# Comprehensive Gene and microRNA Expression Profiling Reveals a Role for microRNAs in Human Liver Development

**DOI:** 10.1371/journal.pone.0007511

**Published:** 2009-10-20

**Authors:** Galit Tzur, Ariel Israel, Asaf Levy, Hila Benjamin, Eti Meiri, Yoel Shufaro, Karen Meir, Elina Khvalevsky, Yael Spector, Nathan Rojansky, Zvi Bentwich, Benjamin E. Reubinoff, Eithan Galun

**Affiliations:** 1 The Goldyne Savad Institute for Gene Therapy, Hadassah Hebrew University Hospital, Jerusalem, Israel; 2 Rosetta Genomics, Rehovot, Israel; 3 Department of Pathology, Hadassah Hebrew University Hospital, Jerusalem, Israel; 4 Department of Obstetrics and Gynecology, Hadassah Hebrew University Hospital, Jerusalem, Israel; University of Calgary, Canada

## Abstract

**Background and Aims:**

microRNAs (miRNAs) are small noncoding RNAs that regulate cognate mRNAs post-transcriptionally. miRNAs have been implicated in regulating gene expression in embryonic developmental processes, including proliferation and differentiation. The liver is a multifunctional organ, which undergoes rapid changes during the developmental period and relies on tightly-regulated gene expression. Little is known regarding the complex expression patterns of both mRNAs and miRNAs during the early stages of human liver development, and the role of miRNAs in the regulation of this process has not been studied. The aim of this work was to study the impact of miRNAs on gene expression during early human liver development.

**Methods:**

Global gene and miRNA expression were profiled in adult and in 9–12w human embryonic livers, using high-density microarrays and quantitative RT-PCR.

**Results:**

Embryonic liver samples exhibited a gene expression profile that differentiated upon progression in the developmental process, and revealed multiple regulated genes. miRNA expression profiling revealed four major expression patterns that correlated with the known function of regulated miRNAs. Comparison of the expression of the most regulated miRNAs to that of their putative targets using a novel algorithm revealed a significant anti-correlation for several miRNAs, and identified the most active miRNAs in embryonic and in adult liver. Furthermore, our algorithm facilitated the identification of TGFβ-R1 as a novel target gene of let-7.

**Conclusions:**

Our results uncover multiple regulated miRNAs and genes throughout human liver development, and our algorithm assists in identification of novel miRNA targets with potential roles in liver development.

## Introduction

The liver transcriptome codes for diverse metabolic functions, such as protein synthesis, biotransformation of molecules and hormone release. While most of these functions fully develop only after birth, they evolve during embryonic liver development through dynamic changes of gene expression. Current evidence suggests that certain genes are critical to normal development of the liver, but the overall pattern of interacting genetic elements remains obscure, especially in humans. Gene expression profiling performed on mouse embryonic liver tissues [1], [2] and on isolated murine hepatoblasts [3] has identified a unique expression pattern relative to the adult liver. In humans, the gene expression pattern of the fetal liver was analyzed using the mRNA differential display technique, and revealed multiple regulated genes, most of which were turned on during organogenesis, whereas some were exclusively expressed in the fetal liver [4].

MicroRNAs (miRNAs) are small noncoding RNAs that regulate mRNA and protein expression [5]. miRNAs have been implicated in regulating gene expression in most stages and processes of embryonic development, such as cell differentiation, cell proliferation, and organ formation (reviewed in [6]).

The function of miRNAs during liver development is currently poorly understood. miRNA expression has been characterized in a relatively late stage of human liver development and revealed changes in miRNA expression between a five-month old fetal liver tissue and several hepatocellular carcinoma cell lines [7]. Such profiling, however, has not yet been applied for earlier stages of human liver development, which are characterized by extensive cell proliferation as well as differentiation.

Analysis of both gene and miRNA expression is an important step towards understanding the developmental process taking place in the liver. Furthermore, integration of gene and miRNA expression data will enable the estimation of the extent to which miRNAs regulate gene expression during human liver development, and the identification of novel target genes of differentially-expressed miRNAs.

In the present work, we measured gene and miRNA expression in adult liver tissue samples and embryonic liver samples corresponding to the early stages of human liver development, and harnessed a novel algorithm in order to analyze the impact of miRNA expression on gene regulation. We detected increased levels for specific miRNAs in either the embryonic or the adult liver, and for specific miRNAs, we show a negative correlation between the pattern of expression of the miRNA, and the pattern of expression of their predicted target genes. This analysis has also led us to discover a miR-target relationship for at least one of the regulated miRNAs, let-7c, with the differentially-expressed gene TGFBR1.

## Materials and Methods

### Tissues

Human liver and other tissues were collected from aborted healthy embryos under the Hadassah Ein Kerem institutional IRB approval, and after obtaining a written informed consent for each embryo. After identification and separation of the liver tissue, the liver and the rest of the embryo were immediately snap-frozen in liquid nitrogen and then stored at −80°C until analysis. The age of the embryos was defined according to the first day of the last menstrual period, and confirmed by ultrasound performed at 7–9 weeks from the first day of last menstrual period. Ultrasound-based age dating accuracy is ±5d.

### Immunohistochemistry

Immunohistochemistry was performed on 5 µm-thick sections from formalin-fixed paraffin- embedded hepatic tissue. Incubation with antibodies to cytokeratin-7 (diluted 1∶2000; DAKO, Glostrup, Denmark), cytokeratin 19 (diluted 1∶75; DAKO), alpha-fetoprotein (diluted 1∶500; DAKO), and cytokeratin 18 (diluted 1∶1000; Sigma, St. Louis, MO) was performed on an automated immunostainer (Nexes, Ventana, Tucson, USA), with diaminobenzidine/H_2_O_2_ as a chromogen.

### RNA Isolation

Total RNA was isolated from cells or tissues with Trizol reagent (Invitrogen, Carlsbad, CA) following the manufacturer's instructions, with the exception that the RNA was precipitated overnight at −20°C in ethanol. RNA quality and quantity were analyzed using gel electrophoresis and Nanodrop (ND-1000 Spectrophotometer).

### cDNA Microarray

Gene expression profiling was performed using the Affymetrix Human Genome U133A Plus 2.0 Array (Santa Clara, CA), for analysis of over 47,000 transcripts, including ∼21,000 well-characterized human genes, according to the manufacturer's instructions. Data was normalized using the GCRMA algorithm [8] implemented in R and bioconductor [9]. T-tests were performed using Excel software. Cluster analysis was performed using Cluster 3.0 [10], and heat maps were produced with Java Treeview software [11]. The datasets have been deposited in NCBI's Gene Expression Omnibus [12] and are accessible through GEO Series accession number GSE15238 (http://www.ncbi.nlm.nih.gov/geo/query/acc.cgi?acc=GSE15238).

### microRNA Microarray

Custom microRNA microarrays were prepared as described previously [13]. Briefly, ∼750 DNA oligonucleotide probes representing microRNAs were spotted in triplicate on coated microarray slides (Nexterion® Slide E, Schott, Mainz, Germany). 3–5 µg of total RNA were labeled by ligation of an RNA-linker, p-rCrU-Cy/dye (Dharmacon, Lafayette, CO; Cy3 or Cy5) to the 3′ end. Slides were incubated with the labeled RNA for 12–16 hr at 42°C, and then washed twice. Arrays were scanned at a resolution of 10 µm, and images were analyzed using SpotReader software (Niles Scientific, Portola Valley, CA). Two groups of positive control probes were designed: small RNA synthetic spikes were added to the RNA before labeling to verify the labeling efficiency, and probes for abundant small RNA (e.g., small nuclear RNAs U43, U49, U24, Z30, U6, U48, and U44, and 5.8s and 5s ribosomal RNA) were spotted on the array in triplicates to verify RNA quality. Microarray spots were combined and signals were normalized using polynomial normalization against the median of each probe in the 7 samples.

### miRNA targets predictions

In this study, we used TargetScan 4.0 predictions (downloaded in July 2007) of miRNA targets [14], [15]. TargetScan uses both evolutionary conservation of the seed, and specific context determinants to predict miRNA targets.

### Mapping of probe-sets to miR-seeds

We assigned each probe-set available on the microarray, a list of miRNA seeds that regulate the transcripts detected by the probe-set, as described in [16]. Only when the sequence corresponding to a given miRNA binding site was directly recognized by a probe-set or was located upstream to the sequences detected by the probe-set in the gene, did we consider this probe-set to be affected by the activity of this miRNA seed. We used for this purpose the UCSC genome browser database [17], human build 17 (http://genome.ucsc.edu). We downloaded the TargetScan predictions from the website (http://www.targetscan.org) and mapped them to the UCSC genome.

### Calculation of enrichment of downregulated genes with increasing number of miR target sites

For the given set of miR-seeds, we performed enrichment tests iteratively for each *i* between 1 and the maximal number of target sites, as follows. The total population size (N) was the number of informative probe sets having at least *i-1* target sites for the considered set of miR-seeds, the number of probe-sets reporting downregulation were counted as successes (m). We tested for enrichment of downregulation in the sample of probe-sets detecting at least *i* target sites for these miR-seeds.

### Real-time Quantitative Polymerase Chain Reaction

Quantification of mRNA and microRNA using real-time RT-PCR was carried out using TaqMan-based assay. For mature microRNA quantification, a two-step protocol including reverse transcription with a miRNA-specific primer and TaqMan MicroRNA Reverse Transcription Kit, followed by real-time PCR with TaqMan assays (human) and Taqman PCR Master Mix Kit, was applied on ten nanograms of total RNA for each sample (all reagents were purchased from Applied Biosystems [ABI], Foster City, CA); reaction protocols were carried out according to the manufacturer's instructions. For mRNA quantification, 2.5 mg of total RNA were reverse transcribed using random hexamer primers and Moloney murine leukemia virus reverse transcriptase RNase H minus (both from Promega, Madison, WI). cDNA was amplified with Fast Taqman PCR Master Mix Kit and inventoried human Taqman assays (both from ABI), according to the manufacturer's instructions. The reactions for miRNA and mRNA were automated by a 7900HT Fast Real-Time PCR System (ABI). Each PCR reaction was performed in triplicate and the average ct was used for the RQ calculation after normalization to RNU43 (for miRNAs) and human GUSB (for mRNAs) (both from ABI).

### Western Blotting

Western blot assays were carried out using routine procedures. Briefly, cells were homogenized in lysis buffer A (0.25 M sucrose, 20 mM Tris pH 7.6, 1.5 mM MgCl_2_, 10% glycerol, 1 mM EDTA and “Complete mini” protein inhibitor cocktail [Roche Diagnostics, # 11836153001]), incubated on ice for 10 min and centrifuged at 12,000 rpm for 15 min at 4°C for supernatant collection. Primary Abs: anti-TGFBR1 (diluted 1∶250, Abcam, Cambridge, MA, # ab31013), anti-β-Actin (ICN/MP Biomedicals, USA, # 691001). Secondary Ab: Dako EnVision System labeled Polymer-HRP anti rabbit (DAKO, # K4003). Proteins were visualized by the EZ-ECL chemiluminescence detection kit for HRP (Biological Industries, Bet Haemek).

### Plasmids and Transfections

For construction of a vector containing TGFBR1–3′UTR fused to the 3′ of a Luciferase reporter, we used the dual luciferase pmirGLO vector (Promega). A 4-kb fragment of the 4.8-kb human TFGBR1 3′UTR (containing both putative let-7c binding sites) was amplified by PCR applied on genomic DNA extracted from HEK293 cells, using primers: 5′- CCAAGTTTAAACAGATCTGCTCCTGGGTTTTA (*PmeI*), and 5′- CCAAGCTAGC-ACCGGTATGCTCTGACAAATATTAAAC (*Nhe*I, *AgeI*), and cloned into the *PmeI/NheI* sites of pmirGLO, to generate pmirGLO-TGFBR1-long 3′UTR. In addition, ∼200-bp fragments containing either the 5′ (75–82) or the 3′ (3889–3895) putative let-7c binding sites were amplified using primers: 5′ site - 5′- CCAATCTAGAAGATCTGCTCCTGGGTTTTA (*XbaI*), and 5′- CCAAGCTAGC-ACCGGTTTTCTGTCCTGGGAAAGAAG (*Nhe*I, *AgeI*) (cloned into the *XbaI* site of pmirGLO); 3′ site – 5′– CCAAAGTCGACAACAAGATTTGTGAACTGAA (*SalI*), and 5′- CCAACTGCAG ATGCTCTGACAAATATTAAAC (*PstI*) (cloned into *SalI/SbfI* sites of pmirGLO), to generate pmirGLO-TGFBR1-3′UTR 5′ site and pmirGLO-TGFBR1-3′UTR 3′ site, respectively. For generation of mutated 5′ and 3′ sites, five mutations were introduced to the putative let-7c binding sites by PCR. The sequence of the 5′ site was amplified from pmirGLO-TGFBR1-3′UTR 5′ site by PCR using primers: 5′- CCAATCTAGAAGATCTGCTCCTGGGTTTTAATTTGGGAGGTCAATTGTTCTTCACATCTGAGAGGGAAC (*Xba*I), and 5′- CCAAGCTAGC-ACCGGTTTTCTGTCCTGGGAAAGAAG (*Nhe*I, *AgeI*) and recloned into the *Xba*I site of this vector. The sequence of the 3′ site was amplified from pmirGLO-TGFBR1-3′UTR 3′ site by PCR using primers: 5′– CCAAAGTCGACAACAAGATTTGTGAACTGAA (*SalI*), and 5′- CCAACTGCAG ATGCTCTGACAAATATTAAACATTATATACACAAATGTGAAAATGTACCTTGG (*PstI*) and recloned into the *SalI/SbfI* sites of this vector. The basic vector pmirGLO served as an empty vector. All vectors were sequence-verified.

Transfection of plasmids and or pre-miR reagents (pre-miR negative control #1 and pre-miR let-7c, Ambion) was performed with Oligofectamine reagent (Invitrogen, Carlsbad, CA), according to the manufacturer's instructions. The cells were plated 24 hrs prior to transfection in 6-well plates for RNA extraction or 24-well plates for luciferase assay (using the Dual Luciferase assay as described by the manufacturer – Promega). The cells were transfected with 25 ng of a Luciferase-containing plasmid, and or 7.5–30 nM of pre-miR reagent, as detailed in the text and the figure legends.

### Statistical Analysis

Data is expressed using the mean and the standard deviation when at least three independent experiments were performed. The two-tailed student's *t* test was used for performing analysis of variance in Excel software. A *p* value of 0.05 or less was considered statistically-significant.

## Results

### Embryonic tissue

Following the very early phase of liver development (upon budding on ∼ day 25 in humans), the liver undergoes a massive proliferation phase to meet the needs during the gestational and neonatal periods (to ultimately harbor ∼10^11^ cells in the adult period), while already maintaining some metabolic functions. During this unique period, hepatocytes and non-parenchymal cells need to both proliferate exponentially in order to meet the fetal demands and to progress in differentiation. The current study aims to investigate the transcriptional networking that enables these functions (proliferation while differentiating) during early human liver development, and to explore the possible involvement of miRNAs in the regulation of these events. To this end, we collected human liver tissues from aborted healthy embryos. We collected a total of six liver tissues of embryos aged 9–12 weeks from the last menstrual period (7–10 weeks from gestation) (Table S1). We verified the identity of the liver tissue histologically and/or by RT-PCR for known liver-specific markers, alpha-1 antitrypsin and albumin.

**Figure 1:**
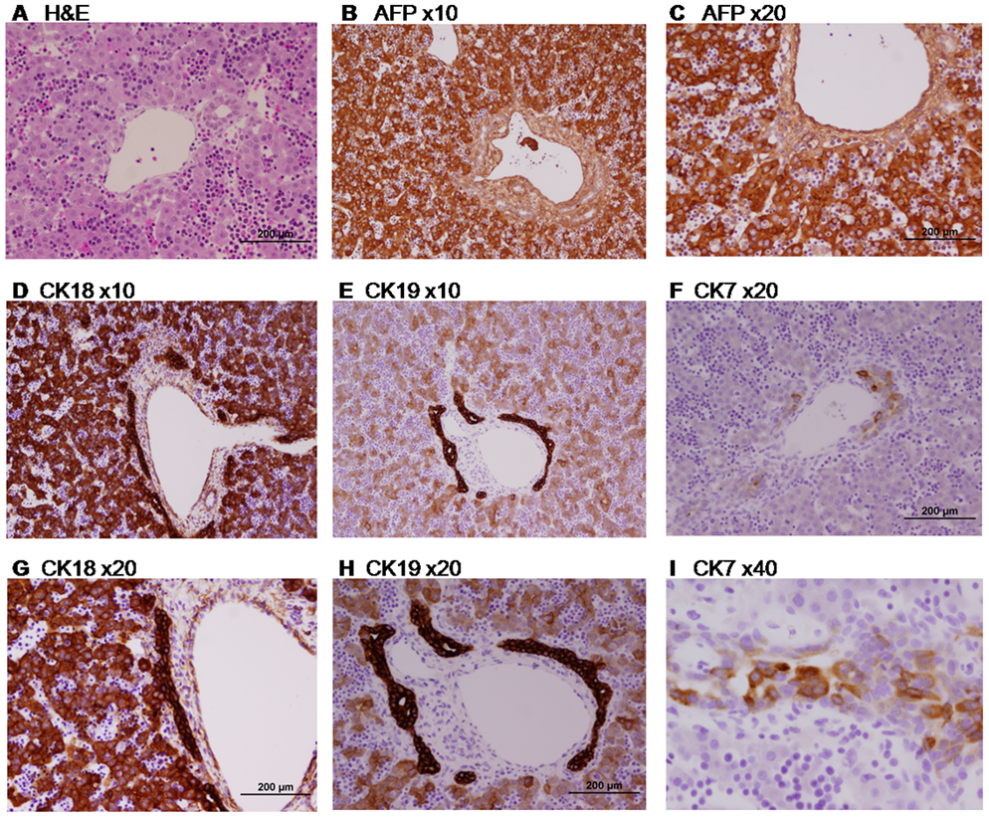
Histology of human embryonic liver at 11 weeks. Paraffin-embedded sections of human embryonic liver at 11 weeks (9 weeks of gestation) stained for Hematoxylin and Eosin (H&E) (A), Alpha-Fetoprotein (AFP) (B, C), Cytokeratin 18 CK18) (D, G), Cytokeratin 19 (CK19) (E, H) and Cytokeratin 7 (CK7) (F, I).

To determine the cellular content and structure of the human fetal liver at this early phase, we first conducted a histological analysis and investigated the expression pattern of known hepatocytes and cholangiocytes markers. In H&E staining (Figure 1A), hematopoietic islands are apparent between non-organized hepatoblasts, teaching the importance of the liver as a temporary hematopoietic site initiating approximately at 6 weeks post-gestation [18]. In some cases, a liver triad (containing vein, artery and bile ducts) can also be observed, with the beginning of bile duct formation. A high level of alpha-fetoprotein (ΑFP) staining (Figure 1B, C) discriminate the hepatocytes (positive) from both hematopoietic cells and blood vessels cells (negative). Cholangiocytes stain negatively for ΑFP. Cytokeratins (CK), marker epithelial cells in the liver, are expressed at early stages in hepatoblasts, while later on, CK18 will be expressed in hepatocytes only, and CK7 and CK19 will be expressed in cholangiocytes. At 11 weeks, CK18 (Figure 1D, G) is still expressed in both cell types, CK19 (Figure 1E, H) is also still expressed in hepatocytes but mainly in cholangiocytes, and CK7 (Figure 1F, I) is expressed only in cholangiocytes.

### Analysis of global gene expression in embryonic and adult human livers

**Figure 2:**
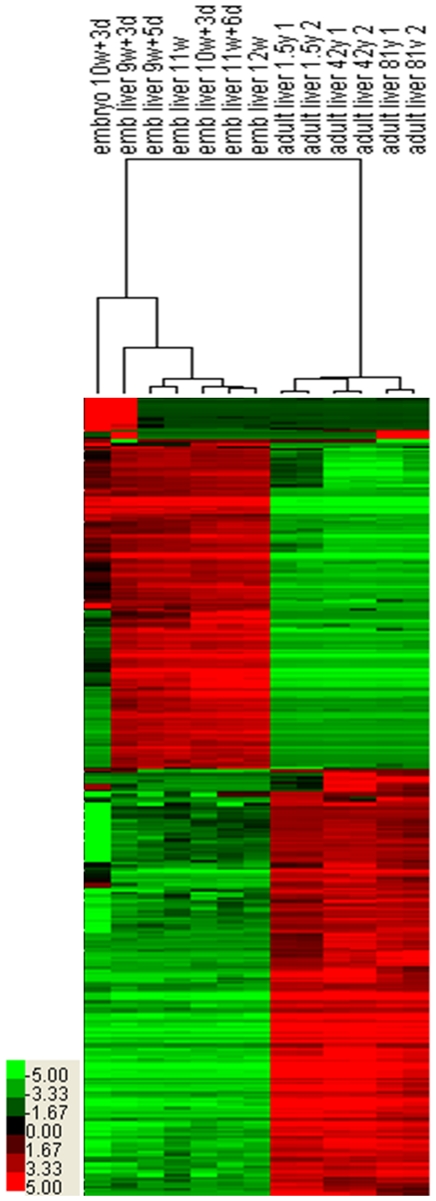
Global gene expression in embryonic and adult human liver. Unsupervised hierarchical cluster analysis was performed on differentially-expressed genes (SD>3) between embryonic (emb) and adult human liver samples (2 samples, named 1 and 2, were taken from each of the three adult liver sources) and whole embryo without liver sample (Cluster 3.0 software, average linkage). A dendogram, demonstrating similarity level in gene expression between the various samples, and a heat map illustrating gene expression changes between the samples, are shown. Samples are listed in columns; genes in rows; red color signifies high expression and green color signifies low expression, according to the color bar shown on the left in logarithmic scale. w – embryonic week calculated from last menstrual period, d – day, y – year after birth.

The significant different tissue ultra-structure of the human embryonic liver compared to the adult liver suggests that it harbors a unique molecular driving force, which navigates its developmental process. In an effort to reveal the functions essential for this process, we characterized the global gene expression in the embryonic livers, and compared it to the expression in three different adult liver tissues and in a whole embryo without the liver tissue of the same age (Table S2). Cluster analysis (Figure 2) revealed a relatively high similarity level in gene expression between the different embryonic livers. The embryo without the liver sample was clustered independently of the embryonic livers, and though it shared many genes with those livers, it expressed some genes higher than the embryonic livers and expressed many genes significantly lower. The gene expression pattern in this sample was closer to the one observed in embryonic liver than the one of adult liver. The adult livers were clustered independently of the embryonic tissues, when each duplicate of samples taken from the same adult was clustered together and seemed nearly identical, confirming the reliability of the microarray results. Generally, 2344 mapped genes were upregulated and 2130 genes were downregulated significantly (p<0.01, at least two-fold) in embryonic liver when compared to adult liver. When compared to the whole embryo, more genes were considerably (at least four-fold) downregulated (2329) than upregulated (1288) in the embryonic liver. The expression of selected differentially-expressed genes was verified with quantitative (q) RT-PCR (see below). In summary, many genes are differentially-expressed between embryonic and adult liver, and between embryonic liver and embryo without liver.

### Functional classification of significantly-regulated genes

In order to investigate what functions the regulated genes may fulfill in the embryonic or the adult liver, we performed functional classification analysis of significantly-regulated genes using the Panther algorithm [19], and searched for significantly-enriched biological processes and pathways. Table S3 lists the top 10 biological processes and pathways which were significantly-enriched in differentially-expressed genes between embryonic and adult livers. Genes that were upregulated in the embryonic liver were classified as related mainly to the cell cycle, and to additional processes that are associated with the cell cycle, such as DNA replication and mitosis. The Ubiquitin-Proteasome pathway was ranked as the most significantly over-represented pathway among these embryonic liver-enriched genes. This result may reflect the importance of ubiquitin-mediated proteolysis as a mechanism for cell cycle control (reviewed in [20]). Heme biosynthesis was the second most significantly over-represented pathway, demonstrating the hematopoietic activity taking place in the embryonic liver at this stage. Genes that were upregulated in the adult liver were classified as related mainly to metabolic functions and immunity. Nevertheless, metabolic function such as cholesterol biosynthesis was ranked among the significantly over-represented pathways in embryonic liver-enriched genes. This result agrees with the enhanced sterol synthesis observed in fetuses compared with adults [21], and is likely associated with the increased proliferation and the requirement for membrane substrates. Interestingly, apoptosis, which is a known regulatory pathway of embryonic development, was over-represented among genes that were upregulated in the adult (and quiescent) liver and not in the embryonic liver. Table S4 lists the top 10 biological processes and pathways which were significantly-enriched in differentially-expressed genes between embryonic liver and whole embryo without liver. Here, genes that were upregulated in the embryonic liver were classified as related to metabolism and transport, indicating that the embryonic liver expresses multiple metabolic genes already at this early developmental stage, while genes that were downregulated in the embryonic liver were classified as related to development of many tissues other than the liver.

### Gene expression changes during early liver development

In order to detect changes in gene expression that occur in the embryonic liver within the developmental window we had access to, we performed a cluster analysis on the six embryonic liver samples only. This analysis separated the embryonic liver samples into 2 groups, which corresponded to different age ranges: the first group included embryos which age was estimated between 9 and 10 weeks, while the second group included samples which age was estimated between 10 and 12 weeks, suggesting that two different stages of embryonic development were present in our dataset (Figure S1). Two samples, which age was estimated by ultrasound as 10w+3d and 11w, near the age boundary of the clusters, were segregated as belonging to the later and earlier stages respectively. This result may be explained by the imprecision of the ultrasound method used for determining the age of the embryos, which although considered to be the most reliable method, is only accurate within a range of ±5 days.

Multiple genes were significantly (p<0.01, at least two-fold) differentially-expressed between these stages (Table S5), when considerably more genes were upregulated (1908 probe sets, 1509 mapped genes) than downregulated (432 probe sets, 301 mapped genes) in the later stage compared to the earlier one. Analysis of enrichment in specific categories of biological processes and pathways among the regulated genes revealed significant enrichments in genes related to metabolism, protein and mRNA processing, cell cycle, and mitosis, among genes upregulated throughout development (Table S6). The earliest sample, 9w+3d, seemed to express a noticeable amount of genes higher than the rest of the samples, and some genes lower. This could reflect the fact that this was the earliest sample taken, and the only female of all six samples. Contamination by other tissues is not likely, as this sample was taken from a nearly undamaged embryo using a binocular microscope, and the liver structure was well preserved during the procedure.

### Analysis of global miRNA expression in embryonic and adult livers

**Figure 3:**
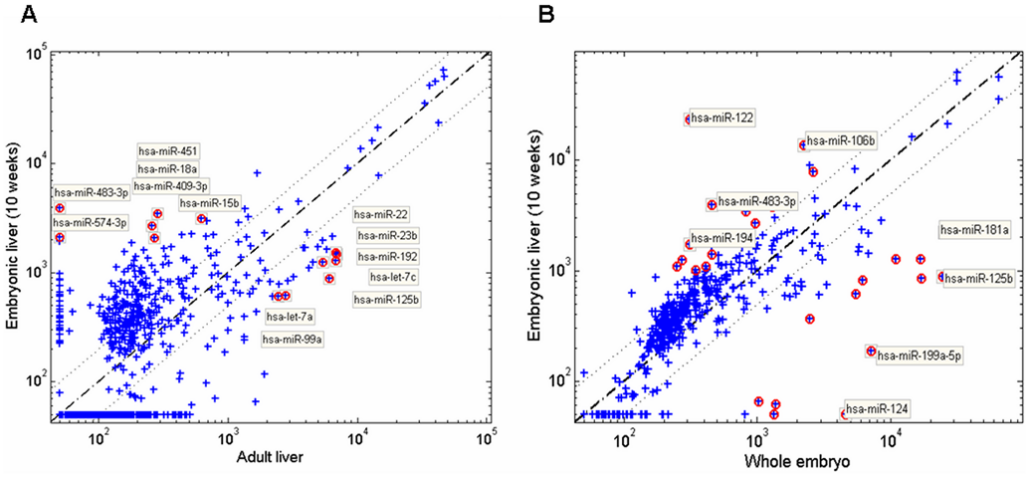
miRNA expression in embryonic liver vs. adult liver and vs. embryo without liver. Scatter plots representing miRNA expression profiles of 507 known miRNAs and additional predicted miRNAs of Rosetta Genomics in samples of embryonic liver (10 weeks) vs. adult liver (A) or vs. the corresponding embryo without liver sample (B). Each blue cross represents a miRNA, and the top 15% of the upregulated and downregulated miRNAs are labeled with red circles. Names of outlier miRNAs are indicated.

**Figure 4:**
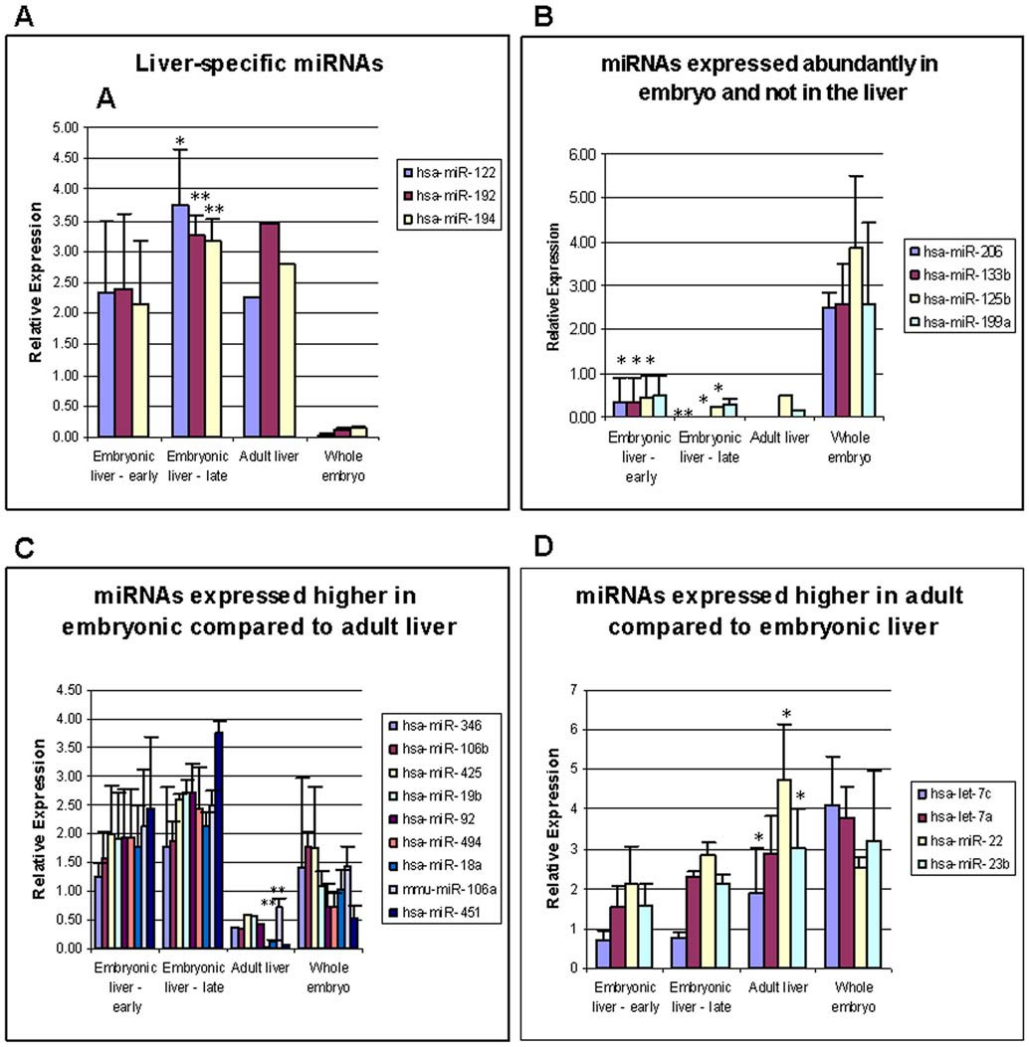
Validation of miRNA expression patterns in embryonic and adult human liver. QRT-PCR results of miRNA expression level in earlier (9–10w) and later (11–12w) stages of liver development, adult liver and whole embryo without liver (10–11w). Results are expressed as relative quantification (RQ) of the miRNA expression level in each sample relative to 9w embryonic liver, and normalized to RNU43. Each reaction was performed in triplicate and the average ct was used for RQ calculation. The average RQ is shown with the SD for 3 different samples (embryonic and adult liver), or 2 different samples (whole embryo). * p<0.05, ** p<0.01 in 2-tailed student's *ttest* when compared to whole embryo (A,B) or to embryonic liver 9–12w (C,D).

miRNAs are estimated to regulate at least one third of all human transcripts [14]. Furthermore, specific miRNAs are important factors in the regulation of cell cycle, differentiation and development (reviewed in [22]). Yet, the role of miRNAs at a state of high cellular proliferation while differentiating at early developmental phases, in particular in the human liver, is unknown. In an effort to investigate which roles miRNAs may play during early human liver development, we profiled the expression of 507 known miRNAs and additional miRNAs validated by Rosetta Genomics in embryonic and adult liver samples and in the corresponding embryos without liver samples (detailed in Table S1), using custom miRNA microarrays (Table S7). The top 15% of the differentially-expressed miRNAs are listed in Table 1, and representative scatter plots, comparing miRNA expression in embryonic liver versus adult liver or versus embryo without liver are shown in Figure 3. We identified several miRNA expression patterns, and verified the expression of selected miRNAs of each pattern with qRT-PCR. The first pattern was of liver-specific miRNAs, represented in Figure 4A by miR-122, miR-192 and miR-194. These miRNAs were not expressed in the rest of the embryo, and were highly-expressed both in the embryonic and the adult livers. miR-122 is known to be liver–specific, and is highly-expressed also in mouse embryonic liver [23], [24]. Our results show that this miRNA is expressed already at 9 weeks (7 weeks from gestation) as high as in adult liver. miR-192 and miR-194, also grouped here, are expressed relatively specifically in endoderm-derived tissues, such as the liver, lungs and the gastrointestinal tract [13], [25], [26]. Reciprocally, some miRNAs were abundantly-expressed in the rest of the embryo, and expressed at very low levels in embryonic and in adult livers (Figure 4B): for instance, miR-206, which was shown to function in skeletal muscle development [27]. A third pattern contained miRNAs that were highly-expressed in embryonic liver compared to adult liver (Figure 4C). The fourth pattern contained miRNAs that were expressed higher in the adult liver compared to the embryonic liver (Figure 4D). These results demonstrate a unique miRNA expression in the early embryonic liver compared to other embryonic tissues and to the adult liver, and suggest a regulatory role for the differentially-expressed miRNAs.

**Table 1:** The top 15% differentially-expressed miRNAs during liver development.

Expressed higher in adult compared to embryonic liver †	Expressed higher in embryonic compared to adult liver ψ	Abundantly-expressed in embryonic liver	Abundantly-expressed in embryo (deprived of embryonic liver)
let-7a	miR-18a	miR-106b	let-7c
let-7b	miR-409-3p	miR-122	miR-124
let-7c	miR-451	miR-192	miR-125a-5p
miR-125b	miR-483-3p	miR-194	miR-125b
miR-192	miR-92a	miR-451	miR-140-3p
miR-22		miR-483-3p	miR-199a-5p
miR-23b			miR-199a-3p
miR-99a			miR-205
			miR-206

† Higher at least 2-fold than the expression in at least 2/3 embryonic livers.

ψ Higher at least 2-fold in at least 2/3 embryonic livers than the expression in adult liver.

### Predicted targets of selected regulated miRNAs are inversely-expressed to their cognate miRNAs

It has been shown that miRNAs accelerate the degradation of their target transcripts and repress their translation. In addition, several studies have shown a clear correlation between miRNAs that are highly expressed in a given tissue and the downregulation of their target transcripts [16], [25], [28]–[30]. The increased levels that were observed for some miRNAs in either the adult or embryonic liver, suggest that many genes that are targets of these miRNA could be repressed in the liver embryo, or in the adult liver. In order to assess the impact of these miRNAs on gene expression, we examined the behavior of predicted targets for these miRNAs using the MiRABELLE algorithm that we have recently published [16]. This algorithm uses the gene expression levels to compute scores that reflect the extent of downregulation of target genes for each miRNA seed in the samples. Using this algorithm, we systematically surveyed the target gene behavior of all the differentially-expressed miRNAs that we found. Among adult liver-enriched miRNAs, we confirmed a statistically-significant downregulation of transcripts that have predicted binding sites for the let-7/98, miR-22 and miR-23 seeds, which correspond to the miRNAs identified previously as expressed higher in the adult liver than in the embryo: let-7a, let-7b, let-7c, miR-22, and miR-23b. The downregulation of these transcripts could reflect other regulatory mechanisms in addition to miRNAs. To provide additional support for the hypothesis that increased levels of miRNAs were instrumental in the downregulation of these miRNA target genes, we verified a known property of miRNA regulation: if the transcripts were downregulated as an effect of miRNA-mediated degradation, then we should observe a more efficient downregulation of transcripts carrying multiple predicted binding sites for miRNAs [31]–[33]. We, therefore, examined the extent of gene target downregulation as a function of the number of binding sites for these seeds in target transcripts.

Table 2 summarizes the expression trends observed for probe-sets that detect transcripts carrying predicted target sites for miRNAs, whose predicted targets were negatively-expressed to them in the most significant manner. To exemplify the targets' behavior trends, we will use the let-7/98 seed, which corresponds to the seed of several miRNAs (let-7a, let-7b and let-7c) that are upregulated in adult compared to embryonic livers. The first line in the table indicates that our microarray platform includes 40,539 informative probe-sets, with 43.67% of them displaying downregulation in the adult liver (without specifying a minimal threshold). Looking further for probe-sets detecting transcripts carrying at least 1 binding site for let-7/98, we find that among the 811 probe-sets detecting such transcripts, the percentage of downregulation increases to 56.60% (p<5.75•10^−14^ by a hypergeometric test). This percentage continues to increase for probe-sets detecting transcripts carrying more miRNA binding sites, reaching 100% for probe-sets detecting transcripts with 4 or more binding sites. The first p-value is usually the most significant, since it reflects enrichment on the largest sample size, and hence has the strongest statistical power. Yet, the trend of gradual increase in the proportion of downregulation can be observed at each increase in the number of target sites, and many of these steps are associated with significant p-values. We calculated a total score summarizing the probability for let-7/98 predicted targets to be increasingly downregulated according to the number of target sites, by summing the (–log) of all the p-values. We observe that this score increases when targets of let-7/98 and miR-23 and miR-22 are considered together, suggesting that many genes are downregulated in the adult by the combined repression of several miRNA species.

**Table 2:** Behavior of predicted target genes of adult liver-enriched miRNAs.

miR Seed	Minimum # of miR binding sites in transcriptsγ	# of probe-sets with at least miR sitesδ	# Down in adult liverε	% Down in adult liver	P value†
**let-7/98**	0	40539	17704	43.67%	
	1	811	459	56.60%	5.75E-14
	2	69	46	66.67%	4.96E-02
	3	13	10	76.92%	3.00E-01
	4	6	6	100.00%	1.22E-01
	5	3	3	100.00%	1.00E+00
					= 15.98 ψ
**let-7/98, miR-23**	0	40539	17704	43.67%	
	1	1591	899	56.51%	7.28E-26
	2	219	132	60.27%	1.27E-01
	3	31	21	67.74%	2.38E-01
	4	11	9	81.82%	2.02E-01
	5	3	3	100.00%	5.09E-01
					= 27.64 ψ
**let-7/98, miR-23, miR-22**	0	40539	17704	43.67%	
	1	1894	1079	56.97%	8.79E-33
	2	285	180	63.16%	1.27E-02
	3	42	28	66.67%	3.72E-01
	4	12	9	75.00%	3.65E-01
	5	4	3	75.00%	7.64E-01
					= 34.94 ψ

Expression trends of probe-sets from the cDNA microarray, according to the number of target sites predicted in the transcripts they detect. The proportion of transcripts that are downregulated in the adult liver gradually increases with the number of target sites for these miRNAs, and this increase is associated with significant hypergeometric p-values. miR – miRNA; # - number.

γ Minimum number of microRNA binding sites for the microRNA seeds. 0 means that no binding site is required.

δ Number of informative probe-sets in the array that detect transcripts matching the requirement for the minimum number of binding sites.

ε Number of probe-sets from **^δ^** that report downregulation in the adult: the average expression in adult liver tissues is lower than the average expression in the embryonic liver tissues.

† P values were calculated using the hypergeometric distribution, as described in the materials and methods section.

ψ Total scores were calculated by summing the (–log) of the p-values. The higher the total score is, the higher the significance of the overall downregulation of the predicted targets.

In Table 3, we used a similar approach to examine the behavior of genes that are targets of embryonic liver-enriched miRNAs. Here, again, we observe a gradual increase in the percentage of downregulated transcript. The best score was obtained for 3 miRNA seeds, corresponding to miRNAs that are expressed higher in the embryonic liver (miR-106a, miR-18a and miR-574-3p), and for which the predicted targets expression displays a negative correlation. In summary, the miRNA seeds that achieved the best scores in terms of anti-correlation with their predicted target genes are presented, and correspond to miRNAs let-7a, let-7b, let-7c, miR-22, and miR-23b, for adult liver-enriched miRNAs (Table 2), and miR-106a, miR-18a and miR-574-3p, for embryonic liver-enriched miRNAs (Table 3).

**Table 3:** Behavior of predicted target genes of embryonic liver-enriched miRNAs.

miR Seed	Minimum # of miR sitesγ	Total transcripts with miR sitesδ	# Down in embryonic liverζ	% Down in embryonic liver	P value†
**miR-17-5p/20/93.mr/106/519.d**	0	40527	21164	52.22%	
	1	4738	2529	53.38%	4.66E-02
	2	1063	600	56.44%	1.24E-02
	3	252	150	59.52%	1.45E-01
	4	73	52	71.23%	1.07E-02
	5	24	17	70.83%	6.33E-01
	6	10	7	70.00%	7.04E-01
					= 6.39 ψ
**miR-17-5p/20/93.mr/106/519.d, miR-18**	0	40527	21164	52.22%	
	1	6284	3308	52.64%	2.39E-01
	2	1757	979	55.72%	1.27E-03
	3	504	305	60.52%	5.88E-03
	4	152	95	62.50%	3.09E-01
	5	62	42	67.74%	1.74E-01
	6	22	16	72.73%	3.71E-01
					= 7.45 ψ
**miR-17-5p/20/93.mr/106/519.d, miR-18, miR-574**	0	40527	21164	52.22%	
	1	6565	3459	52.69%	2.08E-01
	2	1882	1050	55.79%	7.68E-04
	3	570	337	59.12%	3.08E-02
	4	171	109	63.74%	8.41E-02
	5	72	51	70.83%	6.84E-02
	6	27	20	74.07%	4.24E-01
					= 7.92 ψ

Expression trends of probe-sets from the cDNA microarray, according to the number of target sites predicted in the transcripts they detect. The proportion of transcripts that are downregulated in the embryonic liver gradually increases with the number of target sites for these miRNAs, and this increase is associated with significant hypergeometric p-values. miR – miRNA; # - number.

γ Minimum number of microRNA binding sites for the microRNA seeds. 0 means that no binding site is required.

δ Number of informative probe-sets in the array that detect transcripts matching the requirement for the minimum number of binding sites.

ζ Number of probe-sets from **^δ^** that report downregulation in the early embryonic livers: the average expression in adult liver tissues is higher than the average expression in the early embryonic liver tissues.

† P values were calculated using the hypergeometric distribution, as described in the materials and methods section.

ψ Total scores were calculated by summing the (–log) of the p-values. The higher the total score is, the higher the significance of the overall downregulation of the predicted targets.

**Figure 5:**
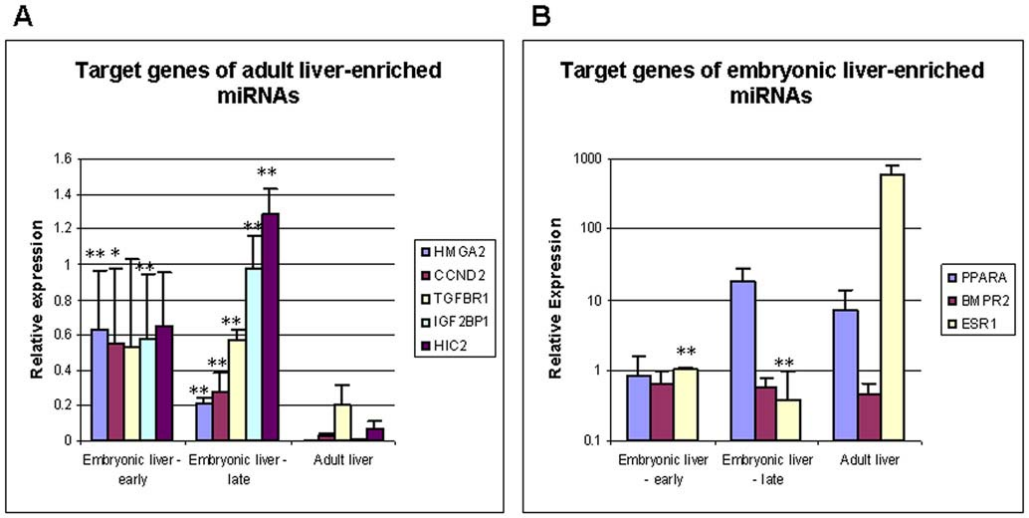
Verification of miRNA predicted-targets expression in qRT-PCR. QRT-PCR results of gene expression level in earlier (9–10w) and later (11–12w) stages of liver development, and adult liver. Results are expressed as relative quantification of the gene expression level in each sample relative to 9w embryonic liver, and normalized to GUSB. Each reaction was performed in triplicate and the average ct was used for RQ calculation. The average RQ of 3 different samples is shown with the SD. * p<0.05, ** p<0.01 in 2-tailed student's *t-test* when compared to adult liver.

We verified the expression of several known and predicted targets of these adult and embryonic liver-enriched miRNAs in the liver tissue samples, using qRT-PCR. Figure 5 shows that indeed, all the predicted and known targets of the adult liver-enriched miRNAs were expressed significantly lower in the adult liver (A), and some of the predicted targets of embryonic liver-enriched miRNAs were expressed lower in early embryonic liver compared with the adult liver (B).

### TGFβ-R1 is a direct target of hsa-let-7c

**Figure 6:**
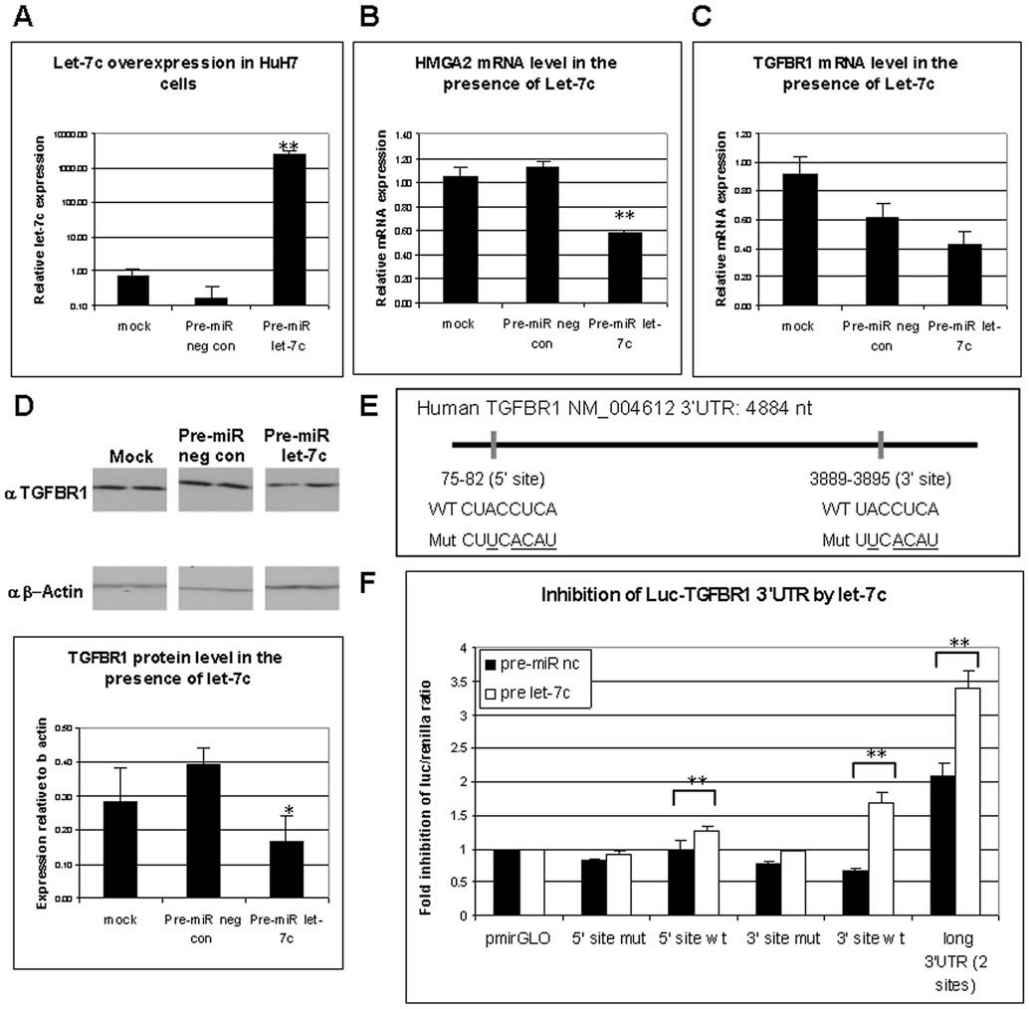
TGFBR1 is a direct target of hsa-let-7c. A. QRT-PCR results of hsa-let-7c expression level in mock transfected HuH7 cells or cells transfected with 30 nM of pre-miR negative control #1 (neg con) or pre-miR let-7c for 48 hrs. B–C. QRT-PCR results of mRNA level of HMGA2 (B) and TGFBR1 (C) in HuH7 cells transfected as described in A. D. Western blot analysis of TGFBR1 protein level in HuH7 cells transfected as described in A for 72 hrs. Quantification of the bands' intensity is shown below. E. Schematic of the 3′UTR of human TGFBR1 with the location and sequence of the 5′ and 3′ putative let-7c binding sites. The 3′UTR fragments that were cloned in pmirGLO are illustrated below. The mutations which were incorporated in the control vectors are underlined below the wt sequences. F. Luciferase/Renilla ratio results for HuH7 cells co-transfected with 7.5 nM of pre-miR negative control (nc) or pre-miR let-7c together with pmirGLO or pmirGLO-TGFBR1 3′UTR 5′ site (wt or mutant) or pGL3-TGFBR1-3′UTR 3′ site (wt or mutant) or pmirGLO-TGFBR1 long genomic 3′UTR (containing both sites), for 72 hrs. Renilla expression from the pmirGLO vector was used for Luc/Renilla ratio quantification. The results are presented as fold-inhibition relative to cells transfected with pmirGLO together with the corresponding pre-miR (nc or let-7c). In all histograms, the average of at least three samples is shown with the SD. * p<0.05, ** p<0.01 in 2-tailed student's *t-test* when compared to pre-miR neg con.

After demonstrating a negative correlation between the expression of selected regulated miRNAs and their predicted target genes, we wished to verify a direct miR-target relationship for one of our differentially-expressed genes and miRNAs. TGF-β signaling plays important roles in the developing liver and throughout adulthood, regulating proliferation and apoptosis [34]–[36]. The type I receptor gene TGFβ-R1 (TGFBR1), which mediates the action of TGF-β, is a predicted target gene of the let-7/98 family according to TargetScan 4.2 (containing conserved sites for let-7a-g and i, and for miR-98). Our results showed a negative correlation between the expression of let-7c and TGFBR1 during liver development, when the first was expressed higher in the adult liver compared to embryonic liver (Figure 4D), and the second was expressed vice versa (Figure 5A). In order to examine whether let-7c may directly regulate TGFBR1 expression, we overexpressed let-7c miRNA in the hepatocellular carcinoma HuH7 cells (Figure 6A), and measured the expression of TGFBR1 mRNA and protein. Let-7c was capable of inhibiting the expression of both HMGA2 (a known target of let-7c [37]) and TGFBR1 mRNAs, though in the case of TGFBR1 mRNA, a partial non-specific effect was shown also by a pre-miR negative control 48 hrs post-transfection (Figure 6B,C). At the protein level, TGFBR1 expression was significantly inhibited by let-7c, and the non-specific effect of the pre-miR negative control was not observed after 72 hrs (Figure 6D). TGFBR1 3′UTR contains two conserved predicted binding sites for let-7c, placed at nucleotides 75–82 and 3889–3895 of the 3′UTR. In order to verify that the inhibitory effect of let-7c is mediated by these predicted binding sites, we cloned the 5′ (75–82) and the 3′ (3889–3895) sites in their native or a mutated form, as well as a long fragment spanning nearly the whole 3′UTR (including both sites) in a luciferase reporter-containing vector (Figure 6E). Transfection of let-7c to HuH7 cells significantly inhibited the level of Luciferase/Renilla ratio when compared to pre-miR negative control in the presence of the wt 5′ and 3′ sites, as well as the long 3′UTR fragment (Figure 6F). Mutations of either of these two sites considerably reduced luciferase repression by let-7c. At the tested let-7c concentration (7.5 nM) the 3′ site conferred a more potent inhibitory effect compared with the 5′ site, yet the inhibitory effect of the 5′ site increased at a higher let-7c concentration (10 nM, data not shown). Taken together, these results show that TGFBR1 is a plausible direct target of let-7c.

## Discussion

In order to enhance our understanding of human liver development and to explore the role of miRNAs during the early stages of this process, we analyzed global gene and miRNA expression in 9–12w old embryonic liver and in adult liver tissues.

Thousands of genes were significantly upregulated or downregulated during liver development. The downregulated genes were related mainly to the cell cycle and hematopoiesis (Table S3), teaching the centrality of these processes at this stage of liver development. Upregulated genes were related mainly to metabolism, despite the fact that multiple metabolic genes were highly-expressed already at this early stage of liver development. This observation may indirectly suggest that some of these genes are regulated at the protein level during the embryonic period. Additional experiments should clarify whether these metabolic genes actively support metabolic activity during this period, and which machineries control their expression.

Cluster analysis of gene expression results may assist in dividing the embryonic development period into sequential stages, as we manage to identify two expression patterns that separate our liver samples into earlier and later stages (Figure S1). Such analysis might ease the identification of the major functions that are accomplished in each developmental stage.

Comparison of miRNA expression profiling between embryonic liver and adult liver or embryo without liver revealed four distinct expression patterns (Figure 4), which can facilitate understanding of their possible roles during human development. Further, the expression pattern of several miRNAs fit nicely with their known function. For instance, one of the expression patterns contained miRNAs that were highly-expressed in embryonic liver compared to adult liver. These miRNAs may regulate processes that take place solely at the embryonic stage (such as hematopoiesis and cell cycle), or may inhibit genes that should be turned on only at later stages. Thus, miR-451, which belongs to this group, was shown to regulate maturation of erythroid cells [38]. Likewise, miR-92, miR-19b and miR-106b, also grouped here, belong to the paralogues and potentially oncogenic miRNA clusters, miR-17-92 and miR-106b-25, and may regulate cell cycle-related genes in the embryonic liver. Another pattern, which contained miRNAs that were expressed higher in the adult liver compared to the embryonic liver, included members of the let-7 family. Let-7 family members have been shown to target proliferation-promoting genes, such as HMGA2 [37] and IGF2BP1 [39], and as such, may mediate cell cycle arrest in the adult liver.

Several reports have recently shown that miRNA activity can be inferred from the relevant gene expression level [16], [40], [41]. Comparison of the expression of regulated miRNAs and their cognate-predicted targets in our arrays revealed significant anti-correlation only for specific regulated miRNAs, which we grouped into two: miRNAs that are enriched in the embryonic liver, including miR-106a, miR-18a and miR-574-3p, and miRNAs that are enriched in the adult liver, including let-7a and c, miR-23b and miR-22. These miRNAs seem to be the most active in embryonic or in adult liver, respectively, at least in terms of mRNA inhibition, since their predicted target genes were negatively-expressed to them at the most significant manner, when compared to other differentially-expressed miRNAs. Perhaps these miRNAs affect mRNA stability more profoundly than other differentially-expressed miRNAs in our study. Additionally, the regulatory effect of miRNAs that do not show negative correlation with their target genes may be masked by additional regulatory machineries such as transcription factors. As for the possible roles of these miRNAs during liver development; while miR-574-3p's role has not yet been studied, miR-106a and miR-18a belong to the oncogenic clusters, miR-106a-363 and miR-17-92, and may regulate cell cycle and apoptosis in the embryonic liver. miR-106a has oncogenic activity in humans [42] and upregulation of miR-18a expression is associated with poor prognosis of serous ovarian cells [43]. Regarding the adult liver-enriched miRNAs, similar to let-7, miR-23b is capable of inhibiting cell proliferation [44], and may mediate cell cycle arrest in mature hepatocytes.

Identification of a miRNA target by usage of gene and miRNA datasets taken from two relatively divergent tissues (such as the embryonic and the adult liver) is relatively complex since, as mentioned above, the mRNAs are most likely regulated by additional machineries. To overcome this hurdle, we focused only on the miRNAs whose predicted target genes showed significant anti-correlation to their cognate miRNA expression by our algorithm. We illustrated this point by choosing the regulated miRNA let-7, which showed the best anti-correlation to its predicted target genes, and one of its predicted targets, TGFBR1, for validation of miR-target relationships. We showed that overexpression of let-7c inhibits both the expression of TGFBR1 mRNA and protein, and further, the expression of a luciferase reporter gene fused to the 3′UTR of TGFBR1 (Figure 6). These results suggest that let-7c, and presumably additional members of the let-7 family, may regulate the expression of TGFBR1 in the liver. TGF-β signaling, which is mediated by TGFBR1, plays important roles in the developing liver and throughout adulthood. Heterozygous inactivation of both Smad2 and Smad3, which are the transcriptional mediators of TGF-β signaling, causes fetal liver hypoplasia [34]. In the adult liver, however, TGF-β has been known to inhibit hepatocyte proliferation [35], [36]. Thus, TGF-β confers opposite effects during different stages of liver development, perhaps by the combined effect of different factors in each stage. Tight regulation of TGF-β signaling activity, therefore, appears essential during liver development and upon maturation. Regulation of TGFBR1 by let-7, which is suggested by our results, may further fine tune the TGF-β signaling activity to the necessary level at each developmental stage. According to a likely scenario, in the embryo, where let-7 is expressed at a relatively low level, it may allow enhanced TGF-β signaling activity that is necessary for hepatocyte proliferation and organization, whereas in the adult, let-7 is expressed higher and may assist in inhibiting TGF-β signaling until it becomes necessary upon specific states, such as liver regeneration.

In this study we identified numerous differentially-expressed genes and miRNAs between embryonic and adult liver tissues. While miRNAs may be responsible for at least some of these expression changes, as demonstrated by our algorithm, additional regulatory mechanisms, such as transcription factors, most likely contribute to the observed changes. The contribution of other factors, such as different compositions/ratios of cell-types in the examined tissues, can also not be excluded. Furthermore, the difficult acquisition of human embryonic liver tissues has limited our liver development analysis to the period of 9–12 weeks, of which 6 different samples were collected. Yet, widening the study to additional stages of liver development is necessary to better understand the impact of miRNAs during human liver development, and reinforce the conclusions of the present study.

In summary, measurement of global gene and miRNA expression in embryonic and adult human livers revealed multiple regulated genes and demonstrated changes in the expression patterns upon progression in the developmental process. Parallel patterns were demonstrated for miRNA and gene expression, when cell cycle and hematopoiesis were the most significant biological process and pathway among the downregulated genes, and accordingly, miRNAs engaged with the regulation of cell cycle and hematopoiesis were downregulated, whereas cell cycle-inhibiting miRNAs were upregulated upon liver maturation. Comparison of the expression of the most regulated miRNAs and the expression of their putative target genes using a dedicated algorithm revealed a significant negative correlation for several miRNAs, identified as the most active miRNAs in either the embryonic or the adult liver, and facilitated the identification of a novel miR-target couple, let-7 and TGFBR1. Future experiments are necessary to uncover additional miRNA targets, and to clarify the role of miRNAs in the regulation of liver development.

## Supporting Information

Figure S1 Gene expression changes during early human liver development Unsupervised hierarchical cluster analysis was performed on differentially-expressed genes (SD>1) between human embryonic (emb) liver samples (Cluster 3.0 software, average linkage). Dendogram, demonstrating similarity level in gene expression between the various samples, and heat map, illustrating gene expression changes between the samples, are shown. Samples are listed in columns; genes in rows; red color signifies high expression and green color signifies low expression, according to the color bar shown on the left.
(0.38 MB TIF)

Table S1 Tissue sample characterizations and types of analysis performed w/o - without, w - embryonic week calculated from last menstrual period, d - day, y - year after birth. ψ For 1.5y-, 42y- and 81y-old adult livers, two samples of the same patient were taken for gene expression analysis. † The age of the embryos was defined according to the first day of the last menstrual period, and confirmed by ultrasound performed at 7–9 weeks from the first day of the last menstrual period. Ultrasound-based age definition accuracy is ±5 days.
(0.05 MB DOC)

Table S2 Affymetrix gene expression
(4.15 MB PDF)

Table S3 Top 10 biological processes and pathways enriched significantly in differentially-expressed genes between embryonic and adult liver * The reference list for the classification analysis was all genes - NCBI: H. sapiens genes. 1 Upregulated at least two-fold in a significant (p<0.01) manner. 2 Bonferroni-corrected for multiple testing.
(0.05 MB DOC)

Table S4 Top 10 biological processes and pathways enriched significantly in differentially-expressed genes between embryonic liver and embryo without liver * The reference list for the classification analysis was all genes - NCBI: H. sapiens genes. * w/o - without. 1 Upregulated at least four-fold. 2 Bonferroni-corrected for multiple testing.
(0.06 MB DOC)

Table S5 Differentially-expressed genes throughout development
(0.39 MB XLS)

Table S6 Top 10 biological processes and pathways enriched significantly in differentially-expressed genes between earlier (9–10w) and later (11–12w) stages of liver development * The reference list for the classification analysis was all genes - NCBI: H. sapiens genes. * NS - non significant. 1 Upregulated or downregulated at least two-fold in a significant (p<0.01) manner. 2 Bonferroni-corrected for multiple testing.
(0.05 MB DOC)

Table S7 microRNA expression
(0.09 MB XLS)
