# Supplementary figures and images for: Comprehensive Gene and microRNA Expression Profiling Reveals a Role for microRNAs in Human Liver Development
Source: PLoS One. 2009 Oct 20;4(10):e7511. doi: 10.1371/journal.pone.0007511 (PMC2760133; doi:10.1371/journal.pone.0007511)

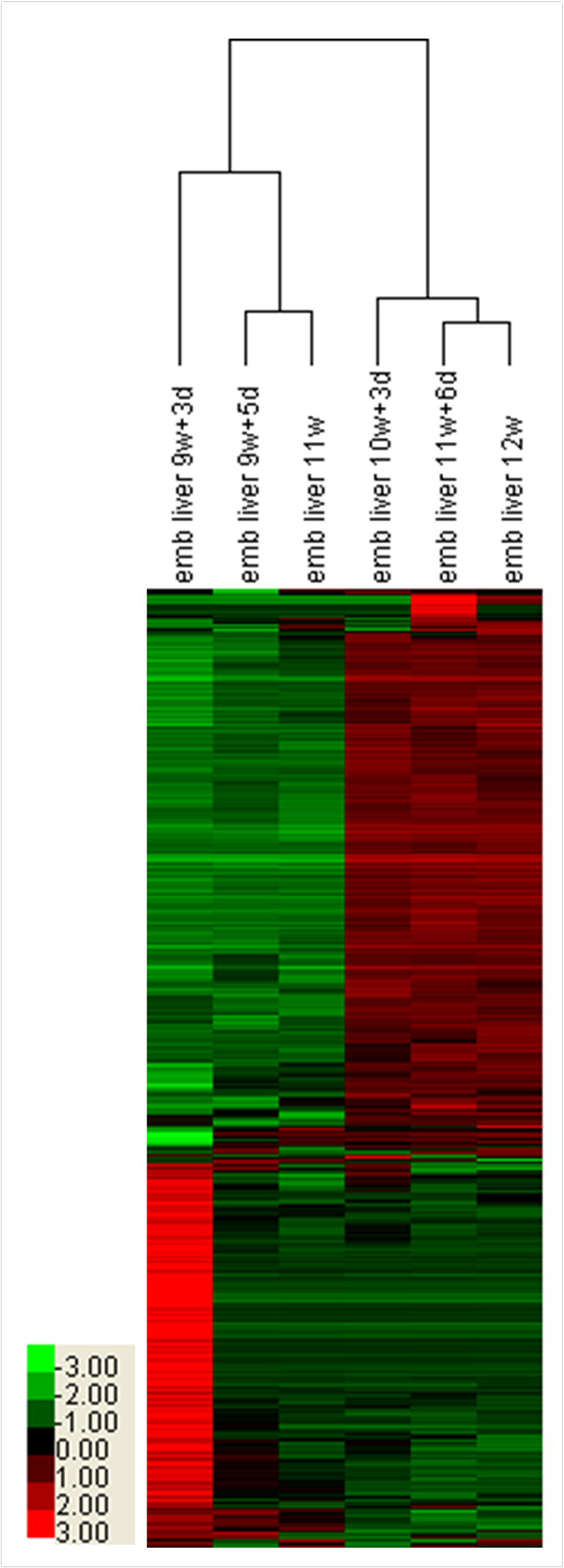

Supplement: Figure S1 — Gene expression changes during early human liver development Unsupervised hierarchical cluster analysis was performed on differentially-expressed genes (SD>1) between human embryonic (emb) liver samples (Cluster 3.0 software, average linkage). Dendogram, demonstrating similarity level in gene expression between the various samples, and heat map, illustrating gene expression changes between the samples, are shown. Samples are listed in columns; genes in rows; red color signifies high expression and green color signifies low expression, according to the color bar shown on the left. (0.38 MB TIF) [file pone.0007511.s001.tif]
