# Supplementary material for: Comprehensive Gene and microRNA Expression Profiling Reveals a Role for microRNAs in Human Liver Development
Source: PLoS One. 2009 Oct 20;4(10):e7511. doi: 10.1371/journal.pone.0007511 (PMC2760133; doi:10.1371/journal.pone.0007511)
Supplement: Table S1 — Tissue sample characterizations and types of analysis performed w/o - without, w - embryonic week calculated from last menstrual period, d - day, y - year after birth. ψ For 1.5y-, 42y- and 81y-old adult livers, two samples of the same patient were taken for gene expression analysis. † The age of the embryos was defined according to the first day of the last menstrual period, and confirmed by ultrasound performed at 7–9 weeks from the first day of the last menstrual period. Ultrasound-based age definition accuracy is ±5 days. (0.05 MB DOC) [file pone.0007511.s002.doc]

**Table S1: Tissue sample characterizations and types of analysis performed**

| **Sample** | **Age ^†^** | **Gender** | **Global Gene expression** | **Global miRNA expression** | **qRT-PCR for miRNA** |
| --- | --- | --- | --- | --- | --- |
| Embryonic liver | 9w+3d | Female | + | + | + |
| Embryonic liver | 9w+5d | Male | + | - | + |
| Embryonic liver | 10w+3d | Male | + | + | + |
| Embryonic liver | 11w | Male | + | + | + |
| Embryonic liver | 11w+6d | Male | + | - | + |
| Embryonic liver | 12w | Male | + | - | + |
| Embryo w/o liver | 9w+3d | Female | - | + | - |
| Embryo w/o liver | 10w+3d | Male | + | + | + |
| Embryo w/o liver | 11w | Male | - | + | + |
| Adult liver | 1.5y | Male | + | - | + |
| Adult liver | 1.5y^Ψ^ | Male | + | - | + |
| Adult liver | 42y | Male | + | - | + |
| Adult liver | 42y^Ψ^ | Male | + | - | - |
| Adult liver | 81y | Female | + | - | - |
| Adult liver | 81y^Ψ^ | Female | + | - | - |
| Adult liver | Commercial tissue (Ambion) | Male | - | + | - |

w/o – without, w – embryonic week calculated from last menstrual period, d – day, y – year after birth.

^Ψ^ For 1.5y-, 42y- and 81y-old adult livers, two samples of the same patient were taken for gene expression analysis.

^†^ The age of the embryos was defined according to the first day of the last menstrual period, and confirmed by ultrasound performed at 7-9 weeks from the first day of the last menstrual period. Ultrasound-based age definition accuracy is ± 5 days.
