# Supplementary material for: Comprehensive Gene and microRNA Expression Profiling Reveals a Role for microRNAs in Human Liver Development
Source: PLoS One. 2009 Oct 20;4(10):e7511. doi: 10.1371/journal.pone.0007511 (PMC2760133; doi:10.1371/journal.pone.0007511)
Supplement: Table S3 — Top 10 biological processes and pathways enriched significantly in differentially-expressed genes between embryonic and adult liver * The reference list for the classification analysis was all genes - NCBI: H. sapiens genes. 1 Upregulated at least two-fold in a significant (p<0.01) manner. 2 Bonferroni-corrected for multiple testing. (0.05 MB DOC) [file pone.0007511.s004.doc]

**Table S3: Top 10 biological processes and pathways enriched significantly in differentially-expressed genes between embryonic and** adult liver

| **Genes upregulated^1^ in embryonic liver** | | | **Genes upregulated^1^ in adult liver** | | |
| --- | --- | --- | --- | --- | --- |
| **Biological Process** | **Number of genes**  **(out of 2344 mapped Ids)** | **P-value^2^** | **Biological Process** | **Number of genes**  **(out of 2130 mapped Ids)** | **P-value^2^** |
| Nucleoside, nucleotide and nucleic acid metabolism | 557 | 1.09E-42 | Immunity and defense | 299 | 4.33E-52 |
| Cell cycle | 250 | 3.06E-42 | Lipid, fatty acid and steroid metabolism | 200 | 4.89E-42 |
| DNA metabolism | 103 | 1.3E-20 | Amino acid metabolism | 79 | 2.64E-23 |
| Pre-mRNA processing | 91 | 1.37E-20 | Steroid metabolism | 71 | 3.39E-23 |
| Mitosis | 100 | 2.27E-17 | Fatty acid metabolism | 63 | 1.7E-17 |
| Protein metabolism and modification | 423 | 2.46E-16 | Other metabolism | 113 | 2.17E-15 |
| DNA replication | 55 | 2.23E-14 | Signal transduction | 412 | 4.04E-13 |
| mRNA splicing | 66 | 2.61E-14 | Carbohydrate metabolism | 110 | 1.3E-12 |
| Chromosome segregation | 42 | 2.18E-10 | Electron transport | 62 | 8.82E-12 |
| Cell cycle control | 89 | 2.21E-10 | Complement-mediated immunity | 26 | 1.02E-09 |
| **Pathway** |  |  | **Pathway** |  |  |
| Ubiquitin proteasome pathway | 27 | 0.000026 | Blood coagulation | 22 | 6.75E-07 |
| Heme biosynthesis | 11 | 0.0000764 | 5-Hydroxytryptamine degradation | 10 | 0.000426 |
| Cell cycle | 14 | 0.000148 | Nicotine degradation | 8 | 0.00047 |
| Parkinson disease | 24 | 0.0136 | Pyrimidine Metabolism | 9 | 0.000663 |
| Cholesterol biosynthesis | 7 | 0.0149 | Inflammation mediated by chemokine and cytokine signaling pathway | 51 | 0.00198 |
| p53 pathway feedback loops 2 | 17 | 0.0332 | Phenylethylamine degradation | 7 | 0.00456 |
|  |  |  | T cell activation | 24 | 0.00639 |
|  |  |  | Apoptosis signaling pathway | 25 | 0.0304 |

^*^ The reference list for the classification analysis was all genes - NCBI: H. sapiens genes.

^1^ Upregulated at least two-fold in a significant (p<0.01) manner.

^2^ Bonferroni-corrected for multiple testing.
