# Supplementary material for: Comprehensive Gene and microRNA Expression Profiling Reveals a Role for microRNAs in Human Liver Development
Source: PLoS One. 2009 Oct 20;4(10):e7511. doi: 10.1371/journal.pone.0007511 (PMC2760133; doi:10.1371/journal.pone.0007511)
Supplement: Table S4 — Top 10 biological processes and pathways enriched significantly in differentially-expressed genes between embryonic liver and embryo without liver * The reference list for the classification analysis was all genes - NCBI: H. sapiens genes. * w/o - without. 1 Upregulated at least four-fold. 2 Bonferroni-corrected for multiple testing. (0.06 MB DOC) [file pone.0007511.s005.doc]

**Table S4: Top 10 biological processes and pathways enriched significantly in differentially-expressed genes between embryonic liver and embryo without liver**

| **Genes upregulated^1^ in embryonic liver** | | | **Genes upregulated^1^ in embryo w/o liver** | | |
| --- | --- | --- | --- | --- | --- |
| **Biological Process** | **Number of genes**  **(out of 1288 mapped Ids)** | **P-value^2^** | **Biological Process** | **Number of genes**  **(out of 2329 mapped Ids)** | **P-value^2^** |
| Lipid, fatty acid and steroid metabolism | 147 | 1.81E-40 | Developmental processes | 538 | 6.86E-100 |
| Immunity and defense | 166 | 6.84E-25 | Signal transduction | 599 | 4.27E-55 |
| Transport | 154 | 2.96E-20 | Ectoderm development | 207 | 5.19E-46 |
| Amino acid metabolism | 56 | 1.14E-19 | Neurogenesis | 182 | 4.25E-42 |
| Blood clotting | 33 | 1.53E-15 | Mesoderm development | 174 | 2.67E-41 |
| Fatty acid metabolism | 45 | 6.25E-15 | Cell structure and motility | 257 | 1.01E-36 |
| Other metabolism | 79 | 4.93E-14 | Cell communication | 262 | 2.65E-34 |
| Steroid metabolism | 43 | 7.04E-14 | Cell adhesion | 170 | 1.92E-33 |
| Coenzyme and prosthetic group metabolism | 36 | 1.29E-10 | Cell adhesion-mediated signaling | 108 | 1.33E-21 |
| Lipid and fatty acid transport | 32 | 1.41E-10 | Cell structure | 153 | 1.54E-20 |
| **Pathway** |  |  | **Pathway** |  |  |
| Blood coagulation | 30 | 7.13E-19 | Wnt signaling pathway | 96 | 3.09E-18 |
| Plasminogen activating cascade | 8 | 0.00259 | Integrin signaling pathway | 71 | 5.48E-16 |
| Heme biosynthesis | 7 | 0.00369 | Angiogenesis | 64 | 3.88E-12 |
| 5-Hydroxytryptamine degradation | 6 | 0.0448 | Cadherin signaling pathway | 50 | 2.85E-10 |
| Cholesterol biosynthesis | 5 | 0.0465 | Inflammation mediated by chemokine and cytokine signaling pathway | 70 | 7.69E-09 |
|  |  |  | Alzheimer disease-presenilin pathway | 42 | 2.42E-08 |
|  |  |  | Axon guidance mediated by Slit/Robo | 19 | 2.56E-07 |
|  |  |  | Metabotropic glutamate receptor group III pathway | 26 | 0.0000169 |
|  |  |  | Axon guidance mediated by semaphorins | 19 | 0.000018 |
|  |  |  | Metabotropic glutamate receptor group II pathway | 19 | 0.000191 |

^*^ The reference list for the classification analysis was all genes - NCBI: H. sapiens genes.

^*^ w/o – without.

^1^ Upregulated at least four-fold.

^2^ Bonferroni-corrected for multiple testing.
