# Supplementary material for: Comprehensive Gene and microRNA Expression Profiling Reveals a Role for microRNAs in Human Liver Development
Source: PLoS One. 2009 Oct 20;4(10):e7511. doi: 10.1371/journal.pone.0007511 (PMC2760133; doi:10.1371/journal.pone.0007511)
Supplement: Table S6 — Top 10 biological processes and pathways enriched significantly in differentially-expressed genes between earlier (9–10w) and later (11–12w) stages of liver development * The reference list for the classification analysis was all genes - NCBI: H. sapiens genes. * NS - non significant. 1 Upregulated or downregulated at least two-fold in a significant (p<0.01) manner. 2 Bonferroni-corrected for multiple testing. (0.05 MB DOC) [file pone.0007511.s007.doc]

**Table S6: Top 10 biological processes and pathways enriched significantly in differentially-expressed genes between earlier (9-10w) and later (11-12w) stages of liver development**

| **Genes upregulated^1^ throughout development** | | | **Genes downregulated^1^ throughout development** | | | |
| --- | --- | --- | --- | --- | --- | --- |
| **Biological Process** | **Number of genes**  **(out of 1509**  **mapped Ids)** | **P-value^2^** | | **Biological Process** | **Number of genes**  **(out of 301 mapped Ids)** | **P-value^2^** |
| Cell cycle | 141 | 1.08E-18 | | Nucleoside, nucleotide and nucleic acid metabolism | 64 | 0.00205 |
| Protein metabolism and modification | 305 | 1.43E-18 | | Cell cycle | 26 | 0.00633 |
| Nucleoside, nucleotide and nucleic acid metabolism | 319 | 2.15E-16 | | Mitosis | 14 | 0.0331 |
| Pre-mRNA processing | 62 | 5.25E-15 | |  |  |  |
| Protein modification | 142 | 1.51E-13 | |  |  |  |
| mRNA splicing | 49 | 1.04E-12 | |  |  |  |
| Intracellular protein traffic | 111 | 2.64E-08 | |  |  |  |
| Lipid, fatty acid and steroid metabolism | 91 | 3.53E-08 | |  |  |  |
| Mitosis | 57 | 1.02E-07 | |  |  |  |
| Protein phosphorylation | 79 | 0.00000168 | |  |  |  |
| **Pathway** |  |  | | **Pathway** |  |  |
| p53 pathway | 31 | 9.2E-08 | | NS |  |  |
| p53 pathway feedback loops 2 | 18 | 0.0000286 | |  |  |  |
| Parkinson disease | 23 | 0.0000343 | |  |  |  |
| Ubiquitin proteasome pathway | 20 | 0.000123 | |  |  |  |
| T cell activation | 20 | 0.00303 | |  |  |  |
| Integrin signalling pathway | 30 | 0.0107 | |  |  |  |
| Angiogenesis | 29 | 0.0284 | |  |  |  |

^*^ The reference list for the classification analysis was all genes - NCBI: H. sapiens genes.

^*^ NS – non significant.

^1^ Upregulated or downregulated at least two-fold in a significant (p<0.01) manner.

^2^ Bonferroni-corrected for multiple testing.
