# Supplementary material for: Comprehensive Gene and microRNA Expression Profiling Reveals a Role for microRNAs in Human Liver Development
Source: PLoS One. 2009 Oct 20;4(10):e7511. doi: 10.1371/journal.pone.0007511 (PMC2760133; doi:10.1371/journal.pone.0007511)
Supplement: Table S2 — Affymetrix gene expression (4.15 MB PDF) [file pone.0007511.s003.pdf]

GENE_ID	PROBE_ID	adult_liver_1.5y_T0_1	adult_liver_1.5y_T0_2	adult_liver_42y_T0_1	adult_liver_42y_T0_2	adult_liver_81y_T0_1	adult_liver_81y_T0_2	embryonic_liver_10w+3d	embryonic_liver_11w+2d	embryonic_liver_11w+6d	embryonic_liver_12w	embryonic_liver_9w+3d	embryonic_liver_9w+5d	embryo_w/o_liver_10w+3d
DDR1	1007_s_at	6.522448	6.385938	5.40254	5.375076	5.968828	5.787293	6.209515	6.720525	6.12282	6.259905	7.282043	6.968684	8.771543
RFC2	1053_at	5.664809	5.070918	5.952055	5.80305	5.095239	5.374251	7.483748	6.656218	7.625738	7.653072	6.603402	6.970982	7.045369
HSPA6	117_at	3.299646	3.469327	4.3317	3.250807	3.384785	3.12445	3.092326	3.131158	3.298891	3.099298	3.148762	3.20031	2.910479
PAX8	121_at	2.214025	2.207699	2.178511	2.18755	2.199826	2.186513	2.214553	2.207642	2.205508	2.191571	2.178471	2.201338	3.116222
GUCA1A	1255_g_at	2.137795	2.115991	2.087238	2.101466	2.135692	2.108786	2.108223	2.119207	2.10851	2.098448	2.104397	2.190141	2.145546
UBE1L	1294_at	5.559987	5.589831	5.453551	5.710275	5.856592	6.572123	3.774828	4.314512	3.949778	3.110369	4.942295	4.95258	3.476631
THRA	1316_at	3.80127	3.792243	3.756312	3.779587	4.389109	4.161021	3.765141	4.292267	3.768146	3.656633	3.977287	4.638319	3.316693
PTPN21	1320_at	2.690671	2.644829	2.648685	2.843114	2.668332	2.611716	2.644	2.651046	2.653559	2.640655	2.644689	2.657573	2.897066
CCL5	1405_i_at	7.651306	7.484134	6.3315	6.420879	7.681773	8.878778	5.765064	5.742788	5.147417	4.812768	5.280088	6.892921	2.63285
CYP2E1	1431_at	13.83814	13.7781	14.23518	14.24921	13.31648	12.65521	2.1453	2.089097	2.09312	2.049282	2.08601	2.128409	2.080654
EPHB3	1438_at	2.085686	2.064072	2.027354	2.045188	2.066013	2.048853	2.061773	2.062213	2.053063	2.049751	2.108812	2.057967	6.566783
ESRRA	1487_at	6.811175	6.908818	6.564318	6.737607	6.876941	6.408709	5.72031	5.661081	5.634153	5.726612	5.782399	5.640239	4.863807
CYP2A6	1494_f_at	12.96769	13.17296	12.74937	12.82671	13.23515	13.13193	3.038082	3.331001	2.85321	2.73259	3.018589	2.793331	3.024091
SCARB1	1552256_a_at	10.41546	10.4421	10.1198	10.2015	10.15424	10.01958	9.829175	9.855032	9.988574	9.995908	9.687535	9.851833	9.225504
TTLL12	1552257_a_at	6.94494	6.907275	6.17341	6.407803	6.655963	7.030431	8.534741	9.33889	9.007844	8.909631	8.45254	8.830611	6.047942
C2orf59	1552258_at	2.603654	2.548774	2.50573	2.585655	2.588149	2.567242	2.714221	3.388614	3.278956	2.633546	2.816469	2.950013	2.388945
WFDC2	1552261_at	2.289924	2.25046	2.243641	2.248474	2.279233	2.247496	2.247797	2.258508	2.249904	2.243026	2.241526	2.257691	2.144534
MAPK1	1552263_at	4.491138	4.630921	3.795219	4.358734	4.345033	4.600684	4.332027	5.08513	4.619708	4.733211	5.071709	5.656678	4.26915
MAPK1	1552264_a_at	3.319645	3.509596	3.59984	3.253295	3.266882	3.015916	4.79365	3.885613	5.190296	4.81007	3.660346	3.927433	5.817451
ADAM32	1552266_at	3.470794	3.372612	3.288538	3.371919	3.590085	3.33105	3.370939	3.387986	3.376808	3.254684	3.378889	3.413824	3.149034
SPATA17	1552269_at	2.317329	2.290504	2.260827	2.271323	2.300409	2.268725	2.278883	2.290774	2.284514	2.268403	2.276659	2.28604	2.19182
MGC24975	1552271_at	1.856377	1.883486	1.834899	1.839027	1.846336	1.947418	1.849032	1.837029	1.830402	1.81189	1.830571	1.842008	1.775251
MGC24975	1552272_a_at	2.280219	2.277595	2.256321	2.298816	2.275176	2.333009	2.263014	2.282604	2.232971	2.251035	2.233201	2.277007	2.154915
PXK	1552274_at	5.160262	4.569631	4.487933	4.559397	4.765035	4.818547	4.743658	4.460628	4.418782	4.616627	4.552366	4.563363	5.763232
PXK	1552275_s_at	2.648169	2.536091	3.715114	2.611845	2.674386	2.574656	3.709255	2.628478	3.76376	3.563586	2.633009	2.594172	4.801739
VPS18	1552276_a_at	2.360844	2.330802	2.308979	2.312627	2.353733	2.460844	2.316518	2.326477	2.307611	2.290348	2.308362	2.325196	2.257604
C9orf30	1552277_a_at	5.200674	5.349735	5.19577	5.296944	4.811542	4.881787	6.441866	5.762124	6.336398	6.452473	4.726452	5.817663	6.778764
SLC46A1	1552278_a_at	3.25815	3.161212	3.420181	3.15141	3.444774	3.152742	3.168306	3.168171	3.153904	3.137138	3.165617	3.174179	2.833963
SLC46A1	1552279_a_at	5.607705	5.458064	5.931905	5.785803	6.867612	6.642582	5.211748	5.091698	5.031272	4.438475	5.019089	4.599086	3.544384
TIMD4	1552280_at	2.572263	2.514443	6.835246	7.001479	4.226726	3.83487	8.175779	7.545009	7.854429	7.767122	5.878586	7.674479	3.203101
SLC39A5	1552281_at	9.142777	9.182644	8.636995	8.740118	8.232812	8.055237	9.26207	9.071267	8.79379	9.092014	8.826121	9.267168	2.860303
ZDHHC11	1552283_s_at	6.474565	7.314634	6.561192	6.441073	5.987878	6.089353	3.900578	4.951004	3.691762	3.902768	4.398109	4.175136	4.924012
ATP6V1E2	1552286_at	2.658149	2.654303	2.634104	2.636585	3.163806	2.988526	2.539499	2.653942	2.643266	2.615007	2.640468	2.653376	2.601439
AFG3L1	1552287_s_at	6.023462	5.98073	6.211896	5.626615	5.940804	5.80248	7.136242	6.510155	7.158593	6.962546	6.573882	6.478765	6.434002

PIGX	1552291_at	6.877859	6.839326	6.754747	6.992307	6.898689	6.598401	7.172079	7.947999	7.242547	7.17189	7.758954	8.058248	7.754834
MGC42090	1552293_at	2.494103	2.473761	2.455022	2.459302	2.492278	2.337613	2.471813	2.463295	2.466892	2.455667	2.454277	2.463649	2.317331
SLC39A13	1552295_a_at	2.426339	2.284883	2.264966	2.266979	2.273059	2.267999	2.281301	2.266552	2.278056	2.266619	2.256135	2.259164	4.079649
BEST4	1552296_at	2.692353	2.658143	2.655222	2.652004	2.713445	2.677491	2.671221	2.671387	2.660241	2.646509	2.655466	2.669099	2.605537
C6orf199	1552299_at	2.483928	2.455386	2.434412	2.424467	2.455986	2.432452	2.458647	2.45343	2.548247	2.441435	2.516143	2.447422	2.543427
CORO6	1552301_a_at	2.735614	2.674675	2.651486	2.669445	2.689427	2.738919	2.663655	2.673148	2.65792	2.657307	2.957178	2.674313	3.555511
TMEM106A	1552302_at	8.500998	8.491239	6.295124	6.565387	7.844715	8.180266	6.86722	7.822779	6.660967	6.398028	7.902301	7.432705	5.536956
TMEM106A	1552303_a_at	6.380839	5.986563	4.517507	4.818409	5.388899	4.995556	5.185154	5.205257	4.993512	4.989142	5.023691	4.984933	4.662483
ALG10	1552304_at	2.390985	2.37006	2.328345	2.347466	2.379188	2.343036	2.361981	2.367148	2.353176	2.345895	2.348016	2.360313	2.277673
ALG10	1552306_at	2.820701	2.685899	2.783206	2.78733	2.820077	3.501667	2.84644	2.89291	2.850143	2.749451	2.78468	2.797078	2.578424
C18orf17	1552307_a_at	9.575257	9.514818	9.840149	10.02806	8.287771	8.12749	4.839039	5.613683	5.573018	5.310634	4.74324	5.450049	2.056577
NEXN	1552309_a_at	2.368981	2.303214	2.722271	3.30518	2.630434	2.749402	2.760394	2.362288	2.628874	2.621083	6.461245	3.374538	6.633699
C15orf40	1552310_at	6.761509	6.642216	6.374689	6.573453	6.099405	5.808162	6.7965	6.535495	7.06111	6.460803	6.351819	6.521699	6.516564
RAXL1	1552311_a_at	3.365247	4.057607	3.179441	3.27659	3.461332	3.94877	3.33896	3.693586	3.333888	3.260866	3.442323	3.376851	2.892769
MFAP3	1552312_a_at	3.236737	2.940137	3.20273	3.215165	2.751844	3.015234	3.457144	3.219866	3.717282	3.81294	3.239688	3.365409	6.362481
EYA3	1552314_a_at	3.124576	3.060147	3.013	3.041609	3.055021	3.034037	3.133737	3.057206	3.050967	3.036067	3.040701	3.052372	3.001419
GIMAP1	1552315_at	2.969706	2.95931	2.941846	2.939215	3.031864	2.951707	2.954906	2.941155	2.945249	2.935189	3.058933	2.943839	2.935233
GIMAP1	1552316_a_at	6.822585	6.75476	6.220071	6.482791	7.383715	7.405842	5.241869	5.622069	5.038191	5.393091	5.643665	5.709339	4.797813
KLK8	1552319_a_at	3.169004	3.057112	3.029673	3.039875	3.158479	3.119053	3.10915	3.072856	3.06162	3.03617	3.057031	3.118771	2.715008
CCDC65	1552320_a_at	3.099394	2.981252	2.808287	2.964816	3.092477	2.86187	2.974874	2.970221	2.99726	2.96556	3.232539	3.190632	2.533625
CCDC65	1552321_a_at	2.7179	2.654918	2.645981	2.643537	2.697696	2.653586	2.656232	2.664691	2.651216	2.632821	2.649359	2.671646	2.626161
FAM122C	1552322_at	2.428482	2.405787	2.381131	2.388695	2.413432	2.386404	2.39392	2.402131	2.390439	2.384288	2.389266	2.400562	2.316547
FAM122C	1552323_s_at	2.804859	2.705639	2.900109	2.697228	2.704502	2.607029	2.697004	2.701878	2.693923	2.676443	2.514771	2.700979	3.030407
CCDC11	1552325_at	4.092809	3.67682	3.625962	3.824638	4.309889	3.686231	3.757346	3.781822	3.876515	3.818645	4.110361	4.20516	3.237205
CCDC11	1552326_a_at	2.025134	2.006158	1.979583	1.986988	2.044009	1.987161	1.994762	2.003938	1.988381	1.981344	1.982951	1.998423	1.926584
ARMCX4	1552327_at	3.128513	3.133194	4.490797	3.026923	3.562586	3.024694	2.891804	2.988889	2.992423	2.958964	2.946694	2.897039	4.932341
RBBP6	1552329_at	4.631909	5.254656	5.289778	5.473943	4.808127	4.416003	5.592958	5.084325	5.795364	5.132678	4.795202	4.852669	5.763172
MGC16385	1552330_at	5.89757	5.710619	5.545529	5.83564	5.099472	4.993364	5.414574	5.447096	5.366699	5.441904	5.460293	5.533771	4.525276
TRIOBP	1552332_at	3.879813	3.875401	3.482897	3.83615	3.865125	3.86295	3.87139	3.898443	3.867349	3.850672	3.849432	4.769087	3.538199
TRIOBP	1552334_at	3.036616	2.692133	3.172786	2.667219	2.602936	2.589362	4.489274	2.688982	4.137228	4.38066	2.764677	2.747683	4.118653
CATSPER1	1552335_at	2.052245	2.033309	2.010061	2.014982	2.031803	2.014823	2.026862	2.03229	2.018442	2.010096	2.015129	2.026412	1.957449
HOXD4	1552337_s_at	2.251885	2.228155	2.198752	2.207223	2.230287	2.210773	2.21632	2.227947	2.210631	2.204418	2.213671	2.221657	2.501486
GSC	1552338_at	1.814102	1.805243	1.784641	1.788595	1.799488	1.781684	1.800096	1.799805	1.797912	1.792317	1.788894	1.794705	1.748411
SP7	1552340_at	2.082293	2.069995	2.049815	2.054497	2.071458	2.056439	2.064807	2.067928	2.05664	2.054922	2.049715	2.061141	3.361165
PDE7A	1552343_s_at	3.971467	4.055279	3.776663	4.187819	4.039416	4.327504	4.229866	4.8522	4.4551	5.443804	4.477739	4.93728	4.806897
CNOT7	1552344_s_at	2.695882	2.671153	2.768459	2.788138	2.678286	2.520805	3.720962	2.788794	4.122343	3.634139	2.784847	2.863905	5.548625
CRYZL1	1552347_at	6.814029	6.958916	6.117823	6.290143	6.796779	6.914597	6.464028	6.352558	6.409763	6.276792	7.018586	6.687871	7.194506
PRSS33	1552348_at	2.871735	2.849754	2.8053	2.835956	2.85695	2.839106	2.845483	2.850336	2.842009	2.834646	2.873256	2.98919	2.700926

PRSS33	1552349_a_at	2.413565	2.333922	2.228179	2.296301	2.312382	2.275473	2.287875	2.31459	2.308554	2.301908	2.291715	2.551643	2.232314
C19orf26	1552354_at	2.626187	2.566337	2.559813	2.555424	2.605773	2.595275	2.570544	2.576534	2.567749	2.549757	2.563115	2.599706	2.449519
C19orf26	1552355_s_at	2.281231	2.270816	2.247288	2.251198	2.281555	2.254602	2.260046	2.274712	2.266993	2.261627	2.257401	2.265456	2.157938
C8orf45	1552359_at	2.751904	2.700324	2.653263	2.76243	2.753056	2.668076	2.702509	2.712344	2.699676	2.676502	2.733846	2.732249	2.501774
TIRAP	1552360_a_at	3.831681	4.02572	4.050287	3.867948	3.476795	3.311819	3.383778	3.567569	3.356315	3.497233	3.360592	3.372588	3.092193
LEAP2	1552362_a_at	13.57144	13.62136	12.95304	12.94388	12.52808	12.37342	9.269309	8.702081	9.722087	10.45866	9.05251	9.108222	5.19634
MSI2	1552364_s_at	2.209888	2.192568	2.170602	2.174959	2.197794	2.18233	2.383524	2.768204	2.348263	2.20629	2.185743	2.216224	2.164169
SCIN	1552365_at	2.858268	2.801024	2.750077	2.791912	2.849731	3.04464	2.809394	2.809951	2.80001	2.779808	2.806389	2.847069	3.714214
SCIN	1552367_a_at	3.097772	3.022401	2.907286	2.970718	3.090723	2.986811	3.006505	3.038153	3.004453	2.950877	3.02362	3.059223	2.912
CTCFL	1552368_at	2.806738	2.70076	2.644421	2.705566	2.776767	2.684341	2.707436	2.71652	2.710924	2.680011	2.7023	2.756733	2.511047
C4orf33	1552370_at	3.121645	3.018962	4.191493	4.074757	3.084781	2.90471	4.475562	2.926133	4.487199	5.517396	3.071059	3.356313	3.781887
C4orf33	1552372_at	3.171524	3.054999	3.026736	3.070115	3.178475	3.046005	3.042517	3.078437	3.07934	3.058075	3.084282	3.158093	2.764374
C4orf33	1552373_s_at	2.978593	2.88276	2.805701	2.880236	2.993756	2.826005	2.88245	2.903515	2.891067	2.872281	2.982763	3.103038	2.658311
ZNF333	1552375_at	2.978963	2.943839	2.897268	2.876697	2.963455	3.045918	2.938425	2.949081	2.933471	2.925949	2.934971	2.943088	3.396268
FAM18B2	1552377_s_at	3.336932	3.331091	3.304722	3.26279	3.422523	3.318235	3.327229	3.306104	3.317367	3.262354	3.406834	3.323346	3.050804
RDH10	1552378_s_at	4.472622	4.44542	4.615691	5.166403	4.674087	4.866141	4.101158	4.232089	4.306779	4.336995	4.407384	4.290482	4.128223
RDH10	1552379_at	3.137253	3.052725	2.959079	3.018594	3.151312	2.963709	3.02692	3.054397	3.040193	3.014957	3.0708	3.103843	2.710342
SRrp35	1552381_at	2.740398	2.691843	2.666359	2.684224	2.74953	2.653546	2.701916	2.703762	2.697606	2.689312	2.700619	2.707058	3.47988
FAM71A	1552383_at	3.734012	4.024171	3.779436	4.061371	4.017532	4.51798	3.664983	4.301899	3.650697	3.861244	3.782515	4.522445	3.149195
FAM71A	1552384_a_at	2.395232	2.373652	2.333193	2.357125	2.381702	2.356284	2.355931	2.36809	2.351729	2.339228	2.352764	2.361423	2.284386
C5orf29	1552386_at	2.474902	2.442573	2.432759	2.436482	2.559184	2.435724	2.448586	2.447432	2.435596	2.427659	2.43575	2.445177	2.33228
FLJ30901	1552388_at	2.109258	2.092155	2.08165	2.073923	2.082143	2.082695	2.09688	2.094601	2.085458	2.223415	2.07854	2.087978	2.085491
C8orf47	1552389_at	6.559434	6.679851	4.266065	3.898081	4.252178	4.188891	4.481157	4.430508	5.675076	5.366399	4.121799	4.142495	2.784653
C8orf47	1552390_a_at	8.473712	8.649178	5.378422	5.819252	6.242148	6.138623	5.763305	7.050815	6.93386	7.31599	5.437751	5.8947	2.75176
C1orf65	1552391_at	3.576576	3.542781	3.491701	3.547793	3.55547	3.521898	3.531756	3.541019	3.527662	3.516236	3.487146	3.538536	3.26175
ENTHD1	1552393_at	3.449966	3.345098	3.214825	3.31001	3.462043	3.276792	3.295543	3.31375	3.328355	3.296763	3.344747	3.422265	2.881443
ENTHD1	1552394_a_at	2.934892	2.834124	2.795339	2.827768	2.938586	2.814559	2.852969	2.854746	2.847117	2.810738	2.856132	2.889998	2.608681
TSSK3	1552395_at	2.133453	2.129431	2.107257	2.1119	2.124988	2.1114	2.123161	2.114776	2.114889	2.110061	2.105826	2.121281	2.071112
WFDC6	1552396_at	2.957695	2.950912	2.928685	2.936077	2.96262	3.021709	2.93341	2.944334	2.860931	2.927699	2.929278	2.950302	2.879693
CLEC12A	1552398_a_at	2.843091	3.048666	3.330972	2.778968	2.95191	2.928087	2.581819	2.54651	2.656154	2.539298	2.678815	2.807244	2.173038
BRF1	1552399_a_at	2.883669	2.843514	2.803513	2.834437	2.874643	2.810675	2.848112	2.854235	2.841644	2.834549	2.860975	2.902095	3.619657
C15orf27	1552400_a_at	2.420613	2.400084	2.379087	2.385724	2.406037	2.509246	2.40138	2.401696	2.383179	2.375633	2.387369	2.518502	2.325269
CALML6	1552402_at	2.882967	2.870643	2.7856	2.855542	2.886813	2.838522	2.867823	2.869759	2.86347	2.850677	2.851639	2.861053	2.805922
NLRP5	1552405_at	2.143493	2.124995	2.100247	2.106205	2.128731	2.1041	2.114334	2.12057	2.108073	2.100815	2.104101	2.117509	2.031666
ODF4	1552408_at	2.501826	2.467791	2.417173	2.447866	2.490694	2.440061	2.455893	2.467881	2.45278	2.431952	2.451315	2.464686	2.3138
ODF4	1552409_a_at	2.683616	2.664289	2.643975	2.647852	2.661183	2.82734	2.655804	2.660191	2.613618	2.626819	2.640817	2.655335	2.622706
CLEC4F	1552410_at	2.239619	2.230227	2.196394	2.209465	2.272904	2.216323	2.218414	2.226405	2.212814	2.211188	2.210751	2.219786	2.120665
DEFB106A	1552411_at	0.493656	0.489365	0.469974	0.475442	0.482079	0.474951	0.493536	0.474674	0.484988	0.473768	0.467701	0.472123	0.492

DEFB106A	1552412_a_at	2.774565	2.72694	2.685618	2.725109	2.761928	2.709434	2.733884	2.742098	2.728794	2.719892	2.731049	2.738618	2.578339
WFDC9	1552414_at	2.700882	2.676742	2.618158	2.654621	2.69008	2.638452	2.721833	2.673934	2.661081	2.646525	2.679923	2.66929	2.527106
WFDC9	1552415_a_at	2.193464	2.186522	2.155307	2.170595	2.186804	2.174472	2.181413	2.183646	2.170488	2.162503	2.168868	2.175619	2.037679
NEDD1	1552417_a_at	3.216065	3.044174	3.190299	3.074166	3.329767	3.052603	4.417702	3.201544	4.548755	4.942972	3.336752	3.192248	5.535411
TTLL10	1552418_at	1.657465	1.647008	1.617784	1.635979	1.660619	1.624678	1.64806	1.64636	1.634714	1.632822	1.628926	1.64411	1.61115
TTLL10	1552419_s_at	1.927537	1.91969	1.898926	1.902524	1.916605	1.908973	1.914315	1.911694	1.900331	1.901175	1.903475	1.910236	1.871264
CALR3	1552421_a_at	2.559532	2.531338	2.499562	2.512207	2.546215	2.515958	2.521852	2.529902	2.517498	2.502254	2.51102	2.526517	2.362016
C10orf25	1552422_at	6.221738	4.826867	5.200593	5.466326	6.417963	6.545155	4.059898	4.41683	4.490228	4.4181	4.76037	4.572841	4.254008
ETV3	1552423_at	2.763423	2.712447	3.188522	2.6907	2.721735	2.679619	2.772696	2.709653	2.696427	2.682971	2.691842	2.704405	3.406494
KLHL10	1552424_at	2.691843	2.61546	2.562294	2.608888	2.701014	2.585773	2.621809	2.631027	2.616278	2.592349	2.613491	2.648043	2.436117
KLHL10	1552425_a_at	2.453045	2.44181	2.400114	2.407121	2.432903	2.404699	2.419219	2.422339	2.408141	2.399249	2.404873	2.418656	2.309689
TM2D3	1552426_a_at	9.754925	9.919562	10.05045	10.18831	9.608026	9.530535	8.817303	8.967373	8.816745	9.110132	8.82787	8.877844	9.621251
ZNF485	1552427_at	4.622256	4.228536	3.996169	4.251029	4.951554	4.170496	4.266672	4.526249	4.36133	4.229862	4.364385	4.576357	4.243933
WDR17	1552430_at	3.492759	3.288117	3.265328	3.320999	3.542119	3.3008	3.295017	3.307444	3.320353	3.260115	3.345061	3.386042	3.370858
FLJ35773	1552432_at	3.910178	3.906198	3.885372	3.889966	3.917872	3.893255	3.725692	3.896975	3.825491	3.887777	3.87951	3.894096	3.862066
CDH23	1552436_a_at	1.863299	1.854199	1.833188	1.836339	1.852042	1.836987	1.848744	1.85005	1.839047	1.832847	1.831057	1.843461	1.796392
ANKAR	1552438_a_at	2.569065	2.518875	2.475655	2.508043	2.557091	2.517565	2.523421	2.530008	2.520367	2.497706	2.518383	2.552302	2.3646
MEGF11	1552439_s_at	2.888754	2.81663	2.771028	2.795748	2.869823	2.784637	2.809448	2.812995	2.799283	2.77487	2.80582	2.839561	2.756738
ADMR	1552440_at	6.631553	6.431029	6.723073	6.786276	5.973819	5.866644	6.317674	7.140722	6.388557	6.235333	7.063448	7.416267	3.590697
ESX1	1552445_a_at	3.275003	3.242993	3.137309	3.23407	3.288306	3.157507	3.222398	3.313135	3.247875	3.244264	3.264003	3.323253	3.010657
C8orf12	1552448_a_at	2.469596	2.43616	2.417635	2.422228	2.459178	2.421412	2.432898	2.435121	2.425065	2.417264	2.420118	2.435567	2.362121
SCGB1C1	1552449_a_at	2.922202	2.89418	2.83638	2.871378	2.948901	2.866864	2.877167	2.87994	2.873064	2.856998	2.873677	2.919605	2.609418
DNAJC5G	1552450_a_at	2.163513	2.153715	2.135808	2.138907	2.15354	2.137099	2.151292	2.146914	2.146713	2.136723	2.133348	2.141688	2.117638
WDR88	1552452_at	2.36152	2.335639	2.308858	2.316848	2.443861	2.319873	2.329376	2.334823	2.331157	2.310001	2.325752	2.334583	2.271433
WDR88	1552453_a_at	2.289677	2.256217	2.237668	2.241352	2.266776	2.240548	2.252853	2.256146	2.24354	2.23648	2.238115	2.251387	2.16276
PRUNE2	1552455_at	2.321668	2.297015	2.266681	2.274567	2.306648	2.274042	2.285464	2.292292	2.278852	2.267998	2.282154	2.288772	2.237089
MBD3L2	1552456_a_at	2.228496	2.213233	2.180553	2.195318	2.21202	2.195935	2.207795	2.213378	2.198685	2.188773	2.190611	2.205219	2.042058
ADAMTSL1	1552457_a_at	2.364497	2.334725	2.302823	2.315197	2.345972	2.311033	2.322835	2.334788	2.319105	2.307951	2.32052	2.328977	2.215619
MBD3L1	1552458_at	3.046299	2.960602	2.879862	2.920618	3.050694	2.912314	2.955578	2.986455	2.955458	2.938627	2.970639	3.018287	2.695088
MBD3L1	1552459_a_at	2.096346	2.261808	2.053974	2.115468	2.082145	2.057344	2.064276	2.072697	2.058931	2.053655	2.060548	2.067522	1.990877
FAM46D	1552461_at	2.293935	2.25211	2.225687	2.245165	2.271495	2.237144	2.249725	2.257248	2.245445	2.235367	2.240595	2.254799	2.142221
SERPINB11	1552463_at	2.347227	2.315839	2.275905	2.295881	2.335522	2.295225	2.30469	2.314866	2.299891	2.288018	2.298004	2.332596	2.189567
C21orf100	1552466_x_at	3.412785	3.256313	3.144413	3.246969	3.571874	3.1647	3.26327	3.251789	3.276038	3.228336	3.342352	3.369701	2.864853
DSCR10	1552467_at	3.109958	3.044397	3.139278	3.036213	3.118282	3.817676	3.018622	3.0281	2.647834	2.999813	2.974166	3.056287	2.843767
DSCR10	1552468_a_at	2.250796	2.228227	2.185995	2.213132	2.233103	2.216043	2.211948	2.22289	2.209255	2.196017	2.208575	2.220828	2.116718
ABHD11	1552470_a_at	2.084329	2.06271	2.040212	2.046749	2.072404	2.04522	2.059841	2.055212	2.049218	2.049426	2.04757	2.053824	2.033064
CENTB2	1552472_a_at	2.941114	2.863134	2.773454	2.912644	2.965647	2.89863	2.932821	3.016865	3.081925	3.011458	2.901416	3.492298	2.994478
GAMT	1552473_at	8.03503	8.095207	8.516389	8.264438	8.970476	9.333023	6.412097	7.782188	6.329832	6.318343	7.526567	7.395057	5.110621

GAMT	1552474_a_at	9.585478	9.624938	9.879897	9.758345	10.2742	9.723382	8.966777	8.927146	9.223819	9.132119	8.687089	8.9876	6.119525
PLCD3	1552476_s_at	2.261609	2.191378	2.201424	2.121533	2.232934	2.94114	2.223748	2.27168	2.188071	2.21039	2.226144	2.229235	2.175457
IRF6	1552477_a_at	5.143983	4.617227	5.507887	5.533816	6.061011	4.676368	2.262243	2.272075	2.269906	2.245924	2.275529	2.26721	3.123142
IRF6	1552478_a_at	2.799835	2.783365	2.765353	2.763016	2.802316	2.767239	2.779671	2.781066	2.765987	2.761849	2.763793	2.776043	2.717674
PTPRC	1552480_s_at	3.320332	3.176326	3.064022	3.164459	3.441103	3.14262	3.159166	3.178228	3.187542	3.136046	3.223821	3.340058	2.796482
MAN1A2	1552481_s_at	2.414015	2.368284	2.309888	2.333746	2.393386	2.332678	2.336186	2.3459	2.348165	2.33701	2.328135	2.362146	2.256314
RAPH1	1552482_at	10.94326	11.0594	10.22083	10.43428	10.33531	10.35511	7.275156	8.306594	6.892358	7.667543	8.133035	7.669363	5.257125
SMCR8	1552484_at	2.128334	2.109315	2.081935	2.089968	2.106028	2.089872	2.102525	2.107931	2.100787	2.088424	2.087635	2.097633	2.014966
LACTB	1552485_at	3.114836	2.728542	4.470496	3.459016	2.528088	3.03786	2.977662	3.272379	2.740732	3.11572	2.499131	2.604759	3.770904
LACTB	1552486_s_at	4.908352	4.825705	6.32328	5.400952	4.728266	4.021703	5.433914	4.347041	5.878022	5.613009	3.242899	3.950383	4.665933
BNC1	1552487_a_at	2.840036	2.744911	2.687799	2.747394	2.841457	2.783531	2.780385	2.76394	2.776866	2.793967	3.60635	2.963606	3.654417
MPP4	1552489_s_at	2.78379	2.736324	2.706214	2.725524	2.777315	2.696007	2.739425	2.724921	2.737252	2.714477	2.74166	2.754864	2.522148
LACE1	1552490_at	2.631143	2.597897	2.585616	2.588871	2.653418	2.593283	2.596981	2.65182	2.59456	2.559595	2.577082	2.857163	2.539588
IDI2	1552491_at	2.018287	2.005461	1.988638	1.989645	2.004802	1.987661	2.000227	2.001515	1.991472	1.985956	1.98659	1.996237	1.92642
CYP11B1	1552493_s_at	2.455135	2.418896	2.37518	2.402613	2.443289	2.392935	2.410583	2.422412	2.406825	2.393437	2.405933	2.41765	4.186027
TAF8	1552494_at	2.074164	2.056002	2.034776	2.036909	2.064178	2.03925	2.050607	2.053865	2.041702	2.033561	2.035346	2.047315	1.972048
COBL	1552496_a_at	5.356065	5.199409	5.30248	5.336151	5.243267	5.49013	4.021716	3.514712	4.029059	3.93298	2.814394	3.529586	2.569569
SLAMF6	1552497_a_at	1.84423	1.791881	1.913927	1.822544	1.902399	2.437632	1.709613	1.825803	1.791317	1.693383	1.815462	1.823793	1.709613
ZSCAN20	1552498_at	2.257471	2.236499	2.213048	2.219588	2.246871	2.21807	2.228869	2.237112	2.222048	2.215303	2.221694	2.23373	2.136411
ZSCAN20	1552499_a_at	2.307162	2.28115	2.259542	2.258978	2.300856	2.262717	2.273962	2.279241	2.295432	2.252482	2.268382	2.292213	2.228052
GPBAR1	1552501_a_at	2.652974	2.629972	2.603554	2.625884	2.643939	3.202758	2.628136	3.357868	2.621848	2.614777	2.887548	3.531688	2.470535
RHBDL2	1552502_s_at	2.520299	2.48785	2.437428	2.466916	2.512875	2.470963	2.476203	2.486911	2.471967	2.465801	2.469583	2.48406	2.404243
FRAS1	1552503_at	2.0203	2.006197	1.982967	1.987346	1.99752	1.986552	1.999156	2.001027	1.98997	1.982228	1.986984	1.996944	1.958656
BRSK1	1552504_a_at	3.012223	3.000958	2.947782	2.986323	3.000318	4.160403	2.965511	2.99793	2.971809	2.973026	3.099543	2.991296	2.914731
CRB2	1552506_at	1.918574	1.910492	1.890364	1.892315	1.908085	1.899653	1.905487	1.906224	1.894552	1.887506	1.893281	1.904096	1.861174
KCNE4	1552507_at	2.229127	2.208972	2.191534	2.19119	2.225722	2.196584	2.199411	2.207221	2.196182	2.184702	2.193338	2.200012	2.259011
KCNE4	1552508_at	2.243676	2.224002	2.198379	2.201507	2.22743	2.212803	2.214671	2.224453	2.210122	2.198517	2.207744	2.219997	2.685697
CD300LG	1552509_a_at	2.560453	2.515598	2.508197	2.524673	2.561849	2.513944	2.517589	2.514653	2.509355	2.490064	2.50462	2.514261	2.446627
SLC34A3	1552510_at	2.274791	2.269052	2.246318	2.185468	2.265569	2.258288	2.265793	2.261636	2.251371	2.245554	2.239451	2.307996	2.202261
CPA6	1552511_a_at	2.597879	2.551559	2.504365	2.540913	2.58726	2.536295	2.548778	2.554347	2.541972	2.521095	2.541774	2.562605	2.465205
WBP2NL	1552514_at	2.494807	2.458861	2.40702	2.439081	2.548691	2.43838	2.446521	2.456209	2.442137	2.432606	2.445191	2.453568	2.522672
HIPK1	1552515_at	2.349391	2.330318	2.303246	2.312985	2.346225	2.300876	2.32734	2.332948	2.318532	2.315104	2.316495	2.325765	2.255179
HIPK1	1552516_a_at	2.090847	2.057786	2.0618	2.061463	2.077703	2.06094	2.731999	2.066341	2.289372	2.974563	2.063552	2.073769	3.801804
MTBP	1552518_s_at	3.363441	3.212081	3.065923	3.22505	3.40709	3.163302	3.229906	3.277292	3.312226	3.204202	3.278188	3.333893	3.625934
ACVR1C	1552519_at	3.569276	4.323602	4.711766	5.071277	4.222321	4.011237	2.93219	2.934737	2.926284	2.919639	2.916551	2.92394	2.434463
TMEM74	1552520_at	1.981549	1.965498	1.945266	1.948193	2.035882	1.947581	1.960122	1.96222	1.9503	1.942962	1.957148	1.956939	2.191232
TMEM74	1552521_a_at	3.413403	3.077881	2.954111	3.062761	3.122911	2.974407	3.035121	3.014839	3.036401	3.004847	3.058584	3.099075	3.011904
TIGD4	1552522_at	3.255634	3.09805	3.022618	3.104195	3.263905	3.042991	3.111175	3.115798	3.131295	3.082286	3.159857	3.206866	2.788187

TIGD4	1552523_a_at	2.884969	2.810045	2.787127	2.736473	2.873731	2.798366	2.818522	2.82907	2.812389	2.789334	2.813357	2.846781	2.77365
ART5	1552524_at	2.296287	2.292727	2.27097	2.272844	2.283105	2.267287	2.43174	2.292454	2.277078	2.275971	2.266113	2.39268	2.252955
FAM71C	1552526_at	2.150651	2.139729	2.123474	2.125396	2.139732	2.12554	2.133084	2.137606	2.126685	2.119062	2.121266	2.131393	2.079569
C21orf67	1552528_at	2.05554	2.047126	2.027158	2.03434	2.040802	2.03505	2.044448	2.04289	2.043421	2.031806	2.027977	2.038709	1.989064
NLRP11	1552531_a_at	2.364152	2.350917	2.694502	2.354164	2.456324	2.334058	2.334837	2.345143	2.330026	2.318326	2.328068	2.339375	2.222664
ATP6V1C2	1552532_a_at	2.933657	2.899247	2.806499	2.875494	2.967916	2.827702	2.887809	2.890513	2.884383	2.852164	2.894598	2.931126	2.618822
CLDN19	1552535_at	2.37132	2.3616	2.333451	2.339993	2.36025	2.35169	3.099196	2.958309	2.41221	3.011637	2.969271	2.940065	2.300477
VTI1A	1552536_at	5.364804	4.718797	3.632516	4.292795	5.513575	5.899969	4.80336	5.776128	4.563623	4.568025	5.698232	5.471312	3.238162
KIF6	1552538_a_at	3.057864	2.993827	3.001539	3.025602	3.03732	2.939305	4.052424	3.952581	3.71429	3.927613	3.237955	3.29284	2.744145
IQCD	1552540_s_at	2.193239	2.170966	2.145284	2.152004	2.225207	2.154126	2.16496	2.170192	2.426401	2.148172	2.152948	2.172462	2.109348
TAGAP	1552541_at	4.188454	4.094367	4.198687	4.209273	4.178096	4.097749	4.019169	4.150828	4.074107	4.238856	4.138132	4.279691	3.869661
TAGAP	1552542_s_at	2.146895	2.131529	2.097781	2.116944	2.139706	2.120755	2.119856	2.129857	2.10606	2.112365	2.117461	2.123828	2.036567
STON2	1552543_a_at	2.058722	2.026449	2.008812	2.009812	2.036576	2.014562	2.024087	2.030937	2.023237	2.011057	2.018006	2.022914	1.972981
SERPINA12	1552544_at	2.779726	2.639764	3.382803	3.372086	2.646145	2.589226	2.62722	2.65602	2.654574	2.614073	2.620002	2.681039	2.465153
LETM2	1552546_a_at	2.412177	2.386675	2.357608	2.366949	2.393399	2.36587	2.371446	2.38217	2.368139	2.360275	2.369523	2.37891	2.242273
BSND	1552548_at	2.286909	2.257991	2.216031	2.239834	2.284621	2.237317	2.249416	2.259587	2.244613	2.237962	2.24273	2.254526	2.160372
BSND	1552549_a_at	2.038946	2.026112	2.009647	2.003781	2.037869	2.006436	2.028088	2.11954	2.022005	2.002335	2.007582	2.046243	1.946699
CLEC4C	1552552_s_at	2.597392	2.556478	2.528941	2.537809	2.580585	2.539207	2.555362	2.563523	2.547812	2.539119	2.553172	2.567732	2.388748
NLRC4	1552553_a_at	2.42894	2.386989	2.410604	2.416269	2.45297	2.414993	2.424161	2.43564	2.420714	2.406989	2.372922	2.427711	2.287337
NLRC4	1552554_a_at	2.470618	2.442674	2.411927	2.427752	2.448833	2.426395	2.437794	2.44315	2.428487	2.422475	2.431228	2.476257	2.337269
PRSS36	1552555_at	2.692288	2.686237	2.623278	2.641382	2.672567	2.629708	2.649609	2.661701	2.643908	2.634924	2.771645	2.653254	2.52318
ZDHHC15	1552557_a_at	2.88662	2.832302	2.780115	2.801264	2.877535	2.791168	2.821182	2.989651	2.816758	2.799188	2.831972	2.843292	3.514968
RAI1	1552558_a_at	1.914971	1.877378	1.8556	1.860716	1.87169	1.86602	1.865381	1.876306	1.86218	1.856545	2.097084	1.867723	1.81067
ALS2CR7	1552559_a_at	2.07359	2.039365	1.987641	2.028967	2.135655	2.035389	2.051121	2.050868	2.033924	2.017827	2.037241	2.06475	1.984511
ZNF570	1552562_at	1.877716	1.86329	1.845598	1.849109	1.863224	1.852076	1.862573	1.861928	1.856192	1.848014	1.847128	1.856131	1.815261
C8orf6	1552563_a_at	2.397894	2.371907	2.350214	2.356642	2.406207	2.354284	2.368634	2.369396	2.357773	2.353024	2.753259	2.382501	3.22049
NUDT9P1	1552564_at	2.885372	2.846605	2.829152	2.823087	2.874003	2.78714	2.830658	2.844686	2.828917	2.812115	2.826251	2.844864	2.618438
BTBD16	1552566_at	1.946147	1.933226	1.919602	1.929578	2.039577	1.922902	1.928033	1.925788	1.915866	1.915686	1.911077	1.910996	1.863654
RTP3	1552568_at	7.168434	7.022852	7.286771	7.296779	6.218102	5.741471	3.963403	2.81226	3.951387	3.867453	2.940574	3.396671	2.533462
RTP3	1552569_a_at	8.8792	8.945481	8.323587	8.417991	8.468471	8.336947	3.013546	2.990546	2.091286	2.083683	2.700509	2.966852	2.809698
C21orf29	1552570_at	2.77639	2.687925	2.714882	2.730646	2.828108	2.674528	2.707922	2.967028	2.734834	2.687582	2.873239	2.874699	2.458674
MIPOL1	1552572_a_at	3.27392	3.152993	3.055821	3.131944	3.282391	3.06778	3.204438	3.151582	3.157181	3.105458	3.184636	3.199588	2.7966
MIPOL1	1552573_s_at	2.999627	2.925327	2.869154	2.910819	3.006646	2.909336	2.912571	2.920308	2.913252	2.881809	2.918599	2.937356	2.661041
C6orf141	1552575_a_at	2.851923	2.815182	2.809758	2.812526	3.249874	2.952124	2.818863	2.809608	2.820627	2.798187	2.800669	2.816398	2.737703
ALMS1L	1552576_at	2.540246	2.891894	2.46233	2.468907	3.710269	3.677934	2.462288	2.474225	2.469679	2.438534	2.538946	2.489732	2.306357
MYO3B	1552578_a_at	2.567959	2.471378	2.40529	2.480226	2.523459	2.470198	2.488679	2.500888	2.486076	2.469678	2.497849	2.498652	2.390675
ADAM21	1552579_a_at	2.310885	2.304234	2.278862	2.27952	2.322953	2.53324	2.290629	2.301707	2.287366	2.273462	2.285915	2.331103	2.220479
FLJ25801	1552580_at	2.552798	2.530395	2.51841	2.522314	2.54461	2.513682	2.540652	2.542253	2.53384	2.635878	2.535548	2.756902	2.473913

ABCC13	1552582_at	2.861072	2.819138	2.738433	2.805845	2.820432	2.798494	7.584961	9.136591	8.443028	7.230982	7.561756	8.153773	2.631133
ABCC13	1552583_s_at	1.796353	1.796163	1.745949	1.779326	1.78791	1.765846	6.750113	5.227405	6.889093	6.847298	2.844281	4.036723	1.732417
IL12RB1	1552584_at	4.425476	4.47834	4.34868	4.369444	4.865413	5.849357	4.251911	4.252599	4.243739	4.099598	4.364682	4.371463	4.205954
GTF2A1L	1552585_s_at	2.471124	2.374271	2.301941	2.344936	2.486504	2.346718	2.325703	2.363488	2.354281	2.345542	2.357597	2.377824	2.290257
TRPV3	1552586_at	2.642423	2.621901	2.600713	2.611417	2.623794	2.610279	2.616462	2.622382	2.613004	2.59924	2.603688	2.615787	2.530231
CNBD1	1552587_at	3.169433	2.998137	2.874057	3.011256	3.206881	2.944798	3.007726	3.158717	3.019755	3.019701	3.13058	3.150703	2.71562
CNBD1	1552588_a_at	2.143344	2.120284	2.095687	2.103784	2.12842	2.102168	2.114288	2.119912	2.113162	2.098388	2.104115	2.122044	2.040939
ABCC12	1552590_a_at	2.108419	2.085639	2.058754	2.068008	2.090554	2.068407	2.076309	2.084834	2.071442	2.063667	2.067608	2.080683	1.994299
MMP21	1552592_at	2.416068	2.314272	2.296597	2.313329	2.43478	2.326975	2.340822	2.328819	2.33087	2.321538	2.348359	2.484618	2.216962
TMEM190	1552594_at	1.73673	1.727934	1.703996	1.715342	1.718659	1.711617	1.728435	1.712097	1.723049	1.71274	1.707726	1.709872	1.678451
GAS2L2	1552596_at	2.274623	2.26194	2.241629	2.245413	2.267589	2.251099	2.245834	2.257836	2.241624	2.231521	2.239686	2.251056	2.060998
KCNG4	1552598_at	1.867753	1.856422	1.83753	1.841922	1.856105	1.839558	1.852261	1.853458	1.842712	1.838259	1.83666	1.850763	1.799347
PXT1	1552599_at	1.708108	1.700283	1.679705	1.684835	1.698834	1.685481	1.694642	1.696244	1.685246	1.681319	1.681783	1.69006	1.647542
CACNG5	1552602_at	2.90508	2.84988	2.591946	2.696734	2.84702	2.794606	2.839047	2.848363	2.837078	2.83389	2.834582	2.910759	2.573262
C21orf74	1552604_at	2.689657	2.633036	2.609514	2.627744	2.686882	2.636823	2.639364	2.647666	2.648093	2.632296	2.635852	2.655833	2.453095
C21orf74	1552605_s_at	2.555944	2.519358	2.460208	2.501556	2.544494	2.487929	2.51644	2.526355	2.5111	2.503937	2.504511	2.532246	2.355331
CXorf52	1552607_at	4.963211	4.532658	3.865678	4.189469	4.409311	5.107931	3.705736	4.213773	3.459646	4.445748	3.968112	4.037183	3.215375
WFDC11	1552608_at	2.213937	2.197234	2.176492	2.160892	2.198176	2.180562	2.192232	2.194138	2.182664	2.175378	2.177603	2.190373	2.12586
IL28A	1552609_s_at	2.601164	2.564812	2.523473	2.540124	2.589191	2.532978	2.546438	2.561529	2.544012	2.541347	2.54867	2.565566	2.412293
JAK1	1552610_a_at	3.968662	4.061005	6.939662	5.269045	3.609938	3.996098	4.761999	3.040984	5.246506	5.387848	3.296702	3.563321	8.120509
JAK1	1552611_a_at	6.392682	5.743007	6.094088	6.596439	6.562812	6.796468	4.474453	5.072645	3.136258	4.561051	5.746453	5.465365	5.069957
CDC42SE2	1552612_at	1.785982	1.76723	1.758543	1.754031	1.766775	1.764106	2.210888	1.765649	2.043364	2.401407	1.756147	1.758723	4.381827
CDC42SE2	1552613_s_at	2.962879	2.877536	2.990034	2.921486	2.800368	2.755525	2.985813	2.899293	3.320737	2.923177	2.899993	2.939005	4.121995
ACACB	1552615_at	5.367532	5.197304	6.18584	6.248537	4.763943	4.739596	3.972347	4.677537	3.352418	3.346796	3.089361	3.931225	3.669473
ACACB	1552616_a_at	3.374053	3.222282	4.715483	4.757207	2.414665	3.263262	2.079371	2.087353	1.946502	2.064913	2.113947	2.082052	1.853389
RFWD2	1552617_a_at	5.296295	4.813719	5.839135	5.292799	4.41291	3.72553	6.565105	4.674364	5.934395	6.530217	4.600049	4.851507	6.763
STX6	1552618_at	2.905346	2.895828	2.651004	2.662381	2.717605	2.801586	3.207187	3.719818	3.503522	3.0948	3.570798	3.96203	4.568384
ANLN	1552619_a_at	2.732154	2.702447	2.620829	2.682749	2.702799	2.654671	6.872509	4.249237	7.190362	6.986842	3.15122	3.808415	7.716056
SPRR4	1552620_at	1.811605	1.80519	1.788214	1.790518	1.796332	1.787282	1.802417	1.803236	1.794051	1.789367	1.787064	1.796982	1.805385
POLR2J2	1552621_at	7.325438	6.997493	8.275921	7.957833	8.350324	8.432889	8.516645	8.009983	9.026527	8.787826	8.756402	8.397567	7.729548
LOC441259	1552622_s_at	5.968655	6.11346	7.312926	6.897944	6.918419	6.238279	7.130728	6.765268	7.851072	7.455709	7.132871	6.892999	7.231072
HSH2D	1552623_at	2.84173	2.810456	2.779586	2.790763	2.830513	3.013099	2.798511	2.80804	2.765086	2.762543	2.791606	2.803682	2.540656
TRNT1	1552625_a_at	3.527474	2.915001	3.50734	3.664303	2.414851	3.471111	4.138125	3.486048	4.51687	4.961939	3.54839	3.561297	5.681022
TMEM163	1552626_a_at	2.934643	2.922912	2.800845	2.888215	2.91936	2.901276	2.92188	2.936669	2.903584	3.081008	3.021682	2.911492	5.109858
ARHGAP5	1552627_a_at	2.839851	2.835406	3.868697	2.884096	2.891163	2.869148	3.428274	2.806424	2.902802	2.865776	2.816014	2.804284	5.96712
HERPUD2	1552628_a_at	9.621897	9.276828	9.810093	9.759961	9.350205	8.983934	9.298109	8.988884	9.298275	9.233893	9.190696	9.032574	8.746719
JMJD6	1552629_a_at	2.114372	2.102939	2.085081	2.087286	2.107199	2.085848	2.100552	2.10352	2.092145	2.088897	2.087786	2.098262	2.037382
SRCAP	1552630_a_at	2.507118	2.479036	2.430347	2.454738	2.470382	2.462582	2.825216	2.46886	2.703953	2.462168	2.456874	2.457476	2.465753

MAP3K6	1552631_a_at	2.489382	2.480597	2.43636	2.46568	2.482201	2.46416	2.469539	2.479638	2.476127	2.448273	2.455354	2.467868	2.410719
ARSG	1552632_a_at	2.796689	2.770511	2.746947	2.747885	2.862791	2.715599	2.764737	2.768261	2.743253	2.747138	2.750853	2.764074	2.614266
ZNF101	1552633_at	4.575856	4.843594	3.774051	4.145047	4.187817	4.849127	3.620561	4.734392	4.092614	3.17179	4.102886	4.082014	3.740256
ZNF101	1552634_a_at	3.64183	3.567277	3.527061	3.503903	3.720021	3.565735	3.787654	3.563426	3.91192	3.937749	3.619072	3.896324	3.202379
PTPN11	1552637_at	2.116753	2.094401	2.06558	2.074885	2.096636	2.07144	2.085661	2.092827	2.07809	2.070251	2.077729	2.087612	2.005397
KLHDC7B	1552639_at	2.120571	2.103461	2.082133	2.086128	2.103567	2.082056	2.101952	2.106976	2.099298	2.089578	2.086771	2.096819	2.020834
ATAD3A	1552641_s_at	3.535303	3.516191	3.480086	3.589657	3.511466	3.50694	3.804389	3.597475	4.970738	4.070404	3.954614	3.511471	3.608605
ZNF626	1552643_at	3.699054	3.514222	3.65463	3.662195	3.675954	3.670225	3.671067	3.94688	3.673795	3.66924	3.642665	3.667662	5.158657
PHC3	1552644_a_at	3.398264	3.308431	3.282893	3.25822	3.342478	3.280334	3.355653	3.304029	3.291418	3.029997	3.294266	3.325998	2.758503
IL11RA	1552646_at	3.095175	3.061366	3.051356	3.044287	3.164654	3.041756	3.051802	3.051897	2.504092	2.980728	3.036775	3.043596	4.274579
TNFRSF10A	1552648_a_at	2.435242	2.532568	2.400263	2.390834	2.410402	2.560999	2.374607	2.385179	2.367935	2.360114	2.367578	2.397559	2.260961
RFFL	1552649_a_at	2.335967	2.331647	2.300203	2.313937	2.326339	2.310304	2.32267	2.327049	2.320973	2.314465	2.313663	2.297947	2.230736
RFFL	1552651_a_at	3.39606	3.408894	3.20386	3.519107	3.543132	3.517012	3.53723	3.699894	3.619973	3.517848	3.506238	3.521539	3.18782
HPS4	1552652_at	3.179048	3.419845	3.090331	3.086438	3.104749	3.097701	3.116913	3.107813	3.044364	3.19062	3.106509	3.134615	2.788723
UHMK1	1552656_s_at	2.330487	2.312948	2.512738	2.295403	2.30466	2.330353	2.305763	2.280975	2.304171	2.260058	2.2879	2.277389	2.583188
TXNDC2	1552657_a_at	2.366468	2.341117	2.299445	2.318468	2.342937	2.313843	2.326425	2.337064	2.323695	2.317948	2.323942	2.332574	2.232606
NAV3	1552658_a_at	2.585589	2.561512	2.537947	2.543497	2.579437	2.512617	2.555345	2.560856	2.546719	2.52526	2.556962	2.556483	7.423302
C5orf22	1552660_a_at	2.576134	2.541172	2.708659	2.529107	2.577029	2.56021	3.691836	2.553584	2.918016	3.080809	2.533321	2.558476	5.459252
PCDHGB7	1552661_at	3.736399	3.478976	3.378867	3.524482	3.977813	3.453131	3.513574	3.4898	3.563857	3.482285	3.721574	3.810956	3.01478
PCDHGB7	1552662_a_at	2.123837	2.103425	2.085667	2.085427	2.096544	2.083844	2.098583	2.10056	2.090775	2.086477	2.084986	2.093246	2.066403
ERC1	1552663_a_at	2.534941	2.527356	2.505384	2.513261	2.521526	2.509775	2.521081	2.517594	2.503781	2.514273	2.501647	2.51371	4.4259
FLCN	1552664_at	6.150471	6.058132	5.492087	5.806591	6.180399	6.558671	6.28115	6.847725	5.917834	6.388979	6.817094	6.745135	4.593067
MGC14425	1552665_at	2.412404	2.398299	2.366505	2.391197	2.397961	2.393933	2.390369	2.475481	2.385702	2.39321	2.70961	2.55475	2.36617
LRRC7	1552666_a_at	2.613362	2.562013	2.521943	2.580581	2.601856	2.530337	2.562869	2.573235	2.559773	2.540585	2.556345	2.581131	2.897425
SH2D3C	1552667_a_at	2.247746	2.850229	2.208104	2.212577	2.22658	2.211354	2.223764	2.225303	2.214243	2.207057	2.210914	2.222915	2.738255
PPP1R3B	1552669_at	2.306242	2.277116	2.242802	2.26296	2.288333	2.256152	2.280597	2.282266	2.265778	2.258237	2.26365	2.276414	2.160936
LOC286044	1552670_a_at	6.806507	6.246122	7.755374	6.562282	3.022597	3.13646	3.015921	2.886581	3.320594	3.257892	2.895308	2.991046	5.417894
SLC9A7	1552671_a_at	2.273646	2.213581	2.254184	2.16441	2.224941	2.20803	2.210024	2.23706	2.201623	2.281293	2.199404	2.208038	2.137472
IGSF3	1552672_a_at	1.761854	1.75393	1.733305	1.737442	1.753819	1.73523	1.750635	1.752556	1.739491	1.737442	1.737465	1.745994	1.769278
RFXDC1	1552673_at	2.366888	2.333894	2.292394	2.322305	2.352165	2.324081	2.331184	2.343469	2.326888	2.349525	2.341613	2.446759	2.325849
DIRC1	1552674_at	3.31164	3.206408	3.099077	3.196866	3.289084	3.119473	3.23241	3.211138	3.206826	3.159791	3.245285	3.26158	2.942957
DNAJB7	1552675_at	2.901607	2.839887	2.756543	2.814382	2.903727	2.777185	2.825619	2.839823	2.827065	2.810392	2.835375	2.871477	2.615158
UCN3	1552676_at	2.334604	2.312886	2.287301	2.556451	2.333975	2.298396	2.301982	2.311361	2.279929	2.289158	2.281243	2.254912	2.218469
DIP2A	1552677_a_at	2.626175	2.541361	2.507588	2.528901	2.550856	2.53202	2.549002	2.485609	2.54297	2.524519	2.536516	2.608745	2.549568
USP28	1552678_a_at	2.668782	2.658628	2.622132	2.63786	2.651843	2.701001	2.645946	2.651207	2.641662	2.645409	2.642628	2.649806	4.484576
CASC5	1552680_a_at	2.780926	2.786196	2.664654	2.651096	2.786965	2.727983	6.617281	6.927873	6.631696	6.000939	6.427539	6.927121	4.396906
CASC5	1552682_a_at	2.585446	2.556235	2.484564	2.53273	2.55333	2.528288	4.127364	3.486684	5.722118	4.833532	2.656613	4.014334	2.473421
SENP8	1552684_a_at	2.304487	2.276133	2.254386	2.258467	2.281651	2.246019	2.268775	2.276179	2.352293	2.256269	2.261366	2.283024	3.012323

GRHL1	1552685_a_at	2.392971	2.374485	2.345023	2.364953	2.391283	2.367242	2.387059	2.377097	2.535026	2.368158	2.36204	2.371512	2.337917
C20orf152	1552686_at	2.754774	2.626228	2.565072	2.602408	2.639275	2.585772	2.570596	2.641443	2.629414	2.60053	2.734049	2.668206	2.505099
C20orf152	1552687_a_at	2.372906	2.346248	2.31214	2.326388	2.35842	2.57827	2.320348	2.346697	2.328248	2.321119	2.33243	2.340632	2.225954
CASKIN1	1552689_at	2.205348	2.195674	2.17758	2.179247	2.189716	2.184594	2.196442	3.111481	2.474141	2.185745	2.653828	2.443122	3.074306
CACNA2D4	1552690_a_at	2.06409	2.051563	2.031577	2.034026	2.046923	2.033983	2.04628	2.047877	2.044963	2.033588	2.034894	2.043143	1.99958
ARL11	1552691_at	4.231531	3.752869	3.886714	4.062647	4.17512	4.335364	3.848897	4.009444	4.004244	4.067804	4.155664	4.39605	3.319461
ARL11	1552693_at	2.60844	2.565239	2.524649	2.546926	2.599241	2.535867	2.554555	2.562176	2.551363	2.541232	2.551509	2.567807	2.467295
SLC2A13	1552694_at	3.594481	3.556654	4.065127	3.873223	3.507937	3.280217	3.397002	3.348166	3.400087	3.416799	3.423442	3.445028	3.288376
SLC2A13	1552695_a_at	3.694904	2.818908	4.530162	4.390708	2.555624	2.477184	2.492629	2.477448	2.464159	2.235192	2.46958	2.418688	2.503807
NIPA1	1552696_at	2.537482	2.469576	2.44659	2.449683	2.49112	2.450203	2.45566	2.46647	2.454065	2.44034	2.447749	2.462793	2.366299
MGC16703	1552698_at	2.757984	2.743051	2.598139	2.705268	2.749956	2.731864	2.739256	2.743094	2.734948	2.718516	2.728786	2.736451	2.995972
RFWD2	1552701_a_at	7.234052	6.596749	7.237506	7.534448	8.072118	8.271505	4.080933	5.919911	6.068829	5.509421	5.229996	5.498079	3.204053
CASP1	1552703_s_at	5.635097	5.603753	6.749957	6.469339	6.299054	6.211366	5.323678	5.003622	5.741864	5.452761	4.258023	4.565899	4.063994
DUSP19	1552705_at	4.288514	4.031493	3.958394	4.023965	4.153421	3.904093	4.040573	3.984238	4.07092	3.970259	4.071739	4.33964	4.083437
DUSP19	1552708_a_at	2.988619	2.958666	2.841007	2.94116	3.010628	2.949408	2.947098	2.956361	2.95025	2.885192	2.980148	3.035675	2.665591
CYB5D1	1552711_a_at	6.073316	5.934807	5.909219	6.279287	6.480269	6.463348	5.802685	5.941918	5.878214	5.871285	5.958016	5.976934	5.186422
NMNAT2	1552712_a_at	2.280774	2.265304	2.24682	2.249183	2.278342	2.249619	2.256767	2.265518	2.252252	2.240118	2.246398	2.256545	4.491386
SLC4A1	1552713_a_at	2.531257	2.492832	2.447747	2.528854	2.535403	2.532265	7.242619	5.665935	7.168503	7.312865	4.433992	4.690623	2.354909
CREG2	1552714_at	2.6158	2.55648	2.5052	2.550887	2.61532	2.537646	2.556669	2.573648	2.55491	2.533127	2.555654	2.565945	2.639521
RXFP1	1552715_a_at	2.224307	2.196963	2.2013	2.174968	2.207787	2.179998	2.185243	2.195734	2.184458	2.168261	2.185053	2.189153	2.093364
SPEF2	1552716_at	3.850142	3.789683	3.678115	3.503992	3.781261	3.772108	3.783921	3.785846	3.777294	3.916956	3.767887	3.80729	4.016426
CEP170	1552717_s_at	2.81763	2.733851	2.715017	2.724929	2.978948	2.703464	2.730396	2.729832	2.705537	2.707864	2.71774	2.728121	6.742327
DTD1	1552718_at	2.432285	2.427737	2.374223	2.411414	2.41785	2.41188	2.423825	2.416951	2.420665	2.412193	2.404874	2.418805	2.409048
CASC4	1552719_at	3.524663	2.983913	3.338895	3.163146	3.531531	4.140807	2.786968	2.988643	2.406362	2.787815	3.118217	2.973678	3.340252
FGF1	1552721_a_at	2.199299	2.174149	2.150744	2.157603	2.181877	2.156062	2.162018	2.170261	2.157741	2.146716	2.163488	2.171223	2.606175
ARPP-21	1552722_at	3.253287	3.127868	3.055443	3.129106	3.240884	3.075503	3.14343	3.150522	3.146306	3.097438	3.162185	3.256102	3.123968
RHOXF1	1552724_at	2.041407	2.289215	2.013146	2.015917	2.030427	2.182755	2.024406	2.029022	2.018954	2.013426	2.020112	2.021654	1.983254
ADAMTS17	1552725_s_at	2.403596	2.33291	2.311535	2.311392	2.332231	2.302153	2.326307	2.313769	2.324091	2.311644	2.324618	2.324407	2.130569
ADAMTS17	1552726_at	1.884602	1.878774	2.440563	1.917547	1.872781	1.858093	1.854996	1.872697	1.862048	1.854609	1.854624	1.857185	4.47348
ADAMTS17	1552727_s_at	3.040234	2.858659	4.036583	3.990726	3.434338	2.841316	2.597291	2.595666	2.580221	2.580417	2.605916	2.589877	5.120016
SNHG7	1552729_at	3.552358	3.410212	3.312432	3.116351	3.406907	3.393501	4.149816	4.285404	3.823638	4.045212	3.711666	3.675501	3.148102
DHH	1552730_at	2.240255	2.219404	2.205246	2.208399	2.226697	2.208685	2.216578	2.228466	2.212116	2.206139	2.206031	2.219014	2.156542
ABRA	1552731_at	2.493266	2.476073	2.449156	2.458083	2.477555	2.455486	2.470992	2.472965	2.460398	2.450697	2.457478	2.471795	2.409569
ABRA	1552732_at	3.024373	2.944173	2.869421	2.934529	2.992082	2.916941	2.950795	2.949527	2.94796	2.928183	2.964005	2.968262	4.55398
KLHDC1	1552733_at	6.409346	6.397029	7.012178	6.695079	6.265241	6.22377	5.330355	4.525008	5.607968	5.200119	4.493702	4.296663	5.914044
RICTOR	1552734_at	2.950616	2.816718	2.841362	2.859338	2.945077	2.855316	2.914529	2.874434	3.005434	2.872582	2.877096	2.89205	2.681128
PCDHGA4	1552735_at	3.927492	3.609629	3.589075	3.638659	4.539415	4.216409	3.497553	3.50217	3.522329	3.446765	3.934673	3.573914	5.624853
NETO1	1552736_a_at	2.432985	2.398043	2.355411	2.380541	2.420729	2.372235	2.38835	2.397698	2.385283	2.372256	2.379917	2.404975	3.65603

WWP2	1552737_s_at	1.95773	1.943414	1.925319	1.928856	1.945108	1.92728	1.939934	1.941583	1.930718	1.925402	1.923217	1.935607	4.061771
ST7L	1552738_a_at	3.768255	3.753155	3.668279	3.73525	3.827622	4.035358	3.657426	3.750214	3.738004	3.726818	3.874691	3.749493	3.643187
ST7L	1552739_s_at	2.961023	2.877538	2.977624	2.93517	2.949802	2.793116	2.974295	2.87629	2.950496	3.314448	2.858315	2.941176	3.617366
C2orf15	1552740_at	3.598316	3.458194	3.311054	3.45832	3.800181	3.453725	3.439013	3.427904	3.481019	3.437887	3.512623	3.570378	3.014886
KCNH8	1552742_at	2.239917	2.214321	2.187002	2.194587	2.222382	2.196954	2.203621	2.212513	2.199127	2.190566	2.194612	2.207827	2.165223
MCF2L	1552743_at	1.798961	1.789208	1.769536	1.773108	1.784337	1.777293	1.786267	1.78722	1.780491	1.774316	1.770912	1.779352	1.733507
SLCO6A1	1552745_at	2.288278	2.25648	2.212029	2.234521	2.26907	2.23709	2.243188	2.254058	2.23974	2.226825	2.238978	2.24957	2.124343
C3orf48	1552747_a_at	2.881499	2.794227	2.729571	2.784749	2.917585	2.786485	2.806087	2.818067	2.805417	2.780404	2.81198	2.844709	2.582668
KLC3	1552749_a_at	3.367097	3.471718	3.411491	3.365418	3.352248	3.610106	3.361034	3.529442	3.344129	3.455117	3.38745	3.696797	2.868832
CIB3	1552750_at	2.877524	2.843526	2.790305	2.825399	2.844014	2.834887	2.83452	2.896649	2.827066	2.831488	2.829507	2.840117	2.575919
CIB3	1552751_a_at	2.727672	2.693834	2.665212	2.673714	2.710208	2.672269	2.680527	2.605283	2.676496	2.663007	2.671624	2.704967	2.570259
CADM2	1552752_a_at	2.876103	2.831647	2.771074	2.813871	2.934149	2.810749	2.822011	2.823067	2.817708	2.794182	2.819403	2.828962	2.674345
CADM2	1552754_a_at	2.763762	2.700824	2.668835	2.713876	3.318541	2.748603	2.690368	2.706824	2.723498	2.676201	2.75267	2.950325	6.145718
C9orf66	1552755_at	2.623935	2.607589	2.452168	2.541244	2.607812	2.588586	3.552071	4.281217	3.078896	4.090682	2.700743	2.947028	2.355884
C9orf66	1552757_s_at	2.492602	2.462705	2.399515	2.448756	2.476292	2.433129	2.457479	2.460691	2.447509	2.444579	2.448267	2.456848	2.323047
HDAC9	1552758_at	2.794725	2.778637	2.760776	2.754399	2.799904	2.735041	2.764689	2.760547	2.762069	2.713831	2.761981	2.770746	2.659497
HDAC9	1552760_at	2.299055	2.27093	2.213864	2.251369	2.323191	2.251471	2.261823	2.270509	2.258881	2.232831	2.260002	2.294778	2.598808
SLC16A11	1552761_at	4.255082	4.180388	3.123293	3.142782	3.423047	3.623952	2.789663	2.990569	2.979497	2.936586	3.130133	3.130659	2.934357
TMEM67	1552763_at	2.842168	2.73143	2.699019	2.745703	2.826756	2.714404	2.758121	2.768365	2.754975	2.732892	2.755553	2.808978	2.560207
TMEM67	1552765_x_at	2.782143	2.715863	2.654894	2.70083	2.782982	2.691141	2.728354	2.730587	2.713599	2.694577	2.883466	2.754959	2.52525
HS6ST2	1552766_at	3.009086	2.885736	2.906288	2.947884	3.025999	2.929917	3.365981	2.906978	3.539076	3.176929	2.989223	3.224148	6.636803
HS6ST2	1552767_a_at	3.016115	3.080604	2.698053	2.885677	3.092829	3.274874	8.238136	8.272674	8.188283	7.571669	9.622172	9.030182	9.477292
CAMKK1	1552768_at	2.290604	2.282249	2.261436	2.266318	2.276259	2.273161	2.276691	2.281629	2.267239	2.262116	2.264097	2.272039	2.161172
ZNF625	1552769_at	3.326923	3.264603	3.2162	3.256868	3.299532	3.445851	3.263168	3.275948	3.260524	3.246206	3.306077	3.27042	3.253902
ZNF563	1552770_s_at	5.970835	5.608132	5.064461	5.268692	5.026223	4.15338	4.209269	4.478682	4.286201	4.136472	4.2854	4.56987	3.387973
LIN9	1552771_a_at	2.512315	2.477875	2.426488	2.457838	2.49826	2.460382	2.469569	2.481536	2.464418	2.454685	2.468336	2.512746	2.363578
CLEC4D	1552772_at	2.339307	2.312764	2.250546	2.290004	2.320079	2.30185	2.29775	2.30675	2.29315	2.28287	2.295125	2.30415	2.185497
CLEC4D	1552773_at	2.205311	2.184155	2.156414	2.16432	2.187801	2.166588	2.175327	2.183176	2.170471	2.160663	2.169254	2.177864	2.082376
SLC25A27	1552774_a_at	4.117232	3.670744	4.32446	3.649528	3.136859	3.096735	2.927918	2.604442	3.129478	3.031707	2.632281	2.832699	2.972981
GPRC6A	1552775_at	1.971011	1.9563	1.934179	1.939817	1.955676	1.940081	1.950195	1.953905	1.94005	1.932525	1.937154	1.94729	1.884747
RAET1E	1552777_a_at	2.160175	2.135839	2.115844	2.120508	2.14405	2.119196	2.130909	2.138146	2.12265	2.115393	2.114727	2.132179	2.043265
SLC44A5	1552779_a_at	3.099256	2.994753	2.902492	2.985417	3.094399	2.922787	2.988819	2.995381	2.996485	2.978554	3.016221	3.050829	2.805123
SLC44A5	1552782_at	3.194931	3.117338	2.992685	3.106154	3.201709	3.039688	3.124324	3.131485	3.105923	3.126339	3.22293	3.176814	2.986438
ZNF417	1552783_at	3.926938	4.556168	3.504951	3.780216	4.17454	3.736902	3.581962	3.628005	3.736717	3.703265	3.844013	3.875668	3.46231
ZNF781	1552785_at	3.578229	3.460367	3.27591	3.383057	3.614875	3.491033	3.45405	3.397367	3.479231	3.396646	3.513914	4.008872	4.686076
HELB	1552787_at	3.5486	3.453154	3.408559	3.387107	3.395246	3.373072	2.960949	3.482838	3.441734	3.374791	3.374167	3.197796	2.789411
HELB	1552788_a_at	2.356167	2.33951	2.309945	2.326373	2.33916	2.322131	2.327621	2.325753	2.345424	2.324038	2.308931	2.318924	2.251691
TLOC1	1552789_at	6.664072	6.151982	7.761179	7.410879	6.154724	5.956243	6.988317	6.051558	7.074732	7.275101	5.97322	5.788539	6.411417

TLOC1	1552790_a_at	7.787249	7.632365	8.821278	8.876404	8.128227	7.649005	7.921087	7.370563	7.973719	8.014987	6.812598	6.670831	6.840723
TRDN	1552791_a_at	2.927353	2.836177	2.790195	2.83432	2.905624	2.813935	2.84688	2.855533	2.843955	2.812851	2.844842	2.887174	3.05914
SOCS4	1552792_at	2.538062	2.500019	2.451923	2.485139	2.520807	2.496837	2.489139	2.499804	2.491966	2.47445	2.488742	2.505419	2.497982
C8orf31	1552793_at	2.365463	2.327029	2.281627	2.309053	2.345797	2.328281	2.319997	2.334147	2.31705	2.304392	2.319137	2.357813	2.483918
ZNF547	1552794_a_at	3.20918	3.154948	3.110998	3.16963	3.233778	3.108412	3.631814	3.463454	3.842124	4.096467	3.382044	3.92938	3.677387
SIM1	1552796_a_at	2.362002	2.324381	2.286294	2.304413	2.337107	2.296361	2.312585	2.324316	2.30857	2.296683	2.308634	2.319327	2.196682
PROM2	1552797_s_at	3.068124	2.992944	2.97272	2.98164	3.152832	3.071921	2.941274	2.979917	2.962996	2.946667	2.976049	2.985863	2.782374
TLR4	1552798_a_at	2.189125	2.176637	2.159896	2.153591	2.297569	2.163325	2.165835	2.172932	2.160741	2.149096	2.156352	2.16652	2.070024
TSNARE1	1552799_at	2.46194	2.431611	2.38328	2.416634	2.43905	2.406211	2.417265	2.428627	2.413937	2.399846	2.417425	2.425176	2.29452
ABHD11	1552800_at	1.818768	1.810707	1.792131	1.794373	1.803857	2.503435	1.806952	1.806373	1.80325	1.785756	1.791702	1.798551	1.771798
CAPN13	1552801_at	2.366185	2.316735	2.287445	2.291827	2.349311	2.293377	2.303819	2.31185	2.300889	2.288563	2.300621	2.311181	2.187105
C1orf215	1552803_a_at	2.728485	2.663326	2.621376	2.650269	2.786606	2.674523	2.694728	2.690413	2.67767	2.682226	2.743945	2.706307	2.640793
TIRAP	1552804_a_at	2.117598	2.109779	2.090563	2.08942	2.240381	2.089256	2.104896	2.096611	2.100538	2.086066	2.086554	2.096678	2.102702
SIGLEC10	1552806_a_at	2.325142	2.292755	2.252528	2.27962	2.30929	2.262475	2.280937	2.290279	2.276949	2.263389	2.273651	2.286017	2.18016
SIGLEC10	1552807_a_at	3.729084	3.495575	4.177902	3.590666	4.141297	4.354541	3.536074	4.140159	3.629124	3.35134	3.700045	3.345728	2.964469
ADAMTSL1	1552808_at	2.93296	2.90312	2.85966	2.848018	3.005655	2.818837	2.874271	2.903537	2.866203	2.869481	2.913957	2.967942	2.704082
RFX4	1552809_at	2.567169	2.51159	2.453902	2.483371	2.536645	2.493663	2.493425	2.507597	2.494291	2.47667	2.490983	2.501369	2.348689
WFIKKN1	1552811_at	3.550691	3.54285	3.509767	3.374514	3.377811	3.521557	3.720856	3.543586	3.53768	3.99064	3.684654	4.212719	3.308497
SENP1	1552812_a_at	2.358444	2.43742	2.303669	2.323358	2.490123	2.327998	2.554117	2.448663	2.328983	2.328915	2.327794	2.336496	3.859417
KLF14	1552813_at	2.036104	2.025363	2.006904	2.008345	2.024731	2.003705	2.019458	2.02225	2.012264	2.004476	2.007034	2.017102	2.014058
KLF14	1552814_a_at	2.397754	2.368413	2.348354	2.352935	2.401867	2.364413	2.381737	2.374033	2.360872	2.359656	2.360474	2.377579	2.260249
NXNL2	1552816_at	3.625594	3.549425	3.493529	3.516301	3.671653	3.528189	3.563882	3.573696	3.54815	3.535536	3.712232	3.775642	3.310522
BRS3	1552818_a_at	2.077532	2.059194	2.033693	2.041168	2.060602	2.040587	2.050322	2.057115	2.043792	2.037035	2.041376	2.051895	1.975033
C21orf86	1552819_at	2.04054	2.025082	1.99829	2.00713	2.025385	2.007085	2.015263	2.021969	2.010941	2.007271	2.004502	2.015743	1.941238
TXNDC10	1552822_at	3.180539	3.145627	3.000088	3.114131	3.244893	3.083593	3.134396	3.146126	3.141803	3.119042	3.219853	3.324476	2.82332
ZNF396	1552825_at	2.883277	2.973224	2.73509	2.813906	2.878846	2.821727	2.805	2.797581	2.809805	2.808507	2.965232	2.877577	2.5694
SLC26A7	1552826_at	3.414026	3.295224	3.193776	3.272478	3.468385	3.214102	3.266072	3.275255	3.296391	3.284704	3.349949	3.405988	2.950568
SLC26A7	1552827_s_at	2.429444	2.398109	2.370759	2.377	2.405724	2.377997	2.38519	2.397006	2.38172	2.368727	2.386238	2.407146	2.358006
SNAG1	1552828_at	2.076405	2.057895	2.03755	2.040503	2.058454	2.039716	2.050549	2.055724	2.048482	2.035386	2.040984	2.048879	1.99545
FBXO39	1552830_at	3.353364	3.324902	3.282286	3.307384	3.380873	3.304923	3.32206	3.320454	3.312131	3.304793	3.314282	3.331208	3.223833
B3GNT6	1552833_at	1.828637	1.821261	1.803863	1.806611	1.815275	1.807213	1.814742	1.822101	1.813699	1.799097	1.796068	1.80762	1.775262
B3GNT6	1552834_at	3.404004	3.177785	3.138868	3.072603	3.319531	3.191971	3.156239	3.226504	3.18028	3.141026	3.186102	3.373991	3.158896
DENND1B	1552835_at	2.537218	2.512293	2.486862	2.495141	2.517088	2.493335	2.501629	2.51121	2.497293	2.496759	2.496844	2.506727	2.347697
ZNF619	1552836_at	1.766792	1.760201	1.743039	1.742946	1.755983	1.741981	1.752934	1.755341	1.753382	1.740336	1.739588	1.747352	1.704065
ICK	1552837_at	2.983902	2.904482	2.811647	2.874964	3.048135	2.880657	2.889269	2.907649	2.888124	2.881347	2.898568	2.960322	2.64433
C14orf54	1552839_at	2.965125	2.932665	2.892438	2.914445	3.112868	2.924976	2.896134	2.902935	2.919306	2.908854	2.943141	2.932459	2.851024
BCL2L14	1552841_s_at	2.120861	2.090186	2.071789	2.076931	2.114989	2.077711	2.090596	2.084873	2.076509	2.072199	2.079893	2.08953	2.007027
HS6ST3	1552842_at	2.37302	2.344677	2.31784	2.324978	2.358587	2.317495	2.333116	2.345001	2.329419	2.322187	2.329932	2.33863	2.227114

SLC26A1	1552843_at	2.561773	2.508321	2.448057	2.476813	2.567828	2.468571	2.482104	2.498398	2.47938	2.467022	2.483581	2.519386	2.34247
CLDN15	1552845_at	1.797608	1.896896	1.687969	1.78409	1.80199	1.779142	1.790357	2.184075	1.927052	1.777298	1.951678	2.011257	1.757539
RAB42	1552846_s_at	2.846132	2.816184	2.796105	2.791226	2.96367	2.806004	3.043309	2.816664	2.795028	2.796697	2.814315	2.810751	2.765421
PTCHD1	1552848_a_at	2.258971	2.242187	2.216115	2.224696	2.246555	2.223494	2.233368	2.23894	2.226071	2.219345	2.663531	2.23389	5.079662
LOC130951	1552849_at	2.862581	2.776805	2.714048	2.746916	2.850101	2.77986	2.783985	2.799089	2.784433	2.752427	2.781574	2.820308	2.567512
ZSCAN4	1552851_at	2.41161	2.377773	2.336128	2.359541	2.395185	2.348001	2.367709	2.377231	2.364151	2.35173	2.362948	2.374503	2.246044
ZSCAN4	1552852_a_at	3.035301	2.985547	2.846515	2.968915	3.126236	2.9452	2.98115	2.961578	2.978283	2.92633	2.975961	3.082069	2.819901
FLJ32784	1552853_at	2.264298	2.225151	2.210604	2.221783	2.307328	2.196381	2.229166	2.240694	2.232616	2.221244	2.230237	2.274715	2.129079
FLJ32784	1552854_a_at	2.094276	2.082856	2.059145	2.062953	2.098695	2.095604	2.078784	2.081783	2.0681	2.06739	2.06767	2.076838	1.959135
TMEM148	1552856_at	2.582174	2.545988	2.500332	2.52877	2.563821	2.536459	2.536008	2.545773	2.533022	2.520234	2.578717	2.549634	2.40172
HTR6	1552857_a_at	2.161609	2.140321	2.113337	2.121773	2.144278	2.12038	2.130659	2.139615	2.124695	2.117659	2.124212	2.134469	2.047308
MAGEB6	1552858_at	3.536993	3.233487	3.330125	3.323014	3.68112	3.181573	3.289957	3.254621	3.351406	3.306379	3.469683	3.56879	2.949382
TCEB3C	1552860_at	2.948476	2.910079	2.85922	2.899412	2.95171	2.906866	2.893917	2.918394	2.904377	2.891719	2.905304	2.916771	2.737083
C1orf104	1552862_at	2.48045	2.491242	2.453094	2.426551	2.473883	2.456273	3.519398	2.649316	2.626696	2.817715	2.568126	3.356816	2.405713
CACNG6	1552863_a_at	2.516469	2.512348	2.488147	2.431981	2.500174	2.493004	2.510157	2.505041	2.502504	2.607662	2.582503	2.498428	2.457752
IFLTD1	1552865_a_at	1.748908	1.747249	1.72444	1.730201	1.735833	1.728047	1.742446	1.740323	1.739065	1.729318	1.722967	1.724754	1.732037
C19orf23	1552868_at	3.153562	3.149246	3.111409	3.023256	3.250792	3.111808	3.13574	3.556549	3.127073	3.105445	3.057505	3.145742	2.930601
C1orf125	1552870_s_at	3.022742	2.929532	2.847483	2.922186	3.010968	2.881495	2.936776	2.946142	2.933581	2.908813	2.951397	2.977942	2.687065
CXYorf2	1552872_at	2.776324	2.744301	2.725952	2.631581	2.744588	2.725728	2.737316	2.736909	2.723312	2.717601	2.721933	2.734555	2.644353
CXYorf2	1552873_s_at	1.734074	1.727395	1.70884	1.713956	1.725801	1.715118	1.724904	1.724831	1.715762	1.787801	1.708938	1.717376	1.677153
CD200R1	1552875_a_at	2.742687	2.693119	2.67535	2.681434	2.721843	2.660136	2.783312	2.709404	2.695992	2.688911	2.691077	2.722033	2.491443
C21orf89	1552876_at	2.454722	2.417908	2.369767	2.400186	2.440449	2.409265	2.409549	2.420668	2.404215	2.386297	2.405063	2.416372	2.273274
C21orf89	1552877_s_at	2.680946	2.608302	2.547833	2.593672	2.65399	2.858908	2.592016	2.603326	2.604075	2.568321	2.585914	2.603894	2.403846
ATOH7	1552878_at	2.491965	2.408384	3.687679	2.56947	2.457317	2.65133	2.397175	2.408874	2.392441	2.380785	2.398682	2.407578	2.650658
ATOH7	1552879_a_at	3.901493	4.191961	5.209257	5.638743	3.775251	4.2487	3.2707	3.278468	3.403971	3.24012	3.20604	3.212617	3.782876
SEC16B	1552880_at	5.183703	4.581336	7.146712	6.375223	4.453978	4.004079	4.559415	3.113841	4.183093	3.838677	3.562305	3.392179	3.40136
FAM123B	1552882_a_at	2.635342	2.611855	2.591401	2.594663	2.620011	2.591915	2.61026	2.606237	2.606953	2.574309	2.595135	2.607502	2.440178
NKX6-3	1552884_at	2.130958	2.124891	2.097961	2.106215	2.122867	2.102444	2.119379	2.122306	2.10897	2.108666	2.107843	2.11873	2.081495
NKX6-3	1552885_a_at	3.187884	3.18264	3.120391	2.986776	3.102186	3.549277	3.178242	3.356171	3.151329	3.164575	3.160439	3.304445	2.724993
C10orf27	1552887_at	2.700412	2.68615	2.545039	2.667166	2.691124	2.53476	2.685788	2.686427	2.670255	2.662242	2.653192	2.683064	2.609849
EXOC3L2	1552889_a_at	6.139026	6.185382	6.150449	6.159534	6.175312	6.722621	5.975943	6.378995	6.138506	6.032393	6.201488	6.503227	5.085438
CABP4	1552890_a_at	2.424406	2.395815	2.358654	2.37503	2.412333	2.380339	2.38318	2.392631	2.379508	2.369062	2.37527	2.389234	2.254524
TNFRSF13C	1552892_at	2.292918	2.284166	2.26562	2.268659	2.286572	2.26775	2.278005	2.286613	2.27713	2.266689	2.267565	2.28051	2.228206
CAMK2N2	1552893_at	1.553269	1.546421	1.527873	1.533074	1.539575	1.529664	1.544973	1.530853	1.539575	1.530665	1.521231	1.523938	1.54676
C21orf99	1552895_a_at	2.843019	2.770354	2.703449	2.755793	2.825184	2.760641	2.777	2.785543	2.768307	2.748701	2.772527	2.809712	2.616799
KCNG3	1552897_a_at	2.412344	2.383108	2.353978	2.367391	2.40121	2.368635	2.375584	2.384275	2.371176	2.36148	2.367388	2.380891	2.279649
KCNG3	1552898_a_at	2.16778	2.143116	2.117176	2.126308	2.148445	2.126614	2.136399	2.14412	2.129108	2.121813	2.126333	2.13813	2.062143
RP4-662A9.	1552899_at	3.892198	3.675803	3.626742	3.748104	3.932729	3.71312	3.709026	3.699605	3.720232	3.629087	3.829052	3.852369	2.998968

RP4-662A9.	1552900_a_at	2.747186	2.664112	2.589736	2.651302	2.705042	2.661231	2.65829	2.671466	2.654643	2.632611	2.655463	2.689379	2.426033
FOXP2	1552902_a_at	2.269191	2.241724	2.209915	2.22249	2.248893	2.22301	2.230183	2.239763	2.226407	2.217889	2.223455	2.23581	3.786512
B4GALNT2	1552903_at	2.280332	2.259196	2.230236	2.239849	2.261446	2.234658	2.24764	2.256177	2.24282	2.235244	2.242969	2.250201	2.157828
NETO1	1552904_at	3.201726	3.041693	2.964656	3.037913	3.235759	2.984983	3.047452	3.055346	3.065113	3.017579	3.087059	3.129704	2.737863
FMR1NB	1552906_at	3.320141	3.250518	3.09704	3.223819	3.355689	3.192552	3.236434	3.248388	3.23334	3.211086	3.24532	3.280174	2.805478
C1orf150	1552908_at	3.196717	3.114237	2.984788	3.115109	3.185388	3.083653	3.128963	3.111272	3.124469	3.101979	3.153775	3.295417	2.929409
SIGLEC11	1552910_at	6.345229	6.251037	5.760903	5.953964	7.027547	7.804075	5.212775	6.433951	5.813139	5.142046	6.091363	6.632581	4.775744
SIGLEC11	1552911_at	2.78719	2.779609	2.762099	2.76515	2.786162	2.747091	2.761382	2.773486	2.764312	2.766956	2.759544	2.806301	2.811073
IL23R	1552912_a_at	2.620132	2.583395	2.55145	2.566002	2.594171	2.557817	2.573894	2.58351	2.569681	2.558471	2.569614	2.585769	2.407087
MAGEB18	1552913_at	3.008884	2.917042	2.856949	2.892718	2.998766	2.876967	2.907081	2.928012	2.919743	2.895079	2.923401	2.960488	2.632471
CD276	1552914_a_at	2.047331	2.042944	2.015672	2.026583	2.044213	2.023876	2.044687	2.044423	2.474416	2.172879	2.467193	2.146514	3.295018
IL28A	1552915_at	2.35327	2.317756	2.301842	2.306297	2.342459	2.298768	2.317274	2.326613	2.314157	2.291878	2.30495	2.31868	2.181017
IL29	1552917_at	2.411069	2.389384	2.342415	2.369056	2.393886	2.36964	2.379774	2.389689	2.37431	2.367119	2.375092	2.384167	2.247391
C4orf36	1552919_at	2.80534	2.75871	2.679698	2.814604	2.838103	2.701684	2.747272	2.759051	2.742719	2.718328	2.744558	2.781187	2.560063
FIGNL1	1552921_a_at	2.497213	2.478642	2.487573	2.458836	2.480551	2.574935	4.682499	2.503738	3.974811	4.618414	2.461821	2.49514	5.563733
RIMS1	1552922_at	2.858616	2.795357	2.737965	2.765902	2.922937	2.778334	2.790425	2.794184	2.781825	2.753079	2.779247	2.79949	2.624749
PITPNM2	1552923_a_at	2.07913	2.135219	2.052647	2.056427	2.072407	2.180259	2.062286	2.068894	2.052625	2.046706	2.050933	2.059238	1.984933
PITPNM2	1552924_a_at	1.713039	2.575384	2.028949	1.635096	1.645547	1.626795	1.62746	1.617255	1.613347	1.615061	1.619162	1.61995	1.636395
PCDH10	1552925_at	2.13769	2.117544	2.092743	2.099264	2.123454	2.097673	2.109059	2.11393	2.101679	2.094277	2.100501	2.108309	2.065283
MAP3K7IP3	1552927_at	3.589174	3.490376	3.331654	3.471857	3.608603	3.422365	3.461634	3.492242	3.517666	3.464552	3.500533	3.550537	3.504222
MAP3K7IP3	1552928_s_at	2.522335	2.572566	2.461991	2.448058	2.512363	2.472507	2.818291	2.589927	3.039392	3.130408	2.486641	2.504758	2.448943
GRK7	1552929_at	2.239591	2.2186	2.196289	2.196337	2.227485	2.184935	2.216036	2.216293	2.203744	2.197421	2.200293	2.211958	2.11157
MMEL1	1552930_at	3.149024	3.173228	3.128965	3.07467	3.201843	3.718656	3.073664	3.091975	3.076661	3.06967	3.070656	3.111304	3.012592
PDE8A	1552931_a_at	3.179779	3.137581	5.41181	4.564486	2.846442	2.555893	3.426132	2.576349	4.194019	4.543904	2.606326	2.570509	4.330648
NLRP6	1552932_at	2.136741	2.117106	2.089406	2.098541	2.118608	2.09598	2.108616	2.112613	2.100141	2.092745	2.094793	2.108643	2.028498
C1orf62	1552933_at	2.590249	2.542288	2.493127	2.534927	2.614907	2.53348	2.545378	2.550739	2.542292	2.536599	2.534853	2.555031	2.475174
ZCCHC5	1552935_at	2.485936	2.454944	2.429764	2.463587	2.471226	2.441266	2.444319	2.452498	2.437618	2.431864	2.43886	2.452659	2.378844
ATRIP	1552937_s_at	2.836467	2.706931	2.497532	2.495353	3.397948	3.607763	4.260574	5.176191	4.020847	4.186977	5.009385	5.114256	3.232011
ZIC5	1552938_at	2.457968	2.420137	2.379943	2.407379	2.445861	2.392471	2.415956	2.425705	2.412371	2.395096	2.410551	2.454142	2.318768
ANGPT1	1552939_at	2.807635	2.732534	2.673869	2.723523	2.815214	2.709992	2.735902	2.745697	2.73294	2.712225	2.730385	2.777199	2.520741
TEDDM1	1552940_at	2.064333	2.04869	2.024637	2.029705	2.048602	2.133666	2.039301	2.047156	2.030963	2.025111	2.032425	2.041071	1.982984
FLJ30430	1552942_at	2.404292	2.393516	2.373925	2.378468	2.393645	2.377567	2.391576	2.393857	2.383166	2.372802	2.376605	2.3856	2.318279
GABRG1	1552943_at	2.253784	2.225753	2.196498	2.211513	2.235998	2.210013	2.221434	2.226994	2.214066	2.206352	2.213928	2.234826	2.117407
PANX2	1552944_a_at	2.803137	2.561866	2.514882	2.540668	2.577138	2.52696	2.548928	2.549247	2.545662	2.529063	2.535127	2.543761	2.387109
ZNF114	1552946_at	2.654958	2.593637	2.499359	2.608441	2.637865	2.580824	2.616449	2.695587	2.612086	2.602258	2.950509	2.810362	3.290454
ZNF114	1552947_x_at	2.946323	2.832596	2.878916	2.854862	2.934825	2.87124	2.903809	2.889371	2.904315	2.866897	3.131006	3.770109	3.212036
CCDC27	1552948_at	2.915064	2.865326	2.835628	2.848485	2.901925	2.804711	2.835375	2.864956	2.843576	2.839099	2.851433	2.87097	2.749391
C15orf26	1552950_at	2.941619	2.867654	2.864909	2.852823	3.079635	2.886516	2.877925	2.899684	2.905652	2.849501	2.898719	2.937838	2.728521

RBMY2FP	1552952_at	2.496992	2.462271	2.427188	2.442425	2.467285	2.426433	2.452659	2.463205	2.448627	2.433359	2.446	2.460015	2.330026
NEUROD2	1552953_a_at	2.006373	1.953868	1.928871	1.933994	1.961086	1.954245	1.962977	1.955516	1.945646	1.949911	1.962773	1.948614	1.951458
C5orf17	1552954_at	2.260763	2.230305	2.207322	2.213028	2.241091	2.211242	2.223324	2.231444	2.215871	2.207757	2.216719	2.225372	2.147382
C8orf14	1552955_at	2.807231	3.221264	2.778437	2.7826	2.794377	2.77792	2.790474	3.117919	2.736507	2.723457	2.73585	2.81305	2.547495
LOC200383	1552957_at	2.679479	2.641267	2.587873	2.62689	2.685544	2.625914	2.6332	2.639179	2.628332	2.617109	2.627057	2.643785	2.441989
LRRC15	1552960_at	2.329824	2.313947	2.291674	2.296108	2.313892	2.297377	2.307515	2.31418	2.299584	2.296873	2.299675	2.358419	2.182536
C10orf93	1552964_at	1.833789	1.822577	1.803628	1.807301	1.820065	1.807905	1.818609	1.819817	1.80827	1.803752	1.804495	1.813166	1.76451
B3GNT7	1552965_a_at	1.953714	1.947036	1.913858	1.930966	1.942544	1.923279	1.944141	1.941691	1.932947	1.932519	1.960875	1.93742	1.907167
ZNF645	1552967_at	2.301306	2.274467	2.252801	2.256702	2.277854	2.254686	2.262952	2.270794	2.258368	2.249986	2.251419	2.262056	2.181137
ZMYM6	1552969_a_at	3.252404	3.104702	3.032482	3.104211	3.259291	3.071762	3.110471	3.127616	3.128794	3.090516	3.138642	3.220393	3.017068
ZMYM6	1552970_s_at	2.782808	2.715804	2.759001	2.707456	2.804786	2.723594	2.744586	2.733091	2.775876	2.71279	2.741267	2.749188	2.572523
SGCZ	1552971_at	2.409593	2.375872	2.334268	2.357713	2.398076	2.349854	2.367119	2.378304	2.364015	2.350355	2.362117	2.372407	2.2598
hCG_20329	1552972_at	3.334262	3.184869	3.045112	3.174417	3.353835	3.121029	3.179061	3.138433	3.207073	3.153547	3.246264	3.481164	2.805531
WNT9B	1552973_at	2.267765	2.241079	2.206861	2.227561	2.250304	2.231126	2.229586	2.23989	2.224302	2.215893	2.225594	2.233128	2.12987
TNRC5	1552977_a_at	6.091013	6.214931	7.368961	7.200593	6.443224	5.997352	6.912489	5.855363	6.95942	6.713474	5.657656	6.161132	6.917512
SCAMP1	1552978_a_at	3.793546	3.169893	5.620806	5.095496	3.250275	3.166189	4.952521	3.253582	4.722474	3.720378	3.193325	3.220018	7.815827
C2orf52	1552979_at	2.50551	2.462594	2.425029	2.443837	2.492857	2.447742	2.456927	2.467693	2.452756	2.444973	2.490773	2.469272	2.347183
HAS3	1552980_at	3.545924	3.558295	3.51433	3.527261	3.63399	3.51615	3.528012	3.530377	3.308005	3.553132	3.299493	3.524005	3.212119
FGF4	1552982_a_at	1.855788	1.847969	1.828517	1.832875	1.846886	1.830587	1.843952	1.844661	1.835969	1.82936	1.831571	1.838372	1.790126
SLC30A8	1552985_at	2.156206	2.13646	2.107855	2.117385	2.138865	2.116079	2.127867	2.13494	2.12018	2.112595	2.119356	2.129649	2.067381
LOC142937	1552986_at	3.272826	3.167987	2.588291	2.625245	2.688468	2.846092	2.633023	2.636892	2.633454	2.625348	2.617933	2.629561	2.404085
MGC42157	1552987_a_at	2.95765	2.894896	2.825338	2.887809	2.999644	2.844896	2.901953	2.894493	2.905498	2.870847	2.906526	2.937105	2.629037
C11orf65	1552988_at	2.654691	2.551322	2.573488	2.590495	2.748086	2.596462	2.490315	2.533744	2.59652	2.585402	2.596031	2.630145	2.088482
C19orf16	1552990_at	2.075975	2.067438	2.047	2.051339	2.064757	2.047316	2.061523	2.066689	2.052365	2.048355	2.050691	2.060947	1.982587
OR5P2	1552991_at	1.645101	1.638388	1.619856	1.623265	1.636985	1.627267	1.633241	1.635504	1.628599	1.624635	1.619671	1.628648	1.59363
DYDC1	1552993_at	3.02994	2.941296	2.87147	2.918236	3.037541	2.918151	2.951136	2.96363	2.9507	2.932449	2.962815	2.999836	2.640071
IL27	1552995_at	1.806717	1.927541	1.927466	2.56324	2.35345	2.526221	1.728309	1.728422	1.369782	1.365497	1.437356	1.720085	1.639561
IQCF1	1552997_a_at	1.739325	1.725941	1.705815	1.710835	1.725255	1.711623	1.720652	1.723087	1.714189	1.709171	1.705635	1.717463	1.669131
DEFB125	1552998_at	2.212149	2.186397	2.157523	2.169938	2.187121	2.169269	2.181781	2.184106	2.172728	2.164691	2.166518	2.178853	2.118735
WFDC10B	1552999_a_at	2.039449	2.020398	1.99045	1.996156	2.023879	1.994789	2.003813	2.006768	1.99185	1.989545	1.990383	2.000256	1.921181
DEFB105A	1553002_at	2.328651	2.307676	2.279314	2.288339	2.310901	2.28581	2.302242	2.306718	2.291379	2.281037	2.287908	2.300945	2.212322
PKHD1	1553003_at	1.955699	1.928655	1.912159	1.915789	1.938505	1.90628	1.930854	1.923984	1.911254	1.911268	1.915634	1.923489	1.867991
PKD1L1	1553004_at	2.214656	2.191085	2.161672	2.17536	2.196761	2.179737	2.179264	2.190783	2.175164	2.166586	2.174578	2.183065	2.088475
GPR112	1553006_at	2.047544	2.022482	2.000684	1.999102	2.035883	2.004181	2.016865	2.020688	2.006515	1.999727	2.000755	2.012977	1.951238
ODZ1	1553007_a_at	2.408844	2.378321	2.358227	2.353573	2.502897	2.363841	2.362834	2.374185	2.376571	2.370525	2.371985	2.374881	2.241751
REXO1L1	1553009_s_at	2.300941	2.270084	2.235064	2.251076	2.282267	2.250018	2.260775	2.271757	2.255901	2.245074	2.254687	2.266619	2.173091
TAF1L	1553011_at	3.496866	3.249592	3.231638	3.222382	3.315554	4.270665	3.178578	3.442093	3.391224	3.175317	3.297687	3.461065	2.981135
CNTNAP5	1553013_at	2.085955	2.064421	2.043513	2.048386	2.068885	2.047437	2.059294	2.061332	2.05073	2.043006	2.04532	2.059467	2.047842

RECQL4	1553015_a_at	3.554997	4.226874	3.169361	3.624288	3.480344	3.879781	6.419879	6.909918	6.365462	6.842453	7.300991	6.831892	5.005885
GPR113	1553016_at	1.91495	1.904434	1.88655	1.885604	1.902004	1.88942	1.900952	1.901911	1.893138	1.888215	1.886819	1.895511	1.850651
TMC2	1553019_at	2.039124	2.029273	2.007663	2.013637	2.027514	2.011445	2.023765	2.025834	2.014666	2.009618	2.011697	2.018857	1.959706
SMCR5	1553020_at	2.601669	2.577425	2.523327	2.535256	2.583639	3.097813	2.566967	2.575596	2.557261	2.542723	2.557029	2.570412	2.426643
BICD2	1553021_s_at	2.289473	2.262812	2.229757	2.240464	2.259106	2.233329	2.261655	2.259482	2.256853	2.241236	2.247197	2.252912	2.284694
ZIM3	1553022_at	2.940548	2.843546	2.779923	2.840546	2.928869	2.835466	2.851056	2.85818	2.847277	2.820016	2.858449	2.895458	2.595522
NOX5	1553023_a_at	2.394332	2.374134	2.341823	2.352324	2.376759	2.354678	2.362214	2.372408	2.360769	2.350258	2.36085	2.366702	2.276565
G30	1553024_at	2.517954	2.483255	2.431224	2.462763	2.504102	2.445101	2.473873	2.481489	2.46907	2.46102	2.46563	2.480691	2.325773
GPR126	1553025_at	2.375975	2.345712	2.319987	2.323591	2.346544	2.325992	2.326837	2.33486	2.319021	2.306286	2.317668	2.328108	2.231575
KLHL4	1553027_a_at	2.383896	2.356353	2.311677	2.338961	2.367552	2.322784	2.341142	2.353254	2.340662	2.323712	2.34006	2.348236	3.447264
GPR156	1553028_at	2.228255	2.210146	2.187067	2.194423	2.285982	2.19453	2.202972	2.208215	2.196078	2.185258	2.190392	2.203597	2.136954
SUOX	1553030_a_at	5.813334	5.572164	6.530566	6.380217	5.376601	5.140106	5.727885	4.599095	5.903778	5.235857	4.196265	4.483238	4.394581
GPR115	1553031_at	1.895278	1.884787	1.864152	1.868544	1.884183	1.866917	1.878759	1.882357	1.875805	1.863451	1.866309	1.877826	1.827764
IL31RA	1553032_at	2.504332	2.471694	2.451595	2.453492	2.483361	2.433964	2.461592	2.467916	2.456769	2.443921	2.45591	2.468227	2.31648
SYTL5	1553033_at	2.539746	2.505461	2.490431	2.484671	2.533805	2.494813	2.495336	2.506347	2.491964	2.479828	2.487037	2.500715	2.377103
SDCCAG8	1553034_at	4.951568	4.899858	4.695296	4.901163	4.939951	4.800037	5.254659	4.862821	5.957473	5.500216	4.947411	4.964503	4.591428
GPR111	1553036_at	2.178412	2.163174	2.13829	2.145022	2.163287	2.144347	2.153946	2.160168	2.147592	2.144837	2.14336	2.154892	2.063637
SYN2	1553037_a_at	2.626118	2.608397	2.568954	2.576115	2.636607	2.579697	2.601639	2.605732	2.580318	2.582775	2.589049	2.603759	2.535862
ASB10	1553039_a_at	0.974431	0.967794	0.947334	0.953001	0.95852	0.949649	0.965468	0.954771	0.960371	0.949034	0.94057	0.955716	0.965718
HTR3C	1553041_at	2.509507	2.499374	2.482068	2.481862	2.498552	2.488008	2.495507	2.49803	2.4831	2.485856	2.483018	2.489709	2.433324
TA-NFKBH	1553042_a_at	2.276419	2.264777	2.245304	2.249753	2.26933	2.248155	2.25998	2.261511	2.252196	2.244482	2.250019	2.256517	2.205664
CD300LF	1553043_a_at	2.628053	3.221344	2.599938	2.630863	4.051272	4.438928	2.615273	2.764368	2.624128	2.602664	2.392714	2.64446	2.439265
CX62	1553044_at	1.981364	1.964167	1.944048	1.947787	1.967311	1.945909	1.95988	1.963828	1.950855	1.943739	1.948954	1.958051	1.894044
WNT9A	1553045_at	2.284252	2.255614	2.245016	2.249066	2.274403	2.237556	2.252385	2.260384	2.248202	2.229437	2.242396	2.258722	2.148244
NEU4	1553046_s_at	1.955987	1.941199	1.912041	1.919524	1.941142	2.079215	1.933823	1.938041	1.925157	1.948089	2.11628	1.933006	1.878872
PIP4K2B	1553047_at	2.839689	2.833208	2.813362	2.817507	2.825694	2.818429	2.828973	2.827355	2.915381	2.811261	2.815659	2.821838	2.648444
PIP4K2B	1553048_a_at	2.548582	2.517117	2.471264	2.537095	2.538311	2.517813	2.518052	2.52882	2.513265	2.505776	2.512049	2.514209	2.92157
ODF3	1553051_s_at	2.527521	2.518204	2.501307	2.500397	2.516058	2.498873	2.510774	2.517269	2.513097	2.504831	2.50104	2.510032	2.474962
WFDC13	1553052_at	2.640028	2.585437	2.531816	2.573163	2.629687	2.575867	2.586885	2.58927	2.580118	2.57231	2.580202	2.622707	2.498543
C14orf48	1553053_at	2.696685	2.660924	2.594788	2.642681	2.818103	2.640249	2.652157	2.660895	2.648439	2.623328	2.647229	2.674444	2.462571
SLFN5	1553055_a_at	3.176812	3.425191	2.874953	2.966171	3.483209	4.370363	2.848115	2.977095	2.843125	2.836564	2.841983	2.970524	2.734364
SERPINB12	1553057_at	2.56512	2.528106	2.515129	2.509556	2.553135	2.521312	2.522657	2.530924	2.517522	2.501942	2.514116	2.523707	2.386316
GIPC3	1553058_at	2.84978	2.57152	2.51866	2.515152	2.580955	2.547753	2.444931	2.571352	2.46327	2.498575	2.54808	2.700777	2.256228
PGLYRP3	1553059_at	2.299757	2.283158	2.237701	2.272061	2.28797	2.268106	2.279535	2.287961	2.27304	2.264857	2.273026	2.295943	2.193046
PSKH2	1553060_at	2.192033	2.166425	2.138905	2.148113	2.172261	2.153989	2.156224	2.168223	2.152498	2.152586	2.151966	2.161929	2.104963
OR6W1P	1553061_at	2.23354	2.220219	2.19073	2.19761	2.238674	2.207287	2.215277	2.217218	2.211368	2.204583	2.200599	2.208165	2.125583
MOGAT1	1553062_at	6.948729	7.085085	5.665382	6.434753	7.545095	7.212947	2.213422	2.130383	2.116537	2.098629	2.113068	2.155781	2.058289
GPR78	1553063_at	1.829938	1.819719	1.795782	1.800115	1.813908	1.801808	1.810093	1.814209	1.802989	1.802004	1.797779	1.80646	1.767965

H1FOO	1553064_at	2.81624	2.769098	2.720624	2.772082	2.810364	2.736566	2.780863	2.78112	2.771456	2.74844	2.764507	2.781072	2.546463
TAAR9	1553066_at	1.881465	1.869774	1.850005	1.853324	1.868718	1.852044	1.864427	1.866067	1.854883	1.849284	1.84708	1.859712	1.809249
GNRHR2	1553067_a_at	2.482895	2.472617	2.44489	2.453555	2.472235	2.436776	2.466004	2.474225	2.458652	2.461101	2.457598	2.468063	2.375711
C21orf87	1553069_at	1.782064	1.769119	1.75074	1.753673	1.770067	1.753867	1.766541	1.769291	1.757013	1.752574	1.749814	1.761155	1.713659
MYOZ3	1553070_a_at	2.018882	2.004849	1.986743	1.990115	2.008518	1.989382	2.002539	2.002763	1.998282	1.990394	1.988455	1.996566	1.943422
MYOZ3	1553071_a_at	2.347829	2.313323	2.295022	2.293567	2.326588	2.302862	2.301844	2.30676	2.298359	2.284813	2.2908	2.304148	2.225709
BNIPL	1553072_at	2.451507	2.415897	2.372026	2.398613	2.438534	2.399681	2.403565	2.41377	2.403876	2.390053	2.403502	2.41562	2.315079
ASB11	1553074_at	2.464443	2.422475	2.351736	2.39387	2.453488	2.387141	2.406484	2.41855	2.401454	2.390816	2.400318	2.429089	2.274816
OFCC1	1553075_a_at	3.026402	2.955989	2.905253	2.958978	3.117585	2.853614	2.96215	2.971982	2.95778	2.899628	2.964746	3.03639	2.63525
OFCC1	1553076_at	2.37174	2.342656	2.324999	2.33096	2.365915	2.329738	2.338472	2.34518	2.333461	2.325608	2.335095	2.343036	2.234912
SDR-O	1553077_at	2.225991	2.206064	2.171869	2.18569	2.210457	2.187033	2.193607	2.205346	2.190309	2.180446	2.189726	2.200125	2.099094
OR5P3	1553078_at	2.232238	2.223508	2.205664	2.209965	2.221365	2.211351	2.221306	2.222223	2.213295	2.208743	2.207051	2.214456	2.176408
TRIM40	1553079_at	2.262887	2.249393	2.182878	2.203713	2.230825	2.198821	2.214759	2.233482	2.210746	2.192874	2.206465	2.219008	2.108824
CSN1S2A	1553080_at	2.72032	2.653584	2.588799	2.633255	2.684689	2.625112	2.641522	2.653766	2.639249	2.622767	2.639814	2.660844	2.456962
WFDC12	1553081_at	2.454208	2.444696	2.424711	2.534241	2.441339	2.395549	2.44522	2.445101	2.448704	2.504958	2.431089	2.572301	2.346502
CRYGN	1553082_at	2.344354	2.308369	2.26749	2.288565	2.332458	2.289229	2.293313	2.301257	2.288816	2.272867	2.291026	2.314525	2.182517
STARD6	1553084_at	2.475616	2.415986	2.362484	2.405145	2.431208	2.40439	2.417045	2.429956	2.413149	2.396917	2.414235	2.479372	2.30947
C11orf40	1553086_at	2.136823	2.117084	2.093738	2.099979	2.120089	2.101987	2.110273	2.116264	2.10165	2.094118	2.101199	2.109224	2.025114
C18orf12	1553087_at	3.071622	2.983073	2.930188	2.939476	3.068273	2.989254	2.970045	2.980731	2.968055	3.11997	2.963869	3.022016	2.797719
BCL2L11	1553088_a_at	3.123339	3.067097	3.093189	3.07881	3.198803	3.296424	3.139636	3.171552	3.10727	3.286675	3.195436	3.167143	3.000435
WFDC2	1553089_a_at	2.004977	1.997448	1.972479	1.977078	1.995436	1.984511	1.99514	1.995538	1.988502	1.982633	1.979849	1.988891	1.944182
DEFB119	1553093_a_at	2.283565	2.25276	2.219657	2.236405	2.266335	2.235685	2.248556	2.252767	2.238315	2.231233	2.237869	2.249677	2.145375
TAC4	1553094_at	2.78265	2.721953	2.668469	2.713099	2.756869	2.687677	2.726118	2.735961	2.681329	2.703844	2.720271	2.748438	2.4685
BCL2L11	1553096_s_at	3.470903	3.701633	3.006211	3.36786	3.532372	3.827405	4.432412	5.059641	4.079436	4.638848	4.313205	4.923783	3.352337
TIGD1	1553099_at	5.206942	5.13589	3.976568	4.031457	4.279664	4.116276	4.765229	4.929621	4.645125	4.653936	5.464217	5.219121	6.035646
ALKBH5	1553101_a_at	5.847568	5.49156	7.083224	6.499143	4.674163	3.991793	7.145397	4.673897	7.072955	7.471918	4.976101	5.801937	6.894374
CCDC69	1553102_a_at	6.771462	6.388745	7.294251	7.449562	6.278384	6.604515	6.900314	7.273844	7.283615	7.088803	6.880077	7.547842	4.93567
NFX1	1553103_at	9.50601	9.358794	9.359119	9.742073	10.01536	10.15225	8.834153	9.346903	8.90817	8.811264	9.220979	9.419709	8.253712
DSG2	1553105_s_at	2.332505	2.320446	3.431592	2.356933	2.343007	2.301483	2.940748	2.312232	4.144784	3.254767	2.303884	2.310186	4.198173
C5orf24	1553106_at	4.899026	4.148938	5.668029	4.988633	4.395701	3.650903	4.834848	4.14587	4.914232	4.479562	4.086008	4.013736	6.816053
C5orf24	1553107_s_at	5.084459	4.935033	4.461685	5.111536	5.231021	5.901688	3.434682	4.42933	3.685641	3.55332	5.738893	5.071531	3.992044
C5orf24	1553108_at	3.762521	3.592852	4.105557	3.470395	3.616217	3.770987	3.75938	3.447955	3.722886	3.825069	3.574702	3.732069	6.091766
KBTBD6	1553111_a_at	4.702346	4.347817	4.783441	4.71584	4.635426	3.234746	5.592863	4.983024	5.943588	6.027309	5.280015	5.23834	7.055786
CDK8	1553112_s_at	2.499502	2.471089	3.440161	2.50372	2.464234	2.446394	4.66107	2.471513	4.591588	4.986659	2.457133	2.465082	6.503453
CDK8	1553113_s_at	6.434437	6.328442	5.965403	6.33346	5.636318	4.9067	7.588774	6.783733	7.37071	7.502756	6.371412	6.594894	8.055923
PTK6	1553114_a_at	2.161145	2.152393	2.067979	2.126131	2.221653	2.337419	2.152571	2.14953	2.137412	2.111515	2.150779	2.187681	2.028735
NKD1	1553115_at	2.448416	2.440581	2.42312	2.425506	2.45276	2.429418	2.424051	2.433666	2.422429	2.402994	2.423756	2.433337	2.348595
STK38	1553117_a_at	2.817728	3.157674	4.617888	2.822651	2.821493	2.686286	4.303454	2.297849	3.915024	3.819506	2.788098	2.770225	5.565416

THEM4	1553118_at	4.31386	4.048679	6.355647	6.105657	4.99203	5.301157	4.866366	4.182372	4.840973	4.936126	5.876746	4.354417	4.57988
CLSPN	1553120_at	2.438987	2.412972	2.365112	2.39131	2.433465	2.390932	2.412092	2.412315	2.398795	2.392337	2.399278	2.407231	2.283992
RBAK	1553122_s_at	2.955637	2.866853	2.855723	2.908042	2.943392	2.825766	2.879642	2.892442	2.877077	2.84872	2.917805	2.911953	3.402689
WDR62	1553123_at	3.406155	3.172598	3.061368	3.25965	3.339493	3.167694	3.406247	3.979012	3.323077	3.59759	3.8775	4.082466	2.856719
WDR62	1553125_x_at	3.333311	3.204999	3.237456	3.257833	3.322485	3.649502	3.295893	3.308131	3.39126	3.28065	3.354969	3.569482	2.855214
SLC39A12	1553126_a_at	2.731169	2.669636	2.612479	2.659053	2.712831	2.636377	2.671965	2.683051	2.668019	2.647899	2.667135	2.688829	3.858765
RNF168	1553127_a_at	3.361246	3.288563	3.199932	3.260484	3.348958	3.257148	3.276175	3.285623	3.276874	3.247459	3.285613	3.383365	2.854535
SVEP1	1553129_at	2.707371	2.643955	2.586541	2.624369	2.72038	2.783064	2.603365	2.63824	2.627183	2.593702	2.63002	2.637972	2.691642
LOC124216	1553130_at	2.478375	2.454927	2.453532	2.444539	2.516956	2.443692	2.444446	2.459927	2.442388	2.429915	2.442538	2.456261	2.328739
GATA4	1553131_a_at	1.92195	1.904145	2.36436	1.891688	1.902738	1.885602	1.901833	1.887538	2.055757	1.888702	1.881016	1.89687	1.850415
TC2N	1553132_a_at	3.180576	3.120172	2.980166	3.006081	3.1729	3.087228	3.098587	3.096105	3.073853	3.007901	3.075433	3.129231	2.889651
C9orf72	1553133_at	3.384352	3.800312	5.770247	6.049556	4.772351	4.984312	3.327084	3.336861	3.32336	3.301558	3.321838	3.348743	3.141229
C9orf72	1553134_s_at	2.510676	2.475932	2.461118	2.463854	2.497714	2.457445	2.466459	2.477367	2.463891	2.454728	2.459582	2.47451	2.340562
KCTD18	1553136_at	2.56302	2.531187	2.474247	2.41397	2.523769	2.489754	2.534007	2.511827	2.517275	2.498682	2.513313	2.50363	2.263283
KLF11	1553137_s_at	2.91157	2.895546	3.003402	2.973746	2.896127	2.87287	2.84889	2.934282	2.877476	2.872544	2.875181	2.886929	2.989649
ANKRD41	1553138_a_at	3.165622	3.097086	3.027394	3.089064	3.113698	3.060884	6.729829	4.784536	6.202781	6.577925	3.770571	4.066429	2.896671
PLXNA3	1553139_s_at	2.152103	2.141136	2.116421	2.127206	2.139939	2.125144	2.136457	2.1271	2.126226	2.123953	2.119416	2.131977	2.525474
PELO	1553140_at	3.658208	3.523845	3.458967	3.518024	3.711094	4.243204	3.53077	3.536757	3.489537	3.501416	3.539183	3.575796	3.279886
C13orf31	1553141_at	2.563028	2.537627	2.515399	2.516288	2.545388	2.629243	2.529378	2.536312	2.523597	2.516642	2.520439	2.53416	2.392671
C13orf31	1553142_at	3.595736	3.305185	3.373787	3.401178	3.73858	3.37866	3.356294	3.428232	3.447036	3.410639	3.546938	3.503446	3.145049
FLJ39653	1553145_at	5.538883	5.462768	4.96657	5.290231	5.943758	5.778962	4.44081	4.295171	4.573459	4.670024	4.884335	4.71593	3.91468
C3orf30	1553146_at	2.482145	2.444494	2.399341	2.440408	2.481607	2.412474	2.437849	2.445894	2.43474	2.425021	2.435014	2.457241	2.334431
RANBP3L	1553147_at	2.710311	2.669271	2.610985	2.658327	2.714804	2.634216	2.659659	2.674525	2.657752	2.637738	2.661366	2.690633	2.573051
SNX13	1553148_a_at	2.455058	2.424674	2.433416	2.411292	2.434	2.40253	2.432121	2.432552	2.694474	2.407304	2.41692	2.423995	4.077544
AOF1	1553150_at	2.548145	2.520606	2.498535	2.505693	2.544006	2.4949	2.515654	2.517535	2.508814	2.503334	2.511849	2.518145	3.537154
ATP6V0D2	1553151_at	2.303967	2.285258	2.269427	2.273037	2.300622	2.271488	2.286033	2.286461	2.274486	2.269102	2.282573	2.28142	2.223183
ATP6V0D2	1553153_at	2.566843	2.440284	2.468179	2.43191	2.543245	2.467239	2.479168	2.441573	2.477501	2.470773	2.466903	2.468193	2.146074
ATP6V0D2	1553155_x_at	2.517021	2.436093	2.446528	2.419589	2.542974	2.446347	2.470451	2.457012	2.467956	2.452581	2.467909	2.52422	2.398045
LHX4	1553156_at	2.167799	2.16145	2.140372	2.1458	2.156878	2.144638	2.1583	2.15932	2.15345	2.143422	2.144118	2.149347	2.110299
LHX4	1553157_at	2.134908	2.095079	2.128548	2.062345	2.413997	2.061771	2.073581	2.081017	2.065951	2.05843	2.063923	2.075042	1.998385
C3orf34	1553158_at	2.499652	2.489532	2.478969	2.474772	2.488863	2.465959	2.535047	2.494573	2.912476	2.966179	2.47957	3.06275	4.137261
DNAH11	1553159_at	2.893688	2.871839	2.84445	2.844826	2.875948	2.854692	2.867495	2.871874	2.856485	2.851997	2.858036	2.866313	2.817683
C19orf55	1553162_x_at	2.863304	2.813025	2.78818	2.802577	3.020221	3.386028	2.786312	2.810891	2.796271	3.189577	2.78773	2.803523	2.821582
ADPRHL1	1553163_at	3.323399	3.191725	3.30015	3.240425	3.315623	3.306371	3.254995	3.297872	3.31103	3.30051	3.317742	3.308004	3.535783
AP1GBP1	1553165_at	4.643736	4.290843	4.731392	4.926336	5.188781	4.71469	4.288216	4.294048	4.28379	4.277233	4.373246	4.089971	3.713892
CDH24	1553166_at	2.01572	2.007062	1.983492	1.98823	2.006982	1.992206	2.00621	1.99194	1.98934	1.992069	1.991287	1.997738	1.970506
SEPSECS	1553167_a_at	6.425227	6.210538	6.748377	6.977156	6.612029	6.476409	4.315697	4.582084	5.005623	4.806926	4.076816	5.09389	2.960303
GRIK5	1553168_at	1.740195	1.732314	1.712986	1.718039	1.731618	1.721271	1.728377	1.730368	1.725468	1.715225	1.711149	1.722918	1.680946

C20orf75	1553169_at	2.037924	2.024889	2.001089	2.00657	2.026165	2.005502	2.01703	2.024548	2.010028	2.011627	2.170715	2.015983	1.953867
C20orf75	1553171_x_at	2.063556	2.045443	2.024109	2.028957	2.048834	2.031807	2.041135	2.043724	2.031708	2.03028	2.171734	2.038527	1.968119
ZNF777	1553172_at	6.150252	6.04849	5.619769	5.781588	6.277435	6.200162	5.574543	6.799133	5.934911	5.854978	6.412078	6.493772	5.087599
JPH2	1553174_at	1.675685	1.666981	1.649969	1.654569	1.663007	1.653591	1.66616	1.666384	1.657472	1.652045	1.650927	1.657668	1.617325
PDE5A	1553175_s_at	2.471279	2.439741	2.389504	2.428571	2.518171	2.426557	2.438188	2.443738	2.432186	2.422341	2.434147	2.441623	2.726806
SH2D1B	1553176_at	2.55334	2.549916	2.480298	2.50816	2.538464	2.490907	2.512988	2.520376	2.508911	2.4954	2.504718	2.516693	2.356845
SH2D1B	1553177_at	2.135546	2.118312	2.093597	2.09929	2.11953	2.106702	2.109362	2.116642	2.101427	2.103514	2.098606	2.108449	2.070721
SSTR3	1553178_a_at	1.720347	1.712946	1.68501	1.696167	1.706315	1.699219	1.709904	1.709277	1.701721	1.693504	1.695371	1.70391	1.687165
ADAMTS19	1553179_at	2.361512	2.345766	2.299958	2.316675	2.347823	2.330717	2.336125	2.343982	2.356833	2.324262	2.365349	2.339345	4.185742
ADAMTS19	1553180_at	3.642031	3.36274	3.421368	3.483916	3.772519	3.323809	3.432142	3.422407	3.494557	3.449941	3.652912	3.586281	3.741947
DDX31	1553181_at	2.066201	2.055394	2.034499	2.041121	2.054793	2.039387	2.053795	2.052448	2.048916	2.042641	2.036741	2.046175	2.000256
UMODL1	1553183_at	2.439646	2.412104	2.383796	2.393385	2.423459	2.392127	2.397041	2.411942	2.396058	2.388727	2.395819	2.406458	2.264406
RASEF	1553185_at	8.442023	8.459407	8.629749	8.687686	8.53038	8.464024	8.204502	7.822893	7.701833	7.738764	7.947385	7.746383	8.372953
RASEF	1553186_x_at	8.435495	8.466417	8.606955	8.518354	8.582315	8.602307	7.849437	7.371761	7.504073	7.800147	7.964183	7.598701	8.482445
PARD3B	1553188_s_at	2.746232	2.720544	2.692021	2.700729	2.738063	2.699836	2.709523	2.708206	2.704085	2.661272	2.699401	2.713042	2.621026
PARD3B	1553190_s_at	2.254847	2.264611	2.258016	2.234398	2.285556	2.242586	2.253612	2.262796	2.2405	2.234452	2.248407	2.261875	2.168958
DST	1553191_at	2.67563	2.627942	2.566662	2.614088	2.665452	2.595589	2.621474	2.631714	2.618709	2.599493	2.618197	2.635092	2.506405
ZNF441	1553192_at	3.625072	3.518023	3.485219	3.481523	3.583475	3.509432	3.526952	3.377991	3.624082	3.520236	3.519427	3.559191	3.973454
ZNF441	1553193_at	4.360463	3.825883	3.74656	4.100679	4.769591	4.070287	3.891962	3.871236	3.928254	3.856468	3.971211	4.019323	3.752715
NEGR1	1553194_at	2.687498	2.666416	2.595101	2.651265	2.692248	2.649019	2.644358	2.657984	2.647639	2.626754	2.651411	2.674549	3.622079
FCRL3	1553196_a_at	2.418348	2.389215	2.366294	2.370314	2.39026	2.368789	2.381448	2.387181	2.371817	2.358122	2.370847	2.381032	2.247309
WDR21C	1553197_at	2.518576	2.486122	2.431822	2.469221	2.510703	2.459408	2.475258	2.486388	2.470971	2.462182	2.473071	2.487367	2.369909
WDR21C	1553199_at	4.03687	3.729689	3.476108	3.665307	4.001244	3.492483	3.627338	3.591603	3.643889	3.570527	3.856769	3.953998	3.096154
STOX1	1553202_at	2.802548	2.760267	2.669852	2.743734	2.784122	2.728107	2.84286	2.764362	2.776745	2.833715	2.757586	2.77062	2.517851
C20orf200	1553204_at	2.752308	2.711861	2.677756	2.700844	2.739662	2.684127	2.710484	2.692168	2.703348	2.680856	2.70386	2.715225	2.661198
C20orf200	1553205_at	2.277438	2.271011	2.20898	2.247949	2.25635	2.252093	2.265966	2.263582	2.256008	2.24937	2.245452	2.290622	2.211391
ARL10	1553207_at	2.96862	3.160335	2.666867	2.909048	2.976091	2.913336	2.921253	2.935513	2.917038	2.854466	2.91286	2.922498	2.755394
ARL10	1553208_s_at	2.008126	1.98692	1.964254	1.971088	1.99115	1.974387	1.982232	1.9797	1.977821	1.965759	1.969091	1.979272	1.940968
TMEM118	1553209_at	2.304204	2.27517	2.239331	2.256311	2.274804	2.248728	2.271399	2.273705	2.26638	2.261796	2.257764	2.269736	2.204225
ANKFN1	1553211_at	2.086059	2.065587	2.041912	2.047794	2.069027	2.047813	2.058541	2.062786	2.049836	2.043552	2.048708	2.05768	2.029282
KRT78	1553212_at	3.244235	3.287114	3.223591	3.290418	3.394587	3.28946	3.299487	3.646413	3.318763	3.689002	3.302354	3.34475	2.846832
KRT78	1553213_a_at	1.95549	1.942289	1.921196	1.925414	1.95332	1.924808	1.936886	1.939318	1.926779	1.920926	1.920714	1.933361	1.873101
CCDC7	1553214_a_at	3.943547	3.594013	3.528785	3.726825	5.318483	3.585483	3.672406	3.636271	3.753123	3.682519	3.976888	4.015476	3.229779
CCDC7	1553215_s_at	3.603239	3.487778	3.44684	3.605738	3.627268	3.362325	3.425128	3.404206	3.400381	3.423599	3.474676	3.532411	3.212153
ZNF41	1553216_at	3.061717	3.00448	2.953587	3.035374	3.051518	2.9488	3.046425	3.017306	3.149956	3.271905	3.045221	3.082542	3.907372
ZNF41	1553217_s_at	3.107011	2.988413	2.915079	2.989694	3.111291	2.929591	2.998492	3.004019	3.015287	2.967097	3.021321	3.108202	3.089644
ZNF512	1553218_a_at	3.165912	4.078211	4.649919	3.819221	3.047755	3.334948	5.278047	3.327733	5.34531	5.044197	3.397246	3.325661	7.889025
AMMECR1	1553219_a_at	1.986721	1.973791	1.952987	1.957382	1.978394	1.956311	2.449095	1.970987	2.710709	2.154985	1.955441	1.964788	2.061555

ALS2CR13	1553220_at	2.082307	2.051419	2.03157	2.036917	2.050725	2.041387	2.046121	2.050706	2.037385	2.0375	2.033655	2.043374	2.291995
ZNF583	1553221_at	2.115756	2.100831	2.080728	2.087571	2.105238	2.078204	2.099893	2.098324	2.094614	2.085143	2.214166	2.090619	3.734269
OXER1	1553222_at	3.638576	4.056513	2.542131	2.886372	3.389788	2.889192	2.5667	3.309264	2.327996	2.324245	2.279994	2.527396	2.114345
LUZP1	1553224_at	2.467299	2.431929	2.409755	2.409861	2.45487	2.413618	2.417848	2.427494	2.417322	2.40901	2.414265	2.431916	2.281249
ZNF75	1553225_s_at	3.23131	3.203747	3.186858	3.179704	3.271155	3.137503	3.191673	3.188968	3.200795	3.190351	3.177139	3.191517	4.459609
TMEM83	1553226_at	2.341125	2.314055	2.272988	2.291886	2.336275	2.299592	2.300083	2.312498	2.298809	2.283537	2.295232	2.308389	2.185743
BRWD1	1553227_s_at	4.457977	4.4654	4.948842	4.332245	3.429651	3.038691	3.887756	2.957684	4.171991	4.292506	3.327763	3.04295	4.548586
CCDC89	1553228_at	3.350857	3.256074	3.117302	3.226506	3.362895	3.207976	3.209511	3.256685	3.237539	3.221997	3.270605	3.322995	2.984104
ZNF572	1553229_at	2.836653	2.763187	2.888652	2.760989	2.78012	2.762134	2.769208	2.77902	2.864265	2.926653	2.762693	2.797267	2.703072
FAM82A	1553232_at	2.953704	3.196616	2.868427	2.931969	3.227697	2.922654	2.904827	2.939355	2.938699	2.89725	2.953863	2.934055	2.565431
ADAMTS18	1553234_at	2.951191	2.920976	2.811806	2.896982	2.929265	3.224147	2.888534	2.922727	2.901738	2.888501	2.910812	2.912757	2.830234
PCDHAC1	1553235_at	1.970968	1.960228	1.931829	1.942978	1.957767	1.950122	1.951171	1.960174	1.954629	1.937392	1.947902	2.021005	1.885355
PCDHAC1	1553237_x_at	2.708658	2.658564	2.639669	2.638573	2.674044	2.646204	2.656908	2.665699	2.648213	2.643544	2.651234	2.673889	2.420493
HIPK4	1553238_a_at	2.81289	2.740986	2.667925	2.684538	2.757645	2.932506	2.692938	2.713892	2.689005	2.701989	2.874467	2.732104	2.329153
FAM124A	1553239_at	2.502939	2.645248	2.455919	2.472423	2.48961	2.470644	2.666639	2.491434	2.474844	2.481147	2.497038	2.484871	2.406894
FAM77D	1553241_at	2.730259	2.706289	2.632355	2.681337	2.713669	2.880148	2.693314	2.702437	2.68596	2.665419	2.684183	2.713191	2.4899
ITIH5	1553243_at	3.697627	3.555931	3.19462	3.304196	3.448916	3.526283	3.18799	3.263659	3.233659	3.186171	3.493081	3.378372	4.262329
FANCB	1553244_at	3.451713	3.423533	3.37399	3.382062	3.517806	3.380367	3.421635	3.510338	3.926394	3.507163	3.627575	3.579521	3.263516
FLJ23356	1553246_a_at	2.022756	2.004538	1.985104	1.983462	2.011911	1.982664	2.000122	2.003034	1.991608	1.98032	1.986082	1.999036	1.998458
ZNF564	1553247_a_at	3.172188	3.074377	2.963259	3.120148	3.33457	3.028796	3.039825	3.125033	3.132148	3.090118	3.109291	3.152031	3.83044
CCDC57	1553248_at	5.442972	5.346392	4.827883	4.617796	3.594893	3.310554	3.19195	3.231429	3.186879	3.261894	3.358964	3.360862	2.914043
SMC1B	1553249_at	2.907081	2.863797	2.844036	2.845426	2.942845	2.846082	2.85887	2.849554	2.852584	2.844285	2.854234	2.86288	2.570799
BRWD3	1553252_a_at	3.08647	3.013853	2.993384	2.996775	3.619103	3.532976	3.028449	3.481899	2.969021	2.946309	2.990965	4.586056	2.977879
ASB16	1553253_at	1.59078	1.583055	1.566481	1.567672	1.574794	1.571339	1.580152	1.578925	1.565965	1.565599	1.56616	1.571537	1.553298
MAGEE2	1553254_at	2.383614	2.326411	2.272311	2.331444	2.374127	2.29685	2.339126	2.531027	2.334888	2.30864	2.33869	2.414301	2.359229
FLJ32214	1553255_at	3.490832	3.491663	3.40057	3.447415	3.487629	3.446555	3.427248	3.463134	3.445227	3.424668	3.458909	3.526481	3.265488
GAL3ST3	1553257_at	2.235296	2.214471	2.19133	2.195002	2.217556	2.196352	2.208191	2.214107	2.198573	2.192442	2.197704	2.208649	3.294205
FLJ30679	1553258_at	2.35358	2.334601	2.309631	2.316209	2.344142	2.314609	2.318897	2.330578	2.315488	2.309953	2.312736	2.32421	2.202147
ALS2CR11	1553260_s_at	2.607291	2.580884	2.520732	2.559438	2.615627	2.561642	2.573146	2.578068	2.567889	2.558121	2.569146	2.586681	2.402278
ALS2CR11	1553261_x_at	3.741256	3.454269	3.403602	3.589838	3.886023	3.540836	3.613693	3.615681	3.621478	3.531514	3.733607	3.860413	3.028632
UTS2R	1553262_a_at	1.036166	1.029351	1.050733	1.014054	1.018567	1.011235	1.027622	1.013747	1.023817	1.01249	1.006653	1.006878	1.027694
USH1G	1553263_at	2.440604	2.426138	2.416196	2.420713	2.446101	2.422574	2.439616	2.518495	2.435603	2.427732	3.006695	2.464524	2.432375
SYN1	1553264_a_at	2.622857	2.900418	2.595974	2.596867	2.660758	2.587778	2.605025	2.607883	2.601692	2.593022	2.585245	2.588729	2.683313
SLC23A3	1553265_at	3.022627	3.840619	2.887589	2.894238	3.455235	2.892701	2.893246	2.99648	2.872961	2.82472	2.896143	2.904142	2.767527
CNOT6L	1553266_at	3.165848	3.026922	2.956483	3.026516	3.19659	2.970938	3.036346	3.042487	3.048638	3.004984	3.084248	3.128071	2.715432
CNOT6L	1553267_a_at	2.960749	2.917321	2.901867	2.984436	2.989198	2.927941	2.92031	2.932275	2.911193	2.893295	2.907653	2.925598	2.832027
RHBDL3	1553268_at	2.043368	2.028165	2.006388	2.010871	2.029781	2.01289	2.02168	2.023765	2.01227	2.006571	2.006722	2.018239	1.947202
ZNF718	1553269_at	4.214327	3.972038	3.563225	3.593302	4.239914	3.939374	5.095285	3.875833	5.22104	4.80003	5.121194	4.044199	5.147335

DIP2B	1553271_at	4.035297	4.349012	3.540407	3.648123	4.218394	3.981849	3.788987	3.578756	3.74249	3.562058	3.754337	3.618054	3.386983
SLC36A1	1553272_at	2.087732	2.066747	2.038299	2.044279	2.070098	2.042798	2.055945	2.058533	2.051743	2.041957	2.044042	2.05398	1.981324
C6orf151	1553274_a_at	6.967262	6.721089	6.084876	6.575028	7.469407	7.18332	6.199804	7.138753	7.189115	6.782473	7.076084	7.63585	5.026583
ZNF560	1553276_at	2.469437	2.437064	2.387782	2.417492	2.446494	2.411852	2.425804	2.435906	2.421186	2.412258	2.423414	2.431734	2.287931
RTTN	1553277_at	3.078532	2.936748	2.870993	2.962549	3.079832	2.946394	2.9721	2.982519	2.96755	2.918752	3.008858	3.069333	2.694035
BTNL9	1553279_at	3.112394	3.047142	3.121424	3.083687	3.094426	3.094741	3.104854	3.094686	3.100924	3.08764	3.081551	3.108938	2.778296
CCDC139	1553280_at	3.912305	3.801197	4.812619	4.303541	3.752993	3.529835	3.674115	3.706351	3.636406	3.390221	3.500075	3.544615	2.728485
PLCXD2	1553281_at	2.028334	2.043812	1.988493	1.992128	2.011774	1.991403	2.004245	2.005955	1.998797	1.98744	1.992768	2.003717	1.933563
C21orf128	1553282_at	2.275228	2.250386	2.227388	2.233754	2.26188	2.233227	2.242961	2.250713	2.236169	2.228567	2.233983	2.247395	2.157291
LMLN	1553284_s_at	2.665097	2.64282	2.620186	2.628768	2.651134	2.645633	2.637532	2.647417	2.63579	2.644081	2.637761	2.678397	2.645015
RAD9B	1553285_s_at	2.731511	2.680154	2.581725	2.666425	2.724242	2.644042	2.678971	2.689137	2.675894	2.655145	2.673568	2.696836	2.527688
ZNF555	1553286_at	3.544189	4.314305	3.440253	4.008395	3.909691	4.152626	4.02725	4.902232	3.692298	4.419288	4.676613	3.786363	5.078714
C7orf51	1553288_a_at	3.223263	3.202478	3.178792	3.153884	3.242053	3.184622	3.188309	3.18567	3.192623	3.186558	3.177822	3.19991	3.118174
FLJ25006	1553292_s_at	4.195907	3.960904	3.904251	3.852156	4.052331	3.771888	4.121643	3.72797	3.989579	3.903278	4.497683	3.721954	6.160674
MRGPRX3	1553293_at	2.171259	2.153304	2.127291	2.136112	2.224864	2.134674	2.145456	2.153073	2.138318	2.130945	2.135256	2.147763	2.058755
ABCA13	1553295_at	2.700398	2.643309	2.593941	2.645103	2.692574	2.604422	2.642386	2.652328	2.638651	2.625725	2.638035	2.667351	2.454964
GPR128	1553296_at	6.254699	6.098172	7.216928	7.328612	5.186775	4.892546	2.377761	2.319121	2.877825	2.283275	2.291582	2.387025	2.159822
CSF3R	1553297_a_at	5.200698	4.909578	6.070602	5.157926	5.959181	6.082126	8.345695	8.629465	8.284187	7.344614	6.885226	7.467877	4.726301
C17orf77	1553298_at	3.595149	3.471404	3.311268	3.465149	3.882243	3.373059	3.474353	3.431432	3.473616	3.446861	3.533352	3.644895	3.061566
RP4-621O1	1553299_at	2.752359	2.702317	2.677757	2.706038	2.773463	2.689323	2.708421	2.723345	2.705592	2.692996	2.709028	2.74699	2.602036
DGKH	1553300_a_at	2.626206	2.605402	2.58541	2.582734	2.652455	2.584481	2.590169	2.601953	2.583236	2.582316	2.585468	2.601167	2.528329
TMEM182	1553301_a_at	2.805222	2.7468	2.693963	2.754846	2.815371	2.700177	2.746047	2.761203	2.764914	2.756804	2.753015	2.773451	4.925969
C16orf46	1553303_at	2.517957	2.509232	2.450438	2.483895	2.527039	2.498877	2.4965	2.505948	2.493047	2.472835	2.4895	2.502915	2.342285
LSM14B	1553304_at	2.621011	2.596552	2.575795	2.579536	2.593689	2.578648	2.591312	2.582137	2.580137	2.572513	2.578629	2.574798	2.39376
CASP8	1553306_at	2.329389	2.211178	2.096464	2.189533	2.220882	2.195263	2.163621	2.194452	2.196092	2.185606	2.194313	2.22625	2.105161
C20orf197	1553311_at	2.613247	2.574677	2.54221	2.551008	2.620421	2.555876	2.55835	2.570566	2.558851	2.524722	2.554773	2.59045	2.393336
SLC5A3	1553313_s_at	2.964475	2.526328	2.508534	2.699405	2.615447	3.811652	2.522996	2.530922	2.485824	2.510741	2.511923	2.536358	2.499916
KIF19	1553314_a_at	2.768744	2.726147	2.659008	2.708078	2.756428	2.715826	2.727955	2.7328	2.719086	2.707966	2.715846	2.733141	2.568352
SLFNL1	1553315_at	3.579607	3.326515	2.929696	2.927014	2.967474	2.812895	2.787332	2.841734	2.720724	2.923902	2.93212	2.791805	2.662339
GPR82	1553316_at	2.172446	2.154239	2.128046	2.135905	2.159717	2.130161	2.144351	2.154182	2.139079	2.130001	2.137804	2.148248	2.057966
GPR82	1553317_s_at	3.09897	2.990469	2.93689	3.421683	3.052683	2.907365	2.96587	2.994196	2.978096	2.969105	2.988641	3.019625	2.69857
RIBC1	1553318_at	2.090277	2.076755	2.055007	2.058302	2.077784	2.06014	2.070699	2.074348	2.06019	2.057625	2.055701	2.068131	2.00244
OXGR1	1553319_at	2.173712	2.160811	2.141888	2.142874	2.160258	2.146818	2.155208	2.160118	2.149714	2.139908	2.147407	2.155159	2.138043
CDC14B	1553320_s_at	3.244052	3.119951	3.05551	3.151598	3.302965	3.063691	3.103377	3.146222	3.147758	3.075718	3.154653	3.242163	2.849304
SULT1C4	1553321_a_at	2.553451	2.500348	2.502055	2.501806	2.582275	2.490477	2.72061	2.521669	2.539424	2.506101	2.503996	2.51741	4.272348
TEAD1	1553322_s_at	2.543793	2.550758	2.507694	2.510827	2.530833	2.510618	2.521315	2.527961	2.513002	2.505535	2.511576	2.522329	2.577521
CATSPER2	1553323_a_at	2.374103	2.354681	2.326468	2.33492	2.368294	2.33286	2.34504	2.352028	2.33859	2.330914	2.338505	2.348117	2.2213
POU5F2	1553325_at	2.605146	2.549845	2.532627	2.539241	2.579935	2.547113	2.544657	2.553983	2.540248	2.51926	2.539122	2.551339	2.380689

RXFP2	1553326_at	2.266164	2.23509	2.211161	2.215681	2.243985	2.219688	2.223972	2.234635	2.221758	2.210192	2.220142	2.229977	2.148362
C10orf30	1553327_a_at	2.447861	2.418996	2.401099	2.399235	2.427741	2.407877	2.403909	2.41697	2.400071	2.386487	2.400957	2.411055	2.271035
SLC18A2	1553328_a_at	2.193726	2.180058	2.167596	2.161789	2.185141	2.156775	2.183377	2.183947	2.169035	2.16219	2.165355	2.177232	2.099807
C7orf45	1553329_at	2.307618	2.276631	2.244567	2.255627	2.334914	2.25308	2.267162	2.274664	2.260454	2.252604	2.265018	2.275605	2.16035
C7orf45	1553330_at	3.090406	2.967768	2.934948	2.967713	3.085881	2.94904	2.998067	3.002038	2.994923	2.974198	3.002302	3.041681	2.676964
C1orf161	1553333_at	2.685973	2.635985	2.640989	2.639647	2.67472	2.608976	2.636277	2.651492	2.637575	2.626076	2.638364	2.648353	2.45618
FLJ34047	1553335_x_at	2.645588	2.604063	2.582433	2.582764	2.627988	2.585193	2.593594	2.59874	2.587377	2.580455	2.581783	2.608449	2.515845
MIER3	1553336_a_at	1.939193	1.926206	1.905248	1.910593	1.927127	1.912506	1.921678	1.924464	1.912179	1.910206	1.906193	1.917931	3.269743
C1orf55	1553338_at	2.638259	2.407904	2.612103	2.578376	2.629917	2.483868	4.687268	4.960303	4.73725	4.757633	3.410678	3.764648	2.867642
AMAC1	1553340_s_at	1.778132	1.766344	1.748082	1.751877	1.765358	1.752553	1.761836	1.762829	1.755901	1.751378	1.757334	1.755452	1.716274
UROC1	1553341_at	5.104219	4.971899	5.000128	4.340249	3.423062	3.298824	3.366721	3.104155	3.091602	3.122718	3.084998	3.09795	2.430506
PCDH15	1553344_at	2.452535	2.379024	2.333372	2.357952	2.394022	2.350383	2.366668	2.377622	2.364768	2.351091	2.367635	2.382483	2.251992
TNRC6A	1553346_a_at	2.06203	2.052076	2.034959	2.039964	2.046434	2.036249	2.050127	2.049751	2.040567	2.036173	2.033296	2.033804	2.047474
KCNA6	1553347_s_at	2.010209	2.002488	1.976623	1.983595	1.996871	1.981968	2.001623	1.99692	1.997968	1.984075	1.986607	1.995279	2.382559
NFX1	1553348_a_at	5.836279	5.679979	5.92782	6.367411	5.653148	5.664147	5.659269	5.514841	5.594064	5.662796	5.284578	5.377376	6.242666
ARID2	1553349_at	5.938833	5.974894	5.750896	5.88802	6.408882	6.055219	5.868154	6.313009	6.323465	6.502885	6.418988	6.065859	5.953719
FAM123C	1553350_at	2.505048	2.466974	2.414618	2.449442	2.476725	2.426624	2.448518	2.461813	2.450629	2.430644	2.442444	2.455693	2.317667
OTUD7A	1553351_at	1.870907	1.85834	1.840145	1.843415	1.857778	1.842958	1.854121	1.857666	1.846199	1.841018	1.838289	1.850826	1.809041
ERVWE1	1553352_x_at	2.13477	2.124792	2.103575	2.107725	2.124366	2.111534	2.118142	2.123398	2.086049	2.081252	2.105178	2.11817	2.075746
FLJ31958	1553354_a_at	2.51982	2.493722	2.478307	2.490122	2.530247	2.485423	2.490013	2.482505	2.497506	2.466311	2.485387	2.515087	2.49275
FLJ30672	1553357_at	2.214085	2.193318	2.162345	2.173678	2.219972	2.169665	2.178771	2.187819	2.175294	2.171012	2.176174	2.184516	2.711162
FBXL18	1553359_at	3.620624	4.316208	3.657748	3.661925	3.675911	3.897482	3.785499	3.697214	3.681896	3.688047	3.556645	3.667584	3.224703
FBXL18	1553361_x_at	2.375793	2.36587	2.340635	2.344818	2.363325	2.361734	2.353959	2.364827	2.340789	2.337091	2.344078	2.348339	2.332762
FLJ37357	1553362_at	2.551007	2.453486	2.402844	2.431679	2.480645	2.432802	2.441929	2.45257	2.442718	2.427493	2.440483	2.482172	2.309847
C6orf195	1553363_at	2.91412	2.855646	2.805125	2.844721	2.933119	2.824569	2.856063	2.861737	2.849835	2.830095	2.871768	2.888345	2.715097
PNPLA1	1553364_at	2.394226	2.333781	2.294586	2.321699	2.351793	2.325802	2.338097	2.342998	2.331808	2.314688	2.333088	2.355683	2.22985
DEPDC4	1553365_at	2.621973	2.599753	2.537672	2.58349	2.631698	2.582048	2.59565	2.601321	2.586657	2.545648	2.626379	2.596033	2.452454
ANKRD23	1553366_s_at	1.903224	1.896764	1.876403	1.880815	1.895825	1.879239	1.887808	1.892753	1.881151	1.871007	1.873326	1.88611	1.83137
COX6B2	1553367_a_at	2.041463	2.035049	2.011877	2.017577	2.027295	2.02439	2.031072	2.030916	2.019952	2.020245	2.018135	2.029053	1.99877
FAM129C	1553369_at	2.275959	2.259864	2.241239	2.242951	2.267057	2.242255	2.252263	2.255981	2.244369	2.238043	2.243031	2.2505	2.148488
EPHA10	1553371_at	2.416865	2.38858	2.363201	2.369981	2.400194	2.370962	2.375347	2.384747	2.37229	2.364922	2.367542	2.379338	2.284355
FLJ23865	1553372_at	3.410572	3.261014	3.18972	3.27384	3.623483	4.069748	3.286868	3.322198	3.29922	3.219097	3.310468	3.501891	2.849135
WDR64	1553373_at	2.509547	2.474237	2.44869	2.453276	2.493837	2.458995	2.460878	2.476568	2.459401	2.45111	2.454012	2.468279	2.318422
PRO2949	1553374_at	2.839138	2.758055	2.683956	2.732412	2.800781	2.733252	2.693297	2.758521	2.747209	2.727702	2.743042	2.763778	2.537191
BTBD9	1553375_at	2.405463	2.374043	2.346717	2.356225	2.384995	2.351194	2.364687	2.376395	2.360613	2.352244	2.360721	2.371066	2.288696
C1orf125	1553376_a_at	2.598083	2.562497	2.519235	2.545368	2.593774	2.542354	2.553747	2.564436	2.54893	2.539942	2.55015	2.570856	2.390301
C17orf66	1553378_a_at	2.532019	2.490931	2.56309	2.483168	2.520073	2.479756	2.492297	2.494859	2.483045	2.475733	2.479675	2.496681	2.385738
PARP15	1553380_at	2.533466	2.491264	2.467052	2.491109	2.521693	2.483539	2.499103	2.510645	2.495363	2.467135	2.56348	2.505137	2.393092

FLJ32810	1553383_at	3.013025	2.901623	2.830428	2.907772	3.058667	2.884243	2.908599	2.928187	2.913939	2.896641	2.926793	2.997078	2.628958
FLJ14816	1553385_at	2.237128	2.218157	2.191181	2.199864	2.220213	2.198266	2.209758	2.215534	2.201951	2.194819	2.198095	2.211802	2.110749
MFSD2	1553386_at	4.863327	4.249495	4.962094	4.896854	4.072047	4.453719	2.618215	2.628959	2.61532	2.596659	2.612026	2.629077	2.465867
ATM	1553387_at	2.658721	2.62225	2.594863	2.60743	2.770371	2.611857	2.60615	2.620175	2.603604	2.60872	2.657487	2.623983	2.525477
FAM26D	1553388_at	2.203684	2.180738	2.154503	2.161456	2.197421	2.160179	2.16965	2.176414	2.164565	2.156886	2.165307	2.17404	2.079595
NPHP3	1553389_at	2.57648	2.545262	2.516585	2.525624	2.564645	2.503171	2.530818	2.538942	2.526848	2.50007	2.524537	2.541139	2.388044
ATG9B	1553390_at	2.520444	2.486407	2.446186	2.465329	2.509931	2.459655	2.474649	2.486723	2.471592	2.458571	2.473534	2.502589	2.324841
CXorf58	1553391_at	2.209761	2.196713	2.172947	2.176835	2.202426	2.179963	2.184926	2.194903	2.183221	2.178373	2.192887	2.188326	2.094678
EFCAB3	1553392_at	2.582686	2.528799	2.479133	2.51863	2.570913	2.524241	2.527366	2.542969	2.523739	2.505493	2.535989	2.538682	2.410513
TFAP2B	1553394_a_at	2.53159	2.4895	2.450986	2.476666	2.518232	2.480662	2.485304	2.497039	2.481658	2.465319	2.481425	2.493079	3.108364
CD200R1	1553395_a_at	2.435368	2.431863	2.384833	2.382292	2.40167	2.367612	2.409355	2.401096	2.387344	2.379431	2.384203	2.394203	2.257203
CCDC13	1553396_a_at	2.805257	2.768102	2.723152	2.752601	2.789986	2.733149	2.763625	2.772063	2.755019	2.741869	2.75285	2.767143	2.532149
CCDC13	1553397_at	2.001106	1.997261	1.974818	1.980187	1.985939	1.979895	2.039876	1.994386	1.985483	1.981188	1.975306	1.984778	1.953652
C17orf69	1553399_a_at	2.732158	2.725318	2.705055	2.710424	2.732721	3.352372	2.730769	2.925204	2.718141	2.712039	2.709035	2.714785	2.690735
C17orf69	1553400_a_at	2.523595	2.703585	2.397802	2.425345	2.653434	2.447108	2.550534	2.575507	2.48765	2.847168	2.488787	2.588562	3.341808
MRGPRX1	1553401_at	2.838976	2.811482	2.767653	2.788637	2.850656	2.75508	2.796507	2.810491	2.792245	2.769357	2.794214	2.82955	2.648231
HFE	1553402_a_at	3.605309	3.341963	3.799402	3.909595	3.57695	4.0955	3.182103	3.025226	3.145722	3.302224	3.331285	3.354359	2.711168
RLN3	1553403_at	2.292249	2.262261	2.240763	2.245534	2.269739	2.247985	2.250787	2.262293	2.247283	2.237529	2.248092	2.256616	2.154556
CSMD1	1553405_a_at	2.809812	2.712422	2.673588	2.720887	2.789125	2.693823	2.739275	2.748924	2.734727	2.721149	2.737055	2.788276	2.614645
MACF1	1553407_at	5.474015	5.542298	4.935128	5.214011	5.241888	5.595365	3.609625	3.59607	3.210547	3.379169	5.200466	3.880639	3.369553
ADAMTS20	1553409_at	2.40735	2.377509	2.355943	2.357623	2.389202	2.364168	2.363529	2.375474	2.361945	2.357322	2.361596	2.370185	2.257568
ABCC12	1553410_a_at	2.22707	2.213073	2.186999	2.199151	2.212098	2.193016	2.209182	2.209796	2.196214	2.1898	2.197425	2.204085	2.14358
SALL3	1553411_s_at	2.198283	2.17852	2.149628	2.160324	2.183186	2.160255	2.166775	2.177313	2.16225	2.155498	2.162932	2.171371	3.719415
AGBL4	1553412_at	2.876784	2.842223	2.795807	2.821983	2.913973	2.836896	2.839629	2.852537	2.835358	2.82629	2.831236	2.882263	2.742513
SLC17A8	1553415_at	3.463116	3.354237	3.185877	3.412245	3.449054	3.399823	3.545105	3.404426	3.414557	3.381875	3.694874	3.42169	5.093142
C11orf44	1553417_at	2.232369	2.21341	2.188697	2.19424	2.221538	2.194756	2.206835	2.209862	2.197659	2.191366	2.197325	2.207542	2.134102
CNTNAP5	1553418_a_at	2.093563	2.079231	2.058285	2.062233	2.091416	2.061431	2.073827	2.076072	2.065089	2.057043	2.065595	2.073844	2.01589
FLJ32063	1553420_at	2.50755	2.479229	2.431754	2.469586	2.491673	2.474652	2.490899	2.491547	2.482688	2.46415	2.476573	2.51037	2.445102
A2BP1	1553422_s_at	1.921936	1.90952	1.887159	1.891669	1.908639	1.914229	1.902445	1.906279	1.893489	1.886963	5.052646	1.899904	7.897151
SLFN13	1553423_a_at	3.598748	3.435971	3.338571	3.366404	3.466488	3.405519	4.223665	5.413239	4.529597	4.856437	3.855925	4.466843	2.954253
C12orf40	1553424_at	2.726267	2.633608	2.598134	2.625662	2.684839	2.614313	2.634596	2.64601	2.630156	2.617047	2.63025	2.654658	2.422489
WDR65	1553425_at	3.19468	3.138118	3.088978	3.128152	3.184976	3.126969	2.90922	3.150332	3.131171	3.072116	3.121916	3.160715	2.863905
FLJ37543	1553426_at	2.754911	2.71286	2.63913	2.69394	2.706229	2.687512	2.701521	2.737311	2.697301	2.687891	2.69685	2.706982	2.489582
ADAMTS15	1553427_at	1.840763	1.828717	1.810857	1.815083	1.82861	1.814209	1.826438	1.827292	1.817103	1.817073	1.811565	1.820665	1.775959
RP3-398D13	1553428_at	2.208684	2.16466	2.117627	2.150994	2.196096	2.128735	2.16252	2.172575	2.159002	2.140785	2.156776	2.179969	2.01396
FLJ31713	1553429_at	2.608438	2.572869	2.557355	2.546794	2.569076	2.525666	2.559704	2.572449	2.563229	2.561934	2.558392	2.565208	2.392461
EDARADD	1553430_a_at	1.871951	1.859775	1.841847	1.843527	1.856851	1.849087	1.858415	1.85712	1.854001	1.847419	1.842218	1.85098	1.816523
OTOA	1553432_s_at	2.427481	2.405556	2.383202	2.388683	2.41022	3.080993	2.372181	2.40138	2.390686	2.38352	2.384894	2.395976	2.35047

C9orf93	1553433_at	3.160217	3.050259	2.948264	3.01961	3.162048	2.987243	3.032335	3.212292	3.040843	3.042612	3.064151	3.115663	2.707331
CYP4Z2P	1553434_at	2.467349	2.443019	2.399297	2.423383	2.478905	2.425447	2.432093	2.441934	2.428238	2.420778	2.426587	2.442247	2.294662
C18orf15	1553435_at	2.127664	2.109304	2.081379	2.093031	2.116225	2.169943	2.107895	2.128803	2.100187	2.088097	2.096797	2.10496	2.020681
MUC19	1553436_at	2.159949	2.137913	2.116096	2.119901	2.137763	2.120269	2.131808	2.135052	2.12152	2.115153	2.117388	2.129837	2.059418
KLHDC7A	1553437_at	2.276969	2.262551	2.245643	2.249679	2.262725	2.250103	2.259843	2.261353	2.243258	2.247195	2.242468	2.255472	2.210459
C11orf72	1553438_at	2.082379	2.074282	2.050669	2.057834	2.074304	2.054533	2.06485	2.068763	2.057994	2.050219	2.050099	2.062637	1.987865
C18orf16	1553440_at	2.470299	2.441134	2.396129	2.425173	2.465545	2.409207	2.434733	2.445053	2.431211	2.422567	2.428085	2.44141	2.29531
CNTNAP4	1553441_at	3.076563	2.976071	2.905025	2.97782	3.224436	2.972379	2.986749	3.020352	2.996359	2.984515	3.014473	3.05311	2.685272
CNTNAP4	1553442_a_at	2.62998	2.597024	2.539767	2.578938	2.615672	2.565491	2.590001	2.597633	2.585926	2.5743	2.582964	2.606078	2.587248
C8orf54	1553443_at	2.185915	2.163037	2.136939	2.144342	2.17067	2.143291	2.15008	2.161552	2.146416	2.139949	2.144222	2.155606	2.065422
C1orf127	1553444_a_at	2.073518	2.063934	2.046733	2.051864	2.063068	2.051454	2.062405	2.063278	2.056	2.049617	2.047884	2.055974	2.016502
FLJ37396	1553446_at	2.612664	2.561917	2.512732	2.562384	2.601077	2.560773	2.572296	2.578558	2.564655	2.556881	2.561599	2.573265	2.496728
AGBL1	1553447_at	2.210918	2.190544	2.156756	2.173006	2.199014	2.167685	2.179079	2.189757	2.184002	2.166017	2.17426	2.183651	2.163267
FLJ34503	1553448_at	2.188329	2.140462	2.115558	2.123076	2.164635	2.123267	2.128818	2.138717	2.135364	2.12034	2.126055	2.132391	2.071168
CCDC79	1553451_at	2.196667	2.160097	2.141947	2.138339	2.166171	2.140909	2.151999	2.158218	2.147218	2.141023	2.142973	2.15626	2.061062
MYO1H	1553452_at	2.201706	2.217758	2.160442	2.166632	2.184067	2.159366	2.175458	2.178657	2.165754	2.159153	2.159306	2.174254	2.086968
ASB14	1553453_at	2.384607	2.352138	2.334365	2.333897	2.37808	2.328258	2.334939	2.343525	2.329992	2.328911	2.330846	2.354569	2.22099
RPTN	1553454_at	2.366555	2.332714	2.296194	2.317461	2.364918	2.318447	2.326923	2.333012	2.321272	2.309892	2.322122	2.340983	2.207162
FLJ30719	1553456_at	2.084566	2.055201	2.031466	2.035106	2.058083	2.030066	2.04765	2.054534	2.040055	2.032407	2.039077	2.048663	1.972918
RP13-153N	1553457_at	2.64441	2.615754	2.612323	2.605702	2.660478	2.611437	2.618971	2.625504	2.615338	2.587548	2.618681	2.621471	2.437124
C10orf72	1553458_at	2.035177	2.004206	1.986739	1.988854	2.006471	1.986442	2.00227	1.996892	1.984962	1.986466	1.987934	1.99115	1.995137
FLJ39660	1553459_at	2.030086	2.016425	1.991786	1.999737	2.041613	1.996199	2.010695	2.01471	2.002853	2.003729	1.999267	2.007416	1.977886
C2orf61	1553460_at	2.089278	2.070926	2.044074	2.052337	2.0725	2.052109	2.064113	2.069822	2.055239	2.047286	2.052522	2.064605	1.998601
FAM9B	1553461_at	2.289122	2.270527	2.253877	2.250254	2.272804	2.711466	2.260184	2.299474	2.254252	2.245669	2.251041	2.266963	2.127717
FLJ40288	1553464_at	1.8936	1.886563	1.868433	1.869494	1.881095	1.868707	1.88169	1.885511	1.87084	1.864317	1.866782	1.880194	1.850858
CES7	1553465_a_at	2.612693	2.605483	2.631985	3.076932	2.61493	3.286943	2.471083	2.582993	2.56778	2.37706	2.571503	2.597927	2.446034
CXorf59	1553466_at	1.879643	1.868078	1.84856	1.853417	1.865977	1.850071	1.864615	1.865135	1.852801	1.851888	1.845561	1.857877	1.818909
FLJ32742	1553467_at	1.860449	1.846417	1.825194	1.828987	1.843686	1.82685	1.839849	1.841714	1.830829	1.824961	1.824029	1.834726	1.784324
HYDIN2	1553468_at	2.533349	2.502254	2.46328	2.478389	2.516028	2.493335	2.49141	2.50188	2.487917	2.475682	2.4849	2.496583	2.375868
DNAH17	1553470_at	2.439453	2.404489	2.377349	2.382015	2.4179	2.360282	2.391834	2.401752	2.389622	2.377448	2.392486	2.404028	2.260869
AMAC1	1553471_at	2.955685	2.910869	2.888773	2.896003	2.979422	2.897188	2.891149	2.897625	2.897059	2.885048	2.904706	2.921694	2.916508
FLJ32955	1553472_at	3.053646	2.94031	2.888938	2.930722	3.083215	3.082359	2.94466	2.951609	2.96184	2.920198	2.969987	3.005895	2.656106
ANKRD21	1553474_at	3.475125	3.323041	3.280443	3.332114	3.529866	3.297175	3.348507	3.220984	3.302847	3.314358	3.353771	3.645411	2.834131
PRO1483	1553475_at	2.438782	2.441817	2.376243	2.389876	2.426384	2.402291	2.412621	2.407449	2.395592	2.381463	2.394367	2.405044	2.266526
PRR10	1553478_at	2.155436	2.134955	2.074194	2.120822	2.133757	2.118893	2.118371	2.1309	2.118403	2.10245	2.114758	2.129676	2.018445
TMEM145	1553479_at	2.006372	1.991343	1.967853	1.972608	2.013156	1.972168	1.986007	1.987738	1.978018	2.016346	1.973906	1.989759	2.218419
C15orf32	1553482_at	2.738925	2.700842	2.669832	2.676511	2.727521	2.678518	2.687501	2.697714	2.68333	2.661021	2.683232	2.703805	2.485651
TSGA10IP	1553483_at	2.079723	2.056183	2.035822	2.085252	2.062831	2.047109	2.051266	2.058451	2.044791	2.042596	2.040557	2.050528	2.012553

FLJ32894	1553484_at	2.547272	2.510198	2.45824	2.491866	2.536209	2.493401	2.50214	2.512988	2.499152	2.490759	2.49677	2.510107	2.356998
CCDC140	1553485_at	2.701204	2.647794	2.603239	2.651201	2.669749	2.654216	2.658413	2.64216	2.651216	2.635034	2.645294	2.671775	2.628435
C17orf78	1553486_a_at	2.38571	2.363792	2.327319	2.344874	2.380804	2.331332	2.35521	2.362522	2.347073	2.339447	2.34627	2.356375	2.227812
TEKT5	1553488_at	2.181919	2.149148	2.104504	2.133124	2.165119	2.130847	2.142262	2.149689	2.13704	2.129928	2.131678	2.145323	2.010653
TEKT5	1553489_a_at	1.993794	1.985063	1.961116	1.968291	1.986225	1.964866	1.975483	1.979397	1.968132	1.96068	1.966784	1.972775	1.916776
KSR2	1553491_at	2.140042	2.104874	2.039104	2.100944	2.165718	2.098068	2.099213	2.118893	2.103547	2.095007	2.104316	2.112238	2.027864
PAX1	1553492_a_at	2.542716	2.528062	2.507756	2.512	2.532198	2.513964	2.52165	2.524325	2.515277	2.506513	2.534812	2.520226	2.440126
TDH	1553493_a_at	3.575056	3.467781	3.363647	3.517742	3.528617	3.314919	4.883656	4.643524	4.833847	4.886474	4.381239	4.273849	3.302866
TDH	1553494_at	3.043465	2.95822	2.869607	2.936056	3.036756	2.903318	5.61915	6.122897	5.812948	5.716432	4.713618	5.350217	2.652029
C12orf37	1553497_at	2.502279	2.470331	2.420199	2.450588	2.488587	2.446135	2.458089	2.468829	2.454385	2.446383	2.457418	2.464998	2.36255
SERPINA9	1553499_s_at	3.318159	3.505267	3.278657	3.28379	3.301519	3.766904	2.478705	3.289255	2.480654	2.530675	3.275541	3.19965	2.856538
FBXL21	1553500_at	2.78258	2.728041	2.685772	2.732177	2.930728	2.689276	2.746124	2.750363	2.743387	2.724971	2.744571	2.770474	3.140456
PALM2	1553502_a_at	2.551092	2.52123	2.472006	2.487791	2.547069	2.464091	2.505497	2.516334	2.502407	2.500669	2.502054	2.51722	2.455726
MRGPRX4	1553504_at	3.272179	3.230049	3.161446	3.200347	3.602148	3.366314	3.157888	3.210939	3.213585	3.164997	3.214718	3.227866	2.861246
A2ML1	1553505_at	2.144557	2.116028	2.090744	2.099035	2.128968	2.097204	2.109303	2.115902	2.102283	2.094294	2.098971	2.108611	2.036964
CPO	1553506_at	2.221101	2.1985	2.170784	2.179081	2.203535	2.178344	2.18491	2.196931	2.181398	2.173073	2.178998	2.190852	2.103218
GPR6	1553507_a_at	1.504137	1.496418	1.479083	1.482749	1.491426	1.481076	1.495096	1.495434	1.489468	1.48272	1.479365	1.487841	1.472841
MDS2	1553508_at	2.595978	2.531627	2.47692	2.523623	2.585239	2.550486	2.503173	2.549331	2.53395	2.812258	2.769966	2.560516	2.338452
RQCD1	1553510_s_at	2.389147	2.366777	2.360745	2.35123	2.356098	2.331546	4.197664	2.373203	4.158964	4.223821	2.357736	2.788288	4.280959
GJC1	1553511_at	1.35131	1.343947	1.328069	1.330187	1.337064	1.327543	1.343744	1.335158	1.341366	1.329793	1.324077	1.329767	1.338116
HOXC12	1553512_at	2.330738	2.307615	2.28591	2.292295	2.307532	2.291103	2.302145	2.305828	2.291977	2.28482	2.286591	2.30475	2.223273
VNN3	1553513_at	3.868213	3.792938	3.820482	3.829787	3.856039	3.775806	3.799877	3.830751	3.794758	3.771232	3.836345	3.862836	3.359659
VNN3	1553514_a_at	3.433725	3.373258	3.263952	3.402176	3.39281	3.192514	3.26561	3.375168	3.41946	3.34722	3.019212	3.130174	2.78138
MYEOV2	1553515_at	2.499174	2.47408	2.428618	2.436743	2.47311	2.448297	2.459173	2.468509	2.451677	2.459357	2.455805	2.463138	2.346629
FERD3L	1553517_at	2.150356	2.132288	2.10539	2.110729	2.136402	2.113983	2.123058	2.130039	2.116536	2.109611	2.114225	2.124474	2.061306
DEFT1P	1553518_at	2.791103	2.759991	2.740155	2.740469	2.810096	2.74609	2.755247	2.75902	2.747877	2.742799	2.742133	2.769281	2.528897
C21orf94	1553519_at	3.330232	3.204053	3.134571	3.194895	3.36367	3.21209	3.208968	3.219351	3.220316	3.181405	3.245567	3.301438	2.942304
DEFB104A	1553521_at	2.633362	2.574832	2.517386	2.560325	2.635577	2.579818	2.585993	2.581187	2.572835	2.576343	2.583361	2.59355	2.470945
NLRP14	1553523_at	2.232655	2.212517	2.189556	2.193776	2.241025	2.202359	2.214486	2.217531	2.2037	2.189145	2.197411	2.21034	2.151558
DGKB	1553524_at	2.881687	2.844827	2.79281	2.815343	2.951208	2.777657	2.84619	2.838384	2.832558	2.815108	2.833023	2.872126	2.581988
NLRP13	1553525_at	2.163243	2.148026	2.126239	2.13029	2.14896	2.129841	2.141875	2.146376	2.133339	2.125131	2.135268	2.142947	2.043539
NLRP8	1553526_at	2.442006	2.409703	2.363409	2.389433	2.412819	2.401664	2.397698	2.409201	2.394378	2.381077	2.392688	2.404685	2.265264
NLRP9	1553527_at	1.94667	1.927698	1.90879	1.909726	1.934891	1.906929	1.920519	1.924139	1.912562	1.909964	2.134138	1.9189	1.924288
TAF5	1553528_a_at	5.144545	5.190739	4.964112	4.831819	5.449491	4.728913	7.121158	6.563192	7.019027	7.255287	6.359016	6.238787	6.283409
TAS1R2	1553529_at	1.867665	1.854905	1.834033	1.836377	1.853394	1.836244	1.846751	1.849872	1.838274	1.830832	1.833765	1.844713	1.783867
ITGB1	1553530_a_at	6.858837	6.669113	8.018343	7.136996	6.763777	6.957314	8.762199	6.493815	8.960103	8.902634	6.829832	7.090299	10.76309
PCSK6	1553531_at	2.320154	2.297119	2.273877	2.287122	2.311024	2.277859	2.278848	2.291738	2.275135	2.266332	2.276218	2.286275	2.11394
ZNF341	1553532_a_at	2.337774	2.316047	2.297531	2.297167	2.31602	2.295392	2.316525	2.301115	2.298121	2.298347	2.294841	2.304697	2.241382

JPH1	1553533_at	2.651711	2.656186	2.595407	2.602388	2.623733	2.602671	2.612958	2.620598	2.605224	2.597693	2.600703	2.680895	2.997969
NLRP10	1553534_at	2.240301	2.205704	2.143465	2.188638	2.228479	2.194441	2.195458	2.202866	2.191485	2.180476	2.192791	2.20509	2.109847
RANGAP1	1553535_a_at	4.90949	4.409812	4.524549	4.278101	3.680558	4.374101	6.279854	3.895164	6.64947	6.75029	4.208117	4.903636	6.03397
KRT73	1553537_at	2.31287	2.273081	2.255289	2.259067	2.322288	2.228882	2.26656	2.272256	2.26186	2.25759	2.255587	2.277461	2.157394
KRT74	1553539_at	2.365676	2.428699	2.2999	2.320724	2.346115	2.32596	2.334313	2.334472	2.324379	2.308606	2.320226	2.331052	2.210421
SLC29A2	1553540_a_at	2.023946	2.013351	1.994987	1.999954	2.012074	1.998279	2.011578	2.011444	2.006201	2.003163	1.994223	2.004233	1.970266
LMX1A	1553541_at	2.055782	2.034815	2.011147	2.016806	2.034734	2.024233	2.026475	2.031979	2.019033	2.011571	2.01611	2.026676	1.954357
CCDC125	1553542_at	5.64717	4.814594	4.793599	5.732784	6.720781	6.935996	4.599557	5.482187	4.247312	4.549513	4.815318	5.017838	3.523534
GPR101	1553544_at	1.948318	1.933684	1.916013	1.919978	1.934621	1.900007	1.93127	1.930503	1.92538	1.915604	1.912658	1.927934	1.865399
ILDR1	1553545_at	2.327171	2.319134	2.298396	2.295604	2.318778	2.301246	2.313779	2.31689	2.30346	2.297185	2.298868	2.310632	2.252176
PRO2012	1553546_at	3.176381	3.035781	2.960891	3.036675	3.171132	2.997271	3.043783	3.054747	3.056655	3.01645	3.089893	3.144533	2.722575
VN1R2	1553549_at	2.164451	2.143472	2.115819	2.124191	2.14812	2.121661	2.132625	2.142563	2.127259	2.119441	2.128187	2.136003	2.047914
VN1R5	1553550_at	2.177863	2.155025	2.129294	2.137448	2.160868	2.136339	2.145337	2.153167	2.139964	2.132923	2.136653	2.146772	2.058929
TAAR8	1553552_at	2.205453	2.183134	2.151602	2.162569	2.187686	2.168573	2.173052	2.182739	2.171916	2.164769	2.165875	2.176396	2.100262
TAS2R39	1553553_at	1.948319	1.931944	1.911133	1.918698	1.931259	1.921194	1.927161	1.92978	1.919782	1.915693	1.913047	1.924811	1.882481
TAS2R38	1553555_at	1.982857	1.965125	1.944474	1.949241	1.965017	1.946345	1.957405	1.963228	1.948974	1.941858	1.946481	1.956804	1.892489
TAS2R40	1553556_at	2.337418	2.309412	2.283484	2.29336	2.328814	2.293042	2.300026	2.306248	2.292257	2.286409	2.290236	2.30284	2.185776
TAS2R41	1553558_at	2.081072	2.063981	2.042407	2.062789	2.065209	2.047192	2.058168	2.06292	2.049072	2.044639	2.046249	2.057232	1.981941
TMEM171	1553559_at	1.927389	1.915441	1.890799	1.900915	1.913293	1.898929	1.90813	1.907697	1.899004	1.891233	1.896094	1.900634	1.846923
VN1R4	1553560_at	2.330509	2.305266	2.267474	2.290067	2.323049	2.290291	2.301673	2.301637	2.292295	2.286685	2.291219	2.295756	2.178804
TAS2R50	1553561_at	2.142854	2.120583	2.097951	2.102425	2.121691	2.1002	2.114391	2.115594	2.104742	2.098615	2.098553	2.112817	2.028804
CD8B	1553562_at	2.417331	2.392625	2.34455	2.36459	2.408415	2.381457	2.379547	2.38444	2.369385	2.357814	2.370199	2.380434	2.227213
MACROD2	1553564_at	1.966744	1.949799	1.931982	1.934409	1.956485	1.932131	1.947073	1.946862	1.935805	1.930202	1.928667	1.94173	2.797486
DDAH1	1553565_s_at	2.732979	2.634886	2.568411	2.62364	2.827476	2.636479	2.733277	2.647455	2.628985	2.611673	2.635539	2.65568	3.117406
C12orf53	1553566_at	2.184252	2.176286	2.151959	2.156569	2.173458	2.163277	2.164848	2.173212	2.16056	2.152596	2.15818	2.165055	2.11903
ATP13A5	1553567_s_at	15.08705	15.1164	15.23637	15.32092	14.37593	13.96081	15.23074	14.12312	15.04064	15.05119	13.94518	14.08389	15.15982
HIST1H1T	1553568_a_at	2.047384	2.036813	2.011142	2.022774	2.035408	2.017459	2.035062	2.034034	2.030943	2.023985	2.020586	2.036254	2.01302
OAF	1553569_at	14.7794	14.78125	14.7936	14.85887	14.59421	14.36216	14.82238	14.03564	14.69967	14.68358	14.0836	14.20021	14.64514
OAF	1553570_x_at	14.78159	14.78631	14.73164	14.85471	14.5585	14.36739	14.79494	14.09565	14.63184	14.60007	14.18763	14.21894	14.61337
CYGB	1553572_a_at	2.133436	2.123588	2.099045	2.105248	2.123567	2.10709	2.121707	2.122244	2.107464	2.10381	2.105637	2.11727	2.07961
EFNA2	1553573_s_at	2.356674	2.332154	2.303557	2.311941	2.336839	2.315248	2.321769	2.328755	2.317376	2.309213	2.311466	2.32461	2.265483
IFNE1	1553574_at	2.096427	2.062142	2.044473	2.048936	2.070989	2.048661	2.060094	2.06611	2.052738	2.045432	2.048365	2.073156	1.989041
SPAG11B	1553579_a_at	2.434764	2.383458	2.341549	2.365467	2.423533	2.369981	2.37877	2.381416	2.372848	2.36403	2.369878	2.381081	2.246589
P18SRP	1553581_s_at	7.645692	7.864453	7.913575	8.294607	8.371716	8.238047	8.167204	9.238897	8.22869	8.155277	8.802926	9.071548	7.395698
SPAG11A	1553582_a_at	3.228675	3.1063	3.019317	3.117612	3.247601	3.194324	3.099844	3.095392	3.119238	3.07231	3.13458	3.207949	2.787021
THRSP	1553583_a_at	7.874968	8.038506	8.199165	7.56396	4.004663	3.183004	2.263124	2.273637	2.224507	2.374509	2.333042	2.231891	2.1724
hCG_19816	1553584_at	2.03752	2.025627	2.005395	2.007102	2.027264	2.005175	2.020772	2.026076	2.017203	2.013067	2.012697	2.019312	1.966643
MAGEA5	1553585_a_at	2.36441	2.350031	2.292882	2.322258	2.346701	2.334571	2.34267	2.345934	2.336141	2.3259	2.332303	2.340716	2.26573

LOC283932	1553586_at	2.03664	2.025742	2.005028	2.010414	2.026989	2.00918	2.020717	2.022829	2.011951	2.007613	2.003997	2.019125	1.897109
POLE4	1553587_a_at	5.882021	5.493459	6.410743	6.716061	6.629495	6.3851	6.727977	6.387335	6.815479	6.629775	6.38713	6.464819	6.809261
PDZK1IP1	1553589_a_at	2.096581	2.086458	2.062483	2.069524	2.09124	2.068357	2.090116	2.580151	2.243512	2.113262	2.542325	2.1725	2.287323
FAM27E1	1553590_at	2.42166	2.351407	2.311228	2.342587	2.355804	2.342276	2.348778	2.353932	2.340485	2.341503	2.337962	2.346871	2.312993
LOC727996	1553591_at	2.885276	2.874264	2.769732	2.852974	2.888845	2.806599	2.865273	2.917936	2.868607	2.815178	2.859569	3.021419	2.596418
LOC727996	1553592_x_at	3.482137	3.467673	3.42983	3.449978	3.515092	3.29201	3.470605	3.469945	3.454844	3.458563	3.448977	3.528307	3.263744
TAL2	1553593_a_at	2.639322	2.58565	2.535256	2.5751	2.610799	2.585319	2.584608	2.606528	2.580148	2.553715	2.972884	2.604138	2.975173
INSL3	1553594_a_at	2.085196	2.077935	2.048811	2.05553	2.071288	2.055827	2.072874	2.190866	2.075983	2.058335	2.055232	2.099419	2.081532
SYCP3	1553599_a_at	3.672095	3.530986	3.415218	3.513581	3.760096	3.619245	3.275508	4.054392	3.461087	3.41292	3.608159	3.670371	2.954416
TMIE	1553600_at	3.211062	3.108927	3.034008	3.083257	3.143144	3.068248	3.246186	3.127862	3.107529	3.139707	3.136677	3.143988	2.749371
TMIE	1553601_a_at	2.638051	2.538384	2.47837	2.518342	3.115326	2.645483	2.525564	2.53505	2.521693	2.511567	2.523996	2.532641	2.376931
MUCL1	1553602_at	2.220579	2.195705	2.178314	2.177626	2.202656	2.184595	2.19181	2.19508	2.179769	2.166277	2.179397	2.186917	2.155121
ARL6IP2	1553603_s_at	2.798494	2.787658	3.810103	3.164739	2.733151	2.706832	4.469777	2.785517	4.407112	4.157618	2.709071	2.741519	4.853954
ABCA13	1553604_at	3.260551	3.111853	3.077328	3.15106	3.353063	3.1186	3.15061	3.158318	3.163237	3.123588	3.185361	3.254183	3.045096
ABCA13	1553605_a_at	2.92032	2.788979	2.711909	2.779419	2.883749	2.761987	2.780769	2.805565	2.788601	2.754132	2.788942	2.983489	2.778159
C21orf109	1553607_at	3.656607	3.505942	3.343018	3.492462	3.857545	3.365195	3.465156	3.413551	3.492655	3.384466	3.572627	3.660911	2.977685
C21orf109	1553608_a_at	2.604604	2.586499	2.508882	2.546807	3.124609	2.520434	2.551265	2.568238	2.554144	2.521861	2.551341	2.614841	2.392323
FLJ33790	1553611_s_at	2.239587	2.213261	2.195357	2.198849	2.223024	2.198792	2.21026	2.217278	2.203975	2.196321	2.204298	2.212057	3.019906
FOXC1	1553613_s_at	2.712054	2.702232	2.688056	2.670969	2.778014	3.780913	2.649478	2.68056	2.550417	2.665035	5.264164	2.69429	6.315804
FLJ25694	1553614_a_at	2.518878	2.44918	2.410141	2.431636	2.467139	2.596204	2.439138	2.445142	2.43363	2.418659	2.447062	2.446498	2.368276
TRIM43	1553618_at	2.711761	2.650023	2.596522	2.641724	2.705416	2.648069	2.653828	2.664728	2.650085	2.630673	2.651397	2.676982	2.542584
TRIM43	1553619_a_at	2.455402	2.445888	2.416553	2.428827	2.447711	2.433065	2.433818	2.437703	2.429886	2.431124	2.428191	2.437724	2.350172
TRIM42	1553620_at	2.434644	2.406098	2.36792	2.386058	2.418626	2.378183	2.394355	2.40646	2.389809	2.380394	2.389113	2.399203	2.261616
FSIP1	1553622_a_at	2.500444	2.464328	2.418692	2.450771	2.48996	2.432334	2.459856	2.470896	2.454653	2.45383	2.461709	2.467527	2.361279
MGC15705	1553623_at	2.257789	2.231905	2.214259	2.218506	2.233501	2.216245	2.226772	2.231433	2.218774	2.208419	2.212099	2.22406	2.123325
FAM98B	1553625_at	3.800503	3.495658	3.475443	3.587368	3.875707	3.459463	3.586038	3.58376	3.639645	3.559095	3.732591	3.898355	3.118978
C17orf57	1553626_a_at	2.38257	2.33406	2.337236	2.345283	2.442059	2.341712	2.353239	2.355212	2.378909	2.386345	2.342766	2.354035	2.277947
C17orf57	1553627_s_at	2.751624	2.683404	2.943168	2.677859	2.857535	2.730401	2.672017	2.723473	2.707672	2.708882	2.698354	2.748386	2.488904
FAM71B	1553629_a_at	2.323429	2.311427	2.279872	2.298786	2.314799	2.286421	2.305939	2.309664	2.296257	2.296056	2.291201	2.310281	2.187414
NHEDC1	1553633_s_at	2.84107	2.909556	2.781947	2.80184	2.955876	2.797185	2.813869	2.821808	2.8011	2.793601	2.806111	2.814591	2.740947
FLJ40852	1553634_a_at	2.050368	2.032268	2.011843	2.017452	2.026686	2.01715	2.028356	2.094166	2.018961	2.016598	2.038817	2.025495	1.987219
TCTEX1D1	1553635_s_at	5.238097	4.891274	4.63334	5.013574	3.972001	3.412071	5.826776	5.920085	5.669668	5.53762	5.201287	6.286552	3.647234
TMCO5	1553636_at	2.299192	2.270881	2.234783	2.251577	2.281853	2.244212	2.263237	2.271448	2.256498	2.245781	2.257225	2.265101	2.154129
TMCO5	1553637_s_at	2.631591	2.609124	2.592047	2.592727	2.603896	2.589819	2.608095	2.668021	2.596058	2.593286	2.597417	2.605697	2.41397
XKR6	1553640_at	2.204264	2.166385	2.150134	2.155375	2.171258	2.199565	2.159883	2.17082	2.156526	2.15093	2.15211	2.162471	2.118401
TSGA13	1553641_a_at	2.603291	2.956582	2.527421	2.582875	2.584912	2.667164	2.545104	2.582278	2.577441	2.542037	2.574	2.575348	2.408858
C9orf163	1553642_at	1.985265	1.981655	1.959828	1.963512	1.971158	1.960025	1.975755	1.971033	1.962312	1.960404	1.954072	1.967538	1.904539
C14orf49	1553644_at	2.461426	2.432217	2.414186	2.427114	2.457974	2.42446	2.432701	2.435207	2.421072	2.533286	2.564315	2.431394	2.287711

CCDC141	1553645_at	3.016348	2.968504	2.917261	2.961011	3.01554	2.93591	2.970058	2.981778	2.965962	2.948368	2.966792	3.000446	2.906197
HDX	1553646_at	2.866537	2.786192	2.789378	2.7926	2.839953	2.793126	2.779419	2.804375	2.774213	2.743382	2.791412	2.803382	3.308353
CDYL2	1553647_at	2.449062	2.439729	2.419382	2.425024	2.43701	2.419945	2.432015	2.439469	2.427116	2.416702	2.418033	2.431413	2.397716
C18orf54	1553651_at	3.253034	3.097322	3.13124	3.181089	3.360631	3.153677	3.141505	3.201808	3.194323	3.195578	3.244392	3.29616	3.141057
C18orf54	1553652_a_at	2.648726	2.61756	2.57488	2.595361	2.650029	2.584024	2.609174	2.620249	2.605039	2.59538	2.601631	2.624976	2.504187
SYT14	1553654_at	2.401061	2.358539	2.325437	2.344632	2.428894	2.34823	2.359749	2.368155	2.356046	2.34058	2.358198	2.365908	2.59544
CDC20B	1553655_at	2.352983	2.325117	2.291905	2.306645	2.33477	2.300391	2.314359	2.324585	2.30971	2.30593	2.307021	2.317924	2.208798
TECTB	1553656_at	2.332048	2.323432	2.306531	2.519486	2.320501	2.330309	2.322659	2.322972	2.315448	2.310228	2.305879	2.314717	2.072012
VWA3A	1553657_at	2.544325	2.513938	2.495323	2.496951	2.533683	3.401556	2.512464	2.514119	2.49947	2.487245	2.499746	2.5122	2.349456
FLJ32575	1553658_at	2.72464	2.674873	2.612781	2.671126	2.699393	2.671752	2.682222	2.679399	2.682995	2.669918	2.663217	2.676381	2.220558
HUS1B	1553660_at	3.128918	3.068353	2.993673	3.039873	3.141396	3.063846	3.101737	3.110989	3.08032	3.056302	3.162325	3.192725	2.689492
HUS1B	1553661_a_at	2.563433	2.52952	2.487565	2.505503	2.552656	2.515912	2.520095	2.536834	2.516416	2.503579	2.520859	2.572124	2.393919
LOC653419	1553663_a_at	2.028674	2.082402	1.999166	1.974662	1.993082	2.010055	2.01973	2.019881	2.016548	2.008003	1.988665	2.01138	2.124169
CCDC34	1553666_at	2.811925	2.781867	2.762901	2.701244	2.801352	2.773655	3.742692	3.455249	3.215059	2.837777	3.737551	3.587323	2.787507
LRCH3	1553668_at	2.564707	2.524639	2.512338	2.475615	2.523086	2.492504	2.941311	2.528496	3.162256	2.664378	2.501969	2.510303	2.893255
INTS4	1553670_at	2.354025	2.332959	2.319237	2.300393	2.369287	2.323733	2.325537	2.335002	2.325926	2.321982	2.318406	2.345327	2.035434
ENAH	1553672_at	3.445814	3.424659	3.395833	3.66119	3.573848	3.401694	3.422216	3.41795	3.401894	3.373415	3.410649	3.415422	3.246553
STK35	1553673_at	2.363288	2.347022	2.327541	2.325691	2.34954	2.333061	2.343926	2.346601	2.335182	2.329465	2.328643	2.468424	2.281323
LRRC44	1553674_at	3.234687	3.167757	3.084754	3.15467	3.242393	3.236113	3.155161	3.167071	3.151073	3.125464	3.152364	3.207441	2.677698
KLC4	1553675_at	1.851125	1.83642	1.949397	1.843674	1.844916	1.827751	1.835098	1.832175	1.832244	1.825225	1.819578	1.826746	1.795536
TIPRL	1553677_a_at	6.705398	6.845838	6.688709	7.16653	7.093485	7.374079	6.547448	6.981453	5.851555	6.143171	7.518069	6.93946	7.171822
ITGB1	1553678_a_at	6.893092	6.927984	8.713787	7.594217	5.402794	3.951523	9.01872	5.102395	9.243159	9.141299	5.357647	5.646516	10.4608
VKORC1L1	1553679_s_at	2.834128	2.813705	2.803541	2.799772	2.841473	2.805037	2.814445	2.805918	2.80583	2.803738	3.112052	2.841502	3.598864
PRF1	1553681_a_at	2.322375	2.32046	2.466597	2.284842	2.307302	2.283886	2.296122	2.306993	2.240515	2.241562	2.274269	2.295863	2.310672
FBXL14	1553682_at	2.209936	2.17656	2.295196	2.167078	2.193069	2.163651	2.1705	2.17714	2.166193	2.155402	2.160001	2.171044	2.109418
FBXL14	1553683_s_at	3.039486	3.103152	3.370543	3.769549	4.199195	3.179293	3.064922	3.098426	3.0822	3.024859	3.035148	2.884142	2.933004
PPIL6	1553684_at	3.425129	3.288184	3.206745	3.348802	3.524109	3.419255	3.292053	3.301912	3.310606	3.267755	3.376533	3.346204	3.280157
SP1	1553685_s_at	2.638659	2.610024	2.604491	2.601219	2.621238	2.565743	2.704317	2.615997	3.351612	3.108672	2.596549	2.609849	5.347248
C18orf25	1553686_at	2.603073	2.583145	2.517772	2.563032	2.603232	2.57311	2.584595	2.587477	2.57415	2.642251	2.570939	2.59058	2.404944
METTL6	1553688_at	2.468971	2.284603	2.254237	2.264	2.303101	2.266491	2.272815	2.284212	2.266333	2.257897	2.269769	2.278782	2.186928
METTL6	1553689_s_at	4.031244	4.05045	4.074853	4.466979	4.716449	4.719805	4.792773	5.057406	4.894396	4.820714	4.956025	5.331713	5.380711
SGOL1	1553690_at	2.053454	2.047682	2.014098	2.019897	2.038951	2.014892	2.03837	2.481218	2.8637	2.077137	2.150256	2.941837	1.975792
B3GALNT2	1553691_at	2.978137	2.888711	2.814562	2.910958	3.055464	2.859336	2.938835	2.921725	2.913318	2.883398	2.921367	2.941914	3.482097
CBR4	1553693_s_at	9.333204	9.319607	8.087255	8.368757	9.311332	9.266631	7.898446	8.741865	8.083593	8.045386	8.375486	8.237988	6.672333
PIK3C2A	1553694_a_at	2.08465	2.063283	3.266613	2.072771	2.079962	2.054791	3.127886	2.061373	3.65165	3.540442	2.038495	2.056078	5.410675
NLRX1	1553695_a_at	2.675954	2.65932	2.649941	2.985053	2.665159	2.654982	3.139934	2.665116	2.669267	2.65334	2.607082	2.625101	2.622547
ZNF569	1553696_s_at	5.00136	5.232298	4.612405	4.761913	5.048991	4.816291	5.240401	5.696415	5.005942	5.146872	4.9588	5.30449	5.961674
C1orf96	1553697_at	3.494449	3.349061	3.288012	3.379134	3.56351	3.296757	3.358349	3.36493	3.366462	3.311524	3.408028	3.506931	3.204389

C1orf96	1553698_a_at	2.722383	2.674985	2.614401	2.65836	2.710602	2.638019	2.673803	2.674859	2.670464	2.647574	2.668975	2.697819	2.77101
DUSP18	1553701_a_at	2.428126	2.513892	2.362978	2.398792	2.654734	2.398137	2.379603	2.411821	2.377743	2.393054	2.39244	2.410023	2.265772
ZNF697	1553702_at	3.158119	3.304242	2.956983	3.085393	3.161601	3.452302	3.040471	3.119724	3.048914	3.04233	3.109285	3.114328	2.982406
ZNF791	1553703_at	8.89849	8.901444	8.194807	8.524525	9.335018	9.471625	8.167721	8.963122	8.061657	8.099507	9.186099	8.988943	7.588911
ZNF791	1553704_x_at	8.702225	8.812322	7.984023	8.60832	9.275386	9.546992	7.622529	8.653807	8.044155	7.760144	9.038153	8.883737	7.410127
CHRM3	1553705_a_at	2.365582	2.336466	2.298017	2.322663	2.349157	2.317331	2.327244	2.3372	2.321852	2.314601	2.321141	2.329276	2.580852
HTRA4	1553706_at	2.918418	2.84606	2.766216	2.82314	2.904791	3.427035	2.830508	2.842968	2.833838	2.806755	2.831709	2.843971	2.599511
MGC16075	1553708_at	1.891202	1.872132	1.855773	1.859504	1.873212	1.853921	1.860471	1.870286	1.855589	1.848112	1.849898	1.863817	1.816851
PRPF38A	1553709_a_at	4.944784	5.157988	5.776617	5.270446	5.599002	4.497469	7.422536	6.666008	7.241683	7.671337	6.648444	6.95443	7.519856
C4orf39	1553710_at	2.906172	2.879606	2.84751	2.855999	2.881177	2.857889	2.870193	2.838984	2.858858	2.83803	2.859806	2.876312	3.526025
C4orf39	1553711_a_at	2.578542	2.537702	2.495214	2.520154	2.626641	2.517553	2.539028	2.539591	2.527465	2.522868	2.523904	2.543231	3.644448
RHEBL1	1553713_a_at	2.010556	2.007365	1.983383	1.989088	2.004233	2.000938	1.99413	2.000947	1.999032	1.993286	1.988149	2.005554	1.989904
C16orf14	1553715_s_at	8.907999	8.937046	8.272449	8.472956	8.547527	8.72789	7.193749	7.671163	7.141239	7.087505	7.91268	7.357769	4.853786
KCTD7	1553717_at	2.855603	2.745023	2.652195	2.729051	2.756764	3.178852	2.739416	2.909482	2.727804	2.638288	2.726876	2.74408	2.741456
ZNF548	1553718_at	4.982842	4.862851	5.983301	5.572176	5.06741	5.487339	6.521284	4.863674	6.164816	6.030625	5.090411	5.672518	7.040055
ZNF548	1553719_s_at	5.040776	4.420671	5.355698	5.063162	5.279343	4.93091	5.213244	5.067689	5.286512	5.003458	5.012586	5.025835	5.817653
FAM123A	1553720_a_at	3.251284	3.172226	3.133635	3.156022	3.177816	3.165045	3.166079	3.176786	3.160976	3.151973	3.158606	3.1702	3.430271
RNF152	1553721_at	3.003399	2.893571	2.834366	2.958901	2.99512	2.854448	2.892592	3.00047	2.903354	2.849299	2.910897	2.950167	2.637323
RNF152	1553722_s_at	2.928299	2.933108	3.655028	3.422565	2.975342	2.929684	2.921336	2.878124	2.878985	2.866414	2.877519	2.888517	2.629045
GPR97	1553723_at	2.711277	2.66801	2.672	2.639249	2.685535	2.679005	2.687628	2.684369	2.671044	2.676663	2.673863	2.680317	2.554588
ZNF644	1553725_s_at	2.716604	2.678095	4.044938	2.671371	2.704608	2.590956	3.288353	2.687409	4.281553	3.106069	2.651637	2.664493	6.812203
C6orf170	1553726_s_at	3.416127	3.25394	3.350455	3.404935	3.527459	3.214638	3.308611	3.305402	3.371962	3.328171	3.402776	3.383334	4.291156
B4GALNT3	1553727_at	2.565149	2.539773	2.509872	2.519194	2.536267	2.513083	2.527069	2.534766	2.522828	2.513656	2.506739	2.528135	2.366642
LRRC43	1553728_at	1.876968	1.872644	1.850981	1.848147	1.858499	1.855052	1.868206	1.861373	1.864688	1.858049	1.852218	1.863069	1.867442
LRRC43	1553729_s_at	2.147942	2.127568	2.099401	2.110006	2.131212	2.107943	2.122735	2.124735	2.113121	2.104703	2.108944	2.122016	2.063323
LRRC43	1553730_x_at	1.90229	1.897263	1.875184	1.879627	1.889709	1.878127	1.89388	1.891608	1.889037	1.882839	1.873225	1.886527	1.833059
CCDC123	1553732_s_at	1.91957	1.911995	1.896043	1.88181	1.901731	1.894481	1.910563	2.789816	1.957748	1.947813	2.565056	2.009667	1.963193
AK7	1553734_at	2.819688	2.680807	2.696468	2.776527	2.797495	3.06008	3.760816	3.675379	3.870853	3.137075	2.966612	2.822477	2.652433
CCDC131	1553736_at	3.038148	2.911472	2.898941	2.94655	3.041726	2.915902	2.95914	2.959782	2.946771	2.941883	2.949356	3.067254	2.702132
IRAK2	1553739_at	2.729234	2.68514	2.611005	2.664419	2.71842	2.647116	2.672444	2.682563	2.670227	2.659911	2.670555	2.718636	2.473878
IRAK2	1553740_a_at	2.466799	2.454231	2.426601	2.438357	2.472229	2.415763	2.441125	2.451006	2.43394	2.434222	2.434579	2.44518	2.266949
C1orf211	1553741_at	2.579722	2.533454	2.48827	2.51512	2.604372	2.499788	2.520693	2.522921	2.51722	2.497769	2.515965	2.565609	2.362646
FAM119A	1553743_at	6.478692	6.749061	6.649297	7.143706	7.003017	6.481826	6.502528	6.474746	6.643643	6.508549	6.400424	6.306194	6.408081
C12orf64	1553746_a_at	2.632894	2.561792	2.508436	2.537382	2.585293	2.522221	2.548945	2.560507	2.545965	2.528605	2.543043	2.561855	2.432297
MGC16025	1553747_at	2.87398	2.757269	2.693146	2.740213	2.85007	2.72193	2.762293	2.764492	2.757113	2.73305	2.762234	2.787909	2.530498
FAM76B	1553749_at	3.068737	3.039689	3.071373	3.053341	3.051979	3.005896	4.299727	3.062334	4.318036	3.361692	3.076611	3.087546	5.676534
FAM76B	1553750_a_at	3.834618	3.584494	4.047888	3.500044	3.787377	4.49481	4.572105	3.70104	5.188428	5.087056	3.510942	3.743549	4.63556
C20orf165	1553752_at	2.291729	2.260717	2.234161	2.242707	2.26502	2.242442	2.246468	2.257874	2.24497	2.234277	2.241581	2.328001	2.167196

NXNL1	1553755_at	2.762485	2.756726	2.328917	2.738118	2.751496	2.783961	2.750604	2.791805	2.742403	2.738496	2.738754	2.743472	2.561154
C9orf70	1553756_at	2.004822	1.988931	1.96894	1.973355	1.989118	2.039728	1.984878	1.986204	1.975786	1.968618	1.974782	1.983391	1.966595
MCM9	1553759_at	3.770196	3.349976	3.29712	4.132696	3.712737	3.481034	3.917612	3.644725	3.700532	4.297311	3.857554	3.690585	3.135258
C22orf30	1553761_at	2.498169	2.48118	2.427117	2.462792	2.486161	2.463417	2.473438	2.482477	2.464243	2.459928	2.466982	2.475993	2.428529
JUB	1553764_a_at	4.660134	4.551802	3.689466	3.162261	3.713842	3.14677	5.712928	4.075657	5.778025	6.474716	4.540597	3.958911	6.577835
KLHL32	1553765_a_at	3.45082	3.283525	3.240621	3.265245	3.460439	3.264457	3.274924	3.325069	3.302814	3.293895	3.302379	3.339881	3.275033
DCBLD1	1553768_a_at	4.654053	4.032778	4.873227	5.95219	5.406533	5.816119	2.973024	4.284536	3.141637	2.780789	3.733947	3.892235	3.572306
SLAMF9	1553769_at	2.846655	2.825225	2.763306	2.797029	2.820701	2.812234	2.805923	2.814825	2.802785	2.78361	2.806999	2.81889	2.645127
SLAMF9	1553770_a_at	2.523491	2.46162	2.550945	2.455079	2.476869	2.434172	2.460484	2.473348	2.457729	2.440908	2.460271	2.46761	2.288023
GK5	1553772_at	3.816767	3.554171	3.449936	3.545127	3.781036	3.521547	3.560738	3.562118	3.551316	3.528735	3.579094	3.616381	3.323612
FLJ31715	1553775_at	2.845896	3.384131	2.804799	2.825383	2.937062	2.823315	3.582619	2.83479	2.826082	2.844842	2.816879	2.843464	2.785498
UBE2U	1553776_at	2.914941	2.829956	2.791275	2.820606	2.915878	2.787264	2.830291	2.845035	2.833996	2.806562	2.851233	2.872918	2.586521
WBSCR27	1553778_at	1.735976	1.724701	1.70616	1.709838	1.741826	1.771291	1.722002	1.725655	1.718196	1.705133	1.707025	1.719929	1.682763
MGC23270	1553780_at	3.101185	3.090736	3.007059	3.042474	3.087304	3.045421	3.07366	3.200741	3.254778	3.243047	3.077618	3.40027	3.014813
ZC3HAV1L	1553781_at	2.777498	2.708732	2.656089	2.711241	2.807575	2.674199	2.713058	2.722238	2.708454	2.687511	2.71193	2.737899	2.647411
RASGEF1B	1553785_at	3.421744	3.21573	3.397627	3.326976	3.85636	3.920579	3.2963	3.31605	3.184456	3.283851	3.238575	3.389652	2.899627
C11orf45	1553787_at	2.857374	2.995645	2.753943	2.780267	2.900533	3.169582	2.798411	2.800862	2.784916	2.751881	2.783589	2.833482	2.641415
C21orf58	1553789_a_at	2.134012	2.119002	2.10072	2.104497	2.123808	2.108266	2.31235	4.735228	4.115581	3.648545	4.68579	4.36845	2.026003
KIAA1109	1553792_at	2.847807	2.767232	2.704567	2.755567	2.873915	2.72891	2.769666	2.772435	2.757712	2.741589	2.770541	2.799732	2.567507
KIAA1109	1553793_a_at	2.474334	2.450691	2.413666	2.424418	2.460712	2.432642	2.436382	2.449506	2.428791	2.419827	2.434389	2.443756	2.346444
STOML3	1553794_at	2.717355	2.833523	2.56807	2.632236	2.67418	2.617294	2.645263	2.654441	2.641477	2.619988	2.648287	2.710938	2.473007
FLJ30594	1553796_at	2.272896	2.239037	2.213352	2.220498	2.247881	2.221683	2.236296	2.239705	2.225637	2.221438	2.23339	2.235154	2.188306
FLJ30594	1553797_a_at	2.785541	2.72032	2.663322	2.730281	2.786408	2.683563	2.728012	2.735559	2.729321	2.716189	2.836834	2.756777	2.693305
FBXL13	1553798_a_at	2.550454	2.516073	2.468768	2.500595	2.538519	2.474713	2.508456	2.514937	2.50245	2.4839	2.500131	2.515262	2.368793
C15orf33	1553799_at	2.947593	2.843522	2.753247	2.827825	2.941428	2.789751	2.840839	2.836383	2.841859	2.81536	2.860359	2.897376	2.578881
C14orf126	1553801_a_at	4.762949	4.791117	5.56255	4.861328	4.718398	4.585086	4.977481	4.790969	5.155311	5.051384	4.662821	4.976193	5.94716
SOX3	1553802_a_at	2.34697	2.356995	2.322001	2.325723	2.329612	2.321524	2.338187	2.339405	2.324411	2.312736	2.315444	2.334161	2.209532
C17orf46	1553803_at	3.301028	3.273106	3.253063	3.255601	3.28883	3.235009	3.259602	3.269355	3.257945	3.247877	3.253614	3.263541	3.190198
C17orf46	1553804_a_at	2.92866	2.891445	2.835383	2.870205	2.942429	2.864	2.878243	2.891767	2.87482	2.848962	2.874469	2.886593	2.658671
C3orf49	1553805_at	3.267184	3.170627	3.12879	3.157544	3.27566	3.165997	3.137492	3.141137	3.160756	3.129823	3.170609	3.178912	2.798296
NKX2-3	1553808_a_at	2.406011	2.361184	2.320237	2.367557	2.389301	2.374925	2.383583	2.385303	2.370859	2.372693	2.413658	2.380122	2.298656
C9orf71	1553809_a_at	2.260502	2.235377	2.222588	2.215701	2.234273	2.217567	2.228633	2.313535	2.223137	2.215112	2.215622	2.226902	2.123137
KIAA1524	1553810_a_at	3.212415	3.14093	3.066475	3.12475	3.216116	3.051295	5.592742	5.594416	4.661708	5.326635	4.261991	4.245075	3.193543
MGC14436	1553811_at	2.420262	2.394104	2.374075	2.37713	2.40239	2.377944	2.38482	2.393374	2.381997	2.373868	2.379474	2.397235	2.253131
TLE6	1553812_at	2.585667	2.55037	2.51683	2.527406	2.558292	2.575848	2.538395	2.548605	2.53449	2.516414	2.531502	2.544142	2.442184
TLE6	1553813_s_at	2.095876	2.066376	2.054428	2.059186	2.154505	2.05821	2.070067	2.07407	2.061824	2.054102	2.052581	2.066162	1.998774
POM121L1	1553817_at	2.105026	2.088457	2.065697	2.069491	2.088372	2.070465	2.083287	2.085219	2.072613	2.066079	2.067101	2.080096	2.01996
POM121L1	1553818_x_at	2.245429	2.223217	2.186917	2.205243	2.25882	2.19532	2.217613	2.220387	2.206557	2.191349	2.203496	2.215842	2.112475

FLJ30092	1553821_at	2.145285	2.120607	2.091899	2.100155	2.118829	2.098522	2.11508	2.126551	2.106038	2.10177	2.103228	2.114235	2.027887
RTP1	1553822_at	2.69769	2.66121	2.595735	2.638079	2.699861	2.615666	2.651453	2.663181	2.647763	2.635893	2.647964	2.675897	2.462004
RTP1	1553823_a_at	2.644151	2.624695	2.597274	2.605796	2.627905	2.603806	2.614855	2.623745	2.607224	2.599336	2.611595	2.61826	2.488621
LOC146325	1553826_a_at	2.354591	2.347857	2.328823	2.330622	2.338956	2.333184	2.334608	2.337403	2.339339	2.330909	2.332271	2.330098	2.272494
FAM55A	1553828_at	2.529709	2.490009	2.46167	2.472217	2.501947	2.63308	2.47898	2.491392	2.477203	2.461707	2.475248	2.494089	2.35072
C2orf58	1553829_at	2.114648	2.093926	2.070484	2.076317	2.106793	2.074241	2.082957	2.091538	2.078124	2.070082	2.078302	2.087961	2.017637
MAGEA2	1553830_s_at	2.351052	2.324748	2.282112	2.301416	2.33466	2.304634	2.315326	2.323703	2.308934	2.301172	2.357544	2.318642	2.198649
APOBEC3D	1553831_at	2.24466	2.20694	2.15445	2.204798	2.219466	2.194613	2.207871	2.219642	2.198604	2.208474	2.207033	2.243673	2.111893
C21orf93	1553832_at	1.612442	1.605594	1.587089	1.591039	1.605938	1.590686	1.602222	1.592076	1.59803	1.591241	1.585148	1.584908	1.571606
FLJ35880	1553834_at	2.638974	2.59456	2.547202	2.581227	2.626722	2.562008	2.591555	2.602339	2.586956	2.577094	2.585116	2.615929	2.414742
FLJ35880	1553835_a_at	2.40725	2.37221	2.348022	2.355536	2.396132	2.354533	2.361964	2.373366	2.357785	2.347887	2.358439	2.373823	2.237751
PGAM5	1553837_at	1.957702	1.946172	1.923083	1.927563	1.943567	1.931889	1.938266	1.934914	1.929305	1.921955	1.9223	1.935481	1.873998
FLJ25371	1553838_at	2.476378	2.446346	2.414116	2.425622	2.458879	2.437423	2.436606	2.445777	2.431825	2.414551	2.429922	2.442157	2.320791
DKFZp761B	1553839_at	2.746771	2.731887	2.719028	2.714343	2.750216	2.723568	2.737487	2.723282	2.724216	2.720917	2.716437	2.758173	2.513958
DKFZp761B	1553840_a_at	2.628463	2.558672	2.455033	2.575987	2.606304	2.568491	2.586075	2.596679	2.547003	2.566916	2.579431	2.597495	2.348468
CXorf20	1553842_at	3.579533	3.408878	3.321492	3.419411	3.699468	3.359436	3.422869	3.423802	3.429653	3.384816	3.479886	3.543508	2.937083
C10orf67	1553843_at	1.989774	1.98202	1.964273	1.966853	1.99516	1.969899	1.978326	1.977453	1.975185	1.963698	1.961607	1.962378	1.965629
C10orf67	1553844_a_at	1.848089	1.837426	1.793758	1.816029	1.837222	1.823435	1.835408	1.836184	1.8332	1.823451	1.823126	1.893015	1.786086
C10orf67	1553845_x_at	2.382363	2.377518	2.357227	2.360539	2.370289	2.363684	2.345668	2.358792	2.367887	2.322353	2.358463	2.364794	2.292116
SPERT	1553846_at	2.140699	2.124216	2.097279	2.103514	2.128119	2.104044	2.113808	2.136048	2.113271	2.101552	2.129596	2.123378	2.04064
SPERT	1553847_a_at	2.294356	2.267501	2.243664	2.248891	2.268958	2.247839	2.259263	2.265872	2.250161	2.243838	2.25117	2.258141	2.18766
CCDC26	1553849_at	2.799779	2.744054	2.667087	2.714906	2.785301	2.700709	2.733124	2.741378	2.906539	2.718286	2.730042	2.754212	2.524681
C21orf88	1553850_at	2.415093	2.394366	2.371819	2.379216	2.398107	2.374404	2.380413	2.38841	2.379954	2.37757	2.372585	2.389037	2.249091
SPIC	1553851_at	2.585098	2.537876	2.512853	2.544983	2.582907	2.585197	2.547321	2.562318	2.551354	2.563671	2.52992	2.888137	2.376225
VPS13B	1553852_at	2.210704	2.188579	2.15615	2.171299	2.19032	2.170467	2.175647	2.186445	2.184947	2.165848	2.169062	2.180787	2.8746
P2RY10	1553856_s_at	2.175846	2.151969	2.122955	2.132126	2.154964	2.12953	2.144223	2.145784	2.135998	2.127879	2.136774	2.14537	2.063722
IGSF22	1553857_at	2.433277	2.410854	2.388909	2.396241	2.406341	2.751401	2.392432	3.117743	2.388212	2.390067	2.372964	2.404562	2.30141
ZBTB3	1553858_at	5.611362	5.091188	4.818913	5.076382	5.979152	6.355564	4.564123	5.249819	4.638214	4.873433	5.290915	5.826347	4.512623
TPH1	1553859_at	2.667778	2.667274	2.590868	2.608242	2.630758	2.607025	2.620575	2.622678	2.609157	2.602938	2.606981	2.61988	2.42701
DCST1	1553860_at	2.001798	1.985157	1.966136	1.96657	1.984938	1.973629	1.979148	1.981517	1.969332	1.960975	1.965075	1.975514	1.897243
TCP11L2	1553861_at	2.636329	2.61834	2.593582	2.57972	2.629292	2.554307	2.848781	2.610328	3.212087	3.212096	2.594153	2.605214	2.409231
C10orf64	1553863_at	2.375103	2.363025	2.332847	2.341191	2.371976	2.340975	2.353716	2.360848	2.349321	2.340966	2.357926	2.358766	2.182914
DKFZp761H	1553864_at	3.224286	3.136114	3.009436	3.126742	3.227953	3.039858	3.14563	3.092406	3.141589	3.062358	3.144449	3.200736	3.241743
DKFZp761H	1553865_a_at	3.182228	3.139532	3.147167	3.150785	3.213294	3.14944	3.144183	3.164879	3.151061	3.116166	3.14943	3.181753	3.108385
C5orf36	1553868_a_at	3.878639	3.611055	3.479726	3.664562	4.163175	3.678183	3.620492	3.667984	3.704076	3.631597	3.834657	3.922121	3.221896
SESN3	1553869_at	1.889774	1.866145	1.846785	1.848457	1.864711	1.849074	1.861482	1.861724	1.861667	1.844585	1.863304	1.856289	1.810598
C19orf34	1553871_at	1.593787	1.58712	1.570538	1.572834	1.578956	1.571373	1.58458	1.583383	1.57898	1.571266	1.565193	1.575409	1.560758
MGC33894	1553872_at	3.226427	3.198765	3.169531	3.20484	3.364216	3.205547	3.166562	3.672804	3.212019	3.1596	3.230052	3.652516	3.103838

KLHL34	1553873_at	3.526505	2.899845	2.747769	2.785331	2.897408	2.73635	2.786131	2.801182	2.810831	2.756792	2.842543	2.854327	2.623156
ZSCAN10	1553874_a_at	1.738214	1.731897	1.711471	1.715715	1.725691	1.714573	1.727763	1.726897	1.72199	1.715969	1.710217	1.72058	1.724571
ZSCAN10	1553875_s_at	1.265615	1.258318	1.241062	1.244475	1.250589	1.242214	1.257006	1.248615	1.253714	1.244438	1.238831	1.243291	1.248513
SAMD3	1553876_at	2.489047	2.458512	2.440101	2.434793	2.472117	2.432681	2.451265	2.457419	2.447048	2.43373	2.442753	2.454799	2.373679
GOT1L1	1553878_at	2.755821	2.670211	2.621037	2.658464	2.732945	2.652847	2.669767	2.678101	2.679428	2.638971	2.839019	2.707907	2.515838
GOT1L1	1553879_a_at	3.189938	3.186302	3.168267	3.154321	3.17783	3.109316	3.180924	3.47009	3.348631	3.143324	3.195246	3.180546	2.86529
C10orf91	1553880_at	2.858249	2.796416	2.728421	2.78529	2.920485	2.788261	2.798291	2.799159	2.816158	2.772804	2.799071	2.820644	2.561132
MGC16142	1553881_at	2.643435	2.60883	2.697555	2.591551	2.609844	2.595085	2.60059	2.608241	2.597528	2.547396	2.591551	2.617754	2.510407
ZNF99	1553883_at	2.96732	2.8604	2.819369	2.867765	2.948034	2.812827	2.880149	2.891467	2.877088	2.854564	2.876827	2.947923	2.692631
ZNF99	1553885_x_at	2.811269	2.731271	2.662747	2.711595	2.808973	2.684593	2.727423	2.735729	2.725719	2.700461	2.734354	2.756048	2.60791
CCDC108	1553886_at	2.547202	2.512659	2.47933	2.501984	2.534585	2.503518	2.500515	2.511953	2.50293	2.479766	2.500109	2.514064	2.405449
FLJ40235	1553887_at	2.447041	2.418754	2.37265	2.394385	2.436076	2.399527	2.4062	2.41512	2.402529	2.385743	2.399552	2.420277	2.283028
LDHAL6A	1553888_at	2.570165	2.521027	2.501617	2.505953	2.571489	2.503341	2.491601	2.51691	2.490793	2.488228	2.498303	2.529803	2.435119
MRGPRX2	1553889_at	2.033591	2.013522	1.989546	1.997568	2.016948	1.996761	2.007289	2.015245	2.000273	1.993584	1.997472	2.015175	1.937914
LOC126147	1553890_s_at	2.352286	2.343566	2.325514	2.329605	2.355489	2.331944	2.342174	2.341438	2.32626	2.321848	2.425826	2.3263	2.511627
KLF17	1553891_at	2.616567	2.588568	2.556647	2.57082	2.58207	2.565096	2.578975	2.583744	2.5745	2.569356	2.576267	2.589403	2.41354
CCDC105	1553893_at	2.191562	2.183032	2.140615	2.161571	2.173675	2.174695	2.474168	2.17723	2.176957	2.165872	2.158749	2.163973	2.132713
C11orf42	1553895_at	2.113946	2.108141	2.081838	2.091105	2.110876	2.088441	2.103097	2.103736	2.098228	2.08959	2.09086	2.101435	2.018135
DKFZp434L	1553898_a_at	3.214815	3.120984	3.06897	3.10597	3.223224	3.027098	3.077531	3.083311	3.104204	3.049881	3.128467	3.21039	2.775922
DKFZp434K	1553900_s_at	2.114215	2.105837	2.088622	2.090558	2.104947	2.093689	2.105211	2.107206	2.101644	2.092208	2.091838	2.245074	2.063279
ZNF486	1553901_x_at	5.351988	5.185712	4.825922	5.204438	5.461646	5.778485	4.96597	5.490824	5.1661	5.214174	5.749388	5.644893	4.965995
CXorf22	1553905_at	2.975943	2.865093	2.784624	2.831639	2.928889	2.844476	2.862399	2.872082	2.859515	2.847609	2.856009	2.928313	2.647361
FGD2	1553906_s_at	8.33487	8.231644	7.202572	7.498904	9.156237	9.95796	8.000378	8.410977	7.683286	7.232186	8.056137	8.256321	6.0761
EXDL1	1553907_a_at	2.747486	2.682712	2.640247	2.661552	2.779479	2.662211	2.671117	2.67904	2.663962	2.630772	2.667182	2.75553	2.444506
C10orf6	1553909_x_at	6.339785	6.499502	5.723265	5.974101	5.662722	6.226354	5.602814	5.924631	5.547004	5.487855	6.825359	6.078254	5.326032
NBPF4	1553910_at	2.507507	2.475322	2.468687	2.461816	2.533556	2.628048	2.471635	2.481664	2.464691	2.45421	2.467363	2.469882	2.325259
ZNF663	1553911_at	2.589214	2.542072	2.500037	2.521178	2.555907	2.52032	2.528695	2.539353	2.526271	2.51562	2.52837	2.538781	2.432908
FLJ35424	1553912_at	2.39762	2.365027	2.324052	2.350102	2.375908	2.347383	2.35631	2.365951	2.353281	2.336093	2.348436	2.370916	2.237698
MGC34800	1553914_at	2.261056	2.234968	2.201108	2.220371	2.249706	2.222779	2.231225	2.233504	2.219177	2.210371	2.214734	2.228884	2.125841
C10orf126	1553915_at	4.101336	3.920455	3.823281	3.946965	4.285349	3.865355	3.943938	3.952356	3.95444	3.917793	4.046938	4.154565	3.565893
PIP5K3	1553917_at	3.13419	3.111833	3.078634	3.092151	3.117127	3.090096	3.099176	3.153161	3.094842	3.086774	3.09239	3.106072	2.986102
C21orf129	1553918_at	2.374264	2.352104	2.332307	2.335978	2.358814	2.336659	2.345481	2.514982	2.496051	2.335384	2.339954	2.479988	2.28561
C9orf62	1553919_at	1.977752	1.952493	1.9267	1.93082	1.965698	1.930214	1.934193	1.945317	1.943371	1.92815	1.927868	1.942099	1.797913
C9orf84	1553920_at	2.756133	2.734861	2.705788	2.70981	2.739992	2.716949	2.729232	2.732899	2.720424	2.696629	2.717231	2.769226	2.659976
MGC34821	1553923_at	2.367394	2.34937	2.329151	2.330131	2.350556	2.334973	2.343161	2.344743	2.332741	2.33073	2.326469	2.338596	2.293884
FMO9P	1553924_at	2.499737	2.457417	2.412769	2.446647	2.481249	2.425035	2.444615	2.460877	2.447233	2.431975	2.444421	2.464606	2.308982
C7orf33	1553927_at	2.689518	2.600321	2.555677	2.59079	2.659391	2.573484	2.610604	2.619477	2.60653	2.589478	2.603979	2.651485	2.448537
ELMOD2	1553928_at	5.56979	4.921121	5.686963	6.092504	5.72309	5.569561	5.854981	6.205709	6.113865	5.820981	5.130419	6.287198	4.831592

ASAH3	1553929_at	2.57292	2.531437	2.485402	2.516843	2.55945	2.499236	2.537293	2.537859	2.521777	2.515398	2.520376	2.535907	2.380743
TAAR1	1553930_at	2.315969	2.287615	2.250929	2.267963	2.29873	2.262859	2.276595	2.276901	2.272158	2.263382	2.270634	2.281561	2.178827
LOC202459	1553931_at	2.729123	2.659988	2.662956	2.657615	2.70786	2.602011	2.674046	2.683996	2.66683	2.655394	2.674493	2.69463	2.546334
C18orf20	1553934_at	2.412951	2.379122	2.337351	2.360988	2.396503	2.352162	2.369216	2.381426	2.377556	2.355695	2.373484	2.378661	2.251216
MGC2848	1553935_at	2.318822	2.28585	2.254318	2.27473	2.410807	2.273861	2.279083	2.286259	2.271044	2.258617	2.273342	2.317834	2.122567
MGC2848	1553936_a_at	2.67713	2.66243	2.594597	2.639315	2.66217	2.639017	2.65711	2.657023	2.648638	2.643041	2.645022	2.657394	2.602008
STK32A	1553938_a_at	2.728444	2.682609	2.62472	2.662995	2.71892	2.652247	2.67524	2.686936	2.671618	2.653992	2.670319	2.694827	2.502221
LRRC28	1553940_a_at	3.484727	4.122135	4.494901	3.824186	3.394177	3.158646	5.696756	3.363574	5.110747	5.468325	3.347134	3.358435	3.539637
NS3BP	1553943_at	2.228041	2.212303	2.193785	2.188091	2.210195	2.187944	2.206981	2.205508	2.194975	2.184639	2.192505	2.198567	2.117561
MIA2	1553944_at	2.947114	2.693057	4.665159	4.328529	2.79101	2.409943	2.862912	2.668406	2.796081	2.67898	2.668884	2.637592	2.347872
GPHB5	1553945_at	2.351537	2.340897	2.305844	2.32693	2.336722	2.32535	2.330051	2.338309	2.326892	2.327417	2.319619	2.333929	2.211672
DCD	1553946_at	2.088716	2.067706	2.044591	2.049249	2.071466	2.050165	2.058063	2.063543	2.052263	2.044809	2.048167	2.060021	2.002222
EXOSC6	1553947_at	2.631203	2.587342	2.561208	2.529576	2.596346	2.445193	3.019362	2.595171	3.608879	2.8586	2.577875	2.582958	3.460541
LOC728056	1553949_at	1.941938	1.927235	1.903143	1.905948	1.923702	1.912328	1.918143	1.920463	1.90992	1.901953	1.903618	1.915025	1.856497
ZDHHC19	1553952_at	2.606854	2.665077	2.581668	2.585639	2.596189	2.586204	2.597579	2.597607	2.589412	2.58446	2.580641	2.589152	2.276874
ALG14	1553954_at	7.941282	7.895954	7.967623	7.983383	7.776887	7.45217	7.058297	7.097908	7.188571	7.181692	6.944677	7.266246	6.342815
CCDC128	1553955_at	8.476351	8.312787	7.425374	7.699672	8.154654	7.936243	8.637579	8.353972	8.760867	8.543821	8.037387	8.423495	8.013565
ALS2CR4	1553956_at	2.772858	2.745641	2.586508	2.618897	2.662593	2.608241	4.610396	2.980045	4.13726	4.471674	3.873073	3.217441	6.323003
ZNF564	1553957_at	5.788624	5.686853	4.731218	4.753252	5.801252	5.351928	4.760947	5.069477	5.018055	4.95698	5.138558	5.075108	4.62548
B3GALT6	1553959_a_at	3.674896	3.489602	3.658771	3.889079	3.619376	3.747889	3.548951	3.3804	3.618768	3.517681	4.272402	3.959508	4.627141
SNX21	1553960_at	5.744043	5.749538	5.351995	5.560635	6.353574	6.792379	3.817098	4.875583	4.131687	4.267243	6.363781	4.913272	6.482637
SNX21	1553961_s_at	2.447542	2.576093	2.413855	2.643695	3.077447	3.481716	2.429739	2.439703	2.423236	2.417573	2.799901	2.421621	3.611409
RHOB	1553962_s_at	3.687053	3.278791	6.72349	4.720018	2.977083	3.195129	2.945789	2.872842	3.282083	3.054627	2.876746	2.953924	6.240314
RHOB	1553963_at	2.705395	2.629137	2.580153	2.62084	2.708687	2.622569	2.635677	2.644027	2.633904	2.606866	2.634256	2.668882	2.453293
RHOB	1553965_x_at	2.879834	2.831634	2.825123	2.828994	2.855694	2.798181	2.830214	2.839985	2.827568	2.807234	2.821707	2.834762	2.784448
ADAT3	1553967_at	2.475494	2.46365	2.450182	2.454654	2.488532	2.447217	2.460834	2.45235	2.454463	2.448828	2.436552	2.445306	2.314543
ADAT3	1553968_a_at	1.542038	1.384652	1.513094	1.521749	1.529011	1.519305	1.538712	1.578464	1.534034	1.520428	1.622545	1.518311	1.543616
CEL	1553970_s_at	2.969763	2.760556	2.718452	2.728256	2.765614	2.751881	2.754161	2.758966	2.754966	2.736677	2.975978	3.525417	3.39918
GATS	1553971_a_at	2.566263	3.078643	2.512357	2.517909	2.531138	2.514289	2.63329	2.532222	2.522835	2.694166	2.849472	2.519327	5.248356
CBS	1553972_a_at	9.647217	9.621825	10.4504	10.556	9.956091	9.917056	8.607006	8.766344	8.862112	8.70501	8.461808	8.134454	4.813051
SPINK6	1553973_a_at	2.018706	2.005114	1.980867	1.985975	2.012032	1.990224	1.993658	1.999281	1.981067	1.980871	1.982345	1.992658	1.90343
LOC128977	1553974_at	7.119059	6.633725	6.619538	6.713909	6.978668	6.555447	7.134662	7.201004	7.363146	7.397966	7.300475	7.42453	7.457761
RP11-529I1	1553976_a_at	3.769153	3.566833	4.075852	3.55682	3.566413	3.591277	6.325079	6.071792	6.560334	6.850311	5.721836	6.156642	6.806948
CYP39A1	1553977_a_at	7.439054	7.511652	8.542972	8.149724	6.377076	5.034228	5.578157	4.299345	6.083091	5.531436	2.630888	2.668268	2.915496
MEF2B	1553978_at	6.264373	6.31276	5.91631	6.268796	6.190442	6.185033	6.196989	6.876786	6.17614	6.184563	6.76636	6.759092	4.330364
RAB7B	1553982_a_at	2.497278	2.456751	2.424618	2.439742	2.460946	2.445976	2.43928	2.598485	2.455926	2.433642	2.446125	3.018298	2.415206
DTYMK	1553983_at	3.543233	3.59334	2.898798	3.504841	3.563614	3.58355	5.739854	6.374994	6.008314	5.832311	6.496497	6.347791	3.208468
DTYMK	1553984_s_at	6.14853	5.734718	5.339167	5.603	5.44906	5.552398	8.437499	8.998493	8.893607	8.604352	8.988968	9.229375	7.246172

RASEF	1553986_at	2.254054	2.216634	2.418233	2.42512	2.24046	2.237162	2.206626	2.236962	2.214455	2.180501	2.199904	2.229587	2.948897
C12orf47	1553987_at	3.486759	3.409775	4.378152	3.444379	3.484758	2.926583	2.466734	2.570175	2.990635	2.928517	2.580243	2.577204	5.873813
ATP6V1C2	1553989_a_at	1.879801	1.870556	1.850304	1.855992	1.867795	1.858867	1.869536	1.866241	1.857393	1.859448	1.851632	1.858442	1.857087
C16orf79	1553990_at	2.100468	2.095914	1.950188	1.976785	2.092771	2.074249	2.895748	4.010275	2.078302	2.067035	3.097475	2.845329	2.225475
FLJ20674	1553991_s_at	2.522781	2.495907	2.469315	2.473547	2.502062	2.460325	2.477225	2.486559	2.473889	2.462256	2.469076	2.487452	2.363308
NBR2	1553992_s_at	4.548335	4.254212	4.862516	4.497297	3.785659	3.780052	3.80638	3.730146	3.695539	3.780052	3.590462	3.763843	3.671256
MED25	1553993_s_at	6.107724	5.672708	5.74177	5.955635	5.822986	6.561002	8.261264	8.810905	8.420428	8.53099	8.419163	8.844266	6.354039
NT5E	1553994_at	7.368454	7.249423	7.59036	7.948455	7.806362	7.916647	5.630048	6.125499	5.694916	5.919823	5.689851	6.394365	3.372281
NT5E	1553995_a_at	6.018901	5.694109	6.798491	7.096607	6.354262	6.059624	5.615138	4.756025	5.995033	5.892027	4.887389	5.475973	4.273069
ASPHD1	1553997_a_at	1.677742	1.67245	1.645364	1.657158	1.664837	1.661006	1.669447	1.751357	1.663787	1.675698	1.655131	1.860685	1.641946
DMRTC1	1553998_at	2.454832	2.421864	2.415066	2.413442	2.433313	2.421358	2.816271	4.550651	2.786021	2.723042	4.046613	2.816671	3.72295
TRIM37	1554001_at	3.889477	3.707985	3.740061	4.49735	4.115397	4.409597	3.692623	3.868782	3.772636	3.457777	3.85416	3.990261	3.130612
RGNEF	1554003_at	6.524627	5.839337	6.158391	6.346648	7.77266	8.012702	4.931611	6.057466	4.506498	5.100246	5.529997	5.330107	4.267866
RGNEF	1554004_a_at	3.920631	3.769093	4.122108	3.960089	4.052187	3.631001	3.650563	3.781804	3.388838	3.743499	3.534933	3.827887	3.628677
LLGL2	1554006_a_at	3.2159	3.21213	3.190584	3.195835	3.51711	3.207452	3.206741	3.22347	3.206826	3.197895	3.191115	3.199247	3.011899
OSMR	1554008_at	2.695129	2.516514	2.547406	2.465996	2.51452	2.496713	2.45784	2.471064	2.448337	2.442606	2.422608	2.454199	2.475663
NDST1	1554010_at	3.469568	2.797664	3.002642	3.138423	2.761642	2.756022	2.178387	2.830382	2.706821	2.632409	2.588822	2.798592	1.896552
RSPO2	1554012_at	2.467668	2.405603	2.366067	2.389124	2.438146	2.373175	2.394352	2.408539	2.39332	2.377706	2.399371	2.423878	4.40693
CHD2	1554014_at	6.31524	6.310864	6.150765	6.316827	6.369039	6.453994	6.320718	6.681574	6.282937	6.228407	6.055215	6.607782	4.799248
CHD2	1554015_a_at	3.271062	3.248583	3.262231	3.241549	3.289702	2.848195	3.578515	3.247905	3.904538	3.965781	3.053804	3.471554	3.750298
C16orf57	1554016_a_at	4.760867	4.949353	5.03055	5.201354	4.825649	5.722898	5.762957	5.601828	5.790109	5.913401	5.628097	5.680476	4.598369
FLJ39378	1554017_at	2.272361	2.255887	2.214763	2.319666	2.255666	2.236361	2.237885	2.252755	2.24062	2.235204	2.596445	2.246771	2.216221
GPNMB	1554018_at	4.422498	3.781443	3.564735	3.859479	4.766891	6.145289	3.731474	3.545663	3.565911	3.677496	3.904249	3.719627	5.651154
C6orf182	1554019_s_at	3.757198	3.510846	3.596921	3.607709	3.81759	3.442711	4.81359	4.300233	5.086974	4.694736	3.907115	4.364889	4.452737
BICD1	1554020_at	2.390646	2.371789	2.330446	2.348812	2.372605	2.347239	3.15117	2.374676	3.600355	2.68256	2.678702	2.487001	2.640238
ZNF12	1554021_a_at	8.724305	8.558526	8.281016	8.310793	8.60629	8.003268	8.576325	8.381043	8.609021	8.572444	8.331336	8.241762	8.876196
CCDC13	1554022_at	2.471083	2.41245	2.369039	2.404826	2.46198	2.380352	2.403055	2.411603	2.400379	2.384301	2.396572	2.46028	2.26566
CCDC13	1554023_s_at	2.948905	3.108089	3.204464	3.096593	3.112941	3.096428	3.100233	3.156786	2.998421	3.079799	3.090432	3.110875	3.04927
MYO10	1554026_a_at	2.331154	2.153467	2.13941	2.140994	2.213596	2.144833	2.13157	2.135789	2.142777	2.100708	2.138278	2.141853	2.137189
SLC4A4	1554027_a_at	3.54103	3.154699	4.918787	4.702043	3.220907	2.849157	2.875605	2.868752	2.871219	2.844543	2.869293	2.892682	2.891887
KIAA0372	1554029_a_at	2.696186	2.535343	2.984824	2.662829	2.676855	2.352752	3.150926	2.981196	3.091762	2.978166	2.753116	3.10496	4.992889
ARSB	1554030_at	3.707704	3.933821	3.50587	3.705411	2.717651	3.489366	3.631587	3.579687	3.562695	3.563053	3.507555	3.759189	2.917574
ARSB	1554032_at	2.260465	2.235653	2.215866	2.217949	2.242845	2.2213	2.23259	2.23728	2.223876	2.216841	2.219312	2.232019	2.12428
FRMD4A	1554033_at	2.940563	2.897836	2.831718	2.858714	2.937861	2.856677	2.893479	2.900141	2.886975	2.867471	2.885627	2.930591	2.695461
FRMD4A	1554034_a_at	2.38275	2.352272	2.313115	2.331363	2.366739	2.323827	2.342147	2.353074	2.338315	2.326707	2.340324	2.348303	2.236785
ZBTB24	1554036_at	3.469908	3.286528	3.360506	3.713246	3.807991	3.400444	3.390759	3.403532	3.395167	3.373469	3.391034	3.423279	3.080666
ZBTB24	1554037_a_at	2.977766	2.910353	2.848458	2.924627	3.051876	2.910075	2.917468	2.997661	2.921545	2.864877	2.928333	2.989465	2.639179
LARP2	1554038_at	2.733393	3.07779	4.168515	4.015488	3.655722	3.646492	2.493637	2.517346	2.4404	2.478658	2.505419	2.524226	2.320015

C14orf159	1554039_at	2.741933	2.722327	2.578091	2.630507	2.725699	3.607404	2.698087	2.859386	2.696877	2.685857	2.805194	2.762826	2.295145
C20orf141	1554041_at	2.825334	2.783085	2.704323	2.756609	2.83341	3.045024	2.767027	2.776945	2.768775	2.743783	2.802058	2.793974	2.572255
C20orf141	1554042_s_at	1.792158	1.781533	1.759748	1.764721	1.776828	1.765331	1.780206	1.7797	1.773642	1.768687	1.765922	1.923659	1.725742
MRAP	1554044_a_at	2.349617	2.338797	2.292771	2.3198	2.336141	2.319728	2.331166	2.336104	2.321814	2.319363	2.319817	2.327754	3.918399
ZNF24	1554045_at	4.161567	4.102222	3.933823	4.445047	4.531111	4.742972	3.200045	3.530248	3.380178	3.304601	3.221931	3.196746	3.822573
TXNDC9	1554047_at	3.49304	3.083433	3.044001	3.377911	3.468995	3.297386	3.362494	3.555051	3.391459	3.339117	3.512913	3.487146	3.293554
WDR42A	1554049_s_at	3.129705	2.962305	2.782841	2.823636	2.912001	2.854151	2.782118	2.840203	2.822014	2.85428	2.82317	2.845275	2.741853
SMPDL3B	1554050_at	2.508205	2.790906	2.447575	2.367421	2.854004	2.48248	2.495605	3.102293	2.48519	2.48469	2.481132	2.970867	2.443689
CNOT1	1554052_at	2.583757	2.550929	2.554488	2.53913	2.571742	2.559643	3.029856	2.620397	2.85557	2.55826	2.54372	2.601172	2.557581
SPTLC1	1554053_at	4.752284	3.884417	4.71639	4.821037	3.893434	3.69289	3.825017	4.028458	3.614586	3.97325	3.710122	3.721627	3.958767
LOC645676	1554057_at	6.241259	6.000091	6.151869	6.152995	6.222553	6.211459	6.234104	7.177285	6.452331	6.219316	7.139416	7.304871	5.724781
SETMAR	1554059_at	1.897556	1.896918	1.860642	1.875729	1.88288	1.881894	2.190313	1.898245	1.890402	2.367633	1.875082	2.561329	1.88155
SETMAR	1554060_s_at	2.76861	2.747622	2.717822	2.73328	2.748342	2.726463	2.736079	2.746331	3.006536	2.729119	2.726206	2.746296	3.111467
XG	1554062_at	3.425613	2.735422	2.713552	2.71119	2.778738	2.806351	2.726291	2.932998	2.969978	3.522263	2.76138	2.821402	2.6102
C8orf76	1554063_at	2.425161	2.405678	2.388152	2.393537	2.410024	2.39335	2.388253	2.405294	2.377754	2.390443	2.380393	2.442702	2.32595
FAM125B	1554065_at	2.539437	2.505498	2.614322	2.497009	2.522762	2.475222	2.509613	2.512234	2.496781	2.499749	2.489596	2.501991	2.36898
FLJ32549	1554067_at	7.799674	7.647003	8.617822	8.613706	7.819446	7.191313	5.932138	6.021682	6.2639	6.377409	5.472222	5.571846	4.532722
FLJ32549	1554068_s_at	4.328902	4.039028	6.001613	5.474958	4.037707	3.902748	3.91588	3.886185	4.011426	3.861074	3.791217	3.929238	3.493637
EPHA8	1554069_at	2.547282	2.524376	2.495917	2.484398	2.531302	2.502855	2.511718	2.52475	2.50789	2.495131	2.510398	2.517366	2.426983
CCDC67	1554071_at	3.306576	3.121955	3.080427	3.160312	3.396686	3.09985	3.153871	3.180493	3.164769	3.122941	3.236255	3.244151	2.857432
CCDC67	1554072_s_at	3.135677	3.001914	2.995479	3.001503	3.142479	2.946815	2.990124	2.995544	3.010969	2.997346	3.045013	3.089474	2.828587
SLFNL1	1554074_s_at	2.865842	2.851152	2.798008	2.831284	2.859396	3.11099	2.842286	2.851152	2.821442	2.825661	2.832745	2.835189	2.740174
TMEM136	1554076_s_at	1.820545	1.812138	1.781685	1.804944	1.808179	1.802238	1.985107	1.811622	2.004031	1.803862	1.79773	1.825991	1.838089
TMEM53	1554077_a_at	6.922804	6.891809	7.40936	7.082202	7.054948	6.575526	5.856239	5.543528	5.626348	5.364505	4.5845	5.454005	3.598105
DNAJA3	1554078_s_at	9.399943	9.200903	9.248664	9.200933	8.980213	8.615068	9.152645	8.841698	9.345978	9.27295	8.898685	9.01183	7.679583
GALNTL4	1554079_at	4.924101	4.839156	4.763982	4.597157	3.767602	4.127568	2.737481	2.739541	2.728083	2.490082	3.718864	2.734115	4.489367
RQCD1	1554080_at	4.496554	4.662776	4.049928	4.314934	3.321807	3.967833	5.109737	4.490776	4.940282	5.712147	4.435581	5.092111	4.675077
NOL9	1554082_a_at	4.864999	4.839973	4.820883	4.827429	4.362303	4.818241	5.874379	5.280642	5.904849	6.009103	5.188032	5.695919	6.227617
NOL9	1554084_a_at	4.777365	4.417458	4.25474	4.002537	4.778883	4.814928	4.584249	4.999561	4.581464	4.535074	5.383148	5.344072	4.569909
DDX51	1554085_at	4.970389	4.923726	4.76783	4.902153	4.927636	5.080616	4.956484	5.485945	4.906161	4.898143	5.275823	5.112814	3.524623
TUBGCP3	1554086_at	1.747266	1.73265	1.721892	1.725745	1.736911	1.724496	1.737989	1.736275	1.733384	2.100491	1.717878	1.729677	1.68778
SBDS	1554089_s_at	7.668601	7.406117	9.343354	8.956578	8.221046	8.181074	8.465727	7.249773	8.352983	8.280836	7.168924	6.911722	9.063532
TIRAP	1554091_a_at	2.059942	2.049051	2.270073	2.155956	2.049921	2.028279	2.035512	2.040061	2.03172	2.027462	2.02871	2.233108	1.964263
SNAPC5	1554093_a_at	6.129387	5.994107	6.795984	6.412038	6.8422	6.740279	6.584845	6.579486	6.980544	6.856458	6.680477	6.818715	6.392403
ENTPD5	1554094_at	5.841781	5.559173	5.254118	5.472812	5.556985	5.508293	4.251166	4.360045	4.238492	4.238566	4.237928	4.706762	3.007162
RBM33	1554095_at	6.450894	6.402454	5.875995	6.356012	5.771069	5.523444	5.03701	5.763488	5.166724	4.989321	5.255073	5.548331	3.6958
RBM33	1554096_a_at	4.47631	4.333901	4.199722	4.668541	5.261926	4.664194	3.580677	4.766323	4.183842	4.060141	4.430118	4.809601	3.122209
LOC554202	1554097_a_at	2.238904	2.214536	2.201165	2.199129	2.228805	2.205915	2.203628	2.210572	2.201054	2.188419	2.194724	2.20402	2.158536

SPIN3	1554098_at	3.610692	3.347109	3.369029	3.46363	3.544545	3.476366	3.488826	3.490235	3.373445	3.457132	3.517935	3.573021	3.345196
SPIN3	1554099_a_at	2.523727	3.398122	2.621939	2.412526	2.810647	2.281992	2.3572	2.301205	2.414722	2.413343	2.418205	2.40095	3.516047
TMTC4	1554101_a_at	2.152827	2.144307	2.135128	2.328501	2.14699	2.122031	2.135796	2.139739	2.128358	2.131193	2.122342	2.132904	3.380256
TMTC4	1554102_a_at	3.232271	3.057191	3.447573	3.041433	3.049807	3.092593	3.44079	3.321851	3.892282	3.850972	3.371639	3.561078	4.29474
TMEM185A	1554105_at	4.090745	4.276189	4.023621	4.020664	4.042806	4.026082	4.038164	4.617272	4.029105	4.013607	4.334726	4.072901	5.204253
ALS2CR16	1554106_at	6.380207	6.165963	7.030006	6.923841	6.115055	4.722636	5.8821	4.612978	5.831724	5.50503	4.994095	5.325008	5.814773
ALS2CR16	1554108_at	3.187511	3.321203	3.077635	3.211051	3.215694	3.176445	3.530276	3.183769	3.179842	3.225011	3.399327	3.627669	4.035174
CDCP1	1554110_at	2.4894	2.422076	2.352054	2.406103	2.465733	2.41659	2.409139	2.41971	2.40894	2.393449	2.408865	2.418595	2.317735
ULK2	1554112_a_at	2.501939	2.487368	2.459351	2.469496	2.480944	2.472192	2.472874	2.478809	2.581378	2.473227	2.47161	2.473556	3.991566
SLC4A8	1554113_a_at	2.036422	2.011719	1.993364	1.997707	2.033234	1.991811	2.003826	2.007835	1.995036	1.990642	1.990527	2.002375	1.946643
SSH2	1554114_s_at	2.348421	2.3198	2.288679	2.294881	2.335027	2.296585	2.308481	2.313373	2.30343	2.290651	2.299076	2.311699	2.246176
LOC133874	1554115_at	2.193654	2.181734	2.152618	2.163874	2.180488	2.163126	2.178345	2.207682	2.167389	2.162901	2.168862	2.173185	2.105656
PARP11	1554116_s_at	3.21147	3.1894	3.485748	3.187298	3.220038	3.630356	3.089822	3.198493	3.188537	3.141404	3.18012	3.775837	3.146972
CCDC60	1554117_at	2.873909	2.982049	2.808309	2.801402	2.866366	2.815474	2.832598	2.832246	2.816205	2.774582	2.84769	2.838739	2.574873
C16orf57	1554119_at	3.6832	3.648972	3.561695	3.630665	3.657897	3.633201	3.638906	3.655045	3.640592	3.793961	3.638657	3.697779	3.36134
HSD17B12	1554121_at	2.313448	2.285607	2.241995	2.264965	2.304793	2.257047	2.277639	2.28314	2.269693	2.26124	2.26822	2.278944	2.152977
HSD17B12	1554122_a_at	3.480149	3.630842	3.188892	3.313162	3.368884	3.231581	3.537667	4.471535	3.425214	3.326779	4.214745	3.463541	3.040301
NLGN4Y	1554125_a_at	3.49628	3.373055	3.347041	3.372038	3.512054	3.27402	3.342794	3.38279	3.379423	3.331022	3.375744	3.681409	3.183181
MSRB3	1554126_at	2.670459	2.664026	2.551542	2.646827	2.653745	2.653162	2.663745	2.791164	2.661364	3.089429	2.740001	3.920369	4.461488
MSRB3	1554127_s_at	2.338767	2.330861	2.299686	2.314856	2.329813	2.318786	2.329635	2.32386	2.31558	2.317679	2.314205	2.320652	5.13849
ADIG	1554128_at	2.506619	2.488433	2.458826	2.466817	2.489538	2.467711	3.007027	2.486306	3.026686	2.575424	2.470433	2.593636	2.375593
ADIG	1554129_a_at	2.730684	2.689254	2.657352	2.629761	2.696927	2.671184	2.682483	2.680411	2.673831	2.665497	2.658554	2.682501	2.46671
KIAA1128	1554131_at	3.89821	3.60934	3.517836	3.724163	4.024192	3.618442	3.664694	3.76195	3.705337	3.648955	3.841761	3.925575	3.14585
KIAA1128	1554132_a_at	3.916511	3.654333	4.524015	4.626073	4.174209	3.674975	3.881233	3.654011	3.494566	3.643385	3.795495	3.633973	5.122083
RUFY2	1554133_at	3.929657	3.583239	3.759242	3.692743	4.045705	3.598154	3.689143	3.615341	3.754221	3.715911	3.858477	4.001708	3.995683
WDR78	1554140_at	2.560166	2.511576	2.470098	2.507983	2.564051	2.485394	2.512757	2.528171	2.512937	2.500043	2.513067	2.528095	2.406066
WDR78	1554141_s_at	2.014177	1.997964	1.975863	1.980224	2.010456	1.979332	1.992021	1.995075	1.981484	1.971547	1.98406	1.993873	1.929064
SUGT1L1	1554142_at	3.93911	3.581584	3.505345	3.70346	4.184525	3.556191	3.670095	3.635862	3.744606	3.65748	3.962068	3.983524	3.124744
SUGT1L1	1554143_a_at	2.590051	2.5285	2.587028	2.827982	2.976811	2.638666	2.51176	2.480152	2.515551	2.502873	2.502215	2.511365	2.390666
CCDC128	1554145_a_at	2.613601	2.348848	2.42154	2.423682	2.259137	2.756789	4.190546	2.435288	4.702885	4.492098	2.416955	2.417219	3.979298
C3orf15	1554147_s_at	2.385752	2.329584	2.771466	2.328633	2.375141	2.318936	2.308689	2.341644	2.329147	2.318065	2.342496	2.342197	2.163351
SLC33A1	1554148_a_at	5.034719	4.401758	5.974932	5.297687	5.049829	4.633603	6.50634	4.355247	6.591929	6.23804	3.789381	4.646452	5.933684
CLDND1	1554149_at	7.004267	6.891446	7.24697	6.991977	6.048964	5.521016	8.146104	7.217288	8.115568	8.124911	7.093587	6.944209	8.403351
OGDH	1554151_at	2.908927	2.891455	2.816314	2.82116	2.907493	2.952324	3.156861	3.211266	2.872641	3.129984	2.872808	3.944562	2.567429
OGDH	1554152_a_at	2.538017	2.527552	2.479095	2.530986	2.51739	2.484426	2.896757	2.52207	2.589553	2.554478	2.504717	2.517941	3.882714
PHF21A	1554153_a_at	2.574055	2.359845	2.522341	2.52611	2.245046	2.509032	3.15288	2.541847	2.744136	2.724778	3.385207	3.14422	4.418114
GDAP2	1554154_at	6.05741	5.960727	6.698099	6.762761	5.852662	5.180435	5.67431	5.13381	5.680321	5.404829	5.016675	5.054022	6.203417
MCPH1	1554155_at	5.197016	4.85426	5.140839	5.267848	6.093364	6.47375	6.348537	5.92807	6.679929	5.946937	6.608242	6.921374	5.952572

WFDC8	1554156_a_at	2.307345	2.27771	2.245494	2.258954	2.315545	2.256888	2.268164	2.278677	2.264492	2.252039	2.264583	2.273369	2.160024
WFDC8	1554157_a_at	2.364339	2.332595	2.305159	2.318845	2.348035	2.309092	2.322467	2.334361	2.319276	2.316892	2.31609	2.325899	2.193677
ZMYND11	1554158_at	3.600707	3.40316	3.261	3.389193	3.471147	3.404874	3.373925	3.456219	3.434272	3.42277	3.461936	3.627769	2.927264
ZMYND11	1554159_a_at	2.515813	2.475835	2.439195	2.596271	2.515289	2.467697	2.470707	2.43278	2.470379	2.450913	2.454642	2.4792	4.896511
ZNF446	1554160_a_at	2.388135	2.384753	2.358411	2.364853	2.384551	3.118456	2.378243	2.383339	2.368782	2.361871	3.139236	2.369336	2.367062
SLC25A27	1554161_at	5.80944	5.579732	5.371157	4.731688	4.730358	4.930622	4.124901	4.32311	4.649432	3.917696	4.13012	4.122786	4.620101
TWIST2	1554163_at	2.330059	2.315731	2.291512	2.297499	2.318032	2.29651	2.308401	2.315093	2.300455	2.293142	2.300152	2.310011	2.278692
FAM53B	1554165_at	2.743164	2.699627	2.633598	2.685169	2.7467	2.66777	2.680511	2.704834	2.691313	2.658307	2.688051	2.707599	2.490813
GOLGA7	1554167_a_at	5.259693	5.325372	6.551216	5.86369	4.841256	3.866331	6.99926	4.781025	6.740223	7.088113	4.858718	5.503313	7.930984
SH3KBP1	1554168_a_at	6.23355	6.13342	6.280822	6.292803	5.898997	6.101344	6.64143	6.48554	6.659137	6.133701	7.020859	6.851939	6.652231
LOC255374	1554170_a_at	4.074244	3.572141	3.575351	3.549819	3.560346	3.558077	3.689561	3.565142	3.556522	3.555985	3.54684	3.55856	3.412279
ZMYM3	1554171_at	4.92001	4.805955	4.421167	4.83474	5.466882	5.852796	4.97071	6.068278	5.465637	5.07615	5.536576	5.82404	4.74578
ZMYM3	1554172_a_at	1.890873	1.885295	1.863263	1.869159	1.876913	1.86653	1.882163	1.895495	1.878808	1.872275	1.867636	2.358181	1.907177
CD300LB	1554173_at	2.6268	2.61637	2.589079	2.595804	2.610901	2.607354	2.606804	2.607753	2.602663	2.596798	2.599149	2.652243	2.427655
CD300LB	1554175_at	2.195787	2.172538	2.144459	2.158433	2.167335	2.14915	2.168304	2.172673	2.156504	2.147983	2.154414	2.162529	2.085563
C3orf33	1554176_a_at	3.047829	2.97997	2.930159	2.979825	3.025669	2.927618	3.243594	3.003697	3.476317	3.183943	3.005689	3.378118	3.603802
ATP5S	1554177_a_at	7.680941	7.671021	7.281479	6.925884	7.524348	7.546077	7.140694	7.357239	7.386011	7.344685	7.369008	7.575952	6.601552
FAM126B	1554178_a_at	7.959193	8.006355	7.940028	7.894753	7.445052	6.902213	6.92268	6.889133	7.15992	7.084676	6.478121	6.77711	6.583778
LYNX1	1554179_s_at	2.463041	2.453176	2.526996	2.520995	2.448977	2.444949	2.437149	2.446611	2.424894	2.424921	2.433192	2.441974	2.431022
FLJ30934	1554181_at	2.222108	2.199738	2.177289	2.183544	2.208097	2.182772	2.192299	2.20183	2.185862	2.181549	2.181438	2.196437	2.142524
TRIM73	1554182_at	3.630012	3.276827	3.123487	3.517232	3.797788	5.166111	2.866756	3.126424	2.856886	3.016147	3.081366	3.082909	2.89559
TRIM73	1554183_s_at	4.267703	4.31971	4.215235	4.218514	4.787885	4.937721	4.13326	4.216536	3.939175	3.893376	4.206447	4.212696	3.930732
LOC554206	1554185_at	3.203882	3.152073	3.132248	3.129848	3.155742	3.138352	3.129177	3.141087	3.123115	3.088653	3.131292	3.132791	3.049046
LOC554206	1554187_at	2.531281	2.522153	2.457918	2.493067	2.560177	3.128471	2.507238	2.518153	2.556774	2.4873	2.50397	2.513658	2.360494
C11orf53	1554188_at	2.493718	2.374917	2.256644	2.361245	2.388011	2.360781	2.368301	2.368598	2.358153	2.356685	2.35726	2.367285	2.236365
C10orf81	1554190_s_at	3.081036	2.977685	2.945569	3.094337	3.097839	2.964436	3.008755	3.020316	2.972597	2.991282	3.007448	3.054016	2.683621
C4orf23	1554192_s_at	2.47833	2.467976	2.448809	2.452681	2.466453	2.443478	2.454482	2.463508	2.448387	2.443569	2.445492	2.458071	2.311206
MANEA	1554193_s_at	2.300495	2.26848	2.343806	2.255481	2.283754	2.254755	2.267489	2.268319	2.257026	2.248808	2.250984	2.26206	2.219593
MGC23985	1554195_a_at	2.152777	2.144266	2.122917	2.127487	2.174134	2.116875	2.138774	2.141681	2.137677	2.124989	2.126517	2.13473	2.080155
IZUMO1	1554196_at	2.561547	2.511533	2.464119	2.498983	2.552552	2.479096	2.507042	2.517434	2.504031	2.484875	2.500851	2.518194	2.33994
SH3YL1	1554198_at	1.989738	1.980291	1.957886	1.966568	1.969996	1.964946	1.973727	1.980175	1.965088	1.959409	1.958775	1.970945	1.906374
PTPRO	1554199_at	2.234415	2.209833	2.187025	2.191396	2.216803	2.190887	2.20254	2.205538	2.193936	2.186472	2.195781	2.224038	2.136783
CABP4	1554201_at	3.550187	3.437242	3.444362	3.159825	4.101997	4.859452	3.435105	3.475174	3.482457	3.405021	3.5949	3.506972	2.888466
CABP4	1554202_x_at	3.087856	3.059148	2.982765	2.96104	3.079977	4.162485	3.074701	3.059714	3.051196	3.329375	3.045679	3.054236	2.903149
C21orf9	1554203_at	3.096875	3.072876	2.998879	3.058612	3.172839	3.064483	3.066582	3.075781	3.059506	3.015523	3.058206	3.121674	2.720961
ICA1L	1554205_s_at	3.938217	3.930244	3.832436	3.895824	3.942163	3.689958	3.904058	3.929008	3.935049	3.9165	3.935305	3.920792	3.918964
TMLHE	1554206_at	3.609294	3.596032	3.440188	3.614297	3.597033	3.590984	3.520282	4.052557	3.595224	3.46528	3.870601	3.713646	2.859381
RP5-821D1	1554208_at	1.838041	1.828363	1.809138	1.813325	1.839494	1.81092	1.823868	1.819061	1.816953	1.814407	1.808278	1.811985	1.781531

ZCCHC13	1554210_at	2.479646	2.449118	2.415018	2.428719	2.456445	2.430182	2.436409	2.446211	2.43253	2.423738	2.428706	2.441875	2.297032
KCNS2	1554212_s_at	2.682985	2.618536	2.575017	2.625262	2.741308	2.611289	2.63229	2.63647	2.627624	2.608817	2.62861	2.69197	2.501403
ARHGEF10	1554213_at	2.565639	2.529465	2.529462	2.531597	2.53783	2.496684	2.530389	2.534944	2.528833	2.507483	2.709784	2.53669	3.729881
CCDC132	1554216_at	3.214848	3.032404	2.982628	3.058238	3.365911	3.097831	3.06541	3.08436	3.102805	3.091281	3.129356	3.240005	3.014287
CCDC132	1554217_a_at	2.831377	2.711596	2.655927	2.738191	2.873309	2.875713	2.744277	2.74215	2.895028	2.718624	2.837343	2.979043	3.346581
MGC45922	1554222_at	2.304546	2.443196	2.588982	2.286305	2.292154	2.316053	2.295922	2.296997	2.286588	2.283299	2.277802	2.278982	2.206446
C8orf56	1554228_a_at	2.003434	1.989509	1.968907	1.972298	1.993446	1.980243	1.98764	2.101873	1.97985	1.968192	1.98119	1.988202	1.918835
LOC153222	1554229_at	2.682053	2.590784	2.558769	2.576411	2.597497	3.687976	2.583259	2.584077	2.574828	2.566589	2.565926	2.584115	2.486326
ZNF396	1554231_a_at	2.769692	2.745971	2.679939	2.705345	2.78044	2.816992	2.734243	2.745759	2.729958	2.7217	2.732771	2.748036	2.611149
C1QTNF9	1554233_at	2.492653	2.462773	2.408819	2.443555	2.478163	2.440494	2.447735	2.459244	2.444372	2.429269	2.442675	2.456582	2.350473
KATNAL2	1554234_at	2.859002	2.811882	3.126746	3.197137	3.294	2.80512	2.810611	2.826979	2.807839	2.788225	2.812439	2.821203	2.662253
CTNNA3	1554235_at	3.080417	2.984014	2.939143	2.973883	3.494763	2.951672	2.932257	2.963983	2.93746	2.925761	2.951389	2.988834	2.755567
SDCCAG8	1554237_at	3.897108	3.613691	4.128784	4.67571	4.150576	3.754724	3.766538	3.76403	3.808263	3.703788	3.990289	3.832898	3.636053
ZADH2	1554239_s_at	6.1267	6.177755	5.745851	5.70757	6.079635	6.035046	4.17817	4.898992	3.30314	3.458513	4.49664	3.983408	6.07506
ITGAL	1554240_a_at	5.663747	5.219648	5.7666	4.810229	5.296766	4.862903	4.82195	3.988965	4.469085	4.096709	3.44134	4.191112	2.264427
COCH	1554241_at	8.632553	8.665356	7.810261	7.871723	8.251422	7.786554	9.011475	8.008781	9.420468	9.285191	8.218319	8.190138	9.871421
COCH	1554242_a_at	2.803258	2.734276	2.681997	2.737486	2.793266	2.729303	3.200357	2.762468	2.743553	4.355862	2.746036	2.756376	5.253184
ARL17	1554245_x_at	4.415099	4.813768	4.257922	3.798527	4.254811	4.449695	4.231812	4.075215	4.268511	4.21875	4.228385	4.246479	4.035045
C1orf210	1554246_at	5.347276	4.89756	3.787233	4.065569	4.087292	4.098918	5.892385	5.41171	6.147569	6.252126	4.511482	4.849483	3.455088
ZNF638	1554248_at	1.826533	1.823194	1.812654	1.821551	2.052389	2.775113	1.818933	1.826377	1.826377	1.799256	1.82175	1.81351	1.764569
ZNF638	1554249_a_at	5.155212	4.384561	5.255615	5.252041	5.516947	5.230443	5.204868	5.089241	5.196389	5.064501	5.193036	5.233632	5.888129
TRIM73	1554250_s_at	7.117056	6.722259	6.334726	6.332806	6.41049	6.146522	5.202665	5.389959	5.208156	4.91053	5.883687	5.177439	6.123505
HP1BP3	1554251_at	6.05632	6.049105	5.935071	6.247039	6.043088	6.209925	7.694512	7.54271	6.28512	7.503645	7.799359	6.418627	6.015995
LASS3	1554252_a_at	2.639955	2.61268	2.580073	2.592625	2.639267	2.592344	2.600776	2.608361	2.596274	2.574456	2.591372	2.605849	2.503925
LASS3	1554253_a_at	2.145374	2.114911	2.090145	2.100958	2.114586	2.19366	2.107162	2.113913	2.099474	2.093709	2.093352	2.106049	2.025566
PCNXL2	1554256_a_at	2.075921	2.058569	2.038	2.04591	2.058456	2.045116	2.047826	2.05469	2.04031	2.030276	2.036331	2.049838	2.00937
DNAJC5B	1554258_a_at	2.256093	2.225009	2.209407	2.208014	2.239544	2.202972	2.216536	2.227234	2.213614	2.213536	2.213199	2.221923	2.119061
GPSM1	1554259_at	1.789837	1.779905	1.759703	1.767633	1.778944	1.857144	1.777094	1.779017	1.770948	1.764721	1.764943	1.770715	1.734792
FRYL	1554260_a_at	3.320397	2.717233	3.75814	3.462634	3.295519	3.497816	5.197819	3.982187	5.154255	5.561184	3.581875	4.032861	4.790104
KLHL29	1554261_at	2.014408	1.986875	1.968906	1.968655	1.986553	1.966661	1.983469	1.98525	1.974084	1.970073	1.967238	1.978074	1.913327
KLHL29	1554262_s_at	2.378999	2.359998	2.332473	2.335577	2.368989	2.326556	2.343815	2.358172	2.340334	2.335611	2.339259	2.350537	2.268588
CKAP2	1554264_at	3.739558	3.482596	3.594442	3.640695	3.979302	3.593227	5.069034	3.718621	5.919528	4.357355	4.923431	4.546355	3.752916
MORN1	1554268_at	2.549237	2.521244	2.473299	2.514163	2.533715	2.508495	2.525431	2.529573	2.516944	2.508633	2.513276	2.524949	2.452509
CENPL	1554271_a_at	2.395502	2.307718	2.28618	2.289244	2.31767	2.281724	5.815638	5.120491	6.158417	5.555909	4.697392	5.521738	4.564011
LRAP	1554272_at	2.939782	2.858878	2.813615	2.838897	2.880222	2.826186	2.850346	2.859095	2.847293	2.833206	2.857675	2.894594	2.595056
LRAP	1554273_a_at	2.647787	2.590221	2.573157	2.578951	2.682534	2.563293	2.578528	2.590449	2.581397	2.563775	2.576197	2.601021	2.412401
SSH1	1554274_a_at	1.987773	1.977338	1.959093	1.96344	2.043938	1.962907	1.974841	1.970464	1.958424	1.951422	1.961365	1.966909	1.904951
SART3	1554276_at	2.226526	2.205289	2.185432	2.189964	2.209674	2.188466	2.197776	2.205826	2.190993	2.18526	2.184227	2.193953	2.142488

FANCM	1554277_s_at	2.587846	2.540346	2.491294	2.528216	2.582643	2.519819	2.548291	2.55639	2.59926	2.535216	2.534475	2.554912	2.501736
CXorf34	1554279_a_at	2.590362	2.563275	2.538735	2.546989	2.573231	2.523642	2.698844	2.554562	2.549601	2.537816	2.549518	2.561581	2.474013
C9orf43	1554280_a_at	2.365723	2.341098	2.322568	2.447918	2.347938	2.318896	2.324221	2.341975	2.32632	2.114663	2.320681	2.335554	2.239961
CCRN4L	1554283_at	3.881095	3.459324	5.437434	6.334091	3.905446	4.211283	3.654245	4.556217	4.269453	3.78176	4.362843	4.406446	3.295376
HAVCR2	1554285_at	2.506349	2.48355	2.458302	2.455337	2.476919	2.458247	2.509262	2.474305	2.461301	2.457499	2.451344	2.470933	2.360706
FLJ25758	1554286_at	1.93421	1.926165	1.90923	1.910849	1.926003	2.085371	1.914138	1.922195	1.909233	1.899622	1.903181	1.916571	1.871557
TRIM4	1554287_at	3.732388	3.059134	3.659586	3.745956	3.634942	3.636944	3.042941	3.042766	3.043274	3.008274	3.024764	3.125994	3.053752
KIAA1600	1554288_at	2.848652	2.745992	2.700062	2.736594	2.76865	2.716712	2.738181	2.747745	2.735133	2.71972	2.731474	2.742771	2.599215
HERC3	1554290_at	3.064263	3.020441	2.964883	2.998741	3.052674	2.994587	3.007308	3.019919	3.008744	2.954367	3.00736	3.020389	2.723761
KIAA0701	1554291_at	3.094477	3.060488	3.041314	3.043191	3.546351	3.047599	3.049915	2.983144	3.002056	2.973947	3.007676	3.041961	2.623526
KIAA0701	1554292_a_at	3.076148	3.008172	2.94334	3.004373	3.302318	2.811668	3.002523	2.846701	2.992282	3.000842	2.988747	3.028816	2.827573
TTBK2	1554293_at	2.861111	2.778534	2.714975	2.767956	2.848517	2.735983	2.784476	2.793223	2.781113	2.755708	2.784541	2.819391	2.675644
TTBK2	1554294_s_at	2.946169	2.882397	2.898268	2.894479	2.963894	2.869309	2.91422	2.885907	2.899238	2.902193	2.899989	2.908781	2.879995
TTBK2	1554295_x_at	2.840273	2.7655	2.711904	2.895445	2.845025	3.796539	2.781006	2.789395	2.778534	2.751844	2.759926	2.815597	2.549814
CYP19A1	1554296_at	3.450988	3.336592	3.246184	3.370342	3.504347	3.304189	4.009278	3.51275	4.666767	3.763694	3.573765	3.580612	2.977111
WDR49	1554298_a_at	3.047848	2.973808	2.867447	2.941736	3.057379	2.887058	2.954716	2.98603	2.961327	2.924463	2.978012	3.083845	2.849721
NPAS4	1554299_at	2.117776	2.100759	2.068125	2.083973	2.104093	2.081821	2.094341	2.09981	2.085658	2.077737	2.083054	2.093422	2.012592
SVOPL	1554300_a_at	2.114543	2.08809	2.064638	2.069854	2.088827	2.068154	2.080912	2.087165	2.073743	2.065144	2.073841	2.080978	2.002049
LOC645591	1554301_at	4.064742	3.762085	3.687575	3.858399	4.136997	3.618479	3.797268	3.807667	3.871066	3.805353	3.989456	4.079532	3.172681
LOC645591	1554302_s_at	1.96127	1.948945	1.930058	1.931997	1.952048	1.936857	1.943269	1.945113	1.9426	1.926915	1.933308	1.940037	1.875324
HNMT	1554303_at	1.873129	1.849883	1.830665	1.834696	1.848519	1.863771	1.847661	1.846306	1.83579	1.828325	1.830715	1.839804	1.788885
MGC20647	1554305_at	2.47914	2.524725	2.430952	2.446128	2.481637	2.724776	2.401201	2.457723	2.441817	2.435218	2.443428	2.449611	2.339583
ITPKB	1554306_at	3.175675	2.988705	2.915345	3.061469	3.111572	2.931899	3.015001	3.022331	3.067391	3.06478	3.126245	3.19301	2.809093
LOC644852	1554307_at	2.24912	2.221345	2.18796	2.201349	2.228805	2.203524	2.215742	2.220896	2.205466	2.19656	2.202637	2.215596	2.113404
GABRA2	1554308_s_at	2.93118	2.785779	2.725633	2.781034	2.956904	2.753949	2.79401	2.806603	2.787825	2.763294	2.791149	2.823353	2.630204
EIF4G3	1554309_at	3.108557	3.035716	2.920743	2.992315	3.101709	2.923613	3.065087	2.996969	2.969505	3.043802	2.986015	3.169409	2.681729
EIF4G3	1554310_a_at	2.485159	2.444113	2.405121	2.424026	2.457127	2.415491	2.430802	2.43308	2.422959	2.423138	2.420609	2.438152	2.401451
SUPT6H	1554311_a_at	2.678637	2.622337	2.569321	2.612971	2.677001	2.64339	2.625027	2.63413	2.622834	2.606531	2.625294	2.649864	2.496821
RNF170	1554312_at	2.176097	2.167642	2.136439	2.151271	2.168999	2.145416	2.165238	2.160702	2.155172	2.1446	2.148891	2.158104	2.138362
C6orf141	1554314_at	2.996618	2.91435	2.858812	2.939386	3.014549	2.923816	2.914048	3.571091	2.912656	2.883031	2.94627	2.960173	2.656872
PGLS	1554316_at	2.99526	2.988367	2.968391	2.970466	2.987328	3.043144	2.983094	2.984722	2.97359	2.965441	4.06964	2.999279	2.926305
C14orf172	1554317_s_at	1.913275	1.905462	1.886112	1.890324	1.898246	1.887992	1.901423	1.90325	1.896533	1.887873	1.892114	1.893521	1.884871
LOC541473	1554318_at	1.756907	1.746621	1.729131	1.73359	1.746082	1.732683	1.74212	1.744558	1.735101	1.730148	1.725936	1.738205	1.695061
RPS6KA5	1554319_at	3.000452	2.930666	2.886754	2.914754	3.103057	2.843188	2.920126	2.906862	2.919746	2.905649	2.946493	2.985638	2.722856
NFS1	1554321_a_at	2.498226	2.483715	2.684152	2.46374	2.48017	2.468159	2.709112	2.487275	2.508057	2.741173	2.451525	2.474205	2.941932
HDAC4	1554322_a_at	2.087669	2.073702	2.055743	2.056415	2.073484	2.056338	2.061238	2.071167	2.059401	2.051765	2.052716	2.065608	2.017163
DYNC2LI1	1554324_s_at	2.755028	2.602596	2.580767	2.609804	2.820822	2.586091	2.598608	2.632207	2.65049	2.594564	2.657056	2.686539	3.15935
DOCK2	1554325_at	2.468185	2.442895	2.411436	2.42779	2.491953	2.424833	2.42459	2.436655	2.421414	2.407979	2.420903	2.437358	2.286869

CANT1	1554327_a_at	1.975202	1.959851	1.938028	1.941076	1.958198	1.941603	1.958378	1.957861	1.953657	1.93711	1.938975	1.951253	2.709017
STXBP4	1554328_at	2.93769	2.852025	2.787979	2.852358	2.929439	2.828876	2.860424	2.865393	2.861366	2.839644	2.873005	2.898638	2.619244
STXBP4	1554329_x_at	3.021471	2.932653	2.877664	2.923552	3.068121	2.921459	2.940223	2.9487	2.944149	2.906385	2.939789	2.978305	2.698911
LRRC18	1554331_a_at	2.611035	2.572578	2.537972	2.57444	2.581254	2.578531	2.587711	2.605802	2.582871	2.576568	2.571532	2.585051	2.433333
DNAJA4	1554333_at	3.59425	3.349858	3.271228	3.420685	3.618059	3.335933	7.679512	6.828712	7.820907	7.695304	6.081056	6.266223	5.800755
DNAJA4	1554334_a_at	3.53022	3.584814	3.351966	3.439612	3.516623	3.120576	8.998824	8.791639	8.977092	9.000666	7.628594	8.564714	6.547122
PSCD4	1554335_at	2.430804	2.345587	2.283119	2.336344	2.365343	2.346758	2.355463	2.362111	2.349975	2.344086	2.348077	2.360366	2.118063
FARP2	1554337_at	2.823852	2.758968	2.692217	2.743672	2.833859	2.752457	2.759712	2.767931	2.756037	2.745931	2.764503	2.788414	2.534571
COG3	1554339_a_at	3.093662	3.042805	3.426126	3.069356	3.315634	3.025845	3.046177	3.052165	3.04013	3.024091	3.201294	3.27348	3.793013
C1orf187	1554340_a_at	2.550197	2.509304	2.461007	2.495914	2.537943	2.481231	2.512416	2.514483	2.503734	2.496535	2.500423	2.517517	3.034386
HEL308	1554341_a_at	4.068321	3.646449	4.406763	3.82326	3.964752	3.597217	3.795829	3.906368	4.260372	4.226034	3.95715	3.786839	5.602331
HEL308	1554342_s_at	5.087173	4.37981	4.975104	5.065342	5.079574	4.683752	5.562123	5.656966	5.391119	4.895275	5.308525	5.578899	5.905502
STAP1	1554343_a_at	2.855558	2.687507	2.668657	2.686492	2.724046	2.655234	2.674892	2.705516	2.672873	2.655893	2.686134	2.699451	2.477022
AQP12A	1554344_s_at	1.85059	1.845483	1.822918	2.040153	1.849268	1.827051	1.836705	1.843275	1.834748	1.822719	1.830687	1.840023	1.756299
GIN1	1554345_a_at	3.775298	3.736927	4.631067	4.357784	4.032577	3.681815	4.238498	3.878208	4.159524	4.316502	3.663482	3.841467	4.640942
GNB5	1554346_at	2.564903	2.528661	2.49807	2.378549	2.571075	2.788285	2.532561	2.514046	2.523081	2.503189	2.514571	2.59298	2.524175
CDKN2AIPN	1554348_s_at	2.54262	2.501177	2.519155	3.364552	2.493235	2.478677	2.482401	2.500574	2.760463	2.490767	2.470522	2.494453	2.496607
XRCC6BP1	1554349_at	2.091712	2.056029	2.039943	2.044291	2.058321	2.040466	2.056653	2.056024	2.046209	2.042005	2.040659	2.050567	1.973642
TIPRL	1554351_a_at	6.351462	6.278266	6.992455	6.462321	6.144355	5.459224	7.634121	6.213668	7.167547	7.067674	5.976018	5.742959	7.955381
DENND4A	1554352_s_at	5.594598	4.99361	6.554389	6.281605	5.417173	5.26048	8.665691	7.609054	8.905989	9.144923	6.719876	7.461707	5.211859
SIAE	1554354_at	3.341543	3.178664	3.07427	3.187589	3.342946	3.128986	3.175828	3.177787	3.21323	3.154598	3.271721	3.332666	2.8754
SIAE	1554355_a_at	2.518957	2.43128	2.379501	2.403185	2.458863	2.406954	2.411839	2.41611	2.408255	2.395181	2.412685	2.418757	2.331795
GINS4	1554356_at	2.312257	2.53198	2.2769	2.322141	2.30066	2.266994	2.576597	3.169843	2.699383	2.706949	2.400698	2.517678	2.239187
FCHSD2	1554360_at	2.792693	2.635478	2.594295	2.615422	2.663986	2.607898	2.63044	2.630171	2.617357	2.599379	2.629142	2.629722	2.514283
BTG4	1554362_at	2.056345	2.040557	2.018781	2.02425	2.05402	2.02245	2.036206	2.038342	2.025904	2.021078	2.022459	2.035992	1.972023
PPP2R5C	1554364_at	1.919532	1.90676	1.889847	1.892474	1.906507	1.894472	1.905626	1.906069	1.901056	1.884873	1.890172	1.898326	1.840216
PPP2R5C	1554365_a_at	5.713207	5.619769	7.396438	7.066645	5.747674	4.019647	7.592318	6.368785	7.827953	7.795228	6.108958	6.195751	6.942568
FAM26C	1554367_at	2.547306	2.48654	2.439295	2.47159	2.484773	2.455146	2.480792	2.491227	2.477584	2.42663	2.473239	2.485372	2.343655
NT5C1B	1554368_at	2.532831	2.508126	2.477255	2.482779	2.567293	2.487092	2.497282	2.506188	2.493576	2.483744	2.494796	2.502952	2.341682
PKD1L2	1554371_at	2.360555	2.348385	2.32656	2.329375	2.351119	2.451765	2.341472	2.334045	2.332925	2.316323	2.32528	2.335715	2.293294
NR1H4	1554375_a_at	8.604712	8.563704	9.542893	9.021329	8.201707	6.738455	9.052653	6.641057	8.770151	8.670831	6.555694	7.040331	3.556146
PTPLA	1554376_s_at	2.122944	2.083219	2.077458	2.095831	2.100786	2.083688	2.094196	2.146344	2.088062	2.078285	2.08838	2.096898	2.01123
CNTNAP4	1554377_a_at	2.811667	2.765043	2.772449	2.779776	2.795624	2.77611	2.788248	2.788838	2.779594	2.752976	2.777269	2.828241	3.552223
PDE1C	1554378_a_at	2.64304	2.603753	2.539348	2.588132	2.632038	2.586756	2.601678	2.601107	2.58872	2.568167	2.586073	2.610869	2.416715
TP73	1554379_a_at	1.904644	1.888731	1.870326	1.871979	1.887665	1.873218	1.884861	1.887185	1.883056	1.873215	1.866958	1.875066	1.823959
NEK11	1554380_at	2.375061	2.360763	2.327983	2.395715	2.343011	2.314921	2.332434	2.344747	2.329553	2.331424	2.330224	2.336597	2.21483
LOC200261	1554382_at	2.157707	2.132967	2.098628	2.113241	2.131517	2.108434	2.121559	2.129265	2.124824	2.107074	2.113662	2.126576	2.061436
TRAM2	1554383_a_at	2.616604	2.590267	2.566563	2.569104	2.588995	2.570524	2.574226	2.588328	2.571333	2.566491	2.569365	2.582877	2.510849

PADI2	1554384_at	2.437963	2.398525	2.358672	2.382014	2.420338	2.381859	2.392642	2.404119	2.391294	2.377624	2.389294	2.442948	2.261984
PADI2	1554385_a_at	2.043946	2.03048	2.002992	2.0182	2.031411	2.023088	2.022438	2.029304	2.019502	2.008803	2.01424	2.051028	1.974345
CST9	1554386_at	2.575317	2.546962	2.500446	2.523615	2.552479	2.52352	2.531964	2.543655	2.530592	2.522976	2.52934	2.539979	2.460541
VPS29	1554388_at	4.141071	4.1345	4.133849	3.909437	3.723261	4.136817	4.515622	4.263794	4.090893	4.354207	4.191705	4.135635	4.5207
ACTR2	1554390_s_at	3.676559	3.511546	4.33492	4.513521	4.285065	4.23311	4.92532	3.966564	4.821839	4.683823	4.452731	3.850625	5.160861
TBK1	1554391_at	2.325736	2.31338	2.287811	2.28585	2.31223	2.286855	2.307251	2.298399	2.30998	2.292178	2.289097	2.350867	2.231457
ADC	1554393_a_at	2.449824	2.423261	2.398495	2.408375	2.467706	2.403619	2.424053	2.426721	2.415082	2.410939	2.411276	2.421014	2.370316
NELL1	1554394_at	2.829979	2.754094	2.691415	2.753071	2.846045	2.738626	2.759478	2.770779	2.748823	2.728283	2.756584	2.795833	2.544981
UEVLD	1554396_at	2.624597	2.586444	2.373214	2.571054	2.579616	2.568625	2.556006	2.568428	2.57916	2.574086	2.56982	2.803034	2.210066
UEVLD	1554397_s_at	1.963185	1.95346	1.936574	1.940176	1.975443	1.939054	1.953738	1.954029	2.045762	2.034173	1.933495	1.946076	3.954575
LYG2	1554398_at	3.342972	3.259705	3.120491	3.23144	3.251386	3.2325	3.185243	3.2485	3.228371	3.062619	3.245577	3.243201	2.928115
TCTE3	1554400_at	3.691948	3.443009	3.945758	3.509342	3.803773	3.549355	3.487456	3.542114	3.542569	3.507297	3.846414	3.613226	3.044087
TCTE3	1554401_a_at	2.429915	2.401011	2.375966	2.380944	2.416831	2.371927	2.388847	2.398086	2.386853	2.372389	2.385763	2.395452	2.275243
C21orf100	1554405_a_at	2.810422	2.719214	2.717011	2.722268	2.870982	2.717448	2.67321	2.76682	2.73083	2.716685	2.745633	2.820972	2.416635
CLEC7A	1554406_a_at	3.57867	3.342427	3.551943	3.560452	4.232229	3.630327	3.274392	3.541175	2.695102	2.987171	2.942667	3.540482	2.784437
TK1	1554408_a_at	3.305047	3.592427	2.977185	3.034595	3.02923	2.985943	7.711644	5.123789	8.009277	8.133619	6.037831	7.035383	5.402756
WBSCR16	1554409_at	3.040329	2.942437	2.84937	2.924277	3.028385	2.913765	2.936717	2.936077	2.934044	2.899055	2.951879	2.990338	2.757282
WBSCR16	1554410_a_at	3.236875	3.26812	3.221088	2.957799	2.833768	3.23346	4.5441	3.148757	4.895875	4.524796	3.250991	2.603794	4.73389
CTNNB1	1554411_at	3.451539	3.48117	4.564361	3.475078	3.473026	3.456547	3.525478	3.27584	4.351341	3.855607	3.416535	3.427755	5.624272
RUNDC2B	1554413_s_at	3.947776	4.509716	4.119104	4.869039	4.113077	3.900164	3.525285	3.700239	3.674747	3.713954	3.927928	3.757753	3.103249
OSGIN2	1554414_a_at	4.537734	4.01995	4.052237	3.812934	2.739656	3.813249	3.513211	3.100572	3.312007	3.077609	3.084188	3.551216	4.6081
TAF5L	1554415_at	5.250759	5.471397	5.924561	6.212897	5.973871	5.407053	6.579405	6.897283	7.263098	7.285424	7.201583	6.961727	4.566934
APH1A	1554417_s_at	1.989647	2.093852	2.240194	1.961882	1.966692	1.960233	2.481425	1.964334	1.97723	1.988316	1.956621	1.957375	4.314927
SPOCK3	1554418_s_at	2.628141	2.575601	2.530025	2.567264	2.615211	2.542766	2.577674	2.585803	2.572334	2.556655	2.570461	2.591113	2.558782
GGNBP2	1554419_x_at	3.336123	3.22393	3.098438	3.218618	3.385564	3.252254	3.251754	3.251023	3.254485	3.190162	3.25026	3.289898	2.98905
ATF3	1554420_at	2.24665	2.218525	2.193221	2.199347	2.221608	2.2029	2.207849	2.204149	2.20197	2.18961	2.196209	2.19712	2.124225
FBXO7	1554423_a_at	9.236817	9.231162	9.80525	9.283797	8.084439	6.505698	12.07636	9.602582	11.89401	11.86493	8.81848	9.495266	9.528602
FIP1L1	1554424_at	3.263601	3.14078	3.064211	3.156855	3.326652	3.113141	3.65824	3.159014	3.833757	4.006324	3.172553	3.251885	3.179583
NLGN2	1554428_s_at	1.146125	1.141243	1.120666	1.125493	1.130173	1.121713	1.142844	1.124821	1.134778	1.125349	1.113951	1.113958	1.141756
DMWD	1554429_a_at	4.230289	4.536235	4.50577	4.101528	4.23722	4.410893	4.535746	5.277935	4.961073	4.607989	4.823811	5.203715	5.521725
C21orf51	1554430_at	2.755116	2.732923	2.728337	2.730152	2.74472	2.741265	2.743341	2.744054	2.732447	2.729537	2.719944	2.723018	2.555827
C21orf51	1554432_x_at	4.089299	4.118218	4.096945	4.303977	4.563318	4.307812	4.111244	4.116884	4.102822	4.097561	4.100022	4.111402	3.703138
ZNF146	1554433_a_at	3.069025	3.060365	4.011777	3.051709	3.053793	2.920405	4.332687	2.902986	4.29002	4.158579	2.917864	2.930741	7.073613
REG4	1554436_a_at	3.281767	3.208836	3.174764	3.181135	3.269908	3.172018	3.185949	3.20362	3.183112	3.166741	3.190197	3.276446	3.128531
KIAA1217	1554438_at	4.527907	4.287933	4.629503	4.391745	4.645575	4.394335	3.446454	4.045678	4.152206	2.974097	3.783099	3.298187	2.488485
KIAA0513	1554440_at	2.606295	2.56711	2.504871	2.54385	2.593206	2.52314	2.555962	2.565695	2.552707	2.541327	2.553935	2.562576	2.437468
WAPAL	1554441_a_at	3.607625	3.603659	4.486765	4.031781	3.128644	3.021866	6.159653	4.03968	6.39531	6.103598	3.783171	4.368158	5.787729
BEST1	1554442_at	1.856578	1.84953	1.834175	1.835977	1.83998	1.833933	1.838581	1.833451	1.828396	1.830635	1.825208	1.827301	1.782854

BEST1	1554443_s_at	2.050767	2.042735	2.025842	2.022459	2.043882	2.032221	2.031066	2.03334	2.023245	2.017178	2.015513	2.035352	1.977099
C2orf18	1554444_s_at	4.505006	4.497268	4.327946	4.484534	4.491879	4.481105	4.497051	4.486857	4.492696	4.486753	4.478749	4.485339	4.362114
ZNF85	1554445_at	2.5208	2.496066	2.450359	2.477219	2.509517	2.458557	2.493796	2.497805	3.638427	2.480194	2.480455	2.696308	2.932273
LOC554203	1554447_at	5.673739	5.089898	5.804809	6.190099	6.955681	7.38279	5.07212	5.201329	4.387365	4.19142	6.466432	5.183911	4.772167
LOC554203	1554448_at	3.863377	3.749687	3.633369	3.993011	5.020276	4.79044	3.706324	3.734817	3.671869	3.69613	4.286442	3.191316	3.455132
MIER3	1554449_at	3.391129	3.236053	3.334172	3.410071	3.441883	3.180977	3.316649	3.241469	4.108774	4.37005	3.3557	3.408533	3.455253
MIER3	1554450_s_at	3.689485	3.613365	4.91946	4.053938	3.851541	3.259797	6.61344	3.637032	6.625116	5.732735	3.832579	3.708304	7.922802
DNAJC14	1554451_s_at	2.553128	2.510111	2.652209	2.500832	2.535885	2.524833	3.798594	2.510748	3.151163	3.743531	2.496402	2.53259	5.246836
HIG2	1554452_a_at	3.689303	3.573364	4.365754	4.264253	4.10698	4.372151	4.741278	4.392389	4.396101	4.372025	5.095659	4.749405	6.275808
HNRPLL	1554453_at	2.135884	2.118176	2.098159	2.102132	2.119604	2.101654	2.116052	2.116632	2.117032	2.104875	2.099055	2.111715	2.034288
LINS1	1554455_at	6.501159	6.544554	6.467065	6.614869	6.74836	7.015828	7.485473	7.860411	7.541254	7.344992	7.84292	7.602552	6.657593
LINS1	1554456_a_at	3.965255	3.915159	4.748497	3.906357	3.895628	3.909799	5.596014	3.969882	6.292681	5.963	3.955943	3.953916	5.811934
CFHR3	1554459_s_at	5.825703	6.532746	11.03277	11.24329	12.13192	12.02794	3.676502	3.81465	3.868721	3.925525	3.6222	3.960809	3.295602
ST8SIA4	1554460_at	3.397247	3.271108	3.191593	3.260864	3.517379	3.175903	3.249226	3.286898	3.28869	3.220864	3.380404	3.461297	3.009506
DNAJB9	1554462_a_at	6.51491	6.324335	8.493891	7.674266	6.211131	4.966503	7.296299	5.562783	7.629606	7.522339	4.828383	5.830722	6.614479
CRTAP	1554464_a_at	2.962907	2.815869	5.223072	4.430792	2.675659	2.468481	5.163573	2.641826	5.362144	5.358165	2.62385	2.65193	7.871958
ZNF673	1554465_s_at	3.317171	4.593159	3.730226	3.44529	3.317704	3.30106	4.321875	3.58555	4.172695	3.94785	3.253773	3.596647	4.41717
C16orf13	1554466_a_at	2.975078	2.957804	4.137949	3.800903	2.954378	2.953044	3.839909	2.845477	3.057218	3.171901	2.946712	2.965851	4.256546
FBF1	1554468_s_at	2.242354	2.239616	2.282214	2.184417	2.22755	2.175218	2.998019	2.222243	2.927829	2.223046	2.209915	2.234596	2.223928
ZBTB44	1554469_at	3.332261	3.565057	3.304717	3.388334	3.503057	3.393196	3.190672	3.277987	3.224681	3.218128	3.215064	3.305981	2.822749
ZBTB44	1554470_s_at	3.101075	3.151291	4.197003	3.689663	2.946948	2.818541	3.698637	3.160582	4.112531	4.367906	2.937181	3.070312	5.044982
ANKRD13C	1554471_a_at	3.044038	2.817319	4.087581	3.353942	2.95793	2.895055	3.733545	3.369539	4.030652	3.895825	2.953254	3.288237	5.245749
PHF20L1	1554472_a_at	3.947282	3.855057	4.770216	4.505208	3.902228	3.827927	3.916329	3.756035	4.464368	4.200194	3.966641	4.007115	6.531458
SRGAP1	1554473_at	3.250386	3.158014	3.038298	3.182832	3.248341	3.199452	3.103214	3.107973	3.163854	3.085996	3.686723	3.193891	4.863098
MOXD1	1554474_a_at	2.152995	2.141151	2.118168	2.12221	2.144327	2.12548	2.130312	2.133954	2.125258	2.117537	2.135276	2.127268	6.857824
C19orf47	1554475_a_at	1.946993	1.938736	1.916089	1.925453	1.929935	1.922167	1.93761	1.930172	1.932002	1.923336	1.922139	1.931987	1.925739
ZNF808	1554476_x_at	3.934803	3.632653	3.554822	3.649335	3.887345	3.724171	3.659084	3.659531	3.628388	3.629512	3.671326	3.710889	3.663262
HEATR3	1554478_a_at	4.197932	4.233378	4.392905	4.220441	4.261795	4.054002	4.943163	4.368459	5.411612	4.886363	4.065716	4.566673	3.765865
CARD8	1554479_a_at	5.933563	5.761783	6.022196	5.822326	5.92087	5.653395	7.15205	5.158823	6.350594	6.527012	5.716826	4.997927	7.029513
ARMC10	1554480_a_at	7.871551	7.69659	7.194437	7.483402	7.569618	7.853326	6.400067	7.335432	6.63247	6.637047	7.347398	7.326964	5.886973
EPB41	1554481_a_at	2.57995	2.564049	2.518135	2.533742	2.570134	2.554183	4.758281	5.092623	5.035685	4.937601	5.057708	6.038205	2.488961
SAR1B	1554482_a_at	6.545777	6.529642	8.278794	7.880068	6.198966	5.048175	6.989125	5.335512	7.281297	7.134076	4.98819	5.375679	6.455311
TMEM37	1554483_at	2.11648	2.108057	3.309748	3.027586	2.098683	2.090758	2.102696	1.918067	2.101546	2.089967	1.884508	1.95258	1.515361
TMEM37	1554485_s_at	8.594923	8.350637	8.643876	8.487125	8.305999	7.84655	7.228125	6.816042	7.411021	7.281484	6.68007	6.780192	3.911373
C6orf114	1554486_a_at	2.792099	2.776417	2.753996	2.758415	3.223123	2.758163	2.318939	2.623171	2.757535	2.379718	2.626141	2.827848	2.26514
CREBL1	1554487_a_at	2.200325	2.180986	2.153273	2.160175	2.182462	2.28454	2.172248	2.179573	2.164038	2.156807	2.165683	2.174149	2.126999
CEP70	1554488_at	2.761876	2.730878	2.705044	2.71132	2.745719	2.71132	2.733062	2.7147	2.714261	2.700982	2.712841	2.724572	2.720074
CEP70	1554489_a_at	3.537352	3.520901	4.371264	5.044246	3.97582	3.532598	3.734222	3.286582	4.012236	3.905686	3.714879	3.577922	5.490498

SERPINC1	1554491_a_at	11.36851	11.47429	12.87538	12.28218	10.57746	9.099716	11.28334	8.638638	11.35908	11.21913	7.675981	8.321604	3.95515
THADA	1554492_at	3.321132	3.192351	3.127489	3.259656	3.499696	3.232268	3.267646	3.277096	3.264657	3.229975	3.311507	3.340118	2.831631
THADA	1554493_s_at	2.517906	2.463611	2.412713	2.479059	2.484237	2.430522	2.472546	2.474705	2.506358	2.63265	2.45849	2.482393	2.798104
MTHFSD	1554494_at	3.424928	3.225691	3.124654	3.237041	3.404641	3.180274	3.231897	3.230902	3.269032	3.212198	3.311447	3.373984	2.84088
MTHFSD	1554495_s_at	2.520363	2.475485	2.43218	2.463016	2.505297	2.46483	2.473698	2.484237	2.470855	2.453392	2.472871	2.484413	2.393621
RAD51L1	1554496_at	3.359045	3.212217	3.181795	3.252457	3.315155	3.202005	3.246573	3.231211	3.255102	3.278576	3.258682	3.27546	2.880663
PPA2	1554499_s_at	1.865683	1.851917	1.83395	1.837119	1.852443	1.835853	1.848181	1.850575	1.843057	1.837778	1.833352	1.843509	1.805802
RGS7	1554500_a_at	3.032352	2.993485	2.890502	2.965955	3.050118	2.926896	2.986346	3.013938	2.996042	2.994933	3.000817	3.049655	2.949185
TSC22D4	1554501_at	5.790681	5.604618	4.578188	4.502251	4.886018	5.950837	6.814049	7.850478	7.085386	6.731096	7.511405	7.682499	5.177476
OSCAR	1554503_a_at	2.199628	2.192286	2.173214	2.171635	2.182638	2.17916	2.179361	2.180357	2.174192	2.168219	2.171737	2.172992	2.088525
NAALAD2	1554504_at	3.149339	3.088649	2.981795	3.042395	3.18131	3.02542	3.081283	3.057563	3.064274	3.064499	3.092012	3.131443	3.189617
NAALAD2	1554506_x_at	2.769153	2.734033	2.649166	2.702057	2.764828	2.704404	2.72036	2.72408	2.720684	2.708014	2.718413	2.753483	3.083168
NAALAD2	1554507_at	3.340076	3.257201	3.203354	3.236141	3.406837	3.10916	3.252468	3.280265	3.411838	3.162579	3.307362	3.351286	3.435352
PIK3AP1	1554508_at	2.406763	2.377125	3.468477	2.859581	2.365681	2.312642	2.347548	2.341502	2.330958	2.329618	2.302405	2.34022	2.16358
C10orf97	1554509_a_at	2.26804	2.257009	3.156228	2.303054	2.264216	2.226943	2.843818	2.262065	3.572999	3.234859	2.240722	2.258852	4.789144
GHITM	1554510_s_at	7.662585	7.667721	8.763082	8.193023	6.834861	6.274704	9.187815	7.415293	9.214519	9.17233	6.568087	7.435368	8.544139
WWC1	1554511_at	2.273309	2.256253	2.220433	2.239292	2.259582	2.239653	2.250878	2.25507	2.241444	2.230026	2.237113	2.249999	2.141267
CCDC123	1554512_a_at	3.85874	3.567995	3.513326	3.821603	4.162009	4.134905	3.582518	3.520608	3.709172	3.591031	4.117652	3.927996	4.3827
CCDC123	1554513_s_at	2.961584	2.894839	2.85834	2.878422	2.938551	2.854639	2.900497	2.906448	3.235139	2.887291	2.890155	2.920824	4.699248
FLJ20581	1554514_at	3.453394	3.675304	4.996035	4.654688	3.123222	3.098552	2.378099	2.382659	2.374621	2.366523	2.366736	2.383848	2.264429
GSTCD	1554518_at	3.066331	3.046115	2.980957	3.036974	3.124947	3.022263	3.119541	3.069353	3.053943	3.07632	3.054639	3.093243	3.587305
CD80	1554519_at	2.086929	2.060263	2.045276	2.050938	2.066886	2.043542	2.055901	2.042015	2.040745	2.008177	2.048164	2.055223	1.962905
LOC283861	1554520_at	3.163414	3.429956	3.07404	3.110679	3.106353	3.077536	3.089905	3.086577	3.088065	3.130002	3.076208	3.084967	3.056333
CNNM2	1554522_at	5.512516	5.376415	4.632162	4.971202	5.121678	4.398757	5.024866	5.562717	5.180149	5.582594	5.070173	5.752348	3.251388
CNNM2	1554523_a_at	3.128498	3.204694	3.252059	3.1824	3.249139	3.181071	3.459414	3.087311	3.197465	3.192076	3.183507	3.193856	2.725652
OLFM3	1554524_a_at	2.331539	2.28739	2.249071	2.263608	2.332725	2.267948	2.277909	2.284551	2.272946	2.265837	2.272349	2.286065	5.241504
OLFM3	1554526_at	3.29221	3.125633	3.117399	3.144533	3.303806	3.135185	3.143767	3.166733	3.1674	3.118107	3.20327	3.248608	4.829038
C3orf15	1554528_at	2.642812	2.635686	2.617798	3.057929	2.651614	2.637538	2.585589	2.578501	2.58384	2.585513	2.761572	2.625054	2.571921
VSTM2A	1554530_at	2.151445	2.126592	2.103869	2.113831	2.13261	2.109694	2.120787	2.124973	2.114754	2.107537	2.11572	2.124546	2.050598
TTC12	1554531_at	1.826096	1.818957	1.796407	1.796897	1.827246	1.80122	1.8123	1.813443	1.802015	1.795956	1.798984	1.806949	2.060248
C2	1554533_at	7.711889	7.339119	8.3761	8.074973	7.516316	6.833701	5.652634	4.285836	6.011699	5.617	3.714601	4.551033	2.392325
DPYD	1554534_at	2.090251	2.084502	2.065134	2.068718	2.078203	2.067202	2.078537	2.07044	2.075565	2.065579	2.060758	2.072631	1.996556
DPYD	1554536_at	2.878113	2.812908	2.800117	3.346415	2.933473	2.746934	2.774858	2.802321	2.813293	2.771738	2.79323	2.840412	2.515978
TMEM126B	1554537_at	1.947855	1.929474	1.912561	1.917443	1.924715	1.918424	1.928864	1.928466	1.914575	1.916229	1.912584	1.920311	1.862861
RHOF	1554538_at	2.189383	2.17995	2.15234	2.226159	2.177851	2.155519	2.170418	2.179214	2.287267	2.167457	2.157555	2.191984	2.078672
RHOF	1554539_a_at	1.934862	1.92574	1.90365	1.907286	1.925092	1.909184	1.925429	1.937501	1.912583	1.909563	1.910621	1.921352	1.873954
C1orf67	1554540_at	3.192878	3.076584	2.995761	3.088137	3.184235	3.061394	3.079933	3.086235	3.101804	3.052625	3.152547	3.169368	2.744381
GPRIN2	1554541_a_at	1.932389	1.918122	1.900126	1.903798	1.915489	1.90215	1.913214	1.914916	1.908977	1.896664	1.900658	1.908786	1.878407

LOC153328	1554542_at	2.532426	3.268047	2.252301	2.327629	2.535493	2.495564	2.506323	2.539146	2.496757	2.631909	2.498982	2.565713	2.479144
SPAG9	1554543_at	4.735954	4.919307	5.617342	5.131987	5.864782	4.941813	4.414461	4.491889	3.867097	4.657627	4.73965	5.000091	4.319377
MBP	1554544_a_at	4.639874	4.570791	5.382438	5.224917	5.560192	5.333911	4.615675	4.501953	5.106836	5.847627	4.773741	5.167844	4.24331
APOBEC4	1554545_at	2.222373	2.20571	2.176174	2.187894	2.217043	2.177247	2.201188	2.208124	2.192858	2.181539	2.203163	2.202733	2.130252
FAM13C1	1554547_at	4.831289	5.028941	5.020569	4.665413	4.1419	4.371095	3.729553	3.988207	4.559224	4.083247	5.87431	4.069226	8.072771
WDR20	1554549_a_at	3.204701	3.184227	3.133823	3.164822	3.345297	3.15372	3.090769	3.352785	3.16765	3.160406	3.10956	3.198856	3.089771
KIAA1430	1554550_at	3.122739	3.033538	2.9991	3.025643	3.201325	3.024142	3.028714	3.071002	3.049681	3.035714	3.084801	3.105918	2.820075
YIF1B	1554553_s_at	4.290271	4.297827	4.407741	4.293965	3.964862	3.398564	6.61651	5.841766	6.428179	6.577046	4.578607	6.012339	5.503311
CCDC57	1554554_at	2.191167	2.159228	2.134089	2.141254	2.174676	2.141183	2.166066	2.162963	2.162439	2.149015	2.144974	2.189953	2.066679
SETD6	1554555_a_at	4.539685	4.491306	4.58036	5.016305	3.883375	4.47293	5.095726	4.39622	5.340241	4.729156	4.460345	4.70779	5.03165
ATP11B	1554556_a_at	3.809765	4.441704	3.454371	3.296627	3.827814	3.679955	4.108551	4.431988	4.068058	4.317574	3.827297	4.117087	3.169155
ATP11B	1554557_at	3.639462	3.384242	2.965661	3.666794	3.645314	3.430624	3.671969	3.692216	4.158852	3.674184	3.888856	3.80078	3.64703
WDR22	1554558_at	3.183982	3.140114	3.108539	3.12642	3.17365	3.118707	3.129981	3.1365	3.126302	3.105587	3.12793	3.195657	2.946606
GPR62	1554559_at	2.206689	2.195332	2.168746	2.175615	2.710962	2.536195	2.183444	2.193646	2.171068	2.170975	2.176619	2.179133	2.091988
PGM5	1554560_at	1.852188	1.841462	1.822481	1.827808	1.844297	1.826405	1.837895	1.839142	1.827473	1.822544	1.823721	1.832876	1.781943
UNQ1887	1554564_a_at	4.620919	4.227151	3.610954	3.660525	4.722023	5.542582	4.023383	5.199429	3.968839	3.963344	5.751826	4.90881	3.557748
UNQ1887	1554565_x_at	6.007693	6.309085	4.907475	5.337625	5.982452	6.663558	5.048387	6.723822	5.544868	5.064643	6.645035	6.268758	4.70933
KCTD17	1554566_at	1.867543	1.860248	1.841075	1.845859	1.857617	1.845514	1.857812	1.856881	1.853642	1.847858	1.842688	1.851763	1.812304
DYNLRB1	1554568_at	2.205435	2.176953	2.157068	2.158547	2.180957	2.157973	2.171328	2.175928	2.161402	2.156086	2.15969	2.170351	2.07697
CUGBP2	1554569_a_at	2.780769	2.709768	2.686907	2.716046	2.786868	2.716857	2.752629	2.75924	2.748307	2.742522	2.74906	2.782467	2.931786
APBB1IP	1554571_at	2.79431	2.80441	2.742279	2.744395	2.777982	2.729482	2.710105	2.745482	2.740487	2.743797	2.742198	2.758808	2.62376
SUV39H2	1554572_a_at	5.013007	5.12765	4.967665	4.874161	4.745017	4.615765	7.384908	7.708138	7.686789	7.515554	7.619941	6.840631	6.787627
CYB5R3	1554574_a_at	4.61299	4.806221	6.002768	5.49934	3.559147	4.102348	6.906995	4.163216	7.031971	6.732849	3.721563	4.156701	8.514399
BPNT1	1554575_a_at	3.520887	3.523048	3.733119	3.402531	3.530741	3.303584	3.371043	3.357158	3.391685	3.387495	3.415887	3.42374	3.743764
ETV4	1554576_a_at	2.763914	2.707309	2.652064	2.740183	2.615873	3.334429	3.019655	2.930972	4.744584	3.157506	2.707566	3.083109	3.224689
PSMD10	1554577_a_at	4.685221	4.992243	5.812254	5.009313	4.316681	3.27457	7.003031	4.648487	7.318262	7.319162	4.362084	4.613952	8.973294
ZNF70	1554578_at	2.029853	2.015757	1.997628	1.995606	2.009145	2.000547	2.005337	2.009013	1.995415	1.988986	1.995796	2.005012	2.002878
MYO18B	1554579_a_at	2.15676	2.142321	2.109933	2.122871	2.146381	2.123248	2.140872	2.177638	2.590362	2.130485	4.181032	2.610608	3.190104
WWP2	1554580_a_at	3.581437	3.432858	3.299051	3.378183	3.653704	3.379969	3.353423	3.353577	3.416702	3.371318	3.361103	3.447201	2.91759
MGC50559	1554582_a_at	4.162741	4.117849	3.6771	3.733947	4.58173	3.879457	3.394944	3.646176	3.38758	3.46323	3.315106	3.534612	2.988095
MGC50559	1554583_a_at	2.742436	2.71454	2.692487	2.693503	2.710445	2.698152	2.708218	2.707234	2.695405	2.688659	2.693197	2.704288	2.513526
LRRC48	1554584_at	2.066244	2.048629	2.026851	2.035705	2.064472	2.035673	2.046819	2.048309	2.034501	2.027973	2.031084	2.056228	1.999283
RHOBTB2	1554586_a_at	2.52333	2.507129	2.425059	2.489887	2.507484	2.489282	2.489919	2.506127	2.491386	2.484763	2.549459	2.79388	2.435213
TTC30B	1554588_a_at	4.896626	4.34401	4.223238	4.084316	4.556568	4.850622	4.614522	4.331593	5.026945	3.963214	4.307461	5.099569	4.96085
LENG9	1554589_at	2.783856	2.774234	2.674298	2.749336	2.834232	2.749597	2.762279	2.774056	2.727071	2.741134	2.759738	2.767457	2.608417
GDEP	1554591_at	1.913733	1.907414	1.885678	1.891369	1.903861	1.895316	1.900514	1.904087	1.891362	1.892785	1.8848	1.895649	1.869308
SLC1A6	1554592_a_at	2.222568	2.200639	2.170625	2.183239	2.204912	2.177754	2.193353	2.199813	2.186199	2.177438	2.182994	2.193866	3.869822
SLC1A6	1554593_s_at	2.199134	2.173731	2.14942	2.158042	2.181429	2.15338	2.166927	2.172166	2.160296	2.153434	2.172137	2.171312	5.887564

LOC201175	1554594_at	2.014864	1.998464	1.975626	1.98319	2.000489	1.982597	1.992248	1.99748	1.982634	1.979114	1.978929	1.990728	1.931508
SYMPK	1554595_at	6.845212	7.046707	6.573793	6.909596	7.069779	7.680256	5.909359	6.372681	5.833799	5.645516	6.523634	6.404642	5.266203
LMNA	1554600_s_at	5.707397	5.448322	6.491508	6.200842	6.026916	5.889495	6.871953	7.072642	7.075299	6.9389	6.924161	6.798953	7.732776
TCBA1	1554601_at	2.132893	2.111685	2.089462	2.093456	2.115843	2.096153	2.106009	2.111675	2.097907	2.089151	2.09669	2.106124	2.048355
RBM8A	1554602_at	2.471529	2.578316	2.486155	2.522429	2.528187	2.434366	2.579002	2.581883	3.404176	2.899884	2.572002	2.570323	3.172607
MBTPS2	1554604_at	2.953364	2.927971	2.826669	2.888787	2.994313	2.910834	2.90342	2.925038	2.914664	2.909516	2.922154	2.970277	2.622761
CCDC100	1554606_at	2.910693	2.886701	2.873181	2.887706	3.013234	2.762485	3.173973	2.719412	2.904204	3.046705	2.857772	2.8797	5.269618
CNKSR2	1554607_at	2.679189	2.652119	2.636474	2.636092	2.654178	2.639177	2.640384	2.652591	2.632073	2.604331	2.637722	2.648302	2.571923
TGOLN2	1554608_at	6.258964	6.071354	5.991835	6.354075	5.345715	5.639408	5.128146	5.138199	4.970043	4.592361	4.677698	4.645806	2.638073
MGC12965	1554609_at	4.291323	4.321212	4.171753	4.108073	4.977915	4.345995	4.034092	4.152981	4.050368	4.131154	4.140224	4.145756	4.953428
ANKMY1	1554610_at	2.914701	2.883212	2.846879	2.841454	2.898259	2.868286	2.855606	2.862712	2.879055	2.858851	2.863711	2.87964	2.666765
KIAA0226	1554612_at	3.141762	3.021481	2.960689	2.99904	3.145883	2.99193	2.998053	3.020076	3.03375	3.002261	3.038809	3.121783	2.704213
KIAA0226	1554613_a_at	3.647565	3.627905	3.615458	3.62079	3.630751	3.620123	3.630182	3.633343	3.620696	3.61508	3.618126	3.624769	3.573048
PTBP2	1554614_a_at	2.309067	2.280313	2.260015	2.265914	2.289942	2.25278	2.279623	2.277518	2.280183	2.536576	2.261889	2.278481	6.974539
SERPINB8	1554616_at	2.170949	2.165329	2.141967	2.143922	2.171946	2.140427	2.158317	2.151105	2.154163	2.140826	2.132686	2.137532	2.063054
HRBL	1554618_at	2.243145	2.232142	2.218758	2.220059	2.229823	2.188153	2.379889	2.231436	2.668713	2.224206	2.212296	2.224129	2.124813
LOC153364	1554619_at	3.404081	3.243562	3.05754	3.192651	3.560812	3.286992	3.190545	3.193138	3.247971	3.166212	3.386907	3.287649	3.065864
DGKE	1554621_at	3.36828	3.215558	3.101252	3.221462	3.341934	3.160523	3.228153	3.234533	3.249008	3.217537	3.41456	3.326221	2.893162
DGKE	1554623_x_at	3.275035	3.19192	3.085624	3.167066	3.269991	3.161424	3.198672	3.274708	3.195486	3.168574	3.181537	3.233446	3.04244
SIRPB1	1554624_a_at	2.509494	2.464627	2.449956	2.449805	2.582329	2.444505	2.458997	2.47039	2.455997	2.439355	2.477581	2.46662	2.324695
BCL6B	1554625_at	2.037308	2.021077	2.013357	2.01139	2.024322	2.027841	2.028397	2.024253	2.01878	2.017485	2.007905	2.019783	2.000525
ASCC1	1554627_a_at	3.161028	3.171276	5.655748	5.066877	2.852524	2.550443	4.551735	3.134213	4.671814	5.0689	3.102227	2.904761	6.741892
ZNF57	1554628_at	3.302059	3.586255	3.216656	3.276396	4.290132	4.119053	3.164719	3.287626	3.034976	3.272872	3.757725	3.321617	3.557042
EPHA7	1554629_at	2.94468	2.868875	2.798577	2.894546	2.950303	2.873192	2.906311	2.915074	2.901836	2.901024	2.911245	2.920373	6.463904
ATM	1554631_at	2.958414	2.855451	2.870307	2.562733	2.94129	2.42356	3.06836	3.277047	3.060081	3.130721	2.922696	2.93172	2.840056
MYT1L	1554633_a_at	2.559655	2.520153	2.452793	2.506203	2.584262	2.482919	2.514978	2.525664	2.512278	2.492971	2.508928	2.524591	5.211879
PDS5B	1554634_at	3.133963	2.994952	2.922639	3.040211	3.21107	2.94302	3.045654	3.034415	3.041429	2.982085	3.062708	3.153902	2.697728
NPAS3	1554635_a_at	1.845466	1.839458	1.820463	1.823465	1.833697	1.826133	1.836003	1.836232	1.827329	1.826498	1.821218	1.827818	1.822024
CBFA2T2	1554637_a_at	2.019858	2.007957	1.983662	1.993132	2.004067	1.995016	2.005407	2.003765	1.999908	1.981512	1.990287	1.99684	3.919722
ZFYVE16	1554638_at	7.509429	7.774435	7.691374	8.000508	8.367932	8.316655	6.766966	6.959695	6.840286	6.713055	7.332944	6.91618	7.18987
PALM2	1554640_at	6.327226	6.197097	7.007583	6.839589	6.321426	6.269687	2.393112	2.811422	2.38886	2.380782	4.661489	3.180378	6.53704
MGC22014	1554641_a_at	2.114186	2.106623	2.079184	2.091814	2.105104	2.090355	2.104449	2.104796	2.099999	2.091583	2.090204	2.098984	2.015731
RNF32	1554642_at	2.069507	2.062538	2.039978	2.04512	2.056176	2.3721	2.050992	2.059567	2.047047	2.034921	2.041408	2.05689	2.014048
RGS11	1554643_at	2.879196	2.799459	2.729006	2.779975	2.866851	2.709378	2.787682	2.807735	2.796229	2.769709	2.832028	2.871743	2.523186
OSBPL1A	1554646_at	2.067723	2.159379	2.038741	2.042569	2.06279	2.049388	2.054677	2.056617	2.051663	2.037301	2.037615	2.049247	1.97701
DUOXA1	1554648_a_at	2.371614	2.353653	2.330498	2.325724	2.362107	2.33347	2.345208	2.349931	2.335783	2.329402	2.335518	2.345038	2.275306
LOC375449	1554652_s_at	1.771851	1.767094	2.108684	2.390555	1.754327	1.747418	1.754395	1.752673	1.749503	1.731697	1.736398	1.739846	1.693337
RPRML	1554655_a_at	2.11201	2.092134	2.066649	2.074084	2.096974	2.080836	2.080205	2.086046	2.07674	2.069221	2.072811	2.096258	2.021123

C20orf26	1554657_a_at	2.49152	2.423658	2.342802	2.406595	2.442635	2.420897	2.414468	2.426475	2.411144	2.400306	2.413076	2.569809	2.285862
NEK4	1554659_at	2.521937	2.496132	2.462768	2.479041	2.532047	2.476198	2.489291	2.489891	2.475023	2.465491	2.473228	2.482844	2.250024
C1orf71	1554660_a_at	2.816532	2.791689	3.344725	2.807836	2.828455	2.806382	2.725881	2.816839	2.81029	2.811318	2.809227	2.79111	2.78227
C1orf71	1554661_s_at	2.72904	2.629178	2.869867	3.426907	2.769211	2.724305	2.626062	2.623474	2.620738	2.630875	2.657131	2.616471	2.660182
C17orf47	1554662_at	2.296139	2.264457	2.239856	2.24533	2.274747	2.244527	2.252953	2.264595	2.258186	2.239076	2.247473	2.257725	2.15063
NUMA1	1554663_a_at	2.429547	2.394311	2.357302	2.382733	2.418378	2.387016	4.239347	2.408395	2.391803	2.377882	4.736392	2.402069	4.175251
C9orf90	1554664_at	2.613598	2.602105	2.580449	2.583772	2.627496	2.587589	2.587965	2.596372	2.584408	2.563791	2.581737	2.593024	2.462334
ZNF587	1554665_at	2.64357	2.619344	2.586714	2.59944	2.688801	2.602436	2.607622	2.621763	2.608495	2.599253	2.613455	2.631938	2.532364
LOC284017	1554666_at	2.617202	2.560823	2.529608	2.535368	2.559766	2.535852	2.547912	3.073359	2.551491	2.53976	2.543259	2.553773	2.466285
METTL8	1554667_s_at	5.313464	4.933917	4.698459	4.832185	5.206769	5.406047	6.272501	6.5372	6.503914	6.31778	6.549427	6.479443	6.055117
C1orf179	1554668_a_at	5.66684	5.168532	3.817982	4.270804	3.788723	3.703371	6.730061	3.283563	6.36155	5.253787	5.883391	5.756763	3.081882
GGA1	1554670_at	3.939366	4.14105	3.875875	3.615537	3.663638	3.626257	3.578563	3.541974	3.569664	3.498626	3.543853	3.437994	3.134137
SRRM2	1554671_a_at	2.501455	2.467992	2.417854	2.449654	2.462679	2.479214	2.658774	3.124121	2.448261	2.486726	2.570409	2.491572	2.333024
TTC26	1554672_at	2.738254	2.705246	2.67047	2.687398	2.727107	2.685817	2.698528	2.704898	2.682903	2.651248	2.684518	2.703184	2.509245
SYCE1	1554675_a_at	2.517743	2.496707	2.445636	2.47651	2.519007	2.472576	2.486621	2.497156	2.482859	2.472469	2.479204	2.489674	2.336574
SRGN	1554676_at	2.702831	2.651013	2.601092	2.632157	2.80549	2.617929	2.634397	2.665642	2.653578	2.627536	2.682247	2.684352	2.404238
CMTM4	1554677_s_at	2.061219	2.046378	2.014442	2.032052	2.05388	2.027047	2.035402	2.04329	2.038814	2.022551	2.029171	2.038226	1.994499
HNRPDL	1554678_s_at	7.031959	7.065142	8.552278	8.164553	7.097444	5.942978	9.733049	7.711496	9.335736	9.273071	8.101935	8.019097	11.25041
LAPTM4B	1554679_a_at	2.776541	3.219805	4.848619	3.843997	3.112069	2.650068	8.202447	3.214086	6.603176	8.324321	2.681676	2.972966	9.689862
KCNS2	1554680_s_at	1.803552	1.792511	1.77184	1.774495	1.794617	1.777647	1.790633	1.792784	1.780582	1.776538	1.772502	1.855014	1.743028
MGC50722	1554681_a_at	2.59251	2.551695	2.504755	2.53342	2.554118	2.530686	2.54123	2.549888	2.535342	2.527336	2.531748	2.544535	2.420477
MGC50722	1554683_a_at	3.023906	3.011704	2.973918	2.979989	3.010933	2.994338	3.006022	3.015246	2.996523	2.990809	3.127462	3.03312	2.921817
KIAA1199	1554685_a_at	2.052999	2.035026	2.012445	2.016778	2.034785	2.016976	2.028944	2.031877	2.018934	2.012274	2.013931	2.026833	1.965276
STAU2	1554686_at	3.321485	3.097755	3.13294	3.185351	3.711143	3.793844	3.15464	3.216042	3.207408	3.133768	3.327328	3.259114	2.895484
NLGN4X	1554689_a_at	2.956065	2.894097	2.877356	2.886547	2.924342	2.890559	2.901028	2.921868	2.890788	2.869836	2.923485	2.916964	5.740857
TACC1	1554690_a_at	5.383283	5.049273	5.493632	5.576499	5.655746	5.525052	5.924286	6.310734	6.395934	5.947052	6.807204	6.241132	4.828676
PACSIN2	1554691_a_at	3.784189	3.883512	3.802454	3.708304	3.746426	3.711802	3.720034	3.577308	4.143464	3.925709	3.474982	3.635875	5.915518
SLC23A2	1554692_at	2.33415	2.324872	2.282822	2.309004	2.326902	2.308348	2.31984	2.321925	2.308589	2.308492	2.306826	2.317874	2.234719
CCNY	1554694_at	2.612231	2.575556	2.527616	2.55628	2.599096	2.561151	2.568041	2.576214	2.56324	2.549229	2.558836	2.575992	2.491602
TYMS	1554696_s_at	4.310689	3.8417	2.620829	2.809599	3.004897	2.984605	9.397044	7.118656	9.318751	9.304266	6.623542	6.862006	8.708165
ADAMTS9	1554697_at	2.798877	2.785819	2.769293	2.754231	2.785109	2.773518	2.791436	2.778784	2.800992	3.013925	2.770411	2.779166	7.919017
L3MBTL4	1554699_at	1.981569	1.931735	1.917024	1.920161	1.931424	1.919255	1.92423	1.92822	1.914237	1.909638	1.916446	1.921057	1.844161
CDH7	1554700_at	3.372982	3.190196	3.107037	3.206639	3.394383	3.144895	3.220598	3.211705	3.24729	3.195539	3.294295	3.334586	2.858578
TBC1D16	1554701_a_at	3.171638	3.137102	3.516389	3.523404	3.687933	4.789942	3.09201	3.977936	3.573315	3.158288	4.058882	4.51874	3.537308
NALCN	1554702_at	1.980139	1.964936	1.945186	1.949212	1.96533	1.94758	1.960053	1.962294	1.95039	1.94432	1.946219	1.956886	1.899582
ARHGEF10	1554703_at	4.835587	4.982724	4.688627	4.900385	4.485569	5.293539	4.274671	4.112001	4.096925	4.075985	6.776589	4.336905	6.899727
ATP8B3	1554704_at	3.232873	3.164795	3.138572	3.173238	3.021638	3.194377	3.320253	4.44543	3.208179	3.727668	3.970483	3.97739	2.714801
SCARA5	1554705_at	2.563784	2.535255	2.488974	2.432766	2.551402	2.601991	2.530674	2.538475	2.527393	2.510947	2.516974	2.528931	2.461489

OR2L13	1554706_at	3.123947	2.943084	2.846934	2.964341	3.07248	2.926296	2.96945	2.989883	2.996025	2.942866	3.044813	3.116151	2.639988
C9orf68	1554707_at	3.116921	3.062131	2.962888	3.017446	3.203561	3.014455	3.024879	3.066035	3.04352	3.029489	3.06742	3.162154	2.705022
C9orf68	1554708_s_at	3.900724	3.588341	3.704438	3.704598	4.020871	4.17031	2.994932	3.124497	3.137229	3.085966	3.135716	2.993534	2.637345
KCNMB1	1554710_at	2.494301	2.469167	2.428242	2.448408	2.485242	2.452948	2.456981	2.469775	2.453363	2.44307	2.454247	2.490225	2.363396
FAM26A	1554711_at	2.085653	2.067787	2.041731	2.057607	2.071123	2.055952	2.067385	2.068867	2.05067	2.056423	2.051058	2.06152	2.017072
GLYATL2	1554712_a_at	2.628866	2.604185	2.588173	2.587705	2.605425	2.593013	2.592951	2.602382	2.594874	2.569813	2.605422	2.609338	2.4242
RELL1	1554714_at	2.087147	2.066626	2.042864	2.052251	2.069393	2.048406	2.0603	2.065688	2.052079	2.044199	2.050627	2.060595	2.020992
C15orf50	1554715_at	2.505331	2.469308	2.41813	2.446831	2.493297	2.431669	2.456125	2.468439	2.453699	2.436465	2.450958	2.464814	2.330498
C15orf50	1554716_s_at	2.098598	2.078262	2.053126	2.059869	2.078208	2.058149	2.069878	2.075397	2.062544	2.055011	2.061921	2.070106	1.991997
PDE4D	1554717_a_at	2.238348	2.200113	2.183138	2.181242	2.225724	2.190741	2.198872	2.198919	2.1896	2.184538	2.200131	2.202512	2.100245
NDUFA10	1554719_at	2.206525	2.17626	2.121539	2.156788	2.188589	2.157017	2.158172	2.170526	2.159222	2.140974	2.160965	2.168296	2.023934
TAF2	1554721_a_at	2.17184	1.998176	2.143723	2.140898	2.159963	2.315137	2.158121	2.159155	2.143377	2.150644	2.144617	2.152813	2.766651
TRPM3	1554722_at	3.047122	2.961508	2.904603	2.952235	3.034667	2.950702	2.996845	2.962789	2.987128	2.939249	2.997662	3.263588	3.270317
SLC6A11	1554724_at	2.330057	2.30874	2.281224	2.29061	2.320161	2.287793	2.299643	2.307605	2.29355	2.280841	2.293037	2.310298	2.183593
RABGAP1L	1554725_at	2.202437	2.18209	2.151175	2.163135	2.188696	2.166794	2.171323	2.181499	2.167104	2.158864	2.165385	2.176374	2.081326
ZNF655	1554726_at	3.225046	2.860547	3.104909	3.171982	3.20417	3.114425	3.180358	3.057589	3.651841	3.184315	3.170854	3.145485	5.72778
SLC9A1	1554728_at	3.556265	3.476817	3.305031	3.454797	3.555435	3.413919	3.462945	3.471464	3.458634	3.408727	3.461786	3.545719	3.357522
MCTP1	1554730_at	3.095798	3.006816	2.980411	2.988451	3.012759	2.994461	2.995424	3.006031	2.997258	2.897701	3.014711	3.000901	2.930068
MGC24125	1554732_at	1.850214	1.836696	1.818656	1.823047	1.834779	1.822769	1.833707	1.834437	1.831923	1.819462	1.816524	1.828616	1.787208
MGC24125	1554733_at	3.585066	3.452814	3.649602	3.461807	3.7017	3.405001	3.45964	3.455882	3.460214	3.36764	3.466388	3.507926	2.94141
TRIM7	1554735_a_at	1.989503	1.97582	1.954626	1.959821	1.976614	1.958579	1.970332	1.971793	1.961246	1.956309	1.953212	1.966112	1.911109
ARHGAP29	1554736_at	3.115434	3.032786	2.967576	3.021319	3.096565	3.003501	2.985823	3.037215	3.030166	2.975013	3.255446	3.050244	2.682933
FBN2	1554737_at	2.184583	2.16156	2.14032	2.143271	2.16409	2.14399	2.153114	2.158188	2.145524	2.138364	2.146223	2.153238	2.219905
IPP	1554739_at	3.244454	3.02347	2.999526	3.104989	3.28232	3.032966	3.086845	3.095237	3.099937	3.061707	3.09265	3.219626	2.966873
IPP	1554740_a_at	2.453187	2.431299	2.591418	3.297645	2.49802	2.492365	2.983954	2.430624	2.783527	3.33849	2.617914	2.423597	4.666742
FGF7	1554741_s_at	6.172916	5.791989	5.639462	5.941736	6.203638	6.363757	5.30473	5.239777	4.863224	5.153952	6.236297	5.783586	7.679535
PMS1	1554742_at	2.751647	2.659511	2.60507	2.6546	2.984115	2.624718	2.649873	2.661604	2.686103	2.638213	2.730589	2.874323	2.564649
PMS1	1554743_x_at	2.388974	2.354684	2.312888	2.3361	2.372224	2.32521	2.357948	2.358221	2.352128	2.328747	2.344152	2.369084	2.311959
RFWD2	1554744_at	2.880627	2.788628	2.733983	2.784535	2.874051	2.754314	2.797393	2.799748	2.794067	2.765963	2.802775	2.839661	2.559044
RALGPS1	1554745_at	2.873615	2.855103	2.774958	3.030395	2.875738	2.821419	2.862465	2.863325	2.845455	2.833633	2.889267	2.910827	2.626616
SEPT2	1554747_a_at	5.522091	5.552924	5.759144	5.346637	4.603481	5.091315	7.121255	5.672251	7.296032	7.469584	6.040249	5.641575	9.924394
CLCNKB	1554748_at	2.62528	2.802285	2.351232	2.351636	2.386078	2.351231	2.358944	2.370988	2.344245	2.606465	2.35544	2.365919	2.607382
CLCNKB	1554749_s_at	1.6618	1.655396	1.636571	1.639564	1.647644	1.638345	1.649882	1.65012	1.647461	1.633505	1.631023	1.63602	1.626941
KIAA0774	1554755_a_at	2.976302	2.955651	2.83008	2.936191	2.963869	2.910517	2.951076	2.957005	2.942238	2.939504	2.943739	2.950493	4.563021
INPP5A	1554757_a_at	2.638223	2.616863	2.644013	2.596493	2.626047	2.593022	3.029624	2.630274	3.706466	4.032699	2.614391	2.624053	5.341494
CD99L2	1554758_a_at	2.450864	2.686101	2.533343	2.4187	2.462507	2.361456	2.415031	2.431918	2.425388	2.410517	2.394417	2.42671	2.709451
SNIP	1554759_at	3.803013	3.63433	3.56189	3.725003	3.789448	3.572666	3.787639	3.789343	3.788489	3.772061	4.050391	4.109991	3.341719
HEATR2	1554761_a_at	2.875297	2.716625	3.133056	3.778217	2.773079	2.689127	4.639181	2.804377	4.841481	4.612083	2.819579	2.859535	4.373891

WWC2	1554762_a_at	4.603432	4.305199	4.700171	4.762878	4.69927	4.314961	4.267089	3.822108	3.806445	4.155885	4.270421	4.247793	4.238036
UBE2DNL	1554763_at	2.122216	2.106066	2.076089	2.086533	2.108096	2.086346	2.09904	2.100582	2.088711	2.087368	2.08467	2.096484	2.031756
C11orf64	1554764_a_at	3.293714	3.126884	3.049195	3.125789	3.305759	3.132049	3.165787	3.137456	3.169736	3.104712	3.203822	3.25036	2.791274
C11orf64	1554765_a_at	2.888284	2.850877	2.805872	2.814744	2.873225	2.827613	2.841619	2.854914	2.838842	2.823593	2.83774	2.885145	2.699889
CRYZL1	1554767_s_at	3.345987	3.2227	3.268577	3.29373	3.329555	3.124099	3.314788	3.198133	3.685318	3.975844	3.28625	3.307225	6.695031
MAD2L1	1554768_a_at	2.468381	2.460783	2.441506	2.435352	2.454812	2.448945	6.937188	2.517131	7.074766	6.663975	2.44511	2.452486	6.946813
ZNF785	1554769_at	4.378178	4.161524	3.651908	3.884658	4.220075	3.931138	4.450112	3.963172	3.998847	4.25194	4.369551	4.38591	5.005073
ZNF785	1554770_x_at	4.971781	4.266562	3.936959	3.963883	4.255605	4.05221	4.568566	4.106343	4.085283	4.407608	5.024559	4.192953	4.028481
C17orf57	1554772_at	2.030803	2.013446	1.996062	1.992441	2.01061	1.987224	2.01389	2.009957	2.010371	2.000438	1.9965	2.005141	1.975223
WFIKKN2	1554773_at	1.778603	1.768735	1.751457	1.754545	1.767625	1.75339	1.764642	1.765804	1.75612	1.755613	1.752783	1.760391	1.756369
MINA	1554774_at	2.084739	2.059339	2.044531	2.053705	2.064407	2.046378	2.112868	2.010762	2.049785	2.051435	2.004965	2.052091	3.258854
ZFP42	1554776_at	1.957389	1.945129	1.924236	1.928373	1.946236	1.927679	1.940358	1.942603	1.930073	1.923007	1.925359	1.937684	1.875816
ZFP42	1554777_at	2.548361	2.504888	2.457855	2.494372	2.595617	2.471714	2.494189	2.511279	2.497166	2.474906	2.49612	2.528305	2.342984
PHLDB2	1554778_at	3.314825	3.162103	3.11027	3.178217	3.524915	3.127515	3.190938	3.197716	3.207903	3.160583	3.254472	3.353568	2.869805
PHLDB2	1554779_s_at	2.465191	2.428482	2.384116	2.412031	2.451112	2.419153	2.421536	2.43066	2.417156	2.410908	2.414779	2.428602	2.280214
PHTF2	1554780_a_at	3.890815	3.382585	3.836676	4.021816	3.865839	3.866329	3.897224	4.130895	3.827115	3.845681	4.544101	3.857145	4.419536
MGC32805	1554781_at	5.869412	5.569762	5.85711	5.36096	4.831483	3.175402	2.743725	2.75318	2.735035	2.725846	2.737127	2.742894	2.531331
C2orf19	1554782_at	2.106767	2.09755	2.079557	2.085274	2.095813	2.085895	2.09078	2.093509	2.078944	2.083139	2.077404	2.127145	2.028254
ARHGEF2	1554783_s_at	2.19628	2.17432	2.143225	2.160501	2.183147	2.157967	2.168208	2.173263	2.163159	2.154851	2.164603	2.173259	2.172078
CNTN1	1554784_at	3.01319	2.938022	2.89052	2.934079	3.06063	2.910567	2.912015	2.961502	2.932281	2.916552	2.949638	3.018726	2.842288
CCDC82	1554785_at	2.267568	2.240883	2.206715	2.220207	2.250747	2.2181	2.234316	2.243265	2.236169	2.218444	2.229015	2.236833	2.4192
C20orf32	1554786_at	2.766075	2.518514	2.487928	2.497461	2.53739	2.486706	2.503382	2.523399	2.507506	2.493027	2.510683	2.518349	2.334056
C9orf96	1554787_at	2.852271	2.838249	2.813753	3.035096	2.84685	2.809745	2.830796	3.255223	2.820791	2.810108	2.822398	2.980869	2.720191
PDE8B	1554789_a_at	2.559061	2.526744	2.53233	2.516657	2.659625	2.515824	2.511641	2.518488	2.503411	2.500979	2.511385	2.538718	3.431921
ZSCAN20	1554790_at	2.098738	2.07968	2.053142	2.059688	2.093114	2.064913	2.073617	2.074493	2.069549	2.063724	2.05945	2.122986	2.044229
FLJ23861	1554791_a_at	2.723447	2.601207	2.583964	2.575753	2.705777	2.590226	2.589524	2.597451	2.58265	2.56529	2.587122	2.592033	2.485034
UBE3C	1554793_at	3.807295	3.575894	3.589878	3.597187	3.928508	3.769018	3.707614	3.683426	3.838308	3.647953	3.795949	4.229637	3.43091
UBE3C	1554794_a_at	3.484937	3.348434	3.335982	3.371644	3.48345	3.357594	4.344629	3.465861	4.266643	4.405004	3.516003	3.550054	4.763954
FBLIM1	1554795_a_at	2.751667	2.915937	2.715636	2.717503	2.741633	2.720289	2.736544	2.740128	2.724851	2.720152	2.725683	2.813993	3.141508
DPP6	1554796_at	1.883599	1.878946	1.858984	1.862273	1.871245	1.867342	1.86991	1.873786	1.860463	1.86353	1.861033	1.863833	1.852723
SYT16	1554797_at	2.499279	2.46238	2.441996	2.446404	2.485191	2.448479	2.455084	2.460742	2.450977	2.435538	2.449334	2.462083	2.318009
SNED1	1554798_at	2.28187	2.255463	2.164926	2.236903	2.314102	2.238182	2.249424	2.25503	2.247439	2.23658	2.225857	2.247234	2.212771
RAB39	1554800_at	2.396242	2.367497	2.333124	2.356485	2.423264	2.357046	2.357177	2.369162	2.35361	2.355531	2.354199	2.414787	2.233395
C5orf40	1554801_at	2.651452	2.596798	2.591598	2.585457	2.61858	2.558724	2.601781	2.635925	2.597027	2.579537	2.601781	2.636796	2.775267
TRIM72	1554803_s_at	3.363093	3.271167	3.14198	3.210926	3.328758	3.259841	3.179704	3.818982	3.260053	3.19277	4.235382	3.28742	3.776333
CLDN19	1554804_a_at	5.900865	5.561947	4.328739	4.909183	4.938224	5.516787	6.979346	7.653569	7.007311	6.293506	6.694982	7.754571	4.943852
CLDN19	1554805_at	2.287491	2.269826	2.224972	2.221035	2.270947	2.221559	2.396382	2.268518	2.288728	2.24657	2.623911	2.336796	2.145365
FBXO8	1554806_a_at	6.44642	6.003737	7.83889	7.380459	5.719728	4.183426	7.249401	5.066033	7.07183	7.339372	4.788611	5.525801	6.754947

SPIRE1	1554807_a_at	2.682644	2.634261	2.962067	2.665825	2.673007	2.632612	2.694736	2.651685	2.667769	2.644829	2.674829	2.66804	5.207275
ACP1	1554808_at	2.590215	2.550411	2.52862	2.531178	2.579156	2.510318	2.542181	2.549929	2.575169	2.520064	2.530967	2.555846	2.543737
LOC389199	1554809_at	3.986244	3.865466	3.836698	3.848431	3.874599	3.846912	3.850039	3.858877	3.842748	3.835175	3.837798	3.855975	3.639428
PLA2G4C	1554810_at	3.182014	3.522799	2.887939	2.932927	2.936866	2.919967	2.895813	2.93374	2.913801	2.848382	2.921621	2.922943	2.859752
CLDN20	1554812_at	2.79402	2.769137	2.706747	2.731183	2.776336	2.746134	2.757404	2.764725	2.753144	2.724385	2.747017	2.781634	2.654325
UBE2O	1554814_at	2.443609	2.431042	2.358474	2.414602	2.429941	2.413114	2.434547	2.460598	2.429639	2.421033	2.499883	2.424615	2.374097
ASTN2	1554816_at	2.590682	2.559585	2.515804	2.538908	2.57405	2.5371	2.549802	2.553944	2.54367	2.53497	2.543026	2.554919	2.38119
C20orf12	1554818_s_at	2.548055	2.506724	2.461711	2.495616	2.51811	2.493817	2.506819	2.516727	2.502417	2.497997	2.508587	2.567358	2.357505
ITGA11	1554819_a_at	2.523405	2.576859	2.437068	2.468385	2.509341	2.468796	2.47687	2.487996	2.473062	2.458044	2.469911	2.486307	2.456001
AGBL3	1554820_at	2.791262	2.718857	2.661085	2.721281	2.780098	2.712066	2.730031	2.740606	2.72437	2.697332	2.724189	2.757363	2.511245
ZBED1	1554821_a_at	4.67639	4.305976	5.343254	5.40354	5.148215	4.953621	4.170145	3.605147	3.559796	3.19748	4.268893	4.272429	4.001472
PHTF2	1554822_at	2.654729	2.616677	2.595226	2.603471	2.635588	2.598104	2.617216	2.605176	2.601067	2.601253	2.592931	2.607619	2.622327
ZNF585A	1554824_at	3.13832	3.132986	3.113655	3.146457	3.143436	3.226508	3.117879	3.079136	3.11473	3.107071	3.114263	3.118886	3.125154
ALS2CR7	1554826_at	2.706251	2.656227	2.602115	2.639019	2.778365	2.757992	2.648109	2.658182	2.642554	2.634313	2.646753	2.730011	2.456456
ADCY7	1554827_a_at	1.792369	1.785257	1.767175	1.769933	1.780081	1.775119	1.783229	1.781725	1.777914	1.773416	1.767501	1.773863	1.746788
PDGFRA	1554828_at	2.159253	2.13213	2.110886	2.107937	2.142533	2.113617	2.128522	2.127261	2.115704	2.105751	2.503856	2.121475	2.057817
STEAP3	1554830_a_at	6.265216	6.030692	6.200315	5.72761	5.776961	6.236593	6.1689	5.67077	5.973726	6.272692	5.058823	5.747826	2.57717
ALS2CR11	1554831_x_at	2.501688	2.476581	2.437441	2.450508	2.480283	2.456793	2.463772	2.470769	2.458942	2.444039	2.458903	2.489789	2.341512
MCTP2	1554833_at	1.734864	1.714774	1.697949	1.701436	1.714066	1.702994	1.713464	1.714706	1.704547	1.700468	1.700321	1.706897	1.669635
RASSF5	1554834_a_at	2.705364	2.690283	2.672986	2.676191	2.689659	2.674267	2.685868	2.68723	2.676364	2.6766	2.667964	2.671995	2.626835
B3GNT5	1554835_a_at	2.118633	2.097249	2.075356	2.081089	2.099399	2.082411	2.089553	2.166663	2.083843	2.079646	2.087553	2.096511	3.898131
USP36	1554836_at	2.197496	2.181476	2.153968	2.160454	2.181195	2.158962	2.163518	2.177979	2.16259	2.146256	2.159576	2.172593	2.02282
CYP4A11	1554837_a_at	6.671314	6.830053	7.725672	7.779369	8.559538	8.720559	3.096785	2.937992	2.219311	2.889152	2.729074	2.428432	1.973904
CIDEC	1554839_at	3.202294	3.095527	3.154168	3.164926	3.189569	3.161686	3.321779	3.16511	3.186157	3.163349	3.176164	3.178034	3.164428
LOC280665	1554840_at	2.486687	2.415267	2.463345	2.465312	2.492752	2.464112	2.475016	2.47285	2.485597	2.243002	2.461431	2.46214	2.111458
MTHFD2L	1554841_at	4.929663	4.568827	5.157835	4.68508	5.677718	5.896401	3.883183	3.867643	3.950975	3.860412	3.945113	3.911099	3.576076
SLC12A1	1554842_at	4.443596	4.552718	4.530888	4.417665	4.571623	4.840667	4.546924	5.44423	4.391825	4.31633	4.531842	4.972106	3.66503
EYA3	1554844_at	2.666918	2.612834	2.578303	2.592632	2.756641	2.587219	2.600798	2.548881	2.595866	2.57684	2.598941	2.709537	2.424055
FLJ25770	1554846_at	2.483842	2.450911	2.404558	2.431028	2.482523	2.421833	2.439551	2.449842	2.434253	2.413298	2.433405	2.448081	2.306784
ATP6V1B1	1554847_at	3.073555	3.056125	2.999576	3.60309	3.082223	3.044738	3.045808	3.055377	3.031842	3.02408	3.039099	3.050286	2.704472
TOR1A	1554849_at	2.568004	2.536703	2.509154	2.521412	2.591836	2.504229	2.53911	2.541653	2.532602	2.506549	2.528742	2.5918	2.339859
LOC90113	1554850_at	2.34063	2.318692	2.291956	2.299059	2.320686	2.297888	2.307673	2.307957	2.298082	2.284523	2.297445	2.305395	2.230004
KIAA1257	1554852_a_at	2.369607	2.259601	2.321238	2.329828	2.352257	2.315155	2.333032	2.349068	2.332588	2.328962	2.335006	2.342605	2.245872
CYP2U1	1554853_at	3.069325	2.976366	2.924398	2.965474	3.060812	2.945934	2.974809	2.986144	2.971433	2.929649	2.974991	3.001589	2.793488
PARK2	1554855_at	2.491492	2.45677	2.407629	2.432689	2.481575	2.422792	2.444137	2.454877	2.443155	2.428135	2.440405	2.452033	2.315925
ELMO2	1554857_at	2.377655	2.362973	2.34651	2.343031	2.379455	2.351048	2.357686	2.376816	2.354367	2.338096	2.341553	2.351477	2.293243
PTPN7	1554860_at	3.334899	3.236354	3.225551	3.22898	3.24602	4.014689	3.180726	3.241764	3.224275	3.203722	3.22651	3.243062	2.808613
CMAH	1554862_at	2.38742	2.362405	2.346118	2.348965	2.381955	2.331326	2.358881	2.366947	2.349593	2.333526	2.35151	2.361518	2.221472

DOK5	1554863_s_at	2.489801	2.457122	2.418089	2.438012	2.465563	2.437799	2.445411	2.452842	2.441282	2.426448	2.440166	2.452535	6.863912
SDC3	1554864_a_at	2.399242	2.373007	2.341429	2.354771	2.391987	2.362136	2.362472	2.373259	2.358623	2.342916	2.357109	2.369428	2.328787
TMEM135	1554866_at	2.856875	2.802575	2.732637	2.787123	2.87178	2.802002	2.812274	2.815874	2.806941	2.79752	2.805123	2.821401	2.62947
PRR16	1554867_a_at	2.349063	2.307556	2.293624	2.287456	2.309902	2.529486	2.295609	2.309592	2.293011	2.286681	2.29161	2.364921	4.18533
PCNP	1554868_s_at	6.132074	5.695089	7.338511	6.573416	5.743338	5.692521	8.644728	6.240898	8.86154	8.782636	6.268484	6.663973	9.741174
WDR37	1554869_at	2.953536	2.893025	2.876974	2.936535	2.94178	2.87759	2.874963	2.868841	2.862623	2.845122	2.885985	2.870512	2.75829
HMGCLL1	1554872_a_at	2.261813	2.232056	2.207671	2.214152	2.228794	2.212122	2.180068	2.226026	2.199694	2.191853	2.208844	2.220988	2.72494
CSPP1	1554873_at	3.818403	3.61099	3.517851	3.638267	3.960729	3.587142	3.697881	3.577578	3.669313	3.634708	3.731568	3.794621	3.927115
MITF	1554874_at	2.45711	2.424331	2.401294	2.404854	2.441202	2.395044	2.41254	2.42409	2.409142	2.398404	2.418594	2.422002	2.278229
C2orf34	1554875_at	2.531063	2.510632	2.499624	2.497261	2.517369	2.506076	2.523059	2.519886	2.69407	2.571004	2.519858	2.516891	2.353915
S100Z	1554876_a_at	2.977431	2.950612	2.916281	2.932389	3.101023	2.932325	2.938984	2.922319	2.93484	2.925287	2.978358	2.954549	2.613229
ABCD3	1554878_a_at	3.522706	3.197345	3.73824	3.357859	3.2352	3.18865	4.024879	3.256395	5.075091	3.963833	2.993752	3.044271	5.056019
DKFZP434K	1554880_at	2.616058	2.672361	2.626489	2.659548	2.701103	2.638806	2.661136	2.673525	2.658479	2.644256	2.655788	2.668108	2.528962
ERCC8	1554882_at	3.217875	3.056121	3.038955	3.091548	3.223508	3.058149	3.113603	3.127678	3.127476	3.078086	3.302896	3.393861	2.715817
ERCC8	1554883_a_at	2.584024	2.543989	2.55202	2.557819	2.577841	2.508343	2.58473	2.632033	2.824376	2.831276	2.568229	2.573397	4.934933
PRIM2	1554884_at	2.64816	2.604492	2.542834	2.58321	2.63692	2.560747	2.595959	2.603857	2.592984	2.582593	2.591981	2.612769	2.420651
PRIM2	1554885_a_at	2.420655	2.385907	2.353841	2.379004	2.409867	2.363359	2.491046	2.338822	2.63942	2.395976	2.427495	2.434721	2.899021
MLXIP	1554886_a_at	2.710456	2.674073	2.554092	2.656518	2.749375	2.630365	2.664927	2.672405	2.957889	3.112875	2.65518	2.675328	2.342606
TIA1	1554889_at	3.853967	3.513463	3.636418	3.657814	3.817287	3.459073	4.24532	3.682152	4.044198	4.117425	3.774706	3.865479	3.88769
TIA1	1554890_a_at	3.914865	3.905933	3.69803	3.893279	3.905506	3.640196	4.776777	3.901324	4.551203	4.717417	4.624931	4.46208	7.007283
MS4A3	1554892_a_at	2.844841	2.775386	2.754977	2.753822	2.817855	2.755131	2.76917	2.780245	2.7631	2.732157	2.766803	2.787057	2.435736
PCBD2	1554894_a_at	5.567824	5.179392	5.448791	5.85558	5.615179	5.572215	6.170214	6.305464	6.349297	6.374713	6.222677	6.308434	4.489838
RHBDL2	1554895_a_at	2.189687	2.143791	2.113706	2.125114	2.178649	2.132278	2.13284	2.142299	2.128901	2.123732	2.126623	2.154896	2.042397
RHBDL2	1554897_s_at	2.652298	2.607453	2.562045	2.592366	2.642217	2.575613	2.595173	2.609588	2.595992	2.575181	2.591965	2.60896	2.514233
FCER1G	1554899_s_at	4.421499	4.639033	6.841741	6.141778	4.453041	3.289678	5.884127	3.953787	6.004677	5.350527	4.186819	4.626089	4.554637
FRMD8	1554903_at	1.039706	1.031259	1.012394	1.016197	1.022622	1.012394	1.030456	1.015674	1.026579	1.015379	1.005703	1.007616	1.031089
FRMD8	1554905_x_at	1.621848	1.605238	1.596541	1.596531	1.604302	1.59467	1.61287	1.601861	1.609717	1.596531	1.588884	1.596593	1.613746
MPHOSPH6	1554906_a_at	3.250748	3.049394	3.079021	3.087127	3.239467	3.726875	3.15834	4.804523	3.223398	3.410613	4.674825	4.54401	2.843805
HYDIN	1554907_a_at	3.131348	3.037475	3.024966	3.041226	2.997003	3.01295	4.104079	3.156668	3.169055	3.763698	3.312175	3.123086	3.513427
HYDIN	1554908_at	2.608669	2.529458	2.500063	2.510124	2.536704	2.511721	2.520401	2.522606	2.512704	2.508915	2.513466	2.523238	2.46081
CCDC36	1554909_at	2.557395	2.528683	2.480349	2.523007	2.563023	2.524654	2.537472	2.539452	2.524709	2.520148	2.528673	2.539594	2.489136
PRKD3	1554910_at	3.084234	2.93301	2.932065	3.015448	3.038911	2.944433	2.966771	2.963846	2.960022	2.949989	2.961795	3.005244	2.856968
ABCC11	1554911_at	1.911979	1.886199	1.86585	1.869269	1.877884	1.868692	1.88025	1.88215	1.870754	1.863091	1.866899	1.876604	1.829838
FAM62C	1554912_at	2.795814	2.73472	2.663888	2.712021	2.784235	2.684705	2.725818	2.7355	2.721648	2.698856	2.730683	2.769974	2.591895
PLA2G4D	1554914_at	2.122592	2.102075	2.081131	2.08417	2.105966	2.084057	2.093513	2.096745	2.087045	2.07936	2.080388	2.09291	2.013298
2'-PDE	1554915_a_at	5.350768	5.635017	5.884867	5.634803	5.548579	5.225447	6.755584	6.626589	7.212554	6.825054	6.076251	6.209899	5.672565
JRK	1554916_a_at	2.429704	2.414152	2.367398	2.396197	2.417947	2.395914	2.401052	2.413625	2.398267	2.391406	2.398432	2.407923	2.301099
ABCC4	1554918_a_at	2.692688	2.656881	2.610626	2.628865	2.675733	2.596782	4.150121	4.118685	3.590978	4.987126	2.658054	3.491669	2.986197

FLJ21062	1554919_s_at	3.33802	3.328492	3.513236	3.343717	3.513655	3.206302	3.332027	3.295977	4.109979	3.272785	3.316026	3.376698	3.976736
SCEL	1554920_at	4.050198	3.706525	3.634946	3.828119	4.267199	3.668404	3.772091	3.767725	3.877369	3.766824	4.003306	4.180656	3.176835
SCEL	1554921_a_at	3.230496	3.078039	2.981357	3.103986	3.267289	3.074801	3.098443	3.136888	3.121964	3.098494	3.372747	3.262419	2.950235
ZNF678	1554922_at	3.251454	3.104988	3.038483	3.111422	3.282407	3.102031	3.117975	3.129067	3.143334	3.090108	3.187026	3.271109	2.925773
ANKS6	1554923_at	2.62621	2.588054	2.524261	2.563751	2.614733	2.54158	2.575798	2.585418	2.572641	2.552812	2.568546	2.590843	2.502567
LOC646982	1554927_at	2.541228	2.504303	2.485744	2.488987	2.512518	3.55373	2.494373	2.505559	2.491219	2.478007	2.48833	2.498431	2.395819
KIAA0999	1554929_at	2.425549	2.404897	2.388539	2.386083	2.407946	2.383038	2.397605	2.404871	2.393419	2.381174	2.389143	2.398378	2.261156
FUT8	1554930_a_at	2.245465	2.216688	2.203438	2.204082	2.218519	2.196098	2.219724	2.214551	2.220933	2.208451	2.196633	2.20962	4.956354
CYP4A11	1554931_at	7.022627	6.789289	7.496304	7.524553	6.352974	6.185565	3.626014	4.394617	3.581675	3.697778	3.570715	3.87083	3.108745
ZSWIM2	1554932_at	2.920149	2.866334	2.780972	2.838869	2.924233	2.828336	2.855315	2.868564	2.857149	2.843513	2.860142	2.886079	2.598301
PSIP1	1554933_at	3.467791	3.35249	3.128576	3.260515	3.488697	3.241409	3.543899	3.605054	3.622284	3.660665	3.531339	3.425792	3.470099
RCBTB1	1554934_at	3.140138	3.117672	2.958284	3.090487	3.123903	3.08811	3.101981	3.11392	3.098154	3.080544	3.096486	3.258384	3.026764
EXOC3L	1554937_x_at	1.929074	1.922933	1.905543	1.901646	1.920966	1.910574	1.921544	1.922106	1.9178	1.910175	2.127402	2.236388	1.875377
ACOT11	1554938_a_at	2.268671	2.253148	2.222264	2.235098	2.252873	2.400577	2.237517	2.250081	2.237194	2.21966	2.232686	2.244455	2.223177
LOC388882	1554940_a_at	2.20386	2.186812	2.158382	2.167182	2.188883	2.297451	2.179463	2.185711	2.17099	2.166489	2.168152	2.180393	2.099547
KLHL14	1554941_at	3.639772	3.489474	3.375817	3.488896	3.743814	3.44844	3.445284	3.43375	3.49172	3.437849	3.550632	3.595106	2.975304
KLHL14	1554942_a_at	2.51824	2.485547	2.432673	2.469137	2.474787	2.464761	2.478446	2.482696	2.479814	2.464983	2.464309	2.476379	2.459178
VIL1	1554943_at	3.07806	2.903055	2.113529	2.121733	4.544331	4.718035	2.574812	2.986848	2.897124	2.970225	2.967271	3.118019	2.708875
VIL1	1554945_x_at	2.898653	3.353423	2.842125	2.847401	4.392053	4.396023	2.858017	2.874867	2.868339	2.871194	2.866568	2.851707	2.805799
ACAT1	1554947_at	2.720234	2.670804	2.650438	2.652234	2.699076	2.625581	2.66472	2.678761	2.657417	2.649647	2.660355	2.684986	2.555926
LOC554174	1554949_at	2.33752	2.341535	2.284369	2.315347	2.358961	2.340025	2.315438	2.325161	2.313986	2.308575	2.310342	2.336076	2.196766
ACAN	1554950_at	2.088102	2.058525	2.023924	2.040372	2.06168	2.304326	2.05617	2.061853	2.048321	2.051039	2.05375	2.062877	1.985943
NLRP12	1554952_s_at	2.239267	2.198823	2.181739	2.180024	2.221766	2.193853	2.188096	2.209382	2.196706	2.191443	2.1992	2.205438	2.126893
C21orf90	1554953_a_at	2.189088	2.176391	2.143068	2.156692	2.186222	2.149285	2.163312	2.166915	2.160176	2.152677	2.155776	2.169568	2.07597
C21orf90	1554954_at	2.167136	2.150266	2.127161	2.133701	2.177475	2.474105	2.142043	2.148186	2.136072	2.128837	2.135689	2.141505	2.029719
ZNF641	1554958_at	1.962829	1.946706	1.928725	1.930274	1.961769	1.932684	1.942577	1.946158	1.93594	1.927671	1.928941	1.941	2.154733
C1orf110	1554960_at	2.4231	2.379177	2.33858	2.366624	2.397784	2.366346	2.370601	2.377912	2.367189	2.354301	2.3665	2.377202	2.284766
FGFR4	1554961_at	2.232276	2.204303	2.180462	2.186944	2.214696	2.186305	2.196979	2.202067	2.189147	2.182177	2.186057	2.214025	2.102267
FGFR4	1554962_a_at	2.302042	2.294401	2.250526	2.282382	2.303899	2.278973	2.294008	2.297568	2.28371	2.279399	2.275173	2.285909	2.292664
FILIP1L	1554965_at	2.418344	2.386184	2.342325	2.36625	2.401647	2.358554	2.375494	2.383866	2.371644	2.360222	2.37039	2.381368	2.247297
FILIP1L	1554966_a_at	2.719787	2.703184	6.613169	5.010932	2.526962	2.527614	2.679492	2.700758	2.530812	2.525941	2.521942	2.545195	7.157919
DIP2A	1554967_at	2.190937	2.176939	2.15445	2.158898	2.18212	2.160093	2.170703	2.161113	2.161365	2.153347	2.176134	2.172336	2.070188
DIP2A	1554969_x_at	2.290581	2.267325	2.23653	2.25469	2.277682	2.23649	2.26308	2.263138	2.252702	2.245862	2.248969	2.263818	2.161671
PDILT	1554970_at	2.27068	2.248936	2.210491	2.231901	2.252023	2.230029	2.232827	2.24457	2.232024	2.222191	2.230505	2.242079	2.13427
ZBTB26	1554973_a_at	2.640632	2.620917	2.545765	2.603982	2.623955	2.609586	2.756871	2.620476	2.614164	2.599156	2.603975	2.624429	3.071665
ACY3	1554974_at	2.994708	2.978034	3.290397	3.807024	3.380041	2.959376	2.970859	2.658537	2.705609	2.723712	2.958821	2.362067	2.625005
C8orf43	1554976_a_at	2.613013	2.530303	2.503497	2.511166	2.616545	2.507895	2.527931	2.53282	2.525675	2.517741	2.536843	2.575287	2.423383
LOC198437	1554977_at	1.981434	1.976847	1.957814	1.960097	1.966885	1.958187	1.970495	1.971958	1.961197	1.960426	1.954543	2.1318	1.960531

C8orf68	1554978_at	2.399702	2.374922	2.351775	2.356761	2.423146	2.353591	2.364937	2.375067	2.361715	2.352336	2.36118	2.372129	2.280183
ATF3	1554980_a_at	2.421495	2.405715	2.558931	2.389733	2.402233	2.414045	2.378015	2.405446	2.392512	2.385337	2.389024	2.398596	2.279653
C9orf11	1554981_at	2.984048	2.924452	2.834329	2.902326	3.004577	2.875722	2.914864	2.926647	2.911359	2.880187	3.028576	3.111921	2.635506
C9orf11	1554982_a_at	2.73705	2.67235	2.61754	2.661479	2.726298	2.63729	2.676432	2.680672	2.67236	2.654351	2.683873	2.722555	2.478735
C21orf117	1554983_at	2.270576	2.252793	2.218222	2.230737	2.262728	2.23163	2.241011	2.250501	2.236958	2.227786	2.234955	2.246309	2.139088
HLA-DOB	1554984_a_at	2.489077	2.480798	2.426103	2.461539	2.49621	2.454412	2.467126	2.480219	2.46539	2.45479	2.462693	2.474062	2.364574
ZNF396	1554985_at	3.736032	3.354032	3.299975	3.930615	3.463047	3.682428	3.182505	3.260412	3.294619	3.235851	3.301354	3.315268	2.891353
SNX19	1554986_a_at	2.231761	2.222823	2.189743	2.209497	2.230821	2.198101	2.221174	2.21694	2.21479	2.2026	2.202374	2.212972	2.218296
GOLGA3	1554987_at	2.363464	2.356536	2.310955	2.324394	2.35643	2.344668	2.340382	2.354941	2.34662	2.342035	2.337758	2.390117	2.29479
SLC9A11	1554988_at	3.100943	2.972919	2.917133	2.992392	3.117761	2.936202	2.978175	2.988235	2.990888	2.949164	2.999475	3.0345	2.774114
KIAA0317	1554989_at	2.206447	2.179589	2.158985	2.176145	2.221449	2.185553	2.181526	2.273043	2.262431	2.186027	2.180011	2.188888	2.091432
RASGRF1	1554992_at	2.671403	2.627824	2.600856	2.604685	2.640874	2.595592	2.617932	2.627007	2.610544	2.598893	2.60496	2.704431	2.436341
RAG1	1554994_at	2.662667	2.621834	2.555471	2.596937	2.654122	2.573798	2.609887	2.619324	2.606072	2.598029	2.604937	2.628078	2.430579
ZNF479	1554996_at	1.923182	1.898273	1.874999	1.881557	1.897871	1.881019	1.891567	1.895741	1.88306	1.877363	1.878804	1.894279	1.833175
PTGS2	1554997_a_at	2.478093	2.43659	2.395637	2.423896	2.533538	2.432049	2.442897	2.438703	2.430121	2.411238	2.42799	2.452278	2.381841
RASGEF1B	1554999_at	2.047973	2.032997	2.013381	2.113974	2.034159	2.015269	2.021181	2.031121	2.015509	2.008045	2.013066	2.025829	1.961241
IQCF1	1555001_at	2.24239	2.223586	2.196498	2.20449	2.234888	2.191666	2.212792	2.221743	2.208291	2.199186	2.203737	2.216973	2.106854
MGC39545	1555002_at	2.841978	2.758491	2.710604	2.749574	2.817754	2.726196	2.755674	2.766516	2.75721	2.738528	2.758433	2.778504	2.541165
RBL1	1555003_at	3.901851	3.74502	3.525334	3.718071	3.974159	3.724362	3.753551	3.755398	3.781672	3.722801	3.848168	3.935397	3.312123
RBL1	1555004_a_at	2.619581	2.599354	2.530239	2.56206	2.602891	2.540524	4.063617	2.70404	4.228269	3.654954	2.669252	3.226338	2.671798
WDR66	1555006_at	1.877494	1.867102	1.848444	1.853071	1.88752	1.852793	1.863716	1.864725	1.853341	1.848249	1.84654	1.858542	1.813475
WDR66	1555007_s_at	2.241186	2.218672	2.19893	2.517932	2.239347	2.411238	2.214293	2.218861	2.209438	2.197547	2.200364	2.214562	2.239412
SYNJ2	1555009_a_at	2.264008	2.24467	2.226667	2.231356	2.287171	2.229426	2.24117	2.242378	2.231587	2.226364	2.223891	2.287081	2.143581
ZFYVE16	1555011_at	2.758924	2.693265	2.700225	2.674978	2.764959	2.681505	2.711428	2.721551	2.707902	2.684645	2.704524	2.851682	2.55328
ZNF398	1555015_a_at	3.162349	3.071628	3.01327	3.110288	3.149974	4.090552	3.173629	3.097115	3.210546	3.058878	3.108737	3.147468	4.698099
IL16	1555016_at	2.12533	2.11045	2.086809	2.091354	2.109513	2.094661	2.108699	2.109306	2.094673	2.087367	2.089448	2.102302	2.034274
OR2C3	1555018_at	2.968837	2.880004	2.829603	2.848973	2.95759	2.85024	2.880319	2.890585	2.874434	2.843206	2.891997	2.927783	2.610854
PCDH21	1555019_at	2.594341	2.568647	2.548402	2.537447	2.567855	2.553695	2.555823	2.564709	2.551614	2.541792	2.551423	2.561164	2.385024
ARHGAP20	1555020_a_at	2.406034	2.631997	2.361689	2.377668	2.422498	2.379732	2.391696	2.398945	2.382391	2.388606	2.369009	2.388298	2.351124
SCARF1	1555021_a_at	2.45337	2.571906	2.532259	2.543941	2.593466	2.522734	2.566368	2.5021	2.531573	2.453731	2.457672	2.374169	2.545707
RGS12	1555022_at	3.583991	3.503943	3.434691	3.495704	3.683114	3.47469	3.533402	3.542798	3.530283	3.488034	3.548588	3.594136	3.352646
ADAM22	1555024_at	2.804859	2.740925	2.692171	2.721817	2.793707	2.710016	2.735201	2.738166	2.72907	2.708575	2.737471	2.766974	2.588767
TMEM26	1555025_at	2.564636	2.524825	2.502706	2.508825	2.547195	2.508161	2.513169	2.5213	2.510996	2.491093	2.512064	2.524225	2.357223
BRD3	1555028_at	2.357579	2.338544	2.296793	2.316378	2.350954	2.315344	2.327226	2.336404	2.32216	2.307372	2.321416	2.331673	2.244017
RGS12	1555032_at	2.117231	2.104216	2.081949	2.086424	2.104032	2.090213	2.097677	2.101293	2.090052	2.084633	2.090419	2.096086	2.043613
RGS12	1555033_a_at	2.134153	2.107247	2.08113	2.08753	2.130061	2.086085	2.095613	2.10668	2.091504	2.082964	2.084412	2.113321	2.048733
CLRN1	1555034_at	2.759191	2.702319	2.659821	2.664698	2.746858	2.671359	2.68124	2.689426	2.688096	2.631312	2.689558	2.698927	2.446492
CLRN1	1555035_a_at	2.21156	2.18302	2.160796	2.170402	2.202318	2.168531	2.186039	2.188768	2.173608	2.169393	2.177842	2.183317	2.089424

IDH1	1555037_a_at	11.48551	11.38294	12.38132	12.21141	11.53085	10.87588	12.14177	10.54757	11.99287	11.9858	10.49299	10.42597	10.80057
EPB41L4A	1555038_at	2.669792	2.607982	2.559926	2.597628	2.65133	2.60183	2.609467	2.618881	2.605468	2.58558	2.604161	2.634646	2.441166
ABCC4	1555039_a_at	2.373713	2.34994	2.336688	2.334286	2.364853	2.341061	2.375891	2.355413	2.658407	2.937778	2.335712	2.354245	2.287631
C12orf33	1555040_at	2.287541	2.260952	2.210309	2.236402	2.270028	2.233919	2.245531	2.255535	2.240576	2.230584	2.239922	2.250417	2.131576
NAGA	1555041_a_at	6.097281	6.065452	6.31168	6.615009	6.426392	6.211041	5.909329	5.825148	6.084011	6.077633	5.611479	6.058243	5.6082
TRPV5	1555042_at	2.531521	2.517468	2.491636	2.498236	2.518789	2.497915	2.506775	2.513398	2.499903	2.4816	2.496058	2.509491	2.427651
LHFPL5	1555043_at	2.438049	2.422883	2.400888	2.405045	2.422908	2.394288	2.416279	2.419949	2.407073	2.400343	2.401767	2.415509	2.305442
KBTBD5	1555044_a_at	2.74447	2.724673	2.704957	2.708369	2.72775	2.706793	2.712312	2.721482	2.708925	2.702348	2.753847	2.715911	4.253164
CENPI	1555046_at	2.538814	2.482264	2.455617	2.457109	2.526454	2.462334	2.47777	2.483308	2.469233	2.456719	2.557671	2.477813	2.388514
C21orf29	1555048_a_at	2.235559	2.209742	2.178398	2.19227	2.217942	2.190799	2.199174	2.211025	2.194735	2.186032	2.196718	2.204467	2.103038
C21orf29	1555049_at	2.448713	2.408196	2.366005	2.440045	2.434764	2.378322	2.400474	2.406752	2.396558	2.359879	2.395616	2.407443	2.234028
C10orf53	1555051_at	2.13598	2.122084	2.098982	2.103599	2.127619	2.101559	2.113568	2.121159	2.109185	2.105464	2.105636	2.117335	2.028916
SYT9	1555052_a_at	3.456882	3.580992	3.396791	3.550113	3.436687	3.404695	3.287027	3.417616	3.405917	3.396384	3.397766	3.406152	3.165206
SYT9	1555053_at	2.588892	2.55413	2.535315	2.529744	2.596724	2.535833	2.544868	2.550629	2.537293	2.536536	2.533355	2.553643	2.470572
KIAA0241	1555055_at	3.048398	2.932533	2.87837	2.943286	3.064268	2.898643	2.945031	2.947158	2.945626	2.900029	2.97659	2.998516	2.62184
CCNG2	1555056_at	2.389822	2.370122	2.34381	2.351495	2.373172	2.353244	2.359766	2.367658	2.35199	2.345827	2.354539	2.360385	2.279361
NDUFS4	1555057_at	4.581444	4.214637	4.102047	4.298439	4.45365	4.859079	3.091516	3.754718	3.275098	3.179844	3.87582	3.789976	3.473181
LPGAT1	1555058_a_at	9.203131	8.625919	7.895718	8.076673	7.636869	7.469368	7.327091	7.665347	6.955218	7.097527	7.388415	7.922841	4.99911
IKZF2	1555060_a_at	2.325512	2.309599	2.267496	2.291159	2.322632	2.294414	2.303944	2.303646	2.289013	2.27434	2.286344	2.295181	2.178453
GTPBP3	1555062_s_at	2.348507	2.338579	2.304347	2.323968	2.331732	2.325895	2.336653	2.337198	2.332002	2.324434	2.31947	2.344073	2.302463
USP6	1555063_at	3.567389	3.702406	3.395338	3.439716	3.580125	4.225572	3.291872	3.493629	3.160166	3.357081	3.471985	3.480704	3.020621
USP6	1555065_x_at	3.025845	3.355392	2.943426	3.003756	3.11448	3.961197	2.884585	3.014827	2.971398	2.774195	3.002368	3.013194	2.880278
WNK1	1555068_at	3.096771	3.091576	2.93933	2.973601	3.077943	3.035422	3.886719	4.114413	3.404503	3.952117	3.143415	4.103395	2.886574
TLL1	1555071_at	2.366398	2.334396	2.301631	2.316604	2.350173	2.317848	2.325847	2.336166	2.321333	2.308403	2.319918	2.330945	2.279084
MGC40069	1555073_at	2.649427	2.612707	2.55302	2.59628	2.624119	2.581071	2.608013	2.616261	2.607	2.586034	2.60568	2.626083	2.468906
KCNH5	1555074_a_at	2.388095	2.347782	2.314993	2.340713	2.371603	2.326319	2.344324	2.355924	2.342226	2.335798	2.336479	2.360311	2.560967
ARHGAP25	1555076_at	2.046676	2.03404	2.016852	2.01852	2.037414	2.008206	2.026216	2.034509	2.023574	2.017084	2.022882	2.032552	1.9551
MGC46336	1555078_at	2.033373	2.009218	1.98779	1.980327	2.017256	1.993571	1.991243	2.002877	1.987885	1.980149	1.984708	2.00991	1.937933
C9orf68	1555079_at	2.353498	2.323665	2.285066	2.301097	2.388427	2.284922	2.314819	2.321561	2.306128	2.288856	2.309633	2.335412	2.194453
NEK11	1555082_a_at	2.822258	2.796645	2.717357	2.814443	2.804592	2.775388	2.78397	2.795432	2.778508	2.749861	2.780324	2.790438	2.758613
MGC34774	1555083_at	3.137795	3.128259	3.060411	3.115841	3.127004	3.111306	3.264021	3.12754	3.119051	3.114459	3.113011	3.242759	3.082364
STAT5B	1555086_at	2.896185	2.901068	2.591003	2.773818	2.860701	2.767228	3.227691	2.881362	2.861214	3.122487	3.050711	3.140259	2.810911
STAT5B	1555088_x_at	4.421329	4.38121	4.314264	4.363316	4.386535	4.362362	4.375346	4.382068	4.365658	4.365203	4.367793	4.372966	4.179751
TMEM182	1555090_x_at	1.994729	1.987933	1.970669	1.972796	1.97956	1.969717	1.985189	1.980084	1.98091	1.974379	1.968499	1.972946	3.477349
PPM1F	1555091_at	2.848635	2.589187	2.568324	2.573613	2.615169	2.600187	2.588952	2.613	2.585991	2.591671	2.774301	2.686856	2.488927
VASH2	1555092_at	1.985003	1.963164	1.944751	1.946749	1.965064	1.9534	1.959782	1.960391	1.949083	1.943767	1.942425	1.958567	1.891193
C6orf123	1555095_at	6.313712	6.202318	5.273901	5.609493	5.18507	5.546212	3.225572	2.723658	3.257951	2.99809	3.050954	2.752749	2.924676
C4orf37	1555096_at	2.099527	2.081365	2.059247	2.063029	2.077119	2.066616	2.07548	2.077577	2.06504	2.059624	2.063626	2.07283	1.993457

PTGFR	1555097_a_at	2.538155	2.49691	2.438292	2.482823	2.553211	2.454623	2.491849	2.502674	2.487881	2.47106	2.48543	2.501566	5.520413
CACNB2	1555098_a_at	2.336564	2.318877	2.297861	2.308405	2.33452	2.298562	2.315968	2.317927	2.304829	2.297811	2.304305	2.30957	2.47359
MPP4	1555099_at	2.532713	2.490379	2.474392	2.48255	2.519531	2.453038	2.473743	2.491996	2.477079	2.464494	2.483069	2.495043	2.389999
C2orf13	1555100_at	2.377829	2.347165	2.297058	2.342506	2.371724	2.34168	2.338667	2.349938	2.335655	2.318977	2.336484	2.343348	2.215751
FGF7	1555102_at	3.409517	3.204547	3.114747	3.263049	3.407466	3.186834	3.220401	3.256897	3.265619	3.194367	3.325365	3.389577	2.833748
FGF7	1555103_s_at	2.916032	2.849784	2.793097	2.835282	2.91064	2.817861	2.843251	2.853091	2.840274	2.799978	2.837461	2.858052	2.699462
MIER1	1555105_a_at	3.195341	3.551331	3.70353	3.814279	3.720623	3.57981	4.13471	3.719741	3.708541	4.420786	3.670069	3.86552	3.984917
CTDSPL2	1555106_a_at	2.539563	2.488318	2.476765	2.481777	2.527413	2.477924	2.496394	2.498682	2.487462	2.473921	2.482303	2.496384	6.115183
KIAA1530	1555107_a_at	2.205225	2.181739	2.151356	2.165895	2.191198	2.16913	2.173499	2.177454	2.166165	2.162399	2.16486	2.178625	2.585829
SLC10A7	1555108_at	2.424433	2.398303	2.372747	2.372407	2.409389	2.37643	2.387406	2.393334	2.377903	2.374904	2.375	2.387144	2.302985
KLHL3	1555110_a_at	2.797334	2.777149	2.713323	2.749186	2.815737	2.757379	2.770023	2.775239	2.762434	2.742718	2.761584	2.787994	2.604367
C1orf114	1555111_at	3.620475	3.359491	3.312615	3.397599	3.723195	3.370951	3.374837	3.351568	3.438733	3.358562	3.57228	3.615181	2.934951
C1orf114	1555112_a_at	2.624939	2.615717	2.599196	2.58294	2.642361	2.607047	2.601213	2.610576	2.605318	2.576176	2.59657	2.618449	3.226676
PARD3B	1555113_at	2.159186	2.138018	2.111532	2.121007	2.141679	2.117749	2.128303	2.133781	2.122122	2.11472	2.122435	2.131671	2.046832
ATP6V0A2	1555114_at	2.746342	2.68894	2.61813	2.698351	2.726962	2.64197	2.708791	3.218397	2.86528	2.711828	2.894061	2.863876	2.393996
SLC11A1	1555116_s_at	2.592688	2.544129	2.49527	2.525996	2.588798	2.560312	2.534007	2.551076	2.530967	2.521647	2.535295	2.545763	2.408773
ENTPD3	1555118_at	2.476495	2.406613	2.328192	2.391445	2.469098	2.392148	2.391533	2.40273	2.391724	2.383762	2.390678	2.418539	2.185186
CD96	1555120_at	2.541867	2.508911	2.469933	2.489478	2.55991	2.499759	2.496607	2.508737	2.493558	2.483401	2.524386	2.518723	2.385229
GPR125	1555122_at	3.059789	2.994923	5.510054	4.63745	3.104388	2.950351	2.98026	2.983917	3.000207	2.959932	3.006976	3.022428	4.857803
ST6GAL2	1555123_at	2.49048	2.460219	2.432115	2.437122	2.459966	2.435543	2.443639	2.448142	2.430767	2.42043	2.448007	2.45074	3.923823
MGC40574	1555124_at	2.232854	2.283394	2.157425	2.177053	2.239771	2.229453	2.182457	2.188229	2.171504	2.157196	2.171271	2.183982	2.0621
C21orf66	1555125_at	2.164818	2.138174	2.163934	2.136099	2.147031	2.134634	2.250155	2.141888	2.146065	2.132127	2.122037	2.182329	4.172877
MOCS1	1555127_at	3.42229	3.394582	3.359584	3.375017	4.521735	3.62819	3.380991	3.39355	3.362229	3.34112	3.380812	3.389283	2.855408
PER3	1555130_at	2.894562	2.869118	2.774189	2.849355	2.940246	2.777602	2.843389	2.854554	2.853122	2.812213	2.869123	3.123223	2.64555
PER3	1555131_a_at	1.932135	1.910813	1.891064	1.893922	1.915385	1.899423	1.905076	1.907003	1.896263	1.890552	1.893832	1.905139	1.848257
FAM9A	1555133_at	3.012851	2.894082	2.824031	2.921788	3.025331	2.923299	6.630283	6.355989	4.537338	5.091822	5.168344	5.453553	2.632564
FGD6	1555136_at	2.710999	2.609965	2.507417	2.583979	2.816236	2.577297	2.589591	2.602312	2.591054	2.58368	2.604661	2.758483	2.440543
FGD6	1555137_a_at	2.746653	2.722741	2.682241	2.671278	2.731869	2.711119	2.712464	2.681992	2.699439	2.699828	2.70461	2.714559	4.454659
OTUD7B	1555139_a_at	2.401326	2.374523	2.244375	2.3521	2.381163	2.351979	2.36341	2.463141	2.356888	2.591773	2.363332	2.46998	2.240041
BCL2L2	1555140_a_at	2.197818	2.172117	2.144835	2.155023	2.213466	2.144066	2.175197	2.174652	2.165754	2.151267	2.161962	2.176923	2.340475
NHEDC1	1555141_a_at	2.958566	2.921552	2.876606	2.857863	3.047981	2.882879	2.912417	2.921823	2.891808	2.881675	2.907445	2.973863	2.80232
NHEDC1	1555142_at	2.30885	2.283868	2.247156	2.264037	2.294581	2.259031	2.277955	2.280741	2.268087	2.259164	2.269723	2.282449	2.163546
ARL17	1555144_at	2.149391	2.12681	2.104777	2.108538	2.130864	2.108351	2.117467	2.124171	2.111144	2.103885	2.109334	2.120485	2.034637
C1orf96	1555145_at	1.95249	1.929997	1.886938	1.926767	1.937538	1.926205	1.932697	1.939025	1.931217	1.91524	1.921907	1.941347	1.796367
ATF2	1555146_at	2.850686	2.79169	2.712878	2.774148	2.829989	2.733578	2.788405	2.78931	2.78288	2.769158	2.782143	2.823688	3.70682
LOC130951	1555148_a_at	2.403988	2.375752	2.334636	2.358141	2.447164	2.361107	2.371969	2.377705	2.353736	2.357471	2.361838	2.371578	2.060298
TDH	1555151_s_at	2.327738	2.311846	2.274132	2.283732	2.312347	2.281677	4.15351	3.962479	3.966271	4.118895	2.933579	4.079924	2.205403
FCHO2	1555153_s_at	4.485371	4.232887	4.186732	4.429972	5.848067	6.121535	4.371481	6.399693	4.12465	4.1273	5.734342	5.885308	2.534224

QKI	1555154_a_at	3.163955	3.075399	4.148955	3.078976	2.568325	2.510364	4.997025	3.079684	4.851143	4.966961	2.534585	2.82359	6.304776
MLLT6	1555155_at	2.062058	2.026396	2.030021	2.035221	2.04898	2.040398	2.050067	2.046181	2.03474	2.029386	2.081102	2.071263	1.98683
TMEM74	1555159_at	2.223109	2.194124	2.169271	2.17695	2.213128	2.176792	2.190084	2.200773	2.305163	2.176273	2.19286	2.194907	2.103914
LOC553158	1555162_at	1.973396	1.957188	1.934007	1.940395	1.957687	1.939735	1.953896	1.955382	1.942437	1.935335	1.941185	1.952035	1.886815
MGC35030	1555164_at	2.253908	2.251574	2.196655	2.209085	2.228517	2.207544	2.219651	2.225689	2.212345	2.210414	2.209257	2.220981	2.14671
PGPEP1	1555165_a_at	2.82341	2.718918	2.714478	2.690778	2.955424	2.674318	2.698369	2.718513	2.69114	2.705913	2.723437	2.752762	2.470032
ZNF396	1555166_a_at	2.762958	2.685817	2.634407	2.66663	2.840517	2.636526	2.675688	2.674444	2.668278	2.660293	2.67201	2.687694	2.615475
PBEF1	1555167_s_at	3.139449	3.18363	6.498642	5.596199	3.143681	3.134344	3.209903	3.006559	3.054485	3.139565	2.931732	3.043647	4.58989
CALN1	1555168_a_at	2.010306	1.997567	1.973402	1.981229	2.00342	2.566652	1.991685	1.995516	1.983396	1.976397	1.97598	1.993363	1.93231
LIG3	1555169_at	2.644085	2.615643	2.563851	2.595724	2.629579	2.585616	2.605561	2.615566	2.602291	2.584397	2.602897	2.6233	2.448962
ST3GAL3	1555171_at	2.754327	2.695924	2.630161	2.675592	2.738494	2.656394	2.695701	2.706578	2.689141	2.671275	2.692066	2.719233	2.499591
STX19	1555173_at	3.090896	2.983265	2.912135	2.962591	3.057903	2.951777	2.959774	2.979462	2.967769	2.949326	2.976985	3.079132	2.752179
PBLD	1555175_a_at	6.286273	5.924249	8.406491	7.561937	5.372525	5.27487	7.460386	4.196616	7.566182	7.251569	4.081238	4.639237	3.004559
PRKAA1	1555177_at	2.379766	2.347616	2.307584	2.329148	2.443661	2.33284	2.339891	2.34823	2.33555	2.321801	2.336481	2.404848	2.223111
IGHV7-81	1555179_at	2.358767	2.328344	2.295402	2.314052	2.344075	2.303896	2.317757	2.328842	2.315634	2.302041	2.308075	2.323385	2.200874
ST3GAL3	1555181_a_at	2.170961	2.161427	2.143615	2.145147	2.150113	2.140573	2.157757	2.141947	2.153001	2.140753	2.131924	2.134506	2.12092
TERF2	1555183_at	2.710879	2.675476	2.571949	2.64971	2.698825	2.651083	2.665209	2.674247	2.662035	2.655615	2.664477	2.675229	2.496468
TERF2	1555185_x_at	3.408751	3.378431	3.322607	3.292093	3.399317	3.364985	3.379492	3.310126	3.370763	3.345145	3.385027	3.373164	3.308649
CDKN1A	1555186_at	2.099261	2.081299	2.056958	2.062436	2.086784	2.068339	2.075818	2.081083	2.070909	2.066808	2.063367	2.074022	1.993224
hCG_20201	1555188_at	2.192019	2.175735	2.145206	2.155962	2.179393	2.159291	2.168063	2.174999	2.159491	2.152041	2.16074	2.175586	2.091869
TAT	1555189_a_at	2.23327	2.212295	2.184967	2.197623	2.215429	2.191503	2.202936	2.209131	2.193961	2.188784	2.195475	2.204928	2.105037
FHL5	1555191_a_at	2.319064	2.285143	2.259261	2.260444	2.291486	2.26705	2.271608	2.280442	2.269017	2.253524	2.266114	2.278783	2.184462
ZNF277P	1555192_at	5.236662	5.118644	4.344127	4.855471	5.295184	5.094635	3.928905	4.899844	4.886857	4.450523	4.980938	5.235377	3.468385
ZNF277P	1555193_a_at	5.758426	5.701711	6.945077	6.43879	5.050293	4.088434	5.991278	4.51689	5.92795	5.58159	4.363195	4.511982	6.467125
FBXO36	1555195_at	2.99828	2.884175	2.808034	2.88168	3.001362	2.881084	2.876622	2.884066	2.898258	2.84786	2.909145	2.979309	2.612892
C21orf58	1555197_a_at	2.154568	2.145363	2.129413	2.13314	2.143988	2.133634	2.140161	2.141231	2.131985	2.130436	2.128307	2.135385	2.090195
C21orf58	1555198_x_at	2.416813	2.407608	2.378367	2.387157	2.41073	2.398984	2.39828	2.40377	2.401727	2.394239	2.390551	2.414153	2.364534
GOSR1	1555199_at	3.24279	3.210042	3.135768	3.121073	3.231086	3.196149	3.221269	3.204785	3.198845	3.183841	3.189541	3.200042	3.159133
RMND1	1555201_a_at	3.647838	3.543973	4.588505	3.781565	3.363671	3.323903	5.043584	3.396876	5.149427	4.819314	3.369814	3.377483	4.597572
P15RS	1555202_a_at	3.255684	3.233201	3.202345	3.161454	3.228006	3.161091	3.233225	3.228603	3.21856	3.211321	3.219359	3.221741	5.512471
SLC44A4	1555203_s_at	2.338608	2.312714	2.292483	2.288722	2.322255	2.296231	2.296797	2.307645	2.296788	2.294081	2.290124	2.303257	2.179785
LOC439951	1555209_at	2.06381	2.045722	2.027795	2.029162	2.046683	2.045491	2.034957	2.042952	2.029038	2.021431	2.022763	2.036173	1.963135
DTWD1	1555210_at	2.830983	2.750569	2.690518	2.766621	3.176586	3.328538	2.757771	2.920093	2.753399	2.714998	2.810438	3.316366	2.569615
OR8B8	1555212_at	1.998932	1.985527	1.96308	1.967674	1.983681	1.96672	1.978829	1.982355	1.96749	1.966199	1.965506	1.974939	1.929834
CLEC7A	1555213_a_at	3.118342	3.113987	2.882279	3.01422	3.4767	3.251073	2.924634	3.023557	3.000148	2.995677	3.052833	3.205336	2.742321
CLEC7A	1555214_a_at	2.623495	2.585684	2.543317	2.567421	2.83022	2.55526	2.572689	2.587188	2.573586	2.559578	2.572427	2.609557	2.403141
HRH3	1555215_a_at	1.475393	1.469095	1.448525	1.452205	1.460647	1.452809	1.463644	1.459328	1.46037	1.45156	1.446858	1.457284	1.43683
LOC645722	1555216_a_at	2.310482	2.29822	2.27872	2.281828	2.964826	3.634424	2.282952	2.26864	2.280991	2.261865	2.540009	2.277633	3.691223

UBE2W	1555217_at	2.637368	2.588774	2.541558	2.575404	2.620449	2.566428	2.584599	2.59602	2.58111	2.564941	2.580391	2.596779	2.432028
RGR	1555219_at	2.128905	2.104464	2.067481	2.089996	2.111814	2.083852	2.095459	2.10275	2.088768	2.076424	2.088577	2.102575	1.989343
AKR1CL2	1555220_a_at	2.336361	2.306974	2.277452	2.287225	2.314068	2.278982	2.309039	2.308184	2.355267	2.416298	2.294261	2.301777	2.183421
LOC554201	1555224_at	2.230617	2.205163	2.174984	2.185114	2.21298	2.180574	2.193175	2.205301	2.190688	2.183089	2.190678	2.204215	2.103
C1orf43	1555225_at	3.560473	3.410692	3.588559	3.46351	3.161542	3.367792	4.828852	3.737087	4.693666	4.670159	4.597365	3.574551	3.72221
C1orf43	1555226_s_at	7.887179	7.975738	9.294955	8.369177	6.317268	4.377506	9.369019	6.04294	9.390059	9.396374	5.506771	5.557232	10.0452
MANEA	1555227_a_at	1.672261	1.662879	1.645934	1.650257	1.661438	1.650797	1.661575	1.662263	1.653885	1.649074	1.64484	1.654447	1.618481
C1S	1555229_a_at	7.5704	7.029113	10.37832	8.566835	4.242211	3.027948	4.466784	2.868691	4.441742	3.831001	2.744294	2.862547	4.823266
KCNIP2	1555230_a_at	2.161922	2.141013	2.117552	2.123352	2.145131	2.121862	2.13557	2.140687	2.127098	2.119776	2.124143	2.134803	2.073044
C21orf88	1555231_a_at	2.192543	2.177411	2.159612	2.164807	2.215137	2.161074	2.171821	2.181064	2.16796	2.160324	2.165617	2.177845	2.08278
RHOJ	1555233_at	1.889579	1.877684	1.860079	1.861255	1.876566	1.859685	1.872142	1.873856	1.865362	1.856724	1.861581	1.867733	6.249296
RHOJ	1555234_a_at	2.633202	2.614893	2.586756	2.588654	2.617749	2.593129	2.600322	2.606528	2.597176	2.576289	2.601136	2.617362	5.175745
IQCF3	1555235_s_at	2.403707	2.376722	2.35706	2.361145	2.881462	2.364563	2.368874	2.380694	2.362576	2.355992	2.359754	2.370105	2.282309
PGC	1555236_a_at	2.405789	2.392891	2.378896	2.380252	2.400783	2.383218	2.383508	2.394699	2.387593	2.367982	2.378183	2.385868	2.34763
TFIP11	1555238_at	5.35917	5.43431	5.305271	5.331929	5.369156	5.337785	5.351029	5.35296	5.34141	5.33555	5.335276	5.347875	5.197864
GNG12	1555240_s_at	3.406629	3.203852	3.260314	3.444144	3.286153	3.264081	3.252718	3.240395	3.231731	3.214411	3.265168	3.337541	3.436699
C8orf59	1555241_at	7.397735	7.244355	7.167871	7.304251	7.138155	6.912662	8.061804	8.153634	7.721138	8.010135	8.441341	8.132633	7.282002
C8orf59	1555243_x_at	7.666688	7.338488	7.39602	7.520515	7.593095	7.446013	8.022793	7.958285	7.754913	7.842147	8.344361	7.982654	7.703826
LOC554207	1555244_at	2.606835	2.555624	2.508367	2.546153	2.602128	2.523454	2.55749	2.567355	2.553543	2.532249	2.550494	2.578393	2.410422
RP1L1	1555245_s_at	2.021348	2.004022	1.980189	1.98615	2.004979	1.984782	1.996813	2.001388	1.988426	1.980859	2.002999	1.997702	1.927429
SCN1A	1555246_a_at	1.943125	1.926369	1.903193	1.908084	1.927673	1.906573	1.921716	1.925655	1.912325	1.905606	1.91259	1.919194	1.882251
RAPGEF6	1555247_a_at	3.089326	3.110624	3.154328	3.093289	3.094841	3.064819	3.686279	3.09503	4.25954	4.366158	3.080385	3.100401	3.811285
WNK3	1555248_a_at	2.755992	2.718903	2.673924	2.740808	2.745881	2.747516	2.711578	2.723337	2.707689	2.675955	2.704388	2.729637	2.903575
CPEB3	1555250_a_at	4.111943	4.304767	5.938225	5.495167	4.136189	3.659074	3.669767	3.687794	3.674659	3.816988	3.670319	4.062583	3.27221
OTOF	1555251_a_at	2.68192	2.661799	2.644362	2.648489	2.666228	2.612759	2.65871	2.663697	2.652804	2.643252	2.658968	2.800396	2.460371
TRPM3	1555252_a_at	2.3409	2.317852	2.29348	2.298414	2.354335	2.293396	2.306363	2.316215	2.29819	2.276775	2.297521	2.311687	2.315172
COL25A1	1555253_at	3.028823	2.995302	2.89825	2.978814	3.04772	2.991462	3.000562	3.004286	2.97672	2.964785	2.989384	3.005595	6.725253
HISPPD2A	1555255_a_at	2.074392	2.028171	2.079722	2.037141	2.05124	2.031472	2.047283	2.012148	2.04932	2.17227	2.038289	2.093228	1.999635
EVC2	1555256_at	2.237393	2.212448	2.180429	2.197034	2.220216	2.184859	2.20559	2.213656	2.199145	2.192759	2.197267	2.208647	2.20385
MYO3B	1555257_a_at	2.581085	2.548878	2.518157	2.531715	2.564682	2.519806	2.535274	2.547796	2.532223	2.526796	2.537546	2.544015	2.386022
ZAK	1555259_at	5.684987	5.996889	6.076904	6.345055	6.036582	6.269445	4.671071	5.399267	4.572428	4.6574	5.321441	4.823316	4.180884
MAGI1	1555262_a_at	3.05459	3.037035	3.021974	3.026337	3.173021	3.006235	3.031558	3.038286	3.026638	3.020957	3.019645	3.032654	2.890093
LOC646982	1555264_a_at	2.134127	2.118334	2.090549	2.100488	2.118194	2.098045	2.108696	2.115687	2.103312	2.095349	2.104726	2.113037	2.021779
ABCC13	1555265_at	2.718502	2.693331	2.677652	2.680491	2.72578	2.661008	2.695823	4.234513	3.082531	2.701218	2.684272	2.728642	2.535899
ASXL2	1555266_a_at	2.089313	2.067545	2.045123	2.044987	2.066286	2.049472	2.062044	2.063665	2.053696	2.044505	2.045002	2.063415	2.250501
GRID1	1555267_at	1.42991	1.424816	1.406979	1.408737	1.41594	1.412474	1.420836	1.417727	1.416755	1.407974	1.402346	1.410228	1.396524
GRID1	1555268_a_at	2.686945	2.671647	2.613461	2.654442	2.698458	2.659597	2.661607	2.674876	2.65949	2.645947	2.654771	2.669678	2.812261
TMEM16A	1555269_a_at	2.191624	2.186596	3.965385	2.243332	2.175887	2.165682	2.181356	2.172364	2.172073	2.1703	2.162581	2.167933	2.878959

WFS1	1555270_a_at	2.031377	2.014253	2.169339	2.130188	2.091705	2.069431	4.414874	3.343609	4.637445	4.567477	3.216616	2.516237	3.509225
TERT	1555271_a_at	1.988529	1.977353	1.953984	1.959673	1.975596	1.966175	1.974122	1.975197	1.961266	1.953017	1.95906	1.968067	1.912604
RSPH10B	1555272_at	4.339061	4.644779	4.148384	4.253111	4.553118	4.696678	4.452847	4.662992	4.756504	4.847039	4.396668	4.644708	4.385741
GALNT17	1555273_at	3.172983	3.145301	3.064519	3.112677	3.275377	3.12452	3.097579	3.138466	3.121937	3.110162	3.127945	3.152898	2.732929
SELI	1555274_a_at	6.237937	6.046882	6.597747	6.654295	6.530991	6.092402	5.903049	5.084047	5.963536	5.757816	5.454257	5.489858	5.077676
KLHL6	1555275_a_at	2.09005	2.067601	2.03635	2.048752	2.07086	2.048409	2.059386	2.065741	2.051392	2.047673	2.051881	2.060497	1.983492
SLC4A5	1555277_a_at	2.014261	2.000126	1.971749	1.983501	1.999952	1.983263	1.990818	2.000491	1.986218	1.978508	1.986152	1.99233	1.933566
CKAP5	1555278_a_at	4.697391	4.668028	5.006145	4.37618	4.396098	3.98981	8.381987	6.444142	8.585998	8.649309	5.406768	5.843703	9.61504
ARMC8	1555279_at	4.27972	4.08977	4.281874	4.420408	4.360382	4.057379	6.840417	6.306713	6.937444	7.15982	5.915868	6.476333	5.187967
ARMC8	1555281_x_at	3.764846	3.80603	4.317501	4.397928	4.03136	3.874778	6.956788	6.867772	7.092807	6.895539	5.727198	6.177699	5.218489
PPARGC1B	1555282_a_at	2.072519	2.040044	2.018075	2.029878	2.040388	2.030276	2.036823	2.058662	2.037564	2.018553	2.024505	2.034296	1.980911
ALS2	1555284_at	2.811571	2.658095	2.595142	2.650648	3.160699	2.646891	2.658313	2.675184	2.665922	2.637007	2.924701	3.896079	2.363584
CDKL1	1555286_at	2.674751	2.65919	2.641481	2.636467	2.66563	2.625659	2.657193	2.658835	2.648928	2.635439	2.646217	2.670463	2.479218
FBF1	1555288_s_at	4.065999	4.289714	4.049494	3.845087	4.051794	4.838429	4.058841	5.347913	4.146789	4.04664	5.143871	4.815085	4.017441
SEC16B	1555289_at	2.307481	2.293995	2.279307	2.284143	2.29793	2.274499	2.293876	2.295244	2.302643	2.281556	2.279258	2.29298	2.231362
TRPV3	1555291_at	2.489373	2.468971	2.457998	2.462813	2.511089	2.460218	2.465228	2.474135	2.463782	2.459822	2.454889	2.467973	2.352473
FAM40B	1555292_at	2.511015	2.49163	2.415459	2.469788	2.491204	2.466383	2.493487	2.489895	3.260991	2.81986	2.47659	2.531885	2.588476
ERC1	1555294_a_at	2.319344	2.299703	2.260386	2.293871	2.285401	2.272488	2.323813	2.28604	2.292503	2.427631	2.300787	2.29372	3.336865
VWA3B	1555298_a_at	2.401042	2.374177	2.345534	2.354344	2.384504	2.355434	2.363799	2.374494	2.35922	2.350227	2.359992	2.368465	2.250817
ERVWE1	1555299_s_at	2.785929	2.730443	2.671881	2.728395	2.80098	2.692699	2.737322	2.750163	2.730737	2.718448	2.734097	2.767219	2.577852
MED12L	1555300_a_at	2.098701	2.074365	2.054642	2.058883	2.084592	2.05954	2.070718	2.073938	2.063384	2.056188	2.062801	2.073136	1.995068
DIP2A	1555301_a_at	3.120127	2.964688	2.743099	2.944467	3.014043	2.936964	3.032912	3.022972	3.256873	3.097571	3.325436	3.843545	2.417753
KCNH5	1555304_a_at	2.330636	2.303843	2.277603	2.28585	2.313975	2.27349	2.291984	2.310284	2.28652	2.277176	2.289692	2.327033	2.191227
FOXJ2	1555305_at	3.091688	3.071086	2.999797	3.057085	3.067672	3.056318	3.033123	3.064454	3.051874	3.05458	3.049102	3.060728	2.789261
ECE2	1555306_a_at	2.082795	2.072179	2.057014	2.060173	2.077128	2.064583	2.072036	2.092751	2.062193	2.047992	2.057604	2.068679	2.011396
TTF2	1555307_at	2.811695	2.737667	2.676926	2.728124	2.82511	2.711957	2.744803	2.757108	2.744131	2.72154	2.754793	2.774198	2.523104
CARD14	1555308_at	2.844332	2.809211	2.70844	2.794085	2.857344	2.796197	2.777297	2.804035	2.78803	2.779514	2.788254	2.797461	2.78887
CARD14	1555309_a_at	2.419526	2.405413	2.359272	2.377762	2.423441	2.389696	2.391675	2.403603	2.387004	2.387226	2.385054	2.400162	2.245226
PAK6	1555310_a_at	2.367779	2.3614	2.342162	2.345948	2.361293	2.349975	2.358984	2.359502	2.351311	2.347325	2.344585	2.352624	2.313244
MCF2	1555313_a_at	3.014857	2.882828	2.809389	2.89672	2.929735	2.829526	2.904507	2.888137	2.902634	2.852042	2.914849	2.952041	2.923875
WDR19	1555314_at	2.963119	2.836103	2.763466	2.86288	2.980752	2.83845	2.848956	2.863635	2.854334	2.817868	2.860574	2.909681	2.594137
MAK	1555315_a_at	2.102822	2.084692	2.059805	2.068401	2.089878	2.072486	2.076844	2.083195	2.070011	2.061454	2.066752	2.077909	2.006526
KCNH7	1555316_a_at	2.450309	2.42175	2.375984	2.395272	2.451247	2.378835	2.395466	2.42031	2.405021	2.385636	2.403727	2.491502	2.320232
POLK	1555317_at	4.074147	3.483475	3.495669	3.478076	3.466919	3.051112	3.397963	3.480282	3.527743	3.433673	3.462433	3.490131	2.940412
HIF3A	1555318_at	3.5491	3.406604	3.24331	3.554663	3.639511	3.293882	7.407077	7.7301	7.40939	5.885026	8.363062	7.093814	8.749295
STAB1	1555319_at	3.481512	3.357551	3.421007	3.467101	3.501959	3.388578	3.479297	3.47013	3.474041	3.435421	3.492369	3.628914	3.416857
STAB1	1555320_a_at	2.503197	2.490407	2.469135	2.476912	2.490245	2.475451	2.47856	2.486502	2.47494	2.473904	2.471267	2.48124	2.330883
ACOT11	1555321_at	2.72833	2.93527	2.645146	2.656638	2.718294	2.663349	2.658479	2.686004	2.646236	2.67741	2.664411	2.657324	2.685158

ABCB9	1555323_at	4.379228	4.273974	4.100175	4.274318	4.341077	4.371479	4.196606	4.294896	4.239008	4.10763	4.308213	4.372601	3.369174
PTK7	1555324_at	2.428868	2.392207	2.378613	2.383262	2.466758	2.382661	2.389633	2.394819	2.384882	2.375125	2.393892	2.411951	2.285731
ZNF26	1555325_s_at	3.784034	4.447946	4.169624	4.069679	4.010842	3.554224	3.882766	3.575686	3.341707	3.694235	3.720755	3.616695	4.43383
ADAM9	1555326_a_at	2.159405	2.136904	2.127924	2.125335	2.153465	2.120953	2.567155	2.138626	3.210666	2.69475	2.131526	2.134597	5.625392
GCLC	1555330_at	2.184658	2.166479	2.155099	2.149737	2.178877	2.155147	3.631791	2.168901	4.358371	4.444839	2.153863	2.162253	2.140301
TPH2	1555332_at	2.253021	2.226763	2.193872	2.20808	2.243699	2.203039	2.216288	2.227526	2.212009	2.202607	2.213546	2.221371	2.118527
SLC30A5	1555334_s_at	3.151893	3.134623	4.520783	3.895	2.692478	2.673917	5.196352	2.689931	5.131934	4.667672	2.672293	2.974323	6.278489
ITGA9	1555335_at	2.786257	2.714418	2.700784	2.71341	2.938253	2.677268	2.723637	2.724668	2.715447	2.707672	2.717229	2.749041	2.508557
ITGA9	1555336_a_at	2.188665	2.16651	2.152522	2.148646	2.260882	2.154398	2.156896	2.164334	2.153196	2.146326	2.150179	2.157787	2.111962
ZNF317	1555337_a_at	2.041249	2.0257	2.003122	2.007616	2.025487	2.006124	2.019352	2.022756	2.017306	2.002103	2.004465	2.016655	3.119038
AQP10	1555338_s_at	2.181425	2.169761	2.150111	2.154434	2.168379	2.143434	2.164794	2.166497	2.155576	2.150704	2.263975	2.182278	2.102811
RAP1A	1555339_at	1.832407	1.823211	1.800261	1.806072	1.820731	1.804522	1.817578	1.822469	1.809377	1.802424	1.806635	1.818111	1.763168
RAP1A	1555340_x_at	2.150086	2.132901	2.113841	2.118458	2.132774	2.114021	2.124613	2.129125	2.114604	2.106225	2.111263	2.12282	2.022994
UNC5C	1555341_at	2.491925	2.453507	2.408061	2.438079	2.493667	2.440486	2.447735	2.462543	2.446732	2.517083	2.448346	2.745766	2.306128
UNC5C	1555342_a_at	2.133772	2.124769	2.100125	2.105142	2.129625	2.103249	2.115494	2.123772	2.108676	2.102825	2.105263	2.11755	2.035304
MEGF10	1555343_at	2.477447	2.469693	2.449239	2.453448	2.469234	2.4521	2.458204	2.468149	2.454515	2.447738	2.44697	2.459477	2.359503
SLC38A4	1555345_at	5.732945	5.615328	9.027197	7.589807	4.950419	4.091781	6.567005	2.606328	5.832495	5.771052	2.424189	3.085176	2.892569
CDC20B	1555346_at	2.902772	2.811736	2.777812	2.799186	2.875669	2.790327	2.805991	2.818541	2.8125	2.780705	2.819903	2.88052	2.599249
PDXDC1	1555347_at	2.843688	3.26645	3.067928	2.921635	2.809773	2.778105	3.124173	2.823515	2.777618	2.801409	2.74338	2.807933	3.256642
TFAP2E	1555348_at	2.231959	2.220945	2.191511	2.206223	2.243188	2.200229	2.214463	2.224314	2.209977	2.197638	2.207454	2.209337	2.174868
ITGB2	1555349_a_at	4.039751	3.972101	5.33277	4.936634	4.467172	4.186446	4.436305	3.985874	4.618745	3.96587	3.724094	3.891567	3.392071
PPHLN1	1555350_at	2.164514	2.136007	2.112185	2.122555	2.173338	2.120409	2.132354	2.135222	2.123787	2.11866	2.118582	2.295088	2.237919
PPHLN1	1555351_s_at	2.39743	2.363089	2.335967	2.348782	2.356996	2.337482	2.358731	2.377223	2.353621	2.395518	2.347939	2.359635	3.38118
FOXP2	1555352_at	2.767618	2.777441	2.513804	2.548492	2.680765	2.548956	2.551895	2.563666	3.509072	2.760162	2.553298	2.565287	4.374228
LRP1	1555353_at	3.075833	3.005474	3.001859	3.00579	3.135035	3.017231	3.015042	3.044408	3.028328	3.016679	3.175246	3.088799	2.702607
ETS1	1555355_a_at	2.733876	2.75599	3.451209	2.799247	2.847274	3.306228	2.700674	2.711258	2.759547	2.733513	2.763422	2.76966	3.924689
SCML4	1555356_a_at	2.874068	2.821498	2.724947	2.801519	2.873143	2.799125	2.795052	2.812908	2.80174	2.797009	2.806633	2.814466	2.558552
DDX53	1555357_at	3.415512	3.262889	3.181102	3.273012	3.454328	3.294071	3.273688	3.284172	3.29375	3.251388	3.331638	3.375726	2.85264
ENTPD4	1555358_a_at	3.592919	3.242291	3.585799	3.574476	3.669601	3.525158	3.562755	3.57154	3.770538	3.526976	3.532353	3.650984	3.771779
PIGQ	1555359_at	2.2481	2.229138	2.169548	2.215332	2.233113	2.238913	4.393425	2.766354	4.289551	4.875218	2.899429	2.947224	1.65979
DNAJC11	1555360_a_at	4.201458	4.12652	4.226406	4.230976	4.098773	4.160378	4.55994	4.648443	5.54257	4.570356	4.189876	4.769527	5.948951
MGC39821	1555363_s_at	6.394893	6.31474	5.448201	5.919238	6.509855	6.82556	4.290776	4.872296	4.32599	3.951143	5.480157	5.166442	4.399795
ZNF479	1555367_at	3.321155	3.221723	3.167251	3.183673	3.377133	3.185684	3.198686	3.243319	3.243883	3.198418	3.233948	3.304465	2.851301
ZNF479	1555368_x_at	2.776384	2.737696	2.683813	2.728591	2.809051	2.760829	2.750301	2.757393	2.746023	2.738481	2.740632	2.806031	2.529234
BAGE	1555369_at	2.964781	2.912396	2.822234	2.914404	2.951886	2.909532	2.920581	2.923104	2.910322	2.890453	2.915931	2.928099	2.784086
CAMTA1	1555370_a_at	2.875895	3.543665	4.050387	3.791735	4.081117	3.728781	6.784721	6.675416	5.824349	6.564106	6.775703	6.417525	8.240624
ABCB5	1555371_at	2.020351	2.004947	1.980005	1.986523	2.004693	1.986714	1.998083	2.002245	1.990323	1.982937	1.986383	2.008514	1.927844
BCL2L11	1555372_at	7.483284	7.626546	7.689597	7.934182	6.606682	6.457321	8.495085	8.240187	8.66552	9.014632	7.546204	8.151861	6.177845

C21orf114	1555373_at	4.352167	4.056067	4.008517	4.396824	4.314548	3.99819	4.094177	4.083229	4.136851	4.070804	4.05747	4.133844	3.778923
GRIK2	1555375_at	2.939549	2.849855	2.785378	2.840532	2.927408	2.81621	2.857065	2.863934	2.855021	2.825867	2.860103	2.956237	2.613757
OR4D2	1555377_at	2.073126	2.057654	2.039915	2.041964	2.053222	2.060314	2.054231	2.055631	2.04867	2.056983	2.035968	2.049071	1.987672
DBF4B	1555378_at	2.350395	2.320472	2.300827	2.323336	2.344021	2.55026	2.309141	2.318152	2.305928	2.293828	2.276432	2.311105	2.206437
MGC52498	1555379_at	2.562659	2.524975	2.470899	2.503084	2.57097	2.484725	2.515016	2.52935	2.514437	2.503337	2.517092	2.526228	2.361566
ADAMTS4	1555380_at	2.097812	2.060557	2.035257	2.04192	2.06762	2.044287	2.051501	2.059553	2.045298	2.043694	2.043054	2.051494	2.01423
POF1B	1555382_at	2.892294	2.782808	2.720314	2.781308	2.877349	2.780721	2.794749	2.796485	2.792792	2.759287	2.794429	2.825979	2.561644
POF1B	1555383_a_at	2.431954	2.403655	2.354355	2.384788	2.415574	2.366113	2.38707	2.401668	2.384299	2.368001	2.385548	2.396918	2.24906
LARP4	1555384_a_at	3.471935	3.597263	4.781565	4.527199	3.900527	3.43417	4.304441	2.606308	3.826908	3.934869	2.676401	3.515033	3.885997
B4GALNT1	1555385_at	2.35796	2.330143	2.305572	2.314758	2.353478	2.311519	2.319444	2.326774	2.316579	2.308001	2.315484	2.329369	2.200321
UBE1	1555387_at	2.019596	2.00297	1.986254	2.001886	2.002167	1.984306	1.998892	1.992712	1.987174	1.979748	1.980816	1.994079	1.933532
SNX25	1555388_s_at	3.881189	4.494805	4.200964	4.019606	3.884616	3.83866	3.926053	3.911192	3.937177	3.414609	3.845003	3.862596	4.278122
C14orf21	1555390_at	3.49354	3.485796	3.612707	3.848451	4.216436	3.786011	3.388713	3.137144	2.87164	2.986053	3.084158	3.427946	2.506881
ALS2CR8	1555391_a_at	3.647879	3.367691	3.354809	3.338179	3.664879	3.543676	3.343224	3.47064	3.401612	3.353334	3.492037	3.467044	2.990391
C21orf67	1555393_s_at	2.789692	2.782437	2.782621	2.769446	2.872668	2.771467	2.766574	2.775043	2.762772	2.751531	2.759897	2.772805	2.710161
PIGK	1555394_at	3.01113	2.892374	2.831833	2.89469	3.001198	2.888805	2.909072	2.934776	2.917897	2.880264	2.925359	2.960564	2.741158
AKAP12	1555395_at	2.327566	2.314255	2.266192	2.299297	2.327243	2.320888	2.302554	2.311695	2.301215	2.292251	2.29285	2.305792	2.189093
LOC340602	1555396_s_at	2.275367	2.251138	2.222653	2.232397	2.263716	2.231458	2.236803	2.248473	2.233245	2.220602	2.232014	2.243636	2.135604
MYO1D	1555397_at	2.620147	2.553387	2.511528	2.543555	2.616302	2.521026	2.55485	2.563782	2.547682	2.528011	2.567924	2.571326	2.38626
DUSP16	1555399_a_at	2.223255	2.216611	2.304905	2.204004	2.216391	2.199451	2.207165	2.211197	2.199608	2.190982	2.192917	2.209413	2.163763
LOC645261	1555400_at	2.964799	2.881701	2.839619	2.852572	3.084201	2.854088	2.873956	2.883824	2.870368	2.859117	2.873337	2.883492	2.607151
SOHLH2	1555401_at	1.888615	1.874098	1.853992	1.858924	1.889826	1.861904	1.870748	1.872929	1.861311	1.85566	1.857009	1.879178	1.83221
CDH19	1555403_a_at	3.207187	3.033073	3.059837	3.050753	3.130882	3.024793	3.054339	3.071774	3.138424	3.022756	3.060159	3.071539	3.317244
DUOXA1	1555404_a_at	2.552108	2.545978	2.526647	2.521609	2.534843	2.526031	2.542609	2.528426	2.529957	2.528872	2.520738	2.52116	2.540431
UBE3C	1555405_at	2.982023	2.952549	2.927853	2.93392	2.965699	2.923104	2.941948	2.9486	2.936239	2.917085	2.940371	2.948085	2.808536
FGD3	1555407_s_at	2.820682	2.767725	2.748726	2.764078	2.771987	2.764342	2.764372	2.775599	2.758307	2.765173	2.755014	2.949957	2.770339
BAGE2	1555408_at	1.70501	1.701247	1.677432	1.681253	1.691786	1.688182	1.692691	1.689958	1.684307	1.68222	1.680405	1.682641	1.661495
BAGE2	1555409_a_at	2.114307	2.100089	2.07457	2.079277	2.105684	2.07978	2.100409	2.100702	2.088668	2.090335	2.136267	2.095981	2.083538
CCNL1	1555411_a_at	7.950328	8.14245	8.489324	8.387339	7.644359	6.587253	8.075473	6.108606	8.116184	8.075724	6.016236	6.059624	8.191147
FBXL21	1555412_at	3.443823	3.227975	3.19088	3.399375	3.479843	3.361407	3.407665	3.241575	3.580599	3.372917	3.418286	3.413909	3.320273
FBXL21	1555413_s_at	2.750108	2.705199	2.6373	2.685078	2.771444	2.687599	2.690071	2.678646	2.687082	2.666335	2.682724	2.694866	2.515816
ALOX15B	1555416_a_at	2.280124	2.251972	2.217716	2.235516	2.260367	2.228469	2.240454	2.252647	2.249162	2.344612	2.230035	2.245852	2.139776
TAS1R1	1555417_a_at	2.255254	2.234024	2.193338	2.215678	2.288624	2.214857	2.224628	2.249037	2.220701	2.210067	2.225513	2.230433	2.184307
ASAH1	1555419_a_at	5.992648	5.533541	7.468357	6.942171	5.051532	4.941115	8.482934	6.170912	8.131442	8.255273	5.123326	5.655976	9.015716
KLF7	1555420_a_at	2.645146	2.61256	2.628045	2.542385	2.630332	2.635263	2.643195	2.616259	2.614671	2.57845	2.831286	2.622165	4.902097
AP1S3	1555421_at	2.688277	2.648167	2.577326	2.630191	2.765791	2.622834	2.637456	2.64577	2.634062	2.628497	2.645927	2.647318	2.532772
SSH2	1555423_at	3.192843	3.149685	3.095908	3.133105	3.279515	3.122968	3.144098	3.158513	3.141261	3.088298	3.137309	3.150129	2.726668
SSH2	1555425_x_at	2.293372	2.281123	2.258823	2.264675	2.278293	2.262914	2.274226	2.26813	2.252053	2.26238	2.258024	2.271572	2.18308

OTUD5	1555426_a_at	3.472865	3.477371	3.690665	3.464759	3.456037	3.433242	3.711291	3.475729	3.959691	4.488198	3.429872	3.467129	4.000705
SYNCRIP	1555427_s_at	3.588147	3.97429	4.314291	3.757711	3.188823	2.709782	5.974171	4.396182	5.850203	5.716074	4.760771	4.41045	6.014134
C9orf97	1555430_at	2.374733	2.342657	2.300519	2.319155	2.362527	2.316067	2.337854	2.343173	2.328733	2.317183	2.329749	2.338153	2.213321
IL31RA	1555431_a_at	2.303237	2.278837	2.241861	2.259844	2.289979	2.261094	2.266571	2.277336	2.263215	2.252218	2.259533	2.27117	2.174302
SLC39A14	1555433_at	2.215608	2.142224	2.122853	2.126916	2.149523	2.126427	2.13658	2.145206	2.12905	2.121649	2.131117	2.147539	2.07381
SLC39A14	1555434_a_at	2.263327	2.253168	3.110491	2.252861	2.227048	2.193006	3.110602	2.241961	2.895232	3.194252	2.204688	2.208887	2.786254
AFF4	1555435_at	3.786813	3.569467	3.445048	3.617492	4.140963	3.511936	3.490549	3.52256	3.666066	3.559227	3.864569	3.844406	3.189187
AFF4	1555436_a_at	3.502432	3.269277	3.343906	3.258348	3.271345	3.207103	3.265285	2.957298	3.16762	3.253537	3.252581	3.271536	3.166364
EARS2	1555437_at	3.497537	3.41078	3.314947	3.460385	3.514563	3.45893	3.471001	3.477339	3.292885	3.456073	3.461225	3.55081	3.298618
GTF3C3	1555439_at	3.764807	3.593719	3.54198	3.596579	4.414639	3.574205	4.18503	4.018869	3.603899	3.645085	4.389342	4.131514	3.688055
UBE1L2	1555441_at	2.854926	2.746461	2.760667	2.77306	2.875739	2.759607	2.792112	2.790321	2.786347	2.762994	2.788846	2.814109	2.743093
C10orf96	1555443_at	2.429246	2.39437	2.351982	2.37677	2.416801	2.390374	2.390172	2.396736	2.383483	2.368862	2.384075	2.39063	2.256196
PPP1R12B	1555444_a_at	3.276822	3.11236	3.063075	3.140402	3.299417	3.101188	3.148503	3.145312	3.164405	3.132491	3.216959	3.299957	2.828299
CA8	1555445_at	2.492846	2.452317	2.407131	2.433187	2.45817	2.420733	2.446651	2.457334	2.442628	2.427638	2.439868	2.453706	2.306005
TMEM1	1555446_s_at	4.556963	4.230273	5.130437	5.021499	5.181296	5.425075	4.971694	5.243403	4.28537	4.488823	5.434232	5.394444	3.349017
GPR114	1555447_at	2.175167	2.159706	2.138021	2.143853	2.159573	2.142379	2.154504	2.156415	2.1484	2.355486	2.144616	2.155323	2.064195
C14orf108	1555448_at	2.528535	2.497377	2.515278	2.500527	2.538228	2.476739	2.510711	2.503491	2.616842	2.550957	2.49751	2.515112	2.484055
NARG1L	1555450_a_at	3.210415	3.075559	3.178382	3.124989	3.106885	2.963008	4.367352	3.141632	3.359504	3.414713	3.22844	3.621603	4.36679
RALGPS1	1555452_at	3.009453	3.000647	2.88425	2.984227	3.068836	2.97911	2.989381	2.991459	2.978852	2.976399	3.15786	2.993429	2.905671
LITAF	1555454_at	2.866306	2.845411	2.786934	2.792578	2.851416	2.74652	2.785783	2.801136	2.785559	2.780665	2.78729	2.797962	2.570993
DBC1	1555457_at	2.765375	2.732288	2.658358	2.717224	2.734475	2.716303	2.722033	2.730803	2.718931	2.711763	2.717808	2.727508	2.532226
SLC39A6	1555460_a_at	2.096491	2.083001	2.375627	2.235014	2.084442	2.065349	2.106499	2.076679	2.619595	2.216539	2.063306	2.076318	6.803473
PPP1R1C	1555462_at	2.9769	2.544749	2.637318	2.532403	2.543831	2.536118	2.532035	2.540697	2.449852	2.411874	2.526651	2.531349	2.458171
CHD6	1555463_a_at	2.369193	2.352625	2.330052	2.336448	2.357645	2.335503	2.347822	2.349942	2.357622	2.335509	2.375332	2.34821	2.289934
IFIH1	1555464_at	2.385312	2.353086	2.300842	2.33522	2.353093	2.309215	2.347394	2.346823	2.351056	2.313358	2.332206	2.358997	2.18551
MCOLN2	1555465_at	2.097144	2.083303	2.058125	2.057034	2.080886	2.064705	2.080267	2.080983	2.069408	2.064847	2.065178	2.108703	2.027037
CUGBP1	1555467_a_at	3.135095	3.106668	3.979639	3.131159	3.155001	2.932173	3.478202	3.111188	3.486475	3.276817	2.961996	3.122096	4.787876
NRP2	1555468_at	2.679069	2.66691	2.646967	2.633294	2.717401	2.648851	2.656409	2.660621	2.621918	2.646828	2.643741	2.655362	2.559869
CLASP2	1555469_a_at	2.467251	2.47068	2.482981	2.445851	2.470397	2.448497	2.465496	2.458598	2.509686	2.466972	2.458045	2.459113	5.675468
PPM1F	1555470_a_at	3.067936	3.229437	3.165553	2.946621	3.032292	3.172007	3.106091	3.17837	3.323564	3.044764	3.158979	3.171624	3.005996
FMN2	1555471_a_at	2.726474	2.677275	2.619548	2.682917	2.709847	2.669861	2.681914	2.695807	2.684664	2.655949	2.682241	2.706968	3.038092
SORBS2	1555472_at	2.535169	2.492488	2.464076	2.482581	2.522771	2.527179	2.490839	2.499763	2.48643	2.469233	2.488591	2.499432	2.338138
TTLL3	1555474_at	3.050118	3.148537	3.03734	2.839559	3.10028	3.326326	2.956976	2.954811	2.973947	3.026393	2.937947	2.980985	2.753925
TTLL3	1555475_x_at	3.910439	3.833232	3.732072	3.853008	3.928293	3.884708	3.853178	3.894634	3.882074	3.883467	3.948956	4.012541	3.425256
IREB2	1555476_at	2.652784	2.569177	2.550962	2.552941	2.644007	2.557213	2.572574	2.572966	2.570919	2.540675	2.56457	2.581297	2.505342
C17orf82	1555478_at	2.339663	2.333153	2.314781	2.314147	2.32636	2.321531	2.331335	2.329179	2.326004	2.31552	2.311359	2.326477	2.291754
FBLIM1	1555480_a_at	4.949263	4.923487	4.869539	4.8721	5.415271	5.14646	4.945051	5.195136	4.876478	4.879305	4.768768	4.973981	2.873186
FBLIM1	1555483_x_at	2.752112	2.793777	2.774795	2.722533	2.791474	2.78776	2.786548	2.79193	2.784402	2.746163	2.775406	2.783918	2.624579

LOC202134	1555485_s_at	2.757636	2.696171	2.699312	2.731519	2.743871	2.671098	4.264904	4.526376	3.257015	4.029393	4.399736	5.51596	2.495918
FLJ14213	1555486_a_at	2.657342	2.652856	2.633808	2.627512	2.64521	2.846947	2.646665	2.647541	2.644395	2.874544	3.156895	2.642482	2.505101
ACTR3B	1555487_a_at	3.396213	3.399019	3.483373	3.463654	3.378768	3.347145	3.739371	3.456699	3.708809	3.455185	3.456903	3.637167	4.354834
PDZD3	1555490_s_at	1.925579	1.91399	1.89236	1.900555	1.921838	1.898472	1.912267	1.915903	1.911069	1.895257	1.900794	1.910515	1.850777
FLJ11286	1555491_a_at	5.757597	5.818125	5.340692	5.509355	5.894614	5.75576	4.520485	4.044069	4.326099	3.719302	4.496836	3.470052	4.040926
BEST3	1555492_a_at	2.385158	2.355897	2.328899	2.340662	2.367898	2.323064	2.345962	2.353689	2.344508	2.326322	2.341064	2.351028	2.29213
SDCCAG10	1555495_a_at	7.523105	7.568464	7.301671	7.862469	8.452387	8.637948	8.50781	9.166001	8.626598	8.609322	9.5321	9.279964	8.321525
CYP4B1	1555497_a_at	2.052016	2.036053	2.013707	2.01793	2.04455	2.02147	2.029417	2.032958	2.018884	2.012482	2.015908	2.027808	1.953136
IL28RA	1555499_a_at	1.825804	1.814749	1.794591	1.805808	1.814901	1.800357	1.811372	1.812546	1.802642	1.798503	1.792793	1.80597	1.76159
SLC2A4RG	1555500_s_at	5.775764	5.998086	6.343998	6.64289	5.705654	5.932657	5.078438	5.698245	5.059664	4.90566	5.396405	5.781397	3.074447
RSRC1	1555501_s_at	4.819981	4.253052	4.73555	4.808947	4.154261	4.14212	5.93238	6.304865	6.333727	6.731184	5.725597	6.032695	5.402879
NPSA	1555502_at	2.491088	2.460048	2.425211	2.436812	2.476384	2.441461	2.453225	2.457766	2.442788	2.437897	2.440527	2.457835	2.363889
TMCC2	1555503_a_at	2.169941	2.148054	2.093368	2.129259	2.14893	2.106758	5.481448	2.147835	5.830288	5.927925	2.133419	2.235896	2.117657
TYR	1555504_at	3.204737	3.086787	2.978274	3.062048	3.200413	3.05362	3.065261	3.066305	3.080678	3.037648	3.109829	3.167356	2.734786
TYR	1555505_a_at	2.464874	2.430349	2.414003	2.426161	2.448534	2.421249	2.432688	2.43818	2.427491	2.405468	2.427058	2.437291	2.378337
SLC25A41	1555509_a_at	2.406102	2.380131	2.312084	2.362047	2.387693	2.362569	2.363924	2.375925	2.360761	2.357343	2.446296	2.374936	2.25409
ZNF215	1555510_at	3.471905	3.368577	3.30649	3.366848	3.649493	3.218044	3.370188	3.383976	3.35036	3.30192	3.408172	3.47016	2.936195
PIAS2	1555513_at	2.660971	2.612356	2.554067	2.595464	2.65015	2.580456	2.606592	2.612959	2.602501	2.584163	2.601469	2.626446	2.429511
PIAS2	1555514_a_at	2.623349	2.57901	3.219311	2.576005	2.528569	2.546411	2.570547	2.590142	2.740667	2.706039	2.572344	2.490317	3.873785
C1orf2	1555515_a_at	2.425518	2.395601	2.350123	2.373568	2.403634	2.361752	2.38246	2.393653	2.38054	2.373175	2.381174	2.407483	2.363481
FOXP2	1555516_at	2.307095	2.266591	2.227617	2.242922	2.282311	2.253081	2.270022	2.259789	2.265895	2.247393	2.248434	2.257913	2.252778
GABRG3	1555517_at	2.652816	2.597295	2.543156	2.587938	2.644341	2.565898	2.598234	2.609589	2.595161	2.577727	2.597284	2.621103	2.448213
PTCH1	1555520_at	2.328202	2.31255	2.305407	2.29536	2.310864	2.292164	2.37804	2.305217	2.295382	2.289673	2.288101	2.301666	4.004318
MEMO1	1555522_s_at	5.707472	5.43169	6.167036	5.825636	4.732886	3.818329	6.324839	5.051686	6.557465	6.323735	4.910536	5.063151	6.988963
FYCO1	1555523_a_at	1.775546	1.769286	1.74694	1.752755	1.766693	1.758545	1.764483	1.762295	1.762355	1.748614	1.745187	1.754003	1.743758
SEPT6	1555526_a_at	4.296911	4.236067	4.209431	4.278763	4.043835	4.18576	4.752981	4.234343	4.273112	4.549707	4.218816	4.254829	7.603097
COL9A1	1555527_at	2.248255	2.226024	2.202249	2.208338	2.233923	2.206966	2.217557	2.224519	2.210236	2.204373	2.209723	2.222075	2.117313
RNH1	1555529_at	2.081174	2.059188	2.033216	2.042141	2.064779	2.316737	2.044195	2.054897	2.036555	2.025	2.039611	2.044239	1.980421
GPR103	1555533_at	2.426191	2.390352	2.349053	2.373232	2.410349	2.353729	2.38144	2.393389	2.37827	2.360634	2.379275	2.388301	2.267819
BPIL3	1555535_at	2.238755	2.21655	2.191054	2.195791	2.220171	2.194939	2.202625	2.212359	2.198296	2.192607	2.199094	2.207282	2.107387
ANTXR2	1555536_at	2.716448	2.663922	2.614442	2.619145	2.705498	2.827584	2.676683	2.66268	2.663633	2.642478	2.661303	2.681353	2.724384
FAM9B	1555538_s_at	2.896764	2.776903	5.977385	6.810147	6.691265	5.89579	2.741325	2.741852	2.73835	2.718298	2.779518	2.773474	2.490954
SDS	1555539_at	6.800049	7.163958	4.740023	3.864213	7.062904	6.995226	2.689124	2.681301	2.678726	2.672428	2.672738	2.685904	2.627239
TGFB3	1555540_at	2.199211	2.172286	2.150773	2.153404	2.176907	2.151135	2.167543	2.169231	2.156524	2.148616	2.155301	2.165475	2.103598
AFAP1L1	1555542_at	2.544071	2.356903	2.302909	2.20405	2.334115	2.988605	2.196693	2.33153	2.314252	2.302592	2.314652	2.665821	2.254993
CLCC1	1555543_a_at	4.586296	4.126253	4.487552	4.587162	3.505604	3.487341	5.392093	3.998159	5.581934	5.769937	4.372664	3.989892	5.360244
CADM2	1555544_a_at	2.739938	2.695553	2.630955	2.669826	2.720657	2.686869	2.682146	2.689414	2.676064	2.653675	2.675248	2.712314	2.703049
KLK2	1555545_at	2.202484	2.189246	2.164683	2.168906	2.186681	2.231663	2.179135	2.299296	2.170987	2.15979	2.173973	2.178769	2.06045

POFUT2	1555546_a_at	2.289732	2.277111	2.219143	2.261666	2.280375	2.272284	2.275376	2.278976	2.264989	2.26693	2.266211	2.277378	2.10783
NDUFA10	1555548_at	3.483381	3.433642	3.222132	3.39788	3.590145	3.422264	3.432589	3.43254	3.426577	3.48751	3.445833	3.52265	3.384815
LGICZ1	1555550_at	1.932214	1.941171	1.902925	1.90627	1.91412	1.91138	1.923021	1.922106	1.952362	1.912337	1.904488	1.919757	1.858594
SERPINB5	1555551_at	2.879079	2.823251	2.719282	2.78309	2.866722	2.772786	2.795868	2.819591	2.806688	2.779698	2.805274	2.838997	2.592948
SLC22A7	1555553_a_at	7.522388	7.070338	7.817881	7.477386	5.113703	4.943822	4.841565	4.490717	4.81303	4.599472	4.199062	4.384917	4.140769
RP11-49G1(	1555554_at	2.357493	2.361298	2.304027	2.311346	2.333737	2.303474	2.31016	2.328107	2.345479	2.307069	2.315031	2.322106	2.240608
BBS9	1555555_at	2.438087	2.378095	2.336453	2.377519	2.414406	2.348196	2.36258	2.373021	2.3596	2.342842	2.364275	2.424178	2.242195
TNK2	1555557_a_at	2.222235	2.209083	2.174471	2.189016	2.224091	2.187993	2.195197	2.206982	2.191607	2.182475	2.184597	2.197967	2.114111
USP25	1555558_at	3.205395	3.065762	2.987223	3.064659	3.239472	3.008815	3.070434	3.077524	3.088075	3.040636	3.111838	3.20761	2.738341
USP25	1555559_s_at	2.626737	2.592103	2.514588	2.574302	2.619321	2.561071	2.582723	2.595113	2.555799	2.566518	2.586724	2.590933	2.836579
UGCGL2	1555560_at	2.772485	2.708703	2.654152	2.714949	2.761562	2.666231	2.713566	2.72733	2.712851	2.688054	2.714059	2.738943	2.546271
UGCGL2	1555561_a_at	3.075551	3.056348	3.243109	3.060867	3.173478	2.942371	3.054904	3.052517	3.584431	3.501564	2.998314	3.042682	3.972898
ZCCHC7	1555562_a_at	4.081194	3.899072	3.754155	3.947878	4.182276	3.781575	4.352657	4.009376	3.996959	4.217901	3.992946	4.01604	5.437071
CFI	1555564_a_at	9.488168	9.563538	11.25035	10.47337	8.743318	6.939348	8.395523	5.876026	8.68151	8.482595	5.054907	3.891176	5.879116
TAPBP	1555565_s_at	3.110558	2.888761	2.868031	2.833576	2.905268	2.951235	2.87815	2.823639	2.811218	2.82652	2.806827	2.844392	2.755032
LMOD3	1555567_s_at	2.970939	2.916295	2.756019	2.892924	3.003232	2.887118	2.902604	2.909469	2.903002	2.897893	3.162038	3.099738	2.752519
MGC22265	1555568_at	3.030496	3.048788	2.998766	3.010214	3.071923	3.005446	3.220844	3.13636	3.02679	3.011752	2.928076	3.046771	3.033492
KCTD7	1555569_a_at	2.231158	2.207899	2.174894	2.18654	2.214593	2.188834	2.202627	2.207195	2.192776	2.185704	2.192338	2.200521	2.123389
IMMP2L	1555571_at	2.85501	2.841015	3.542758	4.319707	3.179502	2.805981	2.827386	2.812576	2.824227	2.802856	2.823823	2.823875	2.702671
CA6	1555572_at	2.348347	2.32097	2.28534	2.296798	2.372897	2.300955	2.317388	2.31888	2.30379	2.29648	2.304354	2.380523	2.196468
C10orf93	1555573_at	2.871638	2.834082	2.752527	2.812791	2.859589	2.808185	2.815935	2.823504	2.83547	2.785276	2.899677	2.917749	2.720199
KDELR1	1555575_a_at	9.927641	10.06391	9.453793	9.679693	9.820184	9.961715	9.719485	10.01144	9.568795	9.553191	10.14016	10.23402	9.969903
PTPRM	1555578_at	3.203547	3.236415	3.065443	3.32143	3.395814	3.720705	3.059014	3.27562	3.089161	2.979677	3.866705	3.147369	3.166457
PTPRM	1555579_s_at	7.173718	6.90511	7.187731	7.015479	6.786634	6.657272	6.603078	6.53659	6.642904	6.428511	6.765544	6.076979	8.117998
TP63	1555581_a_at	2.446179	2.416008	2.367211	2.395852	2.428044	2.396769	2.4042	2.415949	2.401267	2.38947	2.39882	2.411035	2.319273
FAM71B	1555585_a_at	2.277811	2.254275	2.219195	2.240026	2.260683	2.238544	2.252322	2.255226	2.242521	2.240243	2.241178	2.271745	2.173754
FAM71B	1555586_at	2.298555	2.271171	2.246823	2.255157	2.277446	2.252948	2.264002	2.27067	2.257874	2.244042	2.256477	2.267418	2.159517
PDZRN3	1555587_at	2.959562	2.935136	2.863866	2.902723	2.940002	2.918827	2.921567	2.931031	2.927064	2.98109	3.042519	3.129595	2.829022
GATA1	1555590_a_at	2.173674	2.169401	2.148474	2.152114	2.167038	2.160397	6.943745	4.266744	6.927833	6.99723	3.213994	5.333141	2.026865
PIF1	1555591_at	2.861826	2.783731	2.739612	2.774864	2.850527	2.75243	2.796668	2.807567	2.793918	2.767936	2.792267	2.824899	2.566589
MBNL1	1555594_a_at	2.746962	2.712739	2.660547	2.698726	2.744308	2.67577	2.719097	2.719897	2.739474	2.691656	2.727837	2.735382	3.590292
SCRN3	1555595_at	3.487945	3.412912	3.41331	3.424836	3.597919	3.402531	3.428614	3.303365	3.426521	3.413735	3.402082	3.514734	3.454575
DUSP19	1555598_a_at	2.53113	2.488595	2.457728	2.499673	2.507366	2.488339	2.503198	2.502449	2.490358	2.484595	2.488792	2.495138	2.475589
APOL4	1555600_s_at	2.132939	2.113685	2.088539	2.098434	2.115995	2.093769	2.243966	2.112816	2.353384	2.09861	2.097218	2.107168	2.715916
ELAVL3	1555602_a_at	1.955212	1.933239	1.912013	1.916783	1.932922	1.914971	1.927891	1.931238	1.918226	1.911001	1.917147	1.924843	3.531452
BAGE	1555603_at	2.461955	2.362868	2.298852	2.347107	2.375124	2.341159	2.338161	2.348637	2.350237	2.308274	2.353401	2.40792	2.205753
BAGE	1555605_x_at	2.639162	2.607354	2.46154	2.583061	2.615859	2.840172	2.592878	2.602321	2.591919	2.625241	2.613167	2.620159	2.418231
GDPD1	1555606_a_at	2.104243	2.086264	2.058973	2.067205	2.087092	2.078072	2.076027	2.083807	2.069926	2.07191	2.070625	2.078459	2.066418

CAPRIN2	1555608_at	2.934729	2.919214	2.851794	3.167657	2.917961	2.849737	2.914881	2.91627	2.904088	2.898911	2.896004	2.909161	2.858152
ZMAT3	1555609_a_at	2.153351	2.133137	2.10498	2.113461	2.134353	2.111426	2.126066	2.13001	2.116512	2.11073	2.114948	2.126082	2.042708
AGK	1555610_at	2.735679	2.683836	2.632316	2.664901	2.724856	2.636138	2.673049	2.684997	2.67165	2.652004	2.67053	2.701578	2.489828
MBD1	1555611_s_at	3.185484	3.15176	3.141305	3.112584	3.147437	3.111118	3.132895	3.181302	3.198054	3.129481	3.127157	3.14327	4.743524
G6PC	1555612_s_at	6.281525	6.359643	9.600672	8.535167	5.421952	3.076474	2.822935	2.813506	3.009312	2.801579	2.805497	2.817496	2.466508
ZAP70	1555613_a_at	1.838608	1.830553	1.813025	1.814118	1.827603	1.813315	1.824078	1.824947	1.815921	1.811642	1.806296	1.806082	1.828612
SAE1	1555618_s_at	4.297333	4.254388	4.82746	4.082862	3.675625	3.228218	7.676439	4.577482	7.721214	8.185445	4.622501	4.419238	9.675965
PTGIR	1555620_a_at	1.937187	1.929393	1.898961	1.914127	1.919125	1.960676	1.924291	1.926165	1.913529	1.913592	1.907934	1.914311	1.913963
SSH1	1555624_a_at	2.821461	2.797158	2.776116	3.028493	2.79537	2.638821	2.748742	2.790574	2.745768	2.727209	2.772805	2.779938	2.574711
SLAMF1	1555626_a_at	2.574108	2.514139	2.479766	2.524596	2.562181	2.523099	2.5344	2.542168	2.523335	2.509937	2.598093	2.554438	2.374765
C21orf67	1555627_s_at	2.617611	2.594865	2.591072	2.59447	2.62865	2.592347	2.608141	2.933296	2.596789	2.589215	2.590049	2.588431	2.42138
HAVCR2	1555628_a_at	1.864501	1.835496	1.818688	1.820201	1.83524	1.824063	1.830686	1.835584	1.82208	1.816619	1.818481	1.831615	1.776212
HAVCR2	1555629_at	2.388286	2.361074	2.343348	2.343772	2.368093	2.345836	2.352084	2.35498	2.345067	2.327853	2.34313	2.358655	2.1991
RAB34	1555630_a_at	4.998266	4.989927	4.64561	4.374979	4.851752	4.800067	7.327469	7.060269	7.285087	7.319825	7.975426	7.555723	9.188908
PIK3IP1	1555632_at	2.017932	1.993609	1.970602	1.975385	1.987584	1.976248	1.986557	1.989157	1.980151	1.975183	2.01178	1.985756	1.918103
LILRA5	1555634_a_at	3.467767	3.458088	3.423093	3.434875	3.454513	3.437748	3.454303	3.456888	3.442607	3.435189	3.440363	3.842444	3.036883
CD300LG	1555636_at	2.824638	2.767421	2.72167	2.737309	2.789624	3.07655	2.753403	2.827886	2.75647	2.590286	2.770764	2.77316	2.528539
SAMSN1	1555638_a_at	3.406673	3.488518	4.439419	3.796684	3.394928	3.259057	4.329756	3.746875	4.317068	4.566599	3.811749	3.832985	3.271624
RBM14	1555639_a_at	2.757348	2.726474	2.724533	2.720627	2.741424	2.728395	2.738987	2.739861	2.738014	2.732562	2.722069	2.734511	4.546682
DAOA	1555640_at	2.684901	2.6255	2.572856	2.615711	2.672548	2.560092	2.62562	2.64862	2.625685	2.604148	2.622702	2.646053	2.458243
LILRA5	1555643_s_at	2.484321	2.439234	2.449379	2.417879	2.469295	2.429103	2.433109	2.437914	2.430291	2.411715	2.42333	2.434184	2.282334
FOXP2	1555647_a_at	2.19239	2.165311	2.140081	2.153212	2.166897	2.147407	2.162697	2.164146	2.157885	2.149928	2.148622	2.163582	2.35998
FOXP2	1555648_at	3.051527	2.933784	2.861928	2.940194	3.041188	2.931366	2.94165	2.961056	2.946881	2.910612	2.965348	3.030802	2.654309
KLHL17	1555650_at	1.855252	1.843265	1.825064	1.83078	1.844179	1.829199	1.840578	1.841771	1.832092	1.827315	1.832854	1.83507	1.789608
OR10A5	1555651_at	1.916235	1.903844	1.883391	1.887239	1.902829	1.892034	1.900181	1.902469	1.888555	1.882526	1.886797	1.894922	1.838412
HNRPA3	1555653_at	14.25327	14.20814	14.27392	14.32177	13.95816	13.89361	14.37549	13.75198	14.25039	14.15249	13.7344	13.84541	13.66581
OR10A4	1555655_at	1.959621	1.944917	1.92395	1.927472	1.944705	1.926939	1.939138	1.940397	1.929248	1.929361	1.9236	1.93403	1.881871
CD300LG	1555656_at	2.261973	2.255702	2.231393	2.218182	2.247484	2.233663	2.253753	2.235452	2.425533	2.232111	2.227282	2.229982	2.233222
TREML1	1555659_a_at	2.047722	2.106128	2.022735	2.019301	2.028411	2.01232	2.433391	2.03825	2.05534	2.422735	2.013395	2.110783	1.962484
OR8D1	1555661_at	1.90447	1.893508	1.871408	1.876477	1.892404	1.875716	1.887372	1.892944	1.878435	1.87106	1.874308	1.883923	1.827451
DAOA	1555662_s_at	2.165625	2.144585	2.12419	2.127665	2.14905	2.133113	2.134946	2.141988	2.129598	2.13062	2.127555	2.139695	2.050006
PTPRS	1555666_at	2.990393	2.915329	2.838777	2.912764	3.104887	2.877773	2.922252	2.930472	2.921878	2.888357	2.941314	3.008566	2.790137
PARK2	1555668_a_at	2.113355	2.095317	2.078632	2.081361	2.103509	2.070259	2.08833	2.094562	2.081702	2.07194	2.078279	2.08999	2.00837
ICA1L	1555671_at	2.686289	2.637139	2.570165	2.615161	2.677214	2.621135	2.628168	2.61896	2.623818	2.602631	2.638095	2.64543	2.442289
LOC728285	1555673_at	2.533902	2.512395	2.484192	2.492928	2.515098	2.489825	2.501359	2.510081	2.495569	2.486957	2.489952	2.504062	2.313843
BRCA2	1555675_at	2.359733	2.333055	2.310982	2.309897	2.344223	2.315514	2.317525	2.328702	2.313518	2.30055	2.312602	2.324579	2.302623
GSX1	1555676_s_at	2.041785	2.024977	2.000934	2.009245	2.025249	2.00627	2.017119	2.022378	2.009174	2.000551	2.005644	2.014712	1.945614
SMC1A	1555677_s_at	2.923471	2.860979	2.865228	2.852527	2.935987	2.874551	2.885538	2.875714	2.877869	2.86679	2.874248	2.918684	2.819184

ST3GAL3	1555678_at	2.587277	2.551816	2.47608	2.519465	2.56039	2.479613	2.544852	2.548702	2.535956	2.528346	2.532722	2.553194	2.346367
RTN4IP1	1555679_a_at	3.486575	3.359134	3.491314	3.417721	3.26288	3.244859	4.102001	3.721453	4.884453	4.801417	3.423017	3.439142	4.249663
SMOX	1555680_a_at	2.31583	2.297417	2.270005	2.279913	2.298765	2.290345	2.301876	2.299051	2.281972	2.373129	2.287255	2.291738	2.401701
OTUB2	1555682_at	1.995739	1.988055	1.967941	1.974172	1.986479	1.972142	1.983913	1.986315	1.9735	1.971375	1.967216	1.978165	1.913167
H6PD	1555685_at	2.974048	3.181812	2.839116	2.848712	3.461095	2.868079	2.680622	2.868755	2.854837	2.821783	2.851655	2.861734	2.594969
CLEC4C	1555687_a_at	2.8611	2.794874	2.741364	2.781624	2.850141	2.771607	2.802499	2.806122	2.798204	2.776489	2.798745	2.825959	2.606615
SLA2	1555688_s_at	1.998407	1.983549	1.960731	1.964747	1.982573	1.969642	1.983263	2.040283	1.971269	1.964278	1.962957	1.975187	1.939035
CD80	1555689_at	2.986419	2.936649	2.86911	2.915221	3.038957	2.842151	2.927984	2.913276	2.923602	2.87798	2.931219	2.951273	2.627223
KLRC4	1555691_a_at	2.287833	2.259941	2.233565	2.247777	2.268542	2.239383	2.252083	2.258655	2.24673	2.237952	2.243113	2.252306	2.15853
KCNIP3	1555694_a_at	1.987916	1.981059	1.958023	1.962429	1.980238	1.943776	1.980518	1.978062	1.976924	1.965548	2.035004	2.157205	1.982648
CLRN1	1555695_a_at	2.373697	2.347902	2.348799	2.351188	2.371916	2.348638	2.359608	2.349318	2.353122	2.350421	2.353231	2.358411	2.029774
KLK4	1555697_at	2.050731	2.046151	2.022888	2.024131	2.036989	2.025548	2.039715	2.037063	2.026592	2.023945	2.025003	2.024343	1.991515
ST3GAL3	1555702_a_at	2.129111	2.108458	2.161826	2.092536	2.108072	2.091897	2.10466	2.106096	2.102983	2.151763	2.097037	2.099368	2.751109
ST3GAL3	1555703_at	2.672886	2.620232	2.607592	2.611694	2.633895	2.567525	2.617758	2.605208	2.604958	2.582601	2.60441	2.616746	2.445531
CMTM3	1555704_at	2.963989	2.931692	2.920357	2.931859	2.943752	2.906673	2.944732	2.943775	2.929569	2.934276	2.927972	2.940861	2.774129
CMTM3	1555705_a_at	3.267999	3.237778	3.384501	3.432097	3.239086	2.741923	6.780641	5.445968	6.274932	6.759082	5.531608	5.53292	7.028669
TNAP	1555707_at	2.951921	2.852393	2.797971	2.848537	3.131567	2.84659	2.860406	2.855911	2.859829	2.829334	2.871634	2.895466	2.873263
OFCC1	1555708_a_at	2.674757	2.63028	2.591342	2.608327	2.664495	2.602022	2.619009	2.625524	2.616752	2.596898	2.616574	2.640652	2.439031
CPVL	1555710_at	3.272013	3.138514	3.109669	3.142985	3.29571	3.084164	3.162797	3.169084	3.163783	3.123193	3.184162	3.25121	2.783526
CPVL	1555711_x_at	3.072096	3.007931	2.952824	2.999826	3.098702	2.97298	2.992619	3.021216	2.997627	2.957071	3.017127	3.040404	2.81246
PNKD	1555713_at	2.404228	2.374506	2.331689	2.357687	2.459224	2.359126	2.369998	2.373191	2.362003	2.347044	2.354462	2.369255	2.239331
HRH3	1555715_a_at	1.970982	1.963492	1.562402	1.948621	1.954679	1.946976	1.96283	1.963732	1.960067	1.948079	1.939395	1.95283	1.924668
CXADR	1555716_a_at	2.612218	2.572785	2.600052	2.57503	2.597675	2.548208	2.602678	2.597848	2.589324	2.585222	2.583125	2.591599	4.769346
C3orf15	1555719_a_at	2.867648	2.791568	2.737955	2.798381	2.868982	2.792441	2.807934	2.807005	2.802952	2.797795	2.809121	2.84037	2.712077
SCAMPER	1555722_at	2.137108	2.117712	2.098032	2.098218	2.122101	2.104934	2.118064	2.118758	2.111538	2.105509	2.100649	2.112838	2.043206
TAGLN	1555724_s_at	5.22383	5.21509	6.920326	6.145431	5.204056	5.244583	3.10703	3.090528	3.104191	3.088718	4.473097	3.079969	8.208545
RGS5	1555725_a_at	2.984125	2.976163	4.002999	3.241706	2.972646	2.965641	2.912419	2.914643	2.916066	2.887644	2.899969	2.966588	6.25589
MS4A4A	1555728_a_at	3.102205	3.132546	6.318351	6.23585	3.240658	3.11689	3.226999	2.911463	3.653147	3.541941	2.894144	3.119223	3.731527
CD209	1555729_a_at	3.430949	3.401808	3.376032	3.378033	3.409779	3.442235	3.287357	3.401379	3.324682	3.377626	3.379596	3.394612	2.867372
CFL1	1555730_a_at	8.151838	8.24433	9.331984	8.408165	7.226444	5.742255	10.81891	8.493441	10.76418	10.74053	8.739482	8.69069	12.07247
AP1S3	1555731_a_at	2.964459	2.880643	2.828355	2.8706	2.953211	2.814815	2.885389	2.893832	2.85747	2.839712	2.957455	2.912436	2.776203
AP1S3	1555733_s_at	2.762706	2.704396	2.627433	2.683418	2.7119	2.685841	2.688264	2.698148	2.688688	2.661776	2.690531	2.698312	2.629804
AP1S3	1555734_x_at	2.661999	2.63479	2.529626	2.585942	2.634821	2.594149	2.602447	2.628266	2.612227	2.600196	2.616787	2.678084	2.559877
BAP1	1555735_a_at	4.665739	4.448819	5.51822	4.776471	4.15542	3.667406	6.609183	4.720281	6.672081	6.270731	4.628664	4.634932	5.708056
AGTRAP	1555736_a_at	4.180727	3.932393	4.090469	3.915505	3.901108	3.444542	4.248542	3.930582	4.032405	4.546067	3.345755	3.734111	5.340723
KLK4	1555737_a_at	1.911407	1.901529	1.879094	1.883043	1.897887	1.882985	1.894016	1.895195	1.886033	1.881465	1.887596	1.894299	1.833181
CMTM1	1555738_at	2.029526	2.015697	1.995294	1.999809	2.008688	1.995294	2.008811	2.010968	2.000647	1.994506	1.993615	2.008278	1.986782
MRAP	1555740_a_at	2.451105	2.394083	2.375934	2.390279	2.432943	2.373092	2.391705	2.399095	2.382803	2.369799	2.383999	2.415151	3.470328

MRAP	1555741_at	1.993415	1.97292	1.955415	1.960257	1.977047	1.96011	1.98168	1.973709	1.963754	1.955696	1.957143	1.967853	1.899003
LYZ	1555745_a_at	3.366352	3.230335	3.651335	3.128086	3.277133	3.149004	5.562362	3.229499	3.188382	5.235848	3.217299	3.252182	3.905878
CD79B	1555746_at	2.729873	2.722254	2.683003	2.70293	2.724866	2.694908	2.718258	2.721308	2.706372	2.698737	2.717197	2.787389	2.497166
CD79B	1555748_x_at	2.768739	2.748239	2.723389	2.725185	2.756419	2.729188	2.740397	2.745297	2.733362	2.718427	2.727105	2.738751	2.577058
SF1	1555749_at	2.37142	2.333034	2.262713	2.304932	2.360999	2.303798	2.319961	2.331551	2.317077	2.31078	2.336732	2.368998	2.19371
GEMIN7	1555751_a_at	5.015402	4.532415	4.907062	4.837505	4.275716	4.324976	5.936931	4.454597	5.710654	6.239181	4.415347	4.801714	5.818619
STH	1555752_at	2.611838	2.607901	2.660489	2.589905	2.60789	2.58973	2.583912	2.597324	2.581016	2.566406	2.5895	2.599874	2.411462
ATN1	1555754_s_at	2.629159	2.594991	2.546694	2.577726	2.611686	2.576985	2.585337	2.595611	2.578235	2.570297	2.579092	2.591651	2.422661
CLEC7A	1555756_a_at	4.098515	3.261921	4.224788	4.512659	5.742301	5.358388	3.193372	3.107849	3.04612	3.095414	3.290586	3.131901	2.805222
C7orf34	1555757_at	3.494836	3.47222	3.304343	3.409922	3.60839	3.449085	3.411612	3.431223	3.440828	3.384885	3.496841	3.570246	3.087459
CDKN3	1555758_a_at	2.291218	2.257832	2.185251	2.192703	2.248733	2.191705	8.527475	7.154217	8.800781	8.963891	6.68382	7.112016	6.736135
CCL5	1555759_a_at	3.165545	3.095138	2.728972	2.685199	2.804562	3.694737	2.746381	2.572904	2.657031	2.537664	2.528688	2.724033	2.325161
RBM15	1555760_a_at	3.260376	3.258765	3.951863	3.209342	2.884553	2.481131	6.4576	4.939858	6.526483	6.622997	5.017455	3.985353	6.790966
RBM15	1555761_x_at	3.288734	3.282887	3.263655	3.264458	3.265182	3.261735	3.280765	3.266552	3.324581	3.270167	3.266497	3.27025	3.257823
RBM15	1555762_s_at	7.367309	7.166405	7.116214	7.52257	7.606659	7.091808	8.17655	8.348439	8.392919	8.280889	8.236527	8.236476	7.4046
MKL1	1555763_x_at	3.853156	3.570878	3.468264	3.580975	3.653785	3.606406	3.724117	3.63833	3.572163	3.610383	3.54747	3.864014	3.368776
TIMM10	1555764_s_at	8.824533	8.487509	9.37036	9.464646	9.661676	9.691859	9.084641	10.12959	9.042607	9.22314	9.128371	10.28379	8.126596
GNG4	1555765_a_at	2.152925	2.137037	2.117485	2.119721	2.14927	2.135595	2.135033	2.145109	2.138219	2.122384	2.129283	2.139402	2.117799
GNG2	1555766_a_at	2.409532	2.362366	2.313197	2.350634	2.397497	2.342881	2.369933	2.371901	2.359388	2.339544	2.355216	2.370646	3.401562
CDC25A	1555772_a_at	2.594276	2.546475	2.494056	2.531385	2.592675	2.510549	7.733877	7.487264	7.204862	7.367553	7.602504	7.700205	5.621295
BPIL2	1555773_at	2.455066	2.449061	2.400555	2.426759	2.453311	2.426672	2.438293	2.448531	2.432683	2.434686	2.434301	2.442659	2.301268
ZAR1	1555774_at	2.848106	2.816354	2.73669	2.803839	2.831782	2.804339	2.805429	2.817802	2.801768	2.775781	2.801078	2.835242	2.580517
ZAR1	1555775_a_at	2.626186	2.607432	2.558013	2.593603	2.618774	2.571142	2.60325	2.607544	2.602825	2.586507	2.590296	2.88991	2.298828
POSTN	1555777_at	2.310376	2.283319	2.262459	2.263877	2.284858	2.254928	2.27806	2.282623	2.271584	2.257698	2.265387	2.276966	2.215977
POSTN	1555778_a_at	4.411906	3.874979	5.250497	4.24446	3.976599	3.80678	7.117997	4.030599	4.531568	4.447145	7.749949	4.684191	13.62434
CD79A	1555779_a_at	2.74244	3.288352	2.700036	2.71108	2.675726	2.705554	2.722164	2.724022	2.725698	2.70803	2.709599	2.746009	2.664502
RHEB	1555780_a_at	7.931301	7.549673	8.980775	8.575957	7.003164	6.0941	9.289669	7.212823	9.434246	9.373347	7.369747	7.19708	9.472156
PQLC2	1555781_at	5.473647	5.262902	4.451418	5.089106	6.021424	5.712671	5.154597	5.904466	5.150354	5.06053	6.299102	5.509695	3.538393
PQLC2	1555783_x_at	4.57684	4.72487	3.62278	4.711224	4.857055	4.572021	5.25077	5.42256	4.41812	4.546384	4.699004	5.633916	3.222507
IRAK1	1555784_s_at	1.757284	1.755754	1.829046	1.738377	1.746893	1.742943	1.750467	1.744291	1.746299	1.739199	1.725987	1.738065	1.996453
XRN1	1555785_a_at	3.204846	3.193795	3.175968	3.17068	3.193892	3.01463	3.801209	3.19071	3.357141	3.653442	2.966392	3.166951	3.140103
C11orf63	1555787_at	2.187115	2.175783	2.156905	2.162002	2.215314	2.140344	2.165016	2.174921	2.169326	2.139241	2.162354	2.169263	2.139832
TRIB3	1555788_a_at	2.1693	2.157671	2.434238	2.147998	2.154951	2.135488	2.148895	2.156757	2.142055	2.148451	2.140516	2.151336	2.131773
PHF23	1555789_s_at	3.704705	3.796138	3.78748	3.811486	3.603291	3.404115	5.324548	3.791223	5.329307	5.291626	4.140546	3.728275	6.03305
ZNF320	1555790_a_at	10.10434	9.94871	10.11451	9.986217	9.524497	8.971054	8.03142	7.353243	7.91199	7.943019	6.914235	7.291712	7.807612
CCDC116	1555792_a_at	2.146012	2.137799	2.120038	2.12144	2.134421	2.120672	2.128674	2.134316	2.122555	2.11841	2.117161	2.128439	2.040165
ZNF545	1555793_a_at	2.518862	2.523187	2.408803	2.4366	2.494235	2.412883	5.176486	3.984736	4.771844	4.762153	3.658848	4.278313	7.118846
ARL17	1555794_at	2.820558	2.75338	2.685249	2.740151	2.818977	2.78971	2.754175	2.762536	2.751115	2.733052	2.751821	2.786421	2.530969

ARPC5	1555797_a_at	7.05777	6.808643	8.042324	7.475182	7.545062	7.695118	7.888719	7.389566	8.020488	7.605345	7.349099	7.527881	9.560914
DKFZp761D	1555798_at	3.246365	3.038087	3.044465	3.075124	3.273377	3.058138	3.094996	3.107363	3.092115	3.063582	3.142751	3.210683	2.725066
FCRL5	1555799_at	1.984594	1.966295	1.945973	1.948304	1.965925	1.950699	1.960359	1.962256	1.950875	1.949285	1.948298	1.958156	1.901568
ZNF533	1555800_at	5.382278	5.184819	7.336913	6.85796	4.567463	3.593138	3.294178	2.534041	3.334717	3.420528	2.618662	2.528819	3.89698
ZNF533	1555801_s_at	6.340936	5.78945	7.682945	7.102622	6.281002	5.467363	3.528246	3.460764	3.965309	3.477289	2.992731	2.976331	5.27286
C11orf57	1555803_a_at	4.756974	4.676336	4.642151	5.078954	4.756111	4.583844	5.416209	4.784075	5.644031	5.49151	4.952057	4.643722	6.551892
YSK4	1555804_a_at	2.433692	2.405921	2.373477	2.382242	2.435641	2.383516	2.394937	2.402364	2.389947	2.382535	2.390194	2.399339	2.307245
MOG	1555807_a_at	2.448163	2.431795	2.405594	2.414171	2.436588	2.422535	2.430026	2.431344	2.416765	2.402447	2.414176	2.426791	2.284262
EXDL2	1555808_a_at	2.538446	2.491721	2.491132	2.492038	2.509224	2.495171	2.509598	2.50708	2.497931	2.476157	2.488621	2.501459	3.668679
CRISPLD2	1555809_at	2.804511	2.785263	2.757566	2.732076	2.789203	2.764022	2.775163	2.785092	2.770673	2.762298	2.758766	2.821121	2.545288
ARHGDIB	1555811_at	2.484773	2.464855	2.445789	2.444932	2.465487	3.06738	2.468565	2.711371	2.448872	2.445097	2.448994	2.458386	2.386658
ARHGDIB	1555812_a_at	5.018932	4.982095	6.845341	5.828302	4.992895	4.05076	7.499409	4.646689	7.494364	7.15481	4.303177	5.35212	6.318577
RHOA	1555814_a_at	6.111959	5.616238	7.887364	6.872876	3.800452	2.515729	9.080415	4.338261	8.947536	8.949144	5.245886	4.760955	11.3541
L3MBTL2	1555815_a_at	2.886308	3.296553	3.327299	3.596303	3.065555	2.934642	4.901947	3.888166	4.838574	4.844336	3.135193	3.077858	4.178534
SAMD14	1555819_s_at	2.869421	2.867454	2.84115	2.778874	2.8583	2.848386	2.862504	2.857094	3.205634	2.959953	2.842757	2.967673	3.163991
MKS1	1555820_a_at	2.437655	2.423294	2.392676	2.397535	2.528832	2.383405	2.421633	2.417625	2.416167	2.410683	2.400211	2.408571	3.091348
AKT1S1	1555821_a_at	3.930286	3.834661	3.813963	3.817728	3.834284	3.585236	3.833061	3.83207	3.821243	3.869836	3.893925	3.82721	3.819375
RP11-34P13	1555822_at	2.470792	2.44962	2.396847	2.421021	2.45329	2.426902	2.468756	2.941176	2.43339	2.774489	2.467859	2.443044	2.30456
EPR1	1555826_at	2.360596	2.319885	2.246829	2.277576	2.296981	2.291498	3.791225	5.040533	4.121738	5.433736	3.675153	3.828038	2.606081
CCNL1	1555827_at	4.004371	3.732296	4.754608	4.354009	3.85416	3.751804	3.874255	3.300852	4.564214	4.060439	3.693635	3.719654	3.649668
FAM62B	1555829_at	3.014893	2.838728	2.901829	2.874926	3.012785	2.862289	2.859423	2.87841	2.916617	2.833486	2.925729	2.973276	2.641452
FAM62B	1555830_s_at	2.922101	2.823805	3.081782	2.900102	2.906	2.829649	2.912991	2.829355	3.05217	3.364988	2.889722	2.905238	3.899117
LRRC41	1555831_s_at	3.572496	3.641397	4.59593	4.100359	2.347842	2.476988	6.68967	3.848972	6.661868	6.844514	3.301016	4.29019	7.271101
KLF6	1555832_s_at	11.05933	11.13287	10.77003	11.13877	10.73232	10.76044	8.618942	8.730971	8.657365	8.555872	8.989994	9.232976	9.419856
UCHL1	1555834_at	1.974153	1.968924	1.948713	2.01779	1.959899	1.957162	1.959079	1.962778	1.949403	1.950744	1.944547	1.957352	1.943495
POLR2B	1555836_at	3.641657	3.512766	3.429313	3.78652	3.664094	3.459697	3.612878	3.494108	3.868874	3.599013	3.589188	3.616087	3.580135
POLR2B	1555837_s_at	10.66246	10.39031	10.60863	10.62289	10.31245	10.1407	11.19024	10.78026	11.28654	11.29408	10.85043	11.01524	11.68135
LOC152118	1555839_a_at	2.546269	2.498314	2.451435	2.488754	2.539965	2.485166	2.503206	2.514342	2.500418	2.493361	2.501492	2.52196	2.343186
LOC284356	1555842_at	3.958487	3.523189	3.503977	3.281313	3.64685	4.719446	3.520056	4.003689	3.503939	3.376358	5.405392	4.07222	5.053533
HNRPM	1555843_at	1.423352	1.420175	1.421382	1.450064	1.411428	1.398563	1.82135	1.420738	2.501453	1.809753	1.422938	2.396878	1.426164
HNRPM	1555844_s_at	10.17673	10.12564	10.15992	10.14841	10.31933	9.84467	11.43174	11.16922	11.48719	11.50031	11.31082	11.27757	11.69051
LOC284454	1555847_a_at	6.066852	5.476257	6.571903	6.839481	8.988447	9.411838	5.021402	5.09907	4.255716	4.312736	6.318825	5.452581	6.085467
SEPW1	1555851_s_at	8.58299	8.648871	9.553073	9.637488	9.424023	9.632869	8.538812	9.064272	8.402414	8.229409	10.67267	9.498651	11.01277
MICALL2	1555862_s_at	3.176971	3.040698	2.994413	3.00937	3.163808	3.388234	3.083162	3.161086	3.142442	3.142162	3.278747	3.194139	2.746487
PDHA1	1555864_s_at	9.865974	9.869813	9.136574	9.429401	9.727532	9.880391	9.963396	10.45597	10.24686	10.18855	10.36997	10.40127	9.386635
LOC255512	1555865_at	2.45482	2.431428	2.374884	2.406981	2.438971	2.406041	2.428011	2.423459	2.404592	2.410685	2.404919	2.417922	2.276288
HEXDC	1555866_a_at	6.018037	5.98035	5.6455	5.495499	6.771507	6.96445	3.944271	5.423279	3.70381	4.156472	5.464597	5.549121	4.232756
LOC200169	1555867_at	2.131633	2.09858	2.075679	2.08078	2.106267	2.081844	4.116179	5.378761	4.758832	3.030223	5.175879	4.47455	5.978814

LOC729070	1555868_at	3.719064	3.429831	3.333671	3.745475	3.506057	3.374502	3.55838	3.468853	3.57855	3.528075	3.409408	3.5417	3.019293
LOC729070	1555869_a_at	2.548527	2.503408	2.459756	2.49158	2.600111	2.470637	2.495269	2.506454	2.498982	2.478919	2.505556	2.509821	2.347203
RNF207	1555870_at	5.514696	5.000025	4.471655	3.782048	3.664023	3.598897	3.966472	3.60858	3.455108	3.189373	5.366287	3.601361	4.386168
KIAA1648	1555871_at	2.625301	2.558809	2.778904	2.849561	3.338603	3.664638	2.580474	2.568427	2.408487	2.371002	2.704714	2.468354	2.392506
RP11-262H1	1555872_a_at	4.231458	4.562642	4.080973	4.312723	5.402967	5.808303	4.314017	5.138444	4.217416	4.507037	5.79543	5.469338	5.889008
RP11-262H1	1555874_x_at	5.862464	6.211677	5.54442	5.583028	6.40586	6.752794	5.635636	5.891484	5.661553	5.561544	6.173673	6.690159	7.249721
RPS24	1555878_at	7.401205	7.608761	7.303435	7.073684	7.291925	6.864413	7.601242	8.032024	8.046099	7.850912	7.608691	7.758056	5.894669
HERV-FRD	1555880_at	2.140602	2.120769	2.09965	2.101641	2.122071	2.103272	2.11536	2.119002	2.106484	2.09751	2.102385	2.114101	2.04761
LZTS2	1555881_s_at	4.98357	5.128149	3.815353	4.650183	4.998713	5.094394	4.769563	5.455709	4.787586	4.958435	5.554115	5.429163	5.44808
SPIN3	1555882_at	1.888346	1.874453	2.039542	1.859345	1.871314	1.856491	1.869225	1.870311	1.866901	1.853996	1.852719	1.864007	4.181804
SPIN3	1555883_s_at	3.552523	3.606788	3.529433	3.531588	3.541762	4.239519	3.339421	3.521019	3.416176	3.103587	3.528451	3.534907	4.837111
PSMD6	1555884_at	3.085206	2.984615	2.928561	2.976309	3.073847	2.963977	3.021939	3.018516	3.094271	3.260688	3.016593	3.048827	2.800238
PDSS2	1555886_at	7.383976	7.263538	7.341099	7.3802	6.87863	6.575664	6.473467	5.91999	6.656388	6.355181	5.893532	6.063127	4.622901
UBR5	1555888_at	3.802615	3.552591	3.027279	3.370289	3.073358	2.867627	3.030573	3.041274	3.545313	3.105154	2.991947	3.16192	2.874354
CRTAP	1555889_a_at	8.356743	8.186299	8.485902	8.514651	9.003405	9.198061	8.024808	8.150712	7.88199	7.880762	9.16103	8.488283	9.691748
LOC253039	1555892_s_at	6.397866	6.73368	5.595627	5.964486	7.016483	6.843383	6.431606	6.304029	5.959217	6.386552	7.614802	5.672179	4.974018
ABBA-1	1555894_s_at	6.973382	6.785234	5.743489	6.039361	6.3291	6.639264	5.730499	6.175625	5.652815	5.686395	6.174576	6.24426	5.703295
DNM2	1555895_at	2.07016	2.050114	2.035346	2.04892	2.061374	2.035227	2.058899	2.467723	2.05734	3.099991	2.125258	2.053424	1.999146
ADAM15	1555896_a_at	4.054521	4.049905	4.031029	4.03306	4.526018	4.031656	3.34396	4.030316	3.680016	4.023178	4.023245	4.023336	3.975327
AOF2	1555897_at	2.877366	2.662353	2.707246	2.645218	2.667636	2.659683	2.670644	2.66129	2.652035	2.627165	2.815703	2.659713	4.30748
LOC150759	1555898_at	3.240253	3.186856	3.193085	3.269352	3.270773	3.140586	3.189995	3.198304	3.232018	3.136932	3.244151	3.338447	3.329606
ARMCX5	1555902_at	3.341231	3.414003	4.031633	3.821833	4.36144	4.856674	3.28967	3.488115	3.405576	3.297545	3.966587	3.411886	4.663626
C3orf23	1555905_a_at	6.595382	6.342337	6.827741	6.619325	5.626432	4.732953	5.881464	4.673724	5.838495	5.929998	4.29789	5.398845	2.784521
C3orf23	1555906_s_at	8.408837	8.396466	7.873859	8.271927	8.349484	8.289954	7.149026	7.148025	7.367357	7.493125	7.057497	7.74806	3.851348
FAM120A	1555908_at	2.705154	2.712286	2.584302	2.52794	2.732075	2.471005	3.01664	3.579275	2.708374	2.860847	2.811285	3.216275	2.487638
PTCD2	1555910_at	5.621659	5.344858	5.733247	5.471021	5.420722	5.136656	5.689312	5.377824	5.842412	5.889351	5.115136	5.579348	6.106283
ST7OT1	1555912_at	2.236712	2.220993	2.198282	2.211278	2.220832	2.21006	2.201625	2.219158	2.199431	2.191096	2.201207	2.212745	2.180594
GON4L	1555913_at	5.076885	4.855852	5.099985	5.284189	5.79939	5.931421	4.859505	5.909605	4.918832	4.783508	6.039254	5.816282	5.191376
FLJ20433	1555914_a_at	2.453306	2.404963	2.373086	2.38559	2.424922	2.526272	2.395368	2.404661	2.390333	2.37729	2.388261	2.400001	2.361115
RPUSD3	1555916_at	6.261495	6.201234	5.383842	6.021179	6.400069	7.063077	6.581225	7.008136	6.452895	6.346579	7.014234	7.197634	6.048947
CBX3	1555920_at	4.845978	3.841724	4.790439	5.108904	5.180584	5.170595	6.505162	6.025392	5.798975	5.721745	5.868984	6.177327	5.450374
C10orf114	1555922_at	3.594483	3.610262	3.572857	3.599382	3.699595	3.804248	3.195323	3.885353	3.57426	3.571322	3.593564	3.941932	2.447826
C10orf114	1555923_a_at	6.476149	6.63318	5.836269	6.237421	7.012584	7.055038	5.60383	6.822777	5.439435	5.684034	6.248742	6.679602	4.062384
KIAA2013	1555933_at	5.033591	5.513067	4.506366	4.980933	5.118371	5.320897	4.779568	5.458196	4.983182	4.975897	5.236167	5.125942	4.423449
HUNK	1555935_s_at	2.20296	2.192044	2.159175	2.163922	2.19628	2.170377	2.187533	2.177606	2.170617	2.165564	3.178574	2.183894	5.82161
VIM	1555938_x_at	3.301901	3.124626	3.12823	3.286897	3.337473	3.28626	3.231887	3.219803	3.174621	3.184727	3.298854	3.26334	6.186752
LOC642587	1555942_a_at	2.575057	2.526125	2.437399	2.518071	2.531179	2.508674	2.520395	2.531335	2.513087	2.498553	2.514406	2.559821	2.514945
PGAM5	1555943_at	2.359212	2.347747	2.328929	2.638719	2.340291	2.335877	2.343536	2.334704	2.334927	2.38325	2.326445	2.338192	2.290434

FAM120A	1555944_at	1.660486	1.600812	1.580253	1.588271	1.697646	1.592371	1.600501	1.582585	1.596418	1.581888	1.582315	1.58627	1.572615
FAM120A	1555945_s_at	8.477927	8.153269	8.517756	8.682127	8.777427	8.613394	8.168672	8.25629	7.933141	7.779973	8.513125	8.645865	7.22223
FAM120A	1555947_at	2.132883	2.11739	2.114233	2.172906	2.117985	2.097003	2.118111	2.116962	2.103139	2.096464	2.09564	2.108364	2.027203
FAM120A	1555948_s_at	8.294665	8.247449	8.953714	8.211757	7.420096	6.697422	8.802112	7.163859	8.831816	8.556859	6.831209	7.195949	8.323964
CD55	1555950_a_at	6.79652	6.722043	7.973119	7.659557	6.287101	6.064407	7.396723	7.232116	7.950556	7.974768	6.274358	7.322252	6.524061
SLC19A1	1555952_at	2.501844	2.982568	2.068953	2.072251	2.078071	2.071837	2.081964	2.089997	2.082228	2.072881	2.245137	2.080365	2.049147
COL18A1	1555953_at	3.224282	2.556289	2.406018	2.450627	2.474922	2.510119	2.445257	3.029105	2.458473	2.453641	2.685275	2.910594	2.339523
CRTAC1	1555958_at	1.689556	1.682289	1.654022	1.667642	1.684051	1.66745	1.670656	1.675242	1.664604	1.658091	1.660414	1.671779	2.789017
HINT1	1555960_at	7.131098	7.261075	7.442442	7.310003	6.926437	6.66735	6.891992	6.237448	6.793503	6.308913	5.996712	6.704994	6.124763
HINT1	1555961_a_at	12.84855	12.79544	12.87359	13.0169	12.95672	12.80118	12.88824	12.82168	12.80118	12.84292	12.69524	12.90396	12.31422
B3GNT7	1555962_at	4.428114	4.332112	4.222336	4.325923	4.376781	4.225833	4.281372	4.344624	4.328076	4.254919	4.252357	4.315012	4.46822
B3GNT7	1555963_x_at	3.290569	3.172793	3.202427	3.185087	3.234574	3.172081	3.18091	3.508325	3.206784	3.161641	3.243012	3.244657	3.013156
ARL17P1	1555964_at	2.535924	2.487731	2.461377	2.476502	2.60472	2.465573	2.511949	2.502474	2.525813	2.508875	2.505371	2.743935	2.676799
FBXO28	1555970_at	3.803632	3.775625	3.589385	3.639898	3.890296	3.61074	4.213841	4.090104	3.912012	4.555696	4.696589	4.795542	5.833509
FBXO28	1555971_s_at	4.713263	4.927745	5.788507	5.497078	4.031789	4.077454	5.206773	5.263556	5.72957	5.340701	4.487741	5.03982	5.433134
FBXO28	1555972_s_at	3.289128	3.156249	3.230252	3.232898	3.319459	3.233867	3.193327	3.180975	3.191444	3.232561	3.22548	3.256914	3.824239
FLJ39609	1555979_at	1.888866	1.877285	1.852727	1.857083	1.881657	1.859371	1.872917	1.877393	1.861549	1.855084	1.855862	1.871688	1.820527
FLJ39609	1555980_a_at	2.184361	2.248169	2.15148	2.146444	2.742224	4.327809	2.092182	2.174664	2.164569	2.152583	2.202346	2.161755	2.160325
C17orf65	1555981_at	2.77052	2.854596	3.105534	2.7092	3.124508	2.624302	3.301268	2.727351	3.397726	2.950925	2.711201	3.51007	3.133011
C17orf64	1555985_at	3.329538	3.324216	3.297289	3.30521	3.344738	3.304308	3.174745	3.316342	3.30625	3.308156	3.302981	3.310148	2.751005
LOC126536	1555988_a_at	2.967134	2.833579	2.770894	2.823048	2.895024	2.887044	2.729692	2.763019	2.828211	2.799253	2.833488	2.867901	2.616695
RP1-127L4.(	1555990_at	1.643988	1.635512	1.618019	1.624006	1.641599	1.618159	1.63516	1.634161	1.630601	1.621852	1.618308	1.61937	1.596122
RP1-127L4.(	1555991_s_at	2.408487	2.372289	2.352533	2.357544	2.392892	2.357671	2.363786	2.373335	2.359665	2.346311	2.357576	2.37093	2.316782
EIF4A2	1555996_s_at	2.702248	2.512643	2.815337	2.49318	2.522039	2.483536	5.275164	2.541092	5.905736	5.335331	2.537447	2.651283	7.545736
IGFBP5	1555997_s_at	2.226145	2.205919	2.189659	2.194125	2.714695	2.8367	2.190718	2.192369	2.190247	2.178356	2.199003	2.196556	4.589406
ATP5H	1555998_at	3.40466	3.404857	3.777976	3.770458	3.379634	3.109784	3.394202	3.403026	3.385406	3.364849	3.364044	3.375304	3.347304
BTBD7	1556000_s_at	5.739613	5.453677	5.868418	5.622749	5.670738	5.621078	6.475429	6.54875	6.284927	6.693106	6.472465	6.660687	6.219568
LOC284939	1556001_at	1.719429	1.711445	1.694748	1.720927	1.706181	1.69431	1.708471	1.705686	1.705059	1.694973	1.692452	1.70169	1.679234
CSNK1A1	1556006_s_at	5.632966	5.684003	6.374682	5.387601	5.515695	5.625057	6.78091	5.31399	6.667429	6.655778	4.359376	4.966859	6.387978
CSNK1A1	1556007_s_at	7.807411	7.571046	8.781271	8.305844	7.602197	6.774799	8.725423	7.496816	8.36695	8.4731	8.028669	7.905716	8.414293
PEX13	1556009_at	7.296551	7.243473	6.795954	6.8527	6.621986	6.318523	6.380513	6.349987	6.429535	6.034794	5.802918	6.217771	5.653916
KLHDC7A	1556012_at	4.567991	4.36558	3.570156	3.511588	4.032853	3.757847	3.824389	3.487736	3.320449	3.506022	3.129037	3.496869	2.723859
MESP2	1556014_at	2.446741	2.432717	2.41112	2.414847	2.432468	2.557016	2.416994	2.429521	2.413959	2.408443	2.410765	2.427011	2.269524
MESP2	1556015_a_at	2.60463	2.599538	2.532854	2.582152	2.595338	2.585588	2.5945	2.587042	2.582142	2.561581	2.578338	2.579619	2.513181
NBEAL2	1556017_at	2.230396	2.193923	2.147948	2.178633	2.489494	2.179288	2.186377	2.195833	2.180901	2.174463	2.17961	2.190231	2.093305
LOC144874	1556019_at	2.690066	2.657546	3.430712	2.68288	2.705403	2.612934	2.630491	2.641537	2.628298	2.610227	2.626847	2.655995	2.585112
LOC144874	1556021_at	3.627718	3.318902	5.475409	5.364433	3.580027	3.285184	3.736545	3.393685	4.696128	4.628266	3.563779	3.47794	5.672623
EME2	1556024_at	2.039375	2.031409	2.013998	2.016122	2.033606	2.013376	2.030609	2.022743	2.017811	2.067899	2.014389	2.036369	1.973372

IDS	1556026_at	3.419682	3.054955	3.013967	3.525522	3.517945	4.756624	3.042435	3.039089	3.055474	3.025063	3.094733	3.16025	2.794423
NMNAT2	1556029_s_at	1.908969	1.896393	1.878124	1.882561	1.895316	1.88257	1.890373	1.893069	1.88173	1.879476	1.875601	1.886671	2.234108
SMTN	1556030_at	3.066489	3.059266	3.041283	3.04563	3.055063	3.048147	3.057456	3.043646	3.052843	3.041921	3.042339	3.041349	2.682813
MTMR11	1556034_s_at	2.230389	2.220297	2.199672	2.205377	2.21928	2.533677	2.215398	2.215776	2.206793	2.202534	2.205272	2.208262	3.931924
ZNF207	1556035_s_at	6.303446	6.091427	6.800586	5.86467	5.191786	3.918695	8.019384	6.488479	8.012426	8.002139	5.956183	6.824866	7.850835
HHIP	1556037_s_at	2.969374	2.653111	3.288172	3.355683	3.291838	3.888231	3.368083	3.635198	3.223813	3.298291	3.804734	3.807544	3.516688
LOC338799	1556042_s_at	4.52399	4.462383	4.407882	4.280296	4.512316	4.613349	4.471555	4.460638	4.431774	4.448303	4.418903	4.578145	4.19185
LOC157627	1556046_a_at	2.155152	2.144752	2.126856	2.131494	2.142146	2.137218	2.14361	2.141373	2.131072	2.133391	2.132108	2.140784	3.098626
MAGEE1	1556047_s_at	3.144595	3.111505	2.747496	2.986763	3.186699	3.714574	2.918027	3.007733	3.094259	3.073567	3.107544	3.100078	6.126128
RTN4	1556049_at	3.828483	3.28807	3.278354	3.428957	4.765869	4.469159	3.284053	3.284933	3.272415	3.26205	4.044703	3.279879	4.957119
BICD1	1556051_a_at	3.048462	3.101276	2.902922	3.006382	3.097051	2.984008	3.014738	4.02953	3.009967	2.967891	3.839051	3.13158	6.240749
DNAJC7	1556053_at	5.568189	5.247675	5.21283	5.108828	5.296142	5.534785	5.123457	5.135496	4.749227	5.108601	5.210655	5.061961	5.266355
SNF1LK2	1556056_at	6.201647	6.098943	5.772546	6.017281	5.605053	5.594904	3.049513	3.739473	2.920463	2.762154	3.464095	2.951236	2.721644
NEUROD1	1556057_s_at	2.298564	2.268443	2.246819	2.250835	2.273918	2.251847	2.258816	2.267962	2.254886	2.25539	2.255177	2.264948	3.531285
SPEN	1556058_s_at	2.288194	2.280046	2.243355	2.249353	2.277256	2.253055	2.268715	2.267712	2.267929	2.267017	2.258935	2.270753	2.310563
SPEN	1556059_s_at	6.145623	6.08817	6.49635	6.458892	5.592421	4.89433	6.906123	5.371677	6.78606	6.833882	5.355031	5.7098	6.393236
KIAA1702	1556060_a_at	7.583774	7.367346	7.672903	7.521647	7.43614	6.7979	9.866978	9.337211	9.910725	10.02417	9.283719	9.142117	9.232737
LOC283012	1556061_at	6.261865	6.05289	5.996673	6.572962	6.155046	6.856138	6.277474	6.615319	6.152522	6.160722	6.332964	6.562777	5.561072
LOC283012	1556062_at	2.802001	2.720906	2.674545	2.723372	2.790904	2.694716	2.748428	2.740528	2.793739	2.705245	2.737678	2.770014	2.882002
LOC283012	1556063_s_at	2.647798	2.612983	2.597672	2.599889	2.64732	3.050196	2.682283	2.633446	2.622701	2.605786	2.618904	2.629471	2.82185
LOC284926	1556064_at	3.981559	3.778419	3.090303	3.666629	3.724399	3.645204	5.126818	4.088573	5.652699	5.760167	4.739052	4.310732	4.46254
LOC284926	1556065_at	3.1663	3.029363	2.952569	3.055387	3.172341	2.926758	3.07164	3.101459	3.332818	3.043171	3.228831	3.183333	2.834733
JMJD3	1556066_at	3.313964	2.940788	3.290013	3.418061	3.29909	3.289181	2.294578	2.469375	2.017558	3.289664	3.020815	3.215617	3.524438
JMJD3	1556067_a_at	3.658241	3.736991	4.015959	3.72128	3.642446	3.623536	3.676711	3.639688	3.652355	3.451771	3.669348	3.725051	4.419312
HIF3A	1556069_s_at	2.225799	2.180623	2.163075	2.157301	2.180373	2.55635	7.975007	6.448001	7.171431	6.85054	6.888252	5.552059	8.701361
C22orf37	1556072_at	2.442619	2.381609	2.323557	2.369471	2.397234	3.349247	2.367311	2.381851	2.360344	2.352677	2.363941	2.372668	2.218917
LOC143286	1556078_at	5.067313	5.098336	5.082453	5.036845	5.768248	5.125082	4.825864	5.122439	4.913715	4.57936	4.648324	5.064862	3.069621
HNRPM	1556084_at	1.835867	1.818901	1.799864	1.803978	1.816531	1.801315	1.815179	1.815478	1.805325	1.800232	1.798337	1.808166	1.761258
C4orf27	1556086_at	3.285777	3.184767	3.140118	3.205273	3.296226	3.152067	3.220139	3.212502	3.214069	3.118043	3.310525	3.513129	3.062785
OR5T2	1556088_at	5.210674	5.176744	5.760476	5.622118	5.354392	4.526105	5.724296	5.432433	5.865119	5.385101	5.249495	5.234823	5.730923
HEMK1	1556092_s_at	2.116507	2.106397	2.088909	2.220363	2.102994	2.091525	2.09088	2.102037	2.118941	2.099216	2.098252	2.096885	2.055874
UNC13C	1556095_at	3.13536	3.016132	2.938076	3.009895	3.13967	2.964333	3.019502	3.02048	3.026538	2.992919	3.053266	3.09189	3.239327
UNC13C	1556096_s_at	2.87836	2.824926	2.769399	2.811953	2.882935	2.769917	2.82257	2.834282	2.820927	2.800488	2.829649	2.84705	3.946524
hCG_20257	1556099_at	3.115455	2.99365	2.935455	2.98527	3.09965	2.988331	3.00603	3.008691	2.99836	2.970498	3.010363	3.119301	2.728902
LOC286434	1556102_x_at	4.813445	4.73683	4.71213	4.57535	4.726621	4.722268	4.728161	4.730309	4.714064	4.710803	4.827868	4.715126	4.322456
DKFZp451A	1556113_at	4.614242	4.382474	4.215729	4.340897	4.558896	4.383783	4.180701	4.410614	4.261707	4.204809	4.43454	4.722302	3.556057
DKFZp451A	1556114_a_at	2.280835	2.248415	2.2113	2.215529	2.352657	2.271918	2.226639	2.240571	2.218344	2.209459	2.255168	2.242106	2.213022
GPATCH2	1556125_at	2.487565	2.460991	2.432832	2.433422	2.519702	2.427817	2.451426	2.458101	2.447338	2.424036	2.442981	2.456051	2.364186

GPATCH2	1556126_s_at	2.895996	2.84632	2.87059	2.875744	2.853876	2.796835	2.813466	2.848962	2.832231	2.786141	2.816529	2.824276	2.685541
DIP2A	1556127_at	2.048929	2.039091	2.021158	2.026728	2.172101	2.044209	2.036881	2.039578	2.029548	2.024667	2.02447	2.030157	1.989079
RASGRF2	1556128_a_at	2.472655	2.488661	2.393672	2.438373	2.45596	2.421259	2.429743	2.441799	2.426877	2.413071	2.425409	2.437048	2.293029
LOC642533	1556129_at	3.724378	3.67232	3.74073	3.744538	3.706012	3.744986	3.772216	4.640698	3.861798	4.270696	3.749702	3.752422	3.88174
FBF1	1556131_s_at	2.66161	2.636443	2.583439	2.612839	2.820057	2.611429	2.631029	2.636613	2.620476	2.611759	2.621709	2.630198	2.102252
B3GNT5	1556134_a_at	2.080477	2.068306	2.048243	2.051932	2.067297	2.050353	2.063005	2.067049	2.053923	2.047845	2.050315	2.07341	2.005743
RP11-145H	1556136_at	2.514519	2.500687	2.524688	2.483608	2.498956	2.482449	2.495407	2.496044	2.658665	2.47899	2.921467	2.488856	4.054399
DHX30	1556144_at	2.410036	2.390253	2.309492	2.348554	2.371414	2.343609	2.360959	2.370445	2.394617	2.463988	2.410279	2.37623	2.367161
ARVCF	1556149_at	2.210362	2.234162	2.172094	2.181098	2.202808	2.189905	2.19008	2.198729	2.182774	2.186225	2.183371	2.193474	2.109685
ITFG1	1556151_at	10.09711	10.11105	9.853104	10.07744	10.04894	10.12755	9.143883	9.443397	9.000678	9.059221	9.844936	9.537087	9.71118
NFKBIZ	1556153_s_at	2.534674	2.499083	2.480311	2.478607	2.51262	2.474566	2.490056	2.497625	2.486073	2.479679	2.484715	2.499793	2.397833
MGC23284	1556154_a_at	3.077228	3.050936	3.005284	3.00696	3.062664	3.059249	3.072322	3.823413	3.555042	3.579688	3.335445	3.163851	2.953362
ARL8A	1556155_at	2.119375	2.108683	2.090271	2.092367	2.105291	2.0973	2.103078	2.102953	2.086467	2.093137	2.091996	2.104762	2.033566
ESRRB	1556156_at	2.296599	2.266647	2.247986	2.252471	2.279087	2.25119	2.256902	2.26268	2.254029	2.242179	2.248044	2.261766	2.207631
DKFZp666G	1556158_at	3.469152	3.268982	3.117882	3.264271	3.598259	3.29315	3.251662	3.238546	3.286077	3.213253	3.387554	3.328501	3.103951
IGSF3	1556162_at	2.133188	2.120547	2.103677	2.101373	2.134201	2.109188	2.123483	2.114062	2.11987	2.108856	2.101744	2.113602	2.028199
IGSF3	1556163_a_at	2.077989	2.064785	2.041021	2.046282	2.06701	2.045104	2.056949	2.059746	2.047683	2.042462	2.038643	2.057419	1.998295
LOC339766	1556167_at	2.941313	2.874761	5.819544	6.206368	3.028401	2.890587	2.875723	2.82766	2.837089	2.8034	2.888761	2.90534	2.557683
LOC339766	1556168_s_at	2.501939	2.478074	5.373072	6.15692	3.097392	2.464331	2.389053	2.391214	2.466089	2.453197	2.375722	2.370066	2.321255
ABBA-1	1556175_at	2.381584	2.350729	2.321981	2.334221	2.36517	2.322285	2.345924	2.349531	2.33689	2.325387	2.332023	2.346111	2.249789
TAF8	1556176_at	2.131642	2.013091	2.106579	2.1098	2.11724	2.112773	2.123008	2.117809	2.608211	3.421096	2.107832	2.108597	2.10213
TAF8	1556178_x_at	2.619825	2.616463	2.596828	2.585428	2.686391	2.583607	3.087651	2.610981	3.683358	4.37095	2.593641	2.915818	2.60255
LOC729678	1556180_at	2.492449	2.746841	2.493948	2.470603	2.495616	2.389487	2.551489	2.480219	2.478221	2.47295	2.46872	2.473217	3.874198
hCG_20426	1556181_at	2.855867	2.834265	2.823974	2.817134	2.836831	2.811647	2.792847	2.822938	2.832861	2.816845	2.951515	2.831161	2.799387
hCG_20426	1556182_x_at	3.056698	2.87571	2.853335	2.85858	2.876734	2.86507	2.857903	2.868731	2.854113	2.854563	2.859997	2.863873	2.848539
FLJ40330	1556183_at	1.981942	1.94461	1.953779	1.956405	1.967599	1.957467	2.379747	1.96965	2.182862	1.955984	1.950838	1.959854	3.898958
KIAA0090	1556186_s_at	7.130282	7.050855	6.427471	6.525124	6.594901	6.113937	7.204677	7.166199	7.154325	6.761547	7.272549	7.217943	6.841041
PRNP	1556190_s_at	2.447802	2.412045	2.368631	2.394952	2.448547	2.395321	2.404255	2.413954	2.399517	2.386407	2.398092	2.414974	2.294851
RGS9BP	1556199_a_at	2.031607	2.012317	1.990887	2.000466	2.028424	1.997426	2.000994	2.012	2.000617	1.990578	1.995539	2.006695	1.936373
C10orf90	1556200_a_at	2.447597	2.415853	2.372776	2.410054	2.500866	2.4439	2.406908	2.418825	2.402766	2.387276	2.404355	2.41378	2.273812
RNASET2	1556201_at	2.54277	2.521111	2.490827	2.503557	2.529857	2.510988	2.513764	2.520556	2.504803	2.485472	2.509047	2.517688	2.426727
SRGAP2	1556202_at	2.856408	2.794185	2.816215	2.818987	2.932728	2.767639	3.212954	2.794362	3.41243	3.087646	3.747866	2.831387	5.304379
SRGAP2	1556203_a_at	3.280034	2.932402	3.2539	3.165234	3.264324	3.271795	3.880133	3.270452	3.614962	3.743711	4.185509	4.006656	5.382356
LOC730051	1556204_a_at	3.506415	3.416004	3.417828	3.394622	3.67418	3.103093	3.821704	3.328408	3.75895	3.61004	3.311746	3.617209	3.767395
CLEC2B	1556209_at	3.067516	2.974781	2.940574	2.937777	2.996929	2.926181	2.957892	2.933995	2.953259	2.916545	2.967785	2.968805	2.645529
BTG3	1556213_a_at	3.173177	3.130081	2.988769	2.920753	3.461906	3.101678	3.151818	3.155426	3.138362	3.480738	3.161419	3.13843	3.137576
RP11-291L2	1556222_at	3.13584	3.089384	3.092942	3.069905	3.116361	3.087949	3.10892	3.111963	3.115388	3.106575	3.096452	3.104545	3.007958
LOC791120	1556224_a_at	2.164753	2.158677	2.1405	2.13901	2.683235	2.91623	2.144972	2.155747	2.044876	2.130442	2.138092	2.139444	2.065232

VCPIP1	1556227_at	3.610606	3.450825	3.371803	3.728105	3.450396	3.430543	3.414455	3.435419	3.433985	3.372995	3.429015	3.442349	3.032525
VCPIP1	1556228_a_at	2.954534	3.058872	2.918894	2.884441	2.745987	2.648492	2.567043	2.726835	2.611137	2.573303	2.575296	2.691045	2.444803
KIF6	1556232_at	2.794824	2.784249	2.682994	2.743895	2.778492	2.731071	2.865629	2.958903	3.820529	3.707316	2.760424	2.808386	2.510328
KIF6	1556233_s_at	2.242521	2.219131	2.182669	2.19961	2.225747	2.194561	2.217291	2.221895	2.793324	2.430623	2.205229	2.216563	2.110859
LOC375196	1556244_s_at	2.518831	2.486477	2.432404	2.459683	2.505532	2.445441	2.556754	2.484228	2.470331	2.453587	2.474167	2.482065	2.675442
FLJ33065	1556248_at	2.176814	2.252476	2.138083	2.142313	2.18018	2.504159	2.141366	2.158048	2.122338	2.133509	2.169276	2.255616	2.05954
FLJ33065	1556249_a_at	1.974648	1.952992	1.934026	1.939582	1.977425	1.938218	1.950654	1.95267	1.942375	1.93426	1.934614	1.954198	1.896288
LOC400756	1556255_a_at	2.521367	2.491982	2.494374	2.498919	2.523811	2.614148	2.513971	2.496211	2.487364	2.465171	2.461122	2.476707	2.513971
LOC645513	1556257_at	3.184439	3.024219	2.952395	3.023648	3.276199	2.970496	3.030818	3.04001	3.06178	3.04476	3.136583	3.120323	2.940292
LOC400831	1556265_at	2.241643	2.232884	2.212532	2.214323	2.23624	2.215317	2.221637	2.231995	2.218225	2.211763	2.21631	2.392939	2.135456
LOC400831	1556266_a_at	3.218601	3.191227	3.173714	3.178483	3.182209	3.174792	3.139659	3.185504	3.175494	3.165617	3.205813	3.168856	2.981157
C12orf28	1556267_at	3.548842	3.242577	3.28086	3.305938	3.565564	3.30757	3.310936	3.322928	3.308109	3.210433	3.308542	3.326032	2.921758
MYT1	1556269_at	2.266463	2.23887	2.224807	2.220828	2.323748	2.212355	2.224525	2.238257	2.222477	2.215571	2.259907	2.232748	2.109651
PAPD4	1556277_a_at	5.259141	4.711774	6.449441	6.689099	5.81045	5.348263	6.514257	5.822965	5.855518	6.059548	5.479254	5.35223	5.531192
FGFR1OP2	1556282_at	3.342901	3.185969	3.13761	3.231564	3.331373	3.109205	3.259022	3.269634	3.26221	3.227143	3.266546	3.295634	3.427615
FGFR1OP2	1556283_s_at	5.6229	5.890363	5.740743	5.965203	5.674067	6.215376	8.235695	8.435889	8.305834	8.479721	7.937436	8.504074	5.686671
PPA2	1556284_at	4.670863	4.260096	3.853754	4.133864	4.700779	4.708023	4.204899	4.074322	4.006005	4.053239	4.332266	4.520145	3.388065
PPA2	1556285_s_at	7.880737	7.726966	8.915058	8.616697	7.562535	6.884189	9.290189	7.443088	9.239834	9.113709	7.037524	7.411888	8.447913
LOC284274	1556288_at	2.808284	2.760298	2.677876	2.741144	2.804852	2.690793	2.752374	2.750794	2.74359	2.715051	2.742921	2.773966	2.513702
DKFZp564N	1556291_at	1.36122	1.352337	1.330984	1.334844	1.344684	1.33581	1.353833	1.344799	1.348207	1.33711	1.338074	1.341565	1.354909
DKFZp564N	1556292_s_at	2.270283	2.260469	2.237299	2.248909	2.259438	2.239218	2.260166	2.260667	2.255581	2.239218	2.241594	2.250615	2.189033
FLJ40142	1556299_s_at	2.322147	2.230647	2.15978	2.187993	2.24178	2.209077	2.225456	2.204039	2.218771	2.214427	2.219487	2.296217	2.123489
PRRT3	1556308_at	2.031394	2.013858	1.99173	1.996196	2.020517	1.99542	2.007401	2.01177	2.003954	1.990932	2.00036	2.008029	1.994273
C1orf86	1556309_s_at	2.055492	2.034459	2.015896	2.021934	2.044922	2.021213	2.025257	2.035866	2.017744	2.013516	2.01863	2.02621	1.95618
FLJ34931	1556312_at	2.141805	2.129892	2.111198	2.115551	2.131021	2.113335	2.125962	2.12701	2.115452	2.105304	2.111894	2.124336	2.057888
LOC284889	1556316_s_at	1.770592	1.700695	1.677775	1.681986	1.694206	1.685544	1.695579	1.691289	1.691243	1.681058	1.681012	1.68349	1.647344
LOC283270	1556319_at	2.850463	2.819898	2.79821	2.925459	2.877755	2.813512	2.815522	2.80612	2.699296	2.763354	2.797708	2.806823	2.796761
STOML1	1556320_at	2.109783	2.102124	2.08516	2.084484	2.097356	2.085021	2.097999	2.586435	2.093051	2.085111	2.08653	2.081869	2.078293
CUGBP2	1556323_at	3.529347	3.477836	3.450655	3.387765	3.526588	3.465542	3.253327	3.38482	3.398171	3.357684	3.523502	3.555926	3.29738
FILIP1	1556325_at	4.342483	4.246166	4.789584	4.946522	4.213824	3.768753	3.978173	3.643936	3.865416	3.772649	4.978162	3.899132	7.345683
RBMX	1556336_at	7.85687	6.987217	7.11049	7.393315	8.076134	8.6252	7.465869	8.783173	6.984655	6.738311	9.360622	8.80503	7.615691
MAPK12	1556340_at	2.069419	2.072104	2.02646	2.042894	2.072901	2.052253	2.060275	2.05995	2.046746	2.049303	2.045906	2.056166	2.009385
MAPK12	1556341_s_at	2.610016	2.600502	2.58103	2.585582	2.598947	2.587754	2.596609	2.592128	2.586846	2.582385	2.578333	2.590405	2.5453
LOC150051	1556344_at	1.923779	1.910539	1.892198	1.89347	1.910044	2.671528	1.901601	1.907752	1.900329	1.895469	1.891369	1.901848	1.853826
HEATR1	1556348_at	3.584544	3.332774	3.3435	3.482125	3.658192	3.487619	4.012683	3.469435	3.487642	3.456814	3.525323	3.596079	3.411807
EIF4A2	1556350_a_at	3.018992	2.944816	2.83773	2.932703	3.024409	2.91214	2.927942	2.947422	2.932295	2.922884	2.957253	2.986823	2.775368
HCN1	1556351_at	2.975631	2.888092	2.803258	2.879356	2.962433	2.868247	2.900813	2.903524	2.890652	2.866041	2.939728	2.925543	4.385841
RGL3	1556354_s_at	3.280393	3.084801	3.039624	3.083783	3.449048	3.06499	3.069204	3.08164	3.069254	3.027403	3.118901	3.19551	2.737442

RGL3	1556355_x_at	3.987011	3.972309	3.950838	3.956803	4.04472	4.254991	3.97628	3.968215	3.971363	3.965279	3.961967	3.964152	3.296542
ERICH1	1556356_at	2.646409	2.638311	2.538385	2.622554	2.640454	3.118411	2.605328	2.6652	2.598249	2.633925	2.630427	2.636018	2.544013
ERICH1	1556357_s_at	3.345096	3.172583	3.095471	3.220637	3.37592	3.117858	3.240736	3.245384	3.240183	3.243154	3.311488	3.465505	2.988284
C6orf89	1556359_at	2.566776	2.587299	2.951204	2.566972	2.59079	2.559198	2.595347	2.590904	3.048048	2.578252	2.574792	2.610515	2.450025
ANKRD13C	1556361_s_at	2.748569	2.686046	2.668947	2.71586	2.733221	2.642583	2.833306	2.727757	3.278889	2.709465	2.713779	2.776059	2.524796
LOC730057	1556364_at	2.039219	2.20381	3.171151	2.297048	2.263418	2.513209	2.013767	1.998994	1.968472	1.983895	1.982386	1.974134	1.947096
RP3-398D1	1556365_at	2.975732	2.897138	2.878425	2.882728	2.905525	2.878184	2.878117	2.883135	2.874875	2.846692	2.880944	2.887685	2.644017
RP3-398D1	1556366_s_at	2.945791	2.828932	2.778918	2.814375	2.957538	2.787424	2.827949	2.836977	2.827187	2.800948	3.003488	2.871668	2.580279
PHKG2	1556368_at	2.158706	2.13557	2.109688	2.124563	2.147117	2.118089	2.126585	2.137687	2.132472	2.11619	2.122535	2.12974	2.015728
PHKG2	1556369_a_at	2.611129	2.586503	2.568518	2.574878	2.608097	2.568561	2.565209	2.584519	2.562307	2.542435	2.566085	2.579054	2.503867
OLIG3	1556371_at	2.596305	2.56043	2.535354	2.539178	2.578562	2.519178	2.547024	2.552166	2.542538	2.538106	2.537301	2.552132	2.531451
MGC35361	1556373_a_at	6.48995	6.762278	5.750792	5.440052	5.970846	6.192511	5.297335	5.158566	4.802373	4.29819	5.755501	4.490454	5.043753
LMO7	1556377_s_at	2.735398	2.725466	2.692782	2.70024	2.763129	2.711582	2.71893	2.725347	2.713708	2.700907	3.277377	2.720096	3.070446
LOC440896	1556378_a_at	2.321347	2.29274	2.255621	2.270222	2.305288	2.265453	2.281374	2.29338	2.278186	2.270828	2.353358	2.418554	2.17194
NARG1	1556381_at	2.675882	2.386455	2.369442	2.359163	2.384839	2.34972	2.379657	2.382587	2.379554	2.363736	2.364274	2.376033	2.25084
NARG1	1556382_a_at	3.061331	2.965198	2.950414	2.900755	2.97095	2.953318	2.958575	2.873731	2.95559	2.939915	3.019285	2.965494	2.670266
TNRC5	1556389_at	5.393228	5.407756	5.424595	5.930691	6.57591	6.972222	3.621514	4.915807	4.715748	4.472499	5.051645	5.4327	4.655559
LOC441208	1556404_a_at	2.452028	2.369499	2.307043	2.409796	2.433291	2.406462	2.430192	2.47791	2.413317	2.617613	2.414823	2.426075	2.315281
LOC374890	1556405_s_at	1.966709	1.841399	1.861855	1.867961	1.995427	1.865491	1.834069	1.87766	1.8357	1.865014	1.887232	1.933722	1.753272
LOC255025	1556406_at	2.176926	2.15227	2.12579	2.136012	2.172933	2.14126	2.143096	2.151682	2.137397	2.131347	2.134174	2.146932	2.060946
KRTAP19-1	1556410_a_at	2.707956	2.660915	2.637231	2.647024	2.704968	2.628621	2.656184	2.663909	2.651437	2.632106	2.649908	2.690342	2.464814
KY	1556411_s_at	2.324712	2.299057	2.257649	2.275593	2.30578	2.278871	2.290694	2.298223	2.283246	2.2759	2.283031	2.292836	2.174111
C21orf71	1556414_at	3.238219	3.172769	2.960999	3.058853	3.135771	3.068711	3.023679	3.052396	3.048573	2.992961	3.069483	3.097981	3.078676
YPEL2	1556420_s_at	6.540876	6.625965	7.429005	6.656632	6.877739	7.11662	4.613295	4.802831	4.624282	4.095805	4.887488	4.609439	4.628968
LOC286189	1556421_at	2.640335	2.593603	2.583957	2.58717	2.66516	2.589049	2.595571	2.602656	2.592732	2.58386	2.591041	2.633497	2.527231
VASH1	1556423_at	2.882565	2.521849	2.478248	2.479187	2.546568	2.464321	2.909005	2.522377	2.788312	2.761128	2.517907	2.525168	5.047909
LOC284219	1556425_a_at	2.732057	2.702844	2.680082	2.79561	2.715472	3.034397	2.648277	2.696844	2.639839	2.631972	2.680798	2.648437	3.542396
HEXA	1556426_at	2.242531	2.221948	2.199084	2.203354	2.225659	2.785001	2.215194	2.207374	2.201554	2.195619	2.198133	2.210666	2.139911
LOC221091	1556427_s_at	2.371321	2.304961	2.285846	2.287692	2.360016	2.286714	2.300326	2.299441	2.290757	2.280403	2.769793	2.321318	5.64119
WDR67	1556429_a_at	2.367694	2.353648	2.328739	2.329303	2.356073	2.333893	2.351874	2.350242	2.347532	2.466708	2.33722	2.34745	2.292289
LOC400622	1556435_at	2.711271	2.65682	2.610619	2.638827	2.689058	2.711653	2.651891	2.662742	2.648589	2.631909	2.647344	2.675656	2.500665
C8orf50	1556436_at	2.955809	2.640413	2.553989	2.588333	2.635446	2.589397	2.585924	2.605266	2.582911	2.561838	2.587743	2.61124	2.419599
LOC253805	1556437_at	3.031664	2.981038	2.912671	2.962463	3.163772	2.96105	2.926917	2.934492	2.947329	2.899337	2.963013	2.981994	2.644731
LOC285370	1556447_at	2.394318	2.361085	2.323174	2.340828	2.37528	2.334298	2.349926	2.361851	2.345701	2.33462	2.345566	2.357285	2.220717
LOC285370	1556448_a_at	2.733383	2.691031	2.62897	2.67574	2.722335	2.672621	2.68405	2.683884	2.679874	2.65907	2.676277	2.698302	2.498624
LOC283761	1556452_a_at	2.671551	2.652824	2.627061	2.634122	2.654261	2.632063	2.622723	2.651194	2.635999	2.628693	2.629097	2.642267	2.567447
FLJ39739	1556456_at	2.39146	2.365024	2.318048	2.345787	2.374864	2.345418	2.357164	2.545996	2.348965	2.340651	2.344567	2.357923	2.237308
FLJ39739	1556457_s_at	2.330503	2.300252	2.259582	2.280563	2.315238	2.269936	2.286023	2.279088	2.282154	2.267625	2.283481	2.292985	2.184916

LOC257407	1556464_a_at	4.868853	4.416147	4.196671	4.394749	4.593014	4.776362	4.353211	4.258433	4.185551	4.173189	4.176884	3.811001	3.355986
ZNF80	1556467_at	2.515965	2.47593	2.446539	2.462136	2.495525	2.469461	2.470414	2.484143	2.465357	2.455391	2.462906	2.471427	2.341861
SCML4	1556471_at	3.044545	3.904795	2.495246	2.596926	3.020832	2.518366	2.498132	2.543779	3.138024	2.672558	2.529702	2.653718	2.342363
SCML4	1556472_s_at	5.305016	4.860657	3.274682	3.408452	4.055376	5.229015	3.401661	3.791167	3.897294	3.563207	3.833507	3.848716	2.838479
FLJ38379	1556473_at	3.207125	3.126916	3.031861	3.100955	3.255209	3.181912	3.107463	3.142127	3.113406	3.104826	3.168361	3.208434	2.943319
FLJ38379	1556474_a_at	2.485339	2.450387	2.402182	2.43179	2.552055	2.437288	2.444746	2.453054	2.437811	2.428494	3.120753	2.446987	5.629669
LOC283485	1556476_at	2.926892	2.899958	2.836243	2.856589	2.99567	2.945248	2.88953	2.896397	2.884133	2.86386	2.897155	2.912909	2.781999
LOC283485	1556477_a_at	2.402202	2.370984	2.352108	2.35245	2.387592	2.35108	2.360111	2.36906	2.357347	2.344717	2.359873	2.366353	2.236088
LRRC52	1556484_at	2.688059	2.599513	2.532986	2.577192	2.795671	2.584925	2.585406	2.594746	2.584869	2.57294	2.659185	2.693024	2.4125
C3orf15	1556487_a_at	2.970871	2.89497	2.877782	2.882719	2.893437	2.882757	2.893494	2.894481	2.886401	2.881324	2.937053	2.9221	2.688294
C3orf15	1556488_s_at	2.798586	2.717499	2.686271	2.713443	2.803889	2.699128	2.722405	2.7328	2.721485	2.695959	2.719595	2.753025	2.582465
LOC388946	1556489_at	2.597849	2.552361	2.507577	2.534192	2.573573	2.540817	2.552186	2.555816	2.54456	2.535156	2.538747	2.559039	2.382359
LOC388946	1556490_a_at	2.640931	2.596738	2.549701	2.596847	2.62338	2.596712	2.610273	2.615764	2.599916	2.592238	2.606146	2.623749	2.510792
JMJD2C	1556493_a_at	6.36265	6.31445	5.687061	5.919794	6.662634	6.606368	6.892928	7.31847	7.096972	7.428753	7.174435	7.320229	4.68377
RPL5	1556498_at	2.757295	2.710933	2.652779	2.686025	2.729556	2.68332	2.697085	2.702507	2.696382	2.675324	2.6903	2.706276	2.545885
COL1A1	1556499_s_at	11.6328	11.66479	9.12961	9.508548	9.883075	10.87579	11.05041	10.95212	10.32079	10.67807	13.52866	11.51243	13.90483
ZNF573	1556513_at	1.942989	1.929688	1.910036	1.914167	1.932144	1.913931	1.925458	1.928371	1.915762	1.910532	1.9115	1.92098	1.870913
LOC338809	1556514_at	2.889967	2.810432	2.751155	2.792054	2.910794	2.771506	2.814483	2.815396	2.810379	2.788824	2.810649	2.864189	2.56728
C9orf93	1556516_at	2.876603	2.803919	2.764872	2.786811	2.878476	2.790052	2.801154	2.805141	2.791739	2.772458	2.798522	2.835598	2.56011
LOC285326	1556528_at	3.087349	2.928635	2.847317	2.919337	3.006407	2.884368	2.933823	2.945381	2.939708	2.893931	2.939051	2.981609	2.692374
LOC285326	1556529_a_at	3.172182	3.397453	3.116693	3.088328	3.155533	3.073376	3.068019	3.112632	3.080461	3.080695	3.113209	3.117915	2.672848
C17orf52	1556533_at	2.453375	2.427177	2.3495	2.41087	2.418756	2.415649	2.423339	2.420824	2.416141	2.393154	2.405343	2.412623	2.400164
hCG_20441	1556536_at	2.776149	2.747903	2.711601	2.728381	2.799006	2.705831	2.736384	2.988184	2.731212	2.72153	2.719643	2.758384	2.514119
MFI2	1556538_at	2.404186	2.394639	2.374366	2.375884	2.405913	2.387834	2.389774	2.400507	2.385056	2.377648	2.379649	2.386676	2.350119
SLC39A6	1556551_s_at	2.334033	2.323985	2.296689	2.293409	2.317702	2.307315	2.985176	2.281246	2.339264	2.282408	2.304186	2.321628	5.815122
LOC197322	1556552_a_at	2.021777	2.001155	1.983036	1.987793	2.004642	1.988993	1.998653	2.001118	1.9874	1.988302	1.984935	1.992368	1.962773
TRIM50	1556554_at	2.464706	2.422794	2.428818	2.432944	2.448903	2.434645	2.43004	2.445799	2.433098	2.433039	2.428881	2.437911	2.390528
FLJ36665	1556558_s_at	3.443881	3.32878	3.559515	3.843186	3.61299	3.164149	3.175748	3.174337	3.201889	3.115064	3.323034	3.200955	2.805446
TSSK3	1556560_a_at	2.041247	2.81934	1.82032	2.019723	2.028884	2.548999	2.777938	4.084156	2.053602	2.020785	2.014322	3.218225	1.952784
KIAA1822	1556564_at	2.08419	2.066785	2.042347	2.048905	2.068511	2.047162	2.059	2.064294	2.051309	2.043824	2.050078	2.091617	1.991678
NAP1L4	1556567_at	2.549952	2.546102	2.527624	2.527833	2.545895	2.68327	2.540895	2.722249	2.531932	2.523412	2.640386	3.094557	2.446519
C8orf38	1556571_at	3.588451	3.503601	3.299752	3.51825	3.493385	3.257781	3.284161	3.30949	3.301877	3.286618	3.355957	3.345286	2.909854
LOC286178	1556573_s_at	2.800606	2.772988	2.742618	2.75545	2.782815	2.759219	2.763774	2.774346	2.759342	2.741265	2.772963	2.811984	2.539814
FLJ31945	1556578_a_at	2.415468	2.399779	2.37316	2.378958	2.389582	2.369724	2.388888	2.391973	2.37843	2.383473	2.378191	2.388692	2.271006
IGSF10	1556579_s_at	2.818376	2.75701	2.658009	2.87077	2.964142	2.7391	2.751775	2.735617	2.731165	2.697952	2.735387	2.746497	7.387169
ZNF778	1556581_at	2.158154	2.591728	2.114185	2.242769	2.225311	2.122436	2.12416	2.146958	2.137728	2.120027	2.127959	2.130236	2.035448
C15orf37	1556588_at	6.107832	6.116573	5.427109	5.975864	5.376023	5.287963	4.619915	4.980473	4.733056	4.818842	5.094176	4.888721	4.169648
LOC284513	1556597_a_at	2.838972	2.820401	2.788316	2.805743	2.83693	2.814095	2.877434	3.656578	3.223959	3.264487	3.069664	2.863027	3.052097

ARPP-21	1556598_at	2.962995	2.85492	2.799772	2.847001	2.924863	2.808812	2.846004	2.849897	2.850707	2.83596	2.868924	2.9071	2.821065
ARPP-21	1556599_s_at	2.692388	2.663598	2.599801	2.644122	2.704764	2.828179	2.652826	2.664487	2.638293	2.63634	2.663289	2.680802	4.575971
SPATA13	1556601_a_at	6.376925	5.932483	6.61972	7.233224	6.717935	6.328316	3.945572	4.843932	4.211001	3.906641	4.038809	3.683241	2.497095
NAV2	1556606_at	6.201684	6.318871	3.931713	3.875227	3.788386	4.171661	4.15622	3.649633	3.463523	3.334949	3.526819	3.477971	3.416899
EHD4	1556607_at	4.411182	3.939381	4.05252	3.770329	4.3741	3.757374	3.766962	3.945844	3.75855	3.730967	4.476752	4.10277	3.255489
EHD4	1556608_a_at	2.508388	2.483763	2.449997	2.699255	2.496826	2.404091	2.416394	2.42313	2.448784	2.36439	2.466395	2.462255	2.229928
LOC401098	1556609_at	2.736149	2.63718	2.584362	2.628459	2.868892	2.618708	2.644273	2.647822	2.637349	2.616026	2.637189	2.7144	2.460706
LOC203107	1556613_s_at	3.282486	3.17476	3.149019	2.966866	3.176254	3.120742	3.239751	3.173022	3.52512	3.150618	3.255896	3.203998	4.38172
KLHDC4	1556616_a_at	2.942868	2.821397	2.84251	2.893119	2.997935	2.923769	2.890239	2.913075	2.895321	2.875823	3.174105	2.948149	2.681014
DRP2	1556627_at	2.348008	2.320994	2.269654	2.301079	2.329182	2.290448	2.309719	2.317914	2.30478	2.296134	2.305524	2.324914	2.55723
SNAP25	1556629_a_at	2.280429	2.268089	2.165023	2.249746	2.26942	2.227834	2.266554	2.261888	2.258623	2.254153	2.278083	2.236325	3.446765
CASC2	1556630_at	2.732869	2.674992	2.646584	2.671664	2.824375	2.780047	2.684434	2.697368	2.667873	2.660553	2.680667	2.704549	2.492203
C1orf204	1556633_at	2.950272	2.725101	2.67698	2.692674	2.712553	2.678518	2.701268	2.70891	2.69772	2.708216	2.689677	2.708198	2.611624
LOC284530	1556638_at	1.847373	1.838798	1.819263	1.823207	1.837378	1.823667	1.833203	1.834501	1.824399	1.821093	2.789127	1.829024	5.162781
LOC439914	1556639_at	2.308988	2.276912	2.244956	2.258926	2.289132	2.251834	2.265373	2.277482	2.2609	2.250611	2.277829	2.272474	2.158818
FLJ37228	1556641_at	2.200098	2.178128	2.146824	2.158787	2.230949	2.154391	2.166103	2.17552	2.161318	2.15407	2.180472	2.220452	3.129151
FAM125A	1556643_at	3.449279	3.370143	3.909275	3.740574	4.286389	3.696035	3.159188	3.230446	3.152642	3.015142	3.199648	2.985752	2.721282
C10orf40	1556648_a_at	2.728893	2.665913	2.621912	2.663301	2.718033	2.642441	2.67568	2.685588	2.672919	2.661588	2.673459	2.694215	2.497232
ANKAR	1556653_at	3.126233	3.071649	3.00513	3.06268	3.17524	3.024335	3.079373	3.085036	3.06211	3.019092	3.117487	3.12553	2.722184
LOC150384	1556661_at	2.409573	2.375694	2.36042	2.364865	2.393035	2.362468	2.365644	2.377473	2.364568	2.36327	2.361958	2.372257	2.285411
TTC6	1556665_at	3.538405	3.416973	3.723109	3.775409	3.670387	3.430014	3.313091	3.486188	3.396672	3.282793	3.462279	3.502444	2.964625
TTC6	1556666_a_at	2.995059	2.922895	2.904997	3.194453	3.271637	2.894885	2.802287	2.819461	2.880183	2.783923	2.888684	2.900084	2.499281
LOC348751	1556667_at	4.949825	4.983172	6.096112	6.042927	5.872387	5.549109	4.544538	4.492137	4.541731	4.439009	4.32249	4.594777	3.187331
RBM6	1556672_a_at	3.651337	4.147612	3.497336	3.398188	3.554224	3.583625	3.660453	3.636554	3.610836	3.387612	3.523473	3.768136	3.309153
RPPH1	1556684_at	2.906078	2.882788	2.850504	2.863396	3.914002	4.620932	2.45577	2.881079	2.334617	2.858027	2.868071	2.875589	1.982152
CLDN10	1556687_a_at	2.318169	2.295229	2.258948	2.271331	2.300162	2.283825	2.288231	2.289064	2.274571	2.267056	2.276354	2.288494	2.516833
WNT4	1556689_a_at	3.023312	2.931806	2.877267	2.893004	3.197991	2.904178	2.912947	2.91956	2.924597	2.879084	2.906133	2.913	2.777846
FLJ42709	1556695_a_at	2.674042	2.544994	2.545722	2.64329	2.869303	2.563604	2.648543	2.562904	2.594518	2.591359	2.72779	2.604354	3.428334
FLJ42709	1556696_s_at	2.959379	2.856892	2.856614	2.912036	2.945601	2.911077	2.92421	2.916611	2.913502	2.909318	3.542691	3.055952	4.061869
GPRIN3	1556697_at	2.926367	2.816454	2.751822	2.807895	2.944076	2.771078	2.819857	2.829598	2.816838	2.792146	2.827957	2.867557	2.613567
GPRIN3	1556698_a_at	2.799309	2.768913	2.744686	2.756428	2.782895	2.750614	2.721518	2.737985	2.75323	2.751853	2.745495	2.760953	2.69718
DLGAP4	1556701_at	2.001868	1.968536	1.948785	1.953054	2.007796	1.956908	1.963704	1.968181	1.955191	1.952494	1.952481	1.962829	1.898182
LOC286297	1556704_s_at	3.657023	3.552453	3.475377	3.494854	3.593781	3.495509	3.531606	3.543228	3.539004	3.490089	3.541252	3.575341	3.24605
LOC283887	1556709_a_at	1.845546	1.790435	1.771023	1.779808	1.79227	1.777567	1.784721	1.778764	1.782765	1.767007	1.820314	1.783768	1.729443
C13orf30	1556711_at	2.607274	2.566086	2.527432	2.558307	2.605509	2.528216	2.563906	2.572176	2.557236	2.540018	2.555997	2.581633	2.468242
PRPSAP1	1556715_at	2.490244	2.829767	2.664525	2.251803	2.610114	2.277448	2.3026	2.281392	2.277274	2.256702	2.273375	2.602684	2.716162
FLJ33706	1556721_at	1.669023	1.661219	1.640722	1.64584	1.652188	1.644066	1.656692	1.659799	1.647723	1.639146	1.64405	1.65189	1.618578
FLJ33706	1556722_a_at	2.922834	3.372739	2.929641	3.038415	3.846222	3.028699	3.082068	3.32912	3.048101	3.035476	3.045034	3.070466	2.870037

PRCD	1556727_at	2.563474	2.533842	2.513333	2.517645	2.532677	2.512724	2.536949	2.535741	2.530253	2.517688	2.514419	2.532381	2.509351
LOC652993	1556730_at	3.077743	3.551828	2.881141	3.255563	3.106044	2.86418	2.857462	2.889474	2.87773	2.835171	2.871474	2.888767	2.605616
EML4	1556732_at	3.423603	2.690462	4.285427	3.450668	2.051614	2.448507	3.986609	2.002893	4.220584	3.903237	2.04035	2.426873	4.596901
FLJ31222	1556737_at	2.111956	2.094925	2.065257	2.076035	2.201422	2.071199	2.080973	2.084577	2.07656	2.069692	2.071937	2.080361	1.995507
FLJ35785 //	1556739_at	2.433797	2.346934	2.329128	2.15662	2.354878	2.33123	2.400299	2.673193	2.341933	2.330362	2.550344	2.320104	2.546361
LOC728411	1556742_at	3.740152	3.79347	3.699894	3.746122	3.874758	3.703172	3.715403	3.719056	3.715369	3.533289	3.601162	3.714808	3.540507
ZNF654	1556743_at	4.206578	3.697258	3.89028	3.993248	4.150349	3.967133	3.774429	3.869908	3.975689	3.876307	4.067085	3.81356	4.025449
ZNF654	1556744_a_at	3.336498	3.791099	3.705905	4.439219	3.542403	3.785807	3.527065	3.442461	3.510929	3.503553	3.501951	3.083473	4.087494
LOC153577	1556750_at	3.233448	3.256999	3.148302	3.142799	3.325218	3.182207	3.147773	3.195693	3.184316	3.168686	3.187995	3.196626	3.044312
LOC286149	1556755_s_at	2.416425	2.381739	2.369672	2.363233	2.404696	2.375847	2.372519	2.382277	2.36803	2.350346	2.364829	2.381003	2.245744
ALS2CR11	1556757_a_at	2.518635	2.45905	2.423704	2.433801	2.619093	2.433224	2.450653	2.458467	2.445296	2.429429	2.459171	2.529112	2.30262
C10orf18	1556758_at	2.942514	2.856448	2.83872	2.889323	2.925315	2.874033	2.898159	2.878214	2.906325	2.882148	2.922115	2.904599	2.978858
ERICH1	1556766_at	2.616537	2.51341	2.490601	2.50087	2.603699	2.484314	2.50833	2.50828	2.498437	2.502916	2.498894	2.505049	2.274519
FBXL13	1556770_a_at	3.294531	2.624041	2.486487	2.612979	2.728536	2.59126	2.914809	2.841266	2.663791	3.005256	3.839079	3.277699	6.358831
BC038740	1556771_a_at	2.817848	2.785227	2.714943	2.759563	2.831179	2.761115	2.790158	2.787142	2.766572	2.747189	2.802316	2.79491	2.547129
FAM83C	1556793_a_at	3.063514	2.976501	2.959579	2.971047	3.0263	2.968827	2.97679	2.994586	2.968699	2.959966	2.970184	2.97827	2.92698
LOC386597	1556797_at	1.645108	1.636994	1.620791	1.623417	1.630277	1.626647	1.636205	1.634133	1.629006	1.619528	1.620264	1.627347	1.632878
LOC386597	1556798_a_at	2.246679	2.213896	2.19859	2.204198	2.266397	2.197445	2.218647	2.219524	2.206562	2.197488	2.202973	2.215923	2.107214
LOC400794	1556801_at	2.536535	2.456812	2.41488	2.4338	2.500133	2.899929	2.453581	2.458774	2.369815	2.436774	2.546373	2.489108	2.301048
POLR3B	1556803_at	4.058706	3.863296	3.586382	3.852773	4.236607	3.830743	3.758398	3.709971	3.854736	3.749692	3.940187	4.100477	3.370318
POLR3B	1556804_s_at	4.194288	3.840408	3.574804	3.861618	4.104882	3.749894	3.542797	3.648161	3.769616	3.675708	3.758618	3.996342	3.127256
LOC121906	1556808_at	2.028708	2.010885	1.99154	1.995389	2.013059	1.99125	2.007365	2.009288	1.997585	1.994099	1.994743	2.005259	1.967546
LOC121906	1556809_a_at	3.055626	2.930842	2.851725	2.928365	2.98274	2.894064	3.174034	2.99287	3.599548	2.974048	2.970844	2.979107	2.980344
DLEU2	1556820_a_at	8.30131	8.422069	7.15756	7.49259	8.125194	7.978348	8.954843	9.923171	9.132183	9.363674	9.360221	9.482313	7.857463
DLEU2	1556821_x_at	8.299257	8.357966	7.488893	7.458937	7.751802	7.600746	9.809738	9.673825	9.950561	10.11429	9.051019	9.423954	7.987336
LOC116412	1556822_s_at	1.743977	1.733918	1.716116	1.721854	1.732437	1.71936	1.730693	1.731358	1.727861	1.718359	1.712893	1.726064	1.711476
C1orf187	1556826_s_at	2.75253	2.736586	2.706227	2.718562	2.744575	2.796192	2.726614	2.733177	2.720685	2.712595	2.723988	2.731819	3.860016
LOC339929	1556827_at	2.692768	2.654124	2.62515	2.641953	2.678363	2.622033	2.645274	2.658695	2.644347	2.627117	2.642295	2.655671	2.497607
TIPARP	1556829_at	2.705033	2.653001	2.638455	2.641832	2.687241	2.604337	2.648588	2.652253	2.643765	2.635043	2.639517	2.668834	2.454201
DYNC1H1	1556831_at	4.354704	4.245677	3.824078	4.10922	4.305464	4.385754	4.020887	4.245084	4.158378	4.013474	4.212794	4.441751	3.881414
SPTBN5	1556839_s_at	3.076857	3.055535	2.969725	3.001353	3.117471	3.037605	3.043328	3.012149	3.040135	3.035156	3.012449	3.049906	2.748891
ALDH1L2	1556841_a_at	2.041925	2.021354	2.000944	2.005092	2.025328	2.008469	2.016867	2.018475	2.00762	2.004688	2.00274	2.011999	1.967801
LOC286087	1556842_at	2.843862	2.772022	2.719529	2.762129	2.818315	2.725227	2.775181	2.787221	2.770036	2.764216	2.791633	2.807421	2.644526
CEP290	1556850_at	1.92603	1.910398	1.889273	1.892908	1.909541	1.891486	1.904631	1.906714	1.895132	1.88962	1.893312	1.90107	1.844997
LOC283501	1556854_at	2.057836	2.044132	2.022309	2.029243	2.042812	2.026442	2.039055	2.041389	2.029699	2.022252	2.028346	2.036801	1.977326
LOC283501	1556855_a_at	2.121009	2.11122	2.092732	2.092154	2.104637	2.093618	2.109882	2.110446	2.099113	2.090013	2.092827	2.101648	2.04588
LOC285740	1556859_a_at	1.910548	1.901583	1.877925	1.883778	1.900296	1.889527	1.89193	1.893854	1.882978	1.876523	1.884034	1.891749	1.832407
TCTN1	1556864_at	3.989884	3.565886	3.598974	3.602085	3.980589	3.630946	3.556465	3.614946	3.582938	3.601777	3.62133	3.607452	3.570377

IQSEC3	1556872_s_at	2.039081	2.021405	1.995734	2.004518	2.023006	2.002594	2.012623	2.024541	2.011923	1.997748	2.014456	2.060383	1.926098
MEX3C	1556873_at	3.094283	2.967091	2.923902	2.968599	3.111703	2.939484	2.961623	2.982502	2.966699	2.945527	3.005333	3.055767	2.676488
MEX3C	1556874_a_at	2.148958	2.129856	2.10594	2.112743	2.138313	2.1145	2.123098	2.125284	2.114643	2.107174	2.11369	2.119359	2.047399
TPTEps1	1556876_s_at	2.706314	2.644895	2.566974	2.636351	2.696311	2.64206	2.646688	2.642437	2.642788	2.62348	2.723339	2.663595	2.502812
LOC729967	1556881_at	2.467423	2.439771	2.398712	2.420305	2.455707	2.402389	2.425847	2.435695	2.423092	2.409331	2.421038	2.433571	2.332545
LOC440896	1556883_a_at	2.415201	2.385504	2.347084	2.365782	2.401959	2.357221	2.374093	2.385926	2.370596	2.357469	2.371655	2.380293	2.245928
LAYN	1556885_at	2.50671	2.479066	2.446056	2.462519	2.564958	2.454551	2.475078	2.472	2.465331	2.458159	2.466391	2.475154	2.315251
LAYN	1556886_a_at	2.366613	2.324167	2.284008	2.303444	2.332988	2.303813	2.311681	2.32366	2.308818	2.298772	2.307764	2.317869	2.26361
LOC348808	1556887_at	2.001109	1.986519	1.965488	1.969264	1.980976	1.968566	1.981373	1.98423	1.979033	1.969829	1.965885	1.978114	1.939656
SORCS1	1556891_at	2.006734	1.983221	1.964703	1.967945	2.034688	1.969114	1.979264	1.980894	1.969844	1.967601	1.969524	2.006652	1.911392
SNHG8	1556894_at	3.73686	3.428626	3.467726	3.48727	3.860033	3.579589	3.480041	3.493799	3.526963	3.464196	3.618851	3.712768	3.018199
SNHG8	1556895_a_at	2.22242	2.207183	2.186924	2.190875	2.211272	2.189218	2.200275	2.204348	2.192025	2.186678	2.187885	2.19833	2.230193
LOC200830	1556898_at	3.81289	4.073357	3.756042	3.75305	3.836066	3.647391	3.597629	3.730038	3.656155	3.676445	3.728807	3.723627	3.417996
LOC149773	1556900_at	2.248959	2.241452	2.208267	2.224326	2.236065	2.508088	2.236354	2.238381	2.219876	2.224438	2.220744	2.228405	2.00754
LOC149773	1556901_s_at	2.669823	2.605176	2.575845	2.574593	2.615437	2.584094	2.600734	2.590445	2.5923	2.584004	2.594262	2.60194	2.344931
ZNF577	1556905_at	2.587459	2.494061	2.478096	2.486693	2.509082	2.485217	2.495491	2.64274	2.487221	2.481132	2.490606	2.713721	2.342535
ZNF474	1556907_at	2.611198	2.58134	2.538125	2.556925	2.592985	2.548841	2.566922	2.578875	2.563747	2.552154	2.559313	2.574253	2.696942
LEPR	1556919_at	7.301994	7.323209	7.839555	8.195634	4.900183	4.678546	3.639624	3.669777	4.669543	4.026353	2.41789	2.543298	1.832561
LOC646168	1556920_s_at	2.436712	2.417592	2.400615	2.404485	2.421738	2.375864	2.414995	2.418728	2.406037	2.392065	2.400193	2.415672	2.352949
ALS2CR10	1556924_at	2.274566	2.254625	2.224145	2.231998	2.264807	2.225243	2.242368	2.254554	2.239184	2.226577	2.238128	2.246395	2.140085
SMC3	1556925_at	4.556051	4.34676	4.156625	4.393213	4.480844	4.447461	5.213971	5.17275	5.627106	5.07596	5.338182	5.20383	4.031518
PAPOLG	1556929_at	3.011817	2.988595	2.951381	2.987543	3.12162	2.986014	3.00224	2.986894	2.997912	2.902135	2.99499	3.075465	2.860698
LOC283484	1556940_at	2.964114	2.9066	2.862763	2.879075	2.947646	2.873959	2.899008	2.911857	2.897507	2.88324	2.925715	2.92342	2.760622
LOC283484	1556941_a_at	2.760667	2.674124	2.653038	2.691916	2.746317	2.636408	2.704203	2.708062	2.701349	2.671296	2.807433	2.724519	2.637841
SERPINB6	1556950_s_at	2.576346	2.342695	2.333965	2.335782	2.978418	3.456897	2.315187	2.342288	2.314592	2.318668	2.309485	2.339645	2.240343
LOC283854	1556954_at	3.233102	3.102443	3.085495	3.109195	3.211705	3.006527	3.097869	3.080573	3.113899	3.077374	3.116281	3.184608	2.872069
hCG_20073	1556959_at	4.053189	4.040953	3.607964	3.84768	4.038935	4.034946	4.179689	4.039779	4.04466	4.034184	4.053109	4.109355	4.036746
hCG_20073	1556960_a_at	2.328673	2.294419	2.266867	2.275942	2.315368	2.269836	2.285743	2.294411	2.28203	2.269962	2.278201	2.308393	2.175628
ZDHHC14	1556967_at	2.353434	2.324593	2.309476	2.315338	2.337437	2.311671	2.33641	2.325015	2.321476	2.301755	2.312067	2.32678	2.203714
CCDC108	1556969_at	2.09822	2.090104	2.053363	2.065298	2.094343	2.068657	2.080557	2.081568	2.079039	2.065151	2.066199	2.072607	1.97156
TAF8	1556978_a_at	2.747033	3.06435	2.685064	2.677968	2.736049	2.792244	2.687804	2.697729	2.670782	2.661725	2.682493	2.695439	2.506557
OR2H1	1556986_at	2.011191	1.990545	1.966397	1.972738	1.991616	1.971638	1.982799	1.982065	1.972344	1.968173	1.968686	1.982116	1.924711
OR2H1	1556987_s_at	1.879694	1.874341	1.854862	1.856389	1.931098	1.857567	1.869068	1.867176	1.863953	1.852248	1.848328	1.860624	1.841848
CHD1L	1556988_s_at	9.739476	9.786337	8.354181	8.550894	7.971618	8.065456	9.046155	9.514652	8.863779	8.962075	9.208264	9.540832	7.646651
PERP	1556990_at	2.866397	2.76685	2.705208	2.764177	2.937352	2.725374	2.772278	2.782203	2.754681	2.749211	2.772727	2.807649	2.56569
LOC550113	1556992_at	2.295764	2.272463	2.239949	2.252636	2.27814	2.337149	2.260668	2.271908	2.259068	2.255155	2.257481	2.265811	2.16774
LOC285045	1556999_at	2.213458	2.20171	2.168207	2.216766	2.200101	2.176453	2.195743	2.197495	2.185335	2.180433	2.182822	2.192014	2.1401
ESPNL	1557000_at	2.262167	2.249656	2.238779	2.232175	2.246775	2.242499	2.247317	2.685983	2.249471	2.231821	2.23536	2.23996	2.221027

ESPNL	1557002_x_at	2.293526	2.267938	2.227812	2.253294	2.266508	2.257236	2.261214	2.263618	2.262181	2.238219	2.249772	2.258458	2.250384
FLJ25439	1557003_at	2.306141	2.293183	2.26531	2.273923	2.316554	2.288637	2.282575	2.286757	2.275985	2.27041	2.270564	2.286436	2.233914
LOC285389	1557004_at	2.378947	2.349306	2.314864	2.330065	2.362246	2.317979	2.336028	2.34812	2.333572	2.323868	2.332423	2.341883	2.236981
LOC340107	1557008_at	2.71312	3.075676	2.638401	2.680627	2.735295	2.730398	2.691002	2.704369	2.687933	2.667267	2.689742	2.707065	2.517364
LOC340107	1557009_a_at	2.601278	2.563978	2.506746	2.546598	2.619479	2.533112	2.55272	2.563217	2.551272	2.524563	2.553208	2.588745	2.371234
C9orf122	1557014_a_at	2.70406	2.654626	2.638218	2.63605	2.713503	2.609467	2.64629	2.653128	2.643376	2.620697	2.639912	2.669818	2.458116
C1orf177	1557016_a_at	2.359295	2.321268	2.281681	2.346881	2.323222	2.30501	2.313521	2.320629	2.310358	2.293639	2.303549	2.316536	2.188685
LOC651721	1557027_at	2.22933	2.196128	2.163837	2.176887	2.212098	2.173848	2.178835	2.190663	2.175869	2.161944	2.176141	2.188897	2.096948
GAB1	1557030_at	3.455598	3.243048	3.238553	3.186849	3.445722	3.188295	3.547491	3.691133	3.735393	3.385005	3.415985	4.256016	2.88288
RNF212	1557031_at	2.261019	2.239536	2.217975	2.222783	2.244515	2.219843	2.231637	2.224623	2.225307	2.22161	2.224393	2.23442	2.115094
CXYorf1	1557034_s_at	1.956185	1.941669	1.922983	1.928686	1.942678	1.927578	2.321727	1.943194	1.955474	1.942694	1.928751	2.067068	1.896001
ZBTB1	1557036_at	5.431928	4.821147	4.65651	4.668884	4.745325	3.97615	4.719369	4.080883	4.574617	4.375062	4.70685	4.56961	4.402926
LOC728213	1557037_a_at	3.243639	3.157708	3.140438	3.178881	3.249324	3.223301	3.189399	3.195607	3.186647	3.104081	3.189148	3.224748	2.80625
LOC728213	1557038_s_at	2.556299	2.526156	2.517253	2.44924	2.535384	2.506172	2.54039	2.538696	2.535903	2.661045	2.831584	2.539472	2.383764
YEATS2	1557047_at	2.913397	2.900652	2.80828	2.845728	2.900249	2.885218	3.234766	2.90253	2.901761	2.889842	2.890601	2.977523	2.812543
LOC149478	1557049_at	3.6203	3.729649	5.681157	5.021721	3.70136	3.030348	2.759712	2.723519	2.731006	3.685443	2.927431	2.727907	3.602848
HOXA2	1557051_s_at	3.769117	3.747926	3.690145	3.736193	4.177814	3.746061	3.756573	3.757442	3.929628	3.725219	4.721041	3.75159	5.543833
UBE2G2	1557053_s_at	7.504511	7.415427	7.135053	7.494058	7.834245	8.006499	7.035319	8.013916	7.122708	7.372202	8.055988	7.737948	6.522142
LOC643837	1557055_s_at	7.141074	7.237681	7.513595	7.706544	7.028875	6.773387	8.118719	8.068663	8.430888	8.215458	7.553652	7.918601	5.754098
LOC133491	1557056_at	1.913847	1.880675	1.862414	1.866345	1.898325	1.86526	1.876778	1.880536	1.868051	1.864207	1.866695	1.876188	1.820712
LOC133491	1557057_a_at	3.092115	2.962622	2.855803	2.959435	3.107026	2.946842	2.949099	2.954121	2.960465	2.941033	3.006085	3.061066	2.66826
LOC254128	1557059_at	2.345227	2.33557	2.268511	2.314765	2.331714	2.323273	2.334553	2.335722	2.332232	2.321786	2.322424	2.329239	2.315624
LOC148756	1557060_at	2.276739	2.249618	2.232175	2.235389	2.248354	2.239849	2.237902	2.24821	2.231892	2.217979	2.230151	2.245852	2.222154
FLJ45244	1557063_at	2.665639	2.630935	2.612071	2.616725	2.664549	2.615892	2.629136	2.636866	2.625129	2.61942	2.615106	2.621323	2.551037
HGSNAT	1557064_s_at	5.482389	5.107803	4.555812	4.550465	5.125101	4.930229	3.674697	4.562262	4.265736	4.464606	4.536561	4.538381	4.196695
YLPM1	1557065_at	2.576937	2.534003	2.444799	2.527622	2.547184	3.110065	2.658206	2.743467	2.563883	2.545981	3.445769	3.549888	3.432965
LUC7L	1557066_at	4.20549	3.771312	3.528795	3.799618	3.926365	3.428799	3.744526	3.489643	3.753208	3.618877	3.734755	3.799672	4.56257
LUC7L	1557067_s_at	5.091375	5.038908	4.536447	4.214295	4.231386	3.971739	6.288462	4.912959	6.475838	6.291842	5.222946	5.389992	7.858832
KRT37	1557068_at	2.633164	2.594595	2.558673	2.575718	2.621961	2.552703	2.582329	2.591803	2.576315	2.561353	2.580961	2.597854	2.41032
NUB1	1557071_s_at	3.50229	3.217134	3.861219	3.872474	3.583906	3.106598	3.149124	3.04093	3.156002	3.118508	3.14954	3.204453	2.705904
TTBK2	1557073_s_at	4.983152	4.844246	4.882792	5.098878	4.770445	4.794233	4.911404	4.833002	5.123835	4.560085	5.442898	4.691388	6.14043
SLFN5	1557078_at	2.479108	2.440237	2.677255	2.425916	2.465868	2.411456	2.425818	2.436857	2.424502	2.411666	2.417726	2.433841	2.488853
ITGBL1	1557079_at	2.806364	2.744689	2.672931	2.729136	2.824883	2.735363	2.738564	2.747502	2.737284	2.709742	2.749219	2.768255	2.65774
ITGBL1	1557080_s_at	2.334816	2.325094	3.70518	2.589622	3.841266	2.976986	2.263659	2.247065	2.254179	2.239919	2.247054	2.264438	4.239978
RBM25	1557081_at	7.160192	7.051462	7.327991	7.100773	6.936847	6.212815	6.67064	6.302664	6.811974	6.632872	6.563007	6.206484	7.528067
PLAC1L	1557085_at	2.893375	2.817806	2.748899	2.808642	2.900532	2.785899	2.823988	2.826827	2.81703	2.795428	2.828048	2.855018	2.641577
KIF5C	1557089_at	1.996013	1.991348	1.968795	1.974196	2.001027	1.974683	1.986873	1.983553	1.977975	1.972366	1.969026	1.978241	2.053809
FLJ36070	1557091_at	3.273383	3.475027	2.82545	2.845202	3.395066	3.258752	3.266881	3.260052	3.232556	3.250704	3.318023	3.244372	4.210445

LOC653110	1557094_at	3.814675	3.65168	3.577928	3.629449	3.986547	3.435305	3.563461	3.540721	3.639983	3.510831	4.196488	3.82108	3.200323
HAR1A	1557098_s_at	2.961804	2.919809	2.901667	2.90507	2.948987	2.913078	2.914988	2.919177	2.919572	2.894486	2.888492	2.916496	2.881215
HECTD1	1557100_s_at	4.737886	3.743315	5.910384	5.348927	4.256162	3.669428	5.441388	3.397863	5.00603	4.61777	3.862168	3.746794	5.358001
LMTK3	1557103_a_at	2.605664	2.685041	2.519402	2.54988	2.770347	2.795005	2.539579	2.560562	2.316132	2.540401	2.56899	2.517966	2.325531
LOC286002	1557107_at	2.800702	2.758018	2.668419	2.720394	2.815106	2.730431	2.733658	2.74209	2.744355	2.732126	2.744133	2.795446	2.516786
LOC283588	1557113_at	3.165811	3.060047	3.058953	3.040893	3.098602	3.063769	3.061654	3.064709	3.052792	3.039607	3.054886	3.055226	4.604508
LOC284385	1557114_a_at	2.548335	2.527279	2.829257	2.524609	2.537255	2.526253	2.509253	2.512845	2.529273	2.531125	2.495943	2.520346	2.614984
APOL6	1557116_at	7.946351	7.839378	8.026219	7.976833	6.939293	6.626648	3.410058	3.82991	3.825393	3.837935	3.932145	3.248688	4.718381
ZNF575	1557119_a_at	3.590747	3.586021	3.566398	3.569959	4.596089	4.468391	2.765458	3.180698	3.50157	3.569178	3.564636	3.456385	4.522281
EEF1A1	1557120_at	2.778834	2.876849	2.714418	2.74111	2.777028	2.740406	2.754311	2.766077	2.749797	2.738431	2.751906	2.755771	2.743545
GABRB2	1557122_s_at	2.859399	2.765612	2.635405	2.697632	2.861791	2.744698	2.709973	2.729579	2.742524	2.683079	3.389231	2.902492	4.348852
RP4-756G2	1557123_a_at	2.637818	2.573593	2.529618	2.556848	2.564047	2.555656	2.533514	2.557465	2.652978	2.552988	2.550595	2.646159	3.242726
LOC440104	1557124_at	3.555379	3.802133	3.327276	3.230345	3.685884	3.418858	3.333238	3.432413	3.365065	3.406276	3.180668	3.426103	3.368498
PLD1	1557126_a_at	3.362916	4.089529	4.174373	3.211557	2.760373	2.637834	2.732186	2.744117	2.749559	2.610299	2.678625	2.732806	2.583098
FAM111B	1557128_at	2.479339	2.45132	2.396918	2.432249	2.478936	2.425842	2.445107	2.445489	2.438615	2.431961	2.431419	2.445317	2.396809
FAM111B	1557129_a_at	3.309848	3.152288	3.072157	3.116638	3.219032	3.128412	3.149409	3.612012	3.154419	3.12422	3.612196	3.715618	2.933079
LOC254100	1557131_at	2.004131	1.995296	1.972989	1.981985	2.00525	1.979493	1.994244	1.992665	1.982317	1.981591	1.977898	1.986769	1.943263
WDR17	1557132_at	3.245043	3.17777	3.054954	3.145157	3.325488	3.327062	3.135921	3.148546	3.031014	3.118412	3.171066	3.208575	4.033879
LOC286411	1557133_at	2.449584	2.414911	2.37627	2.402598	2.43305	2.387678	2.410321	2.416308	2.403369	2.397014	2.400012	2.413187	4.360298
ATP13A4	1557136_at	3.029527	3.007641	2.920638	2.995405	3.113917	2.991516	3.003476	3.063765	2.989458	2.983973	2.980153	3.011892	2.909165
TMEM17	1557137_at	5.066665	4.567225	4.570645	5.170765	4.987071	4.563252	5.120167	4.691111	4.681182	4.498652	4.588388	4.711115	5.709621
FLJ22374	1557141_at	2.363075	2.354992	2.338082	2.341415	2.351113	2.34383	2.36982	2.513549	2.988519	2.647016	2.363996	2.358409	2.41031
CSMD2	1557143_at	3.087677	3.378134	2.762895	2.951849	3.365037	3.080476	2.803613	2.937844	2.856661	2.899596	2.956156	2.96045	2.801866
STK38	1557145_at	5.090589	4.526519	4.202472	4.562291	6.049697	4.550135	4.282591	4.635464	4.419923	4.377631	4.825642	4.788355	4.748816
FLJ32252	1557146_a_at	2.110661	2.102059	2.082461	2.081988	2.097078	2.085831	2.097805	2.265993	2.095181	2.083881	2.086243	2.092617	2.010535
HM13	1557148_at	2.345065	2.31613	2.27993	2.292985	2.317662	2.298565	2.300671	2.311035	2.297335	2.286267	2.29743	2.305028	2.188008
TRIM31	1557151_at	3.841754	3.564597	3.444793	3.646764	3.801197	3.508564	3.639364	3.571212	3.667359	3.579785	3.660075	3.856575	3.089565
ZIC1	1557154_at	3.79496	3.604115	3.7196	3.756097	3.936028	3.769515	3.702065	3.770558	3.740458	3.734736	3.804425	3.789995	3.136424
ZIC1	1557155_a_at	2.592094	2.772814	4.149395	3.630038	6.045009	6.419461	2.550715	2.648898	2.634309	2.605096	2.374081	2.438289	2.920143
MLL3	1557158_s_at	2.89986	2.839843	2.695512	2.825361	2.888972	2.842512	2.847943	2.84496	2.834424	2.835865	2.833491	2.839149	3.281855
FLJ25404	1557162_at	2.820212	2.785266	2.726475	2.735548	2.806499	2.75669	2.775744	2.785754	2.7774	2.761233	2.772912	2.789588	2.611306
KLHL18	1557165_s_at	1.383033	1.377782	1.357269	1.36165	1.367153	1.363631	1.375954	1.36145	1.592333	1.363843	1.811097	1.448214	1.355706
PDCD4	1557166_at	3.080777	2.980598	2.903737	3.023464	3.279284	3.027217	2.993525	2.999751	3.024814	3.024754	2.988203	3.074219	3.080595
HCG11	1557167_at	2.234662	2.210942	2.193932	2.206134	2.233824	2.187194	2.626134	2.279374	2.555251	2.51453	2.797599	2.436851	4.639915
HCG11	1557169_x_at	2.219885	2.196617	2.165378	2.179202	2.201475	2.172941	2.19094	2.197823	2.670143	2.52593	2.208502	2.275441	4.7149
NEK8	1557170_at	4.434141	4.608563	3.869882	4.122351	4.877097	5.45285	4.066707	4.804783	4.219116	4.041899	5.287476	4.92085	3.625173
NEK8	1557172_x_at	4.612974	4.397962	3.850678	3.922679	4.521237	4.725546	4.075932	4.647082	4.000491	3.863504	5.219352	4.53655	3.832474
C14orf37	1557176_a_at	3.025126	2.985087	2.874215	3.005035	3.112361	2.895697	3.003766	3.105225	3.005048	2.991435	3.502674	3.556309	6.576015

ING1	1557179_s_at	2.468884	2.457119	2.439282	2.443718	2.457062	2.441321	2.453883	2.452654	2.437756	2.440868	2.43693	2.448562	2.403286
LOC399947	1557180_at	3.269468	3.131236	3.041438	3.161894	3.294654	3.063047	3.160462	3.154982	3.170427	3.148801	3.194097	3.239162	3.554102
LOC399947	1557181_s_at	3.104802	2.990288	2.918631	2.983779	3.095739	2.946675	3.015536	3.082401	3.006569	2.966381	3.049275	3.082558	6.083116
TPCN1	1557185_at	3.972697	3.921157	3.832981	4.095374	3.981624	3.917989	3.929875	3.939252	3.912921	3.897355	3.911796	3.929484	3.459846
TPCN1	1557186_s_at	2.825779	3.006083	3.036786	3.72364	3.127692	3.359798	3.146526	3.190202	2.943745	3.040428	3.112441	3.18673	2.306217
DNASE1	1557189_at	2.852952	2.760984	2.692348	2.743697	2.852429	3.060734	2.729289	2.746545	2.745136	2.72415	2.778729	3.52595	2.278557
C1orf136	1557192_at	2.673232	2.653421	2.633955	2.638348	2.675935	2.639644	2.65998	3.020367	2.783999	2.729223	2.641382	2.6534	3.984616
LGALS3	1557197_a_at	3.102264	3.085194	3.000823	3.06674	3.085687	3.072486	2.959623	3.083298	3.043623	2.95072	3.05562	3.077755	3.085932
RBM32B	1557203_at	2.373667	2.306138	2.30238	2.309605	2.324032	2.229116	2.331256	2.343348	2.426481	2.397326	2.387385	2.408297	3.811221
FLJ35848	1557206_at	3.422265	3.235963	3.26659	3.282418	3.510399	3.269069	3.309969	3.32008	3.302613	3.264305	3.39875	3.449922	3.106143
LOC283177	1557207_s_at	3.922688	3.568084	4.174159	4.338615	4.306291	4.485661	3.460976	3.379737	3.428092	3.548782	3.40105	3.404004	2.650865
LOC219731	1557208_at	2.486713	2.459956	2.407775	2.437748	2.470128	2.421384	2.447393	2.455549	2.443503	2.436981	2.439675	2.454375	2.305862
C14orf86	1557211_a_at	2.449234	2.426742	2.377892	2.381495	2.417266	2.383641	2.391538	2.397749	2.383169	2.385218	2.378399	2.394213	2.277122
FLJ33065	1557212_at	2.657084	2.621558	2.60701	2.60194	2.618294	2.593389	2.610296	2.925011	2.609092	2.59497	2.603936	2.612399	2.356223
FANCB	1557217_a_at	2.943306	2.89994	2.804458	2.865317	2.949522	2.826147	2.980831	2.892105	3.172085	2.933704	2.886203	2.909121	2.614964
FANCB	1557218_s_at	2.862195	2.733077	2.743201	2.754789	2.801773	2.756608	2.790889	2.790126	2.779282	2.773869	2.862308	2.967722	2.675688
SDHALP2	1557223_at	2.331396	2.316947	2.300039	2.304234	2.324397	2.308088	2.314531	2.311736	2.307105	2.284668	2.299533	2.30557	2.242936
LOC374569	1557225_at	2.56277	2.576943	2.765449	3.12831	2.567502	2.523109	2.520213	2.543746	2.52731	2.512178	2.526843	2.535524	1.971791
LOC374569	1557226_a_at	4.504556	4.555717	5.529515	5.731234	4.253332	3.817969	3.679391	3.878417	3.770521	3.934647	3.312477	3.24512	1.990613
TPR	1557227_s_at	3.804585	3.524554	3.785641	3.715952	3.833184	3.815646	5.135701	3.590531	4.655003	4.148605	3.899098	4.244543	6.351612
EHBP1L1	1557228_at	2.786599	2.775357	2.675326	2.763141	2.769704	2.762938	2.778798	2.763697	3.17635	3.275238	2.757986	2.770948	2.811748
LOC285441	1557233_at	2.818252	2.767775	2.723871	2.769842	2.825404	2.758227	2.77752	2.782989	2.772957	2.745479	2.774713	2.809696	2.658229
APOL6	1557236_at	6.167646	5.693731	5.917672	5.753289	5.911062	5.979111	3.404004	3.398684	3.446887	3.425335	3.526138	3.580309	2.964298
KIDINS220	1557246_at	2.813083	2.754915	3.041207	3.005525	2.747729	2.630426	3.227783	2.706541	3.649815	3.884033	2.731284	2.711049	3.798805
ZNF587	1557248_at	3.209674	2.974167	2.687763	2.719026	2.857061	2.502315	2.801886	2.545743	3.20358	2.725377	3.500836	2.743197	3.766695
BCL10	1557257_at	2.542446	2.502499	2.45577	2.483194	2.530081	2.471483	2.493333	2.505167	2.49094	2.475753	2.489953	2.506496	2.352215
BCL10	1557258_a_at	2.181771	2.164352	2.144809	2.152531	2.165218	2.152045	2.16351	2.162765	2.148923	2.14065	2.145863	2.156696	2.672941
ZNF382	1557260_a_at	2.844368	3.340549	2.670721	2.658319	2.768105	2.690643	2.754903	2.765454	2.752342	2.945026	2.757691	3.309189	3.524358
WHDC1L2	1557261_at	3.128516	3.110685	3.23849	3.13749	3.157636	3.329464	3.129369	3.407223	3.247042	3.082956	3.247397	3.836181	3.061556
LOC284952	1557267_s_at	3.035539	2.959086	2.880926	2.950335	3.024432	2.921914	3.048422	2.924666	2.976979	2.931404	3.034154	3.001307	3.895845
TNPO1	1557278_s_at	3.233623	3.338627	3.904939	4.256503	3.789497	3.572665	3.343973	3.406469	3.400832	3.029868	4.705139	3.477699	3.388428
ZNF519	1557283_a_at	2.998096	2.981731	2.796254	2.910454	3.11919	3.046074	3.073702	3.53262	2.934071	2.897357	2.974228	4.848039	3.805702
LOC727738	1557285_at	3.48953	3.252457	3.192664	3.335307	3.527491	3.269018	3.308667	3.305454	3.371017	3.370929	3.489854	3.68192	2.96566
GTF2IRD2	1557289_s_at	5.649923	5.88337	5.566554	5.563595	5.91587	6.847316	3.836115	3.727887	3.563878	3.050057	4.662281	3.788081	4.174248
DPY19L2	1557290_at	2.280168	2.266595	2.231495	2.249689	2.266419	2.249084	2.261153	2.267837	2.253636	2.244695	2.2507	2.271844	2.198866
MCOLN3	1557292_a_at	2.383331	2.351101	2.238266	2.327549	2.360202	2.326815	2.335721	2.361716	2.343992	2.41345	2.339186	2.393655	2.229291
LOC440993	1557293_at	8.577369	8.66008	8.162178	8.420454	7.992098	8.079959	5.570701	6.909514	7.169379	7.09638	7.132815	7.422264	5.179886
FLJ12825	1557296_at	2.06442	2.050023	2.025994	2.030936	2.050886	2.034683	2.041307	2.04294	2.031617	2.025847	2.028569	2.036843	1.96675

NT5C	1557303_at	2.175535	2.169661	2.148557	2.145011	2.16235	2.146845	2.174133	2.15829	2.424568	2.15282	2.14494	2.156909	2.346832
TACC1	1557305_at	3.532205	3.441566	3.3814	3.430396	3.617538	3.449655	3.444771	3.457635	3.444951	3.420206	3.52328	4.107376	3.219806
DENND1B	1557309_at	2.971038	2.916934	2.841713	2.880941	2.946796	2.795024	2.8914	2.899519	2.890549	2.855006	2.895618	2.92872	2.739085
C12orf61	1557312_at	2.7103	2.681326	2.654382	2.653397	2.74054	2.665494	2.671938	2.674943	2.670597	2.659344	2.660708	2.688277	2.477341
CAPN14	1557321_a_at	3.140295	3.017667	2.948079	3.00411	3.225267	2.967645	3.024655	3.026583	3.026633	2.993426	3.051217	3.093608	2.840952
ZNF230	1557322_at	2.549211	3.02156	2.508889	2.517076	2.718876	2.507053	2.519501	2.535023	2.875305	2.504148	2.522631	2.522697	2.897642
LOC283665	1557328_at	2.916951	2.845152	2.779142	2.838444	2.946552	2.835384	2.853221	2.859883	2.860804	2.821738	2.891515	2.897662	2.598856
POLR1B	1557331_at	4.123465	3.7463	4.382545	4.132736	3.780348	3.639459	4.869394	4.446937	4.650271	4.456452	4.12304	4.252445	4.904313
C20orf42	1557334_a_at	2.453379	2.421273	2.386864	2.403746	2.441511	2.39696	2.412875	2.423798	2.40873	2.397558	2.407563	2.429622	2.294992
LOC150381	1557342_a_at	2.517217	2.49768	2.484989	2.483012	2.523873	2.489111	2.493348	2.504441	2.49199	2.487693	2.485128	2.512383	2.346862
LOC283516	1557345_at	2.274684	2.245521	2.218121	2.227498	2.256409	2.221134	2.235329	2.24515	2.230373	2.218504	2.230732	2.239692	2.168048
MCPH1	1557347_at	2.897957	2.593778	2.613598	2.793278	2.59501	2.575717	2.60548	2.591376	2.688887	2.570142	2.576929	2.603296	2.435926
RERE	1557349_at	2.568977	2.522808	2.475097	2.510336	2.643511	2.50709	2.522842	2.534316	2.518692	2.499562	2.521174	2.559159	2.424369
G3BP1	1557350_at	4.219252	4.395901	4.730721	4.942237	4.663918	4.305593	4.731784	4.742724	4.68238	4.710894	4.80614	4.746645	4.066125
SQLE	1557352_at	3.208675	3.158125	3.270402	2.72986	5.313334	4.588097	5.970032	5.992119	6.769797	6.065087	5.4269	5.998503	4.82176
SOS1	1557354_at	2.701652	2.642148	2.578029	2.63308	2.684634	2.609611	2.638667	2.650299	2.634446	2.613438	2.635972	2.659605	2.56722
LOC440944	1557357_at	4.374774	3.977894	3.913587	3.918537	4.213444	4.745334	3.966333	4.13594	3.958833	3.933776	3.973765	4.251205	3.696638
LOC285758	1557359_at	5.525897	5.769412	3.979409	4.213331	5.113022	4.755383	3.333922	3.090077	3.426477	3.20271	3.328517	2.907962	2.802766
LRPPRC	1557360_at	6.287365	6.115726	6.173392	5.698992	6.361219	5.963688	6.217647	5.814734	6.223964	5.961715	6.124934	5.9669	5.926375
PHIP	1557362_at	2.076462	2.06132	2.026435	2.042906	2.059897	2.035803	2.057319	2.059962	2.046677	2.040951	2.048174	2.056256	1.994664
PHIP	1557363_a_at	2.93964	2.838738	2.769615	2.831143	2.931918	2.793869	2.85856	2.857869	2.847847	2.832761	2.866504	2.892737	3.434824
CCDC144B	1557366_at	4.560958	4.267627	4.004209	4.238601	4.719219	4.296006	4.360294	4.340309	4.489195	4.253188	4.573045	4.666951	4.354377
LOC285401	1557369_a_at	2.524133	2.492032	2.470558	2.479818	2.492867	2.505743	2.479818	2.490935	2.47744	2.466028	2.472265	2.487147	2.342441
MYCBP2	1557370_s_at	4.357202	3.890408	5.903019	4.334244	4.538255	4.228053	5.667845	3.863875	5.559072	5.349554	4.092723	4.26133	7.859466
LOC158376	1557371_a_at	4.47922	4.434326	4.561126	4.39249	4.904979	4.41158	4.129187	4.293805	4.151479	4.174678	4.409212	4.558045	3.847752
FLJ41757	1557372_at	1.987993	1.970825	1.951162	1.956338	1.971698	1.946125	1.966737	1.968426	1.956652	1.949961	1.950363	1.962635	1.918726
LOC339505	1557373_at	2.075738	2.059568	2.037878	2.043901	2.061347	2.045324	2.0502	2.05404	2.043412	2.03535	2.039768	2.050359	1.973478
ABCC9	1557374_at	3.148081	3.269266	2.464597	2.46591	2.808636	2.70371	2.455579	2.462948	2.303123	2.425092	2.340147	2.450548	3.388299
KIAA1975	1557380_at	2.263287	2.238861	2.202669	2.215409	2.246086	2.211414	2.229098	2.237397	2.221647	2.215292	2.261594	2.235159	2.127476
KIAA1975	1557382_x_at	2.33545	2.307662	2.276549	2.281226	2.315837	2.306026	2.295594	2.302546	2.290578	2.283217	2.292005	2.319576	2.2171
ZNF131	1557384_at	7.086403	6.732809	7.3363	7.536536	7.363194	7.150553	6.994885	7.298236	7.516402	7.156615	7.153679	7.480197	6.399848
FLJ13305	1557385_at	2.562135	2.586977	2.511678	2.570394	2.586699	2.569407	2.579133	3.127776	2.571527	2.565236	3.007265	2.923963	4.631895
RTTN	1557388_at	1.940586	1.931907	1.938408	2.09502	2.288159	1.920226	2.335636	2.478608	1.919607	1.979035	1.914061	1.964945	2.262898
DLGAP4	1557394_at	4.692977	4.260464	4.002719	4.131092	4.198893	4.507101	3.45139	4.143939	3.757006	3.827105	4.541605	3.954973	4.167725
LOC255130	1557395_at	2.77451	2.70039	2.644347	2.698351	2.814742	2.664591	2.706375	2.716146	2.702087	2.679698	2.718919	2.764797	2.563245
DKFZP564C	1557398_at	2.677641	2.63363	2.58392	2.610607	2.66799	2.602618	2.625645	2.638101	2.619982	2.608189	2.623836	2.632976	2.456555
LOC283697	1557405_at	2.91432	2.859607	2.799309	2.843784	2.903349	2.852618	2.860171	2.874338	2.859645	2.852063	2.890462	2.905603	2.660086
LOC283697	1557406_s_at	2.137999	2.117889	2.113173	2.420876	2.117419	2.060064	2.105079	2.117535	2.095522	2.089797	2.109964	2.111557	2.031387

SLC25A43	1557411_s_at	3.714939	3.684457	5.191114	4.971809	3.665258	3.389868	4.344607	3.42981	4.265647	4.541372	3.280623	3.527477	4.301752
LETM2	1557415_s_at	2.284979	2.257809	2.249889	2.248327	2.275481	2.281827	2.248238	2.263757	2.262178	2.250624	2.253021	2.259773	2.998941
RSPH10B	1557417_s_at	2.636846	2.625568	2.607349	2.610831	3.116354	2.607019	2.528161	2.617927	2.608395	2.522482	2.598704	2.614664	2.429834
ACSL4	1557418_at	4.902173	5.031918	2.775454	2.804883	2.873779	2.794157	9.164886	8.411611	9.230829	9.003948	7.753615	8.131481	3.327998
ACSL4	1557419_a_at	6.259811	6.294132	4.451432	4.435779	4.987183	4.790137	9.286839	9.39735	9.085375	9.30839	8.948597	9.190688	4.489183
GALK2	1557429_s_at	2.972366	2.853665	2.784569	2.854197	3.020262	2.80593	2.861824	2.875402	2.859113	2.846349	2.88353	2.954642	2.657105
LOC147670	1557430_at	2.882362	2.818336	2.752663	2.82281	2.860132	2.824698	2.806872	2.982021	2.884632	2.820536	2.826184	2.83912	6.621712
RASAL2	1557432_at	5.33253	5.272495	5.838079	5.987269	5.32265	5.360014	5.464711	5.638955	5.210192	5.140963	6.627458	5.379959	5.000829
XKR6	1557436_at	2.343843	2.320502	2.293011	2.299825	2.325954	2.292006	2.314932	2.319824	2.306279	2.298838	2.304314	2.313724	2.243787
TREML3	1557444_at	2.893261	2.816195	2.755073	2.802543	2.881795	2.777283	2.822346	2.830644	2.821825	2.809209	2.820248	2.86929	2.597927
TREML3	1557446_x_at	2.88929	2.811387	2.751101	2.802194	2.877823	2.77109	2.817135	2.824763	2.813991	2.789425	2.820462	2.856527	2.572811
DGCR10	1557451_at	2.322777	2.301272	2.272562	2.280813	2.304428	2.27904	2.290143	2.29818	2.283555	2.270934	2.281571	2.295001	2.204936
MOSPD1	1557455_s_at	3.86276	4.151805	4.430685	4.435813	4.091849	4.12496	7.743589	7.341445	7.502727	7.724681	6.89279	7.220098	6.288169
SHB	1557458_s_at	8.307007	8.414301	7.747544	7.973383	7.845671	7.958934	5.077987	6.052221	5.282478	5.59719	5.915442	6.021146	6.037075
RP11-327P2	1557465_at	2.185405	2.163065	2.141594	2.145682	2.161978	2.143651	2.159238	2.15907	2.147467	2.141892	2.143302	2.157442	2.095249
FLJ31104	1557466_at	2.356726	2.338569	2.321027	2.318785	2.33771	2.313366	2.326329	2.335933	2.321983	2.309768	2.318476	2.329845	2.234959
CYP2C8	1557468_at	3.626754	3.419255	3.467987	3.493462	3.642752	3.437571	3.473383	3.475575	3.513748	3.447643	3.603921	3.682168	3.110593
SPATA13	1557470_at	2.128626	2.108965	2.09206	2.090198	2.11829	2.085685	2.099117	2.107773	2.092625	2.085276	2.089034	2.099727	2.045348
FLJ30838	1557472_a_at	3.106907	3.021331	2.908613	2.981631	3.085896	2.928903	2.994281	3.002596	3.00383	2.965432	3.175307	3.062634	3.057906
LOC284578	1557474_at	2.543905	2.440082	2.397223	2.426718	2.542165	2.42211	2.415313	2.432774	2.431457	2.424218	2.424201	2.442393	2.390282
PFN4	1557476_at	2.505138	2.471881	2.420143	2.450603	2.506677	2.445565	2.457713	2.470963	2.455656	2.44574	2.453513	2.464864	2.316339
DYSFIP1	1557480_a_at	2.386385	2.373629	2.351422	2.459066	2.372747	2.754564	2.384634	2.372942	2.376393	2.362656	2.359884	2.370025	2.379227
C21orf131	1557481_a_at	3.461413	3.31555	3.258867	3.276332	3.345486	3.275035	3.307899	3.321503	3.305139	3.285153	3.36248	3.419765	3.142619
LOC284788	1557483_at	2.381302	2.35538	2.326124	2.338207	2.36347	2.334067	2.349244	2.352909	2.338271	2.3259	2.335676	2.347189	2.220615
LOC645158	1557488_at	3.006493	2.921433	2.822577	2.914049	3.010613	2.891758	2.901291	2.917661	2.910876	2.902375	2.919092	2.952813	2.758207
PCCB	1557502_at	2.10005	2.088226	2.067049	2.072448	2.092232	2.07111	2.081415	2.089707	2.074797	2.097884	2.075722	2.076893	2.02546
LOC92270	1557523_at	2.520783	2.482365	2.454947	2.469195	2.506999	2.471292	2.474926	2.481596	2.466618	2.455096	2.468438	2.483657	2.304806
LYK5	1557528_at	2.182152	2.173062	2.151085	2.158142	2.172692	2.80483	2.159204	2.167816	2.153382	2.149813	2.153316	2.161445	2.099215
C12orf51	1557529_at	2.343054	2.307332	2.284459	2.293654	2.319319	2.286381	3.163655	2.832657	2.576723	2.841452	2.56808	2.849317	2.210957
C10orf55	1557531_a_at	2.990366	2.918589	2.89256	2.899074	2.991847	2.841223	2.921033	2.918947	2.911418	2.893693	2.912193	2.966811	2.683172
NDUFA7	1557532_at	3.138883	3.226062	2.994141	3.099245	3.119348	3.097704	3.105652	3.382682	3.100631	3.092556	3.092507	3.109495	2.695319
LOC339862	1557534_at	2.483807	2.444921	2.400859	2.429397	2.497278	2.556542	2.439436	2.447088	2.435872	2.419115	2.436241	2.469969	2.299668
PALLD	1557535_at	2.608501	2.57279	2.546338	2.553502	2.623005	2.560767	2.55882	2.568421	2.561134	2.527057	2.562448	2.607711	2.476087
WNK2	1557536_at	2.163126	2.15187	2.133099	2.157183	2.15866	3.266988	2.1364	2.143522	2.131556	2.124623	2.129686	2.135238	2.056144
C9orf122	1557541_at	2.459336	2.412956	2.339833	2.385184	2.480084	2.394224	2.398432	2.408531	2.399002	2.394734	2.396466	2.423825	2.35137
C10orf80	1557544_at	2.461052	2.432945	2.411581	2.41181	2.453944	2.746843	2.428698	2.433995	2.419729	2.416349	2.431861	2.431794	2.36626
RNF165	1557545_s_at	6.071834	6.082688	4.113205	4.793727	5.080043	5.768745	4.138029	5.271866	4.120271	4.032988	5.996428	5.370058	5.557544
C10orf108	1557548_at	2.883468	2.82815	2.813129	3.24196	3.039226	3.257337	2.81739	2.787572	2.807894	2.761883	2.820725	2.83652	2.572956

LOC148145	1557550_at	2.838393	2.806472	2.715198	2.79385	2.824978	2.763845	2.798124	2.804438	2.790214	2.769279	2.795642	2.801597	2.610782
PPP1R12B	1557553_at	6.083554	6.163905	6.443059	6.946877	6.441403	6.690732	4.830619	5.179385	4.764327	5.126912	6.456125	5.001388	5.845664
GRIPAP1	1557562_at	4.098056	4.211844	3.430374	3.465779	3.580898	3.471294	3.599118	4.187773	4.171017	3.640782	3.67604	3.808908	3.090595
LOC148987	1557566_at	3.238747	3.066606	3.000731	3.073422	3.234113	3.020164	3.272458	3.137257	3.123277	3.204799	3.222086	4.190426	2.744009
LOC148987	1557567_a_at	2.917706	2.83944	2.754391	2.815298	2.86039	2.803764	3.133986	3.637526	3.014143	3.095966	2.88571	2.944792	2.63905
MPDU1	1557569_at	3.227079	3.221225	3.201329	3.167228	3.192847	3.76395	3.216452	3.217878	3.20704	3.214284	3.199608	3.209836	3.077558
LOC285084	1557570_a_at	2.472149	2.44711	2.414093	2.424763	2.463083	2.422446	2.434955	2.444728	2.430872	2.415224	2.428352	2.440875	2.371738
VPS13D	1557571_at	2.871307	2.797308	2.763836	2.803977	2.861854	2.796807	2.812131	2.797329	2.807714	2.775989	2.802255	2.832598	2.675347
STBD1	1557575_at	6.898463	7.026824	4.462662	4.676356	6.45289	5.696183	2.868429	2.835014	2.514052	2.886583	2.778195	2.886515	2.397896
PAQR3	1557576_at	2.561933	2.538401	2.486476	2.522007	2.544928	2.521254	2.529384	2.539992	2.525325	2.518223	2.520985	2.535344	2.383278
PHLDB2	1557578_at	6.260635	6.919934	5.536604	6.64043	7.373362	7.806331	3.614147	5.536457	4.481906	4.396303	5.725134	5.537983	3.583019
BIN3	1557582_at	2.599119	2.568735	2.736903	2.575272	2.604837	2.556125	2.804488	2.885315	2.592051	2.564773	2.573615	3.414879	2.433323
ST18	1557583_at	2.945402	2.83851	2.819415	2.849098	2.89955	2.916534	2.857501	2.869379	2.857242	2.814315	2.858587	2.867863	2.579471
ATP6V1H	1557585_at	3.447951	3.413883	3.38066	3.393178	3.482046	3.39553	3.40263	3.409023	3.39915	3.388383	3.395766	3.410881	3.256908
ATP6V1H	1557586_s_at	2.649295	2.651556	2.820892	2.572316	2.624674	3.064268	2.620472	2.384692	2.477012	2.568127	2.686465	2.341727	2.321637
LOC147299	1557590_at	2.372552	2.359425	2.348106	2.32957	2.381925	2.345806	2.363436	2.361444	2.34627	2.344499	2.346096	2.405473	2.223346
LOC283038	1557591_at	2.333677	2.303651	2.270231	2.28428	2.316819	2.279169	2.292613	2.303926	2.289008	2.278339	2.288917	2.298748	2.181112
SPAG17	1557593_at	3.025958	2.956277	2.916286	2.948748	3.056674	2.952122	2.978574	2.928869	2.970157	2.958825	2.971878	3.015753	2.851146
GINS2	1557595_at	2.632258	2.629769	2.612902	2.616444	2.62702	2.594401	2.691659	2.629366	2.620472	2.616998	2.612581	2.64353	2.554463
KIAA1257	1557601_s_at	2.170353	2.146353	2.127686	2.130574	2.14849	2.130244	2.136501	2.14632	2.133331	2.123579	2.131937	2.144141	2.055878
LOC201617	1557602_at	2.612238	2.967265	2.446382	2.494548	2.618567	2.553564	2.95802	2.579152	4.043672	3.426776	2.776513	2.642124	2.324644
LOC401312	1557604_at	2.178634	2.158739	2.135157	2.140111	2.211803	2.139888	2.148739	2.155337	2.143338	2.136155	2.139066	2.153171	2.06178
LOC401312	1557605_a_at	1.876697	1.863121	1.8461	1.849921	1.855104	1.848223	1.860808	1.855884	1.851078	1.844866	1.843074	1.856201	1.822253
LOC284080	1557607_at	2.752544	2.698843	2.692898	2.700752	2.741149	2.700137	2.704884	2.712699	2.698826	2.678403	2.697302	2.708686	2.586143
LOC284080	1557608_a_at	1.970323	1.949905	1.934137	1.932194	1.953492	1.933392	1.944707	1.944339	1.934651	1.927392	1.925998	1.937923	1.894022
TBC1D12	1557609_s_at	3.529043	3.397233	3.356282	3.413033	3.708425	3.326944	3.414916	3.492321	3.495464	3.764079	3.645204	3.476049	3.510818
TTLL4	1557611_at	3.902691	3.933978	3.873256	3.921067	3.90667	3.891723	4.026749	4.783647	3.894506	3.890373	3.885911	3.898027	3.376505
FLJ39534	1557613_at	2.779329	2.807416	2.930388	2.852551	2.817512	2.773422	2.811327	2.807749	2.778791	2.735042	2.800952	2.815076	2.548801
SLIT1	1557615_a_at	2.207893	2.199786	2.168108	2.184454	2.205759	2.179408	2.196543	2.193859	2.184068	2.171442	2.185735	2.194932	2.103316
ZNF496	1557616_at	1.985272	1.977731	1.958797	1.959276	1.974588	1.957432	1.974094	1.974776	1.966276	1.963556	1.957153	1.969556	1.972122
LOC285768	1557618_at	2.27426	2.245632	2.228351	2.23223	2.273207	2.23039	2.239486	2.245208	2.245901	2.227783	2.229072	2.239929	2.151042
CCDC38	1557620_a_at	3.221582	2.976868	3.04619	3.224409	3.51906	3.535235	3.163906	3.174888	3.254323	3.054695	3.226762	3.275045	2.686179
CRIM2	1557621_at	2.329076	2.216982	2.199811	2.193525	2.348399	2.204137	2.188238	2.213345	2.212201	2.202813	2.199054	2.20894	2.131482
LOC283745	1557627_at	2.182525	2.159783	2.135796	2.143053	2.166859	2.141637	2.15243	2.158439	2.146313	2.13856	2.14331	2.155807	2.06431
LOC283745	1557628_s_at	2.181507	2.14726	2.119141	2.125074	2.152194	2.123717	2.134899	2.145111	2.13256	2.122637	2.128296	2.1443	2.052549
SLC25A4	1557631_at	2.592288	2.505129	2.509459	2.52607	2.560583	2.517188	2.534887	2.546727	2.528466	2.516654	2.565392	2.563441	2.417494
DKFZp434K	1557633_at	2.464172	2.413495	2.383667	2.387764	2.479094	2.391915	2.409615	2.406734	2.389301	2.369804	2.388686	2.423498	2.30422
LOC136288	1557636_a_at	3.285687	3.11182	3.049968	3.116946	3.309934	3.119472	3.122848	3.126026	3.149749	3.097316	3.228034	3.249849	2.964049

NFIA	1557639_at	2.495739	2.398976	2.376101	2.382383	2.50356	2.366325	2.377017	2.382221	2.385086	2.372641	2.38393	2.407557	2.333652
IQCA	1557642_at	2.68516	2.62731	2.608848	2.622142	2.674713	2.602771	2.632145	2.642789	2.62942	2.612515	2.631361	2.651216	2.672854
GALE	1557649_at	3.741988	3.943001	4.477644	4.075379	3.951326	4.365622	4.608245	3.408428	3.542476	4.078671	4.228395	3.500357	3.258608
GALE	1557651_x_at	4.527836	4.950611	4.910643	4.348328	5.165232	4.622461	4.680713	4.447898	5.319603	4.362159	4.342005	4.354189	4.020891
LOC348817	1557652_a_at	2.330688	2.298932	2.281929	2.287024	2.310133	2.270098	2.288727	2.29841	2.285137	2.283167	2.284305	2.290958	2.175887
LOC400238	1557656_at	3.122098	3.097885	3.08054	3.083154	3.175032	3.086537	3.086937	3.094581	3.090275	3.017236	3.080965	3.09194	2.722103
LOC400238	1557657_a_at	2.154188	2.137948	2.113036	2.12346	2.143328	2.125163	2.134368	2.134533	2.128533	2.122265	2.119604	2.134486	2.065418
LOC728883	1557660_s_at	2.368435	2.337151	2.299806	2.325004	2.360993	2.310327	2.330288	2.339	2.325728	2.31872	2.322395	2.333421	2.217734
LOC340239	1557664_at	2.297485	2.277837	2.256249	2.260796	2.306094	2.248979	2.275836	2.276484	2.26453	2.256641	2.262108	2.270903	2.202541
EFCAB2	1557674_s_at	2.930884	2.869039	2.865939	2.867034	2.933848	2.863393	2.882886	2.884464	2.841901	2.85036	3.176031	2.918325	2.802272
RAF1	1557675_at	5.885064	5.869465	5.594844	5.805586	6.350623	5.699003	5.814133	6.562995	6.534684	6.694258	6.428559	6.187905	5.740516
TRIML1	1557677_a_at	2.558449	2.504011	2.452901	2.490373	2.54718	2.478698	2.504116	2.513765	2.499371	2.48229	2.501765	2.523198	2.334139
C8orf68	1557679_at	1.772386	1.753884	1.73687	1.767043	1.755197	1.738207	1.747354	1.747956	1.738678	1.738586	1.733819	1.746096	1.698118
C14orf174	1557680_at	2.677681	2.647075	2.595144	2.608556	2.65158	2.624082	2.640464	2.643562	2.638978	2.627357	2.63255	2.680203	2.494262
C14orf174	1557681_s_at	2.892333	2.827665	2.677926	2.807794	2.854054	2.787464	2.816117	2.828006	2.808244	2.801035	2.813642	2.848978	2.660274
LOC284688	1557682_a_at	2.484833	2.448069	2.402911	2.431511	2.469975	2.416177	2.439699	2.451512	2.437205	2.422807	2.43502	2.448666	2.302533
ZNF286A	1557684_at	2.928877	2.881408	2.797554	2.722392	2.91822	2.938047	3.383342	3.436686	3.226868	3.744256	4.371952	2.954499	5.52959
C4orf38	1557685_at	3.334275	3.082175	3.182678	3.006118	3.01979	3.026391	3.480333	4.829821	4.053432	4.448741	4.542631	4.883887	2.866078
POLH	1557700_at	1.807341	1.796102	1.7634	1.782531	1.806185	1.781518	1.792352	1.793291	1.782934	1.778971	1.775957	1.78674	1.741015
POLH	1557701_s_at	2.577315	2.543295	2.486897	2.51204	2.564162	2.513201	2.543387	2.545304	2.627552	2.568455	2.53459	2.540079	2.904161
MARCH7	1557703_at	3.236177	3.150556	3.093547	3.115238	3.265788	3.144976	3.120936	3.16009	3.147216	3.127818	3.215461	3.220239	2.770827
MARCH7	1557704_a_at	3.341164	3.416267	3.231345	3.289627	3.476464	3.323918	3.297197	3.328619	3.322601	3.149614	3.644475	3.569133	2.857985
ZHX2	1557706_at	4.974009	5.157214	5.825782	6.126136	4.958361	4.96034	3.996874	4.042872	4.084876	3.994226	4.825894	4.355875	2.436227
CTBP1	1557714_at	2.059818	2.052925	2.029103	2.035348	2.042649	2.041172	2.050328	2.053082	2.045808	2.039041	2.036453	2.065232	1.974283
LOC338862	1557717_at	3.618636	3.400218	3.324873	3.438805	3.688102	3.341738	3.435121	3.40873	3.466088	3.400811	3.54436	3.62692	2.974623
PPP2R5C	1557718_at	5.674226	5.682109	4.84674	4.741444	6.816987	6.656177	5.531362	5.372791	5.135015	5.645045	5.828793	5.626898	3.982804
PIP5K3	1557719_at	2.413058	2.406106	2.373112	2.382853	2.404752	2.386039	2.397824	2.402891	2.401239	2.381938	2.386385	2.401152	2.324204
MYO16	1557720_s_at	2.14732	2.139237	2.122614	2.12526	2.258801	2.118481	2.129744	2.129862	2.122456	2.126577	2.119218	2.119713	2.125376
LOC285847	1557723_at	1.998631	1.974454	1.957882	1.966094	2.026048	2.247544	1.972907	1.972821	1.968043	1.955311	1.958984	1.972286	1.919358
LOC285847	1557724_a_at	2.499798	2.466366	2.415385	2.453475	2.495177	2.455328	2.455255	2.469981	2.451495	2.452564	2.452914	2.471789	2.312692
ARMCX4	1557726_at	2.116992	2.094741	2.070985	2.07431	2.230555	2.079337	2.083041	2.090241	2.081637	2.073763	2.078634	2.13103	2.043606
GRK5	1557729_at	2.756022	2.754898	2.853587	2.900347	2.757079	2.942378	2.502915	2.739679	2.725041	2.665301	2.68756	2.717832	2.595037
LOC400620	1557731_at	2.808568	2.785645	2.765292	2.770146	2.926498	3.06824	2.769569	2.772484	2.760194	2.735236	2.767495	2.7811	2.547003
NKTR	1557736_at	4.515969	4.020911	4.315917	4.390175	4.43379	4.052689	4.248189	4.541519	4.262359	4.241912	4.212034	4.092678	4.895087
NKTR	1557737_s_at	6.475928	6.290148	5.626967	6.01174	6.241	5.440832	5.498454	6.396483	5.679488	5.599765	6.167212	6.159896	5.871878
EHBP1L1	1557749_at	2.323096	2.292967	2.273023	2.291687	2.324113	2.310113	3.865352	2.38277	4.365235	3.047724	2.414311	3.267882	2.443599
LOC401068	1557754_at	2.76069	2.698665	2.685765	2.681295	2.702837	2.685917	2.767424	2.704058	2.727888	2.931159	2.684205	2.874547	2.379218
C14orf145	1557755_at	2.991173	2.926722	2.863451	2.917566	2.985949	3.066249	2.942647	3.066952	2.977331	2.920568	2.980091	2.956042	2.781504

C14orf145	1557756_a_at	3.087745	2.999782	3.005461	2.999729	3.129513	2.949943	3.035383	3.015725	3.407563	2.982595	3.396474	3.105329	3.459385
FLJ10241	1557759_at	4.432906	3.90415	3.870802	4.43819	5.103816	4.111289	4.300726	4.227598	4.209352	4.070874	4.47886	4.53416	3.935307
LOC400794	1557761_s_at	2.44568	2.416955	2.378068	2.4031	2.430132	2.393265	2.407252	2.417186	2.399976	2.402054	2.402353	2.412829	2.292976
P2RY5	1557763_at	2.624278	2.596144	2.529648	2.578406	2.603777	2.576489	2.586074	2.596093	2.583013	2.575668	2.593058	2.597006	2.457961
LOC643401	1557765_at	3.38638	3.466797	3.023956	3.099231	3.253051	3.095435	3.111163	3.112567	3.129857	3.09044	3.156586	3.200796	2.760885
NANOS2	1557767_at	2.059299	2.040084	2.016	2.023435	2.03923	2.020332	2.032844	2.036286	2.025033	2.02304	2.019031	2.032259	1.959361
CHN2	1557769_at	2.392185	2.429773	2.324421	2.399527	2.380937	2.414775	2.334511	2.342269	2.323883	2.319583	2.323223	2.32697	2.215379
IPO11	1557770_at	2.70934	2.657581	2.603945	2.65411	2.705924	2.65103	2.658349	2.666575	2.655311	2.633267	2.653247	2.672739	2.465739
FHAD1	1557771_at	3.14714	3.092089	3.084524	3.081678	3.147023	3.093108	3.097279	3.10389	3.095334	3.070512	3.087432	3.101416	2.713597
PDE6B	1557777_at	1.712464	1.708167	1.685703	1.690304	1.80595	1.694375	1.687271	1.689703	1.688591	1.68596	1.676555	1.689015	1.802606
C21orf49	1557782_s_at	2.397659	2.342621	2.325405	2.33027	2.3579	2.31365	2.340948	2.340005	2.34401	2.331918	2.324294	2.338067	2.330868
CHIC1	1557786_s_at	3.291981	3.267621	3.117828	3.243438	3.276232	3.250138	3.251737	3.265199	3.249514	3.235559	3.249465	3.258002	3.189223
C9orf130	1557788_a_at	2.212589	2.189234	2.165835	2.176297	2.194663	2.165751	2.177427	2.18424	2.177909	2.168885	2.173959	2.182488	2.086895
FAM62C	1557793_at	2.485123	2.448954	2.40194	2.431591	2.46536	2.415455	2.440723	2.44906	2.434993	2.422012	2.455837	2.449157	2.300746
NTRK3	1557795_s_at	1.96458	1.956159	1.937593	1.941449	1.952905	1.943673	1.950627	1.953977	1.940892	1.941088	1.935636	1.943625	1.919214
TMEM4	1557798_at	2.839527	2.759947	2.697686	2.757579	2.828404	2.718042	2.768122	2.777796	2.773897	2.77888	2.778684	2.807752	2.643297
C11orf31	1557799_at	5.150999	4.956214	4.191821	4.06571	4.670443	4.546309	4.70031	4.820464	4.542275	4.800191	4.876936	5.261188	4.668589
C11orf31	1557801_x_at	5.164685	4.583042	4.356331	4.711392	4.935691	4.641339	4.676386	4.813843	4.844145	4.638845	4.863674	5.299804	4.37666
AFG3L2	1557820_at	11.71154	11.58202	11.2568	11.47194	11.29031	11.13448	11.06701	11.38148	11.18223	11.09572	11.29219	11.2606	10.41133
LOC283547	1557821_at	2.32306	2.296629	2.256251	2.273327	2.310687	2.280532	2.289102	2.293777	2.278511	2.264808	2.280047	2.288876	2.214607
LOC401134	1557822_at	2.983595	2.858138	2.793018	2.860634	2.950832	2.816481	2.87063	2.882679	2.869336	2.837586	2.897104	2.933318	2.607178
LOC401134	1557823_s_at	3.533248	3.299336	3.246072	3.34845	3.648401	3.264934	3.38107	3.348029	3.382959	3.313117	3.484682	3.566688	2.909376
LOC338817	1557826_at	2.556461	2.994391	3.39457	3.602323	3.795184	2.531121	2.386746	2.394915	2.383847	2.231196	2.405585	2.406283	2.381202
C10orf103	1557827_at	2.321347	2.295232	2.273009	2.28284	2.297864	2.281232	2.293614	2.292842	2.284078	2.281206	2.275764	2.287742	2.171509
LOC646916	1557828_a_at	5.458142	5.618108	5.835917	5.483569	5.476528	5.180616	6.023591	5.474996	6.051145	5.992639	5.315722	5.472515	5.800332
ELMOD2	1557836_at	3.745124	3.595588	3.415749	3.520067	3.713397	3.832915	3.617295	3.537426	3.639274	3.607445	3.742911	3.951624	3.495116
ELMOD2	1557837_a_at	2.848929	2.799084	2.735423	2.795638	2.869129	2.787737	2.80653	2.814323	2.802988	2.786155	2.8083	2.84634	2.56612
LOC402125	1557840_at	2.967649	2.963723	3.146053	2.946579	3.080695	2.986334	2.974685	3.000069	2.991481	2.946048	3.015777	3.054921	2.674842
LOC285954	1557850_at	2.469772	2.453554	2.425843	2.434566	2.455763	2.433981	2.54762	2.44963	2.437461	2.429702	2.436138	2.492458	2.319882
LOC654841	1557862_at	2.773411	2.705095	2.681769	2.712805	2.771712	2.694504	2.723356	2.736656	2.723393	2.699721	2.722641	2.737727	2.571442
C9orf117	1557866_at	2.456092	2.382605	2.336447	2.367688	2.395975	2.357067	2.375825	2.412653	2.372771	2.365394	2.376423	2.387165	2.325144
C9orf117	1557867_s_at	3.582495	3.50393	3.363562	3.463912	3.605474	3.484764	3.476397	3.484557	3.472662	3.448599	3.486253	3.491503	3.215261
LOC253573	1557871_at	3.162071	3.0607	2.944172	3.044941	3.191619	2.96579	3.09285	3.038258	3.075546	3.07248	3.130183	3.250073	2.938245
CCDC108	1557879_at	2.71953	2.700865	2.663942	2.684727	2.980859	2.685526	2.688822	2.700089	2.684847	2.684236	2.681394	2.713689	2.571118
PCDH11X	1557880_at	2.210631	2.187074	2.155898	2.167712	2.193655	2.168491	2.175433	2.186756	2.170849	2.163143	2.171907	2.180535	2.085112
C10orf44	1557881_at	2.495136	2.480205	2.427743	2.462412	2.492672	2.462073	2.465026	2.478112	2.46929	2.460315	2.459691	2.472969	2.293648
CCDC46	1557886_at	2.709819	2.601753	2.589326	2.593957	2.645605	2.585331	2.60401	2.611616	2.597923	2.586623	2.601083	2.606396	2.525253
ZNF787	1557888_at	2.605242	2.565913	2.549686	2.555368	2.650292	2.578421	2.56792	2.579733	2.549027	2.548665	2.565709	2.598972	2.479694

LOC729178	1557890_at	3.275818	3.18292	3.054879	3.14915	3.277544	3.16549	3.156577	3.17971	3.153318	3.136622	3.317242	3.236416	2.889345
LOC729178	1557891_s_at	2.821051	2.778566	2.729402	2.763583	2.83767	2.744658	2.774301	2.785259	2.77138	2.753366	2.771846	2.785953	2.545036
FLJ35934	1557895_at	6.909491	6.882402	6.602983	6.809656	6.611884	6.702051	5.233199	5.252404	4.922399	4.629892	6.491799	5.842747	5.531884
CCDC85A	1557897_at	2.481376	2.469221	2.443964	2.454509	2.467038	2.451333	2.459688	2.463142	2.456091	2.448777	2.4461	2.455377	2.391107
SIM2	1557900_at	2.061349	2.042583	2.021293	2.024489	2.044472	2.023069	2.036313	2.03941	2.02688	2.018169	2.02518	2.035943	1.961086
CD44	1557905_s_at	2.919149	3.153219	3.108171	2.834996	3.173432	3.118694	3.14441	3.162396	3.570267	3.638636	3.14476	3.180798	4.80797
MUC12	1557906_at	2.062772	2.049818	2.032761	2.036263	2.06428	2.036731	2.047737	2.052806	2.039289	2.035937	2.038055	2.048155	1.997581
MUC12	1557907_x_at	2.66004	2.594422	2.574574	2.590608	2.62647	2.576484	2.605693	2.635291	2.600773	2.602094	2.645359	2.623449	2.427598
HSP90AB1	1557910_at	5.451528	4.674559	7.257816	6.356081	5.630044	4.367916	9.229814	6.431367	9.15256	8.937009	6.692495	6.896087	11.99443
GSTO1	1557915_s_at	11.4031	11.39109	11.63984	11.72746	11.42822	10.79368	11.82891	11.04079	11.70358	11.78532	10.80977	11.14878	9.177252
SLC16A1	1557918_s_at	2.405937	2.52253	2.867354	2.799207	2.38857	2.571051	2.389926	2.391314	2.380489	2.126429	2.258284	2.342718	4.208146
LOC648232	1557919_a_at	2.007722	1.985536	1.9651	1.969241	1.989813	1.967205	1.978678	1.980516	1.970028	1.962212	1.965353	1.974285	1.917451
ALPL	1557924_s_at	3.180003	3.260088	3.068332	3.146435	3.235991	3.072385	3.082845	3.089189	3.114274	3.106244	3.112343	3.124866	2.854414
PTRF	1557938_s_at	4.687875	4.633694	4.628888	4.304545	4.357375	3.703577	4.083987	3.903845	3.706838	4.346618	4.258774	3.714883	7.384657
CNP	1557943_at	2.807998	2.777611	2.774335	2.786352	2.794053	2.754709	2.79909	3.208502	2.793176	2.786769	2.783491	2.788914	2.774813
CTNND1	1557944_s_at	4.36743	4.354064	4.33697	4.169437	3.789006	3.906551	5.532539	4.352718	5.120062	5.145126	4.311541	4.331219	5.650744
TCTE3	1557945_at	4.085449	4.210858	3.167987	3.419982	3.341491	3.142173	4.782495	4.261556	5.062383	4.556579	4.270134	4.770443	4.303809
PHLDB3	1557948_at	7.403465	7.33217	6.408185	6.654089	7.26076	6.70876	5.717547	5.877057	6.18967	5.919656	5.561964	6.46962	3.978393
SDCCAG1	1557950_at	4.262718	3.914798	4.856297	4.777456	4.186174	3.072483	5.356115	4.227596	6.334339	5.684572	4.597344	4.42956	5.398703
ZKSCAN1	1557953_at	10.71719	10.71038	10.35113	10.49838	10.77162	10.47098	7.63739	7.751107	7.337667	7.456677	8.186605	8.079324	7.731615
CXorf15	1557954_at	3.66186	4.577792	5.623382	5.157339	2.785906	3.263103	5.456969	3.674255	5.891716	5.863397	2.977434	2.879725	5.17614
CDC42BPB	1557963_at	2.391617	2.381162	2.355143	2.345514	2.384725	2.368731	2.379405	2.363791	2.371516	2.352132	2.348611	2.375474	2.332915
EIF4G2	1557964_at	2.337348	2.332947	2.283399	2.502887	2.323404	2.633808	2.284295	2.322925	2.191625	2.318115	2.313346	2.316562	2.038121
MTERFD2	1557965_at	7.928677	8.034082	7.940929	8.363225	7.56332	7.288212	7.688696	7.735158	7.939461	8.287091	7.855822	7.661411	7.979256
MTERFD2	1557966_x_at	8.018963	8.078908	7.996642	8.22774	7.801973	7.949868	7.88332	7.754444	7.924548	8.019773	7.915634	7.630602	7.944082
RPS6KA2	1557970_s_at	2.414052	2.390229	2.360897	2.367551	2.39817	2.370441	2.382334	2.383796	2.372758	2.384443	2.366854	2.377424	2.379766
RPS9	1557981_at	2.976369	2.910783	2.725124	2.873465	2.91365	2.888943	2.892627	3.175557	2.791277	2.85759	2.895285	2.938565	2.6201
RPAP3	1557984_s_at	2.964202	2.950635	3.019579	3.106605	3.205451	3.04051	3.379232	3.116868	3.191369	3.335019	3.381284	3.594155	4.297715
CEP78	1557985_s_at	2.723522	2.71048	2.684746	2.652351	2.711674	2.689728	3.019882	3.10514	2.880104	3.430594	3.297894	2.873054	3.605491
SMCR8	1557986_s_at	2.830891	2.825129	2.737192	2.792017	2.942125	2.778777	2.808567	2.893705	2.817871	2.80774	2.801668	2.846827	2.688714
LOC641298	1557987_at	10.47334	10.65085	9.582538	9.67394	9.6858	9.68966	10.11105	10.26772	9.776228	9.912159	10.15174	10.41746	8.876688
METTL6	1557991_at	2.61249	2.505253	2.477441	2.485442	2.51829	2.486356	2.491509	2.502421	2.488122	2.472419	2.488428	2.497138	2.393909
TTN	1557994_at	3.349328	3.292386	3.244745	3.233374	3.49659	3.272673	3.278307	3.374952	3.243155	3.252149	3.297978	3.302335	3.245079
NAALADL2	1557998_at	2.892839	2.814216	2.765166	2.80498	2.90885	2.785345	2.812566	2.836361	2.818757	2.799763	2.821594	2.868899	2.569912
ARID5B	1558000_at	3.410429	3.353723	3.253316	3.308259	3.424369	3.354049	3.295407	3.333428	3.324064	3.281216	3.337884	3.380043	3.006342
ARID5B	1558001_s_at	2.740003	2.697549	2.629338	2.634006	2.729821	2.649504	2.692229	2.69393	2.682787	2.653353	2.680292	2.70614	2.549995
STRAP	1558002_at	5.734562	5.424186	4.451289	4.714884	5.885063	5.878469	3.773679	4.469448	4.24691	4.091388	4.451183	4.277237	3.990921
SLC1A2	1558009_at	3.113196	2.98063	2.952461	2.978485	3.101812	2.947595	3.00447	3.015987	3.001452	2.959511	3.033972	3.036944	2.87603

SLC1A2	1558010_s_at	2.490557	2.453366	2.707895	2.437875	2.484237	2.421643	2.446263	2.454397	2.445085	2.427591	2.437496	2.453325	3.553804
LOC340089	1558011_at	3.315803	3.108115	3.030899	3.128646	3.656375	3.105624	3.116522	3.420717	3.183679	3.113406	3.292215	3.414131	2.832008
MLSTD2	1558014_s_at	3.286881	3.111963	3.083047	3.14053	3.219536	3.125381	4.060161	3.146155	4.574566	4.309786	3.255795	3.260583	7.205896
ACTR2	1558015_s_at	2.306456	2.286499	2.282718	2.277852	2.293503	2.253534	3.36399	2.28068	2.565619	2.604766	2.271492	2.284397	4.257642
ERGIC3	1558020_at	2.295242	2.261347	2.248361	2.253023	2.266555	2.247643	2.244201	2.263386	2.249881	2.250119	2.247567	2.585364	2.165014
RABEPK	1558021_at	2.639503	2.63324	3.135582	3.66389	3.170922	3.193653	2.607119	2.611788	2.616366	2.591641	2.605821	2.612323	2.549409
PRKAB2	1558027_s_at	8.765477	8.417434	8.894569	8.608979	8.772729	8.431608	7.447113	6.85026	7.374351	6.915564	6.666806	6.882885	7.006258
LOC647979	1558028_x_at	6.589863	6.083876	6.923644	6.688537	7.353271	7.407504	6.589178	6.555152	6.042721	5.970697	6.537297	6.796958	8.267921
CP	1558034_s_at	13.6364	13.45635	13.57595	13.72374	13.87264	14.02149	9.475869	10.32203	9.061465	9.151696	10.0961	10.61292	3.230312
LOC653319	1558041_a_at	2.360318	2.328933	2.438213	2.356745	2.313853	2.303538	2.31801	2.331743	2.173049	2.302891	3.173511	2.325636	5.006457
EXOSC6	1558044_s_at	5.401248	5.136989	4.481719	4.73833	5.521248	5.161708	5.648271	5.316653	5.555784	5.537031	5.357398	5.58368	6.198512
LOC441528	1558045_a_at	1.545284	1.540824	1.557454	1.524882	1.530572	1.52645	1.535886	1.528348	1.534674	1.524289	1.519429	1.525026	1.698154
LOC441528	1558046_x_at	2.312568	2.298104	2.447726	2.268787	2.294996	2.258667	2.274834	2.288553	2.289042	2.281468	2.275114	2.284958	2.213885
EIF2B5	1558050_at	3.622587	3.412784	3.341149	3.395798	3.43652	3.381681	3.406617	3.416487	3.40084	3.382045	3.405641	3.542118	2.925001
TMED4	1558052_at	2.264504	2.236015	2.203996	2.218698	2.247533	2.222973	2.229981	2.234703	2.222242	2.213038	2.219883	2.228715	2.162052
TMED4	1558053_s_at	2.488035	2.468674	2.423091	2.453689	2.814983	2.456858	2.463265	2.469571	2.45918	2.464397	2.641578	2.544346	2.318736
FBXO10	1558062_at	2.102601	2.079909	2.057381	2.067097	2.083307	2.065609	2.074407	2.077604	2.065396	2.059636	2.062318	2.076833	2.06507
TBC1D16	1558066_s_at	1.552045	1.546322	1.522428	1.527605	1.539362	1.53365	1.547562	1.542719	1.534413	1.526727	1.524483	1.535144	1.514595
LOC339047	1558075_at	4.36238	3.997511	4.867087	3.772193	4.157883	3.358191	3.222043	3.430441	3.217128	3.289879	3.016557	3.543867	4.034268
ANKRD32	1558076_at	3.395467	3.204877	3.05896	3.229094	3.410943	3.222241	3.277373	4.661711	3.276499	3.246382	3.384651	3.421637	3.021781
MDH1B	1558077_s_at	2.379685	2.357863	2.330741	2.337458	2.556097	2.338393	2.349033	2.357218	2.343811	2.329776	2.343855	2.352554	2.274695
LOC144871	1558080_s_at	7.765461	7.506593	8.632506	8.203827	7.793155	7.493639	8.158035	7.574049	8.318359	8.038971	7.444632	7.891493	7.882196
SNORA65	1558081_at	1.944632	1.928083	1.910181	1.914661	1.929796	1.914371	1.925153	1.926653	1.915616	1.913012	1.908576	1.920964	1.862276
C9orf93	1558082_at	2.619004	2.569658	2.555752	2.560625	2.597772	2.561876	2.56871	2.577705	2.565862	2.545903	2.560006	2.576155	2.466781
UBE2I	1558088_a_at	4.825002	4.739937	4.720643	4.588645	4.738593	4.555652	4.728454	4.718153	4.72575	4.811562	4.737929	4.730546	4.676353
MATR3	1558093_s_at	3.729565	3.67407	4.520701	4.395783	3.994816	3.782478	5.848064	3.866955	6.110467	5.842568	3.83923	3.744523	10.10872
C3orf19	1558094_s_at	4.866961	4.828391	4.809884	5.358295	4.905911	4.706566	4.700195	4.702498	4.815009	4.725184	4.837607	4.931029	5.255143
C22orf30	1558097_at	9.031901	9.026619	8.882298	9.045707	8.855303	8.29471	9.657507	9.322925	9.662421	9.39613	9.420478	9.46281	8.67408
C20orf80	1558107_at	3.648305	3.34357	3.576232	3.421025	3.647129	3.334569	3.390493	3.368846	3.468838	3.386355	3.508402	3.560707	3.034063
C20orf80	1558109_x_at	3.429074	3.298847	3.203389	3.296212	3.501531	3.174409	3.286064	3.278948	3.380391	3.266116	3.33993	3.419698	3.242206
MBNL1	1558111_at	5.832565	5.61409	6.829375	6.328822	6.041062	5.319873	8.25099	5.797624	8.447446	8.537158	5.388945	6.426366	7.33682
FAM78B	1558113_at	2.411076	2.383927	2.351181	2.368821	2.401094	3.290897	2.369449	2.382116	2.366191	2.356037	2.368086	2.375945	2.251166
RECK	1558115_at	2.749418	2.632339	2.533063	2.558266	2.594334	2.536316	2.587351	2.577497	2.578	2.573259	2.672528	2.601593	3.109749
RECK	1558116_x_at	4.049663	3.229319	3.033656	3.210056	3.247984	3.198436	3.22693	3.342878	3.226331	3.203587	3.299344	3.227422	4.078147
USP31	1558117_s_at	4.536189	4.127423	6.464868	6.555852	4.963817	4.408039	4.898067	4.327823	5.782134	4.183537	4.409516	4.493694	5.238836
DGCR5	1558118_at	2.720014	2.673847	2.634236	2.654141	2.688487	2.644977	2.663087	2.671642	2.659347	2.636061	2.660721	2.667011	2.579794
TSPAN17	1558119_at	2.167047	2.150629	2.129268	2.133493	2.149835	2.124546	2.146938	2.146617	2.13574	2.137569	2.132283	2.142555	2.077831
DDX3X	1558120_at	2.14639	2.131123	2.112951	2.117585	2.134074	2.122135	2.128819	2.133635	2.114265	2.112063	2.141306	2.126836	2.171845

FLJ35390	1558123_at	2.391621	2.807103	2.31291	2.343085	2.356856	2.336887	2.348063	2.357881	2.347093	2.3349	2.340113	2.347267	2.338887
NUDCD2	1558124_at	3.432371	3.224918	3.011525	3.245434	3.257914	3.159666	3.255828	3.256565	3.251355	4.017497	3.344676	3.92198	3.072928
LOC730202	1558128_at	3.307721	3.079376	3.021936	3.068678	3.130502	3.062823	3.740333	3.179344	3.114877	3.667508	3.107432	3.108507	2.94052
TAF11	1558135_at	4.300701	4.300379	3.841179	4.174988	5.074085	5.29925	3.79546	3.854375	3.934623	3.823297	4.004384	4.048172	3.093058
TAF11	1558136_s_at	6.618598	6.44755	6.871652	6.748634	5.19812	4.994861	7.842797	6.58752	7.558671	7.756296	6.463969	6.666985	7.64921
FLJ39632	1558139_at	2.766515	2.688313	2.678933	2.677559	2.92704	2.714627	2.686415	2.695104	2.680082	2.666774	2.689265	2.699995	2.558773
PLXNA1	1558140_at	1.888013	1.862397	1.821796	1.84681	1.871373	1.845365	1.847768	1.859629	1.848302	1.832794	1.843107	1.854118	1.790904
TNRC6B	1558142_at	9.111606	8.899867	8.321177	8.624938	8.74206	8.752706	8.703443	9.279595	8.818847	8.670597	9.040974	9.103845	8.588209
BCL2L11	1558143_a_at	5.005402	5.698081	6.049397	5.80541	4.874318	5.391725	7.978422	7.399578	8.5491	8.633831	7.03537	7.130014	6.569428
MEG3	1558144_at	2.831963	2.786915	2.741422	2.768852	2.805327	2.757862	2.767833	2.775596	2.765528	2.761081	2.769872	2.801991	2.73171
FLJ90757	1558147_a_at	1.830475	1.822927	1.801963	1.807892	1.828227	1.808426	1.81894	1.807527	1.81943	1.8052	1.802934	2.097822	1.82574
FLJ90757	1558148_x_at	2.475297	2.494046	2.475481	2.465773	2.504649	2.478389	2.477334	2.487703	2.488666	2.460932	2.514443	2.494412	2.349579
C9orf93	1558160_at	2.398779	2.372464	2.334615	2.34661	2.383504	2.344618	2.356971	2.367937	2.356524	2.347473	2.358629	2.383894	2.251707
PEX13	1558163_at	3.119272	3.003311	2.922327	3.0278	3.112898	2.967437	3.091297	3.057452	3.039163	3.066617	3.253675	3.154175	2.858293
PEX13	1558164_s_at	2.297844	2.27806	2.290128	2.283348	2.293702	2.256293	2.463814	2.296094	3.013528	2.828802	2.295371	2.502333	2.540437
MGC16275	1558166_at	3.492388	3.450434	3.398923	3.440113	3.494338	3.291868	3.841737	3.480698	3.400175	3.589196	3.666517	3.58593	4.286245
MGC16275	1558167_a_at	3.203699	3.221569	3.163224	3.1985	3.244657	3.165135	3.192868	3.179374	3.16551	3.180296	3.178928	3.240795	3.39118
C3orf47	1558168_at	2.432444	2.403237	2.359454	2.384066	2.441864	2.396995	2.405724	2.490972	2.393282	2.378697	2.389859	2.397459	2.270101
LUZP1	1558173_a_at	5.54731	5.354214	5.11249	5.432064	6.370459	6.878979	5.49555	5.903586	4.922455	5.085866	7.196215	6.052658	5.352035
FAM43A	1558174_at	1.707455	1.700532	1.681086	1.685005	1.697203	1.685441	1.699239	1.697091	1.692347	1.687174	1.679882	1.690072	1.678658
IGL@	1558176_at	2.358161	2.331109	2.300948	2.309327	2.34047	2.316643	2.321886	2.328946	2.317404	2.309075	2.3193	2.333781	2.227164
C14orf83	1558177_at	2.164214	2.145312	2.116689	2.125792	2.153449	2.125276	2.13421	2.143106	2.12878	2.121508	2.126372	2.138065	2.049389
ATP5J2	1558179_at	2.822161	2.856619	2.790113	2.793584	2.807058	2.788101	2.811799	2.806344	2.805813	2.993444	3.118184	2.874716	2.772121
ZNF17	1558183_at	4.054821	3.818594	3.716984	3.858405	4.083114	3.769845	3.833288	3.905792	3.914788	3.815197	4.022513	4.113647	3.299553
ZNF17	1558184_s_at	2.672772	2.648777	2.570765	2.638001	2.666057	2.637374	2.92202	2.652139	2.639665	2.650331	2.57741	3.382581	3.016771
CLLU1	1558185_at	3.095934	2.917014	2.825373	2.905092	2.992555	2.897377	2.902006	2.926407	2.912521	2.870893	2.922195	2.92982	2.8014
CLLU1	1558186_s_at	2.596937	2.566513	2.510769	2.54191	2.584932	2.524386	2.548636	2.560727	2.545529	2.531171	2.551713	2.559887	2.540133
LOC284570	1558189_a_at	2.574607	2.54749	2.515417	2.524159	2.556181	2.523833	2.535813	2.547778	2.532913	2.524153	2.612466	2.604504	2.432737
LOC283404	1558195_at	2.064404	2.053564	2.034921	2.039907	2.053336	2.353452	2.039485	2.050883	2.04204	2.035047	2.029832	2.044511	1.980352
FN1	1558199_at	8.181257	7.662637	8.120977	8.70774	9.466331	9.403999	6.538844	7.431167	6.451218	6.128373	7.773557	7.758049	4.759235
SLC4A1AP	1558201_s_at	7.932495	7.948929	7.933133	7.962237	8.249943	8.08358	8.101191	8.207624	8.415575	8.209535	8.454204	8.346432	7.98099
LOC145783	1558202_at	2.75218	2.721681	2.712997	2.715606	2.772296	2.707801	2.723529	2.729586	2.723536	2.700758	2.721943	2.754092	2.634892
SRC	1558210_at	2.328534	2.29933	2.261243	2.27932	2.311693	2.281923	2.292001	2.291009	2.284204	2.277159	2.281046	2.293648	2.183369
SRC	1558211_s_at	1.830928	1.821711	1.804919	1.80952	1.820124	1.809264	1.821933	1.822373	1.814774	1.80951	1.807533	1.861138	1.781357
FLJ35024	1558212_at	1.723104	1.713956	1.696428	1.701531	1.730539	1.700704	1.711436	1.71193	1.703641	1.699227	1.694418	1.705718	1.678472
CTNNA1	1558214_s_at	2.725424	2.664632	2.656669	2.676461	2.707404	2.63004	3.882795	2.696006	4.315331	3.810596	2.675993	2.690156	7.648358
UBTF	1558215_s_at	5.991337	5.597696	5.662876	5.782606	6.180265	6.696541	7.332446	7.738291	6.896171	7.038823	7.947469	8.597606	6.017448
LOC254848	1558216_at	2.179384	2.16165	2.136741	2.14386	2.159472	2.145329	2.15452	2.157175	2.145933	2.137197	2.14773	2.156815	2.07913

SLFN13	1558217_at	3.233321	3.08181	2.971308	2.977349	3.185741	3.918344	5.505001	6.637	6.265614	5.953694	5.586645	6.219005	2.663329
LOC440993	1558220_at	8.180309	8.215683	8.140915	8.000274	7.475467	6.40383	6.740165	6.130893	7.503867	7.61637	6.525247	7.329923	6.765726
SF3B2	1558230_at	1.344608	1.335845	1.316426	1.32451	1.328885	1.319352	1.336298	1.323204	1.33265	1.32178	1.314427	1.325804	1.320349
ATF1	1558233_s_at	6.075004	5.672854	6.280959	6.058942	5.916886	5.696523	6.982942	6.299828	7.326741	7.210134	5.970957	6.400138	6.853261
FLJ36644	1558234_at	2.872244	2.810346	2.780207	2.801026	2.879218	2.785284	2.816674	2.829922	2.81516	2.777951	2.81351	2.836812	2.591099
TNRC18	1558238_at	2.298981	2.291336	2.234572	2.261392	2.289993	2.273438	2.291234	2.320038	2.275407	2.469783	2.275801	2.283685	2.222164
LOC126147	1558247_s_at	2.14333	2.125164	2.103878	2.101512	2.12509	2.106117	2.119781	2.121922	2.109289	2.105315	2.105514	2.119466	2.053259
GOLM1	1558248_at	2.757215	2.722339	2.684749	2.696252	2.777735	2.707917	2.714914	2.735049	2.70323	2.684428	2.70987	2.717389	2.502034
STX16	1558249_s_at	3.179532	3.128562	3.155214	3.015546	3.166536	3.095658	4.557685	3.026917	3.820366	4.507448	3.033765	3.162322	6.114413
ZNF587	1558251_a_at	3.922846	3.537798	3.459912	3.456233	4.053003	4.171301	4.526628	4.86135	4.216164	3.552875	4.802082	4.95844	3.047612
ZNF587	1558253_x_at	3.245724	3.347105	3.001783	3.14297	3.327492	3.179499	3.157133	3.351622	3.089541	3.052095	3.735597	4.03865	3.084109
SRPK2	1558254_s_at	3.279308	3.302365	4.99878	4.13393	3.26789	3.176629	4.553579	3.19615	4.621334	4.32914	3.213193	3.362401	7.010182
LOC148189	1558256_at	2.482255	2.357021	2.333355	2.337085	2.42372	2.631077	2.345829	2.358546	2.347353	2.336288	2.356701	2.347905	3.988516
ZNF740	1558277_at	3.086951	3.052214	2.925546	2.993331	3.172206	3.013918	3.507436	3.024525	3.022972	3.000217	3.158767	3.194476	2.781281
TMEM184A	1558281_a_at	5.095562	5.201562	4.824699	4.662944	6.00569	5.767281	3.786042	3.832741	3.71105	3.669135	4.415014	3.870956	3.252318
RFT1	1558289_at	2.00797	2.000104	1.983323	1.985858	1.996978	2.014743	1.998495	1.994631	1.99512	1.986059	1.978842	1.989965	1.959386
PVT1	1558290_a_at	2.825036	3.243009	2.952448	3.234523	3.124757	3.066629	6.784441	7.356505	7.346588	6.986235	6.407092	7.763665	3.299698
PIGW	1558292_s_at	8.004103	7.803213	7.613896	7.83948	7.402065	7.380173	8.394042	8.748494	8.323918	8.579623	8.551736	8.559139	7.171862
KIAA1107	1558293_at	4.011536	4.569586	3.557236	3.764161	3.691126	3.72477	3.572064	3.490818	3.567835	3.548011	3.556344	3.587325	4.682066
PPFIA2	1558295_a_at	2.275826	2.254204	2.228558	2.239649	2.258302	2.237781	2.250383	2.251405	2.241559	2.232772	2.239691	2.250689	3.297955
EFCAB5	1558300_at	2.890968	2.866023	2.823397	2.846392	2.935332	2.848013	2.856449	2.862548	2.848316	2.843829	2.847553	2.859731	2.738536
EFCAB5	1558301_a_at	2.790389	2.748977	2.728516	2.712865	2.799492	2.68438	2.72909	2.736358	2.730533	2.699458	2.726474	2.792452	2.486165
TSEN54	1558304_s_at	4.321152	4.310547	3.951841	4.247208	4.322042	4.535579	4.322422	4.871707	4.345574	4.366949	4.709494	4.763599	4.121132
TNRC15	1558305_at	2.842765	2.844322	2.792126	2.837596	2.900941	2.814648	3.18112	2.860979	3.109523	3.112269	2.844876	2.852463	3.239678
THADA	1558306_at	2.343576	2.311861	2.404314	2.501786	2.32261	2.300243	2.320444	2.320869	2.308043	2.383023	2.300346	2.312023	2.2801
FLJ33297	1558308_at	2.624463	2.593922	2.557932	2.643605	2.658078	2.505364	2.588463	2.592303	2.587273	2.566907	2.576047	2.599198	2.453128
FLJ90723	1558310_s_at	2.220364	2.197005	2.164802	2.177848	2.202682	2.176413	2.205002	2.213562	2.18061	2.168923	2.182024	2.189717	2.117665
HOOK3	1558315_s_at	5.670359	5.052487	5.476573	4.992031	5.040815	4.971953	4.900906	4.435387	4.731093	4.574809	4.983142	5.000289	4.911232
PAQR9	1558322_a_at	7.872704	7.893425	6.383307	6.79859	7.169663	6.453028	10.92578	10.43524	10.54912	10.48969	9.393497	10.25409	4.197252
ZDHHC18	1558327_at	3.216091	3.730131	3.139245	3.279523	3.204485	3.414339	3.166168	3.312533	2.634863	3.105526	3.511146	3.1805	2.683983
NFKBIL2	1558329_at	3.934429	3.932356	3.321589	3.913073	3.921819	4.10325	4.192452	4.371468	4.394968	4.078574	3.913363	4.122889	3.132343
NFKBIL2	1558330_x_at	4.036202	4.028094	4.008036	4.009293	4.019072	4.013621	4.028094	5.070296	4.023947	4.012187	4.011653	4.825344	3.396751
SIRT2	1558331_at	6.301854	6.04732	5.239602	5.354667	5.933811	6.007143	3.883529	4.623752	4.684069	4.819539	5.927284	5.393067	4.232618
C22orf15	1558333_at	2.748685	2.728878	2.719124	2.720938	2.763076	2.840745	2.709612	2.727789	2.717395	2.682732	2.719874	2.732031	2.584981
C22orf15	1558334_a_at	2.066025	2.051207	2.031301	2.034044	2.049358	2.035839	2.042469	2.048613	2.03689	2.029568	2.033034	2.042929	1.997428
C12orf9	1558337_at	2.332203	2.298517	2.277561	2.280779	2.3025	2.279681	2.283348	2.295012	2.280296	2.276228	2.278782	2.289991	2.185273
SEMA6A	1558338_at	2.349785	2.334189	3.188785	2.685483	2.344825	2.323855	2.300152	2.334519	2.324551	2.316394	2.316772	2.357664	2.219739
DIXDC1	1558340_at	2.457	2.421189	2.376867	2.406643	2.433471	2.395355	2.423018	3.119303	2.418018	2.41663	2.434008	2.425101	2.555626

DIXDC1	1558342_x_at	2.452773	2.429026	2.394946	2.417646	2.447145	2.416167	2.422183	3.071663	2.41548	2.393797	2.710164	2.439195	3.017147
LOC439911	1558345_a_at	5.770422	5.719974	5.628014	6.257676	5.653217	6.158057	5.085612	5.319981	5.426986	5.243098	5.371166	4.724032	4.80551
COX17	1558346_at	3.753428	3.820063	3.558594	4.035492	3.598201	3.490096	3.715712	3.816351	3.848359	3.89623	3.739211	4.101452	3.271229
UACA	1558356_at	2.590042	2.624343	2.490895	2.53016	2.606814	2.500096	2.512975	2.522592	2.517503	2.508851	2.542303	2.537595	3.373884
C1orf187	1558368_s_at	1.97398	1.959687	1.939821	1.946471	1.960382	1.946165	1.946326	1.946366	1.942756	1.940783	1.939961	1.95235	2.483688
MPHOSPH9	1558369_at	4.532751	3.986358	3.772413	3.949435	3.534632	3.753708	5.928368	5.482036	5.099443	5.899787	3.83882	4.337551	4.908252
LRRC38	1558375_at	3.100404	3.014337	2.818178	2.990601	3.098522	2.993673	3.002882	2.991176	2.998544	2.917224	3.033382	3.0939	2.684957
AHNAK2	1558378_a_at	2.577057	2.508882	2.469543	2.479324	2.567603	2.497731	2.491255	2.507139	2.489554	2.46348	2.534071	2.498685	2.366351
FLJ39198	1558387_at	3.076316	2.977083	2.924261	2.950801	3.101844	2.916926	2.978967	2.99038	2.975789	2.932687	3.106816	3.042569	3.86799
FLJ39198	1558388_a_at	2.920325	2.815081	2.746477	2.806218	2.917784	2.809938	2.822019	2.827313	2.816636	2.807025	2.847345	2.877577	6.807404
ZNF599	1558390_at	2.80358	2.759094	2.699359	2.730529	2.791923	2.716958	2.817255	2.819127	2.753889	2.735516	2.749897	2.768326	2.686212
ZNF599	1558391_s_at	2.941288	2.829303	2.735708	2.810858	2.875592	2.788838	2.821487	2.82719	2.809008	2.795775	2.850118	3.077175	3.054055
SYNE2	1558392_at	2.960747	3.118981	2.920445	2.92575	2.991569	2.925054	2.904035	2.937908	2.923218	2.908292	2.924874	2.934747	2.76059
KRT7	1558393_at	1.609699	1.601057	1.583606	1.588385	1.598895	1.587857	1.599487	1.600098	1.591811	1.586177	1.584324	1.592564	1.552003
KRT7	1558394_s_at	3.004293	2.891892	2.817082	2.86993	3.014537	2.889178	2.875649	2.885608	2.890579	2.891366	2.910747	2.948166	2.57404
ANKRD24	1558398_at	2.012104	1.996093	1.971137	1.978714	1.996141	1.980528	1.997364	1.992409	1.981065	1.974038	1.975896	1.987079	1.925255
ANKRD24	1558400_x_at	2.044077	2.028813	2.005314	2.010134	2.029632	2.008368	2.02295	2.026971	2.016244	2.006011	2.016046	2.021717	2.011291
LOC644242	1558404_at	2.608829	2.883373	2.524038	2.470568	2.578546	2.534608	2.572616	2.570642	2.699249	2.545344	3.201161	2.566448	4.539962
C3orf50	1558411_at	2.796752	2.666318	2.623748	2.649847	2.698781	2.635471	4.589525	4.404796	3.577093	4.146516	4.333852	3.857662	3.881737
LOC113230	1558412_at	3.161251	3.153976	3.150234	3.267971	3.391203	3.144426	3.113585	3.13823	3.111934	3.141761	3.130491	3.129709	2.589427
C9orf4	1558414_at	2.11547	2.090644	2.069291	2.072882	2.097788	2.072931	2.085598	2.089467	2.077155	2.065205	2.076132	2.083758	4.363634
LOC400258	1558420_at	2.157605	2.210771	4.116247	3.562726	4.522521	4.028656	2.126123	2.044656	2.121544	2.113758	2.199069	2.038139	2.200805
LOC400258	1558421_a_at	2.921883	2.884232	4.69132	4.960859	5.89214	6.245386	2.794009	2.803083	2.867706	2.720784	2.870407	2.752699	2.660064
LOC349114	1558423_at	2.204526	2.190302	2.161677	2.176834	2.199198	2.169582	2.188415	2.187077	2.177413	2.169166	2.171919	2.183626	2.168569
LOC349114	1558425_x_at	2.372293	2.356062	2.318984	2.333183	2.35582	2.340431	2.340475	2.349719	2.338165	2.333659	2.334423	2.343391	2.287906
ORAI2	1558426_x_at	7.995623	8.205871	7.099496	7.239121	7.928069	8.300829	7.30917	7.930223	6.937044	7.398908	8.38095	8.010577	7.03732
ALS2CR11	1558428_at	3.014774	2.897985	2.878672	2.89881	3.015143	2.886641	2.899021	2.912123	2.902498	2.89555	2.928195	3.014019	2.629602
FLJ36208	1558431_at	2.841773	2.832214	2.812903	2.697202	2.830207	2.856841	2.828866	2.837399	2.944682	3.450286	2.989811	2.950481	2.69347
IGHA1	1558437_at	2.107445	2.100648	2.081027	2.083129	2.098961	2.082757	2.095284	2.096656	2.086573	2.084599	2.123596	2.089656	2.044757
IGHA1	1558438_a_at	1.938192	1.929047	1.912906	1.906989	1.934445	1.916263	1.917377	1.926613	1.91042	1.902238	1.908386	1.915691	1.856708
A2M	1558450_at	1.99228	1.976141	1.957061	1.959679	1.976612	1.958642	1.971225	1.974105	1.96115	1.953948	1.949318	1.959942	1.905538
LOC285500	1558451_at	2.591851	2.548804	2.494555	2.531917	2.580629	2.521426	2.543224	2.555745	2.540623	2.529099	2.538882	2.556616	2.393875
TMEM144	1558452_at	2.409255	2.379411	2.351502	2.363255	2.379876	2.358528	2.371019	2.380123	2.366262	2.358096	2.365827	2.374033	2.241602
LOC401320	1558458_at	3.664393	3.365043	3.269633	3.348059	3.369527	4.074228	3.351843	3.273409	3.347697	3.350141	3.238121	3.356914	3.352843
LOC401320	1558459_s_at	5.534283	5.716101	5.518104	5.73036	5.713031	5.732352	5.594841	5.55094	5.629433	5.608982	5.560846	5.544353	5.032543
ABCC5	1558460_at	2.597791	2.537702	2.50786	2.519636	2.586129	2.53554	3.538916	4.24515	4.651103	4.609351	4.499615	4.229002	2.431545
LOC650392	1558463_s_at	1.494804	1.484067	1.46578	1.470113	1.487976	1.469301	1.484182	1.485388	1.478623	1.465928	1.466605	1.477783	1.478114
LPP	1558469_at	2.319634	2.290871	2.274459	2.27715	2.284809	2.273628	2.28073	2.290967	2.284687	2.273192	2.274797	2.285147	2.253466

VWA3B	1558470_at	3.084041	3.004752	2.969388	3.050903	3.158001	2.989592	3.055716	3.086209	3.079417	3.027427	3.016907	3.223495	2.72877
C1orf165	1558476_at	2.373206	2.355372	2.306789	2.329915	2.361506	2.343491	2.350966	2.354658	2.346754	2.341922	2.337984	2.349397	3.494689
LRRC27	1558483_at	2.366915	2.335585	2.319308	2.317003	2.346733	2.366928	2.323895	2.335554	2.322459	2.312396	2.317648	2.326893	2.631821
LRRC27	1558484_s_at	1.755523	1.748444	1.725417	1.73038	1.747777	1.790698	1.741103	1.746389	1.741066	1.734536	1.732863	1.738141	1.715579
ZNF493	1558486_at	8.36256	8.377139	6.996439	7.112217	8.075588	7.616812	7.62752	7.960101	8.063938	7.351597	8.082548	8.100055	8.356411
TMED4	1558487_a_at	6.734591	6.402742	6.663719	6.723818	6.350297	6.529273	6.862789	7.163156	6.609669	6.954794	7.624497	7.363303	8.10432
RP11-218C1	1558493_at	2.576408	2.538139	2.489265	2.525229	2.560403	2.532926	2.533702	2.543805	2.529872	2.515713	2.529145	2.539705	2.448176
DNM3	1558501_at	2.631343	2.588517	2.54576	2.586534	2.615868	2.556269	2.62198	3.160994	3.028555	3.066761	3.679068	3.394809	5.097283
DNM3	1558502_s_at	2.89865	2.885369	2.870376	2.869362	2.898999	2.874515	2.877227	2.882977	2.872512	2.868336	2.863961	2.882472	2.888471
C1orf53	1558507_at	3.283581	3.216957	4.081024	4.447029	3.358814	3.103049	3.371525	3.135392	3.469051	3.411181	3.133187	3.370244	2.743315
C1orf53	1558508_a_at	7.710656	7.576336	8.528787	8.839524	8.153488	8.090186	6.92666	7.478422	7.305261	7.075117	7.155681	7.447523	5.051623
FAM62B	1558511_s_at	6.917288	6.811241	7.48614	7.206192	6.480501	6.326806	6.694087	6.598115	6.656449	6.762354	6.381549	6.674367	6.144985
MAP3K7IP3	1558518_at	5.076233	4.3688	4.100077	4.615419	4.50707	4.825859	4.759778	4.774246	4.506711	4.437486	4.500132	4.887207	3.109252
RPE	1558519_at	2.990467	2.884457	2.815118	2.87695	2.994134	2.866219	2.896072	2.89353	2.893247	2.876276	2.905218	2.944682	2.734533
C6orf60	1558523_at	3.531666	3.287169	4.099131	3.9712	3.562051	3.133963	3.154083	3.144389	3.148803	3.142389	3.181358	3.27826	3.564433
LOC283901	1558525_at	2.96048	2.902643	2.549109	2.569451	3.133152	2.637348	2.672746	2.638164	2.563123	2.545479	2.67583	2.735045	2.510351
LRTM2	1558530_at	2.417779	2.389108	2.368275	2.366323	2.399794	2.35533	2.373843	2.385628	2.382246	2.368168	2.368179	2.380351	2.304195
TPM1	1558532_at	1.88665	1.879762	1.860829	1.86486	1.874584	1.865004	1.873086	1.877073	1.873658	1.86273	1.858078	1.866821	1.841398
KRBA2	1558533_at	2.578606	2.305562	2.287839	2.285016	2.319162	2.273431	2.303877	2.301724	2.295678	2.293192	2.286826	2.357897	3.334128
DKFZp547E	1558534_at	2.851804	2.786638	2.731361	2.777166	2.807062	2.759748	2.792744	2.870108	2.789443	2.765468	2.797218	2.859214	2.590883
FLJ14959	1558537_x_at	3.903613	4.394461	3.66619	3.809351	4.661026	3.791916	3.818954	3.769638	3.764231	3.686667	3.86423	3.918157	3.230051
SLC2A11	1558540_s_at	2.109365	2.117649	2.054459	2.105659	2.119685	2.110969	2.118898	2.158974	2.107733	2.095699	2.194914	2.209125	2.534911
C8orf79	1558541_at	2.300099	2.254435	2.222977	2.244589	2.264312	2.234116	2.250313	2.252498	2.239779	2.227882	2.235466	2.248136	2.14683
C1orf86	1558544_at	2.333191	2.333814	2.273701	2.310001	2.543185	2.309773	2.318198	2.329971	2.314843	2.309629	2.315694	2.324288	2.281374
DNASE1	1558546_at	3.653399	3.641385	3.393097	3.485273	3.640324	3.63402	3.635992	4.061277	3.626525	3.631376	4.021331	3.688086	3.592923
VNN1	1558549_s_at	7.755194	7.088976	8.983975	8.588787	7.249115	6.650357	3.999483	4.004809	3.995071	4.065742	3.985925	3.991751	3.486195
C3orf55	1558552_s_at	2.260304	2.226449	2.207111	2.211152	2.240427	2.20972	2.21506	2.223868	2.212755	2.207088	2.213058	2.224211	2.120062
CAMK1	1558556_at	2.658008	2.647389	2.586625	2.642371	2.703232	2.861703	2.733662	2.653204	2.637417	2.638567	3.762111	2.665058	2.67942
MGC16824	1558557_at	3.256745	3.19177	3.11212	3.175264	3.263642	3.132286	3.178961	3.18434	3.164232	3.149927	3.170372	3.210631	2.915417
BLZF1	1558560_s_at	5.36381	4.353619	5.180159	5.184286	4.920328	4.786718	4.551385	4.85985	4.368263	4.265855	4.686913	4.52833	4.347288
HM13	1558561_at	4.601142	3.90982	4.454732	4.246307	5.015309	4.73067	5.017398	5.540425	5.376496	5.161628	5.307932	5.593839	3.958812
LOC158267	1558565_at	2.096031	2.081132	2.060974	2.062374	2.081772	2.060287	2.070732	2.079102	2.06449	2.058688	2.060066	2.071748	2.002134
LOC145783	1558570_at	2.252216	2.228519	2.195126	2.209002	2.235646	2.213851	2.218092	2.228242	2.212434	2.203936	2.225768	2.227	2.124555
ZMYND8	1558571_at	2.586114	2.549632	2.51874	2.531027	2.574178	2.528566	2.53811	2.547728	2.534024	2.526775	2.533559	2.576896	2.422667
C1GALT1C1	1558573_at	2.136771	2.121601	2.104526	2.111476	2.123882	2.110449	2.119368	2.122143	2.107651	2.108513	2.103951	2.115932	2.06359
LOC148709	1558577_at	2.312798	2.292121	2.275312	2.272121	2.326472	2.277283	2.293386	2.42224	2.430096	2.280234	2.276756	2.30605	2.110282
SLC13A4	1558578_a_at	3.187913	3.156456	3.005373	3.121816	3.219635	3.166273	3.144769	3.154572	3.128767	3.128732	3.145909	3.152641	3.07433
FLJ37786	1558579_at	3.125943	3.029397	2.963372	3.009417	3.196786	2.982338	3.020096	3.044698	3.034545	2.988473	3.070173	3.101779	2.694556

MTA1	1558581_at	2.566921	2.697951	2.406271	2.404685	2.463677	2.457268	2.443649	2.825586	2.450264	2.39611	2.489581	2.649874	2.471818
UBL4B	1558584_at	1.889724	1.879439	1.860653	1.859066	1.873253	1.806834	1.876387	1.867639	1.862494	1.856042	1.863197	1.928264	1.777336
ZNF83	1558586_at	3.855446	3.828864	3.504021	3.811281	5.0556	5.004478	4.026596	3.826916	3.415701	3.64404	3.774132	4.21723	3.073521
C21orf15	1558589_at	3.049381	3.017329	2.971577	2.996135	3.035271	2.969674	3.005229	3.015087	3.001005	2.991293	2.997449	3.012203	2.903555
METT11D1	1558590_at	2.224711	2.213389	2.183995	2.195932	2.228293	2.193069	2.208559	2.214316	2.202819	2.198632	2.193881	2.208933	2.163141
C14orf176	1558594_at	2.377493	2.34512	2.325413	2.324156	2.360554	2.324281	2.342685	2.344304	2.331411	2.319357	2.326948	2.338049	2.27391
LOC285194	1558601_at	2.171936	2.150837	2.122123	2.131287	2.154164	2.128122	2.141663	2.149538	2.136096	2.127187	2.135119	2.143633	2.053998
LOC285194	1558602_a_at	2.695298	2.654016	2.583421	2.616673	2.738888	2.629029	2.635553	2.63874	2.636325	2.640933	2.637286	2.661204	2.506077
PLGLB2	1558603_at	5.064362	4.943664	7.569159	5.870654	3.455297	3.418153	3.508743	2.862971	5.067305	4.11983	2.972508	2.981983	2.416441
PAPPA	1558607_at	3.178994	3.093243	3.027171	3.089465	3.390128	3.107626	3.100732	3.123072	3.036792	2.993378	3.101926	3.101883	2.707471
PAPPA	1558608_a_at	2.999333	2.937897	2.865841	2.961672	3.121262	2.974318	2.980014	2.982515	2.982262	2.903506	3.015705	2.994185	2.820793
C17orf69	1558611_at	2.438497	2.420815	2.396468	2.402992	2.421969	2.415989	2.410098	2.418929	2.404723	2.345619	2.40788	2.454297	2.192322
ATP1A4	1558612_a_at	2.076428	2.054939	2.032707	2.041297	2.05678	2.040069	2.052552	2.054262	2.043413	2.039152	2.039133	2.047638	1.995569
SNHG7	1558619_at	2.366419	2.354123	2.326166	2.335864	2.358852	2.333636	2.347959	2.349954	2.338801	2.335672	2.329768	2.34857	2.210928
ZNF621	1558620_at	5.204126	5.074572	4.907449	4.96499	4.862874	4.609867	5.52605	5.399572	5.765294	5.751697	4.999338	4.821682	5.202245
CABLES1	1558621_at	2.561949	3.574915	2.515039	2.519238	2.545735	2.517928	2.520652	2.516338	2.484212	2.516521	2.462905	2.507914	2.362323
ZNF548	1558622_a_at	2.60154	2.581861	2.56278	2.566642	2.606542	2.558103	2.578509	2.577398	2.569559	2.561842	2.55506	2.576631	2.803707
LOC729121	1558623_at	3.168646	3.066353	3.011136	3.128569	3.16787	3.034154	3.039864	3.071283	3.058576	3.038469	3.059206	3.077444	2.911343
C18orf22	1558628_at	2.636909	2.605143	2.547009	2.579479	2.618686	2.584328	2.585644	2.600002	2.580485	2.570068	2.587901	2.597968	2.516733
PPARA	1558631_at	4.355714	3.623344	5.70358	4.720636	4.795534	3.485889	5.605084	3.553733	5.262869	5.21058	3.513392	3.547052	3.292829
ADAMTS5	1558636_s_at	3.291934	3.196244	3.124595	3.173698	3.417859	3.145304	3.143524	3.173465	3.158745	3.184168	3.20412	3.236202	3.454574
LOC728411	1558640_a_at	2.344534	2.312939	2.274382	2.293119	2.328107	2.2964	2.301411	2.312689	2.297867	2.292962	2.299527	2.307379	2.216565
LOC202051	1558641_at	4.758976	4.755281	4.821748	5.143963	4.821093	5.360506	4.759764	4.744761	4.751334	4.744944	4.549986	4.761871	4.235564
EDIL3	1558643_s_at	2.554088	2.523937	2.46611	2.503218	2.542833	2.505634	2.510248	2.521861	2.507041	2.489716	2.507468	2.522168	3.392979
MIB1	1558645_at	2.894618	2.813251	2.758406	2.795983	2.889884	2.760987	2.817898	2.827852	2.812689	2.781751	2.939914	2.842694	4.724286
SH3D19	1558647_at	3.07072	2.942473	2.922576	2.929342	2.940615	2.931262	2.935904	2.945835	2.837842	2.923101	2.858312	3.062573	2.594312
CCDC32	1558648_at	2.405794	2.692729	2.359714	2.384528	2.403905	2.602599	2.413977	2.378395	2.341919	2.385729	2.378664	2.383124	2.384704
LOC145757	1558649_at	2.287719	2.260816	2.237116	2.24174	2.271669	2.24514	2.249987	2.261332	2.245551	2.235952	2.242995	2.26237	2.146082
FLJ10081	1558652_at	2.638162	2.588688	2.567985	2.583463	2.644047	2.566662	2.593319	2.59431	2.587095	2.573083	2.586408	2.71335	2.404144
LOC339751	1558653_at	2.310545	2.251928	2.240381	2.280277	2.300852	2.245305	2.25592	2.265764	2.250606	2.24024	2.258414	2.265654	2.096806
PPM1H	1558654_at	4.355299	4.084845	4.009357	4.07728	4.497239	3.948209	4.125558	4.132435	4.171626	4.161897	4.414831	4.443612	3.623505
RP1-153G1	1558658_at	2.585866	2.567331	2.542286	2.548073	2.570831	2.546556	2.556212	2.565835	2.551423	2.544801	2.549735	2.561538	2.448177
LOC728544	1558660_at	2.168325	2.155613	2.13713	2.139593	2.162677	2.1389	2.151159	2.153594	2.142341	2.135175	2.137188	2.149556	2.060915
BANK1	1558662_s_at	2.277634	2.243723	2.220571	2.231221	2.260146	2.228574	2.243676	2.254037	2.418936	2.233291	2.236563	2.727305	2.163083
SPATA22	1558667_at	3.296311	3.199756	3.124216	3.179843	3.28659	3.1582	3.209292	3.215072	3.206409	3.185189	3.244879	3.241196	2.739785
SPATA22	1558668_s_at	2.796015	2.76298	2.682382	2.742933	2.807335	2.73462	2.750319	2.762224	2.7478	2.733722	2.748611	2.780021	2.619465
ZNF77	1558673_s_at	3.010993	2.990172	2.951551	2.939701	3.001718	2.965715	3.002352	2.990135	3.205899	2.986857	2.975789	2.983541	3.132689
SDCCAG1	1558675_s_at	5.721572	5.744138	6.094551	6.036289	5.565855	5.080733	6.933127	6.146504	7.173609	7.137295	5.664805	6.249805	6.951774

MALAT1	1558678_s_at	13.92322	13.63746	13.86829	14.04076	14.16665	14.37567	13.82961	13.99061	13.54396	13.54306	13.98964	13.95565	13.31609
LOC284804	1558679_at	2.306755	2.291975	2.263554	2.26292	2.291569	2.266477	2.288237	2.293988	2.28719	2.273979	2.325682	2.308375	2.196093
PDE1A	1558680_s_at	2.300321	2.299371	2.271234	2.280208	2.304509	2.207337	2.458558	2.301265	2.285744	2.278257	2.331184	2.306789	3.191869
HMGA2	1558682_at	2.807907	2.744946	2.670331	2.719952	2.923795	2.918963	2.680026	2.759442	2.746066	2.726961	2.746073	2.838705	2.51805
HMGA2	1558683_a_at	2.099872	2.066288	2.046613	2.049229	2.102443	2.047178	2.057298	2.061189	2.050173	2.040261	2.047928	2.057357	1.982444
LOC158960	1558685_a_at	6.386887	6.290695	6.170279	6.363147	6.716135	7.572594	6.143604	6.66488	5.936603	6.213947	6.422534	6.596953	5.561046
LOC441461	1558688_at	6.605175	6.738046	6.14079	6.17576	6.455656	6.48704	10.18357	11.24323	10.22247	9.904141	10.6479	10.99868	8.219465
LOC441461	1558689_a_at	3.988892	3.458124	3.48923	3.993813	3.666234	3.806015	3.462798	3.469853	3.4574	3.37391	3.507871	3.5187	2.913588
DOCK4	1558691_a_at	2.921601	2.811413	2.838937	2.799902	2.945442	2.7926	2.832608	2.840123	2.831108	2.801917	2.835579	2.996358	2.765919
C1orf85	1558692_at	7.227327	6.889731	6.666524	6.927044	7.537558	7.520456	6.500476	6.840798	6.726077	6.416731	6.88361	6.94738	6.425026
C1orf85	1558693_s_at	6.877729	6.987007	6.312153	6.42405	6.748536	6.384976	6.378545	6.277561	6.422386	6.394685	6.760518	6.917253	5.50686
KIAA0430	1558697_a_at	2.229607	2.214853	2.192354	2.197543	2.220615	2.196869	2.209549	2.213317	2.199275	2.189328	2.1937	2.207054	2.270967
ZNF264	1558698_at	5.183479	4.133558	4.074202	4.426528	4.40749	5.007228	4.066482	4.131133	3.912855	3.855201	4.116189	4.155895	3.814693
HERPUD2	1558699_a_at	4.947096	4.77705	5.989157	5.439739	3.945587	3.004532	5.702911	4.682918	5.77149	5.635786	4.302707	4.348911	5.396528
ZNF260	1558700_s_at	6.891047	6.833901	6.475945	6.690445	7.241342	6.682956	7.06461	7.21802	7.278595	6.971122	7.298509	6.82461	8.594655
TEX10	1558702_at	4.956795	4.873764	5.299593	4.763738	4.400969	4.013822	5.084019	4.447305	5.346985	5.142211	4.387893	4.450151	4.588887
SLC46A1	1558703_at	7.890754	7.939013	8.065861	8.395957	8.809847	7.71084	7.148208	6.558754	6.904122	6.449396	5.98959	6.386957	5.064111
ATOH8	1558705_at	2.95652	2.928897	2.854883	2.831569	2.897814	2.826063	2.874051	2.879876	2.870182	2.879462	2.871491	2.879196	2.615866
ATOH8	1558706_a_at	4.505138	4.814659	2.984897	3.184333	4.429117	4.40769	2.459053	2.777544	2.508067	2.581421	3.181377	2.761338	3.048117
NRXN1	1558708_at	3.26883	3.107634	3.030621	3.121569	3.277422	3.074853	3.125254	3.129269	3.154775	3.110821	3.184911	3.233371	2.772943
FAM13A1OS	1558711_at	3.000615	2.930263	3.04331	3.153805	2.918813	3.426862	2.741818	2.844909	2.594549	2.863308	2.99713	2.901745	2.69185
TMEM134	1558712_at	2.013924	2.00157	1.982914	1.985833	2.001475	1.985222	1.997719	1.998919	1.98815	1.979768	1.980485	1.992557	1.938559
ZNF252	1558722_at	4.770494	3.110542	3.333083	3.421603	3.109928	3.152701	2.570258	2.723168	2.913416	2.723445	2.314594	2.421853	2.816865
LOC284014	1558723_at	3.639754	3.793175	3.465238	3.592297	3.489795	3.468024	3.47706	3.481356	3.468583	3.325241	3.455783	3.457838	2.98536
LOC645478	1558724_at	2.829483	2.760573	2.739088	2.753655	2.817861	2.747436	2.766837	2.771948	2.753374	2.745536	2.758621	2.784599	3.025185
C13orf21	1558727_at	4.247151	4.143713	4.040126	4.037825	4.236033	4.822097	4.180885	4.154632	4.101358	4.038423	4.23886	4.19854	3.629108
ZBTB38	1558733_at	3.470811	3.072887	3.082567	3.139233	3.603582	4.366594	2.726373	3.07734	2.689833	2.613426	3.135123	3.134647	3.871269
NOL3	1558738_at	5.881962	6.135588	5.415796	5.080048	5.410807	5.987465	3.959262	4.667199	4.062352	3.94813	4.650842	4.238905	3.976282
ZNF620	1558743_at	4.321733	4.113996	4.10332	4.174298	4.331158	4.186975	4.368192	4.954492	4.23987	4.37755	4.74107	4.319852	3.763826
LOC92017	1558745_at	2.742623	2.754028	2.943375	2.63508	2.744827	2.643933	2.704075	2.723673	2.882609	2.676857	2.675797	2.705941	2.539087
SMCHD1	1558747_at	7.915369	7.665599	7.811942	7.963325	8.319322	8.552378	7.979673	8.642944	8.055558	8.019182	8.687879	8.980943	7.650494
ZNF763	1558754_at	4.589902	4.175716	3.170365	3.433205	3.487227	3.419499	3.435441	3.450199	3.436851	3.422181	3.433202	3.738923	3.048035
ZNF763	1558755_x_at	4.747489	4.918125	4.031092	4.228332	4.260496	4.912034	4.494867	4.485926	4.554214	4.895499	4.721809	4.872647	4.060174
DKFZP586P	1558756_at	2.055717	2.049928	2.026342	2.032237	2.040984	2.026342	2.042025	2.041601	2.032971	2.024696	2.030325	2.03447	1.98994
CLASP2	1558759_s_at	3.108503	3.090042	4.037982	3.168853	3.093627	3.07572	3.099656	2.888556	3.085917	3.086873	3.100401	3.081567	3.529298
FAM120AOS	1558761_a_at	4.640676	5.341496	4.559477	4.488349	4.384738	4.312143	4.927986	4.519226	4.695669	4.871912	4.537013	4.552032	4.604958
ZNF789	1558762_a_at	2.018283	2.003081	1.97824	1.981758	1.997904	1.973701	2.023393	2.000234	1.997513	1.991389	1.982002	1.994943	1.987316
DNAH1	1558768_at	2.359703	2.338263	2.296556	2.323174	2.367148	2.319642	2.327307	2.336816	2.323237	2.304414	2.320564	2.328649	2.257214

DNAH1	1558769_s_at	2.170354	2.148973	2.12967	2.129706	2.152487	2.132727	2.14503	2.146	2.133384	2.129298	2.131506	2.143114	2.081912
C17orf38	1558770_a_at	2.2605	2.253801	2.227	2.240854	2.251252	2.233444	2.252035	2.253112	2.237712	2.239556	2.233947	2.246609	2.190711
RANBP10	1558773_s_at	2.119685	2.101613	2.083505	2.082282	2.106453	2.081148	2.09113	2.099596	2.085761	2.078314	2.086916	2.091642	2.011174
NSMAF	1558775_s_at	3.182218	3.15624	3.020072	3.111142	3.217434	3.051658	3.094723	3.092291	3.097195	3.078614	3.125048	3.147051	3.581265
MKL2	1558777_at	3.486363	3.468819	3.127111	3.450198	3.709911	3.449212	3.460926	3.470755	3.430283	3.302133	3.487548	3.495162	3.202003
MKL2	1558778_s_at	3.094862	3.030814	2.933733	3.011171	3.12943	2.979444	3.011139	3.045962	3.017173	2.993137	3.094117	3.114101	2.699654
H2AFY	1558779_at	2.723114	2.68223	2.616479	2.661069	2.791688	2.605606	2.669978	2.678325	2.666082	2.630997	2.663007	2.68495	2.573117
H2AFY	1558780_a_at	1.972983	1.962966	1.946549	1.950678	1.968184	1.951016	1.96012	1.963747	1.958633	1.949623	1.948799	1.956644	1.890358
NFYC	1558782_a_at	2.168279	2.288844	2.127967	2.184009	2.156623	2.14666	2.152248	2.157823	2.143209	2.138748	2.73604	2.296402	2.017863
RGS5	1558785_a_at	2.967111	2.931454	2.889211	2.919656	2.950092	2.94083	2.908163	2.915165	2.911395	2.841734	2.950935	2.952674	2.805725
C8orf77	1558790_s_at	2.3544	2.336605	2.313673	2.321294	2.345547	2.302801	2.328996	2.333171	2.320594	2.312966	2.318415	2.327829	2.272017
LOC286467	1558791_at	2.276943	2.254573	2.220938	2.235212	2.264226	2.228855	2.319981	4.426099	2.267944	2.242859	3.633873	2.639708	2.198631
AP2A1	1558792_x_at	4.941669	4.69245	4.310593	4.675694	5.010665	5.432841	4.677976	4.935609	4.581027	4.668699	5.042542	4.685924	3.658396
LOC728190	1558794_at	2.814165	2.767782	2.64269	2.636437	2.800598	2.656078	3.108248	2.728288	2.983103	3.743543	2.691211	2.794631	3.320508
LOC728052	1558795_at	3.197553	3.141346	3.004515	3.390836	3.396294	3.003698	3.048206	3.079641	3.126724	3.036934	3.176602	3.162954	5.009429
LOC728052	1558796_a_at	2.349787	2.319274	2.292185	2.302396	2.333033	2.298661	2.310609	2.318141	2.303031	2.295327	2.308355	2.312779	4.606205
ATAD2B	1558807_at	2.34559	2.325823	2.296557	2.306258	2.386911	2.302761	2.329004	2.325086	2.309516	2.302309	2.322837	2.317958	2.229249
LOC284408	1558809_s_at	2.579536	2.551085	2.539499	2.529085	2.548087	2.531038	2.577487	2.555571	2.5473	2.975848	2.538251	2.543969	4.088091
TMED5	1558813_at	2.008117	1.991697	1.970048	1.973943	1.991772	1.973586	1.985898	1.988565	1.975991	1.969108	1.970451	1.983447	1.916658
TMED5	1558814_s_at	2.035594	2.027533	2.008817	2.013025	2.024416	2.009745	2.022345	2.024054	2.014765	2.00829	2.00529	2.017076	1.967822
SORBS2	1558815_at	8.181907	8.121275	8.871012	9.485754	7.862962	7.646534	4.9691	5.610923	4.754985	4.734372	5.507811	5.381317	4.39926
ZNF664	1558816_at	2.67051	2.580064	3.374946	3.039467	2.539987	2.475882	2.808195	2.519634	3.092087	2.990371	2.547612	2.554233	4.059423
C18orf34	1558820_a_at	1.967726	1.961463	1.944944	1.941748	1.982657	2.268074	1.927492	1.942192	1.944077	1.944784	1.922326	1.951993	1.956781
NBPF3	1558825_s_at	2.405899	2.372269	2.330931	2.354059	2.389366	2.361251	2.370683	2.374153	2.363114	2.357874	2.358594	2.392686	2.247608
C20orf174	1558826_at	2.227263	2.202474	2.181505	2.181307	2.204608	2.179672	2.191501	2.198719	2.187381	2.179012	2.185754	2.194205	2.103344
C20orf174	1558827_a_at	2.664389	2.566826	2.542202	2.547785	2.573093	2.546522	2.551549	2.56507	2.541879	2.513074	2.548826	2.591645	2.37488
LOC728264	1558828_s_at	7.038759	7.27545	5.302786	5.017813	6.563382	6.180455	4.228558	4.144407	4.332758	4.261115	5.486666	4.19335	5.21126
FLJ32224	1558832_at	4.106566	3.933941	3.78226	3.813249	4.440346	4.687401	3.891145	4.130349	3.844471	3.942401	4.065981	4.104975	3.628975
C1orf62	1558834_s_at	2.429711	2.3957	2.352354	2.37618	2.423713	2.363955	2.385433	2.396946	2.385429	2.370019	2.42559	2.392207	2.356149
MAPKBP1	1558839_at	1.968545	1.962746	1.926376	1.938289	1.957958	1.937989	1.948225	1.95346	1.945367	1.933552	1.941405	1.952593	1.894116
SDCCAG10	1558841_at	2.638099	2.591444	2.589002	2.7374	2.626605	2.713178	2.589864	2.597797	2.586976	2.563436	2.583277	2.598789	2.563509
LOC387790	1558844_at	3.007455	2.861174	2.664923	2.363096	2.836866	3.062516	2.829621	2.841269	2.770874	2.963543	3.546078	2.949302	3.945961
PNLIPRP3	1558846_at	2.655798	2.599399	2.571898	2.589999	2.869033	2.568316	2.594226	2.601592	2.591115	2.563732	2.585649	2.600615	2.414043
NUDT22	1558848_at	2.220284	2.204239	2.188208	2.188894	2.215556	2.194434	2.197767	2.204614	2.19108	2.183272	2.190733	2.201941	2.102896
LOC284276	1558849_at	1.454604	1.447772	1.429348	1.432131	1.44143	1.435746	1.444973	1.431847	1.440107	1.431542	1.425089	1.425589	1.418895
LOC284276	1558850_s_at	2.740011	2.709917	2.614738	2.694385	2.71766	2.882552	2.689182	2.699176	2.694483	2.676428	2.717366	2.734311	2.572313
C17orf57	1558854_a_at	6.092691	6.299123	5.092454	5.155084	5.626473	6.23251	4.346021	5.224722	4.121399	4.928583	5.530961	5.451609	4.679303
FARP2	1558855_at	1.914912	1.901887	1.883015	1.889043	1.901603	2.15859	1.894873	1.899845	1.886485	1.880668	1.881522	1.893241	1.850105

DMRTA2	1558856_at	3.0507	3.023025	3.011055	3.016537	3.023644	3.088432	3.0268	3.028152	3.008719	2.961599	3.014417	3.026051	2.958185
LOC222159	1558859_at	2.618793	2.572161	2.572418	2.571087	2.775338	2.548778	2.575426	2.583552	2.571252	2.555593	2.565263	2.59425	2.530823
NBPF5	1558862_at	2.675071	2.627682	2.589906	2.596973	2.700491	2.63968	2.627776	2.640702	2.623883	2.613494	2.640822	2.660078	2.446125
DSE	1558867_at	3.257599	3.110217	3.026784	3.109907	3.298915	3.043073	3.122409	3.123704	3.135824	3.093865	3.185449	3.23949	2.766533
DSE	1558868_a_at	2.116295	2.095372	2.06773	2.077452	2.097375	2.077819	2.087072	2.093488	2.078899	2.073193	2.078048	2.13083	2.011643
SREBF1	1558875_at	5.469408	5.482142	5.051189	5.188867	4.777899	5.163	5.198796	6.086741	5.148667	5.186171	6.111419	5.644326	3.386681
LOC145820	1558881_at	2.237707	2.197888	2.190472	2.251353	2.206176	2.19353	2.19238	2.203413	2.166812	2.167506	2.186545	2.195064	2.047061
LOC401233	1558882_at	3.966165	3.711057	3.522617	3.727172	4.409198	5.214302	4.17616	3.767942	5.053801	3.740768	4.140896	4.539173	3.152923
ZNF321	1558887_at	2.37523	2.320259	2.280061	2.301513	2.327283	2.300301	2.446626	2.48269	2.794532	2.732505	2.354305	2.362845	2.208931
ZNF321	1558888_x_at	2.343099	2.314987	2.270708	2.290318	2.326281	2.298033	2.314104	2.311869	2.309156	2.682617	2.29843	2.332498	2.193332
CCDC67	1558893_a_at	4.119131	3.864586	3.644033	3.919871	4.341527	3.701802	3.823501	3.911104	3.955898	3.903693	4.102082	4.195856	3.4476
CCDC67	1558894_a_at	2.828485	2.750371	2.743948	2.751315	2.817054	2.710338	2.759248	2.760647	2.749756	2.728463	2.756549	2.789472	2.533179
C1orf69	1558896_at	6.509805	6.287555	4.904663	5.188062	5.387552	6.208132	5.451313	6.934681	7.043618	5.176272	5.824719	6.962022	4.753325
LOC126520	1558897_at	1.584955	1.577136	1.559662	1.56029	1.57376	1.558614	1.571406	1.572093	1.570051	1.560283	1.56166	1.607775	1.56845
FLJ35946	1558899_s_at	2.675128	2.653676	2.627169	2.632971	2.691012	2.630392	2.639635	2.643933	2.632648	2.626867	2.633796	2.659941	2.521963
LOC284260	1558903_at	2.386877	2.330754	2.299814	2.312639	2.342373	2.307991	2.320605	2.332749	2.313735	2.2836	2.314493	2.326257	2.200812
C1orf121	1558914_at	2.524492	2.519871	2.49693	2.501488	2.518074	2.50079	2.53956	2.552176	2.506092	2.495919	2.50429	2.512618	2.318053
PEX14	1558915_a_at	2.689584	2.418802	2.388173	2.301473	2.592905	2.406236	2.327947	2.417717	2.407149	2.318919	2.63617	2.500733	2.197807
CLIP1	1558924_s_at	5.121157	4.637809	5.398129	5.458345	5.804121	5.83646	4.284184	4.212733	3.955393	3.948973	3.927551	4.559282	3.53161
LOC728192	1558930_at	2.392594	2.376731	2.346467	2.356014	2.37553	2.363287	2.360358	2.371794	2.356952	2.352243	2.356409	2.366357	2.284083
LRRC16	1558931_at	2.585759	2.530934	2.496339	2.506711	2.540748	2.504879	2.518395	2.525875	2.514771	2.496281	2.515912	2.524765	2.418088
ZNF704	1558941_at	2.149272	2.129789	2.104969	2.112591	2.132501	2.118602	2.119333	2.128592	2.114877	2.107108	2.113491	2.1241	2.043312
ZNF765	1558942_at	6.304324	6.591519	6.101538	6.017453	6.506292	5.940636	7.378237	6.595182	7.850002	7.458288	6.672501	6.301321	7.297659
ZNF765	1558943_x_at	6.514789	6.588571	6.444402	6.052648	6.507942	5.523258	7.588439	7.000444	7.857339	7.694148	6.706527	7.081615	7.53139
CACNA1A	1558944_at	3.398849	3.262007	3.09006	3.224449	3.353299	3.211088	3.195498	3.248764	3.243469	3.163871	3.248122	3.307245	2.964426
CACNA1A	1558945_s_at	2.925245	2.823089	2.756994	2.812836	2.927091	2.786345	2.824976	2.837332	2.824589	2.801541	2.833368	2.882307	2.699669
TNRC18	1558949_at	3.475875	3.298264	3.089773	3.15913	3.731564	3.835686	3.183226	3.175401	3.164347	3.028131	3.254829	3.408592	3.220972
CEP164	1558953_s_at	8.24509	8.292176	7.423179	7.64595	8.729408	9.011763	6.457324	7.52366	6.383617	6.259141	8.202458	7.419731	7.299303
SNORA78	1558954_at	1.308656	1.304132	1.283368	1.287106	1.29209	1.283469	1.299688	1.298733	1.295504	1.283774	1.283686	1.284624	1.296557
IFT80	1558956_s_at	8.420333	8.484413	8.28198	8.335565	8.256501	8.40321	9.175349	9.525535	9.373843	9.40005	9.718897	9.20543	10.35668
FAT3	1558964_at	2.573689	2.527533	2.478681	2.514768	2.523151	2.495017	2.583962	2.536174	2.522836	2.515673	5.441211	2.796464	6.125134
PHF21A	1558965_at	4.084415	4.500929	3.163514	3.12381	3.305195	3.661897	4.592497	3.721204	4.885456	3.627686	4.203597	3.693592	4.711305
TRIM26	1558967_s_at	2.013993	2.00423	1.979619	1.980339	1.999769	1.986024	1.994759	1.996415	1.984607	1.97484	1.979535	1.991035	2.614974
RPL32P3	1558969_a_at	4.436503	4.504825	4.157555	4.266064	4.286429	4.244671	4.323577	4.283304	4.551734	4.256364	4.557128	4.597163	4.103817
C6orf190	1558971_at	3.086578	2.97452	2.917683	2.969678	3.090397	2.919761	2.990869	2.994115	2.987841	2.950631	2.997693	3.036686	2.758705
C6orf190	1558972_s_at	2.900619	2.741022	2.681613	2.732262	3.174053	2.718125	2.741819	2.740889	2.725819	2.718035	2.75009	2.762111	2.559222
FAM100A	1558975_at	4.668329	5.337817	4.023971	4.023699	5.361438	4.803952	3.816745	3.82036	3.808251	3.972882	3.791453	3.810618	3.152617
FAM100A	1558976_x_at	5.009397	5.269301	4.116943	4.096327	4.51418	4.962669	4.032464	4.048734	3.967583	4.000512	4.194187	4.088321	2.813127

LOC375010	1558982_at	2.907027	2.847363	2.79495	2.835901	3.201442	2.819181	2.835603	2.842101	2.832593	2.803775	2.87733	2.867477	2.682332
LRRC41	1558983_at	2.935921	2.859405	2.791541	2.86595	2.948764	2.802772	2.885665	2.981055	2.867472	2.835947	2.918173	2.953825	2.644184
MAP3K11	1558984_at	2.27735	2.274301	2.251261	2.255921	2.266694	2.25951	2.267947	2.266997	2.258489	2.25798	2.249367	2.254966	2.222138
FLJ40473	1558987_at	1.915814	1.884241	1.862622	1.871348	2.021615	1.88793	1.882563	1.88301	1.86903	1.879696	1.864654	1.884779	1.835461
C18orf21	1558990_at	2.794062	2.740168	2.671759	2.720679	2.789771	2.911096	2.735302	2.74389	2.728924	2.714392	2.730711	2.779293	2.506522
ZNF547	1558995_at	2.498084	2.46889	2.441582	2.45554	2.474286	2.4579	2.467562	2.462179	2.450148	2.434451	2.452317	2.464637	2.377736
FOXP1	1558996_at	3.853522	3.760674	4.981087	4.698395	3.768157	3.827812	3.579586	3.651881	3.841476	3.751687	3.754144	3.79673	3.788179
LOC283922	1558999_x_at	4.183171	3.959494	3.932082	3.949333	4.625248	3.70194	3.95995	3.663808	3.969077	3.945064	4.218875	4.23766	3.339835
C10orf108	1559000_at	3.202752	3.218768	3.6729	3.674664	3.073095	3.199229	3.183694	3.185582	3.202962	3.16796	3.038659	3.180483	2.222108
LOC340544	1559002_at	2.363303	2.331689	2.301057	2.312365	2.341675	2.359991	2.32954	2.334079	2.315992	2.307408	2.318239	2.328511	2.214088
LOC126661	1559003_a_at	1.742331	1.734709	1.718329	1.717898	1.726996	2.038184	1.736514	1.730802	1.732925	2.253729	1.722709	1.728922	1.707563
C9orf39	1559005_s_at	5.805674	5.823684	5.02853	5.119216	4.448652	4.268788	4.608824	4.231658	4.262783	4.772358	4.204933	4.440831	4.156508
FLJ13773	1559011_at	2.302318	2.279736	2.266219	2.271346	2.348466	2.298925	2.278754	2.282252	2.268355	2.25465	2.329677	2.305445	2.200542
PTPRE	1559018_at	2.731831	2.687379	2.608869	2.670691	2.926939	2.698828	2.682274	2.699983	2.687549	2.666781	2.683584	2.735762	2.515973
KIAA0494	1559022_at	2.303856	2.249391	2.254785	2.232264	2.251066	2.216901	2.226956	2.242009	2.245728	2.214163	2.231293	2.24088	2.157949
SEPT9	1559025_at	3.73051	3.744321	3.402179	2.809434	3.683224	4.589596	3.419474	3.62961	2.812464	2.982158	3.252741	3.534149	3.646885
C21orf15	1559028_at	1.724626	1.714286	1.694079	1.699419	1.786929	1.699564	1.710361	1.710768	1.704133	1.697708	1.700983	1.706622	1.667064
EXOSC10	1559031_at	2.8152	2.766966	2.679989	2.732294	2.767751	2.753028	2.748514	2.756763	2.749779	2.744403	2.745384	2.763731	2.525413
LOC255167	1559033_at	7.5271	7.668702	8.930171	8.22768	6.339311	6.18201	2.323949	2.461676	2.452421	2.377141	2.331931	2.448475	2.206545
SIRPB2	1559034_at	2.714865	2.676472	2.644105	2.668248	2.785478	2.649236	2.671278	2.682669	2.663075	2.589234	2.666354	2.689449	2.490582
SEPT2	1559038_at	7.998007	7.895831	7.582054	7.488404	7.220589	6.851588	8.278529	7.418429	8.323755	8.218812	7.760434	7.560237	7.618794
DHX36	1559039_at	5.347984	5.450361	5.090421	5.35086	5.312549	5.667803	4.59124	5.248368	5.015453	4.572069	5.702083	5.694043	4.228019
NDUFB6	1559042_at	2.587298	2.53906	2.520444	2.537641	2.526032	2.495254	2.585054	2.53752	2.529608	2.518228	2.522584	2.532236	2.482517
EXOSC1	1559044_at	4.377035	3.99166	3.90724	4.010507	4.622119	3.936946	4.441882	4.888781	4.123052	3.97786	5.133111	4.316287	3.624602
BAHCC1	1559048_at	1.550714	1.544859	1.527791	1.529072	1.53421	1.533202	1.541372	1.536046	1.538665	1.528006	1.526718	1.529172	1.537738
BAHCC1	1559049_a_at	2.701269	2.678758	2.648308	2.656242	2.676365	2.655709	2.674728	2.678088	2.659876	2.65507	2.658305	2.699694	2.578531
HCG27	1559050_at	2.401933	2.622852	2.3394	2.356841	2.431918	2.371924	2.368369	2.372734	2.584671	2.474861	2.36087	2.402645	2.240744
C6orf150	1559051_s_at	2.56762	2.533306	2.519506	2.515155	2.738127	2.984577	2.53299	2.535832	2.524753	2.519987	2.515529	2.940288	2.39102
PAK2	1559052_s_at	3.344906	3.667065	4.184226	4.122496	3.61894	3.055922	4.743366	3.435415	4.935392	4.87545	3.660756	3.68388	6.133293
CXorf45	1559057_at	4.2468	4.721294	4.029226	4.048014	4.926214	5.162436	3.930826	4.060134	3.804534	4.027024	4.118505	4.037874	3.385965
ZNF611	1559059_s_at	2.791822	2.742569	2.745775	2.733314	2.851169	2.773315	2.762878	2.77326	2.804266	2.73084	2.765442	2.793079	2.603403
FNIP1	1559060_a_at	3.326079	3.267236	3.133745	3.2476	3.345741	3.380597	3.215388	3.265443	3.250678	3.200622	3.250204	3.260129	2.830461
LOC253962	1559061_at	2.433562	2.422193	2.39487	2.341562	2.424353	2.404209	2.415483	2.478578	2.406318	2.397308	2.405702	2.415072	2.41526
NUP153	1559064_at	2.127976	2.112127	2.090004	2.104265	2.1122	2.098029	2.105357	2.109383	2.094758	2.086762	2.092143	2.105663	2.019402
CLEC4G	1559065_a_at	8.299262	8.136565	8.701404	8.837915	8.729959	8.481264	2.355843	2.813593	2.296067	2.290775	2.900675	2.321525	2.200281
LRRC62	1559072_a_at	2.590612	2.57681	2.556886	2.564681	2.597001	2.558397	2.563244	2.560548	2.560798	2.567358	2.563704	2.566098	4.738681
JMJD2B	1559073_at	2.16795	2.147376	2.120701	2.130631	2.150919	2.129066	2.138891	2.144082	2.13412	2.12451	2.126382	2.139369	2.056046
BAHCC1	1559075_s_at	2.036698	2.029456	1.9726	2.014431	2.019784	2.014747	2.024421	2.020508	2.020892	2.013171	2.006916	2.015859	1.875162

ABI3BP	1559077_at	2.764071	2.717965	2.610277	2.667528	2.747918	2.677704	2.711955	2.721276	2.707291	2.648885	2.707284	2.732285	2.770565
BCL11A	1559078_at	2.529484	2.504231	2.408238	2.487361	2.512745	2.481954	4.852107	5.168843	4.375455	5.150049	5.103266	5.204134	5.38052
LOC283486	1559079_at	3.253111	3.237465	3.069076	3.19004	3.270398	3.180248	3.202045	3.214868	3.199127	3.179162	3.225153	3.279329	2.96579
LOC284600	1559083_x_at	4.130027	4.541953	3.970858	4.027111	4.164189	4.04843	3.95326	4.353888	3.976087	3.900043	4.041994	4.05809	3.794023
FGD2	1559091_s_at	2.122606	2.116575	2.116967	2.116594	2.112385	2.099404	2.107837	2.100804	2.101769	2.094665	2.093219	2.104184	2.111676
SLC9A5	1559092_at	4.106224	4.033016	3.987785	3.960321	4.257342	3.788174	3.980636	4.011193	4.023618	4.008329	4.035206	4.267249	3.796727
FLJ30403 ///	1559093_at	2.781658	2.796708	2.628314	2.672168	2.822502	2.660231	2.65566	2.695329	2.663461	2.649083	2.676819	2.712219	2.38563
FBXO9	1559094_at	4.678774	3.613669	4.453797	3.3995	3.488606	3.304819	5.495411	3.212357	5.822635	5.09834	3.324526	3.335477	4.522589
FBXO9	1559096_x_at	5.163104	4.362005	4.817783	3.974938	3.439644	3.526512	5.719004	3.56002	6.219453	5.562277	3.154105	3.264419	4.874443
C14orf64	1559097_at	2.947899	3.202564	2.845132	2.841583	2.997348	2.844311	2.85466	2.845	2.851216	2.832566	2.842	2.9115	2.597789
FYN	1559101_at	3.879771	3.664869	3.709857	3.609827	4.087632	4.866715	3.668787	3.879054	3.863403	3.386863	4.518232	4.088725	4.237914
LOC152084	1559105_at	2.977476	2.85552	2.891934	2.90934	3.038629	2.908308	2.916897	2.926111	2.917579	2.856355	2.89804	2.931918	2.655202
CXCR7	1559114_a_at	2.385668	2.353219	2.317518	2.336015	2.367246	2.335201	2.342611	2.354765	2.339148	2.327926	2.33501	2.348854	2.230114
ARIH2	1559121_s_at	4.207354	4.196645	4.412092	4.172671	4.073509	4.016551	4.490564	4.702452	4.713168	4.295807	3.728466	4.461962	3.551877
LOC644135	1559124_at	2.849211	2.806703	2.789127	2.782297	2.838661	2.796785	2.798186	2.806748	2.794722	2.781083	2.795954	2.815151	2.560997
RRP12	1559126_at	4.209202	4.173275	3.878953	3.564911	4.207393	4.19264	4.117994	4.821824	4.168522	4.067286	4.275213	4.480518	3.610114
RRP12	1559127_x_at	4.25578	4.226648	3.760536	4.145353	4.669341	4.795184	4.054107	4.339389	3.948747	4.047836	4.434064	4.513021	3.634485
HSDL2	1559128_at	7.036366	6.882261	7.449783	7.58824	7.354014	7.239496	5.854493	6.10122	5.478698	5.665619	6.326747	5.997254	4.820688
LOC158257	1559129_a_at	2.505321	2.468633	2.42403	2.452084	2.591314	2.462975	2.461786	2.470326	2.457429	2.446766	2.455105	2.468358	2.330218
FLJ44451	1559136_s_at	3.180696	3.485618	3.089035	2.941143	3.112191	3.095193	2.88983	3.2885	2.881031	3.053139	3.077083	3.130605	2.863474
SPATA19	1559138_a_at	1.92071	1.907306	1.886797	1.890567	1.910379	1.890381	1.900328	1.90351	1.892028	1.884884	1.88688	1.897823	1.845573
NOC2L	1559139_at	2.091619	2.073122	2.04814	2.049735	2.06672	2.050161	2.065156	2.069088	2.056737	2.867775	2.055752	2.134663	1.986881
FAM87A	1559140_at	2.481643	2.340203	2.282117	2.322484	2.329965	2.322212	2.334402	2.325669	2.321296	2.321543	2.438277	2.32436	2.18731
FAM87A	1559141_s_at	2.502598	2.457634	2.416675	2.444482	2.490096	2.447259	2.454971	2.466264	2.452459	2.437205	2.450077	2.495624	2.299797
MYST3	1559142_at	3.160774	3.110766	3.069415	3.140397	3.2215	3.156489	3.106497	3.170428	3.153568	3.105332	3.154936	3.280315	3.039466
LOC386597	1559145_at	2.278428	2.233744	2.197681	2.21472	2.244171	2.207056	2.223403	2.23368	2.218747	2.209271	2.217376	2.239852	2.12422
CEP68	1559159_at	2.544628	2.518557	2.47033	2.498821	2.521825	2.482011	2.518632	2.521544	2.509607	2.505331	2.550238	2.523243	2.478365
LOC285954	1559163_at	3.335781	3.186104	3.11319	3.176764	3.337615	3.191498	3.193613	3.242131	3.219761	3.264398	3.246347	3.315656	3.179328
MPV17L	1559167_x_at	1.296244	1.287744	1.704655	1.279598	1.05486	1.267615	1.289294	1.273109	1.279598	1.27667	1.18908	1.279598	1.066095
MGC26718	1559170_at	2.589147	2.532652	2.494136	2.507881	2.57333	2.506757	2.52093	2.530622	2.516651	2.497202	2.516662	2.547861	2.366817
LOC147080	1559171_at	2.472341	2.448217	2.423821	2.43377	2.451601	2.428167	2.440141	2.441351	2.435681	2.434676	2.473257	2.446635	2.363371
RAB6A	1559174_at	3.726056	3.576697	3.742047	3.624408	3.705519	3.544669	3.56055	3.675323	3.598085	3.465089	3.716467	3.736561	3.347344
HOMER2	1559180_at	2.317961	2.289798	2.266093	2.270574	2.303655	2.268124	2.273152	2.282515	2.281191	2.265729	2.262958	2.280836	2.54374
RDH13	1559190_s_at	6.363536	6.394099	5.810419	6.132539	7.276614	7.410834	5.376179	5.98326	5.573805	5.271352	6.914292	5.788014	5.95747
CLEC4GP1	1559194_a_at	2.184342	2.69261	2.135507	2.140075	2.17634	2.539115	2.13771	2.132735	2.136533	2.133315	2.122743	2.146784	2.059558
KRAS	1559203_s_at	3.809687	3.519623	3.522962	3.66562	3.848817	3.731273	3.654537	3.746975	3.759056	3.696856	3.858036	3.770272	3.219395
KRAS	1559204_x_at	4.273568	4.028828	4.003816	4.233829	4.254745	4.096408	3.82814	4.087015	3.779618	3.874203	4.08217	4.089867	3.020217
PSEN1	1559206_at	2.89288	2.825531	2.747488	2.817587	2.941056	2.79061	2.819764	2.815152	2.817358	2.774422	2.825482	2.854487	2.574621

ST7OT4	1559208_at	2.446083	2.410222	2.367178	2.393264	2.459812	2.3794	2.401659	2.413058	2.399297	2.38475	2.398772	2.414627	2.269579
NFYC	1559218_s_at	2.250668	2.305984	2.211932	2.216371	2.229483	2.215634	2.299435	2.556407	2.22851	2.225076	2.219656	2.467304	2.210332
LCE1E	1559224_at	1.400544	1.390298	1.370385	1.375965	1.385855	1.379747	1.383851	1.380894	1.381749	1.37285	1.370761	1.374569	1.366934
LCE1E	1559226_x_at	1.610907	1.605093	1.584884	1.589743	1.596217	1.594123	1.600761	1.594116	1.593873	1.586754	1.583993	1.587945	1.560292
VHL	1559227_s_at	3.575491	3.537876	3.550441	3.778792	3.600733	4.495534	3.889808	3.918662	4.335801	3.809899	3.551389	4.38446	3.713274
SOX30	1559235_a_at	2.224931	2.204006	2.184926	2.186582	2.211843	2.185121	2.195992	2.203252	2.188142	2.181684	2.183123	2.196657	2.115823
ACAT1	1559239_s_at	6.167654	6.436995	6.302299	5.923877	5.600747	5.13557	4.354355	5.074053	5.070524	5.025875	5.068865	4.92911	4.041761
FMN2	1559244_at	2.42217	2.387956	2.354987	2.371613	2.414355	2.374035	2.381238	2.391707	2.377612	2.360112	2.374301	2.391367	2.22925
ATXN1	1559249_at	4.964646	4.707757	4.727871	5.003784	4.793662	4.727123	3.998721	4.613786	3.604796	3.719396	4.57599	4.222911	3.950233
C20orf29	1559252_a_at	3.154865	3.141838	2.995698	3.15865	3.290569	3.03579	3.128069	3.10159	3.131523	3.129132	3.178947	3.289615	2.710854
C21orf113	1559254_at	2.276077	2.250103	2.214159	2.226994	2.324136	2.226574	2.238215	2.24397	2.233899	2.223137	2.225427	2.239636	2.135885
MAGI1	1559256_at	2.284145	2.259696	2.22064	2.239667	2.378205	2.243236	2.248871	2.257795	2.245385	2.231052	2.242101	2.254579	3.957085
MAGI1	1559257_a_at	2.661774	2.619224	2.591428	2.593996	2.621734	2.60805	2.607001	2.617087	2.602301	2.595084	2.600962	2.61208	3.533255
CXorf61	1559258_a_at	2.165936	2.114497	2.092295	2.096446	2.122587	2.101498	2.107421	2.10931	2.103235	2.091901	2.100778	2.112935	2.027454
PKD1L2	1559261_a_at	1.929682	1.974575	1.893256	1.972097	1.928572	1.905473	1.908233	1.908739	1.90695	1.8951	1.895666	1.904342	1.861069
ZC3H12D	1559263_s_at	4.751866	4.470616	3.820029	4.168731	4.849696	6.433673	3.970225	4.195496	3.784063	4.08461	4.031653	4.567438	3.201423
LOC286190	1559264_at	2.691253	2.636938	2.579954	2.623319	2.716824	2.599071	2.636338	2.641343	2.628846	2.613183	2.634994	2.657162	2.454033
C10orf140	1559265_at	3.59618	3.377714	3.457263	3.543307	4.328714	3.549058	3.782717	3.578473	3.750209	3.934899	3.756823	3.658227	3.61022
C10orf140	1559266_s_at	6.682405	6.567476	6.162473	6.491084	7.177325	7.121289	6.698019	7.260396	7.044068	6.679163	7.477139	7.686836	6.743856
ADAM23	1559268_at	2.676216	2.650728	2.629729	2.625547	2.683134	2.631755	2.632482	2.640107	2.629476	2.613268	2.632528	2.639546	2.509048
ZFHX4	1559270_at	2.164784	2.140853	2.118471	2.124887	2.147473	2.124432	2.135703	2.14317	2.131499	2.120705	2.124925	2.137386	2.049834
EXOC3L	1559272_at	2.716766	2.707533	2.690725	2.689918	2.74058	2.86309	2.706473	2.815677	2.689109	2.696141	2.688305	2.745934	2.649662
hCG_38480	1559276_at	2.124857	2.112843	2.092165	2.097334	2.182187	2.095899	2.101297	2.110293	2.097301	2.08966	2.091523	2.103785	2.028687
FLJ35700	1559277_at	3.968254	5.305809	3.815091	3.656359	3.618194	3.607187	4.531844	4.502932	3.075434	3.605946	3.652408	3.609794	5.196526
CNPY1	1559283_a_at	2.47018	2.433315	2.406277	2.419037	2.451615	2.415072	2.429426	2.436071	2.424905	2.413606	2.425392	2.434875	2.330962
TADA1L	1559289_at	3.123126	3.004074	2.9305	2.983036	3.152911	3.231577	2.93155	2.9842	2.982079	2.92961	2.993645	3.053461	2.574409
C9orf14	1559291_at	2.043409	2.019531	1.995733	2.006694	2.020471	2.009725	2.011648	2.021191	2.012883	2.007984	2.007364	2.025449	1.976677
C9orf14	1559292_s_at	3.39043	3.199634	3.13703	3.15293	3.30512	3.184389	3.167229	3.19871	3.193105	3.181549	3.202253	3.207219	3.1117
C9orf14	1559293_x_at	2.043379	2.028689	2.007259	2.01122	2.040795	2.008126	2.017524	2.028926	2.019447	2.001579	2.022131	2.0237	1.950295
LOC730057	1559296_at	2.904315	2.8814	2.831527	2.857974	2.894001	2.871248	2.875569	2.877518	2.855178	2.838968	2.89056	2.873294	2.579099
RUNDC2B	1559297_at	3.137325	3.09604	3.101631	3.104125	3.147317	3.109592	3.101054	3.390287	3.096935	3.08629	3.098534	3.105165	2.967959
FLJ42289	1559298_a_at	1.966998	1.957632	1.940574	1.945053	1.956522	1.941491	1.957252	1.952374	1.944814	1.936891	1.937373	1.946735	1.885507
KIAA1467	1559302_at	2.619266	2.580084	2.54854	2.572462	2.591141	2.559434	2.56845	2.578802	2.565108	2.55791	2.559696	2.570322	2.402112
RBL1	1559307_s_at	2.733965	2.64597	2.593219	2.636598	2.693694	2.631946	2.649616	2.659416	2.646878	2.634147	2.646305	2.656307	2.542944
MCFD2	1559309_at	2.736799	2.674367	2.635802	2.656244	3.80105	2.656633	2.663884	2.674087	2.658614	2.642446	2.659835	2.671383	2.46736
LOC646947	1559310_at	2.849985	2.751059	2.695879	2.74081	2.805722	2.736153	2.729237	2.753834	2.74658	2.69941	2.751052	2.780665	2.524871
FLJ40292	1559311_at	1.252069	1.247388	1.22858	1.232099	1.23701	1.23341	1.243297	1.233795	1.238667	1.23018	1.225471	1.226329	1.234411
LOC144481	1559315_s_at	4.465007	4.461829	4.298497	4.211023	4.146283	4.19254	3.429902	4.038313	3.87973	3.72931	3.608739	3.666366	3.470037

LOC727916	1559322_at	5.859592	4.999336	2.970687	3.562583	3.47675	3.079413	3.078006	4.235712	3.766423	3.861292	2.763335	2.995718	2.517715
LOC220594	1559324_at	2.466087	2.605303	2.385458	2.411076	2.448699	2.39867	2.424075	2.431175	2.412069	2.411636	2.414091	2.42701	2.283523
DIO3OS	1559326_at	2.111	2.093248	2.069167	2.074821	2.100274	2.079518	2.085578	2.092649	2.077434	2.077404	2.076669	2.086532	2.013001
C9orf148	1559340_at	2.514986	2.48989	2.471784	2.4749	2.569224	2.459286	2.486316	2.486554	2.476489	2.461938	2.469046	2.481856	2.395172
SNRPN	1559342_a_at	2.828373	2.757688	2.696547	2.753325	3.109877	2.774778	2.782406	2.772784	2.815486	2.773199	2.812468	2.980461	2.541683
SNRPN	1559343_at	3.975812	3.902928	4.223606	4.356528	3.959071	3.895975	3.959975	3.901143	3.909104	4.11059	3.995545	4.004603	3.812504
LOC92659	1559352_a_at	2.511864	2.4978	2.475759	2.470793	2.493549	2.488709	2.496676	2.498071	2.49294	2.754944	3.768079	2.536423	2.549833
NXPH2	1559355_at	2.368993	2.350171	2.306271	2.330189	2.403036	2.332826	2.338993	2.347444	2.332492	2.328659	2.350494	2.342381	2.426936
LOC283587	1559363_at	7.312957	7.828714	6.383097	6.694753	7.365248	6.512416	6.579997	7.473235	6.742372	6.567338	6.690551	7.004166	2.650636
FLJ13611	1559369_at	2.674445	2.582065	2.528911	2.564194	2.667489	2.974421	2.594196	2.593388	2.589445	2.562254	2.615099	2.599248	2.680419
DAB2	1559370_at	2.616834	2.577248	2.563682	2.55822	2.605071	2.56606	2.57898	2.578048	2.564154	2.552001	2.56165	2.581154	2.449549
C1orf203	1559376_at	2.570365	2.466906	2.446238	2.450272	2.472881	2.932349	2.434726	2.725985	2.432798	2.409698	2.442847	2.502501	2.354306
MED26	1559382_at	2.703318	2.810241	2.68443	2.795591	3.091647	3.632602	2.668145	2.817804	2.629595	2.631186	2.80145	2.755415	2.489023
LYNX1	1559388_a_at	2.417826	3.347626	2.420898	2.272462	2.214743	2.206683	2.164947	2.165355	1.69506	2.12675	2.122925	2.156893	1.557842
LOC149643	1559390_a_at	2.444259	2.410706	2.383037	2.404131	2.441902	2.390907	2.403247	2.404624	2.438709	2.403516	2.400764	2.491558	2.310631
SYT7	1559392_s_at	2.524779	2.47578	2.449819	2.468715	2.491156	2.470592	2.472653	2.47792	2.467239	2.46193	2.463897	2.498035	2.348688
ALDH1L2	1559393_at	3.053605	2.981785	2.878201	2.930664	3.044597	2.897903	2.967901	2.977262	2.964615	2.947381	2.978667	3.03359	2.927068
PRR14	1559397_s_at	2.721312	2.667408	2.711836	2.667065	2.737573	2.656083	3.222257	2.698161	2.759311	2.666264	2.673242	2.704057	4.454643
ZCCHC10	1559399_s_at	6.046268	5.997102	5.937466	6.040525	6.153593	5.804885	6.131106	6.376549	6.098603	6.364966	6.305715	6.393426	4.398226
PAPPA	1559400_s_at	3.121377	2.993527	2.925738	2.992138	3.283234	4.795586	2.852956	2.987001	3.000568	2.829941	3.459928	3.060661	2.964778
C1orf61	1559402_a_at	3.01291	3.187172	3.118266	3.05016	3.063035	2.980773	3.059789	3.060853	3.05267	2.997553	3.042552	3.06254	2.929372
TRPV6	1559405_a_at	2.237409	2.202135	2.16983	2.184348	2.200414	2.177735	2.195421	2.196941	2.18577	2.17758	2.181062	2.195399	2.097404
ANKRD18A	1559406_at	2.626154	2.581507	2.534494	2.570277	2.612605	2.66044	2.578955	2.589593	2.575437	2.557505	2.575818	2.58943	2.456752
KIAA1345	1559409_a_at	2.539928	2.486229	2.483308	2.487157	2.513013	2.486615	2.498424	2.49768	2.485254	2.464626	2.481235	2.495211	2.642838
C21orf34	1559412_at	2.387534	2.377913	2.351576	2.36128	2.47602	2.360998	2.367301	2.36239	2.358783	2.345332	2.37364	2.365314	2.901035
TCP11L2	1559413_at	4.138601	4.154427	4.076436	4.831005	4.511636	4.609873	4.473448	5.641214	5.895389	5.649756	4.642741	4.672975	3.152116
CACNB2	1559419_at	5.258609	4.678009	6.505071	6.654073	5.847424	6.182529	3.143804	4.170517	4.034357	3.623368	3.975031	3.776277	3.693841
CACNB2	1559420_x_at	5.170679	5.663418	6.413174	6.337864	5.546746	6.174662	3.751723	4.931582	3.556314	4.042121	4.130991	3.55155	3.267577
MCF2L	1559427_at	1.888124	1.876491	1.855885	1.86022	1.877012	1.859489	1.87149	1.873095	1.861728	1.856064	1.857994	1.867211	1.816698
C3orf43	1559429_a_at	2.77782	2.723645	2.650465	2.686762	2.766842	2.633355	2.718873	2.709265	2.715735	2.699005	2.729986	2.768118	2.571675
CCBP2	1559432_at	2.509799	2.568716	2.37388	2.373583	2.391991	2.381355	2.372012	2.38924	2.352852	2.354511	2.365351	2.380971	2.263371
LOC149773	1559433_at	2.802321	2.732357	2.669672	2.721751	2.783386	2.698012	2.734993	2.744489	2.730897	2.706343	2.729765	2.763949	2.536408
C21orf58	1559438_at	1.614606	1.607248	1.588356	1.590589	1.599985	1.590945	1.608078	1.602373	1.605911	1.593215	1.587258	1.595577	1.58062
C21orf58	1559439_s_at	3.882032	3.656241	3.576725	3.729629	3.904955	3.82531	4.851354	4.614333	4.233065	4.096114	4.143672	4.577599	3.671115
FLJ38576	1559441_s_at	2.086266	2.07107	2.046164	2.053031	2.075397	2.052779	2.065529	2.07005	2.056242	2.048666	2.056882	2.063649	1.997768
LOC283888	1559443_s_at	2.664986	2.755086	2.604174	2.600926	2.624447	2.577784	2.615795	2.626821	2.610652	2.614836	2.611057	2.629606	2.461445
ZNF254	1559449_a_at	5.688455	5.368784	5.777976	6.199831	5.576606	5.004834	4.878572	4.607532	4.882754	4.818098	5.049312	4.568333	4.578572
LOC613266	1559459_at	2.248383	2.229434	2.196634	2.210443	2.233199	2.208985	2.2256	2.228998	2.212718	2.208039	2.211431	2.22239	2.122698

SIPA1L2	1559469_s_at	2.545514	2.538051	2.505473	2.520184	2.548308	2.517124	2.527899	2.536748	2.527938	2.516962	2.521713	2.533633	2.85335
D21S2088E	1559470_at	2.4635	2.431327	2.382669	2.408382	2.51927	2.416042	2.418092	2.428404	2.41943	2.401415	2.417134	2.45482	2.283394
D21S2088E	1559471_s_at	2.668424	2.736786	2.519508	2.558271	2.609183	2.624814	2.577262	2.789268	2.568035	2.559308	2.581618	2.585293	2.437038
SPEG	1559474_at	2.100464	2.083627	2.058642	2.065213	2.083822	2.063597	2.075021	3.001692	2.067841	2.060309	2.06311	2.077007	2.003106
MEIS1	1559477_s_at	2.375252	2.348251	2.496026	3.275697	2.363521	2.269088	3.484808	2.351351	3.631851	2.732244	2.335076	2.344885	8.088356
LOC285540	1559479_at	4.23256	3.738227	3.668859	3.723376	4.386099	4.269215	3.649551	4.64643	3.737001	3.396293	4.628693	3.806257	3.500795
CHIC1	1559481_at	2.429631	2.39819	2.320386	2.378729	2.424091	2.381111	2.386809	2.398721	2.383468	2.375928	2.382471	2.41015	2.309525
ATG2B	1559485_at	2.599026	2.549611	2.489883	2.52889	2.613227	3.103188	2.549764	3.052733	2.552276	2.514791	2.9709	3.167626	2.385803
LRCH3	1559490_at	6.324549	6.613214	6.026344	6.221741	6.308818	6.101497	6.125645	6.402658	6.345428	6.364184	6.391661	6.295155	5.061369
VPS8	1559500_at	4.182024	4.270696	4.66052	4.07003	4.41711	3.852751	4.948329	4.52534	5.45752	4.536094	3.984955	4.035522	4.940803
LRRC25	1559502_s_at	3.810325	3.384256	3.785225	3.791049	3.869909	4.097688	3.799896	3.801356	3.764821	3.783234	3.783346	3.792461	2.716998
LOC441666	1559503_a_at	3.313822	3.23584	3.136658	3.197427	3.359818	3.086002	3.213595	3.236158	3.220924	3.179806	3.224218	3.272572	2.959707
FLJ35409	1559504_at	3.552952	3.343579	3.171137	3.403461	3.68415	3.285674	3.294859	3.420705	3.421205	3.370281	3.490669	3.578104	2.898822
FLJ35409	1559506_x_at	3.452707	3.297674	3.1983	3.356048	3.540323	3.323494	3.361759	3.312179	3.358704	3.350813	3.403699	3.479625	2.954106
BHLHB9	1559510_at	2.733392	2.710237	2.699026	2.706057	2.747445	2.702684	2.704945	2.714707	2.706216	2.697885	2.705074	2.710683	2.646969
FANCC	1559513_a_at	3.942754	4.425359	3.353562	3.99586	2.498505	2.805406	2.378912	2.485414	2.513384	2.465184	2.161735	2.549256	2.248076
SPIRE1	1559517_a_at	2.396729	2.374644	2.363113	2.365565	2.383112	2.358133	2.377647	2.368433	2.363575	2.362297	2.366372	2.379373	2.885358
HSD17B12	1559518_at	3.284467	3.152423	3.042367	3.206396	3.294801	3.144617	3.146274	3.15194	3.160661	3.108665	3.195367	3.255227	2.781561
GYPA	1559520_at	3.2797	3.205341	3.087303	3.200132	3.330839	3.141717	10.13957	8.958092	9.98855	9.893239	8.710941	9.108697	2.880879
PCGF3	1559528_at	3.317106	3.168021	3.071386	3.192472	3.367868	3.142673	3.189486	3.184378	3.222495	3.205221	3.291741	3.305664	3.170957
PTK2	1559529_at	5.880068	5.811437	4.922626	5.189706	5.722008	6.030241	5.714701	5.932928	5.631967	5.61232	6.161692	6.066688	5.509403
LOC646450	1559532_at	2.03866	2.022855	2.003971	2.002767	2.022055	2.001615	2.117685	2.013072	2.002574	1.994701	2.002673	2.009528	2.011696
SNRPN	1559545_at	3.64356	3.460103	3.301212	3.467865	3.711105	3.397669	3.465953	3.364602	3.50147	3.402952	3.540883	4.048738	3.660085
SNRPN	1559546_s_at	3.147863	3.12926	3.087431	3.017499	3.131015	3.108287	3.119579	3.127049	3.112544	3.104884	3.112701	3.122183	3.053806
ACVR2B	1559548_at	2.730394	2.696485	2.689464	2.659833	2.701926	2.662329	2.687504	2.681325	2.692626	2.687934	2.682721	2.697027	4.557055
C9orf79	1559559_at	2.502469	2.461432	2.417868	2.441536	2.478687	2.427591	2.454821	2.462694	2.44978	2.433401	2.446113	2.466947	2.310094
FBXO18	1559561_at	2.844724	2.814825	2.721449	2.848592	2.828518	2.786554	2.812021	2.790303	2.822638	2.883155	2.785402	2.809166	2.689558
FBXO42	1559566_at	2.580041	2.529046	2.461629	2.515284	2.534352	2.543623	2.572767	2.74828	2.517234	2.829348	2.515465	2.670488	2.251534
ATP13A4	1559571_a_at	2.46816	2.436853	2.389699	2.417518	2.456092	2.409727	2.427479	2.438253	2.423268	2.412635	2.42118	2.432985	2.289628
AQP12A	1559575_a_at	2.267091	2.245494	2.22251	2.225712	2.247502	2.225956	2.235701	2.243712	2.227749	2.223403	2.22523	2.233934	2.129352
WDR78	1559576_at	2.074809	2.061755	2.131482	2.051626	2.067675	2.044885	2.05513	2.059587	2.133488	2.037352	2.048984	2.102865	2.541422
FCN2	1559577_at	7.572499	7.209232	7.658596	8.108973	7.029091	6.937614	3.043254	3.131332	4.687441	4.503531	2.502287	3.370834	2.720933
LOC283688	1559579_at	2.261212	2.235616	2.202382	2.217045	2.245056	2.214855	2.224777	2.234942	2.221219	2.211489	2.218388	2.230378	2.125995
LRRC39	1559580_at	3.018166	2.903494	2.911927	2.889225	2.993685	3.466276	2.942368	2.894102	2.847059	2.850942	4.366251	3.180241	4.225316
RHOQ	1559582_at	2.818775	2.747066	3.230369	2.816657	2.830166	2.813971	2.714887	2.696214	2.701981	2.799689	2.808616	2.712873	3.323354
CD276	1559583_at	2.347029	2.341408	2.87285	2.681285	2.256752	2.324446	2.289144	2.302542	2.288137	2.276653	2.316019	2.318206	2.602722
C16orf54	1559584_a_at	4.410075	3.905336	4.430504	3.751677	4.457985	4.636738	3.155412	3.236206	3.09371	3.173658	3.046596	3.570547	2.778874
FLJ31033	1559585_at	2.140589	2.116312	2.098658	2.10054	2.127922	2.11021	2.112588	2.117571	2.104642	2.097381	2.10309	2.112494	2.088475

LOC728868	1559586_at	1.664995	1.656758	1.639197	1.64199	1.654848	1.643528	1.655179	1.654903	1.648087	1.642005	1.639067	1.648431	1.621803
SYMPK	1559587_at	2.770639	2.696173	2.671093	2.689277	2.882073	2.691587	2.695293	2.701954	2.679438	2.671359	2.687955	2.69385	2.540431
CHDH	1559590_at	4.264781	4.182469	4.481977	4.807325	5.26873	4.279315	3.475851	3.803458	4.063228	3.995454	3.22934	3.834251	3.074292
CHDH	1559591_s_at	7.699624	7.741278	7.176433	7.54195	8.372449	8.368972	5.894128	6.862516	6.561451	6.452276	6.531729	6.399928	3.70389
MED26	1559593_a_at	3.749944	3.867166	3.437687	3.527693	3.636421	3.649889	3.944421	3.785752	3.610789	4.216501	4.653382	4.47882	3.357514
KRT85	1559595_at	2.180729	2.163225	2.136956	2.150297	2.171317	2.144139	2.159872	2.162073	2.15131	2.146803	2.144039	2.157718	2.06787
KIAA2018	1559601_at	2.960623	2.851505	2.795372	2.830119	2.908218	2.852323	2.856076	2.866111	2.851947	2.829703	2.886086	2.902324	2.651173
LOC283535	1559603_at	2.509125	2.37964	2.348869	2.378243	2.387031	2.36194	2.369391	2.375485	2.363455	2.440221	2.366867	2.374545	2.763188
LOC285043	1559605_a_at	2.241437	2.217501	2.199465	2.205613	2.226225	2.208038	2.21468	2.218229	2.207467	2.199662	2.204617	2.260357	2.102593
GBP6	1559606_at	3.070046	2.952379	2.866354	2.945976	3.066497	2.943916	2.959148	2.967408	2.958025	2.929376	2.97587	3.012981	2.687905
GBP6	1559607_s_at	2.190441	2.16483	2.146301	2.153927	2.175159	2.153382	2.163084	2.168417	2.155075	2.147104	2.154129	2.165302	2.110418
TMEM75	1559611_at	2.511109	2.484131	2.447501	2.467297	2.55474	2.458655	2.47082	2.478432	2.459682	2.451033	2.46409	2.478018	2.238833
FLJ38773	1559614_at	3.364775	3.244639	3.107847	3.208241	3.384949	3.145247	3.220727	3.256758	3.239219	3.223135	3.284244	3.33578	2.864125
ZNF626	1559616_x_at	4.135319	4.115742	3.763682	4.09737	4.118039	4.4331	4.107643	4.107992	4.098575	3.910808	4.167322	4.105351	4.034333
LOC732087	1559617_at	2.391933	2.357434	2.341558	2.337576	2.375246	2.343656	2.557283	2.580747	2.339109	2.331728	2.428524	2.352225	2.221312
hCG_18208	1559620_at	2.321518	2.305972	2.285391	2.289054	2.306674	2.291743	2.302894	2.304756	2.294495	2.284058	2.286451	2.298721	2.208198
C11orf54	1559623_at	2.783202	2.58018	4.027344	4.147219	2.822432	2.734272	2.633882	2.573661	2.5883	2.556028	2.5698	2.60258	2.390066
STK32A	1559624_at	2.612898	2.575959	2.558448	2.562713	2.576961	2.529637	2.573035	2.575447	2.56631	2.559307	2.556656	2.568873	2.51851
LOC285941	1559627_at	2.670123	2.587362	2.495263	2.554935	2.65905	2.572894	2.572009	2.585175	2.568668	2.565658	2.568604	2.608843	2.381036
DLGAP3	1559630_at	2.040665	2.017688	1.988981	1.997594	2.024474	1.999643	2.007402	2.015679	2.002749	1.992922	2.000555	2.008073	1.954822
CHRM3	1559633_a_at	2.50362	2.46258	2.417007	2.454875	2.475944	2.453826	2.458104	2.467816	2.453842	2.437591	2.453642	2.468065	2.940223
CHRM3	1559634_at	2.025065	1.993054	1.973386	1.978884	1.996419	1.979514	1.990626	1.992265	1.981699	1.975953	1.973701	1.989986	1.937789
CASC4	1559635_at	2.150277	2.121804	2.102371	2.110689	2.195109	2.103392	2.119603	2.119657	2.110141	2.102608	2.11347	2.131869	2.036532
C1orf200	1559638_at	2.852991	2.821711	2.767719	2.799255	2.855202	2.80139	2.807916	2.821561	2.806159	2.78812	2.790962	2.81665	2.628016
ANKFN1	1559640_at	2.234118	2.203709	2.183622	2.186759	2.217578	2.18632	2.192989	2.202636	2.189325	2.181458	2.18575	2.197533	2.134855
LOC161635	1559651_at	2.583344	2.529532	2.511913	2.519444	2.590301	2.502793	2.536643	2.544878	2.531691	2.516467	2.529602	2.548142	2.372767
C15orf29	1559658_at	1.867794	1.858122	1.844361	1.842721	1.853519	1.840792	1.858375	1.854971	1.855026	1.848171	1.841833	1.848957	1.961087
ODZ3	1559661_at	1.93416	1.899338	1.881318	1.884649	1.900695	1.883715	1.895541	1.897478	1.885885	1.880408	1.88184	1.892265	1.838739
C9orf93	1559672_a_at	3.664662	3.31905	3.267097	3.321919	3.357987	3.137704	3.322582	3.30688	3.319219	3.317004	3.317132	3.404659	3.269421
ZNF333	1559674_at	2.028964	2.020591	1.994784	2.00356	2.015736	2.001474	2.019247	2.019561	2.006398	2.007677	2.005089	2.025325	2.008716
LOC730495	1559678_s_at	3.096616	3.007669	2.940309	2.998007	3.089393	2.899299	3.0232	2.95853	3.016633	3.009039	3.017219	3.072006	2.769744
TTL	1559680_at	2.563878	2.522472	2.469625	2.502546	2.551945	2.507217	2.514094	2.52501	2.511404	2.493086	2.508302	2.525943	2.363487
TRIM16L	1559681_a_at	2.774995	2.753408	2.953436	2.853145	2.75804	2.707114	2.746047	2.754681	2.860653	2.832478	2.660036	2.76266	3.094327
TRIM16L	1559682_at	3.360826	3.525941	3.408538	3.446428	3.496197	3.45845	3.458248	3.939255	3.462532	3.435264	3.452718	3.472308	3.431494
NXNL1	1559687_at	1.913569	1.881475	1.863483	1.868378	1.901782	1.876167	1.879318	1.941369	1.87807	1.867039	1.867621	1.875746	1.820759
LOC400581	1559688_at	3.676909	3.615324	3.493998	3.597587	3.889563	3.586909	3.579323	3.586833	3.594192	3.551406	3.668006	3.660172	3.543636
LOC400581	1559689_a_at	2.626622	2.596875	2.530955	2.578192	2.96131	2.570377	2.559519	2.590982	2.576758	2.55611	2.590449	2.594969	2.40443
C20orf74	1559699_at	2.526851	2.484123	2.431434	2.44864	2.515969	2.4552	2.466476	2.477469	2.465195	2.452632	2.470802	2.480959	2.364905

NDST2	1559701_s_at	2.483676	2.476356	2.449484	2.462259	2.472233	2.463582	2.467251	2.831748	2.475192	2.459774	2.45854	2.772098	2.314991
PHKA2	1559705_s_at	2.718825	2.783056	2.82527	2.697835	2.702017	2.695336	2.710733	2.695934	2.706881	3.063254	2.574144	2.68812	2.566684
RGNEF	1559706_at	3.438548	3.98565	3.103733	2.9297	4.034571	2.963975	2.943998	2.967375	2.874292	2.867918	2.884252	3.043109	2.662728
FAM120A	1559711_at	2.514593	2.480021	2.431848	2.462363	2.519792	2.449572	2.472945	2.483811	2.468866	2.454236	2.467024	2.481374	2.326256
LOC154822	1559712_at	2.050362	2.039067	2.016615	2.019376	2.03924	2.021003	2.034688	2.039144	2.024367	2.016986	2.023045	2.032114	1.985192
RGR	1559714_at	2.159015	2.11546	2.09217	2.09636	2.118771	2.095522	2.106637	2.112387	2.099952	2.090741	2.099964	2.108742	2.038986
C18orf37	1559716_at	2.553926	2.536555	2.465784	2.515631	2.590954	2.524943	2.528308	2.781218	2.524015	2.523353	2.544019	2.563532	2.460729
ZBTB40	1559728_at	2.68768	2.655778	2.641677	2.634878	2.654736	2.642453	2.643262	2.653262	2.645666	2.626374	2.636548	2.650198	2.607695
MACROD1	1559731_x_at	4.106573	4.483303	3.331758	3.543775	4.137077	4.093073	3.264346	3.47885	3.37981	3.428436	4.179944	3.847821	2.730577
NUB1	1559732_at	2.194359	2.162215	2.138458	2.144838	2.178147	2.199393	2.155212	2.160146	2.146624	2.139441	2.145619	2.156339	2.136107
CHPT1	1559739_at	7.722503	7.703673	6.948951	6.993939	7.046732	7.283528	7.6064	8.396818	7.917768	8.135848	7.549985	7.897397	4.154938
ZNF718	1559746_a_at	2.71718	2.616149	2.604385	2.646393	2.722791	2.666882	2.658828	2.670901	2.65664	2.639299	2.659932	2.710783	2.536686
SPG11	1559747_at	3.256733	3.857818	3.490895	3.746074	2.815636	2.761111	2.803685	2.811345	2.765971	2.788926	2.757888	2.884831	2.492207
ADAMTSL3	1559748_at	2.273208	2.259288	2.232313	2.238688	2.259149	2.248174	2.254964	2.253756	2.246518	2.225768	2.242495	2.248916	2.223295
LTB	1559754_at	2.951111	2.934141	2.916121	2.906345	2.928107	2.895493	2.931163	2.917398	2.927477	2.970091	2.908882	2.920383	2.776153
C21orf49	1559755_at	2.114782	2.106231	2.08239	2.088742	2.101932	2.094211	2.104547	2.105033	2.092141	2.085237	2.093559	2.603451	2.040797
DKFZp667F	1559756_at	3.084529	2.913319	2.867844	2.947721	3.255998	2.875321	2.914562	3.125894	2.967514	2.882943	2.995014	3.077521	2.656878
LOC401220	1559760_at	2.598179	2.544807	2.49721	2.534227	2.581762	2.513666	2.544966	2.556465	2.543937	2.531752	2.544261	2.558344	2.38132
ZC3H12C	1559763_at	3.495613	3.414023	3.313801	3.375618	3.594009	3.389818	3.413067	3.414649	3.403468	3.392774	3.414232	3.524419	3.218164
LOC286254	1559765_a_at	2.333944	2.32179	2.28772	2.303774	2.353076	2.296223	2.322264	2.310059	2.322306	2.307938	2.30582	2.315816	2.205059
TPPP2	1559785_at	2.460382	2.620858	2.412238	2.418406	2.443815	3.272719	2.413219	2.422336	2.407603	2.393646	2.406042	2.41064	2.287506
ZNF781	1559789_a_at	2.517645	2.47858	2.430944	2.468307	2.503667	2.474596	2.479055	2.482712	2.469817	2.44453	2.4734	2.551451	3.426831
LOC646241	1559790_at	2.491689	2.466067	2.444404	2.44815	2.474243	2.450009	2.452476	2.462895	2.4457	2.44869	2.444232	2.456771	2.343871
EBF3	1559796_at	2.125938	2.099303	2.0739	2.079799	2.100877	2.081857	2.095772	2.096976	2.083921	2.074658	2.080596	2.091544	3.393052
LOC440839	1559810_at	2.387542	2.352195	2.31892	2.347662	2.365551	2.33315	2.356669	2.36139	2.346734	2.340092	2.342211	2.35429	2.269437
ATG10	1559820_at	7.603289	7.50684	6.623516	7.074495	7.544604	7.853416	3.598174	4.41454	3.962612	3.750149	4.808738	4.914775	4.012394
LOC644215	1559822_s_at	6.456363	6.279646	6.83298	6.992683	7.028291	6.994761	6.618271	6.61054	6.195619	6.054364	6.709239	6.998162	5.883074
LOC401074	1559826_a_at	1.539376	1.527794	1.517077	1.516196	1.528143	1.513605	1.531655	1.526437	1.525267	1.520296	1.51466	1.520358	1.51234
LOC401074	1559827_at	2.216449	2.227402	2.186968	2.17841	2.21244	2.169091	2.31011	2.341262	2.183434	2.322199	2.770054	2.198356	2.318983
C10orf73	1559828_at	2.597741	2.555878	2.532054	2.545521	2.594062	2.541973	2.558015	2.569891	2.555436	2.553385	2.554122	2.570948	2.448946
ARHGEF12	1559833_at	3.109843	3.005619	2.909323	2.984265	3.103857	2.963831	3.017229	2.999371	3.013828	2.979261	3.033726	3.075481	2.686941
TBX18	1559839_at	3.047633	3.012105	2.858267	2.995097	3.09416	2.982021	2.993328	3.015306	2.999586	2.986377	3.012619	3.010604	2.652477
TBX18	1559840_s_at	2.582369	2.550971	2.500727	2.535734	2.565154	2.538652	2.543471	2.566183	2.53913	2.529961	2.541549	2.560655	2.445465
NSUN4	1559848_at	4.006337	3.91273	3.802824	3.822188	4.663571	4.054201	3.882004	3.870927	3.874564	3.7428	3.853276	4.300101	3.270883
ZNF605	1559849_at	3.655791	3.349196	3.33233	3.435004	3.928526	3.422952	3.443337	3.44642	3.478943	3.424907	3.608484	3.666614	2.97387
MLL	1559856_s_at	3.190886	3.10928	2.991019	3.130582	3.199175	3.094845	3.127419	3.123932	3.133523	3.11382	3.122727	3.14124	2.77329
COPA	1559862_at	3.969757	3.955586	3.732558	3.80749	3.902591	3.682346	3.77426	3.871881	3.833746	3.811909	3.855368	3.632369	3.316027
AFG3L1	1559863_a_at	1.65181	1.644486	1.621892	1.629218	1.64033	1.633317	1.637565	1.638115	1.637677	1.621218	1.623807	1.633137	1.596066

LCN6	1559864_at	2.327675	2.300081	2.264669	2.291076	2.316654	2.281907	2.286671	2.298467	2.287336	2.272075	2.284489	2.295489	2.188031
ZNF12	1559881_s_at	4.292793	4.082107	5.206769	4.679167	3.902218	3.912539	5.167394	3.544376	5.828942	5.908276	3.919383	3.891818	6.423946
SAMHD1	1559882_at	5.88858	5.902869	5.110737	5.167444	6.372648	6.752783	2.21064	4.021637	2.434077	2.96175	3.758422	4.244682	1.989653
SAMHD1	1559883_s_at	2.542988	2.519313	2.501621	2.50015	2.527277	2.488601	2.5169	2.516491	2.520372	2.495198	2.504773	2.512673	2.484297
RP11-145E5	1559884_at	2.361998	2.327576	2.288382	2.310999	2.348735	2.308011	2.316594	2.329359	2.313454	2.301272	2.313844	2.325271	2.20191
ABI1	1559890_a_at	2.76248	2.704493	2.644925	2.698959	2.755453	2.670776	2.703111	2.717535	2.702465	2.677098	2.701938	2.72973	2.548582
HMGA2	1559891_at	2.220055	2.212124	2.132636	2.446425	2.216726	2.187682	2.20117	2.277825	2.20423	2.199731	2.197901	2.289012	2.18005
CCDC75	1559893_at	3.65803	3.361066	3.318166	3.47829	3.636965	3.688436	3.451061	3.543731	3.502321	3.518149	3.918527	4.334492	3.355124
C21orf34	1559901_s_at	4.586543	4.675785	5.244912	6.117275	5.43241	5.815173	3.63258	4.015771	3.627332	3.619998	3.933233	3.912804	3.588398
MKL1	1559902_at	2.630936	2.599335	2.566293	2.585139	2.619422	2.582389	2.59046	2.602455	2.58975	2.838701	2.591914	2.595795	2.506508
TIMM17B	1559909_a_at	1.849559	1.841918	1.82127	1.822015	1.853031	1.831377	1.840478	1.837351	1.835347	1.829803	1.826025	1.830012	1.778084
LOC56756	1559914_at	2.315689	2.287209	2.266934	2.267876	2.299785	2.266846	2.281356	2.288106	2.272266	2.265752	2.266531	2.283697	2.188233
CECR4	1559920_a_at	2.003537	1.988828	1.973162	1.975183	1.98877	1.969283	1.985433	1.985897	1.976769	1.965701	1.968206	1.980028	1.919255
PECAM1	1559921_at	2.076657	2.061739	2.036028	2.040883	2.053774	2.044464	2.05872	2.056873	2.053573	2.044649	2.038528	2.051998	1.981092
LOC728353	1559926_at	2.540805	2.534594	2.516313	2.506105	2.529975	2.532927	2.529264	2.530126	2.516741	2.518148	2.51409	2.52624	2.363008
LOC728353	1559927_a_at	3.162736	3.070994	3.014897	3.0626	3.152974	3.075324	3.077026	3.067649	3.068338	3.060992	3.056279	3.114473	2.785348
PAPPA	1559928_at	2.3786	2.355844	2.315392	2.334812	2.365722	2.333652	2.345048	2.35374	2.338736	2.327901	2.341514	2.349663	2.232655
FLJ16124	1559939_at	2.659305	2.628179	2.587468	2.608754	2.662915	2.610263	2.616464	2.624075	2.574647	2.603998	2.606693	2.658376	2.497846
FLJ16124	1559940_s_at	3.391103	3.35545	3.330776	3.334979	3.506033	3.17947	3.303862	3.354145	3.367914	3.349675	3.397036	3.461486	2.860234
MDFIC	1559942_at	2.378949	2.360239	2.319327	2.344429	2.509575	3.020881	2.338717	2.353862	2.339572	2.338917	2.474672	2.355778	3.036907
RUVBL2	1559945_at	2.190707	2.152642	2.124563	2.133099	2.151128	2.132576	2.144496	2.148622	2.137453	2.135758	2.130198	2.146428	2.076987
RUVBL2	1559946_s_at	8.834729	8.920685	8.562037	8.730527	9.238058	9.49051	10.08723	10.86868	10.26194	10.3903	10.78844	10.98408	9.255091
FAM66C	1559950_at	3.658041	3.533415	3.477984	3.518311	3.72211	3.344469	3.446378	3.517439	3.52796	3.424711	3.52324	3.686305	3.46123
FAM66C	1559952_x_at	3.390464	3.367168	3.25462	3.329652	3.496664	3.349458	3.338227	3.345934	3.363242	3.30022	3.34541	3.420132	3.164078
DDX42	1559953_at	3.407492	3.268606	3.278384	3.334594	3.488033	3.298057	3.291935	3.340752	3.31379	3.278453	3.397378	3.432745	2.88779
DDX42	1559954_s_at	4.172211	3.761586	4.210384	4.287569	4.112594	3.70488	5.688201	4.282959	6.091872	5.540817	4.555354	4.589492	7.748232
SYT7	1559956_at	3.824038	3.634582	3.540144	3.659011	3.821274	3.626554	3.691402	3.698971	3.703212	3.661232	3.759709	3.843414	3.06352
LOC642852	1559957_a_at	4.175887	4.122376	3.4553	4.003296	4.194066	4.945949	4.34001	5.384181	4.740481	4.176304	6.575161	5.074822	6.256414
FLJ38717	1559964_at	6.758022	6.913987	6.685441	6.459819	6.324215	5.887298	6.116409	6.465176	6.463927	6.655345	6.815339	6.398409	6.263023
LOC730034	1559970_at	2.49492	2.461475	2.431083	2.447358	2.486205	2.451485	2.456151	2.46659	2.453311	2.438659	2.454843	2.46982	2.374301
BSDC1	1559971_at	4.863728	4.779292	3.818294	4.217004	3.992897	4.237274	4.528578	5.227435	5.108086	4.685741	4.596391	4.090542	3.338949
BTG1	1559975_at	3.145279	3.258084	3.241246	3.241419	3.599234	3.913809	2.965328	3.045707	2.948806	3.12115	3.058275	3.164304	2.761353
SLC25A34	1559977_a_at	3.060282	3.657859	4.74788	5.78537	5.009532	4.001226	2.945916	3.037674	2.494676	2.748001	3.037116	2.833876	3.05523
SYF2	1559979_at	3.388033	2.610106	2.779468	2.756019	2.800275	2.970241	2.743947	2.781821	2.768751	2.762682	2.665964	2.896407	2.554188
ZNF7	1559980_at	3.267414	3.178117	3.09881	3.186489	3.392989	3.147361	3.19911	3.189284	3.196851	3.168644	3.233605	3.263809	2.792877
AKR1CL2	1559982_s_at	2.914051	2.929371	2.819513	2.877461	3.382306	3.647681	2.846107	2.876778	3.252514	2.738912	2.873597	2.883903	2.637131
ZNF483	1559988_at	2.473315	2.448131	2.408972	2.42838	2.460217	2.420431	2.437397	2.448064	2.433232	2.421625	2.43121	2.44609	2.29851
TPM4	1559989_at	2.697385	2.690282	2.523503	2.579672	2.682892	2.713616	2.680364	2.680772	2.590578	2.19705	2.662073	2.671957	2.589642

SFXN3	1559993_at	4.099401	3.939154	4.003383	3.838517	4.399142	4.700689	4.116416	4.487958	3.980197	3.304795	4.114439	4.158005	3.413335
SAMD14	1559995_at	2.413544	2.414117	2.406727	2.523757	2.413234	2.390998	2.40375	2.736868	2.41155	2.407886	2.401177	2.408862	2.222969
SAMD14	1559996_s_at	2.159236	2.140119	2.11413	2.120437	2.142404	2.11801	2.129069	2.13954	2.123942	2.116489	2.123329	2.139028	2.052866
SAMD14	1559997_x_at	2.713608	2.706196	2.686932	2.693091	2.702179	2.691514	2.703447	2.70326	2.694623	2.692606	2.685099	2.731112	2.468999
LOC389634	1560001_at	2.863036	2.858613	2.816032	2.832028	2.850927	2.815072	2.852199	2.853199	2.835714	2.841647	2.837506	3.096732	2.751789
FAM27A	1560002_at	2.388731	2.366236	2.326719	2.348585	2.378528	2.336944	2.354633	2.367448	2.350653	2.343706	2.351048	2.36214	2.288431
LOC646762	1560006_a_at	2.708761	2.649204	2.622871	2.639852	2.719263	2.61869	2.656146	2.675013	2.654846	2.663572	2.660935	2.67404	3.251535
LOC645984	1560007_at	2.717347	2.63319	2.652217	2.646561	2.681859	2.666804	2.670224	2.674937	2.662282	2.652006	2.661245	2.671927	2.517628
FLJ32063	1560010_a_at	2.346665	2.318053	2.297305	2.297836	2.319725	2.282028	2.313018	2.306834	2.303986	2.293177	2.330036	2.311728	2.211325
PSCA	1560011_at	2.393955	2.383069	2.349519	2.369128	2.397627	2.360973	2.382355	2.437873	2.37837	2.365966	2.384659	2.420572	2.253782
PDXDC1	1560013_at	6.299014	6.030483	6.65044	6.806013	6.650642	6.427612	5.110692	4.990914	4.500842	4.177828	5.1784	5.180996	4.445912
PDXDC1	1560014_s_at	6.256689	5.743505	6.611648	6.701789	7.057206	7.349645	4.293601	4.491216	4.164688	3.374863	4.795937	4.444379	4.160677
TMTC3	1560017_at	2.650727	2.632946	2.609052	2.60641	2.647146	2.690673	2.624866	2.626506	2.706861	2.568015	2.608949	2.625799	2.469946
ARPP-21	1560018_at	2.32075	2.291884	2.246911	2.276602	2.31265	2.270181	2.280372	2.290232	2.276489	2.265427	2.402224	2.286172	2.26098
MGC11082	1560019_at	2.578822	2.54908	2.511612	2.53729	2.561333	2.51753	2.537525	2.542915	2.541745	2.510648	2.53275	2.5434	2.393025
DNAJC13	1560020_at	2.747642	2.643239	2.520746	2.624659	2.94904	2.624142	2.616729	2.633895	2.624056	2.593253	2.682052	2.707125	2.727694
C11orf57	1560028_at	2.549259	2.50845	2.43773	2.500809	2.511612	2.492008	2.494079	2.505469	2.478124	2.473283	2.487942	2.50015	2.384673
C11orf57	1560029_a_at	3.229765	3.172528	3.025939	3.163166	3.297885	3.155369	3.120889	3.08179	3.154242	3.095811	3.190531	3.167344	3.291511
LOC283692	1560030_at	2.193858	2.170132	2.147629	2.154498	2.205913	2.151439	2.163222	2.160693	2.158283	2.146373	2.160117	2.168259	2.075889
FRMD4A	1560031_at	7.936542	7.75933	6.743337	6.541321	6.699253	6.76169	8.43947	8.371548	8.363272	8.796734	8.438201	7.974246	7.009001
MCCC2	1560033_at	2.988397	2.882258	2.820576	2.873696	3.193521	2.84469	2.889416	2.893949	2.883345	2.856539	2.97494	2.942824	2.621034
FLJ33590	1560035_at	1.893131	1.887349	1.868973	1.870891	1.88234	1.866502	1.882938	1.878734	1.87546	1.870408	1.863999	1.875778	1.839817
FAM82A	1560042_at	3.355161	2.656043	3.914094	3.062964	4.20264	2.694263	2.654268	2.795804	2.646131	2.839591	2.501243	2.590958	2.329517
CYB5R1	1560043_at	3.054237	2.851925	2.778509	2.866813	2.893833	2.871148	2.876892	2.878086	2.864961	2.853253	2.866776	2.87253	2.601224
LOC439962	1560047_s_at	3.124918	3.049478	2.987979	3.037594	3.071631	3.004074	3.037704	3.150076	2.991631	3.000117	3.031799	3.078397	2.641482
LOC399900	1560058_at	4.260256	4.123182	4.23904	4.058359	4.074833	3.932863	5.568459	5.274073	5.986583	5.743734	4.300572	6.053458	3.543763
VPS37C	1560059_at	2.795601	2.774293	2.634393	2.720009	2.799239	2.654413	3.190213	2.738467	2.738958	2.755061	2.743222	2.832049	2.653837
VPS37C	1560060_s_at	7.72179	7.643485	7.416785	7.285625	7.63402	8.010614	8.434475	9.084881	8.437828	8.530247	8.906876	9.05602	7.998753
SLC29A2	1560062_at	4.24728	4.538124	3.032253	3.250753	4.339725	4.277324	3.054874	3.184278	3.054214	2.764388	3.050863	3.104185	2.530787
PAIP2	1560065_at	2.096465	2.085066	2.052765	2.068579	2.093039	2.077667	2.083677	2.086137	2.076326	2.064198	2.074534	2.121128	2.031339
LOC389072	1560069_at	2.791433	2.70036	3.42422	3.53783	3.981489	2.551352	2.396251	2.512501	2.553513	2.547226	2.543541	2.436008	2.567968
PRKCA	1560074_at	2.518214	2.491176	2.42505	2.467433	2.501037	2.467359	2.480616	2.490592	2.474569	2.465657	2.477411	2.49976	2.298348
ZNF622	1560075_at	3.142113	3.001627	2.970058	3.00834	3.16824	3.000712	3.016532	3.050388	3.034993	2.988908	3.063974	3.109331	2.716837
LAMA3	1560078_at	2.683538	2.656593	2.621662	2.641417	2.683757	2.638613	2.657017	2.659323	2.640817	2.636982	2.641665	2.654332	2.505553
DIAPH1	1560080_at	3.171924	3.020781	2.965602	3.01754	3.208208	3.012206	3.012009	3.02333	3.037719	2.999149	3.064301	3.084715	2.7292
LOC90408	1560081_at	3.432979	3.3653	3.342955	3.783568	3.481776	3.807322	3.831957	3.41375	3.629549	3.643903	3.638789	3.40354	3.27169
LOC286208	1560089_at	5.231295	4.79606	4.840891	5.091594	5.80934	6.421136	4.669957	4.903512	4.342116	4.556583	5.066972	4.897647	3.979812
PHF21B	1560091_a_at	2.28207	2.264781	2.246026	2.239593	2.253069	2.246264	2.247835	2.259255	2.243972	2.247823	2.236934	2.256576	2.260261

FHAD1	1560097_at	2.079823	2.062072	2.04433	2.047235	2.076868	2.047259	2.058591	2.0598	2.050972	2.04255	2.044884	2.05445	2.009713
AKR1C2	1560098_at	2.456789	2.419896	2.378066	2.401088	2.433905	2.396545	2.411075	2.419075	2.407325	2.395747	2.404959	2.416651	2.275894
LOC339260	1560099_at	2.855582	2.786458	2.735886	2.780496	3.085319	2.753511	2.788558	2.796172	2.785919	2.763442	2.794038	2.820241	2.557189
DLX1	1560100_at	3.029823	3.021775	2.923646	3.002142	3.011845	3.007656	3.009778	3.021284	2.988556	3.006441	3.015789	3.089538	2.919741
SYDE2	1560101_at	3.901774	3.757556	4.342589	4.289572	3.85467	3.255101	3.002782	3.37645	3.188089	3.12426	3.648844	3.407422	3.442781
PTPRB	1560105_at	2.549619	2.529616	2.511677	2.511837	2.532975	2.517406	2.468782	2.479711	2.514268	2.506994	2.512373	2.520713	2.440821
LOC729231	1560111_at	3.453813	3.308906	3.280625	3.321983	3.518451	3.276271	3.33844	3.344899	3.345169	3.32057	3.360444	3.395863	3.055536
WDFY2	1560112_at	5.244843	4.816019	5.648775	5.608754	4.642109	4.100579	3.521857	3.953376	3.555812	3.629826	3.69619	4.060397	3.396464
KIAA1217	1560115_a_at	2.308058	2.279199	2.251777	2.275699	2.29797	2.277195	2.275956	2.279587	2.215517	2.26146	2.273771	2.281244	2.139841
NEDD1	1560116_a_at	5.418582	5.022431	5.227591	5.293882	5.263914	5.526904	7.234761	7.304973	7.301135	7.442436	7.432234	7.595659	6.664645
ABHD1	1560117_at	2.054968	2.064345	2.002044	2.008921	2.039879	2.015741	2.012702	2.024657	2.00832	1.997023	2.008326	2.015915	1.945883
OR7E140P	1560119_at	3.495924	3.450147	3.254158	3.37893	3.462539	3.43142	4.065616	4.935357	4.111168	4.542605	5.179887	4.571574	3.252862
LOC441108	1560128_x_at	6.003544	6.561798	5.62147	5.8739	6.140441	6.948978	4.402728	4.96719	4.751697	4.171571	4.784711	5.568442	4.679764
C1orf145	1560130_at	3.138066	3.007577	3.006175	3.024149	3.125838	3.055485	3.028388	3.044558	3.052877	3.021728	3.092241	3.094418	2.769064
TNRC15	1560133_at	6.159911	6.184701	6.148287	6.07929	6.107279	5.99662	5.97419	5.899425	5.994182	6.072334	5.850552	6.012323	4.912108
KIAA0226	1560138_at	3.603671	3.46056	3.515193	3.539296	3.690535	3.533129	3.53272	3.51915	3.49104	3.590944	3.576585	3.747983	2.97263
GRIK2	1560142_at	2.525127	2.479297	2.435911	2.461262	2.517584	2.449677	2.470482	2.477236	2.466831	2.449336	2.469499	2.523454	2.413071
MKLN1	1560145_at	7.31215	7.304069	6.917242	7.284252	7.6249	7.818406	6.514193	7.28831	6.805753	6.721532	7.271434	7.308994	5.420924
SLC29A2	1560149_at	2.385046	2.357868	2.348663	2.342148	2.426001	2.347772	2.351106	2.347556	2.348782	2.337607	2.344025	2.353509	2.259379
SLC29A2	1560151_x_at	2.006009	1.996451	1.964827	1.981886	1.99774	1.979473	1.991789	1.984759	1.984272	1.979273	1.976465	1.98971	1.941458
FRAS1	1560153_at	2.129364	2.110403	2.085084	2.092287	2.112672	2.093624	2.103176	2.110257	2.095484	2.087707	2.09014	2.104893	2.02886
CCNB2	1560161_at	2.877564	2.791896	2.766937	2.794881	2.87131	2.780413	2.806115	2.816223	2.801602	2.783686	2.800728	2.825976	2.561366
FLJ40606	1560162_at	1.989288	1.950258	1.928502	1.928928	1.946523	1.935616	1.93739	1.937381	1.937186	1.931224	1.928848	1.954554	1.858969
LOC646168	1560168_at	2.926186	2.828792	2.766605	2.82364	2.914381	2.841797	2.835803	2.84395	2.834384	2.806836	2.842077	2.905432	2.649363
INTS10	1560172_at	4.589638	4.303922	6.424708	6.788723	5.010207	5.098472	4.282961	4.341754	4.658973	4.25238	4.494877	4.563995	3.9339
SPAG16	1560174_at	2.818463	2.725657	2.692836	2.711127	2.772823	2.701904	2.716517	2.728391	2.717235	2.687732	2.713007	2.746159	2.51992
PPP4R1L	1560175_at	2.939152	2.875343	2.809773	2.869888	2.940605	2.876381	2.863666	2.887558	2.87902	2.879805	2.877642	2.924363	2.648902
PPP4R1L	1560176_s_at	2.958699	2.88565	2.887786	2.885979	2.92991	2.863791	2.90208	2.90557	2.893051	2.886495	2.916146	3.302139	2.744047
CCDC66	1560177_at	3.384465	3.317953	3.266727	3.315575	3.373751	3.308745	3.330304	3.344673	3.329241	3.695411	3.325839	3.348206	3.195425
GALNT14	1560180_at	2.215706	2.189636	2.170021	2.173042	2.187983	2.173005	2.180321	2.18763	2.175039	2.167953	2.169717	2.180875	2.068626
TMCO5	1560184_at	2.765207	2.670107	2.649934	2.673714	2.723605	2.667347	2.681887	2.689137	2.67843	2.648687	2.680161	2.698913	2.477032
C8orf70	1560197_at	2.862888	2.785505	2.730218	2.777567	2.850437	2.741626	2.785749	2.795014	2.786903	2.773125	2.785624	2.82015	2.58971
C14orf70	1560198_at	2.197194	2.176684	2.147772	2.16058	2.180102	2.156941	2.166177	2.174613	2.160181	2.153059	2.159264	2.169063	2.07638
FLJ11903	1560199_x_at	8.168594	8.367487	7.252297	7.704227	7.913572	9.074885	6.377112	6.710536	5.387762	5.388288	7.378691	6.699025	6.576106
ZNF713	1560201_at	3.949874	3.601962	3.628877	3.865412	4.060788	4.170999	3.795566	4.133608	3.773347	3.645167	4.130006	4.574447	4.536115
LOC338739	1560202_at	3.886402	3.533335	3.571985	3.665351	4.00952	3.593777	3.666424	3.689078	3.69019	3.602176	3.827068	3.861406	3.07942
NT5DC4	1560204_at	3.304934	3.217522	3.075659	3.203936	3.345267	3.187798	3.215188	3.160868	3.343411	3.192895	3.239802	3.271974	2.805101
LOC644660	1560207_at	3.324461	3.204861	3.072437	3.188446	3.460907	3.326778	3.211572	3.173584	3.193542	3.179225	3.289029	3.400416	2.926808

LOC642032	1560213_at	2.067234	2.135331	2.042333	2.040578	2.060889	2.059776	2.05061	2.055875	2.043008	2.045603	2.043919	2.049858	1.975281
ASB3	1560219_at	1.873533	1.854975	1.845919	1.849823	1.887718	1.848155	1.859449	1.862071	1.852419	1.846812	1.84509	1.856152	1.803843
CBY1	1560220_a_at	2.104652	2.079421	2.059322	2.067452	2.094698	2.065185	2.076403	2.080306	2.068936	2.061715	2.065399	2.07855	2.039368
AHCTF1	1560224_at	2.462021	2.437369	2.42054	2.419319	2.44425	2.404786	2.431562	2.435555	2.422931	2.414611	2.416517	2.432305	2.615749
CNR1	1560225_at	2.432134	2.3959	2.353333	2.37396	2.426767	2.373328	2.385066	2.395944	2.382419	2.37017	2.382932	2.391917	2.318083
GDPD1	1560227_at	3.758606	3.581666	3.331221	3.472355	3.626435	3.571266	3.544426	3.576047	3.570334	3.55516	3.567834	3.724479	3.436176
SNAI3	1560228_at	2.237894	2.228442	2.21047	2.210775	2.262541	2.215812	2.227199	2.227045	2.218845	2.214019	2.216544	2.21917	2.121062
LOC284242	1560250_s_at	2.440707	2.439663	2.407383	2.387151	2.424269	2.376297	2.394142	2.398479	2.386701	2.377126	2.389638	2.441977	2.343366
LOC645485	1560251_at	2.543209	2.507345	2.463742	2.490271	2.533337	2.470424	2.498785	2.508217	2.494358	2.484742	2.493845	2.510218	2.347012
LHX9	1560253_at	2.384965	2.347928	2.310934	2.331569	2.37466	2.322187	2.340471	2.351735	2.337583	2.322003	2.332329	2.351524	2.201744
C10orf31	1560255_at	1.959735	1.947625	1.926921	1.932365	1.950053	1.937583	1.942372	1.944548	1.934056	1.929923	1.930526	1.939917	1.89595
LOC285593	1560260_at	2.762301	2.728981	2.654441	2.703268	2.77197	2.675891	2.716874	2.726699	2.712691	2.690178	2.713794	2.749199	2.506508
GRIK2	1560265_at	3.605653	3.445159	3.418988	3.484097	3.706747	3.797656	3.434731	3.467839	3.477053	3.406208	3.549738	3.622096	4.373587
WTAP	1560274_at	2.902162	2.83869	2.758572	2.826901	2.900978	2.832577	2.859897	3.011372	2.853906	2.829308	2.866027	2.951921	2.66948
TMEM44	1560275_at	3.186929	3.481404	2.784023	3.164746	3.172829	4.273629	2.837453	4.050766	3.172464	2.968289	3.137989	4.678392	2.963381
LOC283403	1560276_at	2.146885	2.114166	2.089373	2.094943	2.133399	2.095945	2.10366	2.113766	2.097409	2.085959	2.09718	2.109248	2.034956
LOC283403	1560277_a_at	2.55584	2.548498	2.467377	2.523099	2.562303	2.533513	2.515773	2.537898	2.522194	2.518148	2.571419	2.532771	2.368178
LOC221122	1560278_at	2.621298	2.608431	2.590439	2.596521	2.636805	2.592343	2.606411	2.602347	2.604465	2.572092	2.660745	2.612624	2.593512
LOC221122	1560279_a_at	2.093139	2.076709	2.05327	2.058203	2.078332	2.059838	2.068916	2.076639	2.061393	2.052601	2.057224	2.080279	2.000498
TMEM95	1560281_a_at	2.557352	2.486739	2.477775	2.490825	2.638466	2.839477	2.505143	2.512989	2.505235	2.490991	2.50134	2.587426	2.380892
RP11-321G	1560291_at	3.762851	3.242127	2.947698	3.006178	3.086299	3.005145	3.00543	3.005003	2.956472	2.909942	2.961537	2.999406	2.653743
TMCO4	1560292_a_at	2.030973	2.015277	1.994099	1.998392	2.016447	1.999323	2.010237	2.012896	2.008609	1.998845	1.997809	2.009094	1.935452
CYP19A1	1560295_at	2.352955	2.31151	2.283282	2.288238	2.346042	2.313946	2.316049	2.317643	2.30363	2.292409	2.380393	2.351698	2.251122
LOC728656	1560300_a_at	1.588697	1.578994	1.559719	1.564296	1.57756	1.563842	1.574232	1.575112	1.569363	1.562903	1.562266	1.568942	1.539099
GLCCI1	1560315_at	2.677609	2.614319	2.609076	2.60416	2.66017	2.600671	2.612867	2.624545	2.607338	2.595939	2.609359	2.637812	2.435928
GLCCI1	1560316_s_at	2.157202	2.14423	2.117959	2.126205	2.143151	2.124418	2.142596	2.144008	2.134835	2.132522	2.129593	2.245997	2.141272
HIP1	1560317_s_at	2.861119	2.827057	2.777947	2.808124	2.827751	2.807105	2.825859	2.855947	2.814716	2.803139	2.808334	2.82042	2.670979
ARHGAP29	1560318_at	3.443531	3.12203	3.135985	3.500733	3.489252	3.723872	3.116235	3.118147	3.10567	3.107292	3.16804	3.128184	3.045699
UNQ2963	1560320_a_at	3.550321	3.486969	3.434326	3.470982	3.539024	3.52621	3.477328	3.482644	3.479479	3.448183	3.461256	3.475432	3.249113
RBMS3	1560322_at	2.661225	2.562158	2.477623	2.488922	2.751605	2.562083	2.520863	2.490412	2.549042	2.500371	2.92273	2.553188	2.480098
PCDH15	1560330_at	2.924853	2.822668	2.759353	2.813356	2.896333	2.7799	2.837569	2.844724	2.832036	2.800402	2.835257	2.877353	2.592864
MEGF11	1560334_at	2.252607	2.222887	2.183955	2.206133	2.236217	2.205497	2.210482	2.219737	2.20747	2.201702	2.205548	2.217596	2.157627
LOC286184	1560337_at	2.733386	2.691544	2.646148	2.682422	2.727973	2.681966	2.683812	2.693444	2.680857	2.666399	2.681315	2.694605	2.517715
NAP1L4	1560339_s_at	6.6318	6.684116	6.7635	6.334831	5.842577	5.658038	9.120951	7.114364	8.865838	8.951087	6.942937	7.30792	9.633419
LOC441212	1560340_s_at	3.039896	3.003476	3.001918	3.007312	3.187064	2.961514	2.989488	3.000474	2.992116	2.962146	3.003439	3.036796	2.888051
RGNEF	1560348_at	2.077418	2.065769	2.041821	2.049804	2.071201	2.05103	2.107238	2.060815	2.046844	2.043846	2.038679	2.053283	2.008325
CCDC127	1560354_at	2.473111	2.45037	2.420749	2.433816	2.454763	2.407951	2.439167	2.44809	2.434125	2.426688	2.431815	2.44207	2.351803
PHKA2	1560358_at	2.744354	2.715062	2.63297	2.691027	2.729178	2.692924	2.699909	2.707941	2.69838	2.69051	2.698317	2.706957	2.617487

PELO	1560359_at	5.381643	5.467143	6.416389	6.385036	6.112288	5.570019	4.503177	4.601819	4.136452	4.312416	4.007677	4.320144	4.114437
ANKH	1560369_at	2.260308	2.227297	2.193691	2.21862	2.241504	2.215969	2.223621	2.228381	2.219821	2.210046	2.220395	2.284836	2.118104
ANKH	1560370_x_at	2.240004	2.199064	2.185577	2.193165	2.239988	2.193293	2.203097	2.210787	2.196867	2.18946	2.198603	2.264374	2.098448
LOC401321	1560371_at	2.112017	2.098789	2.083619	2.081588	2.09842	2.080005	2.093831	2.098147	2.084569	2.088541	2.081933	2.090024	2.073564
C21orf41	1560378_at	2.30209	2.278459	2.25319	2.259044	2.2844	2.258114	2.265117	2.27709	2.261886	2.254023	2.261614	2.271753	2.18417
LOC497256	1560380_at	2.468938	2.422806	2.375874	2.410072	2.486502	2.394193	2.418887	2.430668	2.418045	2.39916	2.414548	2.436749	2.264615
GAB1	1560382_at	2.263457	2.262011	2.234373	2.234258	2.255949	2.237218	2.241989	2.250176	2.236884	2.226882	2.231426	2.243682	2.176693
KLHL6	1560396_at	2.079011	2.06425	1.974311	2.041576	2.269505	2.965817	2.058775	2.064623	2.056875	2.048814	2.042892	2.413292	1.810961
KLHL6	1560397_s_at	2.729064	2.707025	2.691885	2.68965	3.011801	2.823381	2.706586	2.715942	2.69792	2.68123	2.753718	3.202898	2.622355
OTUD7A	1560399_a_at	2.685864	2.670888	2.597914	2.653751	2.680457	2.653128	2.6645	2.667444	2.653399	2.648413	2.650232	2.678464	2.541659
GAS5	1560402_at	3.813179	3.537208	3.621587	3.831238	3.777227	3.712137	3.752642	3.824855	3.823149	3.98026	3.747609	3.820233	3.721178
ATPBD4	1560404_a_at	3.13289	2.979405	2.906086	2.98047	3.21339	2.946481	2.983481	2.99413	3.005495	2.97236	3.026289	3.085566	2.684598
MARK1	1560407_at	2.945719	2.900802	2.827205	2.883317	2.946552	2.842813	2.886646	2.794288	2.872329	2.840506	2.888852	2.91893	2.83448
LOC339788	1560413_at	2.172075	2.154984	2.119048	2.136488	2.163569	2.135718	2.141877	2.149234	2.13752	2.126359	2.132798	2.147478	2.047159
DNAH11	1560416_at	2.367211	2.307586	2.270453	2.292329	2.3205	2.291265	2.298124	2.307712	2.293882	2.280846	2.294949	2.312644	2.200559
C6orf182	1560418_at	2.81255	2.751102	2.727693	2.762042	2.87771	2.723389	2.767607	2.775532	2.767342	2.748119	2.767859	2.800277	2.624028
CHD4	1560419_at	3.19361	3.141172	3.051199	3.112366	3.351577	3.071459	3.129736	3.136442	3.1264	3.096943	3.141063	3.18808	2.909956
C12orf55	1560426_at	2.701516	2.647759	2.596478	2.64944	2.684687	2.622832	2.650623	2.665171	2.647574	2.635129	2.652393	2.666202	2.501913
NKPD1	1560430_at	2.067364	2.045166	2.018679	2.032251	2.049679	2.055074	2.025056	2.028644	2.029611	2.02157	2.025558	2.038895	2.03604
PGM5P1	1560431_at	2.650007	2.608285	2.560159	2.598931	2.675947	2.569851	2.610836	2.622345	2.606811	2.589771	2.613414	2.61705	2.43354
CLRN1OS	1560432_at	2.142845	2.133293	2.10738	2.113703	2.128987	2.116093	2.122476	2.126152	2.114501	2.109376	2.110223	2.120559	2.03912
CLTA	1560434_x_at	2.016648	2.005474	1.986301	1.989844	2.01969	2.132869	2.000915	2.002628	1.993812	1.909772	1.984112	1.996409	1.951969
hCG_20403	1560439_at	1.99351	2.454677	2.235115	2.237312	2.24554	2.230111	2.255944	2.24529	2.282596	2.263203	2.236092	2.238672	2.153304
ARHGEF1	1560445_x_at	7.108164	7.257301	6.373665	6.617871	7.141458	7.553318	6.584395	7.01783	6.60584	6.45744	7.147972	6.789979	5.875733
LOC340037	1560448_at	2.503074	2.477595	2.460008	2.464086	2.474113	2.466356	2.468455	2.472475	2.458002	2.456001	2.460615	2.468018	2.378418
LOC339166	1560455_at	1.972148	1.953707	1.930181	1.93399	1.951741	1.933088	1.94472	1.950972	1.935979	1.929186	1.935319	1.943713	1.881189
LSDP5	1560456_at	2.621325	2.569976	4.110581	4.226771	3.498494	4.772022	2.465149	2.551764	2.264655	2.518476	2.735331	2.543786	2.071251
LSDP5	1560457_x_at	2.656251	2.677073	3.71302	3.605992	3.56275	5.289472	2.45752	2.749625	2.224231	2.219594	2.464231	2.66737	2.004717
CAPS2	1560458_s_at	3.459018	3.250846	3.210336	3.292641	3.553957	3.200333	3.304658	3.286784	3.312949	3.258701	3.37331	3.447934	2.874434
PAQR9	1560467_at	2.411163	2.392923	2.384437	2.390311	2.434144	2.794107	2.396229	2.399863	2.383843	2.399505	2.380053	2.391847	2.257963
NR5A2	1560469_at	5.342984	5.362378	4.686603	3.563373	3.862304	3.461655	4.777441	3.528472	5.675957	5.709926	3.412707	3.535499	3.266574
LOC338588	1560472_at	1.877693	1.870059	1.854326	1.854246	1.869473	1.857459	1.866938	1.86678	1.854275	1.855177	1.847776	1.858482	1.831209
SAMD11	1560477_a_at	1.927167	2.061802	1.567432	1.903391	1.91528	1.905847	1.913438	1.769778	1.847662	1.904174	2.142259	1.907588	2.439699
HIVEP1	1560485_at	2.126626	2.113415	2.098244	2.099119	2.107565	2.096946	2.08802	2.110911	2.099779	2.094566	2.091494	2.104235	2.015844
STXBP3	1560486_at	4.030589	3.792279	3.688146	3.821306	4.131915	3.744652	3.939043	3.830589	3.913327	4.095611	4.156719	4.014325	3.451072
FLJ45224	1560488_at	2.51397	2.504257	2.452517	2.487925	2.506352	2.483755	2.497439	2.489919	2.500836	2.480534	2.485485	2.492547	2.429762
FAT3	1560490_at	2.190918	2.174436	2.145789	2.157773	2.186118	2.146159	2.161814	2.171578	2.158429	2.15263	2.155267	2.16731	2.121107
CPXCR1	1560493_a_at	2.840715	2.813082	2.733303	2.936825	2.914181	2.74702	2.77618	2.782988	2.795817	2.782261	2.822713	2.803475	2.467729

CPXCR1	1560494_a_at	2.800211	2.734877	2.671963	2.716919	2.789468	2.692119	2.732414	2.740888	2.733874	2.703832	2.74512	2.763273	2.515937
C14orf64	1560499_at	2.768851	2.720184	2.689069	2.704842	2.740403	3.232304	2.69945	2.714968	2.700935	2.678538	2.70621	2.71011	2.440244
DGAT2L3	1560507_at	2.475198	2.452388	2.428355	2.435288	2.448643	2.420052	2.513779	2.455507	2.44085	2.43365	2.436605	2.454613	2.36921
LOC400568	1560513_at	1.982394	1.966246	1.943247	1.947396	1.977705	1.948307	1.959974	1.96541	1.951063	1.944828	1.945836	1.959442	1.893643
LOC285205	1560514_at	3.389899	3.276599	3.17892	3.267368	3.349658	3.263006	3.28386	3.29266	3.261842	3.254128	3.300756	3.457919	2.908736
LOC401312	1560520_at	2.067224	2.052716	2.031701	2.035377	2.052624	2.034669	2.047542	2.105056	2.039529	2.039021	2.034132	2.042987	2.005789
LOC201477	1560522_at	2.261349	2.21521	2.174926	2.182431	2.215604	2.197663	2.211716	2.445864	2.199863	2.187309	2.196662	2.206988	2.131313
LOC400581	1560524_at	2.377283	2.355695	2.313892	2.335048	2.369067	2.337156	2.344197	2.354871	2.339871	2.327948	2.337631	2.349087	2.224058
PR47	1560526_at	2.796936	2.736045	3.486595	3.050974	2.737914	2.74592	2.742979	2.734816	2.741629	2.715018	2.749898	2.709608	2.66229
NF-E4	1560527_at	2.494427	2.478952	2.457099	2.463847	2.478718	2.4633	2.472894	2.476212	2.465412	2.461955	2.461168	2.481944	2.406614
LCE1B	1560531_at	2.404704	2.374004	2.328186	2.355695	2.386246	2.356184	2.358005	2.369294	2.354957	2.356623	2.563185	2.35327	2.333423
MCM3APAS	1560542_at	3.285784	3.118947	3.041589	3.133029	3.296522	3.133338	3.17081	3.18103	3.166848	3.144491	3.202363	3.242279	2.778389
TIAF1	1560553_at	2.481981	2.728704	2.729066	2.444851	2.458833	2.443565	2.420624	2.461455	2.455306	2.426794	2.445362	2.451318	2.376644
C1orf145	1560554_a_at	2.298393	2.268042	2.240447	2.254598	2.269712	2.242214	2.261032	2.265378	2.251084	2.235482	2.247809	2.262896	2.170092
PLEKHA8	1560556_a_at	2.713174	2.597661	2.5662	2.58566	2.634846	2.56506	2.599002	2.710979	2.593919	2.6	2.623728	2.611415	2.599448
C9orf80	1560558_at	2.96879	2.828799	2.843966	2.819344	3.23189	2.820745	2.820144	2.840472	2.825139	2.797056	2.841989	2.856865	2.599262
ZNF677	1560562_a_at	3.305772	3.181073	2.933548	2.929133	2.968151	2.922286	3.286789	3.084885	3.049579	3.347701	4.140377	3.063658	6.126113
PCDH20	1560566_at	2.2529	2.22451	2.193713	2.204069	2.235329	2.202049	2.215633	2.223846	2.211218	2.202436	2.211378	2.220973	2.118376
LOC387895	1560573_at	1.920666	1.901982	1.88576	1.893788	1.907288	1.8919	1.895665	1.901008	1.89683	1.886057	1.886176	1.914211	1.841136
PRDX5	1560587_s_at	11.22496	11.17261	11.13714	11.12561	11.41398	11.22904	11.66659	11.36422	11.59431	11.69302	11.52917	11.88837	11.14992
CCDC123	1560599_a_at	2.95538	2.936328	2.786778	2.922063	2.937256	2.925504	2.932925	2.934467	2.921697	3.156643	2.919308	2.942782	2.905767
CRYZL1	1560609_at	3.255011	3.174529	3.039296	3.116596	3.287421	3.112304	3.168896	3.132108	3.141436	3.122629	3.193166	3.250375	2.923812
LOC255411	1560619_at	2.459854	2.43203	2.389937	2.413474	2.449497	2.401418	2.416536	2.432035	2.416227	2.404346	2.410974	2.469486	2.284579
KCNK15	1560620_at	2.013661	1.999816	1.978318	1.982024	1.999509	1.987146	1.997605	1.99828	1.983455	1.984976	1.979872	1.992545	1.92371
FLJ16124	1560628_at	3.175137	3.095816	3.017878	3.068835	3.156867	3.045349	3.08185	3.091864	3.079212	3.058417	3.441561	3.125043	2.869268
CALCOCO2	1560631_at	1.985594	1.97351	1.952531	1.958149	1.986364	1.957345	1.971494	1.972149	1.960122	1.951425	1.955668	1.963957	1.9339
D21S2089E	1560633_a_at	2.816721	2.781721	2.713868	2.756394	2.81934	2.755902	2.773901	2.785454	2.770698	2.75785	2.771594	2.795179	2.57683
TSPYL1	1560647_at	3.272539	3.072984	3.074539	3.370904	3.316397	3.253661	3.052345	3.095296	3.091671	3.095265	3.138702	3.180425	2.829855
TSPYL1	1560648_s_at	4.201428	4.155988	4.136748	4.698028	4.157603	3.912716	3.872837	3.94624	4.165055	4.040876	4.019985	3.922008	3.776609
FLJ37201	1560654_at	2.635396	2.610761	2.564755	2.594315	2.670801	2.550457	2.601171	2.604279	2.705664	2.572346	2.593815	2.62582	2.531449
TIE1	1560657_at	3.188702	3.074367	3.027399	3.038896	3.183245	3.055652	3.037908	3.036778	3.054166	3.021442	3.08996	3.117011	2.717492
WHDC1L1	1560662_s_at	6.360274	6.077639	5.426469	6.055091	5.740596	6.197156	4.913733	5.361708	5.279963	4.90671	5.592841	5.388952	3.61099
SCPEP1	1560665_at	2.643275	2.613067	2.578093	2.594665	2.615548	2.911209	2.606375	2.610253	2.567755	2.593231	2.592885	2.604973	2.538864
KIAA1652	1560671_at	1.909357	1.890351	1.888222	1.875992	1.891312	1.874902	1.886388	1.890635	1.878889	1.875824	1.871736	1.885464	1.845013
LOC283514	1560676_at	3.041789	2.970105	2.914967	2.961207	3.065056	2.940998	2.951659	2.987077	2.972991	2.950601	2.974859	3.015509	4.982286
LOC151438	1560679_at	6.669585	7.100824	6.935206	7.050883	8.172435	7.818337	3.711519	4.065648	4.035332	4.300983	3.424573	2.620863	2.319114
LOC728170	1560680_at	4.245428	4.142838	5.332208	4.92647	4.239772	4.004303	4.084877	4.081552	4.012583	4.405693	3.862027	4.042496	3.767787
BCL8	1560683_at	3.102242	3.031752	2.81124	3.021304	3.065369	3.009882	2.930151	3.024935	2.992877	2.907074	3.013758	3.151388	2.476856

BCL8	1560684_x_at	2.957375	2.856669	2.76312	2.921219	2.947821	2.923905	2.861249	2.850527	2.92127	2.838172	2.92641	2.94956	2.590295
ITGAD	1560686_at	1.904606	1.895134	2.573534	1.88275	1.900317	1.899628	1.892663	1.887182	1.878948	1.87111	1.869928	1.880326	1.867917
AKT2	1560689_s_at	3.946144	4.194387	4.214189	3.990655	4.102548	3.981709	3.998815	4.046379	3.995539	3.983597	4.158157	4.004946	4.272157
LOC285878	1560692_at	2.416092	2.383706	2.347116	2.365178	2.399723	2.357853	2.376243	2.385541	2.371488	2.363683	2.369091	2.379474	2.341871
SFT2D1	1560694_at	1.883583	1.878743	1.857728	1.862726	1.873855	1.859263	1.873118	1.877037	1.864889	1.860956	1.855897	1.866791	1.813054
QTRTD1	1560695_at	3.503505	3.291082	3.214185	3.316251	3.565059	3.377893	3.397742	3.329822	3.371794	3.342583	3.436171	3.499912	3.099242
QTRTD1	1560696_x_at	3.146649	2.887413	2.867771	2.919514	3.089093	2.888401	2.936842	2.957523	2.95404	2.906488	3.016108	3.18896	2.623914
TRHDE	1560697_at	2.674204	2.641967	2.576652	2.614059	2.651874	2.630781	2.627256	2.637299	2.623632	2.603142	2.625726	2.646465	2.55266
TRHDE	1560698_a_at	2.764002	2.739657	2.69205	2.733342	2.755645	2.706814	2.744144	2.750283	2.74164	2.71537	2.79467	2.787037	3.691838
NOS2A	1560703_at	4.95721	5.300733	4.081931	4.653431	4.143184	4.233596	3.840595	3.877075	3.889863	3.86356	4.224008	3.852904	4.217298
LOC283856	1560707_at	1.852647	1.841016	1.819288	1.824994	1.839838	1.8227	1.8351	1.83747	1.825	1.821697	1.821791	1.83166	1.795608
TMPRSS11	1560712_at	2.754656	2.714421	2.643486	2.688255	2.744195	2.696871	2.700354	2.710356	2.698019	2.677128	2.692995	2.720123	2.494706
LRRC62	1560713_a_at	2.358579	2.353919	2.329563	2.331362	2.422507	2.333363	2.328765	2.335532	2.332685	2.313093	2.328314	2.331908	2.351068
FLJ37035	1560714_at	2.741606	2.693834	2.621215	2.668143	2.734437	2.65205	2.680551	2.691342	2.677483	2.666965	2.67437	2.706009	2.529758
P4HA3	1560715_at	2.010567	2.000596	1.961109	1.983126	2.001865	1.989193	1.988771	1.998836	1.988233	1.973055	1.98403	1.992699	1.875578
MKNK1	1560720_at	5.079737	4.782714	4.487688	4.338242	4.443917	4.378316	4.547058	4.361538	4.498148	4.391684	4.346574	4.500209	3.933407
LOC283731	1560723_at	2.417081	2.341033	2.301637	2.322515	2.362791	2.312428	2.332564	2.342298	2.32852	2.312633	2.329369	2.337591	2.26367
HEATR4	1560727_at	2.859082	2.65753	2.623133	2.847769	2.950903	3.915002	2.596947	2.667561	2.628904	2.570486	2.559012	2.644438	2.537304
ALG5	1560733_at	2.32603	2.375213	2.265458	2.287423	2.315896	2.286336	2.289283	2.303908	2.285794	2.282824	2.289797	2.299637	2.180921
OR4N4	1560735_s_at	2.214838	2.189269	2.159721	2.171756	2.196944	2.171197	2.179487	2.186427	2.173839	2.167217	2.168623	2.191428	2.088595
SNRPN	1560741_at	6.66596	6.881964	6.380004	6.695639	7.266221	6.924165	7.992756	8.077703	7.740426	7.748276	8.482537	8.152777	6.86977
C1orf118	1560746_at	3.052758	2.897842	2.81528	2.890342	3.074788	2.934979	2.903972	2.898746	2.907045	2.872351	2.958114	3.012486	2.627016
LOC151121	1560750_at	1.853221	1.83999	1.820932	1.825858	1.841716	1.824438	1.841866	1.843005	1.836993	1.824016	1.831462	1.839419	1.804881
FBXW2	1560752_at	2.706799	2.674033	2.649392	2.653043	2.702475	2.633865	2.66398	2.670733	2.654321	2.630242	2.649353	2.667499	2.468441
CMTM7	1560754_at	5.892606	6.109456	5.336114	5.881122	6.307889	7.117074	5.64927	5.972064	5.46533	5.42156	6.629684	5.908353	5.237095
KIAA0974	1560755_at	3.015741	2.978533	2.941285	2.944703	3.071097	2.940299	3.451736	3.1632	2.970385	3.528207	2.995083	3.213411	2.794616
FLJ39005	1560756_at	2.495337	2.456789	2.441568	2.441277	2.52309	2.424913	2.453949	2.461127	2.447151	2.439014	2.446757	2.49503	2.349759
LOC285972	1560762_at	2.721237	2.632537	2.649094	2.612041	2.908486	2.655049	2.661637	2.667474	2.651434	2.631325	2.659193	2.750599	2.535743
ARHGAP22	1560764_at	2.315501	2.306501	2.28852	2.290045	2.319716	2.287178	2.305496	2.299924	2.298545	2.283765	2.282724	2.300273	2.263941
ARHGAP22	1560765_a_at	2.444459	2.421351	2.371217	2.404845	2.42669	2.403223	2.405238	2.418353	2.414512	2.389609	2.400955	2.417356	2.332574
PABPC1	1560770_at	2.592651	2.548672	2.523302	2.52693	2.620573	2.580091	2.547959	2.56109	2.546586	2.530544	2.547575	2.556332	2.412606
LOC388458	1560773_at	2.513344	2.484251	2.443126	2.460508	2.494154	2.465154	2.468004	2.480347	2.464598	2.457145	2.473645	2.527185	2.322484
LOC653284	1560779_a_at	4.546644	3.760739	3.456151	3.714143	4.150635	3.683329	3.995822	4.243923	3.715122	3.745481	4.517378	3.71327	5.569328
C11orf47	1560781_at	2.95361	2.87583	2.857376	2.862556	2.903178	2.806129	2.862776	2.875213	2.861824	2.830732	2.892328	2.907621	2.732145
DYRK3	1560785_at	2.28873	2.260233	2.23653	2.242625	2.297753	2.316182	2.249782	2.258134	2.246131	2.235343	2.247568	2.255951	2.150199
MYO3B	1560788_at	2.441812	2.408281	2.372713	2.39022	2.428742	2.391438	2.396598	2.408574	2.392302	2.380913	2.393768	2.418665	2.265427
FLJ36144	1560790_at	2.863687	2.830205	2.761867	2.808545	2.847028	2.800558	2.820275	2.831946	2.821318	2.79459	2.818652	2.843203	2.626417
DNAH3	1560803_at	3.1023	3.042893	3.012546	3.030381	3.275961	3.027581	3.028662	3.036794	3.024769	2.950523	3.023896	3.04713	2.679879

LOC150527	1560806_at	2.589426	2.551249	2.529561	2.534197	2.866921	2.491287	2.540308	2.550618	2.538191	2.513948	2.520459	2.647287	2.23887
CCDC32	1560814_a_at	4.697994	4.579623	3.83135	4.12576	4.145161	4.443861	3.60852	3.476339	2.974002	2.504354	3.636663	3.782545	3.94661
LOC387895	1560818_at	2.015226	2.010486	1.987386	1.993239	2.038493	1.994102	2.001143	1.993885	1.994083	1.981191	1.986565	1.9929	1.963028
LOC387895	1560819_a_at	2.951764	2.913048	2.866972	2.896631	2.995606	2.880447	2.911248	2.917105	2.902847	2.88612	2.905024	3.070875	2.586787
ARHGAP22	1560821_at	2.403058	2.357376	2.328407	2.353139	2.386499	2.341783	2.356952	2.3671	2.356168	2.347635	2.352412	2.365739	2.235207
LOC340017	1560823_at	2.797557	2.726785	2.675972	2.71615	2.805117	2.685948	2.732313	2.739294	2.728842	2.703009	2.726571	2.759773	2.514883
LOC147646	1560830_a_at	2.291054	2.244442	2.236242	2.233294	2.273173	2.248197	2.266195	2.276535	2.250511	2.22839	2.252335	2.245982	2.03623
LOC643648	1560833_at	2.954532	2.8677	2.805412	2.873936	2.93856	2.872978	2.875241	2.882907	2.868493	2.8453	2.87289	2.894375	2.606746
RMST	1560834_a_at	3.074218	2.930149	2.909894	2.96924	3.056694	3.150595	2.949171	3.267564	2.998841	3.026794	3.087325	3.099985	2.843089
C6orf146	1560838_at	2.430702	2.401407	2.38064	2.381926	2.444279	2.382395	2.390313	2.396406	2.385386	2.375317	2.380284	2.396191	2.281047
LOC389247	1560841_at	2.236089	2.225736	2.19056	2.207284	2.223102	2.2041	2.212593	2.220311	2.2081	2.202836	2.21011	2.217281	2.160257
C10orf136	1560851_at	2.581689	2.502914	2.458983	2.489896	2.569953	2.482059	2.494199	2.50982	2.49281	2.477448	2.495998	2.508838	2.373962
FLJ44894	1560853_x_at	2.700625	2.608912	2.499379	2.562266	2.725384	2.583996	2.601422	2.616052	2.600104	2.586236	2.61641	2.610834	2.527474
ZNF107	1560854_s_at	2.860747	2.864784	2.317209	2.583393	2.6293	2.961425	2.593315	2.611263	2.611601	2.574001	2.598356	2.766163	2.401836
FGFR2	1560859_at	3.282131	3.308005	3.001714	3.096481	3.272081	3.075362	3.113482	3.320158	3.114117	3.058783	3.120111	3.242882	3.118677
WNK2	1560866_at	2.466963	2.437983	2.418971	2.353333	2.432513	2.713371	2.431394	2.435654	2.42408	2.423394	2.366769	2.429407	2.380256
FLJ33046	1560874_at	2.763558	2.619896	2.753858	2.720455	2.816471	2.712985	2.585434	2.706349	2.72401	2.71267	2.713454	2.990443	2.355338
SYT15	1560878_at	2.431938	2.425504	2.398406	2.411475	2.429636	2.901221	2.370985	2.421344	2.406785	2.40802	2.409756	2.400671	2.186567
SYT15	1560879_a_at	3.012151	2.894413	2.709807	2.873839	2.916266	2.87664	2.889217	2.885844	2.879895	2.972553	2.477811	2.892806	3.169657
C21orf22	1560881_a_at	2.031782	2.019979	1.997324	2.005134	2.016515	1.999843	2.011853	2.019974	2.003991	1.9955	2.005631	2.013389	1.928071
SLC17A1	1560884_at	5.28417	5.033117	5.294346	5.918887	4.995676	4.913572	4.923917	5.075668	5.236342	4.997471	5.356189	5.073779	3.178451
SLC17A1	1560885_x_at	4.563205	4.312507	4.526072	4.769515	4.437888	4.194291	4.286139	4.257708	4.360009	4.243888	4.258611	4.742751	3.451999
LOC645188	1560895_at	2.562269	2.517258	2.480113	2.504163	2.550212	2.50331	2.514756	2.523368	2.51123	2.493669	2.509573	2.526556	2.359336
KRTAP10-1	1560897_a_at	3.333566	3.223815	3.220838	3.274511	3.233738	3.241083	3.911705	4.391401	3.297366	3.291257	3.435751	3.342717	3.087833
LOC728789	1560901_at	1.948378	1.941831	1.924837	1.927093	1.933402	1.924872	1.939235	1.932378	1.935742	1.928672	1.919239	1.926068	1.923428
HUMYZ82H	1560906_at	3.713413	4.047968	4.778226	4.215521	4.288003	4.187802	3.174853	2.986919	2.994068	2.928783	2.987057	3.06698	2.710605
PPIL6	1560910_at	2.662413	2.612448	2.576798	2.637879	2.651957	2.644307	2.651149	2.653237	2.641423	2.618809	2.642378	2.797993	2.544099
LOC389043	1560912_at	2.266555	2.238757	2.216276	2.221759	2.250191	2.224321	2.2291	2.236027	2.223813	2.216539	2.222681	2.233368	2.142155
DPY19L1	1560915_at	2.775806	2.74046	2.662448	2.713062	2.764321	2.691493	2.727011	2.738392	2.722327	2.702144	2.722575	2.755679	2.512298
DPY19L1	1560916_a_at	5.058526	5.005323	4.777999	4.739542	4.783543	4.963725	5.178037	5.324812	5.553189	5.873538	5.001422	5.488294	5.984061
ZNF169	1560921_at	2.455042	2.459218	2.393269	2.425833	2.462489	2.406329	2.435458	2.642747	2.427078	2.412802	2.513233	2.44239	2.389226
ZNF169	1560922_s_at	2.611995	2.361232	2.35076	2.344932	2.796224	3.094414	2.339541	2.355795	2.352247	2.318051	2.35209	2.350356	2.462167
LOC151657	1560928_at	1.756105	1.744853	1.725914	1.730606	1.744274	1.72886	1.742882	1.739631	1.735007	1.728505	1.7259	1.740659	1.698487
FLJ31356	1560932_at	3.119757	3.028126	2.926906	2.994615	3.090938	2.992922	3.037302	3.047403	3.033454	3.010932	3.034621	3.341038	2.755475
LOC284669	1560934_at	3.464036	3.490634	3.174704	3.289745	3.558389	3.260502	3.260292	3.262168	3.323182	3.257983	3.344866	3.412762	2.874455
LOC284669	1560935_s_at	3.225356	3.678107	2.87521	2.797026	4.395874	4.035056	2.720735	2.767126	2.710399	2.632542	2.632586	2.78625	2.472224
RSU1	1560937_at	3.298255	3.130152	3.058157	3.139825	3.296135	3.164628	3.146059	3.116171	3.129469	3.080472	3.252547	3.205051	2.798188
FLJ40434	1560944_at	2.936461	2.805231	2.741915	2.798211	2.936898	2.827207	2.805979	2.827809	2.823269	2.791542	2.837048	2.878155	2.76294

NOS1	1560974_s_at	3.044703	2.973051	2.857595	2.972795	2.991597	2.959306	2.963627	2.975173	2.961686	2.947217	2.958474	2.969905	2.653179
ZD77D08	1560977_a_at	2.514262	2.498039	2.438175	2.480104	2.497469	2.482683	2.479595	2.490474	2.480218	2.481525	2.477402	2.486124	2.432086
PPARA	1560981_a_at	3.983919	3.649843	4.055699	4.207556	4.424969	4.26576	4.136598	4.540297	4.648933	4.022736	3.646695	4.689738	2.958974
LOC400590	1560992_at	3.318017	3.195352	3.111753	3.213046	3.32505	3.132094	3.195472	3.202208	3.210792	3.16567	3.245757	3.264287	2.89811
LOC400590	1560994_x_at	3.129269	2.986926	2.959871	2.983902	3.114869	2.961027	2.992448	3.001111	3.004768	2.962664	3.013051	3.050533	2.712345
MAOB	1561009_at	3.002789	2.887394	2.716817	2.878106	3.169298	2.84665	2.894317	2.908327	2.890818	2.837388	3.001774	2.953893	2.628776
MAOB	1561010_a_at	2.634682	2.596696	2.54382	2.58172	2.617067	2.571624	2.686566	2.599468	2.583736	2.56859	2.586134	2.599154	2.447077
LOC646241	1561027_at	2.507092	2.477417	2.436739	2.45566	2.490871	2.438397	2.4659	2.475945	2.462355	2.450412	2.461132	2.479228	2.363066
ZNF81	1561038_at	3.013936	2.963276	2.90154	2.95322	2.971805	3.348234	2.95512	2.95007	2.959587	2.941745	2.997969	3.000354	2.750598
ZNF81	1561039_a_at	2.279616	2.248485	2.219444	2.232101	2.267306	2.230052	2.240719	2.249777	2.23713	2.224075	2.23524	2.246048	2.192871
ITGB1	1561042_at	2.440247	2.400094	2.347343	2.36559	2.418776	2.390208	2.486988	3.246358	2.574348	2.684776	2.955851	2.550083	2.330567
RARS2	1561048_at	2.217738	2.198371	2.174835	2.17686	2.202332	2.172485	2.200706	2.194703	2.182021	2.178588	2.181478	2.190022	3.410345
CCDC14	1561054_a_at	2.315477	2.287114	2.259823	2.270716	2.291452	2.276658	2.275238	2.28688	2.271566	2.262419	2.270553	2.281021	2.197363
LOC152024	1561059_a_at	3.417994	3.227917	3.283704	3.253951	3.439616	3.168882	3.255591	3.305546	3.282819	3.292758	3.331533	3.376872	2.920849
ANKRD28	1561079_at	5.250431	5.742004	5.420283	5.815025	5.70303	4.784602	4.620136	3.915162	4.835311	4.116561	4.401456	3.354616	4.529756
NID1	1561082_at	2.662171	2.541479	2.522116	2.516175	2.739965	2.6645	2.527501	2.538081	2.522892	2.514832	2.525001	2.533028	2.357658
LOC153910	1561085_at	2.257642	2.241352	2.219514	2.222417	2.24129	2.22853	2.239761	2.238707	2.231436	2.218286	2.227909	2.233694	2.168058
EYA4	1561088_at	2.320693	2.281326	2.263075	2.264697	2.294592	2.268021	2.277571	2.284801	2.267868	2.258011	2.26895	2.285088	2.163554
LOC400654	1561090_at	2.793898	2.751052	2.698124	2.734247	2.822295	2.683336	2.734167	2.748234	2.734321	2.727659	2.740321	2.761297	2.512808
LOC387601	1561093_at	1.422361	1.410781	1.393376	1.401975	1.400595	1.398433	1.256034	1.399387	1.832832	2.219935	1.624615	1.391962	1.258775
LOC285419	1561096_at	2.418926	2.387487	2.3624	2.370603	2.402562	2.355594	2.379629	2.388523	2.375231	2.376997	2.374206	2.379062	2.312109
LOC641365	1561098_at	2.55506	2.506848	2.478495	2.501408	2.573005	2.475344	2.517163	2.522119	2.512454	2.503847	2.513994	2.545472	2.423695
FLJ32756	1561099_at	3.074817	3.042698	3.108105	2.996632	3.110379	3.02997	2.991787	3.002933	3.018056	3.00759	3.029967	3.061876	2.743657
LOC153469	1561101_at	2.263235	2.239089	2.223912	2.313512	2.247542	2.215946	2.225697	2.23742	2.223145	2.21249	2.222902	2.230923	2.129075
C6orf217	1561106_at	3.019996	2.910825	2.849516	2.923053	3.009918	2.915515	2.921902	2.929596	2.934993	2.892861	2.933531	2.970591	2.73939
DEPDC4	1561114_a_at	3.166748	3.112056	3.061043	3.12666	3.150282	2.995194	3.114328	3.123757	3.111223	3.090477	3.126509	3.192859	2.938723
MTHFD1L	1561125_at	2.332877	2.308458	2.275731	2.293631	2.35278	2.285321	2.290857	2.308397	2.292277	2.285933	2.279932	2.31843	2.201145
ZNF285B	1561126_at	2.017076	2.001929	1.984115	1.985799	2.003806	1.987298	1.999334	2.000989	1.990819	1.983792	1.987756	1.99627	1.940721
hCG_17739	1561127_at	1.521202	1.513918	1.495662	1.499234	1.507693	1.502431	1.511907	1.51075	1.507479	1.501238	1.584083	1.504083	1.498408
C12orf51	1561130_at	2.419175	2.421868	2.343038	2.367106	2.42504	2.358096	4.984836	5.224089	5.089094	5.269002	4.822467	4.821361	2.247942
DHX35	1561132_at	2.689248	2.676148	2.555227	2.60517	2.658086	2.609414	2.610276	2.624355	2.610359	2.586658	2.606134	2.619266	2.282736
GYPE	1561136_at	2.783717	2.700324	2.642308	2.696614	2.772199	2.662442	3.722877	2.727782	3.348369	2.683603	2.928834	2.779115	2.497276
GYPE	1561137_s_at	2.796063	2.706296	2.651131	2.730601	2.929296	2.689332	4.429053	5.810671	4.544815	2.758947	3.808999	5.221995	2.511107
VPS35	1561146_at	2.576504	2.543837	2.513536	2.517819	2.567661	2.498297	2.542102	2.545378	2.537774	2.521581	2.530919	2.541043	2.502515
DEFB108B	1561165_a_at	2.654524	2.597496	2.548403	2.57682	2.643343	2.520874	2.601991	2.608507	2.60053	2.589045	2.601711	2.664829	2.428078
LOC727818	1561169_at	2.252655	2.218957	2.186723	2.195766	2.237226	2.194427	2.207539	2.218385	2.202829	2.194463	2.205877	2.213744	2.112296
FLJ36131 //	1561170_at	3.142415	2.94775	2.908081	2.964873	3.169488	2.896394	2.981482	2.971631	2.982986	2.933888	3.025748	3.058641	2.669318
FLJ36131 //	1561171_a_at	2.348664	2.330228	2.293125	2.308357	2.327651	2.315237	2.329513	2.322736	2.315542	2.433849	2.313618	2.380533	2.241075

LOC283482	1561175_at	2.55886	2.482548	2.46458	2.467023	2.526931	2.469562	2.476371	2.487805	2.473202	2.443715	2.465787	2.485178	2.417235
AMZ1	1561179_s_at	2.309793	2.298579	2.504815	2.28839	2.28756	2.377191	2.228299	2.279451	2.282831	2.276198	2.284871	2.471472	2.187092
CLONE7957	1561185_at	2.547166	2.497106	2.483694	2.49571	2.518916	2.502036	2.516535	2.506617	2.497563	2.479731	2.500311	2.50256	2.34787
CDKL3	1561190_at	4.224851	4.258672	4.108448	4.041747	4.188734	5.139708	4.122665	4.611489	4.114042	4.133152	4.145864	4.674246	4.234318
LOC442028	1561197_at	2.225686	2.204624	2.184339	2.183417	2.217217	2.187121	2.193339	2.202752	2.189269	2.179256	2.188167	2.195239	2.098738
LOC387790	1561198_at	2.893132	2.821498	2.767394	2.810195	2.907927	2.786908	2.826087	2.831127	2.818702	2.802074	2.84581	2.856402	2.631125
VWA3B	1561200_at	2.64547	2.590635	2.539878	2.580132	2.652754	2.557758	2.592344	2.602391	2.589914	2.568619	2.589395	2.623694	2.46436
KLHL8	1561206_at	4.331718	4.446603	5.061629	5.526171	5.01181	4.259536	4.092794	3.822066	4.207213	4.142802	3.918114	3.898491	3.696768
LOC728099	1561218_s_at	3.106766	3.01759	2.943774	2.908698	3.13261	2.98501	3.045805	3.056646	3.042497	3.030887	3.037037	3.184418	2.780379
LOC728099	1561221_x_at	2.252504	2.236097	2.22072	2.223564	2.235185	2.224554	2.229988	2.236253	2.226293	2.207265	2.221887	2.244729	2.205817
LOC283432	1561222_at	3.552968	3.377868	3.329446	3.361037	3.595699	3.381873	3.385939	3.384647	3.397107	3.356734	3.417293	3.45486	3.051476
LOC338579	1561225_at	2.028537	2.02035	1.996876	2.003332	2.014706	2.002132	2.015804	2.016369	2.006319	1.999012	2.005573	2.012864	1.941323
LOC283387	1561233_at	2.377541	2.365134	2.34435	2.342262	2.364464	2.348023	2.359198	2.361554	2.349946	2.34541	2.3461	2.355998	2.299826
PXMP3	1561238_at	2.384846	2.357364	2.336571	2.338421	2.360322	2.323828	2.352825	2.343996	2.353319	2.335559	2.338499	2.350115	2.784709
TMEM105	1561243_at	2.664012	2.662827	2.640816	2.643846	2.650781	2.645536	2.661954	2.908146	2.65718	2.651067	2.645911	2.653515	2.634094
DNM1DN8@	1561249_a_at	2.874351	2.856211	2.831152	2.842555	2.857109	2.849363	2.847529	2.855045	2.836783	2.840879	2.837858	2.924113	2.675558
LOC285577	1561251_at	2.418027	2.395079	2.355704	2.377656	2.404071	2.36641	2.384403	2.396272	2.380398	2.369516	2.38124	2.390351	2.254994
LOC648691	1561252_at	2.129428	2.119866	2.079167	2.104842	2.125559	2.111472	2.115077	2.119987	2.105999	2.108297	2.106268	2.114881	2.047986
LOC340340	1561254_at	2.27919	2.240762	2.220675	2.222982	2.276875	2.226312	2.23197	2.242968	2.231391	2.219921	2.225592	2.243559	2.131617
LOC286083	1561257_at	2.372257	2.340931	2.325006	2.330418	2.359388	2.328693	2.334946	2.342774	2.332013	2.313054	2.328068	2.347672	2.271627
C1QTNF3	1561263_at	2.020783	2.007847	1.991437	1.992414	2.006233	1.997659	2.006334	2.002219	2.000739	1.995073	1.989955	1.997238	1.927807
RAPGEF5	1561269_at	2.006648	1.987234	1.965632	1.970366	1.996732	1.969427	1.981176	1.98452	1.972541	1.964679	1.968226	1.979947	1.913539
CCDC144A	1561271_at	2.842809	2.779445	2.77792	2.75468	2.881613	2.746491	2.782048	2.791302	2.782959	2.758126	2.778495	2.804825	2.681438
DOCK5	1561276_at	3.063281	2.925634	4.130005	4.677711	3.300015	2.868202	2.711099	2.750693	2.761514	2.717956	2.802354	2.72344	2.553189
LOC339298	1561277_at	2.139439	2.124887	2.098591	2.109215	2.122479	2.10954	2.11581	2.121162	2.104484	2.101184	2.106024	2.114007	2.021242
LOC284576	1561280_at	2.848791	2.80167	2.780415	2.782565	2.852991	2.769564	2.794307	2.796096	2.789454	2.774477	2.7876	2.823335	2.553548
LOC284576	1561281_a_at	3.142848	3.075661	2.997165	3.058185	3.167676	3.056781	3.05612	3.041485	3.050904	3.019862	3.07098	3.083918	2.807342
DIP2A	1561286_a_at	3.214565	3.09347	3.090125	2.899424	3.155321	3.023744	3.133467	3.242329	3.118885	3.672839	3.874505	4.032724	2.494891
LOC286370	1561289_at	2.623561	2.596042	2.557488	2.580264	2.611577	2.579647	2.586246	2.5936	2.581715	2.573023	2.581912	2.592595	2.403034
LOC339622	1561290_at	3.036442	2.852499	2.803684	2.87738	3.094152	2.835636	2.8634	2.861642	2.88595	2.837176	2.946694	2.942868	2.48366
LOC144678	1561294_a_at	2.62181	2.568562	2.529449	2.543231	2.629065	2.530864	2.556842	2.56819	2.55419	2.530461	2.552235	2.575275	2.358465
WARS2	1561297_at	2.13344	2.108917	2.086598	2.09614	2.158591	2.094614	2.105521	2.105288	2.097344	2.09213	2.095399	2.107059	2.022233
LOC151877	1561301_at	2.150181	2.113586	2.092403	2.095942	2.110218	2.101048	2.112127	2.1122	2.10603	2.099372	2.09671	2.104916	2.024484
RP11-506K6	1561304_a_at	2.410688	2.190221	2.162191	2.170763	2.180182	2.785196	2.186862	2.188462	2.173477	2.164802	2.17441	2.182091	2.086584
STRC	1561306_s_at	2.002052	2.066168	1.970912	1.976752	2.041787	2.034326	1.9878	1.988979	1.980109	1.980963	1.976044	1.988277	1.944752
GABRB3	1561316_at	2.578454	2.531782	2.491551	2.519537	2.579782	2.504681	2.52989	2.539337	2.528194	2.506513	2.521366	2.540464	2.36217
LOC339975	1561323_at	2.70586	2.680395	2.671269	2.67538	2.689395	2.651536	2.685112	2.686969	2.675619	2.65474	2.667766	2.704299	2.572119
C6orf122	1561327_at	2.151575	2.119593	2.103455	2.10954	2.155655	2.108344	2.118685	2.126536	2.114924	2.107709	2.114293	2.121164	2.037698

RPUSD3	1561329_s_at	2.500329	2.478876	2.441452	2.460613	2.486079	2.465959	2.467256	2.479879	2.475046	2.464131	2.460743	2.486154	2.590652
DSG4	1561330_at	2.306159	2.288191	2.270743	2.266075	2.289303	2.310633	2.283581	2.284487	2.272273	2.257746	2.269459	2.280437	2.190793
C1orf99	1561331_at	3.042764	2.925608	2.872553	2.91423	3.034408	2.897468	2.929508	2.917278	2.92504	2.904075	3.001361	3.003847	2.634666
ATP13A5	1561332_at	2.046536	2.036075	2.024162	2.021975	2.03541	2.020553	2.027523	2.034116	2.017726	2.019015	2.017862	2.026778	1.953467
LOC285181	1561334_at	3.255936	3.775386	3.759669	4.109329	5.183131	5.018248	2.951712	2.968309	2.898552	2.926456	2.975193	2.86785	2.445851
LOC440602	1561335_at	2.674038	2.658758	2.628625	2.639828	2.658548	2.635062	2.649618	2.655761	2.640679	2.632133	2.638103	2.650529	2.561842
DNASE1L3	1561336_at	6.101031	5.55777	5.292637	5.103761	3.927201	4.077322	2.468048	2.46564	2.485922	2.455788	2.548832	2.462497	2.408059
LOC150005	1561342_at	2.50223	2.461666	2.414205	2.441954	2.491051	2.429772	2.453518	2.465436	2.450442	2.434229	2.446925	2.461562	2.316382
LOC150005	1561343_a_at	3.365635	3.181798	3.104081	3.203306	3.405381	3.133005	3.207093	3.220277	3.232131	3.180007	3.279048	3.33037	2.827895
LOC339874	1561348_at	2.376928	2.349687	2.31152	2.333316	2.359742	2.331762	2.353638	2.350167	2.338219	2.335797	2.339777	2.346127	2.235279
LOC692247	1561355_at	2.834878	3.057649	2.820378	3.249429	2.935335	3.216326	2.644688	2.932379	2.411778	2.915579	2.999602	2.819035	2.234648
TXLNA	1561358_at	2.734003	2.682938	2.649624	2.665502	2.698746	2.672762	2.676254	2.684287	2.669533	2.657156	2.669562	2.678499	2.551774
ZNF660	1561361_at	2.314537	2.290301	2.252235	2.269447	2.29773	2.263167	2.286007	2.472956	2.480353	2.266048	2.684135	2.390505	2.284282
NRP1	1561365_at	2.651158	2.612021	2.545347	2.593969	2.634922	2.56687	2.602977	2.609208	2.596086	2.588478	2.591777	2.608343	3.385783
FLJ39080	1561371_at	2.797772	2.70938	2.705299	2.70199	2.77074	2.720886	2.720881	2.734525	2.716204	2.710211	2.717237	2.774542	2.505084
LOC285796	1561373_at	2.014253	2.000583	1.982825	1.98499	2.000696	1.988836	1.990802	2.000138	1.992282	1.986228	1.98248	2.012981	1.953251
C12orf42	1561378_at	2.68906	2.671169	2.651697	2.653233	2.670919	2.653916	2.650792	2.667902	2.65793	2.651427	2.650308	2.663219	2.581871
LOC284661	1561383_at	2.658053	2.596027	2.550977	2.58138	2.634923	2.564527	2.592041	2.600768	2.588156	2.570424	2.588854	2.607921	2.258929
LOC284661	1561384_a_at	2.761271	2.738926	2.688892	2.719405	2.748631	2.712847	2.727081	2.7337	2.719706	2.710903	2.733249	2.754237	2.550936
FAM55A	1561387_a_at	3.263718	3.308492	3.143459	3.210161	3.367822	3.232872	3.217313	3.224672	3.221991	3.190889	3.226626	3.292859	2.965106
FAM41AY	1561390_at	1.964281	1.955372	1.935764	1.93947	1.953407	1.937767	1.9503	1.951225	1.94277	1.938305	1.934341	1.949501	1.908177
RP5-1054A2	1561394_s_at	2.278304	2.171169	2.140926	2.139056	2.456497	2.16288	2.149799	2.15642	2.153546	2.139123	2.165888	2.168154	3.337481
EPHA6	1561396_at	2.759064	2.717446	2.680557	2.704988	2.745828	2.684782	2.711102	2.72474	2.711132	2.694408	2.707664	2.729904	2.659417
LOC285627	1561401_at	2.236468	2.217608	2.194891	2.199188	2.219377	2.196722	2.209818	2.215318	2.201715	2.194538	2.200226	2.210668	2.107583
LOC339894	1561402_at	2.726906	2.703778	2.679252	2.687702	2.729227	2.686819	2.692976	2.70531	2.692244	2.676082	2.691178	2.713582	2.614112
SOHLH1	1561403_at	1.623484	1.617581	1.591541	1.595763	1.60893	1.601874	1.602633	1.614977	1.59898	1.600438	1.599834	1.607621	1.580123
CATSPER2	1561405_s_at	2.861446	3.150716	2.724582	2.773408	3.266881	3.775087	2.646996	2.82606	2.783223	2.801626	2.796936	2.833431	2.496331
CENTD1	1561407_at	2.702871	2.641312	2.587207	2.631419	2.66066	2.610453	2.65547	2.654237	2.640621	2.628279	2.639425	2.66754	2.513718
LOC401497	1561414_at	3.346563	3.236414	3.099534	3.222994	3.365549	3.195814	3.226262	3.246944	3.233527	3.211907	3.266303	3.305842	2.789982
LOC642924	1561423_at	2.490446	2.464339	2.419213	2.451624	2.47343	2.657618	2.454898	2.467069	2.451591	2.438502	2.485024	2.463354	2.319227
ZNF568	1561425_a_at	2.146812	2.124006	2.096263	2.105786	2.125967	2.102696	2.112743	2.121882	2.108891	2.101454	2.10741	2.116256	2.194344
C3orf15	1561429_a_at	3.381101	3.174254	3.072593	3.152001	3.57917	3.144418	3.160155	3.179416	3.183899	3.129787	3.207186	3.263566	2.826145
C3orf15	1561430_s_at	2.955123	2.910122	2.909621	2.878051	3.002193	2.880616	2.895896	2.913075	2.894237	2.884362	2.920569	2.907417	2.622978
LOC285103	1561433_at	2.330565	2.189944	2.155385	2.158602	2.19191	2.314322	2.173466	2.184421	2.16708	2.162435	2.188747	2.172769	2.142233
C15orf45	1561434_at	2.76526	2.712143	2.675462	2.69411	2.754638	2.680148	2.70211	2.714488	2.702674	2.675402	2.699825	2.729028	2.626896
LOC728012	1561437_at	2.879902	2.796713	2.729654	2.788238	2.867592	2.763308	2.798915	2.808383	2.795773	2.777319	2.816168	2.834631	2.571677
LOC283585	1561442_at	2.316651	2.296211	2.268883	2.277394	2.305866	2.283241	2.293757	2.293927	2.278349	2.26719	2.27963	2.287886	2.203708
TSPAN18	1561449_at	2.309242	2.280197	2.243498	2.258923	2.290444	2.253127	2.26877	2.280885	2.297568	2.254805	2.265691	2.276125	2.164842

LOC284294	1561455_at	2.232363	2.205852	2.184055	2.190233	2.215998	2.187838	2.200387	2.202397	2.19143	2.183564	2.184572	2.196372	2.100242
LOC441009	1561471_at	2.472866	2.438384	2.407449	2.41967	2.457685	2.419589	2.430925	2.439677	2.424596	2.413456	2.4245	2.435352	2.290708
LOC728805	1561472_at	2.431209	2.395202	2.361227	2.371881	2.39928	2.358238	2.382901	2.395727	2.379648	2.371329	2.379737	2.387845	2.294472
CCDC144B	1561477_at	3.109755	3.006258	2.989269	3.008315	3.113464	3.01462	3.045772	3.055861	3.031166	2.953642	3.067955	3.129051	2.709699
IQCA	1561485_at	3.134182	3.075933	3.057416	3.063303	3.12333	3.074527	3.044781	2.860751	3.036096	3.062526	3.070764	3.084161	2.966239
AAA1	1561490_at	2.031373	2.015029	1.990025	1.994245	2.019639	1.997855	2.007757	2.009232	1.997999	1.989478	1.992694	2.010447	1.948399
LOC283214	1561491_at	2.602993	2.577996	2.545101	2.558226	2.609153	2.53644	2.570136	2.579838	2.567558	2.553398	2.563992	2.75566	2.452736
LOC647107	1561492_at	2.813702	2.802644	2.784916	2.790254	2.839001	2.777591	2.800234	2.789415	2.791689	2.783651	2.780512	2.794469	2.729092
LOC348180	1561500_at	2.240879	2.23272	2.215451	2.199687	2.23182	2.219372	2.18671	2.232005	2.223278	2.218782	2.220768	2.471812	1.951104
LOC348180	1561501_s_at	3.260018	3.169372	3.104234	3.176258	3.348722	3.160871	3.185695	3.183908	3.181022	3.145936	3.209917	3.267648	2.941915
LOC348180	1561502_x_at	2.467938	2.342631	2.148675	2.31684	2.767855	2.322868	2.329476	2.336611	2.302313	2.318334	2.324264	2.33522	2.207265
RP11-145H	1561503_at	2.397467	2.364737	2.349062	2.352896	2.400874	2.35409	2.353469	2.372214	2.35562	2.350613	2.357093	2.382313	2.229019
RP11-145H	1561504_s_at	3.141207	3.031327	3.049641	3.053979	3.21153	3.054418	3.032617	3.046508	3.03665	3.004027	3.091883	3.149027	2.593396
FFAR1	1561507_at	1.987497	1.968185	1.944723	1.953288	1.970349	1.952219	1.961529	1.965272	1.952389	1.945206	1.951384	1.960236	1.895709
MRPL38	1561508_at	2.23875	2.210185	2.188617	2.193115	2.225143	2.187701	2.202273	2.208129	2.196003	2.194534	2.190844	2.204346	2.105649
LOC400655	1561514_at	2.611653	2.569065	2.508291	2.546234	2.757943	2.524894	2.560937	2.55921	2.554565	2.543493	2.553734	2.572044	2.42546
SSPN	1561517_at	2.60206	2.566018	2.503311	2.549207	2.597134	2.548425	2.558466	2.562974	2.549824	2.530303	2.553059	2.576435	2.478578
LOC283914	1561518_at	2.242427	2.096091	2.070052	2.075562	2.098423	2.140809	2.093843	2.089863	2.089075	2.071792	2.063147	2.082279	1.957183
S100B	1561521_at	3.005108	2.900201	2.827148	2.891794	2.997672	2.846577	2.905861	2.912518	2.91215	2.874487	2.925578	2.967436	2.672623
CCDC65	1561523_at	3.216228	3.102008	3.069294	3.092484	3.173655	3.088643	3.10282	3.115848	3.123864	3.080423	3.121306	3.157749	2.692713
LOC283435	1561536_at	2.129063	2.105388	2.066215	2.085896	2.114413	2.088004	2.098191	2.101267	2.090065	2.084168	2.088078	2.098881	2.015879
LOC388906	1561537_at	1.861978	1.852541	1.833366	1.836692	1.849258	1.833534	1.852558	1.849571	1.845624	1.831004	1.834637	1.842583	1.827974
LOC144920	1561540_at	2.428655	2.394363	2.36909	2.378277	2.414251	2.374681	2.389919	2.399392	2.381052	2.371169	2.383757	2.403482	2.213517
LIPA	1561557_at	2.286711	2.263613	2.228404	2.244554	2.268915	2.237736	2.261133	2.261738	2.254576	2.241064	2.245817	2.263672	2.153174
LOC285501	1561566_at	2.418674	2.381726	2.357155	2.361168	2.397802	2.353568	2.369465	2.381335	2.367114	2.360627	2.364375	2.378366	2.243227
LOC730139	1561571_at	2.769992	2.545258	2.625339	2.699917	2.749183	3.693711	2.866388	2.745775	2.734222	2.728309	2.987188	2.914872	2.117921
MCART6	1561578_s_at	3.120682	2.994998	2.982826	2.999186	3.074921	2.992112	3.007142	2.997236	3.004313	2.957387	3.033587	3.023029	2.902716
LOC728445	1561579_at	1.915925	1.906882	1.890045	1.89083	1.923439	1.890535	1.899423	1.902495	1.891685	1.887494	1.885648	1.895728	1.841558
RCBTB2	1561585_at	2.370817	2.355712	2.334031	2.337841	2.372907	2.337799	2.350551	2.35212	2.34015	2.327216	2.336718	2.347845	2.28522
LOC284260	1561587_at	2.403502	2.383174	2.353317	2.367513	2.415773	2.374319	2.391098	2.390349	2.375988	2.357165	2.373509	2.392356	2.246384
NBEAL1	1561589_a_at	2.811049	3.016047	2.752958	2.756683	2.771054	2.755293	2.766576	2.768403	2.7179	2.752789	2.751461	2.762803	2.49999
BC038740	1561590_a_at	3.308913	3.261886	3.190464	3.233374	3.452617	3.104009	3.228084	3.258048	3.224252	3.181086	3.244965	3.254698	3.054211
SLC8A1	1561614_at	3.022916	2.95268	2.840974	2.904499	3.013087	3.325664	2.908072	2.962019	2.948775	2.887142	2.983149	2.977875	2.640224
SLC8A1	1561615_s_at	3.200324	2.720022	2.897578	3.895949	4.904336	5.109157	2.435869	2.577614	2.563036	2.427288	2.721661	2.520997	4.129411
DNAH6	1561616_a_at	2.500571	2.465238	2.446845	2.441679	2.464783	2.432342	2.447074	2.463063	2.437688	2.437733	2.429161	2.488095	2.410732
DNAH6	1561617_at	2.811316	2.77781	2.756204	2.76029	2.846279	2.759632	2.768451	2.76662	2.764822	2.759306	2.768751	2.773195	2.639539
DJ1033E15.	1561626_at	2.076542	2.069802	2.045279	2.054395	2.072541	2.051887	2.064163	2.065755	2.05459	2.049337	2.053561	2.059558	2.054371
HMGA2	1561633_at	3.15562	3.100138	2.961646	3.076649	3.156345	3.078516	3.094675	3.099938	3.063826	3.063769	3.108389	3.146499	2.723234

LOC285692	1561650_s_at	2.277963	2.267422	2.234483	2.249239	2.265906	2.242139	2.263399	2.270625	2.253764	2.250929	2.254814	2.290656	2.207857
TAL1	1561651_s_at	2.416235	2.365938	2.481821	2.570171	2.499062	2.573952	3.589079	5.006302	4.189194	4.762221	3.659826	4.934375	2.232197
BECN1	1561652_at	3.428129	3.333685	3.295016	3.366281	3.412943	3.389538	3.418301	3.414668	3.414208	3.386051	3.62765	4.181213	3.087418
MCART6	1561660_at	2.316653	2.290336	2.252709	2.268082	2.298493	2.262625	2.279201	2.289605	2.274764	2.266001	2.276373	2.284489	2.181447
KIAA1908	1561666_a_at	2.544723	2.594137	2.187751	2.190141	2.21273	2.187681	2.244554	3.057331	2.119048	2.185842	2.175443	2.202741	2.098415
LOC286121	1561671_at	2.168548	2.143985	2.119646	2.127895	2.150724	2.131155	2.136343	2.14411	2.131051	2.123947	2.131084	2.138154	2.051142
MACROD2	1561677_at	2.325732	2.304821	2.271041	2.285614	2.38101	2.282778	2.295048	2.301836	2.302707	2.275242	2.287919	2.297698	2.180329
LOC730015	1561685_a_at	2.524398	2.509212	2.490839	2.493989	2.508939	2.495355	2.495791	2.507456	2.491538	2.493881	2.492856	2.504189	2.409577
ZNF382	1561687_a_at	2.622712	2.586339	2.566103	2.541017	2.597532	2.553403	2.593948	2.599624	2.665986	2.567925	2.58553	2.617641	2.651846
LOC285735	1561691_at	2.855538	2.790652	2.718541	2.781577	2.853589	2.74053	2.784974	2.804422	2.787479	2.754332	2.788587	2.82752	2.605518
LOC400794	1561693_at	2.699723	2.875824	2.663648	2.6675	2.684673	2.666859	2.679681	2.681894	2.6691	2.66232	2.663493	2.675781	2.475515
C6orf150	1561708_at	2.587718	2.558997	2.530281	2.540806	2.570216	2.534199	2.548328	2.558395	2.544156	2.523	2.546334	2.564747	2.382151
SNX19	1561710_at	2.218229	2.190568	2.160891	2.17119	2.19493	2.168831	2.180724	2.187788	2.174086	2.174037	2.174295	2.183693	2.102677
B3GALNT2	1561714_a_at	2.250327	2.215191	2.190814	2.196515	2.295323	2.19054	2.204176	2.212449	2.20038	2.190669	2.201147	2.210216	2.118141
RECQL5	1561720_at	5.08936	4.322183	4.03345	4.377287	5.493572	5.221818	4.170395	5.195486	4.194196	4.227997	5.226274	5.275658	3.299345
C12orf40	1561721_a_at	2.97566	2.911774	2.862746	2.906935	2.965673	3.363454	2.912845	2.929775	2.915984	2.890605	3.09314	3.049395	2.628578
LOC339894	1561723_at	2.72113	2.677536	2.929337	2.99418	2.711526	2.895818	2.664146	2.674064	2.660719	2.63838	2.658103	2.667104	2.525794
LOC400238	1561728_a_at	3.066787	3.02765	2.94747	3.053903	3.053926	3.006318	3.01082	3.022895	3.010882	2.979514	3.036048	3.068808	2.660906
LOC283352	1561757_a_at	2.242405	2.219636	2.191557	2.200119	2.224775	2.197943	2.208616	2.217212	2.203369	2.193087	2.20118	2.212249	2.119618
LOC645513	1561759_at	2.895409	3.126161	2.484593	2.511807	2.81967	2.492393	2.522877	2.53372	2.519989	2.557776	2.516801	2.533737	2.367445
LOC645513	1561760_s_at	4.574658	4.227137	4.368352	4.405363	4.578956	4.268436	4.323439	4.096169	4.415529	4.391518	4.411484	4.433302	4.188156
LOC645513	1561761_x_at	2.547842	2.542067	2.500031	2.5209	2.528803	2.505988	2.503053	2.520607	2.512852	2.482093	2.509944	2.512818	2.363531
MUC4	1561785_at	2.462084	2.339798	2.352618	2.38356	2.45898	2.367742	2.385732	2.397608	2.386352	2.361789	2.428693	2.422971	2.223374
SCN8A	1561820_at	3.290537	3.186058	3.265557	3.180803	3.276152	3.163701	3.188805	3.200696	3.185639	3.17104	3.184329	3.234234	2.695898
FLJ40176	1561846_s_at	2.444106	2.423673	2.401611	2.405176	2.428471	2.402622	2.411461	2.420296	2.406986	2.398314	2.406566	2.425663	2.268238
NUDT17	1561847_at	2.523078	2.492184	2.464606	2.461465	2.49133	2.469309	2.477225	2.491081	2.482613	2.478215	2.47334	2.484056	2.352619
PKD1L2	1561849_at	2.553042	2.509071	2.461933	2.498303	2.552362	2.481862	2.507604	2.516995	2.503807	2.487287	2.501192	2.51518	2.352489
MGC15613	1561850_at	2.429061	2.40305	2.404281	2.384525	2.415976	2.381551	2.395469	2.399733	2.387087	2.379906	2.383573	2.394659	2.331542
IL23R	1561853_a_at	2.493558	2.461695	2.405879	2.43671	2.474707	2.42637	2.446084	2.456945	2.443353	2.426274	2.440776	2.457894	2.304143
SIGLECP16	1561880_a_at	2.337891	2.335652	2.233095	2.271456	2.874413	3.118921	2.274763	2.274572	2.284331	2.249003	2.268267	2.573654	2.166865
SYTL3	1561882_at	3.320415	3.206991	3.219776	3.23499	3.423304	3.23075	3.210869	3.251206	3.212373	3.12243	3.240345	3.248188	3.119294
CEPT1	1561884_at	3.084629	3.077407	3.060665	3.174991	3.106813	3.065825	3.967356	3.11538	3.634005	3.6628	2.962296	3.486267	2.964398
C10orf128	1561890_at	2.249279	2.239633	2.219523	2.223475	2.238388	2.221768	2.23768	2.241662	2.229559	2.225579	2.223455	2.237852	2.149083
ZMYM6	1561892_at	2.294236	2.273972	2.256215	2.26025	2.298731	2.259126	2.208274	2.2185	2.261909	2.257855	2.258205	2.313702	2.030514
LOC653739	1561894_at	3.129322	3.043813	2.924749	3.024255	3.124791	3.022456	3.007868	2.973165	3.026275	2.984135	3.027101	3.036578	2.915805
CLECL1	1561899_at	4.512538	4.453709	4.12065	4.267251	4.709723	4.138258	4.203307	4.284242	4.320705	4.245819	4.450944	4.529318	3.552604
HS3ST3B1	1561908_a_at	2.581693	2.589918	2.645131	2.573817	2.604696	2.578711	2.606566	2.588744	3.04843	3.068477	2.563591	2.6675	3.444523
SF3B14	1561923_a_at	2.662384	2.641225	2.621979	2.655438	2.631031	2.59283	2.636226	2.633767	2.615031	2.61818	2.621481	2.627111	2.586156

C3orf16	1561927_at	3.980731	3.874458	3.69654	3.851332	3.944666	3.712119	3.890028	3.858507	3.882177	3.862733	3.956006	4.038899	3.195951
C3orf16	1561928_s_at	2.696241	2.659222	2.585406	2.639491	2.687374	2.640218	2.649227	2.652422	2.642299	2.634791	2.636325	2.651953	2.454297
MAPKAPK5	1561937_x_at	1.853297	1.838212	1.814485	1.817497	1.828333	1.825523	1.830006	1.830939	1.825249	1.819462	1.827668	1.82073	1.809531
DYNC2H1	1561939_at	2.732449	2.658198	2.599703	2.642301	2.706982	2.64394	2.656721	2.666842	2.65353	2.631698	2.654591	2.677982	3.188109
POLE	1561940_at	2.379927	2.360618	2.321385	2.331866	2.360098	2.545555	2.342406	2.354476	2.34321	2.336173	2.336835	2.353518	2.295028
LOC283874	1561942_x_at	4.005649	3.722354	3.371921	3.580164	3.859857	3.970931	2.825005	2.663063	3.089524	2.810865	3.33637	2.522249	3.34549
COQ10B	1561948_at	2.512619	2.461791	2.454945	2.512293	2.561065	2.429818	2.466335	2.473821	2.472783	2.464481	2.469934	2.48222	2.31309
SLC5A12	1561951_at	3.192564	3.067524	2.994507	3.092228	3.223001	3.06988	3.074823	3.08316	3.099273	3.082364	3.129618	3.169701	2.784687
KIAA1920	1561960_at	2.294552	2.273782	2.254157	2.259175	2.270245	2.258502	2.261618	2.266868	2.255064	2.240678	2.252445	2.263776	2.209501
DKFZp686A	1561961_at	3.031637	2.927642	2.867884	2.933552	3.201944	3.020999	2.942403	2.947919	2.93318	2.906283	2.937096	2.948935	2.646329
CTBP2	1561963_at	2.737456	2.723258	2.659119	2.711204	2.725001	2.71095	3.002593	2.722836	2.989829	3.054974	2.70886	2.743578	2.595882
LOC441245	1561964_at	2.884903	2.870494	2.852039	2.860417	2.861572	2.847243	2.869872	2.886241	2.853125	2.8563	2.847352	2.862887	2.74865
SNRPB2	1561965_at	3.623311	3.430367	3.373546	3.422158	3.461994	3.386403	3.421182	3.350598	3.43021	3.415946	3.42808	3.440846	3.296383
ZPLD1	1561969_at	2.496228	2.459769	2.422469	2.440508	2.47915	2.421224	2.451991	2.461904	2.454564	2.431942	2.445914	2.458537	2.383081
SMARCC2	1561973_at	3.271261	3.235198	4.027814	3.748086	2.983703	3.092169	3.602694	3.255409	4.006183	3.336808	3.39901	3.473522	3.264772
C1orf167	1561976_at	2.314438	2.3006	2.27937	2.287093	2.316346	2.292447	2.296698	2.34965	2.293178	2.284162	2.299587	2.372381	2.259204
LOC284798	1561978_at	2.449251	2.418123	2.369971	2.395639	2.419004	2.408154	2.404856	2.416579	2.398865	2.387844	2.399928	2.411767	2.319973
C14orf39	1561985_at	2.840103	2.760403	2.699119	2.751003	2.850824	2.725541	2.766426	2.776321	2.763565	2.737678	2.778237	2.80032	3.892097
LOC286068	1561988_at	1.981796	1.963425	1.944611	1.943622	1.964017	1.947738	1.95876	1.963483	1.949304	1.943304	1.94934	2.053213	1.894539
LOC157931	1561990_at	1.795112	1.821233	1.768747	1.77343	1.782903	1.77449	1.781023	1.783069	1.780451	1.773012	1.878487	1.778002	1.725278
LOC440061	1561997_at	3.307866	3.159166	3.083147	3.14569	3.586181	3.066725	3.149074	3.166741	3.163066	3.142313	3.199268	3.217427	2.783393
LOC400620	1562000_at	2.320475	2.2819	2.257345	2.262103	2.30973	2.263264	2.274273	2.282112	2.269964	2.259972	2.267043	2.278679	2.162512
MYOZ3	1562002_at	2.37014	2.362545	2.341415	2.344071	2.355573	2.350947	2.362661	2.357625	2.347673	2.334339	2.346213	2.351501	2.358626
MYOZ3	1562004_x_at	2.300227	2.29503	2.24572	2.270753	2.291977	2.280132	2.282809	2.279011	2.278621	2.277003	2.273669	2.364193	2.294666
RIN3	1562005_at	1.99802	1.982274	1.963213	1.970243	1.976585	1.969115	2.022581	1.973702	1.973124	1.956415	1.963771	1.974148	1.908008
NT5DC4	1562019_at	2.876701	2.798517	2.720575	2.791048	2.875931	2.756789	3.662472	4.791441	4.06875	3.668758	3.903508	3.864026	2.659097
NT5DC4	1562020_s_at	2.700951	2.665499	2.625766	2.658842	2.679686	2.658259	2.870958	2.691013	3.011581	2.654408	2.888158	3.081613	2.204148
RAD9A	1562022_s_at	3.812101	4.089813	3.774049	3.726445	3.929476	3.780558	3.810968	3.792836	3.891521	3.787054	3.77323	3.726516	3.709168
WNK2	1562026_at	2.06171	2.056681	2.038741	2.041835	2.048638	2.041525	2.053954	2.053079	2.045499	2.039019	2.034715	2.046172	2.001381
CCND3	1562028_at	2.102455	2.084337	2.05568	2.065227	2.087919	2.065398	2.080837	2.081177	2.066846	2.05928	2.062348	2.075515	1.995842
LOC284898	1562030_at	2.177862	2.161201	2.139304	2.146133	2.160369	2.144606	2.14649	2.155646	2.144287	2.138809	2.139432	2.150111	2.095009
JAK2	1562031_at	5.027685	5.015864	5.171249	4.513793	5.348798	5.141861	6.351964	5.678072	6.73358	6.437598	5.360481	6.158746	3.942861
C21orf134	1562034_at	2.144699	2.125441	2.101335	2.10882	2.136362	2.106009	2.117797	2.120642	2.109678	2.106333	2.108358	2.121739	2.111133
LOC152225	1562048_at	3.035575	3.036951	2.910994	2.975806	3.073465	3.000156	2.983933	3.00169	2.98056	2.9467	2.999354	3.043926	2.641729
LOC284757	1562051_at	2.828276	2.81546	2.707044	2.800552	2.82322	2.788395	2.793682	2.793637	2.782333	2.738281	2.848594	2.806469	2.794153
SMEK3P	1562054_at	3.254834	3.075088	2.997802	3.097259	3.273247	3.036641	3.098519	3.109853	3.126707	3.076668	3.16566	3.265914	2.759955
KIAA1245	1562062_at	9.305964	9.314027	8.522463	8.665205	10.00101	10.46034	7.104768	8.207309	7.176873	6.639433	8.71395	7.806957	7.278647
KIAA1245	1562063_x_at	9.440485	9.478526	8.513252	8.60404	9.994301	10.46243	7.874755	8.726316	7.157808	7.381919	8.853931	8.086797	7.590861

LOC286144	1562066_at	1.90755	1.893356	1.874476	1.878193	1.894083	1.876585	1.888851	1.88868	1.879376	1.875189	1.87272	1.886997	1.829683
UNC13C	1562074_a_at	2.715906	2.667819	2.608977	2.652457	2.69231	2.61944	2.663934	2.672543	2.656482	2.642249	2.655836	2.668039	2.52639
GLYATL1	1562089_at	3.348349	3.163979	3.18035	3.231735	3.357124	3.109114	3.191283	3.198266	3.237252	3.153012	3.22947	3.289978	2.845264
AKR1C2	1562102_at	3.30757	3.299091	4.026687	3.75666	3.38926	3.279774	3.090741	3.152628	3.113632	4.263048	3.088157	3.124751	2.724606
JAK1	1562103_at	1.94543	1.935664	1.917712	1.918406	1.941194	1.918027	1.928095	1.933726	1.919851	1.911817	1.916874	1.928251	1.870165
MGC14738	1562107_at	3.041478	2.983502	2.944456	2.945941	3.062178	2.898582	2.948052	2.954712	2.969463	2.928401	2.98278	2.994998	2.682056
IQCA	1562130_at	2.660641	2.606582	2.555104	2.539797	2.700401	2.578185	2.611047	2.624252	2.607737	2.586496	2.605489	2.646609	2.431264
ZNF90	1562133_x_at	3.477016	3.268712	3.363438	3.382473	3.679224	3.337872	3.312809	3.394755	3.391909	3.318377	3.397719	3.483865	2.887577
FOXP2	1562139_a_at	2.983683	2.861544	2.792736	2.852239	2.98876	2.853894	2.856822	2.868185	2.874197	2.837051	2.877904	2.915143	2.887357
SKIV2L2	1562142_at	3.272272	3.136206	2.960325	3.075778	3.347883	3.716685	3.038656	3.129782	3.121303	3.049245	3.463976	3.296411	2.647016
PVT1	1562153_a_at	2.425471	2.389695	2.347318	2.369269	2.395964	2.368743	2.380155	2.392657	2.375737	2.363638	2.375667	2.388796	2.251911
DEFB122	1562167_a_at	2.439979	2.406266	2.375035	2.387817	2.423247	2.37762	2.39589	2.407683	2.392566	2.380367	2.393444	2.405407	2.264846
WDR21B	1562209_at	3.253062	3.155878	3.128316	3.140313	3.305835	3.046249	3.121893	3.163471	3.150714	3.092296	3.17587	3.24867	3.015384
ZNF491	1562211_a_at	1.863415	1.855001	1.834922	1.839186	1.854236	1.83704	1.850794	1.851052	1.84951	1.886945	1.837524	1.844779	1.911372
LOC151171	1562214_at	2.751772	2.740337	2.721668	2.710467	2.766211	2.72605	2.726642	2.739843	2.724196	2.72478	2.725642	2.734143	2.51139
FLJ34521	1562217_at	2.827086	2.761615	2.687749	2.746676	2.821272	2.745996	2.756674	2.774321	2.749524	2.741807	2.739709	2.764918	2.64303
FLJ41649	1562219_at	2.59301	2.578125	2.559225	2.555996	2.600946	2.837722	2.566787	2.576417	2.563912	2.544002	2.558365	2.572506	2.442715
LOC642426	1562223_at	3.552819	3.357618	3.239655	3.375418	3.606814	3.302922	3.395975	3.382842	3.410391	3.355768	3.473162	3.5601	2.92359
FLJ14712 //	1562226_at	2.682464	2.624968	2.572406	2.615387	2.681454	2.588867	2.627423	2.637952	2.623863	2.601551	2.62222	2.653261	2.688271
PDE5A	1562227_at	1.358118	1.35303	1.333703	1.334029	1.342346	1.339509	1.34528	1.340867	1.341049	1.33493	1.332452	1.335435	1.332512
PDE5A	1562228_s_at	2.477036	2.443492	2.405812	2.433955	2.454386	2.706556	2.41988	2.445368	2.436405	2.582518	2.435739	2.441329	2.385599
NAV3	1562234_a_at	2.465634	2.438671	2.389129	2.417306	2.467047	2.409784	2.42571	2.436531	2.420025	2.410734	2.417874	2.429513	4.022426
MYST4	1562236_at	4.404724	4.041935	3.839052	4.22981	4.852258	4.017981	4.107973	4.10311	4.181131	4.07715	4.415482	4.508662	3.828031
USPL1	1562238_at	4.507486	3.76073	3.679081	3.722174	4.001155	3.778035	3.749619	3.76856	3.990826	3.726294	3.767137	3.893192	3.505959
SGSM1	1562239_at	2.478455	2.465238	2.437971	2.450043	2.465265	2.448108	2.461065	2.463627	2.462154	2.434802	2.44726	2.877137	2.385422
PLXNA4A	1562240_at	2.921254	2.912621	2.793608	2.830032	2.880685	3.070406	2.856457	2.86576	2.853154	2.841275	2.854476	3.055204	2.742202
LOC286058	1562247_at	2.20983	2.19191	2.174391	2.168498	2.186193	2.197918	2.327071	2.190724	2.175695	2.167829	2.173905	2.185276	2.08921
LOC285965	1562249_at	3.538218	3.27182	3.473506	3.304077	3.877412	3.66346	3.319749	3.284727	3.317106	3.282206	3.57613	3.464332	2.948764
LOC574538	1562251_a_at	2.141599	2.115619	2.098756	2.10271	2.13304	2.10054	2.111245	2.113092	2.105125	2.0977	2.097389	2.110542	1.96235
LOC641364	1562253_at	2.097586	2.084881	2.058463	2.100117	2.086781	2.065808	2.077109	2.080585	2.068213	2.063732	2.065015	2.075769	2.017681
SYTL3	1562255_at	3.864798	3.464969	3.948847	4.036576	4.139054	3.683028	3.495551	3.341938	3.527152	3.359292	4.28071	3.822028	3.433401
NLRP1	1562256_at	1.194505	1.187303	1.17024	1.173646	1.179656	1.17196	1.186224	1.173533	1.182524	1.171679	1.165309	1.165119	1.188687
NLRP1	1562257_x_at	1.274529	1.268415	1.250221	1.253429	1.263985	1.254721	1.266148	1.255014	1.261633	1.253378	1.247212	1.245273	1.262652
TEX9	1562259_at	2.87458	2.804051	2.788218	2.784753	2.829065	2.773853	2.794172	2.803783	2.786066	2.76273	2.788495	2.802888	2.559658
AMZ1	1562261_at	2.782732	2.686977	2.661486	2.670595	2.691049	2.646776	2.682302	2.675182	2.679656	2.627635	2.678601	2.72056	2.680892
LOXL2	1562263_at	1.851199	1.837296	1.817359	1.819466	1.833555	1.821612	1.833147	1.831581	1.821017	1.815853	1.815639	1.825152	1.765532
LOC339685	1562264_at	2.064017	2.045874	2.021742	2.027366	2.047327	2.026144	2.038027	2.04429	2.030056	2.031188	2.027993	2.039245	1.975943
ZNF564	1562267_s_at	1.827015	1.816034	1.796874	1.800858	1.814954	1.798588	1.811702	1.810358	1.801932	1.797361	1.793753	1.80601	1.75835

ARHGEF7	1562270_at	4.641356	4.305099	4.261089	4.21984	4.612855	4.841424	4.136285	4.278062	4.368406	4.096972	4.266615	4.27094	3.823444
ARHGEF7	1562271_x_at	5.401945	5.170768	4.54468	4.551031	4.53113	4.389231	4.448743	4.450786	4.491082	4.441768	4.279332	4.449323	4.06751
CNGA4	1562273_at	3.330169	3.310091	3.528974	3.284511	3.31884	3.357907	3.307356	3.31248	3.300331	3.296398	3.292856	3.446283	3.168875
DNAH1	1562278_at	2.167532	2.536219	2.103143	2.111891	2.155705	2.111471	2.119462	2.128765	2.114083	2.1068	2.111389	2.121889	2.034407
ZNF568	1562282_at	2.553797	2.52013	2.456526	2.496659	2.548705	2.501218	2.67858	2.530054	2.504204	2.492504	2.516362	2.524665	3.370645
SNTG1	1562287_at	3.336111	3.186211	3.084092	3.189817	3.402012	3.193259	3.194415	3.193955	3.20999	3.163263	3.254176	3.301692	2.822794
ANKRD30B	1562292_at	3.711995	3.782374	3.339529	3.515663	3.813371	3.346778	3.533214	3.547434	3.572144	3.438033	3.636052	3.659046	3.11343
ANKRD30B	1562294_x_at	3.329221	3.14687	3.074891	3.194803	3.457314	3.140114	3.179141	3.211026	3.217372	3.15304	3.263926	3.327313	2.828205
LOC730057	1562295_at	2.28403	2.247679	2.228659	2.240579	2.3204	2.2351	2.244072	2.255597	2.239286	2.226881	2.241928	2.250288	2.142275
C14orf25	1562299_at	2.510672	2.477923	2.462024	2.456006	2.480491	2.464472	2.466012	2.477021	2.461892	2.449222	2.458749	2.471861	2.35671
C8orf34	1562301_at	3.508906	3.274104	3.246794	3.31879	3.530664	3.303663	3.310936	3.3207	3.342027	3.284785	3.464645	3.467902	3.318793
ZKSCAN3	1562303_at	2.558826	2.545343	2.411664	2.481689	2.602341	2.4416	2.854982	3.531496	3.646751	3.009355	3.772908	3.544164	2.331548
ZKSCAN3	1562305_x_at	2.765719	2.510236	2.473835	2.493091	2.559627	3.007389	2.659847	3.815418	2.577138	2.674305	3.080622	2.618451	2.259477
LOC283682	1562308_at	2.538294	2.467092	2.414335	2.453491	2.474022	2.452491	2.457688	2.470274	2.450041	2.404506	2.453284	2.466758	2.367064
PHF21B	1562309_s_at	2.601116	2.560368	2.508551	2.533814	2.596235	2.543498	2.55824	2.569317	2.556641	2.545029	2.57449	2.565914	4.359265
BCORL2	1562313_at	2.812911	2.713961	2.660782	2.709882	2.779696	2.680835	2.731859	2.83577	2.719415	2.695982	2.730121	2.76131	2.592032
LOC121006	1562317_at	3.071117	2.883506	2.781113	2.843688	2.903043	3.00728	2.862902	2.866939	2.869216	2.829062	2.947457	2.953994	2.599579
FLJ39743	1562319_at	2.428829	2.080236	2.045184	2.058378	2.080247	2.442426	2.054042	2.071659	2.043021	2.037374	2.036114	2.071422	1.971386
LOC399875	1562320_at	2.177275	2.159299	2.131701	2.140625	2.165057	2.143648	2.150791	2.15975	2.14465	2.137453	2.141196	2.15747	2.06324
PDK4	1562321_at	5.019114	5.299286	6.41659	6.11932	6.961869	6.599243	2.958848	2.965774	2.676717	2.512791	2.983238	3.021767	2.665506
FLJ30838	1562326_at	1.990855	1.974246	1.952362	1.957519	1.977106	1.955785	1.967574	1.968306	1.95849	1.951794	1.953729	1.962967	1.92599
CSMD1	1562329_at	3.149475	3.000527	2.926216	3.006433	3.151992	2.982703	3.023002	3.019581	3.028228	2.993197	3.060445	3.101832	2.702897
CSMD1	1562330_s_at	2.203833	2.171773	2.153467	2.157138	2.187493	2.155555	2.160223	2.169164	2.157815	2.148879	2.150367	2.165208	2.071927
CASC2	1562336_at	2.547611	2.535927	2.518096	2.512582	2.557586	2.522174	2.532642	2.533227	2.519272	2.51924	2.548858	2.53422	2.481267
OR7D2	1562337_at	2.625159	2.615589	3.627587	3.623375	2.626232	2.599781	3.370336	4.020678	3.904555	2.569734	2.602603	2.624651	4.3226
MARCH1	1562338_at	2.591101	2.553481	2.501994	2.535656	2.580019	2.529121	2.542486	2.556766	2.540187	2.520049	2.539444	2.555826	2.392905
LOC400680	1562348_at	2.398992	2.368358	2.334736	2.356604	2.376255	2.343132	2.36965	2.403488	2.609158	2.350268	2.354071	2.361601	2.31126
GVIN1	1562364_at	3.058556	2.939299	2.845653	2.908454	3.087763	3.066758	2.972081	2.911558	2.892232	2.892032	2.912653	2.945101	2.64352
LOC286177	1562365_at	3.382472	3.26761	3.164988	3.251826	3.288031	3.205743	3.218991	3.225616	3.25669	3.193122	3.331921	3.368934	2.832785
FLJ39531	1562367_at	2.077049	2.05101	2.04026	2.038587	2.102738	2.429791	2.055856	2.059008	2.054153	2.038886	2.045391	2.053893	1.989939
VWA3B	1562371_s_at	2.512969	2.466032	2.420705	2.455038	2.491571	2.449641	2.464213	2.469744	2.456592	2.441116	2.470068	2.5047	2.316788
SV2C	1562372_at	2.440985	2.410827	2.393075	2.394933	2.437631	2.391565	2.403915	2.411232	2.398686	2.385152	2.392991	2.410161	2.269875
PROM2	1562378_s_at	2.149843	2.128237	2.103938	2.110836	2.125647	2.108529	2.121108	2.128549	2.114473	2.107497	2.114239	2.123053	2.162737
RP3-377H14	1562381_at	3.569374	3.256326	3.180612	3.331915	3.66536	3.272565	3.323989	3.292279	3.364916	3.291522	3.422165	3.595327	2.969279
ZNF501	1562386_s_at	3.153845	3.093354	3.063009	3.029867	3.102271	3.011239	3.096216	3.099405	3.107811	3.088211	3.08964	3.16601	5.147691
FLJ13231	1562387_at	3.120635	3.021103	2.954024	2.998499	3.10864	2.944377	3.007786	3.040212	3.034445	2.994979	3.037736	3.120309	2.94765
LOC285819	1562388_at	2.696266	2.689132	2.667452	2.674074	2.720724	2.670827	2.681753	2.686296	2.673387	2.66638	2.672096	2.681991	2.618008
B3GALNT2	1562391_at	4.053203	3.736082	4.252441	4.642947	3.879914	3.659484	3.943993	3.861279	3.618089	3.348179	3.888547	3.825438	3.583263

MRPL23	1562392_at	2.280409	2.49323	2.222857	2.258741	2.563393	2.297799	2.518767	2.272124	2.279995	2.264434	2.267449	2.268273	2.206601
PP8961	1562396_at	3.438315	3.263758	3.156359	3.274654	3.462415	3.277686	3.314493	3.326433	3.300395	3.241716	3.34002	3.408649	2.863766
LOC283112	1562400_at	3.354916	3.184351	3.134917	3.220429	3.393662	3.154937	3.204463	3.240972	3.241845	3.20357	3.275398	3.294217	2.904006
SLC8A3	1562403_a_at	2.405786	2.385916	2.369096	2.372507	2.388941	2.311746	2.385208	2.382569	2.370508	2.372547	2.369547	2.377215	4.566892
MYLK2	1562411_at	2.20986	2.182828	2.159544	2.16686	2.192083	2.168557	2.196069	2.186256	2.17059	2.162838	2.172199	2.178149	2.10702
C11orf37	1562413_at	2.462166	2.438848	2.413038	2.422364	2.451143	2.418729	2.429049	2.437641	2.425368	2.414266	2.423236	2.436055	2.29133
SPOCD1	1562415_a_at	2.661515	2.754653	2.429451	2.49801	2.62459	2.512293	2.520831	2.526212	2.514362	2.432316	2.670804	2.918246	2.276864
LOC285889	1562424_at	2.880817	2.848301	2.757671	2.834774	2.904052	2.84208	2.844641	2.832277	2.836366	2.825264	2.837679	2.847294	2.730481
FARP1	1562425_at	2.187947	2.162589	2.144959	2.149711	2.185103	2.149517	2.161037	2.161649	2.14965	2.142129	2.143586	2.154134	2.021319
FARP1	1562426_a_at	2.278951	2.171482	2.138814	2.14814	2.179809	2.155154	2.157877	2.167387	2.155953	2.148976	2.151082	2.165817	2.086757
LOC654780	1562428_at	4.53133	4.25282	4.105531	4.31993	4.623593	4.386207	4.060835	4.144844	4.086587	4.130423	4.709581	4.623013	3.7708
FLJ10489	1562433_at	2.01797	2.001945	1.982731	1.983697	2.002198	1.982929	1.9949	2.000709	1.987654	1.981891	1.983075	1.993716	1.926462
UNK	1562434_at	3.068405	3.483354	2.950852	2.902425	3.00112	2.932271	2.911223	4.205419	3.582414	3.212075	3.77442	3.607716	2.901576
CARS	1562436_at	1.948033	1.93176	1.900861	1.913168	1.946546	1.915689	1.928581	1.932699	1.917691	1.911434	1.915756	1.925157	1.830855
NCOA3	1562439_at	2.630372	2.543575	2.494424	2.534745	2.579775	2.533713	2.542711	2.549117	2.539552	2.520716	2.54865	2.56659	2.380559
MAP3K13	1562440_at	5.731504	5.85832	5.430701	5.617681	5.923601	5.846501	3.856837	3.64977	3.694449	3.242423	3.857419	3.683783	3.402545
SSBP1	1562442_a_at	2.424462	2.399903	2.419809	3.442039	2.538991	2.42111	2.420303	2.421656	2.469831	2.355643	2.408429	2.415437	2.429062
RLBP1L2	1562443_at	2.937661	2.887438	2.882485	2.88076	2.926058	2.853658	2.890132	2.897096	2.884584	2.868818	2.896795	2.924879	2.63243
RP1-153G1	1562446_at	2.398808	2.365859	2.326997	2.344955	2.428101	2.33814	2.354439	2.365022	2.350273	2.338339	2.350862	2.359689	2.267893
LOC283331	1562457_at	2.38234	2.328308	2.313517	2.333053	2.371686	2.44684	2.340082	2.351789	2.335629	2.323116	2.371177	2.373116	2.281235
UBE2W	1562458_at	2.37461	2.344638	2.327119	2.330508	2.359275	2.333117	2.332931	2.344046	2.330081	2.318736	2.328887	2.335419	2.303999
CNDP2	1562460_at	3.141814	3.565743	3.104707	3.107292	3.191343	3.588324	3.066492	3.117679	3.065078	3.093229	3.103396	3.111112	2.804167
DNAH10	1562462_at	2.199784	2.187408	2.164539	2.168009	2.190565	2.169017	2.176553	2.321724	2.171564	2.166841	2.162315	2.180937	2.085464
FLJ22536	1562469_at	2.362479	2.340527	2.32226	2.326882	2.354328	2.327072	2.329307	2.338131	2.324251	2.312034	2.325026	2.331485	2.257202
C1orf83	1562470_at	2.446303	2.39847	2.3763	2.381063	2.449947	2.387427	2.393839	2.401099	2.390388	2.372762	2.386657	2.399804	2.305029
DKFZp686C	1562475_at	2.187181	2.162657	2.135499	2.145552	2.169563	2.14592	2.158439	2.160072	2.145903	2.143884	2.150277	2.153723	2.066546
EBF2	1562477_at	2.733027	2.665754	2.608781	2.645891	2.820011	2.6311	2.663676	2.681034	2.666812	2.644583	5.069725	2.694318	6.162679
FLJ35848	1562484_at	2.788008	2.737588	2.686212	2.707625	2.777021	2.682718	2.723599	2.731382	2.717616	2.696642	2.722365	2.769709	2.515746
C18orf30	1562488_at	2.060772	2.047031	2.025286	2.029807	2.046295	2.030086	2.036549	2.043606	2.030329	2.023085	2.028243	2.036969	2.041707
THEM5	1562490_at	2.503722	2.466355	2.427161	2.448705	2.502693	2.442419	2.45833	2.4692	2.451422	2.440697	2.451622	2.46667	2.318778
LOC340090	1562492_at	2.82022	2.685994	2.664361	2.690594	2.757449	2.699753	2.70416	2.726913	2.708098	2.697882	2.746796	2.779504	2.50061
ANKFY1	1562495_at	1.948779	1.936631	1.885343	1.918942	1.93548	1.925536	1.931218	1.932803	1.920501	1.916167	1.920608	1.926689	1.828524
LOC339539	1562496_at	1.975885	1.971386	1.951314	1.95535	1.962724	1.964035	1.96842	2.017103	1.969777	1.955985	1.954017	1.957833	1.923496
LOC255177	1562501_at	2.589075	2.549559	2.504392	2.536658	2.655348	2.515042	2.547217	2.555087	2.540911	2.525088	2.540815	2.555794	2.400988
LOC339442	1562510_at	3.071868	3.06514	2.981198	3.033537	3.157524	3.032711	3.041438	3.053249	3.027521	3.023356	3.023235	3.052535	2.924035
LYST	1562511_at	2.83927	2.754362	2.732244	2.783205	2.763579	2.70235	2.748422	2.755179	2.744093	2.731194	2.73376	2.746134	2.681572
SMPDL3A	1562512_at	2.386102	2.354601	2.3059	2.336306	2.376472	2.325395	2.344205	2.354961	2.346578	2.327646	2.339753	2.350729	2.223011
WDR60	1562516_at	3.135455	3.029149	2.902429	2.993247	3.142936	2.962536	3.028267	3.014191	2.999824	2.956369	3.048879	3.103691	4.238484

LOC283027	1562527_at	2.610765	2.569277	2.499488	2.544878	2.611504	2.527216	2.555901	2.565165	2.550971	2.534377	2.550591	2.584954	2.402643
FCER1A	1562537_at	3.170784	3.216443	3.03836	3.08001	3.151913	3.06868	3.106847	3.109461	3.093328	3.081586	3.099669	3.133135	2.719276
LOC339978	1562540_at	2.123193	2.11187	2.085967	2.092404	2.11317	2.060875	2.107624	2.109761	2.097137	2.09669	2.094446	2.104595	2.02207
LOC285928	1562541_at	2.866234	2.789118	2.712813	2.776649	2.849946	2.780881	2.78538	2.788787	2.787982	2.765812	2.785475	2.812782	2.55205
LOC440704	1562558_at	2.535943	2.493294	2.462944	2.481863	2.548171	2.479938	2.490076	2.500538	2.485082	2.475033	2.483137	2.518927	2.376382
C9orf89	1562559_at	2.396884	2.373016	2.334484	2.354929	2.377519	2.351929	2.369354	2.371216	2.362271	2.356916	2.361581	2.417877	2.245443
FLJ40759	1562562_at	2.276811	2.250495	2.222273	2.236112	2.254701	2.232013	2.243339	2.249858	2.235903	2.228631	2.23148	2.242388	2.136722
HCN1	1562563_at	1.477238	1.47179	1.45336	1.456496	1.519739	1.453823	1.466738	1.457202	1.460101	1.449932	1.45109	1.460717	1.469212
LOC643749	1562566_at	3.017695	2.911938	2.830557	2.892554	2.986191	2.851001	2.90519	2.92695	2.912271	2.874909	2.968619	2.968086	2.670451
LOC400541	1562568_at	2.434594	2.409534	2.366227	2.396425	2.42906	2.420759	2.408768	2.411016	2.389823	2.383874	2.393295	2.473062	2.349266
CYP17A1	1562573_at	4.033565	3.916457	3.594545	3.858531	4.268706	3.895993	3.769238	3.911936	3.901623	3.830673	4.042435	4.113738	3.509429
LOC254028	1562581_at	2.026934	2.012586	1.990332	1.994729	2.013152	1.994123	2.005465	2.008884	1.996473	1.989502	1.989433	2.005137	1.953961
LOC646405	1562582_at	3.543738	3.4196	3.345985	3.396636	3.738627	3.250625	3.429368	3.437302	3.43232	3.400402	3.466153	3.610995	2.917767
LOC646405	1562583_s_at	3.258904	3.155651	3.169951	3.203238	3.416082	3.089357	3.212486	3.204983	3.198102	3.189533	3.206282	3.357321	4.085503
LOC284263	1562585_at	1.938476	1.915899	1.896879	1.900206	1.921596	1.898797	1.912163	1.915132	1.902861	1.895676	1.901149	1.91245	1.850723
MIST	1562587_at	2.615803	2.562655	2.557935	2.550159	2.664696	2.561379	2.563397	2.568877	2.559839	2.542618	2.559442	2.610266	2.394778
PRTG	1562590_at	2.384225	2.687894	2.33407	2.340618	2.367493	2.858788	2.341053	2.352562	2.340786	2.332858	2.337789	2.507445	2.220866
OFCC1	1562591_a_at	2.364287	2.32944	2.282476	2.296462	2.358243	2.301084	2.31794	2.330234	2.316046	2.301813	2.313979	2.327901	2.201869
UNQ6975	1562601_at	2.453752	2.439217	2.367713	2.42197	2.452386	2.859223	2.416165	2.436068	2.429994	2.40506	2.423604	2.44178	2.256223
RFFL	1562603_at	2.189131	2.200547	2.157489	2.148547	2.15669	2.145534	3.612586	2.171193	2.947892	2.230813	2.158111	2.164155	2.069975
LOC440028	1562606_a_at	2.249576	2.230994	2.194306	2.211389	2.232724	2.214484	2.227067	2.228209	2.214187	2.203089	2.213751	2.221653	2.166494
LOC340074	1562617_at	1.920605	1.909783	1.889693	1.895046	1.918635	1.89301	1.904308	1.907013	1.894923	1.88878	1.887879	1.900606	1.846866
TXNDC6	1562619_at	1.900685	1.888318	1.870051	1.873472	1.888535	1.872792	1.887246	1.887601	1.87965	1.874802	1.86748	1.880434	1.826199
LOC146513	1562623_at	2.48594	2.449761	2.409267	2.434053	2.482674	2.417897	2.443423	2.451228	2.439091	2.42345	2.437406	2.472659	2.302921
FRYL	1562625_at	2.397219	2.369289	2.328094	2.351082	2.386162	2.35502	2.35898	2.365736	2.355837	2.343572	2.349786	2.36611	2.266059
KRT40	1562628_at	3.695586	3.446507	3.367304	3.521731	3.832318	3.520115	3.488316	3.485713	3.541967	3.468034	3.635868	3.7202	3.830068
KRT40	1562629_a_at	2.314874	2.298651	2.278656	2.282105	2.312783	2.282269	2.293177	2.300663	2.283684	2.273882	2.279866	2.329262	2.235574
LOC285191	1562632_at	1.985094	1.974118	1.953339	1.96207	1.973194	1.956947	1.966566	2.23767	1.960215	1.963976	1.952787	1.96264	1.91294
RMST	1562633_at	2.290695	2.267616	2.235589	2.246839	2.276719	2.253755	2.257545	2.269184	2.254468	2.240859	2.253148	2.262776	2.188056
SAMD12	1562637_at	3.749566	3.702326	3.438047	3.611517	3.879087	3.678613	3.694781	3.707381	3.658226	3.676753	3.721002	3.703524	3.101469
LOC339874	1562638_at	2.336886	2.308483	2.284482	2.31177	2.320405	2.279428	2.296421	2.308302	2.293147	2.283287	2.290236	2.303001	2.183763
KIF6	1562639_at	2.178595	2.15991	2.138091	2.14139	2.158465	2.142224	2.156144	2.157402	2.148265	2.144051	2.142042	2.154909	2.081816
LOC285943	1562640_at	2.835386	2.794709	2.744379	2.787609	2.836629	2.801587	2.787995	2.802492	2.789142	2.77377	2.818	2.801202	2.624265
FAM122C	1562641_at	2.796794	2.742608	2.982821	2.732096	2.77896	2.733408	2.89909	2.766633	2.741856	2.789895	2.725619	2.757671	2.570212
MTHFD2L	1562644_at	3.321206	3.14703	3.195445	3.200513	3.491512	3.189244	3.16788	3.003963	3.194749	3.147167	3.298388	3.271064	2.70668
LOC401176	1562645_at	3.119919	3.02002	2.971038	2.99799	3.07585	2.996285	2.976979	2.98326	2.996834	2.949562	3.030015	3.034108	2.704126
CCDC88A	1562648_at	3.063342	2.993659	2.978058	2.999059	3.049916	2.940438	3.928581	3.002489	3.759819	3.872218	3.035082	3.073667	5.023139
FRYL	1562650_at	3.472014	3.450616	3.403877	3.423021	3.487582	3.435775	3.446155	4.140233	3.453464	3.427821	3.816025	3.853031	3.110897

ZSCAN1	1562651_at	2.02198	2.006395	1.982629	1.989253	2.006302	1.988119	1.999162	2.003967	1.991162	1.986896	1.987159	1.99856	1.93014
C10orf90	1562657_a_at	3.265944	3.288681	3.213526	3.555746	3.271635	3.365797	3.244436	3.180598	3.205414	3.152811	3.1329	3.247302	3.040581
LOC400548	1562659_at	1.997894	1.98635	1.96573	1.96925	1.984929	1.977608	1.979571	1.983642	1.977212	1.975785	1.971636	1.978341	1.887738
LOC286009	1562664_at	2.267426	2.260031	2.220591	2.241834	2.265859	2.239547	2.251928	2.241072	2.251595	2.235529	2.242027	2.240684	2.199925
C1orf86	1562675_at	2.544741	2.552599	2.533404	2.535567	2.546745	2.541267	2.549362	2.549115	2.544249	2.532792	2.528284	2.533394	2.416241
KRTAP5-2	1562681_at	2.741653	2.730588	2.704006	2.717163	2.738121	3.41421	2.720437	2.730281	2.727002	2.700611	2.702089	2.706599	2.49589
LOC283666	1562682_at	2.286764	2.247163	2.229784	2.240775	2.269213	2.240698	2.246337	2.258853	2.238054	2.230348	2.244815	2.253411	2.143802
LOC285547	1562683_a_at	2.566837	2.505974	2.480922	2.488101	2.538804	2.466284	2.491389	2.503259	2.487934	2.48306	2.490286	2.509785	2.326951
LOC151484	1562689_at	2.17925	2.155483	2.128685	2.139403	2.164061	2.142353	2.146663	2.15696	2.140693	2.136438	2.143492	2.151066	2.05345
C3orf65	1562694_at	2.207848	2.14763	2.125309	2.127802	2.196939	2.129703	2.142342	2.149734	2.134703	2.125711	2.136675	2.147213	2.078345
FOXN4	1562695_at	2.422692	2.407694	2.379604	2.388067	2.405017	2.394049	2.390364	2.402414	2.384233	2.372439	2.390184	2.447748	2.205198
LOC339988	1562697_at	3.731752	3.625577	3.044091	3.182412	3.32613	4.611413	2.848093	2.820845	2.914424	2.822226	3.136778	3.199446	2.443349
LOC339988	1562698_x_at	4.178067	3.586975	2.907397	2.896178	4.887351	4.226034	2.916771	2.931468	2.91327	2.901464	2.914083	2.919848	2.239602
NCAM1	1562699_at	2.665226	2.637191	2.608624	2.614449	2.639577	2.623467	2.62211	2.630804	2.626955	2.621866	2.619058	2.626667	2.500975
LOC157381	1562703_at	2.180034	2.168642	2.136221	2.154492	2.168421	2.143703	2.165357	2.169024	2.156957	2.152948	2.158442	2.162543	2.121242
NETO1	1562713_a_at	2.562533	2.486644	2.452907	2.457757	2.550587	2.456843	2.471533	2.478325	2.470487	2.46344	2.472588	2.563809	2.430885
LOC284632	1562716_at	2.714923	2.692816	2.662556	2.678111	2.693886	2.670312	2.681593	2.691991	2.681785	2.677383	2.67776	2.68547	2.593164
C2orf46	1562717_at	2.406234	2.384739	2.344746	2.367333	2.394785	2.365612	2.368076	2.378736	2.363271	2.35332	2.368624	2.37663	2.243252
FLJ40296 //	1562722_at	2.233884	2.240327	2.175371	2.196775	2.21651	2.189232	2.200577	2.234884	2.199463	2.192216	2.196993	2.206082	2.128069
LOC286114	1562724_at	3.151513	3.10967	3.036962	3.092051	3.29634	3.091689	3.076005	3.105861	3.089469	3.089823	3.061712	3.113876	2.853843
MDS2	1562731_s_at	2.606863	2.762071	2.557182	2.563014	2.584193	2.576525	2.555653	2.585819	2.580425	2.54994	2.570485	2.578285	2.532724
LHX9	1562736_at	2.697394	2.655808	2.586141	2.64132	2.686536	2.640721	2.643215	2.653137	2.642921	2.63748	2.635382	2.649022	4.034333
LOC285593	1562739_at	2.327338	2.302848	2.269688	2.283398	2.32516	2.277878	2.291515	2.302713	2.286593	2.274232	2.285394	2.294356	2.167087
LOC285224	1562740_at	2.537504	2.490499	2.424645	2.47337	2.506026	2.465437	2.480484	2.488489	2.476944	2.466746	2.479758	2.500616	2.300187
UBXD2	1562741_at	1.969973	1.954665	1.932963	1.936831	1.963995	1.938677	1.947101	1.951648	1.938965	1.932002	1.933655	1.944795	1.883752
ZNF83	1562743_at	2.390042	2.371942	2.35299	2.351885	2.371994	2.349005	2.345393	2.365476	2.387396	2.359179	2.408245	2.359014	2.277567
LOC253044	1562748_at	2.263162	2.242278	2.206768	2.45318	2.243062	2.213074	2.226955	2.233131	2.216342	2.208228	2.223642	2.241355	2.131577
LOC641912	1562749_at	2.132266	2.148616	2.094806	2.10051	2.113411	2.10651	2.110547	2.110052	2.100941	2.093716	2.092645	2.107829	2.024664
ST6GALNA(	1562753_at	2.802433	2.778758	2.735678	2.753389	2.827857	2.745863	2.774581	2.77464	2.76266	2.737365	2.764746	2.771617	2.539206
LOC339260	1562754_at	2.150849	2.134467	2.114172	2.111922	2.135818	2.12121	2.130456	2.132543	2.12018	2.113245	2.114872	2.281689	2.065547
C9orf95	1562761_at	3.487784	3.306394	3.507072	3.2995	3.745639	3.413163	3.308894	3.318291	3.330594	3.32309	3.316939	3.314421	3.208063
C3orf56	1562762_at	3.090772	3.029426	2.951085	3.002589	3.095615	3.003709	3.005991	3.033413	3.016111	2.988827	3.020875	3.051333	2.706852
DAND5	1562772_a_at	2.627704	2.594713	2.524141	2.592633	2.611339	2.602084	2.604893	2.609394	2.608495	2.587103	2.621056	2.653606	2.419949
NUDT12	1562775_at	3.118549	3.070863	3.067854	3.24572	3.12854	3.042292	3.068094	3.078453	3.078594	3.046767	3.137698	3.074825	2.927375
LOC339807	1562776_at	2.530193	2.49221	2.459657	2.473176	2.497	2.480453	2.493705	2.49531	2.482186	2.465054	2.479274	2.493523	2.339065
LOC401565	1562778_at	2.0929	2.087068	2.065489	2.070825	2.284512	2.072246	2.074212	2.081854	2.070048	2.060532	2.065027	2.079861	1.974213
HERC6	1562785_at	2.318307	2.305286	2.283607	2.285806	2.304227	2.287129	2.302662	2.303215	2.291531	2.28955	2.284971	2.299893	2.253291
LOC254099	1562788_at	2.594109	2.567277	2.510803	2.547123	2.573938	2.532946	2.555004	2.561212	2.549092	2.533139	2.552919	2.563322	2.381015

ZNF229	1562789_at	2.670483	2.631007	2.587726	2.617869	2.730239	2.624479	2.63574	2.639377	2.629025	2.621132	2.709212	2.648862	2.956513
NCALD	1562790_at	2.467844	2.437966	2.391266	2.420639	2.450652	2.41634	2.427462	2.437535	2.424146	2.412454	2.420499	2.433376	2.289102
MYCBPAP	1562791_at	2.92742	2.892518	2.797372	2.874558	2.922444	2.876784	2.873661	2.936264	2.875135	2.869119	2.883178	2.917133	2.610095
LOC643464	1562792_at	2.291258	2.339219	2.248092	2.250617	2.25511	2.241222	2.249046	2.249	2.245809	2.242886	2.232975	2.241011	2.149097
C9orf29	1562803_at	2.410076	2.37636	2.334743	2.359219	2.393764	2.349777	2.367395	2.374742	2.363869	2.352298	2.362606	2.374106	2.240714
LOC349408	1562805_at	2.156402	2.132279	2.099647	2.107734	2.220378	2.107801	2.124514	2.127627	2.116323	2.107728	2.11789	2.170422	2.033863
C10orf44	1562812_at	2.382019	2.355122	2.31112	2.332974	2.364284	2.322343	2.344368	2.353984	2.339552	2.331095	2.337858	2.354893	2.246505
EFCAB6	1562815_at	2.860003	2.783498	2.740347	2.758761	2.881046	2.888466	2.776324	2.781169	2.775419	2.756566	2.771622	2.791181	2.643402
NMNAT2	1562818_at	2.023043	2.009624	1.987757	1.992295	2.009157	1.991509	2.004154	2.009729	1.994949	1.987199	1.991769	2.003344	1.940687
LOC339568	1562829_at	2.557978	2.528799	2.471686	2.506287	2.534321	2.535682	2.519914	2.525857	2.511231	2.495879	2.5105	2.524288	2.361099
METT5D1	1562830_at	2.147663	2.134413	2.114905	2.118807	2.133646	2.116747	2.13052	2.134784	2.11933	2.112088	2.116577	2.124946	2.074625
LOC283089	1562831_a_at	2.318372	2.304665	2.282954	2.282936	2.310644	2.287039	2.299005	2.301224	2.290027	2.283184	2.28932	2.304124	2.226724
C3orf65	1562832_at	3.031483	3.020255	2.982194	2.998875	3.020149	3.002101	3.016525	3.020639	3.005205	2.993891	2.999031	3.013837	2.621976
FLJ44451	1562839_at	2.610138	2.547787	2.514018	2.520807	2.574513	2.530764	2.533722	2.544993	2.530633	2.532371	2.527712	2.541704	2.374175
LOC339666	1562841_at	2.650188	2.642243	2.552425	2.610722	2.673227	2.723241	2.620077	2.672684	2.623261	2.595986	2.625669	2.646932	2.438004
LOC339822	1562844_at	2.608319	2.567546	2.525993	2.544668	2.607546	2.537613	2.561284	2.572106	2.557079	2.531453	2.55647	2.592902	2.357367
EP400NL	1562845_at	2.107925	2.095743	2.074075	2.078154	2.094433	2.077551	2.085965	2.091202	2.079268	2.074056	2.078948	2.085905	2.029076
LOC541471	1562876_s_at	3.865094	3.633409	3.456031	3.67818	3.979775	3.530267	3.65913	3.67903	3.675387	3.592794	3.90585	3.884113	3.301609
GLB1L3	1562888_at	2.146473	2.127222	2.108614	2.113167	2.122991	2.114522	2.120983	2.115164	2.110383	2.112777	2.106817	2.115735	2.044274
CHCHD5	1562892_at	1.598975	1.591209	1.572962	1.577579	1.583867	1.574166	1.58848	1.586398	1.5836	1.577579	1.574261	1.580128	1.550025
KIAA1856	1562896_at	3.043901	3.035739	3.000927	3.02385	3.026694	3.016071	3.039514	3.248217	3.032363	3.040234	2.935577	3.010091	3.033583
LOC400456	1562901_at	2.466942	2.43025	2.385622	2.413604	2.461343	2.398556	2.422819	2.434663	2.420924	2.406611	2.420487	2.430762	2.29705
FAM86C	1562903_at	2.039093	2.030276	2.008596	2.004646	2.015251	2.010617	4.170165	6.42281	3.99338	4.618008	2.083	3.56602	1.999229
FAM86C	1562904_s_at	2.315159	2.289418	2.270254	2.411107	2.297634	2.260329	2.275458	2.279153	2.271309	2.260684	2.273005	2.280809	2.170443
LOC340069	1562906_at	2.460433	2.438305	2.373862	2.400518	2.494225	2.420596	2.433867	2.436547	2.421835	2.417241	2.433141	2.456524	2.364427
LOC339468	1562908_at	2.70595	2.660302	2.615252	2.657246	2.682533	2.628379	2.656462	2.664355	2.649327	2.634779	2.645408	2.67897	2.514496
C1orf98	1562909_at	3.153878	3.119384	3.10165	3.120409	3.235448	3.097215	3.110177	3.118372	3.105039	3.093166	3.136198	3.11333	2.708522
SH3PXD2B	1562910_at	2.139109	2.126355	2.10729	2.111787	2.123713	2.111749	2.122716	2.128526	2.114843	2.110389	2.111078	2.118439	2.068034
FLJ25328	1562914_a_at	2.152649	2.144209	2.124186	2.13103	2.141674	2.131258	2.131032	2.221483	2.129914	2.114754	2.127859	2.135526	2.043781
LOC729062	1562916_at	4.159915	3.959885	3.871071	3.972777	4.433906	3.743898	3.972574	3.976053	4.027323	3.949062	4.073581	4.303896	3.256302
FAM45A	1562919_at	2.872719	2.783115	2.77453	2.778576	2.902924	2.778916	2.790253	2.814844	2.800597	2.760593	2.803208	2.826322	2.581085
LOC441086	1562920_at	1.882421	1.874777	1.854757	1.85828	1.871321	1.863336	1.870319	1.873472	1.863107	1.857841	1.857605	1.870151	1.850075
LOC340357	1562924_at	2.148333	2.137927	2.117958	2.128004	2.137795	2.128462	2.132355	2.128951	2.122038	2.11518	2.120675	2.123513	2.068577
LOC146443	1562930_at	2.302646	2.27615	2.256687	2.261183	2.512039	2.911069	2.239031	2.274721	2.241007	2.21417	2.258051	2.268326	2.243692
LOC729234	1562938_at	2.497626	2.450859	2.406638	2.430846	2.484189	2.429449	2.43971	2.449743	2.434385	2.430104	2.711959	2.444265	2.357405
LRRC16	1562939_at	2.270726	2.212788	2.220613	2.221787	2.23235	2.748946	2.220266	2.230762	2.22516	2.215758	2.227183	2.198593	2.121545
C4orf12	1562953_s_at	2.66328	2.650649	2.628158	2.632416	2.646168	2.635681	2.645056	2.633071	2.621623	2.628943	2.617643	2.640547	2.590122
LOC338653	1562960_at	2.635321	2.882774	2.567229	2.58421	2.623983	2.561669	2.584027	2.593462	2.580913	2.572784	2.580165	2.593047	2.528313

KIAA1217	1562966_at	2.481244	2.444118	2.428238	2.424676	2.45973	2.417452	2.431807	2.439004	2.42996	2.417803	2.426719	2.438528	2.329359
DNMBP	1562969_at	2.906786	2.844231	2.819934	2.816154	2.894668	2.82494	2.83481	2.843184	2.819782	2.78653	2.850347	2.837114	2.75385
LOC503519	1562972_at	2.410127	2.376173	2.334771	2.361217	2.397967	2.359394	2.372977	2.384772	2.368528	2.35919	2.364441	2.397117	2.275836
HBB	1562981_at	2.214543	2.203615	2.183323	2.18909	2.202779	2.188577	2.20158	2.208994	2.199213	2.187593	2.194724	2.203122	2.165669
LOC441025	1562983_at	2.551451	2.538255	2.514321	2.522094	2.542012	2.520603	2.526695	3.061441	2.524392	2.511677	2.524065	2.529581	2.352376
ZNF292	1562991_at	3.093356	2.93364	2.903634	2.998364	3.080876	2.875782	3.004574	3.014345	3.012066	3.001628	3.01874	3.063571	2.778828
ITGAX	1563003_at	2.414034	2.36833	2.325627	2.349879	2.403445	2.377229	2.360764	2.369885	2.354692	2.343898	2.347559	2.361457	2.233909
LOC730217	1563007_at	2.182167	2.158186	2.13001	2.138331	2.164657	2.138625	2.14751	2.152192	2.14103	2.131174	2.135819	2.151357	2.062324
LOC284930	1563009_at	2.608459	2.570045	2.528919	2.554458	2.582409	2.551698	2.575872	2.576857	2.563406	2.551993	2.680057	2.581179	2.4595
LOC646522	1563013_at	2.249259	2.223787	2.197332	2.204087	2.236228	2.203842	2.21242	2.223204	2.208205	2.202622	2.206564	2.217939	2.117597
RPS15	1563014_at	2.481598	2.446336	2.410955	2.428222	2.466569	2.432412	2.43551	2.445958	2.438467	2.418692	2.433066	2.445248	2.297862
TBX5	1563018_at	1.901463	1.888795	1.868395	1.873086	1.88889	1.872518	1.882985	1.886065	1.874606	1.867817	1.867476	1.880278	1.825064
LOC347475	1563022_at	3.404606	3.311412	3.18355	3.299232	3.454362	3.145923	3.304962	3.311745	3.301203	3.203224	3.313446	3.354729	2.992442
GPD1	1563034_at	3.934565	4.661995	3.644437	3.812769	3.856211	3.748427	3.84717	3.854711	3.860127	3.78122	3.851131	3.887001	3.177242
GPD1	1563035_x_at	3.359458	4.513922	3.045634	3.723442	3.182209	3.065895	3.107069	3.09844	3.116086	3.064688	3.093329	3.178704	2.713179
LOC338694	1563042_at	2.537945	2.503538	2.455674	2.500172	2.532379	2.479899	2.50594	2.515189	2.502644	2.478427	2.50213	2.563382	2.346824
LOC285375	1563043_at	2.076208	2.056819	2.036545	2.040023	2.059315	2.039414	2.046655	2.053907	2.041395	2.034017	2.038938	2.050478	1.973887
OSBP	1563051_at	4.68021	4.635346	4.711072	5.248213	6.086359	5.240618	4.468919	4.632902	4.29197	3.781333	4.777984	4.316234	3.940178
LOC729083	1563053_at	2.502342	2.481637	2.452397	2.460739	2.501826	2.464656	2.470616	2.480908	2.467281	2.453641	2.465245	2.614965	2.366637
LOC340581	1563059_at	2.237295	2.210586	2.186583	2.192872	2.220154	2.192508	2.201182	2.210476	2.197051	2.189576	2.196497	2.206646	2.106116
LOC728192	1563062_at	2.080796	2.060036	2.036837	2.040337	2.072291	2.042432	2.053374	2.060451	2.045739	2.04357	2.045739	2.054366	1.983723
LOC255654	1563074_at	1.674111	1.66409	1.64809	1.652005	1.66301	1.652615	1.66348	1.665308	1.658767	1.650549	1.649479	1.660613	1.583357
LOC285045	1563082_at	2.76878	2.72797	2.680769	2.713143	2.789181	2.703648	2.724155	2.73373	2.719287	2.700329	2.720544	2.734925	2.484454
LOC285045	1563083_s_at	3.105675	3.000773	2.906001	2.976598	3.109727	2.955583	2.978961	3.008059	2.99957	2.983431	3.017826	3.059731	2.684355
LOC284837	1563088_a_at	2.386084	2.485985	2.313275	2.339353	3.251537	4.172283	2.319749	2.329542	2.340215	2.29725	2.312783	2.317031	2.203086
CCDC33	1563090_at	2.702898	3.023715	2.678635	2.675484	2.684948	2.657555	2.701032	2.673696	2.690849	2.680889	2.666566	2.676187	2.602367
DNAH12	1563097_at	2.506359	2.469278	2.417839	2.448172	2.48833	2.431417	2.457821	2.468489	2.454111	2.44446	2.454965	2.465529	2.314411
DKFZp434B	1563098_at	3.136914	3.066479	3.061212	3.198518	3.077314	3.030497	3.068971	3.078027	3.069671	3.005768	3.085351	3.070396	2.76281
ARNTL2	1563101_at	3.002851	2.924646	2.843925	2.911313	2.985641	2.91	2.921627	2.907447	2.894894	2.900693	2.922415	2.93935	2.666014
LRRC62	1563108_at	2.776919	2.750372	2.704249	2.727333	2.75704	2.715498	2.737728	3.056463	2.733546	2.718577	2.733928	2.742812	2.519498
PIGX	1563111_a_at	3.367571	3.412466	4.194237	3.444864	3.300262	3.296291	4.309565	3.449457	4.321824	4.4347	3.432043	3.437889	5.024012
FAM120AOS	1563112_at	2.697175	2.637702	2.659729	2.640856	2.681195	2.604332	2.624018	2.642169	2.640957	2.601046	2.661759	2.65325	2.469416
UBR4	1563113_at	2.319165	2.279968	2.244199	2.267386	2.304647	2.264052	2.278813	2.288778	2.275092	2.263838	2.272783	2.290578	2.223609
LOC388456	1563117_at	1.777265	1.766316	1.748072	1.751768	1.766572	1.75156	1.762151	1.765499	1.755638	1.748786	1.75056	1.759654	1.720196
LOC651900	1563119_at	2.611747	2.591858	2.557263	2.579879	2.595194	2.575306	2.577953	2.592701	2.578729	2.553188	2.5758	2.587151	2.405965
MGAT5B	1563137_at	1.888842	1.876667	1.857481	1.860392	1.875938	1.862528	1.873029	1.872931	1.865759	1.860125	1.85725	1.866154	1.830367
C11orf64	1563145_at	2.383329	2.349168	2.309583	2.328155	2.357488	2.329545	2.33672	2.345028	2.333723	2.324241	2.335289	2.348351	2.233275
F11	1563160_at	7.501576	7.48285	6.360982	6.74233	7.677451	7.538198	3.884557	4.534268	5.090451	4.101517	3.969854	4.463356	2.15288

ZNF627	1563204_at	2.548583	2.535975	2.505265	2.515364	2.617177	2.523549	2.533408	2.534126	2.563037	2.522785	2.525765	2.542139	2.367737
C3orf65	1563207_at	3.080437	3.023806	2.900011	2.977056	3.118851	2.970165	2.990078	2.996613	3.000194	2.958532	3.026681	3.065744	2.739225
C3orf65	1563208_s_at	2.496317	2.462777	2.381842	2.43484	2.477073	2.425721	2.460413	2.462697	2.447945	2.449348	2.451378	2.45535	2.309485
MACROD2	1563209_a_at	2.982946	2.942589	2.937262	2.936754	3.030531	2.940561	2.940938	2.950912	2.941402	2.928684	2.956406	2.976167	2.911994
LOC414300	1563221_at	2.256359	2.227135	2.167356	2.201207	2.247016	2.196138	2.215464	2.221278	2.211213	2.205411	2.204861	2.222263	2.122102
CENPI	1563223_a_at	2.943271	2.868718	2.825322	2.860578	2.96003	2.820165	2.882991	2.893474	2.877307	2.857264	2.988796	2.938112	2.612557
MGC15523	1563226_at	4.223118	3.782162	3.638331	3.371651	4.354521	4.968923	3.874619	3.476067	3.30779	2.718568	3.25543	4.144966	2.715542
MGC15523	1563228_x_at	4.712787	4.16629	4.314201	3.591799	4.533318	5.270947	3.782202	4.053026	3.874686	3.131723	3.868961	3.48934	3.539514
DLEU2	1563229_at	2.887382	2.832018	2.770936	2.827491	2.871536	2.802836	2.941189	2.847136	3.415188	2.997986	2.882854	2.887876	2.794735
ERBB3	1563252_at	3.192107	3.141013	3.020727	3.134395	3.1805	3.108363	3.145594	3.150922	3.137052	3.119078	3.138782	3.209922	2.907246
ERBB3	1563253_s_at	2.808422	3.368491	2.661912	2.890171	2.918182	2.873524	3.288365	3.119981	2.89908	3.104928	2.905763	2.908053	2.811962
LOC728883	1563254_a_at	2.689085	2.623822	2.571268	2.616797	2.678593	2.600769	2.627512	2.637313	2.623586	2.602569	2.625302	2.651327	2.443339
LOC728883	1563255_at	3.242123	3.084593	3.156781	3.175609	3.208347	3.180375	3.184404	3.196382	3.180458	3.168794	3.181183	3.249034	3.075364
C9orf107	1563260_at	2.564129	2.527233	2.470845	2.504585	2.551563	2.48265	2.516168	2.522914	2.522248	2.493457	2.527912	2.527903	2.360347
PLCG2	1563263_at	2.456523	2.449538	2.428173	2.432181	2.442286	2.58331	2.444269	2.446895	2.436402	2.425742	2.429655	2.457051	2.374185
TBC1D8B	1563272_at	2.3859	2.366078	2.300145	2.346331	2.386462	2.350263	2.355287	2.364473	2.350457	2.343528	2.343422	2.358965	2.792063
DNAH3	1563290_at	2.034222	2.016845	1.997878	2.003693	2.014607	2.000107	2.015834	2.015578	2.003641	1.999752	1.995302	2.00827	1.93604
LOC387826	1563300_at	2.106959	2.160627	2.053709	2.06463	2.089404	2.167687	2.073022	2.0842	2.067985	2.060688	2.067636	2.077807	1.973582
ERICH1	1563315_s_at	5.030636	4.627033	5.721071	5.486867	5.32962	4.937012	4.975966	4.354915	5.190071	4.737694	4.224006	4.545371	3.690112
MAGIX	1563318_s_at	2.043889	2.045422	2.015003	2.023352	2.039725	2.019278	2.035991	2.037662	2.029113	2.145659	2.020006	2.019467	1.959007
MLLT10	1563321_s_at	7.560903	7.47591	6.838828	7.284963	7.43211	7.064472	7.181813	7.169459	7.163038	7.131699	7.21874	7.036823	7.039983
LOC389457	1563324_at	2.167158	2.138276	2.101735	2.120468	2.140589	2.118989	2.126333	2.133097	2.12155	2.111407	2.122377	2.13534	2.032527
CXorf31	1563327_a_at	2.318256	2.301451	2.281512	2.282912	2.333667	2.279612	2.289711	2.300934	2.28654	2.271928	2.284843	2.295845	2.178967
IRGM	1563335_at	2.738577	2.677181	2.653086	2.676447	2.738341	2.654561	2.683562	2.696487	2.683649	2.665502	2.681425	2.696466	2.483717
D21S2089E	1563346_at	1.825202	1.813944	1.792495	1.797247	1.827621	1.798891	1.808304	1.808835	1.801073	1.791808	1.793087	1.804344	1.763054
RNF216	1563361_at	2.135421	2.097449	2.035237	2.07884	2.104818	2.077379	2.08678	2.270775	2.081976	2.0759	2.081326	2.101179	2.008651
D21S2090E	1563362_at	2.599039	2.562971	2.519322	2.545221	2.58337	2.531613	2.554353	2.566004	2.549945	2.538984	2.551495	2.560223	2.382323
FLJ42957	1563369_at	2.275896	2.264485	2.23853	2.243685	2.260816	2.231601	2.255148	2.257599	2.245023	2.248696	2.242452	2.280462	2.193452
D21S2091E	1563385_at	2.308149	2.277508	2.228011	2.260151	2.290876	2.241579	2.267753	2.278389	2.264101	2.256796	2.265714	2.31091	2.125591
ATP4B	1563405_at	2.30663	2.267303	2.261357	2.264307	2.281365	2.227177	2.27661	2.278341	2.267829	2.260213	2.264298	2.295893	2.264981
ATP4B	1563407_x_at	2.187244	2.173401	2.138669	2.154295	2.175026	2.145174	2.167915	2.175607	2.162204	2.153659	2.15845	2.167219	2.073466
LOC646182	1563420_at	2.638037	2.606182	2.574994	2.587932	2.608081	2.586582	2.595218	2.602275	2.590892	2.582601	2.584727	2.600383	2.52373
LOC644613	1563426_a_at	2.574017	2.563853	2.557105	2.552219	2.612988	2.56553	2.567267	3.200147	2.555375	2.556106	2.734885	2.615268	2.504376
CALM3	1563431_x_at	8.312875	8.332711	7.621268	8.088905	8.640271	8.758236	8.397446	9.593767	8.506591	8.590187	9.318732	9.480209	7.680389
CTSLL3	1563445_x_at	4.552864	4.499934	5.687514	4.760256	4.037822	3.830942	4.88013	3.846228	4.885718	4.724684	3.650619	4.022367	3.766354
DEFB107A	1563450_at	2.657238	2.602415	2.598815	2.586976	2.663901	2.572497	2.604542	2.616098	2.603525	2.589556	2.602308	2.622438	2.435904
SOX6	1563454_at	2.656266	2.616914	2.559704	2.629222	2.651078	2.627388	3.928753	4.866331	3.032886	3.564595	3.838013	2.883424	2.614087
KIAA0999	1563455_at	5.824068	6.24562	7.328822	7.61006	5.712124	5.88672	4.880363	5.392821	4.714414	4.861059	5.763401	5.417348	3.945996

LOC285026	1563456_at	3.026722	2.931364	2.866598	2.924998	2.995153	2.88678	2.934945	2.938804	2.927983	2.909741	2.931327	2.977627	2.708189
PARVA	1563458_at	1.939858	1.930052	1.9144	1.917849	1.935404	1.921525	1.930171	1.930499	1.921511	1.919115	1.916515	1.922363	1.885139
PKD1L1	1563465_at	3.987306	3.798593	3.558437	3.778228	4.179086	3.767535	3.708661	3.660627	3.804922	3.701894	3.957528	4.04211	3.121037
MYLK	1563466_at	2.89895	2.882553	2.863627	2.865092	2.927024	2.872224	2.865519	2.862942	2.86263	2.844823	2.85934	2.884259	2.589874
KIAA1632	1563471_at	4.285958	4.14813	3.680823	3.746154	3.873344	3.578662	4.276858	3.609285	4.088341	4.472901	4.034431	3.611703	3.553882
MGC50559	1563474_at	4.179006	4.138599	4.218803	3.302729	5.130907	4.187949	3.023828	3.06688	2.99606	2.935581	3.001851	3.069565	2.643215
MGC50559	1563475_s_at	5.348776	4.725358	5.049431	5.171147	4.912052	3.896848	3.925629	3.936719	3.849721	3.955	3.283404	3.757378	3.149961
CTA-221G9	1563478_at	4.189411	4.109563	5.26644	4.649625	3.98701	3.529825	2.992472	3.037727	2.555851	3.150773	3.121655	2.924281	2.16488
FAM91A2	1563483_at	3.001362	2.938665	3.270784	3.093272	2.981211	3.100003	2.843762	2.851159	2.84551	2.819248	2.873967	2.870791	2.882007
TMEM132B	1563488_at	2.511992	2.476851	2.432141	2.459131	2.532162	2.444402	2.466985	2.479466	2.461681	2.4465	2.464654	2.474329	2.381403
LOC285638	1563489_at	2.208353	2.198662	2.177947	2.186158	2.189851	2.184929	2.182956	2.359842	2.18945	2.284256	2.176923	2.189918	2.044456
SLC9A11	1563495_at	2.297633	2.287418	2.250123	2.277542	2.299412	2.235665	2.274084	2.282184	2.283933	2.275988	2.273283	2.294003	2.167233
STL	1563496_at	3.068829	2.939121	2.937348	2.951581	3.083304	2.970038	2.95475	2.960595	2.953881	2.95728	2.999597	3.080325	2.695065
USP25	1563497_at	2.757711	2.63992	3.886654	2.875996	2.742581	2.631294	2.748998	2.649373	2.925009	2.736942	2.647997	2.731296	3.516307
SLC25A45	1563498_s_at	5.714683	5.706085	5.309943	5.301496	5.994353	6.276684	4.842218	5.458504	4.284625	4.696107	5.449121	5.484071	3.550336
ZDHHC2	1563502_at	2.113407	2.09021	2.034719	2.084354	2.109974	2.046855	2.096424	2.092121	2.092246	2.057018	2.251743	2.089906	2.084354
DUSP16	1563505_at	5.935313	5.892091	5.733284	6.140279	6.292559	5.928753	3.267657	3.435157	3.255031	3.123902	3.33759	3.475335	3.078988
DKFZp451B	1563511_at	2.118478	2.112322	2.091612	2.093129	2.11491	2.091604	2.101873	2.109542	2.096051	2.088797	2.093671	2.10269	2.029931
NOS1AP	1563512_at	3.654614	3.857352	3.539321	3.751536	3.734358	3.558592	3.031816	3.250031	2.908293	2.897197	3.21278	2.902951	2.896705
SYTL4	1563513_at	3.398851	3.675326	4.044184	3.412674	3.428836	3.441527	3.36484	2.94177	3.530352	3.437927	3.547161	3.350524	2.717478
MOBKL2B	1563521_at	3.18021	3.287506	3.002397	3.007003	3.02038	3.503495	2.972937	3.018002	3.008074	2.956739	2.984709	3.014357	2.920346
DDX10	1563522_at	2.384102	2.35293	2.319661	2.334686	2.37163	2.328521	2.342334	2.349693	2.337125	2.328616	2.338639	2.348474	2.220985
C14orf85	1563524_a_at	2.65435	2.585068	2.527696	2.56542	2.642755	2.577583	2.579382	2.592059	2.575947	2.551438	2.591563	2.615121	2.369684
LOC91149	1563528_at	2.16914	2.147793	2.11824	2.13713	2.151529	2.135691	2.13893	2.148065	2.138424	2.134321	2.132527	2.141799	2.097089
HYDIN2	1563529_at	4.249334	4.955079	3.838475	3.981662	4.181813	4.129939	4.252605	4.480065	4.17802	4.176612	4.172072	4.440508	4.239927
HMCN2	1563532_at	2.022634	2.015314	1.996447	1.995749	2.015942	2.002028	2.006957	2.00978	2.00586	2.000681	1.996909	2.007918	1.937079
GADL1	1563533_at	2.887491	2.802711	2.735388	2.796726	2.875234	2.784041	2.799593	2.80657	2.802932	2.776073	2.808213	2.844398	2.620987
SCML4	1563542_a_at	2.092955	3.508326	2.026454	2.05517	2.122421	3.445698	2.068465	2.072664	2.059667	2.050212	2.045398	2.066109	1.99772
TMEM16H	1563549_a_at	1.844389	1.834795	1.81792	1.873787	1.897245	1.832194	1.823392	1.826255	1.822365	1.792707	1.817031	1.819189	1.822482
FMN1	1563559_at	2.586326	2.554109	2.532795	2.542677	2.561536	2.53466	2.548536	2.5605	2.545379	2.535153	2.546227	2.554	2.450071
AHNAK	1563560_at	2.030279	2.021808	1.997162	1.995373	2.021303	2.01209	2.01802	2.021535	2.004596	1.989025	2.006634	2.012812	1.951815
CCDC40	1563563_at	2.287972	2.258289	2.24016	2.242022	2.277536	2.243877	2.249359	2.261271	2.245868	2.232763	2.244037	2.255407	2.191434
INPP5B	1563565_at	2.668028	2.602345	2.570612	2.62911	2.660389	2.633731	2.653651	2.699893	2.623748	2.590529	2.625461	2.696626	2.47091
LOC285463	1563571_at	2.790421	2.719312	2.642447	2.669728	2.719041	2.697129	2.71405	2.717392	2.703715	2.697518	2.711526	2.767157	2.470524
LOC152274	1563572_at	2.295695	2.267743	2.240475	2.248097	2.274809	2.245848	2.256085	2.265049	2.251949	2.242555	2.247878	2.260177	2.166937
FLJ37673	1563581_at	3.187979	3.249609	2.933126	3.039746	3.134604	2.935131	3.052798	3.063431	3.051468	2.990563	3.102245	3.18442	2.687444
C18orf30	1563584_at	1.975556	1.962251	1.94381	1.948408	1.962604	1.946747	1.956437	1.959435	1.948148	1.945421	1.949679	1.952998	1.919505
LOC401324	1563586_at	2.443325	2.411239	2.3786	2.395501	2.427152	2.400036	2.405951	2.408787	2.396346	2.392431	2.395306	2.403051	2.28926

CDH4	1563587_at	2.016972	2.001746	1.975687	1.98379	2.001613	1.985179	1.996315	1.994931	1.986012	1.978957	1.979416	1.993064	1.922407
LOC340184	1563589_at	3.201914	3.072107	3.08506	3.122449	3.164753	3.100006	3.087197	3.143752	3.127029	3.050738	3.149343	3.141244	2.743284
SRGAP3	1563595_at	2.609145	2.581962	2.56066	2.564411	2.621474	2.553508	2.569028	2.573298	2.565184	2.542056	2.561391	2.579764	2.718394
LOC286359	1563606_a_at	3.203439	3.135108	3.063169	3.121486	3.25967	3.082297	3.13985	3.155862	3.136163	3.083618	3.163349	3.166747	2.764631
LOC286359	1563607_x_at	3.093381	3.079117	2.965384	3.021339	3.105471	3.064519	3.078977	3.079563	3.067993	3.065295	3.06439	3.082258	2.667287
KCNT1	1563608_a_at	2.007248	1.998199	1.960092	1.965491	1.983142	1.96514	1.97509	1.979534	1.966673	1.960603	1.965259	1.975413	1.908293
LOC157273	1563610_at	2.946391	2.777328	5.382488	4.401483	5.633041	6.261158	2.754204	2.745722	2.732538	2.724247	2.765979	2.802742	2.599427
MTBP	1563614_at	2.397268	2.363494	2.330344	2.347458	2.380443	2.34054	2.356445	2.364621	2.352119	2.346542	2.353926	2.388046	2.230516
BTRC	1563620_at	2.666924	2.610963	2.581599	2.60411	2.661032	2.601277	2.613865	2.623935	2.60958	2.589692	2.608316	2.631723	2.433511
LOC283874	1563629_a_at	4.820583	4.801568	4.984213	4.782094	5.408317	4.841693	4.486469	4.184746	5.481393	5.73708	4.206567	4.38602	4.603169
LOC220980	1563632_at	2.166556	2.164101	2.134077	2.144542	2.166726	2.154993	2.155271	2.161891	2.147161	2.139786	2.142635	2.152042	2.080701
LOC729652	1563637_at	2.981008	2.903176	2.869859	2.86229	2.933833	2.870762	2.900511	2.878865	2.897739	2.841794	2.90633	2.914327	2.589109
FAM18A	1563638_at	2.323123	2.315447	2.266434	2.301586	2.312342	2.271038	2.313734	2.314109	2.301904	2.281944	2.298849	2.330061	3.570929
FHAD1	1563639_a_at	1.690024	1.683382	1.663687	1.667526	1.68257	1.662602	1.686161	1.671115	1.673842	1.664666	1.652773	1.660801	1.636845
SLIC1	1563641_a_at	2.450541	2.421637	2.390654	2.399652	2.421682	2.411994	2.412476	2.422197	2.408824	2.399205	2.408291	2.413408	2.227945
TMEM67	1563646_a_at	3.557882	3.51493	3.481243	3.502817	3.531935	3.688575	3.520111	3.523702	3.863946	3.384093	3.505916	3.520274	4.984268
C8orf66	1563654_at	2.237713	2.230439	2.196659	2.210417	2.238642	2.205002	2.228496	2.223852	2.223964	2.205485	2.213077	2.231714	2.221305
TNNT2	1563655_at	2.120632	2.098873	2.074583	2.095408	2.146969	2.08511	2.091697	2.099937	2.087997	2.141278	2.086842	2.096638	2.090526
PLXND1	1563657_at	1.856672	1.856137	1.818674	1.838815	1.850249	1.877669	1.852337	1.940763	1.846145	1.840047	1.837303	1.84868	1.800281
SYT9	1563658_a_at	2.335283	2.311127	3.389888	2.312916	2.715472	2.905439	2.303096	2.321279	2.301329	2.28703	2.296564	2.312867	2.283511
HERC6	1563659_at	2.263014	2.235832	2.209963	2.216163	2.266771	2.225291	2.224068	2.235127	2.219893	2.210516	2.220482	2.229363	2.142676
LOC285778	1563662_at	2.409687	2.384923	2.362309	2.366152	2.392049	2.36738	2.374124	2.382258	2.369885	2.361591	2.36628	2.375967	2.243723
MORN1	1563668_at	1.806944	1.77944	1.759198	1.76102	1.776062	1.766891	1.775044	1.772284	1.769901	1.758099	1.756856	1.765466	1.723727
ZSCAN23	1563670_at	2.240495	2.198793	2.17575	2.186344	2.228312	2.184993	2.196619	2.204041	2.190286	2.174402	2.187109	2.203394	2.120102
LACE1	1563671_at	2.598201	2.562564	2.507161	2.544244	2.600202	2.543304	2.550246	2.560753	2.547206	2.52675	2.548257	2.560094	2.385267
ALS2CR11	1563673_a_at	3.1187	3.03771	2.973019	3.022995	3.156112	3.037997	3.030629	3.059046	3.044896	3.00213	3.051123	3.07931	2.820365
FCRL2	1563674_at	2.862405	2.816771	2.768873	2.803847	2.850066	3.025695	2.824286	2.822516	2.815578	2.810588	2.805794	2.814713	2.726603
LOC150577	1563679_at	2.568741	2.506809	2.489549	2.488212	2.522431	2.491528	2.495208	2.5056	2.49184	2.47919	2.490555	2.499062	2.369897
LOC284950	1563680_at	2.883983	2.811264	2.754907	2.809818	2.871416	2.786608	2.817396	2.832525	2.817489	2.790615	2.833458	2.871692	2.604691
LOC285422	1563685_at	2.680985	2.640873	2.575215	2.618702	2.692554	2.60916	2.634138	2.645909	2.630796	2.605384	2.63142	2.643814	2.446371
FRYL	1563687_a_at	2.693538	2.675504	2.639213	2.657303	2.693777	2.67551	3.486013	2.671031	2.67975	2.787787	2.655262	2.676304	3.064809
PPP4R1	1563690_at	3.386283	3.271448	3.140375	3.263295	3.445949	3.194067	3.24968	3.286921	3.273647	3.222758	3.310551	3.336185	2.852461
LOC285300	1563691_at	2.047218	2.036788	2.018755	2.022098	2.037384	2.016953	2.028239	2.034488	2.020925	2.020273	2.019374	2.030687	1.955083
HSD17B4	1563696_at	2.689529	2.628923	2.598141	2.618673	2.673972	2.621	2.62526	2.637121	2.62819	2.601386	2.623093	2.648119	2.442504
SFXN5	1563708_at	5.756374	5.876438	4.218429	4.403067	6.061368	6.034015	4.063492	4.397455	3.976408	4.245519	4.305326	3.916122	2.757645
ZNF583	1563725_at	2.322909	2.294412	2.273656	2.273927	2.368614	2.274942	2.286175	2.289849	2.278636	2.267507	2.279129	2.288225	2.172682
C9orf14	1563728_at	2.288623	2.26822	2.240133	2.249355	2.271247	2.252409	2.262757	2.264476	2.253192	2.24404	2.250019	2.261291	2.148134
LOC643650	1563742_at	2.088252	2.067901	2.046228	2.050162	2.071349	2.048912	2.06104	2.064071	2.052835	2.049505	2.04788	2.059768	1.997706

C1orf180	1563743_at	2.15831	2.141267	2.115866	2.121868	2.141498	2.119093	2.130414	2.138649	2.123927	2.122316	2.122224	2.134721	2.048313
DISC1	1563744_a_at	1.875544	1.86475	1.843471	1.850787	1.86453	1.84868	1.860931	1.861939	1.8498	1.843977	1.844103	1.855492	1.809557
LOC283050	1563745_a_at	2.774878	2.756711	2.715057	2.718949	2.799595	2.741245	2.731139	2.756079	2.757291	2.742887	2.753456	2.816096	2.669477
FLJ43944	1563750_at	2.709516	2.662727	2.614819	2.642919	2.696525	2.632048	2.659108	2.671384	2.658039	2.639546	2.658773	2.715571	2.462286
LOC149684	1563753_at	2.175039	2.157385	2.131671	2.138904	2.165016	2.138383	2.149969	2.155116	2.142964	2.133833	2.139495	2.149533	2.077425
GRIK2	1563754_at	3.170661	3.099663	3.089769	3.108775	3.199759	3.106697	3.113928	3.125689	3.109754	3.076851	3.113727	3.121696	3.239135
RP1-27O5.1	1563770_at	2.352861	2.312161	2.269762	2.29222	2.323301	2.83437	2.299389	2.311926	2.295819	2.291091	2.296692	2.307968	2.263269
LAMA3	1563772_a_at	2.749034	2.863284	2.704838	2.711221	2.756519	3.06328	2.718449	2.735492	2.718869	2.700826	2.707687	2.729442	2.507047
LOC285949	1563781_at	5.111229	5.407784	4.247715	4.51051	4.491437	4.847262	4.497076	4.670333	4.316381	4.312237	4.307447	4.497059	3.819877
ZNF333	1563783_a_at	2.475195	2.465467	2.442699	2.44629	2.486309	2.447557	2.455959	2.452257	2.46079	2.451603	2.451071	2.458503	2.610648
CAGE1	1563787_a_at	3.550087	3.320967	3.28243	3.36891	3.661813	3.263419	3.349185	3.333597	3.404606	3.33313	3.58559	3.641436	3.081556
AMN	1563792_at	5.729327	5.457774	3.632325	4.269074	4.817001	4.387129	6.135674	6.304122	5.871198	6.26582	6.018292	6.203557	2.262072
IFT122	1563794_s_at	3.786323	3.897206	3.352849	3.481656	3.497599	3.705939	3.583427	3.756211	3.533513	3.460386	3.717621	3.50687	3.14588
EARS2	1563796_s_at	4.900772	4.669497	4.746522	5.013998	4.904746	5.555883	4.919758	4.909405	5.210916	5.085822	5.723148	5.661188	4.220301
LOC283140	1563800_at	3.648228	3.603296	3.68829	3.587179	3.645511	3.767694	3.603995	3.908501	3.541299	3.587142	3.599003	3.608902	3.475407
LOC284551	1563802_at	2.654184	2.620983	2.591629	2.60108	2.676241	2.600618	2.607348	2.614566	2.599019	2.57652	2.589725	2.608406	2.426252
FAM83C	1563805_a_at	3.642533	3.625392	3.595609	3.578006	3.830867	3.449984	3.617851	3.566594	3.609638	3.601649	3.622639	3.757727	3.090421
MCF2L	1563808_at	1.924825	1.91823	1.839117	1.902862	1.951333	1.87941	1.908708	1.901379	1.904667	1.901449	1.901145	1.960858	1.779379
MCF2L	1563809_a_at	2.319089	2.292878	2.279877	2.276793	2.295334	2.278603	2.289451	2.28679	2.292289	2.279051	2.274941	2.279828	2.301073
C2orf50	1563814_at	2.291586	2.272503	2.250125	2.249352	2.279609	2.251727	2.263072	2.269158	2.255726	2.244573	2.254998	2.262825	2.205858
GAB4	1563816_at	2.018339	2.001835	1.975905	1.979761	2.013196	1.984971	1.994537	1.999238	1.985535	1.972743	1.982351	1.993167	1.920314
LOC170425	1563821_at	2.444245	2.407243	2.375858	2.396361	2.429235	2.385539	2.407738	2.414943	2.406191	2.386535	2.402888	2.421772	2.306022
LOC158434	1563827_at	2.700448	2.662927	2.600994	2.64321	2.801384	2.617385	2.644641	2.654414	2.64664	2.635474	2.642281	2.666381	2.455849
FHAD1	1563830_a_at	2.24772	2.228134	2.192133	2.199124	2.230847	2.397061	2.214079	2.225058	2.209598	2.191059	2.211599	2.237736	2.117257
C1orf62	1563834_a_at	3.404858	3.227608	3.131602	3.219418	3.452073	3.21306	3.237148	3.277438	3.261485	3.288248	3.293268	3.388122	3.144136
TBC1D7	1563839_at	2.319361	2.292267	2.27087	2.274611	2.302824	2.272971	2.284018	2.289183	2.275029	2.264039	2.273838	2.284071	2.202745
PIGG	1563842_at	3.116989	2.991101	2.974464	2.977525	3.121962	2.979624	2.98315	2.983106	2.976253	2.965976	2.970252	2.982706	2.876587
LOC202134	1563845_at	2.874016	2.860103	2.777493	2.797022	2.879935	2.829024	3.380296	3.647897	2.939678	3.103375	3.049554	3.831111	2.572099
SH2D4B	1563849_at	2.213222	2.19084	2.161885	2.171458	2.196585	2.168799	2.180917	2.190734	2.174792	2.163773	2.175784	2.183893	2.074955
LOC283045	1563853_at	1.806803	1.798889	1.780162	1.784036	1.795984	1.782703	1.796458	1.790266	1.78663	1.781728	1.773795	1.789917	1.753224
LOC283045	1563854_s_at	2.764903	2.746714	2.763816	2.720549	2.792861	2.730543	2.734206	2.725429	2.729202	2.707378	2.728859	2.7395	2.505497
INSAF	1563856_at	2.177711	2.155191	2.133391	2.139897	2.16065	2.138398	2.14986	2.156053	2.142324	2.13498	2.14119	2.150155	2.072989
LOC400965	1563860_at	2.584878	2.559585	2.520526	2.54284	2.596998	2.544173	2.560606	2.558662	2.541039	2.535883	2.548354	2.562186	2.352159
MGC17403	1563862_at	2.545089	2.500039	2.434992	2.478717	2.549939	2.478221	2.490735	2.492542	2.490284	2.470263	2.48076	2.500124	2.376867
MGC17403	1563863_x_at	2.521275	2.491253	2.442009	2.479492	2.523964	2.570891	2.491798	2.500743	2.487998	2.477247	2.510527	2.495637	2.336584
LOC283194	1563867_at	1.996259	1.981472	1.958835	1.965997	1.981391	1.970762	1.976066	1.981685	1.967835	1.964115	1.961091	1.977485	1.907125
C9orf79	1563868_a_at	2.503856	2.469036	2.422059	2.452403	2.487059	2.520789	2.462427	2.47278	2.463415	2.442529	2.455931	2.500196	2.372698
LOC284395	1563872_at	2.091868	2.082549	2.065741	2.068808	2.079343	2.071528	2.073619	2.081864	2.066431	2.067968	2.06446	2.073988	1.995419

WDR72	1563874_at	2.192263	2.163587	2.217991	2.170247	2.245209	2.544507	2.175187	2.186502	2.165837	2.162501	2.157526	2.175636	2.088293
FLJ35530	1563876_at	2.854998	2.802718	2.754717	2.744316	2.84148	2.850784	2.801322	2.810216	2.798356	2.779424	2.794551	2.881922	2.699479
LOC338963	1563878_a_at	2.243791	2.224968	2.197126	2.20041	2.227	2.211461	2.215175	2.203971	2.209049	2.200789	2.217729	2.208142	1.950834
RAPGEF5	1563882_a_at	1.815879	1.805131	1.78612	1.788446	1.80448	1.788457	1.800508	1.802092	1.78972	1.787433	1.788192	1.796888	1.748825
FLJ25037	1563887_at	2.582646	2.548991	2.514479	2.529635	2.62393	2.531433	2.552231	2.542807	2.55374	2.51542	2.542126	2.590373	2.453823
LOC730015	1563894_at	2.025244	2.008521	1.987921	1.989973	2.02046	1.997701	2.001137	2.000925	1.99322	1.979574	1.991898	2.000948	1.931127
CRABP1	1563897_at	1.794603	1.782147	1.764666	1.768697	1.781208	1.771173	1.779661	1.778025	1.772055	1.765315	1.761542	1.772844	1.727565
TM7SF3	1563898_at	2.518939	2.478275	2.444508	2.46931	2.481593	2.443246	2.470636	2.478851	2.462021	2.45328	2.462145	2.502651	2.324245
LCTL	1563899_at	2.912725	2.838936	2.766318	2.829711	2.912273	2.800838	2.839728	2.851457	2.840072	2.808872	2.844063	2.870311	2.658082
FAM83B	1563900_at	2.64867	2.616747	2.600866	2.597687	2.631568	2.590153	2.605351	2.594693	2.600873	2.58765	2.60099	2.611258	2.454639
SOBP	1563906_at	2.162756	3.094399	2.087141	2.136885	3.13552	3.118847	3.275683	3.275891	3.095484	3.081895	3.745151	3.423234	3.834768
GARNL3	1563907_at	2.317378	2.293038	2.263558	2.273475	2.305225	2.274337	2.284881	2.292427	2.276728	2.272274	2.278933	2.292102	2.198472
LOC283089	1563916_at	2.225039	2.201578	2.174438	2.181429	2.203234	2.180564	2.193312	2.19859	2.184677	2.176881	2.180917	2.193213	2.115313
CCDC123	1563919_a_at	2.157969	2.156448	2.135896	2.136534	2.153485	2.135458	2.145782	2.154677	2.264039	2.132401	2.140513	2.149353	2.13523
FAM45A	1563920_at	2.986164	2.905975	2.794517	2.891291	3.048826	2.893993	2.870679	2.905338	2.889128	2.881604	2.891269	2.988972	2.608115
LOC401463	1563927_a_at	2.344224	2.309144	2.275362	2.286255	2.311917	2.278021	2.29652	2.307095	2.293062	2.284171	2.289955	2.301638	2.199897
PLD5	1563933_a_at	2.141431	2.115374	2.094245	2.098457	2.121174	2.097429	2.109431	2.1116	2.09969	2.093314	2.097101	2.117101	4.203491
PPARGC1B	1563943_at	2.25666	2.726487	2.236244	2.238636	2.25887	2.310092	2.245321	2.28912	2.241834	2.245003	2.251931	2.251206	2.18383
LOC284100	1563945_at	2.035185	2.025122	1.983617	2.006153	2.018558	1.992158	2.019774	2.026076	2.011026	2.005591	2.012441	2.021806	1.937804
ERC1	1563947_a_at	3.074617	2.838854	2.994966	3.246284	4.231108	4.120248	2.757231	3.244653	2.840779	2.486523	3.031668	3.387146	3.516309
FHAD1	1563961_at	2.637656	2.595645	2.532859	2.577549	2.625914	2.804924	2.585417	2.598694	2.582152	2.566531	2.58227	2.622899	2.412331
FLJ33360	1563969_at	2.518917	2.303819	2.273937	2.292603	2.325647	2.709157	2.309523	2.3129	2.285643	2.278463	2.298208	2.313178	2.143383
MRPS27	1563970_at	2.374019	2.3428	2.303697	2.323903	2.358198	2.321142	2.331606	2.342268	2.328447	2.317372	2.329302	2.401674	2.212958
RNF130	1563975_at	3.741093	3.612767	3.450003	3.576644	3.839592	3.586941	3.534624	3.533103	3.513882	3.511123	3.666903	3.717744	3.017156
LOC339457	1563978_at	2.622016	2.567783	2.555551	2.559851	2.610518	2.532465	2.573161	2.582799	2.568832	2.553225	2.567888	2.595617	2.402316
TSGA14	1563997_at	1.917458	1.902999	1.884307	1.888168	1.903882	1.886733	1.898468	1.900403	1.888972	1.882945	1.884738	1.892831	1.860679
ANKRD31	1564000_at	2.999931	2.887868	2.833581	2.8848	2.973869	2.94621	2.889803	2.900891	2.886878	2.86673	2.893208	2.911425	2.583271
C6orf199	1564002_a_at	3.241921	3.226758	3.632437	3.209973	3.243496	3.226805	3.21659	3.16126	3.213735	3.539376	3.214224	3.220168	4.420091
COL27A1	1564008_at	2.288421	2.265235	2.224616	2.247543	2.275983	2.339893	2.253169	2.260705	2.249004	2.242871	2.249022	2.253083	2.193272
CAST	1564010_at	2.098073	2.043799	2.024882	2.031678	2.044706	2.032745	2.040272	2.038545	2.038235	2.015564	2.02411	2.034246	1.975714
C18orf58	1564015_at	2.443935	2.408992	2.365048	2.393034	2.440094	2.387377	2.400075	2.411393	2.396983	2.38284	2.397315	2.41597	2.291997
C21orf123	1564017_at	2.250385	2.224803	2.198206	2.207543	2.237072	2.202234	2.215812	2.222714	2.208329	2.202508	2.207768	2.21859	2.119
ZNF804B	1564022_at	2.179107	2.155377	2.136621	2.138253	2.161279	2.136326	2.14651	2.154378	2.141177	2.133734	2.137996	2.150061	2.059976
FAM139A	1564027_a_at	2.870706	2.881891	2.772494	2.828318	2.881787	2.841231	2.853091	2.846753	2.8608	2.878073	3.07769	2.931722	2.807646
FAM139A	1564028_s_at	2.562443	2.529139	2.485365	2.54942	2.57147	2.526409	2.517182	2.528876	2.518661	2.494116	2.489494	2.522431	2.408732
USP49	1564029_at	2.619535	2.594978	2.562301	2.563986	2.61565	2.572859	2.584196	2.591622	2.578488	2.570436	2.576289	2.615735	2.48851
RELL2	1564031_a_at	2.172962	2.154532	2.12912	2.115333	2.158208	2.135515	2.515821	2.179079	2.138347	2.106333	2.139527	2.178784	2.057357
ZSCAN23	1564039_at	2.926333	2.855655	2.835436	2.83909	2.860083	2.837711	2.8393	2.83133	2.8443	2.802943	2.845334	2.849037	2.587769

LOC283999	1564040_a_at	2.22707	2.203925	2.180842	2.18626	2.209619	2.179264	2.197819	2.225686	2.191575	2.184244	2.190952	2.201224	2.123843
KNCN	1564044_at	2.241664	2.23218	2.200362	2.211145	2.245452	2.208551	2.22715	2.230805	2.216488	2.210105	2.215769	2.223693	2.164597
LOC339593	1564049_at	2.400806	2.368853	2.334845	2.350336	2.402208	2.344445	2.360324	2.370093	2.354188	2.333169	2.356426	2.378602	2.172622
LOC642808	1564050_at	1.927833	1.915094	1.894804	1.898775	1.91428	1.904544	1.909892	1.911945	1.901292	1.89388	1.898568	1.906973	1.851448
TREML4	1564052_at	2.001921	1.992349	1.965526	1.970875	2.178345	1.977391	1.981647	1.983973	1.973486	1.965689	1.96947	1.978266	1.917124
YTHDF3	1564053_a_at	2.653188	2.674025	4.27686	3.479983	2.008341	2.080871	3.734146	2.315518	3.803092	3.781326	2.092395	2.207814	6.407625
LOC144742	1564060_at	2.27464	2.252975	2.216993	2.233838	2.262873	2.225268	2.241206	2.250229	2.237194	2.227269	2.235652	2.24655	2.139238
ATP11B	1564063_a_at	3.363487	3.323962	3.160791	3.211007	3.029284	3.165673	3.419072	3.950419	3.590067	3.830582	4.014352	4.097303	3.05765
ATP11B	1564064_a_at	3.145791	2.934766	3.587548	3.409788	2.678475	3.112065	4.542051	3.26715	4.12663	4.538256	2.660285	2.856918	3.540917
C6orf137	1564066_at	3.587414	3.899865	3.285878	3.410307	3.585485	3.325211	3.39418	3.400408	3.451064	3.377571	3.501113	3.563699	2.89284
C6orf137	1564067_x_at	3.482698	3.421983	3.246108	3.405258	3.678283	3.399495	3.350451	3.372673	3.378568	3.324053	3.402803	3.457188	2.913985
MYH16	1564072_at	2.676481	2.631364	2.596458	2.618484	2.660621	2.597544	2.632301	2.645841	2.683113	2.614757	2.635537	2.640646	2.472295
FLJ35848	1564075_a_at	2.191312	2.172825	2.120244	2.153485	2.198244	2.149812	2.162682	2.171081	2.155409	2.148604	2.155496	2.190732	2.072895
CAD	1564084_at	2.338372	2.307205	2.28136	2.295659	2.323171	2.295815	2.302367	2.310719	2.297927	2.289563	2.29311	2.306097	2.207983
NEK1	1564093_at	3.717993	3.68222	3.433376	4.033753	3.749385	3.651097	3.616935	5.004489	3.830783	3.664371	4.746162	4.883463	3.257128
LOC284865	1564109_at	2.143682	2.119032	2.096072	2.101102	2.117238	2.100949	2.113559	2.116345	2.103779	2.097147	2.101416	2.108453	2.069968
FAM71A	1564112_at	2.017997	2.010611	1.990216	1.995129	2.008356	1.996026	2.005236	2.012705	1.997073	1.990864	1.988294	1.988915	1.961683
HMCN2	1564117_at	2.104214	2.098392	2.069061	2.080246	2.097413	2.081291	2.093121	2.095731	2.083475	2.080818	2.079083	2.088489	2.037012
LOC283875	1564122_at	2.578207	2.539977	2.517836	2.52237	2.554668	2.500759	2.529314	2.540503	2.522053	2.508319	2.523235	2.539865	2.37112
LOC285857	1564125_at	2.938455	2.867577	2.805192	2.877283	3.099771	2.889352	2.851468	2.897302	2.881939	2.8747	2.882939	2.910103	2.595773
FBXW12	1564138_at	2.132307	2.11738	2.091033	2.097137	2.120453	2.106306	2.117537	2.11694	2.107758	2.10395	2.099745	2.116064	2.065707
LOC144571	1564139_at	4.451377	5.1129	3.845982	4.14315	4.676643	5.064807	3.861737	4.195773	3.944656	3.91498	4.154737	4.190017	4.301558
LOC256021	1564150_a_at	2.268692	2.239999	2.215984	2.224163	2.251096	2.223741	2.230334	2.238298	2.226596	2.219189	2.221426	2.234421	2.13052
FLJ35816	1564152_at	2.113425	2.093812	2.067787	2.074561	2.108258	2.072749	2.08397	2.092117	2.077811	2.069308	2.075928	2.085999	2.001963
FLJ33544	1564157_at	2.332133	2.312179	2.293657	2.297874	2.335098	2.370001	2.302865	2.312045	2.297982	2.293377	2.295849	2.299863	2.186913
FLJ16686	1564160_at	2.455503	2.420352	2.394825	2.400487	2.441711	2.4012	2.408606	2.413632	2.404719	2.399301	2.430086	2.418284	2.275456
C1orf218	1564164_at	5.6453	5.80184	5.523535	5.42812	5.283155	4.309784	4.803079	5.209517	5.199577	5.278108	4.334425	4.763473	5.161084
PRKRIP1	1564165_at	4.653687	4.479814	4.360264	4.449032	5.012077	4.188097	4.421436	4.963492	4.465201	4.480601	4.686446	4.780204	4.11958
PRKRIP1	1564166_s_at	2.994964	3.037232	2.791576	2.869422	3.208265	2.942492	2.936644	2.948334	2.92471	2.914167	2.968916	3.044068	2.93301
LOC93444	1564168_a_at	2.260838	2.22846	2.205462	2.211919	2.226935	2.21068	2.215447	2.224328	2.211572	2.20409	2.206293	2.222361	2.155298
LOC401074	1564175_at	2.526932	2.487323	2.445969	2.470212	2.517958	2.457382	2.480686	2.496346	2.480901	2.470096	2.483337	2.493516	2.399674
LOC283033	1564178_at	2.079272	2.05875	2.035693	2.042342	2.062687	2.041818	2.054013	2.058262	2.044563	2.037665	2.043103	2.052922	1.978654
ZNF519	1564190_x_at	3.04108	3.234032	2.892849	2.922698	2.942308	3.295345	3.177545	3.843196	2.934791	2.91918	2.953672	4.305316	4.008362
FLJ39061	1564193_at	2.082101	2.054624	2.029236	2.036195	2.055634	2.04411	2.045601	2.055062	2.03981	2.031913	2.038899	2.063746	1.944948
MGC35295	1564194_a_at	2.253316	2.23363	2.175	2.219681	2.253274	2.216142	2.224271	2.231528	2.227068	2.212518	2.217213	2.226045	2.179106
C10orf90	1564198_a_at	2.927157	2.873514	2.826859	2.862319	2.897849	2.841566	2.867801	2.878514	2.863769	2.848506	2.865216	2.87716	2.686158
LOC646324	1564200_at	1.988856	1.958921	1.916779	1.947651	1.972303	1.943941	1.946097	1.95661	1.945437	1.939517	1.940214	1.950244	1.844184
LOC147004	1564203_at	1.983851	1.968787	1.906469	1.945318	1.968488	2.75628	1.967226	1.965744	1.954661	1.954776	1.95012	1.959484	1.867789

FAM91A2	1564204_at	3.602006	3.368586	3.413965	3.41593	3.720131	3.327416	3.365055	3.376447	3.405755	3.340633	3.405645	3.405183	3.178754
FLJ35390	1564207_at	5.582281	5.999407	5.368709	5.388312	6.199646	6.179469	5.21901	5.377081	3.807019	5.185999	4.913376	5.396494	4.880618
FLJ35390	1564208_x_at	3.126988	3.318194	3.030561	3.080768	3.425982	3.64452	3.020363	3.103174	2.699693	3.033891	3.110874	3.126104	2.777546
LOC282980	1564209_at	2.167754	2.148793	2.124348	2.130569	2.151762	2.129085	2.14085	2.147541	2.133031	2.125875	2.131453	2.142733	2.066109
C14orf64	1564211_at	3.015212	2.896782	2.868897	2.919108	3.017976	3.019763	2.899746	2.875014	2.92557	2.903205	3.055723	2.915205	2.643001
UNQ2963	1564224_x_at	3.645763	4.525774	3.545959	3.707375	4.676765	4.448295	3.316889	3.357108	3.51644	3.060835	3.521097	3.548238	3.26976
LOC729173	1564229_at	1.789008	1.780345	1.762824	1.766288	1.778648	1.768764	1.773664	1.784053	1.767531	1.760387	1.763973	1.770449	1.728576
IFT80	1564231_at	1.955645	1.942514	1.92071	1.92407	1.941959	1.924136	1.936637	1.940018	1.928696	1.921969	1.92113	1.933882	1.883304
LOC441528	1564232_at	2.250318	2.236297	2.211427	2.220407	2.245632	2.21533	2.230762	2.234424	2.219852	2.217729	2.219489	2.211047	2.217394
FLJ33534	1564233_at	2.131889	2.110584	2.07834	2.092255	2.118634	2.102798	2.102959	2.108576	2.096714	2.086721	2.094057	2.103346	2.019034
hCG_19948	1564235_at	2.196607	2.165641	2.142416	2.146105	2.173963	2.146115	2.154531	2.162344	2.147278	2.141291	2.142545	2.155825	2.065033
WDR49	1564238_a_at	2.258912	2.226556	2.194942	2.215499	2.231869	2.20871	2.214947	2.226683	2.210292	2.212832	2.210906	2.230304	2.117759
ATP1A4	1564241_at	2.469418	2.430752	2.382705	2.410484	2.447744	2.395358	2.418854	2.428691	2.415885	2.40374	2.412054	2.425433	2.285732
C3orf53	1564250_at	2.729701	2.678054	2.61185	2.65817	2.718736	2.63503	2.669264	2.678872	2.667318	2.644693	2.665376	2.6945	2.4741
EMID1	1564251_at	1.958124	1.950388	1.930247	1.936548	1.948475	1.936678	1.948041	1.948072	1.937032	1.935555	1.93058	1.940788	1.907567
LOC285766	1564253_at	2.299401	2.279944	2.241602	2.263221	2.282664	2.260107	2.27422	2.27875	2.265003	2.258035	2.262929	2.274471	2.188136
KY	1564254_at	2.812367	2.805726	2.778876	2.783148	2.816214	2.79415	2.802326	2.795658	2.785925	2.782447	2.790613	2.795422	2.708619
LOC144817	1564263_at	2.348588	2.422107	2.297454	2.31592	2.351758	2.320817	2.318292	2.312513	2.322812	2.311235	2.315625	2.323538	2.208291
KLHDC1	1564272_a_at	3.654745	3.435982	3.394852	3.474773	3.721723	3.378287	3.440258	3.476023	3.498284	3.446656	3.552488	3.636003	3.052873
KLHDC1	1564273_at	2.660855	2.633292	2.566397	2.608996	2.665198	2.614121	2.62199	2.631321	2.619752	2.607298	2.619458	2.654989	2.54963
C9orf47	1564274_at	1.803793	1.793863	1.775511	1.78226	1.793147	1.779175	1.788185	1.789471	1.78088	1.775756	1.779211	1.784626	1.780973
LOC441108	1564276_at	3.395254	3.350782	3.028502	3.226006	3.45877	3.066038	3.107988	3.109354	3.126062	3.080243	3.193581	3.244986	2.782859
LOC286297	1564277_a_at	3.41991	3.453418	3.373564	3.364374	3.470089	4.158797	3.323277	3.393676	3.402498	3.349507	3.421488	3.446419	2.561617
LOC286297	1564280_x_at	3.254311	3.180018	3.151753	3.125776	3.247846	3.365119	3.169248	3.177089	3.163621	3.133319	3.165592	3.204219	2.763838
LOC285708	1564281_at	0.961564	0.947616	0.929191	0.928781	0.939214	0.931697	0.941995	0.933022	0.943346	0.928046	0.922483	0.932823	0.919337
LOC285708	1564282_a_at	2.513201	2.48017	2.459908	2.464813	2.539401	2.462684	2.477121	2.482787	2.4724	2.468487	2.474257	2.52157	2.381829
HFM1	1564285_at	2.658883	2.604523	2.572336	2.597264	2.64866	2.579888	2.60695	2.616041	2.603383	2.587055	2.600273	2.615244	2.431092
LOC144776	1564287_at	2.651692	2.610052	2.589334	2.590834	2.62764	2.588477	2.604497	2.595496	2.592699	2.572335	2.588974	2.633829	2.440794
FLJ25917	1564295_at	4.025351	3.989601	3.924403	3.981171	4.064908	3.864729	3.995637	4.005597	4.012605	3.996643	4.132862	4.13775	3.572535
LOC389124	1564300_at	2.069625	2.057357	2.038022	2.041613	2.05609	2.040948	2.134684	2.059934	2.044511	2.037513	2.042455	2.054449	1.98156
RPAIN	1564301_a_at	2.551236	2.533103	2.478925	2.51615	2.557594	2.515978	2.527803	2.531831	2.5185	2.511194	2.514704	2.527261	2.46692
A2ML1	1564307_a_at	2.765976	2.755598	2.675808	2.71088	2.767448	2.799368	2.726832	2.732193	2.727559	2.700279	2.718907	2.783406	2.509077
MPP7	1564308_a_at	2.657919	2.625185	2.577883	2.615395	2.638128	2.593113	2.626445	2.617851	2.619546	2.601594	2.604062	2.685069	2.467818
LOC285045	1564309_at	2.352075	2.325011	2.285789	2.305518	2.334468	2.296663	2.314019	2.32394	2.310915	2.303109	2.307183	2.321007	2.213519
PARP15	1564310_a_at	2.2235	2.203812	2.185175	2.185302	2.206829	2.18465	2.192056	2.201321	2.187701	2.180245	2.184612	2.197185	2.113903
LOC219690	1564314_at	2.262541	2.241987	2.216555	2.223227	2.245827	2.222562	2.230339	2.240828	2.224344	2.217619	2.226969	2.235689	2.129503
C8orf49	1564315_at	3.162792	2.71269	2.917562	3.024128	2.710877	2.523646	2.590477	2.596959	2.699215	2.736298	2.67847	2.674958	2.090314
POLR3A	1564318_at	2.685641	2.661719	2.625949	2.663111	2.714333	2.633707	3.01376	2.662123	2.646767	2.635214	2.642785	2.656112	2.464457

C19orf16	1564319_at	2.493349	2.482719	2.427747	2.464536	2.540265	3.022875	2.455243	2.482021	2.465809	2.413846	2.465379	2.603658	2.279378
hCG_19838	1564327_at	2.427262	2.394114	2.352551	2.378825	2.441895	2.376418	2.386083	2.394539	2.381084	2.367896	2.379434	2.391547	2.302963
LOC162993	1564331_at	3.176651	3.086865	2.960949	3.072573	3.160115	3.067228	3.081545	3.092693	3.056163	3.072998	3.175551	3.115148	2.947184
PSAPL1	1564333_a_at	3.007731	2.894375	2.983007	2.948187	2.99924	2.983257	2.999965	3.247106	2.992415	2.987585	2.990973	2.992598	2.852717
CHRM3	1564339_a_at	2.224461	2.207431	2.194516	2.194086	2.210389	2.193116	2.204299	2.206718	2.195503	2.188924	2.192434	2.198985	2.335106
ATP9B	1564344_at	2.241907	2.215954	2.174798	2.202512	2.229971	2.197337	2.213307	2.221278	2.344812	2.198822	2.215933	2.215912	2.169767
CECR3	1564352_at	2.279139	2.270175	2.24945	2.319411	2.276989	2.258172	2.254941	2.269501	2.250689	2.240276	2.252615	2.261643	2.202426
ZNF568	1564356_at	2.569311	2.557317	2.539955	2.544979	2.606772	2.54042	2.551424	2.546567	2.54481	2.544328	2.553122	2.549864	2.323006
ABHD12B	1564357_at	2.547972	2.481272	2.435519	2.462681	2.541283	2.449388	2.47655	2.487784	2.469676	2.456716	2.474086	2.574747	2.330043
LOC339260	1564359_a_at	3.152623	3.114524	3.057661	3.091783	3.172554	3.003963	3.097681	3.111749	3.093822	3.07062	3.099558	3.147441	2.751707
LOC339260	1564360_a_at	2.688677	2.643622	2.577669	2.616678	2.678142	2.625224	2.634424	2.643522	2.630161	2.610345	2.678891	2.654605	2.448195
MGC46336	1564362_x_at	3.244199	3.238777	3.187158	3.216414	3.254741	3.214735	3.225502	3.236471	3.21699	3.218051	3.251567	3.219035	2.92119
CXorf25	1564367_at	1.992648	1.974225	1.942203	1.960218	1.988387	1.959202	1.970096	1.971491	1.961293	1.958318	1.958716	1.967644	1.903359
CASC2	1564371_a_at	2.374383	2.338985	2.299407	2.320224	2.356295	2.311742	2.329643	2.34052	2.325574	2.313631	2.326774	2.334983	2.210027
CASC2	1564372_s_at	2.504203	2.472605	2.419388	2.456779	2.491203	2.432968	2.462143	2.470211	2.46039	2.452105	2.477783	2.470488	2.401061
LOC283887	1564373_a_at	1.687219	1.680378	1.660845	1.665027	1.673506	1.664293	1.675561	1.675723	1.675841	1.662612	1.66431	1.668976	1.650454
SHANK2	1564377_at	2.377475	2.339018	2.323698	2.327105	2.410475	2.338648	2.334683	2.339309	2.328584	2.319714	2.332215	2.338753	2.21554
FLJ35934	1564383_s_at	3.035303	3.005754	2.838804	2.99349	3.013922	2.970309	2.988202	3.121086	2.962555	2.959305	3.219146	3.347466	3.205554
LOC219688	1564385_at	2.46128	2.554607	2.430251	2.493808	3.058149	2.6101	2.440743	2.435889	2.43556	2.371324	2.419193	2.440103	2.345802
TXNDC8	1564386_at	2.492568	2.46702	2.433376	2.441468	2.479819	2.425113	2.460552	2.46534	2.450895	2.450058	2.450982	2.460642	2.405587
DOPEY1	1564387_at	2.88019	2.818056	2.731863	2.788893	2.869432	2.784397	2.821461	2.800777	2.817696	2.798182	2.844032	3.024617	2.481718
C21orf131	1564392_at	2.097781	2.078245	2.045196	2.055017	2.083911	2.066239	2.073117	2.071221	2.071041	2.048655	2.064514	2.075529	2.009583
ARMCX4	1564399_a_at	2.761802	2.73889	2.691122	2.721385	2.743977	2.718827	2.729913	2.731119	2.724407	2.717215	2.72073	2.734385	2.730352
LOC284274	1564400_at	2.639661	2.577293	2.543284	2.558122	2.723349	2.663647	2.485789	2.575618	2.529372	2.538077	2.56106	2.573753	2.394189
LOC400748	1564401_at	2.465776	2.447717	2.430133	2.432336	2.463476	2.430255	2.421452	2.443657	2.43018	2.426539	2.424698	2.438132	2.266489
LOC146795	1564402_at	2.43405	2.402538	2.365437	2.385412	2.416436	2.389775	2.391716	2.399667	2.385754	2.372058	2.384012	2.396825	2.338588
TOPORS	1564403_at	3.5355	3.446412	3.467038	3.475481	3.631622	3.473477	3.486048	3.488369	3.47809	3.469251	3.596818	3.487939	3.24591
PNLDC1	1564414_a_at	2.651446	2.627133	2.584731	2.610208	2.63245	2.594161	2.619143	2.625192	2.612948	2.594978	2.610138	2.622793	2.502987
SEC16B	1564423_a_at	2.60814	2.463731	4.76201	2.512217	2.493371	2.460313	2.470732	2.418454	2.462523	2.469263	2.418883	2.464713	2.416352
LOC389634	1564426_x_at	2.534905	2.514371	2.464493	2.485325	2.519342	2.493107	2.491256	2.510703	2.51872	2.476446	2.49881	2.542822	2.368859
FLJ45224	1564431_a_at	1.725191	1.720258	1.699535	1.703757	1.711364	1.708182	1.714469	1.711986	1.710573	1.705719	1.700442	1.704331	1.695527
KRT72	1564435_a_at	2.492858	2.468216	2.407569	2.44768	2.479048	2.453747	2.465602	2.803318	2.444321	2.450912	2.451492	2.461368	2.311502
C11orf36	1564439_a_at	1.60005	1.59153	1.575004	1.578941	1.584731	1.58174	1.591009	1.591221	1.586628	1.578486	1.574122	1.583292	1.554285
DLEU2	1564443_at	2.2406	2.166122	2.151559	2.167005	2.186953	2.162005	2.174924	2.183838	2.183414	2.171815	2.172846	2.178778	2.083564
LOC284930	1564446_at	2.212777	2.195149	2.163566	2.175674	2.201227	2.176865	2.184837	2.19301	2.178295	2.172015	2.176882	2.187086	2.106851
LOC286442	1564460_at	2.378311	2.324623	2.28491	2.310152	2.358568	2.29669	2.324134	2.327264	2.314387	2.305572	2.313754	2.319697	2.224004
LOC283711	1564463_at	2.666352	2.903143	2.59845	2.635062	2.741904	2.633459	2.325018	2.648917	2.617469	2.620537	2.63013	2.682982	2.303217
FLJ37644	1564466_at	2.399154	2.366756	2.324258	2.344798	2.391348	2.334984	2.354659	2.367138	2.351419	2.335082	2.395339	2.362049	2.211294

FLJ13305	1564467_at	2.439458	2.407919	2.37192	2.395719	2.421724	2.375494	2.517786	2.416958	2.406994	2.938005	2.413049	2.413185	5.033866
LMOD3	1564469_at	3.626147	3.388509	3.316675	3.441589	3.707588	3.39372	3.465368	3.432481	3.472963	3.411929	4.21991	3.607624	4.614612
PDGFA	1564472_at	2.168101	2.13987	2.123203	2.127456	2.164156	2.128829	2.138577	2.13935	2.13058	2.123275	2.129886	2.14031	1.97187
ESCO2	1564473_at	3.385966	3.33649	3.335907	3.341807	3.483868	3.303958	3.273172	3.352015	3.333707	3.25113	3.408972	3.420793	3.171875
LOC728723	1564474_at	2.743849	2.650225	2.629451	2.625812	2.654029	2.601315	2.626237	2.646396	2.63477	2.621537	2.632716	2.750833	2.6159
LOC728723	1564475_s_at	3.673012	3.693009	3.721116	3.611232	3.870785	3.66799	3.675701	3.70381	3.654235	3.883535	3.679028	3.697264	3.52001
ATP5O	1564482_at	2.055599	2.036293	2.015037	2.022865	2.040105	2.024495	2.035377	2.033336	2.022487	2.014728	2.020793	2.02797	1.957296
LOC254808	1564485_at	2.526143	2.488719	2.436097	2.475005	2.51082	2.45281	2.477265	2.488363	2.474364	2.459645	2.471082	2.48813	2.328357
CXorf18	1564491_at	2.91567	2.892038	2.859934	2.873313	2.897356	2.867765	2.868496	2.880313	2.871147	2.867193	2.877155	2.911542	2.608343
P4HB	1564494_s_at	6.742402	6.622956	8.393073	7.252661	4.151684	3.457016	8.873301	4.678792	8.880123	8.868208	4.001653	4.654432	8.947564
C14orf81	1564499_at	2.220731	2.195946	2.167732	2.177654	2.203083	2.177164	2.189909	2.195466	2.18005	2.172718	2.181132	2.188705	2.093407
ACCN5	1564504_at	2.164683	2.14111	2.102474	2.12549	2.14747	2.123607	2.129665	2.137335	2.128143	2.120902	2.12128	2.1333	2.070468
FSTL4	1564511_a_at	2.398739	2.386405	2.366113	2.366245	2.397278	2.349773	2.370044	2.382612	2.371069	2.365956	2.363762	2.376782	2.32185
SNRK	1564514_at	2.095441	2.073396	2.050996	2.059854	2.135409	2.056216	2.0707	2.071575	2.061673	2.055513	2.065258	2.069936	1.990229
PRMT5	1564520_s_at	6.94056	6.951823	6.524196	6.693847	7.014312	7.188223	7.651823	8.376782	7.97359	7.857373	8.303322	8.434774	7.462538
PRMT5	1564521_x_at	6.692558	6.630525	6.550625	6.384643	6.921688	6.694804	7.189809	7.51665	7.559996	7.35916	7.548473	7.791091	7.33194
LOC732096	1564533_at	2.300453	2.27347	2.239707	2.253575	2.281623	2.245144	2.262283	2.272548	2.258843	2.248573	2.258276	2.268529	2.164143
ASB10	1564536_at	2.150707	2.127262	2.101339	2.11058	2.133472	2.106785	2.120032	2.126183	2.113987	2.106827	2.111589	2.120181	2.036886
ASB10	1564537_a_at	2.26026	2.224059	2.197005	2.20624	2.231724	2.206702	2.221644	2.225816	2.213316	2.203208	2.21215	2.219775	2.127656
LOC647323	1564539_at	2.165684	2.140441	2.116502	2.124139	2.147867	2.122991	2.133671	2.143699	2.128216	2.120238	2.126716	2.137927	2.0488
LOC644450	1564544_x_at	2.102517	2.094033	2.077003	2.07589	2.088178	2.081386	2.093431	2.093038	2.090591	2.080181	2.077381	2.224777	2.030301
hCG_19998	1564548_at	2.552168	2.511028	2.459923	2.49107	2.535691	2.49833	2.500668	2.509313	2.496868	2.475955	2.500449	2.510709	2.420885
LOC728073	1564559_at	2.742633	2.693671	2.662878	2.675761	2.749639	2.674349	2.696473	2.70515	2.696079	2.669964	2.69107	2.721819	2.390443
LOC402778	1564573_at	2.246158	2.238754	2.241648	2.219553	2.227857	2.226425	2.234455	2.224282	2.230098	2.218911	2.215713	2.216526	2.140641
OFCC1	1564590_a_at	2.178337	2.159961	2.136187	2.140441	2.168344	2.143975	2.152837	2.158227	2.149773	2.140329	2.142499	2.1729	2.072571
TMC1	1564591_a_at	2.208732	2.191354	2.167465	2.17493	2.200454	2.173119	2.188004	2.19139	2.17874	2.16484	2.17315	2.181676	2.093345
VWA3B	1564598_a_at	3.290196	3.250642	3.09314	3.234611	3.278196	3.238081	3.225481	3.236262	3.220911	3.154084	3.23249	3.311619	2.913354
CCDC36	1564600_a_at	2.629632	2.600933	2.544296	2.577511	2.621074	2.582913	2.58727	2.596997	2.583938	2.569797	2.580346	2.592432	2.435936
LOC286123	1564601_at	2.281276	2.259282	2.239013	2.239509	2.267417	2.231284	2.254043	2.256541	2.242916	2.237418	2.243406	2.251225	2.142721
NUT	1564603_at	1.996226	1.989856	1.964349	1.973889	1.986977	1.978471	1.983383	1.984335	1.975109	1.970777	1.966934	1.97681	1.939717
RP5-821D1'	1564621_a_at	2.102094	2.080426	2.060812	2.065917	2.080389	2.062657	2.076503	2.079608	2.066797	2.05975	2.066195	2.072081	1.995731
DNAJC13	1564627_at	2.826525	2.771755	2.700618	2.74712	2.837425	2.725963	2.767608	2.779699	2.762415	2.738377	2.765344	2.794755	2.543872
EDN1	1564630_at	2.019934	2.005238	1.983867	2.024311	2.004265	1.989509	1.999181	2.002152	1.989579	1.985191	1.984625	1.997431	1.928101
FHAD1	1564635_a_at	2.351169	2.321118	2.246585	2.301561	2.33482	2.266962	2.314892	2.319423	2.307541	2.299959	2.307761	2.317183	2.194857
FAM98B	1564637_a_at	7.154182	7.038646	6.697652	6.720845	8.144968	8.686417	9.481201	10.23837	9.251602	9.255977	9.896296	10.33746	8.695144
LOC389906	1564639_at	4.394347	3.702557	3.61544	3.609083	3.833544	4.00328	3.58194	4.017629	3.639605	3.476252	4.459011	3.697606	3.575743
MGA	1564640_at	3.851722	3.831785	3.524283	3.548125	3.753083	3.365017	3.700662	3.884358	3.708207	3.712412	3.661361	3.728638	3.399422
RUNX1T1	1564642_at	2.486519	2.448616	2.403337	2.432859	2.575468	2.432819	2.442099	2.45183	2.43694	2.426717	2.442458	2.449294	2.301844

LOC221710	1564651_at	2.909402	2.863311	3.224565	2.898612	2.789127	2.846773	2.863668	2.910695	2.824008	2.856102	2.721368	2.715549	2.415062
LOC152084	1564653_s_at	2.401235	2.395594	2.352876	2.349632	2.397489	2.333252	2.371497	2.37215	2.358974	2.358924	2.353983	2.361662	2.729909
C7orf52	1564658_at	5.148027	5.748411	4.926629	5.010431	5.038716	5.276847	4.539722	5.01731	4.744013	4.628409	4.830611	5.016761	4.396768
LOC285346	1564662_at	2.369312	2.338548	2.319414	2.319281	2.354112	2.309342	2.327409	2.33858	2.324697	2.310601	2.324776	2.33345	2.209259
CDC20B	1564674_a_at	2.279487	2.263953	2.241453	2.24395	2.264102	2.24512	2.25729	2.261365	2.247245	2.240243	2.245943	2.25574	2.188957
ASB15	1564679_at	2.459362	2.424211	2.38128	2.403506	2.496359	2.393556	2.415474	2.424393	2.410164	2.398667	2.421802	2.439243	2.286251
LOC283868	1564683_at	2.47726	2.451398	2.409294	2.423925	2.590555	2.440121	2.432975	2.443964	2.443536	2.395147	2.460038	2.451945	2.381931
BTN2A2	1564684_at	2.057352	2.038721	2.015746	2.020308	2.039011	2.019332	2.032059	2.0371	2.022945	2.015145	2.021547	2.030928	1.963926
RAD9B	1564688_a_at	3.25932	3.159098	3.020629	3.109853	3.307357	3.132423	3.081176	3.144728	3.101494	3.142358	3.168517	3.223101	2.767578
LOC400752	1564697_a_at	1.719171	1.670082	1.649696	1.648928	1.707977	1.653798	1.665759	1.654928	1.657253	1.649818	1.64613	1.647654	1.644562
GLS2	1564705_at	3.71919	3.620862	3.414435	3.570675	3.865751	3.357925	3.42493	3.472096	3.394449	3.399454	3.454584	3.483105	2.868602
GLS2	1564706_s_at	7.133709	7.751522	9.251489	9.088829	7.869684	6.843045	7.204594	4.554463	6.62369	6.538905	3.340737	3.465752	2.415583
GLS2	1564707_x_at	4.144772	4.257645	4.90179	4.644417	4.664265	3.780827	3.795503	3.606858	3.804585	3.666532	3.831015	3.879436	2.959983
LOC286238	1564709_at	3.054683	2.951882	2.880029	2.906231	3.105438	2.918418	2.90795	2.910296	2.937977	2.880977	2.988717	3.011367	2.546875
FOXN4	1564713_a_at	2.411246	2.402437	2.381113	2.382306	2.40021	2.371596	2.391322	2.40234	2.398207	2.389602	2.385877	2.397016	2.285855
CASP12	1564736_a_at	2.401248	2.373152	2.329855	2.356021	2.387467	2.352949	2.368182	2.375786	2.361345	2.352942	2.360723	2.461328	2.263474
NHEDC2	1564746_at	3.801612	3.780859	4.561397	4.32382	3.630567	3.30109	4.055702	3.777744	4.285333	4.424362	3.540075	3.782691	3.581111
LOC130940	1564757_a_at	3.030077	2.900705	2.935551	2.956778	3.123577	2.95084	2.942958	2.968697	2.958266	2.977181	3.004257	3.088714	2.827519
hCG_20191	1564758_at	2.638634	2.580751	2.538862	2.571289	2.622639	2.566118	2.582865	2.593641	2.579822	2.559408	2.579432	2.604656	2.429646
LENG10	1564776_at	4.066558	3.195912	2.911022	2.865513	3.841614	3.345409	2.970748	2.836011	2.978179	2.875024	2.980035	2.987829	3.317779
HYALP1	1564777_at	2.724484	2.697759	2.665023	2.679199	2.741299	2.633475	2.686523	2.694957	2.681932	2.673408	2.677845	2.710866	2.59092
OFCC1	1564783_x_at	2.679002	2.638981	2.618563	2.622411	2.650541	2.621917	2.634389	2.634109	2.624295	2.60155	2.618605	2.635143	2.568282
LOC196913	1564785_at	1.861563	1.849957	1.830456	1.83393	1.867116	1.835844	1.84482	1.847684	1.835175	1.829451	1.830229	1.846018	1.785145
LOC338667	1564786_at	2.599311	2.564233	2.511884	2.546956	2.587147	2.551504	2.557978	2.568717	2.554902	2.547425	2.551318	2.571214	2.489656
LOC643923	1564787_at	1.889105	1.877255	1.859686	1.857398	1.877064	1.860078	1.87468	2.638905	1.863159	1.858885	1.856538	1.868813	1.816167
ST7OT2	1564790_at	3.115932	3.001646	2.949373	3.001326	3.117708	3.125077	3.022798	3.030098	3.02347	2.991882	3.031439	3.065415	2.771269
EMP1	1564796_at	2.060147	2.056061	2.035313	2.039151	2.069604	2.03724	2.035808	2.053681	2.04081	2.018628	2.036167	2.048993	2.080978
KRTAP11-1	1564803_at	1.821809	1.813975	1.793921	1.799435	1.81258	1.798499	1.811503	1.812145	1.806729	1.799755	1.797251	1.803789	1.774525
OFCC1	1564805_a_at	1.962732	1.948124	1.928472	1.934311	1.950077	1.932609	1.94073	1.946704	1.93536	1.927535	1.927761	1.94204	1.879729
C14orf178	1564816_at	2.121174	2.111819	2.092359	2.093772	2.111597	2.093915	2.112274	2.108991	2.107164	2.102471	2.09683	2.123704	2.064062
CDC42BPG	1564823_at	2.049618	2.033256	2.013401	2.015889	2.03286	2.014461	2.024023	2.028802	2.017483	2.007857	2.013163	2.0225	1.951095
KIAA1211	1564828_at	2.21638	2.196438	2.17727	2.177925	2.199564	2.245622	2.187775	2.193989	2.180966	2.171203	2.18015	2.188074	2.108904
LOC151760	1564837_at	2.520056	2.479393	2.433247	2.464146	2.507449	2.447107	2.474466	2.479344	2.471075	2.453852	2.472839	2.483663	2.32765
LOC151760	1564838_a_at	1.968896	1.953687	1.932861	1.938429	1.95363	1.936225	1.948975	1.950971	1.940504	1.934566	1.93939	1.945289	1.883907
OR4N4	1564855_at	3.540312	3.414959	3.280494	3.594726	3.521494	3.361404	3.421202	3.438664	3.462083	3.410312	3.591328	3.499771	2.854691
OR4N4	1564856_s_at	2.369335	2.344099	2.298512	2.319203	2.356419	2.3271	2.331653	2.34101	2.328302	2.316721	2.324691	2.337604	2.21875
ALS2CR13	1564868_a_at	2.263492	2.243597	2.225416	2.2315	2.247608	2.215243	2.241196	2.239184	2.231809	2.224262	2.239822	2.238722	2.193435
FOXP2	1564876_s_at	3.787859	3.728649	3.705438	3.738993	3.742069	3.711833	3.750872	3.884137	3.745581	4.054962	3.769803	3.922657	3.290845

LOC152578	1564881_at	2.226503	2.204976	2.179769	2.187219	2.211393	2.189417	2.196419	2.205388	2.188299	2.180456	2.190695	2.198123	2.099741
TAS1R1	1564883_a_at	2.27547	2.24851	2.222679	2.22566	2.273105	2.227389	2.23727	2.245522	2.232745	2.223361	2.224398	2.240282	2.13568
SNHG4	1564906_at	2.219263	2.189856	2.161218	2.170893	2.196753	2.168524	2.179281	2.189168	2.172601	2.166905	2.176346	2.223033	2.091614
MATR3	1564907_s_at	2.511081	2.6924	2.265135	2.403185	2.595344	2.584163	2.476115	2.384885	2.362464	2.396859	2.324081	2.337287	2.354547
SNHG4	1564911_at	2.711878	2.6802	2.613188	2.66161	2.695501	2.652608	2.674901	2.677365	2.676947	2.656107	2.666564	2.689926	2.557606
KRTAP13-1	1564921_at	2.066514	2.056113	2.034413	2.036622	2.053282	2.041158	2.049495	2.052478	2.039223	2.031866	2.031738	2.134543	1.965322
TBX20	1564945_at	2.586831	2.547329	2.501674	2.532158	2.60168	2.524654	2.541795	2.550943	2.538296	2.518386	2.536903	2.549741	2.397565
KRTAP7-1	1564960_at	2.42848	2.343073	2.310346	2.329237	2.361835	2.327114	2.338612	2.342306	2.327636	2.317816	2.322905	2.336912	2.189117
ZNF92	1564962_at	3.447869	3.258682	3.233801	3.300109	3.503858	3.263772	3.340219	3.314024	3.339798	3.309864	3.384406	3.439173	3.186866
ZNF92	1564963_x_at	3.030995	2.983118	2.873361	2.965869	3.047007	2.893794	2.973479	2.979367	2.965249	2.940788	2.976672	3.048386	2.933687
SETDB2	1564970_at	3.607995	3.996468	2.766628	2.488976	2.527331	3.397813	2.456892	2.508996	2.46069	2.479388	2.558718	2.487694	2.358432
SETDB2	1564972_x_at	3.231135	3.686094	2.525591	2.609919	2.694351	2.728512	2.400427	2.576762	2.56012	2.543825	2.557704	2.517847	2.400366
KRTAP8-1	1564974_at	2.055996	2.03798	2.013603	2.024532	2.038231	2.024397	2.029909	2.035869	2.02162	2.022162	2.017634	2.02965	1.961719
SLC8A1	1564985_a_at	2.07496	2.051394	2.031896	2.037798	2.057725	2.034119	2.04591	2.052716	2.039769	2.022896	2.038586	2.049415	1.973786
TCP11L2	1565000_a_at	2.33218	2.322415	2.304832	2.305515	2.323848	2.30529	2.31758	2.316097	2.307762	2.306447	2.299617	2.395975	2.190481
PRMT1	1565016_at	2.338424	2.319805	2.298191	2.302143	2.270745	2.321868	2.314691	2.316645	2.303524	2.296979	2.29938	2.851659	2.252498
OFCC1	1565026_a_at	2.981734	2.893596	2.834054	2.87689	2.933239	2.826333	2.885321	2.893541	2.883303	2.865283	2.88406	2.91879	2.714123
OFCC1	1565027_at	2.899868	2.870679	2.873972	2.850498	2.888397	2.879401	2.890403	2.953045	2.883115	3.009268	2.875006	2.882858	2.630119
AFF3	1565034_s_at	2.051133	2.03326	2.006533	2.014824	2.03507	2.01254	2.022803	2.029657	2.017002	2.009977	2.012837	2.024796	1.955389
OFCC1	1565065_at	2.09572	2.080417	2.043014	2.062582	2.122644	2.067091	2.072647	2.077421	2.064766	2.052638	2.059444	2.072453	1.950946
SRC	1565080_at	2.149382	2.127864	2.101022	2.108128	2.132404	2.108717	2.119239	2.127664	2.114678	2.105237	2.113272	2.121492	2.037836
SRC	1565082_x_at	2.077056	2.056438	2.02912	2.04139	2.055549	2.034531	2.053668	2.05751	2.042423	2.038089	2.039049	2.050001	1.966564
OFCC1	1565111_x_at	2.489125	2.44884	2.386263	2.432492	2.455512	2.397396	2.441331	2.449607	2.436937	2.427678	2.440967	2.452789	2.295987
RBMY3AP	1565132_at	2.340076	2.319139	2.298528	2.310164	2.33573	2.349562	2.313146	2.31808	2.318488	2.299409	2.307542	2.432748	2.193278
DYNC2H1	1565149_at	3.286973	3.433049	3.249957	3.170124	3.343394	3.414911	3.173753	3.26264	3.256525	3.149895	3.237911	3.264396	4.867299
MGST1	1565162_s_at	11.65915	11.54901	13.1188	12.65503	11.03228	9.621103	11.01304	8.669592	10.75359	10.87085	8.034327	8.393253	8.326368
ALB	1565228_s_at	15.26264	15.2829	15.34856	15.47533	14.50962	13.5921	15.67622	14.09189	15.67622	15.72807	13.54351	13.90873	13.81422
TNXB	1565242_at	2.583768	2.546914	2.487961	2.524496	2.573477	2.499639	2.538141	2.543772	2.532624	2.511983	2.529595	2.548421	2.355415
ELL	1565254_s_at	2.315385	2.277916	2.260191	2.265421	2.292603	2.263982	2.269432	2.276195	2.266954	2.262936	2.258005	2.270989	2.162051
ATF1	1565269_s_at	2.997182	3.036541	3.960387	3.597734	3.777172	3.036888	5.224169	2.990228	5.012521	4.541499	2.957566	3.848945	5.599666
SLC8A1	1565306_a_at	2.070792	2.05117	2.030034	2.032239	2.065971	2.032106	2.036497	2.046129	2.03558	2.021253	2.033676	2.045404	1.987876
RBMY3AP	1565320_at	4.209477	4.82896	4.073591	4.167945	4.191631	4.036014	4.174006	4.186911	4.172778	4.161228	4.1715	4.147758	3.718479
SNHG4	1565325_at	2.783917	2.723798	2.679676	2.709112	2.747959	2.711003	2.718757	2.7248	2.711369	2.691099	2.714265	2.723623	2.507244
POLE4	1565329_at	3.173965	3.091992	2.985226	3.084736	3.210217	3.054927	3.072468	3.079284	3.086918	3.067944	3.134163	3.198912	2.739741
DNAH6	1565337_at	2.221092	2.182897	2.145028	2.163352	2.190881	2.167811	2.173742	2.176855	2.170043	2.167945	2.166439	2.1764	2.085003
DNAH6	1565338_x_at	2.413717	2.378357	2.349403	2.36066	2.387879	2.35707	2.372757	2.375297	2.364173	2.345107	2.359745	2.373565	2.278792
DNAH10	1565339_at	2.094907	2.076511	2.044771	2.057567	2.076238	2.054738	2.07035	2.071585	2.07063	2.05647	2.059471	2.069501	1.98828
ATP1A4	1565346_a_at	2.634876	2.529925	2.492643	2.514913	2.563724	2.494966	2.518806	2.529013	2.51978	2.504257	2.51652	2.54012	2.363677

TFE3	1565347_s_at	2.827056	2.789497	2.708076	2.771473	2.806896	2.799351	2.75616	2.774887	2.762519	2.741901	2.766051	2.779381	2.703933
RARA	1565358_at	2.031415	2.015418	1.991038	1.998228	2.02056	1.997204	2.012126	2.015288	1.999735	1.994111	2.000532	2.009899	1.991303
GRM5	1565389_s_at	2.749462	2.699535	2.504661	2.70265	2.839241	2.679933	2.609022	2.718256	2.701255	2.59415	2.781916	2.75407	3.253783
LHX9	1565406_a_at	1.579463	1.574158	1.557596	1.558303	1.563911	1.561426	1.563728	1.563709	1.565248	1.551326	1.555984	1.559949	1.542007
LHX9	1565407_at	2.211603	2.193139	2.176115	2.18116	2.202955	2.177133	2.191713	2.192483	2.184829	2.171554	2.175481	2.186886	2.109124
C8orf8	1565424_at	2.446089	2.40557	2.362481	2.388316	2.434055	2.398791	2.409542	2.412459	2.401503	2.394078	2.399445	2.419422	2.290631
MLL	1565436_s_at	2.64667	2.604279	2.550481	2.587314	2.644281	2.57472	2.602837	2.600944	2.595494	2.576157	2.618574	2.620347	2.488781
HPRT1	1565446_at	3.814255	3.593132	3.545741	3.638229	3.881763	3.563775	3.598274	3.627231	3.664444	3.616537	3.734765	3.818977	3.208971
XAGE3	1565454_at	1.728107	1.676204	1.657002	1.65951	1.686192	1.663497	1.671533	1.673993	1.667253	1.685971	1.653709	1.659005	1.653558
EGFR	1565483_at	1.816142	1.80516	1.804118	1.844173	1.831565	3.433472	1.749708	1.802532	1.744976	1.791704	1.786877	1.798143	1.727212
EGFR	1565484_x_at	2.532465	2.50315	2.511405	2.843605	2.637349	2.494058	2.471674	2.493809	2.472193	2.477138	2.479721	2.493949	2.296146
TCP11L2	1565525_a_at	2.522354	2.483855	2.447634	2.47083	2.505732	2.459214	2.606352	3.163897	2.762438	5.057348	2.475052	2.521529	2.33132
TCP11L2	1565527_x_at	1.956555	1.940342	1.92044	1.922634	1.937658	1.926977	1.936938	1.938355	1.932689	1.924892	1.920712	1.933613	1.901481
NKX1-1	1565537_at	1.070664	1.064145	1.046239	1.048703	1.054339	1.047985	1.062481	1.048625	1.059374	1.047842	1.040294	1.040102	1.061833
RNF141	1565544_at	3.411854	3.302741	3.1359	3.257942	3.439155	3.270276	3.269851	3.304567	3.288425	3.280617	3.322989	3.397513	2.848939
RNF141	1565546_at	2.955717	2.870337	2.821217	2.871926	2.979265	2.837883	2.876644	2.882023	2.876316	2.867032	2.877289	2.905361	2.705614
LOC127841	1565554_at	3.721074	3.683366	3.670481	3.675057	3.706705	3.65142	3.645091	3.645559	3.616408	3.672107	3.623493	3.853463	2.858128
LOC127841	1565556_at	2.231463	2.216702	2.198289	2.202863	2.216652	2.202158	2.215303	2.216758	2.20523	2.197589	2.205356	2.210333	2.145279
MAGIX	1565559_at	1.837039	1.825134	1.806335	1.800113	1.833231	1.808251	1.818713	1.818938	1.812384	1.806871	1.808761	1.815994	1.787867
MAGIX	1565562_at	2.501453	2.25443	2.218339	2.234886	2.256855	2.233399	2.245455	2.251512	2.237152	2.230108	2.238188	2.247257	2.139514
TATDN2	1565580_s_at	2.335051	2.311886	2.295615	2.293433	2.306879	2.301941	2.29719	2.307513	2.293898	2.282297	2.294666	2.300399	2.214975
ZSCAN5	1565583_at	2.593904	2.569634	2.532337	2.560925	2.580601	2.555936	2.57048	2.573987	2.56526	2.560768	2.562165	2.577493	2.45
SP140	1565588_at	2.553414	2.509166	2.462087	2.496468	2.540824	2.476843	2.50635	2.515945	2.503343	2.485383	2.498262	2.518669	2.352594
PWP2	1565603_at	2.453776	2.497739	2.418176	2.419621	2.426878	2.419915	2.415656	2.428353	2.253665	2.278799	2.411707	2.41995	2.2116
PWP2	1565604_at	3.54199	3.491405	3.231985	3.405359	3.517622	3.478476	3.470182	3.482074	3.466709	3.447259	3.47064	3.535872	3.275737
MFSD1	1565608_at	2.407545	2.372799	2.318015	2.356631	2.379785	2.361393	2.352884	2.365084	2.355948	2.337388	2.351187	2.375901	2.230037
DYNLRB1	1565611_at	3.639262	4.109829	3.588938	3.504077	3.531926	3.553978	3.56465	3.741149	3.663553	3.585849	3.547227	3.578046	3.31963
DYNLRB1	1565612_at	2.40945	2.398202	2.358618	2.379643	2.401036	2.381508	2.388528	2.397699	2.382205	2.381255	2.383006	2.470546	2.344975
LOC729865	1565618_at	2.36836	2.340282	2.305415	2.321518	2.3838	2.31449	2.329484	2.341428	2.326405	2.31783	2.327728	2.337824	2.211152
CTGLF1	1565620_at	7.956941	7.541671	7.513902	7.650251	7.891749	7.823346	7.977434	7.672288	7.511876	7.304212	8.076567	7.50434	6.947498
C16orf45	1565641_at	2.385399	2.370103	2.358965	2.351903	2.374942	2.36382	2.374713	2.371893	2.370754	2.344123	2.36003	2.422631	2.330545
LOC143286	1565644_at	5.001833	5.309196	4.611189	5.124311	4.921893	4.524162	4.525462	4.537114	4.526931	4.522846	4.565654	4.530079	4.115135
ARF1	1565651_at	4.468229	5.112084	4.944282	4.465162	4.490735	4.532745	4.556961	4.195516	4.67966	4.681949	4.357346	4.231075	3.280542
KIAA1833	1565653_at	3.275751	3.194162	3.034657	3.109727	3.301021	3.068229	3.211147	3.18624	3.171818	3.116105	3.236598	3.266925	2.93899
CMTM6	1565657_at	3.661916	3.515518	3.443147	3.516595	3.735625	3.463771	3.474874	3.533965	3.504211	3.428868	3.580114	3.643821	3.000945
FUT6	1565659_at	2.278418	2.273294	2.234912	2.252612	2.284821	2.244915	2.271267	2.272927	2.266284	2.247998	2.358803	2.266428	2.236623
FUT6	1565660_at	2.630517	2.559937	2.540008	2.542593	2.573028	2.539522	2.512179	2.556524	2.469801	2.543692	2.757853	2.551174	2.143344
FUT6	1565661_x_at	2.743497	3.030024	2.471029	2.673941	2.72648	2.782023	2.658001	2.723145	2.718417	2.700754	2.768915	2.534165	2.53958

MUC6	1565662_at	3.32014	3.250224	3.164548	3.226564	3.310153	3.073593	3.256354	3.406817	3.243637	3.213516	3.253179	3.290193	3.008039
MUC6	1565666_s_at	2.067376	2.049218	2.024527	2.030113	2.051992	2.028365	2.040474	2.047426	2.032494	2.025038	2.030973	2.041961	1.962444
TUBA1B	1565668_at	1.931317	1.921583	1.904293	1.908611	1.920486	1.910087	1.919505	1.920709	1.911358	1.907288	1.903135	1.913114	1.854366
LOC391142	1565671_a_at	2.756856	2.760443	2.725931	2.713023	2.757006	2.865055	2.72833	2.742336	2.723289	2.685872	2.723249	2.758317	2.595432
FCGR2A	1565673_at	2.624533	2.554712	2.506153	2.542423	2.593366	2.522311	2.553459	2.565109	2.551256	2.5328	2.558592	2.569204	2.390031
FCGR2A	1565674_at	2.738983	2.626411	2.639594	2.682786	2.877751	2.668848	2.703139	2.71305	2.725253	2.678012	2.698934	2.824691	2.495155
DIP2C	1565680_at	2.176992	2.140376	2.119152	2.141388	2.170936	2.130876	2.160583	2.165828	2.150229	2.147942	2.158904	2.197809	2.024566
DIP2C	1565681_s_at	3.85518	3.69938	4.85442	4.812257	3.709672	3.107179	4.224224	3.86939	4.148243	4.057093	3.336083	2.63755	5.822487
LOC285144	1565684_at	2.468991	2.447334	2.417908	2.427651	2.450553	2.428447	2.440178	2.446451	2.431292	2.423633	2.435632	2.443615	2.297302
LOC285144	1565685_at	3.297191	3.20199	3.174079	3.192349	3.297132	3.134113	3.202313	3.213241	3.199711	3.181365	3.264357	3.31323	3.031871
DTYMK	1565693_at	3.597352	3.397817	3.347827	3.375623	3.574617	3.317932	3.352356	3.45821	3.360887	3.332025	3.42519	3.499128	2.919979
DTYMK	1565694_at	2.1584	2.1227	2.098826	2.104656	2.121288	2.118165	2.125348	2.120194	2.110093	2.109141	2.105472	2.113107	2.124986
HECTD2	1565699_at	2.831905	2.780182	2.666178	2.748755	2.904371	2.686424	2.755509	2.783815	2.768889	2.754358	2.772859	2.84713	2.716058
SMAD4	1565702_at	2.321509	2.290784	2.253242	2.275059	2.302995	2.267251	2.279732	2.292645	2.277881	2.2673	2.276959	2.295706	2.17004
SMAD4	1565703_at	6.449933	6.402871	6.110604	6.24081	6.011151	5.223692	6.421869	6.360864	6.546892	6.688792	5.817528	6.254194	5.447426
FUS	1565715_at	1.811084	1.800869	1.783832	1.787978	1.959628	1.786963	1.796871	1.797061	1.793398	1.789884	1.779664	1.792212	1.750406
FUS	1565716_at	1.718774	1.703037	1.684731	1.689008	1.701641	1.689978	1.70078	1.698994	1.692368	1.682659	1.686247	1.691197	1.657322
FUS	1565717_s_at	3.263027	3.250588	3.988657	2.746961	2.345716	1.991191	5.684222	2.70223	5.13323	5.553082	3.629986	2.791144	7.362315
LOC284630	1565728_at	3.049559	3.031991	3.003231	3.011288	3.033305	3.013149	3.022954	3.02025	3.015398	3.01222	3.014352	3.037691	2.9397
ALDH3B1	1565731_at	1.371519	1.366284	1.340963	1.347081	1.37678	1.643416	1.357411	1.345864	1.35248	1.339612	1.342442	1.345044	1.364446
SCTR	1565737_at	2.859377	2.794324	2.751434	2.835322	2.90707	2.780444	2.782915	2.79125	2.779117	2.753695	2.778873	2.829526	2.586866
SCTR	1565738_at	2.444171	2.417897	2.470384	2.408547	2.437719	2.399584	2.409776	2.420655	2.402065	2.393332	2.403495	2.414904	2.315709
FGD2	1565752_at	2.087184	2.082968	1.958473	2.063135	2.081998	2.065534	2.080852	2.482751	2.076615	2.128381	2.060044	2.095904	2.070941
FGD2	1565754_x_at	2.251344	2.365017	1.977699	2.206733	2.217746	2.201322	2.369622	2.208183	2.307522	2.405478	2.192618	2.204571	2.18478
MAP1D	1565755_at	2.885508	2.774173	2.711832	2.775916	2.876525	2.732393	2.777638	2.790428	2.782857	2.747331	2.781211	2.837376	2.549458
MAP1D	1565756_a_at	2.325395	2.286954	2.274114	2.28331	2.487393	2.268585	2.295003	2.296562	2.285283	2.28139	2.353917	2.289376	2.286577
RPL13	1565758_at	2.330369	2.319401	2.272352	2.300972	2.31818	2.289385	2.317435	2.323602	2.309252	2.303657	2.308997	2.318034	2.23285
RPL13	1565759_at	2.612344	2.562139	2.511326	2.547922	2.57219	2.552922	2.568373	2.565897	2.663299	2.551717	2.806085	2.589052	3.020941
C13orf33	1565763_at	2.404022	2.398567	2.374306	2.380872	2.413251	2.387111	2.390897	2.397448	2.387343	2.382999	2.381036	2.392117	2.256572
C13orf33	1565765_x_at	3.68304	3.624091	3.393189	3.613295	3.866486	3.591461	3.619434	3.800361	3.596494	3.518676	3.698981	3.65342	3.033815
ZNF268	1565768_at	3.307582	3.265877	3.198558	3.241201	3.298114	3.13693	3.206627	3.265627	3.249693	3.241269	3.251461	3.299938	2.820584
DKFZp434E	1565771_at	2.945184	2.940582	2.88915	2.883809	3.165561	2.89298	2.895563	2.907161	2.889998	2.872579	2.888315	2.938386	2.411891
PAK1	1565772_at	1.55916	1.551212	1.532954	1.535241	1.541319	1.537046	1.549676	1.550115	1.544509	1.535557	1.534614	1.536435	1.513247
FAM38B	1565775_at	2.097903	2.086744	2.064637	2.071981	2.086972	2.066953	2.083729	2.085141	2.075231	2.071119	2.069229	2.079558	2.034007
ABCA8	1565778_at	7.346456	7.060281	7.708821	8.043932	7.624918	7.343718	2.791611	2.802078	2.786315	2.754499	3.102215	3.10096	2.595731
ABCA8	1565780_at	2.041314	2.02701	2.006313	2.012814	2.028122	2.00968	2.022168	2.025167	2.011831	2.003408	2.008784	2.019013	1.947065
FLJ45482	1565786_x_at	6.04513	5.780372	4.216533	4.444395	4.772678	4.27124	6.865842	6.917648	7.478221	6.79325	7.760182	7.81362	6.154279
DUOX1	1565795_at	2.209998	2.185502	2.155531	2.166313	2.192202	2.16534	2.178032	2.179471	2.170736	2.163555	2.16816	2.194726	2.122943

RAB3IP	1565799_at	3.483039	3.260583	3.193834	3.293182	3.593187	3.199107	3.294847	3.339299	3.346757	3.274451	3.36381	3.475419	2.877102
RAB3IP	1565800_x_at	3.65847	3.213367	3.170101	3.244835	3.444572	3.167506	3.289671	3.296577	3.361773	3.260444	3.332716	3.410944	2.851852
LOC728011	1565805_at	1.92903	1.916224	1.894536	1.897447	1.913781	1.897031	1.909713	1.912024	1.900059	1.892183	1.89484	1.907011	1.835214
FLJ37035	1565806_at	3.20085	3.199835	2.955604	3.031819	3.434479	2.95949	3.040239	3.074375	3.064563	3.009616	3.080519	3.119648	2.788478
TRIM36	1565812_at	2.007776	1.991813	1.969272	1.973624	1.99529	1.980464	1.984865	1.988043	1.976204	1.968584	1.972374	1.984298	1.916184
TRIM36	1565814_at	1.964903	1.952386	1.931492	1.935946	1.95137	1.938188	1.946384	1.948435	1.936778	1.930004	1.930094	1.942979	1.885084
IKZF1	1565816_at	2.195072	2.1787	2.158747	2.162242	2.179255	2.160512	2.168663	2.175129	2.163535	2.155639	2.159511	2.170694	2.079128
IKZF1	1565817_at	2.441112	2.396878	2.518559	2.392634	2.406875	2.407485	2.38971	2.846619	2.412137	2.377598	2.979798	2.480356	2.246848
IKZF1	1565818_s_at	2.997993	2.936788	2.828498	2.893416	3.029937	2.928951	2.926394	2.938408	2.923384	2.910081	2.921938	2.981262	2.631254
SEPT7	1565823_at	3.31649	3.044443	3.301471	3.331749	3.307921	3.783124	3.243521	3.506041	3.283349	3.301803	3.319512	3.349996	3.408571
KIAA1731	1565829_at	2.392081	2.362705	2.308114	2.332135	2.374173	2.361445	2.616858	2.360968	2.347097	2.4588	2.372635	2.583887	2.89745
ANKRD15	1565832_at	2.606583	2.528024	2.541239	2.545105	2.585912	2.535184	2.550607	3.159597	2.548329	2.536747	2.542126	2.594201	2.396735
BRPF3	1565843_s_at	1.963848	1.958702	1.93922	1.93911	1.952333	1.940855	1.951343	1.943707	1.941871	1.933753	1.935138	1.937372	1.908991
BRPF3	1565844_at	2.247684	2.19397	2.129625	2.168084	2.221926	2.176262	2.182958	2.191679	2.178879	2.154867	2.177982	2.197994	2.091642
LOC283970	1565845_at	2.129265	2.108575	2.090199	2.090788	2.09733	2.102302	2.102953	2.108444	2.096156	2.090147	2.094045	2.099489	2.107891
ZNF428	1565848_at	2.085565	2.064612	2.124422	2.063453	2.105562	2.148222	2.075815	2.068048	2.071273	2.064496	2.059827	2.066195	1.98706
SNORA71A	1565858_at	2.21683	2.193225	2.159398	2.172764	2.196675	2.167749	2.183449	2.192887	2.176726	2.169143	2.178634	2.221286	2.084819
SNORA71A	1565859_at	2.366946	2.353907	2.341478	2.335479	2.35562	2.334589	2.357747	2.352475	2.351791	2.345207	2.342406	2.349018	2.298764
ZC3H11A	1565867_a_at	3.871099	3.380936	3.488443	3.682061	4.231232	3.813981	3.779639	3.5289	3.622607	3.286233	4.315281	3.843777	3.544913
CD44	1565868_at	2.482298	2.688104	2.592366	2.665587	2.737209	2.677116	2.671015	2.688199	2.675121	2.672111	2.669981	2.726823	3.063469
TRPM7	1565887_at	4.577945	4.356655	3.691481	3.4364	4.186823	3.328204	2.866382	3.362005	3.317066	3.304088	2.986558	2.643978	2.728107
METT5D1	1565898_at	2.955046	2.897363	2.814047	2.88671	2.93885	2.864723	2.890884	2.908577	2.895823	2.859736	2.912146	2.940959	2.803608
METT5D1	1565900_at	2.970614	2.936834	2.919411	2.899689	2.960646	2.922932	2.909761	2.962378	2.913061	2.880266	2.928829	2.95705	2.778166
FLJ46026	1565905_at	1.969321	1.95682	1.937987	1.938847	2.044279	1.95046	1.953412	1.958556	1.94109	1.92863	1.945778	1.951197	1.932853
NADSYN1	1565906_at	2.29305	2.279357	2.21236	2.262415	2.280947	2.261632	2.276659	2.272016	2.259267	2.256872	2.263944	2.276662	2.234014
NADSYN1	1565908_at	2.340101	2.300841	2.277489	2.278828	2.31595	2.271088	2.293242	2.298957	2.283667	2.267532	2.280794	2.293354	2.176698
FSTL4	1565910_at	2.415744	2.387314	2.35234	2.367715	2.397347	2.361475	2.3757	2.384309	2.371556	2.361707	2.367898	2.38095	2.245164
SLC17A4	1565925_at	2.14955	2.1275	2.101438	2.113719	2.132128	2.107874	2.120326	2.124671	2.11686	2.111762	2.111548	2.119645	2.035919
LOC91431	1565934_at	2.245118	2.222723	2.186144	2.204672	2.226868	2.197926	2.217594	2.219606	2.207543	2.198573	2.204616	2.221411	2.118774
LOC91431	1565935_at	4.081382	3.998168	3.682226	3.913231	4.032963	4.089576	4.217882	5.90036	4.025241	4.190685	4.662312	4.614539	3.872285
LMO3	1565936_a_at	3.197733	3.125915	3.013267	3.097961	3.191456	3.130719	3.132709	3.141158	3.128429	3.110394	3.131727	3.167096	2.984886
LMO3	1565937_a_at	2.595484	2.546333	2.509277	2.533189	2.610722	2.531044	2.544242	2.560513	2.546282	2.530724	2.543661	2.573669	2.403049
C5orf22	1565939_at	2.705626	2.645065	2.59763	2.637051	2.693612	2.611244	2.649049	2.656631	2.646412	2.62545	2.668126	2.661944	2.501077
CHML	1565947_a_at	1.38651	1.381707	1.361893	1.364899	1.371896	1.368786	1.377161	1.372393	1.372646	1.366186	1.362555	1.365328	1.356678
CHML	1565949_x_at	1.573101	1.560868	1.543559	1.54648	1.557217	1.54942	1.554363	1.554561	1.554	1.547274	1.542967	1.550532	1.54645
CHML	1565951_s_at	3.872478	3.792698	3.693178	3.782013	3.896825	3.82866	3.861621	4.535555	4.450069	3.779078	5.263515	4.972701	6.227739
LOC286299	1565998_at	2.430307	2.395654	2.37538	2.379992	2.413872	2.370914	2.391631	2.401326	2.419195	2.376325	2.388183	2.39939	2.262884
LOC286299	1565999_at	2.226205	2.202376	2.177368	2.183892	2.205474	2.180329	2.190683	2.20073	2.185711	2.178553	2.18586	2.195445	2.097719

LOC729314	1566037_at	2.494877	2.475338	2.427535	2.457548	2.498405	2.462367	2.467098	2.475297	2.464963	2.454562	2.469276	2.552081	2.321835
DGCR7	1566038_at	2.233666	2.215681	2.191077	2.197436	2.217851	2.194746	2.207257	2.216792	2.202082	2.192522	2.203049	2.21161	2.131387
LOC401220	1566039_a_at	2.311781	2.30179	2.274716	2.281555	2.302873	2.293659	2.289698	2.298738	2.296613	2.275262	2.28103	2.293963	2.285955
LOC647190	1566079_at	8.263262	8.186036	6.176561	6.581694	8.605019	8.764822	7.10029	8.060796	6.851373	7.162548	7.815675	7.666627	5.348473
LOC644215	1566082_at	1.888543	1.878694	1.86154	1.85875	1.939085	1.862895	1.87788	2.441147	1.868622	1.864555	1.860276	1.870918	1.812029
LOC644215	1566084_at	4.623777	5.026491	4.744667	4.83964	4.255202	4.541218	4.765732	4.986862	4.857384	4.758538	5.379725	4.557092	6.114012
ARHGEF12	1566093_at	2.351542	2.293483	2.275014	2.271152	2.330511	2.277789	2.289661	2.28292	2.276555	2.265029	2.273939	2.282226	2.190669
CD6	1566099_at	2.226551	2.210775	2.184882	2.190852	2.210272	2.184916	2.204127	2.203766	2.193441	2.185147	2.187609	2.208049	2.104372
TTLL5	1566101_at	2.586391	2.682738	2.515623	2.530789	2.569588	2.51678	2.542021	2.550791	2.536707	2.528817	2.53855	2.548289	2.389074
TTLL5	1566102_at	2.135936	2.122328	2.110197	2.109429	2.122934	2.11407	2.123511	2.113246	2.119252	2.110806	2.101499	2.107778	2.119252
MFN2	1566105_at	2.627237	2.646496	2.535533	2.588742	2.641945	2.595558	2.606474	2.607163	2.598225	2.561705	2.591096	2.601232	2.409619
MYNN	1566108_at	2.4849	2.447532	2.400682	2.430096	2.468692	2.418856	2.435969	2.447103	2.432257	2.420309	2.432793	2.449642	2.32783
MYNN	1566109_at	2.47839	2.445585	2.424404	2.436712	2.462531	2.436133	2.441401	2.456303	2.436693	2.433499	2.442466	2.448507	2.299199
LOC731495	1566110_at	1.35488	1.348427	1.328985	1.333777	1.340149	1.330853	1.346053	1.335085	1.340934	1.329692	1.327981	1.327944	1.34598
FABP6	1566123_at	1.888149	1.876064	1.853273	1.859375	1.875241	1.858562	1.869891	1.872263	1.861077	1.855322	1.853688	1.866309	1.8125
FABP6	1566124_a_at	2.375915	2.351742	2.340184	2.336059	2.354846	2.318851	2.348702	2.351827	2.339544	2.337121	2.340711	2.389461	2.255834
LIMS1	1566127_at	2.586812	2.550402	2.513425	2.525573	2.570486	2.504433	2.534914	2.545784	2.531197	2.521838	2.53024	2.552796	2.439175
LIMS1	1566129_at	3.888681	3.753517	3.68722	3.869819	4.048758	3.720319	3.672772	4.032254	3.806224	3.815856	3.956393	3.899514	3.310568
CARHSP1	1566134_at	2.409842	2.390626	2.371148	2.375137	2.381592	2.379151	2.366197	2.375483	2.37475	2.375098	2.368627	2.372819	2.274422
CARHSP1	1566135_at	2.252424	2.243374	2.549315	2.23824	2.256807	2.767235	2.200386	2.618328	2.336523	2.13651	2.783719	2.229725	1.948546
HOPX	1566139_at	1.990619	1.974955	1.953217	1.959205	1.97916	1.957024	1.968471	1.972189	1.967393	1.954867	1.957336	1.971886	1.901527
HOPX	1566140_at	3.810259	3.585862	3.402438	3.558832	3.692737	3.550979	3.569132	3.578922	3.607865	3.546292	3.606111	3.671568	3.052603
LOC644450	1566145_s_at	2.731156	2.672038	2.600218	2.621442	2.794444	2.660686	2.647874	2.659863	2.645797	2.600514	2.644383	2.660374	2.466238
LOC375010	1566147_a_at	2.098884	2.074206	2.047087	2.058479	2.086455	2.064552	2.073107	2.07163	2.061057	2.060638	2.060507	2.097884	1.990807
CALML4	1566149_at	2.332215	2.283065	2.264688	2.285227	2.304191	2.284454	2.291206	2.300242	2.287872	2.279854	2.288999	2.339209	2.180198
CALML4	1566150_at	2.836797	2.868547	2.698295	2.762661	2.833735	2.72607	2.771041	2.78469	2.76548	2.745906	2.776641	2.799381	2.568276
LOC729986	1566168_at	2.291801	2.275088	2.253884	2.254142	2.285865	2.25249	2.26322	2.269773	2.259159	2.253254	2.251877	2.26835	2.15717
RFFL	1566171_at	2.771073	2.759328	2.650553	2.847387	2.757882	2.741326	2.759394	3.189657	2.748765	2.742372	2.802584	2.756041	2.696692
SUZ12	1566190_at	2.593648	2.53309	2.512437	2.531276	2.697801	2.516327	2.525211	2.548323	2.535574	2.511036	2.537802	2.556398	2.376811
SUZ12	1566191_at	5.767287	5.940756	5.589769	5.650315	5.71447	5.585596	6.52671	6.345313	6.372459	6.400663	6.372072	6.351456	5.926546
TCEA1	1566207_at	3.830957	4.114815	3.377431	3.824399	3.981085	3.784243	3.800451	4.251265	3.782563	3.620569	4.065944	4.062626	3.359795
TCEA1	1566208_at	2.370215	2.343171	2.308975	2.32021	2.352547	2.317646	2.328697	2.340453	2.324965	2.317389	2.325661	2.334349	2.231611
KRTAP5-10	1566218_at	2.180515	2.173482	2.153264	2.157545	2.172561	2.161168	2.171631	2.168127	2.156937	2.149813	2.153074	2.15931	2.128207
LOC338651	1566219_at	2.46314	2.436434	2.404003	2.418956	2.447331	2.413464	2.430585	2.437024	2.425343	2.414509	2.425941	2.442716	2.350196
DGCR12	1566235_at	2.201549	2.179554	2.157887	2.162413	2.184586	2.174593	2.168131	2.178923	2.16355	2.157157	2.160029	2.166926	2.078583
DGCR12	1566236_at	2.363454	2.351137	2.330806	2.326992	2.35758	2.327161	2.337504	2.349687	2.333342	2.326246	2.328039	2.486026	2.166514
SH3GLP3	1566251_at	3.621715	3.332482	3.219511	3.375085	3.507578	3.367994	3.386112	3.346547	3.386083	3.308433	3.592547	3.499877	3.074926
SH3GLP1	1566253_at	2.974454	2.933407	2.837828	2.900018	3.01025	2.857346	2.914383	2.932182	2.91934	2.899155	2.932736	2.970068	2.791271

LOC144874	1566256_s_at	2.730184	2.658845	3.115922	3.147734	2.688903	2.622303	2.662344	2.673127	2.667451	2.638087	2.669547	2.69488	2.47445
LOC144874	1566257_at	4.139191	4.123745	6.127821	6.379522	4.072037	4.427028	4.984975	4.269969	4.988637	4.632989	4.649078	4.077205	6.117113
GARNL1	1566269_at	2.684878	2.643627	2.62443	2.613438	2.669818	2.589392	2.626013	2.635808	2.629761	2.620492	2.629527	2.646076	2.581886
GARNL1	1566271_x_at	2.908196	2.880847	2.888973	2.871313	3.043689	2.885341	2.88656	2.887347	2.87741	2.859597	2.935196	2.980791	2.793031
GARNL1	1566272_at	2.947804	2.867716	2.827234	2.849473	2.939358	2.850871	2.857079	2.864613	2.852055	2.825882	2.865146	2.89795	2.736262
OR5E1P	1566277_at	2.049798	2.030012	2.008724	2.013781	2.033271	2.020178	2.024448	2.026199	2.016132	2.015633	2.013973	2.02402	1.965033
OR2M4	1566288_at	2.702617	2.671319	2.63032	2.651194	2.685205	2.641739	2.660094	2.67157	2.656679	2.645073	2.657399	2.700184	2.540728
OR2M4	1566289_at	1.965348	1.9505	1.930178	1.932459	1.95125	1.930863	1.944895	1.947368	1.933995	1.92921	1.931365	1.942183	1.879966
PPP1R11	1566301_at	2.832863	2.805336	2.764609	2.782184	2.81595	2.774269	2.798961	2.828887	2.798807	2.810517	2.802548	2.836055	2.644873
PPP1R11	1566302_at	2.807629	2.763027	2.751468	2.754581	2.797412	2.7451	2.752946	2.74926	2.76194	2.727225	2.749698	2.775828	2.520732
PPP1R11	1566303_s_at	5.22119	5.215066	6.528575	5.827879	4.391035	2.771499	5.387463	3.033906	5.529089	6.087137	3.023109	3.614844	6.837549
MAF	1566323_at	3.095331	2.93632	2.866753	2.917371	3.195271	2.932814	2.935712	2.942843	2.952609	2.916246	3.01256	3.006467	2.627771
MAF	1566324_a_at	5.694502	5.059853	5.394407	5.830734	5.339351	5.568883	4.635603	4.668051	4.16353	3.938325	4.463551	4.843966	4.537293
SNORD8	1566339_at	3.002561	2.882321	2.767779	2.862631	2.90984	2.837866	2.871148	2.857643	2.862978	2.820665	2.866844	2.894178	2.571799
SNORD8	1566340_at	3.125039	3.076437	3.062363	3.069693	3.121655	3.044115	3.078975	3.090431	3.134109	3.045831	2.980758	3.088079	3.01508
SOD2	1566342_at	10.60035	10.91132	9.737823	10.5982	11.14983	11.53372	7.144959	9.056399	7.534339	7.441969	8.199491	8.483531	7.674662
DNTT	1566362_at	3.049305	2.975577	2.910071	2.95555	3.05196	2.948027	2.942244	2.975987	2.960126	2.91992	2.962755	2.987871	2.659888
DNTT	1566363_at	2.936169	2.995479	2.886563	2.770506	3.036529	2.985937	2.848829	4.354913	2.903483	3.198777	3.026758	3.758343	2.681477
SNORA68	1566402_at	1.758842	1.747493	1.727332	1.732196	1.742824	1.72949	1.741772	1.743578	1.737617	1.730779	1.82908	1.737061	1.700491
SNORA68	1566403_at	2.427027	2.418515	2.398735	2.403287	2.413517	2.345049	2.36638	2.417845	2.412642	2.394287	2.402253	2.409354	2.36246
PLD4	1566433_at	2.021997	2.006987	1.964312	1.982663	2.006471	1.987844	1.99459	2.003748	1.990108	1.983433	1.991654	1.998443	1.929249
LOC283475	1566437_at	2.686128	2.58447	2.562176	2.589527	2.644037	2.570278	2.585719	2.59464	2.585723	2.571074	2.585805	2.61179	2.321746
LOC283475	1566438_at	2.675054	2.646636	2.595183	2.622666	2.658302	2.62346	2.627557	2.637289	2.630606	2.609747	2.62671	2.646186	2.503875
CD6	1566447_at	2.210206	2.327422	2.146518	2.166587	2.286351	2.175994	2.178292	2.183412	2.170107	2.162097	2.170841	2.175216	2.066012
CD6	1566448_at	2.263378	2.230381	2.194019	2.214502	2.250362	2.220627	2.232098	2.233067	2.22544	2.219677	2.219438	2.272081	2.207347
LRRC40	1566449_at	2.304746	2.267501	2.258181	2.256805	2.305088	2.248714	2.264971	2.273277	2.260796	2.25661	2.257775	2.277079	2.192832
LOC729988	1566456_at	2.399156	2.367601	2.346398	2.352212	2.366515	2.336422	2.362601	2.36753	2.36237	2.346875	2.352118	2.363733	2.28861
LOC729988	1566457_at	2.082312	2.064313	2.038781	2.045529	2.065113	2.045195	2.059417	2.061931	2.04796	2.040489	2.04619	2.057381	1.99439
LOC728987	1566464_at	2.562486	2.52096	2.469629	2.504044	2.555526	2.501531	2.514619	2.525576	2.510649	2.493559	2.508125	2.527126	2.358096
LOC728987	1566465_at	4.917163	4.830965	3.966432	3.906645	5.114242	3.743726	3.601106	3.665252	3.408601	3.476402	3.597131	3.605476	2.835459
LOC647305	1566471_at	2.632553	2.621597	2.600965	2.606853	2.617124	2.613271	2.622626	2.613702	2.61763	2.603986	2.605424	2.613757	2.432169
RETSAT	1566472_s_at	5.71566	5.419284	6.604704	5.885835	4.894794	4.921849	4.985955	3.042706	4.936285	5.219748	2.986443	2.86442	3.230984
LOC647305	1566473_a_at	3.623687	3.514892	3.733598	3.4945	3.62119	3.5042	3.510097	3.489896	3.403513	3.207783	3.491561	3.50309	3.011142
FLJ35848	1566478_at	2.324697	2.293655	2.257632	2.275654	2.314155	2.279636	2.285839	2.294497	2.281958	2.269092	2.286048	2.289882	2.201129
FLJ35848	1566480_x_at	6.750772	6.644449	5.412715	6.047725	6.853557	7.08318	4.738944	6.095485	5.030818	4.915138	6.556253	5.957398	4.70751
LOC283874	1566505_at	2.270309	2.243051	2.208228	2.223672	2.253991	2.225198	2.232212	2.243953	2.227252	2.218137	2.227321	2.236979	2.132485
FBXO9	1566507_a_at	5.128061	4.70176	4.684929	4.654988	4.979713	4.701382	4.957826	5.202594	5.29657	4.93297	5.310857	5.56015	4.443202
FBXO9	1566509_s_at	8.886838	8.578618	8.963794	8.90834	8.563916	8.522127	10.0528	9.181215	10.14384	9.911025	9.052511	9.511005	8.485536

FBXO9	1566511_at	2.872253	2.84322	2.802574	2.807181	2.898419	2.862479	2.828358	2.819614	2.824348	2.810438	2.830928	2.836638	2.613921
LOC200169	1566512_at	1.618939	1.610667	1.592511	1.595998	1.606439	1.598764	1.608718	1.607475	1.603645	1.598574	1.593528	1.600084	1.567718
LOC200169	1566513_a_at	2.179972	2.162114	2.132393	2.144458	2.166456	2.144475	2.150637	2.160831	2.147081	2.138121	2.143976	2.154986	2.635377
CWF19L2	1566514_at	2.014992	1.997153	1.957445	1.978863	1.995168	1.979494	1.991503	1.996069	1.988325	1.981094	1.979682	1.990106	1.953287
CWF19L2	1566515_at	3.532804	3.414653	3.292307	3.387007	3.570508	3.311785	3.406888	3.351267	3.403155	3.327966	3.412273	3.510599	2.897696
LOC283688	1566526_at	2.586753	2.571084	2.529295	2.546982	2.582498	2.5495	2.561342	2.567708	2.55515	2.540577	2.55307	2.561854	2.462091
FLJ90757	1566555_at	1.847968	1.837917	1.821558	1.826003	1.838361	1.825085	1.835692	1.836228	1.826416	1.844043	1.818732	1.829928	1.78091
FLJ90757	1566557_at	7.032988	6.898022	5.602799	6.244954	7.511457	7.901843	6.214056	7.203607	6.298877	6.305115	8.186801	7.704704	6.679436
FLJ90757	1566558_x_at	5.90784	5.798267	4.552417	5.255771	6.265626	6.697109	4.890855	5.85621	4.965266	4.7795	6.701708	6.242108	5.594865
RPUSD3	1566602_at	2.045586	2.02211	2.000255	2.004975	2.019421	2.004283	2.016879	2.02169	2.01325	2.006968	2.008229	2.01665	1.949742
RPUSD3	1566603_s_at	3.875353	3.580223	3.502969	3.750489	3.870829	3.593705	4.021412	4.011341	4.173653	4.493091	3.818827	3.86269	4.227014
TEX9	1566605_at	2.607355	2.572432	2.521691	2.551426	2.590863	2.53543	2.570117	2.574005	2.560281	2.551594	2.563744	2.580916	2.434207
TEX9	1566606_a_at	3.874054	3.739848	3.591543	3.69599	3.837182	3.688842	3.74663	3.752857	3.741257	3.714846	3.807133	3.842515	3.299471
DKFZP547J	1566623_at	2.210539	2.196466	2.177657	2.179181	2.203426	2.178716	2.181997	2.192966	2.181167	2.172863	2.177223	2.186907	2.093562
DKFZP547J	1566624_at	3.1556	2.93619	2.923634	2.975427	3.282116	2.925195	3.001127	3.007929	3.004215	2.956397	3.100281	3.182482	3.305551
PPP2R3B	1566630_at	3.105585	3.306451	3.016736	3.075853	3.089318	3.062318	3.0782	3.086224	3.071584	3.063182	3.06995	3.098211	2.954447
LOC149086	1566646_at	2.218918	2.213077	2.175734	2.181533	2.198522	2.176508	2.192888	2.195244	2.188082	2.177798	2.175025	2.189898	2.130416
LOC149086	1566647_s_at	2.347974	3.015027	2.308919	2.325408	2.336325	2.315381	2.296102	2.318585	2.302022	2.272383	2.309289	2.314248	2.134837
FLJ40176	1566664_at	2.323874	2.297959	2.26811	2.280376	2.306217	2.285862	2.288339	2.296701	2.283366	2.275632	2.283705	2.29141	2.196047
FLJ40176	1566665_at	2.573556	2.517938	2.462061	2.506192	2.55895	2.50225	2.516467	2.522687	2.511724	2.492575	2.510118	2.527213	2.359756
PDXK	1566670_at	3.226788	3.075613	2.985291	3.043383	3.087721	3.514102	2.877195	3.109022	2.981365	2.926886	3.048668	3.039335	2.790091
PDXK	1566671_a_at	2.931918	2.888736	2.525306	2.544423	2.644558	2.63678	3.267103	4.344253	2.825054	2.635236	3.528695	3.603269	2.427958
MMP2	1566677_at	2.729961	2.679254	2.634529	2.664986	2.735545	2.66622	2.676621	2.687547	2.669828	2.656687	2.67616	2.688295	2.474291
MMP2	1566678_at	2.246845	2.233021	2.207686	2.202532	2.241257	2.174087	2.216903	2.213901	2.216094	2.190134	2.219004	2.226628	2.149479
VRK3	1566700_at	2.168972	2.157753	2.136723	2.140472	2.156774	2.133284	2.153404	2.156532	2.142998	2.140303	2.137671	2.175554	2.100221
VRK3	1566701_at	2.835855	2.821146	2.80044	2.786899	2.820794	2.957498	2.815818	2.817577	2.800567	2.799072	2.798934	2.812883	2.563924
LOC376693	1566720_at	3.168723	3.210314	2.767328	2.824029	3.361938	2.836061	2.763417	2.849218	2.838996	2.820943	2.834474	3.154959	2.691516
SVEP1	1566721_at	2.381873	2.316785	2.301005	2.29965	2.368526	2.298512	2.313669	2.327346	2.31242	2.300478	2.313106	2.358025	2.199759
SVEP1	1566722_a_at	3.450119	3.358021	3.288713	3.32708	3.440379	3.528508	3.362266	3.353194	3.35677	3.331314	3.379729	3.405743	2.910894
LOC283454	1566734_at	2.973934	2.901159	2.80206	2.881557	2.954122	2.777722	2.878812	2.899625	2.887511	2.87711	2.919672	2.947856	2.75345
hCG_20365	1566735_at	2.334246	2.283925	2.266782	2.267618	2.295901	2.256598	2.274123	2.278706	2.276976	2.265919	2.269271	2.27754	2.164545
hCG_20365	1566737_at	2.399384	2.384111	2.348308	2.372812	2.386862	2.357644	2.370284	2.384867	2.37388	2.36502	2.370275	2.378292	2.335679
PLCE1	1566739_at	2.852451	2.823879	2.774446	2.801559	2.849304	2.793747	2.8184	2.830539	2.81488	2.8026	2.811755	2.869853	2.674054
PLCE1	1566740_at	3.067799	2.987631	2.945915	2.967971	3.183992	2.963771	2.966908	2.978488	2.897374	2.809163	3.001142	2.974073	2.616497
FLJ34208	1566760_at	2.521245	2.493481	2.472073	2.530026	2.515022	2.475868	2.488253	2.49279	2.485311	2.471713	2.478598	2.493308	2.40658
FLJ34208	1566761_a_at	2.537532	2.405009	2.387254	2.390837	2.418624	2.391149	2.393575	2.405555	2.390825	2.388585	2.412566	2.399067	2.263401
DNAH1	1566775_at	2.634468	2.608472	2.573909	2.586069	2.616786	2.582412	2.597764	2.607468	2.592737	2.585904	2.588852	2.611663	2.432649
DNAH1	1566776_at	2.07282	2.060331	2.038786	2.044036	2.090128	2.041317	2.055358	2.058351	2.047763	2.044087	2.048932	2.057275	2.006518

LOC728806	1566784_at	2.500346	2.464884	2.42961	2.445262	2.474917	2.441755	2.455703	2.466935	2.451567	2.440445	2.45104	2.460921	2.350473
LOC728806	1566785_x_at	6.641524	6.954595	5.343282	5.912329	7.249086	7.750109	6.33608	6.7759	6.155056	6.357002	7.088819	7.752667	6.088234
FGF22	1566814_at	1.993421	1.976361	1.955199	1.962064	1.974068	1.959128	1.974985	1.974978	1.96994	1.958924	1.957489	1.963154	1.931012
FGF22	1566816_at	1.922651	1.914418	1.895373	1.900147	1.911162	1.897272	1.91415	1.913337	1.908947	1.90175	1.895674	1.905638	1.842225
LOC440028	1566830_at	1.63128	1.624035	1.604809	1.608165	1.614622	1.608753	1.622169	1.626134	1.619587	1.621536	1.600368	1.620575	1.609753
LOC283104	1566831_at	2.154603	2.149985	2.130744	2.131895	2.157078	2.137539	2.135079	2.141188	2.130822	2.124625	2.132667	2.138771	2.053289
TOP1P2	1566832_at	2.460217	2.434821	2.404831	2.420308	2.449393	2.421525	2.427877	2.438444	2.42459	2.413996	2.42126	2.434115	2.358577
TOP1P2	1566833_x_at	2.451226	2.434322	2.412897	2.419042	2.45131	2.416641	2.429723	2.433051	2.420144	2.414039	2.416386	2.429834	2.354673
LOC283674	1566839_at	1.903419	1.923401	1.877264	1.902754	1.892523	1.879383	1.890568	1.884047	1.875917	1.877856	1.870091	1.883154	1.788461
LOC283674	1566840_at	2.211089	2.183447	2.149425	2.162817	2.198565	2.157783	2.172777	2.182787	2.167026	2.159351	2.165491	2.17633	2.081921
PER4	1566843_at	2.696847	2.637405	2.613391	2.620785	2.712523	2.590125	2.63765	2.651568	2.637324	2.616202	2.637904	2.668553	2.453581
PER4	1566844_at	2.155995	2.135314	2.107468	2.116166	2.138624	2.11378	2.123552	2.132317	2.118856	2.111119	2.117416	2.12809	2.041164
TRIM42	1566851_at	1.893138	1.884608	1.863634	1.870906	1.882871	1.871116	1.878276	1.880069	1.873937	1.869233	1.864614	1.874345	1.81955
TRIM42	1566852_at	2.547054	2.500994	2.455233	2.477377	2.591862	2.457733	2.50034	2.499449	2.497893	2.463852	2.506866	2.54405	2.228207
LOC145663	1566860_at	3.088611	4.771077	3.081948	3.741523	3.419885	2.881644	2.814435	2.819067	2.817724	2.825985	2.899623	2.842001	2.604614
LOC145663	1566861_at	2.026699	2.010307	1.989679	1.994245	2.013022	1.995105	2.004387	2.008754	1.996679	1.988783	1.993561	2.005035	1.934682
C7orf38	1566865_at	3.044163	2.988482	2.822704	2.977414	3.220746	2.817011	2.941998	3.182563	2.927607	3.025425	3.07889	3.184906	2.726891
C17orf84	1566884_at	2.701765	2.692559	2.675396	2.680037	2.692271	2.679761	2.686864	2.689581	2.676666	2.678502	2.675137	2.698657	2.579422
C17orf84	1566885_at	2.148942	2.136583	2.109623	2.12053	2.132846	2.118885	2.134351	2.135228	2.119757	2.123427	2.120198	2.127061	2.115309
THADA	1566888_at	2.807597	2.762705	2.69626	2.739658	2.828826	2.715386	2.755766	2.760979	2.731907	2.727588	2.757239	2.755657	2.623597
THADA	1566889_at	2.949025	2.919846	2.879655	2.892406	2.92897	2.890463	2.916742	2.91719	2.907518	2.899516	2.905014	2.927554	2.887872
UNQ1887	1566896_at	2.827348	2.767405	2.689049	2.740178	2.815547	2.754481	2.756082	2.769026	2.759134	2.728807	2.764666	2.78784	2.498341
UNQ1887	1566897_at	1.914976	1.900341	1.881692	1.88194	1.901563	1.897154	1.893146	1.897615	1.886638	1.883145	1.888285	1.924324	1.851093
HSSUCCDH	1566898_at	2.325366	2.293189	2.26423	2.27769	2.310216	2.269509	2.287152	2.297905	2.282549	2.273203	2.283603	2.301029	2.175682
HSSUCCDH	1566899_at	2.564587	2.523093	2.456621	2.50527	2.5606	2.503365	2.509903	2.5233	2.506736	2.485981	2.508089	2.526529	2.335315
LOC441476	1566908_at	2.494274	2.462402	2.436403	2.449551	2.476692	2.444669	2.457892	2.464511	2.453138	2.45676	2.453872	2.463052	2.355339
HPYR1	1566916_at	2.684657	2.628662	2.600495	2.620428	2.674077	2.641535	2.632583	2.646475	2.628002	2.607334	2.62845	2.650111	2.44684
HPYR1	1566917_at	1.941239	1.929155	1.90734	1.910035	1.927471	1.909218	1.92272	1.925706	1.913292	1.91369	1.910579	1.917402	1.859418
C21orf104	1566926_at	2.352047	2.324678	2.350992	2.312688	2.40062	2.296079	2.314006	2.324975	2.309773	2.298655	2.310932	2.319538	2.214744
C21orf104	1566927_at	2.48542	2.476122	2.460908	2.464289	2.473841	2.464549	2.4614	2.473604	2.467848	2.460888	2.454747	2.48213	2.432934
TFB2M	1566930_at	3.281754	3.117719	3.044965	3.135271	3.309094	3.081356	3.134905	3.142046	3.155662	3.108829	3.194124	3.237327	2.784735
TFB2M	1566931_at	2.601158	2.590923	2.553377	2.573226	2.591172	2.583794	2.588606	2.591082	2.576883	2.576325	2.570145	2.584938	2.481393
TFB2M	1566932_x_at	3.089093	3.059518	3.012028	3.035061	3.365991	3.086259	3.053772	3.060685	3.04406	3.03695	3.045528	3.054905	2.684092
TYRO3P	1566934_at	2.13195	2.118852	2.099764	2.103145	2.119042	2.102348	2.1138	2.116037	2.104629	2.099729	2.097447	2.109715	2.059497
TYRO3P	1566935_at	2.316149	2.287467	2.250151	2.271519	2.287121	2.275313	2.28269	2.287695	2.272307	2.26084	2.271244	2.287307	2.168533
OR7E104P	1566956_at	1.204538	1.195841	1.177847	1.181541	1.188901	1.184924	1.196319	1.1854	1.189074	1.182254	1.17778	1.189577	1.190007
OR7E104P	1566957_at	2.752264	2.734255	2.709796	2.707381	2.738757	3.010049	2.722341	2.732603	2.717361	2.710025	2.717844	2.726911	2.481008
LOC651250	1566984_at	2.566088	2.499813	2.46811	2.490621	2.589902	2.463091	2.506888	2.511872	2.499547	2.488132	2.499086	2.53193	2.302561

LOC729074	1566986_at	1.534178	1.526442	1.508975	1.511113	1.521507	1.508778	1.524495	1.521261	1.519854	1.51052	1.503193	1.513844	1.525756
LOC729074	1566987_s_at	2.866619	2.840551	2.825451	2.830194	2.889999	2.81775	2.834515	2.821088	2.83144	2.818644	2.822502	2.823356	2.927468
ARID1B	1566989_at	3.680594	3.683078	3.660765	3.66141	3.664652	3.659224	3.679418	3.678073	3.682662	3.666352	4.247795	3.672508	3.629226
ARID1B	1566990_x_at	4.953133	4.950559	4.935016	4.727459	4.794081	4.833244	5.006748	5.437799	5.087099	5.265255	5.505073	4.940999	4.883113
ARID1B	1566991_at	2.254351	2.236452	2.213445	2.218429	2.321732	2.217589	2.224907	2.232045	2.221198	2.212315	2.215997	2.228463	2.160866
NFE2L2	1567013_at	5.278187	4.891525	4.529356	5.034804	5.653262	6.537476	3.313123	3.828097	2.421986	3.275862	4.253157	3.998794	3.04422
NFE2L2	1567014_s_at	2.890991	3.492183	3.612073	3.479787	3.044521	3.336722	4.692361	3.68144	3.540416	3.594005	3.19873	3.48361	6.636537
NFE2L2	1567015_at	2.506414	2.478251	2.400061	2.460036	2.481519	2.4589	2.476122	2.477227	2.462992	2.460492	2.461576	2.498405	2.436349
OR5AK4P	1567022_at	3.4922	3.231738	3.260987	3.2624	4.315823	3.133248	3.256419	3.262004	3.297181	3.237261	3.346548	3.401076	2.870396
OR5AK4P	1567023_at	2.306893	2.28588	2.230616	2.267939	2.296573	2.251009	2.279026	2.292485	2.337188	2.256713	2.265928	2.310617	2.134121
SH3GLP2	1567027_at	1.608917	1.600063	1.585528	1.58552	1.595239	1.58629	1.60144	1.601492	1.596262	1.592792	1.586834	1.591478	1.567253
SH3GLP2	1567028_s_at	1.691635	1.673883	1.655531	1.658335	1.909831	1.787608	1.669921	1.671876	1.665768	1.652786	1.657367	1.663673	1.626611
SH3GLP2	1567030_at	2.160785	2.139312	2.100516	2.12338	2.140846	2.118232	2.129303	2.136187	2.123689	2.116709	2.122762	2.12921	2.045042
ZNF160	1567031_at	2.185061	2.158397	2.121887	2.138946	2.171796	2.138157	2.147823	2.154474	2.142499	2.131336	2.13888	2.152264	2.042314
ZNF160	1567032_s_at	2.876236	2.801049	2.779751	2.783772	2.846404	2.777866	3.2756	2.779599	3.197714	2.940202	2.802296	2.828986	4.490899
C20orf181	1567035_at	1.728045	1.714923	1.701143	1.704461	1.713476	1.701657	1.71935	1.714814	1.71679	1.707154	1.700494	1.706474	1.710478
C20orf181	1567036_at	2.725732	2.712342	2.692037	2.696395	2.711384	2.694852	2.704087	2.70833	2.696921	2.691239	2.689664	2.706753	2.637605
OR1C1	1567054_at	3.318232	3.219192	3.153421	3.214744	3.375012	3.344803	3.197241	3.234452	3.24003	3.214227	3.242722	3.276167	2.995518
OR1C1	1567055_at	1.914112	1.901677	1.884288	1.884457	1.900798	1.883578	1.895325	1.899467	1.886573	1.879966	1.882585	1.893046	1.835643
OR8G2	1567056_at	3.305456	3.213907	3.118437	3.202654	3.370084	3.09041	3.199365	3.209641	3.211462	3.167455	3.217212	3.26957	2.900719
OR8G2	1567058_at	2.285481	2.262951	2.237597	2.244414	2.269506	2.250154	2.251369	2.26318	2.247201	2.240461	2.248874	2.256699	2.166884
OR8G1	1567060_at	1.87849	1.856952	1.828381	1.838569	1.869047	1.844958	1.845942	1.852326	1.841664	1.836256	1.836845	1.846766	1.759872
OR1Q1	1567064_at	2.600977	2.548721	2.502267	2.538584	2.606156	2.518988	2.547693	2.558003	2.544046	2.529052	2.544556	2.565428	2.387076
OR1Q1	1567065_at	2.226007	2.220031	2.201061	2.20428	2.21592	2.241488	2.216698	2.218701	2.204208	2.206106	2.20336	2.213893	2.122373
OR4D1	1567068_at	2.885056	2.867075	2.800122	2.848611	2.948283	2.859706	2.84064	2.871321	2.846015	2.83309	2.867778	2.906914	2.659948
OR4D1	1567069_at	2.036391	2.02714	2.005266	2.013185	2.025478	2.014899	2.026033	2.026334	2.017979	2.01313	2.010809	2.01873	1.981899
CLN6	1567079_at	3.053749	3.044738	2.972206	3.031896	3.145144	3.075417	3.042907	3.053354	3.039969	3.009056	3.035994	3.313365	2.686975
CLN6	1567080_s_at	2.893103	2.842412	2.825398	2.824044	2.683754	2.814843	4.021677	3.19544	4.29297	4.530713	2.901362	3.255687	2.823483
CLN6	1567081_x_at	2.761974	2.757325	2.735763	2.725139	2.803537	2.740234	2.740461	3.010664	2.737836	2.817446	2.737106	2.79742	2.577939
TPM4	1567107_s_at	3.900654	3.830213	3.6758	3.69342	3.964339	4.106396	4.954968	4.17589	4.292856	4.18593	5.632822	4.38518	7.449481
PNN	1567213_at	3.666508	3.725577	3.757903	3.82846	3.540939	3.711555	3.865828	4.61754	4.110134	3.767252	3.703842	3.717053	3.678842
PNN	1567214_a_at	10.04555	10.04274	9.882948	9.962124	9.7176	9.467783	10.4791	10.30866	10.39559	10.46339	10.0947	10.14133	10.60837
ELOVL5	1567222_x_at	2.607915	2.569395	2.546147	2.54648	2.578352	2.527548	2.561648	2.561635	2.557128	2.544623	2.545864	2.553363	2.489336
HMGA2	1567223_at	2.550392	2.484774	2.484748	2.480437	2.545119	2.46616	2.493931	2.510059	2.489648	2.487383	2.495097	2.600907	2.37547
HMGA2	1567224_at	2.745343	2.711324	2.625686	2.624095	2.834178	2.619273	2.696203	2.690202	2.695551	2.677728	2.706652	2.844306	5.901739
OR2L2	1567237_at	2.264395	2.241901	2.203898	2.218614	2.277157	2.22463	2.226324	2.238338	2.226939	2.216332	2.224633	2.235123	2.147112
OR2L2	1567238_at	2.526554	2.487118	2.433759	2.465595	2.524498	2.476363	2.474857	2.481121	2.475387	2.455058	2.469408	2.490524	2.327816
OR2L2	1567240_x_at	2.206071	2.185255	2.152123	2.171957	2.200232	2.159814	2.171164	2.18059	2.17546	2.170942	2.164619	2.178173	2.081503

OR2L1P	1567241_at	1.994559	1.973088	1.948497	1.955478	1.975452	1.954248	1.964173	1.970169	1.957132	1.950081	1.952565	1.961483	1.899816
OR2L1P	1567242_at	2.192869	2.17417	2.156173	2.158086	2.179021	2.151252	2.164898	2.161706	2.158013	2.150465	2.152786	2.161751	2.066048
OR5J2	1567244_at	2.984096	2.953676	2.851357	2.938493	2.967571	3.014079	2.943054	2.955149	2.939625	2.93369	2.931943	2.948894	2.830224
OR5J2	1567245_at	2.07454	2.056418	2.032417	2.038594	2.057493	2.041556	2.048698	2.052064	2.040344	2.032755	2.037851	2.049203	1.976127
OR5H1	1567246_at	3.562298	3.31963	3.239433	3.395943	3.784709	3.315588	3.36286	3.408317	3.412966	3.336309	3.57651	3.688712	2.929397
OR5H1	1567247_at	2.492075	2.456984	2.43938	2.437336	2.473872	2.440638	2.447044	2.455236	2.440294	2.427333	2.440377	2.451456	2.328698
OR9A1P	1567248_at	3.26601	3.064625	2.943714	3.040391	3.187289	3.077852	3.044347	3.108884	3.074173	3.039364	3.111428	3.276001	2.694157
OR9A1P	1567249_at	2.13173	2.106391	2.079244	2.087932	2.108965	2.088993	2.099938	2.10444	2.095635	2.082271	2.087762	2.099589	2.015768
OR10A3	1567250_at	3.343163	3.113508	3.312629	3.278168	3.437971	3.155403	3.321349	3.326676	3.314738	3.273812	3.314581	3.59867	3.110578
OR10A3	1567251_at	2.259669	2.233075	2.212232	2.213948	2.243835	2.218509	2.227036	2.23047	2.217604	2.21191	2.213292	2.22414	2.15174
OR10D3P	1567252_at	2.948789	2.904615	2.858146	2.861335	2.984028	2.884483	2.872636	2.901658	2.87986	2.88701	2.943998	2.972566	2.749494
OR10D3P	1567253_at	1.912428	1.905879	1.883505	1.888951	1.902793	1.890992	1.895811	1.899876	1.886192	1.886924	1.883217	1.894107	1.83602
OR10D1P	1567254_at	2.656257	2.629275	2.593921	2.611977	2.688682	2.601159	2.617533	2.63413	2.620536	2.612223	2.624281	2.640847	2.506161
OR10D1P	1567255_at	2.054561	2.048779	2.028685	2.028176	2.040358	2.0285	2.041363	2.032387	2.040585	2.022769	2.022758	2.036101	2.019656
OR1J2	1567256_at	3.328301	3.267734	3.194085	3.246824	3.317216	3.255079	3.259962	3.2771	3.25548	3.19594	3.256374	3.290247	2.823069
OR1J2	1567257_at	1.964153	1.957396	1.935539	1.942397	1.950252	1.946072	1.95169	1.953376	1.947338	1.934512	1.937505	1.949256	1.89322
OR4C1P	1567271_at	2.262586	2.235785	2.204997	2.21724	2.247209	2.215896	2.224611	2.233604	2.220172	2.212792	2.216699	2.228335	2.12587
OR2K2	1567272_at	2.569647	2.536588	2.493073	2.505441	2.603357	2.512692	2.506642	2.53037	2.519268	2.51277	2.52789	2.562884	2.290804
OR2K2	1567273_at	1.88596	1.876015	1.855286	1.861495	1.874484	1.85943	1.871506	1.871785	1.862403	1.85464	1.853993	1.867839	1.811938
CTTN	1567277_at	2.648089	2.60856	2.546101	2.596179	2.640755	2.58846	2.60075	2.613736	2.596661	2.593404	2.600074	2.639576	2.440954
OR1J4	1567282_at	3.533822	3.388643	3.294744	3.381952	3.549329	3.314834	3.396159	3.439256	3.414755	3.366406	3.444906	3.552418	3.049108
OR1J4	1567284_at	2.547852	2.493963	2.441648	2.488131	2.507914	2.487559	2.495412	2.504813	2.492291	2.474957	2.487835	2.506008	2.343931
OR5L2	1567285_at	3.192266	3.073599	2.98506	3.094678	3.228816	3.085164	3.107364	3.093294	3.106757	3.081627	3.104132	3.130601	2.74907
OR5L2	1567286_at	1.690867	1.682643	1.662224	1.669497	1.680085	1.667424	1.682403	1.682439	1.669681	1.67008	1.666837	1.675632	1.632842
OR5K1	1567287_at	3.475034	3.369539	3.219037	3.316063	3.564395	3.224067	3.310303	3.308218	3.341854	3.329028	3.390603	3.479329	2.944537
OR5K1	1567288_at	2.457439	2.424179	2.375653	2.402986	2.463743	2.406254	2.4173	2.421652	2.410269	2.40461	2.40578	2.421593	2.277176
OR13C4	1567297_at	2.868626	2.839488	2.825169	2.819571	2.851725	2.927453	2.805121	2.828754	2.824086	2.812925	2.820814	2.833883	2.430979
OR13C4	1567298_at	3.500626	3.494429	3.460213	3.478228	3.49306	3.468571	3.488786	3.531786	3.478332	3.469205	3.472164	3.485275	3.037467
LOC57802	1567319_at	2.172799	2.163809	2.146465	2.151042	2.16077	2.147299	2.163811	2.164024	2.153853	2.147584	2.148887	2.303193	2.058783
LOC57802	1567320_at	1.724758	1.718723	1.69927	1.704731	1.710537	2.347657	1.714709	1.712661	1.710203	1.69909	1.696956	1.705937	1.6745
FOXD4	1567341_at	2.7549	2.881144	2.64167	2.640619	2.663803	2.64611	2.651668	2.64275	2.63334	2.636509	2.604899	2.650014	2.437024
NAV2	1567357_at	2.393014	2.383397	2.309263	2.364869	2.391713	2.370272	2.378012	2.38271	2.37323	2.364945	2.373336	2.400838	2.29451
NAV2	1567358_at	3.147415	3.115942	3.06161	3.088264	3.135742	3.101006	3.094626	3.10701	3.087243	3.054494	3.092351	3.112074	2.93979
BDNFOS	1567359_at	3.396017	3.203885	3.133454	3.24574	3.50294	3.148565	3.24051	3.231654	3.252222	3.204363	3.30404	3.36191	2.841445
BDNFOS	1567361_at	3.033596	2.934197	2.893196	2.9477	3.063342	2.924844	2.976846	3.571853	2.956979	2.944637	2.984752	3.029182	2.724839
DNAH1	1567376_at	2.840168	2.804816	2.764501	2.77936	2.820003	2.791027	2.79578	2.814077	2.792548	2.790456	2.791163	2.823004	2.553377
DNAH1	1567377_at	2.948014	2.858134	2.805979	2.847565	2.935857	2.802707	2.863876	2.86826	2.865411	2.829988	2.866625	2.917992	2.597831
DNAH1	1567378_x_at	2.59407	2.553557	2.507616	2.538925	2.58169	2.548477	2.546528	2.557436	2.54192	2.522537	2.541798	2.558333	2.381831

ZNF135	1567410_at	1.552492	1.544239	1.526651	1.525723	1.540627	1.534347	1.542927	1.543295	1.53844	1.525723	1.514266	1.517683	1.539056
PSEN1	1567440_at	1.920079	1.913156	1.893557	1.896269	1.912776	1.898986	1.910736	1.909532	1.903423	1.897948	1.902824	1.912321	1.856366
PSEN1	1567443_x_at	3.672737	3.539856	3.410518	3.530442	3.927951	3.493231	3.573543	3.560969	3.547712	3.516316	3.548731	3.590095	3.13236
RAC1	1567457_at	3.5486	3.505964	3.443562	3.413418	3.528131	3.778041	3.495563	3.508567	3.493568	3.472834	3.492065	3.513169	3.215493
RAC1	1567458_s_at	8.221634	7.775584	9.555014	7.853489	6.921267	5.565262	9.738593	7.467742	9.838356	9.444492	7.130175	7.419196	10.4497
PLXNA3	1567519_at	2.344456	2.33159	2.310606	2.305514	2.371658	2.310261	2.32577	2.327797	2.326999	2.310375	2.313073	2.326609	2.203657
SPAG10	1567539_at	2.373538	2.35834	2.316489	2.353875	2.377607	2.35515	2.363274	2.362147	2.358459	2.352684	2.347485	2.356268	2.225083
SPAG10	1567540_at	2.85456	2.76155	2.753594	2.757798	2.822525	2.754196	2.760406	2.76386	2.75731	2.731188	2.754378	2.813511	2.536342
TREML4	1567558_at	2.24535	2.230794	2.212396	2.215748	2.230132	2.217312	2.226707	2.226416	2.220161	2.21126	2.215296	2.223364	2.115987
TREML3	1567559_s_at	2.169353	2.145736	2.118947	2.127997	2.150803	2.128806	2.140839	2.145206	2.131848	2.127502	2.129394	2.138893	2.083124
ABLIM2	1567622_at	2.447872	2.700609	2.381637	2.415786	2.696012	2.514458	2.252569	2.379197	2.40594	2.238067	2.434689	2.379014	2.312566
ABLIM2	1567623_at	2.117528	2.081847	2.076684	2.083033	2.102361	2.099181	2.0903	2.096691	2.083678	2.075763	2.117329	2.088889	2.020819
TREML2P	1567624_at	1.884925	1.872809	1.852461	1.857693	1.871772	1.859886	1.869549	1.871387	1.862623	1.854234	1.855255	1.865474	1.844839
CD74	1567627_at	2.51024	2.48394	2.444806	2.472191	2.486687	2.470441	2.474208	2.485893	2.477846	2.471761	2.470459	2.479025	2.356145
CD74	1567628_at	3.590468	3.67771	5.337102	4.10618	3.229339	3.401853	2.420397	2.588046	2.603084	2.566076	2.576853	2.481088	2.511704
OR2H1	1567656_at	2.909862	2.818074	2.75272	2.82094	2.89749	2.792259	2.835771	2.840947	2.825584	2.807666	2.840077	3.103227	2.640372
OR2H1	1567657_at	2.953866	2.894748	2.841939	2.864787	2.942652	2.81976	2.898126	2.907351	2.899532	2.878601	2.951322	2.919144	2.72892
SNORA74A	1567679_at	2.453326	2.420987	2.373611	2.400239	2.437421	2.385909	2.411383	2.420319	2.41152	2.406822	2.404682	2.415827	2.27521
SNORA74A	1567681_at	2.494454	2.461076	2.402024	2.448217	2.461939	2.445946	2.458019	2.459916	2.445072	2.445481	2.440711	2.454185	2.306483
SNORA74A	1567682_x_at	2.765495	2.738994	2.653156	2.720668	2.759951	2.727189	2.716067	2.740807	2.726212	2.711147	2.787493	2.744146	2.50548
CECR9	1567686_at	1.787604	1.777823	1.738015	1.755642	1.807302	1.764966	1.767927	1.772754	1.773534	1.763682	1.763879	1.769832	1.729104
CECR9	1567687_at	2.967858	2.87668	2.883394	2.905014	2.975354	2.905773	2.915015	2.893169	2.887243	2.900867	2.907068	2.960564	2.707298
TFE3	1567704_at	2.110631	2.081824	2.054067	2.064413	2.090943	2.057826	2.071981	2.076171	2.067796	2.058045	2.069788	2.073274	1.995698
ZNF28	1567853_at	2.643762	2.589676	2.549323	2.579112	2.691453	2.563008	2.587753	2.595628	2.584383	2.569369	2.596545	2.640557	2.417474
DNAH14	1567861_at	2.956668	2.852351	2.782422	2.848122	2.93979	2.821438	2.869852	2.885012	2.871156	2.824823	2.873808	2.905236	2.603327
DNAH14	1567862_at	3.342778	3.185789	3.110019	3.25971	3.469745	3.359142	3.287349	3.320549	3.270556	3.225524	3.315086	3.416475	3.149063
DEFB114	1567878_at	2.797021	2.723478	2.665446	2.717547	2.792992	2.685626	2.730364	2.740992	2.727506	2.70454	2.728004	2.75984	2.514619
SOX4	1567906_at	1.79199	1.784758	1.766214	1.770206	1.778838	1.770414	1.78026	1.782022	1.778697	1.768538	1.763471	1.77421	1.737001
CT45-1 /// C	1567912_s_at	2.967207	2.873989	2.797114	2.857034	2.955872	2.81698	2.877564	2.879962	2.87471	2.841811	2.882948	2.922451	2.610278
CLIP1	1568012_at	2.006889	1.98837	1.968586	1.972446	2.059853	1.971754	1.985617	1.986302	1.974914	1.967175	1.970979	2.012158	1.915776
ANXA2	1568126_at	2.886525	2.818488	2.773359	2.78928	2.866	2.753255	2.80859	2.831523	2.819488	2.798723	2.818072	2.843167	2.651339
SNORA71B	1568248_x_at	2.253869	2.242203	2.21455	2.218273	2.244557	2.224024	2.236385	2.235788	2.221957	2.213195	2.225924	2.275131	2.125201
SNORA71B	1568249_at	4.697764	4.584785	4.530243	4.582676	4.823721	5.789746	4.336809	4.60032	4.585224	4.178872	4.471127	4.613061	3.616565
HMGA2	1568286_at	2.951368	2.853028	2.784765	2.8435	2.989842	2.816588	2.860152	2.866744	2.860952	2.828855	2.872902	2.921009	2.619911
HMGA2	1568287_at	2.984404	2.890114	2.846535	2.897666	2.973025	2.866661	2.909984	2.921464	2.904302	2.884561	2.948386	2.945316	2.625845
DEFB124	1568375_at	2.200514	2.18816	2.170878	2.174605	2.188521	2.172331	2.179247	2.187119	2.178226	2.171141	2.169095	2.181413	2.132212
DEFB124	1568377_x_at	2.881976	2.858678	2.794229	2.837204	2.871569	2.852983	2.870913	3.335852	2.847509	2.87657	2.845764	3.05166	2.470859
GTA	1568487_x_at	2.982365	2.927474	2.886641	2.923048	3.00739	2.902435	2.89589	2.943282	2.927684	2.911349	2.928054	2.944854	2.615644

GTA	1568488_at	2.024784	2.004573	1.980912	1.987116	2.005819	1.986194	1.998319	2.003347	1.99105	1.983236	1.989106	2.000493	1.922125
IL23A	1568513_x_at	7.969881	8.045958	7.739336	7.692519	8.003825	8.290726	8.137747	8.552553	7.959206	7.969267	7.971354	8.450647	7.379448
C6orf142	1568554_x_at	2.958156	2.874652	2.79584	2.864392	2.976354	2.837841	2.880063	2.888404	2.875783	2.851121	2.885389	2.916063	2.645452
SPP1	1568574_x_at	2.50175	2.462418	2.40955	2.446175	2.486219	2.422629	2.455918	2.466494	2.451333	2.431933	2.454732	2.463485	2.954228
ARL3	1568590_at	2.385887	2.376942	2.340643	2.357324	2.377352	2.358924	2.371265	2.384931	2.361826	2.354743	2.372331	2.369475	2.628234
TRIM69	1568592_at	4.552272	4.033592	4.245353	4.058599	4.414063	4.683095	2.346074	2.652719	2.569372	2.50917	3.169834	2.892018	4.667163
NUDT16P	1568593_a_at	2.92228	2.694848	3.817748	3.165312	2.786708	2.701567	2.709573	2.676031	2.697035	2.784093	2.668886	2.698943	2.481229
TRIM52	1568594_s_at	4.662661	4.175049	4.167329	4.096945	5.006928	4.620451	5.281501	5.094763	5.803992	5.55862	5.49728	5.701754	4.612758
TROAP	1568596_a_at	2.09993	2.124024	2.013649	2.040835	2.032542	2.090292	3.264666	3.909602	3.996766	3.878084	3.539541	4.063099	2.212472
LOC646762	1568597_at	3.750112	3.941391	3.808747	3.863182	3.761231	4.47796	5.482707	5.724558	5.568438	5.359891	5.200324	5.655234	6.058858
KAZALD1	1568598_at	2.563376	2.548037	2.500286	2.529285	2.577225	2.516165	2.540395	2.756164	2.539165	2.518314	2.535408	2.55801	3.504423
CALML4	1568600_at	2.850695	2.838826	2.81964	2.8145	2.880892	2.72867	2.909712	2.835882	3.03089	2.824566	2.860531	2.966087	3.396723
GAD2	1568601_at	1.821948	1.815691	1.793406	1.799159	1.827143	1.794558	1.812513	1.7979	1.793546	1.787531	1.797449	1.799865	1.813494
CADPS	1568603_at	3.266485	3.128024	3.103324	3.150181	3.313311	3.104798	3.163056	3.129618	3.1586	3.109833	3.181249	3.22458	6.198883
CADPS	1568604_a_at	2.384799	2.660085	2.321807	2.34868	2.371486	2.34828	2.358182	2.366454	2.348973	2.336642	2.351662	2.361316	6.888493
JRK	1568605_at	4.0322	3.916868	3.123359	3.176571	3.479309	3.463545	4.793299	5.659428	5.189087	5.265524	5.538999	5.853144	3.611309
FLJ46266	1568606_at	2.136201	2.124904	2.110289	2.108883	2.12473	2.10152	2.117788	2.126735	2.12149	2.104615	2.112682	2.121518	2.099314
FAM91A2	1568609_s_at	6.535409	6.267735	7.422557	7.780139	7.529292	7.70439	5.032067	5.159292	5.425937	5.24569	6.353456	6.088841	7.155346
GABRG2	1568612_at	2.664896	2.61835	2.571419	2.600718	2.644307	2.607685	2.602879	2.613646	2.599864	2.577293	2.598739	2.62038	4.906131
RSPH3	1568613_at	3.522364	3.800963	3.168966	3.595342	3.411409	3.642013	3.536418	3.553711	3.73485	3.677878	3.603344	3.594046	3.298283
KIAA1543	1568617_a_at	4.246218	4.264927	4.11855	4.584014	3.866439	3.690307	2.882798	3.131258	3.608077	2.758164	3.134295	3.455462	2.616055
GALNT1	1568618_a_at	8.683699	8.532686	8.790184	9.073756	8.949866	8.676874	8.597632	8.487864	8.62589	8.541927	8.776665	8.758601	9.188447
LOC162073	1568619_s_at	7.943294	7.734967	7.545464	7.658297	8.397051	8.411088	5.688656	6.414042	5.345454	5.456876	7.645915	6.83021	8.1254
CSAD	1568620_at	1.77027	1.741115	1.727165	1.748955	1.773882	1.733501	1.733589	1.735058	1.726057	1.719939	1.790987	1.734846	1.721804
SLC35E4	1568623_a_at	2.718753	2.675798	2.615489	2.657413	2.761446	2.630444	2.668274	2.678039	2.660263	2.642361	2.671437	2.711529	2.494337
SMEK2	1568627_at	2.192748	2.154043	2.097135	2.155807	2.177187	2.152464	2.172158	2.185332	2.561883	2.227807	2.154455	2.193548	2.136682
PIK3R2	1568629_s_at	6.955451	6.744182	6.003083	6.725784	6.358638	6.541767	9.124546	9.535673	9.037667	8.965561	9.251575	9.35995	7.103722
LOC339977	1568634_a_at	2.848734	2.820649	2.722837	2.809518	2.86837	2.784148	2.82159	2.831195	2.818709	2.79711	2.835162	2.887129	3.028548
INDOL1	1568638_a_at	3.20168	3.152002	8.233138	8.301018	7.232806	7.26126	2.459241	2.471247	2.50413	2.48612	2.500515	2.518379	2.203692
LOC400960	1568640_at	6.649888	6.845653	6.516049	6.605902	7.122948	7.139631	6.648602	6.901908	6.500329	6.623608	7.119384	6.823578	6.29716
ZNF208	1568644_at	2.972622	2.879044	2.811169	2.863841	2.988064	2.840663	2.871972	2.877138	2.868738	2.855034	2.895051	2.936842	2.612778
ZNF208	1568646_x_at	2.748827	2.64615	2.641025	2.638792	2.696041	2.611796	2.648805	2.660716	2.645466	2.632033	2.645257	2.675724	2.472252
LOC339804	1568658_at	7.952338	8.136873	7.589547	7.911866	9.142269	8.737887	7.023901	7.354158	6.667567	7.155132	5.835563	6.307455	6.951442
RNF103	1568665_at	2.306925	2.2717	2.238117	2.245284	2.261359	2.289682	2.252993	2.256465	2.257266	2.239818	2.248331	2.249523	2.218896
LSDP5	1568666_at	2.757402	2.749632	2.781818	2.734398	2.742016	2.73191	2.74926	2.73369	2.710743	2.735594	2.726613	2.747458	2.740948
LSDP5	1568667_s_at	3.086507	3.082793	3.425363	3.064734	3.072075	3.069216	3.07245	3.066087	3.059043	3.056342	3.048708	3.065997	2.958175
EAF2	1568672_at	2.558433	2.518283	2.473429	2.501842	2.546184	2.481425	2.511086	2.520158	2.506967	2.490243	2.506416	2.531924	2.373447
EAF2	1568673_s_at	3.350695	3.172269	3.081755	3.171985	3.510888	3.108453	3.187073	3.195451	3.207911	3.158755	3.272532	3.297069	2.792979

CHRNA10	1568675_at	2.488538	2.603455	2.431496	2.459423	2.493198	2.467546	2.477399	2.800492	2.465492	2.449189	2.460996	2.472221	2.427057
FGFR1OP	1568678_s_at	6.609504	6.722589	6.291805	6.312695	6.218417	6.041549	6.812137	6.655562	6.994155	6.62343	6.493898	6.854266	5.28175
YTHDC2	1568680_s_at	5.394026	5.135134	5.552852	5.691787	5.78664	5.254577	5.510826	5.529313	5.395559	5.126704	5.732143	5.84057	5.472951
MGC23284	1568683_at	2.722738	2.674334	2.610628	2.661694	2.764854	2.663074	2.669979	2.67645	2.67164	2.659189	2.661213	2.671813	2.471971
LOC158381	1568686_at	2.841527	2.774517	2.727543	2.76121	2.830316	2.723562	2.770337	2.776717	2.766197	2.753715	2.766914	3.004586	2.540535
LOC158381	1568687_s_at	2.695642	2.66906	2.600154	2.652114	2.68768	2.619761	2.658813	2.665014	2.654711	2.647922	2.653012	2.681168	2.465246
LOC728853	1568689_at	3.578899	3.468622	3.256815	3.46551	3.721503	3.420614	3.38169	3.483607	3.455173	3.450957	3.496817	3.778823	3.1754
LOC728853	1568690_a_at	2.217922	2.193953	2.164004	2.178345	2.225673	2.171536	2.182141	2.192723	2.18157	2.168305	2.178229	2.188121	2.093751
INTS6	1568695_s_at	2.913453	2.886566	2.884084	2.878989	2.913542	2.857131	2.883558	2.902322	2.89463	2.865621	2.894929	2.948425	2.827372
ARMETL1	1568696_at	3.589312	3.353657	3.988021	4.076724	4.012728	4.625907	3.310118	3.292322	3.306821	3.283986	3.341063	3.46684	2.753977
FLJ43080	1568698_at	2.307632	2.288005	2.263474	2.267301	2.290483	2.27267	2.282652	2.287351	2.272443	2.266252	2.266944	2.279257	2.232735
C14orf179	1568699_at	4.385259	4.361834	4.344443	4.371016	4.366489	4.350195	4.360891	4.353563	4.353146	4.348662	4.337551	4.356084	3.335711
PAAF1	1568702_a_at	4.977659	4.625951	4.448461	4.495139	4.853615	5.429904	4.586952	5.375884	4.644254	4.638442	5.185447	5.153238	4.120466
CHERP	1568704_a_at	1.829936	1.822184	1.879309	1.811702	1.819098	1.805612	1.818112	1.815112	1.809581	1.804024	1.798432	1.811785	1.819013
AVIL	1568706_s_at	6.835431	6.626403	5.752865	4.67801	6.097609	5.172339	4.579497	4.80049	4.198726	4.120179	4.002166	4.358629	3.951768
TBC1D1	1568713_a_at	2.614971	2.597669	2.569384	2.576962	2.605191	2.583253	2.593102	2.583538	2.588582	2.58238	2.577676	2.581246	2.508875
FKBP15	1568717_a_at	2.158069	2.137569	2.112086	2.121902	2.144067	2.120394	2.133028	2.139175	2.125407	2.169564	2.154507	2.121755	2.062673
C6orf85	1568718_at	1.289893	1.281004	1.264096	1.267309	1.273014	1.271129	1.280977	1.272698	1.277394	1.265728	1.259877	1.286098	1.281181
C6orf85	1568719_s_at	2.671818	2.626015	2.581182	2.623212	2.673078	2.631195	2.624882	2.638843	2.625528	2.617714	2.958872	2.634734	2.414467
ZNF506	1568720_at	3.019114	2.914629	2.823937	2.881141	2.977868	2.847211	2.892958	2.898885	2.892295	2.859438	2.896897	2.911083	2.914546
ATP9B	1568723_at	2.06552	2.057781	2.03543	2.043391	2.051365	2.04812	2.056276	2.05362	2.041829	2.026462	2.039941	2.049712	2.006809
ICMT	1568728_s_at	3.178627	3.270943	3.154342	3.090758	3.138939	3.213176	2.891673	2.893967	3.126557	3.108162	3.117376	3.093021	2.730822
C10orf76	1568733_at	2.253481	2.238345	2.496517	2.214058	2.241132	2.232138	2.223794	2.233656	2.218223	2.211633	2.219514	2.227732	2.280755
HS1BP3	1568734_a_at	2.438083	2.42857	2.406374	2.410029	2.429674	2.415579	2.420585	2.422449	2.418693	2.413856	2.409627	2.450173	2.280236
LOC285692	1568739_at	3.044182	2.976508	2.938741	2.967881	3.033625	2.941103	2.979868	2.991009	2.976537	2.960044	2.976997	3.008065	2.778203
ATP10A	1568743_at	2.586031	2.555082	2.517833	2.536545	2.569616	2.531763	2.54486	2.554472	2.543049	2.534995	2.525499	2.547284	2.50571
hCG_16547	1568745_at	2.076643	2.056114	2.015947	2.046695	2.071246	2.044034	2.048585	2.058767	2.042673	2.055912	2.038271	2.190195	1.947664
hCG_16547	1568746_a_at	3.117168	3.006599	2.93518	3.007701	3.16107	2.997178	3.018878	3.034757	3.01017	2.967514	3.067872	3.067007	2.690667
RGS13	1568751_at	3.19928	3.055279	2.98237	3.06078	3.208688	3.002875	3.064492	3.072459	3.085347	3.049582	3.115273	3.162635	2.743346
RGS13	1568752_s_at	3.616765	3.419274	3.427888	3.486065	3.725585	3.361505	3.442602	3.431284	3.495353	3.473225	3.537778	3.651685	2.91953
MYH11	1568760_at	2.46203	2.44827	2.419238	2.404932	2.452141	2.425757	2.444422	2.447597	2.434792	2.433784	2.433336	2.43998	2.43258
PDCD6	1568762_at	3.28078	3.203993	3.031032	3.184583	3.297259	3.194563	3.202404	3.203947	3.191778	3.173994	3.185802	3.206706	2.821427
PDCD6	1568763_s_at	7.607635	7.721801	7.490875	7.518576	7.521398	7.344896	7.759244	7.724291	7.592205	7.297756	6.640477	7.132111	7.016305
PDCD6	1568764_x_at	4.609662	4.464981	4.39729	4.465652	4.604127	4.764037	4.432764	4.299109	4.417438	4.08352	3.979557	4.429302	3.941424
SERPINE1	1568765_at	7.524876	7.712475	5.684276	5.587279	8.596615	8.293234	2.892365	2.980249	2.778126	2.715092	2.856373	2.884042	2.433875
BRE	1568768_s_at	2.519707	2.537831	2.513505	2.499995	2.546441	2.634919	2.511164	2.535351	2.507806	2.475648	2.504515	2.522408	2.322623
MYLK	1568770_at	2.119148	2.099798	2.078147	2.083512	2.097056	2.093677	2.094635	2.094117	2.083167	2.077637	2.078049	2.088006	2.032121
EML5	1568777_at	3.142921	3.135482	3.818797	4.002135	3.2152	3.081321	3.177923	3.15452	3.596289	3.167454	3.176225	3.277178	3.046613

ECM2	1568779_a_at	6.66408	6.221512	5.362062	5.555033	5.331432	5.170101	3.599678	3.470882	4.258576	3.459585	2.673474	3.606035	2.466863
LOC497257	1568780_at	2.665054	2.681939	2.544251	2.597858	2.757632	3.583395	4.187492	5.5966	4.142493	4.183079	5.692465	5.14491	3.651042
RP2	1568782_at	2.204528	2.181593	2.150664	2.162007	2.190922	2.158195	2.170134	2.179359	2.164699	2.157668	2.165711	2.174592	2.088176
SFRS12	1568783_at	2.653734	2.563672	2.541334	2.559261	2.636435	2.558152	2.574979	2.579715	2.56696	2.557157	2.562101	2.579357	2.521188
C6orf107	1568789_at	2.218622	2.194012	2.15084	2.179487	2.199315	2.174886	2.177609	2.193898	2.174652	2.166524	2.179189	2.205145	2.065589
SF3A1	1568796_at	2.858159	2.970653	2.7548	2.656312	2.968017	3.32102	3.390119	3.1025	3.04124	3.473537	2.959624	2.981164	2.524522
C15orf44	1568801_at	2.35574	2.329266	2.312515	2.304151	2.326132	2.29727	2.326717	2.32428	2.323503	2.315797	2.316643	2.354234	2.324801
NCOA7	1568805_at	2.937112	2.890687	2.842687	2.872471	2.925662	2.817405	2.873902	2.897231	2.875209	2.842375	2.883457	2.901525	2.610284
DDX50	1568814_at	2.485827	2.45851	2.430713	2.438833	2.468183	2.449189	2.447616	2.458804	2.448407	2.43709	2.441731	2.453667	2.392438
DDX50	1568815_a_at	5.564088	5.631084	5.625483	5.503673	5.942488	6.086531	4.939844	5.461779	4.901837	5.111837	5.458907	5.456459	4.494914
C6orf65	1568820_a_at	3.041658	2.948661	2.900849	2.943763	3.12008	2.928311	2.956768	2.961945	2.980928	2.926789	2.999691	3.053777	2.744024
TTC23	1568821_at	2.526489	2.493881	2.460461	2.471472	2.512875	2.475835	2.482642	2.492431	2.47941	2.460172	2.475903	2.497201	2.332187
GTPBP5	1568822_at	3.832793	4.101139	3.342547	3.352949	3.159745	3.145797	3.161446	3.127724	3.16194	3.150963	3.211541	3.113086	2.768911
LOC401442	1568827_at	1.999231	1.981967	1.961512	1.964701	1.989216	1.964935	1.976667	1.980764	1.96874	1.961633	1.963369	1.975644	1.909116
IRAK3	1568830_at	2.721021	2.701933	2.511106	2.681093	2.703151	3.130698	2.63517	2.698812	2.678631	2.610414	2.684968	2.673616	2.193012
CCDC90B	1568834_s_at	6.295295	6.681369	5.621713	5.485279	5.845057	5.047701	5.602323	5.622064	5.282651	5.058454	5.000908	5.015761	5.194564
CLK4	1568836_at	2.684196	2.620549	2.56818	2.601552	2.668781	2.593376	2.6544	2.63254	2.833417	2.761545	2.629834	2.644745	4.221606
WDSOF1	1568840_at	2.612941	2.574772	2.512117	2.541433	2.600406	2.528866	2.550625	2.562524	2.559051	2.536357	2.559269	2.576441	2.395046
TTLL13	1568843_at	2.207426	2.190825	2.152653	2.172748	2.197053	2.171018	2.178086	2.188	2.174491	2.167872	2.17341	2.184501	2.111351
C21orf135	1568849_at	2.048827	2.052973	2.021939	2.022857	2.03228	2.027463	2.035264	2.038461	2.032998	2.016034	2.017003	2.029908	1.997131
CCBP2	1568850_at	1.981452	1.970516	1.949515	1.953919	1.995005	1.977781	1.964141	1.970657	1.961638	1.955318	1.953999	1.959543	1.896546
NBR1	1568856_at	4.950403	4.202053	4.019599	4.152408	4.334597	3.908376	4.200307	4.164537	4.299502	4.090828	4.44316	4.212799	4.068201
NBR1	1568857_a_at	5.909242	5.912324	5.29467	4.601957	5.558282	5.472509	5.162056	5.935318	5.519962	5.271923	6.210772	5.4588	4.343401
SLC8A3	1568859_a_at	2.466929	2.45723	2.43435	2.415982	2.44505	2.435304	2.457371	2.450384	2.451589	2.438583	2.455014	2.433302	2.436192
MGC2752	1568864_at	2.270856	2.241492	2.218645	2.222834	2.27797	2.265129	2.234297	2.242016	2.22974	2.222559	2.228078	2.359954	2.13062
FNTB	1568865_at	3.213061	3.592983	3.061046	3.339405	3.357701	3.027989	3.972871	3.841601	4.089346	3.929045	3.072031	3.061353	3.301219
CYP27C1	1568868_at	2.818419	2.757437	2.686318	2.736119	2.812163	2.725346	2.739243	2.746574	2.742591	2.703677	5.256172	2.849952	6.642806
ZNF519	1568873_at	2.30565	2.282466	2.245248	2.236141	2.28939	2.217551	2.474824	2.288388	2.860508	2.696825	2.728262	2.296046	3.308412
NCOA6	1568874_at	2.584941	2.551617	2.510529	2.534214	2.568754	2.52199	2.543218	2.555311	2.540583	2.518058	2.539365	2.568262	2.417482
ACBD5	1568877_a_at	5.31601	4.264659	5.936255	6.51823	6.616705	6.335408	2.364894	2.310363	3.169011	2.3599	2.753623	2.303769	4.104535
LAMA3	1568879_a_at	2.452186	2.435193	2.415078	2.42998	2.440596	2.423399	2.442607	2.438895	2.430309	2.422254	2.425698	2.434523	2.383465
LRRC51	1568882_at	2.179463	2.141155	2.131735	2.135603	2.156683	2.158233	2.146487	2.150786	2.136103	2.123586	2.187916	2.148488	2.015635
ZRF1	1568884_at	2.872302	2.84411	2.819033	2.830757	2.89738	2.826577	2.838339	2.847746	2.833535	2.799209	2.834939	2.870965	2.749065
FANCD2	1568889_at	2.218598	2.21057	2.19079	2.19585	2.306886	2.193127	2.20855	2.20088	2.182546	2.165174	2.297144	2.600244	2.070529
FANCD2	1568891_x_at	3.124388	3.012672	3.013842	3.01077	3.187138	3.077839	3.085117	3.095186	3.047301	3.054703	3.179332	3.130037	2.72639
STXBP5L	1568896_at	2.691961	2.655625	2.617426	2.649611	2.676144	2.64552	2.650553	2.663544	2.649956	2.635763	2.64795	2.659618	2.462135
ZNF568	1568900_a_at	3.517073	3.279395	3.003181	3.257752	3.645607	3.54445	3.185415	3.251481	3.254895	3.252886	3.513003	3.585285	4.292266
LOC728196	1568906_at	2.536919	2.509378	2.495939	2.496174	2.541098	2.501309	2.503895	2.512419	2.503461	2.485571	2.5008	2.514694	2.621847

NSUN3	1568913_at	2.4215	2.389361	2.346189	2.371962	2.399318	2.369595	2.381467	2.388635	2.374236	2.365492	2.373283	2.383918	2.308425
OPN5	1568914_at	2.145662	2.127064	2.097239	2.108454	2.126896	2.115968	2.115539	2.124766	2.110687	2.105856	2.105538	2.120269	2.041322
SPIRE2	1568916_at	4.152373	3.980159	3.653274	3.916302	4.562301	4.617856	3.834897	3.962785	3.972175	3.830827	4.093724	4.232577	3.307586
SLC24A4	1568922_at	2.184656	2.174785	2.153608	2.158632	2.183506	2.158751	2.170513	2.202434	2.162796	2.15937	2.163705	2.166778	2.09283
IQUB	1568924_a_at	2.665466	2.61239	2.563171	2.600716	2.704908	3.274594	2.611749	2.634241	2.609839	2.591784	2.605902	2.644336	2.439536
MYLK2	1568925_at	4.58518	4.199459	4.122402	4.436144	4.944914	4.217808	4.32713	4.270132	4.426055	4.305144	4.691536	4.554636	3.582815
MYLK2	1568926_x_at	3.758215	3.442661	3.359701	3.53256	3.736569	3.452151	3.468336	3.450314	3.557842	3.504214	3.594945	3.643808	2.995808
EFCAB1	1568930_at	2.786623	2.752759	2.664141	2.733622	2.774669	2.724494	2.738203	2.751613	2.733567	2.731451	2.740048	2.776078	2.52
CX3CR1	1568934_at	2.784538	2.748228	2.749559	2.749669	2.776162	2.741879	2.75239	2.766457	2.748373	2.739973	2.750115	2.758622	2.529758
INPP5D	1568943_at	2.998959	2.905864	2.658413	2.768539	2.948716	2.906656	2.909784	2.918197	2.933333	2.888888	2.905735	3.476452	2.713066
PITPNC1	1568949_at	2.041061	2.037054	2.016252	2.0201	2.026987	2.015264	2.032204	2.026056	2.029627	2.01313	2.016255	2.019293	2.019039
SUHW4	1568951_at	2.775889	2.640547	2.686588	2.725539	2.757944	2.718508	2.94887	2.740469	3.15926	2.718001	2.723036	2.836293	3.876225
C16orf72	1568954_s_at	6.185305	6.00567	6.422241	5.859692	5.400828	4.784678	6.742429	5.084465	6.755155	6.772077	5.561482	5.478627	7.852366
LOC653464	1568955_at	6.789751	6.580429	6.065669	5.882504	6.840261	6.404696	6.919141	6.5039	7.376229	7.085085	7.199356	7.028292	8.199694
LOC653464	1568957_x_at	5.294368	5.170655	4.988475	4.796362	5.274685	5.206188	5.43892	5.299405	5.408678	5.608826	5.824427	5.65441	6.945851
SPN	1568964_x_at	3.780443	4.012907	3.172556	3.357734	3.785944	4.408578	6.320451	7.00213	5.97641	6.270506	6.389258	7.081105	2.897691
ADAM18	1568970_at	2.120707	2.101363	2.077696	2.083666	2.103555	2.08204	2.092453	2.100054	2.085939	2.078171	2.084284	2.095164	2.012245
C14orf135	1568971_at	2.634372	2.554942	2.505794	2.775455	2.592908	2.524887	2.555281	2.564988	2.552127	2.53333	2.549414	2.589563	2.410153
RNASET2	1568977_at	2.17209	2.165779	2.145746	2.14103	2.164596	2.523716	2.162755	2.162826	2.144253	2.148927	2.14604	2.154646	2.092062
C11orf21	1568978_s_at	2.667294	2.662234	2.598338	2.643295	2.661706	2.647785	2.851601	3.271033	2.654587	2.72858	2.855976	2.959461	2.463863
PIGT	1568986_x_at	7.009394	7.432741	6.370573	6.660422	7.096788	7.900727	6.648274	7.392426	6.60354	6.650511	7.422788	7.129092	5.988087
MGC57346	1568987_at	1.740644	1.728742	1.709846	1.714395	1.728691	1.713364	1.725144	1.745149	1.716263	1.711914	1.710186	1.726522	1.698509
PSG4	1568999_at	2.429316	2.39366	2.361999	2.380119	2.412495	2.376054	2.39687	2.398571	2.384981	2.381307	2.428228	2.39466	2.277789
BMP1	1569001_at	2.289408	2.27036	2.250324	2.254375	2.282386	2.295371	2.253428	2.266336	2.253534	2.242008	2.24687	2.26018	2.148204
BMP1	1569002_x_at	2.331529	2.308274	2.288429	2.291504	2.314151	2.292026	2.296487	2.306025	2.291133	2.281633	2.290951	2.301148	2.184252
TMEM49	1569003_at	2.442527	2.428494	2.408021	2.413525	2.428278	2.317571	2.388525	2.380318	2.385712	2.37321	2.379702	2.395007	2.978517
LOC284379	1569006_at	3.14039	3.053071	2.988437	3.05151	3.22023	2.9873	3.058899	3.054715	3.056502	3.029626	3.073302	3.099547	2.817453
LOC339742	1569008_at	1.627279	1.626653	1.597696	1.611515	1.635263	1.602161	1.623517	1.618588	1.618506	1.608248	1.609591	1.620161	1.604407
LOC96610	1569013_s_at	2.698529	2.639257	2.610573	2.630251	2.687782	2.622206	2.646638	2.653183	2.644506	2.61603	2.649077	2.664339	3.727476
NEDD9	1569020_at	2.239349	2.230477	2.209292	2.215129	2.22014	2.216398	2.221781	2.221892	2.219967	2.214468	2.209177	2.210401	2.199485
PIK3C2A	1569021_at	3.774699	3.639922	3.502485	3.655161	3.877187	3.597471	3.663007	3.664308	3.69418	3.632547	3.726896	3.81333	3.292491
PIK3C2A	1569022_a_at	2.366985	2.344318	2.32241	2.325619	2.349999	2.307299	2.333533	2.33877	2.329251	2.320486	2.329464	2.468183	2.246362
FAM13A1	1569024_at	2.445147	2.416964	2.380022	2.407472	2.432863	2.411493	2.425526	2.427347	2.423747	2.40783	2.416674	2.4533	2.281015
FAM13A1	1569025_s_at	2.928668	2.868826	2.795675	2.860949	2.95987	2.862686	3.202673	2.89652	2.999717	3.120488	2.889167	2.895641	3.987042
NUB1	1569030_s_at	6.3387	6.427618	6.308114	6.452329	7.543965	7.369517	6.136088	7.370097	6.511494	6.254938	7.158788	6.703628	6.09579
LOC642852	1569032_at	2.27492	2.265434	2.242777	2.24765	2.26169	2.250801	2.261507	2.256544	2.241342	2.231951	2.240209	2.252767	2.099647
IGF2BP3	1569033_at	3.409398	3.387094	3.264207	3.363404	3.396326	3.368887	3.383936	3.638821	3.390602	3.368293	3.485987	3.384984	3.266351
LOC440864	1569034_a_at	2.468616	2.434783	2.399501	2.416056	2.448959	2.421076	2.426122	2.434305	2.422785	2.408704	2.420417	2.431188	2.30649

ZNF677	1569039_s_at	3.294579	3.034025	3.091857	3.078301	3.329295	3.501322	3.071241	3.10746	3.180063	3.103312	3.190317	3.191987	3.645144
FLJ40330	1569040_s_at	3.303686	3.156191	3.054274	3.175039	3.332249	3.394458	3.471378	3.429687	3.461674	3.28674	3.395345	3.617311	3.337796
LRP1	1569042_at	2.356427	2.328597	2.288957	2.307076	2.339429	2.306805	2.315258	2.327397	2.311951	2.306067	2.309555	2.321192	2.199131
CDC42BPG	1569044_at	2.139877	2.120167	2.100325	2.280397	2.128023	2.115854	2.109343	2.120881	2.107566	2.099406	2.105647	2.115641	2.027499
TMEM112	1569048_s_at	2.311095	2.301185	2.269887	2.279719	2.300154	2.283553	2.29049	2.30051	2.288618	2.327179	2.278212	2.290625	2.253732
AP3M2	1569053_at	3.841586	3.760116	3.581924	3.439106	3.602476	3.751605	4.767922	4.597369	4.756005	4.968058	4.044817	4.320314	3.784943
SLC1A3	1569054_at	2.80579	2.751135	2.732546	2.73699	2.794468	2.736027	2.748086	2.746461	2.733084	2.727728	2.73442	2.767692	3.934742
MIA3	1569057_s_at	4.352796	4.34532	6.378076	5.506348	4.311836	3.494623	4.344894	3.268	4.713688	4.670496	2.935996	2.512484	5.825761
GTF3C3	1569058_at	3.05365	2.976062	2.865336	2.968337	3.24454	2.974669	2.983047	2.991412	3.011769	2.955574	3.035642	3.131912	2.759632
IQGAP3	1569061_at	2.677361	2.589629	2.544935	2.56141	2.656862	2.587825	2.613188	2.624538	2.609659	2.59174	2.607816	2.673003	2.47778
IQGAP3	1569062_s_at	2.386913	2.35322	2.324452	2.335231	2.370319	2.331098	2.343405	2.355173	2.340128	2.331283	2.339314	2.349841	2.221041
LOC643338	1569064_at	2.581755	2.549351	2.5052	2.533029	2.574152	2.527597	2.537583	2.548738	2.536973	2.517562	2.530896	2.544141	2.435898
LOC643338	1569065_s_at	2.309961	2.298825	2.277777	2.284734	2.31497	2.484047	2.292531	2.295569	2.283048	2.281671	2.29231	2.286785	2.171773
TDRD3	1569069_s_at	4.047692	3.872736	3.879271	4.180999	4.250479	4.447718	4.106804	4.128563	3.992627	4.029505	4.063672	4.610338	5.094238
ABCB5	1569072_s_at	2.205917	2.189921	2.167179	2.171406	2.21704	2.171742	2.182025	2.187937	2.174287	2.166826	2.172581	2.182026	2.088319
SMARCA4	1569073_x_at	3.243394	3.31799	3.015343	3.0846	3.193448	3.379824	3.088899	3.533963	3.011612	3.131946	3.297037	3.367584	2.79487
FLJ37078	1569074_at	2.164866	2.143943	2.114052	2.122104	2.156102	2.515613	2.126076	2.139804	2.124727	2.113411	2.123595	2.13476	2.048134
FLJ16287	1569076_a_at	2.372692	2.34248	2.304481	2.32296	2.356144	2.31642	2.331342	2.342549	2.327931	2.317396	2.35862	2.35464	2.24917
FLJ16287	1569077_x_at	2.303172	1.624594	1.606307	1.608301	1.718806	1.610862	1.614677	1.621199	1.614991	1.607901	1.605748	1.607072	1.581797
RNF213	1569078_at	3.2599	3.148806	3.141686	3.162272	3.280635	3.137821	3.1359	3.165217	3.158036	3.109791	3.19438	3.238608	3.073729
RNF165	1569080_at	2.417267	2.377265	2.338121	2.358861	2.392282	2.361162	2.370267	2.37563	2.366653	2.348969	2.36319	2.389658	2.239512
EML2	1569086_at	3.039146	2.962557	2.858394	2.923979	3.038696	2.939902	2.93341	2.942649	2.943234	2.906949	2.943331	2.955692	2.749674
FLJ35390	1569089_a_at	2.213355	2.185806	2.162465	2.167119	2.195488	2.166172	2.17817	2.186451	2.173928	2.166832	2.170456	2.182763	2.087529
FLJ35390	1569090_x_at	2.650954	2.643323	2.611853	2.62378	2.648815	2.619799	2.631038	2.640802	2.62736	2.61916	2.625105	2.685596	2.27213
LOC731424	1569095_at	3.057527	2.986562	2.972437	2.978401	2.996813	2.966743	2.992913	3.002024	2.997653	2.991626	2.986209	3.011594	2.109769
TP53BP1	1569097_at	1.907437	1.89533	1.875196	1.878945	1.894119	1.883125	1.889869	1.891819	1.880231	1.874831	1.87498	1.886882	1.846048
TP53BP1	1569098_s_at	2.269392	2.215438	2.180796	2.194717	2.22218	2.190141	2.206255	2.213191	2.203405	2.191452	2.671815	2.209769	3.126135
ABCA4	1569102_at	2.148992	2.121019	2.096984	2.104214	2.133282	2.101961	2.112924	2.119898	2.105962	2.099205	2.10381	2.115352	2.030684
SETD5	1569105_at	2.163995	2.146915	2.126509	2.128209	2.158926	2.136081	2.148889	2.283141	2.139031	2.133432	2.130245	2.132369	2.064988
SETD5	1569106_s_at	3.379835	3.74519	4.522846	3.621893	3.250997	3.161744	3.840198	2.938901	3.324466	3.564247	3.047027	3.185396	7.365691
ZNF642	1569107_s_at	4.01395	3.847218	3.535443	3.7392	4.336808	4.963117	3.8073	3.82831	4.005987	4.084846	4.150337	4.315555	4.256374
ZNF589	1569108_a_at	2.515063	2.479011	2.438588	2.450805	2.501068	2.452594	2.445978	2.480525	2.462275	2.452088	2.476607	2.513906	2.636048
LOC728613	1569110_x_at	8.172459	7.961733	8.121174	8.204176	7.980451	7.809554	8.026481	7.368543	7.674811	6.854254	1.842671	4.845365	7.640264
SOX13	1569111_at	3.040355	3.087904	2.562499	2.685919	3.454893	3.146088	2.633464	2.798282	2.626304	2.621183	2.67036	2.626003	2.529203
SLC44A5	1569112_at	2.255111	2.228286	2.198883	2.208462	2.242571	2.201604	2.21662	2.227464	2.212326	2.21138	2.214251	2.218803	2.194343
SLC25A24	1569121_at	1.999823	1.986241	1.963268	1.967364	1.997348	1.968006	1.978053	1.984761	1.971449	1.971448	1.966742	1.978818	1.929747
RP11-139H1	1569124_at	2.450557	2.42075	2.371663	2.402654	2.435614	2.383577	2.410852	2.423883	2.404611	2.401384	2.40846	2.445587	2.273146
CCNC	1569126_at	3.95718	3.590188	3.917258	4.058277	4.652829	3.939791	4.737023	4.15519	4.083133	4.255545	5.20787	4.410191	3.652626

ARSK	1569132_s_at	2.838948	2.818585	3.110813	2.769557	2.827575	2.767206	3.060458	2.793362	2.824455	2.768861	2.864069	2.821778	2.643375
ARSK	1569133_x_at	3.329505	3.304864	3.279986	3.355589	3.338934	3.355793	3.282088	3.74716	3.691276	3.520319	3.290363	3.367507	3.126218
MGAT4A	1569136_at	3.051468	2.965133	2.964569	2.978593	3.224153	2.985695	2.993843	2.980487	2.986464	2.978856	2.991387	3.06586	2.675107
SRCAP	1569138_a_at	1.991563	1.973742	1.953833	1.958456	1.970857	1.957171	1.96932	1.9731	1.959207	1.952477	1.957103	1.967218	1.899002
FAM53A	1569139_s_at	4.676143	4.700148	3.993197	4.076944	5.632759	5.345497	3.949284	4.288989	3.605585	3.430078	4.524927	3.767376	3.135816
UBR2	1569140_at	2.797355	2.7148	2.652968	2.70784	2.805692	2.682125	2.71471	2.724273	2.711777	2.690662	2.723789	2.75226	2.518601
PPARGC1A	1569141_a_at	2.553336	2.513983	2.465868	2.492486	2.556533	2.540378	2.502392	2.514305	2.498132	2.480298	2.499133	2.517972	2.336466
TRIM13	1569142_at	3.894843	3.817767	4.485594	3.759126	3.579588	3.283783	5.23098	3.547765	4.745175	5.243457	3.444565	3.555662	5.366151
LOC653325	1569144_a_at	2.749853	2.741687	2.724111	2.726435	2.732702	2.729396	2.72393	2.735885	2.719841	2.725399	2.721649	2.729528	2.618565
C6orf174	1569146_s_at	2.100523	2.088248	2.060577	2.067464	2.085583	2.076805	2.087655	2.085427	2.073002	2.075084	2.084741	2.079434	2.007727
PDLIM7	1569149_at	2.322081	2.305939	2.268034	2.267024	2.307548	2.277885	2.313602	2.387238	3.006631	3.568823	3.786085	2.381203	3.156851
PDLIM7	1569150_x_at	2.077791	2.017193	2.030855	2.056205	2.018027	2.058623	2.070422	2.603011	2.066489	2.869739	4.382701	2.275231	3.12205
LOC162993	1569156_at	3.895503	3.964349	3.607465	5.134	3.759258	4.332784	3.510369	3.521833	3.680113	3.764156	3.809308	3.770854	3.075373
LOC162993	1569157_s_at	5.710169	5.951978	6.388488	6.63451	6.406431	6.451101	4.655582	5.554156	4.131073	4.856163	4.672944	5.327912	3.584067
FXR1	1569171_a_at	1.887068	1.86845	1.84949	1.853531	1.867827	1.851348	1.864074	1.87024	1.855158	1.848073	1.850353	1.860175	1.819334
LOC441005	1569172_a_at	2.300036	2.289737	2.265482	2.2713	2.294743	2.269574	2.274951	2.282671	2.271472	2.266406	2.270709	2.281439	2.165874
TMPRSS12	1569176_at	2.159577	2.135832	2.116476	2.117262	2.142014	2.113975	2.127838	2.133034	2.119226	2.111975	2.120484	2.1289	2.041356
GRIA4	1569178_at	3.779219	3.525447	3.446914	3.566236	3.907555	3.522209	3.541156	3.571424	3.608874	3.551839	3.704216	3.796129	3.256838
CHM	1569183_a_at	3.404087	3.209708	3.131051	3.227079	3.499273	3.166425	3.219222	3.24414	3.253221	3.229629	3.324278	3.370687	3.185626
MYO3A	1569186_at	2.752913	2.685335	2.633753	2.677856	2.742359	2.651668	2.688167	2.70044	2.686824	2.663483	2.686044	2.734588	2.487933
RPL10	1569188_s_at	3.700003	3.769462	3.615617	3.639843	3.665965	3.624346	3.595309	3.807889	3.63775	3.624611	3.637986	3.644015	3.286698
TTC9C	1569189_at	3.749279	3.718253	3.701418	3.507692	3.706232	3.701958	4.663952	3.523884	3.931125	4.101487	3.761798	3.739968	4.547662
SCLT1	1569190_at	2.735311	2.633105	2.561242	2.709863	2.727679	2.706898	3.154146	2.729129	3.666785	3.703685	2.715175	2.723143	2.953813
FLJ44894	1569191_at	2.841144	2.793247	2.742296	2.760081	2.813977	2.756283	2.784329	2.877521	2.779374	2.764505	2.824702	3.19663	2.732408
ZNF789	1569194_at	2.293588	2.267668	2.2474	2.250889	2.266762	2.236551	2.753405	2.263884	2.41678	2.254683	2.277669	2.258778	2.268405
CXCL2	1569203_at	5.633249	5.779836	7.790015	6.634539	4.267128	3.397298	2.773739	2.776306	2.800635	2.853009	2.836203	2.770891	2.684592
TCP11L2	1569206_at	1.594952	1.585815	1.566187	1.571235	1.582481	1.57169	1.586461	1.573705	1.592344	1.574286	1.567013	1.574352	1.543134
TCP11L2	1569207_s_at	2.277242	2.265029	2.238605	2.162156	2.264773	2.245655	2.934308	2.263693	2.579033	3.128147	2.251288	2.608486	2.167436
LOC619207	1569212_at	2.175188	2.14771	2.125853	2.128914	2.147184	2.12613	2.137373	2.144537	2.140831	2.128729	2.127371	2.141735	2.058936
LOC400891	1569213_at	2.246819	2.221597	2.188237	2.207051	2.243756	2.203936	2.209978	2.219088	2.208968	2.201434	2.227783	2.383744	2.148097
SCML4	1569225_a_at	5.474763	5.588082	3.24902	3.216678	3.762551	4.166213	2.573208	3.236623	4.185749	4.442528	2.649974	2.604779	2.893877
LOC729303	1569234_at	2.272652	2.248005	2.385582	2.639493	2.27091	2.548415	2.234194	2.2433	2.218735	2.217302	2.229345	2.245178	2.114018
LOC729303	1569235_a_at	3.205296	3.165534	3.028765	3.147482	3.241618	3.160686	3.173019	3.162243	3.170337	3.162016	3.169416	3.1792	2.9329
ZNF93	1569240_at	2.168755	2.144281	2.119583	2.130982	2.156029	2.130746	2.136931	2.143237	2.130883	2.120508	2.128867	2.137471	2.048409
ZNF93	1569241_a_at	2.476951	2.444986	2.392352	2.425738	2.461728	2.407808	2.434498	2.444949	2.431261	2.419207	2.432202	2.440797	2.315031
C8orf74	1569245_at	2.229317	2.208949	2.184097	2.188297	2.214605	2.193128	2.196348	2.207999	2.200535	2.183543	2.188798	2.201253	2.103367
C8orf74	1569246_a_at	2.725491	2.704149	2.648721	2.689837	2.716438	3.697564	2.630138	2.686936	2.687132	2.679692	2.679585	2.694724	2.545159
ZNF333	1569250_at	3.824156	4.049426	3.76888	3.785799	3.852656	3.781093	3.791193	3.806042	3.78741	3.778459	3.790739	3.79069	3.683361

ZNF333	1569251_a_at	4.383927	4.317602	3.321385	3.627597	3.709714	3.652831	3.581682	4.43314	3.411587	3.433419	3.642452	3.64638	3.632802
INTS4	1569253_at	2.282637	2.254896	2.224731	2.235759	2.269245	2.236344	2.243626	2.251433	2.239472	2.232246	2.239172	2.249773	2.173029
INTS4	1569254_s_at	3.192336	3.020475	2.944325	3.05919	3.183193	2.966349	3.039541	3.065174	3.058092	3.033416	3.100318	3.20428	2.712237
FAM43B	1569256_a_at	2.334543	2.325685	2.30907	2.306395	2.370652	2.316006	2.324698	2.318825	2.306551	2.299336	2.310918	2.320747	2.314824
FMNL1	1569257_at	3.575017	3.208479	3.138351	3.333065	3.307676	3.082112	3.115345	3.223281	3.070008	3.153306	3.212729	3.257386	2.789672
C6orf157	1569262_x_at	4.56163	5.060145	3.817937	3.99982	4.672027	4.574134	3.995078	4.144583	4.204702	3.967987	5.002783	4.613777	3.407187
LOC400655	1569264_at	2.125355	2.104836	2.07907	2.087317	2.108784	2.085454	2.093074	2.101625	2.088601	2.082136	2.086758	2.098099	2.015632
GRIN2C	1569268_at	2.590213	2.592152	2.573957	2.564556	2.589621	2.581422	2.576889	2.590798	2.584135	2.57996	2.575363	2.579896	2.406172
SRGAP1	1569269_s_at	2.585988	2.559724	2.540814	2.537293	2.562291	2.544294	2.559164	2.745888	2.545892	2.539737	2.54165	2.562481	3.711288
PIK3C3	1569272_at	2.330509	2.297245	2.257965	2.273723	2.303576	2.273245	2.281722	2.290411	2.278946	2.27987	2.295325	2.304765	2.185947
LOC283551	1569274_at	2.260552	2.224213	2.208995	2.2173	2.532748	2.213148	2.223581	2.235102	2.217659	2.210305	2.218638	2.22866	2.144216
ZNF91	1569277_at	3.681657	3.460463	3.411526	3.511706	3.794349	3.344771	3.562168	3.514581	3.536242	3.444009	3.595432	3.636295	3.22366
hCG_16461	1569283_at	3.297524	3.103609	3.233656	3.846722	3.306468	3.048443	3.150493	3.147535	3.142703	3.113296	3.335067	3.362147	3.218288
BIVM	1569289_at	2.470645	2.444192	2.407303	2.427377	2.461491	2.422979	2.435641	2.448417	2.433457	2.421016	2.425608	2.449739	2.31824
GRIA3	1569290_s_at	3.057097	3.149309	4.566591	4.594185	2.6822	3.116294	2.68122	3.089488	2.922128	3.943591	2.663131	2.242563	3.567042
CROCCL1	1569291_at	2.293255	1.991593	1.974238	1.942693	2.026765	2.152671	1.990956	2.404865	1.98529	1.974542	1.966511	1.967755	1.812979
CROCCL1	1569293_x_at	2.537944	2.534784	2.514528	2.491529	3.285375	3.019655	2.534181	3.613015	2.527228	2.515341	2.5179	2.52423	2.30032
RNF187	1569294_at	2.195914	2.15754	2.168167	2.171552	2.190996	2.173321	2.191989	2.381307	2.175837	2.169287	2.173741	2.234357	2.124081
C1orf148	1569300_at	3.056101	3.036452	3.002822	3.037642	3.199424	3.112546	2.826716	3.033861	2.923439	2.86151	3.027472	3.031444	2.782486
KIAA1731	1569302_at	6.48463	5.820617	5.197544	5.61115	6.190074	5.809087	5.8169	7.41423	6.016998	5.988232	6.823852	7.314794	5.57396
RGS20	1569303_s_at	2.447288	2.439198	2.568614	2.415594	2.432741	2.399557	2.437774	2.434767	2.434129	2.42413	2.421894	2.427037	2.426768
IRGQ	1569310_at	1.65158	1.64363	1.624211	1.629248	1.640449	1.632134	1.642011	1.640382	1.634922	1.627847	1.626258	1.635238	1.601561
LOC554203	1569311_at	4.895594	4.627727	5.345528	5.106442	5.19829	5.14373	3.967316	3.4302	4.119047	3.335052	4.492197	3.500977	4.19994
FLJ44451	1569315_s_at	2.729868	2.693491	2.816993	2.824056	2.74743	2.702346	2.695157	2.705168	2.696777	2.699683	2.691036	2.69682	2.49936
MGC39821	1569318_at	2.351882	2.064101	2.039945	2.042187	2.067819	2.127856	2.052157	2.048584	2.050292	2.03793	2.042006	2.048496	2.015369
GPBP1L1	1569320_at	7.923659	7.911349	7.677768	7.521712	7.28937	6.876591	7.900638	7.647092	7.800816	7.969586	7.37562	7.359829	7.017195
LOC439990	1569322_at	2.25575	2.214848	2.211034	2.214482	2.327253	2.214746	2.219028	2.227155	2.215952	2.195223	2.208545	2.244555	2.051347
PTPRG	1569323_at	3.957113	3.57004	4.609647	3.940912	3.530098	3.695923	3.5277	3.432726	3.633128	3.135385	3.587231	3.435173	5.339677
ARPC5	1569325_at	2.284701	2.251062	2.222029	2.236954	2.251596	2.234048	2.251429	2.257771	2.243702	2.233907	2.243498	2.24368	2.135316
C3orf66	1569332_at	2.626182	2.600578	2.580083	2.583725	2.622064	2.578103	2.589528	2.599957	2.585203	2.56402	2.578154	2.598563	2.415334
STRA6	1569334_at	3.162852	3.139742	2.998748	3.103707	3.147399	3.067549	3.08838	3.138092	3.123578	3.115756	3.137352	3.25657	2.995132
STRA6	1569335_a_at	2.732877	2.703913	2.587218	2.701722	2.926927	2.700056	2.734885	2.854398	2.70564	2.705356	2.705539	2.868379	2.456457
SLC5A9	1569337_at	2.622889	2.590791	2.560065	2.561925	2.614733	2.571577	2.574235	2.587584	2.571166	2.550993	2.567932	2.581445	2.516591
GLI3	1569342_at	2.512012	2.481711	2.456017	2.463953	2.497941	2.463906	2.466704	2.468931	2.466373	2.446788	2.45984	2.473665	3.699937
psiTPTE22	1569348_at	3.033946	2.973561	2.957196	2.934061	3.246866	2.96509	2.959042	2.935519	2.955134	2.88903	3.57175	3.023534	2.963234
C11orf30	1569349_at	2.70654	2.654524	2.601043	2.659187	2.692124	2.639257	2.670007	2.644297	3.067922	2.644556	2.655332	2.669371	2.514672
SOBP	1569351_at	2.07444	2.057977	2.034379	2.040789	2.059551	2.044115	2.052319	2.055854	2.047126	2.035876	2.039794	2.050108	1.985078
KIAA1450	1569352_at	3.116146	2.733275	2.91869	2.088177	2.105069	2.087434	2.131932	2.089343	2.093842	2.272585	2.081388	2.093543	2.033514

CP110	1569353_at	2.816456	2.804914	2.767735	2.79051	2.75604	2.791427	2.806961	2.805106	2.892949	2.789969	2.779624	2.805415	4.029617
NSUN6	1569354_at	5.943842	5.932565	5.230636	5.377209	6.226161	5.8458	5.388566	5.843915	5.441447	5.185542	5.549607	5.466083	3.829763
KCNIP3	1569355_at	2.209137	2.183692	2.166102	2.169802	2.192701	2.159464	2.171753	2.183279	2.167835	2.165916	2.164737	2.177725	2.078157
ALCAM	1569362_at	2.994811	2.873702	2.93337	2.881777	2.971315	2.846236	2.904868	2.86034	2.990363	2.859	2.88572	2.893171	4.12176
KIAA1656	1569365_a_at	3.43353	3.293191	3.17627	3.285555	3.463859	3.26058	3.265595	3.282184	3.287976	3.238859	3.356478	3.562813	2.849669
ZNF569	1569366_a_at	2.603783	2.534562	2.82638	2.718583	2.631123	2.501882	2.603879	2.799104	2.51967	2.570988	2.73004	2.546695	3.849968
LOC648921	1569368_at	3.030009	2.95239	2.944708	2.926861	3.041681	2.949764	2.960375	2.875628	2.970751	3.056678	2.961442	3.144668	2.741522
ZFYVE28	1569369_at	2.987442	2.959019	3.511274	3.319015	3.039026	2.822838	2.897793	2.949354	2.947581	2.881235	2.948807	2.957229	2.67345
LRRC59	1569371_at	2.371818	2.342244	2.313469	2.322944	2.350217	2.324811	2.336617	2.339834	2.324633	2.321147	2.322685	2.333202	2.207062
C3orf62	1569374_at	2.514891	2.477362	2.444665	2.457572	2.498304	2.458823	2.467017	2.475282	2.478497	2.486905	2.465402	2.475179	2.442805
TMEM67	1569377_at	4.013159	3.808579	3.599569	3.743908	4.200521	4.216428	3.736731	3.643393	3.792678	3.651035	3.979884	3.852276	3.901469
FLJ33297	1569378_at	2.109232	2.098786	2.07917	2.083287	2.103491	2.079012	2.094209	2.090175	2.083533	2.076772	2.075853	2.088993	2.034899
ZFYVE28	1569383_s_at	1.654818	1.646737	1.626441	1.630044	1.643738	1.631014	1.640953	1.642781	1.632474	1.625717	1.625637	1.63775	1.601851
KIAA1546	1569385_s_at	3.635784	3.245783	3.460872	3.34703	4.22825	4.105694	3.344104	3.343772	2.779405	3.107397	4.524504	3.346568	3.210314
TNKS	1569391_at	2.096274	2.075111	2.051758	2.058447	2.07934	2.057155	2.068467	2.074755	2.062045	2.054276	2.060023	2.071632	1.990794
GPSN2	1569392_at	3.343653	3.204672	3.179279	3.245023	3.276808	3.541805	3.345428	4.087608	3.369577	3.372431	3.535626	3.48272	2.974635
MGC15885	1569393_at	2.036269	1.996779	1.979252	1.98243	1.993333	1.97916	1.995054	1.995538	1.989101	1.980474	1.979696	1.992361	1.921226
RAB40C	1569396_at	4.957459	5.432519	4.723088	4.881666	5.004099	5.210293	5.274403	5.114495	5.20406	4.924088	4.793736	5.260933	4.37656
CLEC12A	1569401_at	2.909664	2.691555	2.66924	2.723038	2.706513	2.671802	2.66276	2.675929	2.66406	2.630887	2.668235	2.684084	2.470717
MEIS3	1569402_at	2.728167	2.717451	2.713399	2.687035	2.7105	3.036864	2.701803	2.713432	2.697283	2.682979	2.699894	2.699935	2.667495
EIF2C4	1569408_at	2.969578	2.899882	3.004098	3.085276	2.927403	2.89901	2.88108	2.890821	2.890766	2.908291	2.882733	2.920108	2.747798
RP1-14N1.3	1569410_at	2.882429	2.837206	2.758017	2.809816	2.864092	2.805037	2.823988	2.834407	2.825152	2.791976	2.828456	2.949359	2.579532
TMEM67	1569411_at	2.722305	2.645536	2.594303	2.629003	2.688883	2.619825	2.637643	2.648935	2.637085	2.618597	2.637829	2.665477	2.455268
CLDN4	1569421_at	2.436321	2.418608	2.39771	2.403166	2.417657	2.406987	2.412039	2.418185	2.40334	2.388966	2.401507	2.410428	2.298394
FAM129C	1569422_at	3.74467	3.725159	3.686866	3.697583	3.727045	3.70915	3.715945	3.719889	3.63023	3.70012	3.705706	3.728588	3.618937
WIBG	1569428_at	2.551217	2.523726	2.516659	2.522178	2.534936	2.504803	2.532342	2.53295	2.632713	2.513149	2.515038	2.562976	2.484333
PTRH1	1569430_at	2.504592	2.465302	2.419658	2.450233	2.490285	2.43659	2.458466	2.470376	2.456025	2.441884	2.452983	2.467425	2.315945
PAFAH1B2	1569431_at	2.644411	2.605789	2.58716	2.584706	2.635496	2.583904	2.594237	2.604341	2.599224	2.575305	2.587912	2.603443	2.45315
SAMD5	1569433_at	3.105362	2.951098	3.510235	3.229788	3.111263	2.97146	2.958894	2.967003	2.985453	2.932525	3.014347	3.057542	5.176584
C14orf105	1569434_at	3.613437	3.54014	3.702771	3.843006	3.392348	3.391116	3.501226	3.496594	3.45951	3.421122	3.481449	3.188842	2.394667
LOC400128	1569436_at	3.725602	3.832501	3.416761	3.595664	4.25955	3.649159	3.898521	3.58344	3.640091	3.639169	3.512483	3.806953	3.009276
LOC730823	1569445_at	2.212191	2.199318	2.170812	2.413102	2.189808	2.185577	2.194125	2.190428	2.190501	2.14861	2.174985	2.196184	2.180463
MGC45713	1569446_x_at	2.058801	2.037925	2.01768	2.019658	2.042042	2.020379	2.032551	2.034236	2.024518	2.010263	2.022732	2.033654	1.980271
PGM2L1	1569448_at	2.048809	2.074933	1.996839	2.004487	2.012035	2.02849	2.016173	2.000642	1.923889	2.00542	1.986677	1.973291	1.955423
CAPZA2	1569450_at	4.565809	4.087801	4.049365	3.801278	4.379856	4.08991	4.73123	4.415425	4.361545	4.523468	4.363185	4.542372	3.602749
LOC692247	1569452_at	2.30155	2.640761	2.277574	2.282798	2.287317	2.970753	2.202822	2.273577	2.189877	2.175727	2.200112	2.276261	2.097088
LOC692247	1569453_a_at	2.250594	2.227402	2.207122	2.2117	2.232452	2.211513	2.221622	2.225659	2.214136	2.209453	2.213617	2.22276	2.145041
LOC283352	1569454_a_at	3.226815	3.087442	3.049983	3.099561	3.266948	3.070108	3.081259	3.088936	3.107552	3.085233	3.145092	3.127264	2.735391

KCNT1	1569462_x_at	2.028234	2.020682	1.980276	1.997404	2.013363	2.019454	2.005516	2.009678	2.001243	1.98886	2.151385	2.00435	1.911804
PPFIBP1	1569464_at	1.860083	1.849155	1.829645	1.832637	1.848915	1.830859	1.844095	1.846306	1.834431	1.829246	1.828117	1.839496	1.840716
LOC729830	1569465_at	2.357317	2.341751	2.321677	2.325495	2.343799	2.324584	2.338709	2.340983	2.328581	2.318347	2.322305	2.339716	2.228957
C20orf152	1569466_at	2.921447	2.783842	2.77043	2.799109	2.856857	2.852167	2.808528	2.808246	2.802895	2.790043	2.815877	2.85553	2.597112
MGC26356	1569468_at	3.093094	2.973916	2.907561	2.980368	3.085845	2.923823	2.981809	2.989069	2.987111	2.952907	3.013865	3.055688	2.678054
LHX8	1569469_a_at	2.953351	2.870022	2.812919	2.874386	3.001928	2.824422	2.886712	2.898417	2.87108	2.856285	2.884937	2.925842	5.466991
FRMD5	1569470_a_at	2.128878	2.106649	2.079657	2.090913	2.099186	2.095301	2.096672	2.280177	2.089416	2.082638	2.080997	2.094715	2.076351
TTC3	1569472_s_at	2.786079	2.77967	3.351936	2.706276	2.815574	2.680041	3.521969	2.749714	2.865268	2.812002	2.895167	2.819206	5.473373
ZNF783	1569473_s_at	1.357133	1.34999	1.332569	1.33611	1.34315	1.339933	1.348565	1.336974	1.343239	1.333856	1.327652	1.334278	1.349656
DKFZP434L	1569476_at	3.101371	2.977111	2.904263	2.977326	3.097134	2.934083	3.052416	3.935723	3.273306	3.318836	4.255442	4.041811	3.218014
ZNF718	1569479_at	2.542047	2.52091	2.496909	2.506838	2.537924	2.503978	2.508512	2.519472	2.503462	2.48983	2.499737	2.515635	2.368673
SNX22	1569481_s_at	2.767363	2.75491	2.735496	2.73941	2.778319	2.738774	4.794886	5.148163	4.271656	4.621926	3.213883	4.902956	2.683925
MDN1	1569484_s_at	6.65378	6.624637	7.533894	7.881396	8.288587	7.893005	6.342317	7.028004	6.504368	6.528568	6.827152	6.824966	4.533996
TACC3	1569487_at	3.20646	3.165764	3.100842	3.153128	3.136785	3.034024	4.156107	3.678724	3.735977	3.538774	3.17864	3.659998	2.778402
FNDC3B	1569490_at	3.865592	4.025577	3.75926	3.820811	3.844505	3.842956	3.685503	3.81451	3.825553	3.789233	3.819768	3.827925	3.926171
SCLT1	1569495_at	2.390622	2.33573	2.296412	2.317386	2.350569	2.316435	2.329005	2.333644	2.327785	2.318412	2.322378	2.338086	2.316886
SPON2	1569496_s_at	4.678112	3.727233	3.835735	4.162458	4.575653	4.359075	3.283149	3.046602	3.326185	2.895492	3.101411	2.963272	2.85973
KIAA0495	1569502_s_at	2.290281	2.27413	2.247776	2.254764	2.266247	3.116328	2.420913	2.529344	2.361374	2.269492	2.257107	2.593023	2.443403
HEATR5B	1569503_at	3.670145	3.940137	3.253735	3.599205	4.142187	3.43644	3.378757	3.961045	3.505882	3.572845	3.619966	3.63035	3.148808
LILRB4	1569504_at	2.93111	2.839511	2.808661	2.842618	2.89024	2.829448	2.848325	2.864388	2.846409	2.827922	2.850159	2.892206	2.696907
PRDM5	1569508_at	3.072599	2.951531	2.944132	2.97827	3.063537	2.976554	2.982023	2.988948	2.983588	2.966463	2.984304	3.13775	2.836758
LOC646482	1569517_at	2.049099	2.038737	2.021556	2.025391	2.037856	2.021089	2.037943	2.035372	2.030971	2.01998	2.021943	2.029634	1.960184
KIAA1245	1569519_at	7.168744	7.351643	7.077392	7.292908	7.283015	7.254755	4.890149	5.244248	4.870326	4.96911	6.367465	5.172179	5.206552
UNQ2541	1569532_a_at	1.718616	1.708853	1.691518	1.69411	1.705415	1.701662	1.710294	1.701679	1.69867	1.694521	1.695747	1.700383	1.682163
FLVCR2	1569536_at	5.090135	5.228479	5.214313	5.186039	4.663116	4.595896	4.100917	3.883791	3.876397	4.037353	4.344323	4.095433	3.528015
RSU1	1569543_at	4.026929	3.807167	3.955455	3.897694	4.117437	3.844398	3.806762	3.828603	3.941386	3.815548	4.080822	4.157101	3.231832
PTPN18	1569552_at	4.103705	4.034116	4.02064	4.033078	4.180987	4.001665	4.14131	3.941913	4.310684	4.727052	4.036254	4.092463	3.339012
ESR2	1569554_at	2.769729	2.710541	2.657753	2.709514	2.780288	2.676786	2.714502	2.724456	2.710842	2.705188	2.709725	2.735645	2.52695
GDA	1569555_at	3.243276	3.466511	6.091787	6.295936	3.966886	4.539013	2.364041	2.897236	2.759134	2.668116	2.595657	2.524212	2.290535
ZNF248	1569557_at	3.072214	3.05677	2.866999	2.976864	3.265947	3.018883	3.018667	3.275066	2.983017	2.990535	3.127969	3.027594	2.634999
HIRA	1569560_at	3.129714	3.035971	3.012312	3.015598	3.048469	2.945284	3.023553	3.0319	3.020367	2.977934	3.021159	3.063623	2.905115
LOC389328	1569561_at	1.949678	1.934527	1.913953	1.916756	1.936854	1.916795	1.927562	1.928397	1.922033	1.91172	1.917769	1.924465	1.864889
TBC1D1	1569566_at	2.617287	2.586815	2.56737	2.681704	2.600964	2.606815	2.573544	2.58365	2.571899	2.551761	2.567802	2.58094	2.448189
AGBL4	1569570_at	2.473188	2.432131	2.387318	2.421407	2.45341	2.418388	2.430261	2.439067	2.425262	2.417937	2.427756	2.436219	2.339303
LIPJ	1569571_at	3.147458	3.011682	2.939225	3.017945	3.145339	2.954109	3.022433	3.030178	3.034657	3.006183	3.071305	3.098406	2.707568
LOC201651	1569582_at	4.716138	4.493358	5.302033	5.596276	5.695584	5.285431	2.239037	2.573552	2.18081	2.25159	2.225904	2.366463	2.123243
EREG	1569583_at	2.143989	2.124141	2.102878	2.107066	2.121131	2.102805	2.120178	2.109037	2.108361	2.100164	2.099031	2.098933	2.03118
PIK3C2A	1569587_at	2.83228	2.721702	2.73776	2.769473	2.828488	2.764514	2.784693	2.779868	2.770856	2.751738	2.772753	2.796173	2.530682

PIK3C2A	1569588_x_at	2.824296	2.786851	2.774642	2.763689	2.790861	2.776049	2.790815	2.787152	2.776509	2.767329	2.778515	2.868924	2.571152
DOCK5	1569590_at	2.751307	2.694838	2.649416	2.679384	2.703379	2.670946	2.68975	2.725104	2.68393	2.676661	2.684814	2.696015	2.543302
F11	1569591_at	6.31865	5.832889	6.196055	5.522798	4.496262	3.757985	3.793867	3.565507	3.955189	3.906814	3.923111	3.703405	2.996019
F11	1569592_a_at	4.277546	4.332871	4.986523	4.550358	4.176718	3.944221	4.01769	4.022603	4.075113	4.12022	4.076894	4.122005	3.278629
SDCCAG1	1569594_a_at	8.737098	8.703254	8.543601	8.678813	9.041299	9.264989	8.44375	9.014823	8.339289	8.403496	9.107906	8.963655	8.599479
SAMSN1	1569599_at	3.12337	3.084903	2.923787	3.024734	3.261007	3.034443	3.024509	3.019324	3.064875	2.998797	3.070767	3.193153	2.696738
DLEU2	1569600_at	4.202837	4.401555	3.699151	3.936567	4.272466	3.744593	4.472951	4.755155	5.480668	6.243512	4.053047	4.201636	3.515772
ANKRD20A	1569607_s_at	6.278566	6.311616	5.610136	6.230217	7.73792	7.987329	4.006387	2.832954	2.741487	2.432322	2.545346	3.167274	4.446663
LOC643187	1569608_x_at	4.282994	4.028543	3.89294	4.096323	6.414793	6.850241	3.934255	4.282126	4.046091	3.939579	4.34539	4.040785	3.497407
IQCH	1569610_at	2.804199	2.710021	2.653206	2.701697	2.770361	2.682619	2.715428	2.725779	2.710511	2.688889	2.712857	2.744166	2.51709
IQCH	1569611_a_at	2.686362	2.628447	2.57565	2.617376	2.689046	2.626694	2.636822	2.631073	2.632291	2.616937	2.630175	2.652503	2.446662
LCN8	1569614_s_at	2.703218	2.687837	2.672374	2.675845	2.685796	2.676018	2.686823	2.68742	2.682685	2.67451	2.671901	2.679582	2.477062
LOC647309	1569616_at	1.861101	1.847022	1.828258	1.832512	1.846824	1.833702	1.844521	1.844499	1.835557	1.831574	1.82632	1.838104	1.800452
OSBP2	1569617_at	3.26391	3.160891	3.047721	3.052799	3.336455	3.000643	8.445088	7.293859	8.328216	8.232525	6.708485	7.227128	2.957234
LOC389906	1569629_x_at	3.422467	3.404467	3.385356	3.390886	3.398134	3.377802	3.389337	3.358206	3.402593	3.376892	4.629236	3.39371	3.593226
RUFY2	1569630_a_at	3.564083	3.381563	3.240287	3.368793	3.543945	3.540434	3.390813	3.397869	3.404553	3.362956	3.519061	3.532479	2.99961
NMNAT1	1569631_at	3.393611	3.422472	3.195944	3.289795	3.34167	3.226149	3.157108	3.263594	3.190069	3.13861	3.547774	3.452162	3.256198
SEPSECS	1569634_at	1.467811	1.461193	1.443313	1.446165	1.452185	1.445721	1.457394	1.451599	1.453848	1.445385	1.441177	1.444865	1.429415
ZNF100	1569637_at	3.332512	3.270017	3.179602	3.254274	3.323785	3.242054	3.255498	3.275349	3.252978	3.250914	3.262984	3.293167	3.123821
SOX5	1569638_at	2.516537	2.489551	2.459661	2.476552	2.50574	2.457167	2.48519	2.496363	2.482145	2.484184	2.480634	2.490826	2.42734
KIAA1706	1569640_s_at	1.794062	1.786512	1.767029	1.77269	1.782291	1.775678	1.784289	1.778396	1.779469	1.773256	1.770057	1.776451	1.730756
F2R	1569642_at	2.16558	2.460038	2.474035	2.634142	3.009079	2.480852	2.355121	2.490082	2.26634	2.060456	2.44798	2.485085	2.422504
LOC643623	1569647_at	2.753878	2.533203	2.510343	2.526574	2.532766	2.523152	2.525973	2.533875	2.524188	2.509025	2.522796	2.532732	2.361699
DACT2	1569648_at	5.395963	5.544601	3.335734	3.569933	3.578442	3.567896	3.433	3.568357	3.365401	3.541992	3.386253	3.570073	3.647179
MLLT3	1569652_at	2.89954	2.863981	2.748861	2.851287	2.896379	2.866163	3.727984	3.421935	4.340358	5.187163	3.397995	2.991067	3.879442
FOXR2	1569669_at	2.807291	2.770694	2.717624	2.75086	2.794303	2.752741	2.758901	2.771334	2.756331	2.744558	2.75708	2.765596	2.53113
BHLHB9	1569674_at	3.064785	2.959161	2.889893	2.962618	3.070306	2.974471	2.987077	2.987069	2.971781	2.935523	2.990962	3.204736	2.669548
POU2AF1	1569675_at	2.074449	2.056276	2.0361	2.037351	2.057372	2.03775	2.047341	2.054151	2.03966	2.036014	2.038348	2.049088	1.97228
TOR1AIP2	1569676_at	2.67839	2.64164	3.071384	2.628052	2.641567	2.612792	2.647857	2.610854	2.663421	3.112435	2.597735	2.605581	3.059912
C8orf45	1569677_a_at	2.667598	2.646734	2.559463	2.59965	2.756509	2.577844	2.612096	2.622568	2.61081	2.589238	2.629103	2.660753	2.459337
CDH22	1569679_at	3.128636	3.122868	3.105508	3.106213	3.114544	3.113331	3.122038	3.11978	3.118053	3.091526	3.103926	3.104154	3.108555
XYLB	1569683_at	5.217304	4.353965	4.257649	4.444171	4.31982	4.461	4.172167	4.075494	3.997849	4.221423	4.234121	3.816123	3.6904
FMO5	1569688_at	9.848713	9.825743	7.387029	7.57734	8.9881	8.685265	3.253052	3.180235	3.376651	2.857391	3.224152	3.248302	2.43708
LOC730017	1569689_s_at	2.286839	2.265021	2.223762	2.240842	2.269416	2.233415	2.250431	2.263923	2.247075	2.235111	2.248018	2.258768	2.144342
CCDC36	1569690_at	2.427957	2.382107	2.335015	2.375578	2.44969	2.597499	2.386027	2.398716	2.37992	2.367696	2.384389	2.467595	2.255024
BTBD8	1569693_at	2.90913	2.847197	2.810243	2.808253	2.884439	2.789334	2.825094	2.844504	2.825012	2.794765	2.829772	2.852334	2.579394
LOC402779	1569696_at	2.336893	2.215809	2.191716	2.194753	2.214375	2.209146	2.214211	2.329891	2.202468	2.202293	2.198605	2.254326	2.165749
AK7	1569699_at	2.043209	2.027902	2.00842	2.006103	2.031201	2.010204	2.044923	2.030447	2.02555	2.145524	2.01402	2.042326	1.933394

AK7	1569700_s_at	1.985123	1.979902	1.955289	1.963512	1.974863	1.961765	2.016963	1.975116	1.965962	1.961734	1.956368	1.96864	1.923233
PER3	1569701_at	4.800444	4.509143	4.789956	4.496172	3.365029	3.099471	3.110836	3.094624	3.103173	3.07147	3.104039	3.11053	2.679674
MYSM1	1569706_at	3.938287	4.160437	3.766345	4.511484	3.057738	3.417092	3.962993	3.47829	4.245947	3.617251	3.483882	3.639195	3.542967
C1orf84	1569709_at	2.638796	2.515518	2.471143	2.499043	2.672404	2.498132	2.518777	2.521874	2.512405	2.494606	2.567173	2.710809	2.311514
LOC729725	1569712_at	3.042863	2.927523	2.851136	2.915789	3.070472	2.880553	2.930178	2.936504	2.9345	2.891918	2.96563	3.01281	2.647143
ZFYVE20	1569714_at	2.594158	2.564189	2.810256	2.523916	2.584809	2.542747	2.56326	2.575871	3.027781	2.89343	2.559914	2.569025	2.456457
BCL2L14	1569719_at	2.280288	2.252451	2.218375	2.239395	2.263094	2.226525	2.242571	2.252906	2.23714	2.225069	2.238334	2.246538	2.137344
LOC219731	1569722_s_at	3.032102	2.99446	2.928553	2.970025	3.047024	2.963962	2.98806	2.995509	2.986283	2.974243	2.977158	3.001159	2.805616
SPIRE2	1569723_a_at	3.10566	3.475799	2.94116	2.974244	2.985918	3.051441	2.959934	2.976618	2.958885	2.918856	2.987375	3.101123	2.891549
ASZ1	1569729_a_at	2.885966	2.828519	2.74329	2.817109	2.885374	2.80149	2.814768	2.823163	2.81384	2.789344	2.819742	2.83884	2.575794
FLJ40243	1569730_at	1.99242	1.980844	1.959788	1.961375	1.978923	1.960499	1.976047	1.976881	1.968511	1.95952	1.95971	1.973403	1.918443
CASKIN1	1569737_a_at	1.992262	1.979676	1.962165	1.960928	1.977157	1.967997	1.978859	1.978376	1.971069	1.964087	1.962998	1.97136	1.916877
LOC221946	1569739_at	2.06603	2.056237	2.033833	2.039647	2.055995	2.036585	2.045556	2.053451	2.040338	2.035333	2.03238	2.045895	1.996114
PRKCZ	1569748_at	2.702472	2.659773	2.592099	2.636708	2.694941	2.611284	2.649703	2.659204	2.645983	2.618243	2.643791	2.663538	2.459348
ZNF286A	1569751_at	2.557097	2.485056	2.438849	2.469271	2.508232	2.452715	2.480223	2.49247	2.474772	2.467164	2.476008	2.490369	2.412832
ATP8A1	1569773_at	2.737352	2.686234	2.669387	2.686933	2.722348	2.675081	2.695276	2.707261	2.691295	2.684027	2.692063	2.709588	2.488582
RNF157	1569775_at	1.501345	1.48861	1.468989	1.474353	1.478168	1.469278	1.485982	1.47051	1.481236	1.475245	1.469586	1.463089	1.461768
ZPLD1	1569777_a_at	2.088429	2.069503	2.044099	2.050604	2.071321	2.048964	2.061771	2.066879	2.053034	2.051337	2.052406	2.062653	1.984081
LOC285423	1569781_at	2.569468	2.512813	2.474263	2.506632	2.576328	2.496557	2.518403	2.52187	2.513917	2.498655	2.520269	2.534268	2.359179
LOC401387	1569783_at	3.114212	3.014977	2.898427	3.011137	3.147318	2.917061	2.971013	3.01849	2.998989	2.945091	3.032441	3.098314	2.682894
ST8SIA1	1569788_at	2.986133	2.964892	2.876941	2.917278	2.975269	2.891734	3.02994	2.957685	3.068481	3.012323	3.013155	3.026927	4.417835
STK4	1569791_at	2.286287	2.267899	2.219685	2.25058	2.288873	2.250442	2.261894	2.265945	2.252079	2.234103	2.248734	2.261847	2.191703
MGC50559	1569792_a_at	4.232079	3.937302	4.578408	4.387226	4.498796	4.485508	3.251598	3.020528	2.551135	3.581926	2.523796	2.626256	2.826796
SLC25A18	1569793_at	2.908368	2.881363	2.846581	2.863488	2.978021	2.862596	2.8735	2.863581	2.866291	2.85841	2.867492	2.874751	2.768113
ATRNL1	1569796_s_at	2.620034	2.516159	2.541984	2.4987	2.695457	2.48385	4.029302	3.343218	4.281928	3.796026	3.718604	2.925385	3.397021
STRN	1569813_at	2.833453	2.818507	2.795813	2.800248	2.819383	2.798647	2.756721	2.805653	2.816985	2.800403	2.796909	2.810641	2.82245
STRN	1569815_x_at	3.278156	3.262178	3.208491	3.245186	3.263642	3.244449	3.264387	3.249713	3.261327	3.239705	3.238522	3.255842	3.270696
LOC440346	1569823_at	3.423948	3.286515	3.145932	3.277166	3.45433	3.375628	3.249617	3.245341	3.289698	3.243469	3.346373	3.392792	2.856186
ATG7	1569827_at	2.319722	2.301843	2.276507	2.273816	2.307211	2.282031	2.295051	2.295505	2.299417	2.278406	2.283753	2.291231	2.218232
PTPRC	1569830_at	2.776479	2.710879	2.71033	2.693949	2.748927	2.664092	2.701034	2.714009	2.6979	2.677031	2.697771	2.724285	2.492827
LOC339352	1569835_at	2.644489	2.601236	2.543917	2.587778	2.635876	2.56123	2.595086	2.608856	2.592058	2.571153	2.592509	2.613693	2.41988
C9orf29	1569839_s_at	2.41819	2.398708	2.377408	2.37528	2.453977	2.381175	2.385918	2.395156	2.383496	2.367001	2.379232	2.391534	2.254751
LOC728114	1569842_at	2.625659	2.571516	2.511106	2.552996	2.614058	2.529595	2.560238	2.563689	2.548751	2.542539	2.5538	2.575328	2.444504
DYNC1I1	1569843_at	2.427539	2.401321	2.372189	2.381522	2.409706	2.373788	2.389618	2.401533	2.386655	2.373704	2.386693	2.397155	2.316133
CGNL1	1569847_at	4.429789	4.712257	2.699383	2.743057	4.881703	3.35887	3.351703	3.55267	3.216812	3.205682	3.342265	3.322634	2.993581
C7orf53	1569852_at	2.066882	2.050661	2.024541	2.038357	2.083662	2.029086	2.040917	2.048244	2.033139	2.025434	2.032672	2.043617	1.950952
TPP2	1569856_at	2.806359	2.754004	2.6849	2.707781	2.894315	2.589581	2.652763	2.717918	2.76721	2.733212	2.754375	3.070574	2.296539
TPP2	1569857_s_at	3.574411	3.42874	3.354812	3.518117	3.75817	4.082189	3.507277	3.497138	3.556651	3.482924	3.668385	3.752152	3.498935

TRAF5	1569861_at	2.062965	2.045234	2.022736	2.027704	2.046431	2.026703	2.036862	2.041412	2.028759	2.022096	2.024738	2.037989	1.968197
SERAC1	1569864_at	2.751216	2.687866	2.651811	2.694619	2.739132	2.655885	2.693369	2.703291	2.693443	2.680299	2.694159	2.714274	2.55079
EME2	1569867_at	3.346928	3.071839	2.996143	2.975448	3.023241	3.104217	3.995361	3.600783	3.690644	3.189206	3.133746	3.125439	3.664241
EME2	1569868_s_at	6.137176	5.900246	4.402177	4.811549	5.104684	5.424584	5.962481	6.662566	6.054561	5.956596	7.271061	6.475623	5.600448
LOC650392	1569871_at	1.451743	1.444828	1.42707	1.426842	1.436502	1.428403	1.44197	1.436088	1.438043	1.431935	1.566508	1.422844	4.64777
LOC650392	1569872_a_at	2.278188	2.235297	2.201394	2.223532	2.289513	2.274128	2.284441	2.943409	2.237142	2.272121	6.728164	3.392578	7.708679
LIPJ	1569873_at	3.236702	3.115021	3.038286	3.129868	3.268269	3.154485	3.175798	3.138476	3.171916	3.111043	3.172703	3.2079	2.971178
LIPJ	1569874_s_at	2.080316	2.057745	2.040515	2.045278	2.111473	2.04489	2.054862	2.056185	2.04475	2.039142	2.03886	2.05499	1.9776
C17orf57	1569876_at	3.190143	2.99535	2.919894	2.982359	3.186502	2.965635	2.992428	2.997213	2.988457	2.970828	3.021201	3.058647	2.734346
CCNYL2	1569878_at	2.682713	2.643475	2.629625	2.639896	2.688763	2.639253	2.6488	2.656606	2.642418	2.618475	2.640214	2.65288	2.559729
MEGF11	1569879_a_at	1.873505	1.850893	1.816186	1.834935	1.895196	1.838174	1.848407	1.852107	1.839931	1.833549	2.018435	1.850887	1.792495
DRP2	1569881_at	2.822234	2.76093	2.724804	2.736437	2.847976	2.73444	2.751898	2.756965	2.746306	2.735322	2.749577	2.755942	2.529454
LOC348808	1569882_at	2.429682	2.406896	2.364727	2.385173	2.418125	2.380665	2.39335	2.403718	2.388775	2.37686	2.390285	2.397458	2.276662
GLB1L3	1569886_a_at	3.079855	3.000744	2.94229	2.956124	3.075247	2.934661	2.967988	2.994544	2.986721	2.968995	2.994177	3.071164	2.691614
LOC286135	1569887_a_at	2.268113	2.250634	2.218084	2.22474	2.255014	2.241263	2.231014	2.249583	2.235289	2.268406	2.235947	2.245834	2.101765
ATP5A1	1569891_at	1.965983	1.950469	1.92943	1.936895	1.9519	1.935306	1.946703	1.948071	1.936737	1.935481	1.92996	1.943073	1.898195
PPP2R3C	1569894_at	4.138185	3.538889	4.418968	4.053503	3.989032	3.514478	3.331226	3.590436	3.609747	3.575569	3.229017	3.28359	3.334819
FLJ16124	1569900_at	2.151754	2.135253	2.111333	2.117885	2.135278	2.115177	2.124714	2.133932	2.119616	2.112698	2.117496	2.127019	2.029334
HSD11B1L	1569905_at	1.997347	1.996238	1.973704	2.059263	1.982609	1.922253	1.99159	1.984404	1.986884	1.977881	1.967136	2.037168	2.685082
PHF20	1569906_s_at	3.546707	3.483696	3.439934	3.40516	3.530711	3.33392	4.55192	4.575945	4.254934	3.866401	3.804483	3.699241	3.660906
KRT79	1569909_at	3.188464	3.173067	3.13077	3.153574	3.174146	3.159341	3.163623	3.171254	3.077556	3.14868	3.206365	3.165908	3.05359
LOC285708	1569923_s_at	2.60927	2.569275	2.552038	2.556594	2.619709	2.555449	2.559595	2.572783	2.55825	2.536563	2.555762	2.577629	2.489312
SLC34A3	1569926_s_at	2.401247	2.379414	2.357079	2.373282	2.387484	2.359445	2.364798	2.395776	2.364646	2.35886	2.356821	2.375739	2.282158
RP11-262D1	1569932_at	2.027896	2.015742	1.999823	2.003818	2.015127	2.006831	2.015793	2.020145	2.00829	2.004012	2.002444	2.017206	1.944767
DEPDC2	1569934_at	4.291117	4.105322	4.007387	4.072921	4.45644	3.990675	3.961584	4.066637	4.080687	4.009059	4.163831	4.232222	3.936998
ZNF664	1569935_at	1.224536	1.217628	1.196926	1.202823	1.210414	1.19714	1.215336	1.201017	1.210612	1.200957	1.197909	1.192617	1.212706
ZNF664	1569936_a_at	2.753923	2.737982	2.755075	2.840527	2.749014	2.717133	2.720648	2.736063	2.717333	2.57195	2.660581	2.73082	2.634192
SIRT5	1569938_at	4.064134	4.815526	3.625847	4.144126	4.328991	3.294548	3.210994	3.381806	3.336273	3.349104	3.531485	3.461572	2.900125
SLC6A16	1569940_at	4.891398	4.989764	4.502467	4.6231	4.984261	5.35546	2.728505	2.715513	3.231618	2.679088	2.70131	3.030544	2.869814
GRK5	1569949_at	1.186626	1.180846	1.162743	1.165795	1.175374	1.168128	1.178688	1.164949	1.173928	1.164191	1.1592	1.16885	1.1726
UNC13C	1569969_a_at	2.531336	2.492957	2.437605	2.47081	2.587121	2.482149	2.482138	2.494605	2.479757	2.46239	2.481406	2.491509	3.194506
HKR1	1569979_at	1.965412	1.950403	1.930601	1.93441	1.950983	1.933723	1.944995	1.946555	1.935711	1.929515	1.929249	1.942487	1.880019
HKR1	1569980_x_at	1.928513	1.913822	1.894145	1.898198	1.988333	1.896851	1.909423	1.910867	1.89988	1.894304	1.892975	1.915751	1.848016
TNNT3	1569986_x_at	3.215962	3.172817	3.12488	3.202801	3.17071	3.158166	3.157114	3.168047	3.153277	3.140453	5.766162	3.161923	7.649156
NUDT3	1569990_at	2.847963	2.803775	2.777707	2.777633	2.822929	2.863034	2.787741	2.802854	2.785293	2.767047	2.885066	2.850954	2.543989
KY	1569991_at	2.428441	2.400053	2.379801	2.377666	2.41239	2.381763	2.389063	2.396631	2.381231	2.369542	2.380089	2.38892	2.325076
LOC152586	1569995_at	2.545814	2.508863	2.454398	2.487774	2.530601	2.500856	2.498242	2.512301	2.494816	2.482739	2.49394	2.510448	2.345758
MMD2	1569998_at	2.737002	2.676474	2.603162	2.6723	2.799845	2.623323	2.677623	2.733769	2.679497	2.677465	2.69692	2.701537	2.45352

CASP8AP2	1570001_at	3.389974	3.229082	3.131322	3.184593	3.392459	3.144363	3.991489	4.486691	4.532661	4.181269	3.628534	3.859559	3.057766
LOC400958	1570006_at	2.610284	2.559742	2.543177	2.548562	2.623007	2.547497	2.558867	2.56722	2.550619	2.537551	2.553236	2.574716	2.399176
LRRC8C	1570007_at	1.950412	1.941398	1.92427	1.925769	1.939785	1.929481	1.940037	1.939589	1.926798	1.927775	1.923613	1.931395	1.870278
LOC253724	1570008_at	2.39898	2.365711	2.321618	2.347357	2.382867	2.333343	2.354858	2.36685	2.35149	2.339154	2.348964	2.361363	2.231409
LOC643711	1570009_at	2.466397	2.402901	2.35995	2.383972	2.421557	2.37715	2.39384	2.405663	2.390329	2.377442	2.389795	2.40044	2.264997
RP11-165H2	1570015_at	2.48651	2.307875	2.22357	2.288193	2.323741	2.28353	2.295442	2.304609	2.291259	2.28205	2.293705	2.302063	2.056788
AACSL	1570020_at	3.105388	3.007532	2.956963	2.976253	3.099841	2.996975	3.007349	3.111417	3.005367	2.957399	3.018802	3.050864	2.688361
LOC360030	1570021_at	5.781212	5.324788	6.066311	6.258348	6.554928	6.665584	3.65173	4.437197	3.576673	3.489429	5.1919	3.904562	3.306161
TACC2	1570025_at	2.115837	2.108803	2.069741	2.09304	3.016284	2.690176	2.086279	2.101087	2.086832	2.071277	2.08496	2.094988	2.02414
CPXM2	1570026_at	1.967207	1.954987	1.935645	1.936936	1.953779	1.94331	1.948845	1.951533	1.94065	1.931546	1.935144	1.94551	1.895083
ZNF396	1570030_at	2.525384	2.496945	2.470071	2.477254	2.508261	2.468968	2.482162	2.490412	2.479946	2.472795	2.473332	2.489392	2.331427
AP3B2	1570032_at	3.393787	3.204587	3.202153	3.247257	3.518316	4.27792	3.212951	3.2648	3.229851	3.203495	3.304828	3.303836	2.868387
WIPI2	1570033_at	2.111602	2.090692	2.065097	2.07213	2.094499	2.071004	2.081255	2.088793	2.075116	2.067522	2.074944	2.083227	2.003005
TBC1D10A	1570035_at	2.624323	2.511973	2.604109	2.566378	2.604103	2.486974	2.436221	2.614881	2.486436	2.448697	2.569005	2.492968	2.31867
ZNF718	1570038_at	2.533209	2.50146	2.482571	2.486446	2.52042	2.486112	2.493278	2.498087	2.488328	2.48162	2.481146	2.49612	2.337758
MGC39900	1570039_at	2.09262	2.070895	2.046991	2.053094	2.073212	2.053172	2.063126	2.070572	2.055232	2.046742	2.461923	2.160873	3.344424
ADAM9	1570042_a_at	2.451756	2.397991	2.403625	2.389272	2.435857	2.394181	2.401721	2.41888	2.402564	2.390157	2.405426	2.454058	2.310303
DPH4	1570048_at	3.008674	2.897577	2.879262	2.882557	3.012724	3.380223	2.901774	3.002308	2.897207	2.891159	3.026274	2.965811	3.339429
RNF144A	1570051_at	2.592697	2.556292	2.512478	2.538815	2.569575	2.524858	2.54836	2.559666	2.547272	2.530964	2.542941	2.563946	2.413802
MIPOL1	1570052_at	2.502049	2.46302	2.427441	2.448815	2.487788	2.459328	2.466202	2.468811	2.453794	2.437906	2.455783	2.466946	2.322772
LOC390604	1570055_at	2.915658	2.910716	2.950026	2.875433	2.942543	2.887613	2.891956	2.900246	2.88917	2.8622	2.878611	2.887374	2.591101
LOC390604	1570057_x_at	2.780105	2.768429	2.747972	2.752137	2.766871	2.742436	2.756654	2.766298	2.753059	2.747763	2.746724	2.76191	2.590914
SUV420H2	1570058_at	2.825457	2.767791	2.685935	2.739074	2.814058	2.751365	2.75372	2.763198	2.758755	2.752922	2.752438	2.786028	2.579383
C20orf160	1570070_at	3.076831	3.014254	2.972761	3.008299	3.031443	3.012694	3.004099	3.014155	3.000673	2.957888	3.012959	3.020419	2.789154
DOCK5	1570078_a_at	2.874678	2.864358	4.848407	5.6684	2.863227	2.495038	2.640569	2.509833	2.630967	2.49082	2.622828	2.636051	2.203691
TTLL1	1570085_at	2.211826	2.183392	2.165499	2.169663	2.184247	2.170516	2.179818	2.184048	2.169918	2.160047	2.167568	2.175282	2.080999
GNB1	1570108_at	2.112945	2.100318	2.080919	2.083072	2.099867	2.080148	2.095553	2.097493	2.086043	2.081568	2.082611	2.091347	2.105062
C14orf48	1570111_at	2.407274	2.376051	2.331952	2.355197	2.390538	2.360166	2.364703	2.376434	2.359839	2.347349	2.360767	2.369814	2.307975
MDGA2	1570114_at	2.427926	2.39308	2.358262	2.376576	2.468259	2.3769	2.387032	2.395463	2.382152	2.367313	2.382801	2.390906	2.254972
PDS5B	1570119_at	3.100994	3.012768	3.005027	3.01393	3.115123	3.011377	3.020151	3.028169	3.008351	3.008793	3.014706	3.02954	2.780499
ZNF365	1570121_at	2.303824	2.273728	2.253877	2.258034	2.288926	2.257231	2.266376	2.271447	2.258113	2.248991	2.255159	2.268127	2.157631
DDX19A	1570128_at	4.491705	4.177634	3.775939	3.483797	3.630768	3.798495	3.269	3.501384	3.372086	3.472794	3.489699	3.629852	3.189134
SPATS2	1570130_at	3.131136	3.341032	2.896839	3.042495	3.148405	3.014787	3.102637	3.069484	3.063367	3.032196	3.058612	3.092518	3.339979
LOC285847	1570131_at	1.881233	1.874335	1.855625	1.853827	1.86927	1.853103	1.869254	1.867305	1.858936	1.849702	1.851193	1.859596	1.761556
ZNF230	1570135_at	3.766919	3.596704	3.577867	3.806428	3.70023	3.390337	3.890765	3.341247	3.870613	4.549804	3.612424	3.617002	3.479493
LOC653110	1570140_at	1.817948	1.810327	1.791589	1.795973	1.808285	1.804485	1.805906	1.804792	1.802694	1.792089	1.788135	1.797385	1.75345
MYO5B	1570141_at	2.60036	2.52626	2.503656	2.508853	2.641638	2.506551	2.519888	2.532626	2.5171	2.510926	2.521097	2.556541	2.36467
UCHL5	1570145_at	2.059868	2.04192	2.016436	2.024757	2.054394	2.022172	2.035192	2.040159	2.026703	2.019086	2.02832	2.036576	1.959864

SOHLH2	1570153_at	2.478017	2.428252	2.393316	2.415228	2.464424	2.407402	3.650354	2.446825	4.603003	3.014216	2.423693	2.52892	3.562084
FMN1	1570156_s_at	2.866753	2.745616	2.764492	2.776013	2.798581	2.79175	2.95392	2.797197	2.786035	2.724597	2.789359	2.788949	2.428942
C14orf91	1570162_at	2.277753	2.393523	2.220645	2.23815	2.265941	2.245531	2.242258	2.256179	2.240001	2.230074	2.241387	2.249441	2.141533
CSMD2	1570169_at	2.339177	2.327859	2.308083	2.311961	2.328418	2.311149	2.323136	2.325094	2.313302	2.310766	2.309103	2.319912	2.203946
INTS7	1570173_at	3.453485	3.419223	3.300912	3.398472	3.456221	3.400561	3.35491	3.987668	4.042759	3.347606	3.656634	3.554312	3.123248
FBXO34	1570183_at	3.346718	3.307219	3.131269	3.255314	3.48635	3.296842	3.293856	3.312667	3.298219	3.189465	3.536308	3.862491	2.820508
GRASPOS	1570186_at	1.939091	1.923267	1.903008	1.891021	1.944391	1.924453	1.920003	1.918664	1.909412	1.8989	1.911871	1.948655	1.824049
NR1I3	1570188_at	4.596471	5.244933	4.290073	4.546105	3.006509	3.443611	2.512299	2.515955	2.504202	2.477878	2.502986	2.51093	2.361338
FLJ31222	1570189_at	2.603217	2.554764	2.506305	2.541995	2.593494	2.522339	2.55455	2.563801	2.552721	2.532697	2.554804	2.589416	2.394988
LSAMP	1570190_at	3.216115	3.136625	2.994508	3.118269	3.218926	3.071831	3.119797	3.085489	3.120428	3.090833	3.12686	3.152725	2.743909
HELB	1570200_at	4.82374	5.11337	4.504057	4.327462	4.834538	5.439316	3.818735	4.784723	4.316692	4.175936	4.535407	4.545262	4.082915
MKL2	1570202_a_at	2.501913	2.466726	2.431168	2.445882	2.472875	2.441306	2.454932	2.465738	2.451333	2.427268	2.452894	2.460613	2.308529
LOC728723	1570204_at	2.666695	2.583436	2.531978	2.564509	2.644434	2.550716	2.584097	2.591261	2.579535	2.560836	2.581241	2.607066	2.411604
FRRS1	1570207_at	2.584087	2.526996	2.484536	2.524764	2.566918	2.524092	2.525143	2.535587	2.521452	2.501208	2.519744	2.538992	2.368195
LOC284260	1570208_at	2.80911	2.733292	2.672428	2.730708	2.797898	2.707186	2.740721	2.752667	2.739036	2.719571	2.737992	2.767612	2.520282
SAPS2	1570210_x_at	7.741113	7.851095	6.512781	6.914534	7.937984	8.373899	6.852591	7.817758	6.762829	6.281509	8.260558	7.785971	5.317959
LOC253724	1570212_a_at	2.369467	2.346331	2.318143	2.326915	2.349049	2.335962	2.335627	2.344906	2.331699	2.323833	2.32902	2.340887	2.268615
LOXL4	1570219_at	1.911343	1.90071	1.879479	1.886745	1.901165	1.888296	1.897051	1.898209	1.885436	1.881164	1.879362	1.891649	1.80076
NDST4	1570222_at	2.762167	2.610002	2.55825	2.599937	2.702443	2.576587	2.613072	2.622208	2.609859	2.58984	2.617909	2.653573	2.432901
SCRL	1570225_at	2.248653	2.237083	2.201016	2.215433	2.234147	2.212596	2.228495	2.49021	2.280538	2.229052	2.269639	2.279362	2.217805
LOC728852	1570226_at	3.166355	3.12672	3.065862	3.099021	3.154851	3.00448	3.072535	3.123522	3.111446	3.093369	3.111657	3.153159	2.948359
CFHR4	1570228_at	2.830163	2.801328	6.378935	6.500508	7.289867	7.112262	2.725588	2.76717	2.760532	2.735539	2.762638	2.79565	2.537842
LATS1	1570231_at	2.325768	2.296516	2.258608	2.280187	2.308829	2.281853	2.288453	2.296897	2.285281	2.275262	2.291673	2.314916	2.17482
MGC27382	1570235_at	2.85745	2.748962	2.70026	2.738093	2.8526	2.705121	2.742891	2.743114	2.743246	2.72239	2.76809	2.779699	2.54635
ZNF527	1570238_at	2.62242	2.613568	2.589041	2.59943	2.611326	2.601664	2.536004	2.611753	2.599062	2.552668	2.59044	2.603371	2.559957
MIST	1570239_a_at	2.849578	2.828702	2.809346	2.810028	2.831414	2.817712	2.806682	2.823836	2.812652	2.804563	2.805904	2.87149	2.635662
SPATA21	1570241_at	2.922199	2.861023	2.807516	2.84272	2.87274	2.866199	2.859701	2.86984	2.858546	2.839696	2.866413	2.881322	2.605964
SPATA21	1570242_x_at	3.796986	3.789363	3.801796	3.775194	3.804808	3.706257	3.768344	3.788038	3.76526	3.735426	3.762039	3.818047	3.530737
LOC440731	1570243_at	3.177663	3.10322	3.097302	3.158452	3.176904	3.16725	3.300637	3.155656	3.17164	3.16543	3.287777	3.211344	2.998075
RP11-35N6.	1570250_at	3.050168	2.966838	2.879888	2.957486	3.099801	2.970081	2.980753	2.982067	2.970107	2.95906	2.995523	3.041019	2.659734
HECTD1	1570251_at	2.94795	2.88897	2.693568	2.730141	3.865272	3.442252	2.828454	2.804683	2.697745	2.702246	3.163593	2.648709	2.40739
RHEBL1	1570253_a_at	2.415872	2.383594	2.341677	2.365962	2.40092	2.356363	2.374536	2.38559	2.370355	2.3621	2.372775	2.386829	2.280363
ANKRD20A	1570255_s_at	3.546076	3.40838	3.345983	3.358619	3.63027	3.661233	3.463766	3.41968	3.462838	3.341219	3.4317	3.572675	4.221773
LIMS1	1570259_at	3.162674	2.801525	3.075986	3.108618	3.051094	3.106292	3.252102	2.875875	3.414433	3.093884	3.217742	3.138066	3.055727
UNQ9391	1570274_at	2.341016	2.310396	2.290301	2.29557	2.369148	2.319255	2.304086	2.315771	2.30008	2.294219	2.301308	2.311139	2.189302
GATA4	1570276_a_at	2.432866	2.397779	2.385163	2.384728	2.416471	2.366894	2.400655	2.400579	2.395801	2.38469	2.384047	2.432893	2.258999
ABCA1	1570279_at	3.469608	4.225432	3.446623	3.516039	2.965697	2.947537	2.871053	2.875692	2.868862	2.855011	2.882922	2.907897	2.616504
LOC646736	1570289_at	2.737261	2.696082	2.66207	2.64772	2.857825	2.685064	2.646296	2.696656	2.681462	2.661546	2.679912	2.723959	2.273905

TBL1X	1570293_at	2.227193	2.212846	2.190206	2.193954	2.21999	2.194358	2.206531	2.20838	2.197325	2.189302	2.190911	2.203918	2.106323
ST18	1570306_at	2.349657	2.319738	2.280323	2.300078	2.341143	2.291442	2.308251	2.320149	2.304133	2.289256	2.305906	2.321287	2.193908
ST18	1570307_s_at	2.777387	2.7568	2.686718	2.731497	2.760365	2.662495	2.744422	2.756263	2.74113	2.734082	2.735921	2.798034	2.552877
LOC283902	1570315_at	2.436834	2.430221	2.378899	2.40621	2.422261	2.414693	2.427757	2.425436	2.421033	2.411852	2.411997	2.419708	2.375464
C20orf62	1570327_at	2.617717	2.578067	2.556965	2.560746	2.606219	2.558035	2.572838	2.575952	2.564057	2.544623	2.563248	2.571978	2.46248
C20orf62	1570328_s_at	2.259632	2.206075	2.18118	2.248601	2.199988	2.193635	2.164455	2.197342	2.185991	2.181796	2.177891	2.19111	2.088792
BDH1	1570336_at	2.386163	2.366917	2.349437	2.35602	2.484063	2.353114	2.365307	2.372438	2.356999	2.353566	2.353739	2.366477	2.295186
FIGLA	1570337_at	2.182474	2.16117	2.134407	2.141732	2.174085	2.140374	2.151018	2.16138	2.145757	2.138524	2.143677	2.155507	2.070427
NKTR	1570342_at	3.16643	3.029593	2.912645	3.025862	3.146557	3.014081	3.038214	3.03813	3.035452	3.014269	3.05087	3.119069	2.938551
C9orf27	1570345_at	3.948979	3.772809	3.700095	3.81221	3.928267	3.768643	3.803961	3.83425	3.859067	3.781065	3.930496	3.884037	3.342134
MLX	1570347_at	2.077873	2.054681	2.175636	2.047172	2.05135	2.043528	2.059723	2.11788	2.055367	2.044308	2.856973	2.054212	2.018983
ADAMTS6	1570351_at	2.820679	2.720098	2.684495	2.713121	2.811879	2.917702	2.724078	2.727422	2.73638	2.721023	2.718182	2.754398	4.045178
ATM	1570352_at	2.607776	2.553562	2.504533	2.540133	2.596815	2.520212	2.55059	2.561001	2.548026	2.528742	2.5496	2.565039	2.392913
ZNF169	1570354_s_at	2.534803	2.500725	2.445736	2.481542	2.552922	2.463148	2.47135	2.491889	2.49293	2.468063	2.485614	2.498899	2.36673
DPY19L1	1570355_a_at	3.153655	3.050132	2.987887	3.052836	3.336532	3.001306	3.063416	3.070211	3.058662	3.025628	3.137152	3.121947	2.727199
STX8	1570357_at	2.618681	2.571594	2.551827	2.565939	2.589879	2.563438	2.569158	2.578678	2.563224	2.556082	2.562767	2.573014	2.531661
DDX3Y	1570359_at	2.949186	2.856984	2.811004	2.844282	2.96646	2.83207	2.859536	2.871333	2.865002	2.833639	2.885902	2.947238	2.604536
DDX3Y	1570360_s_at	2.051643	2.039122	2.013966	2.01765	2.035036	2.017083	2.029759	2.031449	2.030866	2.0124	2.016837	2.026948	2.027261
ZNF709	1570364_at	3.293329	4.118315	3.229535	3.522777	3.275231	3.244243	3.256501	3.268619	3.238142	3.154069	3.25011	3.261711	2.781494
ZNF709	1570366_x_at	2.467916	2.39068	2.346931	2.371115	2.398919	2.638971	2.379603	2.390876	2.375969	2.36608	2.375229	2.385701	2.266337
SELI	1570371_a_at	1.92403	1.913069	1.896516	1.89896	1.906313	1.902373	1.893555	1.895037	1.907476	1.878459	1.89537	1.86242	1.843973
ZNF746	1570373_at	2.000665	1.988896	1.970442	1.971451	1.9866	1.969901	1.982093	1.983816	1.972899	1.967197	1.968916	1.97814	1.913969
LOC154822	1570376_at	2.54934	2.516824	2.490545	2.500917	2.518481	2.510372	2.51218	2.508246	2.4939	2.492465	2.499493	2.50969	2.429352
BMP1	1570383_at	2.301131	2.272027	2.246829	2.250849	2.276148	2.249044	2.266791	2.268351	2.25585	2.238502	2.254094	2.263528	2.211885
LOC440896	1570388_a_at	2.990671	2.972434	2.862841	2.935124	2.97557	2.951669	2.963257	2.954395	2.95558	2.954467	2.949857	2.962366	2.68763
EML5	1570393_at	2.226834	2.171825	2.145117	2.154393	2.201016	2.151743	2.162951	2.171336	2.157675	2.150308	2.157461	2.167325	2.069138
XRN1	1570394_at	2.251589	2.221449	2.190491	2.206181	2.233878	2.208074	2.22223	2.231139	2.213077	2.201112	2.217031	2.228213	2.117281
FAM66C	1570395_a_at	2.228928	2.200942	2.130708	2.185852	2.237698	2.184036	2.195348	2.1942	2.187249	2.161767	2.18265	2.198497	2.196953
FAM66C	1570396_at	2.287682	2.250065	2.215379	2.235048	2.275434	2.235863	2.238455	2.250498	2.238082	2.231046	2.232281	2.2482	2.185826
FAM66C	1570397_x_at	2.16956	2.151552	2.130691	2.134381	2.156036	2.134241	2.146495	2.148217	2.13584	2.130311	2.136097	2.145806	2.090488
STBD1	1570398_at	2.486095	2.455755	2.417539	2.437245	2.471364	2.42842	2.448729	2.452926	2.442083	2.424588	2.449088	2.484302	2.329883
KLC3	1570402_at	1.728444	1.724523	1.700773	1.706593	1.715759	1.703855	1.71735	1.707921	1.713013	1.703525	1.698117	1.701872	1.685588
CYGB	1570410_at	2.143348	2.139439	2.1023	2.116097	2.645782	2.16948	2.122037	2.133964	2.103239	2.116101	2.112798	2.118919	2.067506
FLJ13197	1570414_x_at	3.005159	3.627174	2.360556	2.499912	2.807161	2.444865	2.376987	2.367225	2.157763	2.152705	2.693269	2.095345	2.371748
DDX52	1570415_at	2.678614	2.602068	2.606394	2.611164	2.635641	2.568833	2.768655	2.614994	2.795978	2.605684	2.597699	2.622085	2.552821
STXBP2	1570420_at	2.451626	2.416142	2.398776	2.395931	2.427017	2.557942	2.404303	2.41672	2.401036	2.400445	2.604117	2.564246	2.271748
SNTG2	1570422_at	2.619614	2.543989	2.485542	2.532252	2.617365	2.498958	2.540947	2.550324	2.536307	2.513906	2.545391	2.56885	2.34093
LATS1	1570425_s_at	2.335802	2.302163	2.258134	2.287583	2.327977	2.267526	2.294162	2.301163	2.287606	2.280091	2.288673	2.300796	2.224725

TMPRSS2	1570433_at	2.003328	1.98741	2.05922	1.974097	1.987577	1.967092	1.979517	1.980937	1.97004	1.963118	1.967525	1.974143	1.918209
NAPB	1570441_at	3.169729	3.135334	3.125309	4.123351	3.132653	3.003707	3.11142	2.955698	3.372996	3.107226	3.11362	3.169596	3.194741
LOC643201	1570444_at	3.240427	3.151059	3.032259	3.142845	3.23062	3.115639	3.139513	3.262883	3.152525	3.10831	3.181976	3.226258	2.934984
LOC643201	1570445_a_at	2.208835	2.194546	2.172095	2.175441	2.192091	2.175162	2.189404	2.190744	2.177693	2.181043	2.17265	2.185512	2.130745
FLJ44054	1570447_at	2.683833	2.685254	2.554955	2.635281	2.667767	2.64822	2.665076	2.664382	2.660339	2.639246	2.649201	2.656422	2.591176
C1orf61	1570451_at	2.046212	2.026927	2.00705	2.010917	2.029837	2.010585	2.023249	2.025287	2.016297	2.005574	2.010235	2.019806	1.952942
EIF4EBP2	1570454_at	4.026292	3.362836	3.532542	3.34174	4.069857	4.788469	2.802373	2.830745	2.800845	2.772422	2.804142	2.565976	2.333596
C14orf161	1570470_at	2.834253	2.77589	2.692304	2.738346	2.870661	2.728121	2.770106	2.782921	2.75562	2.736994	2.766177	2.822435	2.473698
ARHGEF12	1570471_at	4.586665	5.089282	4.034573	4.299525	5.105522	4.107495	3.91682	4.324116	4.379784	4.016116	3.873928	4.200443	3.283869
LOC338579	1570473_at	3.55542	3.440248	3.393669	3.444182	3.945911	3.407875	3.376501	3.368408	3.433167	3.353898	3.447273	3.448003	2.926662
LOC338579	1570474_s_at	3.013393	2.938442	2.868846	2.931154	3.012597	2.935139	2.918551	2.931794	2.932777	2.908337	2.937572	2.963055	2.639968
ART1	1570479_at	1.643995	1.636283	1.617188	1.621701	1.63274	1.625657	1.631867	1.634311	1.626191	1.617558	1.619868	1.627159	1.596694
ART1	1570480_s_at	2.47367	2.452455	2.427339	2.431	2.468312	2.433766	2.444816	2.451607	2.43803	2.43036	2.435422	2.447724	2.303934
LOC729954	1570483_at	2.078801	2.196471	2.053667	2.055801	2.146298	2.046097	2.057336	2.063153	2.063513	2.055042	2.054001	2.065156	1.981237
SOX6	1570486_at	3.566714	3.465619	3.325182	3.495654	3.634324	3.528993	3.554041	4.278919	3.525978	3.594588	4.83289	3.93284	3.423787
ABCB4	1570505_at	7.790217	7.656115	8.284649	8.567097	8.830741	8.84778	2.317441	3.535874	2.279816	2.956746	3.179881	3.427401	2.173712
CTNNB1	1570507_at	4.216216	3.891362	4.489163	4.590255	3.930094	3.872236	4.636645	3.798367	4.444682	4.636011	4.064571	3.908412	5.680985
ARHGEF10l	1570511_at	6.89161	7.051893	6.855183	6.516022	7.271073	7.782801	5.536339	6.294416	5.577574	5.209809	6.444018	6.045694	3.877733
FILIP1	1570515_a_at	3.726907	3.626783	3.822112	3.965405	4.014715	3.698607	3.396936	3.561517	3.576632	3.341373	5.051	3.422412	4.792103
OR51B5	1570516_s_at	1.909451	1.892029	1.871634	1.875136	1.890749	1.874229	1.885382	1.887985	1.876052	1.870846	1.873542	1.882537	1.810068
FHL5	1570517_at	2.848351	2.781846	2.749054	2.765179	2.826471	2.736275	2.7715	2.778755	2.767277	2.758097	2.761579	2.777089	2.535001
ATG10	1570523_s_at	3.032053	3.185491	3.164214	3.168056	3.0182	2.947858	4.864122	3.565442	5.123305	5.175755	3.164098	3.78613	4.68179
XYLT2	1570528_at	2.85589	2.827241	2.782818	2.813145	2.82786	2.818267	2.822566	2.823564	2.81284	2.786112	2.806215	2.819366	2.564889
KIAA0020	1570529_at	3.427547	3.380295	3.26292	3.368311	3.38493	3.210921	3.374386	3.380008	3.340915	3.358193	3.363144	3.381259	3.313268
CMPK	1570533_at	2.221234	2.184796	2.15999	2.172382	2.195817	2.167934	2.179769	2.184791	2.175597	2.168213	2.17201	2.192896	2.096351
ZNF483	1570534_a_at	2.960754	2.857306	2.810933	2.866998	3.155194	2.822655	2.883897	2.895927	2.877361	2.853191	2.886654	2.924569	2.634526
LOC729936	1570541_s_at	2.233199	2.200031	2.182686	2.183411	2.217833	2.187009	2.188682	2.191298	2.188163	2.176034	2.182894	2.193792	2.096095
TACC2	1570546_a_at	2.310598	2.285706	2.252803	2.2659	2.292777	2.261015	2.282913	2.283565	2.268801	2.272677	2.2701	2.301551	2.185105
C18orf50	1570552_at	7.036968	6.994304	7.04395	7.443055	5.894135	5.950072	3.699267	4.495614	4.713521	3.588323	4.037416	4.207672	3.408695
CCDC91	1570571_at	7.146931	7.138032	7.522904	7.364283	6.877946	7.163791	5.863904	6.037658	6.773512	5.993764	6.232068	5.879224	5.440834
LOC729291	1570572_at	2.208985	2.186407	2.154654	2.167965	2.190419	2.176235	2.179819	2.18505	2.170156	2.163057	2.169209	2.179186	2.083893
MPZL3	1570584_at	2.842807	2.792149	2.70972	2.780428	2.811439	2.773081	2.774084	2.793066	2.770886	2.726879	2.778267	2.792398	2.639241
MPZL3	1570585_at	3.01889	2.896737	2.792599	2.880122	3.059802	2.848204	2.882472	2.791748	2.891809	2.805158	2.939119	2.981005	2.547708
TCEB3	1570625_at	2.394698	2.364355	2.342086	2.346222	2.363937	2.345418	2.348345	2.361376	2.348165	2.33742	2.344948	2.354559	2.307523
TCEB3	1570627_at	2.568639	2.523963	2.524169	2.513268	2.563717	2.529947	2.522455	2.537113	2.522018	2.507226	2.521528	2.534088	2.356623
CCBL1	1570650_at	1.798965	1.77955	1.761766	1.765905	1.77959	1.762764	1.776023	1.777177	1.767186	1.76304	1.758789	1.770761	1.729957
CCBL1	1570651_at	2.931491	2.841713	2.757309	2.838371	2.903596	2.809488	2.836772	2.840837	3.021512	2.839168	2.835327	2.876232	2.591296
GAS6	1598_g_at	5.604289	5.220234	4.487373	4.776528	5.676934	5.885684	3.108088	3.095349	3.123141	3.09156	4.689897	3.499799	4.477653

MMP14	160020_at	2.407681	2.383523	2.407307	2.372801	2.438599	2.573939	2.418447	2.477543	2.36411	2.44562	2.425718	2.412921	2.85169
TRADD	1729_at	6.271296	6.18684	6.073719	6.097216	6.502581	6.287426	4.25253	4.502911	4.561224	4.513882	4.739014	4.945737	4.063558
FNTB	1773_at	2.035801	2.028963	1.989642	2.001606	2.019563	2.006484	2.69419	2.26185	2.169619	2.023195	2.010432	2.071435	4.162405
PLD1	177_at	4.569196	4.805449	4.871152	5.026221	4.324927	4.140018	4.086153	4.195162	3.708441	4.172445	3.759134	3.587649	3.779277
PMS2L11	179_at	1.902938	1.892865	1.872551	1.876208	1.891513	1.875147	1.887307	1.888923	1.879836	1.871764	1.870547	1.883466	1.882315
BAD	1861_at	4.83722	4.848438	4.567906	4.736237	4.580689	4.442006	3.821895	3.565651	3.808765	4.117278	4.262512	3.966032	4.563478
PRPF8	200000_s_at	8.154534	8.01393	8.260546	8.345074	8.06889	7.773978	9.069079	8.461398	9.079443	9.108515	8.515489	8.76271	8.758752
CAPNS1	200001_at	8.193626	7.888798	8.404867	8.251531	7.305789	6.757772	9.142452	8.205731	8.985473	9.212022	8.325524	8.269475	9.739096
RPL35	200002_at	12.11717	12.18024	12.08348	12.24509	12.26908	12.22793	12.88875	12.89614	12.87155	12.99628	13.13303	13.03692	13.26098
RPL28	200003_s_at	12.81789	12.82166	12.73083	12.8399	12.80888	12.62582	13.86234	13.72252	13.87028	13.90074	13.79825	13.82537	14.13053
EIF4G2	200004_at	11.7795	11.83987	11.77775	11.54931	11.54403	11.09129	12.35184	11.86968	12.36355	12.40883	12.05112	11.92411	13.38548
EIF3D	200005_at	9.765249	9.737217	9.711578	9.684949	9.98331	9.486241	10.99409	10.61413	10.96898	11.00116	10.75778	10.89009	10.59571
PARK7	200006_at	11.5522	11.57744	11.82647	11.87079	11.92242	11.77556	11.84985	12.09614	12.18168	12.07311	12.15335	12.15891	12.17183
SRP14	200007_at	11.2493	11.29026	11.34781	11.44931	11.19458	11.0102	12.25854	11.48855	11.91549	11.71768	11.53595	11.60886	12.83788
GDI2	200008_s_at	6.916233	6.732095	8.10624	7.008501	5.371705	3.087819	9.235797	5.670557	9.479946	9.151878	4.868927	5.835515	10.28113
GDI2	200009_at	12.26218	12.23844	11.87988	11.95864	12.00142	11.5551	12.06734	12.04275	12.3493	12.38963	12.19724	12.04326	12.47965
RPL11	200010_at	12.30124	12.37567	12.63498	12.58806	12.47751	12.19429	13.21865	12.90113	13.39994	13.40571	13.11426	13.01274	13.75391
ARF3	200011_s_at	7.728938	7.706555	7.916731	7.833059	7.819139	7.738484	7.571687	7.437385	7.869417	7.828251	7.531761	7.626704	8.44322
RPL21	200012_x_at	13.71576	13.65489	13.65025	13.85179	14.04587	14.03773	14.13733	14.40175	14.2687	14.2992	14.41629	14.46491	14.2495
RPL24	200013_at	12.42597	12.59607	12.68955	12.67059	12.50078	12.20007	13.41864	13.07045	13.53822	13.55661	13.31262	13.18164	13.9771
HNRNPC	200014_s_at	10.15056	10.19122	10.30801	10.36285	10.56177	10.17426	11.57243	11.36934	11.67372	11.51322	11.36933	11.42594	11.68954
SEPT2	200015_s_at	10.27965	10.23618	10.07919	10.0218	9.701689	9.543097	10.98234	10.22226	10.88972	11.01913	10.61102	10.26692	11.98321
HNRNPA1	200016_x_at	12.56622	12.57174	12.12587	12.16409	12.44601	12.25706	13.56282	13.55765	13.55572	13.4866	13.65586	13.60702	14.00267
RPS27A	200017_at	13.3603	13.33754	13.52147	13.6211	13.49541	13.48742	14.02353	14.13418	13.96567	14.20988	14.18654	14.05377	14.20093
RPS13	200018_at	13.08795	13.06077	12.96183	13.03254	12.9496	12.85462	13.604	13.47419	13.50528	13.6353	13.53546	13.5238	13.83441
FAU	200019_s_at	12.11273	12.16446	12.08712	12.24702	12.44456	12.47575	12.56293	12.6971	12.62638	12.74482	12.79861	12.83146	12.84042
TARDBP	200020_at	10.22092	10.07553	9.517903	9.557171	9.920256	9.844699	10.35331	10.48988	10.27041	10.27731	10.52588	10.47369	10.13128
CFL1	200021_at	11.57491	11.59029	11.23845	11.32048	11.48846	11.87055	12.45941	12.33005	12.22655	12.27604	12.48617	12.49919	12.66335
RPL18	200022_at	12.29592	12.32933	12.32087	12.38569	12.3234	12.104	13.14083	12.99593	13.17885	13.28532	13.08126	13.08587	13.58412
EIF3F	200023_s_at	10.60346	10.60895	10.99325	10.97015	10.78509	10.62514	11.80807	11.67299	11.81786	11.89794	11.9011	11.70473	12.58967
RPS5	200024_at	11.62402	11.51493	12.26741	12.15627	11.5382	11.27118	13.38538	13.03533	13.52883	13.40516	13.01869	13.11779	13.66287
RPL27	200025_s_at	13.59018	13.57079	13.33871	13.49032	13.64286	13.77014	13.93965	14.09005	13.9024	13.93422	14.19133	14.15126	14.03254
RPL34	200026_at	13.63291	13.60596	13.53699	13.64563	13.83817	13.75495	13.76023	13.9681	13.9439	13.93651	14.06607	14.02917	14.06199
NARS	200027_at	10.37799	10.34329	9.991684	10.08457	9.759611	9.549864	11.24622	10.9158	11.10001	11.29922	10.51557	10.96235	10.93193
STARD7	200028_s_at	9.393473	9.249209	9.282238	9.44468	9.416288	9.263326	9.471639	9.756281	9.528006	9.405562	9.691307	9.677767	9.06932
RPL19	200029_at	12.67563	12.78427	12.827	12.93287	12.87814	13.00356	13.4133	13.20927	13.40905	13.47364	13.41186	13.47145	13.75472
SLC25A3	200030_s_at	11.76751	11.79064	11.90662	11.8998	11.62576	11.3442	12.36796	12.12656	12.32883	12.28971	12.21044	12.21295	12.55649
RPS11	200031_s_at	13.37088	13.4189	13.39855	13.45885	13.34979	13.33964	14.00288	13.91126	14.05687	13.93333	13.99384	13.9135	14.55374

RPL9	200032_s_at	13.40643	13.47475	13.47594	13.55654	13.64677	13.44158	14.12455	14.08784	14.05253	14.17244	14.11949	14.07416	14.32747
DDX5	200033_at	11.9062	11.97484	12.61	12.25781	11.40168	10.83423	13.04554	11.73956	12.83326	12.79173	11.9772	11.90207	13.36923
RPL6	200034_s_at	12.57487	12.51098	12.76906	12.82376	12.70735	12.17569	13.64209	13.27991	13.84604	13.67958	13.34318	13.23807	14.42449
DULLARD	200035_at	7.570832	7.605944	7.459055	7.448912	7.644431	7.617389	8.185477	8.717373	7.993788	8.197701	8.831943	8.83442	7.901205
RPL10A	200036_s_at	11.4377	11.31035	11.805	11.94045	11.53509	11.25656	12.9954	12.70875	12.99037	12.98437	12.91686	12.80636	13.76705
CBX3	200037_s_at	9.752376	9.764862	9.479042	9.466164	9.378853	9.368521	11.38912	11.02768	11.46221	11.50816	10.84425	10.91557	11.43715
RPL17	200038_s_at	12.86173	12.83906	13.02645	13.06101	13.10127	12.89168	13.97604	13.71094	14.04191	14.12299	13.81114	13.81715	14.58186
PSMB2	200039_s_at	9.280878	9.265513	9.463945	9.488559	9.55489	9.475742	9.777724	9.667475	9.711976	9.788758	9.66019	9.801253	9.598659
KHDRBS1	200040_at	10.54931	10.50428	10.13237	10.09033	10.14472	9.807249	11.12595	11.00156	11.02305	10.85634	11.15437	10.95873	12.06713
BAT1	200041_s_at	11.2454	11.28079	10.94273	10.92024	10.98065	10.8608	11.91972	11.56841	11.81859	11.82791	11.88829	11.67118	12.61205
C22orf28	200042_at	8.468554	8.440951	8.493504	8.724618	8.241911	8.636652	8.849776	9.322444	9.052051	9.103239	9.419071	9.573342	8.136736
ERH	200043_at	10.56698	10.32176	10.81692	10.65721	10.00715	10.12144	11.64843	10.73512	11.58889	11.57206	11.29728	11.00779	12.1561
SFRS9	200044_at	8.943003	9.01181	9.242333	9.29019	8.868835	8.459854	9.893024	9.627852	9.667563	9.676868	9.439309	9.559988	10.18053
ABCF1	200045_at	7.409906	7.632996	7.651888	7.304814	7.122404	7.119749	8.315628	8.229055	8.478787	8.373148	8.04494	8.459124	7.281609
DAD1	200046_at	9.333399	9.104373	10.10954	10.11113	9.576825	9.089831	11.01382	9.728138	11.02677	11.0346	9.875516	10.19099	11.08727
YY1	200047_s_at	11.75736	11.69652	11.57096	11.65175	11.53975	11.58128	11.99181	12.03904	12.03487	11.96843	11.77237	11.79229	11.84971
JTB	200048_s_at	11.33523	11.22105	11.47152	11.6208	11.49367	11.00725	10.91656	10.60572	11.01522	10.96536	10.5282	10.81768	10.65829
MYST2	200049_at	4.190563	4.565478	5.04997	4.646261	3.822211	2.517716	5.867904	5.123633	6.205674	5.873861	4.245644	5.289462	5.829646
ZNF146	200050_at	10.07099	10.08946	10.25889	10.15718	10.01155	9.753413	10.30644	10.34343	10.7178	10.68818	10.56885	10.35448	11.5507
SART1	200051_at	6.350925	6.509062	6.250591	6.505246	6.772625	6.409843	6.836735	7.464658	6.526557	6.502331	7.455996	7.436782	6.421072
ILF2	200052_s_at	7.503915	7.567476	8.020081	7.700825	7.01728	6.713772	10.3483	9.207229	10.38184	10.18626	9.330092	9.416854	11.13173
SPAG7	200053_at	8.578404	8.598139	8.490478	8.57968	8.855742	8.558919	8.868613	8.724597	8.616527	8.781744	9.036408	8.87779	9.022977
ZNF259	200054_at	6.822892	6.668644	6.592106	6.930314	6.902282	6.936207	6.370793	6.927639	6.488678	6.506082	7.024022	7.274911	5.961289
TAF10	200055_at	8.617606	8.610176	8.79856	9.027612	9.154897	9.238252	9.565415	9.805745	9.576081	9.549448	9.773544	9.9723	8.034127
C1D	200056_s_at	9.592222	9.517568	9.918572	9.793888	9.227589	8.84634	10.30883	10.16924	10.685	10.46618	9.602357	9.912587	9.14786
NONO	200057_s_at	11.28953	11.26258	11.00851	11.0398	10.82417	10.68414	12.15191	11.19544	11.78618	11.94451	11.67613	11.57014	12.75998
ASCC3L1	200058_s_at	10.53291	10.45276	10.39289	10.3675	10.2074	10.26207	11.65053	11.20244	11.55228	11.71661	11.26656	11.15467	11.06862
RHOA	200059_s_at	9.805943	9.842304	10.25678	9.911488	9.134685	8.835129	10.82331	9.845319	10.91706	10.87803	9.884153	9.910311	11.82761
RNPS1	200060_s_at	8.932735	8.964412	8.699663	8.771604	9.028929	8.981095	10.21513	10.0003	9.925538	9.960945	10.23668	10.09479	10.60685
RPS24	200061_s_at	13.64554	13.67426	13.62221	13.71954	13.75724	13.70727	14.48787	14.25018	14.48109	14.45531	14.40495	14.29407	14.77386
RPL30	200062_s_at	13.57904	13.46726	13.65081	13.77635	13.74916	13.69848	13.87806	13.81069	13.94675	13.89485	13.974	13.91022	14.55873
NPM1	200063_s_at	11.83575	11.76089	11.66626	11.61888	11.4356	11.16837	12.69631	12.40218	12.70275	12.66276	12.55489	12.43613	13.14121
HSP90AB1	200064_at	10.84819	10.81128	10.98088	10.9023	10.33675	9.51893	12.68822	11.38054	12.44738	12.71662	11.86394	11.59879	13.14185
ARF1	200065_s_at	9.867744	9.719244	9.919052	9.84387	9.425736	9.10463	10.67856	9.805988	10.64547	10.79561	9.792814	9.931921	11.32587
IK	200066_at	8.223447	8.398703	8.318677	8.372867	8.639663	8.486712	9.20762	9.270192	9.358831	9.640078	8.908905	8.865244	9.204939
SNX3	200067_x_at	10.1554	10.1886	10.6549	10.75414	9.983153	9.749472	11.46845	10.72264	11.37394	11.42637	10.64288	10.78912	11.37012
CANX	200068_s_at	10.06854	10.08161	10.78485	10.5661	10.03269	9.755616	11.50243	10.82963	11.68485	11.65819	10.8155	10.96581	10.96308
SART3	200069_at	6.141547	6.174889	6.151896	6.226613	6.311605	6.601917	7.700492	7.249126	7.090938	7.588846	7.551535	7.60186	7.690971

C2orf24	200070_at	7.448438	7.478461	6.544638	6.821784	7.243558	7.481075	7.678667	8.316294	7.51653	8.057453	8.00313	8.215029	5.19646
SMNDC1	200071_at	9.139096	9.106431	9.254023	9.302874	8.825605	8.49296	10.13645	9.503735	10.25338	10.16975	9.390858	9.602333	10.50896
HNRPM	200072_s_at	9.281101	9.374173	9.567512	9.31159	9.626159	8.993619	10.63783	10.15942	10.54521	10.69195	10.29539	10.35089	11.2119
HNRPD	200073_s_at	10.6601	10.6136	10.50337	10.70229	10.52484	10.43697	12.41818	11.89989	12.39164	12.43919	11.89054	12.04057	12.54057
RPL14	200074_s_at	11.29186	11.38031	11.58346	11.46651	11.31366	11.04403	12.74047	12.21048	12.84294	12.73724	12.23385	12.30957	13.02916
GUK1	200075_s_at	9.365658	9.271934	9.740489	9.82879	9.343653	9.147716	10.26228	10.10292	10.47607	10.57871	9.730784	10.12776	9.536805
C19orf50	200076_s_at	5.433058	5.381862	5.359385	5.504728	5.725899	5.901642	6.583373	6.585669	6.462284	6.726549	6.470955	6.787137	6.082607
OAZ1	200077_s_at	12.39904	12.3554	12.38678	12.43107	12.60629	12.58864	13.24893	13.33384	13.35101	13.48203	13.15003	13.24873	12.83163
ATP6V0B	200078_s_at	9.227914	9.214364	8.788513	8.91034	9.015704	8.524804	9.384147	8.826483	9.477969	9.637581	8.98924	9.366299	9.068649
KARS	200079_s_at	8.878127	8.839705	9.035382	8.935087	8.857116	8.578403	9.783944	9.367183	9.807361	9.877505	9.393786	9.323323	9.406295
H3F3A	200080_s_at	12.7978	12.79095	12.86604	12.876	12.63475	12.46293	13.8101	13.70263	13.71539	13.83854	13.42942	13.60547	13.79515
RPS6	200081_s_at	12.49844	12.5737	12.49933	12.34491	12.76753	12.94711	13.92759	13.6158	13.88661	13.87983	13.8091	13.77488	14.51548
RPS7	200082_s_at	11.37633	11.40833	11.7471	11.56943	10.91259	10.60158	12.80473	11.81174	12.70478	12.71956	12.12384	12.04279	13.34107
USP22	200083_at	9.675091	9.689754	9.334908	9.268988	9.208171	9.2029	9.925554	9.965848	9.842072	9.903145	10.07405	9.868683	10.45137
C11orf58	200084_at	10.5867	10.56316	10.60286	10.64624	10.31945	10.26545	11.08744	10.8011	10.95059	11.1739	10.77504	10.79015	11.58952
TCEB2	200085_s_at	11.15798	11.21468	10.90718	11.26903	11.52287	11.58858	11.46546	12.14329	11.75844	11.7517	12.26616	12.29852	11.65339
COX4I1	200086_s_at	12.13615	12.16109	11.68157	11.89307	12.00419	12.11948	11.68061	12.05572	11.74967	11.77754	12.11759	12.19852	11.70163
TMED2	200087_s_at	12.09997	12.19124	12.21987	12.36238	12.15626	11.81516	12.23959	12.19403	12.20414	12.31186	12.25698	12.24837	12.3619
RPL12	200088_x_at	12.95824	12.93468	12.73606	12.83199	13.22983	13.33023	13.64135	13.68723	13.62959	13.86962	13.63455	13.82877	13.75495
RPL4	200089_s_at	12.63285	12.73481	12.61922	12.78741	12.99419	12.95266	13.91505	13.95152	14.00947	14.08495	13.7575	13.72176	14.33487
FNTA	200090_at	10.72144	10.78101	10.30639	10.39725	10.57357	10.67285	10.59791	10.7403	10.61647	10.66035	10.82588	10.64192	11.23364
RPS25	200091_s_at	13.20164	13.23314	13.42395	13.41716	13.25229	13.11441	13.62419	13.66145	13.69114	13.69063	13.59939	13.50777	14.13941
RPL37	200092_s_at	13.83004	13.76627	13.52248	13.78135	13.87862	13.91159	13.89411	14.27761	13.96272	13.9923	14.16319	14.14237	14.38502
HINT1	200093_s_at	11.9554	11.81636	12.07277	12.31681	11.94377	11.65767	11.81493	11.4518	11.73484	11.81437	11.44045	11.65588	11.51313
EEF2	200094_s_at	13.01328	12.9037	13.04294	12.8976	13.03289	12.77592	13.26398	13.07015	13.32562	13.28627	13.1577	13.10052	13.60382
ATP6V0E1	200096_s_at	9.329646	9.221977	9.84783	9.83	9.257535	8.668956	9.388113	8.637231	9.314898	9.236827	8.780052	8.862151	9.539849
HNRPK	200097_s_at	11.37829	11.24435	11.75198	11.53797	11.10532	10.42556	12.16542	11.35468	12.3908	12.41865	11.32251	11.28615	12.53451
ANAPC5	200098_s_at	10.18738	10.13688	10.13169	10.28825	10.06616	10.15479	10.76935	10.45285	10.75393	10.59809	10.66584	10.62141	10.58931
HNRNPU	200593_s_at	7.280394	7.519967	8.102385	7.519698	7.788409	7.726967	9.666752	8.746301	9.443559	9.492678	8.613314	8.764363	10.64907
HNRNPU	200594_x_at	10.78009	10.81763	10.70209	10.45606	10.41207	9.918213	12.56157	11.884	12.5601	12.40801	11.85422	12.01769	12.75062
EIF3A	200595_s_at	11.15521	11.15385	10.71497	10.84924	10.6281	10.16634	11.58872	11.40473	11.63329	11.72544	11.10466	11.36253	11.24646
EIF3A	200596_s_at	9.908531	9.740426	9.724837	9.804969	9.795781	9.490143	10.37701	10.05917	10.22938	10.32446	10.10644	10.35607	10.04897
EIF3A	200597_at	7.758314	7.727357	7.534424	7.404207	7.731287	7.746444	8.016217	7.7327	8.412905	8.021771	7.825392	7.752954	7.291003
HSP90B1	200598_s_at	8.769606	8.330256	10.05486	10.05159	9.708649	9.438364	9.720952	8.924201	9.908218	9.76296	9.352608	9.557525	10.31779
HSP90B1	200599_s_at	12.53284	12.59088	12.56598	12.75869	12.75678	12.53355	12.69351	12.64657	12.79857	12.81482	12.72132	12.75972	12.61687
MSN	200600_at	9.613895	9.497367	9.175557	8.97871	9.411566	9.244571	9.385819	8.906932	9.30027	9.593526	9.337879	9.34095	10.47701
ACTN4	200601_at	8.598747	8.445271	7.938101	8.126108	8.671491	8.619543	8.247989	8.402391	8.047628	8.569451	8.53915	8.729729	7.550735
APP	200602_at	11.38215	11.21021	11.03866	11.18937	11.18706	11.18083	8.994349	8.750595	7.65152	8.151842	10.41739	9.180259	11.2933

PRKAR1A	200603_at	12.32007	12.29158	12.27583	12.22315	12.07125	11.8759	12.20144	12.22788	12.29266	12.57077	12.2703	12.15947	12.75798
PRKAR1A	200604_s_at	7.114009	7.225844	7.67368	7.532814	7.091428	6.288076	7.620004	6.871267	7.911926	7.433398	7.62991	6.839949	9.973647
PRKAR1A	200605_s_at	9.655955	9.163261	10.58733	9.947595	8.705953	7.918946	10.83128	8.601004	10.98809	10.9877	9.094476	8.691735	12.27474
DSP	200606_at	10.00235	9.846382	10.06047	9.7532	9.682483	9.557215	9.51748	8.846201	9.627149	9.551928	8.221475	8.805695	8.913295
RAD21	200607_s_at	6.441987	6.144406	6.760488	6.740614	6.634663	6.59528	8.926776	7.934366	8.353436	8.337158	7.910597	8.019308	8.972162
RAD21	200608_s_at	10.48266	10.47138	10.43478	10.36487	9.708445	9.383035	11.95969	11.49857	11.65757	11.99185	11.34289	11.25798	11.76478
WDR1	200609_s_at	8.481769	8.392618	8.714265	8.666714	8.181077	7.915243	9.966465	9.134198	9.817275	9.816254	9.110337	9.673699	9.831271
NCL	200610_s_at	10.45482	10.43629	10.2353	10.08203	10.46696	10.38134	11.88354	11.58912	11.92884	12.00128	11.56591	11.49083	11.90431
WDR1	200611_s_at	8.456616	8.276867	8.709809	8.824648	8.037595	7.971337	9.848241	8.912594	9.550584	9.492626	9.276629	9.394897	10.12295
AP2B1	200612_s_at	5.828967	6.124868	6.237961	6.00615	6.6987	6.657392	7.683551	7.841045	6.731215	8.491873	7.278556	8.136347	7.999558
AP2M1	200613_at	8.405529	8.196764	8.820185	8.717303	8.192862	8.174814	10.24643	9.399491	10.20211	10.55487	9.478793	9.640625	9.81609
CLTC	200614_at	11.27978	11.23894	11.52256	11.38798	10.96979	10.43471	12.89362	12.24247	12.88413	12.86151	12.15092	12.18556	12.41028
AP2B1	200615_s_at	3.004823	3.173216	4.305584	4.178196	3.399704	3.454004	7.044697	6.23534	7.176917	6.93381	6.000549	6.312582	6.766443
KIAA0152	200616_s_at	8.628741	8.285944	8.681041	9.044402	9.609764	9.78801	7.95647	8.45558	7.731488	7.679363	8.64626	8.711996	8.096595
KIAA0152	200617_at	7.64891	7.862659	8.299906	7.992897	8.238176	7.888721	8.03205	7.895001	8.3005	8.336393	8.049201	8.010987	8.036084
LASP1	200618_at	9.981742	9.9629	9.312212	9.099766	9.49268	9.290849	10.25175	10.23756	10.23088	10.11044	10.1121	10.22957	10.05876
SF3B2	200619_at	8.048038	7.94716	7.758675	7.649551	7.942004	8.001542	8.323371	8.576386	8.618408	8.237216	8.881738	8.954395	8.223812
TMEM59	200620_at	11.24535	11.26381	11.88755	11.76639	11.21032	11.02065	10.62743	10.58656	10.84104	10.95305	10.26851	10.5335	10.3745
CSRP1	200621_at	8.653879	8.725382	8.950261	8.840855	9.054912	9.042284	8.074108	8.188729	7.824986	8.14573	8.179823	8.168061	8.159179
CALM3	200622_x_at	6.6611	6.610557	6.782553	6.947037	7.04329	7.253971	9.499856	9.203251	9.210242	9.484727	9.252481	9.390984	8.275511
CALM3	200623_s_at	7.026659	7.04005	6.892494	7.084547	7.40073	7.55589	8.466474	8.725542	8.463178	8.398188	8.889466	8.835589	7.921519
MATR3	200624_s_at	8.433774	8.403614	9.364941	8.808111	7.848013	7.931269	9.941892	8.298483	10.10343	10.01532	8.831289	8.579255	11.296
CAP1	200625_s_at	9.44805	9.212317	9.356459	9.392881	9.483082	9.286348	9.489487	9.539269	9.407137	9.588399	9.644455	9.779962	9.347392
MATR3	200626_s_at	9.756573	9.676254	10.2442	10.10377	9.506685	8.804491	11.0931	10.18965	11.06904	11.26358	10.23101	10.08419	12.24938
PTGES3	200627_at	11.18674	11.1964	11.69192	11.48704	11.19067	10.81311	12.6753	11.72578	12.71939	12.67932	11.53668	11.85887	12.59979
WARS	200628_s_at	2.974383	2.926103	2.954382	2.948417	2.736467	2.580346	3.262735	2.960107	4.148604	4.268826	2.680912	2.946997	4.145621
WARS	200629_at	6.417947	6.319944	5.882648	6.071042	7.222992	8.029051	5.511974	6.608357	5.77103	5.938789	6.760109	6.568127	6.029573
SET	200630_x_at	9.48067	9.618593	9.046819	9.123726	9.614485	9.511652	10.11868	10.3006	9.889977	10.11248	10.37309	10.45933	9.638947
SET	200631_s_at	9.546241	9.433407	9.572358	9.618297	9.478007	9.023667	10.75568	10.33685	10.80035	10.83045	10.55082	10.49017	11.49425
NDRG1	200632_s_at	8.690328	8.701686	9.634014	9.856624	10.12851	10.04256	8.004872	8.814477	8.68168	8.743452	8.740798	8.789737	7.916562
UBB	200633_at	13.80302	13.72642	13.95303	14.14097	13.99085	13.86849	14.12021	14.02088	14.08812	14.22849	13.93845	14.01028	13.80918
PFN1	200634_at	10.85364	10.98439	10.93464	10.82248	10.45614	10.30839	12.07787	11.42512	11.74457	11.92647	11.68	11.75825	11.47029
PTPRF	200635_s_at	8.482537	8.333302	7.957866	8.28261	8.463959	8.465398	6.788731	6.703593	6.307197	6.165407	7.202713	7.402526	6.083891
PTPRF	200636_s_at	10.89173	10.97272	10.50659	10.68925	10.43025	10.26292	8.935042	9.083764	8.957922	9.178306	9.183209	9.393971	8.821646
PTPRF	200637_s_at	6.252956	6.115451	7.036943	6.81369	5.917909	5.57642	4.971817	3.535308	4.129262	5.627108	3.542797	3.550932	5.244664
YWHAZ	200638_s_at	7.31313	7.216505	8.397384	7.563267	7.114624	6.369937	9.799749	7.387085	9.531944	9.488582	8.654702	7.857339	12.18038
YWHAZ	200639_s_at	10.0205	10.11176	10.1493	9.919449	9.438809	9.038701	10.8255	10.37584	10.71856	10.72718	10.60385	10.65395	11.98118
YWHAZ	200640_at	8.337364	8.283348	8.977201	8.352132	7.020438	6.259128	10.16029	8.139118	9.972745	9.998767	8.523283	8.59371	11.84538

YWHAZ	200641_s_at	4.649399	4.900073	5.914091	4.972106	4.452132	4.448532	7.615681	4.403369	7.551006	7.507731	4.525268	4.873118	11.45817
SOD1	200642_at	13.78365	13.77429	13.91432	14.0173	14.15718	13.99441	12.8081	12.54577	12.78857	12.89615	12.21772	12.52626	11.64563
HDLBP	200643_at	8.959178	9.080674	9.566235	9.518153	9.172449	8.712517	9.462685	8.864554	9.395674	9.468541	9.101224	9.115055	9.244549
MARCKSL1	200644_at	8.198931	8.289005	8.599832	8.259069	8.370285	8.213989	11.16261	10.28089	10.54336	10.63774	10.75339	10.63829	12.10473
GABARAP	200645_at	10.92376	10.85684	10.91373	11.00959	10.94274	10.78735	11.1449	10.9574	10.98433	11.29208	10.97484	10.98378	11.61343
NUCB1	200646_s_at	6.964235	6.867799	7.803009	7.576729	6.479369	5.81812	7.208195	5.280464	7.53877	7.490085	5.707115	5.873501	6.816053
EIF3C	200647_x_at	9.94189	9.857157	10.25162	10.24506	10.04373	10.31779	10.70889	9.861332	11.43875	10.12005	11.47663	10.75839	10.99122
GLUL	200648_s_at	10.03911	9.594257	9.829831	9.447795	8.842966	8.714773	10.07661	9.105481	9.906134	9.933566	8.054717	9.081885	9.697098
NUCB1	200649_at	9.856651	9.817737	10.01365	10.0527	9.684234	9.460855	9.107547	8.673792	9.051551	9.259156	8.904331	9.089576	8.080125
LDHA	200650_s_at	12.22689	12.25522	12.63517	12.42904	12.13449	11.78996	12.17505	11.41378	12.1377	12.36139	11.33673	11.33111	12.8807
GNB2L1	200651_at	11.959	12.0165	11.87261	11.96604	11.84413	11.4483	13.48548	12.87036	13.44507	13.38802	13.13541	13.15454	14.11679
SSR2	200652_at	8.882476	8.843953	8.959272	8.977863	9.149412	9.1717	9.807187	9.693768	9.704925	9.856144	10.16894	9.915847	10.90241
CALM1	200653_s_at	11.62587	11.66819	11.90379	11.82344	12.35696	12.26008	11.32668	11.47359	11.71399	11.52259	11.72475	11.3757	12.30169
P4HB	200654_at	11.60564	11.65539	11.53795	11.66321	11.54624	11.38389	11.55436	11.45173	11.54733	11.53589	11.44463	11.55053	11.10698
CALM1	200655_s_at	11.28622	11.19571	11.19129	11.19656	11.74516	11.59727	10.78934	11.00546	11.14101	11.03955	11.0402	11.047	11.56356
P4HB	200656_s_at	11.43825	11.40815	12.15426	11.65927	10.64037	9.681271	12.25791	10.25238	12.18099	12.39472	10.54919	10.73181	11.49686
SLC25A5	200657_at	11.96863	11.9763	11.65193	11.78359	11.28585	11.02933	12.25042	11.74537	12.19863	12.23648	11.72783	11.99403	11.44207
PHB	200658_s_at	7.577759	7.83546	8.263555	7.979153	7.545824	7.108477	8.770366	8.247028	8.691974	8.901874	8.524601	8.340591	9.436437
PHB	200659_s_at	8.027554	7.923083	8.112554	8.270649	7.842944	7.555679	7.737761	7.510165	7.630951	8.058831	7.958521	8.001746	6.848266
S100A11	200660_at	5.953153	6.004445	6.612839	6.387386	6.41542	6.335898	6.395048	6.2796	6.392299	6.400166	7.118147	6.448455	10.04793
CTSA	200661_at	9.601145	9.67081	8.927608	8.881397	8.874096	8.669052	9.387964	8.561656	9.102007	9.113463	8.61563	8.842565	7.686538
TOMM20	200662_s_at	10.41568	10.55124	10.83197	10.51968	10.4137	9.411075	11.29368	10.57217	11.43815	11.21595	10.86438	10.65726	12.30225
CD63	200663_at	11.66144	11.60872	11.52545	11.56422	11.66862	11.63927	11.64356	11.5951	11.53577	11.59051	11.85866	11.7488	12.19765
DNAJB1	200664_s_at	6.249173	6.277405	7.459138	7.173214	6.961974	6.578969	7.732026	7.097839	7.917195	7.799305	6.540649	7.117893	7.327125
SPARC	200665_s_at	10.3781	10.52058	10.38456	10.08585	9.55571	9.05713	10.25126	9.123912	9.822048	9.859428	11.73737	9.859096	13.68111
DNAJB1	200666_s_at	7.382436	7.32819	7.652905	7.219375	7.660746	7.223803	8.290328	8.048305	8.662413	8.326241	7.723718	8.261203	7.765995
UBE2D3	200667_at	10.64081	10.60164	10.97836	11.05026	10.77601	10.26716	10.91336	10.55119	10.94683	10.96222	10.42687	10.64728	10.57008
UBE2D3	200668_s_at	10.15486	10.10829	10.7264	10.54924	9.97149	9.31767	11.17689	10.25979	11.29562	11.12438	10.02656	10.25514	11.17791
UBE2D3	200669_s_at	6.381136	6.104873	7.507412	6.831432	6.241346	6.070832	8.38119	7.222647	8.41874	8.372487	6.71359	7.089521	8.234159
XBP1	200670_at	10.51655	10.54028	10.76698	10.32606	9.850426	8.941396	9.985028	8.919461	10.13594	9.914127	8.410071	8.817329	8.671339
SPTBN1	200671_s_at	5.493094	4.951916	4.576398	4.534357	4.507404	4.537158	3.683094	3.967445	3.160592	3.668704	4.531043	4.18457	5.907852
SPTBN1	200672_x_at	7.307018	7.199551	6.591982	6.098377	5.834809	6.030964	7.016018	5.330446	6.154114	6.219519	5.818969	5.724353	9.833503
LAPTM4A	200673_at	12.27691	12.16614	12.54091	12.386	12.05406	11.7375	12.21637	11.5544	11.93504	12.06303	12.01406	11.69533	13.27071
RPL32	200674_s_at	12.88698	12.90297	12.70204	12.92624	13.2453	13.18014	13.52691	13.47033	13.41662	13.63724	13.50437	13.45559	13.94877
CD81	200675_at	11.19763	11.13646	12.13427	12.16687	11.87263	11.82781	9.305902	9.089847	9.165577	9.18128	10.33003	9.446817	11.24465
UBE2L3	200676_s_at	5.417452	5.51892	5.208382	5.327045	5.434055	5.513194	6.000764	6.127239	6.031715	5.763992	5.839385	6.551171	6.406955
PTTG1IP	200677_at	9.764432	9.850618	10.16576	10.23531	9.208581	9.407245	9.773498	9.178232	9.329409	9.862976	9.686988	9.957467	10.32303
GRN	200678_x_at	8.629476	8.572983	8.544347	8.602243	8.582584	8.764997	8.148347	7.545947	7.517876	7.48642	7.736572	7.878048	7.763127

HMGB1	200679_x_at	10.04826	9.834465	10.5572	10.5949	10.56577	10.45409	12.37266	11.4423	12.28032	12.238	11.66773	11.72903	12.706
HMGB1	200680_x_at	12.68046	12.70857	12.55797	12.62455	12.8147	12.90783	13.90157	13.8591	13.87766	13.96888	13.73789	13.79218	13.62088
GLO1	200681_at	10.28815	10.36867	10.87962	10.86382	10.72321	10.45455	11.73406	10.88103	11.91863	11.96222	10.8232	11.08023	10.71598
UBE2L3	200682_s_at	8.649079	8.634448	9.003274	8.873307	7.953707	7.489536	10.23117	9.358156	10.11483	10.20324	9.396969	9.613207	9.985708
UBE2L3	200683_s_at	6.760852	6.689725	6.910135	6.898338	6.379673	6.071281	7.95413	6.898402	7.751185	7.930075	7.16929	7.012642	9.100871
UBE2L3	200684_s_at	5.001351	4.983331	5.135417	5.208704	5.334418	5.318268	6.160766	6.08524	6.262843	6.322474	6.464204	6.290155	6.799993
SFRS11	200685_at	8.705355	8.721067	8.586233	8.928271	8.784731	8.425955	8.227316	8.099924	8.390434	8.238394	8.171201	8.493048	8.764166
SFRS11	200686_s_at	11.57644	11.68278	11.48874	11.34517	11.25549	11.43419	11.49029	11.50212	11.52962	11.4752	11.51199	11.49605	11.89563
SF3B3	200687_s_at	7.678324	7.53038	7.502669	7.471892	7.591574	7.226263	9.087146	8.827706	9.005081	8.976601	8.784738	8.818077	8.48411
SF3B3	200688_at	4.283077	4.43657	4.113206	4.555333	4.356495	4.883421	4.471508	5.105291	4.976538	4.863932	5.225559	5.549587	4.8894
EEF1G	200689_x_at	12.21279	12.2253	12.07098	12.09968	12.20757	11.84779	12.97696	12.88241	13.12035	13.03399	13.00765	13.01476	13.41933
HSPA9	200690_at	7.99059	7.851975	7.848493	8.267159	7.713514	7.767963	7.28923	7.73955	7.44257	7.221543	7.404676	7.542469	5.950457
HSPA9	200691_s_at	10.48306	10.57915	10.60805	10.45341	10.11118	10.03549	10.79415	10.17903	10.81674	10.71967	9.959647	10.18371	9.704129
HSPA9	200692_s_at	10.07685	9.811801	10.20893	10.22252	10.0289	10.13278	10.17867	9.858336	10.00416	10.034	9.75154	9.778956	9.558999
YWHAQ	200693_at	9.666859	9.675729	10.39182	10.18724	9.528026	9.169346	11.60711	10.71333	11.76461	11.82994	10.80881	10.64717	12.49899
DDX24	200694_s_at	8.478083	8.404339	8.515749	8.695986	8.64891	8.794644	8.470994	8.797255	8.710555	8.646929	8.769668	8.844928	8.74396
PPP2R1A	200695_at	6.998927	6.842302	7.070942	7.098304	6.730395	5.966264	7.150487	6.906756	7.429873	7.449335	7.756661	7.530069	9.353634
GSN	200696_s_at	7.334229	7.743415	8.270903	7.83971	8.590049	8.751865	12.45257	12.03399	11.73256	11.8059	11.71599	12.079	10.24124
HK1	200697_at	7.142899	7.063829	6.841608	6.645277	7.5	8.136285	12.61825	12.70624	12.69483	12.68693	12.44487	12.83428	10.03816
KDELR2	200698_at	9.078093	8.946522	9.987256	9.638948	8.510552	8.104185	10.55689	9.136683	10.43936	10.37385	9.174236	9.083252	10.96061
KDELR2	200700_s_at	8.894892	8.749979	9.559178	9.346226	8.750097	8.724439	10.04944	9.731106	10.13557	9.926245	9.842568	9.620626	10.45409
NPC2	200701_at	8.864232	8.884393	9.458327	9.459083	9.295411	9.344267	9.9041	9.344964	9.431639	9.534893	9.506376	9.552391	9.660559
DDX24	200702_s_at	9.40151	9.293023	9.078748	9.383609	10.31842	10.33528	8.564315	9.430955	8.27636	7.753596	9.649167	9.747153	8.209278
DYNLL1	200703_at	9.871484	9.736598	10.30528	10.16226	9.836295	9.580508	12.17333	11.3566	12.32394	12.29051	11.47543	11.36389	12.37303
LITAF	200704_at	10.46551	10.38817	9.918048	10.05277	9.503508	9.317866	5.838828	5.27221	4.78306	5.251208	6.510139	5.488765	9.240955
EEF1B2	200705_s_at	12.07759	12.00179	12.25371	12.23826	12.23178	11.94993	13.27842	13.12942	13.33959	13.30809	13.00886	13.12036	12.86758
LITAF	200706_s_at	8.42837	8.472031	8.673889	8.258264	7.053343	6.556573	3.784488	3.21401	3.158278	3.668241	4.077691	3.339572	7.409578
PRKCSH	200707_at	9.094149	8.980118	8.841942	8.875491	8.778814	8.173636	8.789595	8.520266	8.507464	8.459492	8.545671	8.306186	8.623912
GOT2	200708_at	10.76231	10.65668	10.86859	10.88861	10.50173	10.05519	9.811252	9.106884	10.13795	9.669567	9.059634	9.149535	7.553941
FKBP1A	200709_at	9.03499	8.956189	8.97882	8.978448	8.666141	8.407342	9.971536	9.425821	9.893205	10.32714	9.461986	9.457828	10.24134
ACADVL	200710_at	11.41279	11.46622	10.94914	11.02929	11.75917	11.58542	10.15231	9.893735	9.921007	9.855014	10.05744	9.831636	8.354276
SKP1A	200711_s_at	9.609188	9.400581	9.493624	9.617552	9.553727	9.543792	9.918268	9.614651	9.629611	9.799442	9.492393	9.627577	10.27336
MAPRE1	200712_s_at	4.560139	4.594274	5.746416	5.004951	4.580884	4.584894	7.851711	6.471985	8.002249	8.13928	5.941113	6.407861	9.306934
MAPRE1	200713_s_at	6.513771	6.428376	7.141379	6.895156	6.316978	6.179851	8.932304	8.330617	8.936085	9.208453	8.409254	8.282741	10.15942
OS9	200714_x_at	10.41516	10.53625	10.19532	10.3331	10.52346	10.69713	9.434271	9.98142	9.254683	9.357823	9.623892	10.03388	9.004421
RPL13A	200715_x_at	10.9108	10.85108	10.83036	10.9449	11.13867	11.28285	11.5792	11.54833	11.86267	11.84813	11.93346	11.74055	12.22143
RPL13A	200716_x_at	12.90839	12.9677	13.05461	13.05889	12.94023	12.99508	13.88671	13.49496	13.85025	13.76683	13.72949	13.64589	14.28632
RPL7	200717_x_at	13.54717	13.58799	13.4579	13.58675	13.65743	13.6719	14.13953	14.06902	14.11377	14.10908	14.22908	14.21181	14.26966

SKP1A	200718_s_at	12.56974	12.62283	12.65443	12.72959	12.74004	12.7189	13.21566	13.2993	13.34107	13.27049	13.23255	13.25528	13.26413
SKP1A	200719_at	5.991172	5.991044	6.716581	6.340876	6.186662	5.587135	6.181131	5.984833	6.552774	6.226451	5.952793	6.060994	6.289721
ACTR1A	200720_s_at	5.771879	5.710025	5.675138	5.902383	6.280489	6.481309	7.735219	7.68114	7.564507	7.957454	7.75557	7.868936	8.954277
ACTR1A	200721_s_at	6.598481	6.660989	6.571535	6.388208	6.645514	6.620374	7.304409	7.81069	8.053046	7.839488	7.966624	7.901452	8.134414
CAPRIN1	200722_s_at	4.276854	4.23754	4.498815	3.828147	3.729219	4.349418	6.000881	4.775074	6.004864	6.264366	4.507471	4.648469	8.099452
CAPRIN1	200723_s_at	7.964138	7.837437	8.158848	7.891645	7.283319	7.137665	10.12406	8.06225	10.25239	10.10542	8.710078	9.485307	11.68846
RPL10	200724_at	2.510759	2.49903	2.481881	2.487163	2.49171	2.490689	2.499425	2.486761	2.485326	2.481881	2.484072	2.494639	2.350343
RPL10	200725_x_at	12.27128	12.13409	12.45366	12.43817	12.40753	12.21118	13.1278	12.94023	13.10218	13.14432	13.06313	13.04367	13.5995
PPP1CC	200726_at	10.22062	10.10198	10.30417	10.20598	10.01848	9.863533	11.515	11.04788	11.54991	11.46566	11.03932	11.19834	11.47134
ACTR2	200727_s_at	3.976196	4.151799	4.37941	4.492308	4.431084	5.041028	4.412823	4.417036	4.087138	4.13035	5.498901	5.404216	5.402119
ACTR2	200728_at	9.338486	9.282224	9.868756	9.558095	9.20268	8.489634	10.07142	9.009349	9.987646	9.966758	9.560873	9.270238	11.41749
ACTR2	200729_s_at	8.904936	8.717855	8.622078	8.01279	8.778624	8.851897	9.738232	8.668453	9.361674	9.295506	8.187692	8.815331	11.16992
PTP4A1	200730_s_at	7.386417	6.878812	9.331731	8.162328	6.457843	5.077102	6.461353	4.301229	7.024761	7.380713	2.737907	3.58723	5.708281
PTP4A1	200731_s_at	9.319653	9.345208	10.28198	9.768558	8.644085	8.050871	7.909056	6.93349	8.666748	8.744024	6.078961	6.780064	7.793469
PTP4A1	200732_s_at	12.21635	12.23445	12.70597	12.70591	11.85443	11.46262	10.73712	10.24199	11.1643	11.27323	9.52531	10.22249	9.624422
PTP4A1	200733_s_at	11.91748	11.76495	12.10063	11.96791	11.36543	11.29025	10.15174	10.10721	10.63926	11.01557	9.745282	9.955463	9.514549
ARF3	200734_s_at	7.753843	7.68619	7.880103	8.193301	8.342802	8.590967	7.464455	7.88803	7.490255	7.181487	8.411867	8.163301	7.626621
NACA	200735_x_at	12.39718	12.42319	12.78359	12.76514	12.51033	12.11782	12.92881	12.87516	12.91483	13.04797	13.03002	12.91558	13.95889
GPX1	200736_s_at	11.01193	10.98051	11.33133	11.39726	11.13047	11.21782	11.66538	11.21752	11.07318	11.23562	11.24684	11.57036	10.33269
PGK1	200737_at	6.30222	6.13125	7.60932	7.443552	6.044372	5.275919	8.14138	6.550531	8.317754	8.37708	6.78073	6.579573	8.972764
PGK1	200738_s_at	10.49655	10.50545	11.12024	10.9927	10.57333	10.21824	11.61601	10.89136	11.76969	11.77615	10.84237	11.0243	11.77681
SUMO3	200739_s_at	5.492681	5.45597	6.09744	6.102995	5.924841	6.04844	6.948061	6.479586	6.641921	6.974117	6.316857	6.660134	6.75812
SUMO3	200740_s_at	9.475768	9.337668	9.777725	9.656829	9.416672	9.089591	10.63613	10.23512	10.67744	10.72371	10.25308	10.23869	10.85962
RPS27	200741_s_at	13.95868	13.9348	14.0723	14.17261	14.02833	13.86378	14.24002	14.09305	14.25607	14.24002	14.20908	14.19686	14.78283
TPP1	200742_s_at	6.266188	6.175942	7.633115	6.934013	6.191401	5.886057	6.843019	5.390814	6.841505	6.863852	3.811114	5.378072	5.831821
TPP1	200743_s_at	10.40982	10.40307	10.27277	10.19853	10.0978	9.882096	10.48934	9.93972	10.49433	10.44459	10.04209	10.17411	8.367754
GNB1	200744_s_at	7.185171	6.762271	7.465678	7.212361	6.450937	5.945105	8.578682	7.764659	8.674909	8.612019	7.599132	7.836297	9.969089
GNB1	200745_s_at	9.115367	8.885461	9.588972	9.233422	8.763619	8.568864	9.99268	8.408116	9.587174	9.391384	9.051359	9.081318	11.49312
GNB1	200746_s_at	9.700101	9.554072	9.368726	9.442698	9.434711	9.502688	9.702493	9.546232	9.441868	9.653049	9.526681	9.458173	10.92905
NUMA1	200747_s_at	8.043013	8.044672	7.790406	7.8752	8.245307	8.418373	7.995196	8.335635	7.842142	8.144404	8.437687	8.465058	8.326365
FTH1	200748_s_at	13.79813	13.76225	14.0308	14.13408	13.91141	13.57136	14.51727	14.28624	14.50738	14.57555	14.01805	14.2885	13.27457
RAN	200749_at	9.476119	9.534148	9.514582	9.521972	9.566696	9.320615	10.62119	10.5779	10.44666	10.59104	10.80804	10.42662	10.99461
RAN	200750_s_at	9.420977	9.351278	9.590942	9.518336	8.900058	8.143608	12.81101	11.7037	12.74006	12.82496	11.9826	12.03024	12.54246
HNRNPC	200751_s_at	8.753385	8.50735	9.516217	8.935082	8.420269	8.231985	11.24449	8.656634	11.51459	11.23821	9.152749	9.329009	12.6429
CAPN1	200752_s_at	5.56027	5.570076	5.573746	5.720742	5.328779	5.183854	5.779523	5.567116	6.421153	6.537541	5.966253	6.077488	5.666734
SFRS2	200753_x_at	6.796222	6.808725	7.149331	6.684803	6.395233	5.750315	9.830034	8.507977	9.746585	9.752135	7.999446	8.593653	9.437885
SFRS2	200754_x_at	9.475208	9.464548	9.614027	9.323074	8.912373	8.794208	11.72023	10.73616	11.65754	11.70684	10.70678	10.7393	11.24828
CALU	200755_s_at	6.723934	6.599949	7.273398	6.970045	5.944841	5.799921	8.404613	6.762613	8.009649	8.256007	7.665444	7.018459	10.1572

CALU	200756_x_at	3.286203	3.769763	4.039931	3.755722	3.228658	3.302711	5.485528	3.961828	5.130476	5.278742	3.754887	4.081802	8.658211
CALU	200757_s_at	6.935153	7.077954	7.466288	7.149954	6.73107	6.206774	8.526415	7.764897	8.425734	8.621737	8.554509	8.092217	10.15938
NFE2L1	200758_s_at	8.494976	8.556772	8.702157	8.605101	8.464955	8.284589	7.054691	6.925502	7.267198	7.330613	7.002934	6.730655	8.616606
NFE2L1	200759_x_at	9.692058	9.659289	9.484321	9.614203	10.09363	9.977367	7.863085	8.168073	7.869892	7.686889	8.589539	8.114498	9.046655
ARL6IP5	200760_s_at	7.830386	7.565418	8.545617	8.286949	7.775897	7.847379	8.396441	7.821817	8.508358	8.340457	8.011966	7.941296	10.29898
ARL6IP5	200761_s_at	10.0015	10.00038	10.00124	10.21966	10.07942	9.803514	10.11454	9.951581	9.978858	10.06217	9.775427	9.941339	10.56303
DPYSL2	200762_at	8.852835	8.847207	9.022214	9.040221	9.661943	9.493982	8.568296	8.422945	8.013072	8.089941	9.303328	8.702589	12.14787
RPLP1	200763_s_at	14.02727	13.995	13.82644	13.98077	14.06685	14.02972	14.52706	14.55968	14.4671	14.54943	14.51415	14.60335	14.71892
CTNNA1	200764_s_at	8.927161	8.674027	8.959561	8.665742	8.568233	7.912633	9.269156	8.490803	9.481315	9.313903	8.658549	8.616521	10.64976
CTNNA1	200765_x_at	9.035968	9.060136	8.94535	8.981607	8.904152	8.645583	10.15862	9.65192	10.19091	10.20292	9.862262	9.471976	11.08155
CTSD	200766_at	10.00832	10.00767	8.411985	8.721839	8.88303	9.05068	7.496231	7.897289	7.480905	7.675559	7.6044	7.959474	5.764989
FAM120A	200767_s_at	4.743404	4.688036	5.444177	4.81283	4.609883	4.364987	5.182432	4.731507	4.92821	4.666958	4.651349	4.083982	4.708511
MAT2A	200768_s_at	9.104778	9.1761	8.919812	8.921624	9.762489	9.803228	9.933359	10.07641	10.14523	10.01279	10.20934	10.32406	9.956213
MAT2A	200769_s_at	1.993681	1.949687	2.11851	1.980797	1.946755	1.981558	4.220707	1.944452	3.712213	4.266369	1.97948	1.983543	5.830304
LAMC1	200770_s_at	6.25687	6.504168	6.247619	6.307176	6.337089	6.350171	5.891031	6.116356	5.754508	5.927965	5.830909	6.142657	7.14001
LAMC1	200771_at	7.486217	7.478572	7.469732	7.348765	7.084993	6.561288	7.823374	7.462148	7.809854	7.905799	9.112127	7.448748	10.93719
PTMA	200772_x_at	10.53359	10.56188	9.963832	10.13527	10.82907	11.23617	12.08405	12.04316	12.12611	11.89956	12.40812	12.23331	12.07786
PTMA	200773_x_at	11.49802	11.49291	11.45469	11.27382	11.43524	11.43241	12.99331	12.52908	13.01798	12.97266	12.50527	12.48014	13.01879
FAM120A	200774_at	11.05497	11.00253	11.60436	11.33392	10.42939	10.31238	11.20703	10.35669	11.39992	11.23086	10.51099	10.47437	10.63786
HNRPK	200775_s_at	10.75703	10.98863	10.97955	10.59404	10.7081	10.48008	12.67842	11.57972	12.20792	12.33489	11.33674	11.59658	12.93625
BZW1	200776_s_at	8.974589	8.75772	9.617027	9.265257	8.558524	8.460448	9.26357	8.028733	9.551929	9.434302	7.83148	7.89727	10.5722
BZW1	200777_s_at	11.1247	11.07797	11.64942	11.39245	10.97256	10.22625	11.33806	10.51482	11.10663	11.27526	10.45424	10.40748	11.53206
SEPT2	200778_s_at	6.422405	6.121647	6.583225	6.050082	5.605429	5.595671	8.10582	7.206671	8.345656	8.082427	7.212131	6.88772	10.47673
ATF4	200779_at	9.946574	10.10111	10.09424	10.04224	10.34199	10.19799	10.67025	10.38799	10.41209	10.66313	10.38516	10.54042	10.3389
GNAS	200780_x_at	11.01416	10.83021	11.48928	11.48519	10.60017	10.19224	12.85598	12.08727	12.75864	12.7245	12.45897	12.39072	13.36988
RPS15A	200781_s_at	13.55678	13.52058	13.62781	13.79899	13.74676	13.72233	14.12995	14.18713	14.34301	14.22848	14.21836	14.27495	14.6225
ANXA5	200782_at	8.353258	8.226701	8.399078	8.126089	8.099098	7.3828	9.949641	8.225323	9.772503	9.563363	8.406345	8.317585	11.22484
STMN1	200783_s_at	5.045503	4.818972	4.007499	3.862642	4.219468	5.082636	8.242907	8.271603	8.286672	8.603853	8.15759	7.87486	9.387483
LRP1	200784_s_at	5.853961	6.066251	6.261493	6.26464	6.540254	6.713621	5.207533	5.425284	5.43007	5.215564	5.312339	4.859023	5.779653
LRP1	200785_s_at	7.7975	7.726458	7.970164	8.04165	8.400761	8.167432	6.32553	6.05037	6.190114	6.051002	6.451227	5.927414	6.834096
PSMB7	200786_at	10.48058	10.48742	10.92139	10.91734	11.07015	10.95597	11.02138	11.05953	11.06974	11.05547	11.03218	11.16841	11.02251
PEA15	200787_s_at	3.322505	3.163537	3.11139	2.585086	3.318908	3.161034	3.444637	3.208984	3.74954	3.206643	3.923775	3.245985	6.978995
PEA15	200788_s_at	8.696595	8.691679	8.555497	8.437618	8.653288	8.846602	8.332135	8.496605	8.231826	8.146196	9.254184	8.588551	10.47825
ECH1	200789_at	9.915635	9.982116	9.857028	9.809051	9.835449	9.25907	9.502957	9.214355	9.502243	9.537414	8.911588	8.808851	8.024192
ODC1	200790_at	8.477934	8.335354	9.009558	8.90648	8.434094	7.81785	12.79967	12.03725	12.2902	12.45193	12.27795	12.50837	10.9986
IQGAP1	200791_s_at	6.630753	6.42841	7.412997	7.198037	6.934842	6.571081	6.882272	6.388729	7.035409	6.704388	6.62771	6.409207	8.564848
XRCC6	200792_at	9.994672	10.0361	9.891669	10.02479	9.96244	9.799849	11.03769	11.0436	11.04275	11.11033	11.12065	11.24632	10.83075
ACO2	200793_s_at	8.796907	8.717114	9.195412	9.214029	8.631284	8.464518	9.495562	8.565143	9.426603	9.637193	8.539821	8.681936	9.283424

DAZAP2	200794_x_at	11.51063	11.50131	11.90188	11.65376	11.34373	10.71087	11.47062	10.88694	11.52145	11.48159	10.69675	10.62952	11.11119
SPARCL1	200795_at	5.764048	6.247379	7.72488	7.711162	7.773562	7.267664	3.362932	3.202579	2.486306	2.171704	7.597109	3.432767	10.99614
MCL1	200796_s_at	2.788494	2.741304	2.800079	3.279541	2.798922	2.755038	2.803327	2.778004	2.792854	2.755642	2.63358	2.774228	3.953856
MCL1	200797_s_at	11.58157	11.42867	11.74513	11.65678	12.39574	12.39446	10.82396	10.86418	10.5877	10.90157	11.30811	11.39541	10.36786
MCL1	200798_x_at	8.186192	7.975595	10.15346	9.474395	8.914106	8.710922	8.062798	6.95315	7.781061	8.146358	6.400338	7.155532	8.403296
HSPA1A	200799_at	11.60146	11.58168	11.73303	11.68667	11.92702	11.80605	11.12645	10.9823	11.01736	11.13997	10.91337	11.32705	11.00247
HSPA1A	200800_s_at	9.734062	9.768942	10.24981	9.97294	10.30478	9.669585	10.01205	8.98767	9.84667	10.03714	9.242339	9.336566	10.22766
ACTB	200801_x_at	13.67593	13.72035	13.11212	13.24392	13.32748	13.21123	14.32307	13.86997	14.24738	14.20252	14.0951	14.01994	14.66018
SARS	200802_at	8.935138	8.798354	9.256389	9.273772	8.796839	8.617377	9.205381	8.696054	9.60871	9.579302	8.771372	9.082846	8.863795
TEGT	200803_s_at	10.8618	10.78172	11.62056	11.54678	10.90389	10.87849	10.28285	10.1326	10.64369	10.47717	10.01287	10.19138	9.277765
TEGT	200804_at	11.74241	11.70788	12.51372	12.31382	11.43491	11.0836	10.69064	10.22944	10.66032	10.71229	10.33756	10.69661	9.729077
LMAN2	200805_at	9.168106	9.112886	9.497238	9.601646	9.531295	9.460198	8.856054	8.990416	8.719066	9.131842	8.845875	8.820179	7.45977
HSPD1	200806_s_at	9.092983	8.830111	10.32036	9.57011	8.432793	7.785118	11.20614	8.745527	11.29326	11.38042	9.164589	9.045305	11.81463
HSPD1	200807_s_at	13.25129	13.19316	13.18256	13.27677	13.07801	12.84447	13.66649	13.50036	13.62188	13.69416	13.43978	13.42422	13.38422
ZYX	200808_s_at	6.434235	6.510181	6.778029	6.908205	6.294981	6.511719	7.467352	7.028775	7.211018	7.119003	8.105165	7.976088	7.786475
RPL12	200809_x_at	12.34002	12.35854	12.18845	12.28399	12.67299	12.74658	13.11003	13.06525	13.13241	13.3391	13.09329	13.25348	13.73156
CIRBP	200810_s_at	11.14175	11.07593	10.29858	10.83045	10.99016	11.0396	10.67391	10.92501	10.28335	10.54644	11.54937	11.20764	12.07902
CIRBP	200811_at	7.856493	8.174564	7.714342	7.86441	7.574409	7.407382	8.179691	7.43827	7.865979	8.050328	7.80546	7.813134	9.927235
CCT7	200812_at	8.425483	8.369475	8.577758	8.583537	8.659889	8.499142	9.875355	9.689518	9.98254	10.09302	9.884983	9.940691	9.752027
PAFAH1B1	200813_s_at	5.207655	4.976646	5.446266	4.86432	4.989859	4.115431	5.868122	4.94332	5.916039	5.675801	5.348001	4.584824	8.773447
PSME1	200814_at	9.899438	9.887027	9.960309	9.982991	9.496457	9.596385	9.425617	9.462017	9.447382	9.570473	9.203945	9.534088	8.281024
PAFAH1B1	200815_s_at	6.763092	6.787734	6.713275	6.784684	6.975807	6.82923	7.597276	7.538579	7.672265	7.89027	7.67494	7.875826	7.003805
PAFAH1B1	200816_s_at	8.270205	8.143153	8.30811	8.402177	7.757462	7.264024	8.329669	7.967692	8.235977	8.113249	8.134401	8.15896	9.555412
RPS10	200817_x_at	13.12801	13.10871	12.96545	13.13442	13.22804	13.10267	13.78734	13.76513	13.63087	13.86951	13.90567	13.88283	14.03453
ATP5O	200818_at	11.46608	11.35198	11.82342	12.05577	11.67841	11.54606	11.73121	11.47648	11.88556	11.85279	11.82176	11.77077	12.04445
RPS15	200819_s_at	12.7702	12.7062	12.69444	12.82699	12.83328	12.87358	13.81614	13.70392	13.8463	13.86336	13.75776	13.85647	14.03218
PSMD8	200820_at	8.533382	8.35633	8.979144	8.927039	8.697384	8.30042	9.553482	8.938323	9.590366	9.657267	8.892803	9.218359	9.538415
LAMP2	200821_at	10.0034	9.916335	9.762249	9.513971	9.624321	9.045638	9.066539	8.711333	9.296777	9.250318	8.699854	8.689284	9.679863
TPI1	200822_x_at	9.76072	9.82263	9.697481	9.72916	9.647367	9.602554	10.15849	9.858223	9.859039	10.14118	10.20826	10.03829	10.09657
RPL29	200823_x_at	11.80284	11.83452	11.89584	11.90357	11.77126	11.68072	13.01951	12.70706	13.25901	13.23749	12.88083	12.89067	13.26499
GSTP1	200824_at	5.043796	5.289909	5.097786	4.581601	5.257653	5.306481	9.40196	9.383241	9.273198	9.444874	9.305371	9.382511	8.700476
HYOU1	200825_s_at	7.127936	6.922852	7.84663	7.620198	7.747002	7.585253	8.576147	8.246328	9.165257	9.017379	8.572384	8.489918	8.180184
SNRPD2	200826_at	10.58001	10.55456	10.721	10.83391	10.84658	10.62394	11.88037	11.91844	11.96323	12.16057	12.05994	12.01947	12.38621
PLOD1	200827_at	8.475014	8.623859	8.258073	8.382694	8.414532	8.480882	7.764164	7.831574	7.853539	7.742652	8.260046	8.057708	8.486891
ZNF207	200828_s_at	6.986831	6.938422	7.871167	7.458631	6.179161	5.945113	9.852404	7.992701	9.779766	9.847815	7.788536	8.306375	10.45723
ZNF207	200829_x_at	8.137067	8.103467	7.839964	7.944651	7.920666	8.097012	9.269858	9.294127	9.275825	9.27131	9.278115	9.357827	9.616923
PSMD2	200830_at	8.786755	8.713195	8.549899	8.758395	8.937602	9.134447	10.11754	10.32727	10.1677	10.30978	10.28653	10.314	9.447134
SCD	200831_s_at	11.24505	11.27006	9.33592	9.148368	8.335722	8.396877	11.73506	10.42446	11.52284	11.67709	10.10993	10.61229	7.29852

SCD	200832_s_at	13.94749	13.9632	12.49296	12.46521	11.96905	11.2291	13.83347	13.3026	13.69569	13.7648	13.09708	13.4026	10.70692
RAP1B	200833_s_at	8.984992	8.902131	9.592276	9.566327	9.019688	8.660974	10.44687	9.63663	10.30335	10.30896	9.655317	10.05849	10.44199
RPS21	200834_s_at	12.8876	12.86043	12.72662	12.90464	13.07203	13.18957	13.66569	13.83613	13.70456	13.72438	13.89566	13.97646	13.88861
MAP4	200835_s_at	2.870214	2.724209	2.82927	3.072309	2.907754	3.121292	2.84568	2.854685	2.83894	2.831655	3.245449	3.046142	2.780153
MAP4	200836_s_at	3.876124	3.64435	3.873748	3.878039	3.859214	3.876778	3.888853	3.889118	3.879752	3.858895	3.870962	3.891022	4.864376
BCAP31	200837_at	9.009169	8.997075	8.61273	8.824483	8.790242	8.927675	9.063774	9.084126	8.78234	8.9834	9.000293	9.137021	8.150767
CTSB	200838_at	12.02014	12.03606	11.98326	11.90316	11.88961	11.51194	11.79724	11.14466	11.85346	11.76349	10.50464	10.92378	9.963807
CTSB	200839_s_at	12.94781	12.99672	12.5612	12.68574	13.01851	12.88488	12.25035	12.54491	12.575	12.37248	11.8821	12.31238	10.53899
KARS	200840_at	11.80341	11.75361	11.21408	11.51751	11.88926	11.93184	12.19236	12.59005	12.38236	12.35108	12.71002	12.67383	11.98028
EPRS	200841_s_at	2.153349	2.013755	2.226713	2.137938	2.75605	2.883804	3.125592	3.327878	3.753462	3.736971	3.415904	4.005239	5.296039
EPRS	200842_s_at	8.337925	8.080549	8.033356	8.243919	8.421417	8.450228	9.632609	9.944273	9.8933	9.882832	10.06516	10.19686	9.216505
EPRS	200843_s_at	7.267585	7.301167	7.493027	7.512597	7.13347	6.741303	9.459759	8.99326	9.746589	9.93098	9.174315	9.292429	8.954391
PRDX6	200844_s_at	12.05898	12.1624	12.59546	12.68212	12.76033	12.63769	12.22653	12.21896	12.27864	12.19424	12.31529	12.28045	11.94934
PRDX6	200845_s_at	10.94342	10.97885	11.62037	11.8575	11.54876	11.58295	11.27628	10.80954	11.28741	11.26016	11.20852	11.22071	11.48232
PPP1CA	200846_s_at	9.214843	9.19673	9.01878	8.967847	8.929057	8.731108	10.37791	9.874924	10.32205	10.29003	10.0589	10.34221	9.620881
TMEM66	200847_s_at	12.12677	12.1593	12.52165	12.37028	12.27326	12.167	11.79893	11.59971	11.73041	11.75916	11.29711	11.3366	12.03244
AHCYL1	200848_at	8.983444	8.635067	9.54348	9.110085	8.860151	8.987642	8.743639	7.65419	8.667747	8.78395	8.145902	8.282195	8.608756
AHCYL1	200849_s_at	9.124515	9.092607	9.016783	9.078188	9.271803	9.188506	8.255166	8.482391	8.227315	8.319135	8.788977	8.723228	7.843695
AHCYL1	200850_s_at	9.217114	9.204177	9.477197	9.409682	9.009729	8.685614	8.952869	8.483601	8.852454	8.761895	8.583582	8.642335	8.998355
KIAA0174	200851_s_at	8.629763	8.63128	8.424587	8.343372	8.406104	8.469724	8.969026	8.791833	8.986587	8.950274	9.077988	8.815203	9.655443
GNB2	200852_x_at	8.284482	8.077819	8.230942	8.219707	8.104976	7.79807	9.550894	8.644378	9.397884	9.487182	8.835941	9.190715	8.714546
H2AFZ	200853_at	8.786814	8.696252	8.575908	8.488964	8.239024	7.57205	11.61305	10.72465	11.52612	11.48086	10.67464	10.87968	11.84616
NCOR1	200854_at	8.511093	8.42384	9.36988	9.314282	8.482142	8.259142	8.566401	7.86535	8.279216	8.252188	7.616091	7.86178	8.70128
C20orf191	200855_at	5.782532	5.888128	7.020842	6.669323	5.8049	5.77401	5.778297	5.784949	5.797234	5.859867	5.459173	5.808075	5.656874
C20orf191	200856_x_at	6.939693	6.90485	7.994527	7.949527	7.231439	6.407086	5.544422	6.184162	6.036978	6.930627	5.519416	5.672546	5.482622
NCOR1	200857_s_at	6.440552	6.208824	7.764908	7.111917	5.949317	6.027758	6.45647	5.417505	6.312942	6.302138	5.005852	5.309313	6.252577
RPS8	200858_s_at	12.87738	12.59977	12.95387	13.08541	12.8091	12.68655	13.69671	13.46929	13.67099	13.70636	13.52132	13.55399	14.68373
FLNA	200859_x_at	6.506108	6.48088	6.815102	6.420389	7.135981	7.525982	7.536573	6.931749	6.798978	7.167991	8.484992	7.628234	9.073469
CNOT1	200860_s_at	8.9406	8.814162	8.689772	8.795051	8.666575	8.763174	9.663797	9.567803	9.897688	9.875661	9.529697	9.703051	9.195125
CNOT1	200861_at	6.854801	6.919297	7.5418	7.449278	7.247015	7.213268	8.417087	7.538486	7.884193	7.940322	7.484478	7.69619	7.510698
DHCR24	200862_at	11.65175	11.64371	11.42406	11.24129	11.80464	11.43297	11.69815	11.51927	11.78966	11.53485	11.61177	11.88208	10.00133
RAB11A	200863_s_at	8.612118	8.606451	8.895535	8.761513	8.424447	8.224184	9.757122	9.124613	9.747667	9.762185	9.217456	9.365241	10.34353
RAB11A	200864_s_at	7.187654	6.893261	7.659766	7.385901	6.993212	6.273076	7.927889	7.497231	7.851753	8.072735	7.798332	7.734857	9.453028
PSAP	200866_s_at	9.577785	9.562882	10.38714	9.979	9.106612	8.223007	10.24665	8.486537	9.976974	9.901096	8.415045	8.640685	9.632557
ZNF313	200867_at	7.989504	7.790146	8.209362	8.175274	7.640751	7.200713	8.146809	7.577419	8.284221	8.140769	7.376576	7.497972	7.693259
ZNF313	200868_s_at	5.789334	5.757032	6.521305	6.685302	5.928758	5.958855	5.828003	5.619791	5.786989	5.730438	5.498883	5.626785	6.034446
RPL18A	200869_at	11.76274	11.82913	12.11371	11.87319	11.52052	10.99666	13.46593	12.65444	13.48397	13.5229	12.96507	13.03801	13.99044
STRAP	200870_at	9.784008	9.775853	10.32904	10.3242	9.412489	8.89511	11.16964	10.34761	11.30629	11.44183	10.38133	10.30735	11.7353

PSAP	200871_s_at	12.49401	12.53614	12.24149	12.45384	12.58382	12.61384	11.991	12.09129	11.62373	11.6517	12.04093	12.09563	11.21187
S100A10	200872_at	9.555135	9.426437	9.043211	9.022587	8.79817	8.390821	10.94585	10.47234	10.44886	10.65272	10.20401	10.48685	11.61833
CCT8	200873_s_at	11.16531	11.16915	10.80106	11.03025	11.04029	10.65748	12.28148	11.9554	12.10261	12.17995	11.90379	12.15433	12.11077
NOL5A	200874_s_at	4.6234	4.369465	4.489442	5.01196	4.187357	4.413017	5.181953	4.735506	4.95046	5.186433	4.694526	5.032856	4.977508
NOL5A	200875_s_at	8.356768	8.330849	7.806939	7.984891	8.536298	8.813856	9.940992	10.58735	10.21625	10.16428	10.82676	10.72101	9.244363
PSMB1	200876_s_at	10.86593	10.88308	10.94905	10.96634	10.85901	10.72074	11.17567	10.90974	11.21406	11.29398	10.89296	11.06354	10.67486
CCT4	200877_at	9.750312	9.567717	9.55236	9.631335	9.728723	9.441061	10.96642	10.77428	11.08898	11.28496	10.83919	10.93066	11.2102
EPAS1	200878_at	10.65344	10.59825	11.29533	11.20355	10.89589	10.69597	10.0384	9.374659	9.914383	9.659553	9.228152	9.684245	8.578176
EPAS1	200879_s_at	2.840651	2.787411	2.779605	2.89647	2.817634	2.773356	2.789327	2.788419	2.782928	2.78525	2.782167	2.797762	2.73381
DNAJA1	200880_at	9.56013	9.505755	10.12286	10.04294	9.248392	8.952928	9.79867	9.340829	9.878628	10.08421	9.434591	9.514093	10.72049
DNAJA1	200881_s_at	10.75902	10.67883	11.08545	11.00663	10.42011	10.05549	11.15624	10.25379	11.23878	11.30027	10.18559	10.49951	10.88918
PSMD4	200882_s_at	10.51765	10.55912	10.23932	10.53652	10.65857	10.75119	10.35462	11.00168	10.45272	10.58447	10.98952	11.00157	10.22893
UQCRC2	200883_at	8.31468	8.196344	9.010234	8.648319	7.543967	6.937816	9.038822	7.41821	9.254737	9.124317	7.278762	7.541731	9.591536
CKB	200884_at	5.042672	5.181783	4.811099	4.176894	5.863211	5.509452	7.514399	8.152985	7.602614	8.089548	9.146815	8.759048	8.699047
RHOC	200885_at	7.739738	7.66639	7.602317	7.386692	7.706129	6.74182	8.622723	7.681424	8.25541	8.655145	8.12049	7.837108	9.54604
PGAM1	200886_s_at	10.41332	10.34269	10.78193	10.63615	10.15768	10.17137	10.4952	10.39185	10.46355	10.5132	10.61566	10.511	11.18472
STAT1	200887_s_at	9.902202	9.863107	9.792833	9.658788	9.728669	9.834317	8.796604	8.70728	8.939323	8.702224	7.828942	8.382518	7.955903
RPL23	200888_s_at	11.97504	12.01787	12.07435	12.05038	12.03656	11.74973	12.73463	12.49387	12.68208	12.76705	12.60431	12.48821	13.61945
SSR1	200889_s_at	7.139797	6.940484	7.921209	7.772074	6.950196	6.841303	8.557882	7.710348	8.903653	8.757047	7.77027	7.947845	8.720676
SSR1	200890_s_at	6.039857	6.144161	7.13985	6.957666	5.932675	5.659651	7.788945	7.359638	7.774212	7.644233	7.465897	7.182286	8.549371
SSR1	200891_s_at	11.63855	11.54509	11.61124	11.78361	11.8626	11.81417	11.8477	12.25324	12.06716	11.94585	12.21089	12.30334	11.96507
SFRS10	200892_s_at	7.225107	7.047041	8.319197	7.473202	6.955886	6.474991	9.726277	8.261752	10.00906	9.896382	8.200596	8.538191	10.27662
SFRS10	200893_at	10.53377	10.65483	10.52126	10.44475	10.47021	10.49492	11.87622	11.95861	11.89803	11.86016	11.89998	11.85369	11.96576
FKBP4	200894_s_at	4.012592	4.387219	5.249117	4.740567	4.56047	3.920998	6.940023	5.29756	6.988596	7.155478	5.20134	5.627511	5.198335
FKBP4	200895_s_at	8.273318	8.108643	8.555335	8.858331	9.119249	9.383876	9.995998	10.32379	9.845509	10.3805	10.18936	10.37359	7.691685
HDGF	200896_x_at	10.1253	10.13379	10.25348	10.23231	10.21271	10.23131	11.39898	11.53437	11.33036	11.51981	11.1394	11.64797	9.02865
PALLD	200897_s_at	8.160813	7.986441	8.362676	8.199297	7.584222	7.56272	7.254991	6.879473	7.192325	7.415897	8.532178	6.513237	11.26949
MGEA5	200898_s_at	5.932707	5.620768	6.505817	6.388837	6.601248	6.281946	6.989075	6.218704	7.097795	7.11856	5.929818	6.200928	7.919564
MGEA5	200899_s_at	8.530871	8.658526	8.743049	8.809924	9.059625	8.959797	9.141478	8.778101	8.991253	9.381374	8.579015	8.806342	9.477746
M6PR	200900_s_at	5.734224	5.39417	6.710489	6.379673	5.699567	5.353035	7.560401	5.139284	6.934001	6.846653	5.708036	5.916286	6.869823
M6PR	200901_s_at	7.79558	8.079715	8.095043	7.974288	7.51693	7.326206	8.492209	7.935548	8.312848	8.450888	8.078785	8.1724	7.072394
SEP15	200902_at	10.53759	10.40316	11.14863	10.94248	10.23479	9.763386	11.2248	10.37588	11.34245	11.31224	10.15754	10.19181	11.63827
AHCY	200903_s_at	9.212196	9.181276	10.03738	9.7856	9.637716	9.044121	10.37086	9.833516	10.69553	10.2972	9.842681	10.00478	9.32247
HLA-E	200904_at	9.130601	9.119216	10.06843	10.05179	9.85939	9.736241	7.188522	7.334407	7.55623	7.465185	6.891681	7.40016	6.663493
HLA-E	200905_x_at	10.61767	10.51722	11.05216	10.99323	11.32132	11.35725	8.770903	8.856629	8.627416	8.649821	9.037261	9.239716	8.187666
PALLD	200906_s_at	4.366052	4.35506	4.652734	5.227861	5.487587	4.791203	3.724043	3.661262	3.322721	3.317533	4.885239	3.963082	7.233931
PALLD	200907_s_at	8.214055	7.909583	7.870467	8.361201	8.814333	8.883295	6.976028	7.583421	6.794655	6.83934	9.58123	7.625519	10.92352
RPLP2	200908_s_at	4.658897	4.630704	4.279344	4.612315	5.064499	5.359736	4.584709	5.517885	4.516749	4.567698	5.317714	4.792164	3.816791

RPLP2	200909_s_at	12.90123	12.7895	12.44361	12.61405	12.95311	13.04486	13.64335	13.61406	13.57605	13.53912	13.80781	13.65082	13.94695
CCT3	200910_at	9.85802	9.865818	9.824669	10.09193	9.739705	9.828463	11.25345	11.32521	11.43407	11.4895	11.54673	11.59692	10.91074
TACC1	200911_s_at	7.939312	7.918406	8.585935	8.554575	8.029328	7.987229	8.910351	8.565742	9.254062	9.29472	8.636163	8.29495	8.398034
EIF4A2	200912_s_at	12.18177	12.0939	12.28015	12.04527	11.59066	10.97941	12.54458	11.48609	12.50169	12.4459	11.58053	11.51704	13.21511
PPM1G	200913_at	6.051633	6.048389	5.809343	6.029902	5.210631	5.61005	7.825976	7.622744	7.82118	7.831101	7.563686	7.776388	6.920862
KTN1	200914_x_at	11.03258	10.78553	11.00444	11.15529	10.934	10.79735	9.8665	9.983182	9.641229	9.712996	10.08651	9.932282	9.524917
KTN1	200915_x_at	11.33204	11.43471	11.38513	11.41841	11.42945	11.107	10.60495	10.69187	10.72353	10.80569	10.60758	10.6756	10.60861
TAGLN2	200916_at	9.52717	9.545106	8.995012	8.812765	8.351448	8.338905	8.374623	8.284361	8.456883	8.404352	8.448524	8.710065	8.4453
SRPR	200917_s_at	3.926409	3.930885	4.50144	4.001265	4.222838	4.44544	3.920221	3.920667	3.912637	3.907781	3.897261	3.6616	4.124395
SRPR	200918_s_at	9.439657	9.27417	10.2567	10.05295	9.175967	8.789185	8.25528	7.45664	8.319791	8.330959	7.582234	7.699967	8.015148
PHC2	200919_at	8.642058	8.716605	8.493996	8.408319	8.379914	8.297769	8.267379	8.480057	8.234128	8.373892	8.667666	8.557769	8.342559
BTG1	200920_s_at	11.58503	11.58952	11.73688	11.74478	11.37786	11.17755	10.80761	10.26996	10.66409	10.63436	10.2519	10.36287	11.28152
BTG1	200921_s_at	11.41402	11.61413	11.77868	11.7511	10.97669	10.48687	10.97089	10.35433	11.01948	11.04213	9.989217	10.12304	11.00589
KDELR1	200922_at	5.254832	5.257455	6.302821	5.565988	4.809081	4.733497	6.808792	5.026901	6.737819	6.833881	5.381899	5.736754	8.102152
LGALS3BP	200923_at	8.484725	8.689224	8.238941	8.336153	7.281728	7.564779	7.100767	6.487074	6.828786	6.846836	6.996824	6.053539	6.949853
SLC3A2	200924_s_at	6.568446	6.417336	7.184817	7.234861	6.85698	6.398841	7.691177	7.119453	7.583965	7.581121	6.858032	7.436811	6.95179
COX6A1	200925_at	12.64041	12.62878	12.68683	12.66474	12.04863	11.807	12.65453	12.31799	12.55175	12.51871	12.06989	12.34588	11.93737
RPS23	200926_at	13.84805	13.71547	13.91804	14.04017	13.93956	13.89762	14.40506	14.39152	14.4758	14.41482	14.50294	14.4611	14.80639
RAB14	200927_s_at	7.817255	7.593542	8.870149	8.393221	6.865931	5.396531	8.200876	5.863142	8.426874	8.199912	5.983593	5.987187	9.219654
RAB14	200928_s_at	5.584219	5.311316	5.223586	5.585477	5.434924	6.098285	4.022719	4.781613	4.01285	4.04715	4.688296	4.960417	4.539401
TMED10	200929_at	8.083825	8.015633	9.042964	8.922858	7.980358	7.218287	8.641969	7.616643	8.796127	8.627037	7.870132	8.000942	8.62781
VCL	200930_s_at	2.720233	2.700575	2.648381	2.66323	2.691136	2.650616	2.693545	2.701474	2.687468	2.825517	2.68734	2.724268	2.615336
VCL	200931_s_at	6.553874	6.541218	7.8167	7.745414	6.658701	6.760156	8.244486	7.119743	7.89186	7.980369	8.107579	7.851777	9.80135
DCTN2	200932_s_at	7.73098	7.623374	7.330356	7.542834	7.864202	8.153012	8.65474	8.820613	8.546611	8.662559	8.891516	8.908743	9.263576
RPS4X	200933_x_at	12.83506	12.85368	12.63444	12.79856	13.43853	13.43314	13.70764	13.58392	13.72078	13.72226	14.03465	13.63424	13.97757
DEK	200934_at	9.10645	8.823796	9.041187	9.008772	8.871469	8.473444	10.98714	10.54838	11.03034	11.03345	10.21032	10.50414	10.60418
CALR	200935_at	7.398398	7.116638	7.99808	7.298671	5.689326	4.996267	7.748497	6.272317	7.810135	7.528809	7.354063	6.848929	7.297279
RPL8	200936_at	11.8068	11.79268	11.91827	11.91388	12.23363	12.16558	13.52222	12.91303	13.49318	13.41389	13.20118	12.95844	14.01682
RPL5	200937_s_at	11.4723	11.48916	12.00185	11.85043	11.59764	11.20637	13.09859	12.63022	13.33736	13.28677	12.7246	12.5191	13.77875
RERE	200938_s_at	2.598359	2.566948	2.551615	2.558064	2.584027	2.562758	2.568374	2.582441	2.565496	2.545061	2.571723	2.580764	2.47811
RERE	200939_s_at	2.57146	2.561337	2.474401	2.50639	2.549853	2.546854	2.55596	2.548185	2.544221	2.538383	2.537166	2.523026	2.708246
RERE	200940_s_at	6.920599	6.781789	7.087854	6.851675	7.24057	6.766068	7.020447	6.390757	7.206353	6.876217	7.167651	6.718079	8.026719
HSBP1	200941_at	8.458246	8.599898	8.05405	8.427784	8.420799	8.756805	7.996076	8.604689	8.189977	8.269663	9.153779	9.026416	9.915195
HSBP1	200942_s_at	8.511645	8.672295	8.77284	8.743385	8.582602	8.210096	9.693834	9.188104	9.672982	9.62496	9.248165	9.3778	10.52045
HMGN1	200943_at	9.931423	9.784047	9.598272	9.484227	9.430719	8.894535	11.7229	10.53397	11.68469	11.79075	10.99855	10.76158	12.27309
HMGN1	200944_s_at	10.25418	10.19147	9.341617	9.369726	9.831766	9.701062	11.71942	11.11824	11.42412	11.50461	11.6086	11.4009	12.10476
SEC31A	200945_s_at	11.15285	11.16344	10.86336	10.82224	10.14344	10.01119	11.28065	10.92088	11.20781	11.21362	11.10465	11.03641	12.12585
GLUD1	200946_x_at	9.91006	9.73133	10.72269	10.33059	8.529687	8.386077	9.671889	8.696779	10.59478	10.44071	9.349627	8.119749	7.768603

GLUD1	200947_s_at	12.1606	12.27008	12.47165	12.03848	10.92863	10.31786	11.44921	11.18651	12.36261	12.38626	11.63761	10.6964	8.720379
MLF2	200948_at	8.266243	8.254917	8.462078	8.349993	7.977407	7.311581	7.625765	6.502427	7.502919	7.723603	7.100529	6.922587	7.9125
RPS20	200949_x_at	12.49536	12.44391	12.71273	12.87523	12.91162	12.82377	13.25326	13.30081	13.25372	13.20739	13.23951	13.19149	13.98189
ARPC1A	200950_at	8.010836	8.087612	8.975375	8.974969	8.096898	7.460768	9.626828	8.120184	9.700499	9.537214	8.136599	8.638873	9.518583
CCND2	200951_s_at	2.31023	2.304992	2.287547	2.24646	2.294099	2.336984	2.388743	2.303417	2.613606	2.967309	4.912054	3.033628	6.489331
CCND2	200952_s_at	1.884047	1.875077	1.855607	1.858659	1.871794	1.857081	1.873909	1.868105	1.867243	1.854381	1.853996	1.863682	7.501115
CCND2	200953_s_at	4.546819	4.493163	4.413565	3.417055	4.818476	4.815198	7.494521	6.157052	7.677122	7.644571	8.250285	6.404836	11.05146
ATP6V0C	200954_at	7.12869	7.033166	7.070368	7.038307	6.701275	6.543222	7.934639	7.494897	7.730654	8.08313	7.305319	7.795494	7.222931
IMMT	200955_at	8.520999	8.633844	8.535705	8.675174	8.809065	8.468937	9.565698	9.489497	9.762964	9.658162	9.612785	9.587282	9.019725
SSRP1	200956_s_at	7.021633	6.703843	7.053047	7.16121	7.719656	7.623461	9.085239	8.876985	8.869856	9.006525	9.325489	9.336058	8.502808
SSRP1	200957_s_at	7.498957	7.24718	7.197632	7.290572	7.543104	7.496671	9.333723	9.251582	9.149812	9.448894	9.144095	9.461836	8.174888
SDCBP	200958_s_at	10.60739	10.3727	10.88076	10.72838	10.31266	10.2293	11.1164	10.28371	11.12614	10.79641	10.13826	10.29794	11.5528
FUS	200959_at	9.188132	9.285683	8.545917	8.219762	9.338588	9.229229	10.34429	10.34638	9.944324	10.43336	11.00208	10.50806	10.66367
CLTA	200960_x_at	10.5378	10.56444	10.41274	10.58715	10.38933	10.38268	11.16947	10.89	10.99262	10.88998	11.06551	11.10492	11.24952
SEPHS2	200961_at	12.92697	13.02711	12.5766	12.68999	12.73169	12.67163	11.07986	11.14836	11.11164	11.09799	10.68898	11.20459	9.033789
RPL31	200962_at	5.639827	4.972449	4.114013	4.398877	3.863551	3.251616	7.14744	5.465582	6.469835	7.338193	5.50522	3.927862	8.647222
RPL31	200963_x_at	13.22756	13.26668	13.25028	13.35268	13.25636	13.14171	14.09386	13.69416	14.09566	14.01967	13.8773	13.81689	14.41775
UBE1	200964_at	9.581169	9.465209	9.83872	9.794368	9.69771	9.43036	10.28711	9.729325	10.17054	10.22123	10.23498	9.737227	10.49319
ABLIM1	200965_s_at	8.639798	8.648745	8.980768	8.992338	8.749452	8.329165	8.404609	7.951479	8.286204	8.239822	8.641193	8.082511	9.774373
ALDOA	200966_x_at	9.171363	9.076561	9.037314	9.135594	9.239918	9.328019	11.11834	10.53898	10.99753	11.0235	11.06147	10.84556	12.12886
PPIB	200967_at	12.09056	12.28449	12.22008	12.09573	12.1512	12.17508	13.17661	13.00178	12.92931	12.9323	13.06616	13.12074	13.04479
PPIB	200968_s_at	10.21554	10.31312	10.68019	10.44932	10.05496	9.806651	11.73954	11.08938	11.4417	11.48322	10.98699	11.04264	12.0317
SERP1	200969_at	10.45461	10.45292	10.90052	10.985	10.79756	10.84397	10.31526	10.50044	10.32643	10.19277	10.58712	10.44609	10.05544
SERP1	200970_s_at	8.824622	8.847473	10.17136	9.6773	8.931304	8.189767	9.869594	8.97318	10.07828	10.02509	8.396561	8.812222	10.03909
SERP1	200971_s_at	10.00035	9.97455	10.35336	10.41208	10.52299	10.44237	10.42027	10.72265	10.60686	10.53191	10.42282	10.63098	9.998833
TSPAN3	200972_at	8.286075	8.502484	8.536546	8.393452	8.069591	7.831968	9.24565	9.204304	9.492529	9.696322	9.63291	9.185779	11.2341
TSPAN3	200973_s_at	5.879875	5.797423	7.07044	6.27054	5.637404	4.546418	8.177702	6.516065	8.455146	8.250529	7.289541	6.603979	10.73295
ACTA2	200974_at	8.55766	8.843576	9.300521	9.313709	8.953039	8.948787	7.024585	5.595179	6.52463	5.922403	8.842377	6.802266	11.59046
PPT1	200975_at	9.169944	9.265501	9.644576	9.602619	9.602899	9.579116	9.845195	9.679127	9.843948	9.684986	9.728212	9.824754	10.43568
TAX1BP1	200976_s_at	9.158499	9.266002	8.958527	9.178146	8.828912	8.600274	9.354119	9.488818	9.880928	9.950157	8.752028	9.016191	9.142946
TAX1BP1	200977_s_at	10.3589	10.25377	10.21008	10.39497	10.31664	10.19894	10.50625	10.48788	10.72263	10.77708	10.15638	10.42341	10.27525
MDH1	200978_at	9.90814	9.680473	10.44029	10.347	9.877512	9.350864	10.19217	9.439319	10.28279	10.32329	9.619711	9.4503	11.331
MAP3K15	200979_at	7.524979	7.500348	7.751955	7.976128	6.88317	6.47912	6.811213	6.79848	6.710745	7.15695	6.485637	6.51235	6.614922
PDHA1	200980_s_at	8.603953	8.613506	8.291407	8.481336	8.184801	7.814948	9.49028	9.134638	9.476587	9.481427	8.935471	9.046803	8.474236
GNAS	200981_x_at	11.00151	10.87496	11.4929	11.55855	10.69762	10.18988	12.97226	12.11087	12.80326	12.79689	12.51708	12.39207	13.35179
ANXA6	200982_s_at	9.058734	9.055823	9.041322	8.794393	7.349225	7.401797	9.563552	8.888418	9.069467	9.212993	9.675044	9.262661	9.617781
CD59	200983_x_at	8.427905	8.443643	8.936727	8.978816	8.546983	8.272326	8.498851	7.67908	8.439437	8.751607	7.349619	8.004521	8.96945
CD59	200984_s_at	8.519911	8.508586	8.675095	8.470566	8.617501	8.459485	8.042359	7.522081	7.837239	8.136967	7.434455	7.710365	9.022387

CD59	200985_s_at	9.320773	9.276785	9.324367	9.237167	9.202212	9.01557	9.043732	8.221502	8.585798	8.871428	8.094799	8.229966	9.477231
SERPING1	200986_at	13.54486	13.53527	13.39105	13.57844	13.47269	13.34023	10.86809	10.62476	10.72094	10.84833	10.3162	10.56298	8.198049
PSME3	200987_x_at	4.265753	4.115183	4.730064	4.659498	4.697022	4.65193	4.667874	5.643131	5.750017	5.520698	5.698905	6.051485	4.926493
PSME3	200988_s_at	3.342682	3.248834	3.298601	3.396153	3.318404	3.198509	3.524905	4.534208	4.906179	5.233029	4.267973	4.181604	3.380344
HIF1A	200989_at	10.76768	10.66958	10.85907	10.8941	10.92311	11.31614	11.05221	10.91138	10.89433	10.85883	11.24087	11.11465	11.81072
TRIM28	200990_at	8.66502	8.416994	8.217164	8.311146	8.152937	8.22357	10.33937	9.712994	10.09606	9.99373	9.913279	9.945459	9.891879
SNX17	200991_s_at	7.746145	7.586616	7.863072	7.659785	7.523611	7.00181	8.536836	7.836755	8.59307	8.667574	7.992094	8.193422	8.804642
IPO7	200992_at	8.644267	8.611985	9.15948	8.747019	8.171228	8.236884	9.650807	8.734462	9.907497	9.831193	8.995653	8.91055	10.57358
IPO7	200993_at	8.161154	8.06034	9.215886	8.827694	7.515038	6.388444	9.592421	7.736687	9.555562	9.47357	8.071392	8.135655	10.41842
IPO7	200994_at	9.365465	9.350506	9.669026	9.670024	9.187858	9.067764	9.919102	9.658296	9.914563	10.15655	9.818836	9.766276	10.56307
IPO7	200995_at	8.005814	7.822573	7.909476	8.099181	8.010269	8.137564	7.950023	8.336541	8.009845	8.109507	8.522025	8.562352	7.268705
ACTR3	200996_at	8.837688	8.791374	9.41257	8.897856	9.021294	8.934652	10.95472	9.558124	10.6218	10.89698	9.489897	9.886169	11.10076
RBM4	200997_at	8.483207	8.495362	8.294318	8.198459	8.227641	8.058876	9.237487	9.251149	9.654888	9.801228	9.783486	9.614926	10.66325
CKAP4	200998_s_at	6.695666	6.736908	7.52344	7.483516	7.406758	7.100742	8.769308	8.631491	8.550899	8.452173	9.299081	8.860636	10.0063
CKAP4	200999_s_at	9.281645	9.223912	8.552518	8.829523	9.273267	9.300246	9.781036	10.34281	9.709446	9.741703	10.93621	10.58695	11.07736
AARS	201000_at	8.585492	8.583188	8.691477	8.540555	8.560983	8.445832	9.374553	8.988455	9.119481	9.587299	8.940944	9.355674	8.821607
TMEM189-U	201001_s_at	7.183363	6.960974	7.568349	7.509266	7.01943	7.428933	9.249235	8.65481	9.120123	9.234415	8.926164	8.85623	8.879247
TMEM189-U	201002_s_at	9.214156	9.320048	9.286903	9.330977	9.348618	9.115482	10.50669	10.55581	10.53565	10.72621	10.53793	10.48858	9.773309
TMEM189	201003_x_at	7.009996	6.517788	7.535239	7.3543	6.582886	6.180124	6.669468	6.706193	6.549614	6.102794	6.778437	6.218958	6.315807
SSR4	201004_at	10.66346	10.59905	11.04622	10.80778	11.18952	11.08887	11.37997	11.29237	11.45779	11.66321	11.38569	11.48583	11.19731
CD9	201005_at	7.281484	7.2453	8.678948	8.372303	8.303743	7.865707	7.578757	6.637355	7.079357	7.272557	6.077222	6.409624	9.176029
PRDX2	201006_at	3.991601	4.059073	3.879184	4.013279	4.069069	3.985852	4.07273	5.391562	5.849318	4.926459	5.035949	4.141059	4.041395
HADHB	201007_at	11.32758	11.28948	11.12186	11.15109	11.42986	11.16028	10.38456	10.0078	10.35256	10.25985	9.799115	9.611806	9.80817
TXNIP	201008_s_at	8.47474	8.384114	9.432834	8.892706	8.076951	8.536315	7.47887	6.449185	7.403292	7.332896	7.391343	6.927236	10.59991
TXNIP	201009_s_at	10.29304	10.26946	10.50522	10.2164	9.402388	8.716794	8.902711	7.787041	8.746142	9.007336	8.507476	7.722607	10.49165
TXNIP	201010_s_at	11.21021	11.13386	11.16251	10.98822	10.0443	9.533932	9.792864	8.76142	9.819478	9.965634	9.340387	8.828621	10.91432
RPN1	201011_at	9.835541	9.842735	9.460107	9.679617	9.755913	9.604471	9.675933	9.506017	9.424008	9.5466	9.573197	9.677223	9.216726
ANXA1	201012_at	5.793828	5.377669	7.796051	6.997668	6.163871	6.013757	5.184655	3.999335	5.762642	5.779073	5.311981	4.224533	10.44643
PAICS	201013_s_at	9.814009	9.708459	10.11381	9.809363	9.182881	8.7624	10.59522	10.08619	10.77142	10.78129	9.827905	9.964414	9.574745
PAICS	201014_s_at	8.183267	7.802339	8.338843	8.139419	7.523302	7.089552	9.632293	9.153926	9.817423	9.783789	8.796744	9.101489	8.698207
JUP	201015_s_at	7.309478	7.5503	7.314732	7.450626	7.556615	7.473192	6.928569	6.429295	6.900206	6.816621	6.792947	6.696831	6.92635
EIF1AX	201016_at	8.269347	8.005141	8.52047	8.426055	8.601506	8.008668	8.729855	8.60098	8.804341	8.822855	8.840785	8.577184	8.514408
EIF1AX	201017_at	7.1438	6.85228	8.109366	7.81495	7.795827	7.158372	8.30217	6.715609	8.272378	8.128109	7.374837	6.223776	8.668532
EIF1AX	201018_at	7.445019	7.409772	8.169926	7.921634	8.077966	7.417181	8.319258	7.419349	8.388417	8.370451	7.714226	7.104302	8.824271
EIF1AP1	201019_s_at	10.63229	10.57848	10.77374	10.81572	11.2623	10.97994	10.84747	10.78313	10.75161	10.815	11.2873	10.92421	10.59728
YWHAH	201020_at	5.256947	5.269207	5.776971	4.900557	3.887616	3.489817	9.158879	7.005527	8.781988	9.200904	6.707782	7.137977	8.552339
DSTN	201021_s_at	8.823596	8.801307	10.15303	9.589182	9.217706	8.901665	9.681391	8.455087	9.837533	9.621864	8.6356	8.505113	12.2664
DSTN	201022_s_at	10.77022	10.82988	11.13572	11.301	11.35582	11.21473	10.24249	9.924449	10.14906	10.08393	10.50702	10.0151	12.14172

TAF7	201023_at	8.82428	8.904298	8.978242	8.971975	8.649495	8.157487	8.984917	8.741903	9.275364	9.460492	8.870352	8.899059	9.926191
EIF5B	201024_x_at	10.02251	9.989448	9.627143	9.830808	10.66611	10.76063	10.06748	11.1502	10.18714	10.17938	11.14022	11.11337	9.433262
EIF5B	201025_at	6.777316	6.643629	6.386766	6.530369	6.649919	6.769922	7.424861	7.897505	7.22888	7.332716	7.485476	7.661029	7.072782
EIF5B	201026_at	2.33504	2.591911	2.639455	2.643984	3.077173	3.113776	2.829365	3.200739	2.927907	2.725416	2.661214	2.885538	2.017276
EIF5B	201027_s_at	7.357913	7.200122	7.364402	7.746102	7.052703	6.820917	8.412624	7.860586	8.754791	8.680328	7.992845	7.886402	8.265721
CD99	201028_s_at	9.035755	9.137914	9.966195	9.613796	8.353396	7.430336	10.09564	8.865602	9.75598	10.05666	8.302313	9.308759	10.82641
CD99	201029_s_at	11.3512	11.32627	11.53915	11.61506	11.11029	10.97495	11.59241	11.41272	11.37948	11.58664	11.17505	11.78982	12.31323
LDHB	201030_x_at	7.628109	7.662355	7.67576	7.783444	8.603159	8.44272	11.81599	11.24139	11.75163	11.64979	11.73186	11.52984	12.57177
HNRPH1	201031_s_at	12.68448	12.80816	12.32428	12.3314	12.54713	12.26276	13.32104	13.28461	12.77917	12.75752	13.07968	12.95896	13.42212
BLCAP	201032_at	9.974768	9.947483	9.780263	10.00053	10.23518	10.27868	7.753997	8.378826	7.763657	7.812189	10.01116	8.547	10.04802
RPLP0	201033_x_at	13.15771	13.15198	13.09002	13.10869	12.94512	12.84243	14.09297	13.89988	13.98221	14.02549	13.96985	14.01428	14.10052
ADD3	201034_at	9.644815	9.473669	9.331163	9.352639	8.553851	8.415767	11.19313	9.894875	11.32675	10.93883	10.34682	10.30852	12.5619
HADH	201035_s_at	8.09991	8.041698	9.205841	8.843287	7.889382	7.979378	7.74796	7.219495	7.80732	8.126831	6.736477	7.644269	6.837679
HADH	201036_s_at	11.44371	11.38778	11.68728	11.68317	11.02434	10.92449	10.25108	10.26955	10.58536	10.23365	10.13012	10.31211	9.22631
PFKP	201037_at	4.570659	4.678777	4.0245	4.13557	5.141759	4.678722	4.627233	4.760821	4.733707	4.81405	4.683624	4.980107	8.045617
ANP32A	201038_s_at	7.29875	7.193379	6.739513	6.888967	7.260479	7.030795	8.355742	8.4861	8.178492	8.391663	7.971557	8.392421	7.902296
RAD23A	201039_s_at	7.281659	6.964265	7.590828	7.644447	7.956688	7.654038	9.418682	9.644049	9.201562	9.35365	9.528621	9.859694	6.789215
GNAI2	201040_at	6.393322	6.781562	6.657529	6.997531	6.487262	6.904642	6.197869	6.113789	6.304974	6.469374	6.556882	6.59192	6.369409
DUSP1	201041_s_at	12.37471	12.33904	11.00422	11.02753	12.24902	11.72372	7.997137	6.755431	8.1722	8.123326	6.868238	7.189594	8.518224
TGM2	201042_at	6.618825	6.490072	7.668539	7.506823	6.688997	6.610927	7.59995	7.649187	7.897322	7.91917	6.86467	7.369816	7.23508
ANP32A	201043_s_at	5.272298	5.05742	5.349319	4.327258	5.075199	4.551334	5.798292	5.143914	6.243641	6.094759	5.30737	5.091101	7.223137
DUSP1	201044_x_at	4.988643	4.516031	4.95462	4.412708	4.058527	4.481841	4.424918	4.379497	4.521273	4.351039	4.019978	4.484141	4.704041
RAB6A	201045_s_at	2.303346	2.285333	2.266541	2.26972	2.287811	2.258833	2.282675	2.280218	2.264692	2.270194	2.26614	2.278155	2.276199
RAD23A	201046_s_at	9.208126	9.183137	8.654084	9.062842	9.305809	9.381217	10.68913	11.49748	10.7584	10.88017	10.81104	11.3559	7.647722
RAB6A	201047_x_at	8.436267	8.313934	9.261273	8.825436	8.430795	8.065255	8.900941	8.395356	9.028442	8.877722	8.085999	8.431712	10.24428
RAB6A	201048_x_at	2.341349	2.323953	2.307156	2.289693	2.323854	2.246933	2.315161	2.323978	2.321392	2.306258	2.305819	2.318948	4.964239
RPS18	201049_s_at	13.26335	13.21173	13.13961	13.16969	13.10383	12.97943	13.95583	13.62172	14.00533	14.05746	13.86131	13.79643	14.49267
PLD3	201050_at	7.8659	7.727712	8.139958	8.175062	7.600615	7.190618	8.756581	7.658051	8.702541	8.618353	8.262731	8.049487	9.313589
ANP32A	201051_at	7.60838	7.67555	8.708922	8.232543	7.888571	7.213536	10.05455	8.789628	9.975628	9.789216	8.814434	9.144432	10.55222
PSMF1	201052_s_at	7.948028	7.9423	8.393098	7.956499	7.425902	6.703295	9.085593	8.185925	9.179025	9.320369	7.950377	8.262303	7.384025
PSMF1	201053_s_at	10.08218	9.901361	9.62203	9.896355	9.920761	9.854835	9.798838	10.38504	9.739253	10.20838	9.935867	10.20007	8.753735
HNRNPA0	201054_at	9.979961	9.877582	10.08179	9.997397	9.921532	9.167373	11.31937	10.92594	11.3609	11.252	11.23441	10.91463	12.9344
HNRNPA0	201055_s_at	5.57952	5.915062	5.699704	5.945269	5.819644	5.944648	6.836496	6.972161	6.65781	6.914546	5.711075	6.842051	7.47673
GOLGB1	201056_at	7.109638	7.130496	6.766542	7.089425	6.849778	6.908619	6.200046	6.624988	6.146097	6.445186	6.496336	6.633285	6.66074
GOLGB1	201057_s_at	7.930861	8.084295	8.135101	8.347009	8.187674	8.298267	7.780558	7.833532	7.947329	7.744486	8.117103	8.0023	7.925685
MYL9	201058_s_at	5.583957	5.828808	6.845375	6.435121	6.928513	7.355503	5.574005	4.121598	5.488206	5.439247	7.876702	6.194281	9.721721
CTTN	201059_at	9.539608	9.444621	9.186273	9.315095	9.683962	9.712155	9.093204	9.562401	9.076016	9.083217	9.824005	9.795737	8.818697
STOM	201060_x_at	9.653299	9.468543	10.05625	9.752114	9.464242	9.312114	10.48117	9.462784	10.41875	10.39464	8.287529	9.378482	8.398102

STOM	201061_s_at	12.04745	12.02884	11.881	12.03162	12.28981	12.25406	11.34739	11.49815	11.51283	11.22853	10.86799	10.81705	9.360851
STOM	201062_at	3.007499	2.946264	2.906333	2.929126	2.964199	2.873964	2.93695	2.942507	2.934563	2.912255	2.934329	2.943765	2.661717
RCN1	201063_at	9.517535	9.631825	9.234004	9.49107	9.316187	9.60548	7.508087	7.720377	7.794251	8.191639	9.373643	8.500958	9.820503
PABPC4	201064_s_at	9.144037	9.202103	9.42778	9.342892	8.558576	7.942798	10.27368	9.24953	10.03957	10.29165	9.438876	9.539263	9.859507
GTF2I	201065_s_at	10.53651	10.4006	10.60943	10.56183	10.47628	10.38611	11.34447	10.89943	11.09946	11.29036	11.21464	11.28832	12.207
CYC1	201066_at	8.631193	8.634807	9.153135	9.173363	8.714144	8.24752	8.75055	7.556205	8.879887	8.72073	7.776611	7.917198	8.496719
PSMC2	201068_s_at	10.18041	10.00114	10.63039	10.34318	9.844644	9.390183	11.31842	10.36256	11.28883	11.32081	10.19926	10.26038	11.05221
MMP2	201069_at	5.674979	6.295287	6.213262	5.63273	6.106046	7.269981	5.850093	5.304435	5.033153	5.398033	8.675878	5.768903	11.73329
SF3B1	201070_x_at	7.382629	7.058453	7.276216	7.443951	7.767411	7.673162	7.482706	7.336234	7.172012	6.958817	7.818339	7.723282	8.48126
SF3B1	201071_x_at	10.21377	10.30342	10.68612	10.39961	9.537277	8.71779	11.5555	10.00502	11.4647	11.33541	10.01525	10.07543	12.12452
SMARCC1	201072_s_at	4.776217	4.332082	4.610694	4.896463	5.270038	5.771178	4.806554	6.178474	4.670688	4.554441	7.010467	6.61918	4.911031
SMARCC1	201073_s_at	2.936836	3.033182	3.036493	2.879222	3.066361	3.021989	3.719787	3.392506	3.6277	4.220952	3.360928	3.74489	5.94878
SMARCC1	201074_at	8.059032	7.96577	7.517359	7.54362	7.359187	7.215245	8.873159	8.874399	9.019225	9.051941	8.997618	8.811229	9.240534
SMARCC1	201075_s_at	5.161182	4.588626	5.505028	4.820639	4.101345	3.605569	7.461032	5.290404	7.554728	7.573006	5.633971	5.85956	8.362851
NHP2L1	201076_at	7.197904	7.109955	7.003997	7.064482	7.349525	7.542299	7.494348	7.871197	7.617221	7.672727	8.254024	8.247081	7.939023
NHP2L1	201077_s_at	9.355937	9.198312	9.352576	9.153887	8.713905	8.359321	10.19764	9.440162	10.23356	10.25959	9.706933	9.549796	10.7841
TM9SF2	201078_at	9.683367	9.733738	10.62081	10.21042	9.091652	8.224558	10.63359	9.120918	10.64272	10.50291	8.877028	9.432287	10.42562
SYNGR2	201079_at	8.033402	7.821326	7.504658	7.776289	7.86514	7.54466	8.357598	7.762103	8.05752	7.998827	7.823709	8.175098	6.279895
PIP4K2B	201080_at	7.140023	7.456248	7.176701	6.90677	7.410089	7.21131	7.430205	7.59584	7.774277	7.234158	8.077172	7.511563	9.059712
PIP4K2B	201081_s_at	3.328243	3.079648	3.159144	3.091202	3.192668	3.92536	3.123246	3.130065	3.418463	3.220558	3.202018	3.124993	6.561503
DCTN1	201082_s_at	6.395355	6.41125	6.753569	6.57396	5.960586	5.699384	6.984023	6.139328	6.618684	6.434638	6.760124	6.36969	8.173243
BCLAF1	201083_s_at	7.552867	7.307243	7.649796	7.688188	7.686706	7.84361	7.972387	7.696477	7.64724	7.611065	8.108067	8.066554	8.579833
BCLAF1	201084_s_at	10.18493	10.33758	10.30151	10.25924	9.801087	9.309664	11.0678	10.4476	11.03155	11.10351	10.54124	10.52889	11.94254
SON	201085_s_at	7.949146	7.468129	8.105035	8.285986	8.481009	8.430241	8.603688	8.648017	8.099909	8.121634	9.153994	9.201441	7.657181
SON	201086_x_at	10.01706	9.990846	10.32279	10.27674	9.786027	9.284895	10.94325	10.1474	11.01496	10.83264	10.19851	10.19132	11.00815
PXN	201087_at	5.62861	5.927344	5.383273	5.427974	6.088929	5.88564	3.841241	4.516462	4.32058	4.377911	5.331028	4.682702	5.887263
KPNA2	201088_at	9.691656	9.522843	8.626638	8.613832	8.602565	8.78369	12.4643	12.53685	12.40415	12.57884	12.45547	12.49931	12.16416
ATP6V1B2	201089_at	7.271017	7.146989	7.754651	7.528372	7.025802	5.976534	7.911169	6.78657	8.107919	8.268633	6.798668	7.161274	8.365947
TUBA1B	201090_x_at	10.07569	10.0819	9.626769	9.400555	9.929598	9.755892	12.80585	12.42729	12.6263	12.76056	12.57103	12.51896	13.22482
CBX3	201091_s_at	9.978652	10.18473	9.043389	9.326874	9.338437	9.728434	11.17648	11.18625	11.32076	11.32725	10.85977	11.39775	11.25856
RBBP7	201092_at	8.900316	8.909899	8.819033	8.692595	8.680039	8.546774	10.47891	10.19135	10.54828	10.56871	10.00619	10.05581	10.72264
SDHA	201093_x_at	9.688704	9.714783	9.960791	9.978478	9.555612	9.23932	8.882203	8.677734	8.764056	8.864342	8.778857	8.864268	7.741711
RPS29	201094_at	13.87851	13.85078	13.7619	13.88186	13.91415	13.97234	14.02921	14.20253	14.13117	14.05949	14.18267	14.25005	14.18693
DAP	201095_at	6.919551	6.981275	7.619874	7.665121	7.680677	6.969862	8.461778	7.861089	8.640941	8.772497	7.867614	7.974392	7.61364
ARF4	201096_s_at	7.867821	8.01464	8.67879	8.136103	7.578444	7.264771	9.912287	8.730127	9.760755	9.797144	8.511978	8.699661	10.9376
ARF4	201097_s_at	11.02114	10.87891	11.24827	11.04542	10.23095	9.909557	12.18477	11.11748	12.08915	11.96894	11.4977	11.18779	12.98871
COPB2	201098_at	9.169201	9.124782	9.590908	9.34921	8.843429	8.261991	10.51389	9.828862	10.54322	10.56287	9.51764	9.711564	10.57136
USP9X	201099_at	9.028449	8.965073	9.031006	9.078625	8.60007	8.349144	9.127131	8.707003	9.222585	9.316029	8.445304	8.787503	8.404417

USP9X	201100_s_at	10.17238	10.22428	10.41334	10.50581	10.07264	10.07979	10.39362	10.33662	10.43202	10.54996	10.01669	10.1849	9.317936
BCLAF1	201101_s_at	5.218067	4.068259	6.063234	5.559696	5.975976	5.463348	6.143123	6.218094	6.015383	5.505133	6.884314	6.400317	7.657091
PFKL	201102_s_at	7.641031	7.599534	7.207948	7.209969	6.847238	7.043516	7.749205	7.752657	7.518373	7.688365	7.992329	8.093145	7.082605
NBPF10	201103_x_at	10.60497	10.75585	10.98283	10.68672	10.19334	9.802748	9.634671	8.911169	9.631418	9.455411	9.610653	8.949011	11.69407
NBPF10	201104_x_at	7.68833	7.757259	8.856632	8.038748	6.164718	5.871524	7.476425	4.95979	7.372654	7.470336	5.869746	4.945528	10.30088
LGALS1	201105_at	8.808426	8.778308	10.22067	10.19881	8.839946	8.708381	7.156161	7.244798	6.819054	7.195617	11.54672	7.810541	12.98693
GPX4	201106_at	12.25483	12.24666	11.81171	11.98171	11.61762	11.40662	10.89096	10.64065	10.81011	11.09569	11.03446	10.74666	10.64847
THBS1	201107_s_at	2.37353	2.354708	2.340247	2.339963	2.358126	2.338786	2.347169	2.355229	2.341388	2.332418	2.337849	2.350333	2.303988
THBS1	201108_s_at	3.436614	3.428095	3.71124	3.065799	2.609717	3.397613	3.427213	3.413593	3.40062	3.415046	3.34139	3.419474	6.106689
THBS1	201109_s_at	4.63351	4.443839	6.410983	4.385681	3.363099	3.662189	6.492674	3.775807	6.274186	5.475235	3.55309	3.502517	8.543509
THBS1	201110_s_at	9.456334	9.29827	10.1336	9.220194	7.483844	7.423968	10.14374	8.240111	9.661104	9.566637	8.440161	8.942419	11.14355
CSE1L	201111_at	7.494214	7.463493	6.79298	7.383305	7.427299	7.513192	9.325389	9.529414	9.445684	9.4249	9.727795	9.419209	9.893862
CSE1L	201112_s_at	9.37038	9.353317	9.250735	9.034885	9.117991	8.788383	11.33125	10.60993	11.45743	11.33944	10.26936	10.67838	10.8636
TUFM	201113_at	10.21868	10.26399	10.30095	10.38738	10.24615	10.03589	10.54695	10.31035	10.36049	10.32567	10.36212	10.53152	9.381624
PSMA7	201114_x_at	9.759464	9.697195	9.845086	9.977798	10.07266	9.92318	10.21238	10.25885	10.18791	10.22201	10.39816	10.36997	10.06732
POLD2	201115_at	9.589638	9.618906	9.194031	9.31756	9.752996	9.554248	8.989951	9.312958	9.22636	9.344656	9.446111	9.485822	8.387953
CPE	201116_s_at	2.834407	2.883597	2.97618	2.979984	3.089283	2.982373	2.992153	2.797277	2.787411	2.93483	4.917705	2.235192	10.09957
CPE	201117_s_at	5.416429	5.070055	4.883661	4.913968	6.210866	5.618963	4.687669	4.535829	3.599025	4.224793	7.997886	5.089107	11.12059
PGD	201118_at	5.654466	5.813475	6.347428	5.785741	4.93245	4.320333	8.173994	6.103958	8.116096	8.093145	6.490429	6.595186	8.692255
COX8A	201119_s_at	11.05712	10.98254	11.01097	11.04474	10.9435	10.79805	11.37528	10.92964	11.12756	11.43327	11.12745	11.15085	11.21087
PGRMC1	201120_s_at	8.624214	8.309368	10.42195	9.805916	8.00479	7.157476	8.883502	5.65686	8.528439	8.408156	5.775318	5.789759	9.595652
PGRMC1	201121_s_at	12.62847	12.64613	12.82143	12.84971	12.35776	11.70639	11.44046	10.4331	11.38127	11.37663	10.5495	10.3932	11.77407
EIF5A	201122_x_at	7.404451	7.543636	7.444477	7.31923	7.181031	7.060517	7.58745	7.382211	8.538213	8.682993	7.999335	8.472647	6.544755
EIF5A	201123_s_at	6.832313	6.326307	7.759878	6.315294	2.907207	2.807101	7.292366	4.355685	9.752939	9.8807	5.865807	6.934784	7.838114
ITGB5	201124_at	5.745866	5.442736	5.752068	5.739199	5.606202	4.898994	4.839981	4.31701	4.790959	5.09756	4.386442	4.748181	5.603042
ITGB5	201125_s_at	8.798602	8.622808	8.379323	8.369266	8.723459	8.903734	7.368466	7.884634	7.469138	7.525604	8.050002	8.167789	7.85847
MGAT1	201126_s_at	8.462385	8.016189	8.456634	8.453681	8.526065	8.584561	7.697216	7.745126	7.603874	7.682301	8.080392	8.287643	7.114197
ACLY	201127_s_at	4.998253	5.0373	5.020154	5.016988	4.334691	4.693071	7.75801	6.282452	8.109586	7.934387	5.550549	5.847766	7.303482
ACLY	201128_s_at	9.258472	9.13536	8.351444	8.265327	8.145129	8.090771	11.88693	11.01437	11.77898	11.68766	10.94408	11.18526	11.26949
SFRS7	201129_at	8.067249	7.995101	8.6602	8.787984	8.808344	8.581337	8.274477	8.451148	8.479439	8.363591	8.812769	8.226607	9.5521
CDH1	201130_s_at	2.377381	2.353972	2.528247	2.30822	2.355556	2.333231	3.299892	2.352202	2.378086	2.862543	2.336709	2.3573	2.583347
CDH1	201131_s_at	11.18353	11.18848	10.85738	10.87724	9.787525	8.935015	10.83456	10.36798	10.79335	10.58736	10.02917	10.21852	8.417855
HNRPH2	201132_at	9.350359	9.421642	9.788964	9.604199	9.086942	8.410607	9.646006	8.843248	9.458992	9.35682	8.785419	8.963693	10.43404
PJA2	201133_s_at	9.124774	9.090383	10.20374	9.796459	9.25893	8.914459	10.16344	8.590045	9.770277	9.770351	9.222205	8.947425	11.5466
COX7C	201134_x_at	12.6884	12.59108	12.64009	12.69629	12.7118	12.55014	12.38868	11.84129	12.23525	12.269	12.17716	12.06861	12.54703
ECHS1	201135_at	13.11316	13.03322	13.00785	13.1979	12.92157	12.85889	10.37852	10.90713	10.48136	10.45721	10.80735	10.72375	9.602192
PLP2	201136_at	2.836287	2.956169	3.380673	3.195157	3.474312	3.230684	5.264865	5.08119	6.569518	5.121122	4.83435	5.08198	6.127692
HLA-DPB1	201137_s_at	8.655196	8.769167	8.279152	8.132101	9.171862	9.619987	4.904015	4.724718	5.565491	5.122392	5.40562	4.819813	4.726106

SSB	201138_s_at	8.194788	8.110652	8.656885	8.638622	8.220853	7.747858	10.38054	9.666923	10.35338	10.20002	9.62987	9.708651	10.09742
SSB	201139_s_at	8.534358	8.418734	8.620644	8.856886	8.412375	8.081781	10.4404	10.38916	10.5229	10.52006	10.11371	10.14835	9.799274
RAB5C	201140_s_at	7.48765	7.474919	7.523113	7.346981	7.223257	6.801517	7.886923	7.195697	7.657841	7.905334	7.749414	7.341967	6.960533
GPNMB	201141_at	7.054693	6.828175	7.600152	7.630557	7.907043	8.347886	7.100917	6.258166	6.164131	5.909226	5.381715	3.245607	7.781663
EIF2S1	201142_at	7.774185	7.699377	7.84069	8.037709	7.356931	7.269174	8.211013	8.088215	8.371064	8.572709	7.872343	8.084832	8.056508
EIF2S1	201143_s_at	7.907508	8.263333	7.964114	7.759801	6.537504	6.762716	7.694412	7.182105	8.27062	8.535212	7.668509	7.849844	8.288016
EIF2S1	201144_s_at	8.516957	8.347882	8.64539	8.64926	8.371268	7.857179	10.01389	9.840402	10.12585	9.754498	9.317662	9.826295	9.06876
HAX1	201145_at	8.902207	8.843053	9.224194	9.38673	9.291735	9.025449	8.837111	8.653929	8.823441	8.814789	8.471578	8.826751	8.583275
NFE2L2	201146_at	11.14535	11.00591	11.2354	11.08311	10.54331	9.672852	10.31577	9.15741	10.31109	10.2755	9.065372	9.491686	9.95614
TIMP3	201147_s_at	5.15195	5.337012	6.653007	6.618565	6.155121	5.852509	6.392875	5.982319	6.672065	6.492567	6.017756	5.760504	10.07801
TIMP3	201148_s_at	5.15515	4.880218	5.554658	5.596954	5.614111	5.441374	5.831769	5.504324	5.634432	5.752258	5.592056	5.629766	8.285907
TIMP3	201149_s_at	4.254542	3.800829	5.853822	5.49796	5.255925	5.137771	5.945082	3.885733	5.974804	5.225381	4.993627	4.903448	8.766099
TIMP3	201150_s_at	8.245122	8.01346	9.614358	9.511214	9.226356	8.761634	8.346258	7.294284	8.654571	8.448302	8.275974	7.621825	11.96216
MBNL1	201151_s_at	5.65857	5.488214	7.27273	6.101126	5.647126	5.63821	8.792331	6.395249	9.023538	9.03675	6.686352	7.071437	9.156001
MBNL1	201152_s_at	9.444934	9.189424	9.605783	9.641412	9.772222	9.579587	10.82489	10.28723	10.69866	10.58955	10.31227	10.5593	9.602458
MBNL1	201153_s_at	9.232576	9.223047	9.22836	9.271884	9.111581	8.800109	10.18161	9.924242	10.21736	10.44929	9.673552	9.879961	8.89893
RPL4	201154_x_at	12.16977	12.40078	12.49686	12.63046	12.52198	12.43304	13.7723	13.52975	13.86651	13.80813	13.5946	13.71443	14.27513
MFN2	201155_s_at	9.521292	9.274973	8.971468	9.02327	9.489286	9.871148	8.430591	8.960052	8.432653	8.629856	9.364638	9.331837	7.883722
RAB5C	201156_s_at	5.215253	5.103955	6.333094	5.8661	4.960977	4.473835	6.554492	4.844672	5.768368	6.615581	4.756993	5.151612	6.12244
NMT1	201157_s_at	8.018288	7.941157	8.351338	8.652134	8.604006	8.699247	8.496903	8.490007	8.533346	8.456111	8.415782	8.597482	6.747966
NMT1	201158_at	7.131177	7.011233	6.991	6.976005	7.137217	7.164396	7.068328	6.763138	6.865771	6.917451	6.782699	6.714187	5.831347
NMT1	201159_s_at	2.943074	2.560244	3.142107	2.53692	2.560651	2.535385	2.563996	2.547722	3.073547	2.773249	2.282267	2.537051	2.652363
CSDA	201160_s_at	9.937599	9.865625	8.691834	8.763806	9.573608	10.21954	12.51552	12.94723	12.45849	12.87546	12.58434	12.54803	11.5604
CSDA	201161_s_at	5.193538	5.38254	4.722755	4.058684	4.814485	3.783179	9.866914	8.955113	9.659758	10.03596	8.355218	8.615987	8.670968
IGFBP7	201162_at	10.5371	10.56894	11.2148	10.88038	10.66209	10.48044	8.039585	5.798367	7.605245	7.636832	7.610103	6.361863	10.83208
IGFBP7	201163_s_at	11.94112	11.98921	12.34001	12.06958	11.92036	11.89654	9.434864	8.355775	8.949655	9.065172	9.284909	8.446498	12.17855
PUM1	201164_s_at	7.91139	7.931762	8.020128	7.919821	7.341415	6.438849	8.431164	7.660128	8.549765	8.447492	7.863179	7.742243	9.296647
PUM1	201165_s_at	8.444717	8.223209	8.702934	8.601152	8.776053	8.333906	9.523412	8.429341	9.491416	9.335081	9.004774	8.814097	9.910847
PUM1	201166_s_at	10.59939	10.49041	10.42316	10.29835	10.08769	9.961846	10.68468	10.24047	10.5864	10.75974	10.4309	10.4681	11.41299
ARHGDIA	201167_x_at	2.894794	2.891671	2.845744	3.066483	2.808865	2.858149	3.100232	3.34409	2.992424	2.866723	2.508398	2.86475	4.887444
ARHGDIA	201168_x_at	7.755333	7.929261	7.117259	7.145694	7.457056	7.067618	6.58456	6.543903	6.44896	6.519374	6.862905	7.020068	6.949487
BHLHB2	201169_s_at	2.35731	2.32399	2.470362	2.356378	2.358926	2.31509	2.378529	2.376383	2.360716	2.352498	2.340158	2.370122	2.310489
BHLHB2	201170_s_at	9.477011	9.300746	9.574015	8.604004	8.912772	8.206957	3.746137	2.837135	3.722043	3.112756	3.046	2.537656	5.55823
ATP6V0E1	201171_at	4.675778	4.672074	5.76685	5.638696	4.762953	4.47441	4.736844	4.312926	4.7519	4.752949	4.54901	4.768601	4.653151
ATP6V0E1	201172_x_at	9.153433	9.124534	9.248149	9.566561	9.478285	9.166257	9.002532	8.677571	9.193254	9.146689	8.825371	8.862385	8.412218
NUDC	201173_x_at	8.659583	8.907501	8.345302	8.573726	8.374844	8.321357	8.753875	9.295089	9.051309	9.11494	9.491944	9.696878	7.377362
TERF2IP	201174_s_at	8.768301	8.8305	8.858818	8.959736	8.755122	8.566265	10.52302	9.964033	10.53328	10.81079	9.8763	10.14589	10.01757
TXNDC14	201175_at	9.257518	9.34884	9.29135	9.300119	8.751046	8.661778	10.07422	9.506471	10.01832	9.939766	9.338667	9.587737	9.10464

ARCN1	201176_s_at	8.63388	8.890236	9.027742	8.925543	8.803579	8.658034	9.320369	8.823149	9.158656	9.017518	9.031619	8.895789	10.34463
SAE2	201177_s_at	10.5681	10.50391	10.44535	10.4189	9.727045	8.635114	11.68583	11.36062	11.90984	12.0546	11.02503	11.24413	11.74015
FBXO7	201178_at	9.994065	10.11474	9.760711	9.881395	9.892896	9.445161	11.48556	11.18028	11.21918	11.40423	10.66047	11.12525	8.254486
GNAI3	201179_s_at	4.868039	5.33475	5.631279	5.318397	4.438788	3.430936	6.936757	5.55075	7.106882	7.12813	5.31664	5.625982	8.629432
GNAI3	201180_s_at	9.402338	9.434293	8.9929	8.93255	8.911012	8.348494	9.99068	9.705372	9.860709	9.935627	9.939491	9.611118	10.72228
GNAI3	201181_at	7.388205	7.27611	6.68296	7.260213	7.314289	7.104934	7.443282	7.290581	7.585583	7.803853	7.499109	7.362158	7.909662
CHD4	201182_s_at	5.032219	5.044191	4.706042	5.173215	5.460554	5.296388	5.52223	5.327265	4.817424	4.87295	6.025426	6.296525	5.622659
CHD4	201183_s_at	8.569924	8.409625	7.947936	7.757321	8.175834	8.411824	8.47303	8.823666	8.360306	8.302082	9.207006	9.092874	8.874018
CHD4	201184_s_at	7.203516	7.084869	5.869215	6.174905	6.691648	6.725992	7.125072	7.614815	6.900255	7.003102	7.837796	7.564775	7.33504
HTRA1	201185_at	8.815782	8.8321	8.407293	8.479556	8.360646	8.188466	2.377981	2.907608	2.13017	2.029617	6.880904	2.611588	9.22264
LRPAP1	201186_at	8.536481	8.440514	8.365503	8.548286	8.49526	8.603634	7.68727	7.980127	7.868679	7.537241	8.172533	8.352541	7.259379
ITPR3	201187_s_at	2.758211	2.740057	2.709145	2.719538	2.753278	2.716208	2.727536	2.737911	2.714707	2.715778	2.724823	2.747693	2.608403
ITPR3	201188_s_at	2.606698	2.575488	2.575395	2.56465	2.479437	2.57471	2.587566	2.588948	2.578068	2.602674	2.573627	2.582575	3.915567
ITPR3	201189_s_at	3.932332	3.958637	3.478126	3.562878	3.628881	4.362909	3.553499	3.115376	3.492146	3.555592	3.728058	3.350959	5.635225
PITPNA	201190_s_at	7.626617	7.588013	7.389887	7.336909	6.675258	6.999384	7.361876	7.388904	7.291222	7.444587	7.347482	7.297053	7.327375
PITPNA	201191_at	6.767253	6.50894	6.403162	6.752435	5.403082	5.132079	6.5584	6.512625	6.920041	7.030029	5.982291	6.39048	6.482121
PITPNA	201192_s_at	6.798655	6.588135	6.359342	6.763672	7.076242	6.923282	7.7057	7.746106	7.596555	7.712687	7.368447	7.818196	6.745246
IDH1	201193_at	11.83955	11.79966	12.66703	12.44208	12.14071	11.57522	12.20066	11.29968	12.03731	11.99141	11.05679	11.20177	10.4719
SEPW1	201194_at	7.64863	7.572393	8.913385	8.769669	8.360985	7.935241	8.005565	7.970771	7.470145	7.587385	9.535923	8.552374	11.03252
SLC7A5	201195_s_at	4.639275	4.794376	5.274033	5.837107	4.944743	4.805647	10.10399	9.652484	10.00299	10.22683	9.051229	9.58071	6.054452
AMD1	201196_s_at	4.587882	4.286736	6.378289	5.87912	4.980712	4.495279	7.813101	6.979124	7.936597	7.899072	6.630973	6.926977	7.694232
AMD1	201197_at	7.720364	7.728983	8.341514	8.097256	7.86023	7.626826	9.746572	9.74834	9.616501	9.843282	9.517201	9.722535	9.179872
PSMD1	201198_s_at	7.380203	7.122762	7.806804	7.700058	7.187734	6.773929	9.097763	8.469155	9.076125	9.176415	8.485234	8.708742	8.492095
PSMD1	201199_s_at	8.618146	8.659404	8.287231	8.481362	8.82152	8.730291	9.79623	10.00115	9.931938	9.878011	9.932698	10.11103	8.613729
CREG1	201200_at	11.866	11.72886	12.13878	12.11459	10.88258	10.42561	10.66	10.11799	10.53965	10.65551	9.565094	10.05471	9.168481
CSTB	201201_at	9.896937	9.679288	10.40054	10.2214	9.696573	9.428037	10.92779	9.248031	11.03336	10.54063	10.08866	9.965083	10.46631
PCNA	201202_at	9.02615	8.767062	8.464569	8.435611	7.923436	7.616547	12.01382	11.39807	11.94921	12.05159	10.87752	11.13608	10.82855
RRBP1	201203_s_at	6.127829	6.093014	6.057584	6.22296	5.905428	5.337789	6.081388	5.791414	6.208591	6.284918	6.078875	6.11466	5.96962
RRBP1	201204_s_at	12.10578	12.10472	11.12036	11.31753	11.80085	11.77307	11.30833	11.82808	10.93861	11.15017	12.15386	12.07622	9.941293
RRBP1	201206_s_at	8.822118	8.650968	7.879412	8.273594	8.735985	8.777141	8.195049	8.313897	7.902471	8.211841	9.186207	9.061688	7.019357
TNFAIP1	201207_at	8.274793	8.284251	7.998443	7.904499	6.864192	6.282408	6.428916	6.293045	6.676971	6.244776	6.487269	6.341856	7.224877
TNFAIP1	201208_s_at	6.140543	5.98745	6.609923	6.549213	5.784046	6.108921	5.430729	4.644139	4.854822	4.34757	5.372373	5.1756	5.345484
HDAC1	201209_at	9.48912	9.462042	8.955698	9.074296	9.560719	9.519687	9.964543	9.549244	9.98399	9.876125	9.54209	9.941181	9.199345
DDX3X	201210_at	10.22497	10.11019	11.2613	10.92743	10.90862	10.53049	11.62634	10.96847	11.79475	11.76896	11.2779	10.88553	11.57651
DDX3X	201211_s_at	3.484605	3.996028	4.437485	3.877832	3.811568	3.748058	5.243596	4.063677	4.758709	4.610756	3.605961	3.998393	7.063771
LGMN	201212_at	9.325057	9.158848	9.670685	9.730045	9.245233	8.963507	9.546862	9.210342	9.259339	9.475463	8.154807	8.951403	7.935407
PPP1R7	201213_at	3.113195	3.087184	3.087287	3.084013	3.099916	3.083188	3.095158	3.098967	3.269495	3.555256	3.078663	3.092624	4.225497
PPP1R7	201214_s_at	7.920283	7.78854	7.741679	7.753877	8.39221	8.263399	7.803832	8.258141	8.100932	7.99318	8.4754	8.470752	7.863221

PLS3	201215_at	11.89759	11.91373	12.17474	11.92982	11.45873	11.02637	10.81308	10.16781	10.69969	10.70919	10.86027	10.29015	11.98574
ERP29	201216_at	10.39851	10.4887	10.60557	10.41406	10.64476	10.34537	10.368	10.32124	10.32828	10.34099	9.868959	10.37457	9.633766
RPL3	201217_x_at	13.53265	13.56384	13.58867	13.57562	13.77428	13.64933	13.86145	13.86426	13.84846	13.81012	14.0041	14.04305	14.12255
CTBP2	201218_at	7.137721	7.354038	7.70466	7.314271	7.21303	6.217189	10.5673	10.17438	11.04918	10.77679	9.574008	10.03176	10.86484
CTBP2	201219_at	4.568827	4.624156	4.219832	4.574597	4.881327	5.043165	6.169214	6.312667	7.204375	6.766863	6.08493	6.623101	5.443635
CTBP2	201220_x_at	7.793417	8.152414	7.699231	7.803091	8.419883	8.459709	10.18327	10.83776	10.44755	10.29428	10.72662	10.80682	10.13632
SNRP70	201221_s_at	8.945004	9.114367	8.284183	8.540356	9.383383	9.620815	8.533056	9.142786	8.466259	8.553623	9.330278	9.36115	8.145199
RAD23B	201222_s_at	8.363629	8.05246	8.628392	7.868894	7.592022	7.652417	7.595575	7.08643	7.558393	7.558751	7.386886	7.667991	7.769426
RAD23B	201223_s_at	10.21832	10.05394	10.38733	10.29693	10.04793	9.851723	9.479441	9.351451	9.976008	9.441338	9.393973	9.45624	9.754674
SRRM1	201224_s_at	6.30313	6.272201	6.524056	6.494128	6.693603	6.493171	7.146198	6.835206	7.008167	7.09694	6.609117	6.996454	7.192928
SRRM1	201225_s_at	8.889296	9.032148	9.240432	9.219646	8.837001	8.309065	10.63553	9.652638	10.78875	10.49287	9.364841	9.574351	10.10314
NDUFB8	201226_at	9.893109	9.79989	9.988189	9.950454	9.704985	9.445988	10.37345	9.844135	10.46483	10.52917	9.990911	9.965891	9.959719
NDUFB8	201227_s_at	11.8175	11.70037	11.27116	11.57055	11.58218	11.75775	11.69195	11.95952	11.66643	11.748	11.93997	12.10229	11.02548
ARIH2	201228_s_at	5.000164	4.922733	4.952795	4.656894	4.793148	4.68505	5.069163	4.916027	5.292485	5.669601	4.999265	4.825951	4.792085
ARIH2	201229_s_at	6.914121	6.89296	7.221074	7.086202	6.423441	5.866692	7.440602	6.372665	7.640683	7.583706	6.579353	6.711471	7.35925
ARIH2	201230_s_at	7.54092	7.169836	6.958055	7.199805	7.338751	7.336377	7.129652	7.137317	7.105551	7.191034	7.441312	7.38509	6.868708
ENO1	201231_s_at	10.11627	10.12341	10.29103	10.25513	10.28894	10.03897	10.90363	10.82653	10.80832	10.96663	11.17473	11.14308	11.03322
PSMD13	201232_s_at	8.698656	8.772326	8.108806	8.445132	9.184431	9.194329	8.877863	9.589663	8.88178	8.999346	9.70233	9.45813	8.691838
PSMD13	201233_at	7.860693	7.760187	7.498655	7.827527	7.975797	7.665076	8.168445	8.196368	8.174815	8.260109	8.517558	8.294946	7.913251
ILK	201234_at	8.281039	8.554673	8.399765	8.216365	8.054931	8.163148	9.295329	9.154963	9.404806	9.315668	9.391465	9.672799	9.247665
BTG2	201235_s_at	2.293001	2.288325	2.239887	2.271205	2.281559	2.266212	2.797104	2.287513	2.591139	2.270845	2.292668	2.296473	2.191636
BTG2	201236_s_at	7.699044	7.761419	5.328663	3.805131	6.609785	6.245938	8.208431	8.292216	8.449939	8.803736	7.475201	7.736793	5.605197
CAPZA2	201237_at	9.055217	9.017006	9.284098	8.967422	8.729419	8.103948	10.04598	9.360426	10.20056	10.26262	8.896966	9.028206	9.865786
CAPZA2	201238_s_at	9.824455	9.721715	9.994311	9.90274	9.139096	8.563518	11.44642	10.23354	11.24509	11.43614	9.670445	10.18537	11.42304
SPCS2	201239_s_at	8.460652	8.237668	9.576691	9.4309	8.563427	8.369947	9.86609	8.69376	9.929241	9.91516	8.605611	8.772358	9.522075
SPCS2	201240_s_at	10.49983	10.36161	11.05723	10.95847	10.4224	10.20075	11.28714	10.90369	11.3827	11.48939	10.75948	10.94225	11.55364
DDX1	201241_at	8.631003	8.627049	9.164226	9.243426	8.783373	8.303441	10.29496	9.962234	10.54495	10.43508	9.852208	9.9452	10.23465
ATP1B1	201242_s_at	12.67816	12.51974	12.21587	12.25888	12.04043	11.91857	10.75247	10.26286	10.71025	10.50923	9.98963	10.20888	11.47626
ATP1B1	201243_s_at	11.38588	11.4377	11.48662	11.34364	10.8049	9.994604	9.951532	8.903106	9.895053	10.14185	8.392419	8.674172	10.31071
RAF1	201244_s_at	8.669219	8.777992	8.804768	8.742569	8.441336	7.918949	8.913054	8.393664	9.170887	9.196774	8.276546	8.185032	8.937851
OTUB1	201245_s_at	7.008006	6.96538	7.085004	6.945841	6.822626	6.410603	7.763105	6.90091	7.827997	7.72273	7.380095	7.497285	8.345053
OTUB1	201246_s_at	3.269482	3.440834	3.345115	3.718022	3.163599	3.397167	3.645327	4.546847	3.995572	3.416446	4.069485	4.614202	4.519337
SREBF2	201248_s_at	4.796852	4.523886	4.041692	4.230428	4.752524	4.735987	5.225528	4.785657	5.131119	4.934716	5.265744	5.113677	4.944375
SLC2A1	201249_at	2.279457	2.262242	2.219475	2.234962	2.264565	2.229607	5.975656	5.308565	6.134713	6.082595	5.126084	5.52311	2.147784
SLC2A1	201250_s_at	2.708211	2.647124	2.642999	2.645401	2.719105	2.939194	10.10016	10.40615	9.996365	9.904285	9.805551	10.21069	5.927144
PKM2	201251_at	5.668858	5.261756	5.469996	5.26115	6.008377	6.081837	6.76158	6.308757	6.230578	6.003433	8.346062	6.348402	10.24
PSMC4	201252_at	7.396238	7.393362	7.551346	7.414685	7.450808	7.407766	8.972918	8.449638	8.993263	8.902341	8.653701	8.865678	8.127227
CDIPT	201253_s_at	11.3304	11.23745	10.91401	10.93915	11.47067	11.49521	10.49132	10.667	10.56055	10.48112	10.57632	10.74598	10.00312

RPS6	201254_x_at	12.97644	12.94148	12.61206	12.58759	13.12601	13.05686	13.94956	13.83628	13.88548	13.89067	13.81198	13.7187	14.00345
BAT3	201255_x_at	9.088539	8.997415	8.936858	9.083876	8.9611	8.621447	9.396949	9.244429	9.546954	9.70145	9.404995	9.584566	8.87061
COX7A2L	201256_at	8.954469	8.867712	8.609577	8.70885	8.860243	8.413375	8.930837	8.556353	9.40516	9.409545	8.671798	8.779283	9.451137
RPS3A	201257_x_at	13.61109	13.62893	13.41619	13.60805	13.79077	13.73415	14.19194	14.30348	14.20686	14.19839	14.3095	14.2003	14.36215
RPS16	201258_at	12.81242	12.75538	12.93994	12.96478	12.87543	12.71278	13.86109	13.53448	13.79556	13.90682	13.61515	13.55819	14.07573
SYPL1	201259_s_at	6.14205	5.536111	6.921412	6.610328	5.08361	5.623679	7.305159	5.577877	6.983556	7.030351	5.3634	5.450988	7.721914
SYPL1	201260_s_at	8.127186	8.047265	8.272329	8.04185	7.398635	7.070081	8.125141	7.536931	7.924567	8.285241	7.200503	7.318396	8.664966
BGN	201261_x_at	9.697054	9.751631	9.108309	9.353573	10.19675	10.82651	6.905608	7.061911	6.150528	6.328112	7.244718	7.472047	7.618737
BGN	201262_s_at	2.691203	2.675443	4.058864	3.546294	2.561009	2.653129	3.181278	2.69442	2.585467	2.547971	2.525099	2.694994	4.912787
TARS	201263_at	8.716886	8.680644	9.192825	9.177267	8.677595	8.1705	10.06402	9.928982	9.986778	9.907963	9.437641	10.00556	9.380216
COPE	201264_at	8.105971	8.094617	8.049667	8.010482	8.01411	7.720355	9.288597	8.782445	9.067659	9.345987	8.797646	9.144217	9.464659
TXNRD1	201266_at	7.493752	7.37842	7.621344	7.646914	6.737665	5.819606	8.746003	7.550448	8.663584	8.730584	7.382708	7.563601	8.010848
PSMC3	201267_s_at	7.634922	7.726752	8.310104	7.781356	6.968903	6.246921	9.514268	8.073253	9.527918	9.570192	8.03217	8.35122	9.361313
NME1-NME	201268_at	10.22596	10.21156	10.2769	10.34381	10.52711	10.40261	10.74622	10.76208	10.71928	10.98376	11.17203	10.98944	11.77509
NUDCD3	201269_s_at	2.458217	2.421002	2.325436	2.39831	2.434311	2.393697	2.416804	2.418534	2.402786	2.386572	2.39916	2.411535	2.2784
NUDCD3	201270_x_at	7.194702	7.296789	6.716	6.676177	7.489974	7.91601	6.266825	7.129141	6.503567	6.50681	7.443679	7.216062	6.642937
RALY	201271_s_at	6.533113	6.58284	6.042783	6.338046	6.631187	6.743385	7.806682	7.907665	7.58759	7.871063	7.9591	8.009605	7.688966
AKR1B1	201272_at	6.346213	6.300081	7.090348	6.937987	6.949799	7.271558	7.849436	7.907864	7.515799	7.770478	8.946534	8.327012	10.35502
SRP9	201273_s_at	11.37209	11.25739	11.63495	11.43865	10.89495	10.18448	12.13791	11.08398	11.98043	12.08216	11.24081	11.26713	12.75795
PSMA5	201274_at	9.069911	8.815079	8.776499	8.973567	8.525186	8.220586	9.38889	8.815487	9.348839	9.339617	8.80535	8.957459	8.285111
FDPS	201275_at	11.04547	11.02607	10.06332	10.03684	11.62653	11.5293	11.97223	11.90976	12.09394	12.00119	11.77858	12.19054	9.78472
RAB5B	201276_at	7.114234	7.071729	7.274249	7.001409	7.352437	7.020207	7.968224	7.196736	7.771405	8.0102	6.965221	7.330883	7.513374
HNRPAB	201277_s_at	9.042668	8.895119	9.02103	8.856648	8.768005	8.541735	10.99071	10.46653	11.25092	11.21655	10.20276	10.21812	10.55974
DAB2	201278_at	8.203021	7.953304	8.302446	8.331911	7.547823	7.188363	9.172098	7.875651	8.33012	7.869975	8.358355	8.475586	9.872723
DAB2	201279_s_at	6.250982	5.331214	6.588218	6.322652	5.071187	4.351313	8.243731	5.204797	7.398074	7.068197	6.027739	6.458558	9.038448
DAB2	201280_s_at	9.305052	9.150119	8.654742	8.739939	8.585691	8.395782	9.6444	8.586804	9.146427	8.660181	9.435559	9.719943	10.33316
ADRM1	201281_at	8.036352	8.095394	7.740108	8.17014	7.989454	8.179919	7.711587	7.910869	7.866776	7.679956	8.015269	8.025912	6.406007
OGDH	201282_at	6.402127	6.344223	6.410834	6.404752	6.007638	5.892786	7.152891	5.986949	6.912848	6.851242	6.108668	6.216797	6.275135
TRAK1	201283_s_at	3.595218	3.835556	2.954166	2.587348	2.585754	2.574859	2.563742	2.607382	2.416605	2.619561	2.561084	2.577293	2.780406
APEH	201284_s_at	7.688026	7.711235	7.39536	7.515754	7.725078	7.097335	8.763334	8.32645	8.700504	8.81849	8.226145	8.510329	6.043136
MKRN1	201285_at	8.722029	8.746755	8.550213	8.792851	8.765416	8.461287	9.847955	10.20689	10.08436	10.3408	9.668958	9.826197	8.574127
SDC1	201286_at	10.79211	10.8077	10.47328	10.74265	10.97436	10.96032	8.67153	9.143276	9.01664	9.016821	9.131221	9.287735	6.494037
SDC1	201287_s_at	10.21005	10.06843	10.17381	10.01182	9.87754	9.476869	8.600909	8.217175	8.905118	9.041712	8.201965	8.428755	6.75526
ARHGDIB	201288_at	8.964398	8.897439	9.368569	9.234613	9.007746	9.17375	9.755124	9.256564	9.631283	9.766045	8.986717	9.481343	7.690401
CYR61	201289_at	9.130842	9.254055	10.12733	10.0332	9.880776	9.842322	2.752259	2.523393	2.516144	2.782404	4.963346	3.320892	8.956358
SEC11A	201290_at	11.67789	11.61589	11.4804	11.53337	11.54055	11.63353	11.49177	11.70557	11.51128	11.40969	11.95178	11.88913	11.8253
TOP2A	201291_s_at	7.077029	6.517957	2.804257	3.064369	3.036964	4.655428	11.37337	11.75652	11.21884	11.25422	11.78336	11.85079	10.44686
TOP2A	201292_at	4.774456	4.770945	2.975059	3.060925	3.087059	3.133828	10.51564	9.490646	10.84112	10.79771	9.162857	9.086935	9.957899

PPIA	201293_x_at	12.09894	12.09541	12.0903	12.11595	11.71619	11.41159	13.17855	12.85272	13.18507	13.08534	13.0429	13.03854	13.17633
WSB1	201294_s_at	6.576873	6.625054	6.162727	6.178885	6.554458	6.647199	7.487688	6.68168	7.874475	7.254986	6.462425	7.260563	9.587768
WSB1	201296_s_at	9.963475	9.997421	9.958088	9.757285	10.04207	10.10636	10.76991	10.57318	10.85924	10.80034	10.78518	10.47411	11.73695
MOBKL1B	201297_s_at	5.345498	4.990722	5.20747	5.320943	5.305762	4.878963	5.380156	5.787823	4.942435	5.19899	4.917573	5.4483	6.074551
MOBKL1B	201298_s_at	6.501506	6.165318	7.282043	6.840863	6.569803	5.715025	7.583323	6.895807	7.63254	7.430209	6.097748	6.892064	7.519751
MOBKL1B	201299_s_at	3.255178	3.250627	3.471529	3.144115	3.362095	3.23069	3.30789	3.248927	3.546604	3.408893	3.232494	3.321868	5.3042
PRNP	201300_s_at	10.70248	10.53553	10.69181	10.76223	10.70374	10.81951	10.22248	10.67863	10.35406	10.63622	10.30293	10.52362	11.03168
ANXA4	201301_s_at	9.15918	9.088489	9.934575	9.701428	9.075338	8.581253	8.437837	6.521114	8.157679	8.208302	6.767531	7.245793	7.858985
ANXA4	201302_at	9.865016	10.01613	10.5611	10.39307	9.609354	8.713899	8.606266	7.617549	8.204837	8.334867	6.991502	7.631275	7.629425
EIF4A3	201303_at	8.497998	8.456972	8.859893	8.763725	8.446832	8.230212	9.633255	8.938853	9.884168	9.85592	9.134622	9.313376	10.11039
NDUFA5	201304_at	10.49196	10.36211	11.11297	10.93921	10.34613	10.16321	10.64165	10.29864	10.45585	10.74142	9.921926	9.951378	11.76639
ANP32B	201305_x_at	11.06125	10.80791	10.38066	10.60532	11.12296	11.34556	12.14829	12.62323	12.10941	12.20794	12.54188	12.85118	10.50089
ANP32B	201306_s_at	11.68542	11.51791	11.17563	11.27467	11.46914	11.58459	13.42988	13.39859	13.28865	13.24872	13.27484	13.41599	12.45759
SEPT11	201307_at	7.326778	7.140951	7.848738	7.642262	6.949123	6.844288	8.436028	7.523191	8.162606	8.048557	8.767455	7.904063	9.929067
SEPT11	201308_s_at	3.129884	3.09022	3.103752	3.108444	3.123685	3.109091	4.34051	3.063958	4.210607	3.492516	3.073219	3.112422	8.41939
C5orf13	201309_x_at	4.740486	4.476091	4.471611	4.170238	3.71694	3.41191	6.896917	4.718588	6.555685	6.78936	4.611757	4.922595	11.15109
C5orf13	201310_s_at	8.31695	8.410753	7.462281	7.488362	7.48247	7.111198	10.62406	10.0804	10.40222	10.43476	10.54126	10.1636	12.62905
SH3BGRL	201311_s_at	6.499707	6.437162	7.005152	6.862112	6.501867	6.458441	9.394009	7.914961	9.63222	9.473498	7.568975	8.635984	9.720057
SH3BGRL	201312_s_at	8.290904	8.112329	9.085093	8.775968	8.022667	7.195935	10.49159	9.596848	10.68814	10.69019	8.835453	8.972188	10.7086
ENO2	201313_at	2.248627	2.221895	2.195851	2.210768	2.245979	2.203285	2.735235	2.31443	3.838291	3.052399	2.925237	3.474462	7.333621
STK25	201314_at	6.19273	6.102173	5.994399	5.853877	6.147419	5.647113	6.560109	6.375597	6.940699	6.474978	7.01334	6.783647	7.287995
IFITM2	201315_x_at	11.48901	11.47684	11.55953	11.42717	11.14294	10.96513	8.201055	7.701321	8.131999	8.511301	8.849878	8.179358	9.903087
PSMA2	201316_at	8.032429	8.062155	8.748498	8.683107	8.36254	8.243395	7.416865	7.497097	7.573672	7.430703	7.5853	7.624853	8.165065
PSMA2	201317_s_at	10.78767	10.73458	11.24401	11.19277	10.50761	10.16094	11.055	10.08976	11.021	11.10115	10.35901	10.36001	10.89569
MRCL3 /// M	201318_s_at	12.52507	12.50746	12.3677	12.2654	11.95902	11.60424	12.33022	11.59294	12.27335	12.51161	11.82108	11.76442	12.99965
LOC645094	201319_at	10.09414	9.997206	10.14962	9.979298	9.866818	9.703722	9.87908	9.732568	9.980203	9.969251	9.872885	9.748975	10.20265
SMARCC2	201320_at	7.211724	7.341036	7.017039	6.89403	7.338959	7.052757	7.367315	7.448098	7.395681	7.431406	7.507312	7.614251	7.129232
SMARCC2	201321_s_at	7.156965	7.255013	7.404576	7.287396	7.337798	7.153852	7.352776	7.498778	7.413397	7.811783	8.114308	7.489353	7.933546
ATP5B	201322_at	11.15177	11.02575	11.29967	11.3163	10.96305	10.51589	12.03766	11.47154	12.00255	12.07095	11.53157	11.58549	11.69311
EBNA1BP2	201323_at	8.251262	8.236019	8.123703	8.514696	8.236391	8.342935	8.083799	8.561716	8.276987	8.262716	8.340018	8.621368	7.333455
EMP1	201324_at	5.215253	5.196641	8.522623	7.39648	6.42635	6.14061	4.42954	4.45224	4.186351	4.444706	7.977479	4.211394	10.88326
EMP1	201325_s_at	3.373925	3.268273	5.710213	4.014555	3.108037	3.145293	3.401796	3.22544	2.777886	3.220994	3.626581	3.092553	8.577563
CCT6A	201326_at	8.923973	8.855987	8.045491	8.536084	8.829928	8.889721	9.61584	9.925056	9.562877	9.671429	10.09205	9.964245	10.01698
CCT6A	201327_s_at	8.884583	8.738995	8.533409	8.753448	8.682828	8.588684	10.7241	10.34518	10.58854	10.81389	10.425	10.51586	11.07806
ETS2	201328_at	9.207294	9.279276	9.754083	9.649051	9.356271	8.891767	6.005264	5.718148	6.250159	6.562209	6.154786	6.217123	7.410543
ETS2	201329_s_at	5.873979	5.865658	7.743884	6.893692	5.709988	5.459036	4.504986	3.787935	4.604902	3.898469	3.429867	3.474854	5.980212
RARS	201330_at	8.848723	8.83142	8.878636	8.827276	8.685228	8.799105	8.839615	8.838751	8.834332	8.827404	8.822604	8.833201	9.602838
STAT6	201331_s_at	9.142597	9.29771	8.810074	8.809909	9.42904	9.376156	7.557059	7.404249	7.750027	7.871493	7.648547	7.767188	6.819845

STAT6	201332_s_at	2.321202	2.307656	4.120133	2.847693	2.145762	2.603842	2.306822	2.298905	2.487852	2.280387	2.10349	2.234456	2.417946
ARHGEF12	201333_s_at	7.215445	6.494828	6.83945	7.134647	7.687761	7.811131	6.152969	7.067768	5.805951	6.254591	7.262364	7.135219	4.644143
ARHGEF12	201334_s_at	10.94937	10.90033	11.41069	11.08991	10.62787	10.15963	10.40757	9.808613	10.64121	10.62062	9.428206	9.811665	10.1918
ARHGEF12	201335_s_at	4.523173	4.330926	4.973505	4.677463	4.200539	4.098743	3.745601	4.06792	4.242453	3.891494	3.519236	4.057411	3.872484
VAMP3	201336_at	10.05698	10.08196	10.30453	10.57828	10.03355	9.799563	10.01229	9.413113	9.64803	9.624055	9.977892	9.77206	10.13475
VAMP3	201337_s_at	3.476242	2.888712	4.665245	3.919521	2.94597	2.866942	5.331073	2.856183	5.084226	4.683361	2.755306	2.974633	7.32798
GTF3A	201338_x_at	11.23783	11.37508	10.7789	11.14414	11.13527	11.02968	11.53343	12.01826	11.60947	11.52956	11.75388	11.96953	10.25499
SCP2	201339_s_at	11.85458	11.61989	12.83205	12.61425	11.43888	10.58513	10.88563	8.76028	10.86009	10.84865	8.538166	9.071271	9.984448
ENC1	201340_s_at	6.866625	6.409441	7.379936	7.032779	7.641455	7.618981	6.796332	6.837228	6.8221	6.767501	7.614919	7.2807	9.771045
ENC1	201341_at	6.924229	6.865889	7.157651	6.846998	6.856483	7.097857	6.904115	6.648191	6.987585	6.984169	6.424701	6.786084	9.861429
SNRPC	201342_at	6.890752	6.813746	6.929237	6.935944	6.845559	6.834412	8.111093	7.939708	8.0061	8.053505	8.055874	8.134699	7.58531
UBE2D2	201343_at	8.549268	8.346708	8.498113	8.460902	7.888493	7.111044	9.437036	8.830186	9.477925	9.281292	8.732818	9.128975	9.915456
UBE2D2	201344_at	6.025454	5.913432	5.224909	5.607533	5.680595	5.987047	5.659263	6.275744	5.584696	5.637305	5.914676	6.457012	7.082089
UBE2D2	201345_s_at	8.035812	8.119577	8.168828	8.193403	8.030436	8.244956	9.02049	9.209692	9.132146	9.154419	9.093236	9.286548	8.825838
ADIPOR2	201346_at	9.284239	9.27876	8.717659	8.91144	8.389612	8.046504	7.679846	7.278587	7.782458	7.812425	6.913744	7.319889	6.746521
GRHPR	201347_x_at	11.37173	11.27216	11.86849	11.71999	11.32952	11.08891	9.114866	8.999008	9.341692	9.034671	8.750277	8.917185	8.329146
GPX3	201348_at	11.15759	11.13812	11.27548	11.10707	10.72384	10.37524	10.18081	8.63228	9.89512	9.755248	8.633479	9.416724	7.39674
SLC9A3R1	201349_at	9.46798	9.47876	8.830857	8.768783	9.221454	9.119045	8.525968	8.723947	8.475576	8.621031	8.497213	8.726394	5.594358
FLOT2	201350_at	4.98905	4.862434	5.270402	5.243984	5.38301	5.644804	7.356024	7.192496	7.112972	7.432165	7.068408	7.573104	4.71134
YME1L1	201351_s_at	8.684824	8.621454	8.909498	8.692314	8.32815	7.527605	9.92473	9.166532	9.853254	9.616505	8.929112	9.36463	10.03308
YME1L1	201352_at	10.91436	10.77299	11.14864	11.0913	10.32559	10.12202	11.15109	10.89549	11.31955	11.31232	10.72523	10.7689	11.31468
BAZ2A	201353_s_at	6.541989	6.91562	6.43485	6.220487	6.664243	6.738495	6.965978	6.72139	6.925374	6.916608	6.56839	6.578287	6.346679
BAZ2A	201354_s_at	6.02327	6.05441	5.70616	5.966495	6.018122	6.286434	6.715176	6.465676	6.735144	6.871388	6.311763	6.346613	5.826554
BAZ2A	201355_s_at	3.050521	3.033173	3.01833	3.337544	3.343702	3.033528	3.034599	3.170646	3.01465	3.017359	3.028569	3.031896	2.969758
SF3A1	201356_at	7.820883	7.682482	7.948235	7.896658	7.442549	7.377144	8.914688	8.696198	8.888875	9.06722	8.575311	8.923239	7.880004
SF3A1	201357_s_at	4.321727	4.236285	4.857758	4.304021	4.301789	4.287307	6.407324	5.316907	6.27339	6.024861	4.499786	5.015734	4.92932
COPB1	201358_s_at	11.5246	11.50058	11.27531	11.40186	11.56827	11.63515	12.01082	12.02687	11.99122	11.85247	12.1011	12.14359	12.18301
COPB1	201359_at	8.657965	8.556834	9.451325	9.142818	8.082994	7.118561	10.07398	8.315829	10.14894	9.985637	8.587999	8.68046	10.94754
CST3	201360_at	10.12923	10.17051	10.81167	10.97947	10.01015	9.93576	9.420743	9.563186	9.381822	9.262564	9.177484	9.528442	8.441114
TMEM109	201361_at	5.970754	5.954802	6.391933	6.559374	6.290704	5.984479	6.64757	6.29839	6.836837	6.735397	6.430421	6.685234	6.331325
IVNS1ABP	201362_at	7.147331	7.167366	7.507942	7.523938	8.158947	7.80697	8.026836	8.094875	7.939409	8.097896	8.004602	7.942728	8.224603
IVNS1ABP	201363_s_at	7.054709	7.04046	8.058364	7.625388	7.720308	7.813314	8.281651	7.782959	8.139669	8.296453	7.429427	7.67514	8.918834
OAZ2	201364_s_at	7.394086	7.191616	7.05428	6.719258	7.0651	7.043414	7.027759	6.559582	6.752532	6.935493	6.493164	6.837313	8.172902
OAZ2	201365_at	7.118017	6.653845	6.851959	6.54822	7.195608	6.976637	6.018703	6.164542	6.167455	6.193415	6.012037	6.438572	7.136368
ANXA7	201366_at	10.52972	10.36819	11.01236	10.9995	10.43009	10.31127	10.51041	10.40595	10.38146	10.60342	10.00911	10.05927	8.951923
ZFP36L2	201367_s_at	2.691057	2.67184	3.875763	2.668787	2.318248	2.315357	4.053425	2.319528	3.83393	3.709306	2.300203	2.311934	5.331523
ZFP36L2	201368_at	10.04052	10.01845	10.71231	10.59415	9.817055	9.577568	10.38196	9.80196	10.30154	10.46142	9.641386	9.736376	10.57985
ZFP36L2	201369_s_at	7.890083	7.922617	7.960981	8.056585	7.615277	7.585811	7.077726	7.187916	6.917516	7.245006	7.11687	7.279269	6.476493

CUL3	201370_s_at	6.447924	6.233885	7.305654	6.829978	6.409722	5.951154	7.323736	6.867886	7.551544	7.693412	6.725742	6.621582	7.95043
CUL3	201371_s_at	9.956456	9.907662	10.1032	10.20414	9.766289	9.393692	10.46938	10.08944	10.40285	10.54642	10.10587	10.03634	10.94344
CUL3	201372_s_at	3.151647	2.821395	3.119691	3.128948	3.129498	3.57307	3.794662	3.938987	3.201403	3.126366	3.121435	3.257798	3.528574
PLEC1	201373_at	5.696596	5.901618	6.005041	6.345753	5.796281	5.549597	5.202311	5.6965	5.316758	4.993768	5.310858	5.355541	5.929247
PPP2CB	201374_x_at	2.167895	2.147431	2.125314	2.130087	2.150145	2.129683	2.140595	2.146519	2.132258	2.125795	2.13335	2.159864	2.067739
PPP2CB	201375_s_at	10.38284	10.33642	10.69265	10.45244	9.341354	8.738501	10.44566	9.5013	10.41295	10.3531	9.447977	9.478628	11.50507
HNRPF	201376_s_at	7.493306	7.538471	8.089185	7.651637	7.00357	6.826918	8.444083	6.904738	8.568683	8.479885	6.372814	7.09714	8.688923
UBAP2L	201377_at	6.923811	6.91998	6.290345	6.513321	6.953195	6.77228	8.128821	8.487148	8.348994	8.26501	8.33691	8.560082	7.101433
UBAP2L	201378_s_at	5.773679	5.723474	5.663434	5.657746	5.581288	5.479343	7.683318	6.877141	7.915046	7.758782	6.870276	6.979582	8.274041
TPD52L2	201379_s_at	7.598118	7.676045	7.533969	7.257971	7.78583	7.330203	8.232851	8.221466	8.299877	8.212928	8.185112	8.063106	8.620299
CRTAP	201380_at	5.856867	5.991699	6.135289	6.133673	6.446094	6.813423	4.834215	5.54446	4.980863	5.095177	6.14656	5.11692	6.729984
CACYBP	201381_x_at	8.246271	8.208728	8.441235	8.557953	8.303721	8.213002	10.43473	10.17008	10.56231	10.57504	9.494686	10.18061	9.826661
CACYBP	201382_at	4.457578	4.205991	4.157979	4.296958	4.379096	4.581522	4.277188	5.021989	4.343865	4.082167	4.462186	4.547795	3.992641
NBR1	201383_s_at	4.759173	4.568621	5.840212	5.33789	4.686054	4.777174	5.228056	4.346367	5.934252	5.566594	4.21153	4.341085	5.995384
NBR1	201384_s_at	10.01342	9.976405	10.44644	10.41588	10.13065	9.370761	9.649685	9.386449	9.958572	9.845356	9.218412	9.406463	9.384707
DHX15	201385_at	10.14506	10.16229	10.37481	10.20421	10.01851	9.75863	11.46423	11.12698	11.56764	11.5119	11.09261	11.13297	11.66143
DHX15	201386_s_at	6.273546	6.431204	7.314466	7.093396	5.569908	4.109425	9.291215	7.029174	9.452778	9.425278	7.197455	6.894387	10.34874
UCHL1	201387_s_at	2.150815	2.143666	2.120455	2.126382	2.136453	2.128031	2.141381	2.142677	2.134605	2.129204	4.758452	2.133814	10.57665
PSMD3	201388_at	7.38483	7.164809	7.432657	7.280586	6.922451	6.72032	7.57277	7.395741	7.328526	7.66143	7.568909	7.554375	6.836232
ITGA5	201389_at	7.144319	7.271455	7.010508	7.475665	6.729796	6.676811	7.212773	6.954106	7.027883	7.1007	7.367459	6.922472	6.701026
CSNK2B	201390_s_at	9.176917	9.078497	9.065106	9.206632	8.7831	8.565167	9.959475	9.845491	9.875394	10.06104	9.497279	9.851786	9.79207
TRAP1	201391_at	9.689583	9.614505	9.170525	9.433404	9.492574	9.565212	9.620664	9.994523	9.889083	9.882917	10.1784	10.21216	7.963875
IGF2R	201392_s_at	6.817444	6.237258	7.216805	7.055122	6.237741	5.567228	6.018133	4.916955	5.74953	5.479578	5.170191	4.698706	6.146472
IGF2R	201393_s_at	8.932745	8.954232	9.469552	9.011545	7.949739	6.967323	7.857355	6.689196	8.272483	8.071063	6.948028	6.87527	8.403258
RBM5	201394_s_at	7.534383	7.361834	7.447406	7.30936	7.13761	6.600205	7.976538	7.022739	8.031501	7.975611	7.572304	7.131189	8.839693
RBM5	201395_at	9.691068	9.764903	8.994783	8.950174	9.639952	9.628602	9.586511	9.769591	9.825585	9.729561	9.90642	9.806885	10.25859
SGTA	201396_s_at	5.123078	4.907729	4.502034	4.797153	4.92787	5.2417	4.910779	5.278621	4.936433	4.92185	5.902715	5.306929	5.434554
PHGDH	201397_at	7.803446	7.817124	8.29853	8.229264	8.716913	8.367912	8.373422	8.522935	7.938529	8.614347	8.509706	8.708423	7.340039
TRAM1	201398_s_at	11.04316	11.01994	11.1169	10.95924	10.94864	10.38411	11.09113	10.76637	11.16693	11.08703	10.58821	10.8588	10.81514
TRAM1	201399_s_at	7.759481	7.445215	8.662504	8.446478	7.762393	7.478884	8.986444	7.234938	9.098311	9.158857	6.891113	7.093471	9.586126
PSMB3	201400_at	9.847601	9.847335	9.887773	9.979625	10.19655	9.859237	10.57028	10.3344	10.58433	10.66016	10.4058	10.60001	9.716848
ADRBK1	201401_s_at	2.858046	2.86507	2.749676	2.80176	3.034793	2.852666	2.863631	3.018449	2.855706	2.84016	3.05313	2.847464	4.013249
ADRBK1	201402_at	2.256248	2.241881	2.205228	2.224256	2.232904	2.226234	2.197944	2.237699	2.224548	2.217007	2.226216	2.232471	2.166719
MGST3	201403_s_at	9.713961	9.501898	10.3614	10.24184	9.792627	8.865431	12.25595	11.03317	12.24923	12.27162	10.83043	11.22843	10.26567
PSMB2	201404_x_at	6.131417	6.090329	6.628147	5.98929	5.819815	5.989331	6.626563	6.088493	6.268324	6.105823	5.798717	6.045765	6.160606
COPS6	201405_s_at	8.138745	8.103577	8.175747	8.046767	8.441958	7.925628	9.466859	9.251386	9.335728	9.512235	9.182666	9.377058	9.142231
RPL36A	201406_at	11.50035	11.46943	11.52721	11.51042	11.64618	11.40011	13.2371	12.85105	13.38791	13.42323	12.8851	12.9654	13.62697
PPP1CB	201407_s_at	8.55236	8.54395	8.850938	8.646233	8.663152	7.670695	9.049604	8.898639	8.978292	9.278391	8.673228	8.842223	9.762754

PPP1CB	201408_at	7.538842	7.487085	8.2549	7.984052	6.936656	6.414496	7.447419	6.807011	7.990541	8.002195	6.751892	6.804651	8.764
PPP1CB	201409_s_at	8.482853	8.477928	8.752114	8.450218	8.67454	8.462313	8.749061	8.930293	9.329438	8.866477	8.796659	8.772176	9.250872
PLEKHB2	201411_s_at	7.111325	6.967597	7.49671	7.149646	6.965506	6.530332	7.848968	7.341038	7.821467	7.579703	6.941406	7.330885	8.294755
LRP10	201412_at	8.353262	8.287122	8.453853	8.44999	8.379116	8.620615	9.21755	9.458225	9.316765	9.279586	9.045598	9.279422	7.944981
HSD17B4	201413_at	10.82789	10.60105	11.57212	11.53583	11.08973	10.52197	11.21916	10.37052	11.40025	11.18024	10.07084	10.3505	9.421368
NAP1L4	201414_s_at	7.118978	6.896895	6.644624	6.796371	7.013469	6.912524	8.717031	8.853011	8.796595	8.760708	8.813464	8.933461	8.034381
GSS	201415_at	8.443909	8.420045	7.844906	8.07081	8.55489	9.000244	7.479836	8.634713	7.496774	7.415876	8.676765	8.716052	7.066287
SOX4	201416_at	1.97555	1.893281	1.864251	1.866558	1.882495	1.954635	2.652078	3.282443	2.091365	2.374519	5.67136	3.78888	6.670068
SOX4	201417_at	5.43058	5.401302	4.977023	4.174372	4.138655	4.17139	8.326649	6.95661	8.019123	8.258505	8.32271	7.214557	11.64289
SOX4	201418_s_at	6.132429	5.870832	4.344802	4.424446	6.3624	7.279659	6.481689	7.905277	6.394925	6.995899	9.596646	8.270465	9.443206
BAP1	201419_at	6.177708	6.218858	6.257379	6.401165	6.173447	6.266561	7.213786	7.472962	7.405838	7.59567	7.078561	7.690252	6.318898
WDR77	201420_s_at	4.173492	4.206772	4.206095	4.151283	3.832615	4.15501	4.733205	4.979916	5.742712	6.056115	4.758747	4.814153	6.094393
WDR77	201421_s_at	6.844028	7.042709	6.864735	6.903768	6.91885	6.901044	7.217904	7.318563	7.369628	7.21974	7.345806	7.280065	7.551891
IFI30	201422_at	7.705037	7.906345	8.229423	8.48956	8.446424	8.570022	6.819473	6.825523	7.187335	6.745379	6.345614	6.666307	5.560051
CUL4A	201423_s_at	7.578125	7.521968	7.59053	7.412525	6.671133	6.380483	8.608785	7.815823	8.698853	8.730614	7.599404	7.79422	7.229849
CUL4A	201424_s_at	10.07416	10.03613	9.592345	9.587681	9.722001	9.340201	10.41778	10.21595	10.48411	10.6318	9.818436	9.83602	9.525015
ALDH2	201425_at	13.50373	13.40033	13.92372	13.80546	13.22317	12.89983	11.19111	10.27156	11.22865	11.14835	10.13815	10.37184	8.425413
VIM	201426_s_at	8.611546	8.859773	9.816327	9.339726	9.095936	8.798747	10.55225	8.633978	9.458578	9.894014	11.29121	9.291511	13.94919
SEPP1	201427_s_at	14.4813	14.55209	14.35259	14.45905	14.32466	14.37564	13.69129	13.79448	13.92148	13.94878	13.49425	13.70091	11.87884
CLDN4	201428_at	3.260893	3.24839	3.207225	3.229419	3.246367	3.21812	3.216528	3.243406	3.230642	3.221266	3.225224	3.23966	3.183884
PLK1	201429_s_at	14.33826	14.30611	13.86119	14.18371	14.36496	14.42257	14.56113	14.59059	14.51707	14.50457	14.75088	14.69545	14.71983
DPYSL3	201430_s_at	2.506848	2.477891	2.439881	2.457867	2.490015	2.452049	2.466471	2.478176	2.464471	2.459935	2.550731	2.470104	7.088367
DPYSL3	201431_s_at	2.312808	2.418648	2.496228	2.262981	2.273699	2.436357	2.204342	2.235419	2.056036	2.136692	5.496437	2.201123	9.145905
CAT	201432_at	12.85421	12.79403	13.6386	13.60617	12.90049	12.48415	13.08341	12.58527	12.88012	13.0693	11.84093	12.41859	9.591203
PTDSS1	201433_s_at	7.708405	7.423038	7.69597	7.990387	8.419801	8.376127	8.637767	8.678383	8.498979	8.420564	8.807666	8.846019	8.57938
TTC1	201434_at	7.342087	7.251615	7.461921	7.470754	7.279629	7.247133	7.476505	7.492836	7.801215	7.915716	7.464359	7.778204	7.929464
EIF4E	201435_s_at	5.844821	5.801153	7.370824	6.790317	5.217616	4.261593	8.976502	6.604464	9.017638	9.093071	6.000141	6.586385	9.243035
EIF4E	201436_at	6.711693	6.65097	6.842941	7.05301	6.732695	6.175778	8.452769	8.407539	8.44558	8.364135	8.068963	8.35331	8.56806
EIF4E	201437_s_at	9.209645	8.963722	9.037128	9.157125	9.169979	9.210285	10.2971	10.35215	10.29045	10.28504	9.873747	10.15622	9.901785
COL6A3	201438_at	8.547996	8.692123	8.722158	8.406858	7.471332	8.063589	9.660735	7.848351	8.639509	8.772691	10.33445	8.591173	13.0151
GBF1	201439_at	6.006036	5.858117	6.028707	6.119467	6.164665	6.24209	6.686642	6.775064	6.661952	6.570678	6.789162	6.995719	6.127298
DDX23	201440_at	7.58129	7.542515	7.380376	7.339911	7.308967	7.377582	7.685931	7.648161	7.923485	7.929374	7.839215	7.963883	7.383179
COX6B1	201441_at	11.36343	11.31147	11.055	11.25853	11.32674	11.31597	11.33165	11.63761	11.27407	11.37002	11.58418	11.85308	11.28417
ATP6AP2	201442_s_at	2.410267	2.396312	2.334892	2.358707	2.393974	2.346603	2.365442	2.379168	2.363665	2.352103	2.362006	2.37379	2.242452
ATP6AP2	201443_s_at	11.66114	11.53894	11.70021	11.77307	11.43403	11.2754	12.31407	11.67596	12.38097	12.42214	11.57515	11.56242	12.64493
ATP6AP2	201444_s_at	7.080409	7.037906	8.244004	7.741036	7.012925	6.56635	9.19354	7.137967	9.123915	9.199551	7.139331	7.432129	10.05369
CNN3	201445_at	6.173121	6.026499	7.928996	7.245055	5.640746	5.010893	7.382565	4.679793	7.481091	6.784102	6.113119	5.735189	10.18716
TIA1	201446_s_at	5.606054	5.833184	5.652995	5.482857	5.707758	5.197202	7.020184	5.595718	6.831878	6.939544	5.578092	5.82819	9.651825

TIA1	201447_at	5.800566	5.881122	6.248025	5.627162	5.493788	5.330899	7.216929	6.167629	6.850508	6.896583	6.341016	6.310905	9.300081
TIA1	201448_at	8.828191	8.862445	8.483523	8.228436	8.519186	8.104339	9.296685	8.405196	9.267813	9.193127	9.000951	8.512381	11.14976
TIA1	201449_at	7.679908	7.791699	7.355963	7.50324	7.647226	7.671819	7.875784	7.772191	7.546149	7.586198	7.684056	7.357888	8.700016
TIA1	201450_s_at	7.90413	7.910833	7.857462	7.496126	7.693761	7.754386	9.094951	8.422875	8.785089	9.063791	8.364394	8.227669	10.90454
RHEB	201451_x_at	3.912343	3.792359	6.530996	6.403025	3.672075	3.102933	3.07151	3.099524	3.090988	2.872768	3.048101	3.092818	2.367418
RHEB	201452_at	2.465608	2.51778	3.537381	3.863433	2.534284	2.497959	2.502532	2.40951	2.858383	2.491758	2.393425	2.507619	3.639163
RHEB	201453_x_at	9.896702	9.834093	10.44563	10.16073	9.771914	9.173164	10.92117	10.13091	11.07308	10.89419	9.997387	10.21227	11.01531
NPEPPS	201454_s_at	7.546334	7.305478	7.302293	7.64666	7.679983	7.497075	7.250918	7.104178	7.001041	6.851813	7.756565	7.399141	7.816527
NPEPPS	201455_s_at	7.409712	7.114332	7.250902	7.032406	6.226357	6.209482	7.110821	6.074024	7.093897	7.098066	6.569883	5.939923	8.738132
BUB3	201456_s_at	6.641195	6.409374	7.162148	6.601508	6.810412	6.227682	8.488883	7.441659	8.108834	8.029244	7.719267	7.651234	8.45196
BUB3	201457_x_at	6.499277	6.476882	7.570838	7.404216	6.862005	6.372995	9.922831	8.990078	9.877143	9.836935	8.682289	8.880947	9.19943
BUB3	201458_s_at	7.338205	7.211269	7.308288	7.294145	6.831199	6.698184	8.713985	7.931506	8.727733	8.74595	7.820425	8.070785	8.995089
RUVBL2	201459_at	8.012917	7.622191	7.756809	7.83845	8.127497	8.226056	9.332465	9.545184	9.559267	9.573006	9.45408	9.713781	9.061192
MAPKAPK2	201460_at	7.223377	7.061086	7.112456	7.315578	7.336485	7.430259	5.907061	6.386616	5.774835	5.964996	7.04808	6.802689	6.799902
MAPKAPK2	201461_s_at	3.305509	3.282186	3.709024	3.278362	3.29351	3.252764	4.284642	3.285703	3.428752	3.552912	3.271314	3.460332	5.101056
SCRN1	201462_at	3.664355	3.684391	3.782197	3.868321	4.249991	3.877674	4.873616	4.219862	4.700815	3.989357	6.612838	4.930534	8.692775
TALDO1	201463_s_at	10.70669	10.67514	10.69394	10.93805	10.98089	10.90701	11.7355	11.89182	11.83997	12.02023	11.85543	11.82944	10.10618
JUN	201464_x_at	8.118816	8.12624	8.505925	8.530135	9.541246	9.58465	6.223657	5.996416	5.292204	6.354688	6.540675	6.095685	8.295498
JUN	201465_s_at	7.233134	6.884595	7.394153	7.946454	10.51492	10.68874	5.744172	5.906822	4.589002	5.20327	6.830063	6.658224	6.179696
JUN	201466_s_at	7.940235	7.800593	8.220678	8.774419	9.850238	9.557498	5.767127	6.062788	5.494261	6.074616	6.29417	5.80584	7.895927
NQO1	201467_s_at	2.471522	2.448136	2.424488	2.430242	2.454533	2.417258	3.006471	2.486713	2.598105	2.687405	2.524764	2.453462	2.317078
NQO1	201468_s_at	2.909046	3.753203	3.990486	3.624748	3.436679	3.839282	6.715483	6.916854	5.605164	5.109452	7.243216	6.703789	4.882263
SHC1	201469_s_at	4.554408	4.065463	4.651319	4.308375	4.37168	4.340662	5.604326	4.616057	5.057858	5.424341	4.517876	4.526332	6.715204
GSTO1	201470_at	12.58403	12.59133	12.37372	12.55957	12.82616	12.81088	12.23826	12.43656	12.232	12.31097	12.08485	12.46553	9.22612
SQSTM1	201471_s_at	10.61305	10.62868	10.14592	10.07011	9.983785	9.863814	9.567264	8.700631	9.355489	9.525637	8.565339	9.057133	7.959688
VBP1	201472_at	8.497595	8.494276	8.662482	8.656486	8.465373	8.149214	10.85076	10.43412	11.00077	11.18362	10.224	10.43445	10.28322
JUNB	201473_at	8.611391	8.388377	8.199615	7.514933	9.132203	8.63927	6.204654	5.458008	6.177115	6.417318	5.103528	5.789275	4.002656
ITGA3	201474_s_at	2.530915	2.500625	2.47315	2.48112	2.528872	2.480446	2.484974	2.496157	2.485955	2.479301	2.561263	2.496464	2.7137
MARS	201475_x_at	6.335884	6.191916	6.735448	6.254668	5.943104	6.156605	8.067656	7.528952	8.156675	8.206311	7.651481	7.739565	7.792005
RRM1	201476_s_at	4.218921	4.23135	4.198191	4.206764	4.242536	4.345771	8.389747	4.69478	8.723925	8.866822	4.706055	5.340777	8.876058
RRM1	201477_s_at	9.264354	9.309913	8.11217	8.528502	8.904968	8.933403	11.40811	11.91567	11.51171	11.7068	11.52746	11.63612	10.03803
DKC1	201478_s_at	5.555367	6.099175	6.008974	5.746929	4.406731	3.088869	8.348682	7.259382	8.784285	8.516152	7.024107	7.192011	7.620719
DKC1	201479_at	7.619653	7.651122	7.235774	7.223953	7.359589	7.016377	9.678411	9.221546	9.654536	9.748672	8.8997	9.247812	8.503864
SUPT5H	201480_s_at	7.394008	7.334172	6.751829	7.197378	7.405558	7.674353	7.150046	7.734206	7.114728	7.320127	7.88075	7.514662	6.88108
PYGB	201481_s_at	4.096049	4.083485	3.891326	3.860033	4.080589	4.36331	4.268826	4.169742	4.101542	4.068778	4.721823	4.073952	5.62737
QSOX1	201482_at	5.870338	6.111005	6.604674	6.415299	7.073401	7.849137	4.414968	4.468612	4.312975	4.905189	5.680374	5.576499	6.045112
SUPT4H1	201483_s_at	6.061035	6.011484	6.65645	6.269914	6.218645	6.232357	7.806393	6.853845	6.732994	7.435179	7.053547	6.733189	7.714205
SUPT4H1	201484_at	4.946784	5.054179	5.087704	5.053011	4.930039	4.8582	6.768711	5.537563	6.818256	6.522207	5.687858	5.707533	5.597051

RCN2	201485_s_at	5.423423	4.919328	5.939455	5.777162	5.481657	5.127433	7.715974	6.746588	7.234705	7.539615	7.724494	7.468082	10.37194
RCN2	201486_at	8.672772	8.585489	8.59453	8.754563	8.338456	8.0463	10.06735	9.639426	10.03652	10.05666	9.903309	9.745339	11.78337
CTSC	201487_at	7.204636	6.91057	8.111559	8.010536	7.826824	7.158205	8.326083	7.57935	8.4436	8.249339	7.469011	7.565377	8.688652
KHDRBS1	201488_x_at	8.70578	8.475734	8.837731	8.818571	8.431867	8.06877	9.290027	8.58304	9.021495	9.020945	9.233924	8.914593	10.3683
PPIF	201489_at	11.09472	11.05138	10.3074	10.62043	10.0861	9.859433	9.375619	9.698776	9.192957	9.246878	9.661152	9.787679	7.980162
PPIF	201490_s_at	7.436216	7.364663	7.87345	7.325568	4.817827	3.969679	7.134979	4.476116	7.222828	6.939838	4.01285	4.22619	6.921225
AHSA1	201491_at	7.797339	7.559103	8.051392	8.040997	7.281959	7.17975	8.975231	8.025831	8.931208	9.246259	7.86585	8.23098	8.713256
RPL41	201492_s_at	14.70921	14.66434	14.60011	14.76834	14.79814	14.72684	14.87295	14.8706	14.89491	14.83736	14.89245	14.94292	15.00977
PUM2	201493_s_at	9.055293	9.024476	9.04785	8.994005	8.809364	8.398501	9.953465	8.697155	9.599064	9.831499	8.60601	8.527603	10.63561
PRCP	201494_at	9.347393	9.236922	9.664939	9.747921	8.836026	8.710177	9.284286	8.590476	9.130514	8.972925	8.795748	8.956035	9.081256
MYH11	201495_x_at	2.280403	2.253116	2.221132	2.237048	2.262832	2.228589	2.248394	2.252338	2.239255	2.23459	2.231982	2.248071	2.180829
MYH11	201496_x_at	2.440703	2.449515	2.411115	2.449691	2.462358	2.725489	2.412139	2.770471	2.427192	2.423071	2.402595	2.441379	2.395967
MYH11	201497_x_at	3.606032	4.139513	5.216312	5.363136	4.762829	5.046362	3.195668	3.28578	3.099934	2.974703	3.697737	3.444339	5.755726
USP7	201498_at	8.96795	9.010597	8.826571	8.93855	9.137282	9.242235	10.27482	10.55572	10.12839	10.24577	10.28841	10.39932	9.273246
USP7	201499_s_at	8.184673	8.068594	8.226269	8.199501	7.879632	7.888642	9.94574	9.544263	9.893467	10.1819	9.157174	9.641337	8.828002
PPP1R11	201500_s_at	7.550867	7.56838	7.806786	7.74735	7.784748	7.477096	7.590533	6.856154	7.634577	7.168331	6.764162	7.367966	7.385582
GRSF1	201501_s_at	8.252673	8.1828	7.961223	8.207492	8.102876	7.990337	7.935548	8.006734	7.625782	7.552167	8.219995	7.890095	8.67693
NFKBIA	201502_s_at	11.5901	11.57046	11.26081	11.55784	11.43594	11.57543	8.116093	8.31278	8.182034	7.966804	8.663122	8.682271	8.681936
G3BP1	201503_at	8.943215	8.902007	9.548395	9.433807	9.375817	9.101113	10.27186	10.08984	10.26241	10.23855	10.28396	10.2621	10.48569
TSN	201504_s_at	4.183247	4.215549	5.078513	4.500866	4.030097	4.233803	5.314034	3.90324	5.866443	5.284442	4.23487	4.246	6.973357
LAMB1	201505_at	8.690894	8.92024	8.662633	8.492801	9.046661	9.040969	9.923022	9.658683	9.613628	9.842828	10.95187	9.812907	12.0164
TGFBI	201506_at	10.85092	10.90634	11.0528	11.06829	9.526557	9.582148	11.04597	9.985647	10.61479	11.02786	9.864888	10.46202	11.84835
PFDN1	201507_at	6.811391	6.632739	6.924704	6.89248	6.908681	6.927967	7.815595	6.967053	7.775964	7.657653	7.286506	7.373879	8.626457
IGFBP4	201508_at	12.31337	12.39922	12.15201	12.18808	11.84695	11.79223	8.468984	7.961818	7.893731	7.793306	9.456868	8.579329	10.12796
IDH3B	201509_at	7.031774	6.963213	6.850669	7.137158	7.273635	7.504063	7.139016	7.508993	7.031615	7.305823	7.799402	7.629279	6.764525
ELF3	201510_at	5.906156	6.270593	5.77468	5.63242	6.225384	5.844473	3.21497	4.092645	2.4404	2.77559	3.19734	3.209733	3.163448
AAMP	201511_at	7.82095	7.753469	7.952592	8.008204	7.888349	7.70155	8.363438	7.930068	8.467123	8.327735	7.939679	7.956832	7.729684
TOMM70A	201512_s_at	8.781496	8.533805	8.169591	8.467227	8.077187	8.033492	8.223101	8.439608	8.40973	8.182769	8.408473	9.025604	8.362755
TSN	201513_at	6.787212	6.957908	6.803678	7.066238	7.400329	7.042465	6.497906	6.76884	6.784361	6.475616	6.619	6.402532	6.097822
G3BP1	201514_s_at	6.395533	6.309352	7.184003	7.070569	6.781176	6.649159	8.071969	6.854261	7.679431	7.600509	6.820124	7.35416	8.70889
TSN	201515_s_at	8.587725	8.36561	8.723122	8.682844	8.135647	7.648314	9.233315	8.927196	9.650857	9.21419	8.961608	8.904749	9.525768
SRM	201516_at	5.409605	5.452394	5.529149	5.882914	5.696191	5.328763	7.688952	7.85456	7.939238	7.750471	7.798329	7.971054	7.623469
NCBP2	201517_at	6.872133	7.033045	7.233122	6.92267	6.679533	6.851344	7.937015	7.226483	8.01477	7.887772	7.857103	7.564809	9.184674
CBX1	201518_at	7.037388	6.981602	7.196954	6.951287	6.976939	6.656962	8.059355	7.801653	7.870818	8.001487	8.281079	7.774384	11.01607
TOMM70A	201519_at	10.34437	10.359	10.18217	10.27983	10.07322	9.878836	9.910601	9.47906	9.968035	9.636854	9.63076	9.364241	9.470459
GRSF1	201520_s_at	8.124645	7.942692	8.168071	8.338035	7.74041	7.050537	8.755412	8.215594	8.464732	8.402617	7.769766	8.062735	8.594513
NCBP2	201521_s_at	4.944612	4.059831	5.029467	4.805529	5.163524	5.687917	6.549601	6.805125	6.041817	6.958483	6.884555	7.086059	7.211881
SNRPN	201522_x_at	8.333253	8.090639	8.636201	8.717699	8.700216	8.274174	10.0745	10.22654	9.960852	10.13332	10.22844	10.45774	10.62549

UBE2N	201523_x_at	4.414474	4.585653	5.694564	5.337132	3.759838	3.880398	8.014849	5.257516	8.056916	7.68016	5.336682	5.372978	9.250664
UBE2N	201524_x_at	8.26721	8.231248	8.269178	8.442636	8.095076	8.022602	9.720699	9.39874	9.740815	9.66992	9.220879	9.388475	9.993728
APOD	201525_at	2.268818	2.24607	3.491546	2.297185	2.251191	2.282859	2.227062	2.235173	2.219583	2.213646	2.235354	2.230011	8.484814
ARF5	201526_at	6.88297	6.721388	6.701411	6.70389	6.720614	6.542849	6.615709	6.690389	6.572361	6.615458	6.871964	6.711726	7.466885
ATP6V1F	201527_at	7.785805	7.726471	7.934268	7.684154	7.690643	7.595169	9.205661	8.351075	8.908039	9.313387	8.236388	8.20766	10.32449
RPA1	201528_at	8.732082	8.58605	8.461726	8.408424	8.69441	8.072049	10.86162	10.24547	10.76022	10.91275	10.41608	10.50397	10.07924
RPA1	201529_s_at	4.312615	4.624286	5.291508	4.640373	3.093707	2.932926	7.866054	5.431339	7.613742	7.901414	5.483674	5.447258	7.638568
EIF4A1	201530_x_at	10.51574	10.5299	10.46421	10.39401	10.15946	9.636087	12.64534	11.88472	12.40243	12.45093	12.14027	12.09269	12.46855
ZFP36	201531_at	8.446854	8.613967	9.748639	9.174879	9.186536	8.650724	5.514659	4.263371	5.369608	5.707628	4.067041	5.091281	5.07549
PSMA3	201532_at	9.455076	9.37124	9.900787	9.631267	9.00383	8.202132	10.08698	8.953147	9.94939	9.972757	8.828927	8.943484	9.980006
CTNNB1	201533_at	10.11231	10.20102	10.08858	9.922046	10.41357	10.01125	9.954551	9.738223	9.970409	9.716219	9.888548	9.814878	11.02399
UBL3	201534_s_at	8.574384	8.702463	9.070067	9.211532	8.294473	7.354759	7.978199	7.054764	7.573334	7.81165	7.609398	7.020562	9.128399
UBL3	201535_at	8.207566	7.940733	8.976643	8.733513	8.238563	7.761354	7.150884	6.704988	7.14687	7.258181	6.678315	6.479415	8.151138
DUSP3	201536_at	10.35029	10.38398	10.19071	10.20269	9.901241	9.762307	8.740512	8.689196	8.736095	8.679915	8.45481	8.169208	7.711356
DUSP3	201537_s_at	4.388	4.454777	5.069228	4.734378	4.26505	4.292298	4.412106	3.824414	4.44414	4.429471	3.5808	4.455182	4.435138
DUSP3	201538_s_at	3.191954	2.632698	3.327563	4.070028	2.81238	2.9926	2.614289	2.59966	2.617652	2.588731	2.589149	2.593232	2.920667
FHL1	201539_s_at	2.917605	2.795117	3.614577	2.895357	2.904658	2.898898	2.908027	2.807176	2.86607	2.848478	3.031215	2.882598	8.318017
FHL1	201540_at	8.800094	8.726145	10.36169	10.13742	9.28214	9.057222	8.191917	7.775813	8.36139	8.0886	10.76696	8.338128	12.3667
ZNHIT1	201541_s_at	10.15851	10.16481	10.43735	10.49561	10.40384	10.30415	10.05599	10.06685	9.967426	9.949207	10.01397	10.03627	8.881367
SAR1A	201542_at	8.996829	9.022945	9.15927	9.264621	9.212421	9.137307	9.543366	9.553223	9.048617	9.33123	9.575736	9.705152	9.876202
SAR1A	201543_s_at	7.410955	7.222336	7.480904	7.616005	7.057696	7.041101	7.829413	7.855231	8.079456	8.052386	7.987679	7.941304	6.735831
PABPN1	201544_x_at	8.12354	8.272599	8.151202	7.716568	7.763396	7.503999	9.323384	8.488612	9.297354	9.31266	8.579177	8.783938	9.360909
PABPN1	201545_s_at	6.474462	6.213949	6.462334	5.816046	6.035348	5.822883	7.380673	6.280293	7.06069	6.710586	6.743464	6.217621	6.927081
TRIP12	201546_at	8.613547	8.610681	8.561262	8.689676	8.460633	8.176919	9.743045	9.379508	9.683793	9.679544	9.302215	9.359442	9.51178
JARID1B	201547_at	2.328302	2.304987	2.268703	2.2873	2.319962	2.290259	2.297086	2.307645	2.29301	2.278133	2.292232	2.30339	2.18346
JARID1B	201548_s_at	4.935153	4.776453	4.400137	4.337184	4.72244	5.150357	6.342717	6.05791	5.729305	5.712705	6.682148	6.543515	8.818864
JARID1B	201549_x_at	6.108688	6.016114	5.949703	5.342558	5.186456	4.482278	7.470906	6.094149	7.449166	7.235886	6.742414	6.485469	9.145465
ACTG1	201550_x_at	12.76582	12.75071	12.11249	11.92804	12.46712	12.39651	12.82428	12.63299	12.5145	12.66338	13.0893	12.81662	13.82398
LAMP1	201551_s_at	4.928783	5.055946	5.993018	5.482689	2.189785	2.4913	6.033054	2.488704	5.280624	5.860704	2.276005	2.909546	6.99809
LAMP1	201552_at	10.11832	10.1219	10.10034	10.06477	9.447303	9.247726	9.504967	8.693355	9.329069	9.448125	8.620962	8.729206	9.507128
LAMP1	201553_s_at	13.52916	13.45349	13.05133	13.27411	13.35028	13.49685	12.18807	12.63254	12.08459	12.13193	12.67001	12.60442	11.96606
GYG1	201554_x_at	5.114988	4.144601	5.50341	5.537704	4.745001	4.314623	6.599722	5.385311	6.807627	6.480187	6.400671	5.749783	8.47687
MCM3	201555_at	6.703692	6.712252	6.005744	6.009337	6.37304	6.012052	10.5099	9.679539	10.23403	10.44126	9.491296	9.990361	9.622251
VAMP2	201556_s_at	5.648198	5.501746	5.764502	5.659093	5.262039	4.745165	5.647544	5.176878	5.481196	6.080585	5.638151	5.600439	6.482597
VAMP2	201557_at	6.484733	6.316762	6.24712	6.319273	6.740634	6.266239	5.426073	6.07036	5.485917	5.713539	6.216773	6.110641	6.149265
RAE1	201558_at	8.387039	8.338895	7.651433	8.137052	8.489344	8.608854	8.557747	9.145622	8.754855	8.839837	9.538445	9.329744	8.583146
CLIC4	201559_s_at	2.83168	2.820406	2.836638	2.821352	2.836999	2.80365	3.405916	2.832931	3.517439	3.109085	2.482497	2.778233	7.797706
CLIC4	201560_at	9.160275	8.985681	9.448478	9.287631	8.682943	8.187247	8.962689	7.687476	8.715068	8.742529	8.088072	7.88627	10.59665

CLSTN1	201561_s_at	6.530601	6.693881	6.419152	6.609982	6.844981	6.864152	6.304111	6.171715	6.248004	6.315454	7.419666	6.819801	9.261344
SORD	201562_s_at	9.316618	9.200787	10.39684	10.30474	9.91785	9.790389	7.750625	7.601007	7.877888	7.927737	7.165062	7.466685	4.49904
SORD	201563_at	12.92229	12.92088	13.26823	13.32666	12.79255	12.59239	10.97785	10.94322	11.10256	10.95979	10.05225	10.53275	7.271652
FSCN1	201564_s_at	5.039055	5.022084	5.354567	5.0036	5.019205	4.835267	7.059854	5.553341	6.452962	6.367812	6.439922	6.334061	8.798034
ID2	201565_s_at	12.02208	12.03988	12.22717	12.1319	11.76575	11.70848	10.27822	9.393787	10.30101	9.863803	8.3365	9.172765	10.89591
ID2	201566_x_at	10.4639	10.49874	11.17476	11.13331	10.36025	10.29124	8.691099	7.745875	8.644174	8.208999	6.905674	7.88909	8.859438
GOLGA4	201567_s_at	9.800169	9.711087	10.30833	10.26713	9.410687	8.927618	10.2138	9.332386	10.12668	10.08593	9.198675	9.443271	9.504599
UQCRQ	201568_at	12.20689	12.28514	12.12116	12.31805	12.14844	12.12992	11.40438	11.58884	11.46111	11.4906	11.54418	11.63204	10.79355
SAMM50	201569_s_at	7.991036	8.026698	7.731144	8.017423	8.047207	8.181358	8.660532	9.462905	9.099354	9.107415	9.060676	9.512313	7.547549
SAMM50	201570_at	8.144794	8.386443	8.443345	8.486562	8.056864	8.142704	9.40804	9.324745	9.489488	9.478721	9.109049	9.582986	8.422408
DCTD	201571_s_at	6.420601	6.57178	8.033742	7.937633	6.310315	6.165152	6.963248	5.927726	6.800788	6.891224	6.464151	6.338767	7.588237
DCTD	201572_x_at	6.884466	6.966887	7.236672	7.292038	6.545756	6.619799	6.95902	6.333865	6.776771	6.781118	6.312519	6.566619	7.504347
ETF1	201573_s_at	7.824829	7.909927	7.965073	8.191438	7.642901	7.856334	8.371973	8.722794	8.391653	8.858214	8.443979	8.451142	6.924554
ETF1	201574_at	8.1629	8.165846	8.819302	8.457088	7.924212	7.585129	9.314369	8.814936	9.417411	9.609147	8.815884	8.985784	8.890615
SNW1	201575_at	10.10641	10.13179	9.748764	10.04921	10.29267	10.33254	11.10196	11.59865	11.26241	11.12023	11.39514	11.5851	10.36296
GLB1	201576_s_at	8.553508	8.377339	8.809979	8.66043	7.824162	7.793667	8.980839	8.257379	9.013608	8.848181	8.035687	8.452899	8.207673
NME1	201577_at	8.057091	8.072811	7.856349	8.209719	7.849173	7.729735	9.75463	9.81969	9.993874	9.928349	10.36299	10.13501	10.26156
PODXL	201578_at	4.793117	5.086448	5.069246	4.782521	5.71883	5.073377	4.944632	4.354042	4.524931	5.071428	6.318586	5.078078	8.611625
FAT	201579_at	8.309638	8.319447	6.303357	6.157578	6.348648	6.192741	9.110176	8.210306	8.860418	8.619331	9.077119	8.280773	10.91963
TXNDC13	201580_s_at	7.461969	7.394134	7.747887	7.510724	5.741057	5.590139	6.731419	6.212686	7.026462	6.916623	5.71611	6.060069	7.029989
TXNDC13	201581_at	9.420209	9.374329	10.17937	10.04224	9.618421	9.346745	9.595299	9.281109	9.780664	9.793435	9.154363	9.195263	10.11565
SEC23B	201582_at	5.518836	5.284355	6.275363	6.240154	6.06732	6.081727	5.891909	5.477535	6.571005	6.185112	5.738029	5.94157	4.509227
SEC23B	201583_s_at	5.51243	4.978928	7.046254	6.841615	5.707762	5.564829	7.268671	6.000055	7.226253	7.359571	5.914269	5.825219	7.069685
DDX39	201584_s_at	6.78657	6.935632	6.259787	6.372279	6.288522	6.619097	9.203608	8.666941	9.463284	9.283575	8.48777	8.68443	8.135088
SFPQ	201585_s_at	6.182651	6.03212	6.752627	6.01707	5.678671	6.335138	8.99111	7.540818	8.827143	9.135682	7.255035	7.557214	9.49819
SFPQ	201586_s_at	9.815446	9.703108	9.741948	9.650718	9.767009	9.483043	11.71506	11.26636	11.80311	11.677	11.29566	11.21411	11.73612
IRAK1	201587_s_at	7.786986	7.808212	7.662252	7.743143	8.072366	8.293759	7.890723	8.481415	8.090225	8.103549	8.684675	8.758255	7.582401
TXNL1	201588_at	11.1602	11.1358	10.75124	11.07044	10.67824	10.65175	12.23111	11.76051	12.29826	12.3533	11.69517	11.88287	11.48897
SMC1A	201589_at	8.744261	8.571115	8.922366	8.927488	9.06184	8.347116	10.53054	9.96633	10.497	10.35571	10.13981	9.862266	9.673489
ANXA2	201590_x_at	10.45122	10.44176	9.550731	9.365614	9.806778	9.762889	10.72327	10.04269	10.03737	10.18887	10.82238	9.967925	12.95336
NISCH	201591_s_at	9.627358	9.652634	8.675023	8.90173	9.426859	9.670889	8.973055	9.151119	8.750149	8.754856	9.963817	9.43582	9.870494
EIF3H	201592_at	9.966088	10.15351	9.973144	10.18728	10.11766	9.902795	10.91953	10.69034	10.77458	10.62514	10.74348	10.79012	11.42891
ZC3H15	201593_s_at	9.755202	9.650267	9.961732	9.944447	9.853437	9.665199	9.822242	9.862612	10.10728	9.990177	10.1677	10.12835	10.14157
PPP4R1	201594_s_at	8.810082	8.784154	8.057382	7.89933	7.978771	8.075585	9.478878	9.406162	9.273177	9.217904	9.698547	9.587144	10.19815
ZC3H15	201595_s_at	9.752602	9.543532	10.1523	10.22774	9.325353	8.54526	10.38078	9.62587	10.4441	10.34297	9.446381	9.430995	10.28039
KRT18	201596_x_at	10.2253	10.24544	10.51813	10.55386	10.42825	10.2922	10.3972	10.2917	10.1917	10.45826	10.44863	10.70109	7.844705
COX7A2	201597_at	12.33325	12.27237	12.12956	12.34577	12.36318	12.25805	12.03797	12.54629	12.30106	12.17332	12.46168	12.55461	12.37367
INPPL1	201598_s_at	2.561614	2.530932	2.476343	2.505692	2.568158	2.505648	2.415151	2.523204	2.508167	2.43915	3.546696	2.52068	3.697504

OAT	201599_at	11.15307	10.65988	12.15565	12.11989	9.919914	9.052735	13.38855	12.38579	13.43378	13.32241	11.92767	12.12992	11.55255
PHB2	201600_at	11.58039	11.57785	11.11333	11.28592	11.03548	10.90506	11.71469	11.44362	11.77902	11.6632	11.45392	11.51477	11.40074
IFITM1	201601_x_at	8.664185	8.551208	8.886344	8.831912	8.158946	8.132159	4.152084	4.029672	4.40854	4.811453	4.301228	4.015382	6.258771
PPP1R12A	201602_s_at	7.066283	7.244931	7.375808	7.340914	7.502515	7.464682	7.913467	8.1769	8.268334	8.157716	8.596924	8.37871	8.52918
PPP1R12A	201603_at	8.227114	8.14479	8.208427	8.134292	8.106779	7.719205	9.417852	9.2451	9.844881	9.965861	9.327971	9.219864	9.977501
PPP1R12A	201604_s_at	4.439745	4.644389	4.818913	4.641149	4.238667	4.268456	6.401803	5.011521	6.87878	6.796943	4.793786	4.683358	7.673718
CNN2	201605_x_at	6.313932	6.497998	6.765496	6.77533	6.705515	6.760618	6.808822	6.807348	6.798869	6.796371	6.792171	6.799376	8.389247
PWP1	201606_s_at	9.151132	9.024279	9.366704	9.323278	9.345554	8.970498	9.870077	9.518405	9.773346	9.63873	9.881171	9.930309	10.04792
PWP1	201607_at	5.382632	5.253579	6.287754	6.168523	5.624977	5.831448	5.857349	5.631033	5.802724	5.895373	5.494105	5.979934	6.222924
PWP1	201608_s_at	7.645588	7.733737	7.442545	7.628815	7.4884	7.363049	8.02644	8.008353	8.143082	8.139639	8.058802	8.246229	8.046411
ICMT	201609_x_at	6.282901	6.199335	6.565785	6.603796	5.539845	5.810873	7.635469	7.47816	7.419739	7.350655	7.356848	7.656609	7.875555
ICMT	201610_at	2.802739	2.752275	2.687511	2.73491	2.791835	2.706549	2.742986	2.762188	2.720078	2.689439	2.73956	2.78864	2.424385
ICMT	201611_s_at	5.611203	5.520548	6.058022	5.856897	4.028381	3.881586	6.488332	6.083668	6.527411	6.231647	5.752594	5.873953	6.300648
ALDH9A1	201612_at	11.25491	11.27161	11.50551	11.64804	11.42118	11.23784	10.08387	10.42139	10.21981	10.04128	10.24143	10.50131	8.909852
AP1G2	201613_s_at	6.113399	6.129496	5.622171	6.038888	6.102596	6.520782	6.10289	6.636301	6.408179	6.090457	6.424724	6.302327	6.265562
RUVBL1	201614_s_at	5.590354	5.638338	6.312149	6.218716	5.846881	5.683393	8.839702	7.64535	8.693844	8.64762	7.732938	7.881804	8.089522
CALD1	201615_x_at	7.389143	6.886445	7.239152	7.742766	8.274286	8.359429	5.350642	5.857284	5.384073	5.338067	6.586123	6.308541	6.398456
CALD1	201616_s_at	7.834615	7.813132	8.087997	7.851371	7.561391	7.11961	7.114851	5.710456	6.179249	5.83967	6.244623	6.398417	8.668682
CALD1	201617_x_at	7.119812	6.871859	8.666533	7.755738	6.960061	6.662515	7.31018	4.561832	7.21131	7.391047	5.314355	5.219872	9.865001
GPAA1	201618_x_at	8.257777	8.193422	7.920355	8.143833	7.905783	7.698514	8.257628	8.785271	8.461147	8.39571	8.650706	8.797704	7.356948
PRDX3	201619_at	10.9947	10.90776	11.67592	11.54646	10.45355	9.848748	11.74858	10.80148	11.69305	11.95312	10.34736	10.49924	10.54234
MBTPS1	201620_at	7.346743	7.111854	7.575924	7.497747	7.05391	6.306016	7.057605	6.307034	7.148538	7.217348	6.554945	6.146161	8.188638
NBL1	201621_at	3.064905	3.255819	3.44013	3.274005	3.440072	4.634311	3.13753	3.243869	3.275343	3.218028	3.837846	3.279317	5.945777
SND1	201622_at	8.160033	8.119542	7.920071	8.006321	8.219548	8.202056	8.531877	8.579352	8.638477	8.605414	8.993083	8.759702	8.404987
DARS	201623_s_at	8.940573	8.997027	8.574185	8.656743	8.691351	8.676658	9.290156	9.048611	9.309662	9.252436	8.950856	8.938087	8.584967
DARS	201624_at	8.382318	7.999248	8.615374	8.399742	7.559403	6.710188	9.270899	8.232278	9.346594	9.625703	7.640583	7.885826	8.863822
INSIG1	201625_s_at	9.628512	9.555972	9.463848	9.487501	9.297269	8.577116	6.560209	5.834773	6.51707	6.3816	5.335403	5.968911	4.046816
INSIG1	201626_at	12.65312	12.77908	12.79031	12.93305	11.3004	11.0593	10.29939	9.57288	10.47818	10.02803	9.385791	10.14536	8.740282
INSIG1	201627_s_at	10.75641	10.76337	11.81291	11.00041	8.732172	8.453295	9.031425	6.195055	8.634494	8.321284	6.43963	6.892654	7.136825
RRAGA	201628_s_at	8.096341	8.072704	8.6732	8.461611	7.703125	7.355731	8.871308	7.823742	8.790021	8.735755	8.014868	8.155668	9.640409
ACP1	201629_s_at	5.173628	5.36708	6.408216	5.866785	4.343693	4.514656	8.526553	7.156604	9.077281	9.317389	6.604127	7.135073	7.962034
ACP1	201630_s_at	9.0015	8.611959	9.523628	9.528909	8.860497	8.381518	10.5677	9.381374	10.56056	10.68094	9.383121	9.458622	10.43763
IER3	201631_s_at	7.467407	7.484689	6.755893	6.511189	8.326938	8.673211	3.868352	4.973534	4.309023	4.745136	5.136552	5.513588	5.766142
EIF2B1	201632_at	7.432024	7.345584	7.587499	7.483858	7.124985	7.089623	7.97553	7.466493	8.206157	8.190736	7.396645	7.479624	8.130937
CYB5B	201633_s_at	5.024164	5.276808	6.024447	5.486114	4.890004	4.536296	6.296148	5.284418	6.956942	7.055674	5.303068	5.454644	7.706392
CYB5B	201634_s_at	7.144542	7.380524	7.135344	7.315371	7.097229	6.623249	8.136022	8.205861	8.395542	8.597306	8.038721	8.423079	8.233423
FXR1	201635_s_at	8.032072	7.483323	7.802265	8.176308	8.785173	9.125148	7.966519	8.687006	8.925551	8.848937	9.257669	9.110459	10.23964
FXR1	201636_at	7.343072	7.694478	7.197654	7.343466	7.477829	6.855782	7.738267	7.360442	7.752507	7.718244	8.010074	7.342805	9.302366

FXR1	201637_s_at	9.191985	8.99575	9.176905	9.233997	9.295236	9.030164	9.655682	9.470161	9.629308	9.541768	9.668049	9.418837	10.86801
CPSF1	201638_s_at	2.080887	2.04243	2.024629	2.028174	2.132264	2.030878	2.015076	2.04068	2.02952	2.017029	2.022614	2.034109	1.837364
CPSF1	201639_s_at	7.50717	7.615565	7.565411	7.722048	7.686646	7.737934	8.434318	8.500019	8.134638	8.049932	8.46263	8.780318	7.973713
CLPTM1	201640_x_at	7.630834	7.542262	7.428444	7.517984	7.350804	7.465522	7.062984	7.20371	6.95089	6.972869	7.295417	7.529765	5.382084
BST2	201641_at	8.791244	8.839063	8.889329	9.051115	9.285448	9.632556	3.869902	4.994526	4.065557	4.237597	4.727322	4.729108	3.42355
IFNGR2	201642_at	7.704485	7.523349	7.032019	6.871901	6.903341	7.230627	7.491761	6.909322	7.382147	7.33244	7.28063	7.093509	8.685067
JMJD1B	201643_x_at	6.673412	6.666726	6.646644	6.208732	6.416423	6.434928	8.302403	6.816864	7.101693	6.706213	6.702563	6.740752	8.121682
TSTA3	201644_at	7.311054	7.306212	7.268536	7.290267	6.900219	7.246137	9.071681	8.900693	8.984683	9.316036	8.889736	9.463118	7.018742
TNC	201645_at	4.615904	4.566628	3.597749	3.328343	4.161369	4.355122	3.414951	3.497482	2.655494	3.396974	7.639604	3.425532	10.84348
SCARB2	201646_at	6.41826	6.044948	7.138189	6.980722	6.713812	5.764651	5.928589	5.026479	5.797122	6.164064	5.000633	5.227919	8.219461
SCARB2	201647_s_at	4.346203	4.05026	5.666359	4.998059	3.721152	4.375112	3.196405	3.251807	2.957341	3.241649	3.312247	3.425904	7.144865
JAK1	201648_at	10.02757	9.902739	10.32375	10.33674	9.429934	8.827729	8.795523	7.776956	8.753381	8.457466	8.06337	8.174229	9.452707
UBE2L6	201649_at	8.593973	8.58531	8.943812	8.935111	8.810207	8.550112	8.198525	8.111973	8.096393	8.078251	7.767248	8.100256	7.807062
KRT19	201650_at	2.563551	2.502001	2.464529	2.493319	2.569653	2.522658	6.784007	6.214863	6.212099	5.918474	7.08468	7.32187	8.062967
PACSIN2	201651_s_at	9.337896	9.232672	9.040418	8.809334	9.235321	9.19127	8.88951	9.359602	8.69177	8.75402	9.301345	9.192553	9.039913
COPS5	201652_at	8.167798	8.134674	8.620786	8.513404	7.813869	7.451792	9.012448	8.294846	9.239452	9.305246	8.238079	8.427723	8.696992
CNIH	201653_at	11.38664	11.26289	11.25953	11.15207	10.38362	9.797651	10.90049	9.992873	10.86799	10.95642	10.14569	10.37708	10.55119
HSPG2	201654_s_at	2.254994	2.242102	2.21945	2.223834	2.238478	2.239994	2.230791	2.240481	2.22222	2.220564	2.220315	2.228586	4.970239
HSPG2	201655_s_at	8.417831	8.172894	6.932485	7.498146	8.446135	8.850122	6.29199	7.135899	6.341049	6.401882	7.757666	7.37175	8.735846
ITGA6	201656_at	5.395624	5.295464	4.950031	5.117432	5.363416	5.130077	6.705377	6.539675	6.656923	6.309082	6.510611	6.476025	6.995938
ARL1	201657_at	5.798578	6.150038	6.817449	6.594883	5.901728	5.871829	6.726138	6.268245	7.026674	6.462951	6.348706	6.24569	7.795111
ARL1	201658_at	8.329557	8.193557	8.585769	8.544361	8.645446	8.63926	8.908469	9.074131	8.916252	8.777896	9.251013	9.149649	9.220804
ARL1	201659_s_at	7.041454	7.126667	7.67786	7.553827	6.77784	6.179847	7.79123	6.782793	7.902916	7.795256	7.138653	6.947658	8.333131
ACSL3	201660_at	9.336232	9.236886	8.492869	8.417005	8.781523	8.519314	9.723111	9.622574	10.07204	9.697303	9.525	9.697982	8.825542
ACSL3	201661_s_at	9.066362	9.008246	8.847597	8.645205	8.699694	8.239814	9.369283	9.766021	9.722677	9.514067	9.558508	9.739389	8.573002
ACSL3	201662_s_at	8.693084	8.86206	8.35919	8.269439	7.735951	7.181806	9.59747	9.287579	9.803581	10.1532	8.822085	9.256996	7.945148
SMC4	201663_s_at	2.450946	2.939367	3.291364	2.975068	2.01836	2.228308	9.0994	8.211645	9.330009	9.406665	7.566713	8.067465	7.626868
SMC4	201664_at	7.766321	7.63415	7.436708	7.314631	6.872436	6.261009	11.44046	10.62401	11.83843	11.49861	10.30194	10.52945	10.36492
RPS17	201665_x_at	13.296	13.23535	13.19559	13.3778	13.49074	13.52246	13.93684	13.93156	13.91393	13.85567	13.98403	13.96724	14.08268
TIMP1	201666_at	9.278011	9.315833	10.235	10.42315	10.34129	10.40611	7.648311	7.232371	7.517134	7.36821	8.700361	8.107383	10.5063
GJA1	201667_at	6.087113	6.370546	7.873278	7.388742	7.181188	7.146581	5.949063	5.748124	6.302554	6.264446	10.04668	7.146256	13.08857
MARCKS	201668_x_at	2.360514	2.295892	2.337966	2.336802	2.349143	2.33997	3.75098	2.816553	2.733336	2.935137	3.865087	3.373645	7.895
MARCKS	201669_s_at	9.240986	9.279396	9.390435	9.184963	8.704468	8.536933	11.44024	10.37122	11.13093	11.21246	11.43093	10.54427	13.48228
MARCKS	201670_s_at	7.537198	7.357719	7.472624	7.013717	7.583808	6.891705	9.575741	8.210405	8.998872	8.931787	9.384979	8.90062	11.73323
USP14	201671_x_at	8.099291	8.100111	7.923926	8.02273	7.714038	7.340176	8.562155	8.650964	8.653757	8.742086	8.466162	8.502709	7.9457
USP14	201672_s_at	8.219549	8.061909	8.394845	8.26645	7.76092	7.291299	10.70115	9.949339	10.70628	10.66112	9.714037	10.03162	9.442073
GYS1	201673_s_at	4.272769	3.921631	3.499652	3.61886	4.012263	3.641514	3.653489	3.636486	4.070234	3.913129	4.820805	3.686527	5.791207
AKAP1	201674_s_at	9.556383	9.514396	9.087144	9.159905	9.702279	9.520702	8.567201	8.500986	8.590075	8.36737	8.545162	8.607656	6.747415

AKAP1	201675_at	10.90339	10.87849	10.16914	10.29572	10.80551	10.44248	9.405578	9.738341	9.608151	9.396994	9.609608	9.55354	7.551021
PSMA1	201676_x_at	9.47376	9.383228	9.922557	9.922719	9.446686	9.041006	10.62081	9.915062	10.61417	10.7145	9.860898	9.895643	10.36175
C3orf37	201677_at	6.722294	6.645313	6.24737	6.325795	6.627542	6.33395	5.806925	6.260942	6.113573	5.736382	5.581003	5.995415	4.705548
C3orf37	201678_s_at	5.695438	5.483734	5.6421	5.767352	5.332066	5.255277	5.881931	6.175049	5.849509	5.78397	5.965975	6.359025	5.547393
ARS2	201679_at	2.99217	2.815048	2.875734	2.931486	2.962768	2.948034	3.454092	2.974697	3.759534	4.219001	2.943132	2.965125	5.112201
ARS2	201680_x_at	8.721212	8.644621	8.232892	8.284784	8.707063	8.907703	9.580336	9.64737	9.450454	9.616965	9.73966	9.923338	8.690289
DLG5	201681_s_at	7.261163	7.228066	6.54098	6.93305	7.158214	7.236779	7.432262	7.658928	7.367392	7.67074	8.691092	7.933843	10.02143
PMPCB	201682_at	10.12481	10.18449	10.06622	10.09955	9.892735	9.90971	10.333	10.1108	10.43002	10.12996	9.752137	10.08002	9.711246
TOX4	201683_x_at	6.074363	5.933074	6.638686	6.669982	6.400234	6.12303	7.028669	6.857878	6.274128	6.507537	6.862615	7.133621	6.167273
TOX4	201684_s_at	7.00458	6.976655	7.08937	6.85591	6.749277	6.956763	7.619801	7.560456	7.725667	7.798627	7.479814	7.666301	7.213426
TOX4	201685_s_at	7.527834	7.81083	7.237662	7.377875	7.485121	7.389891	7.628531	7.367273	7.622969	7.699319	7.394959	7.560045	7.103233
API5	201686_x_at	2.574533	2.721579	3.043197	3.207295	2.52421	3.153494	4.272727	2.46631	3.937512	3.398053	2.599479	2.461002	3.719067
API5	201687_s_at	9.402061	9.43181	9.931035	9.673422	9.201447	8.778548	9.695513	9.388492	9.736564	9.924986	9.03271	9.41869	9.403983
TPD52	201688_s_at	3.641691	3.513531	5.634262	4.241837	3.520844	3.11685	4.568178	3.512469	4.16353	4.836212	2.670275	2.733298	5.575096
TPD52	201689_s_at	7.197646	7.019922	8.418937	7.58261	7.004845	6.660094	6.966804	5.971892	6.877692	7.135723	5.523365	5.863041	6.797537
TPD52	201690_s_at	8.265126	8.47477	8.945125	8.814111	8.61269	8.33566	7.350649	7.27845	7.840764	7.693114	6.964238	7.012772	7.191187
TPD52	201691_s_at	4.085027	4.023756	4.607021	4.991295	5.426995	5.001995	3.740558	3.760529	4.020376	3.950548	3.829701	3.962415	3.573461
OPRS1	201692_at	8.537909	8.498283	8.885575	8.711045	8.666322	8.381285	8.544562	8.341619	8.746035	8.692997	7.974309	8.55285	6.568717
EGR1	201693_s_at	8.83444	8.825337	4.71778	4.141085	8.987297	8.410423	2.382102	3.036119	2.605143	3.24137	3.326779	3.102223	3.291285
EGR1	201694_s_at	11.0088	11.29658	8.704881	8.782301	11.74342	11.41928	4.995803	5.760684	5.386057	5.79455	5.967471	5.675857	6.461545
NP	201695_s_at	6.671058	6.831771	7.673393	7.356523	6.639319	5.668614	9.09904	8.327514	9.288548	9.562777	7.275716	8.089756	3.915839
SFRS4	201696_at	8.879118	8.980818	9.111302	9.129333	8.869502	8.598926	9.572947	8.99228	9.724268	9.587788	8.848823	8.954382	8.868856
DNMT1	201697_s_at	6.881571	6.753047	5.947983	6.057895	6.399485	6.446028	10.34388	9.434072	9.976537	10.06775	9.296612	9.597757	9.594888
SFRS9	201698_s_at	8.202841	7.814468	8.699138	8.550172	7.537663	7.05436	10.05	8.692385	9.82721	9.901335	8.821707	9.005229	9.941168
PSMC6	201699_at	9.952063	9.882146	10.0521	9.719804	9.762371	9.53214	11.01863	10.58182	10.95633	11.16397	10.28972	10.39189	10.94422
CCND3	201700_at	6.747632	6.536072	6.445494	6.33059	6.864757	7.102924	9.36812	9.57134	9.316505	9.340524	9.104909	9.400458	8.053339
PGRMC2	201701_s_at	10.22962	10.1274	10.35655	10.31283	9.77646	9.454315	10.12927	10.34796	10.14004	10.10708	9.853451	10.19378	8.82928
PPP1R10	201702_s_at	5.498681	4.916374	5.54663	5.77627	6.403548	5.859535	3.358879	4.164093	4.517134	4.438136	5.034923	4.331381	4.415015
PPP1R10	201703_s_at	8.132209	7.996439	7.565251	7.447754	7.134399	7.276379	6.578441	5.589588	6.594409	6.613622	6.191702	6.545542	6.211936
ENTPD6	201704_at	7.24215	7.00314	6.930314	6.98591	7.104051	7.095932	6.763807	7.300446	6.667103	7.179928	7.556978	7.231392	6.431785
PSMD7	201705_at	8.807282	8.711529	8.628772	8.689827	8.443641	8.466184	9.383776	9.331394	9.400528	9.639429	9.287142	9.314116	9.058175
PEX19	201706_s_at	7.437176	7.445524	7.925739	8.036734	8.015448	7.205549	5.89008	5.991358	6.602029	5.824374	5.77672	5.775505	6.077013
PEX19	201707_at	7.152708	7.030066	7.479556	7.395873	7.196739	7.155235	5.907364	5.361552	6.096838	6.090507	5.695149	5.722109	6.049663
NIPSNAP1	201708_s_at	7.788177	7.632146	8.659681	8.31049	7.99588	7.797311	9.19294	7.965137	8.586005	8.683808	7.771586	8.672424	7.296369
NIPSNAP1	201709_s_at	10.56838	10.57861	10.4231	10.4756	10.67496	10.52828	10.79015	10.9214	10.55118	10.69039	10.78746	10.92566	8.410754
MYBL2	201710_at	3.941425	4.180862	3.783591	3.841763	4.071345	4.010067	9.006242	9.502012	9.440838	9.538905	8.943963	9.252661	6.155339
RANBP2	201711_x_at	6.520818	5.847819	7.570529	7.564491	7.206637	6.751389	7.185709	6.740054	7.446865	6.843584	6.799586	7.26005	8.295643
RANBP2	201712_s_at	5.96735	5.839645	6.743541	6.607966	5.841949	5.713884	7.164285	6.720595	7.292728	7.415003	6.453158	6.82343	7.772988

RANBP2	201713_s_at	9.353419	9.302401	9.824985	9.856956	9.358611	9.102731	10.38604	10.13166	10.38599	10.59996	9.859075	10.05282	10.12329
TUBG1	201714_at	6.787732	6.256142	6.345568	6.476971	5.809151	5.518487	10.46434	9.750537	10.12118	10.72828	9.587257	10.05992	8.110774
ACIN1	201715_s_at	7.042838	7.360664	6.905516	6.808842	6.643054	6.455446	8.150461	7.642313	8.062474	8.178521	7.378805	7.692478	7.840791
SNX1	201716_at	7.859449	7.733776	8.048294	8.006069	7.153079	6.923931	7.794037	7.193809	7.694252	7.698979	6.95631	7.230283	7.130845
MRPL49	201717_at	7.846069	7.893314	7.637415	7.838161	7.859229	8.049824	8.163787	8.22753	8.179671	8.177871	8.079941	8.287103	7.824534
EPB41L2	201718_s_at	3.510478	3.505671	3.487827	3.483302	3.495061	3.986364	4.238668	3.465465	4.077955	4.202654	3.341824	3.487471	7.59107
EPB41L2	201719_s_at	6.74698	6.631032	6.570558	6.567275	7.190464	6.897498	7.765915	7.23331	7.604505	7.781146	7.803464	7.329391	9.220476
LAPTM5	201720_s_at	5.468718	5.630428	6.430212	6.001973	6.291338	6.572824	6.878435	5.82729	6.381774	6.249373	5.066403	6.592015	5.221352
LAPTM5	201721_s_at	8.272301	8.441587	8.84989	8.856248	9.176984	9.474942	8.766267	8.714869	8.650041	8.344393	8.43497	9.139936	7.278154
GALNT1	201722_s_at	8.168296	7.932316	8.447356	8.386475	8.051217	7.996348	8.093937	7.858774	7.991192	8.386421	7.787856	7.983295	8.895014
GALNT1	201723_s_at	7.709219	7.44559	7.445275	7.772232	8.04536	8.466226	6.672633	7.303839	6.757197	6.990262	7.716184	7.623862	8.071804
GALNT1	201724_s_at	7.538538	7.490839	7.529425	7.664517	7.418107	7.573814	7.337486	7.408535	7.204515	7.221315	7.26874	7.536426	7.690932
CDC123	201725_at	7.635172	7.43914	8.16958	7.972056	7.496633	7.029403	9.027106	8.199406	8.780122	9.021958	8.279517	8.365935	9.165434
ELAVL1	201726_at	8.671521	8.632806	8.814664	8.391737	7.670884	7.239764	10.38088	8.838315	10.15561	10.24522	8.804595	9.02537	10.02889
ELAVL1	201727_s_at	4.765655	4.665602	5.059457	4.874337	4.417238	4.543142	6.323762	5.428699	6.712619	6.220305	5.567123	5.643781	7.089557
KIAA0100	201728_s_at	3.052703	3.03577	3.027642	3.043117	3.804797	3.048017	3.032088	3.465831	2.979029	2.99611	3.233155	3.301558	2.966621
KIAA0100	201729_s_at	7.378066	7.273933	7.61036	7.440394	7.171371	6.809769	7.700074	7.395314	7.838654	7.753504	7.438545	7.495087	7.859802
TPR	201730_s_at	8.481883	8.537944	8.825005	8.762273	8.74848	8.883573	9.557757	9.500545	9.336154	9.002695	9.602648	9.815377	9.86518
TPR	201731_s_at	7.243187	7.298108	7.300969	7.346588	6.437918	6.263212	8.504667	7.296241	8.584409	8.568729	7.348115	7.889565	8.988073
CLCN3	201732_s_at	4.257856	4.018306	5.437358	4.981782	5.395992	5.287077	6.385413	6.713681	6.584872	6.150306	5.761624	6.51432	6.284387
CLCN3	201733_at	6.79108	6.29259	6.766461	7.032325	7.396892	7.807924	7.781316	7.884268	8.093927	7.670534	7.727991	7.937267	6.460752
CLCN3	201734_at	7.392116	6.946629	7.877083	7.595147	7.349501	6.629141	9.649619	8.663065	9.856221	9.836635	8.094703	8.54593	8.870245
CLCN3	201735_s_at	6.817733	6.296369	7.306878	7.243459	6.7019	6.296868	8.596624	7.788082	8.65025	8.436955	7.503953	7.416211	8.191062
MARCH6	201736_s_at	8.35921	8.219041	7.756711	7.938326	7.192649	7.160317	8.091397	7.528523	8.332031	8.010919	7.501567	7.488125	8.381559
MARCH6	201737_s_at	8.379192	8.588502	8.16724	8.131577	7.464832	6.776307	8.517436	7.354869	8.885576	8.773678	7.379434	7.544554	10.17567
EIF1B	201738_at	9.907857	9.728917	9.930264	9.898051	9.694229	9.225149	10.82255	10.39574	10.91761	10.80986	10.19215	10.28889	11.04354
SGK	201739_at	9.710052	9.701642	10.34493	9.99271	9.020708	8.729091	8.005308	7.280915	7.924067	8.149919	7.478053	7.428138	8.833944
NDUFS3	201740_at	8.465471	8.539712	8.633208	8.810297	8.63075	7.950655	9.0266	8.655018	9.03262	8.986189	8.64424	8.749386	8.607471
SFRS1	201741_x_at	3.492465	3.474865	3.421681	3.45714	3.477175	3.416656	3.923202	3.446135	4.640031	4.684139	3.455678	3.468235	5.064558
SFRS1	201742_x_at	5.05222	4.838909	6.567534	4.558641	3.478139	3.040269	8.871583	5.170669	8.950648	8.777602	4.445797	5.277436	10.31547
CD14	201743_at	11.96236	11.89391	11.89518	12.02249	11.57232	11.58524	8.284774	7.574286	7.166379	7.688557	8.079405	8.523527	6.982486
LUM	201744_s_at	9.386871	9.754657	10.00934	9.433578	10.11218	9.917074	8.024142	4.959497	5.927693	6.690511	11.09228	7.311388	13.76873
TWF1	201745_at	8.683166	8.660457	9.055551	8.911383	8.338494	7.830118	8.774382	8.317884	8.805714	8.800123	8.065233	8.238478	9.066155
TP53	201746_at	5.814468	5.722691	4.990967	5.117708	5.179359	5.256177	6.775577	5.930183	6.555244	6.365321	6.082042	6.312004	5.61284
SAFB	201747_s_at	3.28854	3.173315	3.756166	3.252719	3.150904	3.238765	4.296118	3.267968	4.497687	4.826287	3.029271	3.269141	5.730337
SAFB	201748_s_at	8.185731	8.076048	8.101521	8.150748	8.36208	8.361768	8.65499	8.694555	8.685389	8.624389	8.821278	8.826211	8.299322
ECE1	201749_at	7.71221	7.504116	6.932359	7.143666	7.7838	7.819197	6.654494	6.932403	6.781195	6.996019	6.756129	6.958334	5.726093
ECE1	201750_s_at	2.351514	2.320048	2.373719	2.31315	2.380935	2.28813	2.504838	2.325092	2.322133	2.309647	2.306284	2.316245	2.19521

JOSD1	201751_at	6.579789	6.423627	7.557274	7.424674	6.52062	6.141041	7.781403	7.070066	7.582518	7.701652	7.373033	7.382918	7.836268
ADD3	201752_s_at	7.511269	7.184026	7.069494	6.758895	7.217232	7.181982	9.170331	8.413366	9.040722	8.750638	8.956724	8.851982	10.43644
ADD3	201753_s_at	9.265622	9.151693	8.685819	8.503924	8.039447	7.428189	11.00007	9.57129	11.19546	10.77403	9.831966	9.684268	12.13579
COX6C	201754_at	12.83445	12.85631	12.82203	13.01682	12.81795	12.75728	12.48152	12.45055	12.47019	12.47623	12.60533	12.69136	12.60802
MCM5	201755_at	3.637433	3.368005	2.600876	2.61851	2.627477	2.722297	7.636528	7.310997	7.544972	7.818784	6.57539	7.106481	5.139909
RPA2	201756_at	5.674419	5.071886	5.795114	5.644633	5.419924	4.856832	7.739596	6.837937	7.619371	7.791048	6.977482	7.117791	6.450967
NDUFS5	201757_at	10.98529	10.96366	10.82008	11.04933	11.41181	11.37052	11.13312	11.55265	11.39574	11.26307	11.90321	11.50189	11.66801
TSG101	201758_at	8.89533	8.743167	8.411067	8.517503	8.135079	7.617752	9.350613	9.23907	9.252111	9.367231	8.907309	9.251735	8.80723
TBCD	201759_at	5.359844	5.165738	4.814208	4.654342	5.347985	5.418924	5.344916	5.739646	5.964746	5.97131	5.327103	5.603418	4.145006
WSB2	201760_s_at	8.913662	8.85597	8.798563	8.853528	7.975072	7.742426	8.676534	8.057672	8.631801	8.739884	7.638543	8.129821	9.864358
MTHFD2	201761_at	4.699709	4.785177	4.335481	4.019202	4.915724	5.491984	8.55543	8.630087	8.866414	8.826144	8.221799	8.554831	9.03986
PSME2	201762_s_at	10.75617	10.788	10.46234	10.5466	10.60155	10.96398	9.195185	9.927842	9.28754	9.40281	9.994513	9.964394	9.032379
DAXX	201763_s_at	6.429549	6.182833	5.514523	6.071933	6.413135	6.774076	6.449805	7.291852	6.586067	6.665112	7.481381	7.45777	6.109316
TMEM106C	201764_at	7.578324	7.74015	6.789668	6.960235	7.064343	7.198462	7.73902	8.427113	7.673747	7.773877	8.53967	8.54316	7.793177
HEXA	201765_s_at	8.182302	8.170532	8.350965	8.478281	7.753952	7.630363	7.267138	7.024033	7.45208	6.899887	6.415338	6.902228	7.82574
ELAC2	201766_at	2.573372	2.487123	2.45794	2.464424	2.509859	2.628488	2.482247	2.487006	2.469257	2.457678	2.468943	2.478849	2.412949
ELAC2	201767_s_at	7.083955	7.311966	6.949929	7.181242	7.377007	6.909245	7.945917	8.378891	8.200352	8.628514	8.284218	8.37905	7.065228
CLINT1	201768_s_at	5.362454	5.161725	7.418753	6.079679	5.456934	5.083244	6.302482	4.495857	7.033221	7.093216	4.565927	5.014433	7.308449
CLINT1	201769_at	10.40377	10.32678	10.46704	10.38163	9.892081	9.462259	10.41968	9.693824	10.53754	10.29381	9.583913	9.742975	10.02076
SNRPA	201770_at	7.043243	7.150963	6.464829	6.922388	7.090409	7.289649	8.884992	9.119719	9.047828	9.101929	9.373153	9.383063	8.336836
SCAMP3	201771_at	7.754495	7.834475	7.684707	7.561851	7.523625	7.715339	7.844347	7.698342	7.825883	7.717296	7.771197	7.956592	7.711737
AZIN1	201772_at	8.337889	8.189268	8.82639	8.384411	7.84485	7.312086	8.748025	7.824107	8.5314	8.725926	7.179205	7.744869	9.730881
ADNP	201773_at	7.519897	7.444717	8.137794	7.81033	7.114656	5.913405	9.14669	7.553228	9.131906	8.963381	7.972359	7.749849	10.72491
NCAPD2	201774_s_at	3.725153	3.576655	3.676154	3.390697	3.714009	3.53352	8.013573	8.031388	7.840255	7.834772	7.499409	7.073236	6.334924
KIAA0494	201775_s_at	3.312407	3.405623	3.50295	3.470267	3.408444	3.387898	3.798061	3.156841	4.141059	4.028096	3.148454	3.22538	5.01954
KIAA0494	201776_s_at	6.587407	6.209813	7.283706	6.690606	6.566232	6.451537	7.084093	5.892318	6.859267	6.940423	6.14697	6.11632	7.948055
KIAA0494	201777_s_at	3.264828	2.878316	3.168995	3.588944	3.247842	3.208011	3.223914	3.231453	3.218967	3.209249	3.204655	3.4487	3.43671
KIAA0494	201778_s_at	8.237666	8.111854	9.057952	8.745224	8.368055	7.540229	8.249835	7.406757	8.345703	8.325054	7.737582	7.622271	8.97516
RNF13	201779_s_at	8.715866	8.65796	9.691376	9.554757	9.187268	8.656579	8.553795	7.502451	8.278673	8.362203	7.300558	7.910183	7.928225
RNF13	201780_s_at	9.172173	9.156804	9.627061	9.661298	9.422939	8.912835	8.538876	7.909382	8.622571	8.435741	7.55931	7.926648	7.933357
AIP	201781_s_at	4.246262	4.15027	5.297884	4.993779	4.223879	3.619323	4.285776	4.177872	4.673875	4.954678	3.92037	4.229194	5.996958
AIP	201782_s_at	7.656641	7.613697	7.451039	7.805068	7.915125	8.307187	6.647672	6.981504	6.890687	6.742298	7.540212	6.913082	7.288637
RELA	201783_s_at	8.272546	8.190409	7.695127	7.848989	7.806714	7.59381	7.343642	7.54764	7.657175	7.465231	7.711829	7.920685	7.121894
C11orf58	201784_s_at	9.863735	9.553642	10.09378	9.961856	9.225549	8.863211	10.86124	9.94714	10.71998	10.81111	10.09705	10.07795	11.6277
RNASE1	201785_at	5.543351	5.393646	6.232088	6.016849	6.347122	5.814899	6.526094	6.100922	7.118206	6.601607	6.377329	8.183912	8.207547
ADAR	201786_s_at	10.21213	10.25582	10.45311	10.66716	10.32238	10.18535	9.670983	9.768889	9.84228	9.688223	9.883068	9.686939	10.0006
FBLN1	201787_at	2.255634	2.237305	2.223323	2.231397	2.241358	2.239481	4.201392	3.595726	3.938688	3.416883	4.725241	3.564067	6.7894
DDX42	201788_at	8.830661	8.972921	8.392886	8.221789	8.68347	8.732396	8.971692	8.743603	8.790081	8.871423	9.060667	9.006111	9.196044

DHRS7	201789_at	2.442679	2.404663	2.384424	2.388291	2.40612	2.390444	2.39137	2.403035	2.390158	2.383426	2.394985	2.397945	2.254136
DHCR7	201790_s_at	8.057332	7.92861	7.174217	7.112491	8.462283	7.921326	9.689375	8.070831	9.566307	9.397592	7.325743	8.546689	6.175482
DHCR7	201791_s_at	9.261824	9.397636	7.732794	7.809686	9.919444	9.824284	10.04453	9.890526	10.00713	10.00672	9.703573	10.30565	6.30539
AEBP1	201792_at	6.783338	7.100572	6.471471	6.090199	7.415347	8.449588	4.742062	4.904465	3.717073	3.587312	6.918532	5.247476	8.385689
SMG7	201793_x_at	2.541452	2.536171	2.516884	2.519145	2.531265	2.512481	2.530365	2.531006	2.526594	2.521017	2.513315	2.525368	2.502467
SMG7	201794_s_at	7.513732	7.576861	7.386625	7.268851	7.336214	7.299843	7.441086	7.853275	7.722103	7.803646	7.788248	7.973418	6.96249
LBR	201795_at	9.897241	9.902361	9.333217	9.498098	9.92844	9.778925	11.87371	11.91984	11.82128	11.90027	11.68644	11.79588	10.16518
VARS	201796_s_at	2.664119	2.646088	2.584858	2.631583	2.770746	2.61393	3.402847	2.64036	2.652091	3.566375	2.640801	3.042257	3.441537
VARS	201797_s_at	6.630276	6.59467	6.653302	6.77914	6.713064	6.723002	7.328008	7.726043	7.595906	7.801364	8.229798	8.238614	6.211755
FER1L3	201798_s_at	3.623462	4.37193	4.767125	4.118402	4.535821	4.123244	4.160169	2.340611	2.492392	4.135473	6.329634	2.335087	9.462499
OSBP	201799_s_at	5.531965	5.609521	6.633503	5.963053	5.765412	5.780076	6.02424	5.601891	6.17751	6.158964	5.040385	5.491152	6.585661
OSBP	201800_s_at	7.546532	7.612736	8.00327	8.01711	7.835178	7.501112	7.618973	7.182014	7.705341	7.497088	7.175055	6.921094	7.202555
SLC29A1	201801_s_at	6.147178	5.755229	5.391133	4.863827	2.954445	2.310828	8.025503	5.195753	8.000203	7.725274	4.950447	5.234298	5.28894
SLC29A1	201802_at	6.645119	6.453587	5.133388	5.138523	4.87895	4.810817	6.944773	6.303972	7.392229	6.832751	5.932758	6.671641	3.91407
POLR2B	201803_at	8.860627	8.747986	8.84122	8.854229	8.64019	8.296275	9.828687	9.093437	9.767867	9.876939	9.184446	9.145719	10.06835
TBCB	201804_x_at	7.260334	7.094083	7.080634	7.176826	7.486557	7.603755	7.926227	7.534411	7.32601	7.503778	8.245012	7.763812	9.346697
PRKAG1	201805_at	5.436223	5.450012	5.919405	5.52955	5.058219	4.929482	7.041384	6.018152	6.952083	7.136346	5.976283	6.16691	6.585419
ATXN2L	201806_s_at	5.187412	4.975244	4.741694	4.432825	4.687244	4.728866	4.561542	4.987115	4.716	4.585108	4.629026	4.884682	4.028438
VPS26A	201807_at	9.55892	9.498741	9.675807	9.668999	9.258537	9.385352	10.41131	9.652642	10.49641	10.36029	9.67403	9.773545	10.54867
ENG	201808_s_at	2.349904	2.344294	2.321648	2.325779	2.343515	2.327294	2.395417	2.340886	2.330017	2.321163	2.320867	2.337171	2.310759
ENG	201809_s_at	9.095502	8.713479	8.689099	8.974096	9.573316	9.658564	7.885126	7.667096	7.93516	7.60304	7.960997	8.07713	7.068407
SH3BP5	201810_s_at	6.920417	6.566175	6.944508	6.665529	6.900041	6.856203	5.628438	5.910652	6.211672	5.670435	7.021353	6.393882	8.561009
SH3BP5	201811_x_at	9.420358	9.368138	8.950824	9.01081	8.889537	8.750209	8.048171	8.252348	8.638265	7.99706	9.045093	8.484593	10.60609
TOMM7	201812_s_at	11.37718	11.30471	11.20904	11.56424	11.90732	12.02322	11.012	11.15153	11.19027	11.13916	11.32157	11.41684	11.14103
TBC1D5	201813_s_at	5.748414	5.942264	6.49219	5.857672	5.806083	5.71347	6.89405	5.929189	6.804317	6.907473	5.647674	6.155367	7.560731
TBC1D5	201814_at	7.537438	7.493549	8.105226	7.940799	7.709413	7.597452	8.315173	8.071919	8.255371	8.262144	7.906757	8.053857	7.946156
TBC1D5	201815_s_at	2.712617	2.679683	2.683995	2.660559	2.68076	2.640563	3.351039	2.680898	3.315184	4.65679	2.665268	2.672478	4.951981
GBAS	201816_s_at	6.966121	6.812964	6.694972	6.719657	6.977393	6.767027	8.106884	7.632579	8.307362	8.298896	7.844043	7.731754	9.582289
UBE3C	201817_at	8.157723	7.97981	8.569107	8.379552	8.120084	7.637168	8.566235	8.035497	9.049338	8.823826	7.923478	8.112154	7.835094
AYTL2	201818_at	5.407844	5.467081	5.360586	5.541443	6.236149	7.050923	7.433557	7.586897	6.85292	7.160941	8.300168	8.200828	8.182816
SCARB1	201819_at	8.383912	8.428991	8.050985	8.340213	8.163739	7.912197	7.700562	7.980008	8.033238	7.846417	7.795024	7.919968	6.8252
KRT5	201820_at	1.960304	1.946558	1.926911	1.928128	1.947488	1.928491	1.940217	1.942754	1.929019	1.922863	1.923837	1.937011	4.909584
TIMM17A	201821_s_at	7.798717	7.912224	8.890706	8.593088	7.686747	7.3246	8.813196	8.120633	9.165065	9.197281	8.12624	8.459236	9.305036
TIMM17A	201822_at	3.515961	3.288471	3.717427	3.601854	3.504657	3.219195	3.473709	3.818638	3.304036	4.046172	3.405361	3.760184	2.970037
RNF14	201823_s_at	6.420169	6.379233	7.241367	7.095821	5.930577	5.481374	7.855733	7.071243	8.527576	8.334134	5.347154	6.852848	7.197301
RNF14	201824_at	7.585641	7.850206	7.903154	7.795019	7.369329	7.282102	8.24238	8.14112	8.522884	8.384738	7.520513	8.096599	7.135227
SCCPDH	201825_s_at	8.930147	8.896358	9.429677	9.617658	8.649014	8.180284	8.285309	7.832806	8.583592	7.971151	7.442532	7.740297	7.218956
SCCPDH	201826_s_at	8.212766	8.323943	8.703243	8.781387	8.015813	8.193762	6.932746	7.470697	7.095043	5.908918	6.52465	7.189794	6.001409

SMARCD2	201827_at	7.615956	7.842183	7.660429	7.595051	8.269536	8.292831	7.163648	7.751921	6.839994	7.394187	7.782095	7.589222	6.011101
FAM127A	201828_x_at	7.540032	7.624007	7.854843	7.537186	7.433462	7.737662	9.666408	9.503573	9.539531	9.613865	10.03763	9.16872	11.02318
NET1	201829_at	10.1483	10.15696	9.721334	10.02738	10.11002	9.832812	9.874201	9.955315	9.822609	10.01503	9.980899	9.749225	10.7144
NET1	201830_s_at	7.209309	7.091896	7.776714	7.132281	6.225355	4.886191	8.508231	6.784013	8.642103	8.407128	6.822587	6.739885	10.21602
USO1	201831_s_at	6.255883	6.11636	6.954455	6.732596	6.317529	6.022703	6.755691	6.389132	6.668299	6.390652	6.406894	6.361352	7.610429
USO1	201832_s_at	9.35016	9.272747	9.845419	9.554212	9.295317	8.701838	10.24189	9.209868	10.23642	10.2435	9.18878	9.038122	10.72495
HDAC2	201833_at	8.396045	8.221939	8.29984	8.402536	8.447154	8.323145	10.31336	10.3779	10.33959	10.52668	10.5167	10.42571	11.06081
PRKAB1	201834_at	6.965521	6.925896	6.711475	6.63366	6.705164	6.991857	9.268614	9.374038	9.227679	9.259632	8.988275	9.264435	7.369544
PRKAB1	201835_s_at	2.133581	2.106399	2.084481	2.090725	2.110863	2.088116	3.729672	2.104511	5.578331	5.788988	2.090958	2.102534	4.73889
SUPT7L	201836_s_at	4.635858	4.591248	4.444472	4.564961	4.342624	3.880899	5.543716	4.327436	5.310562	5.226551	4.838657	4.490387	6.48986
SUPT7L	201837_s_at	6.93384	7.148681	7.187827	7.287566	6.757109	6.808158	7.863574	7.446503	7.782703	7.733384	7.630999	7.510994	7.90509
SUPT7L	201838_s_at	3.328614	3.482648	3.238664	3.797796	3.316583	3.233824	4.284856	4.044512	4.52277	3.890544	3.369788	3.854341	4.352509
TACSTD1	201839_s_at	5.86574	6.244915	4.844371	4.920294	5.922149	5.611698	10.40505	10.31582	10.4163	11.0035	10.00166	10.59145	8.503394
NEDD8	201840_at	9.500935	9.519213	9.61219	9.763486	9.729301	9.608461	10.3987	10.23962	10.47991	10.41174	10.55797	10.54209	10.23459
HSPB1	201841_s_at	9.779601	9.806947	9.607582	9.493987	9.445079	9.27678	10.18981	9.820819	10.25032	10.55463	10.20468	10.23792	11.83395
EFEMP1	201842_s_at	4.008405	4.267679	4.954498	4.127418	5.837951	6.401911	2.855903	2.836032	2.826831	2.797978	3.805489	2.939688	8.308894
EFEMP1	201843_s_at	3.292667	2.675784	3.463278	2.655486	3.597593	5.039112	2.610572	2.612168	2.60441	2.583006	2.711331	2.699314	6.262625
RYBP	201844_s_at	7.540127	7.799134	8.051933	8.257609	7.694241	7.256421	8.310701	8.394658	8.440311	8.490439	8.303818	8.77055	8.628977
RYBP	201845_s_at	9.078277	8.906843	9.416055	9.451501	8.997413	8.42864	9.940559	9.523233	10.05366	10.17246	9.412265	9.755036	10.38311
RYBP	201846_s_at	6.998848	6.649864	7.873852	7.71277	7.515109	6.836362	8.245168	7.4196	8.272756	8.06585	7.780568	7.964416	8.488018
LIPA	201847_at	9.628189	9.425201	10.33766	10.24187	9.606377	8.947046	10.91138	9.140193	10.72491	10.90244	8.985963	9.335753	8.605251
BNIP3	201848_s_at	7.732355	7.61445	8.976984	8.772239	7.937903	7.572306	8.376377	7.22247	8.350595	8.509543	6.85527	7.311284	6.847687
BNIP3	201849_at	11.32809	11.29111	11.46734	11.61374	11.76455	11.61086	11.35275	11.35572	11.39215	11.45744	10.93645	11.19914	9.873077
CAPG	201850_at	3.731473	3.443986	3.309823	3.879335	3.872017	4.021792	5.050397	5.701769	4.468986	4.564331	5.562472	5.873601	5.658481
SH3GL1	201851_at	6.698714	6.5246	6.557233	6.565243	6.562738	6.403476	7.180069	7.523379	6.937539	7.360806	7.603768	7.289106	6.895763
COL3A1	201852_x_at	9.903562	10.14974	9.373014	9.090906	7.835597	7.318211	10.11111	6.710509	9.143136	9.644678	11.92619	8.24771	14.93976
CDC25B	201853_s_at	7.157986	7.387221	5.780569	6.417902	7.196019	7.434858	7.987154	8.46585	7.721163	8.339495	8.604997	8.553598	7.678295
ASCIZ	201854_s_at	6.189039	6.094701	6.661128	6.588721	6.614136	6.49745	6.142516	5.962514	5.859936	5.789369	6.03902	5.945374	6.308327
ASCIZ	201855_s_at	7.88784	7.779682	8.371875	8.234792	7.653922	6.890652	7.483354	7.256977	7.63734	7.65792	7.156095	7.028082	8.237431
ZFR	201856_s_at	5.068014	4.379692	5.242085	5.036759	5.477965	6.053432	5.960384	5.560926	5.960368	5.723579	6.241541	6.234858	7.258283
ZFR	201857_at	9.707432	9.601955	9.934863	10.01831	9.954127	9.526128	11.04863	10.50993	10.83817	10.81737	10.42531	10.53824	11.19213
SRGN	201858_s_at	4.816936	4.493038	8.536827	7.622021	5.405187	3.992198	7.940912	3.967825	7.764286	7.771389	3.883556	5.113616	6.373298
SRGN	201859_at	10.38823	10.21524	11.66298	11.55773	11.36404	11.15202	10.68862	10.57461	10.71778	10.99682	10.46135	11.10822	9.608135
PLAT	201860_s_at	3.57132	3.365074	3.82912	3.648389	4.100672	4.171124	3.598481	3.201485	3.40965	3.829198	6.008572	3.519192	8.468701
LRRFIP1	201861_s_at	7.864447	8.059632	7.911198	7.9231	7.870615	7.804259	4.763414	6.25257	6.092989	5.186355	6.090069	6.215511	5.32282
LRRFIP1	201862_s_at	10.60399	10.79809	10.35471	10.20434	9.658431	9.318935	8.47118	7.760079	8.566278	8.658922	8.228643	8.287515	7.522084
FAM32A	201863_at	8.304054	8.228709	8.165672	8.203848	8.341494	8.188741	8.22541	8.074658	8.151038	8.291113	8.358351	8.330334	9.054541
GDI1	201864_at	8.06656	8.169542	7.523787	7.870283	8.501635	8.905581	7.790363	8.332542	7.783776	7.719476	8.890972	8.332173	10.03304

NR3C1	201865_x_at	9.723144	9.79205	9.888255	9.645281	9.293971	8.87596	8.676666	8.302726	9.407693	9.239068	8.61837	8.457714	9.291833
NR3C1	201866_s_at	5.097741	5.265936	5.447797	5.217881	5.009864	4.919067	4.73588	3.597644	5.389489	5.137889	3.904148	4.085107	6.273698
TBL1X	201867_s_at	8.446647	7.780487	8.615883	8.751815	9.168135	9.317681	8.433734	9.182295	8.535485	8.355228	9.243082	9.288411	7.015882
TBL1X	201868_s_at	6.332675	6.057951	6.658617	7.026499	7.054522	6.454364	6.590178	6.799917	6.296342	6.313321	6.6364	7.314774	5.060526
TBL1X	201869_s_at	4.385259	4.327685	4.872296	4.487897	3.870472	3.57061	5.24722	4.262575	5.69239	5.814759	3.849141	4.143814	4.496496
TOMM34	201870_at	5.363125	5.362434	5.044551	5.230101	5.358265	5.207762	5.156704	5.716257	5.323529	5.318452	5.653777	5.460578	5.292316
LOC51035	201871_s_at	7.771333	7.82179	7.14454	7.470143	8.019852	8.199806	7.796506	8.640346	7.948408	7.913097	8.560345	8.693301	7.351431
ABCE1	201872_s_at	7.54289	7.367547	7.722968	7.445108	7.191936	7.076716	8.898603	8.33691	8.955913	8.861317	8.430956	8.22231	9.136911
ABCE1	201873_s_at	7.437473	7.435169	7.700889	7.494875	6.857273	6.531248	8.489535	7.881987	8.537073	8.553586	7.76335	7.904794	8.047114
MPZL1	201874_at	5.061887	5.08727	5.427842	5.332246	5.286218	5.874903	6.287797	5.892629	6.562561	6.604633	6.205344	5.496544	8.11236
MPZL1	201875_s_at	3.606622	3.613509	3.448948	3.441701	3.120768	3.600896	4.475257	3.852931	4.386489	4.260563	4.182189	3.63132	6.219478
PON2	201876_at	10.66769	10.54886	10.83445	11.01776	10.11997	10.16753	11.07352	10.97353	11.65777	11.52823	10.59138	11.16722	9.419044
PPP2R5C	201877_s_at	8.339457	8.3611	8.974275	8.952306	8.227891	7.782725	8.654146	8.198415	8.805294	8.900436	8.427852	8.335179	9.736727
ARIH1	201878_at	5.484499	5.281087	5.807082	6.04612	4.636436	4.61247	5.652768	5.642716	5.697444	5.729012	4.900737	5.08775	7.32314
ARIH1	201879_at	5.79258	5.50491	6.620448	6.454012	5.496255	5.763084	6.409118	5.49946	6.114619	6.115115	5.648245	5.768422	6.853078
ARIH1	201880_at	8.369149	8.338776	8.666098	8.667338	8.300883	7.743499	8.334259	8.187224	8.622933	8.804877	7.986739	8.074038	8.219621
ARIH1	201881_s_at	5.516075	5.141829	5.951119	5.880017	5.173027	4.543423	5.659419	4.660902	5.93548	5.674649	5.022644	5.576309	6.815884
B4GALT1	201882_x_at	2.999322	2.897643	2.957941	2.955889	2.9709	2.959078	2.97352	2.970521	2.968552	2.944002	2.952611	3.064879	2.923709
B4GALT1	201883_s_at	7.487225	7.649274	6.992431	7.398945	7.281868	7.223422	6.72657	7.380939	7.363837	6.744762	6.454938	7.508406	5.180809
CEACAM5	201884_at	2.593945	2.497293	2.426937	2.483751	2.591805	2.631955	2.467192	2.50463	2.490575	2.43843	2.515887	2.503922	2.342406
CYB5R3	201885_s_at	10.51415	10.44382	10.35239	10.41047	10.14609	10.48798	10.33618	10.41033	10.45667	10.34367	10.37689	10.45447	10.03017
WDR23	201886_at	9.191717	9.193526	9.493863	9.723148	10.17698	10.27609	9.151362	10.16828	9.519169	9.588128	9.498793	10.06066	5.695028
IL13RA1	201887_at	10.47854	10.4819	10.30534	10.43764	10.72379	10.43993	7.915353	7.532701	7.850821	7.823669	8.019296	7.788153	7.847187
IL13RA1	201888_s_at	8.362647	8.189045	8.168296	8.195359	8.021076	7.801826	4.329043	4.763291	4.934656	4.602547	4.806515	4.810323	4.759874
FAM3C	201889_at	9.092374	9.002898	8.901214	8.432371	8.582701	7.938719	8.855972	8.157271	9.221953	8.80386	8.548245	8.106495	10.60922
RRM2	201890_at	7.865274	7.544876	3.983091	3.608411	3.855491	4.886705	12.47956	12.35812	12.79961	12.9051	11.91389	12.12141	10.11223
B2M	201891_s_at	13.96863	13.99609	13.89765	13.98715	13.64363	13.65973	13.0963	12.61641	13.08535	13.06907	12.12787	12.39496	12.82747
IMPDH2	201892_s_at	8.985283	8.934575	8.802589	8.845867	9.240085	9.062423	10.63906	10.71889	10.56541	10.7295	11.04332	10.97432	10.65097
DCN	201893_x_at	9.493714	9.605043	10.8468	10.33516	9.850946	9.466721	8.810038	7.002124	8.484639	8.580868	9.640814	8.033501	13.22578
SSR1	201894_s_at	8.412147	8.613476	9.526853	9.090772	8.866727	8.065288	10.44552	9.202941	10.56795	10.3931	9.124758	9.302038	10.54969
ARAF	201895_at	7.671433	7.512764	7.446946	7.545174	7.411784	7.335942	7.446956	7.267036	7.40108	7.411824	7.111773	7.39633	6.149531
PSRC1	201896_s_at	2.717483	2.706054	2.568588	2.582283	2.604712	2.576282	5.07449	3.867516	5.110719	4.917308	5.824533	5.659716	6.274621
CKS1B	201897_s_at	7.977761	7.734311	8.005412	8.280899	7.379705	7.612847	10.37925	10.19907	10.37015	10.42518	10.16609	10.31639	9.348757
UBE2A	201898_s_at	7.098978	7.036285	7.229016	6.970178	6.33324	5.553676	8.31107	7.915174	8.292777	8.405297	7.398357	7.609447	8.102309
UBE2A	201899_s_at	8.99032	8.791427	8.678838	8.737653	8.328881	7.755041	9.166501	8.791112	9.078014	9.111852	8.931128	8.880097	9.96143
AKR1A1	201900_s_at	10.0114	9.896391	10.23647	10.23737	10.24985	10.15007	9.311937	9.179194	9.315111	9.308155	9.037545	9.226888	8.890116
YY1	201901_s_at	9.371428	9.308952	9.827541	10.00597	9.373106	9.268617	10.37531	9.942764	10.05004	9.774034	10.01131	10.20025	9.863155
YY1	201902_s_at	2.588877	2.646624	2.626414	2.630268	2.645393	2.628042	2.641331	2.644255	2.633112	2.746576	2.623319	2.636541	2.634998

UQCRC1	201903_at	11.06909	11.1402	10.75675	10.88873	10.93247	10.8152	10.96293	10.67805	10.99197	10.93096	10.65603	10.97514	9.849862
CTDSPL	201904_s_at	5.862543	5.697858	5.363841	5.15207	5.590076	5.818455	4.894259	5.340415	4.509822	5.129856	5.747689	5.570961	4.767521
CTDSPL	201905_s_at	5.116353	4.269653	4.04143	4.004102	4.010298	4.106199	3.797452	4.025542	3.696405	4.139479	4.170105	4.01696	3.928001
CTDSPL	201906_s_at	8.115041	8.166388	7.460963	7.675857	8.099335	8.259018	7.405971	7.578416	7.014944	7.5036	8.067772	7.938416	7.037462
DVL3	201907_x_at	2.629452	2.639853	2.594337	2.608021	2.636837	3.503089	2.594781	2.620284	2.585453	2.59631	2.760113	2.616838	2.527863
DVL3	201908_at	5.824048	5.724435	6.245747	5.022051	4.307263	3.855098	8.163897	6.197683	7.703519	7.866106	5.921492	6.484968	8.461842
RPS4Y1	201909_at	11.13695	11.36972	10.98792	10.99196	2.499464	2.270718	12.28809	11.97719	12.43859	12.45848	2.486857	12.23179	12.61853
FARP1	201910_at	7.747558	7.857542	7.424628	7.667987	7.711992	7.965234	7.484919	7.110935	7.198154	7.401885	7.471349	7.33042	8.196756
FARP1	201911_s_at	7.847591	7.868777	7.573596	7.826398	7.904399	7.66841	6.904811	6.766622	6.901621	6.877777	7.335563	6.612287	8.141045
GSPT1	201912_s_at	7.162472	6.945064	8.204497	7.838029	6.647617	6.18443	9.707466	8.625341	9.875606	10.03885	8.345269	8.609537	8.189066
COASY	201913_s_at	8.027602	8.113512	8.014246	8.191775	8.296728	8.096095	8.345844	8.557922	8.087925	8.080751	8.668857	8.916566	6.088084
SEC63	201914_s_at	6.76148	6.790447	8.16695	7.61218	7.094015	6.908953	7.034577	6.58162	7.146276	6.930744	6.861084	6.88056	7.348478
SEC63	201915_at	6.253969	6.158342	6.958095	6.802864	6.007702	5.677997	5.523549	5.222566	6.034768	5.767937	5.490522	5.820471	6.199881
SEC63	201916_s_at	9.800021	9.669292	10.23181	10.11148	9.597914	8.973054	9.220051	9.220451	9.775479	9.562478	9.136661	9.104156	9.406866
SLC25A36	201917_s_at	4.49672	3.04232	5.069818	4.31207	4.888583	5.87541	7.397547	6.736676	6.887269	6.714478	7.979774	7.446155	9.446889
SLC25A36	201918_at	5.764396	5.979895	5.753251	5.736231	6.649064	7.040284	7.815709	8.129778	7.438968	7.713412	8.959061	8.459086	9.489198
SLC25A36	201919_at	4.624792	4.618958	5.367364	5.348019	4.665573	4.876783	7.96278	7.206089	7.833949	7.671558	7.489447	7.260926	9.58182
SLC20A1	201920_at	9.330192	9.526597	8.552459	8.640994	8.682453	8.28413	10.38183	10.10059	10.43137	10.2687	9.751697	10.06652	8.762954
GNG10	201921_at	10.2479	10.28421	10.24929	10.09802	10.33552	10.32573	9.883107	10.00309	9.78922	9.85659	10.10494	10.24286	10.70187
TINP1	201922_at	10.11058	10.06749	10.08515	10.11329	9.878074	9.297855	10.36324	9.80239	10.30591	10.41901	9.929714	10.0192	10.86154
PRDX4	201923_at	11.05589	11.04952	11.42335	11.49628	11.36795	11.01792	11.29352	11.1063	11.49793	11.47922	11.2149	11.25344	10.57018
AFF1	201924_at	10.85531	10.79145	10.55184	10.62486	10.54221	10.29394	11.5574	11.28912	11.62333	11.80814	10.8762	11.17261	9.03225
CD55	201925_s_at	7.521389	7.401995	8.539083	8.833085	6.395566	6.127812	7.743971	7.861769	8.237754	8.361673	6.468838	7.561347	6.909445
CD55	201926_s_at	5.786205	5.9144	7.145458	7.219803	5.741779	5.658957	6.664309	6.973418	7.411936	7.38649	5.570955	6.426774	6.347426
PKP4	201927_s_at	6.290409	5.622759	5.913194	5.994352	6.033434	6.084693	6.402033	6.080254	5.792932	6.224476	6.011527	6.101765	4.989983
PKP4	201928_at	8.805126	8.876443	7.954171	8.296475	8.48055	8.475261	8.842968	8.918461	8.909743	9.044252	8.70552	8.8643	8.256554
PKP4	201929_s_at	4.928576	5.26639	5.67238	4.979913	3.567607	2.441356	5.718807	4.07591	5.985027	5.963841	3.944433	3.966621	5.5291
MCM6	201930_at	6.839602	6.905042	5.861198	5.797031	6.241835	6.397107	10.9725	10.85233	11.11386	11.00295	10.5538	10.88625	9.659851
ETFA	201931_at	11.92615	11.88686	12.73598	12.68074	11.85125	11.23115	11.84549	11.10369	12.05312	11.60303	10.82693	10.99124	10.4782
LRRC41	201932_at	6.413883	6.282973	6.582703	6.652716	5.969152	6.133441	7.757683	6.530714	7.438253	7.214138	6.440198	6.888203	7.487765
CHMP1A	201933_at	7.848352	8.100894	7.046441	7.065826	7.980041	8.344358	7.646674	8.348981	7.661184	7.77423	8.566326	8.688306	7.533174
WDR82	201934_at	9.342094	9.267003	9.418928	9.335278	9.460682	9.442536	9.780645	9.962948	9.979374	10.15733	10.21657	10.04842	10.96307
EIF4G3	201935_s_at	7.214165	7.009126	6.707095	6.54265	6.064502	6.563952	7.236623	6.263553	6.691844	6.76636	6.908024	6.778417	8.430612
EIF4G3	201936_s_at	7.515551	7.512963	6.938291	6.88618	7.033136	6.980426	7.105719	7.028567	6.960308	6.801635	7.423376	7.142737	7.827985
DNPEP	201937_s_at	6.53158	6.459198	6.735548	6.624099	6.292756	6.278332	6.276591	6.078001	6.217314	6.146459	6.134328	6.176889	5.876188
CDK2AP1	201938_at	8.778006	8.617916	9.101073	9.020537	8.480264	8.274045	8.723577	8.384108	9.004048	9.061575	9.744401	8.865331	11.46674
PLK2	201939_at	8.156043	8.1044	7.468001	7.254345	7.841064	7.410161	6.785736	6.272741	6.63463	6.600522	8.249614	6.555951	9.168149
CPD	201940_at	6.084297	6.194217	7.15256	7.065356	6.298253	5.936673	6.402735	5.865343	7.26839	6.747949	5.951407	5.981027	6.451751

CPD	201941_at	7.436477	7.283066	8.347067	8.15622	7.810226	6.698135	8.00065	7.36441	8.673092	7.745799	7.162073	7.417617	7.362675
CPD	201942_s_at	3.097006	2.972884	3.892858	3.160363	2.823643	3.573759	3.057852	2.974398	3.384497	3.032295	2.756801	2.593197	4.526259
CPD	201943_s_at	5.985133	6.105465	6.805096	6.093846	5.607889	5.581263	6.089752	5.946596	6.832566	6.30228	5.876865	6.09111	6.397528
HEXB	201944_at	10.3004	10.05352	10.01722	10.17818	10.15477	9.818145	9.437274	9.115682	9.402675	9.344085	8.93925	9.182159	8.528769
FURIN	201945_at	9.161473	9.062384	8.963455	8.940567	8.412875	8.534388	8.598257	7.622245	8.157659	8.417503	7.699921	7.903106	5.834225
CCT2	201946_s_at	4.870101	3.815815	6.776322	5.956814	4.450938	3.539699	9.35235	7.347715	9.283053	9.359049	7.181698	7.8353	9.864425
CCT2	201947_s_at	9.688314	9.668296	10.03103	10.16487	9.523911	9.050804	12.44211	11.79145	12.43925	12.57305	11.72217	11.98225	12.1147
GNL2	201948_at	7.053065	7.269568	7.344588	7.567971	7.457151	7.148611	8.203949	8.306405	8.445395	8.359854	8.450223	8.707204	8.280931
CAPZB	201949_x_at	9.227493	9.055328	8.886355	8.845257	9.155754	9.217743	9.078847	9.194858	8.856366	8.831244	9.696519	9.590542	8.792238
CAPZB	201950_x_at	7.100899	7.208369	7.721907	7.717179	7.171812	7.133088	8.122134	6.837727	7.664116	8.208851	7.504042	7.529714	8.949004
ALCAM	201951_at	8.438001	8.431642	8.33792	8.198653	8.082088	7.908209	8.226919	7.842677	7.746458	7.940086	8.189673	8.082657	9.65779
ALCAM	201952_at	10.70194	10.86521	10.00406	10.2675	10.57697	10.50743	10.0306	10.32938	9.674203	9.718449	10.72577	10.60444	10.83395
CIB1	201953_at	9.195858	9.192402	8.996651	9.173823	9.339319	9.281578	8.229504	8.29746	8.001862	8.262161	8.251007	8.203286	6.420851
ARPC1B	201954_at	8.233576	8.254262	7.689533	7.705585	8.869435	9.130714	7.754818	8.199273	7.702415	7.703487	8.351609	8.436414	7.444548
CCNC	201955_at	9.994053	9.854107	10.27782	10.36308	9.730005	9.492823	10.71649	10.21722	10.79993	10.81379	10.04684	10.37419	9.887199
GNPAT	201956_s_at	8.148697	8.217244	8.198599	8.038676	8.348827	8.079031	8.903155	8.913383	8.968828	8.947409	8.647412	8.797503	8.625368
PPP1R12B	201957_at	5.739386	5.91832	6.489845	6.472498	5.357075	5.632358	5.724593	4.404257	5.589233	5.626111	5.758924	4.72796	7.116282
PPP1R12B	201958_s_at	2.066621	2.058141	2.039193	2.041127	2.056345	2.040022	2.054585	2.058174	2.044327	2.037104	2.041761	2.049441	1.992474
MYCBP2	201959_s_at	8.722957	8.675821	9.16362	9.155955	9.527056	9.169412	8.515714	8.972039	8.469522	8.374562	9.07587	8.985533	9.530434
MYCBP2	201960_s_at	9.624483	9.676466	10.11761	10.35051	10.50591	10.62193	9.123929	9.83421	9.248375	9.34128	10.00161	9.934862	10.55844
RNF41	201961_s_at	4.256643	3.801372	4.357287	4.235421	3.950262	3.723634	4.8606	4.309905	4.886805	4.855073	4.228166	4.274329	5.739879
RNF41	201962_s_at	4.714311	4.216929	4.740606	4.73599	4.529511	4.773135	5.317121	4.916242	4.860639	5.477117	4.913636	4.74932	5.857758
ACSL1	201963_at	13.01473	12.99661	13.43259	13.22075	12.64588	12.14182	9.67295	9.60379	10.3595	10.28952	8.100167	8.498769	7.981389
SETX	201964_at	6.903995	6.692806	7.362902	6.833793	6.271045	5.44298	6.672697	6.069358	7.197795	7.047148	5.748711	5.801178	7.566037
SETX	201965_s_at	5.560292	5.55084	5.443047	5.53561	5.655266	5.539241	5.349294	5.115832	5.529246	5.322856	5.135019	5.518161	5.61359
NDUFS2	201966_at	9.94801	9.913145	10.01046	10.15018	10.07871	9.821879	9.692786	9.646085	9.54272	9.431318	9.747412	9.977706	8.786132
RBM6	201967_at	8.133051	8.281416	7.331353	7.728889	8.206868	8.37245	7.03376	7.645594	6.989635	6.857832	7.873092	7.50432	7.136131
PGM1	201968_s_at	11.70569	11.64866	12.17668	12.2051	11.90796	11.80568	10.28709	10.35491	10.52274	10.34469	10.36079	10.02988	9.554278
NASP	201969_at	5.306426	5.606643	5.256979	5.29769	5.288625	5.568757	7.439915	7.418125	7.270811	7.447512	7.573803	7.712191	6.799675
NASP	201970_s_at	8.92659	9.012826	8.789145	8.988783	8.910129	8.80636	10.78846	10.65705	10.81753	10.65938	10.69055	10.58103	10.36426
ATP6V1A	201971_s_at	3.602751	3.557748	6.603798	4.874251	2.695882	3.023423	7.38675	3.370989	7.309934	7.277072	2.83458	3.063093	7.962773
ATP6V1A	201972_at	9.395584	9.511484	9.834755	9.947925	9.185742	8.846855	10.22088	9.660949	10.23579	10.31149	9.421956	9.710981	9.587991
C7orf28A	201973_s_at	9.708823	9.65429	9.91699	9.705689	9.644194	9.158354	10.73986	9.7749	10.61509	10.77678	10.28514	10.05554	11.41799
C7orf28A	201974_s_at	3.018319	2.961929	2.937619	2.916314	2.998339	2.949161	2.94671	2.944172	2.942477	3.001688	2.966167	3.263734	2.783849
CLIP1	201975_at	9.584874	9.394029	9.27963	9.304833	8.936218	8.463681	8.301114	7.95002	8.125305	8.23551	6.800713	7.850873	8.010885
MYO10	201976_s_at	7.118348	7.094478	7.47463	7.342476	7.507091	7.304205	5.874385	5.495374	5.654803	5.785753	6.444715	5.585036	8.263059
KIAA0141	201977_s_at	5.476686	5.249268	5.670175	5.716485	5.44935	5.246652	5.382111	4.833655	5.534432	5.501189	5.012175	4.954876	5.541886
KIAA0141	201978_s_at	7.394369	7.374112	6.90997	7.036077	7.26081	7.201383	7.061451	7.086187	7.05576	7.047426	7.047416	6.9847	6.593032

PPP5C	201979_s_at	2.762874	2.687475	3.164638	2.776615	2.733664	2.670771	3.53212	2.696199	3.651173	3.248678	2.665377	2.775059	5.351259
RSU1	201980_s_at	6.579254	6.638829	7.235069	7.00574	6.890042	6.324251	8.870799	8.337665	9.216513	8.805877	7.866423	8.375856	9.096022
PAPPA	201981_at	2.901097	2.874984	2.794854	2.846003	2.921531	2.856071	2.858545	2.872163	2.858657	2.832934	2.859676	2.866861	2.819961
PAPPA	201982_s_at	2.16177	2.148819	2.130982	2.134502	2.15136	2.135163	2.146375	2.147954	2.136039	2.129773	2.130061	2.140808	2.101693
EGFR	201983_s_at	9.894989	9.868911	10.24212	10.28967	9.38113	9.047503	8.264948	7.389957	8.011695	7.868696	7.850952	7.399917	8.655613
EGFR	201984_s_at	7.459576	7.209971	8.035431	7.849122	6.487302	6.224343	5.43794	4.988582	5.265421	5.339589	4.899714	4.983254	5.499005
KIAA0196	201985_at	6.782286	6.462031	6.869959	6.975923	6.630991	6.326203	7.40172	6.974869	7.628259	7.530617	7.055443	6.950304	7.040447
MED13	201986_at	8.414045	8.387701	8.809483	8.355971	7.73083	7.067409	8.694498	7.265882	8.464062	8.578384	7.731556	7.509206	8.290679
MED13	201987_at	10.89042	10.86111	10.37073	10.39837	10.8982	10.44969	10.1501	10.33665	10.35497	10.19657	10.27606	10.42198	10.13456
CREBL2	201988_s_at	3.363418	3.638953	4.99277	4.191998	3.351094	3.097172	3.141397	2.820849	3.10566	3.095429	2.806481	2.815631	4.09184
CREBL2	201989_s_at	8.875332	8.719092	9.351567	9.307458	9.2147	8.969525	7.294851	7.22507	7.319236	7.45544	6.619563	6.943894	7.81232
CREBL2	201990_s_at	7.920452	7.862116	8.618951	8.555629	8.337113	7.716984	6.195712	6.09529	6.489441	6.298509	5.425	5.870287	5.48124
KIF5B	201991_s_at	10.25005	10.29037	10.18666	10.18677	10.05854	9.91815	10.15389	10.25981	10.46008	10.47164	10.57385	10.30725	11.74975
KIF5B	201992_s_at	3.346738	3.699666	3.81521	3.761898	3.778676	3.761095	3.84534	3.858134	4.380241	3.744016	3.943034	3.880385	6.888474
HNRPDL	201993_x_at	9.087573	9.024468	9.384499	9.421962	9.175553	8.988906	9.865039	9.516182	9.803795	9.778659	9.854314	9.804124	10.86996
MORF4L2	201994_at	11.06047	11.14177	11.23461	11.19728	10.99624	10.81405	11.79278	11.57232	11.93374	11.8663	11.66209	11.4688	12.61321
EXT1	201995_at	9.653991	9.57271	9.260315	9.413807	9.063178	9.053597	8.049487	7.920204	7.297905	7.742925	8.539452	8.16623	8.530468
SPEN	201996_s_at	8.064152	7.749192	8.093922	8.246827	8.853886	9.135767	8.101112	8.317587	7.810295	7.607201	8.756883	8.849554	7.217283
SPEN	201997_s_at	7.944459	7.755711	7.915318	7.772364	7.787788	7.652927	7.997064	7.57039	8.068161	8.040495	7.559157	7.470021	7.742339
ST6GAL1	201998_at	11.55248	11.54248	11.85779	12.01574	11.64205	11.41355	9.731609	9.636685	10.37278	10.54003	8.546368	8.978262	6.7721
DYNLT1	201999_s_at	9.388151	9.181729	9.475274	9.538984	9.032352	8.754196	9.969199	9.716279	9.725585	9.872099	10.0803	9.822668	11.07869
NDUFA6	202000_at	6.114822	6.143598	5.730524	5.879878	5.955962	5.934445	7.002607	7.318923	7.298674	7.18709	6.778094	6.905348	7.691549
NDUFA6	202001_s_at	11.16363	11.03688	11.28766	11.52802	11.29001	11.33346	11.66324	12.15376	11.66306	11.64845	11.63145	11.84172	11.36275
ACAA2	202002_at	4.523142	4.463448	4.478272	4.962527	4.275399	4.509867	4.73829	4.485745	4.847199	5.108347	3.844652	4.478883	4.250512
ACAA2	202003_s_at	11.13802	11.10578	11.82868	11.96651	11.36081	10.97933	9.216634	8.590872	9.220256	9.041644	8.063457	8.434294	7.755555
SDHC	202004_x_at	8.217737	8.156201	8.576745	8.256189	7.953541	7.799327	7.695732	7.180768	7.828225	7.797746	6.803224	6.961619	7.17641
ST14	202005_at	6.640389	6.363687	4.603783	5.345826	4.74709	4.960768	4.519346	4.277935	4.13505	4.23967	4.259261	4.035166	3.891455
PTPN12	202006_at	7.398878	7.154659	7.794218	7.463457	6.828477	6.322698	8.372772	6.695034	8.554978	8.345359	7.218172	6.908828	9.646772
NID1	202007_at	9.944854	9.733398	10.37119	10.09043	8.380046	7.538955	10.37332	9.018748	10.00369	10.02476	9.466367	9.111807	11.15218
NID1	202008_s_at	4.987556	4.898167	6.744676	5.651523	3.58171	3.578512	6.851485	4.108133	6.375287	6.39766	3.688453	4.19874	7.956063
TWF2	202009_at	6.010078	5.961738	5.444802	5.55502	6.743996	6.307782	5.749387	5.928769	5.770154	5.758792	6.57096	6.056948	4.880985
ZNF410	202010_s_at	6.184792	6.745782	6.311834	6.660465	6.327989	5.877403	6.7649	6.968982	7.103371	7.173418	6.915718	7.056516	6.973418
TJP1	202011_at	9.923474	9.756905	9.270157	9.199626	9.216015	8.776035	8.699498	7.891155	8.571252	8.430353	8.36572	7.815428	10.35305
EXT2	202012_s_at	7.011122	7.117593	7.289541	7.055886	6.3593	6.213526	6.892346	6.589078	6.851391	6.723363	7.055943	6.888151	8.374696
EXT2	202013_s_at	6.139441	6.124857	6.060713	6.198362	6.148883	6.109309	6.197577	6.249978	6.024593	6.359634	6.520987	6.522153	7.086067
PPP1R15A	202014_at	2.624374	2.657897	4.138078	3.483148	3.174446	3.148161	5.292416	5.213426	5.886213	5.740521	4.318356	4.949634	3.109989
MEST	202016_at	8.251293	8.279316	9.207928	9.042293	8.554711	8.137053	10.44955	9.392885	9.416379	10.05206	11.28217	9.799915	13.35629
EPHX1	202017_at	12.8323	12.82164	13.15364	13.20418	12.52639	12.32481	10.37627	9.878101	10.35216	10.18474	9.718775	9.490474	7.288735

LTF	202018_s_at	2.465231	2.521687	2.412092	2.490465	2.487482	2.506323	2.518757	2.521824	2.530546	2.499358	3.01782	2.671182	2.26148
LANCL1	202019_s_at	2.609688	2.590185	2.529383	2.572673	2.599527	2.568875	2.830294	2.595317	3.132135	3.000812	2.581055	2.583011	4.754454
LANCL1	202020_s_at	7.775452	7.737307	8.164871	8.047947	7.526334	7.24969	8.39238	7.761271	8.577421	8.602421	7.74064	7.816412	9.044832
EIF1	202021_x_at	11.20524	11.23632	11.24903	11.22332	10.83848	10.3342	11.49993	10.58855	11.52701	11.51167	10.92042	10.86815	12.45699
ALDOC	202022_at	7.844629	8.110414	6.814925	6.783933	8.611692	8.068361	6.682074	6.602736	7.070129	6.581713	6.127019	6.597467	6.185839
EFNA1	202023_at	11.34419	11.22222	10.40175	10.43302	11.58142	11.54473	8.195827	8.02518	8.291853	8.340866	8.488884	8.654769	7.395693
ASNA1	202024_at	5.636219	5.601237	6.602595	6.632838	5.603599	5.081944	7.362212	6.607719	7.590903	7.602634	6.423551	6.293395	7.526264
ACAA1	202025_x_at	9.137355	9.047119	9.900793	9.993904	10.14875	9.731006	9.105895	8.847698	8.497637	8.52161	8.401795	8.749502	5.505286
SDHD	202026_at	11.12519	10.93796	11.7699	11.79476	10.68479	9.958353	10.6873	9.330671	10.63822	10.62585	9.135673	9.2761	9.941107
TMEM184B	202027_at	5.382298	5.376279	5.018374	5.094605	5.326174	5.613648	5.325991	5.49325	5.307342	5.070623	6.517734	5.805887	6.481342
RPL38	202028_s_at	8.485291	8.850967	7.969559	8.756512	9.096485	9.368291	8.780289	9.536509	8.74948	8.559293	9.154742	9.265861	8.340473
RPL38	202029_x_at	12.29585	12.21669	12.17458	12.27449	12.48	12.57748	12.80378	13.02391	12.91281	12.82143	13.13077	13.16753	12.94306
BCKDK	202030_at	8.195753	8.097752	7.119714	7.411989	7.02853	7.056381	5.72876	5.995167	5.586408	5.71822	5.945563	6.196908	4.995965
WIPI2	202031_s_at	9.308143	9.248144	8.50884	8.9317	9.036073	8.864803	9.002048	9.810172	9.27269	9.248918	9.539883	9.662433	8.012223
MAN2A2	202032_s_at	6.665753	6.911502	6.811686	6.863818	6.42464	6.650579	8.813779	7.987514	8.688378	8.662653	7.692938	8.206545	6.10683
RB1CC1	202033_s_at	9.236559	8.803003	9.928616	9.73469	8.826623	8.203377	9.618941	8.357316	9.494657	9.834463	8.159321	8.456873	9.794362
RB1CC1	202034_x_at	10.09921	10.05851	10.21568	10.40577	9.724213	9.539645	9.705329	9.79646	9.842245	10.16672	9.378519	9.568084	9.528135
SFRP1	202035_s_at	2.633842	2.585228	2.541448	2.571434	2.706732	2.629358	3.073969	2.689542	2.607246	2.599953	7.10962	5.330857	6.719779
SFRP1	202036_s_at	4.770916	4.632212	3.960539	4.446636	4.793473	4.327084	5.573471	6.068372	4.590547	4.552503	9.385877	7.898461	8.961403
SFRP1	202037_s_at	4.624034	4.593616	4.792227	4.81662	3.579268	4.094033	6.609171	5.354743	6.151563	5.941497	8.562978	5.952292	10.32898
UBE4A	202038_at	9.220128	9.186026	9.212171	9.409832	9.028452	8.954139	8.944549	8.809662	9.141851	8.916467	8.774771	8.904516	8.931603
MYO18A	202039_at	8.442503	8.568172	8.38666	8.459265	8.508172	8.300358	7.443478	7.508301	7.233712	7.31067	7.678348	6.797918	6.452292
JARID1A	202040_s_at	8.260463	8.097927	7.436843	7.839164	8.651066	8.689722	8.107771	8.762773	8.053654	7.813901	8.812047	8.752036	7.926415
FIBP	202041_s_at	7.714508	7.730242	7.910324	8.01722	8.062689	8.221538	8.056876	8.31248	8.064439	8.304745	8.896031	8.726345	8.808975
HARS	202042_at	7.573577	7.533723	7.473649	7.637124	7.510789	7.60843	8.866311	8.817261	8.832982	8.70156	8.716522	8.826233	8.6312
SMS	202043_s_at	7.994139	8.104851	8.080993	7.820797	7.875235	7.025181	10.05652	8.928887	9.864273	10.08654	9.003153	9.12189	9.916963
GRLF1	202044_at	6.150058	6.531986	7.575155	7.31357	7.272681	6.698901	5.56226	5.544319	5.013293	5.20468	5.205638	5.632455	4.206945
GRLF1	202045_s_at	6.333975	5.859422	5.471648	5.741365	5.474461	5.429859	5.434631	5.432087	5.427128	5.419866	5.419906	5.422345	5.459357
GRLF1	202046_s_at	2.655128	2.636889	4.693735	3.102769	2.491122	2.602266	3.228524	2.500039	2.739037	2.83761	2.47675	2.48549	2.504063
CBX6	202047_s_at	5.527148	5.420106	5.384369	5.160284	5.669004	6.22908	5.530001	6.446102	6.286369	6.383311	7.100077	6.844334	7.082062
CBX6	202048_s_at	5.449873	5.383158	5.252965	5.429582	5.956607	6.184546	5.459674	5.807364	5.44047	5.500105	6.587299	6.206711	5.44552
ZMYM4	202049_s_at	6.72847	6.430897	5.849455	5.973189	6.247354	6.277106	6.312485	6.681522	6.232609	6.153997	7.018379	6.959468	8.713294
ZMYM4	202050_s_at	6.169641	6.081142	5.97606	5.899423	5.42783	5.201088	6.817787	5.2308	5.934665	6.27674	5.716412	5.309713	7.894616
ZMYM4	202051_s_at	7.45522	7.253495	7.526586	7.116206	6.876729	6.554349	7.444184	6.752846	7.19288	7.299392	7.003251	6.895068	9.119557
RAI14	202052_s_at	9.102632	9.084886	8.974361	9.152377	8.851691	8.833322	8.248062	8.001039	8.04316	7.692716	8.527617	8.179537	9.704754
ALDH3A2	202053_s_at	10.15026	9.980896	10.2026	10.10697	9.503422	8.92117	7.755082	6.839741	7.853052	7.657795	6.82502	6.627023	7.548485
ALDH3A2	202054_s_at	11.80483	11.70851	11.2787	11.43924	11.64939	11.37718	9.292562	9.624694	9.359095	8.960738	9.094832	9.354734	8.342019
KPNA1	202055_at	7.337891	7.165909	7.558697	7.476019	6.634177	6.073949	7.839372	6.995729	7.755822	7.718714	7.032101	6.741833	7.498515

KPNA1	202056_at	7.280987	7.075443	7.449146	7.209182	6.577253	6.162954	7.377306	7.073844	7.249245	7.22593	6.881333	6.906199	7.726254
KPNA1	202057_at	6.843202	6.57119	6.45994	6.885008	6.068781	6.099395	5.921334	6.557014	6.365736	6.424698	6.20114	6.10467	6.017232
KPNA1	202058_s_at	4.191481	3.626126	5.40963	5.008385	2.81157	2.949663	4.203088	3.183364	5.022246	4.398939	3.378811	3.239911	4.743492
KPNA1	202059_s_at	8.111681	7.962085	8.180625	8.385232	7.365311	7.086308	7.407482	7.367747	7.611208	7.606096	7.062766	7.548412	5.749055
CTR9	202060_at	9.218935	9.039858	9.277585	9.116842	8.867043	8.309574	9.820925	9.32527	9.854089	9.901203	9.142383	9.433832	9.200079
SEL1L	202061_s_at	9.126114	9.151097	10.03724	9.700364	8.910275	8.025275	8.879035	8.000992	9.27484	8.88124	7.876621	7.994841	8.803255
SEL1L	202062_s_at	4.098078	4.126259	5.044284	4.434325	2.910448	3.96489	4.027657	2.579459	4.517077	3.991767	2.753763	2.872268	5.63865
SEL1L	202063_s_at	2.960938	2.936858	3.507307	3.430611	3.027171	2.767109	2.823186	2.789374	2.976333	2.923077	2.85512	2.933314	2.300571
SEL1L	202064_s_at	5.692625	5.874182	6.245763	6.403058	5.716504	5.862778	5.949427	5.556207	5.786033	5.837589	5.679004	5.878857	4.406145
PPFIA1	202065_s_at	4.26598	4.177225	4.160077	4.016391	4.135175	4.749379	4.180633	3.767612	3.991289	4.16823	4.166139	4.168955	4.458999
PPFIA1	202066_at	9.315389	9.250929	8.997128	8.984947	8.572613	8.481591	8.462072	8.385331	8.605377	8.555123	8.490313	8.358635	8.958499
LDLR	202067_s_at	4.758446	5.070073	6.414169	5.873051	4.35549	3.905064	5.307042	3.294333	4.620324	5.360017	3.37766	3.896959	5.141053
LDLR	202068_s_at	8.998351	9.064788	10.45267	10.34014	8.632834	8.263862	9.093188	7.772349	8.809562	8.957258	8.257144	8.265971	9.111279
IDH3A	202069_s_at	5.643018	5.08088	6.205095	6.072221	5.783904	5.197234	5.909102	5.223336	6.344988	5.914341	5.244095	5.55776	6.888342
IDH3A	202070_s_at	5.629163	5.621464	5.984417	5.607435	5.531203	5.252996	5.640451	5.490826	5.770447	5.684353	5.369413	5.608463	5.372602
SDC4	202071_at	10.8539	10.68371	11.48815	11.45056	10.7654	10.32017	8.413176	8.856156	8.440125	8.335051	8.937346	8.795729	6.628616
HNRNPL	202072_at	6.628592	6.477186	6.733754	6.481106	6.819366	6.35732	7.637381	6.770461	8.036861	8.33724	6.881636	7.269099	6.71102
OPTN	202073_at	8.475644	8.392526	7.490565	7.363792	6.921245	6.499572	7.089684	6.394505	6.450795	7.225909	5.420993	6.824354	6.646373
OPTN	202074_s_at	9.932239	9.793747	8.785848	9.103869	8.750377	9.082932	8.522972	8.968312	8.418444	8.538061	8.86726	9.275433	6.611227
PLTP	202075_s_at	5.627127	5.108304	6.622541	6.622311	4.822962	4.229281	8.05631	7.044938	7.638106	7.534904	7.245375	7.590445	7.620255
BIRC2	202076_at	10.34193	10.35941	10.18997	10.3611	9.947798	9.799992	10.52837	10.61682	10.70828	10.68534	10.50098	10.4596	10.77578
NDUFAB1	202077_at	10.774	10.70786	10.95844	10.93434	10.71926	10.36264	11.04672	10.78615	11.13109	11.01109	10.77926	10.79825	10.7784
COPS3	202078_at	7.494773	7.543069	7.533796	7.928361	7.504603	7.2159	9.883322	9.945192	9.920267	10.29578	9.899902	10.13417	8.771851
TRAK1	202079_s_at	3.28626	4.06335	3.332384	3.21268	3.222711	3.210053	2.99071	2.818879	3.054396	3.205642	2.882204	2.799883	4.988437
TRAK1	202080_s_at	6.638829	6.667635	5.96458	5.924364	5.890221	5.720947	5.715004	4.423835	5.66087	5.928732	5.862876	4.78025	8.186245
IER2	202081_at	11.76945	11.86466	10.02418	9.954588	12.67441	12.64387	8.605332	9.091934	8.73764	8.506643	9.127096	9.202824	9.246183
SEC14L1	202082_s_at	6.147077	5.892826	6.025568	6.25739	6.438241	6.462838	6.671468	6.628686	6.404751	6.304476	7.204439	7.457739	6.324793
SEC14L1	202083_s_at	4.830274	5.210186	5.675957	6.022625	5.312157	5.525239	6.786395	7.550273	7.163116	7.574709	7.443323	7.808466	4.643541
SEC14L1	202084_s_at	6.529855	6.462606	7.428643	7.196438	6.583158	6.117525	8.590871	7.501881	8.479111	8.619638	7.796356	7.878661	8.562368
TJP2	202085_at	9.818671	9.671326	10.20672	10.0346	10.19597	9.94796	9.675185	9.019945	9.307843	9.323448	8.694687	8.995188	7.757619
MX1	202086_at	6.62175	6.388157	7.151722	7.131771	5.53621	5.918933	4.150654	3.596815	3.600472	3.665389	2.833719	4.318683	3.731423
CTSL1	202087_s_at	10.36002	10.34663	10.58922	10.73646	10.0068	9.552794	9.615548	9.163962	9.728168	9.640014	9.027322	9.414468	8.938746
SLC39A6	202088_at	9.239118	9.208974	9.174463	9.425555	8.716375	8.539302	8.977303	9.075428	8.946267	8.705922	9.638402	9.254068	11.18412
SLC39A6	202089_s_at	5.442995	5.438789	7.040043	6.352198	5.105291	4.821158	6.619801	5.354576	6.572677	6.931778	5.445514	5.424083	9.522668
TMEM54	202090_s_at	12.07558	12.06783	11.84972	12.27773	12.1106	12.12891	11.34565	11.88278	11.55661	11.62245	11.90643	11.91268	10.48284
ARL2BP	202091_at	2.562049	2.527589	2.510132	2.504716	2.550488	2.47172	2.525296	2.524692	2.520899	2.51387	2.51084	2.517471	2.597688
ARL2BP	202092_s_at	6.096311	5.527286	6.213932	6.228443	6.197148	6.135052	7.571156	7.099429	7.489835	7.407204	7.046814	7.208328	8.149041
PAF1	202093_s_at	7.110848	7.077567	6.832519	6.932085	7.6329	7.965141	6.730175	7.907339	6.771292	6.97516	8.01517	8.003235	6.331126

BIRC5	202094_at	2.234709	2.226515	2.209114	2.213434	2.235326	2.213692	5.630067	6.209472	5.691658	5.962984	5.036601	5.100172	3.909816
BIRC5	202095_s_at	4.715712	4.287048	2.006471	2.019697	2.095948	2.400015	8.654309	9.535909	8.942401	8.68282	8.821031	9.447346	7.160013
TSPO	202096_s_at	5.804682	5.809676	6.711375	6.946229	7.16757	7.524033	4.655606	5.186625	5.077761	4.570444	5.7334	5.441675	6.868496
NUP153	202097_at	7.892	7.923613	8.401871	8.100522	7.581002	7.206842	9.82303	8.68437	9.848711	9.637982	8.424965	8.777524	9.05008
PRMT2	202098_s_at	5.041732	5.035205	4.940705	5.012481	5.038413	4.897084	5.370681	5.034131	5.344102	5.702977	5.839821	5.241023	8.858505
DGCR2	202099_s_at	2.877319	2.621131	2.517129	2.606679	2.631068	2.900575	2.572547	2.619119	2.5598	2.602662	2.508492	2.610527	3.44531
RALB	202100_at	7.050965	7.16996	7.273217	7.307116	6.90937	6.66819	7.791703	6.821257	7.580345	7.849008	6.928696	7.639978	8.829663
RALB	202101_s_at	4.224626	3.975157	4.836344	4.088788	3.765412	4.244063	5.081899	4.26628	4.250826	5.009676	4.074727	4.176885	7.034413
BRD4	202102_s_at	9.024747	8.912672	8.472201	8.674005	9.348392	9.565308	8.669153	9.400681	8.477321	8.49656	9.66914	9.627836	7.609086
BRD4	202103_at	5.859172	6.17715	6.062397	5.955244	5.380097	5.220412	6.700039	6.27422	6.959657	6.883267	5.88415	6.092885	5.301684
SPG7	202104_s_at	8.00396	7.910041	7.870388	7.589526	7.11705	6.599234	7.545931	6.571675	7.701078	7.449431	7.195614	6.802537	7.720977
IGBP1	202105_at	6.785198	6.898356	7.032244	7.157104	6.993917	7.02819	7.259871	7.0544	7.116837	7.423669	7.630069	7.306355	8.49033
GOLGA3	202106_at	8.197758	8.330805	7.959168	8.230259	8.458895	8.148589	8.632584	9.064481	8.613944	8.73496	9.296232	9.018506	8.8598
MCM2	202107_s_at	5.156819	4.435325	3.14347	3.148221	3.052852	3.255594	9.601483	8.967808	9.697885	9.728959	8.727394	9.289804	8.03109
PEPD	202108_at	8.037302	8.030041	8.886214	9.080221	7.940926	7.474578	7.020238	6.5324	6.771063	6.652601	6.497873	6.525867	6.408743
ARFIP2	202109_at	6.692853	6.579699	6.714137	6.762337	6.674658	6.550713	7.27989	6.940466	7.002109	6.861548	7.308013	7.10164	6.523104
COX7B	202110_at	12.08226	12.05851	12.01566	12.1218	11.98093	12.01826	12.46881	12.11584	12.38925	12.3492	12.03544	12.08943	11.93589
SLC4A2	202111_at	7.63198	7.954949	6.837347	6.752454	7.375519	6.827597	6.799609	6.708783	6.778938	6.716575	7.198968	6.749415	6.171599
VWF	202112_at	4.896937	5.198197	4.949131	5.026815	7.321775	7.768699	4.510765	4.511986	4.502742	4.489652	5.789063	4.505669	6.82839
SNX2	202113_s_at	7.489061	7.419118	8.879856	8.337345	7.021319	6.17937	9.538891	7.65848	9.454959	9.448379	7.513141	7.625779	9.656075
SNX2	202114_at	8.418165	8.248713	8.45597	8.432068	8.197454	8.044873	8.634217	8.419475	8.817845	8.861898	8.354484	8.488261	8.427079
NOC2L	202115_s_at	5.303	5.588402	4.784135	5.122837	5.360635	5.529324	6.022817	6.448797	6.416588	5.914102	6.517003	6.242254	5.2904
DPF2	202116_at	6.813918	6.740851	6.642384	6.780573	7.072935	7.074291	7.949381	7.592795	7.867362	7.944202	7.662868	7.582928	8.518747
ARHGAP1	202117_at	9.914599	9.647662	9.192452	9.257526	9.911533	9.798133	9.297846	9.088483	9.069294	9.062086	9.31729	9.245085	10.18158
CPNE3	202118_s_at	7.676722	7.512767	8.345561	8.009186	6.879855	6.523636	7.411553	6.470235	6.405794	6.819063	6.582892	6.7034	8.742737
CPNE3	202119_s_at	9.199709	9.237948	10.10286	9.734642	8.717853	8.303193	8.87509	7.69318	9.06274	8.8017	8.080313	7.88966	9.819254
AP2S1	202120_x_at	8.982453	8.98109	8.802535	9.054085	8.695155	8.833549	9.592685	9.66998	9.543396	9.616343	9.626745	9.747547	8.844238
CHMP2A	202121_s_at	10.29765	10.40248	10.86083	11.0048	10.95499	10.84545	10.34341	10.46608	10.38469	10.49163	10.36583	10.44488	9.657122
M6PRBP1	202122_s_at	6.760406	7.2	6.033647	5.933967	6.211887	6.926084	6.918284	7.660654	6.750069	6.978352	7.940702	7.746568	7.418662
ABL1	202123_s_at	5.58614	5.376203	5.816758	5.457112	5.360023	4.936861	5.85435	5.052134	6.211143	6.149484	5.675047	5.363979	8.78234
TRAK2	202124_s_at	7.824529	6.684175	7.81564	7.971917	7.641297	7.286931	11.74723	11.64153	11.72721	11.71402	11.05311	11.31142	8.912836
TRAK2	202125_s_at	7.246946	7.229755	7.042493	6.971832	7.127176	7.048636	10.8997	10.82182	11.14099	11.27806	10.32455	10.14712	7.794762
PRPF4B	202126_at	8.45203	8.583896	8.832355	8.498827	8.332551	8.312195	9.602483	9.498545	9.684301	9.491663	9.433033	9.595573	9.659428
PRPF4B	202127_at	7.574464	7.536099	7.628573	7.643151	6.852346	6.471395	8.451319	8.01984	8.727675	8.525605	7.782173	7.970514	8.114917
KIAA0317	202128_at	5.281545	5.326513	5.692665	4.997684	4.971902	3.957452	7.522557	6.480247	7.669991	7.59387	6.618317	6.239831	6.817763
RIOK3	202129_s_at	5.151682	5.345365	6.560181	6.150316	4.140768	4.518579	7.83078	5.934736	7.977863	8.194599	5.660684	5.804705	6.817134
RIOK3	202130_at	10.184	10.0469	10.40823	10.37982	9.899597	9.705285	10.76283	10.62623	10.78707	10.99783	10.01843	10.23368	9.388117
RIOK3	202131_s_at	6.895939	6.534808	7.595121	7.282447	6.526738	6.160875	7.857179	6.241961	8.496595	8.420973	6.640604	6.927548	6.956744

WWTR1	202132_at	5.76847	5.410731	6.352738	6.449891	6.415379	6.574009	4.567896	5.223156	4.385669	4.418822	6.157899	5.185116	6.490614
WWTR1	202133_at	9.303845	9.145448	9.506773	9.710366	9.455373	9.401124	6.845211	6.52179	6.789917	6.612752	9.494071	7.10748	11.24672
WWTR1	202134_s_at	2.449755	2.318803	2.271785	2.298277	2.331562	2.289724	2.244623	2.302601	2.269305	2.280647	2.302811	2.296183	4.052235
ACTR1B	202135_s_at	7.43951	7.447929	7.355368	7.240333	7.149349	7.234972	7.304752	7.227011	7.257412	7.191411	7.105954	7.200842	7.185495
ZMYND11	202136_at	9.699136	9.721405	9.700525	9.767084	9.851074	9.326513	9.4862	8.996946	9.535332	9.379567	9.437652	9.282134	10.72613
ZMYND11	202137_s_at	3.512512	3.468827	5.1542	3.490026	3.495949	3.399532	3.669768	3.355053	5.009622	4.453805	3.464658	3.475153	7.437147
JTV1	202138_x_at	8.641224	8.549443	8.504656	8.637414	8.236723	8.424135	8.680478	8.367656	8.633172	8.797502	8.424413	8.612423	8.078246
AKR7A2	202139_at	8.254341	8.213034	8.73077	8.751168	8.430311	7.774455	8.349199	7.978313	8.61336	8.284485	8.03664	8.185588	8.405762
CLK3	202140_s_at	5.255032	5.372941	5.173555	4.750379	5.309754	4.915106	6.97077	6.961685	7.001126	7.243809	6.223125	6.328885	5.024717
COPS8	202141_s_at	8.429326	8.252021	8.464856	8.671587	8.213265	7.977644	8.970306	9.032134	8.883036	9.091536	9.373006	9.024612	10.14049
COPS8	202142_at	5.697147	5.539417	6.558556	6.589696	4.607191	4.548571	6.96672	6.12268	7.176062	7.577426	6.342843	6.12336	8.356105
COPS8	202143_s_at	4.875733	4.871878	5.914554	5.064607	4.496623	4.140017	6.431663	4.656785	7.172946	7.274639	4.877306	4.927113	8.863323
ADSL	202144_s_at	8.485277	8.567901	8.287066	8.548345	8.210372	8.286079	9.818206	9.904371	9.909737	9.88364	10.07428	10.0509	9.253923
LY6E	202145_at	7.920847	7.926733	9.352976	9.396766	8.381331	8.299333	3.379018	4.375738	3.910854	4.035972	5.479376	4.490578	6.245601
IFRD1	202146_at	9.599887	9.505814	9.742166	9.962625	9.154907	8.983529	8.395916	8.634918	8.356942	8.361419	8.20593	8.659732	8.862428
IFRD1	202147_s_at	5.98112	5.846023	7.196172	6.844006	5.389104	4.906766	5.870303	5.104488	5.507669	5.938542	4.47485	4.861978	6.557956
PYCR1	202148_s_at	5.055044	5.457448	4.781519	5.060282	5.157601	5.824976	7.178458	6.513884	6.24412	6.964186	7.265051	6.808842	7.805971
NEDD9	202149_at	6.355746	6.417226	7.553079	7.605269	6.16953	6.284837	4.657872	5.858704	6.35838	6.863592	5.88498	5.047727	8.135795
NEDD9	202150_s_at	3.590349	3.770079	5.060031	4.85012	3.926897	3.412949	3.581184	4.030158	3.84539	4.727209	3.285693	3.483953	5.119099
UBAC1	202151_s_at	7.008063	6.794859	6.982878	7.236476	6.841887	6.431405	11.0399	10.76722	11.02753	11.19215	10.38463	10.80515	6.70516
USF2	202152_x_at	8.640747	8.788536	8.116912	8.203631	8.537141	8.415072	7.836351	7.773854	7.903766	7.800894	7.991236	8.00237	6.78128
NUP62	202153_s_at	5.643631	5.642869	5.048404	5.263621	5.697501	5.566889	7.064391	7.041284	6.915706	7.083543	6.982562	7.140315	7.169475
TUBB3	202154_x_at	8.962982	8.943372	8.294566	8.00672	8.313186	8.209961	9.927412	9.980978	9.930967	10.01868	9.911062	10.15281	12.19292
NUP214	202155_s_at	6.392792	6.356983	6.182119	6.342011	6.385611	6.454089	6.422265	7.038939	6.619534	6.339843	6.828029	7.005804	5.39436
CUGBP2	202156_s_at	3.698756	3.520306	3.591439	3.574718	3.951674	3.603331	3.319575	3.533611	3.541568	3.53881	4.733936	3.675384	6.932258
CUGBP2	202157_s_at	6.691623	6.503216	7.037957	6.735303	7.252197	6.901742	6.596195	6.62262	6.544251	6.222172	8.848623	6.923837	10.3708
CUGBP2	202158_s_at	4.744917	4.673648	4.408793	4.351796	5.264345	5.142181	4.546868	4.68912	4.135434	3.950914	6.071259	4.782336	9.010289
FARSA	202159_at	5.799741	5.605535	5.167709	5.418969	5.807745	6.083959	6.503064	6.847044	6.775492	6.637761	7.258291	7.050132	5.869634
CREBBP	202160_at	8.715091	8.658837	8.537272	8.597933	8.658353	8.525829	8.644253	8.4677	8.691028	8.729197	8.834847	8.574231	9.137672
PKN1	202161_at	6.173641	6.028028	5.267876	5.645164	5.621161	6.264494	5.591547	5.681284	5.807767	5.702849	6.096015	6.500448	5.457116
CNOT8	202162_s_at	5.117691	5.023405	5.740798	5.026959	4.382731	4.857165	7.10527	5.454319	6.957865	7.401378	5.296586	5.912801	7.458276
CNOT8	202163_s_at	7.03	6.940004	6.784538	7.049551	6.855309	6.695996	7.419318	7.537427	7.729407	7.388104	7.242154	7.265649	7.818606
CNOT8	202164_s_at	5.148346	5.315292	6.425666	5.583247	4.313582	4.584911	7.210941	4.90188	7.142493	7.205876	5.124112	5.020275	8.468198
PPP1R2	202165_at	6.546978	6.418036	7.059052	7.283505	6.409583	5.550064	6.587464	6.088755	6.60848	6.587605	6.277107	6.378237	7.762853
PPP1R2	202166_s_at	5.949097	6.114542	6.949971	6.681589	6.027989	4.817706	7.324327	6.004123	7.916188	7.577894	5.499075	5.835248	8.185061
MMS19	202167_s_at	6.837149	6.889969	6.535028	6.697278	6.476758	6.316529	7.643032	6.973414	7.694142	7.463156	7.1024	7.447357	7.308882
TAF9	202168_at	7.593806	7.558762	7.984853	7.819773	6.810845	5.770691	9.986096	8.7249	9.785172	9.962375	8.399128	8.828254	8.845515
AASDHPPT	202169_s_at	8.848781	8.592804	9.62971	9.265747	8.358523	7.672016	9.839288	8.9105	10.07256	9.889791	8.827809	8.846558	11.06613

AASDHPPT	202170_s_at	7.181586	6.815226	7.645058	7.806616	7.397836	7.307035	7.506839	7.526353	7.939666	8.179235	7.935986	7.701514	8.30907
VEZF1	202171_at	6.900416	6.523012	7.512959	7.241645	6.981588	7.115137	8.476463	7.975229	8.33499	8.361827	7.669976	7.956593	9.19029
VEZF1	202172_at	7.793496	7.65383	7.546114	7.56243	7.727281	7.805316	7.596969	7.68943	7.952932	7.975841	7.79988	7.876415	8.354341
VEZF1	202173_s_at	6.967011	7.048492	7.548349	6.964956	6.921842	6.319154	7.876914	7.012019	7.784578	7.627474	7.064251	7.346915	8.516495
PCM1	202174_s_at	8.77673	8.874538	8.869239	8.751405	7.946435	7.578284	9.694403	8.696388	9.630757	9.699584	8.707952	8.80438	9.662224
CHPF	202175_at	5.301994	5.535341	4.941745	5.059331	5.063279	5.079907	4.793104	5.056965	4.647079	5.055598	5.066324	4.844202	4.941345
ERCC3	202176_at	5.527433	5.572945	5.715115	5.778041	5.395072	5.327414	6.299998	5.787937	6.148967	5.957487	5.844435	5.858836	6.542069
GAS6	202177_at	4.655102	4.602363	3.954338	3.974945	4.428468	3.961578	3.250455	3.261308	3.024348	3.238121	3.953162	3.091234	3.956338
PRKCZ	202178_at	6.42369	6.392619	5.668241	6.03626	6.521935	6.703181	5.933148	6.529014	5.498595	5.712282	6.670817	6.52301	5.389196
BLMH	202179_at	4.067581	4.109882	4.342539	4.261807	4.174242	3.612474	5.511309	4.760544	5.572367	5.417269	5.080687	5.245437	6.115665
MVP	202180_s_at	6.64282	6.632794	5.019874	4.966173	6.826929	7.082724	2.884676	3.787072	3.418212	3.511863	3.391933	4.080275	3.486277
KIAA0247	202181_at	7.889001	7.567752	8.348916	7.998539	7.101836	6.862099	7.30159	6.368111	7.193929	7.305547	6.695557	6.225655	7.744635
GCN5L2	202182_at	7.029444	6.991173	6.770997	6.779179	7.427686	7.654375	6.231522	6.541694	6.150315	6.556457	6.865805	6.552995	6.98964
KIF22	202183_s_at	6.975691	7.047065	7.11904	7.34176	7.660793	7.694591	7.912305	8.580074	8.447977	8.105336	8.317023	8.654719	6.117429
NUP133	202184_s_at	8.523423	8.357177	8.078206	8.330101	8.149659	7.986691	9.403783	9.081427	9.347745	9.414149	9.297228	9.314845	9.09043
PLOD3	202185_at	8.4382	8.427079	7.85198	7.759868	7.887311	7.94827	7.617746	7.607485	7.763794	7.854941	7.823187	7.659174	7.889583
PPP2R5A	202186_x_at	2.275187	2.245064	2.220909	2.226941	2.254448	2.223483	2.236223	2.247919	2.231432	2.221708	2.228592	2.261491	2.134753
PPP2R5A	202187_s_at	8.025461	8.142922	8.755381	8.693351	8.506429	7.951821	7.041825	6.668122	7.326981	7.280543	6.718183	6.881285	5.908875
NUP93	202188_at	3.67812	3.682425	3.620931	4.020483	3.038982	3.685238	7.051167	6.254709	6.986418	6.981127	6.709249	6.571351	7.053118
PTBP1	202189_x_at	9.329902	9.294994	8.84303	8.945286	9.128133	9.241961	10.49523	10.52617	10.28134	10.33605	10.30237	10.42129	9.561297
CSTF1	202190_at	4.261509	4.108237	4.876807	5.101501	3.530397	3.823566	5.631903	4.493301	5.574294	5.434017	4.235465	4.784038	5.390584
GAS7	202191_s_at	5.055816	4.991439	4.931819	5.031395	5.913174	7.021472	4.15426	5.26699	3.879457	4.005665	7.760099	5.391478	7.84428
GAS7	202192_s_at	3.15427	3.111575	2.926515	3.097165	3.145951	3.868045	2.995385	3.109556	3.069024	3.095879	5.465025	3.67456	6.412055
LIMK2	202193_at	6.392815	6.390527	5.122675	4.911769	5.832212	6.252898	6.64496	6.772392	6.465387	6.380082	6.787564	6.588745	7.769099
TMED5	202194_at	10.44303	10.33987	10.63458	10.54352	9.98077	9.512167	10.89828	9.979384	11.17006	11.0477	9.858167	9.819861	9.76866
TMED5	202195_s_at	9.088403	9.19989	9.192189	9.41397	8.996997	8.556256	7.644041	7.652698	7.686662	7.495895	6.994657	7.284727	6.741642
DKK3	202196_s_at	3.934601	3.786635	3.802157	3.768969	4.02312	3.75614	3.72434	3.58424	3.713996	3.829375	5.340254	3.926959	8.05886
MTMR3	202197_at	6.240425	6.246412	6.14721	6.192665	5.730911	6.381632	7.402293	7.024066	7.52192	7.639298	6.537351	7.134553	7.047718
MTMR3	202198_s_at	2.958854	3.016925	2.930285	2.952923	2.958923	2.955698	3.115397	2.959079	3.220191	3.030838	2.921475	2.934526	2.830372
SRPK1	202199_s_at	3.383092	3.57772	3.561671	3.548196	3.258947	3.563656	4.523618	3.596058	5.063702	4.492275	3.572175	3.803077	4.441336
SRPK1	202200_s_at	7.811541	7.423738	7.997358	8.132801	7.286729	7.33269	9.847144	9.736742	10.10045	9.817347	9.408543	9.55129	8.371043
BLVRB	202201_at	9.040916	9.151132	10.23953	10.49699	10.16688	10.1015	12.80485	12.72123	12.78521	13.11016	12.33647	12.78378	7.369587
LAMA4	202202_s_at	3.979268	4.195795	4.613263	4.969473	4.664331	5.092377	4.77708	5.227026	4.398761	4.549789	7.569577	4.610384	9.435445
AMFR	202203_s_at	8.008491	7.62268	8.117099	7.674971	6.583272	6.167527	8.310713	7.079247	6.237188	8.740833	6.157384	5.001306	7.339086
AMFR	202204_s_at	6.275961	6.145523	6.251295	6.236413	5.396686	5.11807	5.51912	4.920013	4.914896	6.023793	4.86752	5.533348	4.297302
VASP	202205_at	6.602366	6.683055	6.667371	6.498272	7.210936	7.471519	7.175183	7.098652	6.872245	7.012423	7.272639	7.846955	6.231501
ARL4C	202206_at	3.855478	3.659346	3.984325	3.658471	3.615523	3.966804	3.269724	3.265063	3.265475	3.259319	3.204621	3.257994	6.487294
ARL4C	202207_at	5.673513	5.687189	6.600589	6.237833	6.008636	5.750111	3.292901	2.570339	4.059084	3.740051	3.432029	2.908112	8.212689

ARL4C	202208_s_at	5.411448	5.541263	5.810993	5.548449	5.760922	6.637826	4.983545	5.147662	3.714574	5.246327	5.203266	5.192093	5.396463
LSM3	202209_at	8.651225	8.524683	9.216993	9.475355	8.890471	8.530943	9.882863	9.316325	10.0101	9.994423	9.261294	9.425182	9.501639
GSK3A	202210_x_at	5.413567	5.34488	5.021519	5.332654	5.455344	5.345601	5.751657	6.449064	5.403063	5.457015	5.406347	6.071802	5.301818
ARFGAP3	202211_at	8.085721	7.952393	8.886599	8.519946	7.799899	7.428005	9.204089	8.197869	9.269515	8.907888	7.979748	8.015183	9.285817
PES1	202212_at	7.803952	8.035207	7.184002	7.462238	7.669499	8.030518	7.965091	8.940533	8.170923	8.373118	8.904024	9.17417	7.058826
CUL4B	202213_s_at	6.316766	5.73103	6.11192	6.284175	5.75504	5.878849	6.610926	6.345343	7.107316	6.567175	5.737366	6.390651	8.174701
CUL4B	202214_s_at	7.335104	7.295512	7.631154	7.459676	7.070911	6.843361	8.371351	7.466134	8.245459	8.125604	7.372764	7.461271	8.718811
NFYC	202215_s_at	7.467284	7.440974	6.929162	7.250667	7.56601	7.331144	7.563	7.287894	7.628899	7.735184	7.540684	6.647077	7.82997
NFYC	202216_x_at	3.536995	3.127014	4.475286	3.634556	3.485659	3.441469	6.090585	3.525153	5.753783	6.281877	3.647603	3.494497	5.749891
C21orf33	202217_at	10.99833	11.10727	10.7215	10.82443	10.9931	10.91044	10.23231	10.48301	10.3929	10.35124	10.54052	10.52254	9.298844
FADS2	202218_s_at	7.129648	6.662373	5.87985	5.850737	5.655838	4.649281	7.060798	6.435381	6.894674	7.244049	7.933785	7.818765	5.194687
SLC6A8	202219_at	3.618696	4.050337	3.173378	2.560678	3.21197	4.314134	9.502924	10.4052	9.224072	9.50872	9.868145	10.21853	6.113719
KIAA0907	202220_at	8.004124	7.985914	7.77358	7.637495	7.486941	7.506474	8.529671	8.247445	8.662985	8.770597	8.302317	8.318373	9.17609
EP300	202221_s_at	8.111375	7.983505	7.559259	7.61288	7.373355	7.277195	8.640972	8.686511	8.640058	8.778937	7.932874	8.386928	7.406939
DES	202222_s_at	2.575389	2.563084	2.466024	2.533587	2.562274	2.545747	2.548902	2.560266	2.655768	2.726322	7.51752	2.553588	8.203142
STT3A	202223_at	6.346836	5.880198	7.099928	6.840335	6.076632	5.993046	8.474895	8.004362	8.298936	7.917421	7.32417	7.891606	8.45376
CRK	202224_at	8.080573	8.111042	8.423938	8.388144	7.847036	7.431845	7.762048	7.327357	7.730835	7.689073	7.391151	7.802421	7.588806
CRK	202226_s_at	4.374536	3.765972	5.813424	4.776405	3.755288	3.249113	5.671825	3.741733	5.627839	5.435312	2.578048	3.272179	6.407339
BRD8	202227_s_at	8.602416	8.603199	8.256391	8.203857	8.857014	8.796297	9.103263	9.367618	9.17212	9.302039	9.522956	9.195163	9.183434
NPTN	202228_s_at	9.957563	9.874263	10.28477	9.918585	9.368793	8.764186	9.866236	8.687031	9.714658	9.731404	9.160689	9.126087	11.41698
CHERP	202229_s_at	2.134867	2.115998	2.088483	2.096704	2.119557	2.103407	2.105499	2.114067	2.099102	2.092784	2.099219	2.109713	2.023383
CHERP	202230_s_at	10.08714	10.04471	9.662284	9.775138	9.714658	9.710608	9.499271	9.598308	9.483218	9.497148	9.861297	9.69594	9.36339
EIF3M	202231_at	11.84608	11.82291	11.52211	11.72278	11.65862	11.50216	12.47781	12.46326	12.28277	12.5145	12.40383	12.38761	12.27842
EIF3M	202232_s_at	10.06159	9.960169	10.20541	10.17158	9.652801	9.132437	11.18322	10.233	11.23996	11.28175	10.32033	10.169	11.73856
UQCRH	202233_s_at	10.47078	10.41151	10.57252	10.71227	10.20127	10.07679	11.71999	11.33233	11.69071	11.67371	11.36253	11.37901	12.24778
SLC16A1	202234_s_at	7.537788	7.586122	8.075607	8.199661	7.759376	8.100102	5.940781	6.63862	6.507037	6.269046	6.81793	6.662768	5.487237
SLC16A1	202235_at	6.365828	6.463661	6.79751	7.166649	6.958095	6.637541	4.449819	4.939359	5.471729	4.918166	5.324556	5.291604	4.256721
SLC16A1	202236_s_at	7.361705	7.390145	9.040222	8.305677	6.622012	5.732054	8.380347	6.169947	8.566253	8.593033	5.983714	5.966711	8.37171
NNMT	202237_at	13.79476	13.82972	14.61195	14.72807	14.45362	14.70906	3.203117	3.340784	2.879811	3.379992	5.697679	3.692827	8.245188
NNMT	202238_s_at	11.66715	11.77645	13.53108	13.42199	12.75182	12.60861	2.977471	2.966736	3.091832	2.99537	3.007263	3.242766	7.031563
PARP4	202239_at	7.112914	7.132246	7.520356	7.491382	6.654916	5.909681	7.204314	6.258541	7.123212	6.922916	6.477384	6.209463	6.757833
PLK1	202240_at	1.866586	1.854724	1.835173	1.839412	1.852663	1.837966	6.170962	4.766866	6.17222	6.303569	4.580392	5.509402	3.473299
TRIB1	202241_at	11.25124	11.24609	11.42408	11.52409	11.50414	11.57319	7.571699	7.454862	7.971737	8.024588	7.696984	7.831702	7.436512
TSPAN7	202242_at	6.890671	6.465272	8.32614	8.011056	5.799592	6.338156	8.795871	7.749334	8.293922	8.332519	8.123088	8.009138	9.167817
PSMB4	202243_s_at	10.12811	9.981726	9.971232	10.10758	9.866621	9.296042	11.05806	10.40723	11.04268	11.02362	10.51123	10.52569	11.08574
PSMB4	202244_at	11.73938	11.55275	11.359	11.57151	11.59194	11.39009	12.16253	11.96059	12.15159	12.25232	12.06114	12.11288	11.699
LSS	202245_at	9.852658	9.893874	9.090817	9.232013	10.31729	10.16206	10.71619	10.59506	10.65638	10.69532	10.13546	10.61934	8.466374
CDK4	202246_s_at	7.408807	7.304496	7.192361	7.262376	7.229186	6.695582	9.712081	8.731189	9.799902	9.501223	8.807147	9.044317	9.777969

MTA1	202247_s_at	3.390933	3.310508	3.351159	3.368294	3.287716	3.360133	3.860555	3.378001	3.362515	3.669347	3.355782	3.368854	3.652998
E2F4	202248_at	3.719015	3.69133	3.656722	3.687853	3.706857	3.672784	4.640403	4.752809	4.452045	5.379099	3.876453	4.62503	3.650212
WDR42A	202249_s_at	7.423216	7.407355	7.489611	7.42762	7.169374	7.201289	6.719345	6.774639	6.778389	7.279553	6.548881	6.929667	5.920433
WDR42A	202250_s_at	8.174254	8.352929	7.919644	7.714801	8.154155	7.909364	7.29471	7.628373	7.294383	7.28463	7.279946	7.282422	5.600208
PRPF3	202251_at	8.748406	8.641333	8.287002	8.30844	8.493433	8.678691	8.032278	8.409744	8.321778	8.326489	8.71403	8.593069	7.776232
RAB13	202252_at	10.59474	10.6852	10.34138	10.58037	11.20605	11.51093	9.989154	10.78003	10.07241	10.23863	10.97016	11.05613	10.3363
DNM2	202253_s_at	4.785265	4.616336	5.068569	4.466294	4.367362	4.254805	6.403748	5.747734	6.403879	6.25191	4.817892	5.833761	4.43552
SIPA1L1	202254_at	2.891442	2.880604	3.068215	2.867335	3.371889	3.027525	2.951727	2.788511	2.782499	2.889835	2.346756	2.654077	4.713468
SIPA1L1	202255_s_at	6.39141	6.730126	6.626316	7.043671	7.185382	7.131398	6.181572	6.468869	6.142068	6.121965	6.293735	6.472672	6.322198
CD2BP2	202256_at	6.257472	6.574656	5.267694	5.370436	6.390671	6.733037	6.468053	7.311563	6.286547	6.485081	7.134701	7.239806	7.168962
CD2BP2	202257_s_at	6.46749	6.505476	5.977884	6.344053	6.627175	6.721404	7.142001	7.089945	6.993095	6.634364	7.498151	7.51115	6.257844
PFAAP5	202258_s_at	10.48097	10.42313	10.02899	10.21742	9.949439	9.512386	9.903368	9.896294	10.03368	9.999004	9.733128	9.610035	9.938878
PFAAP5	202259_s_at	6.5418	6.410165	6.558299	6.486476	6.330307	6.236433	5.66982	5.347934	6.217072	6.278093	5.769595	5.623111	6.988237
STXBP1	202260_s_at	4.688635	4.378146	4.654907	4.638952	4.984091	4.902731	4.779981	4.7037	4.487161	4.669932	4.611201	4.572697	9.082165
VPS72	202261_at	5.747176	5.797491	6.002009	6.140564	5.925447	4.807904	7.210838	6.154812	7.108687	7.236495	6.48399	6.777219	7.917011
DDAH2	202262_x_at	6.041344	5.81862	5.354339	5.270347	5.461669	5.144709	6.297102	6.373195	6.489215	6.384723	7.296725	6.619054	8.063936
CYB5R1	202263_at	7.958244	8.097017	7.963169	8.122647	7.660275	7.729146	8.456062	8.363685	8.467193	8.731359	8.556665	8.328225	7.829341
TOMM40	202264_s_at	6.74792	6.979738	6.500736	6.938986	7.000101	7.327095	7.753356	7.695971	7.698544	7.714539	7.804078	8.025833	6.324312
BMI1	202265_at	9.733439	9.69085	10.11889	10.19745	9.878561	9.844494	9.864457	9.570376	9.781463	9.691525	9.410382	9.360544	10.35849
TTRAP	202266_at	9.318751	9.209787	9.124122	9.135504	8.76323	8.084989	9.794083	9.050161	10.02455	10.01758	8.90127	9.124251	9.024239
LAMC2	202267_at	2.463233	2.443601	2.414364	2.720047	2.451725	3.033672	2.430926	2.442875	2.432981	2.420114	2.418369	2.437858	2.428132
APPBP1	202268_s_at	8.357973	8.233688	8.541239	8.698091	8.160217	8.040531	9.321301	8.890973	9.489522	9.687423	8.892071	9.058942	9.641552
GBP1	202269_x_at	8.115034	8.169509	8.980492	9.386034	9.053896	9.062379	3.266186	3.00645	3.990984	3.208907	3.839855	3.105152	4.34338
GBP1	202270_at	8.012782	7.739786	9.196288	9.289738	8.361528	8.090056	3.29491	4.340227	4.217187	3.350263	4.308322	3.591282	4.755151
FBXO28	202271_at	8.583339	8.606375	9.275307	9.327814	8.41604	8.011749	7.217338	7.014275	7.629867	7.441785	6.749213	7.067891	7.388963
FBXO28	202272_s_at	3.79676	4.04147	5.160615	4.826311	3.816336	3.777824	4.706456	4.196425	5.264739	5.165992	3.942493	4.061316	5.639178
PDGFRB	202273_at	5.377493	5.636834	5.228329	5.23567	5.280037	5.225342	5.245544	5.253248	4.690747	4.776245	5.973462	5.247227	6.916912
ACTG2	202274_at	2.601786	2.601406	2.618797	2.596261	2.585449	2.558476	2.579272	2.572408	2.56382	2.528214	3.249455	2.612404	9.493694
G6PD	202275_at	3.653637	3.426598	3.616665	3.052625	3.465616	3.620187	4.178513	5.459435	4.453685	4.154134	4.652304	5.378107	3.568093
SHFM1	202276_at	9.52898	9.527137	9.124719	9.14871	9.201445	9.131151	9.107292	9.00291	9.026134	9.127894	9.216374	9.13788	8.971063
SPTLC1	202277_at	9.398843	9.342199	9.596902	9.392699	9.145478	8.944953	10.27155	9.995806	10.73745	10.51491	9.933458	9.942908	11.01021
SPTLC1	202278_s_at	2.591379	2.581123	2.823204	2.568803	2.561126	2.556212	2.581607	2.580582	3.495773	3.441247	2.503086	2.520984	4.762636
C14orf2	202279_at	8.777462	8.734586	8.296771	8.701412	8.777686	8.575494	9.631423	9.748432	9.413982	8.987857	10.01852	9.721643	10.44625
GAK	202281_at	7.17344	7.045792	6.152626	6.66446	7.028637	7.204119	6.087686	6.674407	6.252907	6.083038	6.923253	6.729684	5.635191
HSD17B10	202282_at	9.613296	9.618627	10.63044	10.69434	10.16022	9.949871	9.072132	9.266242	8.857034	8.715729	9.172115	9.148668	8.711868
SERPINF1	202283_at	12.42584	12.383	13.30865	12.98953	11.80441	11.29472	12.30786	11.39969	12.65151	12.24494	11.23248	11.49427	11.00542
CDKN1A	202284_s_at	8.695179	8.635938	7.863923	7.580189	8.031661	8.273689	5.153503	4.313301	4.789121	4.089242	6.522059	5.452463	6.305598
TACSTD2	202285_s_at	2.20135	2.192591	2.158814	2.173123	2.218162	2.185163	2.188313	2.188097	2.175827	2.168252	2.17413	2.181984	2.101076

TACSTD2	202286_s_at	2.798496	3.459661	3.797305	3.277807	4.656999	4.597231	2.129934	2.130776	2.124473	2.117196	2.112549	2.124624	6.195586
TACSTD2	202287_s_at	2.441155	2.411019	2.362903	2.388516	2.425028	2.390582	2.396842	2.407391	2.393463	2.380261	2.38814	2.403698	2.265503
FRAP1	202288_at	6.172954	6.138045	6.118028	6.16895	6.128476	6.124774	6.2561	6.149257	6.141334	6.177621	6.147057	6.131133	5.803911
TACC2	202289_s_at	7.55839	7.508176	7.419625	7.571506	8.443136	8.62309	6.487567	6.665912	5.764271	5.738441	8.265346	7.061062	9.195295
PDAP1	202290_at	5.068174	4.95803	5.514001	4.877917	3.849799	4.370088	5.621204	4.367292	5.841399	6.175118	5.123262	5.256103	6.856606
MGP	202291_s_at	6.40759	7.115817	8.034871	7.232461	7.817035	7.872134	2.385549	2.397936	2.381343	2.705384	7.773231	2.405272	10.91155
LYPLA2	202292_x_at	7.897769	7.965245	7.229947	7.246757	8.185248	8.239775	7.759875	8.34713	7.821424	7.874078	8.195983	8.446044	6.770716
STAG1	202293_at	5.617888	5.651987	6.049622	5.925784	5.199848	5.358945	7.209839	6.587127	7.60166	7.118655	6.574622	6.55361	7.663172
STAG1	202294_at	7.304978	7.491283	7.11731	7.52461	7.105655	7.349308	8.314059	8.206163	8.233574	8.103764	8.278031	7.859032	8.954964
CTSH	202295_s_at	10.36942	10.50094	10.81361	11.1316	10.52866	10.45978	8.561513	7.911542	8.412431	7.516907	8.55374	8.976961	6.682998
RER1	202296_s_at	8.896362	8.823996	9.556252	9.403212	8.855051	8.024129	9.5422	8.645147	9.734087	9.599701	8.715751	8.937673	8.507922
RER1	202297_s_at	9.038484	9.118908	9.168741	9.318702	9.001081	9.006569	8.916043	8.887196	8.77797	9.066276	8.914494	8.877231	7.935824
NDUFA1	202298_at	10.53873	10.51267	10.59904	10.82915	11.06548	11.00159	10.26619	10.51086	10.38863	10.25459	10.60859	10.53704	10.2351
HBXIP	202299_s_at	10.34216	10.27544	10.58156	10.47161	10.39223	10.10794	10.75593	10.56615	10.73233	10.84396	10.5176	10.63918	10.57125
HBXIP	202300_at	8.69831	8.645877	9.191679	9.159763	8.757975	8.355767	9.517751	8.933987	9.422799	9.551415	8.967463	9.035162	9.653197
RSRC2	202301_s_at	7.119116	6.911783	7.842666	7.611322	7.641249	7.769153	8.088951	7.941842	8.137657	8.154485	8.443219	8.324042	8.11903
RSRC2	202302_s_at	8.135971	8.116526	8.556827	8.756626	7.964964	7.380689	9.562863	8.752078	9.687212	9.55386	8.834645	8.865221	9.987448
SMARCA5	202303_x_at	6.085519	5.908328	6.235771	5.995599	5.880202	5.829117	8.481218	8.070086	8.80018	8.90633	7.956343	8.085009	8.483451
FNDC3A	202304_at	9.754836	9.574009	10.2775	9.959321	8.811316	7.645039	8.582496	7.420166	9.158441	9.014042	7.656997	7.220921	10.39242
FEZ2	202305_s_at	7.167349	7.306181	7.652214	7.937988	7.621455	7.559572	6.83357	7.187128	7.270827	6.741513	7.914501	7.585844	7.972486
POLR2G	202306_at	8.472384	8.562942	8.026836	8.222804	8.59901	8.560054	9.305023	9.481702	9.455629	9.459829	9.947804	9.80457	9.752257
TAP1	202307_s_at	6.600549	6.771636	6.355186	6.301243	6.567527	7.214304	3.847494	4.348874	3.773118	4.106558	3.48022	3.215495	4.002409
SREBF1	202308_at	10.23049	10.26161	7.775622	8.031012	7.335335	7.436205	8.071988	8.513593	7.867392	7.786973	8.485313	8.682316	4.65961
MTHFD1	202309_at	11.06987	11.07528	11.82923	11.95798	11.61409	11.48518	9.849655	9.399343	9.799974	9.872875	9.272642	9.241209	8.339769
COL1A1	202310_s_at	8.889179	9.052398	6.585146	6.796507	6.953878	7.665098	8.75332	7.582258	7.783762	8.389143	12.57325	8.604211	14.09893
COL1A1	202311_s_at	5.528514	5.416179	3.841279	3.498816	3.678011	4.082477	6.93823	4.996145	5.722548	5.876786	9.561714	5.556096	12.319
COL1A1	202312_s_at	2.707316	2.675992	2.606898	2.672487	2.68654	2.704953	2.678861	2.731741	2.673539	2.665927	2.762152	2.697319	2.470593
PPP2R2A	202313_at	7.392917	7.340897	8.389306	8.250924	7.393772	6.953353	8.007502	7.507118	7.774615	7.979978	7.856063	7.699462	8.249908
CYP51A1	202314_at	11.41887	11.22037	10.58899	10.7486	12.38467	11.92416	11.67293	11.97799	11.68307	11.5713	11.26231	11.79707	9.457612
BCR	202315_s_at	7.682686	7.677416	6.503138	6.803466	7.132953	7.365909	6.049191	7.058332	6.065233	6.043957	6.827776	6.566478	6.046858
UBE4B	202316_x_at	2.135939	2.119766	3.118128	2.162633	2.121212	2.100469	5.487169	2.116511	5.442554	5.304405	2.10183	2.107162	6.273105
UBE4B	202317_s_at	8.045744	8.099746	7.818239	7.686832	8.20583	7.963181	8.371546	8.688225	8.441328	8.391008	8.779243	8.729689	8.222501
SENP6	202318_s_at	8.709362	8.594778	8.628822	8.595986	8.33702	8.086293	8.713652	8.399325	8.896043	8.658127	8.468188	8.578242	9.603878
SENP6	202319_at	4.843155	4.708267	4.760683	4.73208	4.620852	4.482368	4.836443	5.246014	4.662535	4.750293	4.82764	5.10188	6.285935
GTF3C1	202320_at	5.274415	5.500014	5.47386	5.485098	5.208866	5.295106	5.553556	5.50679	5.490653	5.598085	6.09397	5.802178	6.010304
GGPS1	202321_at	5.449493	5.497417	5.352446	5.219869	5.162066	5.069314	4.451766	4.9108	5.364828	3.97047	4.731364	4.398953	6.10095
GGPS1	202322_s_at	6.530458	6.454494	6.435411	6.620039	6.522071	6.59357	6.844227	6.494248	6.481639	6.672686	6.815869	6.524367	8.277746
ACBD3	202323_s_at	5.856089	5.312766	6.640374	6.672557	6.241772	6.13007	4.514704	5.5023	4.553908	5.507647	6.18919	4.760581	5.884952

ACBD3	202324_s_at	7.940191	8.000722	8.010741	8.00798	7.876683	7.804258	8.544859	8.501629	8.532302	8.64518	8.659358	8.574396	8.962303
ATP5J	202325_s_at	13.0595	12.98223	13.31774	13.3706	12.95304	12.95592	12.72979	12.43785	12.65559	12.6502	12.51969	12.45682	12.61495
EHMT2	202326_at	5.390655	5.225469	5.249799	5.359462	5.655449	5.501388	5.596825	6.205099	5.829346	5.555403	6.828286	6.662465	7.003364
PKD1	202327_s_at	2.286704	2.269095	2.238996	2.250472	2.273403	2.251857	2.26453	2.271197	2.256367	2.248398	2.255284	2.265275	2.191567
PKD1	202328_s_at	6.072981	6.193324	5.228651	5.656655	6.366825	6.863212	4.980151	5.664123	4.46213	4.811803	6.352582	5.666469	6.149395
CSK	202329_at	7.769989	7.144241	7.072435	7.177821	7.882623	8.251854	6.474629	6.763994	6.425262	6.606283	7.420735	7.304966	6.368576
UNG	202330_s_at	8.112719	8.306256	7.588654	7.858888	7.504189	7.432906	8.76765	8.779566	8.818361	8.897488	8.410223	8.346856	8.436139
BCKDHA	202331_at	8.505787	8.401535	8.314552	8.696756	8.645393	9.106723	6.288641	6.986434	6.587122	6.491947	7.150732	7.305532	6.37827
CSNK1E	202332_at	4.664281	4.65597	4.035143	4.213838	4.822707	4.977749	3.80013	4.654932	4.02269	4.400452	5.592238	5.097894	6.284481
UBE2B	202333_s_at	9.549418	9.554145	9.661266	9.702866	9.470116	9.228492	10.13873	9.538219	10.04083	10.06764	9.622805	9.637332	9.651862
UBE2B	202334_s_at	8.497223	8.498328	8.394145	8.657931	8.354651	8.495015	7.611535	8.579186	7.772833	7.966893	7.864281	8.107095	7.206166
UBE2B	202335_s_at	3.906997	3.877273	3.615794	4.301072	3.902462	3.701173	3.732495	3.730201	3.664392	3.602547	3.627188	3.773779	3.322996
PAM	202336_s_at	7.673171	7.615	8.136345	8.093146	7.933692	7.803814	8.590527	8.469112	8.333089	8.676316	9.198105	8.344322	11.22741
PMF1	202337_at	7.504286	7.212503	7.45107	7.479419	7.461738	7.24746	7.069319	7.152092	7.315633	7.240039	7.269437	7.450358	7.165362
TK1	202338_at	3.031851	3.027391	2.728596	2.732822	2.49333	2.734319	6.844187	7.030769	7.3526	7.022005	7.410903	7.710194	5.219366
SYMPK	202339_at	5.708899	5.329152	5.247155	5.687401	5.569501	6.26789	4.768657	5.674184	4.68394	4.832256	6.016636	5.299258	4.004025
NR4A1	202340_x_at	6.677163	7.276214	7.69904	7.98809	7.686483	7.502021	4.375167	4.38595	4.293985	4.363101	4.371853	4.6668	4.286818
TRIM2	202341_s_at	5.474465	5.61631	5.249154	5.215526	5.098994	5.046276	4.49268	5.020526	4.742226	5.069598	4.696171	4.961585	7.289569
TRIM2	202342_s_at	6.729456	6.773596	7.601683	7.286372	5.943513	4.931489	6.324949	5.660919	6.290714	6.231732	5.895398	5.580461	9.428215
COX5B	202343_x_at	11.41287	11.46518	11.60693	11.63623	11.51729	11.33936	11.53521	11.18474	11.44709	11.40578	11.23588	11.47193	10.92304
HSF1	202344_at	6.197139	6.05989	5.541109	6.040881	6.176126	6.051701	6.747962	6.758958	6.372772	6.626455	6.71954	6.26123	5.639283
FABP5	202345_s_at	6.712678	6.295115	7.216269	6.761603	6.106977	7.009703	10.09882	9.702386	10.14976	10.35848	10.44861	10.01646	11.68822
HIP2	202346_at	8.48747	8.476388	8.562965	8.385515	7.742269	7.166837	8.574701	8.044454	8.817584	8.915567	7.918587	8.106008	8.364886
HIP2	202347_s_at	7.094594	7.026079	7.150256	7.011227	6.863263	6.858142	7.443017	7.197951	7.406934	7.343065	7.226152	7.53642	6.959757
TOR1A	202348_s_at	8.089557	8.185298	8.274477	7.976924	7.517082	7.216115	7.338761	7.628958	7.769693	7.728841	6.4971	7.353596	7.541095
TOR1A	202349_at	6.396634	6.319849	6.448769	6.499579	6.079698	5.528753	5.838356	5.643054	5.719139	5.767598	5.862623	5.657342	5.972315
MATN2	202350_s_at	8.620124	8.500494	9.400226	9.552227	8.638317	8.275618	7.238411	6.251793	4.45955	6.989419	9.443277	6.248272	10.89769
ITGAV	202351_at	8.78986	8.802964	9.03521	9.022775	8.704238	8.749834	8.071585	7.784937	8.357445	8.000304	8.949896	7.576392	10.6807
PSMD12	202352_s_at	7.039779	7.081499	8.17558	7.964569	7.302056	6.580318	9.115363	8.240513	9.147677	9.210584	7.940437	8.179451	8.038823
PSMD12	202353_s_at	7.789218	7.756012	8.450836	8.133541	7.200643	5.769809	8.072391	6.980259	7.880045	8.105095	6.367834	6.810443	7.77519
GTF2F1	202354_s_at	4.813031	4.473237	4.776966	4.621976	5.108815	5.089162	5.10065	5.384033	4.975703	4.824125	5.929857	5.934017	5.811377
GTF2F1	202355_s_at	6.32579	6.76286	6.417727	6.686703	6.881577	6.798969	6.978117	7.14664	7.212727	6.907107	7.555321	7.752017	7.318072
GTF2F1	202356_s_at	5.056037	4.536126	5.034329	5.082131	4.628488	5.226063	6.385185	5.852651	6.446278	6.061075	6.267084	6.223795	6.002396
CFB	202357_s_at	14.06726	14.16489	13.774	14.02299	14.04652	13.9378	10.10326	10.54003	10.29811	10.12472	10.22059	10.26245	3.313948
SNX19	202358_s_at	6.618801	6.378026	6.635594	6.630539	6.443876	7.079985	6.401964	6.304784	6.66493	6.688184	6.565031	6.617568	6.636852
SNX19	202359_s_at	7.024672	6.791571	7.805819	7.482831	6.158669	6.221655	7.184246	6.684821	7.191483	7.224687	6.327146	6.663252	7.603268
MAML1	202360_at	7.486055	7.365694	7.393697	7.594247	7.462511	7.017316	8.019822	7.509173	8.027728	7.961718	7.55228	7.430973	8.933104
SEC24C	202361_at	8.195023	8.265557	7.879451	7.840885	8.143306	8.102118	8.797069	8.648175	8.721426	8.711707	8.887819	8.656467	7.807645

RAP1A	202362_at	8.930798	8.86258	9.041951	8.709871	8.50226	8.537183	9.744308	9.024236	9.724361	9.437963	8.506872	8.863471	8.712961
SPOCK1	202363_at	2.083515	2.065192	2.038937	2.045919	2.066356	2.041949	2.053294	2.06326	2.044518	2.0409	2.56673	2.053666	8.975805
MXI1	202364_at	9.072931	9.151659	9.858652	9.897731	9.714494	9.195052	10.42541	9.584176	10.61827	10.43002	9.049227	9.435886	8.66821
UNC119B	202365_at	7.841778	7.962559	7.502296	7.643028	8.103736	8.056484	8.294495	8.365223	8.289093	8.196879	8.269196	8.545451	8.321504
ACADS	202366_at	8.929419	9.072211	8.567104	8.837682	9.330601	9.588608	6.202987	7.228498	6.466797	6.391485	6.854096	7.201737	3.568526
CUTL1	202367_at	6.427103	6.193775	6.469044	6.597904	6.112164	5.948751	6.021675	6.208606	6.121324	6.170201	6.012935	6.153611	6.43819
TRAM2	202368_s_at	5.689823	5.914155	6.847949	6.855198	5.661267	5.340512	4.51024	5.364728	5.670779	5.342382	5.078335	5.012842	5.229114
TRAM2	202369_s_at	6.733018	6.662381	7.827064	7.52854	7.24343	6.755363	7.168378	6.3929	6.96696	6.979412	6.533878	6.770674	8.026053
CBFB	202370_s_at	6.277032	6.051419	7.145905	6.660084	5.908185	5.561906	7.6058	6.667748	7.398676	7.431037	7.071298	6.831617	9.742976
TCEAL4	202371_at	9.976722	9.953261	9.809797	9.871472	10.03317	10.09835	8.14076	8.763191	8.291708	8.31749	9.494923	8.905987	10.35382
RAB3GAP2	202373_s_at	8.270938	8.372737	8.39728	8.378458	8.281488	7.980624	8.502653	8.305902	8.430022	8.716752	8.197274	8.202713	8.383224
RAB3GAP2	202374_s_at	4.437198	4.286627	5.466971	5.113077	4.044644	4.151969	5.996463	4.284529	6.292725	6.591881	3.646279	4.28565	6.786051
SEC24D	202375_at	7.454225	7.242243	8.28136	8.178173	7.169818	6.210166	8.026846	6.75215	7.720468	7.759272	7.051019	6.904809	9.000556
SERPINA3	202376_at	13.31612	13.39944	13.60979	13.65666	13.90262	13.80627	8.509343	8.453325	8.258251	8.621346	8.355323	8.510074	5.38826
LEPR	202377_at	9.231164	9.126452	9.662205	9.522926	8.990808	8.394255	9.6986	8.795305	9.687986	9.968971	8.690736	8.709083	9.210021
LEPROT	202378_s_at	9.029573	8.974533	9.466883	9.322377	9.108854	8.938437	8.915805	8.758862	8.935388	8.924772	8.933177	9.01669	8.887414
NKTR	202379_s_at	9.686012	9.688079	9.13893	9.321699	9.713589	9.428392	9.80412	9.65107	9.518626	9.616351	9.93013	9.803728	10.3184
NKTR	202380_s_at	6.723458	6.968071	6.854671	7.092846	6.526591	6.121137	7.57441	6.301688	7.345404	7.165827	6.54186	6.521799	8.461527
ADAM9	202381_at	7.421761	6.941393	8.201214	8.248453	7.572885	7.113559	9.745734	8.471089	9.690353	9.464438	8.463858	8.588284	9.886745
GNPDA1	202382_s_at	7.247546	7.295606	7.215788	7.33923	7.623844	7.443448	8.396032	7.590269	8.228378	7.98845	7.191202	7.708994	8.370953
JARID1C	202383_at	4.99191	5.105355	4.580118	4.963168	4.979288	5.428113	5.11886	4.973076	5.070124	4.95088	5.046509	4.969129	4.457374
TCOF1	202384_s_at	4.648434	4.632985	4.169665	4.765228	5.16118	4.790676	5.280929	6.715053	5.047904	5.697534	7.03819	6.450514	4.691532
TCOF1	202385_s_at	2.40925	2.284032	2.168707	2.359439	2.692196	2.834836	3.708259	3.956481	3.249029	3.027487	3.506377	3.588155	2.924224
KIAA0430	202386_s_at	7.98788	7.824732	8.347448	8.235892	7.834839	7.237707	8.586441	7.967447	8.979273	8.792014	7.642673	8.093055	8.327654
BAG1	202387_at	8.582791	8.630889	7.903405	8.363554	8.53784	8.684835	8.435612	9.284602	8.413779	8.505619	8.892731	9.293804	6.909382
RGS2	202388_at	7.012741	7.573265	9.261008	8.440711	9.01688	8.417109	5.358778	4.77438	4.825459	5.678961	7.961677	5.201807	10.17642
HD	202389_s_at	5.054573	4.979752	4.940879	5.06765	4.978849	5.468036	4.975267	5.170211	5.169068	5.198986	5.14513	5.435716	3.481169
HD	202390_s_at	5.407613	5.228512	5.487636	5.585651	5.616019	6.469351	5.414824	6.139316	5.734896	5.470036	6.616747	6.503488	5.030966
BASP1	202391_at	6.214538	6.044123	7.680262	7.48922	7.549205	7.259564	6.579201	6.708113	6.74554	7.182713	8.263038	7.035928	11.90763
PISD	202392_s_at	5.829946	6.11631	6.246751	6.357973	6.553699	6.646312	7.769856	7.643364	8.074226	8.086677	7.371577	7.634858	6.068975
KLF10	202393_s_at	10.95816	10.82865	11.80394	11.69414	10.89191	10.91817	8.20365	7.70072	8.170865	7.534213	8.779715	8.398795	9.882903
ABCF3	202394_s_at	6.474887	6.372137	6.728961	6.24424	5.959714	5.801811	5.735326	5.44328	6.260861	5.683997	5.617256	5.920995	5.897692
NSF	202395_at	7.056584	7.158262	7.60349	7.360966	7.198393	7.039313	7.471529	7.208633	7.423274	7.356595	7.619413	7.505656	8.250874
TCERG1	202396_at	7.864252	7.437849	8.003718	7.846918	7.283052	6.987905	9.632042	8.933054	9.733373	9.833494	8.941068	8.820713	10.15212
NUTF2	202397_at	6.894586	6.736603	6.869283	6.948598	6.720459	6.895913	8.872182	8.497294	8.710129	8.77686	8.157285	8.352722	8.794744
AP3S2	202398_at	3.855681	3.128987	3.387136	3.056706	3.225141	3.65564	3.225685	3.461553	3.173945	3.520485	3.014142	3.030353	3.127755
AP3S2	202399_s_at	7.95989	8.005954	8.278747	8.142867	8.315387	8.311384	7.395918	8.080807	7.39099	7.408169	7.620378	7.619192	7.031891
SRF	202400_s_at	2.294858	2.263253	2.238631	2.252428	2.280347	2.269523	2.28629	2.448314	2.281778	2.2743	2.268157	2.383766	2.215812

SRF	202401_s_at	5.529309	5.411298	5.914883	6.159415	6.736323	6.450298	5.475898	5.755133	5.468083	5.812553	6.099389	5.745498	6.008191
CARS	202402_s_at	7.737461	7.344073	6.507235	6.743514	6.676541	6.681915	6.251015	6.692	5.856418	6.327759	6.581012	6.683842	5.41371
COL1A2	202403_s_at	9.080142	8.871497	8.27246	7.863603	7.924695	8.433319	9.330485	7.789674	8.111099	8.576981	12.65504	9.131988	14.24915
COL1A2	202404_s_at	9.24529	9.427827	6.13318	6.026284	6.94563	6.87766	9.936242	8.599455	8.873024	9.378752	13.32209	9.391448	15.0414
TIAL1	202405_at	6.189545	6.097495	6.670978	6.686837	5.886407	6.046889	6.028015	6.300648	6.075031	6.650194	6.449552	6.339437	7.616897
TIAL1	202406_s_at	8.842258	8.699722	8.808393	8.998437	8.639778	8.550152	9.420919	9.260134	9.56269	9.398185	9.248539	9.288309	9.120449
PRPF31	202407_s_at	2.543558	2.537972	2.915972	2.707481	2.529888	2.5267	4.465187	2.068732	4.237307	4.21956	2.591029	2.552935	5.236314
PRPF31	202408_s_at	7.831424	7.888748	7.397127	7.908021	8.174884	8.343279	7.583776	8.478116	7.65631	7.723462	8.795439	8.504374	7.934536
IGF2	202409_at	14.00038	14.07415	12.28194	12.22553	11.18958	10.49183	14.59346	14.23135	14.72912	14.75833	14.27092	14.07077	13.59456
IGF2	202410_x_at	10.08227	10.12461	8.316428	8.24088	6.510081	5.661754	11.71567	10.47323	11.25924	10.73142	10.37276	10.60038	9.683306
IFI27	202411_at	6.752308	6.360769	9.401579	9.364818	6.850847	6.881728	2.472328	2.476795	2.808341	2.532241	2.459575	2.473905	3.650293
USP1	202412_s_at	7.749623	7.13753	7.835169	8.037007	8.060124	7.88342	9.468621	9.331733	9.353302	9.177146	9.617542	9.795586	8.952393
USP1	202413_s_at	8.126716	8.044896	8.162191	8.036856	7.850021	7.714793	10.34332	9.625155	10.37418	10.30454	9.405952	9.583908	10.42999
ERCC5	202414_at	9.136643	9.352541	8.534294	9.045132	9.474663	9.524299	7.850034	8.507167	7.917845	7.799453	8.213168	8.531387	7.304486
HSPBP1	202415_s_at	5.994302	5.995934	5.805722	6.171462	6.037431	6.314619	6.338576	7.08946	6.314748	6.585295	7.251314	7.280833	6.027082
DNAJC7	202416_at	7.320248	7.023733	7.054451	6.734557	6.903247	6.89223	7.170204	7.184793	6.905975	6.98988	7.794196	7.376772	6.700355
KEAP1	202417_at	8.324982	8.200392	7.908422	8.025104	8.355455	8.471275	8.362295	8.714554	8.367761	8.282863	8.4103	8.75358	7.131266
YIF1A	202418_at	8.120433	8.098738	7.857397	7.948549	7.976381	7.853006	8.197656	8.097685	7.981299	8.186024	8.262207	8.366974	6.815543
FVT1	202419_at	9.098902	9.023653	8.621454	8.800184	8.601724	8.495434	6.103786	6.45854	6.167399	6.087748	6.342708	6.353386	6.408831
DHX9	202420_s_at	6.961198	6.779794	7.124631	6.961402	6.964737	6.575314	8.784395	8.415549	8.939716	8.847539	8.368225	8.635086	8.6683
IGSF3	202421_at	3.264823	2.952325	2.919095	2.906014	2.979967	3.555818	4.758738	4.58455	4.535698	4.558249	5.653929	4.83415	8.47433
ACSL4	202422_s_at	9.671147	9.444517	4.253044	4.481224	3.926142	4.259447	10.86008	10.43304	10.57394	11.07741	9.590295	10.00589	6.707039
MYST3	202423_at	8.218962	8.362566	7.976043	8.053174	8.248135	8.231114	8.750447	8.715413	8.78497	9.023429	9.097986	8.66296	8.774663
MAP2K2	202424_at	8.781299	8.734024	8.362387	8.503874	8.657124	8.79906	9.419313	9.848432	9.449767	9.412876	9.669258	10.10403	8.393808
PPP3CA	202425_x_at	7.037664	6.674045	6.785526	6.962557	7.678711	7.630929	6.762394	6.771743	6.563298	6.532657	7.000526	6.926425	7.282409
RXRA	202426_s_at	7.242863	7.334972	7.601487	7.697542	8.219063	8.184368	7.066755	7.151637	7.021738	7.517135	6.508013	6.786143	3.376919
BRP44	202427_s_at	10.83362	10.84681	11.59191	11.33861	10.76734	10.19175	11.95694	10.68783	11.80318	11.907	10.37539	10.78141	9.07547
DBI	202428_x_at	12.48014	12.40058	12.70376	12.83875	12.54602	12.52577	12.35772	12.26967	12.42367	12.35795	12.38896	12.5363	11.1324
PPP3CA	202429_s_at	7.399377	7.545634	8.590689	8.412678	7.668085	7.042118	8.375251	7.480278	8.499057	8.448298	7.509883	7.760139	9.567824
PLSCR1	202430_s_at	7.750274	7.703704	8.769533	8.64701	7.450676	7.165343	7.573063	6.820684	7.893492	7.554701	6.962548	6.95671	7.154023
MYC	202431_s_at	6.37871	6.832326	8.560912	7.449868	5.845102	4.991615	9.23198	7.756585	9.393435	9.235253	7.428598	7.929077	8.006566
PPP3CB	202432_at	8.198707	8.347133	8.110085	8.166693	8.586775	8.229852	9.728327	9.356116	9.448859	9.558021	9.835919	9.337058	10.58625
SLC35B1	202433_at	7.321933	7.290055	8.087717	7.699138	7.391702	6.322978	8.118119	7.699162	8.404549	8.482717	7.558383	7.515791	7.718234
CYP1B1	202434_s_at	2.152155	2.148228	2.127104	2.129704	2.14044	2.133207	2.142998	2.139303	2.126702	2.130944	2.127735	2.133196	2.105987
CYP1B1	202435_s_at	3.321199	3.059813	5.941878	5.153909	3.298386	3.222771	2.919853	2.924384	2.928657	2.921714	2.91613	3.245993	6.878673
CYP1B1	202436_s_at	4.60783	4.236722	7.289612	6.759758	5.470366	5.672379	2.706084	2.702789	2.719277	2.667893	2.701823	2.713827	7.805638
CYP1B1	202437_s_at	4.504125	4.455832	7.337285	7.173992	4.143095	3.916527	2.654493	2.664299	2.651434	2.630085	2.66145	2.673489	8.042225
IDS	202438_x_at	2.785161	2.683764	3.002086	2.985895	2.820156	2.663562	2.566825	2.548526	2.568252	2.619627	2.682378	2.689668	3.903359

IDS	202439_s_at	4.732463	4.344598	4.795937	4.997943	5.685676	5.902942	3.268818	3.732368	3.427069	3.106522	4.123609	3.594228	5.6788
ST5	202440_s_at	7.730437	7.688754	7.113225	7.441184	8.194427	8.096765	7.521986	7.24432	7.234479	6.99748	8.097347	7.515253	7.546801
ERLIN1	202441_at	10.54222	10.45862	10.89767	10.90833	10.3205	10.14241	10.93981	10.67444	10.6765	10.75454	10.49003	10.64449	9.312511
AP3S1	202442_at	8.885983	8.761045	9.492055	9.54329	9.134322	8.828401	9.96264	9.509815	10.13388	9.909385	9.641479	9.527396	10.85469
NOTCH2	202443_x_at	10.11659	9.891297	9.785551	9.719743	9.786335	9.833943	9.414708	8.949284	9.356042	9.287541	10.13958	9.047842	11.16182
ERLIN1	202444_s_at	2.845282	2.774752	4.219305	2.985449	2.548283	2.722939	4.768645	2.883159	5.067866	4.864975	2.717618	2.880611	5.529819
NOTCH2	202445_s_at	4.464057	4.459707	4.836573	4.787454	4.433534	4.336419	4.352144	4.057512	4.09684	4.029257	4.139103	4.350932	4.43898
PLSCR1	202446_s_at	8.196286	8.023134	9.248382	8.784655	7.967815	7.741308	8.45866	7.137923	8.329051	8.195558	7.50164	7.333827	8.298083
DECR1	202447_at	11.58308	11.47469	11.76812	11.88527	11.41487	11.23615	9.840081	9.929631	9.895398	9.653976	9.828406	9.849307	8.854898
ZER1	202448_s_at	2.29495	2.287462	2.246015	2.275172	2.294729	2.27282	2.285296	2.284197	2.283465	2.275464	2.270047	2.277442	2.230586
RXRA	202449_s_at	9.807957	9.689575	9.010061	9.21507	9.721696	9.781149	8.065237	8.404094	7.977195	7.994696	8.376363	8.535357	6.43124
CTSK	202450_s_at	4.823112	4.579457	5.07866	4.645825	4.026835	4.546624	3.919638	3.991	3.904001	4.473057	4.86505	2.805637	9.365869
GTF2H1	202451_at	8.182941	8.159596	7.495303	7.82092	8.263541	8.468468	7.865005	8.329939	7.975711	7.577933	8.111147	8.126992	7.978001
ZER1	202452_at	3.861273	3.960975	3.802089	3.80652	3.816286	4.324505	3.818645	4.821705	3.815689	3.809901	4.701162	4.602459	3.777259
GTF2H1	202453_s_at	3.418066	3.448651	3.411031	3.509909	3.392082	3.373341	4.362103	3.464313	4.232574	4.262213	3.393173	3.400729	6.2275
ERBB3	202454_s_at	9.740428	9.871213	9.403165	9.258882	9.061821	8.463557	7.850165	6.910162	8.023122	7.72635	7.204922	7.039937	6.668996
HDAC5	202455_at	6.562264	6.347448	6.439286	6.334487	7.210836	7.564896	5.105396	6.312888	5.236592	5.708642	6.399225	6.31955	6.225483
ZER1	202456_s_at	4.424492	4.559484	4.535779	4.563403	4.609583	4.544126	4.812373	4.758438	4.895385	5.21397	4.948821	5.100339	4.092743
PPP3CA	202457_s_at	7.509467	7.387161	8.568839	8.263216	8.302015	8.241194	7.669843	7.452775	8.136525	8.117521	8.019927	7.922537	10.25873
PRSS23	202458_at	6.3179	6.113645	6.478525	6.375921	6.514681	6.437773	3.857105	3.728534	4.209375	4.089948	6.391033	3.864759	9.044884
LPIN2	202459_s_at	9.039046	8.955309	9.040896	8.988837	7.092963	6.725517	7.699624	6.753186	8.090215	7.981928	6.601886	6.214588	6.208965
LPIN2	202460_s_at	10.90702	11.03553	10.70129	10.80017	9.557114	9.233916	9.348567	9.327312	9.505422	9.66069	8.308068	8.706739	7.537234
EIF2B2	202461_at	6.645538	6.537776	6.586527	6.794283	6.76883	6.698915	7.442394	7.325281	7.486823	7.640783	7.775406	7.459687	7.628571
DDX46	202462_s_at	8.477852	8.314525	8.589185	8.629985	8.407603	8.437178	9.216829	9.066059	9.225515	9.415168	9.009419	9.177225	9.366701
MBD3	202463_s_at	5.654749	5.746642	5.317109	5.43633	5.881589	5.901097	6.176085	6.71782	6.341538	6.303597	6.984728	7.317492	5.046456
PFKFB3	202464_s_at	7.000673	6.906831	8.987695	8.773855	8.075338	8.454894	6.361853	6.498667	6.048326	5.908634	6.833848	6.337485	7.398211
PCOLCE	202465_at	8.445641	8.683987	9.198797	9.522933	10.11952	9.864451	8.332286	7.685806	8.062334	8.133229	9.094185	8.097781	10.70646
POLS	202466_at	8.209599	8.286048	8.416731	8.563051	7.94948	7.779782	7.817892	7.491081	8.07681	8.098879	7.481704	7.615522	8.228105
COPS2	202467_s_at	9.748326	9.701994	9.822422	9.882754	9.274604	8.551663	10.60387	9.883038	10.7077	10.744	9.831336	9.989551	11.23718
CTNNAL1	202468_s_at	7.541021	7.343383	7.927414	8.122158	7.675803	7.371824	9.206632	9.023611	9.320543	9.206091	8.637381	9.041973	8.149347
CPSF6	202469_s_at	6.628424	6.712315	6.621677	6.683107	6.873425	6.516256	8.43313	8.350913	8.649245	8.547426	8.300976	8.516836	8.653412
CPSF6	202470_s_at	3.113686	2.915337	2.744178	2.896991	2.927869	3.298304	4.598206	3.491598	5.078883	5.085864	3.251063	3.622981	5.693196
IDH3G	202471_s_at	7.278373	7.074108	7.48826	7.243523	6.731613	6.113027	8.117107	7.31601	8.137474	8.153233	7.617846	7.402683	8.694313
MPI	202472_at	4.375455	4.039941	4.858488	4.56822	4.934313	4.573244	4.667175	4.87333	4.64809	4.603893	4.286229	4.743502	4.498313
HCFC1	202473_x_at	3.306253	3.491859	3.458085	3.148536	3.503357	3.698069	3.491768	3.499733	3.492985	3.48678	3.698002	4.164873	2.566739
HCFC1	202474_s_at	5.949271	5.988801	5.774691	5.539889	5.996186	5.9345	7.19026	7.297058	7.335753	7.349828	7.28746	7.445183	6.315444
TMEM147	202475_at	7.511976	7.32117	8.004846	8.217447	7.847051	7.79328	7.908827	7.880664	8.151992	7.553101	7.951808	8.153648	8.386109
TUBGCP2	202476_s_at	3.176028	3.143007	3.064678	3.156317	3.158401	3.352666	3.165356	3.16743	3.171137	3.152632	3.378952	3.318798	3.528506

TUBGCP2	202477_s_at	7.320295	7.428816	7.116836	7.376846	7.462395	7.775835	7.805212	7.975403	7.697571	7.612327	8.073192	8.376647	6.817489
TRIB2	202478_at	6.519343	6.115548	6.628755	6.566118	6.008279	4.430855	7.070453	6.421394	7.321414	7.274524	7.525257	6.636087	10.52508
TRIB2	202479_s_at	2.519468	2.485095	2.858146	2.470783	2.469456	2.471189	2.482544	2.50231	3.033261	2.624007	3.092075	2.509397	7.693939
DEDD	202480_s_at	2.917127	2.858694	2.903842	2.90648	2.907135	2.829928	3.924198	2.904616	3.212797	3.776132	2.906068	2.878736	4.752933
DHRS3	202481_at	10.45181	10.47193	10.04503	10.4048	10.39817	10.03636	8.767263	9.113454	8.975358	8.719083	8.87427	9.119691	7.161286
RANBP1	202482_x_at	3.86588	3.795759	3.763474	3.813798	4.14569	3.783436	3.817187	3.825197	3.763644	3.79357	3.829205	3.833111	3.651029
RANBP1	202483_s_at	7.787793	7.777793	8.323038	8.322727	7.90103	7.679004	10.2551	9.562149	10.30417	10.40052	9.552303	9.644777	9.659039
MBD2	202484_s_at	8.593241	8.494421	8.888413	8.753949	8.435595	8.279723	9.20327	8.713385	9.364691	9.330023	8.455657	8.864506	7.864428
MBD2	202485_s_at	2.195468	2.182032	2.161961	2.165334	2.180854	2.162432	2.170799	2.177517	2.164561	2.158145	2.159264	2.172059	2.111649
AFG3L2	202486_at	7.381883	7.222391	6.942352	7.187837	7.032803	6.938177	6.644579	7.239426	6.616659	6.908859	7.022112	7.477888	5.251071
H2AFV	202487_s_at	7.59771	7.420471	7.768125	7.740807	7.028731	6.733338	9.904739	8.709707	9.989394	9.92155	8.937463	8.990407	9.558284
FXYD3	202488_s_at	2.445583	2.422883	2.404143	2.405216	2.427087	2.40705	2.420193	2.426061	2.412338	2.408859	2.408562	2.418472	2.351215
FXYD3	202489_s_at	2.130786	2.13645	2.099981	2.116817	2.144115	2.127073	2.143834	2.142043	2.137544	2.116881	2.127287	2.199337	2.119243
IKBKAP	202490_at	4.773269	4.707581	4.624264	4.67234	4.685557	4.628359	4.674169	4.724451	5.079868	5.085632	4.677065	4.768636	4.075211
IKBKAP	202491_s_at	9.616022	9.350928	8.900283	8.54143	8.08813	7.547234	10.3971	9.402822	10.59948	10.77761	9.618673	9.595652	10.3307
ATG9A	202492_at	7.616631	7.702491	7.274736	7.470185	7.736335	6.945096	7.693023	8.544622	8.023365	7.835408	7.45214	8.447303	6.187475
CSH1	202493_x_at	2.316549	2.297374	2.255376	2.27771	2.302716	2.280727	2.288912	2.294626	9.301101	5.255267	2.45399	2.287345	7.099253
PPIE	202494_at	5.961802	5.590789	6.194531	6.16849	6.139529	6.115134	6.140941	6.365227	6.212191	6.970314	6.863441	6.865629	5.626543
TBCC	202495_at	6.686464	6.952115	6.699862	6.657433	6.685697	6.600005	6.791963	6.614183	7.03435	6.915087	6.555779	6.529843	8.016082
EDC4	202496_at	5.154979	5.356423	4.836129	4.970384	5.164403	5.787891	5.314764	5.667931	5.345793	5.278915	6.529485	5.787669	5.526699
SLC2A3	202497_x_at	4.227951	4.02344	4.667822	4.562429	4.79876	5.029646	5.384574	4.920984	5.538904	5.233367	4.928033	5.302824	6.267204
SLC2A3	202498_s_at	3.096482	3.15579	3.044895	2.880222	3.059049	3.008229	4.159824	3.280076	4.960132	5.293295	3.013959	3.325287	4.721489
SLC2A3	202499_s_at	4.62742	4.193137	5.46772	4.18995	6.615925	7.176938	6.578828	7.202261	6.532994	6.856857	7.329972	7.943703	6.667161
DNAJB2	202500_at	6.685913	6.522586	5.683057	5.952531	7.029004	7.539734	6.783045	7.935437	6.934772	7.224076	7.726443	7.766729	5.712529
MAPRE2	202501_at	7.652022	7.593747	8.54657	8.31645	7.255276	6.996107	8.682372	6.666977	8.798462	8.566999	7.045887	7.275424	8.177324
ACADM	202502_at	11.23243	11.13347	11.79649	11.61943	11.28408	10.93109	10.45931	10.16476	10.64777	10.49035	9.453683	9.739128	9.920994
KIAA0101	202503_s_at	6.931531	6.662534	4.755469	4.454128	4.21131	5.271236	12.05662	11.45195	12.18844	12.16095	11.12538	11.32469	11.13143
TRIM29	202504_at	2.278858	2.268884	2.234216	2.247622	2.267057	2.251278	2.255166	2.265012	2.254594	2.250588	2.249652	2.258709	3.67983
SNRPB2	202505_at	9.674936	9.589285	9.576119	9.683891	9.622197	9.508499	10.10791	10.31701	10.21589	9.997284	10.42807	10.36936	10.68769
SSFA2	202506_at	9.729956	9.497331	9.833162	9.31906	8.758415	7.530684	8.501231	7.01341	8.416392	8.551483	7.027306	6.911845	8.37857
SNAP25	202507_s_at	2.428115	2.625927	2.350984	2.375268	2.387214	2.362462	2.383888	2.395286	2.355092	2.36664	2.356818	2.36486	6.060382
SNAP25	202508_s_at	3.102845	2.653577	2.5869	2.620141	2.663904	2.588362	2.920063	2.6202	3.248921	2.639723	2.840532	2.64163	9.197192
TNFAIP2	202509_s_at	1.803421	1.909831	1.750103	1.764513	2.148673	2.112966	1.762267	1.767606	1.75565	1.749295	1.753612	1.759416	1.69198
TNFAIP2	202510_s_at	5.999757	6.376336	5.727653	5.078813	6.06841	6.31549	3.861003	3.80264	3.374825	3.665788	3.930629	3.708214	4.266001
ATG5	202511_s_at	7.151772	7.195793	7.235253	7.290513	6.905209	6.646988	7.06357	7.313309	7.286501	7.433665	6.899914	7.138245	6.875894
ATG5	202512_s_at	6.54629	6.830329	7.269924	7.077363	6.649512	6.306199	5.928257	5.272934	5.988617	6.267568	5.50907	5.839077	6.386857
PPP2R5D	202513_s_at	6.603041	6.951531	5.971923	6.042693	6.385174	6.456821	6.703538	6.801592	6.424695	6.363523	6.680815	7.064281	6.170566
DLG1	202514_at	5.820294	5.939924	6.859031	6.041756	5.742203	5.223991	6.393692	4.876123	6.765312	6.435493	5.264917	5.1746	7.960636

DLG1	202515_at	8.565144	8.565944	8.532405	8.517081	8.40003	8.26203	8.582378	8.110138	8.593563	8.282595	8.442568	8.319125	9.013557
DLG1	202516_s_at	3.205415	3.014949	3.588223	3.119198	3.022708	2.868979	4.307842	2.97003	4.467713	4.361179	3.010266	3.013322	6.046868
CRMP1	202517_at	2.171291	2.160929	2.144473	2.192585	2.059115	2.168362	2.22764	2.49231	2.177262	2.712545	3.536394	3.19833	8.645782
BCL7B	202518_at	5.928213	5.828827	6.139193	6.361911	5.853745	5.507741	5.862299	5.686608	6.025303	5.82685	5.705872	5.963737	5.158091
MLXIP	202519_at	6.133782	6.433999	6.248611	6.473633	7.087239	6.766086	6.092004	6.08964	7.200786	6.205084	7.202808	6.669165	5.573573
MLH1	202520_s_at	7.751658	7.549922	8.053678	8.013494	7.49481	7.212276	9.673837	9.153777	9.699875	9.789648	9.058938	9.234201	9.073071
CTCF	202521_at	7.181113	7.188228	7.30176	7.278047	7.059598	6.94679	8.745459	8.476119	8.755019	8.75445	7.858573	8.386729	8.488143
PITPNB	202522_at	9.682988	9.635691	9.735061	9.756573	9.491319	9.328427	9.437491	9.327839	9.411622	9.426504	9.314812	9.247674	9.595584
SPOCK2	202523_s_at	2.253941	2.241319	2.22212	2.221438	2.240707	2.219645	2.236303	2.238122	2.226804	2.216608	2.218963	2.232188	2.179705
SPOCK2	202524_s_at	2.51842	2.532154	2.723236	3.299261	2.55337	3.782636	2.332467	2.351907	2.29734	2.325234	2.298093	2.185294	5.059281
PRSS8	202525_at	4.478933	5.5416	3.461319	3.598976	3.467427	3.460572	3.250365	3.280115	2.997197	3.232628	2.479482	2.498473	2.929639
SMAD4	202526_at	3.901519	3.873966	4.478733	4.431259	3.620953	3.591763	3.468109	3.610245	3.524245	3.611033	3.047214	3.08157	3.256293
SMAD4	202527_s_at	7.244599	7.265919	8.094445	7.726016	6.560623	4.973634	8.174112	6.276372	8.190515	8.299591	6.54456	5.797746	9.56662
GALE	202528_at	7.561334	7.456464	7.344026	7.456636	7.659868	7.732438	6.692077	6.235225	6.505913	6.578429	6.639654	6.837524	5.150642
PRPSAP1	202529_at	10.20231	10.15518	10.01651	9.959028	9.71449	9.241646	8.996722	8.536927	9.182583	9.241305	8.289333	8.619691	8.941465
MAPK14	202530_at	7.241268	7.135569	7.518086	7.323305	7.467015	7.181762	8.275809	7.763007	8.231058	8.428385	7.673164	7.828891	7.027299
IRF1	202531_at	6.325472	6.312607	6.749571	6.083212	6.275209	6.363167	2.44257	3.114314	3.314307	2.569445	3.371669	3.080389	2.806331
DHFR	202532_s_at	7.714842	7.518439	9.135045	8.899889	7.324959	7.495615	8.812394	9.579964	8.861227	9.023555	8.373072	9.372115	6.954204
DHFR	202533_s_at	3.559462	3.86825	4.372222	4.41185	3.781541	3.382811	6.885298	5.937962	6.383025	6.850905	5.096708	5.242846	4.30635
DHFR	202534_x_at	9.051271	8.812075	9.776988	9.964747	8.680588	8.374518	10.33209	10.68399	10.29824	10.39758	9.961237	10.73294	8.123254
FADD	202535_at	6.028897	6.028469	5.675147	5.673279	6.058953	5.655551	6.142138	6.159115	6.472848	6.764802	6.088962	6.432028	5.967037
CHMP2B	202536_at	8.716027	8.584867	9.143175	9.263733	8.605892	8.558389	8.886306	8.7685	9.026565	9.187554	8.840031	8.957953	9.276691
CHMP2B	202537_s_at	7.168104	7.063772	7.772892	8.042271	7.118882	6.790133	7.130744	7.093308	7.360494	7.158388	6.261335	7.410115	7.937482
CHMP2B	202538_s_at	6.748979	6.649957	7.728895	7.357689	6.19312	5.834393	7.700584	6.502331	7.702688	7.950191	5.556419	6.343891	8.090322
HMGCR	202539_s_at	8.062183	7.346708	7.643741	7.222943	8.694303	7.968946	11.34259	9.7335	11.68537	11.51531	9.117357	9.861038	10.00579
HMGCR	202540_s_at	7.081257	7.092428	7.382027	7.36575	8.653803	8.435827	11.06691	10.51037	11.17057	11.12454	10.17588	10.61665	9.337435
SCYE1	202541_at	7.538737	7.415006	7.594377	7.36535	5.98286	6.460158	7.462628	6.579614	7.362724	7.412129	7.035231	6.797458	7.889739
SCYE1	202542_s_at	7.740674	7.632608	8.069889	7.911492	7.832471	7.954354	8.275252	8.023334	8.680898	8.557593	8.075267	8.091843	7.927685
GMFB	202543_s_at	6.128625	5.854853	5.343169	5.398769	5.783478	5.454125	6.143876	5.483829	6.213201	5.661911	5.878993	5.342944	8.377417
GMFB	202544_at	9.045722	8.930211	8.89947	8.704552	8.572847	8.678188	9.290929	8.868123	9.044949	9.139966	9.455499	9.200717	10.38632
PRKCD	202545_at	2.50023	2.454263	2.382997	2.456184	2.464873	2.459836	2.669672	2.691512	3.139627	2.660382	2.490001	2.491592	3.008328
VAMP8	202546_at	10.11126	10.23741	9.928183	10.02041	10.2248	9.875506	10.24454	9.88542	9.843901	9.892268	9.455141	10.07504	6.894203
ARHGEF7	202547_s_at	5.520658	5.56057	5.054375	5.304503	5.522215	5.943128	4.910155	5.508264	4.969635	4.732883	5.488105	5.5387	5.64224
ARHGEF7	202548_s_at	8.166626	8.113299	8.021272	8.078724	7.643224	7.172178	6.988481	7.071308	7.293466	7.479807	7.245317	6.917037	8.729423
VAPB	202549_at	5.115807	4.534694	4.980093	5.489047	6.092239	5.155755	4.661151	5.339581	3.996251	4.149054	6.259345	5.370157	4.57125
VAPB	202550_s_at	8.737575	8.758925	8.810249	8.572587	8.339245	8.334907	8.435466	8.33566	8.610576	8.387524	8.340065	8.23146	7.89535
CRIM1	202551_s_at	7.991701	7.951221	8.541382	8.237123	9.053102	8.581487	7.82982	7.017734	7.475233	7.129192	7.556702	7.175809	9.94896
CRIM1	202552_s_at	7.211983	6.854679	6.65212	6.72881	8.147746	8.564917	5.69397	6.028694	5.737387	5.908912	6.635236	6.105963	7.649897

SYF2	202553_s_at	9.497085	9.343182	9.007122	9.297224	9.596164	9.70723	8.371052	8.906989	8.344687	8.488381	9.072847	8.98842	8.734401
GSTM3	202554_s_at	4.839678	4.476628	3.37745	2.91545	8.580861	8.429128	7.831877	7.993255	8.657355	7.906568	9.299437	8.467793	8.455965
MYLK	202555_s_at	8.886344	8.97373	10.3167	10.39202	10.08623	10.06542	6.477166	6.634567	6.532262	6.157034	7.182708	7.197703	8.085613
MCRS1	202556_s_at	4.819958	4.210844	4.10245	4.761527	4.728471	4.961922	6.440728	5.7769	6.997258	6.659138	6.064721	6.531187	6.951138
STCH	202557_at	6.642347	6.525713	7.530872	7.431915	6.933787	6.891619	9.162065	8.754905	9.410461	9.433426	8.543299	8.99409	10.14391
STCH	202558_s_at	4.952012	4.798061	5.419212	5.324869	5.241661	5.170677	7.298265	6.178179	7.706847	7.327197	6.412488	6.568481	8.421256
C1orf77	202559_x_at	7.378752	7.199553	7.259417	7.391289	7.445107	7.350584	7.803812	7.833261	7.862534	7.824219	7.811444	8.09192	7.546532
C1orf77	202560_s_at	7.596744	7.651021	7.667645	7.590366	7.794556	7.437721	8.491553	8.101535	8.651121	8.366436	8.293133	8.275847	8.853605
TNKS	202561_at	6.762149	6.704521	6.660818	6.63405	6.300022	6.016121	6.805155	6.46164	6.690149	6.65725	6.570831	6.553239	8.29958
C14orf1	202562_s_at	9.667633	9.455373	9.55779	9.630916	10.87172	10.57385	10.1963	9.940321	10.29568	10.17517	9.545636	9.907072	8.351608
C14orf1	202563_at	4.48104	4.144137	3.952209	4.24104	4.439518	4.353307	4.177362	4.090446	4.154382	4.15001	4.284282	4.419694	3.443773
ARL2	202564_x_at	4.716852	4.72028	4.188975	4.206837	4.71986	5.600182	5.638631	6.198573	5.608041	5.553904	6.312433	6.384883	6.09441
SVIL	202565_s_at	9.123693	9.034068	8.57347	8.627133	7.75169	7.801502	6.936077	6.399982	6.426036	6.363571	9.293714	6.438446	11.29975
SVIL	202566_s_at	4.78088	4.677643	6.098699	4.851226	3.966113	3.527584	4.083253	3.918692	3.911171	3.698867	4.609528	3.527971	8.684396
SNRPD3	202567_at	9.762699	9.793144	10.01248	10.0867	10.01692	10.00401	10.6633	10.91942	10.61676	10.88167	10.92171	11.04746	10.12369
MARK3	202568_s_at	7.25212	7.14058	7.097682	7.229971	6.862443	7.027369	9.255829	9.097634	9.342836	9.472308	8.550722	8.682468	7.891738
MARK3	202569_s_at	7.240724	7.155416	6.694041	6.687076	6.62391	6.842062	8.719129	8.913195	8.886288	9.237968	8.415997	8.457478	8.216438
DLGAP4	202570_s_at	4.346922	4.883321	4.651205	4.892265	4.993796	5.664334	3.8268	4.700931	3.820947	3.909542	4.84509	4.876116	4.822095
DLGAP4	202571_s_at	2.527545	2.470838	2.4825	2.480084	2.509943	2.486288	2.488016	2.495169	2.483802	2.459864	2.479376	2.496032	2.554516
DLGAP4	202572_s_at	3.547261	3.648033	3.418948	3.755901	3.292458	3.144463	3.218894	3.090986	3.311842	3.319907	3.824965	3.030739	5.282548
CSNK1G2	202573_at	7.448718	7.37553	6.815707	7.069856	7.597802	8.243331	7.331051	8.073976	7.435485	7.294588	8.159001	8.259139	7.175933
CSNK1G2	202574_s_at	5.504647	5.50752	5.245634	5.330694	5.490155	5.955065	5.5023	5.637594	5.491933	5.806948	5.553797	5.834327	5.371341
CRABP2	202575_at	2.25491	2.236353	2.199023	2.218903	2.236319	2.225654	2.230991	2.231039	2.217733	2.208715	5.764942	2.22743	7.49928
DDX19A	202576_s_at	6.676102	6.702064	6.991224	6.781456	6.634343	5.762052	6.51954	6.052306	6.589777	6.796503	6.12891	5.571813	7.254986
DDX19A	202577_s_at	6.675972	6.448718	6.477487	6.460069	6.573451	6.732191	7.455072	7.623713	7.273726	7.299998	7.651468	7.779277	7.403214
DDX19A	202578_s_at	5.519922	5.564626	5.573994	5.662671	5.39439	5.438757	6.012154	5.615235	6.395352	6.125805	5.451479	5.265058	6.370006
HMGN4	202579_x_at	6.825598	6.833993	6.404836	5.941814	5.70494	5.746431	7.494652	6.057255	7.496997	7.636462	6.468391	5.984206	9.282945
FOXM1	202580_x_at	2.751643	3.28291	2.253344	2.017632	2.269769	2.344516	7.283205	7.542839	7.609503	7.478116	7.620879	8.000815	5.689373
HSPA1B	202581_at	7.036891	6.74456	7.484205	6.673843	6.931889	6.664983	8.323055	7.128268	8.165659	8.403611	7.133528	7.86186	8.315287
RANBP9	202582_s_at	10.00871	9.897603	10.27097	10.26926	9.426784	8.925991	10.4911	9.856758	10.26357	10.38212	9.745791	9.906878	10.31608
RANBP9	202583_s_at	4.562246	4.131714	6.150971	5.419279	2.917868	2.44378	6.340842	3.219172	6.483497	6.520387	3.433061	3.598503	6.302139
NFX1	202584_at	5.55429	5.558237	6.611396	6.314885	5.389454	5.293654	5.887291	5.579322	5.724822	5.742348	5.391692	5.691188	5.503725
NFX1	202585_s_at	3.99194	3.735665	5.12391	4.696169	3.79109	4.078345	4.523372	4.043173	4.469384	4.230768	4.012129	4.072436	4.686513
POLR2L	202586_at	2.717951	2.825744	2.601697	2.647065	2.681973	2.655876	2.706117	2.677566	2.662124	2.653162	2.663333	2.671739	2.581093
AK1	202587_s_at	7.31034	7.344075	6.689857	7.060213	7.410999	7.416751	8.852795	9.201659	8.901661	9.164382	9.584037	9.164536	9.211581
AK1	202588_at	3.625579	3.659115	3.599477	3.639644	3.627416	3.871692	3.64753	3.771809	3.643213	3.61882	3.740471	3.652996	3.729205
TYMS	202589_at	8.680045	8.457331	5.699866	5.887524	5.706713	6.25675	12.38989	12.31098	12.32542	12.54063	12.01104	12.21029	11.35527
PDK2	202590_s_at	7.123657	7.005493	7.022879	7.200435	6.924416	6.853392	3.733489	5.087915	4.375233	4.016842	4.754667	4.834741	5.151397

SSBP1	202591_s_at	10.07383	9.908233	10.40442	10.29536	10.02957	9.39212	11.39601	10.63646	11.24295	11.25816	10.69909	10.73112	11.42692
BLOC1S1	202592_at	9.139015	9.04219	9.254014	9.317369	9.636069	9.865766	8.791718	9.397464	8.771643	8.990894	9.276282	9.572185	7.927639
MIR16	202593_s_at	4.388634	4.493289	6.048861	5.235243	4.377793	3.391064	7.752199	4.861738	8.106807	8.08773	4.563369	5.065142	8.054114
LEPROTL1	202594_at	7.544448	7.680569	7.428054	7.751672	7.60706	7.573683	7.125374	7.161391	7.86193	7.856155	7.905921	7.693278	8.819214
LEPROTL1	202595_s_at	7.329033	7.091902	7.564861	7.533115	7.24277	6.98516	7.024609	7.054748	7.475886	7.302285	6.954818	7.250606	6.792319
ENSA	202596_at	9.527906	9.729128	9.862119	9.849082	10.52394	10.50325	9.959415	10.28113	10.02554	10.02057	10.27412	10.21962	9.305096
IRF6	202597_at	7.03356	6.956803	7.521615	7.613815	7.428616	7.291194	2.173582	2.901122	2.331754	2.272498	2.146827	2.156558	3.126985
S100A13	202598_at	9.170547	9.27843	8.488802	8.740707	10.27478	10.52163	7.860093	8.671679	8.346156	7.928906	9.432068	9.598769	8.744303
NRIP1	202599_s_at	9.434425	9.080979	9.368088	9.239216	8.572654	8.03496	8.273417	7.640623	8.185102	7.899247	8.009421	8.114917	9.280353
NRIP1	202600_s_at	9.436869	9.289591	9.926586	9.684569	8.608968	8.075215	8.192965	7.202933	8.373237	7.704926	8.468217	7.791591	10.02199
HTATSF1	202601_s_at	3.466143	3.461861	3.621998	3.600284	3.637338	3.626608	4.219512	4.925101	4.355585	4.690003	4.738395	4.685848	5.763561
HTATSF1	202602_s_at	8.828389	8.680001	8.799441	8.576406	8.318187	7.893023	9.988429	9.647944	10.14361	9.886944	9.785935	9.5551	10.45257
ADAM10	202604_x_at	3.812375	3.609203	3.818543	3.545137	3.358602	3.314805	4.270454	3.079041	4.061563	3.829909	3.11384	2.779675	5.834337
GUSB	202605_at	10.81694	10.87605	10.48653	10.86802	10.96738	10.97402	9.878979	10.23951	9.747551	9.802974	9.961885	10.31852	8.357
TLK1	202606_s_at	9.400175	9.313935	9.453567	9.316122	8.901412	8.569007	9.07408	8.859485	9.220941	9.23982	8.486	8.672429	9.148115
NDST1	202607_at	7.382577	7.101711	6.902554	6.889409	7.181096	6.603034	6.492509	6.420587	5.856408	6.424926	6.373554	6.411079	5.755749
NDST1	202608_s_at	3.186241	3.177883	3.13724	3.16507	3.182977	3.166926	3.160411	3.177307	3.156152	3.144737	3.549889	3.165644	3.051943
EPS8	202609_at	8.969179	8.912675	10.02635	9.60768	8.728067	8.011319	8.836715	7.263051	8.318154	8.330377	7.996205	7.795565	10.51211
MED14	202610_s_at	7.073819	6.759071	6.887452	7.052119	6.88188	6.702191	6.843695	6.761693	6.910353	7.192473	6.741784	6.827373	7.386548
MED14	202611_s_at	6.329283	6.35268	6.872251	6.266048	5.848541	5.925192	6.583176	5.834561	7.03904	6.690882	6.074258	6.365197	7.947723
MED14	202612_s_at	2.505921	2.464333	2.4403	2.450602	2.483325	2.447472	2.455385	2.467067	2.452391	2.438637	2.450982	2.470434	2.317044
CTPS	202613_at	8.278867	8.080513	8.155958	8.187497	6.108699	5.947598	7.449785	7.29674	7.551345	7.57658	7.39846	7.448342	7.221139
SLC30A9	202614_at	7.937936	8.173882	8.222367	8.242132	8.113191	7.874681	8.66304	8.460536	8.857261	8.909091	8.454856	8.586037	8.706644
GNAQ	202615_at	8.476055	8.392773	8.580053	8.37432	7.924668	7.717653	8.941166	7.878608	8.898884	8.742602	7.985932	8.111294	9.336467
MECP2	202616_s_at	4.016628	3.920208	4.893535	4.687144	4.020262	3.899855	4.555937	3.86123	5.075032	4.114726	3.957607	3.910051	5.960309
MECP2	202617_s_at	4.640035	4.726949	4.89237	4.701917	4.739802	4.521051	5.042557	4.854225	4.796469	4.800621	4.712848	4.985962	5.142718
MECP2	202618_s_at	4.061873	4.164952	3.933459	4.041592	4.62777	4.249052	4.159905	4.163818	4.155628	4.121694	4.147081	4.204271	3.442533
PLOD2	202619_s_at	7.820484	7.567718	8.031074	7.951678	7.156764	6.587695	7.13566	6.443369	7.173081	7.301232	6.659932	6.845658	8.066025
PLOD2	202620_s_at	10.14335	10.16599	9.89181	9.826864	8.878191	8.574603	8.593625	8.599001	8.965096	9.124452	8.661385	8.834636	9.718753
IRF3	202621_at	8.441546	8.507471	7.861046	8.079638	8.79655	9.102207	7.883033	8.592003	8.216518	8.236492	8.298865	8.584334	7.37405
ATXN2	202622_s_at	7.121902	6.837344	6.826324	6.709986	6.479931	6.57031	6.714802	6.22719	6.393921	6.597587	6.729033	6.263651	6.961155
EAPP	202623_at	8.150082	8.051686	8.528351	8.583284	7.993188	7.549517	7.973713	7.467664	8.267998	8.464412	7.631935	7.617922	8.259031
CABIN1	202624_s_at	5.319458	5.333015	5.445182	5.003824	5.596507	5.780114	5.59289	5.79793	5.730093	5.606148	6.181763	6.000128	5.733146
LYN	202625_at	7.080928	6.753955	6.906937	7.179695	7.202517	6.711666	6.839916	6.263373	6.628392	6.377878	6.39158	6.635636	5.155768
LYN	202626_s_at	5.361002	4.784572	6.162455	6.086794	5.565777	4.35237	5.760766	4.627763	5.605346	5.51124	4.730855	5.163774	3.969002
SERPINE1	202627_s_at	4.763291	4.387708	6.864147	6.299356	4.923772	5.389655	3.323061	3.326258	3.315865	3.883169	3.365635	3.621154	4.972634
SERPINE1	202628_s_at	6.695894	6.359859	8.240622	7.365131	7.402046	6.767912	3.006346	2.847834	3.145246	3.558121	2.848196	2.842614	5.380407
APPBP2	202629_at	6.3792	6.383134	6.801615	6.804782	6.116937	5.69121	6.898516	6.259143	6.603859	6.971354	6.589913	6.461227	8.349107

APPBP2	202630_at	7.211916	7.02759	7.248872	7.24534	6.957398	6.594483	6.892063	6.553273	6.674349	6.635019	6.798975	6.828671	8.369902
APPBP2	202631_s_at	4.948629	5.260375	5.255659	5.056143	5.251398	4.858523	5.570874	4.708251	5.281841	4.946633	5.096281	5.129914	6.861852
DPH1	202632_at	6.538539	6.650975	6.779864	6.674529	6.405804	6.498497	6.396189	6.578106	6.614054	5.977889	6.007132	6.758212	5.986299
TOPBP1	202633_at	7.180377	6.784719	7.186167	6.911853	6.601678	5.960296	9.054101	8.24686	9.316254	9.238127	7.946929	8.123179	8.777202
POLR2K	202634_at	7.850629	7.887736	8.569196	8.212437	7.857731	8.020021	8.65292	8.256031	8.497034	8.77487	8.026419	8.320413	8.418145
POLR2K	202635_s_at	8.266184	8.047817	9.022043	8.780301	7.808762	7.364309	9.84695	8.510206	9.925439	9.83495	8.345114	8.590469	10.10775
RNF103	202636_at	8.810957	8.958296	9.26799	9.09646	8.203756	7.560877	7.670358	7.086132	7.731931	7.584691	6.310106	6.560896	8.018706
ICAM1	202637_s_at	4.672894	4.538557	5.770799	4.697727	4.748888	5.027023	3.939918	3.426385	3.501917	3.688508	2.612	3.366857	2.780269
ICAM1	202638_s_at	5.705192	6.020107	7.201082	6.558462	6.594126	6.862347	4.067483	3.540312	3.869532	3.596466	2.7841	3.724371	2.808864
RANBP3	202639_s_at	4.843534	4.440878	4.700919	4.795908	4.839217	4.793152	4.919082	5.309694	5.103634	4.96053	5.198174	5.743304	5.013384
RANBP3	202640_s_at	5.784079	5.556641	5.758254	5.734037	5.644267	5.762529	5.911096	6.678704	6.051283	6.392292	6.548102	6.605797	6.000061
ARL3	202641_at	7.575834	7.861626	7.784196	7.996201	8.638318	8.61453	7.786388	8.406482	8.203363	7.785721	8.838764	8.745982	9.276642
TRRAP	202642_s_at	5.22162	5.190833	5.16638	5.178501	4.908723	4.675754	5.815283	5.216936	5.828143	5.820537	5.68351	5.191303	6.407024
TNFAIP3	202643_s_at	4.917678	4.967561	5.586692	5.080658	4.415218	5.025559	3.947888	3.759167	4.026262	3.922301	3.849388	3.859947	4.112402
TNFAIP3	202644_s_at	7.41209	7.302085	7.445229	7.550108	7.474218	7.932435	4.631493	5.47575	5.34593	4.851384	6.498966	5.381707	5.63076
MEN1	202645_s_at	6.417364	6.035019	6.080537	6.314071	5.336389	4.505743	6.120437	5.697218	6.344553	6.23207	5.62785	5.936671	6.28644
CSDE1	202646_s_at	9.897933	10.02428	10.00847	10.22334	10.41912	10.39861	9.616909	10.32944	9.827645	9.884901	10.56327	10.5107	10.05087
NRAS	202647_s_at	4.77859	4.655092	4.833789	4.438497	2.985157	3.532511	7.017943	5.736353	6.971467	6.99	5.419994	5.455459	7.986475
RPS19	202649_x_at	12.51287	12.49618	12.42217	12.58057	12.40779	12.45936	13.29008	13.16031	13.28129	13.17645	13.36175	13.22138	13.86995
KIAA0195	202650_s_at	5.955064	5.893315	5.413324	5.792137	5.812757	5.715891	5.326866	5.714153	5.677003	5.725406	6.188625	5.762779	5.6333
LPGAT1	202651_at	10.6751	10.65174	9.403991	9.365735	9.203392	8.908604	9.408139	9.446356	9.289065	9.344974	9.184213	9.740896	8.432528
APBB1	202652_at	3.339564	3.31259	3.345925	3.303754	3.448183	3.302726	3.300978	3.315036	3.302902	3.188704	3.30347	3.314889	3.65613
MARCH7	202653_s_at	9.311883	9.231962	9.27645	9.30524	9.03416	8.566027	9.668774	9.383668	9.652813	9.56791	9.350371	9.388147	10.10359
MARCH7	202654_x_at	6.262682	6.105158	7.890633	6.477229	6.201248	5.415375	8.479442	6.084297	8.410879	8.264561	6.143834	6.232217	9.80351
ARMET	202655_at	7.734338	7.905645	7.909948	7.849344	8.869966	8.869249	8.893247	8.912624	8.970331	9.003754	9.233562	9.18126	8.846285
SERTAD2	202656_s_at	7.005048	6.904293	7.642381	7.685287	7.537865	7.274278	8.281923	7.986238	8.354563	8.339965	7.717379	7.914662	7.600425
SERTAD2	202657_s_at	8.655046	8.488924	8.285412	8.494847	9.090279	9.02872	8.751484	9.127953	9.091607	9.060467	8.72153	8.770758	8.118519
PEX11B	202658_at	6.834494	6.993892	7.353132	6.942783	6.562755	5.450563	6.838551	5.990134	6.831931	6.885888	5.254933	5.562747	7.365744
PSMB10	202659_at	7.587059	7.479409	7.545583	7.639443	8.336946	8.798935	6.354094	7.313931	6.67793	6.110951	7.408976	7.743722	5.369341
ITPR2	202660_at	11.46773	11.28784	11.00932	10.9211	11.11315	10.65838	9.248113	9.539116	9.250167	8.831067	8.81397	9.18702	6.203686
ITPR2	202661_at	6.142673	5.999137	5.496094	5.799134	5.931872	5.81786	4.955872	4.885404	4.855864	4.745665	4.054434	4.797499	2.116698
ITPR2	202662_s_at	6.440083	6.474145	7.83746	7.022322	5.895396	5.181119	5.460146	4.63339	6.068579	6.115395	3.818255	4.622777	3.178033
WIPF1	202663_at	4.243665	3.902258	4.213507	3.997878	4.135484	4.210074	3.978127	3.963775	4.033112	4.01838	4.063207	4.099074	5.653146
WIPF1	202664_at	7.918187	7.642558	7.099948	7.43692	8.018031	8.357573	6.647145	6.911796	6.837514	6.599143	7.878479	7.343533	9.379409
WIPF1	202665_s_at	3.114332	3.307814	3.527095	3.526814	3.532492	3.530187	3.542454	3.539218	3.069434	3.529801	3.44614	3.837943	3.891851
ACTL6A	202666_s_at	7.169495	7.048443	7.132711	7.022443	6.800608	6.499988	8.350894	8.208857	8.531599	8.282436	7.869835	7.96037	8.264779
SLC39A7	202667_s_at	3.637461	4.049444	3.57824	3.42563	3.83815	3.844407	4.866928	4.635423	4.683388	4.30716	4.87564	4.844316	4.934735
EFNB2	202668_at	6.411396	6.492899	6.812019	6.74314	6.849163	6.528654	5.901228	5.45386	5.422304	6.01211	6.664865	6.084257	10.37195

EFNB2	202669_s_at	6.193765	5.917414	6.019699	6.275604	6.672904	6.433666	5.336836	5.055368	4.419812	5.127305	6.620819	5.872247	9.307625
MAP2K1	202670_at	5.566818	5.537978	8.388154	8.099116	7.354291	6.44615	6.160429	5.066605	6.245318	6.277405	5.227237	5.203498	7.161914
PDXK	202671_s_at	7.763252	7.577179	7.126193	7.287134	7.181957	7.043244	6.833766	7.125728	6.693797	6.660008	7.274428	7.102395	6.176908
ATF3	202672_s_at	5.825588	5.826855	7.880339	7.432934	7.246756	6.762299	2.343411	2.428167	2.335809	2.328563	2.409493	2.781202	3.237118
DPM1	202673_at	10.253	10.21427	10.76282	10.61658	10.01487	9.264362	10.69238	10.0413	10.80297	10.95875	9.869847	10.06936	10.85056
LMO7	202674_s_at	2.974103	2.932504	3.575346	3.940512	2.963052	2.981062	2.826084	2.90462	2.867502	2.882906	2.863557	2.902563	2.861142
SDHB	202675_at	9.655852	9.537101	10.20855	10.408	9.372336	8.863506	9.244004	8.297153	9.155259	9.197121	8.147722	8.20273	8.578299
FASTK	202676_x_at	6.780184	6.776205	6.051034	6.583505	6.62613	6.885888	6.748343	7.235171	6.575907	6.775427	7.233254	7.312343	6.147064
RASA1	202677_at	8.498481	8.481547	8.435903	8.426739	8.213363	8.316807	9.000118	8.848066	9.085675	8.797517	8.79076	9.007581	9.453098
GTF2A2	202678_at	8.015754	7.893463	8.227853	8.231855	7.378026	6.773585	8.899599	8.083306	8.961424	8.948988	7.71691	8.07951	9.217708
NPC1	202679_at	6.032475	6.181938	6.133125	6.491087	6.359766	5.476918	8.000077	7.672493	8.498636	8.421893	7.382343	8.081537	6.756193
GTF2E2	202680_at	6.393596	6.574061	7.137914	6.890764	6.377336	6.062838	6.289303	6.253471	6.649807	6.66561	6.554512	6.36877	6.761463
USP4	202681_at	6.220905	6.513011	6.030787	5.870066	6.23291	6.453782	6.326362	6.288342	6.244196	6.190418	6.490979	6.916051	5.897694
USP4	202682_s_at	8.52504	8.728203	7.817345	7.838641	8.434687	8.84085	8.191748	8.998034	8.338234	8.410697	9.032508	9.08719	7.917753
RNMT	202683_s_at	7.653956	7.607842	7.712226	7.738074	7.390692	7.422012	7.843911	7.741535	7.930159	7.971388	7.962127	8.096519	8.185536
RNMT	202684_s_at	3.44635	3.123993	3.685975	3.899252	3.775702	3.123649	3.196584	3.203069	3.368868	3.271709	3.371013	3.356096	3.294345
AXL	202685_s_at	2.587187	2.435422	2.9324	2.434555	2.503151	2.962222	2.435222	2.446179	2.363779	2.416709	2.437873	2.428632	3.53594
AXL	202686_s_at	4.65052	4.495285	6.685017	6.627246	5.392966	4.929531	6.004756	4.481399	6.198287	6.061329	4.886696	4.339054	7.783591
TNFSF10	202687_s_at	10.31184	9.95154	9.595576	9.734337	10.40019	10.57129	7.026472	7.738379	7.240045	6.599825	6.724765	6.439683	3.086453
TNFSF10	202688_at	12.77603	12.6845	11.62914	11.91278	12.17973	11.97936	9.91604	10.29187	9.800693	9.529355	9.344421	8.830611	5.294654
RBM15B	202689_at	6.377044	6.207768	6.561611	6.367433	6.367232	6.69519	6.621823	6.66896	6.420889	6.498452	6.430619	6.757247	7.021109
SNRPD1	202690_s_at	8.307334	8.330751	7.969677	8.198172	8.227325	8.246595	10.44946	10.5299	10.23577	10.33527	10.43416	10.70267	10.15735
SNRPD1	202691_at	3.727564	3.400979	3.865574	3.796639	3.203561	3.4293	6.260739	4.763752	5.845853	6.053045	4.858012	5.067884	5.68839
UBTF	202692_s_at	6.41694	6.048676	5.789483	5.533252	6.539605	6.925255	7.375174	7.726588	7.256201	7.072158	7.834915	8.044787	6.841519
STK17A	202693_s_at	6.262134	6.131575	6.014256	6.023361	6.042882	6.617642	8.541449	8.51332	8.880451	8.50111	8.115889	8.56963	6.693728
STK17A	202694_at	2.888496	2.843188	2.806358	2.863078	2.872395	2.795944	2.876333	3.021536	2.8641	3.031671	2.862239	3.485837	2.689006
STK17A	202695_s_at	2.121862	2.286843	2.0998	2.178505	2.204154	2.153884	3.52038	2.244418	3.312878	3.269459	2.348202	3.084496	2.475496
OXSR1	202696_at	5.538635	5.417081	6.198351	5.730464	5.035457	5.054185	7.643868	6.697344	7.840158	7.706701	6.194092	6.607563	7.111529
NUDT21	202697_at	6.621783	6.571742	6.814244	7.084474	6.263128	5.875445	7.889369	6.8933	7.644306	7.773077	6.797885	7.161286	6.991957
COX4I1	202698_x_at	11.90878	11.85774	11.54077	11.66458	11.68339	11.60925	11.4896	11.57922	11.45861	11.50727	11.78414	11.74738	11.42382
TMEM63A	202699_s_at	5.289603	5.00496	4.443005	4.577988	4.222503	4.22126	4.159085	4.226431	3.829653	4.088434	3.581095	4.232074	3.042576
TMEM63A	202700_s_at	6.431493	7.079367	5.970554	6.232877	5.93199	5.786326	5.994561	5.989818	5.849242	5.972741	6.196217	6.347231	4.251817
BMP1	202701_at	6.14552	5.888077	6.442042	6.526883	6.606353	6.703063	4.834375	3.924675	4.890818	3.869984	6.392884	4.890035	6.628524
TRIM26	202702_at	7.218792	7.191106	7.16334	6.829066	6.810683	6.922652	6.627782	5.362796	6.525515	6.000063	5.762078	5.651014	5.567152
DUSP11	202703_at	6.487127	6.501021	6.966865	6.683822	6.232841	5.934978	6.724956	6.133054	6.876745	6.934368	6.397135	6.463752	7.72618
TOB1	202704_at	11.97672	11.73455	12.28075	12.25886	11.45451	11.06341	9.47128	8.910889	9.800283	9.687798	8.520857	8.971622	9.268178
CCNB2	202705_at	4.767425	3.372756	3.02248	3.085874	3.135488	3.125443	10.37689	9.82565	10.31946	10.4168	9.37691	9.89802	8.440997
UMPS	202706_s_at	6.976319	6.988048	7.446077	7.453227	7.118185	6.719834	8.204729	7.758198	8.230688	8.133488	7.573154	8.065787	6.849277

UMPS	202707_at	2.260513	2.219347	2.202355	2.200983	2.216408	2.20378	2.218188	2.218689	2.212924	2.200745	2.203119	2.210722	2.177802
HIST2H2BE	202708_s_at	6.258178	6.233756	5.408468	5.431784	6.18114	5.800707	7.31487	6.960948	7.713341	7.462146	6.37182	6.226393	7.228896
FMOD	202709_at	3.516828	3.511407	3.384719	3.494983	3.631415	4.019158	3.279438	2.93386	3.893947	3.841931	4.728969	3.26072	8.05651
BET1	202710_at	6.605865	6.315002	7.797922	7.457407	6.718269	6.190955	7.574447	6.424833	7.648302	7.580503	6.530887	6.81705	8.165574
EFNB1	202711_at	3.680701	3.27607	3.438447	3.260265	3.347712	3.253273	3.275037	3.27552	3.260265	3.255182	3.450959	3.271458	3.81549
CKMT1A	202712_s_at	2.384893	2.365055	2.327861	2.347886	2.372587	2.343268	2.337061	2.52025	2.352858	2.353254	2.346286	2.357447	3.872183
KIAA0391	202713_s_at	6.544771	6.194289	7.232714	7.402414	7.307618	6.873971	7.322728	7.525266	7.399275	7.538538	7.19672	7.456799	7.048514
KIAA0391	202714_s_at	2.361433	2.304095	2.350359	2.821032	2.345545	2.325783	2.342753	2.334411	2.287697	2.32525	2.313799	2.407441	2.233015
CAD	202715_at	6.233851	6.643639	5.793957	6.213258	5.402369	5.720721	7.536386	7.072854	8.096628	8.027084	7.169484	7.301591	7.736321
PTPN1	202716_at	5.45173	5.491785	5.812429	5.538954	5.731837	5.502063	5.643006	5.972205	5.626595	5.474995	5.721044	5.67714	5.552532
CDC16	202717_s_at	6.746576	6.557905	7.199492	6.935465	6.105208	4.539049	8.618894	7.503198	8.542451	8.538028	7.073947	7.43053	8.401145
IGFBP2	202718_at	12.2134	12.2439	11.66548	11.72958	14.0336	13.66862	9.345102	9.409912	9.876412	9.713157	9.805502	9.971117	10.39784
TES	202719_s_at	3.803317	3.776237	3.850683	3.410342	3.786119	3.75039	3.087688	3.660915	3.002473	3.012018	3.927904	4.626639	6.246459
TES	202720_at	6.358395	6.489228	6.119488	5.860447	6.260433	6.345516	6.812869	6.72266	6.511173	6.547079	6.854177	6.847072	9.460643
GFPT1	202721_s_at	2.353055	1.993968	2.685222	2.335903	2.340015	2.247782	2.357319	2.333012	3.121858	2.340519	2.328672	2.329715	4.053594
GFPT1	202722_s_at	6.788204	6.767289	7.441192	7.239186	6.774707	6.051045	7.358239	7.166694	7.593993	7.484775	6.879735	6.844657	8.328867
FOXO1	202723_s_at	4.786448	5.020476	5.769393	5.429346	4.552055	4.469577	3.023532	3.577502	3.72414	3.69057	3.932687	4.392133	3.624572
FOXO1	202724_s_at	8.162467	8.108928	9.559316	9.4961	8.43582	8.552064	6.33236	6.60961	6.328343	6.320108	7.318236	6.965535	7.348442
POLR2A	202725_at	7.128736	7.124678	6.311564	6.551219	7.162559	7.405773	7.439629	7.789082	7.20871	7.855575	8.090705	7.984516	6.851879
LIG1	202726_at	4.989495	5.093603	4.167545	4.280107	4.090158	5.174967	7.608602	8.166849	7.700068	7.893266	8.022917	8.294537	5.461338
IFNGR1	202727_s_at	9.854492	9.674935	10.00637	9.88003	8.948752	8.595693	9.098805	8.244436	8.73285	8.481656	7.865807	8.509978	9.52045
LTBP1	202728_s_at	4.327547	4.243533	6.145579	6.328084	5.851563	6.074128	7.14685	7.033994	6.208314	6.298468	7.627036	7.658653	8.620043
LTBP1	202729_s_at	7.42342	7.334854	8.497912	8.647201	7.989634	7.663128	9.424233	9.198515	8.841585	9.245831	9.030192	9.167861	11.1629
PDCD4	202730_s_at	4.386967	4.43473	5.969173	5.755693	4.858355	3.812727	7.650234	5.78462	7.699024	7.80478	5.782774	6.419519	7.451057
PDCD4	202731_at	3.984001	3.787775	5.307497	4.350884	3.240475	3.572499	7.109984	4.633035	7.249591	7.158715	4.479465	4.668082	7.101593
PKIG	202732_at	8.936131	8.954455	9.170665	9.259017	9.42592	9.246658	8.281461	8.757136	8.14642	7.971405	8.845815	8.544345	9.111012
P4HA2	202733_at	5.082198	5.003352	5.7347	5.819304	5.585939	5.428525	6.328773	5.625743	5.925825	6.15814	6.79397	6.00958	7.796094
TRIP10	202734_at	6.031098	6.310621	6.014211	6.132972	6.139929	6.177479	6.193132	6.149029	6.090033	6.41621	7.324247	6.784099	5.765291
EBP	202735_at	12.39679	12.4679	11.71053	12.24118	13.28853	13.16493	12.34881	12.8677	12.19959	11.60914	12.49929	12.97551	7.719618
LSM4	202736_s_at	6.491919	6.667526	7.042557	6.862495	6.376168	5.736037	9.596627	7.636176	9.567259	9.428073	7.993317	7.952037	9.181365
LSM4	202737_s_at	7.682422	7.765009	7.44242	7.658371	8.331324	8.2092	9.340615	9.528428	9.264428	9.180021	9.375643	9.537924	8.558691
PHKB	202738_s_at	7.549803	7.157785	7.999545	7.942848	6.99629	6.384523	8.368113	7.449064	8.336235	8.523258	7.645742	7.721112	8.117552
PHKB	202739_s_at	8.520984	8.465953	8.670055	8.679495	8.420162	8.59944	8.217722	8.98427	8.421891	8.594455	8.888491	8.937891	7.995968
ACY1	202740_at	10.22251	10.26854	10.78753	10.66557	9.749645	9.245504	9.264329	8.700628	9.150096	9.261968	8.174656	8.738323	5.548756
PRKACB	202741_at	7.977659	7.852113	8.565042	8.622356	8.565945	8.158273	8.431461	8.016379	8.385044	8.446506	8.066736	7.92818	10.35544
PRKACB	202742_s_at	3.103032	2.824644	3.438825	3.389637	3.292671	3.533134	3.29974	3.038317	3.469333	3.102431	3.071884	3.10793	7.007883
PIK3R3	202743_at	4.815948	4.803059	4.782645	4.785224	4.923946	4.788737	4.55705	4.790142	4.79123	4.772332	5.671442	4.624618	8.267218
SLC20A2	202744_at	4.659276	4.980723	5.712922	5.647682	3.888798	3.231985	3.364187	3.367718	3.868764	3.415538	2.734633	3.073274	4.531987

USP8	202745_at	8.006035	7.915241	7.978979	8.0938	8.044996	7.584665	8.20275	7.596767	7.885437	7.783214	7.711264	7.724355	7.65263
ITM2A	202746_at	4.523229	4.266434	4.623536	4.241789	5.671105	5.932403	6.438102	6.193682	6.336211	6.247756	9.022641	6.156402	11.93103
ITM2A	202747_s_at	3.29397	3.561364	3.934228	3.145968	4.324771	4.211846	5.48217	5.12734	5.191363	5.995492	8.328722	5.032059	11.9624
GBP2	202748_at	7.858877	7.939217	7.150965	7.312437	8.583357	9.320938	4.027103	4.52008	4.055977	4.364078	4.009453	5.070623	4.45341
WRB	202749_at	8.791106	8.672897	9.242811	9.064064	7.846565	6.909403	8.203688	7.479818	8.69987	8.694668	7.577917	7.730501	10.02078
TFIP11	202750_s_at	7.861854	7.744848	7.654256	7.974073	7.892303	8.021539	7.804171	8.537116	8.011455	8.11496	8.219698	8.602044	6.495455
TFIP11	202751_at	2.84388	2.746102	2.674643	2.678695	2.696469	2.677888	2.88395	3.752215	3.611473	2.739716	2.677118	2.718037	3.19475
SLC7A8	202752_x_at	4.858288	5.303365	5.439707	5.419441	5.054028	4.898063	6.085678	5.125818	5.608965	5.617796	5.338632	5.765683	5.152565
PSMD6	202753_at	8.774556	8.670874	9.655942	9.478449	8.768173	8.227868	10.81288	10.20203	10.84521	10.81152	9.963043	10.12113	9.736545
R3HDM1	202754_at	6.912592	6.832138	6.767231	6.849801	6.520783	6.538242	8.286263	7.921228	8.266342	8.170357	7.922472	7.959272	8.803749
GPC1	202755_s_at	2.100182	2.102658	2.093303	2.342694	2.819217	2.084637	2.077795	2.087438	2.075017	2.087599	2.052324	2.081248	2.057253
GPC1	202756_s_at	4.850212	4.39445	4.596161	4.770863	5.744721	5.139619	2.32973	2.645707	2.444276	2.028793	5.244614	2.67608	6.96482
RP13-122B2	202757_at	7.206593	7.200667	6.243129	6.363339	6.808962	7.125377	7.183758	7.642717	7.219022	7.121759	7.72144	7.828335	6.625382
RFXANK	202758_s_at	6.40173	6.36577	6.349038	6.226496	6.500433	6.681459	7.791155	7.953739	7.917583	7.827477	8.324596	8.123048	8.043818
AKAP2	202759_s_at	6.833656	6.887862	8.731058	8.090513	6.589238	6.001726	6.401013	4.701626	6.274615	6.365242	5.453634	4.386608	9.343058
AKAP2	202760_s_at	7.747097	7.472238	8.892424	8.409052	8.077337	7.701944	6.486687	5.680693	5.867193	6.069299	6.984995	5.36511	9.263157
SYNE2	202761_s_at	5.834516	6.02037	6.875161	6.165451	5.382642	4.818011	3.72999	3.448364	3.595131	3.518394	3.942895	2.916751	8.19145
ROCK2	202762_at	7.297611	7.330515	7.398333	7.582661	6.982863	6.398753	7.456592	6.755011	7.595774	7.652972	6.851425	7.176052	7.865636
CASP3	202763_at	7.082571	7.072111	7.271577	7.185693	6.834171	6.496568	8.851904	8.152374	8.699469	8.768534	8.183623	8.267337	7.982587
STIM1	202764_at	5.412164	5.27257	5.090963	5.200684	5.141098	5.596822	4.754791	5.115763	4.668008	5.017346	5.086324	5.128999	4.11677
FBN1	202765_s_at	2.310438	2.275862	2.27733	2.417405	2.303095	2.956208	2.305549	2.293308	2.891393	2.260545	3.510409	2.297338	8.947757
FBN1	202766_s_at	7.443087	7.299704	6.880549	6.674375	6.93911	6.82137	7.12665	6.273023	6.96566	7.371607	9.52894	6.864029	11.60972
ACP2	202767_at	8.674109	8.108063	7.835881	7.893563	8.05885	7.680093	7.713607	7.667639	7.563211	7.712112	7.90116	8.023701	5.821651
FOSB	202768_at	3.223746	4.19339	9.318722	9.016005	10.26764	10.24533	2.235026	2.246829	2.295137	2.287943	2.232781	2.304902	2.131656
CCNG2	202769_at	9.407788	9.320904	8.090562	8.352228	8.683652	8.820759	9.140009	8.833537	8.969769	8.790377	10.87031	8.947327	12.00682
CCNG2	202770_s_at	3.928758	4.021191	4.011917	3.362222	3.286916	3.137512	5.364701	3.122933	5.084823	4.631942	3.459645	3.310199	9.7431
FAM38A	202771_at	6.921979	6.629281	7.046758	6.918428	6.920041	6.836005	8.651862	8.017788	8.585266	8.038399	7.840716	7.595009	6.159263
HMGCL	202772_at	10.81404	10.81392	10.5018	10.60279	10.87201	10.62034	9.12667	9.206948	9.130233	8.984283	8.874215	9.167953	6.109322
SFRS8	202773_s_at	3.301484	3.375404	3.383635	3.358655	3.371067	3.778349	4.557295	4.699975	4.196968	4.430157	5.197469	5.761556	4.080444
SFRS8	202774_s_at	7.228501	7.48615	7.232607	7.314894	7.522658	7.458105	7.87826	7.937709	7.595982	7.703832	8.334598	8.384695	7.489755
SFRS8	202775_s_at	6.460969	6.347716	5.663947	6.15295	6.380121	6.73383	6.756858	6.946869	6.364722	6.371203	6.889003	6.744778	6.158202
DNTTIP2	202776_at	8.395548	8.295786	8.654279	8.746183	8.597373	8.241102	9.108746	9.144475	9.171394	9.125274	8.884641	9.193681	8.703791
SHOC2	202777_at	7.083946	7.072146	7.267019	7.17663	6.748443	6.330763	8.157165	7.480649	8.254479	8.39837	7.167037	7.502946	8.524842
ZMYM2	202778_s_at	8.300373	8.333705	7.587459	7.849369	7.947215	7.971662	7.78322	8.015975	7.77347	7.602053	8.483721	8.136353	9.273979
UBE2S	202779_s_at	4.758946	4.732895	4.42051	3.991829	3.497276	4.097851	9.8807	9.188875	9.755918	10.0722	9.0772	9.361824	7.418802
OXCT1	202780_at	3.444728	3.939271	3.802683	3.801767	4.024902	3.991691	7.129781	7.061426	7.268682	7.292982	7.185242	6.895437	7.995905
SNW1	202781_s_at	4.327769	4.338316	4.639904	4.989021	4.873302	4.307275	5.351575	5.076057	5.997562	5.728645	4.338033	4.937121	4.219976
SNW1	202782_s_at	6.009318	5.874757	5.769716	5.704176	5.784683	5.976291	5.624676	5.985532	5.619106	5.769564	5.78018	6.093105	5.280064

NNT	202783_at	8.720972	8.346713	9.67101	9.181629	7.332078	6.803581	7.573556	6.327319	7.892298	7.815381	5.411202	5.843952	8.240281
NNT	202784_s_at	6.776626	6.206426	8.51837	7.635249	5.193526	4.69166	6.448664	4.710502	6.791772	6.787621	4.694809	4.942484	7.783978
NDUFA7	202785_at	7.436049	7.119065	7.451031	7.409726	7.278981	7.245657	8.148417	7.624485	8.037471	8.183566	7.468428	7.932213	6.726822
STK39	202786_at	3.030314	3.2528	2.982503	2.878716	3.565607	4.206641	4.689533	4.329492	4.905304	4.421365	6.285355	4.762637	8.5348
MAPKAPK3	202787_s_at	5.97576	5.686083	5.116525	4.847641	4.821051	5.043542	5.078677	5.575347	5.487081	5.255083	5.14212	5.215629	4.983346
MAPKAPK3	202788_at	6.831873	7.08701	6.76815	6.91216	7.122822	7.440092	7.058172	7.591526	7.096319	7.262137	7.867104	8.01276	6.782841
PLCG1	202789_at	5.47054	5.723681	4.679378	4.448365	5.733791	5.715864	6.199483	6.548603	6.13095	6.080363	7.390967	7.104973	7.277781
CLDN7	202790_at	3.228415	3.753004	3.189182	3.193794	3.520329	4.531417	2.284071	2.400776	1.863345	2.614177	2.245791	2.320675	2.852009
SAPS2	202791_s_at	7.029701	6.817274	6.561039	6.847993	6.906204	6.946996	6.790121	6.847791	6.786941	6.761	6.921166	6.970413	4.798101
SAPS2	202792_s_at	7.643575	7.866105	7.211202	7.504574	8.030381	7.792196	7.245139	7.325268	6.89794	7.102148	7.626977	7.891003	5.21327
MBOAT5	202793_at	6.236746	5.63706	5.932799	5.379938	4.310234	4.123642	6.378408	5.070196	6.490745	5.877171	3.838668	4.940372	3.917892
INPP1	202794_at	8.128892	8.190742	8.781467	8.66803	7.958317	7.722728	8.283441	7.827407	8.302508	8.49332	7.545033	7.692969	7.397609
TRIOBP	202795_x_at	8.031135	8.078047	6.895752	7.066648	7.547538	7.581987	7.461077	7.981463	7.382827	7.282633	7.969683	8.053908	7.543061
SYNPO	202796_at	8.680677	8.928102	7.867514	7.90652	8.638951	8.738117	3.649094	3.710893	2.531171	3.135444	5.629883	3.405694	7.021961
SACM1L	202797_at	9.464354	9.506614	9.595696	9.455934	9.398787	8.959567	9.43567	9.49375	9.56708	9.659058	9.204057	9.337116	9.480001
SEC24B	202798_at	10.41613	10.24406	9.810495	9.89247	8.817922	7.857098	8.650967	7.812411	8.760354	8.730291	8.041563	7.526043	9.551763
CLPP	202799_at	7.288242	7.058062	7.653702	7.723942	7.887563	7.856328	7.107854	7.564489	7.407565	7.240767	7.78831	7.70829	7.047545
SLC1A3	202800_at	3.358038	3.405037	3.558404	3.591835	3.62671	4.081672	3.604476	3.600031	2.889445	3.565638	4.09778	3.597026	9.097466
PRKACA	202801_at	7.485843	7.418049	7.602981	7.348639	7.580388	7.796497	6.175312	6.765849	6.309055	6.17757	7.060543	6.515575	6.983871
DHPS	202802_at	6.435593	6.554297	6.499317	7.171289	6.440547	6.385982	6.227688	6.258957	6.474977	6.430973	5.997194	6.569054	6.311784
ITGB2	202803_s_at	5.730388	5.463095	6.305096	6.051838	6.423726	6.858142	5.59282	5.590909	5.528061	4.866768	4.755066	5.299654	4.192719
ABCC1	202804_at	5.493543	4.891968	4.958495	5.133862	5.365866	5.323004	5.123925	5.424365	5.705531	4.707573	5.588958	5.199073	6.733433
ABCC1	202805_s_at	2.858966	2.818657	2.820617	2.825691	2.834357	2.782255	2.838242	2.831662	2.823988	2.823729	2.82276	2.829876	4.338305
DBN1	202806_at	3.866747	4.177851	3.175844	3.551889	4.256615	4.753665	5.491763	5.782954	4.401936	4.587358	8.777195	6.443252	9.636627
TOM1	202807_s_at	3.651631	3.526632	3.210833	3.488908	3.462561	3.678865	2.566222	3.405334	2.712717	2.992583	3.808734	3.290462	2.084449
INTS3	202809_s_at	7.851851	8.049099	6.969387	7.265789	8.41235	8.382918	7.723845	7.650751	7.488391	7.268152	8.600602	8.144935	8.741963
DRG1	202810_at	8.052875	8.0564	7.986183	8.027785	8.046579	8.210451	8.624587	8.843142	8.7459	8.993005	8.681547	9.138015	8.291647
STAMBP	202811_at	6.224317	6.473162	6.586589	6.62826	6.157787	6.331695	6.632046	6.995954	6.760217	6.935476	6.556958	6.802877	7.041505
GAA	202812_at	7.812329	7.532452	7.765682	7.944725	8.008631	7.541284	5.373132	4.619531	5.365019	5.274385	5.776907	5.606825	4.64508
TARBP1	202813_at	6.589923	6.507561	6.283562	6.580713	5.513048	5.918875	6.87935	6.556206	7.380266	7.013535	6.906125	6.463423	7.301376
HEXIM1	202814_s_at	7.092556	7.130445	8.118935	7.499036	8.008389	7.712306	9.234116	9.026483	9.706153	9.075785	8.292377	8.634431	8.197052
HEXIM1	202815_s_at	5.142727	5.266441	5.575214	5.606102	5.377908	5.553131	6.009878	5.809912	6.259683	6.19135	6.251585	6.149193	6.200609
SS18	202816_s_at	6.808315	6.672432	6.803448	6.585291	6.535305	6.222316	6.619681	5.469155	6.27747	6.576726	5.703833	5.97588	8.149719
SS18	202817_s_at	7.366912	7.207575	7.996483	7.559815	6.181685	5.234025	8.063038	6.089157	7.820321	7.913778	5.924332	5.678073	8.846168
TCEB3	202818_s_at	7.175409	7.319473	7.018882	7.534515	7.883758	7.964766	6.8087	7.551786	6.394377	6.233234	7.371634	7.297917	5.60417
TCEB3	202819_s_at	5.802872	5.546653	5.837915	5.782553	5.354298	5.725908	5.905309	5.926345	6.173953	6.208538	5.769975	5.810782	5.205405
AHR	202820_at	10.61896	10.48717	10.65175	10.30644	9.899471	9.309671	7.712169	7.295156	7.563605	7.809862	7.265225	7.154515	8.416509
LPP	202821_s_at	4.544251	4.259238	4.076744	4.104485	5.808655	5.997002	3.281387	4.100423	2.592739	2.399049	4.467985	4.414935	2.138715

LPP	202822_at	7.817766	7.783708	7.741776	7.766534	8.060738	7.656165	6.573588	6.592093	7.060321	6.721227	7.101983	6.77239	7.009194
TCEB1	202823_at	6.770031	6.804523	7.079323	6.811598	5.870438	6.299561	7.813304	7.275627	7.84627	7.872645	7.070683	7.39869	8.340896
TCEB1	202824_s_at	9.980803	9.86025	10.1917	10.21234	9.882366	9.601824	10.87312	10.37863	10.76376	10.81454	10.07603	10.40556	10.69492
SLC25A4	202825_at	7.311921	7.453143	7.836229	7.899404	7.377694	6.713297	6.878927	6.447055	7.155402	6.849556	6.86108	6.492542	8.334541
SPINT1	202826_at	2.793384	2.692959	2.654073	2.657067	2.825721	2.670602	2.681798	2.69209	2.677095	2.651242	2.672568	2.694027	3.741385
MMP14	202827_s_at	2.148083	2.13842	2.119101	2.124313	2.13562	2.122081	2.137649	2.137979	2.124257	2.125413	2.124255	2.13153	2.807531
MMP14	202828_s_at	2.329072	2.446359	2.28097	2.288891	2.31661	2.501073	2.294954	2.307666	1.99675	2.291127	2.294573	2.331366	2.240745
SYBL1	202829_s_at	7.769022	7.777832	8.793339	8.492959	7.130584	6.268547	8.232242	6.912349	8.396751	8.248897	6.168519	6.319134	8.071018
SLC37A4	202830_s_at	10.10063	10.07726	9.927439	10.05117	9.762148	9.382205	9.161574	8.62956	8.862523	9.008454	8.215581	8.481588	4.672655
GPX2	202831_at	7.186676	6.92392	8.071837	8.097123	6.484622	6.883546	2.617736	2.674345	2.625472	2.630495	2.624907	2.826376	2.610705
GCC2	202832_at	7.319794	7.423339	7.491844	7.430467	6.68646	6.427582	7.517197	7.265	7.464307	7.864329	6.911843	7.077163	7.109188
SERPINA1	202833_s_at	15.09163	15.12653	15.08186	15.22335	15.14093	15.04059	15.2	15.0665	15.22532	15.23406	14.8573	15.06137	12.18251
AGT	202834_at	13.10107	13.16942	13.06412	13.19722	12.95369	12.52048	13.4573	13.28175	13.59652	13.62275	13.09665	13.17236	8.001906
TXNL4A	202835_at	3.056789	3.052345	3.030669	3.130463	3.045106	3.034982	3.208032	3.045092	3.039259	3.032809	3.03049	3.039158	3.004373
TXNL4A	202836_s_at	9.309604	9.150501	9.256446	9.643741	9.654305	9.720334	10.1174	10.53565	10.32915	10.29502	10.72478	10.66723	9.784133
TRAFD1	202837_at	2.965174	2.792612	2.682239	2.782958	2.792853	2.798385	2.791965	3.537039	3.122452	2.779824	4.266704	3.670224	2.827331
FUCA1	202838_at	9.607573	9.685597	10.10613	10.33247	9.908219	9.463872	9.031036	8.823079	8.567351	9.007031	8.012555	8.6804	7.046612
NDUFB7	202839_s_at	9.998568	9.965499	10.04171	10.2366	10.09477	10.04785	9.956268	9.907501	9.964998	9.968514	10.1417	10.22967	9.613081
TAF15	202840_at	5.791107	5.89374	5.723396	5.671915	5.985022	6.17184	6.012068	5.764079	5.292978	5.556882	6.820497	5.843031	4.343331
OGFR	202841_x_at	6.086105	6.058829	5.237021	5.244991	5.663795	6.092638	4.4871	5.210799	5.272255	4.881456	4.684416	5.210749	4.474545
DNAJB9	202842_s_at	9.219381	9.061801	10.5925	10.48375	9.92496	9.639392	9.530919	9.235099	9.82804	9.876711	8.692552	9.333514	8.660595
DNAJB9	202843_at	7.911098	7.909231	9.391638	9.155622	8.291873	7.578373	7.645096	7.555586	8.020765	7.980403	6.98411	7.668983	7.048383
RALBP1	202844_s_at	8.450904	8.167922	7.968816	8.330171	8.822753	8.965931	8.303193	9.191207	8.255059	8.399187	9.31691	9.504683	7.395423
RALBP1	202845_s_at	7.241528	7.170906	7.593241	7.447154	7.275134	7.072242	8.014125	7.563608	8.210545	8.186216	7.655284	7.595023	7.717306
PIGC	202846_s_at	6.981445	6.899055	6.20118	6.360678	6.570787	6.55074	6.990726	7.028027	6.791215	7.463493	7.119695	7.429505	7.479209
PCK2	202847_at	11.5306	11.53489	11.64977	11.9426	11.55633	11.39984	8.402075	8.568677	8.35673	8.179161	8.239253	8.338476	3.29196
GRK6	202848_s_at	2.401759	2.50534	2.335134	2.45871	2.351258	2.497569	3.283728	4.516432	4.466497	4.027892	3.448477	4.218519	3.185956
GRK6	202849_x_at	6.399729	6.394483	5.717757	5.860132	6.100141	6.374798	6.41608	7.019838	6.464884	6.648315	7.037024	6.623555	6.322101
ABCD3	202850_at	11.42209	11.46369	11.20761	11.22299	11.14045	10.67596	10.9485	10.09307	10.91815	10.80019	10.09653	10.10422	9.968195
FLJ11506	202851_at	3.078712	3.069662	3.016539	3.05683	3.091315	3.00095	3.058239	3.06964	3.062269	3.282216	3.073316	3.417456	2.938373
FLJ11506	202852_s_at	6.67491	6.41612	6.40587	6.562873	6.672826	6.695559	7.286565	7.088567	7.263288	7.681996	7.525846	6.911257	6.52675
RYK	202853_s_at	8.490569	8.488283	8.791604	8.61026	8.44338	7.962621	8.288448	8.01594	8.204592	8.108584	8.526443	7.866573	9.838255
HPRT1	202854_at	8.929301	8.918348	8.733693	8.678131	9.545342	8.633703	9.180725	8.311218	9.441526	9.518251	7.903586	8.539282	8.136192
SLC16A3	202855_s_at	2.211117	2.199179	2.178072	2.181697	2.205133	2.185717	2.197763	2.196339	2.191698	2.181093	2.182446	2.189539	2.166225
SLC16A3	202856_s_at	3.785007	3.645465	3.742033	3.720438	4.060499	3.847084	3.862093	3.904009	3.784459	4.013303	4.441346	4.376314	3.785886
TMEM4	202857_at	10.47578	10.54746	10.77768	10.81118	10.52541	10.40985	10.31527	10.05847	10.21533	10.42375	10.18074	10.30535	9.990095
U2AF1	202858_at	10.15093	10.05779	10.00493	9.994018	10.29268	10.41143	10.78442	11.32526	10.78871	10.67412	11.35949	11.41775	10.19504
IL8	202859_x_at	3.327236	3.216687	6.298872	4.642221	4.279862	4.426098	2.876988	2.877431	2.868659	2.859586	2.859429	2.870116	8.248692

DENND4B	202860_at	7.438164	7.642698	6.901025	6.904292	7.20845	7.51312	7.613074	7.456086	7.384983	7.384054	7.20708	7.293193	6.756348
PER1	202861_at	7.564395	7.565768	8.123369	8.239433	6.960877	6.463803	4.545833	4.203012	4.907705	4.168978	4.858278	4.293311	4.913128
FAH	202862_at	11.07027	11.0748	11.06433	11.17388	12.12932	12.10662	9.40384	10.03258	9.746516	9.954406	9.609142	10.22076	5.901232
SP100	202863_at	9.071776	9.020427	9.039726	9.148234	8.974485	8.598536	8.057309	7.936105	7.896107	8.519884	7.125664	7.590505	4.889136
SP100	202864_s_at	8.254417	8.270922	7.953128	8.133396	8.262458	8.284301	6.845799	6.95721	7.21372	7.725579	6.740411	7.186169	4.663302
DNAJB12	202865_at	4.76461	4.838093	4.378711	4.693199	4.855921	4.694264	4.552565	4.92816	4.61576	5.031756	4.530201	4.861994	4.428704
DNAJB12	202866_at	7.032706	6.687446	6.771864	6.928618	6.55858	6.409423	6.805314	6.625872	6.856232	6.849515	7.085669	6.828085	6.081426
DNAJB12	202867_s_at	5.201477	5.506964	5.750278	6.067616	6.528258	5.883441	5.543726	6.239219	6.001889	5.563683	5.733693	6.520407	5.024941
POP4	202868_s_at	7.937757	7.967744	8.393958	8.384698	8.42471	8.249598	8.598969	8.660157	8.713669	8.67257	8.66743	8.860906	7.873454
OAS1	202869_at	5.152496	5.056879	5.777684	5.918083	3.802318	3.68231	1.647791	1.875353	3.423913	2.761629	2.232308	1.794443	1.621639
CDC20	202870_s_at	3.271074	2.353577	2.347551	2.308165	2.330821	2.329851	9.479009	10.11162	9.778616	9.893284	9.816464	9.960073	6.692814
TRAF4	202871_at	6.765319	6.249032	5.7707	5.745122	6.282322	6.297657	5.032448	4.968624	4.426934	4.802081	6.536026	5.617344	5.256261
ATP6V1C1	202872_at	6.47431	6.292632	6.457677	6.193767	5.825682	5.362688	6.593175	6.196062	6.74278	6.664091	5.804462	6.325438	6.335718
ATP6V1C1	202873_at	2.860251	2.845546	2.825409	2.829103	2.851941	2.827883	2.968308	2.84922	2.850694	2.826682	2.822672	2.845186	3.139679
ATP6V1C1	202874_s_at	5.244059	5.564529	6.23053	5.937038	4.698569	3.348816	7.459896	5.312875	7.035945	7.470467	5.302844	5.618973	7.027291
PBX2	202875_s_at	5.058921	4.884305	4.815347	4.992342	5.264253	5.474245	6.711925	6.827586	6.229479	6.462229	6.545209	7.178084	5.217544
PBX2	202876_s_at	4.879041	4.891225	4.380832	4.341533	5.192978	4.864622	4.876716	6.096049	4.911653	5.21194	5.371955	5.577106	5.203954
CD93	202877_s_at	2.941158	2.893762	3.202444	3.185884	3.37196	4.427784	3.022658	2.921879	2.902198	3.183053	3.220949	3.19994	4.109006
CD93	202878_s_at	7.428235	7.527776	7.970294	7.606594	8.160381	7.758123	6.830902	5.905569	7.321893	6.372894	7.021491	6.585831	8.729262
PSCD1	202879_s_at	2.396642	2.365733	2.326598	2.346751	2.365695	2.348622	2.360954	2.364873	2.351208	2.34469	2.34746	2.360561	2.23783
PSCD1	202880_s_at	7.339013	7.231929	6.895525	7.169838	7.064391	7.011778	6.020113	6.124344	6.059832	5.606003	6.549852	6.163914	5.453951
NOL7	202882_x_at	7.93338	8.099025	8.373092	8.524433	8.215762	7.770995	9.905559	9.279558	9.944884	9.975587	8.909535	9.337403	9.265618
PPP2R1B	202883_s_at	10.05121	10.09748	9.958327	9.865883	9.771762	9.514097	9.721221	9.123035	9.522454	9.546413	8.83049	9.164427	6.701141
PPP2R1B	202884_s_at	6.254504	6.503896	7.036764	6.683834	5.584669	4.890714	5.670982	4.36316	6.089279	6.075992	3.320434	3.882657	3.834575
PPP2R1B	202885_s_at	2.142132	2.136804	2.108642	2.119027	2.142829	2.116333	2.121259	2.111591	2.123692	2.088976	2.113891	2.122931	2.042274
PPP2R1B	202886_s_at	8.43788	8.248978	8.412148	8.495813	7.560111	7.567491	6.952191	6.134719	7.183473	7.172111	5.613327	5.850728	4.944381
DDIT4	202887_s_at	7.033272	6.498191	8.430662	8.700686	6.939403	6.717804	5.787599	5.772188	5.479995	5.928733	7.064854	6.443186	8.961634
ANPEP	202888_s_at	11.36921	11.42219	11.54884	11.7868	11.40925	11.17438	9.473373	9.803304	9.312626	9.399659	9.513153	9.634329	5.036844
MAP7	202889_x_at	3.725912	3.708359	4.749268	4.09697	3.968509	3.698443	3.241108	3.517556	3.269371	3.216521	3.165101	3.176331	3.086688
MAP7	202890_at	8.980096	8.762666	8.815119	8.913201	8.968106	8.955832	5.964953	7.052939	6.548547	6.631993	6.473222	6.670356	5.846287
NIT1	202891_at	9.00755	9.020012	9.298402	9.29954	8.872784	8.857475	8.688879	8.477926	8.593521	8.639895	8.336998	8.592856	7.196074
CDC23	202892_at	8.021385	7.804727	7.728731	7.663662	7.255302	6.628118	9.11949	8.714989	9.24267	9.079674	8.409497	8.667415	8.98879
UNC13B	202893_at	7.747679	7.721675	7.009904	7.138452	6.94846	7.114405	7.605059	7.559321	7.548305	7.65851	7.363799	7.364288	6.36951
EPHB4	202894_at	8.707088	8.40791	7.896324	8.105978	8.688293	8.781262	8.534569	8.313506	8.480725	8.34448	8.860654	8.99917	7.888488
SIRPA	202895_s_at	2.121478	2.10974	2.083421	2.09497	2.108521	2.088149	2.098683	2.099515	2.092351	2.084788	2.086978	2.102174	2.050308
SIRPA	202896_s_at	6.7391	7.036333	6.615917	6.869517	6.97584	6.82367	4.807273	4.729839	4.697547	4.425653	5.248325	5.313662	4.690507
SIRPA	202897_at	5.03452	4.93842	5.879024	5.070101	4.911403	5.620212	4.344563	3.59498	4.334532	4.318728	4.32953	4.33639	5.321123
SDC3	202898_at	6.850389	6.492854	6.658387	6.778026	7.588744	7.326807	6.083353	6.470782	6.247408	6.419151	6.577566	6.403453	7.013542

SFRS3	202899_s_at	7.549069	7.088697	8.238066	7.707354	7.354573	7.204927	10.61373	8.769906	10.48799	10.49531	9.202119	8.804767	11.89852
E2F1	2028_s_at	2.713365	2.699059	2.603516	2.678353	2.701282	2.642126	3.561945	5.33072	3.770989	4.658026	4.511694	4.402427	2.6629
NUP88	202900_s_at	8.446179	8.481358	9.262983	9.094361	9.479934	8.99655	9.546082	9.562521	9.74495	9.676545	9.283103	9.770276	8.306575
CTSS	202901_x_at	8.460246	8.249779	8.421794	8.235115	8.533029	8.23471	6.750631	7.248848	6.502852	6.650655	5.841245	6.116405	4.413431
CTSS	202902_s_at	6.707201	6.168722	8.233456	7.633121	6.794647	6.794613	6.46905	4.915726	6.049698	6.342511	2.63412	3.901285	3.842332
LSM5	202903_at	5.109476	4.495432	4.826987	5.072896	5.167537	5.082743	5.177334	5.417456	5.577123	5.176843	5.948509	6.003084	6.725032
LSM5	202904_s_at	6.334366	6.583414	6.334855	6.612213	5.992418	6.171646	7.321771	7.503704	7.55138	7.633103	7.266325	7.495584	7.198505
NBN	202905_x_at	6.37309	6.223577	6.412124	6.359721	6.893188	6.415147	6.566307	6.377705	6.267003	5.729929	6.164135	6.120964	6.518002
NBN	202906_s_at	7.613342	7.57562	7.224957	7.654887	7.259545	7.259607	7.642485	6.978876	7.371886	7.024036	6.700726	6.716769	7.67365
NBN	202907_s_at	7.843551	7.708218	8.187528	8.258222	7.19493	7.115563	8.306191	7.916973	8.652636	8.674053	7.705921	7.78939	8.903596
WFS1	202908_at	5.08644	4.90461	5.163846	5.706934	6.200642	5.992084	6.996631	8.109959	7.415971	7.2064	8.124831	8.129949	6.258231
EPM2AIP1	202909_at	8.802186	8.978615	8.478581	8.378669	8.827954	8.688365	9.543174	9.384918	9.515871	9.578796	9.584104	9.458851	10.09392
CD97	202910_s_at	4.757111	5.071262	5.785389	5.882385	5.991884	6.161612	2.635893	3.425212	3.240367	3.416998	5.050699	3.526885	3.929717
MSH6	202911_at	7.936778	7.940628	8.095395	7.951585	7.62919	7.469212	9.807009	9.507968	9.971279	9.833135	9.299225	9.44131	9.45899
ADM	202912_at	7.348715	7.103313	7.850058	7.738752	7.036648	7.267621	5.313455	5.883022	6.024741	5.784644	6.712416	5.892207	7.141043
ARHGEF11	202913_at	5.640177	5.498203	5.263445	5.498218	5.196311	5.004849	5.070031	5.007886	5.101864	5.025724	5.109214	5.088173	5.504008
ARHGEF11	202914_s_at	2.565871	2.523017	2.316788	2.237101	2.294768	2.198891	2.39976	2.214375	2.220665	2.199269	2.204522	2.217843	3.197373
FAM20B	202915_s_at	6.997022	7.055109	7.042987	6.805793	6.864087	6.712796	8.82588	7.482686	8.521412	8.49559	7.135315	7.590186	7.99442
FAM20B	202916_s_at	7.433421	7.389709	7.357432	7.540642	7.467249	6.688911	8.772632	8.801846	8.755644	9.083329	8.599364	8.880231	7.739867
S100A8	202917_s_at	5.717825	5.670056	9.751996	9.078468	8.720956	6.503507	8.449735	7.612191	8.160281	9.067125	8.939624	9.251433	8.567029
MOBKL3	202918_s_at	7.85208	7.78504	8.26861	7.772389	7.583547	7.221313	9.450992	8.378937	9.529926	9.592304	8.196385	8.671586	10.90643
MOBKL3	202919_at	6.030836	5.913499	6.202304	6.15329	5.839749	5.287341	7.105039	6.610966	6.888006	7.039732	6.513961	6.712036	8.199285
ANK2	202920_at	3.392746	3.958433	4.13009	3.960041	4.248655	4.92271	3.265339	3.509002	3.178859	3.822855	7.456076	3.854382	10.31584
ANK2	202921_s_at	2.311923	2.29806	2.269193	2.281909	2.317466	2.276582	2.295679	2.301911	2.286875	2.288447	2.286383	2.295252	6.764963
GCLC	202922_at	9.144444	9.097934	9.580549	9.228558	8.216133	7.344683	9.558613	8.912305	9.580477	9.493816	8.195059	8.769726	6.200039
GCLC	202923_s_at	9.828004	9.928601	9.549682	9.367429	8.540389	8.032343	11.08091	10.02624	10.94971	10.96594	9.202306	9.841303	6.50285
PLAGL2	202924_s_at	2.364659	2.415092	2.395072	2.334262	2.350702	2.381891	3.084876	4.069959	3.785558	3.610111	3.490568	3.507799	2.339475
PLAGL2	202925_s_at	3.510181	3.282163	3.202034	3.203502	2.820309	3.013181	6.185039	5.920651	6.336301	6.304094	5.406636	5.825718	5.039247
NAGLU	202926_at	6.67026	6.451193	6.622766	6.691084	6.697151	6.677748	8.419803	8.50568	8.531572	8.235534	8.472318	8.607211	7.479735
PIN1	202927_at	7.319957	7.122362	7.118458	7.407539	7.808782	8.044937	8.390149	8.988367	8.343242	8.606429	8.669808	9.189571	8.134011
PHF1	202928_s_at	3.94144	3.707208	4.013512	3.736504	3.858973	3.985977	3.815587	3.64457	3.637754	3.730965	3.763092	3.70765	3.826708
DDT	202929_s_at	10.79582	10.80868	10.71608	10.79924	10.14494	10.01105	9.119889	8.944153	8.995379	9.385077	9.129551	9.8529	7.518846
SUCLA2	202930_s_at	8.098457	8.18766	9.061266	8.812238	8.094103	6.926503	9.288117	8.344289	9.309315	9.291815	8.394024	8.288866	9.520498
BIN1	202931_x_at	5.321597	5.393648	5.378797	5.477471	5.461064	5.616048	5.219787	5.45984	4.872038	4.981376	7.523044	5.934209	7.572569
YES1	202932_at	8.855193	8.608822	9.670117	9.302915	7.712074	6.352116	8.316854	6.381157	8.185185	8.117314	6.874719	6.429975	9.324187
YES1	202933_s_at	10.72881	10.67163	10.3045	10.52176	9.910953	9.717377	9.133247	9.403822	9.228578	9.128294	9.722012	9.373778	10.10304
HK2	202934_at	3.336924	3.392986	2.857303	2.73959	3.680818	3.480589	3.90821	4.721422	4.133543	4.788555	4.564875	4.520146	5.439376
SOX9	202935_s_at	3.940448	4.521322	3.490709	3.580408	4.075123	3.584857	3.903655	3.145408	2.56003	2.859225	3.579322	3.582183	9.062284

SOX9	202936_s_at	4.679456	5.032385	4.309754	4.498124	4.958708	4.667506	4.600599	4.017188	3.677162	4.054	4.312583	4.175118	8.874268
CTA-126B4.	202937_x_at	5.544271	5.520049	4.462456	4.584489	5.760076	5.997475	5.781977	7.220921	6.551975	5.985638	6.280674	7.521615	4.562965
CTA-126B4.	202938_x_at	2.254216	2.251265	2.19742	2.209673	2.242492	2.213172	2.727552	2.243887	2.687778	2.766836	2.227981	2.804803	2.935804
ZMPSTE24	202939_at	8.954196	8.903572	9.493134	9.532153	8.506723	8.407838	9.356572	8.669202	9.243701	9.273234	8.769	9.024438	9.1081
WNK1	202940_at	3.386671	3.370622	3.045243	2.914229	3.184957	3.305113	4.053657	4.20269	5.036349	4.354907	4.193647	3.628678	3.649236
NDUFV2	202941_at	10.42991	10.41807	10.80874	10.65768	9.881335	9.148958	10.22538	9.222253	10.15516	10.23376	9.029941	9.491147	9.213479
ETFB	202942_at	11.15266	11.12261	10.88783	11.1138	11.59476	11.7003	10.33984	10.90873	10.55836	10.56074	11.04086	11.18815	7.502422
NAGA	202943_s_at	6.042639	5.97754	6.695027	6.336742	6.631214	6.265588	6.014737	5.863234	5.927333	6.041356	5.217353	5.676107	5.956022
NAGA	202944_at	6.046539	5.921986	6.639916	6.776672	6.339142	5.883786	5.917372	5.011014	5.914469	5.896754	5.239545	5.399575	5.952976
FPGS	202945_at	6.614587	6.332431	6.447056	6.726148	7.305189	6.859226	6.084907	6.396343	5.59748	5.539771	6.567805	6.448854	4.741363
BTBD3	202946_s_at	7.090664	7.030019	6.944611	7.269479	6.854308	6.445577	8.098061	7.770791	8.271947	7.966458	8.347988	7.928107	10.73609
GYPC	202947_s_at	6.381649	6.028092	6.472804	6.32726	6.616058	6.678834	12.63003	12.00025	12.48863	12.47768	11.53338	12.08506	8.431852
IL1R1	202948_at	9.756284	9.635752	10.02604	9.814174	9.579291	8.748471	7.170194	6.290361	7.233547	7.628327	6.459182	6.569346	8.18087
FHL2	202949_s_at	4.205734	4.147349	5.802293	5.496082	6.976845	6.045938	5.880869	5.892816	6.297294	6.731945	6.30515	5.453517	9.082016
CRYZ	202950_at	10.48195	10.35108	11.254	11.01871	9.707124	9.064703	10.76139	9.861867	11.07596	11.16496	9.620769	9.879629	8.340603
STK38	202951_at	8.445285	8.53521	8.091073	8.415178	8.934586	8.432228	8.464983	8.516355	8.459163	8.560773	8.686498	8.648712	8.979372
ADAM12	202952_s_at	2.4131	2.380643	2.351376	2.369202	2.399142	2.351619	2.371259	2.383201	2.394361	2.358696	2.434234	2.38246	5.567617
C1QB	202953_at	7.752882	7.840155	9.895459	10.13701	10.11878	10.00782	8.400948	8.837744	8.866566	8.664544	7.625241	8.146492	5.286731
UBE2C	202954_at	5.070589	5.464285	4.127941	4.339296	4.183416	4.156107	10.05869	9.063605	10.2296	10.00252	8.937768	9.279806	9.223882
ARFGEF1	202955_s_at	2.202331	2.193013	3.512794	2.497378	2.14773	2.134028	3.127699	2.145192	3.029044	3.120963	2.126171	2.13925	5.5656
ARFGEF1	202956_at	7.488937	7.570496	7.426241	7.608808	7.416856	7.417907	7.5678	7.361037	7.375054	7.485906	7.627235	7.488042	7.827854
HCLS1	202957_at	6.683604	7.039977	8.186026	7.938485	8.226142	8.083714	6.031962	6.800362	6.75432	6.732953	5.874349	6.59589	4.014283
PTPN9	202958_at	4.696301	4.684195	5.011939	5.216203	4.765763	4.449148	4.902712	4.136274	4.669194	4.66386	4.746795	3.665259	6.048436
MUT	202959_at	11.29938	11.40458	11.1498	11.49119	11.39272	11.13374	9.50763	9.61943	9.371236	9.290367	9.165187	9.274386	7.935175
MUT	202960_s_at	12.48347	12.3323	12.44307	12.46986	12.33127	12.15202	10.81258	10.55934	10.79891	10.24252	10.34123	10.56161	9.1294
ATP5J2	202961_s_at	10.44738	10.40295	10.5461	10.64579	10.58896	10.40111	11.73202	11.4255	11.64709	11.58703	11.48773	11.45497	10.95843
KIF13B	202962_at	7.736555	7.531369	7.358597	7.540396	7.441058	7.121171	6.535664	6.600488	6.5999	6.490657	6.653836	6.713566	4.864461
RFX5	202963_at	5.60856	5.720321	5.281138	4.741232	4.764059	5.433115	6.792982	6.390501	6.813453	7.21163	6.295454	6.597556	6.866273
RFX5	202964_s_at	4.035459	4.030126	3.670542	3.923755	4.022948	4.331442	4.063311	4.029621	4.468252	4.225732	4.71472	4.766934	4.640113
CAPN6	202965_s_at	2.09274	2.07415	2.048308	2.058034	2.075724	2.056504	2.071352	2.073068	2.05949	2.05218	8.218947	2.07488	10.85617
CAPN6	202966_at	2.603653	2.549284	2.528305	2.55251	2.5922	2.536619	2.549172	2.546573	2.554368	2.525557	4.778261	2.611949	9.48709
GSTA4	202967_at	8.224951	8.102991	7.946199	7.982323	7.479279	6.984545	9.751365	8.818978	9.546372	9.455354	8.886688	9.311889	10.98442
DYRK2	202968_s_at	3.528444	3.507871	3.341703	3.298856	2.967285	3.429237	3.525422	3.48459	3.644011	3.88229	4.086	3.648225	6.332338
DYRK2	202971_s_at	4.315866	3.727748	4.527302	4.229844	4.396995	4.11031	4.799415	4.210915	4.87322	4.928506	5.501291	4.298862	8.047916
FAM13A1	202972_s_at	7.657308	7.552819	8.828168	8.758896	7.453178	7.204293	6.553787	5.651455	6.509359	6.618799	5.953605	5.625404	6.498518
FAM13A1	202973_x_at	10.50664	10.33357	11.40681	11.37515	10.50291	9.960747	8.787104	8.68188	9.049834	9.080805	8.427341	8.047361	8.937618
MPP1	202974_at	7.000191	7.140294	7.138315	7.173622	6.572503	6.551935	10.60289	10.07843	10.80281	10.81761	9.663539	9.965675	6.207747
RHOBTB3	202975_s_at	8.344444	8.164682	9.216191	9.118467	7.521113	7.029611	8.414163	7.491036	8.14781	8.049097	8.629023	7.998572	9.434451

RHOBTB3	202976_s_at	8.03474	8.311062	8.600944	8.514855	7.267263	6.676843	7.456307	6.747215	7.956932	7.815604	7.181782	6.685465	8.940998
CREBZF	202977_s_at	2.967815	2.91079	3.014311	3.380001	2.950567	2.971035	2.917928	2.924856	2.915626	2.886057	2.935554	2.972488	2.827196
CREBZF	202978_s_at	4.590279	5.179862	5.298027	5.170511	4.737835	3.954514	5.656623	4.719112	5.781498	5.625821	4.087859	5.110128	6.438333
CREBZF	202979_s_at	6.837246	6.762494	7.195998	6.700231	5.861137	5.628527	7.249354	5.788125	7.30901	7.249376	5.487942	6.198595	7.540337
SIAH1	202980_s_at	5.006077	4.857639	5.346889	5.016888	5.046554	4.319622	5.950446	4.596851	6.117545	6.135699	4.942194	4.980386	6.726892
SIAH1	202981_x_at	7.597312	7.551046	7.696858	7.727635	7.119521	6.962301	7.957825	7.594745	7.985978	7.916825	7.518302	7.676497	8.95878
ACOT1	202982_s_at	7.805887	7.530969	9.834277	9.826375	9.236012	8.584846	8.34327	8.31437	9.1998	9.105787	8.493885	8.549128	6.538094
HLTF	202983_at	7.551145	7.475588	7.621738	7.431749	6.660436	5.784156	8.951946	8.341346	9.360549	9.058793	7.825808	8.198439	9.085982
BAG5	202984_s_at	5.411942	5.078553	5.965875	5.325077	5.281726	4.554876	6.2718	4.964704	5.503535	5.469034	4.845781	5.085238	6.794976
BAG5	202985_s_at	8.108217	7.85251	7.991075	8.184526	8.099129	8.008872	8.143675	8.03292	8.251021	8.074413	8.037495	8.313364	8.155651
ARNT2	202986_at	2.590667	2.580085	2.527106	2.58865	2.567301	2.503672	2.570911	2.556941	2.535655	2.522216	2.998021	2.571853	7.157143
TRAF3IP2	202987_at	2.995122	2.899482	2.831158	2.882506	2.998011	3.010816	2.89892	2.925646	2.899126	2.875418	2.907982	2.921815	2.590549
RGS1	202988_s_at	3.584563	3.529567	5.682229	4.55477	4.7964	4.847024	3.55002	3.560908	3.545499	3.457082	3.449999	3.552661	2.9122
RGS1	202989_at	3.560899	3.467853	3.41521	3.469243	3.618741	3.457301	3.485642	3.493784	3.485964	3.457022	3.481509	3.531488	2.954396
PYGL	202990_at	9.522702	9.419196	10.2136	10.07729	9.474623	8.822455	10.2128	9.438169	10.12785	10.14159	8.827783	9.3472	6.997373
STARD3	202991_at	6.665395	6.259275	6.100584	6.112611	6.676008	6.630073	5.89601	6.311376	5.965366	5.770041	6.492531	6.248754	5.387646
C7	202992_at	9.728025	10.03436	10.92295	10.13723	9.507981	9.306868	3.419223	4.370807	3.409693	4.166023	9.448103	3.759766	7.709029
ILVBL	202993_at	8.091754	8.133281	8.539117	8.564635	7.889376	7.793726	8.077441	7.811695	8.289334	8.270227	8.269734	8.021596	7.252637
FBLN1	202994_s_at	5.207675	6.008242	6.067325	5.521349	6.053154	7.002054	6.377774	6.572953	6.271448	6.319272	7.785812	6.885038	9.018728
FBLN1	202995_s_at	3.461027	3.654485	5.106777	3.422656	3.250238	3.420993	5.797907	3.686116	5.69563	5.413631	4.194225	3.659085	8.528871
POLD4	202996_at	7.536747	7.561164	7.359935	7.567483	7.726441	7.163882	6.494276	6.122618	6.582813	6.670954	6.347411	6.437364	5.022657
LOXL2	202997_s_at	2.212415	2.1937	2.172259	2.169957	2.195377	2.175829	2.191095	2.192493	2.17676	2.164543	2.174542	2.186511	3.470535
LOXL2	202998_s_at	4.101177	3.886823	3.491399	3.561959	4.225595	4.059361	5.307742	4.079811	4.390853	4.468363	7.194052	4.181079	9.643347
LOXL2	202999_s_at	2.291707	2.263965	2.233578	2.249278	2.274845	2.253487	2.25483	2.267164	2.253973	2.242293	2.252206	2.261292	2.128288
STMN2	203000_at	2.500805	2.699638	2.451531	2.447328	2.475604	2.437491	2.457461	2.470354	2.45759	2.473241	2.775326	2.695604	12.26454
STMN2	203001_s_at	2.720825	2.680541	2.636184	2.65973	2.715145	2.662139	2.696507	2.687512	2.669892	2.715271	2.776322	2.683178	11.7268
AMOTL2	203002_at	7.35552	7.318119	7.603438	7.785834	7.352191	7.382941	5.387281	4.859884	4.885645	4.716932	7.296113	4.994035	8.937887
MEF2D	203003_at	3.345112	3.200818	3.34863	3.44857	3.608586	3.662989	3.087549	3.19763	3.194881	3.17757	3.180385	3.192574	2.94071
MEF2D	203004_s_at	3.467542	3.642483	3.409305	3.41379	3.461188	3.377779	3.449833	4.066543	3.364369	3.374952	3.297635	3.445101	3.356679
LTBR	203005_at	7.728402	7.523903	7.405678	7.56549	6.966967	7.03738	4.891289	5.110105	5.171051	5.12133	5.151551	5.439768	4.727445
INPP5A	203006_at	8.206149	8.377285	7.985412	8.53112	8.306224	8.634024	8.146344	8.608019	8.441285	8.70028	8.708604	8.687846	8.421416
LYPLA1	203007_x_at	9.29354	9.266411	10.20608	9.865642	9.346097	8.717712	9.861614	8.929602	9.813118	9.470288	8.628691	8.778979	9.971571
TXNDC9	203008_x_at	7.771526	7.775764	7.978289	7.82261	7.436713	7.032149	9.174177	8.544029	9.248997	9.211023	7.926172	8.761307	8.827824
BCAM	203009_at	3.036508	3.030406	3.010886	2.8991	3.02922	3.365441	3.027898	3.470093	3.299059	3.825343	3.751571	3.298976	2.717893
STAT5A	203010_at	5.400438	5.495858	5.46367	5.114495	5.493111	6.241983	5.562856	6.678845	5.943942	6.089607	6.19209	7.020007	4.038258
IMPA1	203011_at	10.02159	9.938872	9.731175	9.682324	9.574213	9.17815	7.878206	7.290057	8.035417	7.66308	7.327233	7.255847	8.564053
RPL23A	203012_x_at	14.04217	13.9573	13.80829	13.95452	14.09401	14.23618	14.24657	14.33774	14.531	14.29072	14.49754	14.52903	14.79959
ECD	203013_at	5.669774	5.465394	5.951342	5.954868	5.384297	5.512959	6.383575	5.81144	6.4485	6.428618	5.986253	6.195016	6.033358

SGSM3	203014_x_at	4.786067	4.837547	4.815552	4.791564	5.276235	5.291211	4.833137	4.975385	4.780034	4.960705	5.622736	5.265558	4.857798
SSX2IP	203015_s_at	2.464803	2.4575	2.445711	2.453206	2.474485	2.378139	5.890776	4.585831	5.288218	5.765327	3.183453	4.095111	3.887876
SSX2IP	203016_s_at	4.046852	3.41864	6.074673	5.777039	3.823612	3.816587	7.800694	7.024764	7.447925	8.074676	6.968363	7.132518	6.173841
SSX2IP	203017_s_at	3.756207	4.086075	4.612412	4.708258	3.676269	2.577334	7.567609	6.554113	6.983396	7.767486	6.203518	6.436833	5.859783
SSX2IP	203018_s_at	5.374455	4.846413	5.886591	5.738641	5.133571	4.899907	7.086644	6.953293	6.819471	7.185346	6.643582	6.527024	6.377367
SSX2IP	203019_x_at	2.731043	2.701771	2.793368	2.858525	2.66619	2.39183	4.707725	4.194702	4.172109	4.902159	4.32508	4.483662	3.768583
RABGAP1L	203020_at	8.00707	7.915123	8.257667	8.094205	7.559195	7.593082	8.303951	7.840303	8.236698	8.269561	7.721808	7.973837	8.376974
SLPI	203021_at	7.197819	7.33157	8.553944	8.981551	7.127476	7.354495	2.537222	3.018253	2.5563	2.47234	2.553034	2.77974	3.163829
RNASEH2A	203022_at	3.990789	4.099247	3.330117	3.891159	3.687743	3.784487	7.611923	7.31509	7.416559	7.783146	7.551434	7.699197	6.409731
HSPC111	203023_at	5.838601	6.032239	6.324969	6.371465	5.768779	5.421694	7.600895	7.028217	7.218679	7.520386	6.982663	7.180186	6.625239
C5orf15	203024_s_at	10.3212	10.1062	10.50042	10.4172	9.570688	9.671337	9.701123	9.236479	9.818904	9.730349	9.471911	9.370606	10.39662
ARD1A	203025_at	6.950642	7.177654	6.503316	7.108313	7.421453	7.412448	7.496483	8.401453	7.778194	7.919333	8.668503	8.326051	6.996211
ZBTB5	203026_at	6.875115	7.068887	6.588311	6.712154	6.894508	7.125431	7.069541	7.441706	7.175217	6.933027	7.455541	7.480241	7.50427
MVD	203027_s_at	2.957987	2.972545	2.911742	2.594536	3.391625	2.918091	5.208923	4.513015	4.294273	5.303966	3.780504	5.857066	3.190144
CYBA	203028_s_at	7.256954	7.250924	7.854598	7.687261	7.603169	7.493184	9.264593	7.989787	10.10717	9.61124	9.055422	8.790051	8.496745
PTPRN2	203029_s_at	3.003044	2.91654	3.356714	3.232875	3.369397	3.409591	5.357056	3.720258	4.471843	3.967213	4.417368	5.030933	6.365258
PTPRN2	203030_s_at	2.248327	2.239909	2.223023	2.22652	2.237047	2.228468	3.686638	2.904368	3.200584	3.072538	3.842866	4.689407	3.261155
UROS	203031_s_at	7.395789	7.42722	7.353375	7.297947	7.385654	7.350306	10.32175	9.986558	10.42122	10.67517	9.77641	10.25489	6.377004
FH	203032_s_at	4.929743	3.905713	7.264097	5.713254	3.863249	3.174318	7.148064	3.236267	6.742146	7.084979	3.883803	3.89072	6.810345
FH	203033_x_at	9.976722	10.00657	10.3598	10.53794	10.05218	9.958658	9.915032	9.426316	9.751924	9.964682	9.297648	9.481027	8.237989
RPL27A	203034_s_at	13.71194	13.63871	13.70875	13.73599	13.60423	13.56827	14.47162	14.53409	14.46619	14.54385	14.53854	14.58531	14.65161
PIAS3	203035_s_at	2.080563	2.055923	2.05669	2.041701	2.061761	2.046864	2.605158	2.048462	2.074362	2.942374	3.215708	2.047401	5.547951
MTSS1	203036_s_at	3.95504	4.050214	4.374019	4.736204	4.449188	4.150241	3.363073	3.915973	3.731262	3.459859	3.722571	4.304892	3.657961
MTSS1	203037_s_at	12.26351	12.17631	12.13682	12.02494	11.93355	11.74669	10.7924	10.10905	11.03874	10.74482	10.26156	10.27089	10.61439
PTPRK	203038_at	10.56591	10.51957	10.44855	10.77576	10.4113	10.27605	9.041894	9.127958	9.211473	9.099533	8.940488	9.099207	9.095737
NDUFS1	203039_s_at	9.179493	8.957675	9.565415	9.783946	9.283077	9.081013	9.050361	8.443706	8.878859	9.091643	8.626814	8.932936	8.349055
HMBS	203040_s_at	6.169246	5.811779	6.20029	6.433867	6.517458	6.237208	12.3736	12.36196	12.43225	12.56652	12.13615	12.38612	6.723413
LAMP2	203041_s_at	11.16031	11.03616	11.17205	11.09697	10.17081	9.821515	8.937651	8.01845	9.095649	9.127236	7.622465	8.008232	7.3068
LAMP2	203042_at	11.956	11.88404	11.54647	11.66662	11.30818	11.31852	8.870782	9.159638	9.333849	9.086157	8.648447	8.940337	7.275569
ZBED1	203043_at	9.62582	9.634448	9.922779	9.993305	9.206483	8.884587	8.419523	8.343056	8.388611	8.737339	8.351577	8.798613	8.505846
CHSY1	203044_at	8.097267	8.146962	8.26378	8.205702	8.223545	8.273283	8.70047	8.551997	8.630653	8.752645	9.138672	8.597131	11.17768
NINJ1	203045_at	7.743565	7.66111	7.354103	7.512595	7.24095	7.147976	7.41805	7.272976	7.256453	7.181858	5.664768	7.347298	5.749598
TIMELESS	203046_s_at	5.444965	5.196319	5.564721	5.540731	5.426526	5.318546	8.010925	8.013264	8.160389	7.726354	7.885524	7.958998	6.342349
STK10	203047_at	4.640657	4.940744	4.183037	4.687743	4.979081	4.925226	5.573121	5.829982	5.586867	5.637833	5.612333	6.205227	4.965466
KIAA0372	203048_s_at	6.516068	6.450686	7.353758	7.160582	6.021698	5.361603	7.565694	5.603243	7.761912	7.152609	6.202635	6.032937	8.543651
KIAA0372	203049_s_at	8.675561	8.736584	8.992038	8.93245	8.265341	8.015105	8.982365	8.759656	9.192979	9.119925	8.48163	8.499048	9.85721
TP53BP1	203050_at	5.076487	5.291919	4.83794	5.278526	5.291246	5.543058	5.715227	6.094744	6.055987	5.609572	6.611323	6.414332	7.788975
BAHD1	203051_at	5.761874	5.545896	5.468813	4.798374	5.403988	5.036238	5.487388	5.047013	5.589124	5.759159	5.533143	5.632212	5.39969

C2	203052_at	10.69571	10.77908	10.72618	10.78202	10.48138	10.37082	9.619587	8.900314	9.652458	9.710682	8.467448	9.173718	3.710606
BCAS2	203053_at	9.517722	9.414522	10.01422	10.06024	9.516281	9.164361	9.422387	9.678318	9.762832	9.494492	9.456676	9.615985	9.693413
TCTA	203054_s_at	6.995759	6.717288	7.239058	6.916159	6.554847	6.535616	6.503063	6.001988	6.10206	6.362034	6.124261	6.43783	5.706745
ARHGEF1	203055_s_at	5.114892	5.029982	4.720648	5.122975	5.187873	5.764109	4.301958	4.541922	4.182513	4.472427	4.530702	5.015191	4.018389
PRDM2	203056_s_at	2.884749	2.867253	2.843745	3.108188	2.868062	2.847561	2.860425	2.856194	2.875441	2.810084	2.976605	2.859484	3.815034
PRDM2	203057_s_at	5.477518	5.078318	5.866772	5.130651	4.993003	4.018044	5.892147	5.188708	6.278575	6.036313	5.346059	5.069865	6.705965
PAPSS2	203058_s_at	8.543276	8.508267	9.562178	9.777655	9.332107	9.283886	5.917102	5.644771	5.833152	5.852034	6.408179	5.490105	8.498385
PAPSS2	203059_s_at	2.544997	2.631492	4.749438	4.409237	2.536748	3.044538	2.534151	2.646497	2.448329	2.521529	2.516598	2.524817	4.790179
PAPSS2	203060_s_at	11.28952	11.22869	12.1878	12.22282	11.77312	11.72778	7.926621	8.21775	8.461572	8.354846	8.772438	8.08484	10.96561
MDC1	203061_s_at	2.55834	3.093791	3.196006	2.867701	2.770735	3.167787	4.644209	3.237482	4.276731	4.038215	3.521333	3.263247	4.035329
MDC1	203062_s_at	7.241022	6.932576	6.781032	6.777852	7.352467	7.703443	7.981698	7.941764	7.78447	7.759817	8.177181	8.017103	7.407144
PPM1F	203063_at	6.610702	6.504725	5.879946	6.060028	6.847023	7.403954	6.367694	6.720699	6.359532	6.259911	7.910773	6.554665	6.829676
FOXK2	203064_s_at	4.6513	5.086723	5.180355	5.256634	4.472914	4.404237	5.579074	5.525791	5.654454	5.494028	5.823628	6.096937	5.567043
CAV1	203065_s_at	3.784572	3.754713	4.533031	3.758567	3.503539	3.317632	4.167769	3.498329	3.759536	3.771897	3.751098	3.724072	7.280027
GALNAC4S	203066_at	6.914097	6.586514	8.303575	8.286613	7.947935	7.982074	6.081019	5.933913	5.60386	5.748996	6.372188	6.249823	7.782163
PDHX	203067_at	8.530782	8.493764	8.419353	8.493734	7.826415	7.02442	8.10144	8.267823	8.58594	8.544082	7.833646	7.955379	8.453087
KLHL21	203068_at	7.472149	7.564331	7.013669	6.871659	7.820479	7.934517	6.924899	7.239641	7.391184	7.24553	7.268288	7.211914	6.577021
SV2A	203069_at	2.840741	2.829671	2.637293	2.819145	2.830467	2.936021	2.829432	2.835012	2.82161	2.805293	3.572385	3.489533	5.732854
SEMA3B	203070_at	2.740078	2.692219	2.658434	2.672649	2.701451	2.680937	2.680759	2.687457	2.673077	2.66739	2.67594	2.684488	2.582529
SEMA3B	203071_at	3.016764	2.96969	2.927836	2.978455	3.044166	3.104731	2.960086	3.091193	2.961261	2.940424	3.107321	2.967216	4.000179
MYO1E	203072_at	3.374421	3.59539	4.463165	4.451777	3.359826	3.39819	2.918863	2.636884	2.762437	2.867054	2.438698	2.909735	3.070993
COG2	203073_at	5.71715	5.624872	5.507258	5.927129	5.38678	5.05456	6.74454	6.579323	7.640357	7.253158	6.467941	6.956488	6.605471
ANXA8	203074_at	2.674223	2.553589	2.95619	3.711317	2.545373	4.152016	2.532883	2.570143	2.083879	2.617168	2.625298	2.720811	2.499311
SMAD2	203075_at	8.267323	8.173787	7.963624	7.831889	8.139415	8.284534	9.10644	9.071854	8.897935	8.967283	8.863983	9.016328	8.884705
SMAD2	203076_s_at	6.213884	6.052317	6.596565	6.547367	6.03082	6.077156	7.655581	6.208394	7.618531	7.553488	6.292938	6.377034	8.040599
SMAD2	203077_s_at	6.708228	6.92369	6.536567	6.765584	6.752453	6.822094	7.960994	7.875264	7.941164	8.001652	7.737834	7.944814	8.215913
CUL2	203078_at	6.01589	5.9405	6.134171	5.846961	6.045936	5.7625	5.965041	5.956035	6.067579	5.975034	5.768744	6.27247	5.649822
CUL2	203079_s_at	6.622067	6.418992	6.643425	6.739689	6.088689	6.174167	7.515219	6.514923	7.523198	7.511971	6.435403	6.482309	7.890479
BAZ2B	203080_s_at	8.518963	8.350281	8.291631	8.435887	8.104628	7.979658	7.402504	7.313482	7.598467	7.528191	8.203309	7.534314	9.155947
CTNNBIP1	203081_at	4.579237	4.737809	5.005753	5.142733	4.122872	4.646188	5.232981	5.010444	5.347789	5.736854	5.682845	5.475641	5.715616
BMS1	203082_at	6.170318	6.17533	6.048872	6.288371	6.651843	6.551163	6.138802	7.090567	6.256574	6.302386	7.033931	7.070953	5.968879
THBS2	203083_at	7.93699	8.172146	5.518669	5.445167	7.298704	7.482698	3.192026	1.765205	1.837893	3.078463	7.769275	1.842837	10.37759
TGFB1	203084_at	3.315246	3.280668	3.202718	3.244256	3.272704	3.251179	3.276758	3.433128	3.247682	3.238192	3.347414	3.257514	2.978283
TGFB1	203085_s_at	5.34272	5.326375	5.702993	4.986856	5.590333	5.418364	7.217002	6.277834	6.685894	6.937212	6.096555	7.443061	5.364278
KIF2A	203086_at	2.728592	2.668902	2.614254	2.66082	2.692155	2.696826	3.215745	2.71124	3.32794	3.880383	3.1697	3.445249	5.48766
KIF2A	203087_s_at	3.880803	3.54009	3.394588	3.332269	2.510505	2.774352	6.886391	5.432012	6.532492	6.691756	5.839132	5.696808	7.940932
FBLN5	203088_at	4.041689	3.776633	5.202033	4.048312	4.030948	3.967877	4.031059	3.791174	3.791689	3.42784	4.505001	3.695717	9.230047
HTRA2	203089_s_at	6.537865	6.501883	6.342703	6.308137	5.902729	5.441212	9.955714	9.329439	9.937103	10.19076	8.941312	9.522865	6.676093

SDF2	203090_at	6.506404	6.410931	6.755063	7.02546	6.751776	6.350442	6.97178	6.455742	7.1322	6.709159	7.036131	6.686727	6.577903
FUBP1	203091_at	4.597241	4.288598	3.814256	3.854965	4.055775	3.510086	6.001222	4.250204	6.018258	5.549313	4.794685	4.306508	6.52436
TIMM44	203092_at	6.173373	6.121555	5.652171	5.936874	6.232975	6.147778	5.961369	6.668835	5.770358	5.753905	6.656188	6.685485	5.387483
TIMM44	203093_s_at	5.158964	4.718507	5.280576	4.714135	4.187749	4.694994	6.00728	4.959012	5.755372	5.876776	5.40538	5.468787	5.356589
MAD2L1BP	203094_at	5.572553	5.562109	6.063822	6.025599	5.334887	5.095438	7.10756	6.615099	7.427636	7.336057	6.551722	6.791817	6.210918
MTIF2	203095_at	9.5166	9.492544	9.133034	9.472232	9.585831	9.481091	8.679729	9.027552	8.74544	8.598073	8.879083	9.055324	7.193398
RAPGEF2	203096_s_at	8.150349	7.966923	7.925551	7.882349	6.465306	6.522141	9.68298	8.577205	8.784221	9.674738	6.552539	6.980015	8.339887
RAPGEF2	203097_s_at	9.833623	9.743997	10.18113	10.26517	9.960987	9.822163	10.97787	10.4219	10.83222	11.04373	10.10848	10.32777	9.813437
CDYL	203098_at	5.640846	5.674291	5.659379	5.890043	5.287812	5.589268	8.496696	7.62003	8.562483	8.481779	7.612215	7.869827	7.377733
CDYL	203099_s_at	3.367242	3.988487	3.34767	3.977934	3.998412	3.976708	4.454111	5.030382	4.67683	4.73077	3.983528	5.003194	4.226048
CDYL	203100_s_at	4.183036	3.995081	4.669062	4.209084	3.552981	3.823598	7.633747	5.043627	7.775344	7.806228	4.291025	5.156247	7.018789
MGAT2	203101_s_at	2.247565	2.236311	2.216442	2.216758	2.232515	2.218272	2.230854	2.23241	2.221516	2.228138	2.210512	2.226052	2.198809
MGAT2	203102_s_at	9.694216	9.708365	9.860542	9.561583	9.042965	8.659294	9.66405	9.208535	9.579038	9.501544	8.989432	9.463672	9.424215
PRPF19	203103_s_at	8.716374	8.6017	7.784266	7.936255	9.432572	9.562592	8.622364	9.254847	8.318629	8.522777	9.511179	9.372956	7.896395
CSF1R	203104_at	6.708665	6.779113	7.461627	7.33023	6.905642	6.53726	6.626823	6.737628	6.943922	7.019436	6.162525	6.56119	6.333831
DNM1L	203105_s_at	4.393737	4.429967	4.862988	4.519194	4.532666	4.858074	8.058594	7.166902	7.930287	8.134884	7.184089	7.241138	7.7298
VPS41	203106_s_at	2.713263	2.641874	2.596451	2.642594	2.689061	2.654051	2.738165	2.665393	3.319788	2.673865	2.650002	2.698278	3.041498
RPS2	203107_x_at	12.78101	12.69405	12.78964	12.80013	12.75668	12.58588	14.18546	13.62926	14.29693	14.22522	13.89795	13.84038	14.44051
GPRC5A	203108_at	2.047604	2.031055	2.009828	2.013767	2.030741	2.016002	2.026207	2.029884	2.015292	2.015148	2.01048	2.022549	1.985382
UBE2M	203109_at	4.69336	4.807115	5.554549	5.112598	4.034721	3.095283	7.518029	5.786136	7.294162	7.513128	5.196213	5.924991	7.148572
PTK2B	203110_at	3.896887	3.644708	4.414946	4.352138	3.934927	3.752876	4.389664	4.805623	4.452088	4.259218	3.85897	3.701961	2.950253
PTK2B	203111_s_at	2.199964	2.184192	2.174903	2.164367	2.182816	2.156606	2.17672	2.17783	2.167679	2.163878	2.16741	2.173988	2.082462
WHSC2	203112_s_at	5.40014	5.281523	5.259809	5.254137	5.09215	4.61839	6.223379	5.3915	5.996798	5.760009	5.457237	6.052564	5.995333
EEF1D	203113_s_at	12.02349	12.19395	11.59586	11.84004	12.32206	12.52584	11.55485	11.94878	11.73298	11.68238	12.52748	12.43055	12.19935
SSSCA1	203114_at	5.203247	5.089378	5.414179	5.638352	5.341454	5.34853	6.326156	6.432494	6.560719	6.538069	6.324039	6.473205	4.899982
FECH	203115_at	7.021323	6.865817	6.473786	6.65115	6.203133	6.067898	11.2389	11.59819	10.49406	10.89262	10.67368	10.56582	6.080088
FECH	203116_s_at	6.299472	6.177568	6.844917	6.83703	5.942299	4.895403	11.93477	10.88119	11.38386	11.68837	10.39377	10.97635	7.000477
USP52	203117_s_at	7.657676	7.692199	8.416616	8.43641	7.79236	8.002139	7.280709	7.035832	7.356842	7.399591	7.200051	7.25846	6.473897
PCSK7	203118_at	5.831155	5.862549	5.934391	5.62508	5.866867	5.24559	6.456181	5.86432	5.90687	6.373961	5.845112	5.855383	5.836548
CCDC86	203119_at	4.680575	4.140975	4.188049	4.446499	4.359745	4.600397	5.915037	5.617598	6.062094	5.743604	6.026067	5.548484	5.677843
TP53BP2	203120_at	6.017943	6.018062	6.55835	6.880784	5.379579	5.504326	8.158854	7.725724	8.448445	8.463362	7.459351	7.528007	8.183316
TTC15	203122_at	6.72458	6.612	6.374371	6.637476	6.722399	6.715759	6.660673	6.885861	6.642298	6.647775	7.114397	7.025885	5.994262
SLC11A2	203123_s_at	7.609455	7.329722	8.278901	8.160787	8.029677	7.592568	9.096122	8.478426	8.979164	8.817618	8.350018	8.83006	6.839683
SLC11A2	203124_s_at	9.082842	9.116082	9.148311	9.161247	9.063687	9.164967	9.573885	9.703539	9.682209	9.607052	9.437953	9.785246	7.373698
SLC11A2	203125_x_at	3.942024	4.065798	4.047634	4.000239	4.062674	4.045319	5.295737	4.194765	5.577504	5.637823	4.049295	4.436428	4.04459
IMPA2	203126_at	6.786819	6.864991	7.851115	7.719466	7.924261	7.45319	6.821458	6.672298	7.040077	6.74955	6.655932	6.405061	6.239454
SPTLC2	203127_s_at	4.168017	4.020147	3.972002	4.34041	4.080082	3.746652	5.460294	5.260382	5.72434	5.357345	5.028618	5.515434	4.504321
SPTLC2	203128_at	3.306927	3.197922	3.297486	3.358363	3.299147	3.185127	4.748064	4.245183	4.462686	4.542606	4.273511	4.482722	4.976941

KIF5C	203129_s_at	2.103842	2.088719	2.061901	2.069262	2.090906	2.068461	2.080429	2.087104	2.072401	2.064723	2.071922	2.081787	6.913457
KIF5C	203130_s_at	2.254458	2.236106	2.214965	2.216558	2.238394	2.221204	2.225369	2.233792	2.218941	2.211204	2.219894	2.226696	9.852689
PDGFRA	203131_at	8.640137	8.850176	9.714203	9.495062	9.497031	9.563193	7.634502	6.282765	6.680198	6.68635	10.51427	7.453116	12.45404
RB1	203132_at	8.346084	8.086576	8.234079	7.76509	7.657152	7.378417	10.12823	9.387858	9.481391	9.879968	8.625527	9.08687	9.279807
SEC61B	203133_at	10.90441	10.68636	11.29174	11.39407	11.0668	10.81348	11.43378	11.23547	11.51238	11.48055	11.27058	11.09138	11.22155
PICALM	203134_at	4.093339	3.819613	4.222728	4.032342	4.029543	3.890454	5.316299	3.988165	4.96271	4.304039	4.005072	4.043478	4.985288
TBP	203135_at	6.428559	6.387373	6.196593	6.125115	6.460802	6.241414	6.478435	6.465008	6.805406	6.370536	6.441666	6.409676	6.29368
RABAC1	203136_at	7.88159	7.868297	8.107469	8.203947	8.217028	8.096395	8.183662	7.963057	7.823881	8.057547	8.207214	8.137252	9.319711
WTAP	203137_at	8.675125	8.481423	8.396614	8.500539	8.226208	8.17671	9.211019	8.863504	9.453919	9.428232	8.614754	8.647089	10.10019
HAT1	203138_at	8.102641	8.003698	8.741662	8.751667	8.030109	7.49186	10.72196	9.624926	10.64275	10.65902	9.36542	9.615393	9.868778
DAPK1	203139_at	10.6375	10.64217	10.085	10.16333	9.731818	8.826662	9.238033	8.182761	9.392388	9.104456	8.417807	8.308945	8.894084
BCL6	203140_at	9.836357	9.738799	9.764787	9.860022	10.0733	10.31297	8.471799	8.35781	8.776725	8.596628	8.4485	8.491285	8.33055
AP3B1	203141_s_at	5.456592	5.352205	5.763668	5.833404	6.258309	6.197361	5.858091	5.78793	5.445169	5.367939	6.090952	5.591525	5.889585
AP3B1	203142_s_at	3.993525	4.063797	5.349119	4.828729	4.287383	4.052303	6.502889	4.936723	6.542104	6.827102	4.777375	4.695876	7.44245
KIAA0040	203143_s_at	4.834809	4.654796	5.498595	5.17674	5.15832	4.9773	4.595354	4.506678	5.359355	4.716239	3.945048	4.035737	4.55346
KIAA0040	203144_s_at	3.509719	3.372034	3.5898	3.433695	3.199838	3.305445	3.219507	3.30352	3.248195	3.174615	3.197294	3.23838	2.835608
SPAG5	203145_at	4.899544	5.050595	4.185215	4.08332	3.769427	3.911115	8.238602	8.058513	8.107154	8.127757	7.446859	8.175502	6.122561
GABBR1	203146_s_at	3.154151	3.146586	2.647065	3.13108	3.440845	3.512876	3.091661	3.102249	2.740764	3.06751	4.55081	3.534736	7.67143
TRIM14	203147_s_at	5.398992	5.419416	5.900496	5.611849	5.433271	5.84597	5.229808	5.840224	5.291324	5.397218	5.233156	5.466314	5.070075
TRIM14	203148_s_at	8.079739	7.952941	8.341025	8.466841	8.134818	8.195809	7.199401	7.47101	7.279305	7.197884	7.148855	7.577416	2.973948
PVRL2	203149_at	6.744206	6.749862	6.516861	6.730349	6.769136	7.119871	6.130202	6.497873	6.212464	6.277013	6.734424	6.882479	6.324817
RABEPK	203150_at	6.886782	6.976397	8.739315	8.86958	9.065989	8.692636	6.482419	7.001949	7.036785	6.847326	7.0821	7.162207	6.75189
MAP1A	203151_at	3.248464	3.483515	3.172941	3.159639	3.195456	3.121456	3.13516	3.19252	2.951992	3.171235	5.933283	3.481447	7.543166
MRPL40	203152_at	8.875351	8.893077	9.482608	9.614216	9.187959	9.032168	8.838318	8.61876	8.86998	8.712557	8.622009	8.770964	7.957335
IFIT1	203153_at	8.131736	8.05874	9.201931	8.938514	8.680503	8.068625	5.619218	5.35963	6.091974	5.529746	3.974256	3.820636	6.176999
PAK4	203154_s_at	6.832969	7.045111	6.543599	6.483827	6.598353	6.700243	7.208005	8.139512	7.205876	7.478138	8.047775	7.974119	6.532109
SETDB1	203155_at	5.612335	5.739368	5.28197	5.086816	5.354875	4.89433	5.774222	5.143708	5.700854	5.673386	5.475379	5.194219	6.488365
AKAP11	203156_at	7.716398	7.6223	8.184784	7.843568	7.138714	6.621137	8.432485	6.715957	8.45121	8.334053	7.082754	7.07294	9.546621
GLS	203157_s_at	2.908085	2.900033	2.873279	2.911596	2.922305	2.890874	2.898858	2.87845	2.907387	2.885354	2.860353	2.873389	4.75586
GLS	203158_s_at	2.632552	2.610711	2.549358	2.594733	2.772935	2.592611	2.579894	2.605007	2.592136	2.586514	2.587623	2.600415	3.677953
GLS	203159_at	6.034104	6.421776	5.927077	5.733353	5.793267	5.789117	6.241029	5.262307	5.920135	5.703628	5.782929	5.736412	7.074595
RNF8	203160_s_at	5.527161	5.191235	4.698763	4.921543	4.980408	5.112472	6.619958	6.127753	6.392519	6.489806	6.356277	6.58216	6.545314
RNF8	203161_s_at	2.039113	2.022512	1.998923	2.00373	2.019302	2.001507	2.015321	2.019459	2.007323	1.999291	2.008747	2.014274	1.944238
KATNB1	203162_s_at	5.296704	5.357172	5.368163	5.694785	5.724912	5.981841	6.641172	7.587583	7.023666	7.175853	7.75922	7.492471	6.818101
KATNB1	203163_at	3.105074	3.146185	2.992948	3.122719	3.093573	3.022719	4.828416	4.545853	4.9402	5.02849	4.435861	4.947998	5.211842
SLC33A1	203164_at	7.770649	7.340491	8.398233	8.350336	8.242012	8.194202	8.282538	8.163133	8.556859	8.132649	8.394147	8.445124	6.500741
SLC33A1	203165_s_at	5.997062	6.050905	7.318193	7.069042	5.464638	5.331004	7.416778	5.873468	7.513118	7.153476	5.136819	5.857086	6.667175
CFDP1	203166_at	9.038702	9.146863	8.998321	9.093541	9.215576	8.977726	9.368469	9.412313	9.44998	9.47109	9.601561	9.51156	10.4966

TIMP2	203167_at	4.320924	4.088215	4.197955	4.747998	5.271853	5.258256	4.145422	4.093569	4.086564	3.256954	4.794885	4.495474	5.20256
CREBL1	203168_at	2.683215	2.661196	2.625151	2.642213	2.666224	3.209063	2.653502	2.705177	2.660685	2.699341	2.640777	2.668709	2.602947
KIAA0258	203169_at	5.229661	5.241607	5.06719	4.712402	5.394946	5.493221	4.775037	4.996702	4.688751	4.472821	5.530618	4.993364	5.101398
KIAA0409	203170_at	3.740572	3.726067	3.751994	3.702032	3.996741	3.683003	3.723341	3.721749	3.709391	3.710136	3.704615	3.790371	3.636761
KIAA0409	203171_s_at	5.592047	6.252591	5.598598	5.863678	5.65723	5.915637	6.58686	7.000479	6.736513	6.637429	6.568784	7.124288	5.157575
FXR2	203172_at	6.476043	6.532594	5.60665	6.004756	6.048203	6.257825	5.656988	6.015698	5.719027	5.713827	5.917835	6.036461	5.708822
MGC16824	203173_s_at	5.690772	5.692704	5.370615	5.340026	5.362522	5.72807	5.992297	5.881379	5.953004	6.018717	5.831431	5.889012	6.201961
ARFRP1	203174_s_at	6.067782	5.707075	5.346345	5.331535	5.682801	6.018046	5.776408	5.999871	5.864485	5.696501	6.162887	6.151738	4.715149
RHOG	203175_at	5.788794	5.530515	5.952845	5.773121	6.037595	5.938113	7.053772	6.930585	6.979861	6.894036	6.773349	7.408896	5.91908
TFAM	203176_s_at	5.986936	5.350919	6.556783	6.281697	6.393211	6.747873	6.587463	6.705847	6.483715	6.332612	6.760063	7.291224	5.97175
TFAM	203177_x_at	8.393256	8.222045	8.553606	8.384294	8.011826	7.985357	9.105286	8.781348	9.238368	9.166232	8.459248	8.871633	8.026377
GATM	203178_at	13.62943	13.64709	13.83568	13.8313	13.19715	12.89786	12.7954	12.57858	12.27386	12.54524	11.98051	11.98118	10.06018
GALT	203179_at	8.790548	8.649955	8.480457	8.714401	8.894466	8.768208	8.495087	8.480124	8.664447	8.447627	8.544081	8.571485	6.540314
ALDH1A3	203180_at	3.54141	3.939453	4.768493	4.090235	6.360449	5.623366	2.548485	1.860479	1.89582	1.946272	1.98684	1.93409	5.691897
SRPK2	203181_x_at	5.530465	5.693789	6.093907	6.499309	6.361381	6.531242	5.67927	6.110909	5.398589	5.293438	6.368659	5.95245	6.437297
SRPK2	203182_s_at	6.4422	6.400905	6.703085	6.510304	6.428188	6.369523	6.176521	6.013275	6.423336	5.775169	6.442953	6.035251	7.766382
SMARCD1	203183_s_at	2.179806	2.209351	2.100124	2.171087	2.148143	2.147592	2.16189	2.161736	2.17306	2.147277	2.126463	2.140134	2.399196
FBN2	203184_at	2.884145	2.799534	2.767308	2.907002	2.872049	2.788922	3.23977	3.130358	3.794492	3.383973	9.698508	3.72345	12.89406
RASSF2	203185_at	3.900524	3.728878	5.333485	4.39655	3.954353	3.855161	3.853113	3.073211	3.583122	3.311403	5.267774	3.354808	7.676822
S100A4	203186_s_at	7.359001	7.598028	7.389965	7.059862	8.250253	9.101074	11.65909	12.43396	11.98655	11.87894	11.79271	13.25764	9.429734
DOCK1	203187_at	3.767878	3.754094	4.770722	4.435356	3.751612	3.660272	4.12727	2.617288	3.738558	3.737356	3.54182	2.978037	6.481825
B3GNT1	203188_at	8.234202	8.00895	7.621892	7.807583	8.217215	8.263301	7.547787	7.741426	7.204784	7.60036	7.588191	7.670699	8.21452
NDUFS8	203189_s_at	7.774097	7.512761	7.95237	8.196935	7.603025	7.288678	8.743864	8.626683	8.636925	8.800491	8.557457	8.387913	8.400303
NDUFS8	203190_at	8.871224	8.957337	8.728984	9.08584	9.144801	9.233767	9.581434	10.06409	9.550412	9.886132	10.27088	10.23628	8.786062
ABCB6	203191_at	2.94979	2.926349	2.882485	2.900617	2.934702	2.892761	2.914612	2.923818	2.910885	2.890897	2.907428	2.948735	2.684192
ABCB6	203192_at	7.268257	7.055939	7.214955	7.742047	7.450507	7.192974	9.77818	10.09875	9.705518	9.837631	9.713813	10.17556	6.346239
ESRRA	203193_at	5.607036	5.583289	5.642344	5.841744	5.643292	5.5587	5.425587	5.364178	5.348916	5.534289	5.229379	5.436847	4.387936
NUP98	203194_s_at	5.173436	5.207238	5.449008	5.289494	4.482818	4.918289	7.080019	5.696349	6.802665	6.947887	5.196856	5.673308	6.55431
NUP98	203195_s_at	6.381636	6.365921	6.485849	6.543565	6.083325	5.673444	7.102753	6.665739	7.178459	7.237938	6.248206	6.612932	6.119808
ABCC4	203196_at	3.879874	3.864409	3.814702	3.831794	3.862627	3.824119	7.562877	7.208984	7.709889	8.089549	5.661306	6.854919	4.902271
C1orf123	203197_s_at	7.032122	7.044787	7.887411	7.97637	7.743231	7.541876	7.732348	7.566911	7.65356	7.605118	7.537585	7.450266	8.930994
CDK9	203198_at	5.440062	5.361934	6.589317	5.971688	5.547868	5.253153	5.5273	4.984828	5.530062	5.438073	4.357566	5.092756	6.32257
MTRR	203199_s_at	4.728446	4.352438	5.348429	4.34805	3.63764	3.887248	5.051005	3.932232	4.63039	4.671352	3.633016	3.64111	4.985714
MTRR	203200_s_at	7.89158	7.855741	8.437601	8.466774	8.073557	7.843181	8.186017	7.877408	8.068097	7.695889	7.613638	7.677211	7.037773
PMM2	203201_at	6.493315	6.481472	6.039002	6.465014	6.480809	6.469603	5.717197	5.918905	5.562971	5.732792	6.28456	6.472251	5.727502
KRR1	203202_at	7.966516	7.676362	8.287205	8.696838	8.630937	8.809298	8.086888	8.529935	8.130902	7.64607	8.61983	8.589833	8.007734
KRR1	203203_s_at	7.849967	7.759592	8.305776	8.348155	7.913468	7.835689	8.552176	8.497237	8.183012	8.372784	8.046554	8.310916	8.208305
JMJD2A	203204_s_at	4.120985	3.820872	3.840359	3.822628	3.810196	4.856847	3.815529	3.807637	3.682265	3.670638	3.800769	3.96804	3.799091

JMJD2A	203205_at	4.675751	4.607737	4.626492	4.361963	4.638228	5.081757	4.641978	4.810309	4.341518	4.48311	5.080062	4.521321	4.881234
FAM53B	203206_at	4.383254	4.652766	4.203222	4.638983	4.755182	5.07704	5.254778	6.028663	5.421716	5.30646	6.328524	6.015375	4.463567
MTFR1	203207_s_at	6.134837	6.389973	7.017449	6.74921	5.237406	5.076193	6.452622	5.689475	6.550445	6.771441	5.463758	5.213727	5.87766
MTFR1	203208_s_at	9.013873	8.946963	9.495657	9.440766	9.096312	8.697922	8.902808	8.407478	9.084687	8.929583	8.432285	8.46928	7.827619
RFC5	203209_at	6.663897	6.55909	7.254794	7.29766	6.012818	5.763068	8.216394	8.038278	8.267759	8.220963	7.944997	7.993255	7.142523
RFC5	203210_s_at	2.504216	2.501139	3.284996	2.460732	2.486863	2.299415	5.442839	2.480539	5.874463	5.924432	2.463307	2.644969	4.628645
MTMR2	203211_s_at	5.098521	4.944353	5.653488	5.867514	4.867067	5.87935	7.609885	7.18604	7.438129	7.741162	7.420961	7.278054	8.599441
MTMR2	203212_s_at	2.376556	2.34208	2.32387	2.477071	2.473892	2.583096	4.852783	4.220351	4.760068	5.426252	4.69785	4.327182	5.909373
CDC2	203213_at	5.723937	5.301095	2.524082	3.670525	3.234964	3.697926	10.27882	10.17111	10.42699	10.57682	9.747221	9.853605	9.227941
CDC2	203214_x_at	2.215734	2.201963	2.169849	2.183916	2.198909	2.195219	8.883723	5.435313	8.208324	8.911973	5.330505	5.376522	8.028567
MYO6	203215_s_at	6.516547	6.331056	6.764065	6.920909	6.673687	7.227476	5.718984	5.532039	4.905211	5.339463	6.312167	6.323931	4.968603
MYO6	203216_s_at	7.658854	7.62096	7.764123	7.807468	7.245154	6.35354	7.423768	6.910818	7.007796	7.310628	6.702688	6.65967	7.356054
ST3GAL5	203217_s_at	6.654695	6.418465	6.186117	6.193848	6.382493	6.415026	5.931269	5.858664	5.254094	5.592292	6.151612	5.743031	7.03323
MAPK9	203218_at	6.343308	6.092626	6.233162	6.055775	5.9359	5.928346	7.179522	6.79442	7.301748	7.295776	7.12017	6.841866	7.578155
APRT	203219_s_at	6.534943	6.403653	7.109141	7.052953	6.937917	6.630453	7.854186	7.78886	8.029255	7.968082	7.559655	7.892925	5.513768
TLE1	203220_s_at	2.365222	2.342929	2.317982	2.322343	2.348862	2.321442	2.331626	2.318092	2.302961	2.306334	2.303494	2.329895	3.47386
TLE1	203221_at	9.596362	9.541932	9.955408	10.06206	9.147202	8.841135	8.553502	8.744174	8.553297	8.240554	8.724973	8.61561	9.233876
TLE1	203222_s_at	7.764785	7.285384	8.760451	8.919912	7.294648	6.847321	7.356783	6.946744	7.340374	7.096663	7.500514	6.920805	7.87298
RABEP1	203223_at	5.377359	5.288311	5.67251	5.285827	5.032888	5.231354	5.837209	5.310231	6.196624	6.228664	4.847541	5.637221	6.419883
RFK	203224_at	9.01737	8.868229	9.161552	9.265208	8.924643	8.791969	9.065228	9.473676	8.866466	8.949278	8.956797	9.068802	8.361769
RFK	203225_s_at	7.188527	7.054791	7.12767	7.238536	7.184182	7.279958	7.187316	7.741284	7.236507	7.25388	7.40095	8.061629	6.377875
TSPAN31	203226_s_at	4.646881	4.64107	5.806667	5.298936	4.605281	4.62965	5.127897	3.925171	5.130338	5.705129	4.380174	4.597276	5.429603
TSPAN31	203227_s_at	9.409472	9.433026	9.364468	9.440676	10.02367	10.21947	8.679201	9.141104	8.557191	8.628041	8.956423	9.051563	8.233865
PAFAH1B3	203228_at	2.641824	2.61475	2.598937	2.594994	2.616973	2.603072	6.678493	6.644728	6.858001	7.044862	6.591789	6.744335	7.640434
CLK2	203229_s_at	7.025767	7.094119	6.856683	6.437899	6.648215	6.755556	7.76059	7.182081	7.798267	7.46514	7.009864	7.02936	7.35993
DVL1	203230_at	8.158272	7.942416	7.039621	7.505065	7.948737	8.110528	5.823537	6.722323	5.881457	5.832216	6.767484	6.580526	5.533338
ATXN1	203231_s_at	6.187062	6.238716	7.339591	7.028127	7.533595	7.182983	5.571492	6.221302	5.507324	5.67327	4.992115	6.111424	6.476415
ATXN1	203232_s_at	8.289763	7.978152	8.763792	8.860968	8.58461	8.10686	7.686724	7.272114	7.588287	7.617072	7.767907	7.515089	8.8912
IL4R	203233_at	8.006869	7.892931	8.170892	8.058	7.784717	7.56175	6.133315	6.336176	5.715088	5.652912	6.225349	6.407301	4.622927
UPP1	203234_at	5.681493	5.633569	5.915208	5.919257	5.668485	6.447937	4.40759	5.630379	5.402442	4.992331	5.347729	5.625298	4.019834
THOP1	203235_at	6.150715	6.79413	7.15484	7.230524	8.103395	7.751908	6.314756	6.973317	6.640256	7.03053	6.71182	6.80785	5.197464
LGALS9	203236_s_at	5.235881	5.631147	5.625653	6.073443	6.138944	5.672397	4.935603	5.817868	4.948751	4.757898	4.979047	5.747655	4.453156
NOTCH3	203237_s_at	2.258404	2.248859	2.214967	2.227421	2.251556	2.241018	2.247361	2.249077	2.24559	2.345242	2.239225	2.242736	2.214891
NOTCH3	203238_s_at	2.93094	2.820535	2.720631	2.580066	2.819055	3.2094	2.903393	2.782895	2.346087	2.785453	6.232318	3.32726	7.087244
CNOT3	203239_s_at	5.153586	5.049379	4.865783	4.844823	4.57395	4.768014	5.897314	5.308735	5.993556	5.84929	5.619579	5.306871	5.533347
FCGBP	203240_at	3.02625	3.413238	2.871596	2.757897	3.112711	3.15166	3.026693	3.481169	3.09567	3.021642	4.081072	5.199111	2.679729
UVRAG	203241_at	5.538539	5.317263	5.857103	5.69944	5.698024	5.554501	5.512476	5.520279	5.549238	5.400995	5.088618	5.26704	5.231078
PDLIM5	203242_s_at	7.747592	7.12197	7.771865	7.789228	7.887863	7.963858	7.360486	6.618382	6.837191	6.396569	6.615225	6.908968	7.229546

PDLIM5	203243_s_at	10.28007	10.21572	10.56709	10.22746	9.617315	9.218332	9.904021	8.802769	9.816667	9.784621	8.619219	8.91283	9.093259
PEX5	203244_at	7.911451	7.907795	8.139364	8.234524	8.183214	7.936597	6.997998	7.375648	7.346612	7.154056	7.291104	7.223219	6.910924
FLJ35348	203245_s_at	2.966157	2.973805	3.310094	3.53764	3.324514	3.867245	4.447383	3.31897	4.541489	3.605346	4.320668	4.054326	6.351286
TUSC4	203246_s_at	6.694428	6.824339	6.772434	6.98062	6.951102	6.985494	6.790015	7.045096	6.636118	6.664105	7.198277	7.266076	6.892111
ZNF24	203247_s_at	7.782714	7.650571	7.897019	7.980717	7.673407	7.477179	7.649818	7.312421	7.639889	7.655427	7.078513	7.480428	8.026645
ZNF24	203248_at	3.510393	3.410881	4.749824	3.480618	3.354832	3.362173	4.066539	3.432544	4.150411	3.582797	3.357225	3.369343	4.837994
EZH1	203249_at	5.835747	5.611822	5.886739	5.742813	6.207259	6.280279	7.168892	7.268145	7.10277	7.347311	7.042499	7.045931	5.590149
RBM16	203250_at	8.237412	7.955815	8.353099	8.292808	8.526539	8.227407	8.829924	8.663361	9.050155	8.794731	8.556831	8.357298	9.047354
CDK2AP2	203252_at	5.946237	6.049412	6.933209	7.051236	7.060837	6.313126	7.663878	6.975137	7.736252	7.808416	6.915905	7.40408	6.177176
HISPPD1	203253_s_at	7.886535	7.900825	7.974439	7.906567	7.416165	6.884253	9.747807	9.328042	9.931819	9.966603	8.887745	9.147311	9.527633
TLN1	203254_s_at	6.413786	6.600538	6.810237	6.39331	5.908227	5.267156	8.145819	6.801872	7.82856	7.827034	6.421305	6.739832	7.025324
FBXO11	203255_at	8.857333	9.122009	8.52455	8.898044	9.119201	9.403335	8.825279	9.746213	8.798035	8.852932	9.540536	9.564248	8.673284
CDH3	203256_at	2.204857	2.179978	2.150975	2.163425	2.183963	2.331606	2.171273	2.178871	2.16374	2.158846	2.171356	2.169164	5.394734
C11orf49	203257_s_at	5.338195	5.250774	4.731435	4.876989	5.322673	5.300228	5.798941	6.272163	5.743656	5.963332	6.498346	6.406833	7.643356
DRAP1	203258_at	7.028881	7.070298	7.170917	6.961947	6.920652	6.921854	8.182864	7.732862	7.925156	8.420492	8.193804	8.500504	7.372149
HDDC2	203259_s_at	7.103007	7.121495	7.329943	7.21507	7.401884	7.19571	8.997653	8.357947	8.979051	8.770494	8.690679	8.715053	9.33451
HDDC2	203260_at	2.77603	2.789177	2.802124	2.805939	2.620588	2.769306	3.367001	3.48943	3.128179	3.265345	3.309052	2.906024	3.759134
DCTN6	203261_at	7.67314	7.676487	8.596996	8.496734	7.099104	6.649536	8.683217	7.717828	8.592502	8.506073	7.78594	7.693551	9.364373
FAM50A	203262_s_at	7.363269	7.500097	6.871165	6.92082	7.53118	7.612085	7.842987	8.34427	7.813872	7.817413	8.460979	8.477233	7.027621
ARHGEF9	203263_s_at	3.717015	3.685618	4.028083	3.796532	4.221297	3.808243	4.237771	3.860218	4.052624	3.79837	4.088398	3.692944	5.132396
ARHGEF9	203264_s_at	3.244184	3.488774	3.746901	3.461996	3.542732	3.539335	4.091349	3.540254	3.749194	3.627189	3.644377	3.943004	6.358371
MAP2K4	203265_s_at	4.924066	4.312687	4.796101	5.090774	4.05446	3.967582	5.586296	5.4136	6.185072	5.659026	4.765547	5.136828	6.049795
MAP2K4	203266_s_at	6.813152	6.657878	7.089408	7.013899	6.2373	6.281866	6.888098	6.730132	7.474265	7.562116	6.815946	6.867542	7.622368
DRG2	203267_s_at	3.976066	3.945418	4.133288	4.180979	4.010645	3.87483	5.841543	4.62576	5.996275	5.969982	4.010605	4.115497	5.638317
DRG2	203268_s_at	4.712122	4.638039	4.097293	3.799928	4.279311	5.176533	5.096025	5.354731	4.916943	4.913849	4.837191	5.226161	4.297878
NSMAF	203269_at	6.327098	6.736513	6.524405	6.675425	7.015086	6.45106	7.394495	7.140759	7.672983	7.326542	7.661676	7.392469	8.827357
DTYMK	203270_at	2.906583	2.829183	2.747322	2.814308	2.827874	2.759037	5.666648	6.162881	5.795995	5.65961	6.067712	6.113081	3.988748
UNC119	203271_s_at	2.801355	3.130508	2.736664	2.285514	2.818424	3.170831	2.737148	2.716331	2.523003	2.495809	3.23207	3.016541	4.018632
TUSC2	203272_s_at	5.955009	5.712172	6.318599	6.312389	6.089649	6.178324	5.558769	6.290971	5.69613	6.564861	6.02178	6.595269	4.908535
TUSC2	203273_s_at	5.89187	5.646767	5.663558	5.638007	5.826163	5.725328	6.349871	5.910613	6.107977	6.288454	6.254007	6.10565	5.360142
F8A1	203274_at	6.93281	6.926911	7.665901	7.702366	7.699964	7.875873	7.170572	6.858709	7.405062	6.362274	6.782563	6.997129	6.622079
IRF2	203275_at	5.709205	5.484238	5.615647	5.570111	5.783837	5.466805	6.22931	5.466745	6.47005	6.131693	5.226057	5.602045	4.625685
LMNB1	203276_at	3.831	3.776198	3.266893	3.211962	3.426786	3.737483	7.016273	5.497762	6.614799	6.483472	4.988034	5.808789	7.404417
DFFA	203277_at	6.711225	6.919834	6.41552	6.699663	6.082766	6.558329	5.715694	5.845265	5.958727	5.765175	6.059723	5.960911	6.335953
PHF21A	203278_s_at	5.881647	5.678193	5.005664	4.970295	6.374568	6.401891	5.791686	6.174209	5.988649	5.682425	6.94697	6.482254	7.006064
EDEM1	203279_at	8.979875	8.977696	9.90297	9.638826	9.38602	8.860993	8.488618	8.231621	8.820601	8.836104	7.702834	8.172174	7.295769
SAFB2	203280_at	3.365852	3.356374	3.339486	3.342288	3.353299	3.341867	3.615921	3.843671	3.500264	3.852065	3.334269	3.475234	3.270287
UBE1L	203281_s_at	6.96705	6.794007	7.197288	7.332429	7.369388	7.733754	4.983149	5.391609	5.154803	4.84226	5.463532	5.441066	4.262813

GBE1	203282_at	10.99656	10.97804	11.09394	11.04647	9.862936	9.411552	8.163316	7.589255	8.393594	7.950888	7.306172	7.663066	7.73899
HS2ST1	203283_s_at	5.887235	5.608906	5.99474	5.663735	5.638087	5.497157	6.946812	6.121974	6.975672	6.560935	6.231308	6.267426	6.807884
HS2ST1	203284_s_at	5.176369	4.873057	5.662646	5.538792	4.30842	4.238566	6.75241	6.70571	6.889596	6.600188	6.665569	6.70368	8.436995
HS2ST1	203285_s_at	7.556039	7.38971	8.368438	8.246338	7.268002	7.246168	7.822346	8.042291	7.405696	7.582737	7.645122	7.926241	8.039167
RNF44	203286_at	6.891999	6.904288	6.793462	6.530396	6.419092	6.107861	6.806342	6.321608	6.744493	7.033635	6.433359	5.861768	7.539546
LAD1	203287_at	2.282293	2.871799	2.851286	2.894832	3.555315	2.85672	2.878035	2.860603	3.093227	2.847629	2.849221	2.864481	1.731337
KIAA0355	203288_at	6.42219	6.235825	6.251716	6.400494	5.864455	5.440637	6.220068	6.220929	6.284021	6.475809	6.25168	5.701677	6.567336
C16orf35	203289_s_at	3.050676	2.998356	3.50994	3.021381	3.037129	3.343217	5.964847	3.181539	6.672853	6.48353	3.179739	3.596338	4.644382
HLA-DQA1	203290_at	2.985317	3.089867	6.69901	5.690522	7.327624	7.060537	2.478653	2.538846	2.496938	2.459402	2.546054	2.556733	2.386672
CNOT4	203291_at	7.393652	7.156198	6.970032	7.209815	7.131779	7.091685	7.262251	7.435169	7.693509	7.268943	7.672776	7.660083	7.230286
VPS11	203292_s_at	5.281882	5.371576	5.445437	5.603992	5.754368	5.855042	5.984999	6.331017	6.511178	6.080324	6.268955	6.417734	5.641899
LMAN1	203293_s_at	2.700119	2.612851	4.046506	3.21532	2.67947	2.537576	3.349395	2.62642	3.493745	3.940828	2.558815	2.642321	5.753399
LMAN1	203294_s_at	3.010288	3.326231	3.505744	3.074295	3.095525	3.025276	3.346854	2.91883	4.044851	3.193244	2.959365	3.12	5.084795
ATP1A2	203295_s_at	2.665652	2.621558	2.591856	2.613075	2.652532	2.837662	2.609561	2.616981	2.599377	2.599055	2.674484	2.621867	5.887587
ATP1A2	203296_s_at	2.768545	2.735021	2.69077	2.717213	3.134771	2.700099	2.706761	2.736869	2.712431	2.679851	4.773523	2.73153	9.312685
JARID2	203297_s_at	7.001691	6.607225	6.704178	6.661286	6.887344	7.229474	8.002607	8.59865	7.866854	8.12845	8.555795	8.648687	7.268975
JARID2	203298_s_at	5.348773	5.338226	5.289405	5.523823	5.853949	5.958259	7.81915	7.924595	7.863114	8.139094	7.416763	8.041083	7.533586
AP1S2	203299_s_at	7.1158	7.134918	6.988133	7.217033	7.485735	7.771368	6.97907	7.403577	7.016892	7.206455	7.571684	7.570617	7.984238
AP1S2	203300_x_at	5.047764	4.795924	5.723007	5.537423	5.027203	4.691905	5.834283	4.568384	5.915911	5.360177	4.212299	3.597497	8.677826
DMTF1	203301_s_at	7.863655	7.748169	7.826445	7.535613	7.710537	6.97726	8.255854	7.599466	8.107315	8.367061	7.891878	7.648729	9.509393
DCK	203302_at	7.433933	7.230276	6.733333	6.993182	6.684362	6.211038	11.33841	10.70118	11.45984	11.08374	9.817471	10.45382	9.247147
DYNLT3	203303_at	9.733312	9.627588	10.19142	10.04448	9.872539	9.592176	8.014256	7.878332	7.94823	8.104016	8.084908	8.059133	8.327401
BAMBI	203304_at	7.042248	7.177575	7.046986	7.219799	7.310796	6.755472	8.549865	7.757063	8.253197	8.283423	7.967443	8.345769	7.527616
F13A1	203305_at	2.225616	2.203284	2.181056	2.181917	2.61293	3.125398	5.596773	3.675734	4.719347	4.140143	4.370854	4.829923	6.41032
SLC35A1	203306_s_at	8.938378	8.783679	9.203195	9.149048	8.539275	8.241106	8.007669	7.960487	8.090683	8.234658	8.022134	7.992842	8.541975
GNL1	203307_at	6.000039	5.896153	5.273677	5.730569	6.208355	6.246073	5.884726	5.937086	5.72454	5.641051	5.94976	6.327099	5.444765
HPS1	203308_x_at	5.599808	5.674505	5.40718	5.707381	5.673539	6.232124	5.605851	6.373289	5.834443	6.395599	5.88759	6.215182	4.701302
HPS1	203309_s_at	3.93458	4.041928	3.784244	4.133094	4.089703	3.817409	3.927267	4.726695	3.885332	4.047429	3.861685	4.018897	2.287847
STXBP3	203310_at	8.913817	8.929973	9.178748	9.221099	8.378944	8.262498	9.381304	8.882967	9.532014	9.540131	8.477372	8.650065	8.970988
ARF6	203311_s_at	5.012146	5.096828	5.819851	5.177345	4.511648	4.464515	5.947354	4.802098	5.488322	6.030649	4.633789	5.09353	5.704462
ARF6	203312_x_at	7.625715	7.848903	7.671862	7.561309	7.798895	7.835401	7.892287	8.402368	7.843396	8.172154	7.727899	7.854818	7.203594
TGIF1	203313_s_at	6.42185	6.402285	6.754137	6.273845	5.887293	5.279492	6.475882	4.438479	6.240543	6.366518	4.245213	4.803261	7.707309
GTPBP6	203314_at	8.014584	8.019815	7.48157	7.868635	8.549968	8.638881	7.862426	8.357682	7.707064	7.741084	8.259682	8.63723	6.832728
NCK2	203315_at	5.385503	5.496558	5.352362	4.980054	5.371783	5.35533	5.545585	5.368797	5.357183	5.346679	5.923849	5.495047	7.410638
SNRPE	203316_s_at	10.89877	10.67178	10.75135	10.99496	10.63769	10.53529	12.21151	12.04844	12.18054	12.17367	12.05862	12.13365	12.4649
PSD4	203317_at	7.25689	7.227112	6.992757	6.932241	7.359249	7.595736	5.476341	6.114313	5.779733	5.392483	5.443434	5.789297	3.624553
ZNF148	203318_s_at	9.238222	8.979474	8.857492	8.987908	9.444887	9.614252	8.199662	8.950652	8.341803	8.195293	9.289321	9.263936	8.557601
ZNF148	203319_s_at	7.13306	6.567285	7.404143	7.372537	6.134735	5.250104	7.663085	6.297103	7.498425	7.822999	6.457834	6.174344	8.773283

SH2B3	203320_at	8.099772	7.946271	7.516992	7.45356	6.926554	6.477903	7.368926	7.169721	7.362052	7.77434	6.645255	7.188842	5.907718
ADNP2	203321_s_at	6.349034	6.177192	6.08574	6.444298	6.802645	6.391842	7.110767	6.691716	6.651995	6.996128	6.920521	6.618285	7.139956
ADNP2	203322_at	6.017602	6.258528	6.179486	6.086874	6.090783	5.860305	6.714495	6.5174	6.585675	6.757649	6.352669	6.557638	7.032848
CAV2	203323_at	7.578027	7.571487	8.310515	8.264948	7.490989	6.703789	4.795678	3.683021	4.503798	4.259092	4.637195	3.877313	7.078675
CAV2	203324_s_at	9.051254	9.004908	9.390055	9.049687	8.981406	8.86869	6.209118	5.939903	6.043281	5.824846	6.343906	6.102851	8.233103
COL5A1	203325_s_at	6.411389	6.40305	5.13441	4.912595	5.462409	6.14793	5.857216	4.868641	4.98018	5.301369	10.07475	5.860385	12.52003
IDE	203327_at	7.5421	7.721555	8.048317	8.098765	8.014479	7.973218	7.860347	8.50563	8.366657	8.389817	8.396032	8.696254	6.254296
IDE	203328_x_at	2.242505	2.151068	3.115639	2.826538	2.237807	2.174163	4.183355	3.105132	4.712647	4.951857	2.691337	2.965417	2.65711
PTPRM	203329_at	6.335474	6.40318	6.473311	6.270528	6.221707	6.185714	6.1679	6.184891	6.216862	6.300749	6.207206	5.680647	7.034035
STX5	203330_s_at	5.96544	6.073639	6.032829	6.147456	6.012649	6.048831	6.105298	6.055789	6.303824	6.514257	6.643079	6.175811	5.836495
INPP5D	203331_s_at	2.188858	2.174609	2.153668	2.157427	2.174878	2.153976	2.170751	2.172392	2.182208	2.28259	2.153598	2.166173	2.076588
INPP5D	203332_s_at	4.367888	4.550677	4.357631	4.339936	4.54116	4.6086	5.877723	5.189046	5.722182	5.377752	5.014369	6.024394	3.383171
KIFAP3	203333_at	5.850608	5.987972	6.419555	6.410233	5.638012	5.483964	7.194966	6.23179	7.069933	7.128174	6.213605	6.225546	9.127193
DHX8	203334_at	5.336901	5.309268	5.322664	5.289451	5.319418	5.131803	6.059495	5.296483	5.297621	5.803312	5.297707	5.16843	5.731526
PHYH	203335_at	13.37829	13.40862	12.73656	12.89739	13.0855	13.22724	12.3483	12.5019	12.12891	12.15156	11.82583	12.15027	8.429404
ITGB1BP1	203336_s_at	6.196003	6.353337	6.20885	6.415931	6.954314	6.500279	7.495748	7.862035	7.936556	7.866692	7.853127	8.202688	8.213516
ITGB1BP1	203337_x_at	2.697498	2.690009	2.83831	2.752955	2.689658	3.220019	3.095831	2.684107	2.430767	2.645415	3.606908	2.683118	5.017956
PPP2R5E	203338_at	8.632342	8.432305	8.589556	8.371003	8.237624	7.654252	9.060962	8.171992	9.35063	9.126878	8.245384	8.409945	9.664982
SLC25A12	203339_at	3.567644	3.101091	4.241382	4.141169	4.892502	5.331553	3.409303	3.602184	3.541501	3.573357	4.683553	4.162514	4.909774
SLC25A12	203340_s_at	2.445538	3.334305	3.346279	3.428403	3.381995	3.712701	3.337436	3.346287	3.333847	3.326647	3.719829	3.340253	6.635562
CEBPZ	203341_at	8.966435	8.710802	8.889557	9.164793	9.28814	9.621624	8.747939	9.52463	8.847093	8.732979	9.304951	9.600913	8.528724
TIMM17B	203342_at	4.491514	4.402294	4.213424	4.469132	4.46864	4.472241	5.350351	5.236697	4.889524	5.14895	5.194776	5.153481	4.475508
UGDH	203343_at	9.79092	9.765419	10.17112	10.00422	10.26603	10.04384	9.370034	9.270296	8.932204	8.875029	8.664902	8.75771	9.272767
RBBP8	203344_s_at	6.005621	5.724224	6.778431	6.641879	5.91056	5.975626	7.486004	7.365188	7.458723	7.170534	7.083708	7.402611	7.04689
MTF2	203345_s_at	6.753875	6.636599	7.196595	6.839644	6.894675	6.558544	8.415109	8.15057	8.381613	8.4786	8.105796	8.298998	8.753259
MTF2	203346_s_at	7.015422	6.995427	6.644229	6.436876	6.657268	6.38536	8.533839	8.177528	8.693879	8.510789	8.161014	8.12054	8.589846
MTF2	203347_s_at	5.969434	5.795358	5.723568	5.545511	5.836522	5.696428	7.345002	7.035973	7.627995	7.685836	6.549351	7.07588	7.789656
ETV5	203348_s_at	4.719529	5.073289	5.912725	5.800877	5.474231	6.008606	7.301085	7.175148	6.862225	7.054787	6.948308	7.16675	6.874585
ETV5	203349_s_at	5.801398	5.759491	5.585764	5.869582	6.580411	6.289932	7.188031	7.783035	7.309914	7.630643	7.66381	7.832569	6.850616
AP1G1	203350_at	6.561308	6.583632	6.649845	6.551065	6.069804	6.144628	6.588154	6.287135	6.526117	6.229846	6.549372	6.069645	6.734055
ORC4L	203351_s_at	7.959257	7.891909	7.74778	7.878488	7.705261	7.454567	8.480889	8.267138	8.680664	8.624059	8.116179	8.157193	8.72947
ORC4L	203352_at	2.853969	2.824021	3.038527	3.155237	2.850467	2.621551	4.188418	3.052976	4.077384	4.288557	2.761938	2.788188	4.721205
MBD1	203353_s_at	8.294459	8.091007	8.079383	8.389987	8.046155	8.261295	8.493188	8.334754	8.640427	8.383211	8.293565	8.473376	8.354296
PSD3	203354_s_at	7.635107	7.26801	7.484513	8.102739	6.395934	6.210562	4.388057	4.568785	3.873588	3.208058	4.043879	4.0336	6.732263
PSD3	203355_s_at	8.218223	8.260029	8.653014	8.807339	7.497663	7.431636	5.544178	6.0276	5.245558	5.186912	5.230333	5.643693	6.315825
CAPN7	203356_at	7.722138	7.527303	7.995499	7.899001	7.008515	6.78957	8.045723	7.121062	8.313927	8.049317	6.933596	7.353325	8.122651
CAPN7	203357_s_at	3.683403	3.507282	5.753937	4.811601	3.402043	3.11282	5.719493	3.285624	6.119089	5.761635	3.150167	3.340747	6.909538
EZH2	203358_s_at	5.509025	5.261628	3.287522	3.559875	3.617601	4.577949	9.149736	9.337345	9.227042	9.261192	8.984563	8.914278	8.364757

MYCBP	203359_s_at	6.533879	6.692268	6.473091	6.983251	6.581574	6.551218	8.63488	8.74384	8.875459	8.670639	8.542336	8.628041	7.303779
MYCBP	203360_s_at	6.045175	6.025059	6.318114	6.487816	6.040323	6.01295	8.038514	7.553003	8.547056	8.345288	6.902941	7.681458	6.796325
MYCBP	203361_s_at	3.058531	2.780529	3.144099	3.196608	3.162368	3.445338	3.765476	3.819761	4.479611	3.260981	3.88157	4.503226	3.155868
MAD2L1	203362_s_at	5.846239	5.165099	3.175069	3.609961	3.490118	3.853592	9.354653	9.024724	9.525152	9.460082	8.700069	9.056392	9.108684
KIAA0652	203363_s_at	6.547356	6.567186	6.498643	6.619446	6.513433	6.442323	6.520332	6.445762	6.514996	6.497708	6.496921	6.457726	6.508995
KIAA0652	203364_s_at	6.799233	6.800648	6.939198	6.79387	6.683016	6.531577	7.038099	7.385242	7.555413	7.321662	7.154245	7.112166	6.795871
MMP15	203365_s_at	6.611172	6.489111	5.817431	5.992619	6.704249	6.989578	4.619706	5.656654	4.484451	4.896292	5.904639	5.709353	5.027588
POLG	203366_at	7.554498	7.644854	7.37428	7.729747	7.68251	7.653352	7.467852	7.724069	7.431916	7.318944	7.768291	7.8017	6.780854
DUSP14	203367_at	6.460844	6.143989	6.300351	5.94811	6.01396	5.819196	5.076825	4.846278	5.065695	4.723487	4.938752	5.083222	5.220928
CRELD1	203368_at	5.276958	5.259064	5.23487	5.385055	5.269428	5.304803	5.251944	5.257198	5.242892	5.23512	5.24087	5.251965	4.841008
PDLIM7	203369_x_at	2.777071	2.766118	2.727891	2.750967	2.771415	2.744691	2.762363	2.76661	2.753748	2.747636	2.752316	2.763225	3.229836
PDLIM7	203370_s_at	2.672414	2.84898	2.595253	2.807756	2.661915	2.819067	5.053924	5.460796	5.833517	5.481964	5.578018	5.756541	6.297388
NDUFB3	203371_s_at	9.084114	8.976758	9.256549	9.471916	9.077219	8.830437	9.809723	9.415182	9.849499	9.959489	9.516392	9.505365	9.384651
SOCS2	203372_s_at	5.327128	5.312904	6.983363	5.725744	3.191793	3.178122	4.532945	2.937591	5.119678	4.982495	2.926351	2.962429	4.63493
SOCS2	203373_at	9.922507	10.01646	10.0345	9.756767	8.230758	7.766887	7.449343	7.468491	8.072121	8.042839	6.443744	7.083391	7.351536
TPP2	203374_s_at	6.648915	6.33329	7.19442	7.205204	7.139663	7.356549	7.418225	7.186907	7.585012	7.614499	7.443023	7.516707	7.046111
TPP2	203375_s_at	8.546033	8.383751	8.744679	8.720635	8.156209	8.430744	8.143291	8.342851	8.235867	8.248534	8.101937	8.181114	7.78421
CDC40	203376_at	7.254622	7.023345	7.962918	7.642123	6.948549	5.805909	8.045377	6.996649	7.842849	7.998391	6.812649	6.589698	7.565556
CDC40	203377_s_at	4.987108	4.262993	5.033322	5.159404	5.179086	5.098919	5.798659	5.251257	5.608612	5.742501	5.182367	5.953666	4.952932
PCF11	203378_at	8.094043	7.820034	8.013074	7.909644	7.990656	7.960343	8.4834	7.851827	8.165795	8.050373	7.781252	7.607043	8.616793
RPS6KA1	203379_at	4.126146	4.260811	4.29127	4.463668	4.391763	4.637736	4.440582	5.024921	4.444285	4.81732	5.339693	5.391804	2.307418
SFRS5	203380_x_at	10.59854	10.68563	10.59302	10.76471	10.75672	10.83193	10.53917	10.3729	10.31547	10.5222	10.24067	10.27357	9.948333
APOE	203381_s_at	13.00978	12.97691	13.11073	12.97905	12.17939	11.28299	12.19031	10.50923	12.10857	12.08542	10.76754	11.14414	8.549893
APOE	203382_s_at	13.27727	13.40692	12.84245	13.11398	12.92009	12.90509	12.07965	12.04719	12.02032	12.0783	11.7747	12.31805	7.285889
GOLGA1	203383_s_at	4.711047	4.978871	4.922409	4.818225	4.664786	4.793783	5.197488	4.966708	5.425587	5.142028	4.288474	4.316555	4.679863
GOLGA1	203384_s_at	5.83402	5.788032	5.171627	5.290437	5.2224	5.633722	4.987103	5.207238	5.111873	5.167276	4.400353	4.881596	4.007386
DGKA	203385_at	4.155115	4.645829	4.3263	4.089149	4.212142	4.86662	4.305002	4.204896	4.808286	4.19756	4.183668	4.224713	4.647916
TBC1D4	203386_at	8.546883	8.428787	8.168874	8.225448	8.131115	8.015583	6.801128	6.784733	6.804426	6.826253	8.079018	6.961892	8.042666
TBC1D4	203387_s_at	6.202368	6.403671	6.356653	5.866441	4.231614	3.487682	5.070381	3.063028	5.2807	4.605717	2.960087	3.138254	7.635134
ARRB2	203388_at	5.139956	5.27095	5.36393	5.36881	5.759717	5.64094	5.875927	5.313148	5.584642	5.496683	5.368524	5.39913	5.016407
KIF3C	203389_at	2.842522	2.824596	2.79857	2.809131	2.819026	2.805717	2.885478	2.822362	2.813421	2.809214	2.813586	3.056984	6.662019
KIF3C	203390_s_at	2.29633	2.290995	2.271266	2.274982	2.285054	2.281057	2.288989	2.287445	2.283788	2.277519	2.274151	2.276701	5.991557
FKBP2	203391_at	9.615682	9.738569	9.763206	9.867017	10.44275	10.51448	9.322054	9.858913	9.298793	9.313106	9.756341	10.22705	7.735132
CTBP1	203392_s_at	7.751187	7.680457	8.715621	8.360223	8.563483	7.522914	8.317581	7.718749	9.532637	8.930196	7.960812	7.862716	8.835049
HES1	203393_at	2.867405	2.825758	2.761978	2.804904	2.855956	2.825342	2.775472	2.828248	2.795147	2.691292	2.818831	2.857444	2.617285
HES1	203394_s_at	8.040158	7.977285	7.400098	7.147835	8.487831	8.65618	3.348204	3.459961	3.268699	3.639319	4.915165	4.136903	6.68132
HES1	203395_s_at	8.284731	8.205408	7.738514	7.58531	8.680902	8.577347	5.489274	5.625933	5.076153	5.049823	6.471758	6.326352	7.368184
PSMA4	203396_at	10.46893	10.32148	10.42956	10.53919	10.65446	10.79004	11.01404	11.12555	10.84962	11.01467	11.21616	11.30358	10.98596

GALNT3	203397_s_at	3.467827	3.580804	3.120279	3.141208	3.206788	2.981666	3.171365	2.858098	3.017924	2.901576	3.119267	2.989893	4.053762
GALNT3	203398_s_at	2.655663	2.620808	2.549984	2.589991	2.64064	2.604569	2.602677	2.620384	2.607313	2.600032	2.610042	2.651737	2.425448
PSG3	203399_x_at	2.230196	2.210833	2.173733	2.194116	2.215941	2.194179	2.197639	2.209069	5.026194	2.189752	2.193298	2.206591	2.289726
TF	203400_s_at	15.14113	15.05905	15.01504	15.17693	14.94964	14.78417	14.80813	14.7781	14.98974	15.052	14.83213	14.74445	10.20147
PRPS2	203401_at	6.928322	6.89692	7.35471	7.404416	7.102193	6.882441	7.679953	7.580881	7.824947	7.715526	6.879844	7.706292	7.207211
KCNAB2	203402_at	3.849685	3.725221	3.724197	3.828378	3.942077	3.8287	3.942574	3.939289	3.815088	3.757229	3.80743	3.837701	3.477377
RNF6	203403_s_at	8.302903	8.142459	8.406226	8.081225	7.526585	6.836767	9.394651	9.141646	9.495225	9.499317	8.437489	8.596518	8.984716
ARMCX2	203404_at	3.936532	3.757854	4.073061	3.915316	3.802597	3.785058	6.253909	4.599533	5.7803	5.865543	7.12482	4.953396	10.28847
DSCR2	203405_at	7.281257	7.284922	7.653245	7.876096	7.356107	6.852591	9.626914	8.889988	9.52569	9.750165	8.211439	8.797171	8.100593
MFAP1	203406_at	6.59092	6.676031	6.570639	6.546126	6.75292	6.403486	8.02767	7.941873	8.288553	8.190874	7.89589	7.960973	8.180877
PPL	203407_at	5.322428	5.207258	5.430772	5.697612	5.459192	5.196292	2.746844	2.766439	2.738107	2.733965	2.747038	2.762179	4.352211
SATB1	203408_s_at	9.441315	9.493479	9.064573	9.106376	9.357943	9.121193	7.938829	7.755834	7.288945	7.496175	7.989168	7.8373	8.926066
DDB2	203409_at	8.170804	7.975833	6.75293	7.165559	7.783489	7.799666	5.535462	5.600511	5.478247	5.19918	5.307728	5.574616	3.529862
AP3M2	203410_at	2.57996	2.594395	2.411474	1.887569	2.610924	2.63208	3.230587	3.922784	3.555351	3.163242	4.595126	3.721385	5.275717
LMNA	203411_s_at	8.456501	8.497485	8.383456	8.325619	8.7452	8.745825	9.146354	9.996823	9.311396	9.376368	10.14539	10.09092	8.969251
LZTR1	203412_at	6.030315	6.203564	5.718462	6.088456	6.060563	6.194055	5.51089	5.62838	5.245726	4.841739	6.306491	6.018633	6.323695
NELL2	203413_at	3.132083	3.066272	2.910614	2.990704	3.093699	3.070732	2.907392	3.05519	2.913076	3.048087	4.040391	3.04926	10.41155
MMD	203414_at	6.015363	5.858491	6.313893	6.457016	5.955867	5.182779	8.600163	7.845083	8.740852	8.992623	7.782844	7.892956	8.821735
PDCD6	203415_at	7.261551	7.140867	7.946628	7.585912	6.753136	6.119556	8.132008	7.218553	8.08967	8.199452	7.282329	6.927669	8.734647
CD53	203416_at	6.241534	5.770748	7.247277	6.984103	6.643876	6.284698	6.493025	5.709533	6.294323	6.517059	5.354297	5.719165	5.549861
MFAP2	203417_at	3.254647	3.066323	3.198925	3.01035	3.017874	3.182967	5.805024	3.944218	4.613982	4.303558	8.463953	5.82174	10.57421
CCNA2	203418_at	2.482085	2.471743	2.415698	2.434293	2.459356	2.435381	8.483832	6.920148	8.284499	8.424232	5.372539	7.357442	5.639669
MLL4	203419_at	6.395894	6.622067	6.324693	6.434392	6.12526	6.174408	6.389968	5.694549	6.972829	6.870799	5.781131	5.983765	6.355145
FAM8A1	203420_at	11.16861	11.13155	11.30647	11.29259	10.72637	10.29321	9.885221	9.002932	9.565292	9.578989	9.12386	9.088347	9.970131
TP53I11	203421_at	4.736242	4.643163	4.59174	4.448503	4.799203	4.828348	4.391769	4.460148	4.400427	4.535661	4.404764	4.452563	4.380906
POLD1	203422_at	5.272923	5.434335	4.10723	4.310941	4.936163	4.905518	8.202179	8.423723	8.240908	8.533588	8.361298	8.315592	6.092841
RBP1	203423_at	8.307349	8.233149	8.677209	8.636751	8.475268	8.909033	8.727899	8.881755	8.832369	8.948269	9.697376	9.298946	9.93265
IGFBP5	203424_s_at	2.377111	2.358161	2.348847	2.76382	2.516398	2.818628	2.356999	2.364346	2.354123	2.344645	2.352356	2.358654	5.869399
IGFBP5	203425_s_at	3.274953	3.262594	3.247081	3.252091	3.893928	3.405723	3.157086	3.380466	3.206088	3.130391	3.392498	3.253007	4.588385
IGFBP5	203426_s_at	2.86099	2.800626	2.75173	2.805999	2.895343	2.780574	2.804526	2.831502	2.784334	2.796526	2.941869	2.841348	3.408652
ASF1A	203427_at	7.834234	7.700517	8.125305	7.977356	7.36542	6.822993	10.27848	8.965	10.08124	10.03466	8.363717	8.759462	9.146423
ASF1A	203428_s_at	5.446646	5.174119	6.378817	5.590705	5.144596	4.52722	8.718417	7.003378	8.467706	8.559856	6.227554	6.516847	8.304947
C1orf9	203429_s_at	7.124612	7.064279	6.70398	7.341743	6.932545	7.224918	9.495621	9.570691	9.79647	9.487964	9.27846	8.910384	9.024172
HEBP2	203430_at	7.979168	7.992817	9.151038	9.205572	9.312645	8.871205	7.420571	7.195083	7.521052	7.350529	7.176863	7.356416	7.751317
RICS	203431_s_at	2.51393	2.297021	3.253916	2.976777	2.344291	2.199988	2.217107	2.228968	2.212584	2.201555	2.214789	2.222425	3.00463
TMPO	203432_at	6.569704	6.106303	6.874504	6.581094	6.050179	5.592023	9.256512	8.503102	9.577773	9.312313	8.013115	8.488047	8.117579
MTHFS	203433_at	8.724439	8.862602	9.576481	9.613748	8.924492	8.356798	7.672834	6.97799	7.264856	7.343815	6.916514	7.240069	5.705061
MME	203434_s_at	2.670557	2.616856	8.41143	8.64502	4.935485	4.230684	2.604977	2.677358	2.601654	2.590351	3.066329	2.611068	7.663933

MME	203435_s_at	2.676385	2.508994	7.088039	6.554246	3.803751	3.937505	2.653039	2.660939	2.313663	2.156935	2.93298	2.414472	4.901884
RPP30	203436_at	6.634339	6.197015	6.443085	6.429855	6.356357	6.600325	8.413513	8.390334	8.433985	8.457151	8.072543	8.48926	7.493557
TMEM11	203437_at	6.26284	5.602803	6.183774	6.200057	6.126938	6.375784	7.738691	7.964766	7.697983	7.77726	7.465573	7.872658	6.958177
STC2	203438_at	2.365017	2.331883	2.310043	2.317593	2.486541	2.324675	2.319608	2.304798	2.315584	2.300587	3.949651	2.323944	4.428449
STC2	203439_s_at	2.068596	2.052281	2.031274	2.039214	2.052024	2.043575	2.04417	2.04935	2.037503	2.030309	2.505253	2.044976	2.096449
CDH2	203440_at	11.45615	11.51993	10.57462	10.86873	11.23372	11.46597	9.898166	10.02073	9.566028	9.456657	10.50938	10.47055	10.56038
CDH2	203441_s_at	6.350077	6.477269	6.980523	6.511878	5.621158	4.935923	6.322524	5.706943	6.097965	6.127158	4.930443	5.447394	7.064813
EML3	203442_x_at	6.108621	5.978962	5.952718	6.176163	7.013139	6.79917	5.02656	5.180603	4.966337	5.10893	5.773518	5.698805	4.850451
EML3	203443_at	2.40114	2.394631	2.372207	2.37493	2.386375	2.371746	2.392457	2.390149	2.388216	2.381766	2.377791	2.399445	2.341214
MTA2	203444_s_at	2.593498	2.574392	2.551325	2.548206	2.566718	2.563089	2.570804	2.572879	2.56495	2.560203	2.556934	2.562714	2.462828
CTDSP2	203445_s_at	9.426755	9.282813	9.451708	9.384917	9.343388	9.012461	8.778898	8.520703	8.785346	8.700573	8.589891	8.566042	9.532124
OCRL	203446_s_at	5.1166	5.094508	5.36995	5.079239	5.075928	5.052958	5.27838	4.968478	5.297951	5.070948	4.804921	5.068799	6.026731
PSMD5	203447_at	7.000598	7.083149	7.387612	7.400567	7.257269	6.371977	8.483027	7.300221	8.121946	8.23737	7.004713	7.316878	7.799509
TERF1	203448_s_at	7.859566	7.834154	7.83333	8.026554	7.852032	7.479419	7.747662	8.127098	7.745068	7.588842	8.129758	8.18591	7.988614
TERF1	203449_s_at	7.804872	7.480908	8.040954	8.151914	7.907017	7.6054	7.834946	8.066228	8.122058	7.9024	8.09262	8.122101	8.823672
CBY1	203450_at	5.897209	5.83797	5.739779	5.857183	5.784265	5.726292	5.648829	5.842315	5.953408	5.724656	5.983546	5.917748	6.569698
LDB1	203451_at	4.143617	4.020957	3.408955	3.888463	3.480441	3.822431	6.498284	5.699347	5.946653	6.138551	5.674308	5.544675	4.322555
B3GAT3	203452_at	3.918177	4.019239	3.274678	3.242319	4.363191	4.564724	3.585455	4.166206	3.403263	3.592813	4.772634	4.738818	3.439981
SCNN1A	203453_at	3.28872	2.672492	3.718143	2.65718	3.266645	3.961712	1.977811	1.978586	1.97049	2.166078	2.173709	1.970756	2.045388
ATOX1	203454_s_at	9.958703	9.795727	9.472385	9.840116	10.34179	10.42066	9.088007	9.969628	8.96773	9.136172	9.946293	10.20851	8.160486
SAT1	203455_s_at	12.75471	12.80854	12.51602	12.78327	12.66341	12.72814	11.17256	11.65734	11.257	11.19419	11.04865	11.5267	10.05511
PRAF2	203456_at	4.566754	3.750307	4.537002	4.55212	4.629058	4.866765	5.929704	5.431609	6.006674	5.634505	6.237628	6.084917	7.69064
STX7	203457_at	3.849811	3.770413	3.78863	3.79479	3.719715	3.768174	4.367229	3.816748	4.429651	4.512207	3.79751	3.846869	4.621003
SPR	203458_at	8.016874	8.144892	8.386186	8.489557	8.310984	8.051387	5.850248	6.424844	6.714191	6.820859	5.955406	6.702904	4.672157
VPS16	203459_s_at	7.225601	7.222642	6.900188	7.338571	7.880232	8.1453	7.301397	7.941432	7.2339	7.50151	8.121652	8.258932	6.889636
PSEN1	203460_s_at	7.583227	7.671238	7.237706	7.317506	7.18434	6.850898	7.161875	7.391422	7.442685	7.490192	7.12294	7.388942	8.10618
CHD2	203461_at	2.457552	2.427893	2.408449	2.404498	2.426794	2.410651	2.423245	2.4265	2.416698	2.395803	2.408683	2.420095	2.356574
EIF3B	203462_x_at	9.206788	9.196568	8.810363	8.844498	9.260542	9.243639	9.696235	10.05	9.774235	9.935176	9.965521	10.25683	9.046452
EPN2	203463_s_at	3.030357	2.926413	2.998176	2.971241	3.02331	2.916145	3.549808	3.581189	4.114107	4.472557	3.277413	3.97446	4.755974
EPN2	203464_s_at	5.869454	5.349344	4.776089	4.586438	4.655991	5.992872	5.987652	5.845168	6.039106	6.364061	6.363546	6.44172	6.26645
MRPL19	203465_at	8.259631	8.373013	9.09328	9.009064	8.232717	7.752648	9.082225	8.524525	9.478532	9.292396	8.199488	8.570593	8.298106
MPV17	203466_at	2.970311	2.931365	3.596378	2.960626	2.616033	2.727959	4.817975	2.951365	4.844762	4.504335	3.115005	3.215991	5.873118
PMM1	203467_at	7.287501	7.184002	7.407311	7.762735	8.171984	8.241336	7.280893	8.033536	7.399726	7.405121	7.91601	7.99322	5.95735
CDK10	203468_at	8.379565	8.285984	7.093638	7.438252	8.631247	8.990717	6.837509	7.875344	6.799557	6.984876	8.046987	7.748398	6.217898
CDK10	203469_s_at	6.871821	7.151227	5.954374	6.176349	7.012117	7.512603	5.810099	6.451493	5.99542	6.044066	6.752535	6.679674	4.329178
PLEK	203470_s_at	4.615091	4.948405	4.830118	4.386727	3.944874	5.416927	7.090516	6.701594	5.946	6.391687	6.77295	7.571906	3.478145
PLEK	203471_s_at	4.440612	4.996783	5.942103	4.795357	4.346071	4.906679	8.170468	7.070633	8.393084	8.560721	6.610137	8.010952	4.735833
SLCO2B1	203472_s_at	4.395188	4.157066	5.329354	5.04214	5.105125	5.158337	3.045529	3.008234	3.710846	3.101847	2.307626	2.64236	2.12545

SLCO2B1	203473_at	9.986778	9.906077	10.12918	10.29658	9.772436	9.205202	8.525645	7.688584	8.879783	8.593313	7.383463	7.71475	4.640692
IQGAP2	203474_at	10.94897	10.81835	10.95245	11.0575	10.83507	10.41443	10.77402	9.965899	10.75158	10.66797	9.389482	10.13814	8.345398
CYP19A1	203475_at	2.372642	2.310904	2.266195	2.300256	2.345006	2.277027	8.803721	8.409812	9.928839	10.03835	6.531216	7.526448	2.379335
TPBG	203476_at	3.213754	3.553354	3.728734	3.896418	3.672439	4.139234	2.797029	3.58797	2.967576	3.514625	7.035849	3.581161	8.906246
COL15A1	203477_at	2.412944	3.730964	2.368337	2.360816	2.396134	2.355371	2.369043	2.380186	2.365751	2.338149	5.163354	2.377436	10.99295
NDUFC1	203478_at	10.97601	10.92272	11.05159	11.20822	10.97015	10.7473	11.47306	11.04067	11.21279	11.26969	11.07695	11.24139	10.76155
OTUD4	203479_s_at	6.729198	6.716995	6.635754	6.669434	6.824347	7.114095	6.682036	6.718628	6.677784	6.667194	7.08219	6.959617	6.506607
OTUD4	203480_s_at	8.361124	8.214102	8.243701	8.241712	7.952038	8.163113	7.921983	8.116485	8.020597	7.929425	8.161768	7.937749	8.104003
C10orf6	203481_at	5.705611	5.706828	5.052584	4.647129	5.466037	4.690545	6.263105	5.367645	6.290459	6.39184	6.06287	5.773617	7.122362
C10orf6	203482_at	5.504982	5.714192	4.887643	4.988202	4.429774	4.644704	6.397804	5.1162	6.080819	6.263346	6.627841	5.541415	7.374056
SEMA4G	203483_at	2.165364	2.145704	2.118284	2.126311	2.145331	2.12657	2.135278	2.140807	2.128976	2.120623	2.129346	2.13442	2.048534
SEC61G	203484_at	9.665412	9.573282	9.667522	9.701411	10.09743	9.848855	10.73302	10.85053	10.89984	10.91254	11.07721	10.93627	11.12077
RTN1	203485_at	3.667013	3.587841	3.525021	3.674047	4.351292	4.5028	3.250958	3.997815	3.593287	3.365982	4.991133	3.696194	12.5304
ARMC8	203486_s_at	6.534637	6.619341	6.921486	6.59101	6.619239	6.318428	8.736262	7.398022	8.761935	8.608535	7.061737	7.55671	7.304574
ARMC8	203487_s_at	6.082047	6.078006	6.062738	6.040344	6.24098	5.795928	7.490184	7.525426	7.606177	7.60548	7.346714	7.556788	6.555979
LPHN1	203488_at	2.901776	3.016254	2.547256	2.873648	2.888814	3.631602	2.880938	2.885609	2.862748	2.872637	5.292413	2.878806	4.99848
SIVA1	203489_at	7.233019	7.223012	7.16391	7.302991	7.232586	7.052228	7.562068	7.222326	7.393005	7.652971	7.586528	7.403835	6.903591
ELF4	203490_at	2.856979	2.829092	2.813535	2.735423	2.891463	2.613991	3.472927	3.128408	2.987691	2.887212	3.564766	3.364716	2.919428
CEP57	203491_s_at	7.454998	7.384569	8.051719	8.547312	8.971406	9.239514	7.939226	8.81203	8.027044	7.342331	8.491994	8.395027	7.724419
CEP57	203492_x_at	5.884974	5.169356	6.955222	6.745891	6.516727	5.940204	7.597205	6.452263	7.922088	7.678326	6.347697	6.412387	7.917926
CEP57	203493_s_at	7.681283	7.608877	8.560604	8.30238	7.832024	7.3585	8.653464	8.404118	8.832202	8.818702	8.033999	7.900439	8.31373
CEP57	203494_s_at	8.427663	8.613579	9.065667	9.41067	9.688079	9.189952	9.148162	9.431342	9.438065	9.249841	9.052383	9.020489	8.409148
LRRC14	203495_at	3.039204	3.771889	2.996324	2.567923	3.022098	3.025966	3.096742	3.735346	3.146449	2.998402	3.504031	4.003129	3.252983
MED1	203496_s_at	5.126626	5.106832	4.915677	4.722784	5.619798	5.795796	5.105309	5.800767	5.091299	5.089399	6.048346	6.026795	5.746137
MED1	203497_at	7.625993	7.398843	7.705413	7.218249	7.496794	6.818796	8.656184	7.916847	8.797729	8.884506	7.891814	7.531274	8.910868
RCAN2	203498_at	5.308839	5.260967	5.910938	5.448943	5.993661	5.882256	2.818315	2.846172	2.892169	2.798339	3.224715	2.920827	7.61528
EPHA2	203499_at	5.216042	5.272562	5.453738	5.070352	3.716346	3.69107	3.758659	2.942999	3.45256	3.879258	3.224767	3.160726	3.586262
GCDH	203500_at	9.068076	9.083438	9.442403	9.644068	8.748408	8.783344	8.002742	8.972392	8.182699	7.745139	8.617133	8.651727	5.087068
PGCP	203501_at	8.533526	8.699284	8.989562	8.801447	8.13396	7.497902	8.320513	7.481855	8.050342	7.766619	7.298828	7.538816	7.871775
BPGM	203502_at	5.035071	5.170925	5.24609	5.562037	4.89825	4.810726	11.12622	10.97308	11.56576	11.44703	10.17173	10.46938	7.471118
PEX14	203503_s_at	6.256482	6.219034	6.350372	6.490899	6.027412	6.175657	5.43109	5.447728	5.436167	5.254498	5.693733	5.525685	5.312586
ABCA1	203504_s_at	8.601154	8.439755	8.745652	7.908019	6.532082	4.214693	9.385318	6.926031	9.0794	8.873364	6.173845	6.973207	6.741874
ABCA1	203505_at	11.61091	11.56288	10.96253	10.78118	10.99026	11.01759	11.138	11.11072	10.97279	10.98077	10.8176	11.0776	8.87964
MED12	203506_s_at	4.009367	3.998777	3.969261	3.982452	4.002019	3.977093	5.690968	4.495306	5.707111	5.257353	4.547601	4.062176	5.41531
CD68	203507_at	6.124497	6.155188	5.536947	5.548894	6.500614	7.056626	5.818401	6.730095	5.144882	5.308765	5.872034	6.443282	3.573428
TNFRSF1B	203508_at	7.414922	7.451786	7.952824	7.943322	7.711412	8.161662	6.028268	7.090639	5.964617	6.41262	6.890748	7.228063	4.447383
SORL1	203509_at	10.82212	10.7742	11.21718	11.37228	10.24387	9.555378	6.835557	6.70473	6.84807	6.854957	6.946019	7.032148	5.191547
MET	203510_at	11.09412	11.07946	11.0858	11.20599	10.93083	10.74693	9.862785	9.411874	9.665224	9.4613	9.250315	9.601165	6.946947

TRAPPC3	203511_s_at	7.194937	7.216989	7.7927	7.535664	7.865083	7.710338	8.653044	7.903693	7.836776	8.114679	7.563562	7.990778	8.189891
TRAPPC3	203512_at	7.516599	7.71585	7.425038	7.563573	7.042871	6.822951	6.350089	7.933565	7.322099	7.497591	7.925919	7.575827	6.516046
SPG11	203513_at	8.652775	8.591825	8.878415	8.437883	8.08503	7.65766	8.384643	8.127418	8.813849	8.589749	7.929476	8.056712	8.468115
MAP3K3	203514_at	4.877331	4.712018	4.417613	4.100916	4.631291	4.546836	4.020969	4.11333	4.079952	4.212645	4.495336	4.087134	4.375274
PMVK	203515_s_at	7.530079	7.548657	7.529107	7.586765	8.420399	8.173582	6.575209	6.644612	6.552437	6.577663	7.011318	7.415657	6.437999
SNTA1	203516_at	4.18096	4.770683	3.954094	4.438303	4.18817	4.692593	3.754838	3.793506	3.615588	4.080322	4.086017	4.084751	4.514016
MTX2	203517_at	7.32803	7.23275	7.580466	7.760333	6.884043	6.381753	8.01872	6.897736	7.992708	7.902604	6.695833	7.026162	8.644844
LYST	203518_at	6.542054	6.325943	6.981279	6.92647	6.019022	5.01354	5.563325	4.751113	5.950406	5.933504	3.876768	4.47745	6.114114
UPF2	203519_s_at	7.023504	7.259779	7.090238	6.898467	6.902817	6.465012	7.931427	7.082828	8.098867	7.713996	6.956616	7.312444	7.4121
ZNF318	203520_s_at	3.229791	3.155193	3.09446	3.174617	2.962245	2.726635	4.244189	3.099385	4.283189	3.587718	3.177447	3.19267	4.621664
ZNF318	203521_s_at	5.025578	5.051367	5.308881	4.92766	4.34292	4.240418	5.67051	4.883847	5.641456	6.189721	4.653327	5.007759	5.550433
CCS	203522_at	9.310648	9.192113	9.107567	9.453951	9.996392	10.03258	8.135169	9.060206	8.185683	8.473382	8.901042	8.628772	7.20916
LSP1	203523_at	5.214939	5.425743	4.692943	4.425173	5.39601	5.742911	4.908182	4.497712	4.619985	4.966226	4.80773	4.912533	4.10142
MPST	203524_s_at	10.09111	10.15678	10.07799	10.4418	10.94421	11.01847	9.348288	9.745674	9.171668	9.304374	9.762405	9.939945	6.482691
APC	203525_s_at	6.887728	6.84775	7.204655	7.057256	6.736403	6.298706	7.946744	7.298564	8.214771	8.237218	7.051973	7.17842	9.642662
APC	203526_s_at	3.175943	3.089495	4.046144	3.339875	3.121523	3.047142	4.641244	3.125739	5.415826	4.54848	3.233272	3.258439	7.17415
APC	203527_s_at	3.125593	3.03637	3.022636	3.144473	3.114111	3.059364	3.262011	3.083858	3.123162	3.075649	3.067535	3.090001	5.942583
SEMA4D	203528_at	3.020323	2.840043	2.68074	2.841134	3.224345	3.352848	3.775555	3.511923	4.524501	4.204284	3.545786	3.848674	3.656614
PPP6C	203529_at	9.729634	9.719397	9.25847	9.471733	9.420556	9.52044	10.15511	10.33717	10.08421	10.21169	9.979766	10.36937	9.187786
STX4	203530_s_at	7.14072	6.70655	7.054501	6.877527	7.066737	7.231079	6.308193	6.903278	6.205363	6.433266	6.981079	6.899467	6.397407
CUL5	203531_at	9.011141	8.904345	9.172935	9.242984	8.714233	8.477688	9.058054	8.752906	9.344746	9.190737	8.771513	8.789483	9.540598
CUL5	203532_x_at	4.656093	4.565679	4.506464	5.299857	5.691303	5.598218	3.885071	4.937865	4.495388	4.658031	5.38687	5.878884	4.059492
CUL5	203533_s_at	9.040118	9.187883	9.178696	8.65973	8.719771	8.989469	8.731112	8.785477	8.756347	8.767473	8.654111	9.057611	9.231347
LSM1	203534_at	8.282044	8.502068	8.584533	8.798279	8.304218	8.219752	9.770068	9.098275	9.434734	9.55058	9.163089	9.431466	8.932882
S100A9	203535_at	3.606175	4.209172	7.02482	6.150083	6.523928	5.641415	5.521241	5.607485	5.249365	5.638879	6.237035	6.834959	5.497005
CIAO1	203536_s_at	6.462262	6.455676	6.543894	6.871299	6.574464	6.587635	6.56208	6.509629	6.456436	6.613712	6.897005	6.832199	5.995428
PRPSAP2	203537_at	6.845848	6.736586	7.127187	7.0451	7.27835	6.971595	8.420228	8.259256	8.471164	8.493451	8.016262	8.235379	8.608114
CAMLG	203538_at	9.514834	9.377035	9.207995	9.452996	9.863667	9.880755	8.175032	8.899613	8.212115	8.388512	9.85015	9.300064	10.06813
GFAP	203540_at	2.149074	2.133768	2.10409	2.115296	2.135489	2.114455	2.124102	2.167817	2.116982	2.109402	2.117505	2.126563	2.072947
KLF9	203541_s_at	2.378012	2.355406	2.328373	2.335603	2.370828	2.487611	2.350005	2.355948	2.333799	2.330942	2.341673	2.449456	2.217065
KLF9	203542_s_at	9.036029	8.936903	9.551554	9.530883	8.733129	8.852946	2.998474	3.418913	2.960786	2.62477	2.980577	3.522306	3.770305
KLF9	203543_s_at	10.15708	10.08155	11.09862	10.84137	9.364759	8.083136	4.363124	4.151406	4.684686	4.729896	3.61915	3.74447	4.366102
STAM	203544_s_at	5.775716	6.358034	6.718279	6.315063	6.052259	4.996784	8.892625	8.329011	9.141123	9.06228	7.36751	8.060866	8.447107
ALG8	203545_at	7.535744	7.535405	8.250979	7.974615	7.942691	7.633862	8.183676	7.52203	8.608581	8.417661	7.408401	7.733863	6.686371
IPO13	203546_at	6.041213	6.054297	5.408779	5.632583	5.861897	5.888501	5.817174	6.886451	5.498512	5.801559	6.310611	6.031553	5.053565
CD4	203547_at	7.527012	7.363612	7.971028	7.929913	7.894709	6.967513	6.823615	6.926794	7.201793	7.23862	6.088715	6.959894	3.769096
LPL	203548_s_at	2.082076	2.065153	2.039717	2.047776	2.066065	2.048594	2.055694	2.063028	2.048245	2.046244	2.046086	2.05697	5.007157
LPL	203549_s_at	2.335324	2.300958	2.24182	2.275726	2.304761	2.305946	2.290684	2.294236	2.278225	2.2769	2.279505	2.297332	5.338299

C1orf2	203550_s_at	5.892213	6.010277	5.60607	5.795578	5.987557	6.188345	6.368689	6.846159	6.055575	6.299948	7.065153	7.079784	6.730856
COX11	203551_s_at	6.979044	7.159839	6.046513	6.923618	7.102055	7.709086	6.93888	7.432864	6.939538	6.950832	7.181236	7.292747	7.373593
MAP4K5	203552_at	7.182779	6.897648	7.397475	7.167225	7.164123	6.548481	8.945006	8.376823	8.82803	9.119008	8.267095	8.168244	9.384517
MAP4K5	203553_s_at	3.993962	3.947502	4.57547	3.996848	3.457866	4.33898	7.01471	5.840579	7.092592	7.168696	5.59733	5.610751	8.225074
PTTG1	203554_x_at	5.683814	5.73311	3.204242	3.207519	2.586638	4.715349	10.92888	11.22995	10.91088	11.12782	10.9478	11.26166	8.996217
PTPN18	203555_at	4.390945	4.373367	4.369766	4.256351	3.91582	4.333247	4.67485	4.515229	4.357204	4.439884	4.180341	4.389135	4.259039
ZHX2	203556_at	8.215247	8.053921	8.549779	8.468919	8.112032	8.097688	7.588405	7.750764	7.668014	7.807557	7.621792	7.933638	6.435078
PCBD1	203557_s_at	9.255706	9.192505	10.34347	10.14908	9.327142	8.741444	7.710618	6.602208	7.523233	8.018291	6.454612	6.824108	6.986753
CUL7	203558_at	4.456396	4.823694	4.637284	4.656981	3.891976	4.089651	5.563928	5.133166	5.289188	5.180871	5.713519	5.17083	6.325185
ABP1	203559_s_at	2.729689	2.791897	5.763009	5.928573	5.175887	5.36987	2.604089	2.605697	2.587907	2.588812	2.59127	2.784664	2.416844
GGH	203560_at	10.22699	10.02053	10.38107	10.43289	10.20334	9.787805	10.74613	10.37522	10.43558	10.67446	9.808793	10.18858	9.010142
FCGR2A	203561_at	3.484648	3.974627	4.333051	4.290243	4.15346	3.91848	4.017239	4.000147	4.025524	4.146784	3.387246	3.993822	3.623474
FEZ1	203562_at	6.000901	5.692994	6.143594	6.521058	4.740517	5.390616	7.088516	6.253049	6.890188	6.442808	6.403927	6.906762	8.10677
AFAP1	203563_at	3.665964	3.45013	3.37817	3.498	3.717945	3.965091	3.492382	3.489504	4.11089	3.671036	3.764942	3.642143	6.390008
FANCG	203564_at	4.102513	3.383263	3.551309	3.311407	3.548119	3.51916	6.177293	6.453088	6.270806	6.334585	6.809147	6.757013	6.294244
MNAT1	203565_s_at	4.372633	4.579994	4.629273	4.631592	4.638765	4.558128	4.644077	4.639472	5.236954	4.633868	5.0848	4.632508	5.394572
AGL	203566_s_at	10.70684	10.59611	12.06084	11.85274	11.42139	10.95531	9.859273	9.446761	9.950133	10.14158	10.07118	9.485953	11.43345
TRIM38	203567_s_at	6.558637	6.350764	6.438102	6.706522	6.877623	7.272117	4.871259	5.507089	4.845455	4.367321	5.779387	5.517969	5.395259
TRIM38	203568_s_at	8.172118	8.323819	7.688495	7.828978	8.399798	8.439662	5.616623	6.673755	5.632881	5.905765	6.229313	6.355833	5.760245
OFD1	203569_s_at	7.391532	7.434683	6.509311	7.05452	7.478115	7.846172	7.270072	7.838594	7.168164	7.323429	8.036197	7.727215	6.891488
LOXL1	203570_at	2.631072	2.608158	2.562777	2.5561	2.639219	2.588669	2.600706	2.580629	2.584467	2.576892	4.484111	2.677103	7.491612
C10orf116	203571_s_at	9.933938	9.792968	8.610534	8.954319	9.158913	9.241543	3.73518	3.917261	3.717475	3.823658	3.920579	3.727266	3.319607
TAF6	203572_s_at	6.481895	6.501013	6.442687	6.131305	6.723596	7.089047	7.820937	7.164138	7.911465	7.805111	7.701373	7.678934	8.196419
RABGGTA	203573_s_at	5.317075	5.45396	5.345493	5.664204	5.726147	5.569446	5.355113	5.596874	5.52732	5.212866	4.968342	5.644798	4.943719
NFIL3	203574_at	7.508897	7.215884	10.11038	10.16736	8.779614	8.430973	6.700901	6.044373	6.813725	6.966644	6.239822	6.376184	7.494825
CSNK2A2	203575_at	6.238707	6.318921	6.30331	6.167237	6.334981	6.058277	6.698314	6.113753	6.224893	6.899785	6.756023	6.638901	6.487666
BCAT2	203576_at	5.551674	5.239433	5.384064	5.511562	5.534103	5.509099	6.236615	6.028122	6.157723	6.189279	6.013362	6.114526	6.30602
GTF2H4	203577_at	5.740943	5.251955	5.431359	5.1912	4.911313	4.76542	4.32145	4.966622	3.655697	4.273872	5.071342	4.340087	3.69421
SLC7A6	203578_s_at	2.948696	2.913399	2.819119	2.870877	2.948216	2.916721	2.902753	2.93396	2.891083	2.820172	2.968624	2.922428	3.584263
SLC7A6	203579_s_at	2.731127	2.529763	2.496724	2.502096	2.540508	2.509928	2.525667	2.528468	2.515295	2.506077	2.523143	2.525057	5.724204
SLC7A6	203580_s_at	3.139126	2.786724	2.766403	2.674134	2.643865	3.085392	4.19646	3.613178	4.288355	4.264849	4.213191	3.210088	6.179328
RAB4A	203581_at	8.898128	8.642701	9.519943	9.785463	8.983504	8.68291	8.842792	8.550954	9.014742	8.854031	8.426517	8.661825	8.817842
RAB4A	203582_s_at	7.574084	7.375872	8.70691	8.539469	7.683057	6.778077	7.937947	6.264373	7.998782	7.941641	6.60311	6.653666	8.189491
UNC50	203583_at	8.507523	8.493141	8.994827	8.840573	8.239895	8.053795	8.684269	8.219067	8.572356	8.834252	7.807759	8.434823	8.596431
TTC35	203584_at	8.660462	8.379617	9.048324	8.867892	8.307235	7.949169	8.002954	7.837	8.278441	8.284965	7.273437	7.73903	8.612851
ZNF185	203585_at	2.962375	3.095407	3.09905	3.062894	3.308451	3.10681	3.212594	3.04442	3.118439	2.943263	3.115808	3.127036	7.725746
ARL4D	203586_s_at	5.926679	5.99891	6.131478	5.405458	6.696314	6.143282	5.037648	5.215158	5.098952	4.921064	4.982678	5.257376	5.117852
ARL4D	203587_at	3.090461	3.058268	3.029397	3.044441	3.072855	3.047622	3.026255	3.061425	3.041351	3.01088	3.049224	3.102806	2.709825

TFDP2	203588_s_at	4.998276	5.027675	6.348455	6.624069	5.29138	5.67558	8.742046	8.513763	8.473238	8.563302	7.717057	8.28849	5.799323
TFDP2	203589_s_at	4.66284	4.835428	5.05666	5.236654	4.791511	4.823913	7.780891	7.313161	7.852417	7.953596	6.555577	6.84478	4.572848
DYNC1LI2	203590_at	8.12058	8.120937	8.083703	7.993622	8.174344	8.093572	7.831399	7.703754	7.445596	7.527662	8.46304	7.83708	9.629194
CSF3R	203591_s_at	4.571016	4.588265	4.15519	4.175041	4.602194	4.645509	8.232432	6.874399	8.17549	7.595056	7.137192	6.975939	2.582823
FSTL3	203592_s_at	2.145808	2.149977	2.126172	2.130653	2.41867	4.036739	1.917751	2.148295	2.115054	1.901028	2.13138	2.133031	2.246882
CD2AP	203593_at	8.974225	8.893958	8.750366	9.006582	8.710921	8.266915	9.999024	9.553926	10.26826	9.95474	9.160438	9.530623	7.26694
RTCD1	203594_at	8.18817	7.992833	8.28331	8.027489	7.583859	6.740573	9.287425	8.177675	9.212386	9.06988	8.003668	8.222272	9.324145
IFIT5	203595_s_at	7.893476	7.582892	7.259793	7.291153	7.547893	7.884193	6.239851	5.45194	6.194367	5.781328	6.745572	6.43959	8.708135
IFIT5	203596_s_at	6.788819	6.680546	6.496449	6.816391	7.223769	6.448128	5.409487	5.467958	5.613109	5.153391	5.231455	5.101476	6.823741
WBP4	203597_s_at	7.115415	6.850837	7.087572	7.138395	7.131173	7.107042	7.19942	8.021689	7.425937	7.332762	8.000219	8.170023	6.619058
WBP4	203598_s_at	6.415588	6.405328	6.214579	6.240194	6.523092	6.12307	7.968033	8.428247	8.140428	8.09089	7.763503	8.05095	7.247352
WBP4	203599_s_at	6.255341	6.345159	6.914618	6.776415	5.522164	5.606794	7.597135	6.23674	7.306865	7.44602	6.281843	6.323285	6.673616
C4orf8	203600_s_at	5.858333	5.850437	5.514195	5.833146	5.950766	5.585908	6.200082	6.251034	6.342542	6.105145	6.307649	6.383966	6.775662
ZBTB17	203601_s_at	2.896888	2.891934	2.875111	2.879796	2.889925	2.880212	2.890724	2.892273	2.880555	2.878576	3.014384	2.8843	2.850349
ZBTB17	203602_s_at	6.265966	5.936287	5.17909	5.544994	6.092351	6.472564	5.64928	6.348336	5.89437	5.929148	6.605982	6.696758	5.969591
ZEB2	203603_s_at	7.237834	7.277928	6.99982	7.161087	6.98298	7.34343	7.656404	7.39903	7.396097	7.399447	7.883793	7.692958	8.64317
ZNF516	203604_at	4.878893	5.460682	4.560177	4.094929	3.815485	3.738026	4.945593	4.275608	5.034422	5.022262	4.739221	4.095893	6.772624
SRP54	203605_at	7.327624	7.407133	7.856269	7.79442	7.29696	7.289293	7.973229	7.6711	8.133857	8.035172	7.433677	7.390465	7.685545
NDUFS6	203606_at	10.81746	10.71599	10.36943	10.88474	11.24032	11.70329	10.72874	11.62109	10.85345	10.89339	11.87355	11.82118	10.2527
INPP5F	203607_at	5.223458	5.506613	4.903156	4.90745	5.126646	5.398223	8.135469	7.869931	8.133061	8.116135	8.583885	8.007634	10.96802
ALDH5A1	203608_at	10.95823	10.91327	11.08337	11.0611	10.33896	9.821798	9.104673	8.707841	9.716115	9.144808	8.168869	8.482475	7.753931
ALDH5A1	203609_s_at	4.514835	4.209249	4.967076	4.696544	4.049516	3.983256	3.582974	3.797818	3.764954	3.046282	3.706764	3.642274	2.15308
TRIM38	203610_s_at	3.096893	2.792843	3.972312	3.710296	2.786392	2.779305	2.674494	2.521411	2.670485	2.489892	2.539904	2.64744	2.832519
TERF2	203611_at	6.981046	6.737757	6.810486	6.738083	6.855636	6.358022	7.250747	7.200343	7.834184	7.391223	7.014196	7.06524	7.666534
BYSL	203612_at	5.896341	5.873505	5.753278	5.850618	5.745893	6.136844	7.048291	6.68828	6.960768	7.016805	6.764215	6.856998	6.479065
NDUFB6	203613_s_at	10.55033	10.37836	10.9732	11.04937	10.45786	10.28669	11.39172	10.80138	11.34986	11.2983	10.85463	10.83056	10.87989
UTP14C	203614_at	7.843952	7.608643	8.229524	8.109561	7.464888	7.301406	8.45596	8.165412	8.673802	8.481853	8.198955	8.045317	8.408074
SULT1A1	203615_x_at	11.07326	11.2224	11.21344	11.29658	11.44214	11.3767	9.26745	9.744386	9.18205	8.244092	9.996323	9.863563	5.792027
POLB	203616_at	7.493652	7.42611	8.546862	8.51276	7.780045	7.44264	6.715711	6.605053	6.873688	6.960508	6.973723	6.708598	8.499212
ELK1	203617_x_at	7.080081	7.039515	7.110697	6.927016	6.836508	7.214513	6.438039	6.143203	6.061192	6.116938	5.882101	6.594319	6.486629
FAIM2	203618_at	2.433436	2.409495	2.393096	2.394765	2.431726	2.392742	2.403263	2.399353	2.393783	2.38768	2.39464	2.400085	2.38062
FAIM2	203619_s_at	2.745339	2.708485	2.67745	2.67721	2.715333	2.685385	2.693449	2.704384	2.700472	2.695233	2.694445	2.700347	4.751107
FCHSD2	203620_s_at	7.461156	7.122383	7.305534	7.250682	7.429687	7.013505	7.07025	6.378658	6.849288	6.658425	6.710371	6.357192	7.930432
NDUFB5	203621_at	10.4001	10.42426	10.61103	10.64789	10.307	9.935384	10.21987	9.944129	10.23329	10.38278	10.18336	10.11399	10.52312
PNO1	203622_s_at	4.732093	4.654006	5.824301	5.393117	4.847701	5.068811	7.040136	6.153489	7.340194	7.235921	5.939772	6.176622	6.6451
PLXNA3	203623_at	2.492744	2.441398	2.65639	2.494423	2.694624	2.503122	2.445636	3.182284	2.410599	2.419422	3.825155	2.858556	5.254481
SFRS17A	203624_at	9.078644	9.13525	8.743665	8.65939	8.964369	8.623347	9.783254	9.333714	9.50294	9.437477	9.383765	9.307653	9.24953
MCAM	203625_x_at	9.06619	8.992238	8.110179	8.419567	8.675614	8.760312	10.01708	10.39541	10.12777	9.96097	10.21201	10.23675	9.648079

SKP2	203626_s_at	2.51159	2.472194	2.425529	2.460549	2.496904	2.446554	2.825763	2.481014	3.484391	2.706941	2.468715	2.478008	5.84828
IGF1R	203627_at	2.691493	3.103832	2.972139	2.977194	3.174716	2.977786	4.849377	3.390447	3.862177	3.738464	4.653618	3.510189	7.632622
IGF1R	203628_at	4.665204	4.562696	4.750691	5.220447	4.80614	5.15023	6.704268	6.321547	5.46092	5.81413	7.637896	6.698925	8.78284
COG5	203629_s_at	4.942462	4.862955	5.49603	5.273031	4.834571	4.954649	5.949937	5.953964	6.427945	6.036052	5.921227	5.669839	6.940746
COG5	203630_s_at	4.811737	4.602162	5.46572	5.298997	4.792243	4.773468	6.659248	5.185734	6.787895	6.381388	5.583091	5.655804	7.836495
GPRC5B	203631_s_at	2.185015	2.163114	2.143295	2.143632	2.206792	2.142098	2.151801	2.156008	2.144814	2.139497	2.143971	2.156595	2.107828
GPRC5B	203632_s_at	2.54317	2.319262	2.343919	2.251983	2.5586	2.72173	2.169915	2.119722	2.120851	2.082745	3.423131	2.194285	5.991239
CPT1A	203633_at	8.080811	8.047751	9.170532	9.444756	8.951624	8.830059	6.869002	6.661414	7.437838	7.173184	6.073106	6.69258	5.921552
CPT1A	203634_s_at	2.388449	2.427726	2.597814	2.750977	2.765259	2.513416	2.311323	2.527285	2.516288	2.603897	2.418543	2.521033	2.196138
DSCR3	203635_at	6.738693	6.357722	7.149207	6.610283	5.775083	5.676727	6.468978	5.437676	6.330195	6.539568	5.519202	5.63319	6.152027
MID1	203636_at	6.59327	6.987071	6.57198	6.731335	6.2407	6.58348	5.916851	6.472625	6.029451	6.213612	8.636841	6.530272	9.33718
MID1	203637_s_at	6.584367	6.397245	7.25407	7.221725	5.842344	6.050443	5.523831	5.942344	5.814875	5.566563	8.389296	6.198295	9.193445
FGFR2	203638_s_at	9.245881	9.149467	8.1308	8.147594	7.338302	7.218528	9.053726	7.530007	7.993931	8.064802	7.301347	8.051541	9.347999
FGFR2	203639_s_at	3.467717	3.506393	4.104728	3.20627	2.679874	2.610534	5.523034	2.666006	4.156501	4.494245	2.659523	2.672087	6.404795
MBNL2	203640_at	8.87729	8.716874	9.490692	9.304948	8.427745	8.024154	7.037924	6.711225	8.179926	8.296324	6.738429	6.551628	9.040641
COBLL1	203641_s_at	3.921236	2.977466	6.941457	5.818803	3.189664	2.674323	4.57584	2.235964	4.795118	4.63944	2.602451	2.229574	2.782642
COBLL1	203642_s_at	10.42532	10.29223	11.62615	11.1187	10.63698	9.996241	8.747967	8.876625	9.232495	9.234668	7.516414	8.087579	6.117024
ERF	203643_at	5.747204	5.155866	5.064678	5.198642	5.078115	4.85179	4.840108	4.851259	4.386051	5.039837	4.834088	4.843974	5.467144
MON1B	203644_s_at	5.123786	5.073993	5.472279	5.47959	5.288837	5.673917	5.717429	5.894788	5.769302	5.745147	5.316043	5.936404	4.28535
CD163	203645_s_at	8.530404	8.322053	10.31659	10.47558	9.420684	8.6699	8.750413	8.014705	8.604459	8.452613	7.402205	8.334751	6.687192
FDX1	203646_at	9.254583	9.13354	9.344423	9.335626	9.191941	8.928517	8.124997	7.789006	8.233077	8.140827	7.273607	8.010113	10.08302
FDX1	203647_s_at	10.22738	10.04414	10.55685	10.57164	9.738422	9.559684	8.767851	7.588867	8.766729	8.572068	7.690987	7.833969	10.97878
TATDN2	203648_at	6.371475	6.397053	5.905594	5.683848	6.319283	6.735284	6.091387	6.9965	6.437463	6.275082	6.588851	6.866704	5.782093
PLA2G2A	203649_s_at	9.204098	9.107304	6.639808	4.045578	10.20409	9.389603	2.886465	2.974543	2.88324	2.623669	3.271921	3.0514	3.633737
PROCR	203650_at	5.386472	5.357025	5.177753	5.348159	5.656126	5.407027	4.448472	5.170904	5.277077	5.164014	6.104372	5.30314	6.8113
ZFYVE16	203651_at	8.544866	8.655739	8.648896	8.851284	8.923446	8.886889	8.309008	8.37968	8.354153	8.108811	8.911626	8.375653	9.720585
MAP3K11	203652_at	7.760607	7.930945	6.852845	7.179076	7.826975	8.06131	6.070071	7.022693	6.067401	5.877315	6.916143	7.379857	4.451912
COIL	203653_s_at	4.710903	4.628409	4.824657	5.079367	4.958391	4.855918	5.625625	5.151813	5.263903	5.55178	5.610867	6.095492	5.024045
COIL	203654_s_at	7.777219	7.863322	7.673534	7.741203	7.543824	7.309842	8.616228	8.665536	8.783275	8.969983	8.743613	8.692406	8.683363
XRCC1	203655_at	4.85791	4.773824	4.174828	4.755446	4.222246	4.465445	6.05837	6.46625	6.082092	5.87234	6.011266	5.824132	5.622596
FIG4	203656_at	6.620789	6.127784	6.082768	6.569721	6.229086	6.529959	6.064291	6.378297	6.119697	6.102181	6.637878	6.496908	7.064693
CTSF	203657_s_at	9.683639	9.708175	9.329459	9.677799	10.09644	10.23744	6.806121	6.857345	6.807861	6.753251	7.130038	7.056704	6.106187
SLC25A20	203658_at	8.451787	8.425658	9.386222	9.117134	8.631087	8.097606	8.571411	8.10149	8.626091	8.287385	7.452534	8.042653	6.038025
TRIM13	203659_s_at	6.093034	6.058962	6.231475	6.327242	6.499939	6.040346	6.089282	5.95569	6.289531	6.303284	6.29156	6.224731	7.283182
PCNT	203660_s_at	6.233654	6.364295	5.913896	6.054872	5.829834	5.569467	8.46909	8.405789	8.485889	8.658882	8.028138	8.17616	6.743719
TMOD1	203661_s_at	3.50082	3.393828	4.816994	4.840788	4.75764	4.171875	8.016935	7.793437	8.032521	8.512009	7.420602	7.900592	6.304964
TMOD1	203662_s_at	5.234853	5.37148	6.312058	6.422796	6.655346	6.418889	8.522412	8.724537	8.654867	8.900911	8.311318	8.641136	6.625809
COX5A	203663_s_at	11.9861	11.76882	11.86	11.94086	11.72773	11.79466	11.58139	11.54993	11.6079	11.57985	11.83536	11.65372	11.58084

POLR2D	203664_s_at	5.876019	6.271565	6.435612	6.618496	6.129156	6.31746	7.840065	7.649794	8.020021	7.714763	7.037843	7.965862	7.150322
HMOX1	203665_at	7.605839	7.403766	7.74226	7.627067	7.867373	7.608567	8.95957	8.374277	8.253763	8.567271	5.661996	8.966119	5.189942
CXCL12	203666_at	8.747592	8.808536	9.16225	9.046218	9.485336	9.005014	8.62312	6.532972	7.760639	7.961718	8.386571	7.6884	9.447659
TBCA	203667_at	12.39427	12.06726	12.62618	12.81625	12.37277	12.35322	12.48074	12.36401	12.45395	12.45398	12.53831	12.58702	12.4021
MAN2C1	203668_at	6.600562	6.508619	6.04509	6.290478	6.513706	6.889291	5.73836	6.105222	5.36133	6.072203	6.228865	6.116225	5.29318
DGAT1	203669_s_at	7.170963	7.086534	7.279835	7.556674	7.337908	7.06509	7.025582	6.9159	7.037256	7.262598	6.781503	7.306638	5.243477
TTLL3	203670_at	2.347158	2.321691	2.294272	2.307227	2.331667	2.358524	2.317131	2.302413	2.307774	2.302819	2.31133	2.312021	2.411214
TPMT	203671_at	6.959283	6.828348	7.316646	7.668951	7.314929	7.348419	4.482941	4.879831	4.706347	5.001371	4.609073	5.386064	3.630238
TPMT	203672_x_at	4.889774	4.880656	5.670407	5.777351	5.016423	4.862949	4.143746	4.878765	4.304269	4.860187	3.699646	4.309825	3.83508
TG	203673_at	2.299808	2.271695	2.235512	2.257296	2.270723	2.273717	2.260199	2.27113	2.256116	2.245299	2.251624	2.265995	2.156008
HELZ	203674_at	6.644888	6.804065	6.793092	7.107786	6.622336	4.988942	7.040017	6.091423	7.29261	7.196038	6.41784	6.039612	6.712428
NUCB2	203675_at	8.976751	8.795836	9.251469	9.661809	10.1878	10.27425	9.778555	10.42034	10.17034	9.904744	10.26474	10.61195	8.998316
GNS	203676_at	2.243998	2.233613	2.214171	2.201256	2.232554	2.223969	2.234588	2.227477	2.227935	2.370132	2.196672	2.225722	3.653064
TARBP2	203677_s_at	5.786559	5.950353	5.673998	5.685592	5.797597	6.290879	6.627548	7.285149	6.689844	6.653389	7.127885	7.41151	6.081
MTMR15	203678_at	7.285015	7.300847	7.477139	7.393007	7.415967	7.536964	6.857616	7.052634	6.926135	6.849222	7.157416	6.997058	7.154353
TMED1	203679_at	7.039702	7.177124	6.456884	6.750912	6.840684	7.090386	6.440806	6.747342	5.961218	6.227386	7.006829	7.06686	6.03721
PRKAR2B	203680_at	3.645807	3.273556	5.161744	4.848417	3.672869	4.066553	10.22376	8.716593	10.02248	10.17325	7.939691	8.879694	8.513554
IVD	203681_at	2.248201	2.230625	2.206847	2.209785	2.232604	2.21469	2.217999	2.224226	2.213508	2.205045	2.212471	2.223331	2.120306
IVD	203682_s_at	7.799907	7.72488	8.372216	8.379926	8.8958	8.41853	4.910562	5.243064	5.577098	5.05475	4.655802	4.839489	5.898894
VEGFB	203683_s_at	4.415957	4.417121	4.632156	4.510018	4.398823	4.707258	4.389021	4.341344	4.380983	4.377442	4.376568	4.557448	4.634769
BCL2	203684_s_at	2.501763	2.473511	2.449997	2.438151	2.474314	2.437205	2.469357	2.470129	2.454082	2.440862	2.453283	2.466905	2.468872
BCL2	203685_at	4.687421	4.653565	4.479573	4.564857	4.65988	6.005878	3.852472	4.106655	3.809079	3.669344	5.556701	3.984501	8.095172
MPG	203686_at	7.666135	7.60349	7.342423	7.614824	7.487777	7.608613	7.080157	7.0274	6.856042	6.940951	7.306235	7.484746	6.038232
CX3CL1	203687_at	2.644726	2.624871	2.603246	2.608547	2.794206	2.619718	2.614533	2.625524	2.613212	2.590533	2.607778	2.617666	2.474175
PKD2	203688_at	6.612775	6.832955	9.130619	8.828004	8.300616	7.469333	7.491062	6.894951	7.399413	7.37233	6.612172	6.297048	9.564318
FMR1	203689_s_at	9.211568	9.263256	9.194243	9.009139	8.837161	8.289489	8.661979	7.921009	8.718468	8.766265	7.992834	7.595995	9.378425
TUBGCP3	203690_at	8.060219	8.061495	8.368109	8.413893	7.886596	7.70837	8.793762	8.538211	9.231962	9.048655	8.230207	8.667409	8.500964
PI3	203691_at	2.272355	2.257599	2.23417	2.240744	2.261108	2.239139	2.25162	2.254374	2.243301	2.235405	2.233865	2.252548	2.123176
E2F3	203692_s_at	3.163804	3.927436	3.441408	3.583929	3.677832	3.146545	4.101223	3.930085	3.927237	4.137956	4.963685	4.037456	4.277497
E2F3	203693_s_at	4.883012	4.865498	4.087971	4.64681	4.92612	5.05311	6.528253	7.049327	6.841708	6.845258	7.610849	7.011008	6.405893
DHX16	203694_s_at	7.367464	7.16732	7.142983	7.129779	7.144897	7.116012	7.216585	6.987709	7.398251	7.265811	7.305463	6.998498	7.066178
DFNA5	203695_s_at	4.503219	4.423838	4.620032	4.629972	4.602014	5.431955	5.379381	6.049582	5.362858	5.187402	5.905441	5.600379	6.896472
RFC2	203696_s_at	5.691104	5.357179	5.559873	5.509271	4.816345	4.454292	7.309798	7.337207	7.565587	7.419999	6.629248	7.180617	6.897222
FRZB	203697_at	2.905065	3.244623	4.068701	4.215894	3.311162	3.477091	3.127485	2.645359	2.6952	3.622362	3.87216	2.954154	7.725032
FRZB	203698_s_at	2.509493	2.618422	4.370854	2.621415	2.600203	2.937965	2.623852	2.628504	2.678443	2.893613	2.624307	2.589273	7.669797
DIO2	203699_s_at	2.223443	2.20246	2.173749	2.182825	2.205636	2.177821	2.191083	2.199931	2.184647	2.177337	2.186989	2.194807	2.188523
DIO2	203700_s_at	2.733344	2.711977	2.779337	2.667372	2.714554	2.69402	2.707098	2.711778	2.71849	2.690345	2.697517	2.706604	2.885737
TRMT1	203701_s_at	5.808365	5.966148	5.921134	5.81812	5.971529	5.950558	6.0457	6.215745	6.122517	6.166986	6.404006	6.939672	5.866885

TTLL4	203702_s_at	2.786108	3.488071	3.34266	3.377076	2.774515	3.455169	7.107732	6.539128	7.127425	7.024075	5.766442	6.714205	4.86155
TTLL4	203703_s_at	2.521841	2.551977	2.498948	2.537018	2.567656	2.519098	2.531789	2.549017	3.095024	2.673324	2.531382	2.545113	2.397246
FZD7	203705_s_at	2.72155	2.699862	2.679054	2.680584	2.690307	2.649709	3.252453	2.688301	2.681817	2.68543	4.667462	2.685905	8.736198
FZD7	203706_s_at	3.820959	3.811251	3.881869	3.74309	3.796719	3.798245	3.808941	3.80962	3.795568	4.004921	7.200583	3.800715	9.599323
ZNF263	203707_at	7.261068	7.462551	6.219186	6.136502	6.761598	6.869989	6.723432	6.90218	6.594925	6.840975	6.851232	6.941283	7.094801
PDE4B	203708_at	3.656406	3.594363	6.363299	5.928383	4.768071	5.222103	3.200058	2.60498	3.059897	3.316266	3.495878	2.593806	7.567356
PHKG2	203709_at	5.664672	5.406987	5.309657	5.423658	5.328944	5.434901	5.453116	5.36084	5.321217	5.608265	5.358476	5.558364	4.429319
ITPR1	203710_at	6.317154	6.198795	6.25979	6.059213	6.355855	6.147104	4.63652	5.038773	4.706959	4.577983	6.477162	4.872325	6.904608
HIBCH	203711_s_at	9.170571	9.238247	9.165561	9.121412	9.409652	9.028526	8.324991	7.746668	8.878914	8.436355	7.825671	8.37424	7.288469
KIAA0020	203712_at	6.80321	6.750719	6.011337	6.325698	6.757817	6.809808	6.813873	6.808367	7.192768	6.801331	6.810976	6.972334	6.956263
LLGL2	203713_s_at	6.093687	5.92463	5.731179	6.023597	6.495089	6.335162	4.156512	4.294951	4.389512	4.474312	4.351759	4.252404	3.802991
TBCE	203714_s_at	7.125081	6.777415	6.816006	6.951057	6.881313	6.674748	7.234021	7.175658	7.469731	7.398384	7.015966	7.433103	7.114696
TBCE	203715_at	5.576735	5.37529	5.926522	5.324025	4.535287	4.115081	6.474105	6.215959	6.818257	6.314536	5.63241	4.846144	5.925057
DPP4	203716_s_at	6.937062	6.358717	7.595942	7.45647	6.339611	6.204266	5.170188	4.038231	5.895843	5.836559	3.543462	3.996078	3.698527
DPP4	203717_at	9.992613	10.01825	9.78594	9.848244	9.534348	9.259555	7.239089	7.941683	8.285016	8.053292	7.280151	7.193387	5.774638
PNPLA6	203718_at	8.041634	7.949515	7.077819	7.516008	8.067377	8.184407	9.003843	9.416858	8.846506	8.976755	8.851597	9.443974	6.015546
ERCC1	203719_at	7.001418	6.97073	6.779811	7.10407	7.777789	8.152046	6.974704	8.241277	7.017533	7.218114	8.305533	8.445111	7.512615
ERCC1	203720_s_at	7.04541	7.080625	7.363182	7.354881	7.377206	7.640729	7.379055	7.847573	7.372025	7.382379	7.966961	7.737171	8.03534
UTP18	203721_s_at	8.37071	8.37548	8.628762	8.497076	8.527068	7.859282	9.525967	9.13305	9.504239	9.375543	8.876541	9.105221	9.156317
ALDH4A1	203722_at	10.73315	10.53003	10.61073	10.54373	10.03856	9.69361	9.561782	9.258353	9.218502	9.098085	10.00019	9.087849	4.375287
ITPKB	203723_at	5.046617	5.023273	4.912351	5.010854	5.17988	5.0216	5.032597	5.022549	5.027976	4.932799	5.742299	5.043804	7.049043
RUFY3	203724_s_at	3.420619	3.332263	3.479379	3.689455	3.236024	3.219395	3.175071	3.16824	3.200166	3.221948	3.351086	3.260103	8.566743
GADD45A	203725_at	10.14763	9.898374	10.55319	10.41628	9.696436	9.417245	9.453852	8.869089	9.960098	9.962819	8.393652	8.583899	7.389414
LAMA3	203726_s_at	2.168514	2.138289	2.104561	2.189853	2.128729	2.108606	2.119346	2.127057	2.111959	2.104464	2.112146	2.120461	2.758477
SKIV2L	203727_at	7.186397	7.290984	6.818688	7.138378	7.815173	7.96263	7.098898	7.265558	7.099322	7.10298	7.986249	7.648155	5.351989
BAK1	203728_at	4.269348	4.277777	4.237808	4.241881	4.272869	4.425698	4.276021	4.264204	4.270929	4.260745	4.834725	4.267636	4.251391
EMP3	203729_at	4.882571	5.177976	5.471237	5.222402	5.551435	6.152026	7.309416	6.4309	6.858756	6.672333	7.100315	6.864939	9.289015
ZKSCAN5	203730_s_at	4.188395	3.302747	3.982084	3.93362	3.748494	4.394341	4.489223	3.993608	5.153013	4.291681	4.192769	4.274417	5.748079
ZKSCAN5	203731_s_at	3.162108	3.126986	3.484651	3.110535	3.126073	4.103425	3.122628	4.220173	3.116848	3.418135	3.671039	3.837554	3.807342
TRIP4	203732_at	6.589339	6.690157	6.979247	6.957125	7.039208	6.528638	7.388249	7.156171	7.936046	7.655853	6.899557	7.48709	8.188707
DEXI	203733_at	7.696816	7.845282	8.264164	8.667011	8.135722	8.153343	6.938218	7.373276	6.49395	7.082046	7.38978	7.314054	6.839984
FOXJ2	203734_at	6.964387	6.710665	6.336492	6.596924	6.930209	7.456553	7.632225	8.241748	7.710954	8.134005	7.778592	8.043737	7.2901
PPFIBP1	203735_x_at	6.924489	6.897174	7.265919	7.35553	7.201127	7.21288	7.027842	6.201027	7.179063	7.110843	6.012215	6.195273	7.538755
PPFIBP1	203736_s_at	2.696005	2.67207	2.627968	2.640899	2.700899	2.724038	2.625669	2.658934	2.643415	2.598742	2.638406	2.646643	4.76063
PPRC1	203737_s_at	6.119712	6.306448	6.651228	6.460646	6.310442	6.051438	7.387274	7.497933	7.823162	7.729408	8.08898	7.620832	7.329696
C5orf22	203738_at	7.301522	7.140398	7.464559	7.436391	6.86793	6.079659	8.287802	7.844692	8.063935	8.621148	7.412954	7.971704	7.868843
ZNF217	203739_at	8.101339	7.895256	7.705924	7.490623	8.098831	7.835349	8.208873	7.602907	8.478773	8.271023	7.701821	8.210355	7.66266
MPHOSPH6	203740_at	5.895013	5.690181	6.235488	6.242088	4.350026	5.070325	7.553603	6.441027	7.541461	7.661666	7.247454	7.270228	7.907614

ADCY7	203741_s_at	5.233112	5.079094	4.836365	4.257832	5.163965	4.989396	7.500824	7.47749	7.40665	7.262347	6.547882	7.195816	5.096396
TDG	203742_s_at	6.933862	6.722967	6.400492	6.636847	7.024639	7.393623	7.6894	7.78163	6.977688	6.792026	8.069346	8.353315	7.588774
TDG	203743_s_at	8.528204	8.380283	8.156527	8.44711	8.321686	8.046344	9.763161	9.730901	9.948135	9.820021	9.622431	9.887129	9.936001
HMGB3	203744_at	3.557474	4.344026	4.7302	4.750623	4.197634	4.155555	5.769463	6.227154	5.850941	6.030516	5.971342	6.305324	7.107065
HCCS	203745_at	7.069046	7.109195	7.002055	6.654203	6.89278	6.422923	7.573596	6.939057	7.022552	7.763022	6.767433	7.153341	7.135044
HCCS	203746_s_at	6.592072	6.360554	6.626713	6.684324	6.65321	6.635283	7.16359	7.131514	7.40965	7.458467	6.778456	7.121057	5.477074
AQP3	203747_at	4.127188	4.216359	4.744894	4.422791	4.35157	3.884472	4.930796	4.123923	4.744023	5.143883	4.056989	4.389962	3.849971
RBMS1	203748_x_at	7.012582	6.765989	7.733299	7.171168	6.551292	5.211806	7.20934	6.055305	7.156513	7.010897	6.4738	6.543171	8.331681
RARA	203749_s_at	4.512427	4.665192	5.299116	5.097929	5.069502	4.754017	4.56144	4.434543	4.461587	4.52541	4.816086	4.852056	5.284471
RARA	203750_s_at	4.611792	5.01488	4.554387	4.744525	4.788409	4.867939	4.797186	5.129751	4.666512	4.71967	4.811633	5.058259	4.572833
JUND	203751_x_at	3.189632	3.375137	4.709004	4.583424	3.64876	3.790791	3.919828	3.526773	3.915514	3.915521	3.021695	2.962841	4.21452
JUND	203752_s_at	11.12917	11.19978	11.58513	11.80303	11.16845	11.18715	11.00224	11.34026	10.82687	11.12331	11.38526	11.26912	10.81764
TCF4	203753_at	7.842604	7.585086	7.977179	8.205942	8.586605	8.697674	7.437522	7.801585	7.107506	7.039098	9.757795	8.179222	11.12984
BRF1	203754_s_at	4.08092	3.899209	3.759637	3.796149	3.786918	3.791005	3.800576	4.326211	3.767597	3.793107	3.811124	3.800556	3.970059
BUB1B	203755_at	2.524386	3.697982	2.341072	2.356187	2.499822	2.356521	8.748015	7.986358	9.085474	9.071331	7.928054	7.83115	8.177381
ARHGEF17	203756_at	3.906579	4.191469	3.764189	3.683286	4.04253	4.035294	3.912354	4.032085	3.436278	3.631792	4.714225	3.860107	5.237974
CEACAM6	203757_s_at	2.167252	2.147077	2.119275	2.128338	2.146441	2.129083	2.14143	2.143138	2.131918	2.126821	2.129633	2.140935	2.07861
CTSO	203758_at	8.69286	8.294956	9.092071	9.13137	9.022732	8.423979	6.61024	5.86696	6.682567	6.895648	6.042349	6.088956	6.305391
ST3GAL4	203759_at	5.189442	4.60389	3.799095	4.30114	4.250332	4.432571	3.250892	3.170525	3.514588	3.219747	3.796955	3.527062	3.639084
SLA	203760_s_at	5.401303	4.785743	6.643335	6.437705	6.028603	6.72363	6.064562	5.911665	5.527382	5.889947	6.284756	6.178878	4.698257
SLA	203761_at	5.429944	5.460595	6.532666	6.269502	6.01259	6.549019	5.416617	5.812336	5.296932	5.222222	5.358499	5.784786	5.097551
DYNC2LI1	203762_s_at	5.349588	4.665248	5.939175	6.005324	5.337318	5.300317	5.055644	5.173379	5.685087	5.689389	5.62365	5.319041	6.993446
DYNC2LI1	203763_at	7.20899	6.889832	7.256057	7.266257	7.469313	7.431829	5.706677	7.055478	7.200162	6.647937	7.418763	6.803815	7.59212
DLG7	203764_at	2.759259	3.393434	2.533965	2.538234	2.567189	2.538577	10.37236	9.816787	10.58077	10.56256	9.530944	9.488738	8.043219
GCA	203765_at	5.913714	5.809173	6.072127	5.82386	5.491395	5.38586	6.62144	5.539351	5.865719	6.015567	5.636071	5.970639	5.801814
LMOD1	203766_s_at	2.184728	2.175515	2.252871	2.341635	2.192144	2.368557	2.168619	2.169445	2.157975	2.156361	2.151622	2.165682	2.960896
STS	203767_s_at	3.362041	2.611693	5.175996	5.301225	6.416845	5.9734	3.740719	3.390123	3.595089	3.684094	4.017562	4.171213	4.979275
STS	203768_s_at	2.275767	2.266777	2.249664	2.258155	2.535321	2.252256	2.262158	2.26377	2.254748	2.220271	2.244869	2.278201	2.318045
STS	203769_s_at	4.115568	4.088696	5.085686	5.366959	5.955499	5.666531	4.476347	4.580978	4.335294	4.557955	4.575889	4.588028	4.509397
STS	203770_s_at	2.362884	2.33959	2.295078	2.315472	2.346533	2.316092	2.323385	2.336209	2.320475	2.313993	2.32004	2.332705	2.237317
BLVRA	203771_s_at	3.338726	3.278178	3.318869	3.267119	3.373331	3.239562	6.890048	5.129665	6.595111	7.603689	3.829372	5.491969	5.32279
BLVRA	203772_at	2.959054	2.917677	2.910779	2.919513	2.943351	2.917143	2.943172	2.930807	2.921678	2.914159	3.05637	2.932	2.844833
BLVRA	203773_x_at	5.980195	6.032387	5.738697	5.926123	6.322321	6.361723	8.88715	9.18824	8.667665	8.71682	8.719452	9.349525	7.028526
MTR	203774_at	8.561317	8.523238	8.980683	8.958191	8.477137	8.434056	9.236518	9.18733	9.42692	9.332479	9.2431	9.012341	9.055481
SLC25A13	203775_at	11.67802	11.73172	11.30364	11.44519	10.88452	10.87183	10.04981	10.42282	10.10376	10.01881	10.20512	10.3253	7.533092
GPKOW	203776_at	6.258009	6.263806	6.551869	6.620833	6.241019	6.517466	6.769755	7.151129	6.94029	7.079323	6.877242	6.75925	5.931359
RPS6KB2	203777_s_at	5.283542	5.001206	5.651112	5.454946	5.800885	5.745013	5.041506	6.237471	5.597167	5.759975	5.615554	6.435116	4.068571
MANBA	203778_at	6.131783	6.115524	5.925779	6.198041	6.038833	5.864521	6.386074	5.971937	6.102019	5.921594	6.216795	6.24354	5.185229

MPZL2	203779_s_at	2.334728	2.314082	2.290407	2.29413	2.317215	2.292164	2.311748	2.313488	2.301409	2.288586	2.295302	2.317244	2.284437
MPZL2	203780_at	7.251183	6.661008	6.526232	6.766293	5.94752	5.934728	6.776529	6.629107	6.67641	6.268188	6.238577	6.991021	7.340214
MRPL33	203781_at	10.14707	10.08565	10.3199	10.55845	10.35291	10.45853	10.31882	10.44177	10.59348	10.43013	10.41429	10.50007	9.859834
POLRMT	203782_s_at	7.480688	7.421783	6.808842	7.311432	7.476537	7.740264	6.741404	7.362074	6.932151	7.059532	7.720111	7.876822	4.977725
POLRMT	203783_x_at	2.060451	2.052986	2.031598	2.034463	2.046464	2.039625	2.05118	2.062096	2.046095	2.114914	2.031697	2.044827	2.018444
DDX28	203784_s_at	2.077597	2.062364	2.04108	2.045831	2.062758	2.044582	2.058974	2.058889	2.046936	2.040891	2.040731	2.053791	1.99623
DDX28	203785_s_at	5.595608	5.88743	5.83064	6.35267	5.283428	5.563802	5.16904	6.372124	5.411245	5.792412	6.150341	6.067866	4.6193
TPD52L1	203786_s_at	8.819334	8.629277	9.052788	9.087308	8.48013	8.492116	6.606511	6.707959	6.639191	6.505567	6.87262	6.978354	6.867983
SSBP2	203787_at	5.812534	5.415739	5.889134	5.330765	5.394267	5.278976	8.373198	7.833965	8.194797	8.20773	8.28062	8.009094	9.711454
SEMA3C	203788_s_at	2.450235	2.444475	2.420774	2.420467	2.585637	2.398995	2.430126	2.442427	2.423917	2.420485	2.433379	2.437713	6.34226
SEMA3C	203789_s_at	2.894319	3.026883	2.850722	2.84621	2.897435	3.488047	2.820172	2.82768	2.838355	2.815495	5.137496	2.859786	10.51603
HRSP12	203790_s_at	11.80013	11.71351	12.85513	12.52249	11.4826	10.46418	10.18555	8.739266	10.4137	10.03174	7.90645	8.13899	6.794249
DMXL1	203791_at	8.755101	8.711064	9.305644	9.142173	8.514113	8.166791	9.23208	8.650449	9.223448	8.874394	8.405816	8.578085	8.600849
PCGF2	203792_x_at	2.55067	2.569182	2.524227	2.574813	2.574568	2.552654	2.80301	2.573553	2.564575	2.956578	2.711163	2.989565	2.526158
PCGF2	203793_x_at	3.607404	4.107619	3.320516	3.367187	3.202955	3.464838	4.262204	4.051589	4.710932	4.49704	4.628917	4.753286	5.992729
CDC42BPA	203794_at	7.603135	7.555621	7.646948	7.861102	7.352053	7.559191	7.980202	7.675387	7.474107	7.703465	7.411396	7.665595	7.624027
BCL7A	203795_s_at	4.693572	4.704261	4.351458	4.444969	4.454357	4.358757	5.227149	4.904316	5.007805	5.073985	4.988339	4.687544	5.66456
BCL7A	203796_s_at	4.957569	4.915306	4.745078	4.899725	4.916452	4.901253	4.952426	5.094279	5.246416	4.889021	5.31677	5.394374	4.834577
VSNL1	203797_at	7.762295	7.587366	8.357586	8.142784	7.68219	7.288422	9.547597	8.459553	9.291662	9.174223	8.24491	8.487594	7.611596
VSNL1	203798_s_at	5.254348	5.029753	6.740911	5.925053	5.266235	4.11089	7.613106	5.993526	7.710927	7.531213	5.724371	5.835703	6.301525
CD302	203799_at	11.29258	11.18441	11.97269	11.74046	11.20197	10.76832	10.4134	9.570073	10.55753	10.21089	9.352594	9.699414	7.490231
MRPS14	203800_s_at	7.549222	7.529386	7.640344	7.844145	7.533654	7.470714	8.070013	7.715744	8.040044	7.872589	7.755537	8.276961	7.233874
MRPS14	203801_at	7.505101	7.142729	7.368382	7.311481	7.280048	7.1604	6.86395	6.939931	6.927981	6.653518	6.953527	7.083734	6.907876
NSUN5	203802_x_at	7.89962	7.972656	7.078621	7.216721	7.468559	7.148937	7.196758	7.649396	7.01521	6.973278	7.822591	7.879758	7.375473
PCYOX1	203803_at	9.135362	8.647636	9.996256	9.999847	9.284406	8.595403	8.08578	7.061738	7.776675	7.535684	7.304409	7.158988	7.755402
CROP	203804_s_at	10.39568	10.4377	9.509631	9.586004	9.830925	10.18357	10.67265	11.00336	10.72331	10.62198	10.85823	10.93846	10.98799
FANCA	203805_s_at	4.872321	4.854936	4.102302	3.332587	4.841247	4.81711	7.280383	6.699146	6.539257	6.757406	5.145224	5.424294	4.850528
FANCA	203806_s_at	2.541534	2.600626	2.314361	2.332179	2.390267	2.579685	3.877667	3.469382	3.41458	4.806595	3.885402	4.235603	2.566192
CSH2	203807_x_at	2.264274	2.247505	2.211829	2.227035	2.248645	2.228168	2.239791	2.246308	9.679297	5.73914	2.269095	2.239988	7.335077
AKT2	203809_s_at	2.365062	2.346527	2.31818	2.327296	2.431439	2.325698	2.343213	2.343499	2.32794	2.316234	2.329181	2.339252	2.315916
DNAJB4	203810_at	7.241841	6.833404	7.806283	7.855814	7.114704	6.136539	7.96679	7.822855	8.414437	8.202458	7.665431	7.689469	8.369613
DNAJB4	203811_s_at	3.302608	3.125599	4.288468	3.385995	3.371037	3.386409	5.072297	3.301397	5.079788	4.71002	3.240539	3.339027	7.113784
SLIT3	203813_s_at	3.078347	2.961656	3.167303	2.903514	2.960598	3.310522	2.95091	2.944159	2.930807	2.945401	3.435846	2.938576	7.49042
NQO2	203814_s_at	9.288229	9.50114	8.892813	9.05268	9.276033	9.256207	7.805072	8.039596	7.13032	7.923126	7.483244	8.243846	6.440928
GSTT1	203815_at	8.491241	8.636184	9.883703	10.07538	9.771599	9.679681	6.588596	6.62692	6.564663	6.395957	6.196515	2.251087	4.850189
DGUOK	203816_at	5.53144	5.536687	5.515661	5.453862	5.028749	3.864756	6.754864	5.854297	6.520089	6.54101	5.823156	5.528424	7.701897
GUCY1B3	203817_at	6.944708	6.940091	6.900915	6.992464	6.679101	6.572652	7.580919	6.786576	7.298752	7.161075	6.891765	6.898797	8.49291
SF3A3	203818_s_at	7.027799	6.929552	6.577891	6.857667	7.028827	6.991951	6.526013	7.976797	7.472988	7.733611	7.555773	7.887986	5.758683

IGF2BP3	203819_s_at	2.576756	2.556509	2.392181	2.499041	2.550616	2.412142	9.480962	8.554853	9.255826	9.404698	8.987705	9.06775	9.418665
IGF2BP3	203820_s_at	3.361466	3.17258	3.124553	3.129132	3.302057	3.128314	9.571532	9.022624	9.876942	9.704356	8.786771	8.794243	10.02346
HBEGF	203821_at	2.779514	2.977312	3.405985	3.379243	3.427366	3.813281	2.712334	2.395611	2.477179	2.314861	3.070585	2.977856	4.096535
ELF2	203822_s_at	7.230943	7.237742	7.24477	7.278371	7.240945	7.042846	7.297998	7.305741	7.332077	7.369319	7.087054	7.418377	7.660231
RGS3	203823_at	7.036103	7.196895	7.793991	7.754962	8.189126	8.071071	6.182917	6.795293	6.583162	6.576481	6.888049	6.467736	7.779784
TSPAN8	203824_at	4.07103	5.109175	9.343313	9.564081	8.767574	8.006722	6.180251	6.889191	7.251468	6.996421	5.906841	5.208464	5.965308
BRD3	203825_at	8.251482	7.99839	7.412982	7.517373	7.764555	7.911226	8.359331	8.767588	8.0983	8.082775	9.560187	8.790991	8.634966
PITPNM1	203826_s_at	4.74952	4.689247	4.665606	4.672814	4.548261	4.867137	4.681331	4.686017	4.645198	4.664596	4.668881	4.737562	4.530067
WIPI1	203827_at	6.052173	6.041658	7.051221	7.022591	6.195032	5.647498	8.178713	7.113455	8.451084	8.468512	7.074656	7.42193	7.504625
IL32	203828_s_at	10.00529	10.08103	8.234723	8.127759	8.510227	9.342444	4.848945	5.387604	3.523569	4.320684	5.843434	4.992672	6.314445
ELP4	203829_at	5.046121	4.736112	5.598478	5.683746	4.985842	4.146432	6.777249	5.472088	6.640622	7.242471	5.750148	5.532383	6.226208
C17orf75	203830_at	5.537178	5.075344	5.84003	5.540851	5.583702	4.655999	6.247819	6.216253	6.282561	6.115974	5.634463	6.38571	6.445187
R3HDM2	203831_at	8.01332	8.029367	8.16166	8.321513	7.935261	7.933632	6.884169	6.734616	6.799015	7.292882	7.175173	6.767386	7.595589
SNRPF	203832_at	7.785347	7.639491	8.176764	8.211006	8.00615	7.709758	9.385093	9.460263	9.649807	9.470256	9.392155	9.593842	9.225711
TGOLN2	203833_s_at	7.907885	7.502873	8.059891	8.372534	8.607809	8.903681	6.720855	7.338611	6.005388	5.840513	7.770231	8.101374	4.697344
TGOLN2	203834_s_at	5.641277	5.632207	6.485952	6.493222	5.60253	5.692282	5.46197	4.756272	5.136769	5.484363	4.981509	4.664909	4.471117
LRRC32	203835_at	4.889842	5.381326	4.828156	4.789108	5.729674	5.520438	4.851768	4.874358	4.876191	4.947131	4.865157	4.662334	4.777518
MAP3K5	203836_s_at	6.242583	6.23788	5.824897	5.98437	5.966904	5.694358	4.130725	3.53386	4.317025	3.953057	3.385734	4.15984	4.645195
MAP3K5	203837_at	9.262486	9.073749	8.460226	8.635065	8.965902	8.854656	6.788284	6.918778	6.97956	6.787555	6.523962	6.900077	7.433703
TNK2	203838_s_at	2.356537	2.334346	2.294475	2.313992	2.337491	2.312489	2.323491	2.333411	2.319531	2.310696	2.317151	2.328347	2.205545
TNK2	203839_s_at	4.481117	4.699885	4.249561	4.299382	4.261548	4.858234	4.383403	4.63246	3.937317	4.410639	5.546297	5.160693	3.962918
BLZF1	203840_at	6.215699	5.934396	5.88043	5.85523	5.929156	5.443312	5.909188	5.946002	6.043746	6.338231	5.886547	5.973102	6.317215
MAPRE3	203841_x_at	2.011067	1.994981	1.978273	1.975005	1.995196	1.975878	1.989899	1.991959	1.978917	1.974794	1.974993	1.9871	2.808987
MAPRE3	203842_s_at	3.73067	3.738066	3.577475	3.900194	3.773341	4.105723	3.528524	3.525458	3.504859	3.540576	3.510732	3.793709	3.458657
RPS6KA3	203843_at	6.59955	6.614108	6.589651	6.421804	6.005661	5.404884	8.113386	6.647688	8.149166	8.009839	6.63902	7.04331	6.741859
VHL	203844_at	3.877943	3.794478	3.687422	3.769364	3.892056	3.776709	3.801402	3.809696	3.797871	3.736544	3.823967	3.853028	3.444432
PCAF	203845_at	9.274712	9.25773	9.56697	9.463758	9.176847	8.802093	10.58677	10.53076	10.69205	10.68802	9.401618	9.839724	6.663583
TRIM32	203846_at	4.150467	4.217586	3.447967	3.313373	3.900128	3.749773	4.040347	3.895404	4.201913	4.326	4.181247	3.888069	6.794671
AKAP8	203847_s_at	6.187221	6.063965	6.032191	6.257543	6.164522	6.27945	6.514708	6.282551	6.588673	6.615523	6.628864	6.88825	6.268563
AKAP8	203848_at	7.331972	7.066233	7.399911	7.500155	7.548456	7.594765	7.021448	7.443536	7.431407	6.802298	7.715356	7.531099	7.011719
KIF1A	203849_s_at	2.585571	2.569676	2.547129	2.540866	2.646771	2.779278	2.540885	2.549019	2.534083	2.5201	2.549701	2.559012	3.405405
KIF1A	203850_s_at	3.069251	2.992155	2.969095	2.975911	3.065807	2.981171	2.986808	2.988435	2.978257	2.956092	2.982425	2.999259	2.67278
IGFBP6	203851_at	3.433152	3.733161	3.892982	3.722777	4.675871	4.953186	3.40953	3.425707	3.350897	3.299366	5.045005	3.796198	6.869226
SMN1	203852_s_at	7.400193	7.134399	7.374805	7.383518	5.736267	5.659292	8.199792	7.759477	7.711693	7.552944	6.864305	6.575857	8.242816
GAB2	203853_s_at	6.244038	6.062325	5.750354	5.53964	5.830745	5.750794	5.797089	5.798421	5.793454	5.559757	5.795312	5.803593	7.058396
CFI	203854_at	12.25048	12.30033	12.76021	12.57782	12.19076	11.7963	9.529732	9.596577	9.685725	9.775868	8.544392	8.324719	5.935543
WDR47	203855_at	5.233033	5.260305	5.391985	5.266701	5.358652	5.292153	5.312088	5.253817	5.394676	5.414731	5.807462	5.293065	8.804943
VRK1	203856_at	5.914441	5.875307	5.467993	5.725443	5.939466	6.246242	10.63148	10.60112	10.48316	10.61832	10.04964	10.41528	8.015691

PDIA5	203857_s_at	9.680565	9.782363	10.179	10.14653	9.710671	9.037887	8.684844	8.302976	8.63548	8.610172	8.039371	8.152302	7.057662
COX10	203858_s_at	5.101815	5.39392	5.516075	5.575212	4.749038	5.478466	5.620646	5.589786	5.685664	5.998927	5.634989	5.518648	5.859052
PALM	203859_s_at	3.902834	4.135453	3.726539	3.871536	3.885613	4.421275	3.498569	3.882154	3.867519	3.681404	4.16691	3.871464	5.536712
PCCA	203860_at	9.117307	9.004792	9.165885	9.42657	8.980307	8.456395	7.844836	7.518371	7.510327	7.781302	7.384685	7.342529	7.214488
ACTN2	203861_s_at	2.094102	2.074005	2.051208	2.055639	2.077444	2.059284	2.066414	2.07225	2.05883	2.053748	8.054762	2.068171	9.753448
ACTN2	203862_s_at	3.122571	2.984052	2.898711	3.008899	3.124734	2.949144	2.986282	2.935925	2.966211	2.955121	7.058782	3.134831	8.901299
ACTN2	203863_at	2.391262	2.358604	2.327187	2.342221	2.37468	2.348246	2.351623	2.362078	2.347268	2.336138	6.033433	2.371253	7.710445
ACTN2	203864_s_at	2.240824	2.215641	2.196099	2.196464	2.214095	2.289511	2.20868	2.21507	2.201272	2.191628	3.67435	2.210257	7.118735
ADARB1	203865_s_at	5.159051	5.049736	5.270521	5.033474	5.236209	5.309604	4.122418	4.099824	4.596811	4.041466	5.40232	4.382728	7.051298
NLE1	203866_at	2.182535	2.173109	2.155982	2.160569	2.173839	2.163826	2.167833	3.029297	2.166232	2.156975	2.648061	3.034609	2.125218
NLE1	203867_s_at	2.396564	2.388419	2.468344	2.47327	2.423465	2.474313	3.464781	3.993991	3.159647	3.273304	4.294011	4.46624	2.481523
VCAM1	203868_s_at	7.652555	7.888735	9.162907	9.421928	9.454453	9.272032	11.05565	10.79814	10.91037	10.76156	10.24202	10.19352	8.708271
USP46	203869_at	3.752436	3.391884	4.152584	3.683497	3.741744	3.640661	5.618578	5.010882	5.364715	5.51774	4.901401	5.193856	7.398053
USP46	203870_at	3.767561	3.703423	4.168266	4.212235	3.632407	3.256892	5.99853	4.453489	6.372676	6.352443	5.759744	4.151418	8.115099
SENP3	203871_at	6.123663	6.03841	5.756878	5.73863	5.548538	5.860876	6.587029	6.454102	6.535206	6.765073	6.814799	6.642828	7.602851
ACTA1	203872_at	2.561825	2.549815	2.488481	2.526549	2.655211	2.532176	2.54486	2.546447	2.53261	2.521887	9.431128	2.540491	11.67245
SMARCA1	203873_at	2.005051	1.969453	1.949412	1.950408	1.99094	1.956918	1.960033	1.967251	1.955892	1.940341	1.949635	1.972451	1.919461
SMARCA1	203874_s_at	8.927251	9.021366	9.571087	9.417268	8.549538	7.673278	10.57339	9.458981	10.80705	10.61626	9.432663	9.562095	10.41493
SMARCA1	203875_at	9.39686	9.178757	9.508091	9.503379	9.224491	8.97513	10.05549	9.646075	10.05308	9.938015	9.767748	9.838861	10.16607
MMP11	203876_s_at	2.78454	2.25833	2.208871	2.239022	2.245082	2.237529	2.250568	2.254473	2.241349	2.234147	2.208286	2.246484	2.340183
MMP11	203878_s_at	6.086291	5.362574	2.498999	2.730872	2.751106	2.715053	2.870895	2.928433	2.912981	2.731789	4.173632	3.082648	4.499684
PIK3CD	203879_at	5.227271	4.897689	4.513715	4.614564	6.571284	6.898019	4.12958	4.961599	4.280842	4.775957	4.668022	4.936214	3.321427
COX17	203880_at	9.416841	9.268705	9.861367	10.04642	9.639404	9.241947	9.790391	9.138159	9.918835	9.930835	9.18628	9.635099	7.999092
DMD	203881_s_at	10.1268	9.961697	10.45177	10.51262	9.722641	9.159824	8.799384	8.245886	9.100625	9.156635	8.409758	8.175312	8.77322
IRF9	203882_at	7.250864	7.21375	7.624937	7.26849	6.86561	6.82478	6.302117	5.586805	6.755152	6.289587	5.490521	5.896431	5.849903
RAB11FIP2	203883_s_at	6.606987	6.526451	6.974479	6.860225	6.648739	6.380286	6.82739	6.774311	7.053019	6.85164	7.018848	6.922999	8.066562
RAB11FIP2	203884_s_at	5.971642	6.000371	6.531928	6.233553	6.057286	5.971225	6.589606	6.374301	6.74647	6.69246	6.236867	6.168721	8.218262
RAB21	203885_at	7.71753	7.671086	8.219522	8.029379	7.629083	6.816906	7.78625	7.274764	7.877319	7.844493	6.896311	7.219494	7.756448
FBLN2	203886_s_at	2.965962	2.805996	2.723955	2.789342	2.786188	2.788388	2.800162	2.805185	2.791946	2.779531	4.616268	2.800239	7.038071
THBD	203887_s_at	3.829325	3.521588	6.101883	5.69527	4.035675	3.939818	2.280724	2.835509	2.303636	2.265805	3.103682	2.332996	5.569659
THBD	203888_at	3.914996	3.842267	5.456062	4.612218	4.115133	3.831846	3.761359	3.735604	3.79486	3.775357	3.830251	3.562982	5.613243
SCG5	203889_at	7.383996	7.267643	6.788694	7.00403	8.131938	7.903868	8.214127	8.073037	7.982275	8.28671	8.200347	7.900077	9.783066
DAPK3	203890_s_at	2.40419	2.353057	2.335984	2.285255	2.33948	2.335403	3.300325	2.352635	2.345469	3.397124	2.34189	2.342086	2.940088
DAPK3	203891_s_at	4.80723	5.068347	4.703092	4.67843	4.984807	5.779541	4.572122	4.790913	4.272076	4.679303	4.861619	5.147539	4.274831
WFDC2	203892_at	2.156278	2.146878	2.127745	2.129674	2.145267	2.130606	2.1419	2.143017	2.139375	2.133555	2.125343	2.137385	3.059145
TAF9	203893_at	8.084423	7.907046	8.271062	8.378407	7.643644	7.363391	8.724492	8.279032	8.822018	8.671846	8.012624	8.214938	8.162418
TUBG2	203894_at	4.648481	4.529793	3.83549	3.795109	3.888376	4.69162	3.862751	3.985534	3.74088	3.822417	3.895753	3.768125	4.866386
PLCB4	203895_at	2.450423	2.416537	2.3748	2.398531	2.492429	2.388464	2.407571	2.419252	2.401762	2.393049	4.086694	2.422558	7.361519

PLCB4	203896_s_at	2.675905	2.635945	2.568164	2.6088	2.685882	2.585584	2.623758	2.633518	2.620957	2.611135	3.438454	2.804852	5.435807
LYRM1	203897_at	10.44362	10.53756	10.5617	10.44446	10.25966	10.31269	10.09117	10.32126	10.12766	10.30417	9.874752	10.34098	8.607974
RCP9	203898_at	6.591574	6.716338	6.700871	6.720871	6.175417	6.326863	6.501552	6.269486	6.352122	5.99996	6.116459	6.307611	6.472049
RCP9	203899_s_at	5.199269	5.191968	5.174056	5.232229	5.190856	5.174473	5.141664	5.187973	5.149194	4.897244	5.203325	5.495541	5.111276
KIAA0467	203900_at	4.156799	3.977136	4.20998	4.199624	4.57225	4.146376	4.617938	4.239093	4.338307	4.550548	4.130478	4.100231	4.25035
MAP3K7IP1	203901_at	2.943604	2.9977	2.856866	2.883067	2.938371	2.724336	2.892675	2.897512	3.056251	2.933971	2.881694	2.887424	2.854786
HEPH	203902_at	2.642218	2.583368	2.540793	2.581921	2.724482	2.682706	2.590972	2.599475	2.588104	2.560572	2.590426	2.614862	2.545317
HEPH	203903_s_at	3.311766	3.542821	3.406105	3.454807	4.004804	4.029706	2.451758	2.96837	2.434778	2.486219	6.34679	3.208698	8.177851
CD82	203904_x_at	6.286874	6.107562	6.126331	6.119006	7.310519	7.329445	5.90059	6.204157	5.499817	5.733458	6.556559	6.203314	5.16118
PARN	203905_at	6.664687	6.47216	6.578757	6.453373	6.520391	6.315384	6.020067	6.356136	6.092475	5.918239	6.087297	6.008795	6.253367
IQSEC1	203906_at	8.306026	8.426412	7.754781	7.926643	7.971164	7.960021	7.873966	7.930584	7.740466	7.736275	7.962548	8.117791	6.583361
IQSEC1	203907_s_at	7.085306	6.891131	6.832177	6.702889	6.363047	6.28983	7.510498	6.960882	7.695733	7.308398	6.830067	6.795017	6.345657
SLC4A4	203908_at	8.483332	8.458848	9.493691	9.105517	7.259793	6.245842	4.588077	3.138267	3.726996	3.871788	2.871847	2.874983	6.992043
SLC9A6	203909_at	5.129492	5.194969	5.923592	5.837731	5.459748	5.31891	6.41506	5.842849	6.400146	6.224423	5.977268	6.154723	7.738025
ARHGAP29	203910_at	9.930085	9.871131	10.46458	10.49417	9.653827	9.192102	8.20591	7.743156	8.210572	8.382161	8.055841	7.647839	9.983625
RAP1GAP	203911_at	6.227535	6.291049	5.636616	5.328184	5.920133	5.815107	7.227129	6.492689	7.684647	7.284228	8.042896	8.766024	5.262964
DNASE1L1	203912_s_at	5.758301	5.409257	5.937655	6.210682	5.212117	5.319745	4.53737	4.469534	5.248435	4.662607	4.474977	5.011258	4.967716
HPGD	203913_s_at	6.890389	7.025286	9.422091	9.290274	8.379136	7.645569	4.352985	4.549877	4.365014	3.939072	3.226278	3.897861	4.937855
HPGD	203914_x_at	8.764435	9.070885	11.44564	11.11499	10.35901	9.674233	4.551686	4.346714	4.761984	3.865131	3.239356	4.148926	6.887264
CXCL9	203915_at	3.722955	3.612534	2.437262	2.367604	2.912428	5.890197	2.377433	2.380468	2.368845	2.361328	2.362128	2.372458	2.319039
NDST2	203916_at	6.888766	6.382272	6.621468	7.025881	6.830579	7.545319	6.751634	7.19067	7.145371	7.254145	7.154011	7.028288	6.62326
CXADR	203917_at	10.44917	10.41023	10.33124	10.23901	10.01753	9.192891	9.277526	8.965645	8.960564	9.350418	8.845448	8.937814	10.13289
PCDH1	203918_at	5.292927	5.635385	5.256591	5.409347	6.425277	6.15887	4.938089	5.149762	4.85927	4.736413	5.240316	5.123314	3.669713
TCEA2	203919_at	7.200876	7.529468	6.487116	6.916359	5.674908	5.604494	4.703058	4.751388	4.851404	4.226633	5.659353	4.867751	6.768686
NR1H3	203920_at	8.742393	8.68705	8.589422	8.819071	9.075352	8.969582	7.734072	7.955754	7.677474	7.766877	7.968927	8.236521	5.128678
CHST2	203921_at	4.430204	4.608368	4.35577	4.722098	4.247597	4.762447	9.357268	8.644147	9.4581	9.525472	7.823791	8.280669	7.015757
CYBB	203922_s_at	3.138681	2.469544	2.748291	3.078177	3.488291	3.294627	3.490017	4.083583	3.113794	2.999012	3.178974	3.713321	2.296974
CYBB	203923_s_at	5.762585	5.925075	6.916771	6.041575	6.265419	5.702915	7.548995	5.976126	7.503073	7.423997	5.843995	6.351901	5.255876
GSTA1	203924_at	13.32511	13.3344	13.55823	13.64526	13.30374	13.00347	12.81611	12.51309	12.78877	12.76931	11.69496	11.77916	8.539123
GCLM	203925_at	8.965697	8.885269	9.257931	9.115583	8.4401	7.977505	10.22549	9.163574	10.44615	10.29673	8.39397	9.039179	6.343244
ATP5D	203926_x_at	6.926196	6.895577	6.594595	7.058128	7.224617	7.459088	5.962556	7.092058	6.253996	6.369626	6.912354	7.144089	5.648964
NFKBIE	203927_at	4.802898	4.698305	4.008323	4.311555	5.409855	5.795845	4.685663	4.69792	4.593699	4.44431	4.681998	4.692075	3.587916
MAPT	203928_x_at	2.660856	2.641293	2.604125	2.609554	2.670171	3.222386	2.629656	2.615648	2.613556	2.620065	2.679534	2.622858	7.246604
MAPT	203929_s_at	2.509124	2.488536	2.457908	2.457757	2.488139	2.466305	2.472048	2.48833	2.469479	2.455804	2.472852	2.478535	4.938737
MAPT	203930_s_at	2.387367	2.358012	2.317291	2.339798	2.378351	2.333269	2.348223	2.358955	2.343664	2.332193	2.344414	2.378238	3.191623
MRPL12	203931_s_at	8.406175	8.565609	8.18314	8.490793	8.508535	8.31421	8.033218	8.468339	8.203385	8.180818	8.641003	8.637387	6.750446
HLA-DMB	203932_at	6.845693	6.895764	7.152534	6.957946	7.348457	7.977603	5.353968	5.478446	5.836122	5.770633	4.452315	5.014454	4.22997
RAB11FIP3	203933_at	5.778461	5.674904	5.467498	5.530714	5.60222	5.616215	5.563964	5.218913	5.293206	5.422696	5.441966	5.425077	5.761613

KDR	203934_at	7.635412	7.635547	8.635167	8.64951	6.81789	6.189147	8.801478	7.309485	8.635545	8.377916	7.749574	7.923662	7.914695
ACVR1	203935_at	7.728347	7.642628	8.112318	7.819476	6.927993	6.058325	7.700894	6.080967	7.201388	7.527852	6.61503	6.126984	9.125299
MMP9	203936_s_at	2.989377	3.598262	3.696414	3.447444	3.84021	5.989495	3.640987	3.821484	3.524693	3.732941	3.948797	3.913703	4.404822
TAF1C	203937_s_at	3.848402	3.566907	3.383062	3.385451	3.45667	3.815827	2.820043	3.383718	3.386474	3.293639	3.363636	2.915911	3.324913
TAF1C	203938_s_at	4.124695	4.120271	3.438596	3.724207	4.273075	4.203841	3.447789	4.036852	3.526769	3.342089	4.282374	3.758661	3.886347
NT5E	203939_at	8.55131	8.459863	9.380515	9.436542	8.5496	7.930231	9.397578	8.671459	9.153051	9.114455	8.553555	9.013486	6.518857
VASH1	203940_s_at	4.916461	5.019297	4.396485	4.121744	5.001395	5.480047	5.167827	5.34641	4.79177	4.944849	6.25118	5.677323	7.228981
INTS9	203941_at	4.734703	4.682419	4.207577	4.164911	4.226869	4.360863	5.308488	5.678581	5.49299	5.601235	5.941609	5.860769	5.886607
MARK2	203942_s_at	2.140166	2.128664	2.102748	2.109398	2.11244	2.102277	2.121218	2.12313	2.120557	2.114141	2.10493	2.11854	2.061139
KIF3B	203943_at	7.376132	7.412681	7.122024	7.027141	7.176183	7.298789	5.388277	6.190194	5.625924	5.540472	6.313754	5.9249	5.759913
BTN2A1	203944_x_at	6.317702	6.175567	6.151187	6.181044	6.388788	6.417564	6.152646	5.837511	6.161683	6.163824	6.460185	6.467607	7.599747
ARG2	203945_at	4.151882	3.797161	4.116995	4.421386	4.435223	4.806293	3.728932	5.050214	3.590334	3.710375	4.210151	4.31736	3.387474
ARG2	203946_s_at	3.090723	2.862724	4.677302	5.416728	3.302478	2.974372	3.17453	3.428287	3.468783	3.644605	3.708748	4.757622	5.699794
CSTF3	203947_at	7.412817	7.26545	7.933218	7.998462	7.351399	7.287374	7.295213	7.280193	7.247809	7.456755	7.350449	7.10262	7.92345
MPO	203948_s_at	2.129536	2.116708	2.08447	2.100263	2.112694	2.091131	3.846258	3.788808	3.42485	5.374592	3.866344	4.675024	2.09664
MPO	203949_at	2.452248	2.430228	2.373013	2.387312	2.438062	2.404157	6.126627	6.606508	5.178917	7.19007	6.708066	7.141905	3.126293
CLCN6	203950_s_at	4.270348	4.251871	4.176101	4.253794	4.473323	4.538433	4.573024	4.418395	4.7141	4.252729	4.302962	4.615168	4.223048
CNN1	203951_at	2.495214	2.488422	2.468964	2.464317	2.48168	2.889542	2.469506	2.478989	2.46598	2.459476	3.253855	2.476616	4.74235
ATF6	203952_at	7.103508	6.986386	7.134252	7.427976	6.838461	6.772244	6.375933	6.32372	6.804838	6.903001	6.553071	6.815977	5.922595
CLDN3	203953_s_at	4.043403	4.170894	5.199777	4.850433	2.856043	2.793193	2.644949	2.174245	2.803108	2.779705	2.279758	2.799145	2.736019
CLDN3	203954_x_at	6.869649	6.949522	6.664556	7.279043	7.056541	6.690737	4.197289	5.891092	5.366134	4.64847	4.666026	6.187889	4.404473
KIAA0649	203955_at	5.333751	5.819811	5.326418	5.252473	5.371303	4.923442	3.826284	4.008898	3.847521	4.169066	4.09935	4.317799	4.416984
MORC2	203956_at	8.796167	8.568911	8.259713	8.393119	8.658365	8.860446	8.864536	9.376736	8.873989	9.062	9.649106	9.487109	8.437347
E2F6	203957_at	5.834096	5.650548	5.779658	5.453827	5.618576	5.215134	5.798217	5.939401	5.948943	5.87346	6.349402	6.032058	7.079042
ZBTB40	203958_s_at	2.911257	2.743022	3.039452	3.040931	3.101776	3.57418	3.857411	3.97561	3.650564	3.350885	4.319522	4.118828	2.739905
ZBTB40	203959_s_at	6.313525	6.160044	5.966445	6.497366	6.229557	6.293334	6.423205	6.158949	6.346726	6.136243	6.316963	6.496744	5.843183
C1orf41	203960_s_at	6.725138	6.775242	6.810771	6.859947	6.944736	6.306405	7.356802	6.918272	7.07679	7.325629	6.77174	7.10909	7.167799
NEBL	203961_at	2.430695	2.386912	2.382265	2.758555	2.419883	2.336272	2.375644	2.381964	2.370675	2.36411	2.368564	2.384439	7.869541
NEBL	203962_s_at	2.602979	2.558572	2.547028	2.554332	2.591079	2.515954	2.558844	2.518177	2.547494	2.554756	2.553492	2.566922	8.028405
CA12	203963_at	2.776031	3.634888	2.236847	2.427247	2.029063	2.485826	7.072219	6.649153	5.275585	6.359275	7.303841	7.360365	5.582896
NMI	203964_at	8.714006	8.627582	8.646252	8.718479	8.559823	8.424452	8.559442	8.132647	8.573561	8.702237	7.77275	8.595317	6.59414
USP20	203965_at	4.241349	4.289891	3.593118	4.091037	4.542655	5.125405	5.074157	5.831723	5.799085	5.5856	5.237287	5.521696	3.548712
PPM1A	203966_s_at	9.825274	9.607708	9.760785	9.828101	9.23356	8.45209	9.927117	9.458584	9.759496	9.843091	8.684491	9.147374	8.190888
CDC6	203967_at	2.286696	2.265161	2.245485	2.246031	2.269035	2.254506	7.261567	6.946875	7.609626	7.370851	6.282837	6.972006	4.756083
CDC6	203968_s_at	2.316448	2.309121	2.272776	2.277422	2.297987	2.27832	6.357849	5.249886	6.576099	6.743104	5.443104	5.382291	4.333093
LOC153914	203969_at	4.257393	3.856375	4.265412	4.378833	4.14906	3.79969	3.740658	3.845576	3.835047	3.750114	3.775156	3.973571	3.777508
PEX3	203970_s_at	7.368742	7.227886	7.612058	7.745196	6.963599	6.64284	7.010104	6.511466	7.097436	6.977171	6.354592	6.452323	6.952549
SLC31A1	203971_at	8.402319	8.246202	8.657363	8.685798	8.567907	8.408757	7.195585	7.425717	7.241163	7.208432	7.125751	7.405348	5.919152

PEX3	203972_s_at	6.307228	6.470476	7.388249	7.402819	5.608026	5.220453	7.192754	5.635332	6.898362	6.68649	5.38695	5.685756	6.71508
CEBPD	203973_s_at	12.18692	12.18732	12.06286	12.11887	12.07356	11.9092	7.156764	7.512195	7.216869	6.915418	7.707196	7.182079	7.99172
HDHD1A	203974_at	5.950153	5.630712	5.480338	5.386988	6.786464	6.269623	6.694658	6.660315	6.875554	6.580994	7.40111	6.440243	6.974926
CHAF1A	203975_s_at	4.141242	4.297305	3.777378	3.39875	4.222007	4.311545	6.034847	6.307728	5.583439	6.03327	6.620922	6.85147	4.762142
CHAF1A	203976_s_at	4.15292	3.821796	3.227939	3.581232	3.595291	3.563113	6.588037	6.740576	7.211324	7.253629	5.700156	6.543033	5.106018
TAZ	203977_at	4.163461	4.146506	4.115644	4.130335	4.14293	4.987422	4.07017	4.140408	4.124575	4.132644	4.318468	4.13351	3.767616
NUBP1	203978_at	6.372123	6.313215	6.831217	6.735991	6.660128	6.293295	6.748775	7.017989	7.237124	7.24952	6.413029	7.219955	6.858225
CYP27A1	203979_at	11.72774	11.61943	11.4722	11.54391	12.01889	11.91491	8.665403	9.437286	8.850805	8.678374	8.881377	9.099319	5.112066
FABP4	203980_at	2.359696	3.596561	4.243135	3.775601	5.885002	4.799741	2.480136	2.225939	2.240808	2.192789	3.773283	2.489371	5.617387
ABCD4	203981_s_at	8.029258	8.129487	7.807448	8.07428	8.193806	7.891917	7.764386	7.315202	8.094052	7.806287	7.866569	7.803145	7.183672
ABCD4	203982_s_at	2.389965	2.357451	2.467292	2.344233	2.361917	2.325654	2.356144	2.359032	2.344241	2.34398	2.342196	2.354149	2.219161
TSNAX	203983_at	9.838619	9.751576	9.467545	9.589416	9.698217	9.690496	10.0326	10.43635	9.878447	10.17822	10.05079	10.22144	9.177495
CASP9	203984_s_at	6.345356	6.358354	6.17737	6.629567	6.359322	6.607962	6.326755	6.417265	6.312916	6.229499	6.252509	6.750846	6.207597
ZNF212	203985_at	6.857491	6.902918	6.599812	6.920712	6.94015	7.026783	6.955291	6.938983	6.825322	6.716466	6.933516	7.036397	6.821925
STBD1	203986_at	9.717321	9.650627	8.590675	8.870729	8.492468	8.076336	5.864909	5.839873	5.8724	6.536576	5.177509	5.519869	4.23162
FZD6	203987_at	2.65142	2.639847	3.79269	2.812361	2.458596	2.409606	3.607644	2.429003	4.127642	2.735768	2.514477	2.453091	5.990358
FUT8	203988_s_at	3.478923	3.892912	3.805136	3.716344	4.189228	3.812161	6.15454	5.179025	6.041197	5.485761	5.09381	5.363834	7.590976
F2R	203989_x_at	7.24996	7.07405	8.573666	8.075416	7.283626	6.722246	7.736211	7.086183	7.555047	7.468211	7.554094	7.417526	8.157405
UTX	203990_s_at	3.010841	3.022232	2.984927	3.059599	4.14788	3.013519	3.02909	3.018436	3.172449	2.986448	3.313706	3.362223	3.262429
UTX	203991_s_at	7.510653	7.362247	7.029244	7.183063	7.897341	7.87662	7.399311	7.89518	7.436675	7.372841	8.25841	7.809825	6.856057
UTX	203992_s_at	5.47633	5.465806	5.915575	5.737216	5.560321	4.339067	6.824732	5.516129	6.97972	7.284115	5.92719	5.404172	6.420613
C21orf2	203993_x_at	1.765557	1.754087	1.735076	1.739705	1.753123	1.740797	1.750451	1.751075	1.7418	1.735964	1.732801	1.745217	1.738768
C21orf2	203994_s_at	6.269245	6.22566	6.138603	6.140605	6.219379	6.290062	5.760305	6.918401	5.749359	5.844607	6.705147	6.13162	5.44758
C21orf2	203995_at	3.324775	3.079012	3.853059	4.176499	3.897647	3.616759	3.203599	3.303796	3.201542	3.629445	3.290213	3.297854	2.651121
C21orf2	203996_s_at	5.489711	5.546751	5.352266	5.650518	6.664368	6.675709	4.935868	5.859207	5.133452	5.58277	5.760029	5.915948	4.739112
PTPN3	203997_at	8.599001	8.312105	8.322986	7.966203	7.572581	6.851917	4.48379	3.451002	5.028566	4.849374	3.51806	4.130559	3.70332
SYT1	203998_s_at	2.925896	2.88105	3.132992	2.8667	2.91157	2.761944	2.877585	2.864051	2.87151	2.856883	2.872764	2.89403	5.871931
SYT1	203999_at	1.906972	1.897135	1.880999	1.909316	1.898515	1.88524	2.686738	1.896659	1.888678	1.883093	1.87922	1.897139	7.027121
GNB5	204000_at	6.718503	6.81219	7.039863	7.392582	7.389215	7.580992	5.746782	6.49241	5.853822	6.027916	6.03759	6.192243	5.908607
SNAPC3	204001_at	5.543267	5.527307	5.519642	5.535375	5.52742	5.482876	5.970984	6.200632	5.946142	6.215309	5.98565	6.104543	5.891399
ICA1	204002_s_at	2.287602	2.272321	2.250091	2.253341	2.265937	2.246676	2.269497	2.286221	2.257405	2.257249	2.259003	2.277295	2.230002
NUPL2	204003_s_at	4.762998	4.714662	4.791094	4.806119	4.216022	4.111625	6.107936	5.263463	5.888405	5.716466	4.938335	4.814544	5.91234
PAWR	204004_at	7.911198	7.853905	8.474258	7.860465	6.490336	6.027898	6.559733	4.450666	6.085828	6.593284	4.725242	4.939115	5.647628
PAWR	204005_s_at	7.314475	7.3327	7.013026	7.133743	6.814815	6.334966	5.336529	4.32701	5.574771	5.185975	4.449249	4.53422	5.869155
FCGR3A	204006_s_at	2.372474	2.34608	2.439018	2.3535	2.369577	2.341855	2.364481	2.365648	3.70506	2.561352	2.327296	2.3486	2.214632
FCGR3B	204007_at	3.899707	3.591308	5.839395	5.256793	4.740271	4.605046	4.267717	4.314292	5.270928	3.947441	3.066146	3.866572	2.116879
DNAL4	204008_at	3.555956	3.545046	3.711351	3.578617	3.562886	3.530973	3.535539	3.901705	3.545622	3.529481	3.548228	3.970096	4.495405
KRAS	204009_s_at	8.139869	7.979041	8.487387	8.412091	8.123055	7.21212	9.354415	8.826668	9.28513	9.52168	8.818336	8.746269	9.959945

KRAS	204010_s_at	6.604655	6.622714	5.94241	6.469498	6.605662	6.090375	4.581805	5.552279	5.358973	5.046034	5.381924	5.724746	3.740414
SPRY2	204011_at	7.326305	7.090846	8.105152	7.530813	6.853528	6.10805	7.184618	5.990976	6.996969	7.152072	7.303645	6.112774	9.736238
LCMT2	204012_s_at	5.886885	5.825219	6.122117	6.099028	6.034423	6.159605	7.583629	6.841001	7.636728	7.419821	6.783972	6.65607	6.772946
LCMT2	204013_s_at	3.159	3.176083	3.100027	3.114277	3.90372	3.567099	3.71576	3.132304	3.133149	3.108334	3.117887	3.126539	3.11013
DUSP4	204014_at	2.8168	2.776098	2.727196	2.748415	2.814494	2.752517	2.76134	2.786327	2.78624	3.976816	2.769879	2.779322	5.236518
DUSP4	204015_s_at	3.2468	3.246465	3.218905	3.035254	3.239824	3.170045	3.235738	3.244661	4.5719	5.502401	3.230236	3.116543	4.666405
LARS2	204016_at	5.751927	5.760132	5.898117	6.019955	5.946106	5.87632	5.890247	6.29747	5.882268	6.538533	6.011142	5.884286	5.464074
KDELR3	204017_at	4.859798	5.464665	5.557454	4.752523	5.688568	5.685111	9.392213	8.424483	9.28342	9.46567	8.213121	8.269513	9.289354
HBA1	204018_x_at	9.603326	10.69705	8.922085	8.108303	12.06607	10.79809	15.62717	15.32696	15.59522	15.58703	15.44764	15.48644	14.87077
SH3YL1	204019_s_at	8.555824	8.686223	7.529564	7.618924	7.362022	7.017105	9.660196	9.356544	9.464718	10.08326	9.05938	8.572644	9.379059
PURA	204020_at	8.905013	8.698536	8.871169	8.717766	8.281707	7.898466	7.971566	7.251778	8.034788	7.882235	7.370754	7.521684	7.75617
PURA	204021_s_at	6.447693	6.447563	6.079354	6.294159	6.792803	6.578858	4.886868	5.501233	4.894287	5.181557	5.60589	5.396489	3.962089
WWP2	204022_at	6.879951	6.981133	6.036059	6.217801	7.141249	7.49394	6.70627	7.17087	6.611841	6.872328	7.315049	7.46803	8.184337
RFC4	204023_at	5.807182	6.251451	5.33757	5.425809	5.356885	5.922105	9.700231	9.313066	9.718268	9.814544	8.691897	9.135564	8.376298
OSGIN2	204024_at	4.291816	3.955741	4.245347	3.937336	2.978206	2.837791	2.943591	2.670801	2.64242	2.633512	3.026575	2.806226	3.088788
PDCD2	204025_s_at	6.224546	6.050325	6.518381	6.913065	6.501566	6.311661	6.700346	6.16262	6.737577	6.762321	6.204273	6.100718	6.591085
ZWINT	204026_s_at	6.911368	6.55916	3.429281	3.096766	3.429782	4.81884	11.31711	10.94147	11.34773	11.30846	10.44246	10.75857	9.924285
METTL1	204027_s_at	4.340554	4.322522	4.562454	4.623232	4.443485	4.740363	4.715912	5.157958	4.893044	4.756927	5.714991	5.938068	4.586444
RABGAP1	204028_s_at	8.169008	8.325022	7.880867	8.028954	8.13156	8.024922	7.948163	8.022241	7.771932	7.832426	8.579592	8.508525	8.7849
CELSR2	204029_at	2.040186	2.029829	1.993977	2.006164	2.029533	2.056224	2.089991	2.027865	2.012842	2.01447	2.012348	2.162198	4.899824
SCHIP1	204030_s_at	4.2092	4.227445	5.543508	5.431689	5.627304	5.498018	5.345437	5.407186	5.2087	5.154555	6.053589	4.793251	8.874483
PCBP2	204031_s_at	10.6296	10.53653	11.20781	11.03104	10.12092	9.510308	11.58132	10.47355	11.91131	11.68446	10.33167	10.5862	11.49808
BCAR3	204032_at	5.275238	5.424508	5.327701	5.504729	5.252611	5.359152	4.503907	3.941376	3.922329	4.389373	4.049737	3.995332	5.130039
TRIP13	204033_at	2.304612	2.287609	2.246075	2.291122	2.286868	2.254365	8.048623	7.506848	8.273561	8.331824	7.558792	7.442685	6.730674
ETHE1	204034_at	6.558273	6.626528	6.966753	6.965806	7.127308	6.964195	7.020742	6.558224	7.048514	6.271935	6.650931	6.929587	4.832112
SCG2	204035_at	2.89685	2.86654	2.840924	2.85001	2.894618	2.847721	2.84996	2.86873	2.853273	2.797588	2.854293	2.864509	7.374125
EDG2	204036_at	3.983675	4.126548	3.739791	3.963445	3.828544	4.196218	4.310255	3.862705	3.80225	3.940472	6.890183	5.0498	9.230033
EDG2	204037_at	5.381006	5.734456	4.806518	5.043372	5.257664	5.485483	5.105756	5.229654	4.857539	5.175445	7.312472	5.608785	9.110927
EDG2	204038_s_at	4.342292	4.330413	4.307166	4.308168	4.331495	4.316896	4.323331	4.326929	4.311245	4.307638	4.311029	4.439985	6.44844
CEBPA	204039_at	10.9553	10.69824	9.841829	9.706547	10.19824	9.786344	9.721176	9.153205	10.06542	9.906064	9.068894	9.515682	4.556304
RNF144A	204040_at	2.561638	2.253923	2.936454	2.954515	2.928249	2.789812	5.089404	4.778022	4.659593	5.534203	5.18074	5.103431	6.604838
MAOB	204041_at	11.11872	11.13521	11.55473	11.42644	11.16444	10.80617	10.01928	9.735911	10.00063	9.949765	9.395702	9.456981	6.638612
WASF3	204042_at	3.124255	3.281955	4.09518	4.127018	4.100877	4.157882	5.665664	3.929979	4.857417	4.391355	4.739562	3.726436	6.80151
TCN2	204043_at	4.705054	4.634278	4.691648	4.709136	4.716918	5.372111	4.691227	4.72077	4.70682	4.702381	4.738031	4.993216	4.621618
QPRT	204044_at	9.806026	9.681341	10.00095	10.17071	10.02173	9.966358	8.773223	8.792614	9.028866	8.99379	8.963565	9.215875	7.057371
TCEAL1	204045_at	6.182807	5.937052	7.040045	6.764176	5.555511	5.579522	5.846723	4.942125	6.007576	5.913525	5.956577	4.908345	8.099316
PLCB2	204046_at	4.044082	4.034299	3.90624	4.017852	4.50836	4.347009	4.030572	4.026975	3.929618	4.010483	3.977185	3.934401	3.447796
PHACTR2	204047_s_at	3.205787	3.186867	3.167258	3.179346	3.18892	3.401191	2.976417	3.185061	2.909327	2.904471	3.172163	3.174646	4.205068

PHACTR2	204048_s_at	6.820473	6.946238	7.376094	7.238124	6.840478	6.324827	6.928338	5.830855	6.684384	6.087232	6.626121	6.126407	8.647384
PHACTR2	204049_s_at	7.168217	7.09978	7.858421	7.25436	6.432694	5.742882	7.625917	6.70221	7.551313	7.217297	7.047826	6.567285	10.33908
CLTA	204050_s_at	9.797321	9.780979	9.799204	9.81473	9.652927	9.471625	10.23679	9.602513	10.30209	10.22689	9.864315	9.983545	10.80569
SFRP4	204051_s_at	1.99553	1.984132	1.961436	1.968629	1.989909	1.969331	1.979787	1.981407	1.971393	1.965391	2.193159	1.97854	5.714973
SFRP4	204052_s_at	2.422365	2.38743	2.361277	2.375725	2.403032	2.363932	2.377934	2.388994	2.376043	2.364792	2.377389	2.387333	3.650539
PTEN	204053_x_at	8.850214	8.689549	9.217373	9.436044	8.466421	8.221538	8.192681	8.206656	8.301843	8.111357	7.881015	8.206752	8.493712
PTEN	204054_at	4.810834	4.381392	4.980537	4.887342	4.364455	4.107534	4.375654	3.867948	4.576213	4.360531	4.142128	4.109393	4.372566
CTAGE5	204055_s_at	6.487523	6.372988	6.667684	6.339637	5.880936	4.938532	5.34983	4.870925	5.606636	5.584622	4.453569	5.055265	3.965072
MVK	204056_s_at	4.793491	4.598096	4.012927	3.998769	4.282428	3.972072	4.494849	4.674856	4.728047	4.775892	4.393103	5.273703	3.868918
IRF8	204057_at	8.270589	8.368223	8.548342	8.358234	7.591023	7.552677	7.406172	7.109708	7.424052	7.26712	6.722888	6.978825	4.747538
ME1	204058_at	7.305854	7.004008	6.355181	6.39465	5.964849	6.145272	6.33707	6.324518	6.341296	6.349059	5.756091	6.131407	5.430376
ME1	204059_s_at	8.200524	8.043956	7.913563	7.417477	6.378608	6.17511	8.192478	7.407031	7.828922	7.884869	6.747259	6.881901	6.6326
PRKX	204060_s_at	5.342886	5.1474	4.630841	4.351391	4.473683	4.219311	4.549301	4.688313	4.679675	4.675592	4.786751	4.681772	5.817923
PRKX	204061_at	2.716784	2.713018	2.576311	2.68721	2.703296	2.686349	2.745341	2.700153	2.746766	2.770967	4.039463	2.702238	4.704811
ULK2	204062_s_at	5.226082	4.415168	4.393323	4.378938	4.414995	4.374466	4.49777	4.33157	5.006265	5.074862	4.415082	4.403968	7.693273
ULK2	204063_s_at	3.63128	3.619555	3.571258	3.074047	3.592058	3.601633	3.751095	3.620738	3.835048	3.703852	4.771771	4.006737	6.268203
THOC1	204064_at	7.958922	8.110665	7.932094	7.908454	8.213095	8.355556	8.692844	8.919503	8.958385	8.755609	8.655655	8.940197	7.95126
CHST10	204065_at	3.218071	2.90703	2.613221	2.866523	2.631454	2.970499	5.35548	5.60935	5.430326	5.289072	5.544049	4.97411	5.886363
CENTG2	204066_s_at	5.510455	5.084016	5.461565	5.098775	4.938658	5.058971	6.446897	4.409744	6.005283	5.094866	5.636807	5.549482	7.692416
SUOX	204067_at	8.017786	8.042928	8.3964	8.411612	8.412114	8.317943	7.519359	7.400612	7.131771	6.984876	6.944101	7.643615	5.329669
STK3	204068_at	8.140862	8.295368	8.491375	8.989666	9.005743	9.021086	7.471819	7.850506	7.807236	7.70455	8.284119	8.053021	8.285506
MEIS1	204069_at	4.932755	5.043169	5.630799	5.543121	4.915937	4.759161	6.841536	5.874053	6.684195	6.381237	7.637955	6.761595	9.829597
RARRES3	204070_at	8.436765	8.534937	8.314079	8.368411	8.885748	9.320838	3.074096	4.258359	3.864214	3.354236	3.046282	3.587414	3.006018
TOPORS	204071_s_at	8.039834	7.969255	8.497822	8.195719	7.509139	6.982232	8.867213	8.088174	8.977557	9.045073	8.009494	8.073503	8.706906
FRY	204072_s_at	7.920454	7.817467	7.754278	7.73112	7.488332	7.0104	5.162393	5.294811	6.007404	6.045416	5.880288	5.140396	8.443442
C11orf9	204073_s_at	6.373732	6.210151	3.933182	4.106712	5.364397	5.486387	4.335991	4.953186	4.657512	4.42051	5.938658	4.667302	4.748988
KIAA0562	204074_s_at	5.614117	5.671296	6.166962	6.051215	5.944385	6.014038	5.769662	5.66102	5.083114	5.184797	5.733492	5.448794	5.231313
KIAA0562	204075_s_at	6.560869	6.333276	6.873004	6.765217	6.333593	6.31472	6.226788	5.285076	6.233059	5.887824	5.615287	5.158829	5.775239
ENTPD4	204076_at	7.70381	7.883715	7.615024	7.770063	8.000287	7.779984	8.262427	8.428465	8.361937	8.42238	8.960095	8.444731	9.690125
ENTPD4	204077_x_at	4.101757	4.758524	4.10922	4.02921	4.569091	4.819542	4.053347	3.951066	3.935712	3.831228	3.929624	3.98665	4.261932
SC65	204078_at	4.605634	4.561622	4.403446	4.627332	4.955984	5.523994	5.16101	7.322135	5.351837	5.96904	8.019544	7.060977	7.910464
TPST2	204079_at	9.975382	10.03656	9.280713	9.501926	9.149795	8.917066	7.547476	7.984012	7.951862	7.816466	7.683323	8.308064	5.844934
TOE1	204080_at	5.95944	5.961718	5.764218	6.081309	6.148864	6.314342	6.503544	6.66691	6.464775	6.605362	7.454154	7.010739	5.67678
NRGN	204081_at	3.909188	3.898896	3.245024	3.277584	4.08235	4.82532	6.067993	6.623575	5.770028	5.487969	6.666717	7.57423	3.235517
PBX3	204082_at	6.163628	5.709341	6.381113	6.170201	4.723833	4.524238	4.966897	4.531979	5.820536	5.185039	4.370459	4.443334	8.439025
TPM2	204083_s_at	5.667048	5.910942	5.623018	5.540687	6.618165	7.335961	5.571066	6.080183	5.268722	5.010482	10.31896	6.865572	11.13745
CLN5	204084_s_at	8.311095	8.113442	8.613406	8.87669	9.261069	9.110139	7.436501	7.638035	7.161175	7.09073	7.405573	8.163159	6.540629
CLN5	204085_s_at	6.205852	5.765685	6.691	6.626668	6.663763	6.642186	5.91464	5.466282	6.237231	6.148132	5.450682	5.962522	6.179951

PRAME	204086_at	2.177227	2.161647	2.142241	2.151754	2.160555	2.152017	2.158008	2.164802	2.155113	2.150093	2.150567	2.155717	2.119241
SLC5A6	204087_s_at	7.030086	7.005509	6.96219	7.099062	7.128362	7.237172	6.954641	6.624393	6.330349	6.242493	6.679161	6.64321	6.511138
P2RX4	204088_at	5.950167	5.765728	6.687278	6.156931	5.558092	5.159246	7.211371	6.183895	6.906242	7.27325	6.701456	6.968486	5.916689
MAP3K4	204089_x_at	7.962146	8.014639	7.617814	7.609998	7.959428	7.71163	8.61195	8.73815	8.580484	8.500165	8.664284	8.941778	8.297901
STK19	204090_at	6.415445	6.211856	5.786589	6.061589	6.186024	6.166378	6.178502	6.169983	6.169949	6.145043	6.649283	6.234757	6.108668
PDE6D	204091_at	5.61871	5.578284	6.661283	6.425571	5.989752	5.432923	6.337406	5.727766	6.512407	6.672675	5.833649	5.431109	7.658346
AURKA	204092_s_at	3.56876	3.447483	3.287725	3.430316	2.02469	2.806646	8.896849	8.67122	8.909842	8.915384	8.292066	8.515989	5.929109
CCNH	204093_at	6.198903	5.91831	6.979114	7.015833	6.390628	5.81791	6.518496	6.536338	6.811524	6.790834	6.238684	6.458906	5.86269
TSC22D2	204094_s_at	8.511713	8.259555	10.02563	9.917077	8.438252	8.17284	9.793863	8.705598	9.891274	10.0887	9.430285	9.548696	10.52973
ELL	204095_s_at	2.357739	2.354341	2.371603	2.419381	2.543199	2.411248	2.348524	2.339984	2.343746	2.295516	2.512134	2.28507	2.297125
ELL	204096_s_at	2.887668	2.935228	2.92218	3.093094	2.967635	2.916726	2.93176	2.706058	2.934686	2.923299	2.909922	2.931323	2.686967
RBMX2	204097_s_at	4.36603	4.420396	4.01273	4.270675	4.600173	4.373721	4.093233	4.641824	4.352213	4.290139	4.847314	4.970289	4.41369
RBMX2	204098_at	4.734617	4.797184	4.541977	4.440585	4.669419	5.034229	4.419729	5.205021	4.483753	4.492328	5.038522	5.093051	4.296729
SMARCD3	204099_at	2.394682	2.481473	2.364023	2.368067	2.381132	3.278179	2.378618	2.379123	2.364834	2.364597	5.630449	2.383763	7.37043
THRA	204100_at	2.094368	2.073605	2.056711	2.060615	2.084996	2.022924	2.073759	2.076321	2.150793	2.058929	2.065932	2.080902	3.78191
MTM1	204101_at	3.90151	3.671868	4.765614	3.601149	3.312566	3.455098	3.522412	3.253308	3.514038	3.251201	2.830563	3.456721	2.947836
EEF2	204102_s_at	12.47725	12.37068	12.43308	12.23305	12.2241	11.80069	12.96017	12.38785	12.9695	13.10258	12.51745	12.45793	13.25764
CCL4	204103_at	3.761667	3.746295	6.647757	5.998937	6.256663	5.632986	3.65738	3.673728	3.65348	2.597983	3.69463	3.66261	2.598509
SNAPC2	204104_at	2.612332	2.604254	2.554077	2.589856	2.606161	2.583915	2.592533	2.640011	2.588236	2.582728	2.590027	2.598284	2.498556
NRCAM	204105_s_at	3.531799	3.351261	3.303179	3.310498	3.287214	3.130244	3.442702	3.935092	3.886972	3.776839	5.286936	3.47345	8.335662
TESK1	204106_at	6.138931	6.195539	6.125687	6.235658	6.191041	6.283929	6.189947	6.2081	6.169148	6.077661	6.280857	6.418086	6.450107
NFYA	204107_at	2.451069	2.442415	2.423369	2.428752	2.438245	2.392715	3.209311	2.439295	3.539425	3.539924	2.412612	2.432758	3.680411
NFYA	204108_at	5.10748	4.946054	5.334727	5.342256	5.286505	4.593174	7.259305	6.59732	7.388495	7.32185	6.734651	6.874428	7.591899
NFYA	204109_s_at	2.811102	2.786866	2.783372	2.818069	2.79552	2.781019	2.991212	2.816403	3.936556	3.882515	2.87554	2.795573	3.815121
HNMT	204110_at	2.398095	2.37087	2.426945	2.353383	2.378497	2.354503	2.360954	2.37575	2.361759	2.361007	2.359092	2.370285	2.227103
HNMT	204111_at	4.015777	3.395843	5.228561	3.83215	3.194543	3.315886	3.450548	3.415482	3.450425	3.391683	3.242714	3.430943	3.095153
HNMT	204112_s_at	11.80957	11.81278	11.51027	11.29967	11.04014	10.72607	10.81023	9.927796	10.82517	10.59467	9.53554	10.13697	8.563729
CUGBP1	204113_at	3.495826	3.486102	3.615356	3.514287	3.559723	3.357262	3.753261	3.48436	3.943417	3.811358	3.299797	3.406152	3.849744
NID2	204114_at	6.753313	6.80704	6.67498	6.362124	6.274596	6.295629	9.449276	7.817568	8.450669	8.325429	8.962923	8.257573	11.55862
GNG11	204115_at	8.051534	8.088457	8.26007	8.322287	8.082654	8.220502	8.077653	7.745189	7.918321	7.812841	8.682979	8.229348	9.454932
IL2RG	204116_at	3.495062	3.988886	3.584032	3.414994	3.625017	3.589482	3.33939	3.375436	3.229481	3.229839	3.368161	3.273874	3.123119
PREP	204117_at	4.988891	4.865884	5.508421	5.228163	4.510701	3.414103	5.90581	4.660819	5.953613	5.638559	4.656096	4.666333	6.636494
CD48	204118_at	3.424979	3.265615	4.01723	3.190335	3.555655	4.370162	2.946566	2.939227	2.607321	2.926924	2.924053	3.196199	2.079343
ADK	204119_s_at	8.679557	8.650258	10.06379	9.682742	8.218583	7.439214	7.780148	7.174993	7.647448	7.52681	7.055548	7.435631	7.243582
ADK	204120_s_at	7.564931	7.470455	8.194926	8.1202	6.40726	6.54416	5.844661	6.065368	5.809984	5.63179	5.528608	5.987846	5.146034
GADD45G	204121_at	8.433837	8.409604	8.00706	8.364399	9.115832	9.455946	3.153737	4.39665	3.704206	3.778145	3.927701	4.262383	3.590507
TYROBP	204122_at	6.461423	6.206785	7.820132	7.792243	7.671395	7.606989	6.479363	5.980024	6.175079	6.338284	5.619117	6.376086	5.17653
LIG3	204123_at	4.071464	3.993722	4.010123	4.028158	4.041306	3.708904	4.868038	4.893383	4.85839	5.104613	4.644704	4.728079	5.291226

SLC34A2	204124_at	3.118949	3.007679	2.978961	2.993301	3.035809	2.961364	3.004585	3.009136	2.996925	2.998173	3.020431	3.074149	2.7534
NDUFAF1	204125_at	7.908597	7.764534	8.240513	8.231161	7.99356	7.678456	6.615271	6.943142	7.096419	7.410964	7.039625	6.952157	6.5588
CDC45L	204126_s_at	2.402075	2.819804	2.233247	2.238028	2.032159	2.237079	5.504679	5.39772	5.294939	5.58403	5.002933	5.539097	3.556838
RFC3	204127_at	5.723544	5.673599	5.770235	5.079036	4.371869	4.169883	9.299151	8.263397	9.494782	9.31327	8.166575	8.24442	8.004684
RFC3	204128_s_at	2.436166	2.417712	2.444251	2.358728	2.390257	2.33938	6.598652	2.423182	6.699515	6.668583	2.40293	2.416969	6.349584
BCL9	204129_at	3.446685	3.400915	3.249917	3.379599	3.391013	3.386505	3.394446	3.38641	3.343126	3.384727	3.458088	3.382391	5.412519
HSD11B2	204130_at	2.253906	2.247863	2.305815	2.232065	2.23981	2.258285	2.182942	2.212112	2.239535	2.18694	2.180666	2.225732	2.192017
FOXO3	204131_s_at	8.570768	8.606722	8.419197	8.698154	8.769552	8.805194	9.122072	9.831155	9.187365	9.204747	9.788313	9.630719	8.133872
FOXO3	204132_s_at	5.484993	5.368203	6.638983	5.960034	4.608595	3.568067	7.724559	5.632555	7.700163	7.65964	5.474523	4.488218	7.317069
RRP9	204133_at	3.807442	3.774392	3.513726	3.631146	3.774501	3.755577	3.771783	3.771652	3.779674	3.759593	3.980594	4.957171	3.764719
PDE2A	204134_at	5.955664	5.745025	5.712542	5.816166	6.501986	5.804452	4.873316	4.884679	5.408116	4.86097	5.110168	5.667765	3.904879
FILIP1L	204135_at	3.604443	3.587839	7.146504	6.674074	3.588067	3.568527	2.760807	2.768965	2.756758	2.73174	3.568478	3.285384	7.254084
COL7A1	204136_at	4.107023	4.47995	4.037317	4.178637	4.409865	4.042089	3.858163	4.052917	4.036789	4.017799	4.03443	4.012358	4.339288
GPR137B	204137_at	8.041243	7.815056	7.899921	7.804902	8.142346	7.289798	7.085453	6.287271	6.007787	6.274758	5.452306	6.874263	7.255595
MZF1	204138_s_at	2.617177	2.603644	2.535813	2.578639	2.620781	2.596301	2.604328	2.600897	2.58963	2.581902	2.581854	2.657357	3.186108
MZF1	204139_x_at	6.216313	5.920303	6.178257	5.777623	5.863568	6.363848	6.440696	6.213774	5.868421	5.67647	5.858767	5.79224	6.224821
TPST1	204140_at	6.850616	6.476852	7.203402	7.144135	6.466398	6.223212	5.594686	4.817823	5.565109	5.544095	5.602187	5.370667	6.259329
TUBB2A	204141_at	12.38556	12.37076	10.25498	10.19561	11.09073	11.09923	10.72371	11.4616	11.19462	11.57045	12.02353	12.21516	13.2989
ENOSF1	204142_at	9.478248	9.412087	9.532919	10.08571	8.681291	8.520141	8.79956	8.688081	9.417008	9.367118	8.947336	9.332868	7.417747
ENOSF1	204143_s_at	8.947123	8.99474	10.12781	9.934058	8.047891	7.172704	8.612481	7.589252	9.483139	9.521268	8.062845	8.527974	6.5865
PIGQ	204144_s_at	3.809723	4.152341	3.983169	3.924217	4.487749	4.642739	6.891927	7.767254	6.574865	6.986854	7.254795	7.500251	2.978666
FRG1	204145_at	8.431769	8.645246	8.338385	8.720932	8.948541	9.175976	8.703955	9.547366	8.836653	9.129995	9.056578	9.524539	8.326499
RAD51AP1	204146_at	4.885199	3.643782	2.999887	3.013683	2.924872	3.538436	8.768853	8.610681	8.939489	9.07744	8.17384	8.470341	7.549413
TFDP1	204147_s_at	3.052117	3.205257	3.302093	3.18873	3.034887	3.156148	7.580542	3.446884	7.683683	7.700827	3.218805	3.255769	5.878554
POMZP3	204148_s_at	5.754217	5.732216	6.205633	6.457738	6.376861	6.435891	6.187805	6.865702	4.151099	6.523357	8.544798	8.476234	6.389414
GSTM4	204149_s_at	4.278272	4.390168	4.097015	3.779256	3.819954	3.746074	4.633861	2.990461	4.639386	4.445038	2.700342	3.728414	4.276862
STAB1	204150_at	8.139568	8.048022	8.151652	8.341417	8.509066	8.47141	6.273171	5.90036	5.866367	6.11598	6.725852	6.966803	5.250843
AKR1C1	204151_x_at	13.28011	13.23533	13.07349	13.14348	13.11744	13.16759	12.79333	13.23884	13.11353	13.32694	12.68544	13.04227	9.005483
MFNG	204152_s_at	1.981608	1.968839	1.949613	1.952085	1.968364	1.951697	1.964974	1.965647	1.956581	1.949257	1.946703	1.959012	1.949405
MFNG	204153_s_at	5.49614	4.967786	5.439583	5.523843	5.626093	6.268237	5.692738	6.054557	6.083994	5.739201	6.094107	6.554015	3.278124
CDO1	204154_at	12.1685	12.15846	12.66199	12.50815	11.82908	11.42526	10.17291	9.333477	10.47011	9.561021	8.974195	8.989422	9.09829
KIAA0999	204155_s_at	6.125948	5.928338	6.255988	6.039664	4.089139	4.652103	5.411927	4.346531	5.462297	5.278945	4.377975	4.803227	5.224557
KIAA0999	204156_at	5.946108	6.018847	6.483184	5.767685	3.370113	2.830203	6.580755	4.333914	6.942705	6.959523	3.982669	3.62391	6.436927
KIAA0999	204157_s_at	7.743092	7.561174	7.385441	7.619876	7.19811	6.99685	7.184594	7.501501	7.390633	7.17142	7.225616	7.519734	5.110308
TCIRG1	204158_s_at	7.917737	7.760233	6.603513	7.085611	7.692262	8.425124	6.480378	7.282618	6.213413	6.112888	7.431145	7.615133	4.773604
CDKN2C	204159_at	6.700275	6.603489	5.2742	5.556149	6.276462	5.682728	8.512428	8.784499	8.581207	8.998559	7.904955	8.337327	5.553019
ENPP4	204160_s_at	5.379954	5.279024	5.761977	5.570869	4.664242	4.635832	5.723317	5.215998	5.628691	5.349309	4.760111	4.830902	6.066681
ENPP4	204161_s_at	5.605234	5.149356	5.404666	5.326003	5.037006	4.125572	5.668471	4.841171	5.401618	4.950174	4.726127	4.805281	5.581203

NDC80	204162_at	5.052956	4.418584	2.9636	2.878707	3.03164	3.100249	9.393346	9.695841	9.665529	9.679173	9.1192	9.420475	7.781021
EMILIN1	204163_at	5.781712	5.460933	5.212505	5.485692	5.398737	5.443322	5.46105	4.263006	5.199052	5.284951	6.598644	5.641413	7.860682
SIPA1	204164_at	6.734727	6.86713	6.232001	6.461483	6.311781	6.308831	6.522419	6.831104	6.510116	6.462565	6.566554	6.973602	4.493035
WASF1	204165_at	5.664516	5.563028	5.733964	5.707273	5.223385	5.18712	7.198254	7.072251	7.43225	7.188117	7.044535	7.182639	8.3362
SBNO2	204166_at	4.351747	4.33945	4.277597	4.09408	4.340449	4.334434	4.34229	4.336478	4.32594	4.330624	4.326302	4.330237	3.92424
BTD	204167_at	7.186186	7.177862	8.192794	8.239305	7.04021	6.732704	5.9523	6.047446	6.104418	6.231933	5.457356	5.90885	3.21202
MGST2	204168_at	10.17527	10.12316	10.4224	10.43202	10.18512	10.06507	9.871863	9.915705	10.14766	10.03695	9.917406	10.07878	6.657755
IMPDH1	204169_at	4.420088	3.566935	4.405668	4.380271	4.500105	4.478515	4.442584	4.468591	4.135168	4.435317	4.4522	4.676124	5.536973
CKS2	204170_s_at	6.949556	6.925315	4.768571	5.374611	5.028185	4.919432	10.97621	10.67925	11.09451	11.13552	10.32319	10.60942	9.160784
RPS6KB1	204171_at	6.543525	6.513051	6.606749	6.545106	6.175856	6.046498	7.07272	6.58039	7.117268	6.957075	6.889097	6.9171	7.09831
CPOX	204172_at	8.201434	7.769169	7.76987	7.900871	7.284086	6.941878	12.077	11.81792	12.06897	12.38128	11.06513	11.61599	7.589553
MYL6B	204173_at	7.715827	7.48787	5.245157	5.640419	7.192354	7.394555	8.420531	9.241939	8.499244	8.527546	11.28697	9.656391	11.63714
ALOX5AP	204174_at	2.72169	3.086656	3.763456	2.69096	2.725635	2.26135	2.682825	2.688054	2.675288	2.638376	2.551332	2.615157	3.81272
ZNF593	204175_at	8.436911	8.191439	7.522427	8.082429	8.599135	8.570016	6.698139	7.605813	7.3586	6.996061	8.128039	8.085028	6.059228
KLHL20	204176_at	7.00609	6.664618	7.417064	7.219898	6.588556	6.101128	6.665158	5.962753	6.672014	6.641464	6.098963	6.134636	7.749366
KLHL20	204177_s_at	6.586388	6.377516	7.02783	6.940783	6.34679	5.809829	5.683166	5.245629	6.23984	5.928868	5.472888	4.971289	6.854145
RBM14	204178_s_at	5.894709	5.98871	6.143326	6.129858	6.126199	5.982594	6.840898	6.905997	6.764312	6.666364	6.596925	6.87214	6.193335
MB	204179_at	2.336408	2.323371	2.294589	2.304663	2.337387	2.303823	2.310599	2.320939	2.307567	2.299131	6.460587	2.318001	6.615037
ZBTB43	204180_s_at	4.261924	4.687098	4.842512	4.585541	4.358719	4.585613	4.356062	4.588651	4.613954	4.018591	4.580945	4.652691	4.50528
ZBTB43	204181_s_at	5.467707	5.601711	6.03892	6.152063	5.556392	5.233145	5.682274	5.752383	5.56405	5.575008	5.85904	5.399127	5.466524
ZBTB43	204182_s_at	5.747373	6.055534	6.020178	5.966483	5.476375	5.438667	6.171464	5.692801	6.024847	6.094042	5.754385	5.974437	5.566985
ADRBK2	204183_s_at	2.569356	2.665138	2.466371	2.463142	2.545899	2.494209	2.510851	2.521823	2.659517	2.50932	2.506248	3.020504	2.41933
ADRBK2	204184_s_at	3.229094	3.12744	3.099221	3.123169	3.167421	3.121904	3.10447	3.135518	3.116646	3.103734	4.034013	3.391922	3.602842
PPID	204185_x_at	8.161633	8.178868	7.891357	7.810122	7.557651	7.568089	8.278233	7.967933	8.43566	8.529384	7.658788	7.967941	8.107202
PPID	204186_s_at	6.904864	6.890809	7.402425	6.96704	6.178248	5.400665	7.956999	6.167904	7.888884	8.134856	6.308019	6.736998	8.505164
GMPR	204187_at	2.163853	2.175285	2.587171	2.741051	2.831275	2.613115	6.907255	7.347311	6.890307	7.209069	6.561205	7.242937	3.467059
RARG	204188_s_at	2.394972	2.364101	2.358724	2.417457	2.366569	2.345679	2.494919	2.364908	2.361585	2.338246	2.350537	2.368507	3.235084
RARG	204189_at	3.500041	3.704598	3.685114	3.604755	3.69894	3.688592	3.894767	3.702021	3.720502	3.926045	3.726594	4.214366	4.182107
USPL1	204190_at	6.618765	6.256947	6.759787	7.025217	6.217438	6.534735	6.652108	5.561699	6.693455	6.290997	5.757708	5.860713	6.8106
IFNAR1	204191_at	4.946409	4.998258	4.81562	4.66215	5.596747	6.133799	3.023671	3.689936	3.672479	3.26442	3.605132	4.190078	2.806549
CD37	204192_at	2.205051	2.194111	2.177236	2.180907	2.195359	2.17914	2.191789	2.191989	2.150395	2.178318	2.173654	2.185401	2.13902
CHKB	204193_at	8.469953	8.683611	7.857439	7.964952	8.098286	7.892737	7.532618	7.897059	7.311298	7.298661	7.804917	7.942358	8.089124
BACH1	204194_at	7.452485	7.247415	7.759356	7.529523	6.939918	5.957421	8.085099	7.031486	8.017051	8.146368	6.615149	6.95936	7.695574
PKNOX1	204195_s_at	4.904785	4.74738	4.751598	4.774566	4.8929	4.877489	4.719108	5.184005	5.0707	4.876411	5.003721	4.975586	5.19433
PKNOX1	204196_x_at	3.331991	3.297478	3.178662	3.247356	3.317584	3.27637	3.286837	3.265363	3.270111	3.249183	3.278219	3.29079	3.089369
RUNX3	204197_s_at	2.541928	2.320546	2.291677	2.296254	2.319633	2.47761	2.292229	2.306921	2.247217	2.260102	2.298083	2.280938	2.301367
RUNX3	204198_s_at	4.52339	4.518738	4.657799	4.564049	4.9365	6.284243	3.063694	4.574824	3.916224	3.775683	4.486849	4.12291	4.115172
RALGPS1	204199_at	4.496493	4.302439	2.454216	2.462269	2.45938	2.831962	2.456157	2.430872	2.342695	2.442854	2.473964	2.458231	6.211745

PDGFB	204200_s_at	3.229202	3.204433	3.160064	3.190485	3.352327	3.190451	3.199007	3.190702	3.189419	3.17254	3.179535	3.136709	3.142335
PTPN13	204201_s_at	4.735133	4.413962	4.674762	4.669104	4.233212	4.603068	5.209133	4.705666	4.678921	4.658022	6.071886	5.507107	8.593491
IQCE	204202_at	5.738185	5.302597	4.988208	4.994666	5.119901	5.066319	4.848239	4.983695	4.822776	4.819788	4.833196	4.479467	5.917667
CEBPG	204203_at	6.897567	6.884557	6.980948	7.058874	6.478563	5.82729	5.842102	5.530782	5.557614	5.667953	5.045121	5.322461	5.170621
SLC31A2	204204_at	6.339918	6.323782	8.011371	8.018584	7.39466	7.440989	4.194611	4.722047	4.642964	4.033459	4.187905	4.447379	4.435832
APOBEC3G	204205_at	6.032292	5.870462	5.752726	5.866183	6.426791	6.472053	5.340563	5.881607	5.634053	5.783132	5.699688	6.05772	3.98425
MNT	204206_at	7.587454	7.498687	7.387898	7.682361	7.502015	7.654227	6.428214	7.034546	6.326088	6.053289	7.235727	7.057516	7.162054
RNGTT	204207_s_at	3.243517	2.940655	3.261367	3.12702	3.158819	3.266329	4.08511	3.108539	4.024151	4.461645	3.260492	3.150252	4.783913
RNGTT	204208_at	6.62402	6.498855	6.601823	6.592409	6.724225	6.228675	7.441544	7.524374	7.829974	7.922675	7.042373	7.451901	7.211558
PCYT1A	204209_at	2.797586	2.419902	2.617141	2.435858	2.442772	2.72764	2.425637	2.662548	3.231769	2.514707	2.477398	2.46429	3.319199
PCYT1A	204210_s_at	2.880819	2.782303	2.856569	2.860396	2.874915	2.856405	2.873578	2.923502	3.116051	2.858756	2.86483	2.875987	2.795329
EIF2AK2	204211_x_at	7.033655	7.075768	6.898319	6.997949	7.012006	6.851615	7.083592	7.184057	6.998995	6.994463	7.027202	6.985045	6.956612
ACOT8	204212_at	4.346308	4.278511	4.596365	4.401992	4.242483	4.518421	4.543725	4.90955	4.675247	4.826204	4.381454	4.954793	3.573357
PIGR	204213_at	2.263587	2.249676	2.217794	2.231687	2.26272	2.23407	2.249809	2.251703	2.236954	2.237186	2.256335	2.243335	2.204081
RAB32	204214_s_at	7.00386	6.967921	6.947778	7.050061	7.129135	6.872205	7.583341	7.022214	7.049749	7.138186	6.879777	7.688621	6.665485
C7orf23	204215_at	5.094999	5.085289	5.129009	4.887154	5.241014	5.157868	6.117455	5.335765	5.934387	5.599134	5.781552	5.537918	6.326355
ZC3H14	204216_s_at	6.983917	7.054141	7.505396	7.391324	6.633464	6.212113	7.433097	6.627738	7.641125	7.262065	6.78961	6.743479	7.720257
RTN2	204217_s_at	2.586062	2.572808	2.541699	2.549934	2.571842	2.682642	2.561959	2.564607	2.558889	2.552925	3.821494	2.57628	5.777944
C11orf51	204218_at	6.404363	6.658014	6.402516	6.924717	6.815746	7.100775	7.386402	8.324831	7.959722	7.604804	7.914031	8.368421	6.021943
PSMC1	204219_s_at	10.03026	10.02296	9.784926	9.992319	9.934991	9.875631	10.60845	10.68865	10.48501	10.42461	10.46719	10.73279	9.953931
GMFG	204220_at	6.55122	6.600692	6.701045	6.672752	7.385563	7.753289	6.462842	6.658631	6.012493	6.317968	6.692372	7.274555	4.998903
GLIPR1	204221_x_at	2.572958	2.547773	2.846592	2.565915	2.568412	2.501019	2.627886	2.555747	2.694439	2.562946	2.545668	2.551218	3.362337
GLIPR1	204222_s_at	3.598825	3.730224	4.282726	3.719251	3.539539	3.72048	3.866412	3.373046	3.722592	3.660525	3.381765	3.510729	4.671812
PRELP	204223_at	4.887799	4.722637	5.070125	5.104535	5.880846	6.148191	3.409134	3.906198	2.210901	3.179884	3.073557	3.7861	5.637522
GCH1	204224_s_at	10.10466	9.914876	11.15175	11.19829	10.31928	9.630962	10.42798	9.271449	10.54052	10.40703	8.713742	9.062374	6.578609
HDAC4	204225_at	5.451617	5.421083	5.381956	5.520152	5.709993	5.185447	5.438469	5.537277	5.496772	5.449463	6.297986	5.752944	7.590435
STAU2	204226_at	5.22936	4.643298	5.999441	5.672758	5.050149	4.023983	6.128306	4.702233	6.089359	5.289374	5.288083	5.218281	6.424918
TK2	204227_s_at	6.112039	6.515991	5.754503	6.149409	6.672582	6.540956	4.385276	5.019866	3.865718	4.245979	5.089259	4.714145	3.985702
PPIH	204228_at	6.58996	6.466059	6.28975	6.497236	6.551621	6.560589	8.412285	8.513058	8.717232	8.655791	8.304812	8.422436	8.096878
SLC17A7	204229_at	2.07402	2.058798	2.023654	2.045586	2.353643	2.044099	2.05658	2.056142	2.042883	2.046624	2.039281	2.050811	2.055211
SLC17A7	204230_s_at	2.491991	2.48963	2.472653	2.486819	2.495801	2.49051	2.490885	2.499653	2.483366	2.487964	2.403163	2.495235	2.467088
FAAH	204231_s_at	7.191318	7.371721	6.686892	6.894242	7.822676	7.989773	3.730614	3.203416	3.041251	2.950633	3.753867	3.763128	3.091393
FCER1G	204232_at	4.839933	4.758305	6.438085	6.152549	5.328991	4.823593	6.09274	5.04785	6.162272	5.612235	4.419981	5.306708	4.007796
CHKA	204233_s_at	5.647842	5.416852	4.733479	5.408937	4.997304	4.566851	5.968875	4.766971	6.214218	5.833878	4.717115	5.388856	5.583748
ZNF195	204234_s_at	4.556739	4.965013	5.268515	5.33516	5.265956	4.539227	5.91755	4.76491	5.739815	5.749406	5.990436	5.44282	6.856314
GULP1	204235_s_at	2.875348	2.865681	2.807211	2.878305	2.986875	2.796663	4.001087	4.152955	4.878077	3.97758	5.499812	2.920168	8.263403
FLI1	204236_at	5.70828	5.513147	6.200914	5.882891	6.122254	6.024666	6.47289	6.265236	6.513291	6.298564	6.005019	6.375388	7.242358
GULP1	204237_at	3.117175	3.061941	3.420554	3.443626	3.871089	3.508179	5.713937	5.448603	6.710007	4.666013	7.363931	4.642356	9.313899

C6orf108	204238_s_at	7.145614	7.269489	8.070255	7.901874	7.293196	6.807749	7.509911	6.622457	7.624737	7.821558	6.733299	7.259207	6.285511
NNAT	204239_s_at	2.870832	2.984746	2.867744	2.8187	2.896579	2.783369	3.429261	2.882362	2.912902	3.191288	5.95114	3.171524	10.91851
SMC2	204240_s_at	3.428303	3.309236	2.884236	3.167047	2.546579	2.58733	7.975466	7.508139	8.412725	8.224865	5.776638	7.224811	7.182377
ACOX3	204241_at	7.57599	7.478684	7.046411	7.061491	7.801647	7.744593	6.059278	6.474156	5.859911	5.930209	6.847093	6.350257	5.53026
ACOX3	204242_s_at	4.279558	4.462143	3.97463	4.021005	4.028011	3.797413	3.52202	3.302823	3.518362	3.503977	3.913927	3.510133	3.265211
RLF	204243_at	5.584318	5.669085	6.709463	6.539293	5.684869	6.007816	6.43826	6.396212	6.598421	7.042744	6.219138	6.38065	6.730108
DBF4	204244_s_at	4.932856	4.965696	5.32727	5.202229	4.911153	4.448864	9.077491	8.537203	9.197851	9.069879	8.374741	8.568081	7.472211
RPP14	204245_s_at	7.200364	7.213667	7.519996	7.230947	6.385533	5.795138	7.127618	6.463713	7.250831	7.014548	5.779403	5.927945	7.398418
DCTN3	204246_s_at	8.14869	8.044663	8.368687	8.366191	8.287039	8.051207	9.103184	8.990975	9.2785	9.295417	9.104389	9.253878	9.200315
CDK5	204247_s_at	4.47099	4.260859	4.483349	4.515589	4.408267	4.091176	4.464016	4.507582	4.585742	4.550672	4.447728	4.489856	5.574837
GNA11	204248_at	4.625286	4.650243	4.960487	4.651508	4.690672	4.630374	5.064584	4.880708	5.337661	5.061739	4.792343	4.623983	5.462957
LMO2	204249_s_at	7.13988	7.042401	7.091195	7.175884	7.541368	7.189075	11.93247	11.28528	11.79086	12.14714	10.67964	10.98707	8.115133
CEP164	204250_s_at	2.522474	2.492663	2.473785	2.471795	2.504579	2.46934	2.48124	2.518028	2.480555	2.474861	2.483102	2.49186	2.818704
CEP164	204251_s_at	5.923812	5.747319	5.524252	5.652949	6.072817	6.840735	4.795212	5.746583	4.519349	4.951257	6.192977	5.121027	5.674502
CDK2	204252_at	5.224273	4.947718	4.981976	4.501202	4.664007	5.041446	6.612043	5.561441	7.075922	6.563916	5.154661	5.724017	6.275444
VDR	204253_s_at	2.748331	2.709401	2.625767	2.688433	2.852004	2.861427	2.696495	2.700773	2.687409	2.683965	2.687088	2.701692	2.475352
VDR	204254_s_at	4.423433	4.732536	4.714665	4.614861	5.111429	5.51455	4.371549	4.719916	4.642586	4.425837	4.713342	4.654099	4.602852
VDR	204255_s_at	2.779019	2.744146	2.704103	2.73023	2.76867	2.703386	2.737681	2.744045	2.734407	2.722432	2.731771	2.762693	2.61057
ELOVL6	204256_at	11.53055	11.4632	9.698021	9.844613	9.315955	9.381835	9.840509	10.58414	9.831919	10.01075	10.29119	10.91336	7.012086
FADS3	204257_at	6.487566	6.635567	6.572109	6.785643	7.511923	7.229838	6.494064	6.654162	6.670319	6.51894	6.711243	6.851267	6.704586
CHD1	204258_at	7.159453	7.030601	7.88972	7.653352	6.749142	6.724177	8.406474	7.474776	8.422689	8.410152	7.34007	7.516167	8.161898
MMP7	204259_at	2.126684	2.119055	2.09606	2.10259	2.169393	3.020513	2.111626	2.113199	2.102131	2.094775	2.095326	2.109548	2.027213
CHGB	204260_at	2.531331	2.516005	2.494455	2.500259	2.521726	2.495372	2.510254	2.515648	2.499044	2.486098	2.504384	2.508054	7.329606
PSEN2	204261_s_at	3.568414	3.545272	3.5063	3.392754	3.606734	3.541102	3.521732	3.564945	3.570349	3.546072	3.55419	3.556274	3.514744
PSEN2	204262_s_at	4.513828	4.984358	3.756287	3.57595	3.808901	3.911361	3.449752	3.584664	3.343826	3.439278	3.702944	3.377112	4.767976
CPT2	204263_s_at	7.69987	7.651891	8.032259	7.845126	7.51525	7.313085	6.064182	5.945041	6.108616	5.999519	5.900591	5.784286	4.836863
CPT2	204264_at	10.05332	9.854682	9.575296	9.657696	9.793836	9.842778	7.508898	8.204234	7.611119	7.754436	7.827595	7.631279	5.589167
GPSM3	204265_s_at	5.600134	5.31103	5.396081	5.234443	6.022423	6.562116	5.31538	5.605303	4.79053	5.231745	5.684279	5.728995	3.850653
CHKA	204266_s_at	6.622908	6.629022	6.468365	6.491296	6.565234	6.503442	6.578973	6.278948	6.549234	6.521257	6.547802	6.555061	6.262021
PKMYT1	204267_x_at	2.166481	2.173818	2.148771	2.134034	2.160025	2.150811	4.522187	3.921654	3.943135	3.999131	3.452136	5.040973	2.088939
S100A2	204268_at	2.142809	2.120453	2.095186	2.103547	2.13027	2.101722	2.113193	2.121224	2.111979	2.102356	2.103009	2.117294	2.069358
PIM2	204269_at	2.9827	3.45926	2.776539	2.642561	3.566404	3.836819	5.902576	5.890822	6.04881	6.053182	5.24286	5.632901	2.785658
SKI	204270_at	4.256354	3.965012	4.139295	4.051566	4.338437	3.965388	4.168196	3.971483	3.966268	3.939228	4.294773	4.068275	4.416236
EDNRB	204271_s_at	8.383582	8.61682	8.781967	8.707053	8.899447	8.844815	8.354571	7.752276	6.760828	7.007045	8.033979	8.36622	8.290384
LGALS4	204272_at	5.39116	5.262483	6.380462	6.662864	8.122286	8.073245	3.211248	3.21198	3.201545	3.196131	3.193387	3.204494	3.166334
EDNRB	204273_at	9.161907	9.026841	9.724136	9.410605	8.772598	8.509954	8.965706	7.121752	7.498322	8.066937	7.491549	7.443227	9.730013
EBAG9	204274_at	9.657572	9.747173	9.861602	9.936082	9.683714	9.655833	9.102576	9.193086	9.149169	9.169118	9.039537	9.242216	8.47721
SOLH	204275_at	4.794307	4.522634	4.54818	4.551193	4.812125	4.312603	5.543961	5.072103	6.110972	5.582813	4.522566	4.946322	3.755763

TK2	204276_at	6.977309	6.884819	7.187365	7.442968	7.698675	7.607981	5.059627	5.445281	5.393408	5.19056	5.41147	5.364305	4.924125
TK2	204277_s_at	4.109339	4.25246	5.271677	5.690554	4.921399	3.731475	3.504992	3.516229	3.496968	3.577417	3.503773	3.511889	3.439158
EBAG9	204278_s_at	7.64672	7.369406	8.40272	8.22477	7.986908	7.762848	7.513527	7.195599	7.39446	7.578915	7.287652	7.322317	6.745667
PSMB9	204279_at	6.206914	5.97318	6.391264	6.131036	5.910127	6.211641	1.747087	2.421078	2.308671	1.831444	1.711458	1.755321	1.591688
RGS14	204280_at	3.632811	4.116088	4.188291	4.059017	4.308131	3.43022	3.036644	2.923517	3.238294	2.967746	2.765579	2.948255	2.681479
TEAD4	204281_at	5.403228	5.399609	5.354103	5.38173	5.401132	5.724631	5.393961	5.404353	4.915777	5.053448	6.717926	5.622195	6.029422
FARS2	204282_s_at	6.51193	5.968311	6.023959	6.391365	6.417435	7.035683	5.99223	6.436859	6.075675	5.865639	6.876157	6.83172	4.648794
FARS2	204283_at	6.328488	6.264499	6.763476	6.826166	5.830215	5.57581	7.141561	6.399017	7.199226	6.715978	6.60848	6.49794	6.105047
PPP1R3C	204284_at	9.156804	9.211392	9.47669	9.098864	9.447342	8.843717	8.647176	8.305898	8.956707	9.228211	7.644051	8.228195	9.203486
PMAIP1	204285_s_at	4.4153	3.550789	4.957077	4.560871	5.482845	5.265216	6.29162	5.404736	6.168429	6.055788	5.319369	5.346556	5.7586
PMAIP1	204286_s_at	3.225034	3.090082	3.065948	3.143151	3.21213	3.091968	3.253992	3.347383	4.045698	3.492898	3.226269	3.288511	4.001463
SYNGR1	204287_at	3.862943	4.021411	3.890511	4.004251	4.050509	4.057217	4.206795	4.608813	4.503137	4.029871	4.324392	5.041948	5.08014
SORBS2	204288_s_at	9.579824	9.630222	10.36199	10.26889	10.04777	9.767991	7.044799	7.169027	7.434952	7.242048	7.398399	7.151166	8.731276
ALDH6A1	204289_at	4.79942	4.469387	6.39623	6.091412	3.888258	2.969811	2.814318	2.801587	3.128464	3.124222	2.78424	2.818953	2.726409
ALDH6A1	204290_s_at	10.02672	9.921706	11.15661	10.73257	9.869487	8.788752	9.393853	7.80954	9.205924	9.265802	7.55235	7.655189	6.644307
ZNF518	204291_at	7.087547	6.979184	6.595944	6.7486	6.816785	6.59148	7.754636	7.622958	7.71423	7.596996	7.646468	7.583575	8.396141
STK11	204292_x_at	5.14079	5.135867	4.948505	4.720006	5.125492	5.121074	5.327144	6.809105	5.356136	6.010843	5.952316	6.419619	3.905607
SGSH	204293_at	2.473153	2.453924	2.442719	2.458808	3.182099	2.447131	2.101627	2.478829	2.293181	2.274719	2.442884	2.434173	2.40217
AMT	204294_at	8.726544	8.78577	8.895447	8.937296	8.916398	8.854959	8.618127	8.236648	8.465974	8.478287	7.698067	7.939438	5.72506
SURF1	204295_at	10.85413	11.02204	10.37601	10.63188	10.85519	10.90059	9.757343	10.59676	9.924389	9.982935	9.853476	10.51399	8.05433
DCTN1	204296_at	2.925452	2.907214	2.78242	2.88556	2.923106	2.888515	2.901942	2.905947	2.892979	2.881417	2.893849	2.901147	2.788901
PIK3C3	204297_at	7.87169	7.815675	7.853198	8.038123	7.880695	7.559075	8.430324	8.343157	8.448196	8.385584	8.363361	8.448366	8.143261
LOX	204298_s_at	2.62824	2.54448	2.492902	2.516249	2.537069	2.496198	2.526641	2.538452	2.536312	2.51003	2.526932	2.534556	7.434964
FUSIP1	204299_at	6.926146	6.403293	7.368557	7.170692	6.098209	5.407274	8.187661	7.182725	8.216983	8.12486	7.110706	7.469072	8.572139
PET112L	204300_at	5.347801	5.21019	5.615794	5.690034	5.438433	5.200692	5.693992	5.765339	5.840318	6.08624	5.492822	5.635833	4.948352
KBTBD11	204301_at	4.127584	3.676406	5.617698	5.74312	5.636718	4.929919	3.697557	3.710116	3.08865	3.861635	4.821507	4.368609	6.305015
KIAA0427	204302_s_at	4.118286	4.373908	4.080937	4.202032	4.395376	4.920408	3.930649	4.084193	3.686438	4.082329	3.780641	4.077066	3.985219
KIAA0427	204303_s_at	6.361853	6.521436	5.868154	6.265718	6.746046	7.061281	3.768624	4.007597	4.013229	3.619407	4.808064	3.744592	4.936496
PROM1	204304_s_at	3.952021	4.493269	3.444846	3.718822	4.19287	4.504226	6.279414	4.993155	4.791451	4.889895	6.782375	5.611116	8.641627
MIPEP	204305_at	6.047262	6.253923	6.533158	6.496565	6.112651	6.206297	6.670434	6.275494	6.255792	8.431441	6.03443	6.236972	4.971897
CD151	204306_s_at	7.53259	7.603878	5.708276	5.803742	7.826758	7.825322	5.414095	5.533671	5.360054	5.29425	6.17404	6.36552	6.260391
KIAA0329	204307_at	5.290293	4.721089	3.946734	4.314471	4.880923	5.118435	3.58885	4.858805	3.584732	3.655401	4.959813	4.721097	4.782004
KIAA0329	204308_s_at	4.609296	4.326229	4.571235	4.724351	4.778745	4.674486	4.443103	4.953182	4.779664	4.427759	4.763594	4.655131	5.413888
CYP11A1	204309_at	3.274927	3.490094	2.32347	2.475353	2.347411	2.342218	2.331284	2.041769	2.279077	2.314022	2.042886	2.063457	7.32535
NPR2	204310_s_at	6.004064	5.777855	5.930395	5.854145	5.73037	5.684987	6.827436	5.867519	6.600644	6.679607	6.068961	5.884383	6.695798
ATP1B2	204311_at	4.658545	4.609986	4.603459	4.63121	4.76398	4.693845	4.851941	5.175879	7.436099	6.69847	4.628644	5.562256	4.490363
CREB1	204312_x_at	2.733921	2.679057	3.096436	2.768685	2.695614	2.754683	3.684826	2.774057	4.501668	3.961058	2.771981	2.783988	5.985914
CREB1	204313_s_at	5.124281	5.368028	6.519779	5.867594	5.219742	5.354984	7.696848	6.042684	7.956887	7.634556	6.028917	6.088607	8.987602

CREB1	204314_s_at	7.269665	7.157238	7.522346	7.29472	6.991032	7.089578	8.363103	7.689313	8.402204	8.30906	7.809981	8.083193	8.896086
GTSE1	204315_s_at	2.148013	2.140052	2.11973	2.124732	2.140137	2.125251	3.0201	5.72191	2.991889	2.832996	4.086691	5.310664	2.345559
RGS10	204316_at	3.564051	3.518703	3.537493	3.542199	3.553603	3.550543	3.558715	4.265054	3.694685	3.548348	4.400863	4.581134	3.538144
GTSE1	204317_at	2.789834	2.751525	2.699483	2.736456	2.774561	2.720612	4.673604	3.61985	4.928233	4.491381	3.742423	3.142497	3.631114
GTSE1	204318_s_at	3.778635	3.084461	2.779572	2.81655	2.947046	3.853421	7.636676	8.661036	8.061128	7.687469	8.419733	8.554798	5.224393
RGS10	204319_s_at	5.006028	5.19449	4.507064	3.866711	5.566942	6.271344	10.21079	10.31141	10.15576	10.15506	9.895081	10.40143	8.032576
COL11A1	204320_at	2.059366	2.051795	2.0285	2.03472	2.050005	2.033594	2.049128	2.051708	2.045695	2.127628	8.490847	3.238278	12.60883
NEO1	204321_at	2.742462	2.555568	2.404153	2.40994	2.082449	2.408393	2.997961	2.55508	2.55275	2.847708	3.611712	3.106691	5.616081
GOLIM4	204322_at	2.396054	2.319371	2.349907	2.867671	2.379356	2.357935	2.36696	2.37633	2.35997	2.352824	2.362002	2.366559	2.26978
NF1	204323_x_at	2.022693	2.020073	2.000493	2.007949	2.022095	2.079486	2.018307	2.018937	2.01005	2.006186	2.098691	2.011993	1.972185
GOLIM4	204324_s_at	8.028203	7.433786	7.829857	8.454443	8.789967	8.561755	6.039723	7.11427	6.046135	5.650012	7.473195	7.386817	6.646064
NF1	204325_s_at	2.241884	2.190747	2.170409	2.173947	2.198666	2.16914	2.180855	2.187715	2.179199	2.167777	2.171269	2.182245	2.467195
MT1X	204326_x_at	13.13816	13.15952	13.58115	13.60969	13.85575	13.99258	13.69917	13.88031	13.69561	13.73316	13.64081	13.58489	8.90104
ZNF202	204327_s_at	4.481711	4.337013	4.416549	4.444095	4.141336	4.462565	4.471995	4.987769	4.925065	4.644576	5.293821	5.172867	6.303508
TMC6	204328_at	5.37562	5.540218	4.967369	4.881882	5.592635	6.611237	7.614115	7.938254	7.23483	6.982604	7.494631	8.264267	4.942311
ZNF202	204329_s_at	2.423306	2.405843	2.386777	2.393768	2.406381	2.390241	2.404699	2.40665	2.396898	2.389224	2.413291	2.636439	3.266659
MRPS12	204330_s_at	2.707572	2.648544	2.947926	2.621935	2.699284	2.616138	2.629862	2.669814	2.656475	2.637514	2.61475	2.659248	2.4847
MRPS12	204331_s_at	6.803886	6.811135	7.426638	7.450596	7.023831	6.578121	7.902926	7.009985	7.828048	8.006003	7.167536	7.399078	7.029324
AGA	204332_s_at	8.526477	8.348099	8.351264	8.408936	8.362595	8.280559	8.784764	9.183873	8.473768	8.793555	8.505136	8.982292	5.554843
AGA	204333_s_at	5.126332	5.723972	5.555184	4.977848	4.637583	4.968726	5.389905	6.050737	5.269089	5.999913	5.166116	5.732078	4.608231
KLF7	204334_at	4.037196	4.224867	5.326303	4.933204	4.427942	5.071498	3.318392	3.865022	3.296102	2.976606	4.321408	4.318871	5.42387
CCDC94	204335_at	5.848614	5.729327	5.70453	5.780539	5.858312	6.024899	5.980372	6.191341	5.848717	6.223735	6.100664	6.073045	5.543099
RGS19	204336_s_at	5.556652	5.557269	5.625774	5.709585	5.706097	5.813376	5.517183	5.6175	5.599562	5.596518	5.833014	6.04202	5.502997
RGS4	204337_at	2.116047	2.108386	2.105531	2.092708	2.616292	3.893748	2.065685	2.061885	2.059875	2.052656	2.295053	2.052077	6.12811
RGS4	204338_s_at	2.57337	2.556362	2.533136	2.53028	2.671417	2.539877	2.541257	2.55316	2.538284	2.525486	2.53988	2.548894	4.685481
RGS4	204339_s_at	2.047289	2.034682	2.020939	2.021613	2.034703	2.025344	2.035598	2.037198	2.024866	2.023971	2.019299	2.029387	3.137909
TMEM187	204340_at	6.055547	5.942727	6.141027	6.545334	6.304348	6.779284	5.857712	5.505708	6.058456	5.20926	5.405631	5.756661	4.668803
TRIM16	204341_at	3.500847	3.448565	4.198414	3.677156	3.471705	3.436767	3.579513	3.302581	3.43997	3.190141	4.129866	3.521699	6.130854
SLC25A24	204342_at	6.591277	6.339525	6.407461	6.501691	6.79476	6.491513	6.804562	6.539762	6.543153	6.467921	8.068091	6.986571	9.873658
ABCA3	204343_at	2.648168	2.605359	2.539347	2.593251	2.622314	3.174115	2.592291	2.603288	2.53797	2.580475	2.59138	2.589063	3.152624
SEC23A	204344_s_at	3.538429	3.737668	5.496367	4.512947	3.573055	3.471375	6.031062	3.484292	5.867113	5.707025	3.498042	3.541436	7.418377
COL16A1	204345_at	5.022574	5.27361	4.577978	4.572807	5.568533	6.324785	4.736036	4.89571	3.930175	4.597948	9.070315	4.988578	11.23086
RASSF1	204346_s_at	5.641783	5.643036	5.617513	5.568668	5.698268	5.630465	5.641514	5.607611	5.633616	5.905841	5.624385	5.768656	5.640384
LOC645619	204347_at	8.582145	8.451819	8.410216	8.270587	8.27542	8.151654	8.571157	7.709865	8.359666	8.206455	7.544476	7.74693	5.472477
AK3L1	204348_s_at	10.8286	10.87527	10.9828	10.78496	10.66576	10.24311	11.1533	10.75113	11.02667	10.89164	10.57627	10.78455	7.229102
MED7	204349_at	8.027797	7.984139	7.736263	8.07165	8.262748	8.405999	8.37766	9.438457	8.784527	8.6674	9.058695	9.236058	7.822204
MED7	204350_s_at	5.532793	5.066428	5.977378	5.460123	4.836054	5.033835	6.898305	5.698078	6.855251	7.35175	5.741441	5.733446	6.951751
S100P	204351_at	2.657377	2.613531	3.145993	2.97601	3.526752	2.674594	2.724505	3.402244	4.164503	3.258643	5.00122	5.324979	3.281612

TRAF5	204352_at	3.5072	4.26813	3.509065	3.318933	3.434516	4.715294	4.06343	4.243568	3.963409	3.948119	6.494585	3.758626	7.400746
POT1	204353_s_at	3.874489	3.871854	4.965878	4.661221	3.915557	3.370551	5.97709	4.150818	6.221448	6.004877	3.864381	4.270826	5.92741
POT1	204354_at	7.273632	7.041267	7.846767	7.878723	7.510061	7.332939	7.803562	7.341904	7.826896	7.790793	7.20444	7.309689	7.57295
DHX30	204355_at	6.645822	6.751393	6.661973	6.824271	6.31988	5.877602	7.114144	6.806497	7.135414	6.924207	7.019279	7.331054	7.353379
LIMK1	204356_at	2.226115	2.211158	2.17906	2.193115	2.210281	2.191788	2.205599	2.209968	2.195828	2.188493	2.194216	2.202984	2.14059
LIMK1	204357_s_at	2.388684	2.361533	2.332905	2.342215	2.376765	2.351478	2.349788	2.362055	2.346367	2.334511	2.350553	2.358287	2.314146
FLRT2	204358_s_at	2.98261	2.97203	2.735843	2.962122	2.971977	2.961744	3.161496	2.96975	2.94988	3.079264	3.18768	2.955756	8.611567
FLRT2	204359_at	6.562892	6.824951	5.527635	5.442945	6.840948	6.302337	6.822203	6.315843	6.197352	6.372612	9.762765	6.954671	12.39075
NAGLU	204360_s_at	7.569657	7.709582	7.260842	7.4987	7.518579	7.513573	6.335292	6.313702	6.190364	6.408911	6.49668	6.50275	4.737932
SKAP2	204361_s_at	3.344214	3.304238	3.971977	3.335074	3.334333	3.291431	4.272004	3.313338	4.401969	4.691889	3.261749	3.265508	3.779045
SKAP2	204362_at	7.073935	6.93067	7.146406	7.380406	7.654796	7.509123	7.395331	7.804329	7.81724	7.910142	7.430348	7.241436	6.979989
F3	204363_at	4.196182	4.188995	4.777914	4.247731	4.145909	4.370713	4.155152	4.11338	4.258504	4.188264	3.970161	4.446436	6.951135
REEP1	204364_s_at	3.481865	3.096812	3.481601	3.282009	3.069695	2.971444	4.281586	2.926586	4.287302	3.579781	5.791449	3.341446	10.42137
REEP1	204365_s_at	3.885241	3.858395	3.850668	3.979781	3.717739	3.772265	3.867982	3.945802	3.862952	3.850311	7.419837	4.770991	9.296118
GTF3C2	204366_s_at	6.30676	6.343527	6.290447	6.302728	6.309976	6.293085	7.497653	6.754445	7.397034	7.59028	6.556408	6.659182	7.42954
SP2	204367_at	5.435983	5.344017	4.958598	5.084929	4.855199	4.413989	6.647852	6.313867	7.030225	6.884953	5.587443	6.293832	6.11252
SLCO2A1	204368_at	2.938401	2.915916	2.879152	2.905624	2.943631	3.749484	2.908229	2.940431	3.213873	2.922969	2.922178	2.939807	3.734432
PIK3CA	204369_at	5.099508	5.457648	6.861654	6.188838	4.241882	4.550933	6.427194	4.232318	6.526373	6.626828	4.122754	4.865481	7.966519
CLP1	204370_at	7.094173	7.033514	7.172532	6.941832	6.811189	6.307133	7.526696	7.594302	7.628599	7.897265	6.960338	7.591766	7.0256
KHSRP	204371_s_at	2.55262	2.538889	2.517957	2.520222	2.538951	2.518506	2.534406	2.946702	2.528301	2.518081	2.518868	2.563449	2.441417
KHSRP	204372_s_at	9.732142	9.505351	8.94825	9.254704	9.373955	9.717447	10.69167	11.15983	10.45147	10.37662	10.99583	11.11489	9.505653
CEP350	204373_s_at	8.764423	8.6203	8.426061	8.485349	8.473512	8.097548	9.040053	8.706523	9.003412	9.092156	8.751616	8.732611	9.097472
GALK1	204374_s_at	6.292948	6.385656	6.032396	5.962444	5.014211	5.266502	6.417046	6.66463	6.993309	7.663685	6.198507	6.704643	4.205165
CLSTN3	204375_at	4.470101	4.305565	4.642715	3.997463	4.755588	4.710289	4.528254	4.504914	4.376258	4.208562	4.197858	4.729194	3.20367
VPRBP	204376_at	3.018336	2.614615	2.529859	2.60475	2.660895	2.601204	2.802325	2.611675	2.614141	2.829965	2.611542	2.694054	2.19339
VPRBP	204377_s_at	2.902973	2.892256	2.874437	3.047805	3.090947	3.277142	2.885129	2.851691	2.844162	2.823242	2.98727	2.926508	2.556541
BCAS1	204378_at	3.331807	3.229078	3.154566	3.310243	3.356827	3.290669	3.585317	3.775477	3.477149	3.299843	3.314596	4.253804	3.274454
FGFR3	204379_s_at	9.888858	9.85708	8.608203	8.907617	9.681506	9.826879	9.312427	9.679587	9.108871	9.119987	9.617557	9.792502	8.336449
FGFR3	204380_s_at	2.432865	2.420901	2.400171	2.386199	2.420874	2.523322	2.43749	2.415216	2.408192	2.403897	2.399676	2.411973	2.365409
LRP3	204381_at	7.21529	7.385338	6.499188	6.655632	7.283778	7.744602	5.077822	5.785633	5.077583	5.440313	6.245039	5.828358	5.367005
NAT9	204382_at	5.82632	5.585954	5.3779	5.372226	5.998477	6.192945	5.610002	6.140963	5.704682	5.882782	6.540189	6.198267	6.043713
DGCR14	204383_at	3.571529	3.634967	3.363981	3.530574	3.569839	3.552056	3.870178	4.389271	3.848893	3.766871	4.217243	3.943405	4.093453
GOLGA2	204384_at	5.970568	5.816511	5.942538	6.111767	5.904765	5.865851	4.75214	4.189023	5.121543	4.568348	6.008302	5.777154	6.245531
KYNU	204385_at	6.372957	6.037009	8.007918	7.680319	5.746059	4.882344	6.26816	4.781265	6.316861	6.565483	4.31026	4.810474	2.508378
MRP63	204386_s_at	10.62839	10.6728	10.55143	10.80518	10.64488	10.74633	10.56557	11.06851	10.7063	10.66569	10.947	11.11538	9.172136
MRP63	204387_x_at	9.295141	9.287056	8.717111	8.958922	9.248954	9.738453	9.309622	9.561052	8.673164	8.654012	9.691751	9.861123	8.191222
MAOA	204388_s_at	8.461944	8.479382	9.867559	9.784316	9.3037	8.898029	5.920222	6.390536	6.539514	6.220778	5.532601	5.868347	3.625785
MAOA	204389_at	6.864945	6.911931	8.418313	8.539856	7.815768	7.441284	5.061867	5.198362	5.546786	5.515582	4.549001	4.41039	3.176961

TRIM24	204391_x_at	8.14333	7.931791	7.672387	7.814588	7.915614	7.821532	9.060885	9.065781	9.047972	8.813453	8.893994	9.147507	7.996255
CAMK1	204392_at	5.688521	5.87171	5.610821	5.811961	6.203855	6.296665	4.837876	5.762538	5.6355	5.457474	5.726225	5.405021	5.308429
ACPP	204393_s_at	2.801502	2.744024	2.704345	2.72487	2.779757	2.733298	2.734375	2.743434	2.730546	2.706749	2.728183	2.735936	2.520209
SLC43A1	204394_at	7.926012	8.011272	7.760518	7.946803	8.005333	8.056108	8.729675	8.405659	8.731896	8.690063	8.572107	8.525708	2.844663
GRK5	204395_s_at	2.284315	2.270995	2.330931	2.253008	2.190952	2.252147	2.32809	2.258823	2.251599	2.247462	2.2526	2.259945	4.084212
GRK5	204396_s_at	8.332475	8.091646	7.7092	7.826994	8.270718	8.481011	7.199953	7.716911	7.214681	7.188438	8.092848	8.170731	8.65943
EML2	204397_at	2.03286	2.012899	1.992305	2.001322	2.016306	2.004432	2.004052	2.013781	1.999705	1.991797	1.999558	2.005815	1.937377
EML2	204398_s_at	3.374409	3.140438	3.122008	2.80165	3.55699	3.256173	3.078576	3.405512	3.165577	3.088251	3.067702	3.388815	3.023388
EML2	204399_s_at	1.856309	1.848105	1.829971	1.833283	1.848843	1.836848	1.84252	1.84612	1.832496	1.832425	1.831278	1.839776	1.796194
EFS	204400_at	4.52298	4.515815	4.509236	4.452029	4.811407	5.076892	4.370655	4.525184	4.516313	4.354297	7.958597	4.518464	9.840585
KCNN4	204401_at	2.600582	2.581675	2.526083	2.56966	2.607986	2.93024	6.070887	6.941885	5.977762	5.967832	5.677523	6.712277	2.514813
RHBDD3	204402_at	6.111189	6.231189	5.071887	5.458178	5.96597	6.28972	5.538887	6.498103	5.513199	5.885403	6.795505	6.804926	5.292355
FAM115A	204403_x_at	8.587665	8.754639	7.980744	8.050189	8.384023	8.657035	8.388151	8.485042	8.089404	8.314867	8.526363	8.450249	8.461209
SLC12A2	204404_at	2.622535	2.712712	2.819945	2.3501	2.681639	2.648481	2.595353	2.372268	2.488998	2.659438	2.65974	2.328756	5.118619
DIMT1L	204405_x_at	7.176437	7.115541	7.36523	7.296375	6.722484	6.732907	8.238943	7.679181	8.155993	8.135217	7.428025	7.485859	7.906336
FLT1	204406_at	2.430801	2.404273	2.383441	2.38721	2.413779	2.373995	2.394651	2.401084	2.530678	2.379979	2.382918	2.39595	2.259013
TTF2	204407_at	4.192811	3.973762	3.818637	3.972956	3.894596	3.666834	6.816752	5.635978	6.447755	6.380528	5.558626	5.424076	5.652829
APEX2	204408_at	5.451203	5.52842	5.311796	5.265686	5.503642	5.543088	6.252424	6.57524	6.141282	6.712448	6.165892	6.139911	5.480676
EIF1AY	204409_s_at	8.795202	8.677394	9.033164	9.146167	3.182262	3.245475	9.90389	10.78721	10.37646	10.87722	3.008817	10.04547	8.172994
EIF1AY	204410_at	6.191033	6.340892	6.884831	6.683608	2.890514	2.934761	7.282564	7.781968	7.808708	8.193316	3.099613	7.35953	5.859651
KIF21B	204411_at	2.101091	2.100442	2.08062	2.083308	2.10009	2.203336	2.09503	2.096935	2.084784	2.067092	2.061287	2.091959	4.707739
NEFH	204412_s_at	2.665806	2.630665	2.586399	2.590026	2.649403	2.59287	5.185952	4.378234	5.801231	5.75217	4.958816	4.735078	5.5042
TRAF2	204413_at	2.859199	2.862703	2.436112	2.748506	2.810037	3.482403	2.807244	3.180867	2.916706	2.674898	3.233221	2.852483	2.415884
LARGE	204414_at	2.771961	2.712085	2.662878	2.714473	2.760996	2.683465	2.713586	2.722522	2.716201	2.69405	2.707206	2.737279	2.55214
IFI6	204415_at	6.480857	6.120201	9.185889	8.424021	6.052476	5.480997	4.730416	4.692303	5.548804	4.025042	4.575837	4.169834	6.393731
APOC1	204416_x_at	14.65768	14.63178	14.34187	14.4835	14.68646	14.71691	13.08743	13.24009	13.30334	13.05278	12.80384	13.30162	8.095985
GALC	204417_at	6.823758	6.507226	7.96043	7.902781	6.822204	6.454616	8.601824	8.206467	8.585344	8.711608	8.083536	7.817692	9.173191
GSTM2	204418_x_at	8.92984	9.105462	5.137126	5.109344	9.603934	9.156896	5.880709	7.52869	6.792398	5.793841	7.570603	7.579945	7.723298
HBG2	204419_x_at	3.274207	4.890924	3.428904	2.466079	3.399699	3.24961	15.43174	15.34264	15.43174	15.40254	15.29818	15.428	14.55404
FOSL1	204420_at	2.603742	2.634	3.800441	3.101593	2.76712	3.062986	2.378229	2.412561	2.37222	2.366953	2.37089	2.42035	2.250523
FGF2	204421_s_at	2.54005	2.330909	2.241878	2.246493	2.273218	2.244744	2.255516	2.263201	2.247045	2.220718	2.249029	2.256553	4.318832
FGF2	204422_s_at	5.442597	5.212404	3.613451	3.191002	3.095667	2.911588	3.035596	3.004867	2.736322	2.978701	3.005844	3.042882	6.642889
MKLN1	204423_at	5.702962	5.451521	6.539822	6.61048	5.649908	5.47267	6.221114	6.194664	6.375388	6.386229	5.596366	6.113445	5.166649
LMO3	204424_s_at	2.141045	2.124168	2.093784	2.107345	2.125254	2.107691	2.120279	2.122567	2.109894	2.119235	2.127433	2.117537	5.813775
ARHGAP4	204425_at	5.030068	4.954748	4.315204	4.702748	5.423958	5.881719	5.0068	5.276151	4.790795	4.787435	5.587633	5.428701	4.322918
TMED2	204426_at	4.074852	3.636204	5.382504	4.114262	3.772426	3.423361	6.71138	3.393475	6.816729	7.075538	3.614915	3.678704	8.349189
TMED2	204427_s_at	4.341529	4.485468	7.069322	5.229602	3.489498	2.939952	7.672014	3.663011	7.8044	7.861994	3.686331	3.555028	9.158313
LCAT	204428_s_at	10.19684	10.10617	10.315	10.33894	10.27857	9.961149	8.855593	8.451367	9.022133	8.419372	8.604171	8.492073	5.77421

SLC2A5	204429_s_at	2.058047	2.039821	2.014298	2.021389	2.034842	2.021844	2.031843	2.039638	2.024046	2.017163	2.022019	2.034387	2.088619
SLC2A5	204430_s_at	2.591454	2.876112	2.534871	2.444421	2.555694	2.526152	2.538374	2.547248	2.545332	2.521968	2.539223	2.54961	2.757843
TLE2	204431_at	3.142768	3.906825	3.485814	3.068348	3.194884	3.062307	3.072658	3.075494	2.979028	3.056935	3.067272	2.970971	4.590637
SOX12	204432_at	4.323333	4.339921	3.945028	3.511425	4.570326	4.330871	5.424471	6.514453	5.408823	5.214142	6.311303	6.512216	6.007979
SPATA2	204433_s_at	4.469519	4.379387	4.587384	5.075828	4.46405	4.684269	4.014993	4.164704	4.377967	4.459978	4.373963	4.335539	3.758738
SPATA2	204434_at	4.978105	5.039931	4.925307	5.048245	4.958464	4.935918	5.036093	5.350448	5.029528	4.94092	5.022184	5.23587	5.034293
NUPL1	204435_at	7.202772	7.244206	7.094839	7.326	6.930864	6.787533	8.227541	8.002266	8.199536	8.310591	7.777956	8.402758	7.464898
PLEKHQ1	204436_at	6.067661	6.136941	6.168973	6.133326	6.676508	7.113422	4.881836	5.382855	4.472588	4.47494	5.738344	5.393977	5.433461
FOLR1	204437_s_at	2.171378	2.151508	2.125197	2.134231	2.162925	2.167743	3.754864	2.167054	2.415794	2.762484	3.227896	4.288574	4.655608
MRC1	204438_at	9.1714	8.96987	9.786829	10.12331	9.420837	9.322909	8.959534	7.657681	8.642235	7.782018	7.574623	8.379914	7.324482
IFI44L	204439_at	3.265718	3.097074	6.539508	6.125349	3.145619	2.861078	2.722674	2.724547	2.680624	2.707029	2.601939	2.614758	3.856257
CD83	204440_at	5.063781	5.544969	6.494062	6.393672	6.895197	7.32319	4.892619	4.940334	4.787115	5.076372	4.986055	4.937086	7.372613
POLA2	204441_s_at	4.760122	4.95666	4.264535	4.683663	4.99359	5.088496	7.255988	5.982979	7.063726	7.53964	8.095588	7.941051	6.099697
LTBP4	204442_x_at	5.324523	5.442449	5.947744	5.718488	5.943589	6.19654	4.736676	4.742382	4.557314	4.707187	6.025974	4.785462	7.262068
ARSA	204443_at	4.676303	4.978794	6.082717	6.017158	4.720976	4.799321	4.118575	3.604589	2.918896	3.786489	3.670708	4.291537	2.075949
KIF11	204444_at	2.778619	2.738519	2.57441	2.611202	2.674795	2.796861	9.000222	8.666623	9.051852	8.936687	8.075716	8.555463	8.443368
ALOX5	204445_s_at	2.459796	2.432043	2.407864	2.416765	2.443209	2.424088	2.427688	2.438714	2.420317	2.41217	2.421889	2.432919	2.319045
ALOX5	204446_s_at	6.277288	5.778109	5.596822	5.435089	6.519968	6.419466	3.055705	5.149947	4.460202	4.490115	4.914128	4.961246	3.383509
ProSAPiP1	204447_at	6.999504	7.418751	6.185823	6.191715	6.998724	6.107742	6.492873	6.610384	6.451019	6.570535	7.031878	7.240563	6.182667
PDCL	204448_s_at	2.626223	2.705475	2.641598	2.500029	2.765126	2.799767	4.697538	5.149454	5.030932	5.053606	4.543708	5.164932	5.107424
PDCL	204449_at	7.619249	7.480602	7.07937	7.520695	7.811104	8.09219	7.366625	8.451266	7.808436	7.5097	8.894869	8.238099	8.517935
APOA1	204450_x_at	14.97849	14.91773	14.653	14.84325	15.14048	14.98746	14.76834	14.70632	14.7616	14.84038	14.71566	14.83706	10.37789
FZD1	204451_at	6.504823	6.323069	6.527061	6.17032	6.214898	6.123915	6.42929	5.728204	5.974048	5.660416	8.667802	5.811234	9.799723
FZD1	204452_s_at	2.020285	1.989206	1.97121	1.975745	1.989156	1.961773	1.98575	1.989172	1.977621	1.973412	2.158505	1.981269	2.702337
ZNF84	204453_at	6.975546	6.981235	6.366685	6.480273	7.207799	6.951354	6.715115	7.134832	6.934339	7.375821	6.996769	6.887758	7.634954
LDOC1	204454_at	2.415308	2.672206	2.76428	2.770578	2.698035	2.682176	5.774297	6.379791	5.676086	5.033446	5.740891	5.540205	6.689245
DST	204455_at	2.554626	2.498766	2.46976	3.022566	2.527372	2.475249	2.482212	2.487543	2.473165	2.45972	2.499653	2.510819	3.747616
GAS1	204456_s_at	2.533536	2.487733	2.45657	2.486414	2.519695	2.469504	2.496614	2.497638	2.482506	2.471089	2.479908	2.496746	7.322574
GAS1	204457_s_at	5.009357	5.181701	6.577972	6.336495	5.004218	4.992534	7.511445	5.828235	6.906528	6.315009	9.65742	6.193081	12.44298
LYPLA3	204458_at	6.747376	6.549373	5.434996	5.844037	6.939978	6.795118	4.522843	5.529035	4.475319	4.574385	6.033098	5.85221	4.6827
CSTF2	204459_at	2.897625	2.919842	2.870091	2.90884	2.643413	2.887051	5.821565	4.841883	5.002465	5.563755	5.425203	4.520738	4.514985
RAD1	204460_s_at	3.808852	3.766863	4.915506	4.294867	3.709753	3.597176	6.320717	5.399015	6.613886	6.523959	4.152741	5.231522	6.891602
RAD1	204461_x_at	8.363873	8.231453	7.623512	7.870715	8.085244	8.134767	8.259618	8.809319	8.785088	8.614915	8.887454	8.820337	8.738798
SLC16A2	204462_s_at	7.250705	7.365618	8.636499	8.411353	7.472752	7.076578	5.278262	5.358181	5.415405	4.593781	5.221028	4.920685	5.995871
EDNRA	204463_s_at	4.881778	4.683422	3.984004	4.069016	3.587278	3.779193	7.168917	5.325355	4.791267	5.116859	8.593838	6.871006	10.39325
EDNRA	204464_s_at	6.269909	5.948966	5.482545	5.347048	4.4394	4.626216	7.945991	6.490312	5.843726	6.107267	9.060807	7.472303	11.00086
INA	204465_s_at	2.134013	2.112257	2.087073	2.09652	2.125711	2.718066	2.103911	2.111334	2.106619	2.090231	2.076055	2.103868	9.951434
SNCA	204466_s_at	2.335841	2.316852	2.288666	2.279844	2.313653	2.296993	11.86114	11.45741	12.37785	12.11733	10.42513	11.11186	7.517572

SNCA	204467_s_at	2.652716	3.067976	2.853602	2.814157	3.086685	3.039304	10.50456	10.56342	10.93534	9.760512	9.252464	10.54945	6.71384
TIE1	204468_s_at	5.954619	5.583666	5.775759	5.488355	5.299389	4.592397	4.837514	4.772597	4.867738	5.320179	6.477148	5.005886	5.482504
PTPRZ1	204469_at	2.682711	2.649798	2.569964	2.581488	2.726455	2.599466	2.644674	2.625579	2.639152	2.611738	3.429642	2.658125	9.259797
CXCL1	204470_at	3.056398	3.436218	3.774788	3.424488	3.501587	4.029785	2.938804	3.241891	2.935283	2.393618	2.405814	2.958789	3.221744
GAP43	204471_at	3.19659	3.191872	3.102715	3.141811	3.306908	3.074562	3.136355	3.160935	3.160521	3.143889	3.491702	3.30155	10.44301
GEM	204472_at	5.10079	5.628695	6.914702	5.860648	4.893384	4.725587	3.094589	3.372555	2.928135	3.079445	3.358102	3.389709	6.658918
ZNF592	204473_s_at	4.866953	5.237137	4.923864	4.910311	4.930186	4.48906	5.797171	6.145574	6.02757	6.006471	5.209934	6.570816	3.851011
ZNF142	204474_at	4.6046	4.602835	4.698665	4.821324	5.11614	5.490595	5.355744	5.29146	5.1347	4.916607	5.323281	5.132429	5.178266
MMP1	204475_at	2.491276	2.453513	2.408071	2.43741	2.483679	2.422606	2.447744	2.457411	2.44262	2.428678	2.452907	2.478474	5.550869
PC	204476_s_at	9.343844	9.313787	9.187102	9.624256	10.2277	10.16894	8.440455	9.078682	8.687277	8.537477	8.694292	8.948396	4.413816
RABIF	204477_at	2.969663	2.910053	2.986025	3.160123	2.995943	3.501805	3.237773	2.960172	3.392018	3.071092	3.899765	4.009778	3.117293
RABIF	204478_s_at	5.067348	5.115093	5.511686	5.455978	5.317651	5.249513	5.72607	5.984226	5.781783	5.497974	5.876803	6.24662	5.50603
OSTF1	204479_at	6.419071	6.53679	6.919237	6.680879	6.439209	6.155077	6.745217	5.92967	6.465367	6.173238	6.052837	6.295319	5.566586
C9orf16	204480_s_at	3.86445	4.39508	4.877095	4.541222	4.003769	4.167178	5.040839	5.011905	5.143321	5.28801	5.225896	5.714268	6.728605
BRPF1	204481_at	5.322348	5.359538	5.053329	5.343854	5.378379	6.048855	5.356804	6.397381	6.21189	6.053008	5.548197	6.255736	5.24324
CLDN5	204482_at	8.008487	8.16731	7.409366	7.381002	7.904853	8.081088	7.940103	7.829912	7.699456	7.7109	8.270657	8.121657	7.814117
ENO3	204483_at	9.897913	10.02684	7.742041	8.326252	6.028609	6.576038	4.106831	5.114653	4.102855	4.913957	8.868671	4.588529	8.732332
PIK3C2B	204484_at	7.186216	7.330908	6.801015	6.806178	7.060564	7.341778	6.648803	7.007556	6.381929	6.333859	7.752923	7.160488	8.589346
TOM1L1	204485_s_at	9.048296	8.930014	9.416324	9.445777	8.777574	8.436521	6.977223	6.720851	7.098354	7.041875	5.71335	6.895373	5.394872
KCNQ1	204486_at	3.076974	2.981456	2.910035	2.95538	3.12086	3.131518	2.974807	3.000977	2.954848	2.940358	3.00333	3.043443	2.733097
KCNQ1	204487_s_at	4.09039	3.986616	4.282907	4.288083	4.53848	4.582925	4.001175	4.298687	4.29016	4.073228	4.519068	4.923203	3.965206
DOLK	204488_at	6.560968	6.665994	6.392906	6.757704	6.269451	6.093311	6.252773	6.80975	6.568808	6.692527	6.27743	6.514188	6.206469
CD44	204489_s_at	4.375521	4.18023	4.88658	4.699809	4.865056	4.717441	4.944137	5.165457	4.938941	5.024559	4.693593	4.99001	7.382896
CD44	204490_s_at	3.993675	3.889062	3.636028	3.142636	4.092758	4.041762	3.838395	4.091077	4.761958	4.420651	3.795942	4.164958	4.833059
PDE4D	204491_at	2.996772	3.107301	3.185625	3.055587	3.115064	3.356369	3.716886	3.07982	3.533653	3.906943	3.415302	3.136032	6.82753
ARHGAP11/	204492_at	2.760821	2.736739	2.669782	2.712053	2.756943	2.689756	5.002788	4.024988	4.110734	5.486172	3.929202	4.176557	2.994561
BID	204493_at	6.621303	6.608235	6.383636	6.357311	5.81277	6.079219	5.481288	5.842446	5.861204	6.058595	5.394279	5.75892	5.561774
C15orf39	204494_s_at	3.489729	3.40122	3.376996	3.31154	3.033516	3.471076	4.47194	4.214559	4.085685	3.792362	3.670618	4.914423	4.176802
C15orf39	204495_s_at	4.429227	4.741568	4.456704	4.451743	4.95169	4.787681	4.801022	5.785843	5.115373	5.554949	6.635609	6.026303	4.722222
STRN3	204496_at	7.976854	7.912809	8.351378	8.365373	7.59568	6.670268	9.048243	7.883457	9.153062	9.275185	7.691736	7.944247	8.886663
ADCY9	204497_at	8.279234	8.243042	8.131481	8.221706	8.117771	8.004355	7.158779	7.212651	7.395442	7.223012	7.517731	7.241678	6.609933
ADCY9	204498_s_at	2.903809	2.8965	2.87757	2.972264	3.155967	3.981974	2.88863	2.896942	2.879214	2.876037	2.840332	2.883304	2.805232
AGTPBP1	204499_at	2.508943	3.088402	3.070235	3.073664	3.081725	2.639913	3.072705	3.627806	3.0808	3.123784	3.231273	3.802255	5.307674
AGTPBP1	204500_s_at	6.11817	5.845588	5.935132	5.928604	6.115114	6.225137	7.463835	7.762279	7.713151	7.819676	7.217289	7.420466	8.110473
NOV	204501_at	2.580156	2.630899	2.530999	2.540857	2.579385	2.510537	2.554484	2.556419	2.540747	2.536831	2.582764	2.556091	7.817464
SAMHD1	204502_at	6.024564	5.93716	6.099886	6.037559	7.048767	6.980163	5.554478	5.744305	5.30772	5.300618	5.296911	5.623567	5.42373
EVPL	204503_at	2.57988	2.53612	2.47471	2.520912	2.554904	2.619802	2.532081	2.570026	2.527104	2.510016	2.519078	2.53465	2.372421
HIRIP3	204504_s_at	6.489256	6.601347	6.396791	6.384372	7.110629	6.853013	7.221835	7.534919	7.17424	7.522136	7.298068	7.645665	6.455301

EPB49	204505_s_at	4.854941	4.640835	4.823456	4.955377	4.008749	4.346258	10.38235	9.208419	10.4627	10.56922	8.43039	9.399144	4.360372
PPP3R1	204506_at	3.614721	3.868048	3.557425	3.558601	3.597072	3.560969	3.578508	3.590207	3.658241	4.650739	3.522475	3.573789	4.787999
PPP3R1	204507_s_at	4.504172	4.473485	4.173751	4.521871	4.325455	3.917904	5.659561	5.365464	4.970669	6.098178	4.35193	4.864396	4.367308
CA12	204508_s_at	2.991939	3.253411	2.915016	2.936376	2.970305	2.993556	5.42039	4.655344	4.021957	4.314563	4.79981	5.214024	4.542571
CA12	204509_at	2.483511	2.460465	2.420646	2.448397	2.470564	2.453806	2.794338	2.469556	2.457252	2.441051	2.455046	2.472601	2.394076
CDC7	204510_at	3.701029	3.654503	2.653858	1.789082	3.090906	3.082362	8.467366	8.425724	8.370397	8.493693	8.33365	8.385179	6.671039
FARP2	204511_at	5.469277	5.512103	4.92388	5.306825	4.897693	4.474503	3.346386	3.558255	3.175237	3.769583	3.057288	3.577604	3.426864
HIVEP1	204512_at	7.838857	8.031972	8.759811	7.899875	7.358543	7.06714	6.614954	6.419689	6.449717	6.676839	6.460331	6.238166	8.287343
ELMO1	204513_s_at	5.147172	4.775111	6.308968	6.070594	5.284641	5.148016	4.197605	4.397631	4.21768	4.753118	4.108107	4.196064	5.69026
DPH2	204514_at	5.648791	5.640745	5.52241	5.552941	5.632432	5.631539	6.038902	6.054643	5.856505	5.997288	5.646929	6.384255	5.65799
HSD3B1	204515_at	2.508853	2.482629	2.445351	2.478638	2.504273	2.453803	2.477911	2.489905	2.632459	2.45808	2.47189	2.484236	2.418189
ATXN7	204516_at	8.703748	8.844591	9.017098	8.835166	8.531739	7.868186	8.212025	7.510389	8.446588	8.451005	7.549478	7.650097	8.225291
PPIC	204517_at	9.0622	8.94365	9.406246	9.264023	8.295494	8.273	8.581147	8.4184	8.672654	8.839823	8.884984	8.45225	9.607283
PPIC	204518_s_at	5.420009	5.907289	6.145167	6.01555	4.664451	3.806694	5.445098	5.127214	5.530142	5.166084	5.158185	5.111736	7.183582
PLLP	204519_s_at	4.577504	4.559383	4.57312	4.981257	6.274051	6.098119	4.439849	4.634136	3.468652	4.176193	3.298069	3.464461	2.487829
BRD1	204520_x_at	7.226015	7.283121	7.173806	6.909018	6.920189	6.723112	7.114837	6.638803	6.939584	6.992331	6.963708	6.975682	7.632761
C12orf24	204521_at	6.054268	5.958542	4.654679	4.483618	5.029919	5.803389	6.146881	6.379351	6.403739	6.268924	6.907286	6.845445	6.595859
DOM3Z	204522_at	4.13489	4.102257	4.095135	4.034048	4.742446	4.388019	4.143089	4.035003	4.575958	4.09425	4.088906	4.110661	4.231885
ZNF140	204523_at	6.706591	6.648738	6.57888	6.685506	6.252156	6.226685	6.466367	6.215865	6.477281	6.438198	6.108869	5.895956	7.552637
PDPK1	204524_at	6.517426	6.556291	5.624534	6.111112	6.110975	6.46639	5.757098	5.925595	5.674955	5.287442	5.765101	5.960149	4.928093
PHF14	204525_at	2.842604	2.893979	3.127147	2.764971	2.999489	2.729761	2.686721	2.806744	2.785556	2.757343	2.804905	2.802863	4.179914
TBC1D8	204526_s_at	7.351781	7.319214	7.615376	7.786612	7.508799	6.445793	5.553652	4.917133	5.054967	5.079009	4.868888	4.962308	5.002074
MYO5A	204527_at	2.960281	2.914686	2.974857	2.942596	3.227441	3.421387	2.894671	2.83314	2.912124	2.870356	2.913374	2.922603	3.957351
NAP1L1	204528_s_at	9.661614	9.695333	9.209459	9.483699	10.05371	10.32732	10.9521	11.45779	10.9725	10.95056	11.90314	11.45193	12.08457
TOX	204529_s_at	3.416057	3.488655	3.366389	3.312058	3.470103	3.819432	3.380464	3.381915	3.358877	3.37476	3.91927	3.437466	8.500494
TOX	204530_s_at	2.842629	2.811819	2.721542	2.777578	2.825823	2.793763	2.798615	2.812279	2.796569	2.782194	2.797681	2.805185	3.214973
BRCA1	204531_s_at	5.351211	5.393439	6.45038	6.835208	4.340774	4.758661	7.749049	7.942598	7.747726	8.121553	7.515071	7.970626	5.73298
UGT1A4	204532_x_at	12.22317	12.1301	12.61962	12.60912	11.41869	10.91384	2.937624	4.219842	2.341062	2.335394	2.634663	2.848458	2.852616
CXCL10	204533_at	7.323252	6.786967	3.221518	2.955081	3.389774	2.940891	2.924176	2.951529	2.922034	2.892195	2.839409	2.924604	2.643418
VTN	204534_at	13.80055	13.78746	13.62494	13.69345	13.61784	13.51044	13.38602	13.03608	13.33323	13.41662	12.87588	13.05824	7.324382
REST	204535_s_at	3.143644	3.050797	3.07627	3.147189	4.341274	4.257164	3.065687	3.113277	3.037463	3.048186	3.385452	3.634862	3.008883
GABRE	204537_s_at	2.240406	2.091424	2.072393	2.076002	2.09267	2.07389	2.081955	2.088689	2.07554	2.067428	2.077159	2.083675	3.904736
NPIP	204538_x_at	9.238812	9.314252	9.395342	9.230289	8.679081	8.646596	9.290492	8.766255	9.113756	8.686226	9.133835	8.732723	10.24894
CELSR1	204539_s_at	2.305859	2.263939	2.238792	2.243462	2.274205	2.246108	2.255188	2.261155	2.248365	2.240607	2.246211	2.253875	2.150623
EEF1A2	204540_at	2.938575	2.950103	2.731248	2.83695	2.985863	2.774684	2.94444	2.932953	2.924502	2.910429	5.871801	2.932193	8.299482
SEC14L2	204541_at	7.558719	7.454356	8.040262	8.214782	8.057731	7.767612	3.173975	3.136727	3.187474	3.186744	3.011281	3.214706	2.48736
ST6GALNAC	204542_at	2.264089	2.254538	2.373944	2.241515	2.261263	3.183181	2.244643	2.240488	2.225169	2.211427	2.237339	2.234465	3.00529
RAPGEF1	204543_at	2.275695	2.257469	2.202769	2.245644	2.257416	2.239132	2.251868	2.254699	2.270153	2.294496	2.236444	2.249539	2.226368

HPS5	204544_at	7.598707	7.317346	7.838239	7.668108	7.358809	6.874808	7.403536	6.611148	7.040456	7.087465	6.860022	6.605948	6.930474
PEX6	204545_at	6.198413	6.280579	4.552382	5.338063	6.32577	5.84155	2.062737	5.446186	2.552893	5.092315	5.7672	5.328403	2.035796
KIAA0513	204546_at	3.849762	4.220883	4.15373	4.073553	3.962028	4.202606	4.214964	4.826505	4.248832	4.506846	4.821376	4.827074	4.978148
RAB40B	204547_at	8.613682	8.519494	7.544876	7.903412	8.098409	7.868675	6.84211	6.091589	6.457747	6.653602	7.094138	6.430078	8.484358
STAR	204548_at	2.456821	2.420618	2.379583	2.410313	2.439125	2.403924	2.518148	2.430996	3.068718	2.558106	2.416202	2.425369	10.66024
IKBKE	204549_at	2.096491	2.084701	1.988639	2.028197	2.089777	2.723131	2.083882	2.385253	2.02935	2.060389	2.535505	2.247937	2.086934
GSTM1	204550_x_at	8.561631	8.589685	4.545745	4.812911	8.954697	8.467713	5.328778	7.058743	6.400046	4.873753	6.941135	7.136198	6.750554
AHSG	204551_s_at	14.60305	14.63999	14.31534	14.35666	14.02462	13.5538	14.96728	14.89399	15.2307	15.40661	14.7136	14.74312	10.41205
INPP4A	204553_x_at	2.167326	2.143903	2.126419	2.120989	2.136173	2.129923	2.143677	2.143964	2.135204	2.131997	2.12653	2.135571	2.70453
PPP1R3D	204554_at	3.064766	2.975487	3.143248	3.051107	3.52173	3.468166	3.035565	2.983763	3.633386	3.057204	3.029681	3.028131	3.690999
PPP1R3D	204555_s_at	2.795919	2.783477	2.761077	2.764609	3.123899	3.128548	2.770461	2.784972	2.765986	2.82057	3.00657	3.726256	2.724247
DZIP1	204556_s_at	3.536102	3.566788	3.325128	3.466699	3.566483	3.537837	4.568964	3.897659	3.553537	4.003886	5.477803	4.582083	7.418976
DZIP1	204557_s_at	3.672103	3.613168	3.32909	3.474662	3.228408	3.328167	5.525098	3.907592	4.30955	3.913245	5.446208	4.662399	8.468552
RAD54L	204558_at	2.497387	2.483418	2.453003	2.46229	2.486402	2.471661	6.097526	6.316371	5.83368	6.073143	6.037697	6.103851	4.10982
LSM7	204559_s_at	9.142688	9.167221	9.114876	9.342177	9.204528	9.229286	10.41642	10.50809	10.57753	10.51632	10.84457	10.70927	10.81369
FKBP5	204560_at	2.909148	2.888015	3.046019	2.962478	2.901198	2.878126	2.876429	2.887317	2.882643	2.880005	2.873022	2.890163	3.849409
APOC2	204561_x_at	14.10717	14.03571	14.01499	14.09025	14.01078	13.89795	13.47637	13.32899	13.46401	13.31946	12.81655	13.29676	7.852228
IRF4	204562_at	2.029805	2.021715	1.997777	1.9747	2.021975	2.438523	2.013537	2.02006	2.005923	2.020584	2.00006	2.013604	1.942517
SELL	204563_at	2.815014	2.774368	3.804627	2.774791	2.784617	2.778581	2.731941	2.772836	2.702762	3.046235	2.771004	2.77865	2.266569
PCGF3	204564_at	3.098109	3.082393	3.190748	3.201661	3.17033	3.058615	2.89841	2.794192	3.066513	2.889308	3.179675	2.867842	3.607079
THEM2	204565_at	8.866196	8.92238	9.894191	9.919065	9.149972	8.702875	9.230806	8.173839	9.276289	9.133915	8.235492	8.630239	6.610328
PPM1D	204566_at	6.179075	5.710889	6.676989	6.475573	5.446276	5.126008	7.247858	6.560878	7.568785	7.920202	6.366958	6.722164	7.885885
ABCG1	204567_s_at	9.097257	8.744462	6.990126	7.157346	5.336938	4.679394	5.668458	5.962466	5.888865	5.983834	5.880726	4.720718	5.346403
KIAA0831	204568_at	6.843424	6.806059	6.724923	6.821926	6.901887	6.195221	8.436136	7.75916	8.334409	8.632844	7.656737	7.870272	7.423245
ICK	204569_at	6.425468	6.119078	5.88427	6.119696	6.031941	5.602697	5.291538	4.72052	5.788896	5.682072	5.555886	5.24389	7.099052
COX7A1	204570_at	6.096564	6.044017	5.737303	6.118636	7.308992	7.514463	3.354334	3.451365	3.395764	3.338053	8.015017	3.481162	8.701344
PIN4	204571_x_at	9.06276	9.07961	9.206714	9.455942	9.642529	9.778233	9.447513	9.770904	9.209629	9.346489	9.695505	9.804102	8.420782
hCG_17897	204572_s_at	2.618406	2.615345	2.65095	2.852972	2.592868	2.834576	4.161928	2.60931	3.238819	2.797809	2.582497	2.724431	3.777353
CROT	204573_at	9.971147	10.00849	9.440757	9.819566	9.918719	9.877033	9.908994	9.883844	9.372488	9.538473	9.593351	9.514592	7.554972
MMP19	204574_s_at	2.712562	2.682668	2.654772	2.676541	2.859414	3.387054	2.677934	2.741179	2.680381	2.670363	2.682533	2.685517	2.030954
MMP19	204575_s_at	3.396063	3.241572	5.730924	4.367607	3.626314	3.620956	3.617809	3.611753	3.358149	3.584143	3.543794	3.373191	3.542855
CLUAP1	204576_s_at	5.269973	5.380276	5.243102	5.216961	4.827114	4.769084	4.737982	5.020216	5.454748	4.758316	5.730753	5.839637	5.945346
CLUAP1	204577_s_at	3.227771	3.163478	3.111065	3.092129	3.478235	3.649114	3.186881	3.180338	3.181818	3.058089	3.173056	3.194636	4.105121
HISPPD2A	204578_at	4.447681	4.112106	4.241738	4.174548	4.367158	4.482348	4.599482	4.954849	4.185552	4.215289	4.242591	4.238108	3.628536
FGFR4	204579_at	8.998278	9.201777	8.656332	9.009586	9.274589	8.940231	8.007016	8.643304	8.023458	8.017583	8.880604	8.9282	7.006044
MMP12	204580_at	2.344844	2.319881	2.294019	2.30255	2.327259	2.290755	2.31225	2.31868	2.306911	2.299718	2.302804	2.313906	2.304072
CD22	204581_at	2.686872	2.631066	2.581893	2.632099	2.649592	2.629028	2.630261	2.621264	2.62763	2.572007	2.645445	2.675286	2.564024
KLK3	204582_s_at	2.568101	2.530118	2.479122	2.515114	2.553447	2.509103	2.524251	2.535126	2.519513	2.500832	2.515609	2.534502	2.201232

KLK3	204583_x_at	2.721594	2.68845	2.665737	2.662859	2.687305	2.669167	2.677272	2.681329	2.672008	2.664758	2.670286	2.699382	2.462603
L1CAM	204584_at	3.426954	3.252805	3.173478	3.288293	3.535291	4.805446	3.277529	3.404054	3.319217	3.369803	4.287855	3.454857	7.295785
L1CAM	204585_s_at	2.021991	2.009192	1.98878	1.992452	2.009315	1.991664	2.004375	2.002488	2.002537	1.990633	1.990459	1.995093	1.977312
BSN	204586_at	2.197769	2.189627	2.122902	2.176676	2.18992	2.179076	2.186278	2.219792	2.183383	2.176412	2.173469	2.182716	2.479746
SLC25A14	204587_at	5.879561	5.860715	5.889065	5.904372	6.018557	6.308703	5.915101	6.286612	6.236234	5.833433	6.639529	6.27584	6.598419
SLC7A7	204588_s_at	5.871477	5.921269	6.332709	6.273237	6.466652	6.4947	6.21554	6.273347	6.270512	6.056204	6.269237	6.666677	5.600836
NUAK1	204589_at	8.614151	8.693454	8.187399	8.537578	8.78297	9.402357	6.82147	7.960792	6.697274	7.037664	9.192455	8.62146	8.661725
VPS33A	204590_x_at	3.894263	3.829091	3.765334	3.834105	3.860761	3.857733	3.834254	4.600053	3.830937	4.0005	4.039532	3.960535	3.331507
CHL1	204591_at	2.847316	2.778918	3.66694	3.635035	2.913223	2.902669	2.743585	2.749694	2.764342	2.718779	2.805891	2.812572	8.822211
DLG4	204592_at	2.122299	2.111293	2.092869	2.094216	2.226617	2.162412	2.061182	2.104041	2.095146	2.085366	2.094226	2.100949	2.138747
SMCR7L	204593_s_at	6.562705	6.512816	6.490944	6.624122	6.793063	6.679862	6.850265	6.428629	6.647367	6.72468	6.447451	6.803894	5.913824
SMCR7L	204594_s_at	7.08876	7.084513	7.017335	7.14113	7.230858	6.828613	7.516178	7.711656	7.507487	7.444875	7.284232	7.336626	6.354837
STC1	204595_s_at	2.008565	1.994554	1.974481	1.978468	1.993692	1.981023	1.985513	1.992394	1.980269	1.972607	1.98253	1.989057	4.966451
STC1	204596_s_at	2.205119	2.166915	2.147492	2.147301	2.1723	2.149401	2.157143	2.163322	2.151553	2.13883	2.144147	2.156686	2.098849
STC1	204597_x_at	1.969691	1.95803	1.938972	1.94315	1.957254	1.951078	1.952637	1.950403	1.942526	1.936513	2.359964	1.95199	4.426022
UBOX5	204598_at	3.766948	3.723527	3.728476	3.694823	3.764609	3.914204	3.752828	5.108085	4.038978	3.7397	4.459068	4.962329	4.071702
MRPL28	204599_s_at	7.174859	7.227203	6.934431	6.983776	7.359488	7.415147	8.199755	8.369053	8.249472	7.591882	8.547691	8.545203	6.945595
EPHB3	204600_at	4.535776	5.061531	4.358642	4.40171	4.608177	4.934774	4.692584	4.670051	4.65448	4.622361	6.232702	4.789116	6.505384
N4BP1	204601_at	3.473831	3.469657	3.450338	3.37655	3.42243	3.371202	3.64073	3.371997	3.447765	3.455597	3.374196	3.384633	3.990799
DKK1	204602_at	2.635111	2.609105	2.595269	2.606472	2.619309	2.603177	2.625126	3.159679	3.163744	2.630883	2.620438	2.899029	8.8101
EXO1	204603_at	2.71549	2.694494	2.628281	2.640649	2.68683	2.641132	6.324055	6.068988	6.612766	6.659572	5.001451	5.748372	3.95999
PFTK1	204604_at	8.328559	8.388512	8.525143	8.733068	8.362992	8.186941	8.224884	9.042232	8.810631	8.809792	8.832964	8.867739	8.97293
CGRRF1	204605_at	6.014955	6.0852	6.812629	6.628622	5.84461	5.897907	6.652271	6.396038	6.82701	6.618246	6.355512	6.609232	6.802426
CCL21	204606_at	6.239059	7.36917	5.066933	5.178306	6.091657	6.634075	3.524333	3.599166	3.518783	3.511538	4.380632	3.54032	5.421486
HMGCS2	204607_at	12.87376	12.78766	13.50838	13.48078	13.05638	12.66414	11.10267	10.37242	11.4284	11.1398	9.559296	10.06255	4.110976
ASL	204608_at	10.2982	10.26727	11.27051	11.48475	11.00084	11.00373	9.633963	9.562081	9.260664	9.534633	9.707249	10.09796	5.838751
CCDC85B	204609_at	2.195021	2.166383	2.144722	2.148176	2.194935	2.149219	2.160845	2.167222	2.151609	2.144456	2.145284	2.171814	2.068683
CCDC85B	204610_s_at	7.088949	7.155588	6.38246	6.586707	8.139587	8.585514	6.411298	7.341258	6.530872	6.630768	7.470728	7.744706	6.479942
PPP2R5B	204611_s_at	5.055278	4.91618	4.859509	5.017066	4.953242	5.035691	5.043338	5.042996	4.930528	5.025711	5.029213	5.037486	5.043648
PKIA	204612_at	2.765352	2.729865	2.645098	2.63168	2.655943	2.676178	5.414601	5.208731	5.239624	5.303629	6.039629	4.893282	9.910008
PLCG2	204613_at	9.165048	9.198272	8.554614	8.93038	8.512322	8.310118	6.018482	6.077529	5.851284	5.851274	5.307312	5.504408	4.167688
SERPINB2	204614_at	2.610892	2.579449	2.514358	2.558382	2.595114	2.556203	2.571287	2.582454	2.567099	2.55656	2.566064	2.576052	3.285919
IDI1	204615_x_at	10.13483	9.99976	9.806706	9.784789	11.43204	11.02527	12.61919	11.90707	12.83022	12.51222	11.72321	12.25353	10.28776
UCHL3	204616_at	9.282142	9.143927	9.436542	9.389667	8.597043	7.825528	9.890015	9.204846	9.916701	9.882827	9.100201	9.359836	8.874521
ACD	204617_s_at	5.466939	5.523657	5.063098	5.257753	5.41416	5.595913	5.809564	5.763216	5.675432	6.153828	6.253237	6.309504	6.121979
GABPB2	204618_s_at	5.69283	5.428698	6.325056	6.106723	5.609219	5.614655	7.199415	5.89024	7.23305	7.165475	5.994341	6.557629	7.13746
VCAN	204619_s_at	3.238908	3.190183	3.268908	3.281176	3.237039	3.278243	3.289716	3.163847	3.070081	3.125871	8.166028	3.506417	10.09228
VCAN	204620_s_at	4.065078	4.283695	5.691297	4.831498	5.159655	5.094623	5.38329	3.898212	3.859366	3.341848	9.70553	5.111871	13.11867

NR4A2	204621_s_at	7.157135	7.09224	7.612107	7.887653	8.303783	7.663005	3.18278	3.098366	3.422432	3.083614	3.426596	3.462354	4.316098
NR4A2	204622_x_at	5.501107	5.856081	7.408832	7.555851	6.513216	6.622137	2.762627	2.692729	2.680232	2.557559	2.66263	2.699695	4.174425
TFF3	204623_at	2.398841	2.420577	2.436799	2.406072	2.680958	2.431783	2.416879	2.418226	2.408961	2.416115	3.197629	2.631571	2.42124
ATP7B	204624_at	7.516971	7.244103	7.655437	7.640375	7.117484	6.300586	9.192494	7.9562	9.173515	8.916875	7.716553	8.008479	5.622156
ITGB3	204625_s_at	1.909721	1.900157	1.881752	1.885018	1.908537	1.884763	1.896487	1.897195	1.887823	1.883641	1.879388	1.891789	1.847916
ITGB3	204626_s_at	1.94791	1.936438	1.916318	1.921233	1.935401	1.919494	1.932398	1.933847	1.921728	1.917886	1.914849	1.926505	1.868891
ITGB3	204627_s_at	2.349941	2.434017	2.300314	2.314099	2.326231	2.366266	5.940008	4.085975	5.783652	5.065086	3.007698	4.750474	2.838064
ITGB3	204628_s_at	3.645025	3.949431	3.822372	3.578972	3.987163	4.712784	5.574662	4.504113	4.372066	3.9362	4.312574	5.507673	3.678646
PARVB	204629_at	3.261943	3.248093	2.931573	3.146741	3.237375	3.396193	5.909023	5.810686	6.245259	5.756196	5.559586	6.484795	2.732633
GOSR1	204630_s_at	9.400913	9.512132	9.067476	9.386613	9.663294	10.02327	8.752352	9.418338	8.998623	8.695454	9.635628	9.557323	8.589738
MYH2	204631_at	2.628205	2.580444	2.494658	2.565321	2.614674	2.563266	2.574881	2.577386	2.572611	2.552261	5.405034	2.584359	3.558054
RPS6KA4	204632_at	5.288364	5.603432	4.937118	4.971688	5.345418	5.130354	4.819128	5.420096	4.720136	4.899617	4.974718	5.456037	4.40476
RPS6KA5	204633_s_at	4.981311	5.239282	5.814316	5.409839	5.256239	5.301354	4.669577	4.66417	5.052403	4.832876	5.691646	5.006246	7.125686
NEK4	204634_at	7.65467	7.547535	7.757691	7.483852	7.375747	6.872828	8.689879	7.887963	8.538262	8.714552	6.981856	7.801921	7.68099
RPS6KA5	204635_at	3.665467	3.606126	4.162806	4.167527	3.606711	3.593978	3.619596	3.621197	3.627763	3.660872	4.381999	3.62403	5.197801
COL17A1	204636_at	1.992077	1.976016	1.954082	1.958059	1.976294	1.957676	1.967969	1.975381	1.960538	1.953447	1.959054	1.968357	1.93703
CGA	204637_at	1.917805	1.90735	1.886449	1.889575	1.905965	1.887985	1.901143	1.903131	8.089221	2.325742	1.89311	1.895828	6.476306
ACP5	204638_at	6.836251	6.139258	6.078636	6.389457	5.283945	6.326822	6.511124	6.35568	5.937743	5.842111	6.576367	6.782187	2.243125
ADA	204639_at	4.450427	4.524103	4.148752	3.737915	4.183589	5.400901	4.672331	5.09343	4.604662	4.67879	5.343205	5.437533	5.05661
SPOP	204640_s_at	8.57032	8.727945	8.379219	8.362908	8.869018	8.693205	8.371359	8.755845	8.4417	8.607157	8.875041	8.711398	8.629946
NEK2	204641_at	3.78942	4.736735	2.954369	2.965644	3.156759	3.027742	9.420338	9.789847	9.671564	9.876815	9.022494	9.608373	7.182428
EDG1	204642_at	6.15889	6.036676	5.946834	5.862946	5.900159	5.917044	4.947682	4.510334	4.861051	4.176014	4.739901	4.295116	5.643647
ENOX2	204643_s_at	5.587701	5.639185	5.33888	5.47966	5.602077	5.565184	5.804246	5.783615	5.666984	5.709166	5.938254	5.728299	5.580901
ENOX2	204644_at	2.514664	2.499074	2.464603	2.473409	2.541502	2.478139	2.480428	2.481017	2.469618	2.473288	2.467879	2.476025	2.3214
CCNT2	204645_at	7.195448	7.020332	7.272653	6.893904	6.70407	5.80785	7.293135	5.920549	7.396205	7.512773	6.51223	6.640869	8.408873
DPYD	204646_at	10.50559	10.43818	10.84601	10.75187	9.919288	9.580718	9.403865	9.108356	9.340061	9.211068	8.195365	8.578374	6.444209
HOMER3	204647_at	5.304332	5.412081	4.71953	4.98467	5.034013	5.353941	4.68272	5.066066	4.652199	4.607219	6.204094	5.476843	6.134399
NPR1	204648_at	4.276744	4.729092	4.954046	5.268539	4.595065	4.842447	4.817914	4.932315	5.012523	4.782164	4.91544	5.169773	4.263302
TROAP	204649_at	2.602049	2.613859	2.448403	2.500983	2.551921	2.467699	6.588324	5.851083	7.306127	7.299442	3.9691	5.613093	5.762221
APBB3	204650_s_at	5.082785	4.54964	4.033737	3.791793	4.140585	3.82411	3.44882	2.921691	2.136662	2.403286	3.606252	3.529411	3.77149
NRF1	204651_at	4.886637	5.207631	4.209321	4.385266	5.22996	5.797116	6.401062	6.89316	6.448274	6.756877	6.468986	6.98706	5.633588
NRF1	204652_s_at	2.71583	2.532638	2.683388	2.687139	2.786507	3.457837	3.116259	3.014419	3.677195	3.068788	2.891338	3.629634	3.206051
TFAP2A	204653_at	2.188844	2.165079	2.132416	2.145028	2.171364	2.148956	2.157662	2.164563	2.151975	2.144163	2.15222	2.161433	6.693752
TFAP2A	204654_s_at	3.267719	3.210067	3.282975	3.189403	3.276172	3.069653	3.272368	3.227606	3.218433	3.174575	3.279975	3.419701	3.696863
CCL5	204655_at	7.081131	6.75466	5.805391	5.919268	7.36798	8.503072	5.072005	5.52057	4.544448	4.467495	5.011679	5.991801	3.434381
SHB	204656_at	6.083797	6.316311	5.850548	5.866433	5.874544	5.772717	3.307565	4.139817	3.926529	4.267262	4.518179	3.745117	3.80754
SHB	204657_s_at	5.425017	5.43024	4.98531	5.059291	5.021912	4.921025	3.025547	4.150239	2.87042	2.946617	3.591688	4.231914	2.966495
TRA2A	204658_at	7.412128	7.34717	7.2605	6.827016	7.016051	6.318825	8.617573	7.297137	8.749799	8.617355	7.346408	7.112009	9.113875

GFER	204659_s_at	6.336959	6.169459	6.216024	6.599028	6.998195	6.843273	5.22425	6.880135	6.33746	6.280591	6.791452	7.260019	3.344224
GFER	204660_at	4.217245	4.135504	3.853539	4.116804	4.254671	4.314052	4.125861	4.327679	3.817123	4.109938	4.133789	4.616567	2.991238
CD52	204661_at	5.202607	4.57504	5.253756	4.920165	5.746579	5.705325	4.247251	4.096425	4.160593	5.054192	3.812267	4.52671	3.72502
CP110	204662_at	6.39559	6.412342	5.41701	5.360023	5.761443	5.666881	7.70538	8.024582	7.574501	8.012569	7.062207	7.595246	7.873588
ME3	204663_at	4.293205	4.315616	4.321484	4.878216	4.989426	5.086826	3.8625	4.039781	4.105779	4.194173	4.280806	4.307326	5.986362
ALPP	204664_at	2.372726	2.350575	2.327514	2.331128	2.355869	2.333185	2.343606	2.350155	2.336217	2.329816	2.332783	2.344747	2.275434
SIKE	204665_at	2.084879	2.066445	2.040733	2.050015	2.063168	2.047714	2.0599	2.062161	2.051062	2.046611	2.047686	2.054522	1.981207
SIKE	204666_s_at	3.185027	3.271224	3.131368	3.075566	3.171261	3.134364	3.14562	3.092556	3.122733	3.052931	3.095835	3.135442	3.660575
FOXA1	204667_at	4.159754	3.802818	4.113871	3.174886	3.401706	3.119207	3.187081	2.934279	3.081889	3.053658	2.823054	2.871217	2.668509
RNF24	204668_at	2.019687	2.003479	1.980635	1.984478	2.00401	1.984656	1.997677	2.035175	2.013203	1.990089	1.987073	1.996108	2.327217
RNF24	204669_s_at	2.154608	2.170816	2.103119	2.106943	2.194085	2.140128	3.032297	2.458643	2.196273	2.741903	4.211012	3.180832	3.894729
HLA-DRB1	204670_x_at	7.79433	7.836293	9.169929	9.084805	9.433172	9.679249	3.965549	4.563001	4.904223	4.544701	4.1049	4.309878	4.183327
ANKRD6	204671_s_at	6.105387	6.245874	5.233312	5.558703	6.269393	6.209555	4.726884	5.059926	4.314957	4.113178	5.576103	4.626638	6.559449
ANKRD6	204672_s_at	5.507448	4.818577	5.763158	5.00756	4.402581	4.456398	4.649096	3.818159	4.381021	4.458286	4.261996	3.857631	6.586137
MUC2	204673_at	2.254497	2.242793	2.220835	2.223619	2.270649	2.229154	2.235253	2.411402	2.227035	2.222389	2.220059	2.236874	2.131248
LRMP	204674_at	2.762123	2.659307	3.048628	3.014578	2.499596	3.027012	4.681731	4.006382	5.695955	4.96691	3.134212	3.4427	2.671584
SRD5A1	204675_at	10.03853	9.991315	10.74703	10.83758	11.06961	10.98124	4.867267	6.17376	5.426012	5.770268	6.309664	6.319615	6.788327
TMEM186	204676_at	6.383917	6.901589	6.245538	6.286831	7.122472	7.053657	6.267085	6.537721	6.792943	6.144924	6.718351	6.731865	5.920298
CDH5	204677_at	5.450384	5.430391	6.243823	6.152734	4.534428	4.179651	5.817332	4.715562	6.117578	5.823701	5.177624	5.283122	6.779209
KCNK1	204678_s_at	4.7258	3.929979	4.648186	4.598748	3.322677	3.988815	2.746583	2.738806	2.742333	2.717703	2.743873	2.839617	3.325295
KCNK1	204679_at	8.954227	8.757347	8.290162	8.371656	7.712769	7.47179	5.952876	6.266024	4.520619	5.691206	6.22253	6.294347	7.556363
RAPGEF5	204680_s_at	2.39468	2.38579	2.388862	2.4514	2.617673	2.373389	2.337871	2.37297	2.33999	2.322856	2.36659	2.367381	2.189671
RAPGEF5	204681_s_at	7.068072	7.066295	7.377603	7.065907	6.530676	6.621444	5.680133	5.588806	5.538452	6.300739	5.527604	5.387369	6.501705
LTBP2	204682_at	2.571522	3.166314	3.208461	3.377086	2.998705	3.991483	2.608651	2.939324	2.480215	2.76234	3.60618	2.933579	4.282107
ICAM2	204683_at	3.884983	4.787261	5.002063	5.045819	4.654442	4.775891	5.466143	5.463239	5.533792	4.883975	4.789969	5.796903	4.405498
NPTX1	204684_at	2.808193	2.789849	2.743774	2.757328	2.781344	2.784749	2.764666	2.775963	2.7607	2.729054	2.760258	2.769836	4.652771
ATP2B2	204685_s_at	8.082084	8.085675	8.189451	8.206592	8.264957	7.855586	8.041877	6.892584	7.895936	7.911481	6.779229	7.189867	4.374849
IRS1	204686_at	10.77268	10.68727	11.05348	10.83834	10.71261	10.29615	8.124285	8.338579	8.001679	7.925681	8.620313	8.059328	9.024886
DKFZP564C	204687_at	2.306766	2.317231	2.242795	2.261963	2.289187	2.258403	2.268938	2.288627	2.276233	2.263219	2.479033	2.276114	5.397251
SGCE	204688_at	7.320591	7.021855	4.360215	4.117771	3.198016	2.906635	7.111624	5.861102	7.387328	7.374291	5.883527	5.937313	8.153773
HHEX	204689_at	8.542493	8.297906	8.235399	8.071654	8.711285	8.329941	8.157221	7.334942	8.035396	8.136767	7.017378	7.650418	4.457735
STX8	204690_at	7.676739	7.879907	7.467868	7.804044	8.218451	8.180564	7.633942	7.835387	7.420578	7.684003	8.176741	7.957379	7.379962
PLA2G6	204691_x_at	5.276554	5.377397	5.237677	5.225608	5.267137	5.325561	5.267104	5.339451	5.25714	5.252829	5.247855	5.258446	5.218668
LRCH4	204692_at	3.665413	3.647152	3.071699	3.62694	3.648728	3.712331	3.639614	3.644596	3.628526	3.624496	3.750225	3.6424	3.590943
CDC42EP1	204693_at	6.377164	6.423072	6.293932	6.278032	5.803419	5.777874	6.29151	6.287662	6.4279	6.444101	6.26913	6.286124	5.425321
AFP	204694_at	2.805905	3.118092	4.246137	4.548658	3.17005	3.402001	14.90378	14.83656	15.00233	14.9515	14.90738	14.96944	12.99676
CDC25A	204695_at	2.892337	3.099546	2.500808	2.545308	2.554786	2.548225	7.904688	8.265566	8.34737	8.340721	7.613423	8.361221	4.577508
CDC25A	204696_s_at	2.269144	2.255518	2.219398	2.2349	2.264504	2.228567	3.331926	2.253501	2.245956	2.271312	2.24061	2.304121	2.144696

CHGA	204697_s_at	2.393997	2.459201	2.335572	2.363398	2.380437	2.357239	2.380463	2.383243	2.366517	2.361302	2.681443	2.376772	4.702756
ISG20	204698_at	3.990099	3.344297	3.668356	4.006533	4.030801	4.106328	5.444688	5.722085	5.081886	5.0006	4.343427	5.167773	2.057475
C1orf107	204699_s_at	5.133134	5.064448	5.039773	5.091086	5.106086	4.658909	5.104099	4.96547	5.777291	5.382259	4.894819	4.485486	5.391933
C1orf107	204700_x_at	6.016657	6.187623	6.082008	5.859792	6.489258	6.04941	6.066377	5.844408	5.888272	5.838273	6.022919	5.971752	6.432712
STOML1	204701_s_at	4.861998	4.9435	4.113611	4.845085	5.005018	5.645714	4.703093	5.107346	4.746826	4.841396	5.559231	5.615248	4.747593
NFE2L3	204702_s_at	3.125938	3.086544	3.02906	3.105898	3.137849	2.981525	3.105147	3.069611	3.08136	3.118553	3.072047	3.100026	2.911997
IFT88	204703_at	8.288497	8.050146	8.063611	8.096682	8.291747	8.119693	7.362756	7.204267	7.276239	6.705088	7.098437	7.090865	7.787894
ALDOB	204704_s_at	12.5025	12.55386	12.50261	12.48941	11.62339	11.32469	9.265796	8.961613	9.182221	8.9544	8.183008	8.34309	2.574145
ALDOB	204705_x_at	14.35591	14.35376	14.24843	14.36449	14.05962	13.68326	13.11945	12.24078	13.11731	12.97429	12.15791	12.21499	7.346256
INPP5E	204706_at	6.427244	6.44358	6.270682	6.273847	6.618294	6.330221	6.394793	6.088885	5.531997	5.954044	6.46992	6.107776	6.848875
MAPK4	204707_s_at	2.274389	2.258764	2.241043	2.244905	2.28144	2.245343	2.253607	2.260832	2.246647	2.240415	2.239408	2.252308	2.200485
MAPK4	204708_at	2.466168	2.435859	2.427178	2.421927	2.451202	2.436388	2.441819	2.448206	2.439092	2.434816	2.432999	2.581999	2.296534
KIF23	204709_s_at	3.366435	3.333705	3.235877	3.166162	3.349737	3.05977	9.010906	7.902892	8.923317	9.140987	7.318977	7.513208	7.5061
WIPI2	204710_s_at	9.539131	9.401207	9.064048	9.40264	9.285682	9.254233	10.18916	10.41305	10.1975	10.30577	10.15592	10.35255	8.53107
KIAA0753	204711_at	5.650588	5.435421	4.235348	4.438071	4.755326	4.751998	4.762362	4.792167	5.049296	5.054687	4.727294	4.745157	6.185058
WIF1	204712_at	3.025194	2.937145	2.850091	2.974104	3.013597	2.957019	2.975581	3.10762	2.976929	2.911844	5.673104	3.012753	10.89152
F5	204713_s_at	11.75723	11.70427	12.1888	12.11124	12.0997	11.54574	10.70985	10.42106	10.68333	10.41888	10.06797	10.2765	4.60096
F5	204714_s_at	11.8325	11.78301	12.1404	11.95579	11.74126	11.23371	11.0789	10.44753	11.17339	10.93145	9.740223	9.745384	3.892513
PANX1	204715_at	4.304256	4.573954	4.48524	4.404884	4.568088	4.192086	3.606826	3.452036	3.15878	3.641814	3.228794	2.715334	4.575239
CCDC6	204716_at	4.381676	4.355263	4.284954	4.147812	4.357006	4.339225	4.325679	4.351525	4.342158	4.338947	4.48742	4.585391	5.627288
SLC29A2	204717_s_at	2.57473	3.397229	2.490018	2.503868	2.568531	2.547313	4.01115	4.761852	3.498786	4.023327	4.120959	3.965965	2.574718
EPHB6	204718_at	3.473732	4.071366	3.430749	3.429877	3.456178	3.619775	3.146926	3.446763	2.9999	3.156667	3.525642	3.498671	3.848017
ABCA8	204719_at	11.19701	11.18546	11.71223	11.62397	11.04788	10.75515	7.374254	6.966202	7.504151	7.118037	7.762199	6.831465	7.980399
DNAJC6	204720_s_at	2.667217	2.642483	2.591241	2.626816	2.75651	2.625048	10.43865	10.40872	10.63047	10.72018	9.166829	10.20275	8.645435
DNAJC6	204721_s_at	3.040912	2.951612	2.893566	2.935783	3.041875	2.914661	2.953838	3.068173	2.967134	2.933638	2.965594	2.998725	2.736692
SCN3B	204722_at	2.084145	2.076149	2.050625	2.05519	2.072477	2.055188	2.066382	2.074441	2.059144	2.051864	2.650017	2.069352	5.453687
SCN3B	204723_at	2.15919	2.13852	2.110275	2.119111	2.141684	2.120204	2.131985	2.137518	2.123125	2.114933	2.613896	2.131066	6.716
COL9A3	204724_s_at	4.013354	3.976413	3.4478	3.40635	5.151731	5.215454	3.965799	3.963937	2.752095	2.799354	7.323059	3.850352	10.57714
NCK1	204725_s_at	5.034902	5.095146	6.665638	5.892727	4.973869	4.633067	5.609771	4.431991	5.589562	5.443895	4.138364	4.416705	6.967071
CDH13	204726_at	2.651815	2.618559	2.614114	2.615616	2.651957	3.770546	2.616473	2.631732	2.615147	2.59491	2.728055	2.625072	4.44905
WDHD1	204727_at	3.12582	2.983508	2.860242	2.942367	3.113877	3.048299	5.427568	5.851715	5.843755	5.358562	4.780598	4.389727	4.16636
WDHD1	204728_s_at	2.673336	2.618845	2.562713	2.609831	2.686171	2.61831	5.836754	6.627276	6.333603	6.30993	5.272561	6.25544	5.129595
STX1A	204729_s_at	2.175361	2.166711	2.137958	2.148285	2.159494	2.172935	2.167358	2.165053	2.150543	2.143691	2.153024	2.159462	3.678241
RIMS3	204730_at	2.579262	2.571385	2.55402	2.562602	2.56519	3.110314	4.459107	2.949024	3.05623	3.87742	3.186865	3.855113	6.937584
TGFBR3	204731_at	7.341589	7.098227	8.484243	8.742383	8.850976	8.756845	7.488911	6.682734	5.895596	5.78239	8.278819	7.386126	8.586466
TRIM23	204732_s_at	7.09344	6.601271	7.04672	6.990007	7.21642	6.832334	7.687847	7.486359	7.274342	7.580224	7.375897	7.416772	7.807339
KLK6	204733_at	2.078947	2.063504	2.039854	2.044742	2.063149	2.044227	2.056256	2.061983	2.047218	2.039838	2.046751	2.055022	1.979156
KRT15	204734_at	2.397076	2.370921	2.304509	2.351356	2.379438	2.345465	2.338228	2.37135	2.35777	2.350695	2.357095	2.364782	5.390052

PDE4A	204735_at	5.442904	4.685021	4.442471	4.446199	4.533738	5.793034	4.398752	4.401989	4.388952	4.381931	3.543279	3.752888	4.272382
CSPG4	204736_s_at	3.074746	3.052372	2.931098	3.004559	3.064525	3.281889	3.015089	3.049402	3.027238	3.036608	4.015583	3.047369	5.757654
MYH6	204737_s_at	2.83185	2.768546	2.716837	2.771665	2.806587	2.768827	2.774542	2.789346	2.770834	2.749041	8.181306	2.796933	10.02246
KRIT1	204738_s_at	5.274756	5.392492	5.485267	5.232682	5.282542	5.229593	5.471347	5.249735	5.472276	5.237681	5.152806	5.245714	5.152695
CENPC1	204739_at	6.445457	6.752532	7.096514	7.195826	6.383289	5.849142	7.063705	6.43496	7.331277	7.215609	5.568734	6.381916	5.914628
CNKSR1	204740_at	3.777649	3.737528	3.60132	3.160814	3.631967	3.698562	4.393434	4.80861	3.71483	3.998956	4.304274	4.802597	4.001629
BICD1	204741_at	2.350519	2.352653	2.286909	2.307477	2.367235	2.320127	2.327911	2.727408	2.31668	2.299561	2.653706	2.421524	3.600542
PDS5B	204742_s_at	4.389925	4.163033	4.272441	4.656778	4.704527	4.751143	4.924678	5.016355	5.605571	5.388852	5.465974	5.101427	6.815298
TAGLN3	204743_at	2.707385	2.687038	2.663024	2.698174	2.689717	3.331515	2.674799	2.68321	2.649297	2.657044	2.663949	2.677428	7.634113
IARS	204744_s_at	8.707406	8.58723	8.663005	8.60574	8.199975	7.752802	10.87482	10.62017	10.98486	10.94418	10.21586	10.45625	10.52722
MT1G	204745_x_at	12.75514	12.74709	12.97765	13.10562	13.68427	13.64281	14.07049	14.06679	13.91151	13.98372	14.01015	13.84069	10.25421
PICK1	204746_s_at	4.012212	3.999572	3.934133	3.959718	4.176384	4.131847	3.90917	3.898735	3.825491	3.943317	4.192706	3.843248	3.718683
IFIT3	204747_at	6.324815	6.40111	6.230809	6.028196	6.474665	6.581768	4.99494	4.748938	4.574668	4.613155	3.768578	4.242222	4.713759
PTGS2	204748_at	2.900279	2.845053	4.957901	4.306404	6.200526	5.520911	2.839593	2.894181	2.835551	2.81801	2.835044	2.884605	3.967168
NAP1L3	204749_at	3.231577	2.900474	2.998798	2.963688	3.047169	2.86538	4.65654	3.640772	4.256185	3.729194	5.899815	3.985137	9.756615
DSC2	204750_s_at	3.41055	3.262329	3.353563	3.446712	4.168115	4.208985	3.411196	4.71004	3.502639	3.395391	4.032255	4.972803	2.894048
DSC2	204751_x_at	3.259451	3.233785	3.488197	3.246252	3.239288	3.300502	3.394474	3.233992	3.267123	3.215437	2.984483	3.148879	3.510354
PARP2	204752_x_at	6.000585	5.854525	6.019306	6.068229	5.956048	5.206922	7.831176	6.847869	7.68617	7.43377	6.905584	6.725874	7.612467
HLF	204753_s_at	9.745266	9.532728	10.43872	10.36106	8.696112	7.79502	5.752071	4.854226	5.874041	5.495565	4.434436	5.138739	4.301836
HLF	204754_at	7.705278	7.628135	8.308072	8.312796	7.316799	6.749347	4.556541	5.212837	4.266472	4.09034	4.261611	5.327642	3.41552
HLF	204755_x_at	8.700205	8.708043	9.526437	9.323623	8.471884	7.851713	4.628407	5.101199	4.722717	4.054206	4.646146	4.678144	2.805678
MAP2K5	204756_at	6.224023	5.792031	5.55794	5.890698	6.575145	6.675967	4.341191	4.954907	4.431931	4.295094	6.069972	5.479091	4.949969
TMEM24	204757_s_at	4.573615	4.56537	4.133743	4.321918	4.814293	5.233438	4.547175	5.03654	4.314071	4.338465	4.946021	4.556703	3.968674
TMEM24	204758_s_at	2.671578	2.622536	2.584408	2.617795	2.66996	2.77314	2.625723	2.632044	2.618673	2.612523	2.619184	2.634545	2.478198
RCBTB2	204759_at	6.899217	6.802817	7.308784	6.664718	6.848049	6.165619	6.108509	6.127731	5.840011	5.692675	7.582471	6.206547	10.15099
NR1D1	204760_s_at	2.391987	2.412188	2.362902	2.404271	3.549372	2.442295	2.34973	2.363753	2.353004	2.33069	2.342574	2.357169	2.317511
USP6NL	204761_at	7.703047	7.613442	7.833141	7.462635	6.816642	6.689156	7.354775	7.061634	7.771614	7.985586	6.956792	7.243563	7.170795
GNAO1	204762_s_at	2.049793	1.975846	3.317703	2.960415	1.98319	1.964319	1.959734	1.969109	1.952877	1.944841	1.943926	1.956192	4.213911
GNAO1	204763_s_at	6.75496	6.30924	6.007065	6.544581	6.266096	6.773957	3.526543	4.014369	3.598307	3.334851	3.95272	3.560103	5.824711
FNTB	204764_at	1.999344	1.986901	1.96206	1.972511	1.983209	1.965474	2.030067	1.980767	1.975942	1.97104	1.96329	1.977407	4.297367
ARHGEF5	204765_at	7.131935	7.23387	6.593017	6.750991	7.230054	7.084363	6.04496	6.356099	5.341969	5.361474	6.433321	6.26541	4.93228
NUDT1	204766_s_at	3.54514	3.433518	3.040442	3.071968	3.083254	3.418033	6.454456	6.564595	6.833098	6.833573	7.207026	7.472487	5.285322
FEN1	204767_s_at	6.852395	6.72151	5.67789	6.123166	6.847454	6.879844	10.29815	10.52718	10.11056	10.1181	10.25769	10.65436	7.621122
FEN1	204768_s_at	2.490775	2.546303	2.515019	2.326648	2.238317	2.191958	7.90252	6.352062	7.656064	7.368715	5.753626	6.455768	5.304611
TAP2	204769_s_at	2.364781	2.3199	2.296224	2.304551	2.317528	2.30022	2.312219	2.314266	2.303277	2.297123	2.30581	2.307852	2.174484
TAP2	204770_at	1.756512	1.746165	1.72787	1.733347	1.743521	1.732141	1.743432	1.744308	1.735132	1.729846	1.729016	1.737314	1.695807
TTF1	204771_s_at	7.561869	7.254488	6.61652	7.186477	8.221182	8.180444	7.6641	8.384895	7.603997	7.491808	8.611815	8.484327	7.168846
TTF1	204772_s_at	3.755383	3.753195	4.001713	4.112125	3.656946	3.425849	5.339171	4.924779	5.687149	5.478514	4.408234	4.436752	4.943116

IL11RA	204773_at	6.93346	7.125062	6.563725	6.633495	7.531322	7.373699	5.52872	5.878837	5.569554	5.56054	8.059033	5.912028	10.28692
EVI2A	204774_at	4.733731	4.603806	5.142311	4.682345	5.003783	5.690268	3.67828	3.334292	3.214397	3.529946	3.537301	4.173359	4.184202
CHAF1B	204775_at	2.506533	2.616226	2.431985	2.560804	2.588987	2.576921	3.264048	3.516659	3.473701	3.974344	3.111941	3.508572	2.638677
THBS4	204776_at	2.222845	2.226452	2.21536	2.225417	2.234329	2.222419	5.417342	2.929797	2.426821	2.223279	6.588345	5.495568	9.574527
MAL	204777_s_at	2.329287	2.289256	2.116432	2.290634	2.307302	2.297545	2.386081	2.308649	2.300681	2.286271	2.429488	2.319819	3.392038
HOXB7	204778_x_at	2.67488	2.638957	2.601765	2.632375	2.644363	2.609016	2.646449	2.646731	2.630043	2.606177	2.690489	2.65768	4.992569
HOXB7	204779_s_at	3.704924	3.647293	3.657183	3.838849	3.784958	3.623945	3.583449	3.619116	3.595542	3.592115	4.184983	3.613666	6.449483
FAS	204780_s_at	7.980056	7.560292	7.556886	7.353397	6.487653	5.905506	4.53856	3.423061	3.713571	3.358652	3.638178	3.527786	5.000427
FAS	204781_s_at	7.57754	7.50742	7.988713	7.574547	6.428946	6.404381	3.994153	4.793713	3.654561	4.046081	4.868925	4.556197	4.454538
MLF1	204783_at	2.841679	2.792801	2.876331	2.799559	2.823976	2.675214	3.12245	2.827539	2.854049	3.036778	2.806226	2.809268	6.371026
MLF1	204784_s_at	2.927246	2.839738	3.602308	3.538681	2.914942	3.300329	3.977806	4.536805	4.328407	3.610527	5.348841	4.850397	7.955427
IFNAR2	204785_x_at	5.849058	5.843828	5.733044	5.831372	5.741709	5.829238	5.83718	5.829455	5.834436	5.827414	5.821245	5.822058	5.558603
IFNAR2	204786_s_at	8.412321	8.400749	7.276038	7.132048	8.744392	8.928394	7.706038	8.540549	7.660526	7.370178	8.243682	8.378329	6.147625
VSIG4	204787_at	5.363033	5.162452	7.67083	8.04934	6.427653	6.495701	6.230573	5.711171	6.6671	6.425798	5.173837	5.341939	5.947232
PPOX	204788_s_at	5.633181	6.021182	5.604667	5.664127	6.038674	6.042051	10.38339	10.77051	10.34373	10.49649	10.14757	10.76998	5.961159
FMNL1	204789_at	3.213921	3.460132	3.104258	2.761919	3.520392	3.110533	3.060771	3.082507	3.031618	3.055602	3.059175	3.277194	2.691573
SMAD7	204790_at	5.712171	5.413978	5.788827	4.980959	4.563646	4.593084	4.561334	3.547527	4.480263	3.983534	4.276162	3.687422	5.331642
NR2C1	204791_at	7.983218	8.065547	8.245333	8.209736	7.588674	7.269496	7.842032	7.309223	7.99442	8.071937	7.196264	7.059612	7.890768
IFT140	204792_s_at	2.95926	3.123041	2.877512	2.98991	2.909877	2.865579	2.867363	2.891959	2.880843	2.673869	2.881249	2.904464	3.388069
GPRASP1	204793_at	4.326257	4.428809	4.030496	3.843861	4.297479	4.352295	5.591799	5.084459	5.592436	5.402002	5.447606	5.05539	7.301531
DUSP2	204794_at	2.720095	3.455045	2.993433	2.973229	4.335098	4.742391	3.530654	3.534935	3.521604	3.522444	3.544779	3.561998	3.462486
PRR3	204795_at	2.277765	2.317792	2.2364	2.235695	2.239946	2.312303	2.595411	2.249694	2.364032	2.709007	3.215782	2.780473	6.00472
EML1	204796_at	3.658712	3.370374	4.368474	3.93207	3.525108	3.489367	3.304482	2.974019	3.06916	2.95587	3.500098	2.959901	6.41913
EML1	204797_s_at	4.521038	4.045309	3.934244	4.173771	3.317959	3.254433	3.293004	2.272886	3.238571	3.112109	4.632514	3.204609	6.856473
MYB	204798_at	2.489148	2.461019	2.435066	2.440814	2.473504	2.44892	8.903639	8.482492	9.011624	8.780176	7.370011	7.859959	3.324562
ZBED4	204799_at	2.760087	2.475448	3.26565	3.170202	2.533425	2.709671	4.558342	3.309777	4.464522	4.871484	3.844256	3.502784	5.493022
DHRS12	204800_s_at	5.690893	5.480445	6.1796	5.836148	5.183424	4.98903	5.394142	4.209341	5.23447	4.938301	4.308528	4.365444	3.903892
DHRS12	204801_s_at	2.67432	2.961026	3.087325	3.170291	3.205455	3.019992	2.954249	3.091886	2.948922	2.965611	2.950043	2.95861	2.849249
RRAD	204802_at	2.459231	2.452452	2.430003	2.436131	2.451266	2.432328	2.43758	2.447089	2.436335	2.418088	2.435791	2.443559	3.701712
RRAD	204803_s_at	2.371952	2.35625	2.36354	2.344117	2.153585	2.345278	2.327651	2.353088	2.323695	2.34229	2.335111	2.348002	3.041956
TRIM21	204804_at	4.166987	4.289624	4.37483	4.080362	4.145104	4.410342	5.255507	4.348637	5.484335	4.240041	4.130916	4.36925	3.257238
H1FX	204805_s_at	8.015242	8.129872	8.157145	8.188047	7.729511	7.245687	9.263094	9.163214	9.661084	9.893548	8.940255	9.211426	8.964531
HLA-F	204806_x_at	9.212772	9.106318	9.100569	9.156877	8.950869	9.116882	6.920282	6.82509	7.645772	7.728269	6.607534	7.238568	7.068957
TMEM5	204807_at	5.559	5.295822	6.154255	6.337509	6.003091	5.770729	5.13884	5.471972	5.923952	5.172221	5.205141	5.408483	6.463739
TMEM5	204808_s_at	8.543156	8.521845	8.13479	8.727142	9.141907	9.085978	7.274052	8.294017	7.532373	7.658523	8.61154	8.28066	7.971585
CLPX	204809_at	9.284018	9.125069	9.62355	9.733129	8.956214	8.700547	8.619865	8.275599	8.763501	8.687236	7.776466	8.037934	7.75442
CKM	204810_s_at	2.488708	2.452302	2.400067	2.431969	2.515021	2.436246	2.440657	2.452706	2.437556	2.431957	9.40372	2.455303	10.976
CACNA2D2	204811_s_at	3.93912	3.929485	3.78402	3.914738	3.923313	4.018978	3.870937	4.257031	3.6206	3.359552	4.253751	3.883254	6.087934

ZW10	204812_at	5.748075	5.611421	6.223242	5.942859	5.28831	5.127159	7.015725	6.202014	6.812411	6.89218	5.992812	6.389643	6.363103
MAPK10	204813_at	2.035035	2.028454	2.007637	1.862354	2.211516	2.440293	1.83959	2.013477	1.97939	1.988283	2.006632	2.008256	3.810804
CADPS	204814_at	2.058961	2.04275	2.022694	2.020448	2.041915	2.02542	2.033213	2.038815	2.027685	2.022631	2.020157	2.032894	1.989781
DHX34	204815_s_at	2.130588	2.114623	2.082133	2.10039	2.114799	2.100139	2.107511	2.111935	2.10144	2.095036	2.0935	2.106701	2.07491
DHX34	204816_s_at	3.614686	3.448556	3.266317	3.404843	3.754619	3.398954	3.36592	3.384487	3.434854	3.430377	3.484016	3.488373	2.956534
ESPL1	204817_at	4.626732	4.575621	4.175102	4.355194	4.576192	5.014443	6.533114	6.622618	7.114786	6.626379	6.608096	6.628699	5.093659
HSD17B2	204818_at	10.25449	10.30566	10.26995	10.33513	10.03342	9.721574	10.55086	9.740939	10.11352	9.879795	9.287282	10.0042	3.369016
FGD1	204819_at	2.186414	2.356889	2.255976	2.070449	2.448743	2.324537	3.159188	2.369674	2.826138	2.96893	2.827626	2.361019	6.134142
BTN3A2	204820_s_at	9.354186	9.183574	8.9072	8.878337	9.608572	9.915856	7.127828	6.79125	6.95313	7.00231	7.572525	7.262978	6.409498
BTN3A3	204821_at	6.955843	6.659614	6.442986	6.370129	6.777936	6.372194	4.182466	4.087366	4.758203	4.16852	4.342413	4.393129	4.461096
TTK	204822_at	3.437532	3.052852	2.435439	2.458081	2.448463	2.875705	8.77318	9.414498	9.043886	8.9654	9.212838	9.468499	7.329377
NAV3	204823_at	2.373422	2.365396	2.350689	2.346864	2.592554	2.345939	2.361635	2.362829	2.348195	2.334256	6.38105	2.354827	8.338297
ENDOG	204824_at	8.077247	8.038851	8.055459	8.384542	8.558934	8.770583	7.066867	7.843329	7.44449	7.72807	8.106503	8.049472	6.080043
MELK	204825_at	4.108823	4.072883	2.731708	2.736191	2.835823	3.66667	8.969285	8.634697	9.012867	9.098102	8.510264	8.702002	8.008183
CCNF	204826_at	4.854928	4.291437	3.224433	3.755451	4.686736	4.699932	7.924852	8.456097	7.984462	8.072345	7.999535	8.387118	5.350139
CCNF	204827_s_at	2.063781	2.044225	2.032184	2.036983	2.044251	2.037493	4.809295	3.307906	4.430715	4.578556	3.761002	3.418927	3.061801
RAD9A	204828_at	4.651497	4.629633	4.612615	4.446951	4.657931	5.036462	4.670148	5.169507	4.621678	4.629955	5.41094	5.371823	4.367192
FOLR2	204829_s_at	4.857215	5.760194	6.665225	7.049354	6.813484	6.745776	6.420246	6.423173	6.327617	5.940482	5.86353	7.08273	4.533927
PSG5	204830_x_at	2.480776	2.447668	2.423204	2.427455	2.452838	2.427159	2.44557	2.44699	4.631118	2.420657	2.432948	2.441343	2.387593
CDK8	204831_at	7.049811	6.829252	6.634249	7.013201	6.350381	6.617922	7.854816	7.697364	7.89955	7.843551	7.638664	7.628196	8.747501
BMPR1A	204832_s_at	3.429525	3.720188	4.257856	3.754458	3.390066	3.154294	3.461554	3.260525	3.416525	3.386666	3.268821	3.369267	7.465403
ATG12	204833_at	4.970826	4.959785	5.243492	4.945499	4.468819	4.61812	5.395217	4.872706	5.733678	5.46489	4.941191	5.207576	6.988288
FGL2	204834_at	5.66346	5.21697	6.579216	6.393599	6.308092	5.715556	3.092523	4.150802	4.252135	3.997152	2.8561	2.891984	3.804035
POLA1	204835_at	3.765989	3.577619	3.928596	3.822436	4.179512	3.526231	6.885661	6.457853	7.121639	7.146149	6.407197	6.749578	6.739206
GLDC	204836_at	10.78071	10.87124	10.61513	10.62988	10.32319	10.23699	11.01704	10.46497	10.28296	10.26804	9.625269	9.171041	5.525466
MTMR9	204837_at	5.494347	5.05306	5.473327	5.400202	5.297082	5.781801	5.098799	3.604798	4.964144	4.815229	5.166124	5.15828	6.645443
MLH3	204838_s_at	6.151284	5.608778	5.470712	5.239139	6.130645	5.994481	4.322951	4.504843	4.831469	4.678049	5.562622	4.494215	5.652958
POP5	204839_at	7.77791	7.748467	8.476807	8.548179	8.312224	8.450619	8.39966	8.741723	8.343008	8.246406	8.348228	8.542215	7.700591
EEA1	204840_s_at	5.23352	5.450288	4.853629	5.528955	6.133964	5.530712	4.709296	4.985224	4.613981	4.383714	5.002765	5.596427	4.268636
EEA1	204841_s_at	2.538539	2.47681	2.455288	2.483538	2.525434	2.468361	2.491934	2.475408	2.461366	2.474902	2.462169	2.500281	3.183291
PRKAR2A	204842_x_at	9.955514	9.967706	9.297971	9.557639	9.836675	9.978935	9.272434	9.627046	9.287246	9.153652	9.721341	9.648345	9.479524
PRKAR2A	204843_s_at	2.174545	2.153927	2.124865	2.140433	2.156992	2.132141	2.142407	2.152085	2.137181	2.130172	2.137029	2.14703	2.635003
ENPEP	204844_at	9.739306	9.695079	9.976355	9.597282	8.001089	7.466217	2.878481	3.31702	2.938096	3.20803	3.159883	3.17406	5.837598
ENPEP	204845_s_at	6.83225	6.628087	8.08592	7.262414	3.893697	3.080314	3.099409	2.936039	2.955113	3.033847	3.049956	3.062231	4.583739
CP	204846_at	12.25323	12.11741	12.91147	12.73069	12.05593	11.54656	8.176796	7.072611	8.45891	8.574701	5.871739	7.123489	3.115586
ZBTB11	204847_at	7.991828	7.99017	8.281816	7.899308	8.130989	7.307695	8.47006	8.241718	8.755771	8.658271	8.059396	8.02545	8.753589
HBG1	204848_x_at	4.247966	5.44547	4.47907	4.051375	4.068894	4.452915	15.44365	15.42089	15.31874	15.34151	15.40166	15.43032	14.33312
TCFL5	204849_at	5.942687	5.90334	6.364768	6.534912	6.329943	6.012494	5.897732	5.897066	5.597368	5.469121	5.694741	5.23833	7.285171

DCX	204850_s_at	2.499905	2.46412	2.399024	2.449719	2.485705	2.435809	2.454599	2.466485	2.450112	2.441464	7.187123	2.462906	10.37089
DCX	204851_s_at	2.529029	2.498016	2.440979	2.478235	2.512487	2.466038	2.485303	2.498394	2.483218	2.472387	7.376031	2.500931	10.61531
PTPN7	204852_s_at	2.659235	2.043432	2.620288	2.62386	2.650557	2.640096	3.15215	2.945234	3.797481	3.524164	3.185283	3.360306	2.148921
ORC2L	204853_at	4.922498	4.79269	5.343461	5.053835	4.403706	4.094073	5.78073	4.634626	6.046707	6.15722	4.762931	4.936536	4.800191
LEPREL2	204854_at	2.676297	2.663117	2.645819	2.651073	2.703253	2.65758	2.609636	2.661375	2.613346	2.639209	3.316296	2.655047	5.120015
SERPINB5	204855_at	2.141449	2.119921	2.09757	2.104346	2.121231	2.101567	2.113511	2.118765	2.105157	2.10617	2.101074	2.113391	2.178615
B3GNT3	204856_at	2.598619	2.677645	2.586042	2.672395	2.662325	2.792207	2.762111	2.678068	2.662133	2.73804	2.918525	2.560987	2.426662
MAD1L1	204857_at	3.803383	4.509156	5.298562	5.375272	4.91019	4.41112	4.356448	4.801763	4.796477	5.328281	4.909974	5.220787	5.628951
ECGF1	204858_s_at	5.865675	6.033558	4.489066	4.659935	5.194515	5.551505	2.067303	2.142766	1.861566	1.752006	1.855171	2.818124	2.030113
APAF1	204859_s_at	7.301906	6.845693	6.705949	6.629743	6.945112	6.748368	6.81653	6.791998	6.798198	6.669318	7.408662	6.633266	8.442527
NAIP	204860_s_at	3.828197	3.640743	3.806868	4.314096	3.793326	3.628826	3.796868	3.967152	3.963102	3.362293	4.064359	3.568032	4.002417
NAIP	204861_s_at	3.50143	3.608889	3.273967	3.280687	3.304412	3.277262	3.235541	3.293302	3.310172	3.207193	3.307158	3.286877	3.210537
NME3	204862_s_at	6.140622	5.942825	5.987216	6.253137	6.705469	6.628879	4.231586	5.052143	4.055267	4.271303	5.684752	5.488496	5.827631
IL6ST	204863_s_at	10.37688	9.99582	10.08188	10.36555	10.91754	11.02842	8.563793	9.117063	8.247246	7.946779	9.726631	9.772934	6.843024
IL6ST	204864_s_at	2.105826	2.08482	2.069999	2.080095	2.087997	2.071768	2.080626	2.085316	2.070456	2.069282	2.06731	2.079293	3.471574
CA3	204865_at	3.465657	3.354952	3.327475	3.366568	3.623655	3.452693	3.353825	3.351129	3.372457	3.333676	4.679391	3.426698	7.232623
PHF16	204866_at	7.256936	6.948918	7.14867	7.166335	7.152409	6.370289	6.68589	6.389123	6.681408	6.626421	5.870855	6.595472	6.124613
GCHFR	204867_at	8.256141	8.638014	9.273497	9.263377	9.500086	9.426417	8.552224	8.731031	8.527789	8.138195	8.571078	8.696434	5.688853
ICT1	204868_at	7.200975	7.223283	7.24761	7.64791	7.310821	7.273347	7.421369	7.626283	7.879169	7.721031	7.363468	7.706875	5.836068
PCSK2	204869_at	2.318525	2.301821	2.26365	2.283322	2.309658	2.251845	2.287721	2.298952	2.286042	2.273181	3.536538	2.293963	5.003583
PCSK2	204870_s_at	2.452267	2.429799	2.411734	2.411639	2.437709	2.420313	2.484609	2.434004	2.421307	2.414981	2.690559	2.428873	5.071498
MTERF	204871_at	5.108927	5.207373	5.137826	5.264464	5.113901	5.088908	5.130201	5.353506	5.21211	5.165413	5.155418	4.965421	5.627958
TLE4	204872_at	7.426518	7.185151	7.526754	7.557276	7.700583	7.75261	7.592154	7.599814	7.20548	7.389876	8.417425	8.142754	9.255916
PEX1	204873_at	7.181289	6.858265	7.230419	7.061329	6.80905	6.53778	6.597281	6.748849	6.658716	6.55563	6.701703	6.934947	6.674577
BAIAP3	204874_x_at	2.141895	2.255471	2.104881	2.108668	2.131011	2.890601	2.121103	2.125934	2.102653	2.103925	2.107981	2.118542	2.034767
GMDS	204875_s_at	5.532858	5.332807	6.351015	6.610275	6.763895	6.426053	5.59427	5.838875	6.145922	5.923687	6.062127	6.062644	6.536871
ZNF646	204876_at	2.701504	2.685366	2.664284	2.666179	2.70527	2.667493	2.675652	2.686639	2.668878	2.660176	2.664084	2.689948	2.543386
TAOK2	204877_s_at	2.855678	2.842167	3.243428	3.257806	2.843083	2.800524	2.838282	2.785464	2.821904	2.806126	2.817575	2.828288	2.585046
TAOK2	204878_s_at	2.862515	2.856537	2.838898	2.84218	2.85959	2.845369	2.853433	2.855089	2.843989	2.84319	2.917929	2.851547	2.822384
PDPN	204879_at	2.09811	2.092654	2.061984	2.07025	2.086054	2.120355	2.078967	2.08489	2.071281	2.064482	2.07195	2.077476	5.447559
MGMT	204880_at	9.734389	9.738927	9.707375	10.16154	9.603059	9.759568	8.251217	9.245085	8.082145	8.08404	8.764252	8.872631	6.171093
UGCG	204881_s_at	9.189249	9.093319	9.088658	8.676876	8.822901	8.604181	9.493688	8.616315	9.5945	9.497035	8.397213	8.613078	9.508862
ARHGAP25	204882_at	3.621552	3.607362	3.586379	3.581825	3.928302	4.649849	3.022629	2.523238	2.523457	3.571601	2.507317	2.930329	1.973439
HUS1	204883_s_at	6.024451	5.861434	6.119965	6.274303	5.789729	5.511076	6.511452	6.107288	6.393242	6.091556	6.124936	6.211373	4.997731
HUS1	204884_s_at	2.788595	2.69477	3.007508	2.738059	2.776601	2.737365	3.063931	2.775868	2.872508	2.758938	2.76263	2.799737	2.718696
MSLN	204885_s_at	2.703858	2.666808	2.629502	2.646245	2.69019	2.641173	2.656162	2.669173	2.65498	2.644636	2.680768	2.679781	2.607238
PLK4	204886_at	3.433826	3.120567	2.962466	3.148525	3.184726	2.987157	7.728927	8.045673	7.999434	7.88627	6.717524	7.998765	6.51608
PLK4	204887_s_at	2.468256	2.450808	2.421303	2.432383	2.453127	2.431326	7.647132	5.469168	7.711672	7.925837	5.16129	5.855209	5.330408

NEURL	204888_s_at	2.235667	2.218061	2.200763	2.205401	2.218089	2.204312	2.211723	2.215791	2.206735	2.195656	2.197863	2.21005	2.165966
NEURL	204889_s_at	3.185744	3.151611	3.010741	3.088289	3.146918	3.12803	3.131984	3.17237	3.118768	3.117918	3.148571	3.147078	3.065397
LCK	204890_s_at	2.394269	2.360291	2.342964	2.338327	2.361427	2.335303	2.346779	2.359312	2.342044	2.327396	2.342797	2.355857	2.263602
LCK	204891_s_at	4.939813	4.29069	3.341437	2.921668	4.63517	5.698265	2.545996	2.627128	2.528411	2.503277	2.604587	2.622068	2.539876
EEF1A1	204892_x_at	13.98844	13.90944	13.933	13.99231	13.86578	13.59432	14.35133	14.02522	14.39889	14.33483	14.18769	14.12391	14.81501
ZFYVE9	204893_s_at	3.298845	3.165542	3.109808	3.43997	3.13752	3.746051	3.132782	3.115205	2.624567	3.107492	3.110342	2.670091	3.088582
AOC3	204894_s_at	7.57955	7.239084	7.237197	7.473977	7.320824	8.010636	4.436298	4.681787	4.509429	4.602951	5.126447	5.449629	5.355621
MUC4	204895_x_at	2.102616	2.090261	2.063359	2.070115	2.092086	2.062057	2.083566	2.089817	2.078228	2.06782	2.072518	2.084614	2.023179
PTGER4	204896_s_at	1.901331	1.888494	1.869381	1.872806	1.885156	1.87577	1.887768	1.886655	1.878464	1.871164	1.873815	1.880373	1.980718
PTGER4	204897_at	6.673563	6.518019	6.860101	6.865096	8.19533	8.572434	2.767641	2.758118	2.886611	2.739603	5.225848	2.755317	7.912767
SAP30	204898_at	1.964524	1.944633	1.922861	1.927488	1.943851	1.927254	1.939603	1.944762	1.930341	1.924467	1.923073	1.936785	1.87815
SAP30	204899_s_at	2.965969	3.23518	3.207867	3.215943	3.226354	2.65302	4.309871	3.638684	3.811041	3.456345	3.219284	3.228435	3.719471
SAP30	204900_x_at	6.289857	6.094986	6.190524	6.194467	6.068851	5.680952	8.254042	8.288124	8.39314	8.342973	7.409982	7.972383	7.723967
BTRC	204901_at	2.570115	2.727376	2.654861	2.539675	2.722673	3.519638	4.626328	6.006713	5.183464	4.890419	5.592858	5.417673	2.811588
ATG4B	204902_s_at	5.150569	5.279949	4.566193	4.570585	4.982819	5.3062	5.027212	5.438352	5.021748	5.015009	5.139467	5.018958	5.585781
ATG4B	204903_x_at	7.204092	7.218924	6.791493	7.083736	7.258826	7.378367	7.378139	7.813159	7.398126	7.497317	7.717679	7.798801	7.007996
GJA4	204904_at	5.067686	4.998233	4.978802	4.977152	5.37832	5.172437	4.522052	4.995288	4.902335	4.735056	5.290306	4.989741	5.021219
EEF1E1	204905_s_at	6.652457	6.033772	6.756158	6.583391	6.545485	5.067921	9.540653	8.703055	9.440065	9.496944	8.203631	8.817844	9.258809
RPS6KA2	204906_at	5.298183	5.820334	5.753891	6.308674	6.223889	6.554688	5.028664	5.07629	5.010294	4.758439	6.235482	5.128669	6.129013
BCL3	204907_s_at	1.894726	1.884252	1.865129	1.866195	1.883072	1.865445	1.868201	1.878867	1.867801	1.862145	1.854935	1.874308	1.819992
BCL3	204908_s_at	7.326383	7.444001	7.917727	7.657845	7.456659	7.746141	3.817318	4.302042	3.96636	3.882321	4.520625	4.006184	3.714707
DDX6	204909_at	4.070369	3.930253	3.756076	3.89687	4.209733	4.354077	3.9535	4.11692	3.9358	3.884981	4.013516	4.074358	3.185075
TRIM3	204910_s_at	2.568017	2.515676	2.468258	2.510292	2.545366	2.502063	2.517204	2.529001	2.512095	2.503683	2.516033	2.524236	2.406677
TRIM3	204911_s_at	2.583257	2.533891	2.533948	2.538094	2.564065	2.503786	2.548406	2.545385	2.540213	2.534529	2.513936	2.543091	3.307494
IL10RA	204912_at	6.513192	6.248065	6.587603	7.174071	7.396824	7.800749	4.758203	5.856412	5.094114	5.250901	4.491578	5.462191	3.460341
SOX11	204913_s_at	2.17525	2.146978	2.119609	2.127955	2.167393	2.125197	2.150322	2.145913	2.133156	2.124563	8.035866	2.16454	11.49644
SOX11	204914_s_at	2.39601	2.368981	2.338754	2.350749	2.38582	2.350941	2.363532	2.370709	2.363406	2.359573	8.629384	2.868606	11.92973
SOX11	204915_s_at	2.250705	2.228526	2.205367	2.216879	2.233806	2.217569	2.223567	2.235714	2.221105	2.217013	3.8794	2.225917	9.173975
RAMP1	204916_at	5.885624	5.760039	6.765699	7.066438	7.181085	7.507984	4.001603	5.406309	5.372518	5.401198	5.743711	5.883609	4.837507
MLLT3	204917_s_at	2.146041	2.133413	2.110341	2.122428	2.133149	2.112118	5.219038	3.247419	5.54043	5.888296	2.179068	2.829645	6.296163
MLLT3	204918_s_at	3.900956	3.454265	3.445123	3.73539	3.883128	3.915398	4.992473	6.097557	5.872465	6.35557	5.955471	5.765486	6.192072
PRR4	204919_at	3.416964	3.168249	3.225883	3.133596	3.156999	3.119231	3.177037	3.161594	3.172547	3.458057	3.145308	3.182139	3.420451
CPS1	204920_at	14.03132	14.14045	14.63719	14.64834	13.97878	13.76977	14.08225	13.86838	14.00236	13.88766	13.42644	13.6581	8.130501
GAS8	204921_at	2.964275	3.105026	3.076191	3.073026	3.093382	3.093625	3.101276	3.103709	3.098192	3.096088	3.105456	3.240262	3.005755
C11orf80	204922_at	2.537595	2.519215	2.432504	2.49772	2.513711	2.498368	2.498051	2.517401	2.502351	2.50054	2.499727	2.534113	2.512335
CXorf9	204923_at	5.101155	4.829146	4.582073	4.320289	5.620726	6.311891	4.184465	4.662686	4.269154	3.717794	4.459668	5.335673	3.927562
TLR2	204924_at	3.377204	3.585486	5.002909	4.756064	4.136059	4.600581	4.132368	3.032659	3.771917	3.219115	3.174941	2.780608	3.014461
CTNS	204925_at	4.162612	3.736659	3.715918	3.721669	3.733056	3.724744	3.731498	3.734423	3.726278	3.71843	3.757554	3.726677	3.542756

INHBA	204926_at	3.454221	3.341148	3.283507	3.325133	3.443282	3.229494	3.348277	3.309373	3.343469	3.31777	3.385373	3.417288	3.114147
RASSF7	204927_at	6.325776	6.642274	5.830819	6.026853	6.701214	7.265903	5.54585	6.126954	5.633766	5.573325	5.989784	6.262845	3.857647
SLC10A3	204928_s_at	3.557392	3.757375	3.621112	3.755433	3.877049	3.860703	4.163994	4.343985	4.436039	4.77963	4.27204	3.868137	4.059753
VAMP5	204929_s_at	6.665002	6.412089	5.93018	6.016185	6.74017	7.238461	6.022839	6.179126	6.054607	5.763272	6.024648	6.102475	4.524081
BNIP1	204930_s_at	4.312928	4.269746	4.222952	4.315402	4.60698	4.313339	4.328902	4.336308	4.303394	4.245679	4.693867	4.281528	4.422588
TCF21	204931_at	3.753905	3.760429	3.559315	3.247098	3.592872	3.242389	3.274552	2.754072	3.059731	3.320069	2.580402	2.872536	4.230198
TNFRSF11E	204932_at	5.844725	5.404618	4.990609	5.220972	5.078209	4.765488	5.1221	3.980427	5.217718	4.915103	4.046347	4.554169	4.314205
TNFRSF11E	204933_s_at	3.508471	3.497124	3.980338	3.470611	3.364521	3.20596	3.494927	3.157241	3.577316	3.484157	3.138953	3.15192	3.381047
HPN	204934_s_at	11.02868	11.03334	10.93491	11.07637	11.08377	10.69749	8.789645	8.611199	8.799236	8.951973	8.736409	8.77815	3.140112
PTPN2	204935_at	3.389431	3.291187	3.187544	3.355597	3.370887	3.386158	3.518631	3.275942	3.715871	3.62708	3.390147	3.451382	3.52443
MAP4K2	204936_at	3.133875	3.12005	3.07081	3.102282	3.121339	3.087677	3.67993	3.446881	3.379169	3.806777	3.109003	4.108029	3.320644
ZNF274	204937_s_at	7.750431	7.836921	8.494295	8.290612	7.924889	7.516538	6.675298	6.618801	6.990562	6.743371	6.625046	6.308125	6.744695
PLN	204938_s_at	2.13852	2.118001	2.091286	2.099613	2.130602	2.106982	2.108233	2.116011	2.102106	2.094821	2.102041	2.110936	7.147611
PLN	204939_s_at	2.525337	2.488836	2.613987	2.496006	2.643498	2.570026	2.478475	2.511126	2.492423	2.44944	2.503854	2.511002	8.906283
PLN	204940_at	3.423292	3.230427	3.238272	3.271072	3.409908	3.253567	3.268635	3.2727	3.296494	3.267322	3.34492	3.382476	8.22553
ALDH3B2	204941_s_at	2.674394	2.648632	2.622537	2.622194	2.662478	2.629329	2.642218	2.644922	2.633265	2.625076	2.62918	2.645079	2.558135
ALDH3B2	204942_s_at	2.822335	2.787398	2.749903	2.769284	2.822434	2.773069	2.777454	2.784975	2.771849	2.760787	2.769803	2.783647	2.580838
ADAM12	204943_at	2.227658	2.20758	2.181793	2.18841	2.210492	2.188552	2.197089	2.205185	2.215114	2.184415	2.190259	2.201713	2.117015
PTPRG	204944_at	5.049101	4.774196	5.472132	5.505008	4.521274	4.24863	3.708934	3.207369	3.717361	3.558888	4.353026	3.017903	6.569677
PTPRN	204945_at	2.737277	2.729817	2.71285	2.600897	2.728917	2.717256	2.728266	2.734367	2.769407	2.715292	2.709119	2.755357	2.684821
TOP3A	204946_s_at	3.407255	3.504254	3.173625	3.174009	3.497069	3.386244	4.174265	3.561046	3.996909	3.539592	4.330467	4.505828	3.335613
E2F1	204947_at	4.394695	4.300025	3.212614	3.621416	4.141234	3.977218	4.850535	6.134576	4.729538	5.702205	6.553164	6.202538	4.010675
FST	204948_s_at	10.19212	10.46594	10.89629	11.48306	9.584681	9.122281	10.15094	10.42473	10.42794	10.70212	10.44505	10.99123	7.844416
ICAM3	204949_at	8.759132	8.542902	9.521859	9.685471	9.502619	9.500682	7.378629	8.048607	7.51535	7.314861	7.768952	8.328305	3.468059
CARD8	204950_at	6.729847	6.470264	6.470381	6.044107	6.402238	6.544302	6.62853	6.752995	7.502663	6.952988	6.812446	7.324403	5.015512
RHOH	204951_at	2.552662	2.543909	2.523571	2.519771	2.542599	2.529282	2.851687	2.539856	2.538818	2.532275	2.522892	2.533735	2.454837
LYPD3	204952_at	1.999713	1.982501	1.959141	1.963975	1.979322	1.969574	1.980556	1.981796	1.972492	1.960361	1.967259	1.975968	1.935796
SNAP91	204953_at	2.460726	2.427605	2.38016	2.407646	2.452097	2.40206	2.416019	2.428054	2.414759	2.402601	2.413783	2.42329	7.135152
DYRK1B	204954_s_at	2.72918	2.695707	2.638078	2.667264	2.71822	2.817374	2.68058	2.68931	2.674868	2.654215	2.904829	2.688013	2.655397
SRPX	204955_at	8.223756	8.443632	8.768776	8.331323	8.575516	8.370295	7.186964	6.520243	6.272319	6.461983	8.463744	7.124515	10.37229
MTAP	204956_at	3.770455	3.79726	3.610226	3.955773	4.011398	3.630658	3.630191	4.023811	3.740362	3.254129	3.884049	4.137511	3.769861
ORC5L	204957_at	6.60567	6.908932	6.58919	6.711107	6.801663	6.882989	6.955536	7.102119	7.184584	7.025371	7.0293	7.291201	6.287058
PLK3	204958_at	3.006755	3.308374	4.612848	4.298086	3.053607	3.315139	3.158769	3.131502	2.970346	2.962176	3.032436	3.226491	3.425336
MNDA	204959_at	5.603125	5.525094	6.817395	6.338142	6.521081	5.946845	4.644329	3.885942	3.678702	4.279397	4.825677	4.936351	4.715308
PTPRCAP	204960_at	3.326504	2.93642	2.827337	3.01692	3.871945	4.1604	2.848171	2.880942	2.704707	2.700603	2.862676	2.997834	2.64569
NCF1	204961_s_at	2.173297	2.162242	2.172205	2.177438	3.782183	2.983484	2.134471	2.137352	2.130385	2.109287	2.141775	2.126928	1.946716
CENPA	204962_s_at	2.610087	2.561394	2.467858	2.473935	2.530768	2.519537	8.545472	8.40043	8.854013	8.670449	7.724351	8.263836	6.76491
SSPN	204963_at	3.685333	3.590531	3.929219	3.659298	3.872985	3.61186	3.561504	3.588631	3.57532	3.534064	5.310287	3.651937	6.316242

SSPN	204964_s_at	2.003093	1.979777	2.170344	1.977034	2.061403	2.379579	1.98634	1.994151	1.954264	1.970955	5.489104	1.987429	6.528121
GC	204965_at	14.38863	14.40187	14.34029	14.40105	14.18272	14.06818	13.40404	12.94231	13.61401	13.57299	12.36802	12.41029	6.537084
BAI2	204966_at	2.507783	2.500725	2.454383	2.477578	2.50797	2.465222	2.483564	2.497563	2.481264	2.485217	3.219852	2.490665	5.875853
SHROOM2	204967_at	5.537472	5.250151	5.56865	5.842246	5.10599	5.006766	4.019615	3.334285	3.717766	3.359635	3.989657	4.818225	5.275476
C6orf47	204968_at	3.574398	4.011962	3.690708	3.422414	3.709747	4.471517	3.4276	3.430676	3.427656	3.50378	3.688383	3.733971	3.653863
RDX	204969_s_at	3.060478	3.028152	3.132753	3.011315	3.089215	2.973201	4.079837	3.050225	3.787592	4.220811	3.048521	3.023646	6.28794
MAFG	204970_s_at	3.235997	3.279703	3.059299	3.131826	2.974888	3.334383	4.160852	3.345019	4.704309	4.525176	4.170311	3.963217	2.41096
CSTA	204971_at	6.832313	6.462879	8.933377	9.285907	9.242201	9.593614	3.389631	4.469006	3.740611	3.526415	5.130358	4.509533	3.411794
OAS2	204972_at	3.301963	3.289418	5.028157	4.467292	3.279131	3.229007	3.250608	3.278492	3.24707	3.255424	3.231321	3.037664	2.234617
GJB1	204973_at	10.58931	10.62573	10.34323	10.29027	10.67764	10.41852	9.319285	9.414713	9.340632	9.240884	8.866379	9.384223	3.518031
RAB3A	204974_at	3.380033	3.415258	3.323143	3.328567	3.659596	3.350401	3.362498	3.908775	3.146145	3.370976	3.525109	3.371252	3.646753
EMP2	204975_at	6.850874	6.916412	7.639685	7.545657	7.774394	7.493086	4.86853	4.795427	4.94988	5.175663	5.346358	5.060994	6.679017
AMMECR1	204976_s_at	6.079711	5.955704	6.190237	6.55006	6.836116	6.821296	10.16445	10.97436	10.38579	10.49171	10.54687	10.72497	6.599808
DDX10	204977_at	5.729251	5.043031	5.654347	5.581494	4.666953	4.647252	6.34997	6.453872	6.840608	6.222731	6.286709	6.333248	6.929345
SFRS16	204978_at	8.052289	7.706181	7.66827	7.773327	8.133565	8.366259	7.040949	7.597354	7.225359	7.131263	8.261801	7.679272	6.190031
SH3BGR	204979_s_at	3.588459	3.567901	3.628717	3.92693	4.582715	3.516843	3.376103	3.390414	3.522615	3.517143	5.28022	3.579373	7.319888
CLOCK	204980_at	5.969953	5.934767	6.453329	6.095902	6.117217	5.936251	6.11955	6.116537	6.251644	6.107326	6.022002	5.319233	6.340218
SLC22A18	204981_at	7.873246	7.978984	8.756828	8.973274	8.20341	8.311623	7.184953	7.596246	6.783024	7.245781	7.312368	7.813176	2.68393
GIT2	204982_at	2.628113	2.577346	2.527553	2.600974	2.578781	2.59875	3.015202	2.597346	3.04919	3.564623	2.593632	3.126863	2.297564
GPC4	204983_s_at	2.279924	2.252945	2.214633	2.247995	2.264393	2.223063	2.267464	2.268102	2.27575	2.254828	2.285292	2.278844	7.065332
GPC4	204984_at	2.590125	2.58118	2.560931	2.563984	2.594047	2.562805	2.457793	2.576092	2.565778	2.560742	5.165687	2.569879	8.146807
TRAPPC6A	204985_s_at	7.600341	7.578298	7.589638	7.377376	8.27132	7.819798	7.363536	7.33015	7.57923	7.913738	7.64511	7.700531	5.886247
TAOK2	204986_s_at	4.792474	5.016884	3.951491	4.235828	4.440844	5.043579	5.050496	4.759332	5.307572	5.090931	5.146157	5.119448	4.676955
ITIH2	204987_at	13.33736	13.31908	13.34882	13.35935	13.05771	12.63144	13.60253	13.06139	13.56407	13.5322	12.93902	13.28366	8.334701
FGB	204988_at	14.8046	14.78342	14.73055	14.90729	14.93649	14.88177	14.39139	14.34908	14.32853	14.19288	14.11755	14.15619	9.356221
ITGB4	204989_s_at	2.269483	2.246103	2.224579	2.228181	2.244972	2.222899	2.239981	2.241752	2.227596	2.220404	2.225063	2.242258	2.133066
ITGB4	204990_s_at	3.169885	3.146204	3.06717	3.126163	3.148598	3.122388	3.140128	3.140913	3.129927	3.128165	3.122973	3.360202	2.962355
NF2	204991_s_at	3.299971	3.15935	3.205442	3.237752	3.786777	3.448568	3.143571	3.670407	3.34626	3.301277	3.369795	3.313488	2.877499
PFN2	204992_s_at	7.081411	6.887724	7.951735	7.786993	7.857831	6.895228	8.576643	7.951879	8.391664	8.402108	9.077447	8.033979	12.20662
GNAZ	204993_at	2.418173	2.414402	2.339825	2.349736	2.341717	2.394865	2.411048	3.743597	3.193873	3.256849	3.286132	3.34171	4.340748
MX2	204994_at	2.784758	2.885105	5.172868	4.949288	2.653929	3.10254	2.395708	2.345445	2.391841	2.38071	2.393909	2.339422	2.287361
CDK5R1	204995_at	3.300264	3.255437	3.092477	3.219612	3.414694	3.178798	3.22678	3.700312	4.070033	3.989057	4.203894	3.354346	9.116621
CDK5R1	204996_s_at	2.054951	2.05433	2.027344	2.02926	2.043006	2.026949	2.033065	2.040523	2.02912	2.028252	2.027627	2.03425	1.9872
GPD1	204997_at	5.708263	5.577702	6.037485	5.868469	4.737065	4.220347	4.173482	3.88785	3.551431	3.565678	4.147716	3.503077	2.367505
ATF5	204998_s_at	12.44343	12.30366	11.26636	11.25314	11.27576	10.92263	9.491521	10.06159	10.54662	10.17821	9.217041	9.230066	4.866502
ATF5	204999_s_at	10.79766	10.59021	9.961825	10.15461	10.12928	10.02297	7.824743	8.812562	8.532054	8.241077	7.997288	8.216251	3.292027
DDX3Y	205000_at	9.26225	8.96699	9.705403	9.690088	2.63482	2.558921	9.499078	9.452244	9.767001	9.699888	2.822396	9.404594	9.820879
DDX3Y	205001_s_at	5.134871	4.704057	5.786627	5.794786	2.624117	2.487554	5.906444	5.984857	5.924516	5.766342	2.499612	6.178408	5.989159

AHDC1	205002_at	3.263346	3.210522	2.527877	3.070269	3.551385	3.858186	3.210054	3.468784	2.90379	2.959404	4.387213	3.327055	4.038139
DOCK4	205003_at	9.204972	9.008634	8.882942	8.862987	9.082421	8.606664	7.875594	7.612299	8.107137	7.837964	7.646638	7.653789	8.687428
NKRF	205004_at	6.434392	6.495678	6.102231	6.498266	6.508521	6.470329	6.1007	6.438437	6.381825	6.373706	6.565684	6.503297	6.469567
NMT2	205005_s_at	8.429357	8.046235	7.750436	8.025897	7.461073	7.590955	6.761438	6.779946	7.026275	6.972275	6.703211	6.983651	6.255868
NMT2	205006_s_at	8.831009	8.691062	7.889165	8.133907	8.48762	8.345448	6.591636	7.389967	6.928373	6.541767	6.97949	7.3227	5.952727
CIB2	205007_s_at	2.043088	2.031262	1.964545	1.985666	2.032092	2.018144	2.144016	2.030578	2.023808	2.040003	2.01489	2.226476	3.327791
CIB2	205008_s_at	2.864628	2.845861	2.440386	2.772235	2.848374	2.897597	3.67287	5.146996	4.227655	4.379269	5.565169	5.38293	6.00947
TFF1	205009_at	2.363088	2.34691	2.325483	2.327398	2.357892	2.330742	2.34213	2.343788	2.330685	2.327163	2.325587	2.338604	2.235424
GNL3L	205010_at	7.301326	6.717735	6.367881	6.459713	6.373916	6.405753	6.773223	6.685114	7.291515	6.582501	6.367002	6.851405	6.943331
LOH11CR2A	205011_at	3.572953	3.673068	3.734548	3.653554	4.380819	4.906343	3.661249	3.715584	2.928442	3.067954	4.203127	3.802334	5.476513
HAGH	205012_s_at	9.908851	9.775345	10.20034	10.3657	9.845904	9.670376	9.297159	9.117052	9.250798	9.067454	8.453855	8.983897	6.104706
ADORA2A	205013_s_at	4.400512	4.466897	4.158036	4.240965	4.383383	5.62022	4.440809	4.380479	4.343797	4.328923	4.564811	4.765957	4.356286
FGFBP1	205014_at	2.553271	2.523222	2.484667	2.502107	2.532842	2.489047	2.5118	2.52118	2.508378	2.493023	2.50218	2.517639	2.415817
TGFA	205015_s_at	2.544036	2.51398	2.457314	2.49549	2.527536	2.491902	2.502679	2.513113	2.498918	2.486357	2.496282	2.507348	2.347352
TGFA	205016_at	2.348411	2.477659	4.093842	3.403272	2.83532	2.607704	2.300271	2.319136	2.30419	2.290293	2.272713	2.285289	3.172327
MBNL2	205017_s_at	2.509617	2.520469	3.031944	2.623287	2.473107	2.511654	2.439401	2.80421	2.583398	2.553652	2.446988	2.443863	3.547523
MBNL2	205018_s_at	2.574633	2.542654	2.511295	2.530141	2.555917	2.527038	2.542119	2.552454	2.573316	2.594733	2.537076	2.559433	5.755555
VIPR1	205019_s_at	7.194668	7.402081	8.552397	8.751955	7.790168	7.453399	4.134728	4.603575	3.892294	3.539236	4.588148	4.880248	3.153961
ARL4A	205020_s_at	5.935273	5.690667	5.896662	6.073237	6.441194	6.759084	9.629594	10.21404	9.753598	10.12735	9.722654	9.712197	7.782271
FOXN3	205021_s_at	2.9946	3.581006	3.258323	2.817896	3.148394	3.277635	3.275748	3.569687	3.257965	3.262445	2.838179	3.23703	3.161097
FOXN3	205022_s_at	9.169792	9.18675	9.459657	9.202698	8.953807	8.544107	8.402272	8.349548	8.933085	9.092933	8.511885	8.304434	8.461808
RAD51	205023_at	2.917561	2.845153	2.766587	2.818164	2.901728	2.830499	3.10967	3.035618	2.863573	2.872612	2.847783	2.870139	2.601766
RAD51	205024_s_at	4.431034	4.384163	3.79405	4.243579	4.39752	4.974732	7.911755	8.55904	7.960794	8.322198	7.942123	8.484394	6.125701
ZBTB48	205025_at	6.980025	6.797037	6.387678	6.923969	7.208278	7.04653	5.873803	6.188838	5.599311	5.610387	6.374403	6.385497	4.869821
STAT5B	205026_at	2.770625	2.801917	2.887984	2.795964	2.853834	2.608361	3.718063	3.905722	3.825736	3.894727	3.431899	2.989381	2.654381
MAP3K8	205027_s_at	4.956734	4.385887	5.971421	5.299912	4.991795	4.853454	4.018845	3.925557	3.923757	3.949894	4.536667	3.743315	4.16107
TRO	205028_at	3.671071	3.529392	3.478211	3.530817	3.684509	3.624126	3.636163	3.628988	3.615425	3.510554	4.124521	3.917331	4.899684
FABP7	205029_s_at	3.122082	2.997953	2.924306	2.997704	3.124233	2.954857	3.013847	3.021092	3.019484	2.999567	3.048087	3.078819	8.940959
FABP7	205030_at	2.307259	2.294902	2.262268	2.275247	2.293872	2.272607	2.283224	2.294828	2.278733	2.270399	2.8862	2.293763	9.507555
EFNB3	205031_at	2.099063	2.082971	2.065188	2.03034	2.08223	2.070595	2.320634	2.072796	1.790058	2.054097	2.830577	2.213178	6.693075
ITGA2	205032_at	2.45801	2.439355	2.412955	2.419308	2.440524	2.418917	2.428053	2.437615	2.424977	2.42155	2.421852	2.432395	2.448711
DEFA1	205033_s_at	2.48369	2.459815	3.197756	5.551725	2.478759	2.426901	8.809978	8.238847	8.833767	9.525079	9.041168	10.18416	5.602971
CCNE2	205034_at	3.771791	3.481112	3.963537	4.634395	3.519136	3.861743	7.845733	7.165423	7.960636	8.277357	6.503256	6.816243	6.368622
CTDP1	205035_at	5.01445	5.107654	5.328537	5.106081	5.017216	5.527153	5.20701	5.185014	5.266785	5.704624	5.237719	5.799287	4.376201
LSM6	205036_at	7.392818	7.233173	7.676219	7.912255	7.233228	6.990644	8.262314	7.771494	8.241355	8.425133	7.825616	8.042883	7.908309
RABL4	205037_at	5.692449	5.579078	6.146884	5.996062	6.466811	6.299119	6.080917	6.494208	6.500458	6.401704	6.925731	6.390713	6.484029
IKZF1	205038_at	2.623987	2.782842	2.752311	2.765818	2.721422	2.762902	4.557669	4.01147	4.544444	4.628959	3.028067	2.891038	2.15126
IKZF1	205039_s_at	2.843413	3.168374	2.760712	2.810106	2.837334	3.291201	6.205462	6.208769	6.734116	6.779619	7.068348	6.536376	2.768397

ORM1	205040_at	14.83922	14.79602	15.02458	15.20926	15.23324	15.13603	9.636709	10.33683	10.08804	9.482107	9.844091	10.19622	3.721962
ORM1	205041_s_at	14.94056	14.93258	15.03307	15.29773	15.14948	15.04085	9.822436	10.28989	10.72218	10.19375	9.96583	10.07368	3.822774
GNE	205042_at	10.65972	10.63594	11.27981	11.30566	10.91105	10.40335	7.595924	7.355268	7.503423	7.616507	6.930707	7.624618	6.851602
CFTR	205043_at	2.710187	2.978302	2.759264	3.342856	2.695472	3.693324	2.629583	2.676807	2.3464	2.502294	2.66235	2.634385	2.540017
GABRP	205044_at	2.548297	3.058017	2.514329	2.516289	2.549096	2.520207	2.528928	2.531566	2.516782	2.49983	2.512875	2.526152	3.936352
AKAP10	205045_at	2.432717	2.399874	2.373421	2.377527	2.407321	2.371455	2.609974	2.397445	2.474039	2.377547	2.38128	2.389951	2.943537
CENPE	205046_at	2.089089	2.069009	2.043868	2.048936	2.071063	2.048334	8.341319	7.831393	9.112446	8.827428	6.999711	7.573722	6.50827
ASNS	205047_s_at	4.090525	4.193908	4.391613	4.479168	3.864728	4.324518	6.966469	7.7323	7.241771	7.534811	8.04538	8.468799	7.884748
PSPH	205048_s_at	8.971601	8.983749	4.593325	4.697353	8.514826	8.038478	8.185285	9.827877	8.039364	11.30233	6.131443	9.418676	5.484286
CD79A	205049_s_at	2.763133	2.722709	2.60888	2.696636	2.701572	3.533215	2.707797	2.870839	2.657692	2.631543	2.689182	3.060425	2.465423
MAPK8IP2	205050_s_at	2.632231	2.626245	2.562607	2.59749	2.622144	2.620555	2.62288	2.621833	2.618066	2.608667	2.608884	2.617246	2.628144
KIT	205051_s_at	2.717233	2.774723	3.046522	2.629025	3.019314	3.575854	6.184272	4.813076	6.470336	6.2654	4.295511	5.247358	7.531677
AUH	205052_at	8.869583	8.532228	8.943415	9.161266	8.602315	8.082392	8.509601	8.41444	8.938961	8.853858	8.035105	7.77317	7.75077
PRIM1	205053_at	3.894867	3.264596	4.576014	4.603143	3.920621	4.218839	7.869036	7.545001	7.884505	7.964382	6.982757	7.696004	5.743461
NEB	205054_at	2.680849	2.341579	2.754108	2.289254	2.264841	2.235379	2.246075	2.276412	2.25468	2.238156	8.246919	2.27113	10.4052
ITGAE	205055_at	7.341487	7.375351	7.445244	7.65242	7.897185	8.146474	8.773564	8.719395	8.704215	8.364382	9.222404	9.035525	9.126578
GPR162	205056_s_at	2.693946	2.703265	2.501755	2.789658	2.812217	4.505496	2.581006	2.690984	2.590306	2.645234	2.676708	2.663119	2.782779
IDUA	205057_s_at	1.932653	1.913816	1.894549	1.899542	1.916902	1.898717	1.910009	1.912812	1.900964	1.896021	1.895865	1.906404	1.848387
SLC26A1	205058_at	3.058672	3.076865	2.958178	3.060199	3.127282	3.226435	2.942808	3.391039	3.03426	2.991182	3.089312	3.044834	2.706074
IDUA	205059_s_at	4.726982	4.765106	4.153857	4.242815	4.191073	4.843357	3.723925	4.169879	3.766794	4.112325	4.169381	4.168421	4.450175
PARG	205060_at	5.989409	6.127875	5.950294	5.998898	5.967584	5.913595	6.306786	6.378021	6.351766	6.146397	6.096683	6.61648	6.447275
EXOSC9	205061_s_at	5.847521	5.962391	5.98257	6.094743	6.220532	5.933648	8.149442	8.59383	8.227766	8.247109	7.846419	8.431467	7.096311
ARID4A	205062_x_at	6.796802	6.354842	7.415594	7.332211	6.968985	6.178607	6.517626	6.097883	6.909113	6.474485	5.808648	5.842645	6.457996
SIP1	205063_at	6.289877	5.625546	6.118801	5.955843	5.911498	5.601013	7.012989	6.896524	7.051744	7.050621	7.179747	7.042201	7.174895
SPRR1B	205064_at	2.103255	2.081904	2.057095	2.0633	2.085928	2.063273	2.074157	2.080452	2.06763	2.060035	2.06609	2.074645	3.512688
ENPP1	205066_s_at	10.36879	10.08776	10.40975	10.44742	9.751767	9.336368	7.172686	7.17446	6.964252	6.899081	7.321189	7.409037	6.821413
IL1B	205067_at	2.270005	2.241908	2.51791	2.242116	2.254755	2.883813	2.231539	2.245887	2.240699	2.231333	2.225442	2.234732	2.196995
ARHGAP26	205068_s_at	5.61043	5.323791	5.027382	5.356441	5.052976	5.724704	4.698184	4.803185	4.994838	4.044116	5.06275	4.644732	4.97033
ARHGAP26	205069_s_at	4.021986	3.619829	3.37844	3.608736	3.863877	3.410061	3.499577	3.403375	3.52114	3.578649	3.664074	3.686421	3.212056
ING3	205070_at	7.612599	7.523874	7.480653	7.756622	8.148518	7.75529	7.576659	7.983643	7.680849	7.523458	7.552538	7.734256	7.997816
XRCC4	205071_x_at	3.042529	3.050717	3.034835	3.005136	3.011752	3.02151	4.491447	3.052382	4.601509	4.000342	3.335219	3.043331	4.133656
XRCC4	205072_s_at	4.422041	4.012152	4.128489	4.179244	4.214504	4.153938	4.472815	4.816536	5.844274	5.09625	4.420731	4.539989	4.462547
CYP2J2	205073_at	8.946801	8.960027	9.707853	9.774681	9.55937	8.904706	7.797139	7.479037	8.154369	7.966441	7.119459	7.031792	4.683224
SLC22A5	205074_at	3.269031	3.226618	3.226171	3.228153	3.35218	3.203848	3.399488	3.252561	3.902008	3.925048	3.335534	3.226886	3.669128
SERPINF2	205075_at	10.95754	11.10658	10.81502	10.84507	11.15402	11.11557	10.60546	10.45778	10.38968	10.22646	10.53353	10.77105	3.820046
MTMR11	205076_s_at	2.164134	2.149738	2.121111	2.116858	2.137124	2.127362	2.139341	2.144403	2.131314	2.123232	2.296404	2.13609	4.53779
PIGF	205077_s_at	8.694418	8.59872	8.751072	8.807014	8.570347	8.473481	8.97094	9.040775	8.944116	9.09694	8.905219	9.218918	8.540082
PIGF	205078_at	6.168569	6.215953	6.484206	6.479205	4.993867	4.198909	7.427124	6.128182	7.392656	7.455599	6.165034	6.746557	7.239269

MPDZ	205079_s_at	10.32155	10.01294	10.28012	10.20924	9.835911	9.210868	8.772764	8.280699	8.570532	8.292992	8.474815	8.546453	7.757478
RARB	205080_at	3.2463	3.116285	4.109164	3.63245	3.534232	3.637997	3.463393	3.043542	3.516052	3.462612	3.970729	3.257551	5.491265
CRIP1	205081_at	4.795058	5.263061	4.521657	3.820982	5.697781	6.454803	3.436877	3.798517	3.368381	3.679313	4.9145	3.737567	7.120752
AOX1	205082_s_at	9.310944	9.358864	11.30851	10.79504	9.216027	8.194566	3.11274	2.78123	2.586073	2.743732	2.761871	2.806377	2.547819
AOX1	205083_at	12.15887	12.09195	12.74455	12.66957	12.41755	12.19586	2.945359	2.464823	2.714245	2.094644	2.230796	2.033347	2.809957
BCAP29	205084_at	4.899244	4.909995	5.64907	5.841476	5.259523	4.804012	6.732994	6.572978	6.565713	6.702323	5.638406	6.082631	6.919344
ORC1L	205085_at	2.415503	2.399832	2.358567	2.363821	2.376412	2.361458	6.550726	6.213305	6.58294	6.722935	4.919409	5.906507	2.665604
NCAPH2	205086_s_at	5.704182	5.636539	4.824961	5.199326	5.57622	5.64423	6.361698	7.359096	6.431423	6.638993	7.332981	7.492	5.044789
RWDD3	205087_at	8.729346	8.492659	8.568321	8.843672	8.137342	7.735508	8.860251	8.066223	8.682273	8.748758	7.704436	8.166478	8.304187
CXorf6	205088_at	4.143823	4.16641	4.223455	4.529759	4.193696	4.185501	4.744295	4.256359	4.309073	4.839337	4.906295	4.263275	5.095174
ZNF7	205089_at	6.357603	5.971165	6.263919	6.150593	6.138106	5.770972	6.125111	5.970844	6.163935	6.245416	6.023943	6.103135	6.886425
NAGPA	205090_s_at	5.459468	5.536585	5.252184	5.486117	5.571607	5.613786	5.718026	5.948072	5.777026	5.647476	5.498813	6.798679	3.619384
RECQL	205091_x_at	6.630051	6.242747	6.343177	6.236433	6.285132	6.493453	7.621851	7.058771	7.548617	7.493223	6.681014	7.045536	7.852811
ZBTB1	205092_x_at	4.658787	4.134644	3.856679	3.793397	4.035289	3.950516	4.09288	4.137997	3.832621	3.700827	4.506998	4.37299	5.21154
PLEKHA6	205093_at	5.788707	6.053132	3.622615	4.575788	3.959668	4.420778	3.116263	3.069814	2.643815	3.369081	3.180161	3.044908	2.791278
PEX12	205094_at	6.485005	6.349507	6.406707	6.083724	6.189112	5.803928	5.914005	5.596263	5.859553	5.760504	5.046942	5.378864	6.301827
ATP6V0A1	205095_s_at	2.081486	2.070908	2.219263	2.052284	2.074875	2.059812	3.909363	2.075332	3.783755	4.494928	2.057846	2.063783	4.223462
POM121	205096_at	3.197538	2.934132	2.912407	2.917211	2.948449	2.893244	3.019918	3.052317	2.92948	2.910883	2.929072	2.936514	2.806111
SLC26A2	205097_at	2.987235	3.174105	3.023321	3.206947	3.082461	2.869757	5.701113	5.261703	6.295195	5.677584	5.869493	5.458673	7.551748
CCR1	205098_at	4.047717	3.778057	6.028289	5.680545	5.563732	4.646439	5.143801	4.380474	4.693474	4.261221	3.584658	4.606863	3.352091
CCR1	205099_s_at	5.892151	5.73842	6.179497	6.509879	6.821584	6.833055	6.035631	5.801132	4.762576	5.328996	5.201008	5.949476	3.500823
GFPT2	205100_at	2.193728	2.185385	2.350692	2.15289	2.184821	2.172383	2.168872	2.183738	2.16039	2.1709	2.167251	2.177116	4.285273
CIITA	205101_at	4.493158	3.958428	3.567694	2.91782	4.595662	5.292257	2.972849	3.18006	3.01745	3.245882	2.905436	3.108254	2.513966
TMPRSS2	205102_at	4.483118	4.47776	4.447972	4.568904	4.425645	4.501118	4.409027	4.443507	4.316256	4.415558	4.406424	4.415053	4.208014
C1orf61	205103_at	1.997022	1.978589	1.961312	1.965558	1.98559	1.963921	1.975852	1.977954	1.966188	1.960608	1.962065	1.97239	8.023614
SNPH	205104_at	2.293305	2.278421	2.256135	2.245343	2.298191	2.262908	2.271917	2.278077	2.270266	2.259231	2.277974	2.271758	2.550974
MAN2A1	205105_at	8.711805	8.610606	8.904278	9.047564	8.360989	7.734652	8.545739	7.400353	8.532262	8.482349	7.085577	7.480632	7.598406
MTCP1	205106_at	2.790144	2.752271	2.709476	2.736931	2.771634	2.737093	2.743419	2.750215	2.742959	2.739039	2.734599	2.750372	2.610314
EFNA4	205107_s_at	3.065007	3.04509	2.868104	2.931854	3.040778	2.954779	2.978569	2.977061	2.967448	3.078013	3.322724	3.289495	4.585103
APOB	205108_s_at	14.57336	14.59198	14.50907	14.55034	14.1212	13.87723	14.63328	14.22072	14.49657	14.57298	14.14159	14.36314	10.27564
ARHGEF4	205109_s_at	2.430302	2.405187	2.377913	2.39056	2.404533	2.415822	2.386732	2.399353	2.383162	2.370399	2.388409	2.393397	4.251606
FGF13	205110_s_at	2.878328	2.841787	2.827651	2.828754	3.475735	2.954335	2.846745	2.8492	2.835677	2.740318	6.041362	2.849433	9.7462
PLCE1	205111_s_at	3.697744	3.683833	3.62233	3.645348	3.883445	4.112946	3.624206	3.663015	3.666737	3.624459	7.491355	3.699085	8.215589
PLCE1	205112_at	3.03655	3.011103	2.911022	2.925377	3.005365	2.92285	3.007334	2.973904	2.980458	2.970196	5.007158	2.993863	6.607849
NEFM	205113_at	2.41324	2.379198	2.337442	2.361078	2.396631	2.361944	2.373181	2.381237	2.363001	2.344337	7.761941	2.375836	11.52855
CCL3	205114_s_at	4.765305	4.787088	6.308143	6.264573	7.953853	7.743353	2.951956	2.931885	2.544887	3.211328	2.781825	2.968085	2.715868
RBM19	205115_s_at	4.157033	4.144767	4.124466	4.04383	3.594888	3.742575	4.402034	4.949231	4.754126	4.581146	4.647309	5.306409	4.080306
LAMA2	205116_at	4.132139	4.164845	4.928067	4.173898	4.174436	4.643131	3.91994	3.94142	3.551026	3.945369	4.153187	3.676443	7.716646

FGF1	205117_at	2.117169	2.094128	2.075397	2.108058	2.103758	2.083426	2.079938	2.09656	2.081138	2.073907	2.083495	2.091184	3.748739
FPR1	205118_at	3.385555	3.297998	3.288879	3.364906	3.622426	3.336942	3.260685	3.261107	3.26172	3.234157	3.328215	3.350903	2.970657
FPR1	205119_s_at	2.944437	2.932188	4.788879	4.526835	4.02136	3.938284	3.598172	3.603083	3.589686	2.918422	3.180875	3.357782	2.72737
SGCB	205120_s_at	4.572699	4.166009	3.635419	3.745037	3.40303	3.754184	2.9952	3.323326	3.174662	3.089898	4.903278	3.224921	8.345983
SGCB	205121_at	2.98121	2.895835	2.830364	2.838484	2.959927	2.876104	2.821915	2.88974	2.859894	2.824058	2.894549	2.949189	5.425286
TMEFF1	205122_at	3.404279	3.279757	3.190844	3.298786	3.570254	3.234809	3.367791	3.299738	4.150559	3.867798	5.884507	3.945487	10.27839
TMEFF1	205123_s_at	2.628594	2.578771	2.531339	2.565853	2.618014	2.546267	2.579084	2.589325	2.576317	2.556566	2.572541	2.592935	8.313263
MEF2B	205124_at	2.416321	2.411591	2.352173	2.466747	2.402187	2.389787	2.400741	2.432169	2.403135	2.396302	2.472643	2.404614	2.281318
PLCD1	205125_at	5.713646	5.183352	5.423947	5.435148	5.608501	5.893451	5.579957	5.453286	5.413871	5.423148	5.523769	5.910025	4.93358
VRK2	205126_at	5.688134	5.685085	5.667606	5.68466	5.710388	5.554679	7.560803	7.057501	7.638345	7.51114	6.384783	7.121636	6.141058
PTGS1	205127_at	1.947434	1.934596	1.915131	1.919769	1.93392	1.924042	1.953301	1.935322	2.174392	1.927339	1.918802	1.928568	1.944302
PTGS1	205128_x_at	2.835356	2.866028	3.322148	4.115841	3.751787	4.566497	4.375294	5.592832	4.814218	5.14113	4.585724	6.136478	2.937748
NPM3	205129_at	4.108453	4.081023	3.025836	4.033418	4.079972	4.07062	4.295437	4.11764	4.071574	4.117027	6.150763	4.263111	4.435139
RAGE	205130_at	3.345099	3.284517	3.302678	3.319531	3.328819	3.321982	3.326733	3.697531	3.322402	3.312268	3.665629	3.432096	3.816306
CLEC11A	205131_x_at	2.111187	1.990882	1.946533	1.973516	1.992026	2.670836	1.965601	1.978052	1.96034	1.966692	4.112391	2.483407	3.781142
ACTC1	205132_at	2.979277	2.917969	2.857225	2.924257	2.940523	2.883731	2.924069	2.929274	2.918458	2.911707	12.94443	2.949355	13.8226
HSPE1	205133_s_at	10.34737	10.20012	11.21789	11.19977	10.23264	9.678341	11.0197	10.25448	11.09397	11.11629	10.2243	10.28438	10.67401
NUFIP1	205134_s_at	4.33974	4.317077	4.344604	4.304216	4.609671	5.48464	4.488328	5.417414	4.252364	4.313788	5.122705	4.778474	4.244981
NUFIP1	205135_s_at	4.44544	4.654172	4.711937	4.797295	4.621495	4.482774	5.411683	5.48428	5.411876	5.441979	5.222747	5.347757	4.578918
NUFIP1	205136_s_at	3.901903	3.894206	3.977067	3.976812	3.949191	3.882821	4.419314	4.065341	4.524281	4.432812	4.4458	4.061651	5.356782
USH1C	205137_x_at	2.190941	2.202999	2.158556	2.18323	2.197026	2.222752	2.200364	2.203185	2.183389	2.176893	2.188703	2.362399	2.42414
UST	205138_s_at	2.406003	2.37776	2.342924	2.363981	2.382813	2.354591	2.368241	2.377062	2.364666	2.35403	2.36128	2.370976	2.338888
UST	205139_s_at	2.757186	2.606623	3.280037	2.693091	2.669083	2.641157	3.591079	2.65977	3.513265	3.006567	3.239328	2.87329	5.891189
FPGT	205140_at	5.92046	5.825514	6.049178	5.748204	5.514976	5.089037	5.885345	5.231372	5.98382	6.067763	4.996857	5.279872	6.423416
ANG	205141_at	11.63148	11.61802	12.87755	12.8627	11.93776	11.64952	7.959696	8.052435	8.563724	8.341	7.620255	8.8322	3.487811
ABCD1	205142_x_at	4.588782	4.467635	3.954733	4.321876	4.176223	3.714795	4.358956	4.079855	3.697024	4.022535	4.095822	4.028401	3.5754
NCAN	205143_at	2.14542	2.125166	2.105677	2.107104	2.128491	2.106291	2.117588	2.125334	2.111509	2.104383	2.111099	2.121108	5.974455
MFSD7	205144_at	2.322847	2.314496	2.28826	2.298202	2.327312	2.297303	2.311982	2.317318	2.306112	2.300711	2.301265	2.311187	2.229305
MYL5	205145_s_at	6.879409	7.093214	5.80652	5.831904	6.320176	6.576884	4.33569	5.36591	4.242627	4.521566	8.654414	5.482739	7.384995
APBA3	205146_x_at	4.610123	4.664776	4.399156	4.400577	4.410563	4.608885	4.412959	4.414071	4.402918	4.395746	4.39747	4.408062	4.372797
NCF4	205147_x_at	2.587085	2.836443	2.508	2.812606	2.926776	3.045803	2.931204	2.879861	2.9271	2.854997	2.85175	3.248532	2.720238
CLCN4	205148_s_at	1.842849	1.830252	1.813819	1.821836	1.828599	1.822142	1.872833	2.319029	1.880329	1.830709	2.469234	2.030181	3.390395
CLCN4	205149_s_at	2.214791	2.194101	2.17179	2.17604	2.197714	2.175332	2.247446	2.1912	2.18723	2.210833	2.178226	2.216262	2.907228
KIAA0644	205150_s_at	2.646651	2.602713	2.584797	2.606196	3.215601	2.610048	2.586601	2.597372	2.58446	2.572077	6.988142	2.6119	8.672054
KIAA0644	205151_s_at	2.837829	2.82959	2.909357	2.898757	2.844302	3.000703	2.816931	2.828913	2.813253	2.807211	6.494338	2.823504	8.219391
SLC6A1	205152_at	10.80196	10.77442	10.94523	10.71126	9.413637	8.557829	2.824265	2.215684	2.201945	2.180566	2.195135	2.208502	6.752348
CD40	205153_s_at	3.729114	3.908496	4.459144	3.823857	3.457209	3.357403	3.015489	3.114738	2.769728	3.081952	3.15365	3.011221	2.763133
LRRN2	205154_at	2.665464	2.625485	2.601423	2.606639	2.622566	2.613273	2.675346	3.001197	2.636076	2.782349	2.817816	2.632686	2.763845

SPTBN2	205155_s_at	3.691015	3.793488	5.074992	5.44685	3.905996	3.907525	3.54915	3.501354	2.808422	2.900047	2.790942	2.914276	3.50818
ACCN2	205156_s_at	2.315215	2.30596	2.287992	2.293026	2.303808	2.29439	2.294884	2.305632	2.294946	2.290868	2.290792	2.297061	2.966947
KRT17	205157_s_at	3.596947	3.521517	3.427693	3.464111	3.76881	3.508705	3.499631	3.521709	3.500781	3.232721	3.504141	3.517318	5.833878
RNASE4	205158_at	9.472554	9.410053	11.34257	10.60305	9.192711	7.855159	9.119639	7.227901	9.407037	9.010601	6.295707	7.186382	6.168485
CSF2RB	205159_at	3.040798	2.990086	4.831942	3.778438	4.482411	3.135729	5.821286	5.978121	6.116824	5.459108	3.406862	5.602503	3.368961
PEX11A	205160_at	7.952715	7.832851	7.859414	7.803166	7.934618	8.53653	6.380005	6.3291	6.335171	6.143349	6.090088	5.921537	5.93998
PEX11A	205161_s_at	3.415278	3.25789	4.331189	4.530712	3.841878	3.107293	2.701662	2.742018	2.954766	2.728738	2.702268	2.696307	2.650371
ERCC8	205162_at	2.460073	2.411196	2.537094	2.420381	2.430273	2.420833	2.62404	2.46885	3.125715	2.944462	2.638614	2.461812	4.103296
MYLPF	205163_at	2.168224	2.156164	2.137513	2.142417	2.154476	2.141592	2.152464	2.153845	2.140156	2.192182	10.93975	2.245469	12.07529
GCAT	205164_at	8.825635	8.786303	8.268948	8.710201	8.807403	8.918545	7.980164	8.949915	8.097038	7.77999	8.895864	9.023533	4.522709
CELSR3	205165_at	2.215668	2.199173	2.16885	2.180609	2.20518	2.183354	2.193148	2.199772	2.192547	2.437167	2.459697	2.229447	2.473045
CAPN5	205166_at	2.768985	2.830021	2.995411	3.117484	2.803934	2.775164	2.721112	2.75879	2.727432	2.735078	2.744521	2.737021	2.62357
CDC25C	205167_s_at	2.538528	2.49744	2.383999	2.460134	2.505844	2.446454	6.335572	6.23461	7.07854	6.957666	6.232578	7.070642	4.706473
DDR2	205168_at	3.439667	3.320278	3.762065	3.325395	3.35159	3.299568	3.27939	2.830792	3.084587	2.792731	4.159899	3.312923	7.514221
RBBP5	205169_at	5.991481	5.879613	6.161218	6.013568	6.055171	6.017841	6.024771	6.384411	5.855903	6.035461	6.140817	6.37645	5.920638
STAT2	205170_at	2.347719	2.311476	2.315639	2.299682	2.338772	2.279587	2.389023	2.316476	2.320794	3.092807	2.311505	2.316252	3.572006
PTPN4	205171_at	5.538719	5.103367	5.541	5.754579	6.149082	5.747123	5.671753	5.547036	6.009783	6.327048	5.668142	5.868678	5.143884
CLTB	205172_x_at	6.032865	6.01762	5.965024	5.802488	6.015008	6.072489	5.391421	5.848484	5.444677	5.427088	5.996428	5.997809	6.07728
CD58	205173_x_at	6.078771	5.614713	6.283078	6.012804	5.562614	4.870424	8.827477	7.551137	8.718122	8.741352	7.110853	7.500354	6.058084
QPCT	205174_s_at	3.739533	3.406593	3.776085	3.87328	3.752727	3.734174	3.674819	3.552956	3.825179	3.599522	4.013705	4.14887	6.560692
KHK	205175_s_at	7.356556	7.213891	5.854418	6.28231	5.97949	4.0214	3.246475	3.246681	2.889222	2.780556	2.782421	3.486192	2.386758
ITGB3BP	205176_s_at	4.938381	4.956239	6.166673	6.348987	6.647679	6.508478	7.482248	8.018815	8.728215	8.192948	8.103419	7.995816	8.093014
TNNI1	205177_at	2.836886	2.80262	2.763479	2.77864	2.822929	2.771087	2.792961	2.805755	2.787496	2.777077	9.20686	2.801143	11.16504
RBBP6	205178_s_at	7.586899	7.395761	7.506962	7.581355	7.3935	7.287684	7.125521	7.306593	7.48003	7.397182	7.272163	7.566778	7.29842
ADAM8	205179_s_at	2.467402	2.402942	2.355719	2.377209	2.425191	2.502959	2.395356	2.399783	2.385689	2.36606	2.391387	2.414861	2.259637
ADAM8	205180_s_at	3.046617	3.145604	3.102025	3.336833	3.596726	3.176814	2.726154	3.143712	2.453136	3.123021	2.643282	2.782663	2.579338
ZNF193	205181_at	2.579303	2.553593	2.527654	2.543239	2.555984	2.540725	2.553547	2.553899	2.543548	2.608619	2.536163	2.743886	4.515825
ZNF324	205182_s_at	2.350557	2.446268	2.28469	2.321537	2.36939	2.333879	2.341404	2.343957	3.074108	2.60806	4.263264	2.80679	3.148628
HNRPD	205183_at	2.809034	2.781896	2.730862	2.758663	2.831474	2.750847	2.764707	2.778657	2.761338	2.740431	2.768023	2.802735	2.541118
GNG4	205184_at	2.152128	2.130918	2.1066	2.11252	2.134901	2.119385	2.120651	2.126423	2.117359	2.108191	2.113116	2.121059	2.115996
SPINK5	205185_at	2.225985	2.211119	2.189448	2.194327	2.21387	2.193365	2.206314	2.208174	2.196229	2.188876	2.194383	2.20534	2.152016
DNALI1	205186_at	5.776865	6.002144	4.271673	4.103902	5.50713	5.180939	3.385428	4.084654	3.837972	3.425462	4.105856	4.116152	3.880441
SMAD5	205187_at	7.935321	7.383638	7.765009	7.826614	7.977139	7.600519	7.712078	7.296888	7.219952	7.118031	8.172345	7.856391	8.37289
SMAD5	205188_s_at	2.921174	2.314723	4.535279	3.656062	2.742544	2.88685	4.193244	2.663175	3.851548	3.498595	2.885219	2.617608	6.276052
FANCC	205189_s_at	7.99003	8.299643	7.569368	7.94748	6.49045	6.379559	5.43152	5.734618	5.271066	5.716473	5.628569	5.843034	4.792506
PLS1	205190_at	8.519996	8.449659	8.448438	8.338798	7.610484	6.94194	6.529403	6.286236	7.205485	7.205922	5.416215	6.121088	3.195798
RP2	205191_at	6.638001	6.333323	7.214795	7.010175	6.435582	6.055642	7.051568	6.530513	6.70462	6.734051	6.278816	6.536806	7.173728
MAP3K14	205192_at	4.218214	4.22481	4.142102	4.095992	4.40418	4.533596	3.217047	3.218446	3.258431	3.219233	3.199289	3.211785	2.714021

MAFF	205193_at	4.47281	3.882186	5.584089	5.339917	4.725981	4.73696	3.62264	3.780712	3.75464	3.673731	3.755449	3.773002	3.760563
PSPH	205194_at	7.02375	6.994239	5.966501	6.182277	6.68361	6.694454	9.105704	9.282722	8.775868	8.863722	8.666818	9.59228	6.559896
AP1S1	205195_at	2.715693	2.728718	2.73145	2.718221	2.750006	2.681064	2.82538	2.746795	2.734567	2.706646	2.728664	2.741785	3.442332
AP1S1	205196_s_at	2.46028	2.452332	2.402019	2.429157	2.453131	2.431185	2.448629	2.451762	2.436863	2.441963	2.435349	2.470737	3.605361
ATP7A	205197_s_at	3.824695	3.812498	3.792642	3.739555	3.891854	3.923684	7.825184	7.008515	4.938352	5.798133	6.583982	5.265708	7.427669
CA9	205199_at	2.441721	2.43683	2.41483	2.419678	2.427647	2.486918	2.424906	2.434169	2.413533	2.407841	2.419781	2.424091	2.341894
CLEC3B	205200_at	7.86574	7.732906	8.331251	8.579294	8.718079	9.121249	5.406854	6.349072	4.968121	4.825223	5.924984	6.330182	7.998297
GLI3	205201_at	3.824181	3.81975	3.798172	3.928572	3.629289	3.798518	3.809737	4.59941	3.807292	3.795253	3.817087	3.846928	5.846908
PCMT1	205202_at	10.17689	10.13778	10.2312	10.34733	10.09802	9.967522	10.67403	10.78855	10.82874	10.74169	10.28292	10.59262	10.59889
PLD1	205203_at	6.188824	6.40397	6.206475	6.46637	6.186292	6.226448	5.265091	6.183386	5.719687	5.68424	5.992172	6.177518	5.231945
NMB	205204_at	5.087863	5.144517	4.745801	4.842381	5.018272	5.125892	5.021393	5.836302	5.015955	5.001298	5.83967	6.198201	5.183425
RELB	205205_at	2.595534	2.898411	2.166588	1.951467	1.98476	2.27858	2.024455	2.089816	1.959451	2.168363	1.967452	1.955879	1.928938
KAL1	205206_at	2.514762	2.432294	2.3981	2.407774	2.202786	2.201703	5.430906	5.235205	5.638392	5.276284	5.392831	4.060442	9.471151
IL6	205207_at	2.494863	2.560991	2.994509	2.552264	2.57113	2.558854	2.557403	2.569299	2.39596	2.538774	2.551758	2.563048	2.49496
ALDH1L1	205208_at	11.30089	11.2195	12.48081	12.50896	12.56193	12.383	9.24954	9.471522	9.161849	8.960743	9.495243	9.373004	3.240737
ACVR1B	205209_at	2.694375	2.676468	2.646628	2.107601	4.039063	4.225107	2.665003	2.558375	1.859263	1.804213	2.661217	2.669558	2.921044
TGFBRAP1	205210_at	2.113626	2.103243	2.083016	2.090326	2.106827	2.303413	2.100621	2.097563	2.134535	2.08241	2.087626	2.120798	2.053131
RIN1	205211_s_at	3.320059	3.57395	3.35823	3.424932	3.459347	3.302854	3.299302	3.782999	3.413378	3.300363	3.411741	3.421735	2.886518
CENTB1	205212_s_at	3.306509	3.254799	3.300779	3.699951	3.49154	3.630186	3.31428	3.252856	2.84993	3.465808	2.953611	3.304457	2.597176
CENTB1	205213_at	2.691127	2.681596	2.619907	2.666614	2.679474	2.671254	2.679118	2.681151	2.673269	2.669358	2.658117	2.673388	2.468769
STK17B	205214_at	2.587893	2.558848	3.546245	2.553065	2.614478	2.542779	3.6514	2.588942	3.098162	3.441467	2.42598	2.626627	2.325656
RNF2	205215_at	5.223977	5.282143	4.535508	5.185709	5.287657	5.290392	5.668407	5.283684	5.453258	5.901829	5.353015	5.277721	5.562501
APOH	205216_s_at	15.38996	15.30438	15.22563	15.40812	15.40221	15.27574	15.01447	14.99381	15.06907	15.00253	14.6987	14.757	10.17162
TIMM8A	205217_at	5.221935	4.886849	5.209963	5.192751	5.153129	5.122438	5.946643	5.890656	5.573596	5.731378	5.645793	5.926379	4.8994
POLR3F	205218_at	4.605513	4.586222	4.869876	4.752395	4.007162	2.964913	5.646116	4.554773	5.422976	5.653164	4.568635	4.569912	6.174519
GALK2	205219_s_at	3.259196	3.731319	5.325474	4.757092	3.821357	3.496981	4.656352	3.240292	4.388586	4.778288	3.491434	2.96719	4.333511
GPR109B	205220_at	2.19754	2.169018	3.537403	3.605479	4.57953	3.080459	2.061037	2.10104	2.126034	2.048956	2.045746	2.053323	2.189539
HGD	205221_at	10.40993	10.68924	11.93896	12.03033	11.26472	10.89479	9.00207	8.728253	9.37685	9.178805	8.34042	8.600312	2.801907
EHHADH	205222_at	11.91001	12.01858	12.25995	12.37407	11.98848	11.69578	9.84401	10.01817	9.970558	9.759714	8.9379	9.409655	5.451662
DEPDC5	205223_at	4.938207	4.72432	5.273329	5.339468	5.288298	5.430038	4.07485	4.159849	4.071354	3.971133	4.021753	4.199187	3.803747
SURF2	205224_at	4.650148	4.684098	4.491292	4.611285	4.528063	4.974024	4.133482	4.924318	4.364258	4.619524	4.818195	5.227439	3.536019
ESR1	205225_at	8.418266	8.384584	9.000713	9.038559	7.887011	7.692739	1.812055	1.81939	1.832127	1.797071	1.928157	1.941259	2.040779
PDGFRL	205226_at	2.366922	2.335597	2.296073	2.317924	2.350368	2.30791	2.329458	2.339102	2.325718	2.317875	2.755447	2.358687	7.243438
IL1RAP	205227_at	8.450172	8.529756	9.194837	8.785694	8.022552	7.561068	8.799374	7.540595	8.295235	8.644095	7.10754	6.978429	6.679359
RBMS2	205228_at	1.802108	1.791766	1.776897	1.784194	1.790211	1.778306	1.79299	1.790039	1.790119	2.143905	2.593704	1.881121	3.026277
COCH	205229_s_at	2.313583	2.289442	2.257482	2.272189	2.301891	2.294841	4.98895	3.853839	3.599746	6.299007	4.515558	2.61019	6.051704
RPH3A	205230_at	2.670727	2.653147	2.62935	2.635936	2.64046	2.610303	2.639691	2.647826	2.637037	2.626321	2.608021	2.685301	2.699064
EPM2A	205231_s_at	6.702193	6.77991	6.606057	6.853162	6.798653	6.762863	5.486456	6.141441	5.469537	5.563782	5.737192	5.487194	5.47111

PAFAH2	205232_s_at	3.301464	4.498657	4.532615	4.00196	2.963578	2.880455	3.670642	3.304551	3.657344	4.613443	3.050364	3.038516	3.623126
PAFAH2	205233_s_at	5.567865	5.402371	5.237344	5.147115	4.951197	5.100423	4.433531	4.81253	4.746021	4.661511	4.667469	4.479319	4.274911
SLC16A4	205234_at	6.42411	6.325813	7.313215	7.603417	8.031129	7.142931	6.465395	6.257621	6.411937	6.275446	5.726522	5.773356	6.274485
MPHOSPH1	205235_s_at	3.247952	3.438403	3.403429	3.518073	3.575836	3.383079	6.312409	6.555996	5.848635	6.455407	6.351372	6.087798	5.00381
SOD3	205236_x_at	3.743253	3.706357	3.701858	3.71683	3.710602	5.372865	3.698644	4.212592	3.675119	3.561095	3.791961	4.113859	3.663081
FCN1	205237_at	3.050535	3.191795	3.413241	3.196277	3.2258	3.514327	2.864866	3.11934	2.819302	2.87771	2.917959	2.868535	2.966831
CXorf34	205238_at	3.97344	3.688206	3.503237	3.464025	4.335706	3.694797	4.392615	3.468523	3.657733	3.948029	3.529589	4.606281	3.237967
AREG	205239_at	2.923162	2.927017	3.854301	3.141952	2.932815	2.871147	2.895661	2.934009	2.920842	2.899613	2.919235	2.939654	2.764362
GPSM2	205240_at	2.365311	2.770266	2.309842	2.297536	2.348305	2.339574	5.763349	4.017634	5.686632	6.112286	4.08073	4.251039	6.050582
SCO2	205241_at	7.615271	7.501874	7.098323	7.377847	7.436426	7.559081	6.346922	7.022724	6.775986	6.201967	6.826735	7.06617	5.670333
CXCL13	205242_at	2.855452	2.67187	4.258081	3.643209	6.001534	5.208368	2.676317	2.660175	2.656148	2.650256	4.235302	2.687568	4.319981
SLC13A3	205243_at	3.159445	2.576097	3.229478	2.562136	2.570726	2.625388	2.553786	2.52763	2.515605	2.559014	2.55111	2.554368	2.521435
SLC13A3	205244_s_at	6.649117	6.667577	5.473904	6.132007	5.166809	5.120588	3.271338	2.966749	3.221705	3.254564	3.383703	3.382668	3.16667
PARD6A	205245_at	2.317733	2.303248	2.585139	2.648619	2.311677	2.366435	2.277733	2.29971	2.259518	2.287439	2.281845	2.292055	2.205563
PEX13	205246_at	5.180905	5.182171	6.219701	5.876256	5.108282	4.673804	5.386018	4.999194	5.346486	5.578345	4.13349	4.941989	4.702474
NOTCH4	205247_at	5.786262	5.715733	6.265518	6.198164	5.713093	5.972037	4.05652	5.411663	5.085515	4.745847	5.588917	4.822098	4.687829
DOPEY2	205248_at	3.209594	3.670116	5.227042	5.094988	3.533062	3.339606	4.349728	3.617598	5.376564	5.376065	3.109268	5.072001	3.19003
EGR2	205249_at	7.005653	6.911696	6.373282	6.610605	8.997868	8.737212	4.299781	3.874057	4.295474	4.240987	4.051528	4.28748	4.28173
CEP290	205250_s_at	6.158327	6.133756	5.786057	5.891796	6.277101	5.967596	6.671174	7.34837	6.779778	6.380755	7.644233	7.038732	7.571011
PER2	205251_at	6.992436	7.183696	8.374378	8.010445	6.885855	6.564873	4.709886	5.68158	5.219772	5.419205	5.447773	5.369407	4.843455
ZNF174	205252_at	3.154359	3.061966	2.977014	3.051802	3.278918	3.054984	3.047578	3.064518	3.065514	3.043276	3.0495	3.056002	3.627137
PBX1	205253_at	2.394239	2.415024	2.524371	2.326484	2.37974	2.337019	3.486164	2.555928	3.510106	2.815642	3.382984	3.131123	5.04765
TCF7	205254_x_at	2.206861	2.186965	2.164213	2.168236	2.190312	2.167157	2.178947	2.184962	2.169766	2.162537	2.17075	2.179363	2.114498
TCF7	205255_x_at	7.783826	7.805647	7.020909	7.195515	7.753002	8.158902	7.16375	7.809867	7.177252	7.03676	7.811051	7.596097	6.775904
ZBTB39	205256_at	4.158486	3.873999	3.825366	3.86408	3.934359	3.783438	4.085578	4.459806	3.968755	4.091084	3.900611	4.191716	4.203031
AMPH	205257_s_at	2.172044	2.15972	2.144694	2.186616	2.17455	2.148324	2.152832	2.154838	2.137258	2.13304	3.260532	2.143523	7.325885
INHBB	205258_at	6.790858	7.001527	8.029963	8.249473	7.895637	7.67594	1.994399	1.994657	1.985803	1.977622	1.978865	1.986572	3.736288
NR3C2	205259_at	7.730751	7.557869	8.080766	7.834027	7.28029	6.697615	3.229883	2.939225	3.216542	3.306592	3.536811	2.741767	6.158928
ACYP1	205260_s_at	4.004931	4.297903	3.845786	3.969329	3.619842	3.937237	6.269143	5.794533	6.394716	6.624646	5.671631	6.065092	6.565759
PGC	205261_at	2.066484	2.057461	2.036412	2.045146	2.054975	2.044821	2.056404	2.056782	2.049251	2.043213	2.036816	2.049961	1.974503
KCNH2	205262_at	2.109514	2.088052	2.066161	2.070491	2.092437	2.072376	7.159449	7.62051	7.243161	7.241838	7.072411	6.937938	4.326981
BCL10	205263_at	8.164645	7.782157	7.476467	7.330826	7.453372	7.163607	8.161971	7.649396	7.838534	7.656314	7.718401	7.723611	8.671462
CD3EAP	205264_at	4.412855	4.298724	4.162011	4.59133	4.366152	4.586387	5.361003	5.399046	5.570807	5.635715	5.352539	5.291396	4.495243
SPEG	205265_s_at	2.561735	2.535205	2.510835	2.520571	2.544124	2.517494	2.529086	2.537671	2.523783	2.512624	2.856988	2.533429	3.113032
LIF	205266_at	3.410106	3.410827	3.374919	3.394106	3.394317	3.531253	3.374102	3.396293	3.384948	2.954442	3.371428	3.383045	2.930119
POU2AF1	205267_at	3.427121	3.438177	2.546099	2.533159	4.295246	5.220142	2.856883	5.082853	3.349223	3.426854	3.809759	4.928059	2.363119
ADD2	205268_s_at	2.171425	2.15898	2.138877	2.142265	2.196019	2.144222	9.920207	9.970637	10.01988	10.55159	9.37919	9.64056	3.802545
LCP2	205269_at	6.39712	6.386315	6.685387	6.327044	6.726219	6.098472	6.310659	6.243433	6.373851	6.388089	5.909769	6.268226	4.73451

LCP2	205270_s_at	4.160618	4.048355	4.678918	4.48283	4.449622	4.481349	5.085545	4.562266	4.851425	4.427966	3.544079	4.421643	3.37295
CCRK	205271_s_at	2.852206	2.849171	2.828893	2.831241	2.840869	2.833453	2.843316	2.830159	2.833648	2.831352	2.826959	2.834782	3.246174
PRH1	205272_s_at	2.676147	2.637105	2.600149	2.624963	2.71417	2.617629	2.634633	2.646595	2.632303	2.625243	2.629783	2.649213	2.475466
PITRM1	205273_s_at	7.547482	7.436731	7.530656	7.583217	7.464825	6.986769	7.076563	7.179108	7.119247	7.506506	7.177955	7.189117	7.276767
GTPBP1	205274_at	2.031485	2.020759	1.999642	2.004376	2.015832	2.006322	2.01644	2.020256	2.011455	2.003005	2.00551	2.014206	1.939438
GTPBP1	205275_at	2.411707	2.405752	2.386735	2.386896	2.398372	2.394341	2.40234	2.401746	2.388998	2.381168	2.388257	2.396785	2.103765
GTPBP1	205276_s_at	4.102613	4.26578	4.015089	4.055124	4.361117	4.083234	4.100284	4.111882	4.096428	4.036718	4.097162	4.129673	3.676284
PRDM2	205277_at	3.361416	3.705966	3.378606	3.887794	4.143738	4.228387	2.738095	3.126278	2.389034	2.847215	3.445581	2.67327	4.638022
GAD1	205278_at	2.397012	2.364057	2.322969	2.347716	2.380718	2.344489	2.353304	2.364561	6.000171	2.356461	2.350147	2.361749	5.73556
GLRB	205279_s_at	2.698915	2.64591	2.566371	2.648318	2.657903	2.606847	2.652111	2.622345	2.726997	2.776908	2.617211	2.717535	5.830515
GLRB	205280_at	3.154066	3.565546	2.753613	2.876559	2.975427	2.787467	3.651106	3.088737	3.478535	3.059598	3.085437	3.170484	6.095684
PIGA	205281_s_at	6.868602	6.720035	6.975018	7.264419	6.71863	6.60468	7.020795	6.762042	7.541305	7.146807	6.843367	7.027246	6.173848
LRP8	205282_at	2.571136	2.524207	2.492676	2.518666	2.569143	2.52004	2.527078	2.533156	2.523386	2.517775	2.521015	2.53351	4.021928
FCMD	205283_at	5.421484	5.394282	5.14889	5.465442	5.212223	5.116094	5.145484	5.416976	5.75813	5.203044	5.766243	5.318594	5.972115
KIAA0133	205284_at	3.411366	4.288621	4.429246	3.746269	4.28604	4.276902	5.276803	5.710182	5.270859	5.250462	5.076694	5.320748	4.64758
FYB	205285_s_at	3.215511	3.187684	3.17085	3.109173	3.441329	3.96166	3.197063	3.070207	3.173924	3.069777	3.08736	3.195257	2.780373
TFAP2C	205286_at	2.2304	2.20528	2.174001	2.191232	2.212937	2.182001	2.194672	2.203216	2.194893	2.181875	2.188121	2.239112	3.948579
TFAP2C	205287_s_at	2.01474	1.98804	1.962551	1.971593	1.996892	1.975859	1.983372	1.987175	1.97511	1.962706	1.970562	1.983497	1.929677
CDC14A	205288_at	3.642399	3.460899	3.329491	3.483485	3.666777	3.467014	3.493181	3.517387	3.509284	3.536922	3.602797	3.733549	3.69068
BMP2	205289_at	5.934104	5.380805	6.895417	6.977281	5.537344	5.765712	6.237997	5.98845	6.556994	6.710208	5.864314	5.893818	7.037451
BMP2	205290_s_at	4.055889	4.007496	5.444894	5.821368	4.493939	5.548596	4.304493	4.611082	4.481022	4.571746	4.728887	5.062521	4.630408
IL2RB	205291_at	5.253028	4.913356	4.42683	4.077816	3.332377	4.234588	2.558783	3.009236	2.56179	2.437285	2.116836	2.444775	4.107205
HNRNPA2B	205292_s_at	11.00731	10.98069	11.05285	10.72855	10.92562	10.35072	12.53344	11.84053	12.50425	12.47458	11.66689	11.83065	12.63494
BAIAP2	205293_x_at	3.853451	4.052578	2.23497	2.705756	2.541803	3.064626	2.173331	2.133293	2.169251	2.120749	2.195059	2.095795	2.202617
BAIAP2	205294_at	5.132431	5.054243	5.08201	5.294724	5.283606	5.087143	4.684259	5.075999	5.045951	4.180201	5.029305	5.182123	4.224813
CKMT2	205295_at	2.475407	2.450372	2.407224	2.437135	2.455347	2.443629	2.492601	2.457652	2.459522	2.448627	2.595765	2.450152	5.100251
RBL1	205296_at	4.485074	4.719295	4.141533	3.922827	4.595092	4.253489	7.836664	7.776325	7.372607	7.673179	7.578701	8.004174	5.089629
CD79B	205297_s_at	3.927247	3.981864	3.456727	3.688186	4.213292	3.930233	3.319983	4.438675	3.31179	3.424028	3.45453	4.044378	2.97001
BTN2A2	205298_s_at	4.859848	5.09986	4.6263	4.132271	4.666941	5.122041	4.337886	4.331397	4.499822	4.311421	4.508536	4.598031	5.250029
BTN2A2	205299_s_at	2.782228	2.808539	2.670652	2.720768	2.770525	2.699589	2.732353	2.730359	2.725847	2.709689	2.725415	2.793748	2.743793
U1SNRNPB	205300_s_at	5.94688	6.192729	5.325084	5.595049	6.242509	6.839455	6.013249	6.325369	6.008204	6.584939	6.796417	7.029165	5.911691
OGG1	205301_s_at	6.064579	6.20403	5.918164	5.890286	6.218474	6.218481	6.181115	7.144889	5.89464	6.155869	6.556021	6.792759	5.813888
IGFBP1	205302_at	13.854	13.99818	13.52053	13.77891	13.60735	13.45009	8.635309	9.889062	11.14892	11.11105	9.151243	9.778606	12.89266
KCNJ8	205303_at	9.220383	9.290261	9.454461	9.843032	9.013526	8.839623	5.631755	5.93945	5.769069	5.801467	6.029807	6.212544	6.209857
KCNJ8	205304_s_at	8.0575	8.104003	8.813907	8.978893	7.918489	7.493738	4.784928	4.830299	5.165412	4.753654	4.759788	4.585183	5.224626
FGL1	205305_at	13.68517	13.66217	13.46058	13.55804	13.73679	13.51148	11.0938	11.42638	12.74558	12.57238	11.4723	10.02501	5.111564
KMO	205306_x_at	8.991987	8.997191	9.010199	9.062815	8.20758	7.857714	5.906744	4.948397	5.069767	5.050884	4.743501	4.885821	1.785047
KMO	205307_s_at	8.356045	8.204971	8.03163	8.368271	7.398486	7.017379	5.759911	3.79036	6.054725	4.521993	4.459363	5.59816	2.903548

C8orf70	205308_at	4.294607	4.525799	4.434388	4.552704	4.66219	5.585786	4.529594	5.286101	4.527891	4.483275	6.851477	5.928848	8.45892
SMPDL3B	205309_at	2.074603	2.05745	2.036847	2.040459	2.057205	2.038332	2.051728	2.053914	2.04071	2.033381	2.038784	2.050258	1.973556
FBXO46	205310_at	6.092284	6.022893	5.544341	6.045857	6.502124	6.542198	5.703121	6.062301	5.521442	5.379245	6.295672	6.357611	5.666171
DDC	205311_at	8.757974	8.680547	9.117821	8.979238	8.818446	8.632526	10.06071	9.440151	9.892834	9.719679	8.880704	9.038503	4.402582
SPI1	205312_at	3.390699	3.317655	3.365152	3.368815	3.541932	3.36545	3.376025	3.372379	3.356881	3.362733	3.327949	3.379673	2.696595
HNF1B	205313_at	6.812553	6.955537	6.852048	7.415197	7.320621	7.543673	6.522538	6.816647	6.154919	5.829091	5.818245	6.554294	4.035266
SNTB2	205314_x_at	2.20762	2.184082	2.160483	2.165951	2.196623	2.161662	2.176957	2.183719	2.168389	2.161727	2.163643	2.178584	2.054192
SNTB2	205315_s_at	4.768959	4.938369	4.995152	4.783891	4.71546	4.853225	3.934839	3.800042	3.708205	4.030005	4.883596	4.265182	5.527973
SLC15A2	205317_s_at	2.849295	2.737054	2.692505	2.720915	2.758677	2.709633	2.72958	2.738858	2.72389	2.70148	2.722959	2.727633	2.694508
KIF5A	205318_at	1.666889	1.659413	1.640588	1.643745	1.656101	1.646816	1.656754	1.656695	1.651188	1.644812	1.641205	1.648895	1.626341
PSCA	205319_at	2.871304	2.840012	2.757927	2.788053	2.864263	2.822536	2.826767	2.831113	2.823002	2.807486	2.817902	2.834331	2.683744
APC2	205320_at	5.449046	5.22642	5.069216	5.1778	5.3212	5.345139	5.208639	5.217904	5.145755	5.188862	4.991112	5.134933	4.849807
EIF2S3	205321_at	4.338221	4.247905	5.665826	4.294039	2.947272	3.210194	6.968415	3.862157	7.334399	6.829999	4.255391	4.257662	8.117906
MTF1	205322_s_at	5.718015	5.717529	5.704015	5.942896	5.702309	5.840619	5.706708	6.083274	5.700973	5.693176	5.89907	5.822468	4.577797
MTF1	205323_s_at	4.504879	4.564218	4.554658	4.683248	4.355416	4.562166	4.656412	5.179044	4.384206	4.741468	4.66915	4.55549	3.871642
FTSJ1	205324_s_at	5.92918	6.193797	6.348654	6.681189	6.770055	6.514747	8.105421	7.670355	8.440741	8.244945	7.60389	7.784044	8.17642
PHYHIP	205325_at	1.922271	1.912616	1.893299	1.898683	1.911281	2.366811	1.905527	1.909548	1.900303	1.891671	1.891052	1.903338	1.851896
RAMP3	205326_at	4.895511	4.355106	5.350467	5.114636	6.215323	6.58668	2.250159	2.266816	2.241944	2.236279	2.251997	2.231639	2.484443
ACVR2A	205327_s_at	6.780197	7.054579	6.722925	6.663734	6.229784	6.724609	6.622865	5.306395	6.52102	6.213662	6.458679	6.01378	7.691767
CLDN10	205328_at	2.429017	2.520139	2.248327	2.254367	2.363392	2.251215	2.253297	2.255154	2.240176	2.226039	2.237948	2.257276	2.559422
SNX4	205329_s_at	6.91773	6.645529	7.964542	7.794037	6.896875	6.715583	7.371087	6.657519	7.451521	7.717368	6.242652	6.852255	7.570892
MN1	205330_at	2.381404	2.356872	2.327884	2.342129	2.36653	2.344173	2.345162	2.355395	2.340669	2.338299	5.777309	2.355294	9.236741
REEP2	205331_s_at	2.824838	2.816667	2.789954	2.794609	2.806546	2.77277	2.805811	2.802372	2.787922	2.789062	2.794571	2.795076	2.896933
RCE1	205332_at	2.410801	2.404635	2.374544	2.390135	2.404622	2.39389	2.557941	2.401969	2.382477	2.381027	2.384582	2.394023	2.330316
RCE1	205333_s_at	3.940915	3.844208	3.762042	3.704098	3.692055	3.782118	4.371776	4.96318	3.983708	4.286048	4.923732	4.379952	3.777954
S100A1	205334_at	2.726614	2.701792	2.640564	2.68859	2.698144	2.686387	2.685539	2.695255	2.689111	2.684985	2.674965	2.69114	2.702315
SRP19	205335_s_at	8.868459	8.860361	9.34033	9.269967	9.074942	8.745189	10.04867	9.986232	10.38711	10.32602	9.879543	10.17913	9.422184
PVALB	205336_at	2.906865	2.561229	3.950869	4.536393	2.849684	2.861996	2.863219	2.866718	2.877514	2.833543	2.86369	2.685919	4.24349
DCT	205337_at	2.633283	2.552654	2.689612	2.613489	2.620853	2.612601	2.542959	2.554698	2.545352	2.52666	2.54782	2.575067	4.485493
DCT	205338_s_at	1.848447	1.836236	1.816781	1.82091	1.836697	1.826357	1.833625	1.833782	1.822588	1.817902	1.81772	1.826473	2.147285
STIL	205339_at	4.900437	4.636959	4.445793	4.478345	4.492041	4.590301	8.914545	9.076803	9.124329	8.870892	8.765896	9.262799	7.279141
ZBTB24	205340_at	5.850922	6.080039	5.468143	5.843473	6.071289	5.935609	5.356618	5.474183	5.08212	4.548566	5.504875	5.473347	5.660985
EHD2	205341_at	2.712861	2.693931	2.648081	2.618238	2.702304	2.661893	2.765847	2.699777	2.685331	2.83162	2.685847	2.745376	3.602277
SULT1C2	205342_s_at	2.333011	2.346007	2.249131	2.254385	2.283431	2.229824	6.622701	5.531222	6.636957	6.371084	5.145174	5.531333	2.190975
SULT1C2	205343_at	2.561744	2.238575	2.180783	2.209509	2.22228	2.208557	6.223539	6.614923	5.868347	5.907998	2.614054	4.913091	2.117138
CSPG5	205344_at	2.267925	2.249241	2.22112	2.23242	2.250331	2.231708	2.240602	2.249406	2.235084	2.227428	2.227488	2.245735	2.222411
BARD1	205345_at	4.156377	4.181131	2.968444	3.642526	3.633422	4.156356	6.638796	7.284509	7.13502	7.098528	7.458164	7.383536	7.633839
ST3GAL2	205346_at	2.344051	2.339489	2.281974	2.322446	2.328815	2.303034	2.438712	2.342636	2.33005	2.324294	2.753634	2.321675	2.81461

TMSL8	205347_s_at	2.639917	2.564671	2.513471	2.552765	2.609903	2.553796	5.126919	4.664256	4.559109	3.690664	7.247402	4.116305	11.64117
DYNC1I1	205348_s_at	2.726795	2.707531	2.674695	2.695685	2.719273	2.703826	2.714066	2.716021	2.766809	2.736297	2.701278	2.712288	4.958604
GNA15	205349_at	4.558671	4.53703	4.381009	4.517658	4.744984	5.59022	4.912148	6.141979	5.165533	5.669226	5.154778	5.842488	3.218678
CRABP1	205350_at	2.22526	2.206758	2.178378	2.184752	2.208497	2.187523	2.277856	2.205595	2.191241	2.183466	4.951303	2.383746	10.3534
GGCX	205351_at	8.607425	8.543097	9.525528	9.358231	8.276541	7.993399	8.74732	7.927642	8.857269	8.76014	7.961382	8.358247	5.025916
SERPINI1	205352_at	5.221159	4.941845	5.725693	5.211896	5.104565	4.60229	10.03929	9.877572	10.12443	10.38927	9.61757	9.517082	8.586965
PEBP1	205353_s_at	13.29332	13.35864	13.1406	13.2192	13.40922	13.3016	11.91052	12.26674	12.11623	11.97511	12.04844	12.19211	10.02058
GAMT	205354_at	10.88127	10.84246	11.00028	10.9868	11.84262	11.68781	9.659867	10.17733	9.940357	10.03606	10.20593	10.37653	7.051531
ACADSB	205355_at	9.910656	9.763409	10.58357	10.64966	10.1079	9.578015	9.762663	9.811249	10.60493	10.27417	9.040962	8.44128	4.594354
USP13	205356_at	6.80647	6.47127	6.969627	7.349574	6.94893	6.587415	6.255752	6.019781	6.394964	5.979623	6.612065	6.638133	7.562557
AGTR1	205357_s_at	10.01867	9.902726	10.83262	10.5514	9.213169	8.545106	10.35639	9.345788	10.52359	10.36203	9.104805	9.392429	6.482628
GRIA2	205358_at	2.199792	2.178892	2.150336	2.161847	2.274168	2.155984	2.16793	2.1765	2.164166	2.156611	2.30413	2.173234	8.332193
AKAP6	205359_at	2.061169	2.046636	2.021302	2.037842	2.045489	2.028594	2.034233	2.042583	2.031382	2.020867	2.027914	2.037243	3.540288
PFDN4	205361_s_at	9.157659	9.224274	9.293852	9.349218	9.101148	8.937972	10.75797	10.49099	10.83922	10.8837	10.60707	10.69263	11.42978
PFDN4	205362_s_at	5.126538	5.08222	4.797715	5.210417	5.412338	5.119877	6.028184	6.536946	5.96919	5.581746	6.276353	5.823137	6.881251
BBOX1	205363_at	8.366857	8.071399	10.05908	10.09111	9.279368	8.823798	3.296723	3.064801	3.54198	3.413151	3.293473	2.973858	3.306971
ACOX2	205364_at	10.22569	10.21186	10.79762	10.69379	10.52358	10.08186	8.995481	8.186364	8.79525	8.745743	7.622205	7.688762	5.043408
HOXB6	205365_at	2.51891	2.485856	2.452493	2.467334	2.495494	2.459896	2.476877	2.48654	2.475831	2.467645	2.47308	2.480534	2.516779
HOXB6	205366_s_at	3.345958	3.300854	3.844688	3.828993	3.309198	3.392355	3.363172	3.200889	3.325608	3.282273	3.302862	3.054276	6.570079
SH2B2	205367_at	7.130523	7.627379	6.841603	6.57502	7.481072	8.020594	6.440145	7.355078	6.167343	6.643048	7.369239	7.216239	6.262831
FAM131B	205368_at	2.817558	2.79338	2.750489	2.773448	2.819022	2.769002	2.776721	2.788739	2.76269	2.76943	2.77441	2.795481	4.696442
DBT	205369_x_at	4.327203	4.002405	5.122747	4.780572	4.312217	4.290606	4.303408	4.038294	4.45609	4.307383	4.021017	3.98879	4.62971
DBT	205370_x_at	11.91404	11.88884	11.15162	11.42421	11.8539	12.28551	10.95405	11.52759	10.84376	10.79336	11.61247	11.43636	10.31635
DBT	205371_s_at	5.509747	5.212123	7.079091	6.619036	5.317613	4.467587	5.678204	4.407781	5.54092	5.077757	4.334007	4.289355	4.809674
PLAG1	205372_at	5.179711	4.70892	3.576216	3.682962	3.275948	3.792563	7.269469	7.446321	7.090828	7.006086	6.721519	6.559293	7.633123
CTNNA2	205373_at	2.534634	2.51625	2.443986	2.476833	2.519235	2.458041	2.499819	2.510921	2.495323	2.484479	2.49997	2.52633	6.212635
SLN	205374_at	2.134063	2.122676	2.05872	2.09599	2.121111	2.094427	2.115852	2.119888	2.106839	2.085249	9.317359	2.114594	10.36068
MDFI	205375_at	2.797714	2.76513	2.674562	2.752767	2.780831	2.743411	2.945177	2.774641	2.759563	2.744759	3.179816	2.771046	3.019892
INPP4B	205376_at	2.826341	2.772096	2.75152	2.789392	2.842428	2.773457	2.779085	2.798709	2.777056	2.79653	3.140883	2.972438	3.5447
ACHE	205377_s_at	2.17653	2.157864	2.128972	2.139144	2.161275	2.138112	4.303728	2.309404	3.772241	4.578996	2.153972	2.893922	2.727595
ACHE	205378_s_at	2.56475	2.553597	2.451996	2.515154	2.552752	2.48725	4.807848	4.321525	4.938704	3.712309	5.370302	4.845663	3.045615
CBR3	205379_at	2.739888	2.847464	2.700993	2.70547	2.763197	2.718107	2.715793	2.724	2.707736	2.707293	2.882439	2.722384	2.655591
PDZK1	205380_at	9.088081	8.904927	9.143785	8.939328	8.82166	8.488773	9.514876	9.106612	9.882448	9.799157	8.637949	8.927873	2.970657
LRRC17	205381_at	6.380277	6.315081	5.881584	5.18711	5.425366	4.963009	9.354574	8.17848	8.527322	8.411423	9.956802	9.054329	11.48771
CFD	205382_s_at	6.023895	6.117475	6.451221	7.292777	6.369351	6.701626	5.011463	6.03975	5.565877	5.659877	5.188337	5.639301	4.15483
ZBTB20	205383_s_at	10.63947	10.65752	10.50738	10.57431	10.515	10.32715	7.821058	7.89208	7.869749	8.082263	7.54128	8.100115	7.719391
FXYD1	205384_at	10.21667	10.35426	10.43937	10.66686	10.24699	10.15133	7.844331	8.250709	8.422715	8.843099	7.760451	6.933816	6.358952
MDM2	205385_at	2.395983	2.369383	2.336492	2.346733	2.381588	2.334308	2.356408	2.363007	2.352455	2.347081	2.349461	2.36392	2.252222

MDM2	205386_s_at	3.126583	3.105707	3.001899	3.069999	3.164712	3.092754	3.073461	3.102569	3.086738	3.006981	3.085824	3.088541	2.857881
CGB	205387_s_at	2.469186	2.598534	2.402447	2.432361	2.457321	2.430935	2.441191	2.451468	4.642974	2.42794	2.574876	2.448314	3.475736
TNNC2	205388_at	2.122316	2.10635	2.081903	2.088402	2.106223	2.088279	2.102543	2.105941	2.091236	2.094411	11.58165	2.136394	12.29049
ANK1	205389_s_at	2.539353	2.570792	2.478928	2.501405	2.649233	3.014641	9.334593	9.714964	9.318113	9.491466	8.917157	9.640162	3.84859
ANK1	205390_s_at	2.895827	2.86181	2.825049	2.844533	2.85997	2.842299	10.03834	10.19339	10.20939	10.12816	9.238274	9.991784	2.837028
ANK1	205391_x_at	2.352268	2.328222	2.299753	2.308226	2.336117	2.307263	9.972098	10.10158	9.481741	10.18066	9.822101	9.988628	4.726394
CCL14	205392_s_at	8.96538	8.793578	10.31039	10.10301	10.40445	10.23461	6.579944	6.120543	6.690761	6.894535	6.384863	6.469968	4.520351
CHEK1	205393_s_at	3.493743	3.265431	3.151299	3.278018	3.377778	3.187907	7.078777	6.613405	7.320454	7.342776	5.734965	6.557128	6.342626
CHEK1	205394_at	2.916735	2.866031	2.82137	2.853272	2.903263	2.842567	7.207706	6.52127	7.42927	7.341547	6.522084	6.686193	5.983872
MRE11A	205395_s_at	3.912666	3.846601	3.720258	3.870544	3.895676	3.844546	5.291479	5.171847	5.424178	5.716386	3.994952	5.323203	5.305677
SMAD3	205396_at	1.897017	1.885913	1.866205	1.870439	1.886267	1.86469	1.875533	1.889525	1.872142	1.870519	1.86461	1.883287	3.088392
SMAD3	205397_x_at	2.246719	2.224122	2.219527	2.209672	2.213685	2.211629	2.201818	2.225277	2.211132	2.189248	2.203737	2.216306	2.290923
SMAD3	205398_s_at	3.481484	3.591434	3.938849	3.829398	3.492281	3.530886	2.416429	2.791827	3.349939	3.504239	3.343227	3.038325	3.563314
DCLK1	205399_at	2.535743	2.503066	2.446549	2.479894	2.501676	2.48962	2.488642	2.497792	2.483023	2.474969	2.514207	2.49367	9.538245
WAS	205400_at	2.776809	2.71523	2.661579	2.706583	2.749903	2.684754	2.718353	2.728466	2.711788	2.690575	2.711953	2.865806	2.502743
AGPS	205401_at	4.623544	4.661759	5.542226	5.729816	5.752703	5.74491	7.070368	6.462805	7.012635	7.445391	6.721654	6.966391	6.587337
PRSS2	205402_x_at	2.510531	2.495613	2.461459	2.459466	2.504661	2.800209	2.474425	2.496199	2.479192	2.470427	2.481852	2.522564	2.175945
IL1R2	205403_at	7.770422	7.849418	7.448905	7.818255	6.94228	7.182619	5.462146	6.669072	6.534293	7.469613	7.503554	7.355037	5.733462
HSD11B1	205404_at	11.77762	11.78302	13.14558	13.05236	11.77171	11.31872	2.457214	2.460305	2.445444	2.556877	2.509626	2.456675	2.72627
SEMA5A	205405_at	6.391788	6.732962	7.185624	6.45607	6.321328	6.317979	6.18922	5.745687	4.995395	5.676993	6.310819	6.304187	8.286165
SPA17	205406_s_at	3.055314	3.050751	3.028551	3.024728	3.050843	3.031729	5.863214	6.63607	6.169095	6.146752	4.468679	5.602482	5.233797
RECK	205407_at	8.039162	7.97653	7.424219	7.529481	7.409612	7.527047	7.396627	7.091025	7.127628	7.076052	8.429282	7.279739	9.83647
MLLT10	205408_at	3.238033	3.11048	4.088293	3.103038	3.010201	3.091288	4.193025	3.00696	4.224745	4.992794	2.541988	3.072611	5.925852
FOSL2	205409_at	2.695014	2.697454	2.698713	2.702439	2.791835	2.696667	2.694735	2.710957	2.703755	2.677379	2.691335	2.710661	2.575279
ATP2B4	205410_s_at	1.961537	1.952608	1.932522	1.939752	1.949961	1.940075	1.949388	1.951489	1.939467	1.932213	1.934596	1.943709	2.762206
STK4	205411_at	2.252468	2.228516	2.192083	2.210072	2.238222	2.211165	2.216563	2.224963	2.21087	2.2045	2.213314	2.221425	2.147665
ACAT1	205412_at	12.58462	12.51488	12.48665	12.46841	12.0317	11.76617	9.927416	9.623601	9.83608	9.726733	9.534347	9.632858	8.718189
MPPED2	205413_at	2.617414	2.55025	2.583116	2.862832	2.637511	2.586343	2.591205	2.565011	2.559433	2.549763	6.810778	2.584546	9.92227
KIAA0672	205414_s_at	2.708936	2.679342	2.688765	2.744241	2.746992	2.66821	2.642843	2.67769	2.668803	2.658166	2.737015	2.695065	4.491408
ATXN3	205415_s_at	6.609948	6.566394	6.090982	6.647754	7.139245	7.09361	6.107904	7.114565	6.253354	6.461942	6.787923	6.718266	6.844264
ATXN3	205416_s_at	2.622422	2.590904	2.549692	2.530039	2.612701	3.005983	2.487193	2.599674	2.578131	2.577371	2.72155	2.588461	3.591567
DAG1	205417_s_at	8.793682	8.835633	7.8994	8.066889	8.290872	8.040932	7.210844	6.961495	7.04396	7.205086	7.788299	7.139985	8.262025
FES	205418_at	5.050751	5.225195	5.159838	5.365637	5.930736	5.691569	4.956008	5.041607	5.034845	4.81297	5.002665	5.518852	4.251253
EBI2	205419_at	4.765767	4.595947	6.031668	5.444592	4.195773	5.06508	4.543136	4.327975	4.081407	4.289012	4.1587	4.86653	3.619186
PEX7	205420_at	6.790979	6.777018	6.824929	6.982878	6.880649	6.242624	6.238326	5.688312	6.062704	6.354301	5.899525	5.978347	5.822871
SLC22A3	205421_at	6.800301	6.453169	7.207512	6.861335	6.2179	5.864125	5.68578	4.019358	5.907595	5.973165	4.655489	4.775134	5.177557
ITGBL1	205422_s_at	2.824294	2.798449	4.274362	2.991892	3.140442	2.788753	2.768663	2.79994	2.750862	2.728223	2.76589	2.797733	4.216787
AP1B1	205423_at	6.251769	6.116701	6.305611	6.44181	6.116809	6.413746	7.257611	7.355398	7.103884	7.330775	7.02644	7.311102	5.599183

TBKBP1	205424_at	3.671124	3.678609	3.646289	3.649501	3.887654	3.742528	3.662193	3.682916	3.648866	3.640362	3.855794	3.54737	3.389273
HIP1	205425_at	3.593679	3.466927	5.582542	5.692699	4.558748	4.648325	4.478572	3.83354	4.67166	3.897833	4.389378	3.593208	7.719315
HIP1	205426_s_at	2.229839	2.213726	2.204183	2.200299	2.220749	2.19576	2.211215	2.213866	2.206515	2.200924	2.201649	2.211315	4.123719
ZNF354A	205427_at	5.053096	5.010864	4.717781	4.508618	4.815971	4.919283	5.426408	5.309004	5.379202	6.232026	5.497432	5.315219	6.836714
CALB2	205428_s_at	2.66789	2.659365	2.643357	2.639968	2.656354	2.650286	2.651264	2.669968	2.643085	2.646112	2.692401	2.65286	3.78519
MPP6	205429_s_at	4.017763	4.064963	4.260332	4.69576	4.06575	4.04366	4.1236	4.086236	4.338936	4.729575	4.082107	4.081159	4.099031
BMP5	205430_at	3.618283	3.471938	3.49333	3.455892	3.930685	3.765007	3.643787	3.289539	3.324269	3.429071	3.447837	3.469367	7.062233
BMP5	205431_s_at	2.046796	2.028356	2.046517	2.015911	2.230156	2.015475	2.026638	2.027515	2.010448	2.000338	2.032889	2.026358	5.608822
OVGP1	205432_at	7.200866	7.081247	6.258513	6.9675	7.273896	7.078003	5.42915	5.437994	5.424263	5.637513	5.619291	6.383056	4.035528
BCHE	205433_at	10.98776	10.9221	12.51076	12.46852	12.05892	11.70064	8.496068	8.735334	9.191149	8.767204	8.021113	8.564502	8.661876
AAK1	205434_s_at	7.33325	7.756544	6.576929	6.940876	7.62987	8.264661	6.196393	6.849694	6.027037	6.251489	7.515937	7.074631	6.709284
AAK1	205435_s_at	3.27424	3.229987	3.171168	3.227948	3.26148	3.251971	3.22223	3.232018	3.234573	3.202022	3.217548	3.266028	3.05956
H2AFX	205436_s_at	7.03685	7.102709	5.286548	5.570593	6.400065	6.414577	10.75996	10.97444	10.68914	10.79816	10.90589	10.91819	9.691707
ZNF211	205437_at	5.636079	5.315434	5.34992	5.259505	5.258174	5.189062	5.77836	5.700643	6.164789	5.903019	5.74562	6.059689	6.582578
PTPN21	205438_at	2.293416	2.274407	2.874961	2.65067	2.28314	2.253938	2.264591	2.277909	2.255188	2.243835	2.297813	2.264753	3.779907
GSTT2	205439_at	5.812286	4.664664	2.549753	2.315807	2.339949	2.515169	2.327378	4.746253	2.319857	3.089984	2.32265	3.065898	2.260146
NPY1R	205440_s_at	7.40046	7.009165	7.56256	7.598957	7.111525	7.280474	2.341778	3.714679	3.546857	3.002382	4.842996	2.685996	6.795214
OCEL1	205441_at	6.218891	5.93093	6.742013	6.933812	7.105256	7.301206	5.095994	5.506474	5.007279	5.414702	5.339878	5.571151	3.079261
MFAP3L	205442_at	9.039509	8.912177	10.24016	10.34326	10.43985	10.27496	8.348203	8.341665	8.560527	8.502803	8.057254	8.779277	5.266268
SNAPC1	205443_at	6.086587	5.747378	6.144783	6.620549	6.397686	7.271514	5.524966	6.119947	5.621393	5.589939	6.323994	6.103452	6.074623
ATP2A1	205444_at	2.072792	2.053459	2.026426	2.038462	2.055129	2.033866	2.043935	2.050313	2.037908	2.030693	2.389344	2.046322	4.663546
PRL	205445_at	2.288603	2.268917	2.251203	2.247559	2.27288	2.253254	2.261059	2.485783	2.251344	2.244824	2.246412	2.260796	5.81468
ATF2	205446_s_at	2.823545	2.738739	2.71106	2.848516	2.78654	2.686943	3.875087	2.764704	3.041171	2.803848	2.775137	2.769823	4.811249
MAP3K12	205447_s_at	2.469261	2.454092	2.415734	2.421536	2.441782	2.762886	2.452884	2.452452	2.426433	2.440675	2.434637	2.773358	2.572086
MAP3K12	205448_s_at	3.025114	3.482917	3.537685	3.390608	3.16772	3.556439	2.87758	2.991823	2.654096	2.702331	2.820346	2.661689	4.499694
SAC3D1	205449_at	8.042471	8.12848	6.801244	7.411426	8.078347	8.578122	8.526116	9.924383	8.565117	8.779053	10.15008	10.15199	7.63495
PHKA1	205450_at	2.173575	2.22493	2.344982	2.144074	2.153334	2.137714	2.149567	2.154138	2.144369	2.133919	2.133158	2.138317	2.146408
FOXO4	205451_at	6.002199	5.783577	6.167419	5.977909	6.268032	6.08548	6.823339	7.653061	7.401904	7.41717	7.48542	7.104631	5.409387
PIGB	205452_at	5.534165	5.762569	5.506846	5.818195	5.470924	5.423585	5.422489	5.582496	5.49151	5.418724	5.008284	5.415686	5.328188
HOXB2	205453_at	3.194324	2.881835	2.608497	2.505188	2.857495	2.863631	4.104138	3.468319	4.287458	4.027702	4.285156	3.503425	6.702939
HPCA	205454_at	2.818497	2.777242	2.683196	2.732287	2.80949	3.017296	2.766161	2.77789	2.745061	2.739241	3.12772	3.635736	2.831239
MST1R	205455_at	2.526669	2.655174	2.488261	2.492796	2.588498	2.491067	2.503803	2.440229	2.494364	2.489857	2.485872	2.498484	2.401009
CD3E	205456_at	2.092062	2.082839	2.067338	2.070747	2.076272	2.995961	2.079509	2.080697	2.069506	2.067073	2.065895	2.074465	1.943738
C6orf106	205457_at	3.602372	3.532956	3.519695	3.52465	3.593905	3.536569	3.547463	3.547912	3.38322	3.534864	3.530477	3.567693	3.432826
MC1R	205458_at	2.917638	2.873295	2.852458	2.858506	2.870524	2.969601	2.826686	2.853163	2.833499	2.846291	2.849062	2.86155	2.579455
NPAS2	205459_s_at	2.94375	2.917473	2.898034	2.895726	2.91626	2.900862	2.906124	2.91404	2.9036	2.88886	2.894636	2.907626	3.278597
NPAS2	205460_at	2.293042	2.272115	2.245512	2.253824	2.276013	2.254282	2.25952	2.272183	2.255444	2.249521	2.248923	2.265122	2.180627
RAB35	205461_at	2.426975	2.39504	2.357901	2.375414	2.398996	2.366913	2.393617	2.395486	2.38438	2.376365	2.379698	2.390057	2.344695

HPCAL1	205462_s_at	4.251184	4.381228	4.413275	4.390681	4.431159	4.059635	4.560637	4.428605	5.38347	4.755683	4.007467	4.194566	5.539288
PDGFA	205463_s_at	2.836292	3.025718	2.631913	2.667902	2.81997	3.399398	2.834429	2.806857	2.791746	2.622375	4.076329	2.803961	5.504418
SCNN1B	205464_at	2.120953	2.109976	2.074901	2.084298	2.108803	2.116548	2.094022	2.100308	2.138552	2.077746	2.105373	2.100404	2.033058
HS3ST1	205465_x_at	2.198292	2.185122	2.159736	2.168304	2.188929	2.165654	2.176841	2.184362	2.169747	2.162429	2.17017	2.178458	2.085257
HS3ST1	205466_s_at	2.847576	2.683346	2.632269	2.65745	2.7607	2.644983	2.656952	2.665949	2.660882	2.644588	2.675878	2.67711	5.648911
CASP10	205467_at	3.708756	3.696442	4.008318	4.208502	3.694498	3.67676	4.477342	4.144453	4.648735	4.818111	4.244564	4.685681	2.31414
IRF5	205468_s_at	2.554138	2.524107	2.472581	2.515183	2.529736	2.513146	2.525568	2.528915	2.515546	2.500229	2.517565	2.525994	2.432102
IRF5	205469_s_at	4.155365	4.206187	4.056233	4.118384	4.143775	4.417557	3.956956	4.118125	3.369994	3.597932	4.10198	4.114812	2.893935
KLK11	205470_s_at	2.378603	2.36609	2.332103	2.272604	2.572224	2.45116	2.338306	2.399273	2.364702	2.32391	2.351835	2.435953	2.358315
DACH1	205471_s_at	4.646344	4.688677	4.959897	4.943828	4.693618	4.662607	4.484367	4.666605	4.579504	4.436662	4.632097	4.795279	7.209342
DACH1	205472_s_at	4.279224	4.218435	4.004921	4.366281	4.353254	5.058712	3.918596	4.019798	3.114227	3.136491	4.729459	4.698051	4.85729
ATP6V1B1	205473_at	2.169718	2.154164	2.125938	2.135525	2.153161	2.132144	2.152047	2.155304	2.147183	2.14107	2.139017	2.147995	2.121174
CRLF3	205474_at	5.86899	6.164975	6.050352	5.949978	5.671826	4.910453	8.573116	8.425754	8.90652	8.716357	7.133558	8.174842	6.923151
SCRG1	205475_at	2.077447	2.06878	2.048105	2.05584	2.070127	2.058835	2.058761	2.067001	2.063528	2.044853	2.059454	2.060072	8.033702
CCL20	205476_at	2.681757	2.645859	2.680762	2.632125	2.605789	3.148663	4.004516	3.172783	3.671593	6.255631	3.161104	5.467597	2.93446
AMBP	205477_s_at	14.61635	14.63464	14.39222	14.59899	14.47593	14.39226	14.06577	14.02707	14.09567	14.19569	13.8896	14.20863	8.298398
PPP1R1A	205478_at	10.32533	10.24659	10.8882	11.10108	9.96982	9.776652	5.758014	7.056398	5.710396	6.148819	5.066701	6.140669	5.83227
PLAU	205479_s_at	2.366629	2.351411	2.334394	2.335319	2.352429	2.375676	2.347454	2.349085	2.336779	2.336083	2.33148	2.343375	3.547046
UGP2	205480_s_at	13.00224	12.99643	12.99009	12.93575	12.19539	11.38272	9.936529	9.122643	9.9949	9.885262	9.23016	8.990237	11.00744
ADORA1	205481_at	2.111501	2.095712	2.055392	2.075933	2.095719	2.076862	2.074929	2.091103	2.079914	2.071254	2.07565	2.087674	2.036493
SNX15	205482_x_at	4.751541	4.708975	4.622757	4.65484	4.727301	4.806259	5.415846	5.506066	5.439129	5.604294	5.082563	5.401918	5.178092
ISG15	205483_s_at	8.144569	8.138523	8.58076	8.764858	8.097841	8.659772	5.427652	7.082375	5.958726	5.681799	6.215867	7.135382	5.62594
SIT1	205484_at	2.457672	2.449353	2.411226	2.427009	2.456773	2.436563	2.435173	2.446701	2.441295	2.420674	2.42988	2.634696	2.296984
RYR1	205485_at	2.104266	2.086084	2.065675	2.067176	2.08567	2.068533	2.080794	2.083816	2.070772	2.067196	5.952242	2.077118	7.471046
TESK2	205486_at	5.181151	5.475612	5.531217	5.433671	5.135787	4.689172	5.349198	5.033711	5.402638	5.026693	4.430998	5.026275	3.652271
VGLL1	205487_s_at	2.602583	2.590956	2.570414	2.578157	2.5966	2.579577	2.584417	2.590376	2.561662	2.556528	2.554618	2.58055	2.512977
GZMA	205488_at	5.125735	4.36028	4.643211	4.042845	4.425621	5.166432	2.823988	2.847653	3.466781	2.984677	2.81983	2.841265	5.88045
CRYM	205489_at	6.064848	6.055698	6.826035	7.248114	6.87233	6.666238	7.956538	6.804691	6.217791	6.815962	6.251287	7.820663	7.134753
GJB3	205490_x_at	2.72724	2.721548	2.694336	2.699226	2.719597	2.708126	2.711648	2.717247	2.704963	2.697092	2.700273	2.711619	2.65422
GJB3	205491_s_at	1.977664	1.963822	1.94527	1.949541	1.966189	1.944981	1.960741	1.961673	1.947501	1.943516	1.947732	1.956261	1.893874
DPYSL4	205492_s_at	2.219836	2.210807	2.188564	2.196171	2.214754	2.192254	2.198435	2.207133	2.191344	2.194895	2.191764	2.203401	3.874786
DPYSL4	205493_s_at	2.243928	2.23158	2.174903	2.203464	2.237445	2.203861	2.217374	2.223308	2.208986	2.211122	4.843635	2.222845	6.997445
LOC55565	205494_at	2.188705	2.173013	2.152501	2.155338	2.173108	2.331942	2.161816	2.169888	2.15768	2.159362	2.152509	2.148547	2.831917
GNLY	205495_s_at	3.309064	2.67845	2.528006	2.527232	2.55627	2.731331	2.422978	2.545521	2.530643	2.433701	2.532552	2.540203	8.550867
KIAA0408	205496_at	2.950201	2.841806	2.763848	2.82714	2.933834	2.824238	2.873437	2.874797	2.848466	2.806083	2.869969	2.883025	3.318449
ZNF175	205497_at	5.129805	4.636286	4.968541	5.379274	5.023767	5.092039	6.062105	5.59528	7.033975	6.792332	5.090939	5.450106	5.418229
GHR	205498_at	12.58842	12.54438	12.13285	12.21843	10.96171	10.87395	6.773586	5.764277	6.26323	5.978039	6.824705	5.968188	7.879516
SRPX2	205499_at	2.709865	2.648106	2.857041	2.712797	2.767762	3.162381	2.697239	2.662099	2.649614	2.711664	4.806035	2.708114	5.360859

C5	205500_at	12.46742	12.53122	12.83369	12.84634	12.65442	12.29734	10.65069	10.52854	11.00685	10.42655	9.434433	10.12827	5.051566
CYP17A1	205502_at	2.683771	3.002068	3.820065	3.808654	2.380729	2.329872	2.15655	2.153483	2.150291	2.136802	2.142785	2.147607	10.71317
PTPN14	205503_at	2.332754	2.310168	2.287436	2.293935	2.316265	2.292479	2.304708	2.306448	2.301594	2.29181	2.288883	2.301599	2.266845
BTK	205504_at	3.561904	3.686046	3.710701	4.073137	4.329673	3.926871	6.409809	7.098955	6.163285	6.479011	5.948372	7.143577	3.389814
GCNT1	205505_at	2.524357	2.512865	2.483543	2.490426	2.602754	2.49557	2.501194	2.561654	2.502453	2.494188	2.521433	2.559153	2.552165
VIL1	205506_at	5.465313	4.866741	3.627363	3.706601	6.362675	6.205154	6.511427	5.721921	6.342169	6.423655	5.900748	5.854974	1.752814
ARHGEF15	205507_at	3.463093	3.647095	3.273482	3.332926	3.68831	3.332242	3.367528	3.346973	3.321432	3.32787	3.333439	3.366501	3.295793
SCN1B	205508_at	4.434494	4.669446	4.322343	4.316474	4.797531	6.191572	4.270189	4.281835	3.406398	3.617833	2.770871	4.297532	2.689672
CPB1	205509_at	2.294915	2.279824	2.248126	2.264248	2.279959	2.252609	2.276475	2.277648	2.26995	2.259302	2.266519	2.272271	2.226512
FLJ10038	205510_s_at	7.091534	6.625076	5.835948	6.084732	6.843582	6.678267	6.094273	6.247377	5.765896	5.60118	6.187912	5.981798	5.490531
FLJ10038	205511_at	4.910774	4.562295	5.161442	5.507443	4.986366	5.251051	4.282474	4.713592	4.159653	3.233462	4.902158	4.973119	4.517234
AIFM1	205512_s_at	8.432011	8.339172	9.041065	8.895681	8.23491	7.602004	8.867344	8.197211	8.75648	8.942305	8.081361	8.051917	6.339543
TCN1	205513_at	2.171115	2.154179	2.132029	2.135914	2.154725	2.135082	2.147742	2.15223	2.139317	2.136351	2.134953	2.143407	2.079169
ZNF415	205514_at	2.901216	2.842522	2.930013	2.90008	3.073201	2.932515	4.48622	3.359351	3.182813	3.215711	3.25946	3.000555	6.618461
PRSS12	205515_at	2.368396	2.339726	2.30163	2.319851	2.365329	2.312417	2.330439	2.344607	2.327351	2.312938	2.328069	2.341877	6.473372
CIZ1	205516_x_at	6.181192	6.229044	6.133647	6.145703	6.295152	6.450595	7.491618	7.703701	7.681057	7.568117	7.681194	7.828191	7.487069
GATA4	205517_at	7.535173	7.659584	7.574276	7.617533	7.291391	7.033058	7.496888	6.997327	7.727536	7.135174	7.230984	7.253892	5.687221
CMAH	205518_s_at	4.378532	4.66368	6.113483	5.419777	5.127848	4.232982	5.279407	3.871236	5.51841	4.669961	3.870592	3.864069	3.180753
WDR76	205519_at	3.165239	3.0548	2.980116	3.04235	3.071533	2.992813	5.165646	3.57884	5.875074	5.352875	3.462757	4.533401	3.722205
STRN	205520_at	2.384288	2.366939	2.34692	2.354491	2.380189	2.353507	2.354164	2.363665	2.350084	2.340525	2.348913	2.358611	2.317678
ENDOGL1	205521_at	2.961398	2.941559	2.647804	2.666982	2.820548	2.92629	3.005229	4.539966	2.928949	3.412128	3.431859	3.781177	5.593608
HOXD4	205522_at	2.107753	2.100377	2.077226	2.081671	2.101232	2.086787	2.091529	2.099295	2.083785	2.076597	2.5333	2.093481	6.683629
HAPLN1	205523_at	2.933223	2.842433	2.816505	2.862481	2.940603	2.836119	4.504784	3.019898	3.84113	2.851673	3.46356	4.868661	10.24189
HAPLN1	205524_s_at	2.121746	2.109065	2.086105	2.087193	2.108927	2.090005	2.545917	2.112593	2.095087	2.09096	2.089408	2.104437	5.954198
CALD1	205525_at	5.878844	5.884366	6.677736	5.92573	5.755504	4.751038	4.75266	4.426816	4.123981	4.13647	4.336493	4.057382	6.251409
KATNA1	205526_s_at	5.584195	5.663277	5.737937	5.837919	5.932971	5.518036	7.998503	7.549856	8.088892	8.222602	7.457748	7.788706	7.156571
GEMIN4	205527_s_at	5.856456	5.92335	5.530157	5.989868	5.909563	6.502407	5.576703	6.500072	5.635974	5.818867	6.465951	6.243877	6.206223
RUNX1T1	205528_s_at	3.924704	3.456444	3.596466	3.723339	3.855798	3.668542	5.635134	4.001812	4.908615	4.600889	6.127893	3.808271	9.130694
RUNX1T1	205529_s_at	3.876684	3.891015	3.441681	3.347375	4.312363	4.353065	5.177257	4.949715	4.258884	4.429768	6.993196	5.588513	8.822059
ETFDH	205530_at	8.663859	8.76638	9.802261	9.764803	9.124798	8.594208	7.707322	6.941793	7.961237	7.162342	6.466031	6.701765	5.968372
GLS2	205531_s_at	8.701926	8.852149	10.07632	10.35149	9.94124	9.427902	7.91346	7.639494	7.745523	7.183782	6.250505	6.413144	2.562062
CDH6	205532_s_at	2.632132	2.633967	2.368579	2.576981	2.704295	2.546636	3.063675	2.61134	2.581385	2.979597	2.815517	2.737026	3.859927
CDH6	205533_s_at	2.580923	2.541118	2.513735	2.525127	2.569255	2.513787	2.533042	2.544978	2.529866	2.510986	2.532865	2.545573	2.463226
PCDH7	205534_at	2.7246	2.673932	2.598893	2.639229	2.707993	2.715187	3.196843	2.700247	3.070098	2.754358	3.704347	3.088367	3.97248
PCDH7	205535_s_at	2.906799	2.884528	2.794245	2.867085	2.954412	2.843156	2.873319	2.882683	2.871452	2.850728	2.881102	2.918899	4.297043
VAV2	205536_at	5.22279	4.548189	4.60124	4.23754	3.849286	4.142908	4.105292	2.502343	3.360626	4.160489	3.865478	3.367937	2.756186
VAV2	205537_s_at	2.717803	2.655846	2.602262	2.658731	2.665076	2.665998	2.659928	2.665215	2.649698	2.628692	2.654458	2.657868	2.467958
CORO2A	205538_at	3.155691	3.034158	3.604002	3.948693	3.771771	4.219313	3.719957	4.45664	3.710377	3.785366	3.918355	4.286812	2.738985

AVIL	205539_at	4.748866	4.797626	4.845719	3.416109	3.129009	3.261688	2.848164	2.847577	2.850709	2.822457	2.841216	2.840965	3.566015
RRAGB	205540_s_at	2.340235	2.797709	2.303095	2.305282	2.312303	2.278461	2.324478	2.321998	2.621681	2.39205	2.306491	2.300753	4.867691
GSPT2	205541_s_at	7.234571	7.133105	7.451645	7.593155	7.533659	6.945457	6.769409	6.467713	6.882045	6.510959	6.878989	6.729423	7.832766
STEAP1	205542_at	6.632439	6.451973	7.243731	6.937541	5.483036	4.381735	8.942939	7.679708	9.240967	9.298485	7.659873	7.765608	4.877299
HSPA4L	205543_at	2.941869	2.637684	2.598146	2.650402	2.702565	2.617308	2.662675	2.631669	2.664597	2.630343	2.618889	2.627532	2.829619
CR2	205544_s_at	2.175493	2.163846	2.144303	2.148327	2.163091	2.146183	2.161972	2.161128	2.147879	2.144831	2.143815	2.171043	2.064762
DNAJC8	205545_x_at	8.414214	8.358573	8.394283	8.423044	8.372363	8.414629	9.024719	9.193187	9.190217	9.123304	9.097891	9.306608	9.122492
TYK2	205546_s_at	8.126063	8.198803	7.846345	7.802864	7.838203	7.539895	8.192534	7.820881	7.896837	8.135854	7.851491	7.926818	6.792816
TAGLN	205547_s_at	7.554077	7.647366	8.29455	8.269399	8.594599	8.71453	3.316916	3.409427	3.087962	3.588092	7.000108	3.701673	9.641562
BTG3	205548_s_at	4.880559	4.351268	5.082169	4.330045	3.538366	3.66936	8.659209	7.258215	8.743477	8.87339	7.269052	7.568235	8.272839
PCP4	205549_at	2.965186	2.896256	2.798583	2.871272	3.21431	2.876388	2.888546	2.852711	2.861769	2.860184	2.882091	2.889285	7.058218
BRE	205550_s_at	5.7647	5.828739	5.812673	5.851804	5.817796	5.778582	6.032675	5.741531	5.986172	5.811581	5.805652	6.033564	5.804994
SV2B	205551_at	2.0122	1.994419	1.991011	1.996109	2.007432	1.995096	2.311658	2.547407	2.058639	1.988763	2.120596	2.013298	2.548709
OAS1	205552_s_at	3.892299	3.965089	6.423694	6.231093	4.618823	3.487252	3.657274	3.212317	3.254355	3.181008	2.87298	3.205626	2.774244
CSRP3	205553_s_at	2.272881	2.242505	2.209237	2.223315	2.256853	2.220627	2.23139	2.243368	2.226767	2.217283	6.070432	2.237623	8.938649
DNASE1L3	205554_s_at	10.72493	10.5092	11.59353	11.69127	11.03807	10.81279	8.919806	8.623226	9.315618	9.197021	8.117198	8.417906	2.651945
MSX2	205555_s_at	2.532813	2.491741	2.468444	2.475895	2.620668	2.449465	2.489416	2.49934	2.485685	2.467121	2.48663	2.495673	2.466703
MSX2	205556_at	3.234107	3.14123	3.032739	3.112713	3.24521	3.052926	3.157014	3.157147	3.143312	3.138025	3.150294	3.383366	2.756293
BPI	205557_at	2.410853	2.49636	2.238476	2.425597	2.392803	2.37637	2.818014	3.526801	2.480891	3.43442	3.539452	3.034215	2.394471
TRAF6	205558_at	4.682302	4.732592	5.012606	5.226172	4.725372	4.718103	4.685022	4.467167	4.709623	4.716101	4.557857	4.476325	4.897502
PCSK5	205559_s_at	3.01168	3.089228	3.017204	3.067737	3.664234	3.232405	2.683158	3.091872	2.969759	2.67242	5.313524	3.352441	6.305681
PCSK5	205560_at	2.713752	2.682702	2.638849	2.671883	2.703303	2.671096	2.683362	2.693535	2.671364	2.666426	2.678084	2.687049	4.635722
KCTD17	205561_at	3.892092	3.864778	3.774792	3.846488	3.94035	4.627232	3.853023	3.870404	3.647738	3.670413	5.130318	4.1715	3.777474
RPP38	205562_at	5.946797	6.323859	6.709529	6.944539	6.151044	5.593774	6.619567	5.806117	6.642579	6.300563	5.904717	5.625016	5.926939
KISS1	205563_at	2.403765	2.695828	2.374633	2.374146	2.390288	2.381414	2.364937	2.443073	2.779776	2.380221	2.374023	2.3818	2.296338
PAGE4	205564_at	2.327454	2.31284	2.275165	2.28519	2.310926	2.291935	2.305576	2.310597	2.392814	2.288473	2.295087	2.305268	2.224561
FXN	205565_s_at	6.581367	6.911709	7.622466	7.854884	7.328275	6.993768	8.12402	7.544131	8.081278	7.943815	7.40965	7.833532	5.924016
ABHD2	205566_at	6.22471	5.933147	6.288092	6.666353	5.930523	5.361094	3.416254	4.426327	3.979723	3.880645	2.407525	3.30019	2.882417
CHST1	205567_at	2.186994	2.528805	2.141118	2.150791	2.174298	2.157151	2.166776	2.171061	2.157651	2.151744	2.588449	2.164872	5.407764
AQP9	205568_at	11.36591	11.36255	11.38708	11.32171	11.06871	10.84517	2.076061	2.07671	2.067822	1.985766	2.058526	2.069382	1.951381
LAMP3	205569_at	2.527313	2.472427	2.424818	2.446378	2.58289	2.796515	2.463046	2.466647	2.452331	2.460662	2.450709	2.463675	2.31298
PIP4K2A	205570_at	3.115832	3.581565	3.287905	3.109139	3.583914	3.563887	6.029319	6.443675	6.07726	6.438193	6.349776	6.356098	3.043322
LIPT1	205571_at	6.091643	5.919256	6.533522	6.33793	6.171458	5.303039	5.933	5.010918	5.878054	5.669248	5.545141	5.408425	6.369946
ANGPT2	205572_at	3.210377	3.139496	3.190038	3.650801	4.080603	4.134362	2.985206	2.753714	3.092501	2.972115	2.976185	2.97101	4.879385
SNX7	205573_s_at	7.063871	7.148761	6.724254	6.772165	6.411816	5.27743	7.982919	7.25766	7.791147	7.822698	7.477599	7.355314	8.155544
BMP1	205574_x_at	4.858105	4.958424	5.927895	5.256696	4.847289	4.30092	4.743851	4.336435	4.31393	4.81809	5.035829	4.872976	6.534921
C1QL1	205575_at	3.589476	3.573791	3.544132	3.556442	3.746424	3.554757	3.585611	3.569028	3.563001	3.55009	3.559018	3.563553	3.954961
SERPIND1	205576_at	13.23756	13.18794	13.45872	13.26661	12.74521	12.21952	12.70165	11.78606	12.78674	12.73056	11.44876	11.91841	6.209294

PYGM	205577_at	1.943628	1.935024	1.915555	1.920978	1.932666	1.917737	1.924277	1.930135	1.917451	1.91139	1.919208	1.924938	3.415814
ROR2	205578_at	3.026885	2.96941	2.846232	2.956384	3.026382	2.998108	3.004179	2.994986	3.0013	2.996715	3.001765	3.026335	4.432949
HRH1	205579_at	2.561132	2.54711	2.527165	2.53475	2.55627	2.530021	2.599003	2.551173	2.537365	2.534722	2.592206	2.579846	3.276279
HRH1	205580_s_at	2.44997	2.427315	2.407382	2.41417	2.433129	2.39496	2.423607	2.424837	2.41305	2.400095	2.411229	2.68304	2.577403
NOS3	205581_s_at	2.121765	2.108148	2.085769	2.095654	2.097443	2.095974	2.107503	2.101143	2.090796	2.08807	2.082655	2.096064	2.015975
GGTLA1	205582_s_at	6.017487	6.011416	5.352237	5.666464	6.567386	6.959707	4.568608	4.557875	4.4275	4.551383	4.278049	4.565183	4.116854
CXorf45	205583_s_at	8.725815	8.684155	8.424958	8.444591	8.90247	8.825129	7.508843	7.937571	7.759413	7.700896	8.369785	7.744475	8.309982
CXorf45	205584_at	6.71314	6.567914	7.244454	6.94384	6.645422	5.345451	6.135273	5.322409	6.316168	6.170071	6.321835	5.928884	6.823563
ETV6	205585_at	2.73156	2.730946	2.69928	2.909065	2.749208	2.701158	2.71492	2.71729	3.092109	2.696334	2.718628	2.712573	2.646635
VGF	205586_x_at	3.547454	3.488361	3.477356	3.483583	3.56406	3.491932	3.502163	3.502426	3.353669	3.467096	3.484498	3.495385	3.304829
FGFR1OP	205587_at	1.707008	1.699791	1.680921	1.683485	1.698429	1.685972	1.696736	1.694727	1.68591	1.683342	1.681229	1.689972	1.65811
FGFR1OP	205588_s_at	6.572778	6.742221	6.407429	6.267386	5.774413	5.857115	6.793317	6.351072	7.067901	6.918862	6.150311	6.263128	5.378067
MYL3	205589_at	2.074542	2.055156	2.021753	2.039334	2.059988	2.038449	2.048925	2.056785	2.042257	2.03388	6.774086	2.050318	8.537278
RASGRP1	205590_at	3.133219	3.103671	3.044865	3.113949	4.096325	5.86212	2.675491	2.721852	2.707156	2.649357	2.774007	2.810241	3.917332
OLFM1	205591_at	2.439183	2.361149	2.287638	2.330756	2.739454	2.905992	2.355553	2.357104	2.424189	2.340515	2.367705	2.38474	5.59846
IL8	205592_at	3.934206	4.023667	4.005851	4.002081	4.548694	4.581097	13.40113	13.66752	13.56668	13.60293	13.05997	13.43749	7.315361
PDE9A	205593_s_at	5.364477	5.440039	3.57531	3.745675	5.032719	4.911074	4.331394	5.177724	4.405874	4.609671	6.516263	6.01135	5.31145
ZNF652	205594_at	7.390844	7.326125	7.594157	7.6843	6.795588	6.772976	6.742999	6.256152	6.405042	6.45887	6.213839	6.231551	8.23584
DSG3	205595_at	2.148787	2.131289	2.10749	2.109639	2.136689	2.113428	2.126023	2.132215	2.118021	2.111285	2.116695	2.124262	2.244234
SMURF2	205596_s_at	6.544555	6.625085	5.951841	6.15928	6.837892	6.835817	6.231556	6.754487	5.943728	5.718853	7.697788	6.971228	8.114242
SLC44A4	205597_at	2.482496	2.459829	2.430175	2.443841	2.488989	2.447883	2.476518	2.469236	2.45342	2.446295	2.449368	2.462652	2.346863
TRAIP	205598_at	2.098562	2.060124	2.049825	2.047005	2.059811	2.054945	2.076475	3.638169	2.086822	2.51346	4.568894	3.044565	2.060904
TRAF1	205599_at	2.490974	2.484624	2.463712	2.468796	2.480169	2.607437	2.477601	2.479502	2.418313	2.460294	2.467284	2.472593	2.414159
HOXB5	205600_x_at	2.41982	2.40089	2.362933	2.381167	2.398491	2.370065	2.397291	2.398003	2.389364	2.385203	2.644724	2.391343	5.174917
HOXB5	205601_s_at	3.579633	3.68653	3.193103	3.359776	3.379673	3.461056	3.277943	3.376806	3.274985	3.256033	3.366787	3.510731	6.131117
PSG7	205602_x_at	2.156228	2.140025	2.110554	2.119862	2.138653	2.119768	2.130439	2.12179	4.011962	2.119208	2.123211	2.131176	2.119209
DIAPH2	205603_s_at	7.601071	7.515628	7.878789	7.852111	7.724436	7.797606	5.505202	5.635613	5.5425	5.179238	6.332726	5.714533	5.741051
HOXD9	205604_at	2.171035	2.162322	2.141284	2.140229	2.155506	2.147126	2.157371	2.153093	2.143589	2.139418	2.135208	2.139873	3.933015
HOXD9	205605_at	2.511112	2.493523	2.464362	2.473982	2.514565	2.472541	2.48166	2.493374	2.476196	2.46894	2.477794	2.486383	2.333404
LRP6	205606_at	5.45252	4.788174	5.262852	6.200081	5.011636	5.259446	4.256021	4.293169	4.312213	4.266441	4.57039	4.542004	5.534709
SCYL3	205607_s_at	5.721983	5.662891	5.450438	5.224102	5.238934	5.222342	5.122619	5.230017	5.260745	4.91718	5.047701	5.191192	5.178795
ANGPT1	205608_s_at	3.528301	3.354845	3.502283	3.439683	4.064617	3.755475	4.323699	4.391658	3.954337	3.899172	5.357958	4.854677	6.072872
ANGPT1	205609_at	3.478035	3.454568	3.100971	3.128893	3.898476	4.46946	5.548479	5.777882	4.47787	5.523924	6.679959	6.060164	7.149643
MYOM1	205610_at	8.135979	8.261849	5.97388	6.560273	6.932111	7.595845	2.649505	3.126214	2.641301	3.034038	6.685096	3.624563	7.585911
TNFSF12	205611_at	3.453487	2.98538	3.247917	3.45886	4.014531	4.147189	3.470009	3.47059	3.437297	3.453248	3.794552	3.979355	2.923489
MMRN1	205612_at	3.09909	2.650968	4.510761	3.428364	4.036107	3.047858	5.128177	3.243177	5.42318	4.506557	3.184738	4.364443	4.931084
SYT17	205613_at	6.33811	6.40305	6.307296	6.534678	7.785359	7.838947	3.961099	5.140428	4.539227	3.956402	4.406091	5.437772	3.976998
MST1	205614_x_at	12.10247	12.19413	11.10588	11.34766	11.82215	11.92543	11.20428	11.58405	11.09959	11.22986	10.98231	11.34258	5.788608

CPA1	205615_at	2.37209	2.337362	2.305331	2.328753	2.398982	2.324756	2.332276	2.342251	2.332902	2.317462	2.329901	2.337915	2.186064
PRRG2	205617_at	1.964911	1.952795	1.932614	2.182731	1.96069	1.931756	1.947251	1.952638	1.937604	1.967087	1.929929	1.943138	1.879825
PRRG1	205618_at	6.026757	5.824442	6.463872	6.77919	6.266273	5.707504	4.389399	3.516075	4.435015	4.524723	4.160063	4.167547	6.662084
MEOX1	205619_s_at	2.790829	2.717873	2.662307	2.724077	2.799054	2.732525	2.73021	2.734826	2.725809	2.695733	5.455668	2.753893	7.565123
F10	205620_at	9.3596	9.480137	9.002145	9.235126	9.176522	9.084641	8.004242	8.632277	8.466902	8.397673	8.416524	8.563732	3.567828
ALKBH1	205621_at	6.173792	6.138068	6.139308	6.304853	6.253563	6.225545	7.267499	6.266717	7.19525	7.065642	6.37751	6.322004	6.732537
SMPD2	205622_at	2.500054	2.385379	2.45844	2.468282	2.477688	2.942429	2.485619	2.491824	2.694776	2.824703	2.792761	2.601824	2.48149
ALDH3A1	205623_at	2.324994	2.299324	2.278692	2.280049	2.315635	2.279056	2.294898	2.58895	2.28491	2.274346	2.309834	2.29434	2.198724
CPA3	205624_at	2.049707	2.039118	3.480916	2.04554	2.051623	2.027887	2.121865	2.718886	2.193137	2.045232	2.038118	4.691902	1.946321
CALB1	205625_s_at	2.5398	2.499612	2.469941	2.495826	2.523427	2.473989	2.496568	2.507165	2.493017	2.479083	2.491938	2.503955	4.613909
CALB1	205626_s_at	3.064225	3.012496	2.943647	2.957817	3.0761	3.010024	3.004384	3.014198	2.974488	2.942843	3.034443	3.032395	5.992627
CDA	205627_at	7.312363	7.181863	8.432387	8.141137	6.822943	6.269477	2.943526	2.759538	2.290135	2.736983	2.291035	4.111665	2.18202
PRIM2	205628_at	3.984631	3.869254	3.740617	3.932573	3.957918	3.933842	5.279468	5.854303	5.217441	4.932629	4.519632	5.21253	4.891435
CRH	205629_s_at	2.865551	2.837491	2.755427	2.817789	3.235346	2.810721	2.790927	2.834009	2.95127	2.83204	2.81564	2.827605	2.29734
CRH	205630_at	2.124249	2.112097	2.088974	2.096248	2.118095	2.095832	2.105042	2.112829	2.09718	2.091192	2.110876	2.107662	3.125138
KIAA0586	205631_at	4.239834	4.50275	4.115423	4.433541	4.153678	4.559451	5.964639	6.103087	6.044607	6.683383	5.202337	5.955418	5.425489
PIP5K1B	205632_s_at	2.801008	2.733419	2.744582	2.749188	2.770312	2.705275	9.033665	8.259228	9.221055	9.151699	7.712741	8.437149	4.468481
ALAS1	205633_s_at	10.12317	10.04673	11.92232	11.64441	10.56911	10.38598	7.44487	7.303725	7.921362	7.888018	7.004462	7.175702	7.769652
ZDHHC24	205634_x_at	3.343237	3.336302	3.313921	3.321441	3.336104	4.178694	3.239946	3.334358	3.004332	3.313076	3.077464	3.328182	2.455207
KALRN	205635_at	2.473025	2.476144	2.393937	2.419766	2.466014	2.424555	3.199554	2.459949	2.482995	2.627862	2.446934	2.463339	2.439126
SH3GL3	205636_at	1.962769	1.952196	1.926802	1.935682	1.948534	1.940777	1.943057	1.950576	1.936366	1.931871	1.933855	1.941137	1.878229
SH3GL3	205637_s_at	1.887394	1.864472	1.838466	1.845767	1.861801	1.849878	1.862773	1.891286	1.850321	1.850977	3.049609	2.040263	7.345293
BAI3	205638_at	2.2308	2.186374	2.156437	2.188577	2.217974	2.167039	2.188055	2.191738	2.172976	2.171964	2.184281	2.192936	7.614035
AOAH	205639_at	4.759771	4.523566	5.235973	5.465453	5.803432	6.320196	4.170223	4.650523	4.084141	4.119951	3.747664	4.60446	2.550199
ALDH3B1	205640_at	3.510487	3.336264	3.471906	3.429911	3.497337	3.479084	3.490551	3.740479	4.075526	3.840871	3.780616	3.495471	2.586844
TRADD	205641_s_at	7.187968	7.372719	7.323358	7.71177	7.678286	7.881957	6.29928	6.929906	6.29454	6.271619	6.294778	6.957445	5.434496
CEP110	205642_at	2.942777	3.016164	2.798864	2.998556	3.010032	2.997635	4.440612	3.076774	4.36373	4.028822	3.502397	3.481977	4.497915
PPP2R2B	205643_s_at	2.52181	2.480695	2.434778	2.46326	2.491171	2.436599	2.476164	2.48719	2.476064	2.469606	2.469404	2.499951	2.410355
SNRPG	205644_s_at	11.28289	11.17937	11.48982	11.59278	11.46943	11.40092	12.54405	12.2248	12.44549	12.60952	12.17582	12.14288	12.0562
REPS2	205645_at	3.640033	3.577736	3.938563	4.008137	3.538886	3.567739	3.41709	3.809498	3.533755	3.542021	3.279242	3.576872	3.251361
PAX6	205646_s_at	2.725724	2.708079	2.68873	2.698092	2.704867	2.772039	2.698352	2.699329	2.691704	2.691951	2.684974	2.697951	4.65242
RAD52	205647_at	3.174638	2.662512	3.346736	3.152625	3.638442	3.234197	3.908506	3.855192	3.155998	3.141256	3.1511	3.324729	4.057624
WNT2	205648_at	2.888203	2.482997	3.743219	2.475083	3.013311	2.507017	2.4816	2.485713	2.411178	2.46901	2.659682	2.420549	4.309527
FGA	205649_s_at	13.21105	13.17974	13.82476	13.69515	13.22966	12.74155	12.55343	11.99008	12.58557	12.33341	11.08042	11.60638	6.216874
FGA	205650_s_at	14.3504	14.32828	14.53132	14.5795	14.48663	14.07724	13.70352	12.76194	13.60937	13.43224	12.48183	12.60916	7.147089
RAPGEF4	205651_x_at	6.979281	6.47785	7.075863	7.061472	7.168459	6.209172	3.59584	2.999609	3.417347	3.412342	3.183206	2.921442	5.312426
TTLL1	205652_s_at	3.328032	3.299664	3.276359	3.278141	3.298765	3.282145	3.286176	3.294539	3.289362	3.271418	3.358626	3.292313	5.275616
CTSG	205653_at	3.494129	3.496132	3.470878	3.475384	3.489766	3.051761	3.485577	3.487227	3.293663	3.91968	3.691448	4.607793	3.139245

C4BPA	205654_at	13.48363	13.48589	13.7908	13.91947	13.63813	13.29323	3.919471	7.257136	5.852352	7.485364	8.249635	6.13715	2.456616
MDM4	205655_at	3.434938	3.324442	3.189766	3.303769	3.429165	3.279778	3.379625	3.303488	3.344523	3.33815	3.346753	3.44251	4.878081
PCDH17	205656_at	5.288958	5.33874	4.7521	4.805652	5.327502	5.798615	6.015675	6.598879	4.719038	4.943353	7.646421	6.81142	7.749703
HAAO	205657_at	8.27384	8.286345	8.861141	8.87376	9.2559	8.752191	4.93128	5.537203	4.977362	5.227878	5.478545	5.484101	2.813244
SNAPC4	205658_s_at	6.412285	6.579723	6.307798	6.339984	7.192398	7.36841	5.870509	6.441194	5.604616	6.151092	7.039222	6.593102	4.854545
HDAC9	205659_at	4.126377	4.205897	3.984478	4.001174	4.696577	4.381264	4.062397	3.622486	3.547866	3.669918	4.185338	4.152102	5.731344
OASL	205660_at	2.957751	2.942429	5.670856	5.748392	4.338533	4.693058	2.35403	2.250401	2.299482	2.348583	2.236417	2.282252	2.432741
FLAD1	205661_s_at	4.695032	4.736071	4.837755	4.860484	3.993899	4.648365	6.038983	5.127725	6.102675	6.057627	4.913095	5.424282	5.881655
B9D1	205662_at	4.702182	4.873922	4.224428	3.984255	4.538444	5.452134	4.501099	5.602339	4.654432	5.459254	5.32246	5.337955	3.645204
PCBP3	205663_at	1.9286	1.985051	1.898728	1.907908	1.91795	1.910866	1.920879	1.918761	1.908532	1.907308	1.898247	1.91185	2.0089
KIN	205664_at	6.697278	6.506174	6.503126	6.907651	6.502902	6.156538	7.312196	6.901895	7.276609	7.257017	6.786839	7.016761	7.144492
TSPAN9	205665_at	3.973257	3.777832	3.760495	3.757532	3.775308	3.727134	3.737843	3.761622	3.759634	3.739921	3.754983	3.756781	3.347207
FMO1	205666_at	3.485812	4.182099	2.710852	2.727134	2.742045	2.728323	8.834038	8.534255	8.25584	8.219608	8.07932	8.103426	6.135461
WRN	205667_at	5.615771	5.757424	5.201012	5.467562	4.982535	4.240497	7.794653	7.933985	7.708396	7.848935	6.764707	7.204215	6.075535
LY75	205668_at	5.205265	4.975757	5.068093	5.089022	5.091242	5.682669	4.3694	4.581739	5.420763	5.797837	5.25888	5.058749	3.232139
NCAM2	205669_at	2.632297	2.588877	3.677056	3.734206	2.618548	2.594035	2.589616	2.60048	2.581597	2.574125	2.597095	2.602717	2.546071
GAL3ST1	205670_at	5.62484	5.237155	4.152425	3.913417	4.553419	4.540693	5.434357	5.508541	4.627458	6.08273	5.03935	4.948161	4.102888
HLA-DOB	205671_s_at	2.147946	2.257006	2.108882	2.114964	2.130747	2.110705	2.117384	2.126564	2.111627	2.104527	2.105964	2.119982	2.034533
XPA	205672_at	8.595945	8.389521	7.788364	8.113785	8.30577	8.517048	7.264738	7.898124	7.575133	7.317259	8.009635	8.031146	7.518864
ASB9	205673_s_at	6.088641	6.183557	7.338486	7.289737	8.198577	7.994944	7.198425	7.206775	6.360905	6.475113	6.549018	6.747108	4.858122
FXYD2	205674_x_at	5.775945	6.266842	4.722503	5.029861	5.460129	4.908659	9.536101	9.54629	9.663333	9.892284	9.325173	9.682629	4.70898
MTTP	205675_at	11.44417	11.39337	11.64748	11.75361	11.01086	10.69238	10.29605	9.918313	10.75474	10.69424	10.17028	10.37101	3.921283
CYP27B1	205676_at	2.08418	2.076344	2.056165	2.062589	2.071522	2.066282	2.071945	2.070886	2.06484	2.063718	2.062364	2.06739	1.987981
DLEU1	205677_s_at	6.632657	6.212335	7.121586	7.28853	6.759253	6.582549	7.193252	7.08243	7.572398	7.23901	6.773975	6.961398	6.302548
AP3B2	205678_at	2.441032	2.412204	2.363727	2.389806	2.426313	2.395456	2.401053	2.409851	2.396755	2.3857	2.395127	2.406169	3.906061
ACAN	205679_x_at	2.221818	2.546987	2.518277	2.532338	2.550609	2.531635	2.547451	2.546858	2.53388	2.523625	2.530465	2.613264	6.900612
MMP10	205680_at	2.514711	2.480248	2.452976	2.4676	2.530639	2.461372	2.473868	2.484884	2.471168	2.453991	2.473137	2.482434	5.576153
BCL2A1	205681_at	3.4325	3.052186	5.068912	3.514523	3.703398	3.41847	2.709523	2.719411	2.705816	2.681104	3.089318	2.731893	3.302985
APOM	205682_x_at	11.63724	11.74864	11.83307	11.70832	11.63594	11.26771	12.94132	12.38467	12.94693	12.90278	12.09313	12.43626	6.703346
TPSAB1	205683_x_at	2.676174	2.666897	2.944844	3.601107	2.783306	2.655355	2.669599	2.666755	2.650057	2.577257	2.650664	2.658837	2.574874
DENND4C	205684_s_at	7.350467	7.549245	7.481706	7.679003	6.94441	6.536448	6.562444	6.410197	6.786269	6.78683	6.169772	6.170218	6.454868
CD86	205685_at	4.521071	4.351107	4.624282	4.763012	4.670765	4.729661	4.651856	4.557389	4.440314	4.034782	4.646274	4.657172	3.829039
CD86	205686_s_at	2.241239	2.243001	2.400513	2.231221	2.486665	2.772399	2.108205	2.232306	2.151921	2.081117	2.187651	2.234462	2.002445
UBFD1	205687_at	4.823912	5.354236	5.195063	5.172836	5.146893	5.025652	5.343327	6.013603	6.202932	6.218293	6.471704	6.197822	6.21033
TFAP4	205688_at	3.273085	3.260768	3.233853	3.245862	3.304844	3.242164	3.257695	3.258127	3.244234	3.478892	3.237579	3.395304	3.193424
PCNXL2	205689_at	2.041869	2.024672	2.00385	2.007214	2.025202	2.013253	2.016422	2.022636	2.008078	2.00049	2.008656	2.016991	1.993025
BUD31	205690_s_at	8.576988	8.55357	8.256069	8.528296	8.909097	8.864893	8.767809	9.283263	9.025756	8.973992	9.212594	9.496855	8.507757
SYNGR3	205691_at	2.302775	2.278467	2.256139	2.259549	2.287062	2.262598	2.389944	2.306975	2.27681	2.270027	2.264178	2.292692	5.759838

CD38	205692_s_at	2.566889	2.644639	2.628573	2.523867	2.742594	2.634663	3.083196	2.531256	2.687911	2.633615	2.513897	2.714092	2.325101
TNNT3	205693_at	2.191774	2.176938	2.158206	2.162845	2.174494	2.159177	2.166892	2.175036	2.170059	2.161367	7.622696	2.169653	7.511451
TYRP1	205694_at	2.275593	2.248265	2.215335	2.229582	2.25768	2.224443	2.237051	2.246881	2.232735	2.223033	2.234238	2.243315	4.307178
SDS	205695_at	10.7832	10.89724	11.87122	12.09991	9.156302	8.992529	1.869224	2.041146	2.168446	2.020243	2.023884	2.035886	1.969405
GFRA1	205696_s_at	4.681809	4.679302	4.810292	5.153551	4.146328	3.846962	2.369288	3.892385	2.350122	3.138658	2.444132	2.335456	4.086118
SCGN	205697_at	2.224555	2.199226	2.175224	2.180866	2.257942	2.18566	2.191626	2.19759	2.183343	2.175473	2.183018	2.192405	2.09366
MAP2K6	205698_s_at	4.547427	4.605985	6.314517	6.087044	4.154574	3.752732	4.098869	4.15137	3.798628	4.218366	4.134827	4.089573	5.348263
MAP2K6	205699_at	2.459085	2.426023	2.387713	2.406012	2.436861	2.412627	2.414902	2.426019	2.41096	2.411423	2.411025	2.421047	2.386821
HSD17B6	205700_at	12.48993	12.50718	13.46471	13.38554	12.57617	11.97105	3.094834	3.501653	3.832839	3.079373	2.471865	3.091517	3.035099
IPO8	205701_at	2.261516	2.237635	2.21374	2.222567	2.273903	2.223835	2.231244	2.241039	2.225608	2.220865	2.221414	2.235495	2.57374
PHTF1	205702_at	2.448401	2.766881	3.102083	2.910905	2.821578	2.2497	3.098131	2.816809	3.109062	2.794343	2.693942	2.741923	2.724628
ATP6V0A2	205704_s_at	3.229161	3.191595	3.333265	3.203036	3.084398	3.074183	5.007225	4.275482	5.675957	5.220093	3.521339	4.38994	4.121523
ANKRD26	205705_at	2.551912	2.528904	2.884814	2.511978	2.530256	2.502318	2.525524	2.524005	2.554802	2.502453	2.533314	2.535773	3.542456
ANKRD26	205706_s_at	3.346647	3.636658	3.320716	3.39294	3.944047	3.398029	3.872218	3.343333	3.503614	3.345157	3.323411	3.426758	4.173912
IL17RA	205707_at	5.300229	6.069942	6.284894	6.168377	5.708856	5.368854	4.903175	4.721729	5.089752	4.89098	5.212801	5.082733	4.71433
TRPM2	205708_s_at	2.322182	2.278127	2.25744	2.26038	2.283299	2.266667	2.273719	2.278552	2.263275	2.258232	2.26114	2.687458	2.186022
CDS1	205709_s_at	3.736728	3.499405	3.618672	3.808292	3.720049	3.932539	3.587349	3.707676	3.680192	3.604435	3.878966	3.666806	4.977742
LRP2	205710_at	2.688076	2.580095	2.530123	2.594459	2.735968	2.601295	2.581988	2.613436	2.598012	2.579616	2.672501	2.608145	2.553626
ATP5C1	205711_x_at	11.10945	10.99061	11.36271	11.42831	10.44	10.13519	11.31948	10.84007	11.43805	11.41362	10.84909	10.6763	11.29089
PTPRD	205712_at	2.334705	2.309228	2.304588	2.308679	2.349206	2.310754	2.308184	2.317245	2.303528	2.284173	2.300788	2.312747	6.946845
COMP	205713_s_at	2.394019	2.368293	2.330413	2.34896	2.377246	2.331703	2.353099	2.365231	2.347389	2.336333	2.354512	2.361903	2.945455
ZMYND10	205714_s_at	2.861263	2.833502	2.803763	2.816812	2.849253	2.801498	2.825118	2.836763	2.818563	2.808311	2.816817	3.290262	2.729879
BST1	205715_at	2.417672	2.397738	2.337292	2.378077	2.401381	2.376224	4.62997	2.443056	4.020182	4.409445	3.461617	2.565547	2.304843
SLC25A40	205716_at	2.594576	2.574857	2.55473	2.558809	2.609793	2.563386	2.604423	2.571599	2.818025	2.644831	2.563675	2.575685	2.989458
PCDHGA1	205717_x_at	5.8829	5.872072	5.873024	5.85035	6.435486	5.860792	5.857685	4.954021	5.676383	5.765253	6.811177	5.477131	8.365474
ITGB7	205718_at	2.616827	3.053109	2.855913	3.616172	2.692696	2.875483	2.661648	2.667922	2.656633	2.53702	2.643116	2.661697	2.609968
PAH	205719_s_at	13.60292	13.66203	14.11921	13.91297	13.03928	12.91086	13.0592	12.16957	12.95761	12.90009	11.43717	11.78199	7.096718
POMC	205720_at	2.597187	2.586967	2.570503	2.664526	2.543023	2.560052	2.5862	2.590853	2.655088	2.5738	3.273351	2.830071	2.528728
GFRA2	205721_at	2.540338	2.512065	2.196543	2.485562	2.512575	2.467555	2.511056	2.793816	2.502989	2.77435	2.648074	2.808866	3.027372
GFRA2	205722_s_at	2.058206	2.026915	2.003282	2.007536	2.027742	2.007162	2.019234	2.159997	2.01267	2.01059	2.006936	2.019501	2.129552
CNTFR	205723_at	2.387032	2.382224	2.355576	2.363147	2.369987	2.3618	2.37731	2.397068	2.365623	2.35802	2.365198	2.359522	2.332088
PKP1	205724_at	1.937017	1.922739	1.903094	1.907273	1.932715	1.909019	1.91655	1.920546	1.908498	1.901284	1.903397	1.913634	1.855774
SCGB1A1	205725_at	2.048087	2.029113	2.003594	2.012676	2.031346	2.011283	2.024706	2.028654	2.016899	2.007815	2.009933	2.022717	2.07651
DIAPH2	205726_at	7.615057	7.504823	7.79165	7.732088	7.194931	6.873989	4.806864	4.974793	5.486225	4.842065	5.139518	5.072664	5.354934
TEP1	205727_at	3.647141	3.348076	3.159894	3.069734	3.358017	3.222615	3.147973	2.977361	2.948407	3.029005	2.974572	2.991713	3.168156
OSMR	205729_at	2.617071	2.574705	2.545689	2.55476	2.605566	2.556433	2.566834	2.576543	2.560136	2.552881	2.558926	2.581696	3.188762
ABLIM3	205730_s_at	7.751936	7.845715	7.761768	7.787979	8.267055	7.896257	4.423911	5.11324	3.74642	4.496442	7.315035	5.461492	7.189702
NCOA2	205731_s_at	5.639974	5.039757	5.248998	5.630985	4.839422	4.718569	4.690288	4.227181	4.718038	4.207495	4.657551	4.673936	4.883399

NCOA2	205732_s_at	2.772195	2.706627	2.694798	2.674206	2.740974	2.669516	2.709524	2.698719	2.741791	3.369432	2.69641	2.718264	5.750097
BLM	205733_at	2.126289	2.130047	2.07515	2.088727	2.109184	2.091426	6.563397	6.657594	7.023942	6.774889	6.244123	6.659197	4.386104
AFF3	205734_s_at	2.758849	2.707451	2.640583	2.691308	2.748328	2.682455	2.70345	2.712755	2.698976	2.685395	2.69768	2.731366	2.493047
AFF3	205735_s_at	2.410463	2.355863	2.310249	2.353164	2.380827	2.931805	2.357845	2.367547	2.355095	2.341573	2.345831	2.362313	2.311673
PGAM2	205736_at	2.196606	2.182558	2.154633	2.173027	2.184665	2.172474	2.18344	2.389816	2.441798	2.173401	2.234807	2.177439	4.872515
KCNQ2	205737_at	1.866237	1.855348	1.837104	1.841498	1.856622	1.841574	1.852793	1.853605	1.844347	1.837271	1.835081	1.84728	5.173701
FABP3	205738_s_at	2.682498	2.591818	2.609667	2.615408	2.634171	2.613592	2.813534	2.991815	3.229284	2.674376	3.108858	2.721441	5.433285
ZNF107	205739_x_at	3.518677	3.379109	2.976318	2.676925	3.405136	3.351981	5.486255	5.051879	5.952897	5.820359	4.345164	4.847001	5.466142
RBM42	205740_s_at	7.117327	7.105901	7.185549	7.309461	7.315759	7.310095	8.068738	8.422365	7.901869	7.928543	8.537272	8.638394	7.468538
DTNA	205741_s_at	2.465047	3.175219	2.050653	2.053719	2.092629	2.182185	2.455651	2.206285	2.147075	2.190077	4.823481	2.199855	5.646803
TNNI3	205742_at	2.759868	2.731698	2.706325	2.667418	2.731908	2.718292	2.72975	2.979045	2.897619	2.876052	4.266129	4.05129	5.591482
STAC	205743_at	2.18886	2.494701	2.183544	2.167271	2.178067	2.175172	2.168146	2.11794	2.117926	2.160278	2.315676	2.165812	3.994125
DOC2A	205744_at	2.208797	2.188482	2.17883	2.172089	2.195499	2.181016	2.193284	2.192819	2.181638	2.184125	2.208332	2.188689	2.092173
ADAM17	205745_x_at	6.05539	6.205977	5.86001	5.900484	5.884956	5.830035	5.932793	6.056293	5.97627	6.22525	6.133076	5.991147	6.257707
ADAM17	205746_s_at	2.673175	2.649182	2.646254	2.637738	2.655386	2.620969	2.776818	2.653932	2.657247	3.120961	2.636935	2.642021	4.516793
CBLN1	205747_at	2.397345	2.314572	2.279849	2.293521	2.360126	2.291693	2.299454	2.310047	2.290844	2.27989	2.293136	2.307145	2.824865
RNF126	205748_s_at	6.380989	6.038002	6.300312	6.044756	5.990074	5.697583	6.256808	5.835476	6.408608	6.302405	6.02161	5.971284	6.257105
CYP1A1	205749_at	7.030338	6.672152	8.828763	8.683225	7.330545	6.829109	3.709833	3.722188	3.748718	3.6975	3.688591	3.803046	2.40779
BPHL	205750_at	9.71886	9.752963	8.92254	8.978857	8.477038	8.034191	8.628611	8.187725	7.366777	6.965527	7.158675	8.008994	6.001968
SH3GL2	205751_at	2.444182	2.408103	2.365313	2.394472	2.451592	2.396101	2.399841	2.411258	2.3979	2.383073	2.399806	2.406915	5.452531
GSTM5	205752_s_at	3.636072	3.811473	3.503173	3.061689	3.847639	4.794216	3.806809	3.511159	3.722858	3.516951	4.939202	4.899888	6.695799
CRP	205753_at	8.722443	8.826601	9.210712	8.382001	7.040231	7.107107	2.74464	2.792142	2.741743	2.729223	2.741452	2.750979	2.306212
F2	205754_at	13.10934	13.13476	13.12841	13.24499	13.26612	13.15177	12.37255	12.23792	12.47031	12.25578	12.03288	12.37425	5.311175
ITIH3	205755_at	13.18297	13.21761	12.61346	12.72879	13.65708	13.5848	12.35226	11.83577	11.31146	12.22032	12.06894	12.53881	5.679546
F8	205756_s_at	6.46456	6.553835	7.98921	7.803587	7.346133	6.513768	4.984755	4.941643	5.396515	5.577693	4.532945	5.282706	3.785146
ENTPD5	205757_at	9.250039	9.061938	9.313898	9.383205	8.798275	8.485405	6.50868	5.903674	5.448606	5.344534	6.145195	5.993124	3.311165
CD8A	205758_at	4.384689	4.198906	4.41829	4.242054	4.134053	5.427159	3.424334	3.090063	3.07248	2.538866	3.399361	3.465639	3.175513
SULT2B1	205759_s_at	3.938342	3.914006	3.862752	3.894626	3.949969	3.902589	3.902971	3.90946	3.899457	3.873035	3.900299	3.91261	3.429872
OGG1	205760_s_at	6.589335	6.297351	6.110741	6.14168	6.526409	6.483575	6.528331	6.691297	6.533432	6.551436	6.577998	6.792551	7.644486
DUS4L	205761_s_at	4.620749	4.777787	5.565121	5.710928	4.784429	4.467042	5.308324	4.432284	6.017675	5.617064	4.613963	4.629443	5.086649
DUS4L	205762_s_at	5.344267	5.232975	4.847681	4.720101	4.813802	4.410049	4.38773	4.646711	4.365983	4.348472	4.242239	4.708259	4.609467
DDX18	205763_s_at	5.813977	5.910933	6.724531	6.131276	6.021194	6.045741	7.233866	6.467209	7.252933	7.424428	5.978167	5.826841	7.356475
CYP3A5	205765_at	12.18834	12.24554	12.46799	12.42298	13.12058	12.66152	6.743828	8.978177	7.311046	7.16555	6.44057	7.05965	2.648008
TCAP	205766_at	3.460133	3.433084	3.464486	3.246912	3.442998	3.925885	3.785201	3.586378	3.494043	3.976684	3.711636	3.977217	3.888129
EREG	205767_at	3.027085	2.962654	2.897926	2.921042	3.017793	2.943059	2.951102	2.987224	2.974868	2.982856	2.972851	2.991729	2.641938
SLC27A2	205768_s_at	11.02633	10.97394	12.41869	12.2306	11.20177	10.66804	10.00299	8.669627	9.833787	9.299199	8.792878	9.11687	3.91778
SLC27A2	205769_at	10.9064	10.73788	12.1516	11.96161	10.88919	10.39349	9.457891	8.598616	9.321786	9.112941	8.547742	8.766358	4.044529
GSR	205770_at	4.999199	4.783473	4.932312	5.042622	5.531305	5.534963	6.382018	5.986304	6.212344	6.129411	5.854204	6.055534	4.600933

AKAP7	205771_s_at	8.684549	8.734649	8.25072	8.29648	8.217472	8.22192	8.322657	8.360081	8.605316	8.284436	7.186986	8.239033	8.330617
AKAP7	205772_s_at	2.383374	2.354813	2.313942	2.340958	2.367425	2.32537	2.342206	2.349893	2.334861	2.324784	2.336986	2.345357	2.232609
CPEB3	205773_at	8.505283	8.384022	9.249507	9.178329	8.180565	7.799222	4.757971	5.149658	5.285097	5.267555	4.949243	5.140077	4.902659
F12	205774_at	12.94667	12.99691	12.79729	13.15553	13.20053	13.35629	11.05116	11.81211	11.11173	11.01103	11.18468	11.74386	4.581744
FAM50B	205775_at	6.799045	6.914165	6.648646	6.854852	7.608732	7.230279	6.654891	6.810611	6.36744	6.611229	6.596189	6.538266	5.89838
FMO5	205776_at	11.76815	11.80393	11.26005	11.18947	10.83867	10.43654	7.472945	6.983299	7.889293	7.379476	5.415233	6.883248	2.679497
DUSP9	205777_at	2.591412	2.480753	2.335079	2.417037	2.361917	2.410702	10.57631	10.74631	10.75432	10.6649	10.23629	10.60986	3.067582
KLK7	205778_at	2.324286	2.303593	2.284366	2.296174	2.318314	2.276747	2.303762	2.314158	2.299113	2.450273	2.30057	2.308584	2.222971
RAMP2	205779_at	3.384321	3.37333	3.355949	3.342583	3.360801	3.358905	4.446343	3.405353	3.442916	3.427505	4.869447	4.869222	5.624211
BIK	205780_at	2.555239	2.554931	2.51573	2.517399	2.539623	2.519255	2.535972	2.53717	2.525948	2.516009	2.522235	2.541701	2.753392
C16orf7	205781_at	5.862779	5.750333	4.936793	5.26228	5.766743	6.298999	3.504538	5.008973	3.9489	4.289276	5.314857	4.940039	4.19998
FGF7	205782_at	2.507085	2.471552	2.401091	2.458423	2.483513	2.468696	2.463408	2.474879	2.460199	2.45507	2.463579	2.469401	3.673198
KLK13	205783_at	2.153526	2.105482	2.088475	2.085054	2.102525	2.093704	2.096092	2.104749	2.093174	2.088028	2.078288	2.097009	2.059146
ARVCF	205784_x_at	5.41879	5.587794	5.221886	5.296771	5.327421	5.524058	5.312241	5.320548	5.325904	5.274109	5.308652	5.380664	4.821518
ITGAM	205785_at	2.415398	2.399234	2.377645	2.383743	2.398708	2.383232	2.39357	2.397977	2.387299	2.37907	2.381096	2.392814	2.314679
ITGAM	205786_s_at	4.03715	3.693879	4.08823	4.090687	3.95713	4.022005	3.821278	4.040294	3.054945	3.700424	3.941794	4.069144	2.963763
ZC3H11A	205787_x_at	6.073682	5.39148	5.442848	6.164803	6.718782	7.320502	5.513676	6.311646	5.323315	4.379637	6.947575	6.579554	6.485329
ZC3H11A	205788_s_at	9.791324	9.74764	9.835329	9.781375	9.424659	9.195725	9.708295	9.316706	9.697423	9.610097	9.555295	9.310128	10.59513
CD1D	205789_at	5.942469	5.906097	6.846981	6.778808	6.963108	6.76148	3.166202	3.215276	3.825792	3.714996	3.371157	2.989697	2.515143
SKAP1	205790_at	7.587479	7.885477	7.928667	8.012994	8.009316	7.229198	3.800422	3.812384	3.797328	3.796757	2.606992	3.80669	2.874869
ZNF230	205791_x_at	2.466206	2.435223	2.418708	2.41334	2.445083	2.400169	2.583376	2.446211	2.645271	2.703534	2.417302	2.433413	3.613789
WISP2	205792_at	2.365469	2.353653	2.30536	2.338699	2.837873	2.34623	2.34912	2.354751	2.340296	2.335164	2.337391	2.347328	2.240665
TNK1	205793_x_at	2.377022	2.341386	2.321577	2.331772	2.349879	2.332068	2.338637	2.346344	2.331437	2.328262	2.324254	2.34098	2.215164
NOVA1	205794_s_at	2.47765	2.471562	2.442935	2.455489	2.464076	2.422451	2.607218	2.466672	2.463223	2.498172	3.90511	2.69908	7.674917
NRXN3	205795_at	2.171043	2.160979	2.137732	2.147571	2.160027	2.141998	2.160704	2.160925	2.152134	2.141194	2.145168	2.159169	3.276479
TCP11L1	205796_at	2.483523	2.597452	2.506666	2.496417	2.525872	2.480762	2.520962	2.797496	2.51163	2.617582	2.779859	2.822419	2.487406
TCP11L1	205797_s_at	3.760812	3.735047	3.734481	3.739139	3.574327	4.202398	3.750213	3.748795	3.735176	3.696905	3.732976	3.743663	3.704943
IL7R	205798_at	2.988993	2.811554	3.822623	3.059319	3.024065	4.971193	1.964735	2.955583	1.956195	1.952133	2.816562	1.999792	1.915445
SLC3A1	205799_s_at	10.73595	10.74631	9.82523	10.16899	9.554957	9.254721	7.331669	8.358777	8.85618	9.053941	6.90169	6.62999	3.560404
SLC3A1	205800_at	2.703472	2.680538	2.649737	2.650176	3.122237	2.660326	2.666287	2.673229	2.662278	2.647849	2.663646	2.680244	2.59107
RASGRP3	205801_s_at	5.436866	5.168284	5.336411	5.578286	4.825484	4.871254	5.752652	5.265264	5.746531	5.656603	5.450738	5.599424	5.207477
TRPC1	205802_at	3.81875	3.766207	3.744086	3.858437	4.449798	4.103549	5.409194	5.849789	5.31645	5.565765	6.102192	5.429272	7.499959
TRPC1	205803_s_at	2.858129	2.67337	2.785874	2.809701	2.78945	2.706015	4.976893	3.568037	4.619351	5.126734	3.563939	3.130753	7.132882
TRAF3IP3	205804_s_at	3.612532	3.952944	3.500084	3.602556	3.604295	4.1322	3.041222	3.561899	3.093954	3.022586	2.745836	3.591137	3.00213
ROR1	205805_s_at	2.32101	2.27364	2.243862	2.263795	2.273128	2.259038	2.272405	2.784811	2.268621	2.338396	3.356916	2.278276	6.615655
ROM1	205806_at	2.504406	2.485583	2.448974	2.462462	2.862384	2.464129	2.475923	2.481504	2.466012	2.458606	2.470938	2.477271	2.382763
TUFT1	205807_s_at	5.955227	5.562256	5.747976	6.024	6.381014	6.692855	6.361284	6.73362	6.299031	6.24215	6.320142	6.542203	6.35629
ASPH	205808_at	2.338149	2.322473	2.302389	2.312395	2.32196	2.305762	2.320335	2.318854	2.30862	2.301328	2.301908	2.31601	3.012474

WASL	205809_s_at	8.699958	7.984427	8.88482	9.10814	9.404516	9.443824	7.673539	8.10612	7.075687	6.523129	8.536444	8.578879	6.725168
WASL	205810_s_at	2.890147	2.885372	2.864401	2.856841	2.874566	2.79819	2.80595	2.802871	2.864125	3.153152	2.879513	2.859478	3.936145
POLG2	205811_at	5.258744	5.360959	5.151145	5.470311	5.055576	5.073048	5.775886	5.905697	6.257271	5.831363	5.487386	5.858519	6.418163
TMED9	205812_s_at	10.6029	10.32359	9.844346	9.96936	10.5011	10.53782	10.31226	10.74211	10.24403	10.4026	11.3722	11.02149	9.879381
MAT1A	205813_s_at	12.3664	12.36109	12.76588	12.93451	12.75692	12.52893	9.298506	8.612972	8.858929	8.69033	7.746484	7.7335	3.639114
GRM3	205814_at	2.35828	2.338429	2.312082	2.31891	2.361923	2.318304	2.325305	2.337102	2.322631	2.310581	2.319475	2.332196	4.637779
REG3A	205815_at	2.309245	2.299284	2.281968	2.286324	2.2982	2.287647	2.287316	2.29915	2.2895	2.285066	2.284703	2.290738	2.245429
ITGB8	205816_at	2.729497	2.684403	2.62213	2.660982	2.717128	2.662736	2.674731	2.683708	2.670161	2.662277	2.66916	2.697829	2.600057
SIX1	205817_at	2.458533	2.433528	2.392485	2.412504	2.435953	2.399367	2.420617	2.430926	2.417488	2.403664	2.417157	2.426866	5.092777
DBC1	205818_at	2.211923	2.189176	2.171516	2.389769	2.190795	2.863736	2.152464	2.187791	2.146142	2.125248	2.177829	2.18236	4.310239
MARCO	205819_at	6.127738	5.995534	7.589005	8.170354	8.823077	8.66532	4.496522	6.603839	5.964062	4.638598	3.200378	4.945947	2.100429
APOC3	205820_s_at	14.79843	14.84908	14.61963	14.83534	14.92437	14.78833	14.21771	13.9427	14.20326	13.96724	13.63866	13.6965	8.230539
KLRK1	205821_at	5.479093	5.292774	4.321236	4.357712	4.416075	5.876431	2.774873	2.711594	2.763924	2.095965	2.695177	3.128765	2.827196
HMGCS1	205822_s_at	3.638461	3.512053	4.361033	3.853482	5.582167	4.657732	8.555501	6.787024	8.521099	8.163133	5.9124	6.438931	5.378819
RGS12	205823_at	4.973051	4.891342	4.870674	4.874867	6.069446	6.52591	3.528679	4.887491	3.525692	4.078608	5.270762	5.007476	3.00646
HSPB2	205824_at	2.622732	2.588261	2.567452	2.615174	2.785639	3.180115	2.600474	2.601869	2.56178	2.545891	6.66887	2.59681	7.450884
PCSK1	205825_at	3.047101	2.938378	2.844077	2.940358	3.006332	2.904945	2.922445	2.951587	2.941839	2.936746	2.944287	2.962354	6.808241
MYOM2	205826_at	4.385303	4.38085	4.647756	5.344042	4.375494	4.366373	4.358512	4.343115	3.844059	3.83496	6.614381	4.359337	6.750894
CCK	205827_at	2.745813	2.716891	2.676295	2.696107	2.716723	2.692796	2.703466	2.713075	2.753806	2.691277	2.696522	2.71072	4.432506
MMP3	205828_at	2.338356	2.315295	2.281483	2.299081	2.320481	2.289729	2.312913	2.332674	2.304685	2.291653	2.300549	2.31869	2.388106
HSD17B1	205829_at	2.454075	2.486344	2.40597	2.411918	2.818182	2.629561	2.399929	2.423929	2.41416	2.360719	2.413602	2.418275	2.327744
CLGN	205830_at	4.842763	4.689745	3.377889	3.748321	4.841094	4.160112	7.085519	7.228862	6.920682	6.697936	6.951748	7.67713	5.358727
CD2	205831_at	3.495373	3.105668	2.967533	3.113711	4.041183	5.938068	2.477229	2.682207	2.467026	2.560327	2.466712	2.489883	2.383034
CPA4	205832_at	2.168101	2.159023	2.137011	2.142217	2.161862	2.141058	2.155322	2.158902	2.145192	2.141061	2.145801	2.15367	2.08086
PART1	205833_s_at	2.119785	2.105167	2.077779	2.08914	2.123173	2.088702	2.100081	2.10231	2.090462	2.087647	2.086134	2.097157	2.078736
PART1	205834_s_at	2.352065	2.326794	2.298824	2.302693	2.329716	2.315273	2.315997	2.322697	2.309921	2.295761	2.308149	2.319991	2.233339
YTHDC2	205835_s_at	2.909205	2.834854	2.817072	3.000943	2.898666	2.86364	2.875466	2.857541	3.304243	3.5213	2.850426	2.876852	3.087814
YTHDC2	205836_s_at	4.430328	4.847451	5.366336	4.761034	4.593198	4.682612	5.180476	5.093013	5.675274	5.51277	4.678319	4.890124	5.479289
GYPA	205837_s_at	3.32217	3.146497	3.044735	3.19419	3.306832	3.100796	12.09281	10.83135	12.44468	12.36189	10.4219	11.07871	5.763698
GYPA	205838_at	2.210547	2.193289	2.161702	2.173904	2.195444	2.169628	11.28783	10.74905	11.61388	11.37917	9.749438	10.15715	3.472948
BZRAP1	205839_s_at	5.409916	5.400258	4.48362	4.465669	4.543954	5.167078	4.138448	4.458677	3.787074	4.062663	3.679916	4.452035	5.07944
GH1	205840_x_at	2.199374	2.168941	2.142177	2.152855	2.172883	2.146102	2.159652	2.16503	5.235697	2.149246	2.153559	2.15902	2.243853
JAK2	205841_at	3.635805	3.54463	3.505779	3.559776	3.623604	3.186399	5.613179	4.673094	6.306676	5.331573	4.472976	4.388782	4.983914
JAK2	205842_s_at	3.463785	3.060041	3.949614	3.871641	3.8152	3.4579	7.008868	4.915321	7.214664	6.776708	5.124148	5.466076	5.802444
CRAT	205843_x_at	7.06607	6.820969	6.734417	6.755714	7.231476	7.610823	5.482708	6.103723	5.332313	5.628886	5.936486	5.987905	3.857043
VNN1	205844_at	11.2523	11.30615	11.9259	11.6268	11.17337	10.80284	3.433869	3.426218	3.020105	2.741764	3.079776	3.442633	4.680203
CACNA1H	205845_at	2.604508	2.593713	3.40139	3.298061	2.597303	2.589267	2.591092	2.576987	2.587735	2.576368	2.581385	2.583514	2.588109
PTPRB	205846_at	3.095539	3.219932	3.797915	3.62994	2.852699	2.800798	2.64722	2.578154	2.561959	2.553064	2.621374	2.315689	2.88453

PRSS22	205847_at	3.922313	3.905523	3.662323	3.868919	3.90741	3.855299	3.890221	3.906155	3.887171	3.891955	3.888111	4.146132	3.415273
GAS2	205848_at	10.64137	10.53363	10.6287	10.49963	10.13889	9.526694	10.75463	10.27799	11.34174	10.95801	10.38867	10.11895	11.81849
UQCRB	205849_s_at	13.05463	12.84241	12.87916	13.16313	12.95607	12.88285	12.72966	12.86085	12.65217	12.72122	12.83577	12.82975	12.79954
GABRB3	205850_s_at	2.646776	2.601289	2.571538	2.598323	2.643234	2.583107	2.607174	2.612921	2.599827	2.585152	2.59686	2.62098	3.656559
NME6	205851_at	5.437142	5.348837	5.161717	5.050402	5.015112	4.836567	5.022234	5.429186	5.635275	5.377488	5.663588	5.624084	4.93393
CDK5R2	205852_at	2.840034	2.919033	2.743016	2.780972	2.877755	2.777401	2.803824	2.813685	2.789932	2.744664	2.797934	2.808928	3.482019
ZBTB7B	205853_at	2.52225	2.503926	2.448546	2.484935	2.509337	2.492652	2.504697	2.672352	2.502267	2.496844	2.487558	2.707853	2.401071
TULP3	205854_at	6.189724	6.484976	6.380576	6.226423	6.71661	6.891845	6.971312	6.753961	6.66228	6.406574	7.357507	6.455873	8.33025
ZNF197	205855_at	3.425456	3.415292	2.727099	3.068741	3.400913	3.349546	3.914753	4.502827	3.389313	3.44183	3.858497	3.644763	4.567602
SLC14A1	205856_at	3.185716	3.048274	3.28903	3.046184	3.197277	3.029749	7.609524	7.914583	9.216325	8.784977	7.150085	7.661967	2.808228
SLC18A2	205857_at	2.650233	2.624478	2.582589	2.642523	2.850359	2.601558	2.615718	2.622528	2.606339	2.59466	2.60714	2.646826	2.495843
NGFR	205858_at	5.025779	5.763491	6.036969	5.106719	5.668794	5.006561	5.17177	4.319089	4.605024	5.007782	5.124961	4.91388	6.087702
LY86	205859_at	5.084976	5.647604	5.630683	5.998379	5.48065	6.05732	5.706326	5.406562	5.169466	4.587915	4.965528	6.026073	4.416903
FOLH1	205860_x_at	8.376818	8.293784	9.207528	9.350801	8.271221	8.139072	7.415217	7.317168	6.747557	6.922901	6.403899	7.072798	3.849375
SPIB	205861_at	2.487132	2.450292	2.419097	2.432256	2.451263	2.437494	2.437633	2.447481	2.434585	2.417832	2.434746	2.449966	2.29409
GREB1	205862_at	3.229207	3.800211	4.056858	4.197642	4.324367	3.904424	5.928106	5.837067	6.179296	6.04524	6.006478	6.116701	5.678657
S100A12	205863_at	3.281795	3.198971	4.172173	4.1355	3.907485	3.180834	3.497595	3.846081	3.25067	4.150141	4.914663	5.80759	2.787903
SLC7A4	205864_at	1.915974	1.904807	1.885869	1.889556	1.929916	1.88889	1.901439	1.902415	1.897547	1.888817	1.887867	1.897506	1.849666
ARID3A	205865_at	2.864341	2.821865	2.756614	2.809023	2.821524	2.841222	4.620837	4.347431	4.826853	4.592069	3.787312	4.575149	2.842729
FCN3	205866_at	11.26627	10.96174	12.17352	12.16711	9.623796	9.145782	8.657335	8.98086	9.23638	8.813332	7.983969	8.923286	3.682604
PTPN11	205867_at	2.729308	2.683153	2.623233	2.67441	2.823549	2.674376	2.696831	2.69685	2.683232	2.668628	2.683032	2.741888	2.59609
PTPN11	205868_s_at	2.673689	2.654548	2.588805	2.632261	2.675697	2.633422	2.643879	2.653077	2.632885	2.63205	2.626108	2.679797	3.530629
PRSS1	205869_at	3.210263	3.183377	3.056812	3.152064	3.231738	3.166857	3.089493	3.183864	3.171041	3.165841	3.167247	3.180535	2.843477
BDKRB2	205870_at	2.424212	2.413273	2.389905	2.400526	2.414818	2.394007	2.405958	2.407655	2.395571	2.389594	2.395501	2.402795	2.397084
PLGLB1	205871_at	13.77505	13.79271	13.75274	13.89971	13.97961	14.00473	10.30749	11.31893	11.80809	11.43945	10.51378	10.86828	5.254039
PDE4DIP	205872_x_at	3.282108	3.815861	4.51766	4.622559	5.656627	5.197104	3.081443	3.948359	3.497614	3.063657	5.465496	3.521304	8.58126
PIGL	205873_at	1.91526	1.904674	2.129057	2.133506	2.138407	2.135309	2.14485	2.187176	2.141363	2.176479	2.127387	2.149242	2.127637
ITPKA	205874_at	2.351753	2.329622	2.314467	2.318376	2.345665	2.318082	2.32469	2.330515	2.320619	2.303178	2.312434	2.328625	2.232863
TREX1	205875_s_at	4.195634	4.481698	5.201452	5.11982	6.18373	5.552274	4.953413	4.947428	4.946066	5.087776	4.970575	5.444979	4.292447
LIFR	205876_at	4.386489	3.762692	4.568494	4.387698	5.043816	5.14622	3.346037	3.212167	3.714136	3.022288	3.209727	3.29006	3.978847
ZC3H7B	205877_s_at	3.861615	4.091436	3.82832	3.653412	3.850152	3.841282	3.852068	4.246713	3.844883	4.051343	3.836097	4.447429	3.809804
POU6F1	205878_at	3.705444	3.54469	4.110088	3.970249	4.245864	4.905583	3.188298	3.69676	3.291095	2.849402	3.73709	3.540324	3.724972
RET	205879_x_at	2.418525	2.384455	2.359518	2.371073	2.392064	2.628945	2.380908	2.480751	2.37767	2.367852	2.382789	2.380997	2.479701
PRKD1	205880_at	3.115787	3.094233	3.06551	3.103621	2.89089	3.071816	3.104287	3.089298	3.075902	3.825423	5.952013	3.029186	7.068146
ZNF74	205881_at	3.216426	2.802198	2.634714	2.789665	2.826821	2.788237	3.007263	2.799893	2.800415	2.787968	4.092712	2.794505	4.641598
ADD3	205882_x_at	6.944468	6.945851	6.46101	6.470375	6.575112	6.387133	9.192111	8.263201	8.958819	8.566879	8.579381	8.604701	10.12672
ZBTB16	205883_at	6.318888	6.38409	8.429794	8.37458	7.229465	6.74628	4.742223	5.506834	4.882125	4.638785	5.076566	5.092903	3.947394
ITGA4	205884_at	3.199622	3.117898	3.015063	3.177842	3.117523	2.972411	6.924725	3.676349	7.010229	7.244231	3.122641	3.673127	5.904203

ITGA4	205885_s_at	2.820771	2.754576	2.733976	2.73771	2.812743	2.736262	7.289115	6.441554	7.860913	7.360403	5.479685	5.814335	4.957982
REG1B	205886_at	2.001678	1.975881	1.952347	1.956477	1.97546	1.961131	1.970295	1.97209	1.960304	1.954374	1.955868	1.98842	1.909492
MSH3	205887_x_at	8.245365	8.225194	8.008055	7.990225	8.165627	7.881871	7.873709	8.048606	7.713766	7.81517	8.156888	8.070337	6.949614
JAKMIP2	205888_s_at	3.875417	4.162543	5.668249	5.045037	4.087457	4.033169	4.034479	3.563847	3.419005	3.770452	3.509438	3.799178	5.13587
JAKMIP2	205889_s_at	2.311843	2.275324	2.251204	2.255465	2.315399	2.229954	2.25936	2.250724	2.252288	2.228731	2.279062	2.289024	5.853521
UBD	205890_s_at	9.105021	9.069619	6.908351	6.801689	6.242913	7.761473	4.979438	6.683842	3.854179	5.082785	4.073896	6.167349	2.843735
ADORA2B	205891_at	1.795402	1.786868	1.774476	1.773626	1.790915	1.771075	2.619461	1.878495	3.633309	2.969337	2.028711	1.808163	2.251665
FABP1	205892_s_at	13.82847	13.75256	13.76641	13.62374	14.26319	14.07182	14.01121	13.93688	14.20233	14.15704	13.74402	13.94904	8.332873
NLGN1	205893_at	2.798035	2.740814	2.681574	2.725131	2.821547	2.705038	2.742942	2.755344	2.739932	2.716741	2.818469	2.762269	6.728847
ARSE	205894_at	9.775204	9.900446	9.820014	10.0346	10.804	10.70618	9.309372	9.777999	9.914276	9.60902	10.39315	9.923257	3.38976
NOLC1	205895_s_at	4.479291	4.174626	4.684818	4.847645	4.174099	3.791856	6.719405	5.440463	7.212414	7.293771	5.631688	5.757071	6.935028
SLC22A4	205896_at	2.361302	2.338603	2.30881	2.331885	2.343696	2.31423	8.603055	7.333063	8.555645	8.669013	7.683751	8.096467	3.628232
NFATC4	205897_at	2.127715	2.116373	2.035392	2.10036	2.122384	2.100136	2.109058	2.208999	2.102371	2.095414	2.036704	2.112727	2.069035
CX3CR1	205898_at	5.209477	5.301098	4.81774	4.906085	6.380957	5.950547	4.363632	3.992812	3.155968	2.928891	4.662305	4.998732	5.449318
CCNA1	205899_at	2.208696	2.184923	2.154462	2.165973	2.199797	2.147398	2.173471	2.184173	2.169383	2.161186	2.169257	2.178027	2.47692
KRT1	205900_at	2.136202	2.122865	2.096879	2.102714	2.127163	2.105982	5.204562	6.130273	5.267961	4.914863	5.433238	5.431019	4.506419
PNOC	205901_at	2.197529	2.181579	2.157747	2.162313	2.181079	2.160136	2.171227	2.178512	2.16521	2.156841	2.166172	2.174988	2.989244
KCNN3	205902_at	2.402466	2.383652	2.362395	2.367191	2.41674	2.370658	2.377151	2.381987	2.368416	2.361711	2.54772	2.378191	3.200694
KCNN3	205903_s_at	2.563646	2.539685	2.471465	2.517877	2.586548	2.819521	2.53225	2.539412	2.526314	2.518464	2.526289	2.537641	3.121022
MICA	205904_at	6.388375	6.477739	5.513953	5.646018	7.124689	7.026826	5.269798	5.902204	5.703308	3.916471	3.622496	5.388265	4.624679
MICA	205905_s_at	5.315889	5.809079	4.929859	5.141169	6.542512	5.902969	4.833725	4.759814	5.191075	4.199507	4.524683	5.13894	4.056077
FOXJ1	205906_at	2.973052	2.952981	2.90351	2.937034	2.977684	3.098662	3.134632	3.900591	3.152499	3.011948	3.324978	2.96635	2.795491
OMD	205907_s_at	2.755414	2.70228	2.690467	2.687679	2.744917	2.701634	2.675671	2.708147	2.671553	2.66693	2.696171	2.703515	6.067092
OMD	205908_s_at	2.652698	2.600171	2.569193	2.594662	2.640312	2.605025	2.588545	2.615342	2.591571	2.573665	2.597876	2.611099	6.015596
POLE2	205909_at	2.824114	2.614973	3.945112	3.439625	2.607963	2.535237	7.155197	6.379119	7.346829	7.229625	5.888109	6.198175	5.935835
CEL	205910_s_at	3.190593	2.77544	2.714807	2.765991	2.84656	2.77956	2.764938	2.802507	2.757898	2.828372	3.353323	2.817334	3.210104
PTHR1	205911_at	7.932344	7.906147	8.144201	8.252806	8.750743	8.832376	5.854199	5.987749	5.276176	5.848807	7.040438	6.514443	7.111023
PNLIP	205912_at	2.599682	2.565392	2.51014	2.548125	2.581409	2.550419	2.561764	2.569671	2.557469	2.549496	2.559058	2.580667	2.441022
PLIN	205913_at	2.680985	2.673386	4.161159	3.651592	2.671086	2.679533	2.687696	2.668141	2.682551	2.608966	2.639092	2.623947	1.850463
GRIN1	205914_s_at	2.313224	2.282878	2.315952	2.269604	2.304202	2.326758	2.321951	2.28879	2.272884	2.264043	2.274389	2.32751	2.18249
GRIN1	205915_x_at	2.234582	2.20795	2.187956	2.365539	2.207241	2.190375	2.196953	2.204149	2.1935	2.182108	2.188057	2.198982	2.124996
S100A7	205916_at	2.588036	2.565626	2.504428	2.53945	2.617144	2.518156	2.555744	2.560496	2.548745	2.528221	2.550506	2.557557	2.530647
ZNF264	205917_at	7.199661	6.848554	6.774411	6.781122	6.881179	6.695235	6.820989	6.7519	7.329745	6.590657	6.703312	6.630078	8.013514
SLC4A3	205918_at	2.468078	2.440187	2.423556	2.42722	2.443533	2.429079	2.438282	2.440728	2.428728	2.423007	2.533282	2.43444	4.198399
HBE1	205919_at	2.777645	2.660739	2.665335	2.641101	2.774754	2.867043	13.22589	12.13832	11.64823	11.87067	12.64515	13.3521	13.32508
SLC6A6	205920_at	2.174645	2.158759	2.13545	2.140535	2.161415	2.140017	2.151711	2.156521	2.143796	2.136373	2.142269	2.151471	2.079719
SLC6A6	205921_s_at	2.668265	2.62893	2.563399	2.610716	2.664614	2.582495	2.618344	2.630446	2.621331	2.609044	2.614047	2.632075	2.497556
VNN2	205922_at	4.18456	4.234758	4.350329	4.2835	4.301883	4.409149	4.420922	4.604957	4.411283	4.903128	4.281856	4.666571	2.787194

RELN	205923_at	10.03288	9.782083	10.05009	9.951878	9.301361	9.068598	11.36068	10.5018	11.3003	10.9355	10.86907	10.77633	8.924156
RAB3B	205924_at	2.37057	2.345997	2.310958	2.329382	2.399233	2.327597	2.356594	2.357713	2.702084	4.183601	2.338935	2.373168	3.818024
RAB3B	205925_s_at	2.134461	2.116626	2.091637	2.0968	2.117425	2.093952	2.107876	2.114544	2.103395	2.100031	2.098327	2.108268	2.814767
IL27RA	205926_at	3.927042	4.006481	3.619807	3.591908	4.059333	3.985091	4.011526	4.021171	3.978058	3.990405	4.042399	4.696356	3.81482
CTSE	205927_s_at	2.730939	2.721607	2.702828	2.709538	2.719429	2.763818	7.452938	8.070555	8.230549	7.705608	6.755221	7.495575	2.991416
ZNF443	205928_at	5.681343	5.680939	5.309305	5.324252	5.906775	5.269921	5.71163	5.640504	5.038533	5.525638	5.400495	5.336891	6.138976
GPA33	205929_at	1.849102	1.836545	1.817302	1.82157	1.835861	1.822236	1.832949	1.834453	1.827888	1.818367	1.815441	1.827692	1.7852
GTF2E1	205930_at	5.780152	5.443187	5.753643	5.81568	5.239507	5.593448	6.499228	6.341137	6.665079	6.795658	6.344842	6.650784	6.88784
CREB5	205931_s_at	2.958041	2.92548	3.281833	2.971907	2.915798	3.088417	2.822779	2.830324	2.827969	2.801272	3.133833	2.879357	6.637777
MSX1	205932_s_at	2.787776	2.776903	2.730668	2.714763	2.786684	2.767712	2.772439	2.778652	2.761901	2.707456	2.763886	2.771735	6.682859
SETBP1	205933_at	5.859018	5.84186	5.724432	5.753206	5.439797	4.845415	3.19763	3.538829	3.081214	4.351878	5.138781	4.156314	8.082402
PLCL1	205934_at	2.830107	2.77269	2.68944	2.772975	2.826959	2.771491	2.750811	2.785916	2.77289	2.752042	2.772373	2.796248	6.711926
FOXF1	205935_at	3.595989	3.558408	3.570887	3.567483	3.584085	3.577211	4.02169	3.587052	3.371901	3.527001	3.5715	3.597488	3.644607
HK3	205936_s_at	3.794975	3.634412	3.543309	3.596658	4.483405	4.866674	2.983791	3.632714	3.57572	2.856023	3.067675	3.389405	2.599802
CGREF1	205937_at	2.762844	3.507097	2.659856	2.727852	2.751395	2.727217	2.712053	2.743314	2.709178	2.68869	2.724798	2.736674	2.700753
PPM1E	205938_at	2.315362	2.293942	2.269327	2.277936	2.318925	2.281309	2.286424	2.292723	2.280054	2.273251	2.275024	2.286839	3.654171
CYP3A7	205939_at	12.71875	12.63201	9.508472	9.290839	9.283875	8.43899	13.02995	12.53516	13.2967	13.07217	11.42033	11.95472	7.679317
MYH3	205940_at	3.510108	4.151152	3.101474	3.263743	4.146889	4.144127	3.086306	3.332217	2.915162	3.058526	12.59724	3.115451	13.0376
COL10A1	205941_s_at	2.380287	2.348729	2.324007	2.330837	2.350856	2.328985	2.343269	2.331204	2.316237	2.318177	2.327861	2.342363	2.386593
ACSM3	205942_s_at	5.548992	5.2553	7.944323	7.042768	5.37385	4.520236	5.617216	2.830156	6.134938	5.206625	2.769582	3.54964	1.718375
TDO2	205943_at	12.85556	12.78917	13.71428	13.71163	12.99286	12.3441	11.93475	10.01713	11.49296	11.00413	9.018818	9.373392	4.994555
CLTCL1	205944_s_at	3.467485	3.792763	3.068701	3.094709	3.216054	3.080053	2.757309	3.088505	2.76699	2.734633	3.118461	3.199663	2.946069
IL6R	205945_at	11.73347	11.65806	11.86853	11.90255	11.77523	11.67199	8.553932	9.173733	8.501256	8.404736	8.82103	9.199708	4.462457
VIPR2	205946_at	2.286627	2.263222	2.23366	2.25092	2.262195	2.241069	2.266375	2.261647	2.254992	2.248867	2.249397	2.255651	2.167326
VIPR2	205947_s_at	2.144582	2.129936	2.109766	2.114486	2.13776	2.114826	2.125405	2.130493	2.116345	2.114042	2.115766	2.12427	2.06358
PTPRT	205948_at	2.314659	2.308306	2.28827	2.292807	2.664693	2.292718	2.292755	2.294424	2.280944	2.277984	2.278302	2.298025	2.264265
CA1	205949_at	2.175889	2.15454	2.123488	2.138367	2.155754	2.136235	2.150302	2.152907	2.140436	2.133744	2.135663	2.147769	2.053662
CA1	205950_s_at	3.54484	3.378337	3.33914	3.145016	3.647276	3.75369	7.195119	6.531207	5.804129	7.136362	5.706183	5.190035	3.039219
MYH1	205951_at	3.247382	3.138016	3.155675	3.169765	3.238416	3.090299	3.180157	3.180901	3.172131	3.120968	3.187553	3.181185	2.851807
KCNK3	205952_at	2.596333	2.556519	2.532306	2.541844	2.557434	2.74646	2.541003	2.550681	2.527089	2.517044	2.532461	2.545752	4.68162
LRIG2	205953_at	2.429737	2.515397	2.263548	2.482849	2.504879	2.45767	3.225035	2.849558	3.193787	3.000272	2.740972	2.764594	4.14186
RXRG	205954_at	2.125419	2.115165	2.08472	2.100851	2.11931	2.092833	2.113002	2.114699	2.103787	2.095639	3.723734	2.114005	3.244565
PSMC3IP	205956_x_at	4.961784	5.056439	4.466349	4.78516	4.853512	5.180531	5.432463	5.47887	4.840258	4.911839	4.843343	4.854264	4.843831
PLXNB3	205957_at	2.695497	2.675889	2.660118	2.652203	2.685905	2.637522	2.857578	2.84474	2.652395	3.036006	2.666625	2.722241	2.429155
CSHL1	205958_x_at	2.452962	2.439182	2.3862	2.423391	2.439971	2.42552	2.434594	2.437621	9.209	5.381004	2.427313	2.434248	6.523116
MMP13	205959_at	2.49484	2.456395	2.438373	2.44479	2.479431	2.446711	2.44655	2.459008	2.446667	2.431387	2.443786	2.457844	2.392855
PDK4	205960_at	2.537311	2.463613	7.221425	5.294505	2.455386	2.406948	2.416002	2.451759	2.426861	2.403038	2.410521	2.442967	2.345333
PSIP1	205961_s_at	5.496958	5.997117	6.258661	5.553024	3.773476	3.401346	8.79563	6.78237	9.004149	8.830665	6.589485	6.88513	9.613982

PAK2	205962_at	2.862049	2.784721	2.68695	2.764087	2.811051	2.706341	2.772488	2.777541	2.769279	2.751581	2.766577	2.782015	2.514972
DNAJA3	205963_s_at	8.257687	8.118828	8.632758	8.172599	7.705134	7.386753	8.015447	7.889645	8.182637	8.21353	7.806704	7.821361	6.533177
ZNF426	205964_at	5.470716	4.744262	4.428517	4.580358	4.999565	4.604173	4.866203	5.079426	5.297779	5.315996	5.702717	5.460381	5.989072
BATF	205965_at	4.095014	4.085549	3.928924	4.068219	4.083906	4.070817	4.070042	4.081485	4.066613	4.111254	4.063014	4.064133	3.934284
TAF13	205966_at	3.457499	3.345883	3.164572	3.304649	3.511468	3.298907	3.342272	3.275803	3.34629	3.293386	3.454348	3.40204	3.099797
HIST1H4C	205967_at	8.653222	8.505547	8.453278	8.471609	8.447121	8.545142	10.70178	11.117	10.44285	10.68133	11.19898	11.36136	9.174181
KCNS3	205968_at	3.544144	3.819497	4.939309	4.756176	4.588869	4.10225	2.718386	2.641012	2.532568	2.489228	3.762616	3.272867	3.803001
AADAC	205969_at	10.80508	10.69363	11.84764	11.62545	10.50532	9.917936	9.499906	8.397305	8.464832	8.962853	6.544135	7.473278	4.072869
MT3	205970_at	3.879787	4.313502	4.001842	4.247374	4.272191	4.123071	4.868338	4.640532	4.446146	5.331576	4.307794	4.303883	4.250979
CTRB1	205971_s_at	3.71285	3.548155	3.519037	3.538268	3.571142	3.525625	3.546454	3.539497	3.534415	3.574182	3.533808	3.544548	2.885869
SLC38A3	205972_at	9.721418	9.949315	9.509241	9.647657	9.104512	8.666219	9.146225	8.353058	9.092666	9.035821	8.116715	9.104586	3.082442
FEZ1	205973_at	3.562794	3.320231	3.36537	3.440449	3.468058	3.411933	3.44415	3.942141	3.443026	3.420738	3.43733	3.459556	3.273061
HOXD1	205974_at	2.528224	2.487437	2.441158	2.473632	2.551737	2.460019	2.483828	2.494212	2.485671	2.463123	2.478206	2.492229	2.302877
HOXD1	205975_s_at	2.362053	2.342142	2.312816	2.319001	2.342669	2.328937	2.329663	2.335548	2.323548	2.312127	2.324098	2.333152	2.983265
FASTKD2	205976_at	3.522294	3.404372	4.415127	4.217094	3.623887	3.835153	3.336268	3.449097	3.610538	3.308677	3.227835	3.1322	4.112436
EPHA1	205977_s_at	6.288745	6.260726	6.110014	6.138677	5.16699	5.027296	6.17177	5.796945	5.846349	5.840876	5.774511	5.872156	3.00086
KL	205978_at	3.594337	3.874136	3.117589	3.20364	3.261439	3.212563	3.229875	3.239278	3.211541	3.206293	3.294853	3.241368	3.260113
SCGB2A1	205979_at	2.684605	2.641448	2.621587	2.635026	2.741398	2.625511	2.646811	2.655164	2.641239	2.629391	2.641903	2.660616	2.53478
ARHGAP8	205980_s_at	2.577991	2.569107	2.549295	2.541431	2.568164	2.510103	2.567719	2.891223	2.556165	2.522489	2.811988	2.56085	2.526273
ING2	205981_s_at	6.964999	6.59315	7.461545	7.407808	6.946675	6.715388	8.209753	8.04915	8.28675	8.184616	8.338818	8.285229	7.954829
SFTPC	205982_x_at	1.876024	1.869769	1.849713	1.853025	1.879394	1.851836	1.861933	1.865788	1.854443	1.849083	1.84657	1.858804	1.806977
DPEP1	205983_at	2.075381	2.055706	2.031899	2.036478	2.058402	2.037436	2.047285	2.055213	2.03932	2.031515	2.039115	2.062102	1.997079
CRHBP	205984_at	7.105241	6.822093	9.008679	8.565407	5.920525	4.36054	8.424458	5.394992	7.946758	7.277064	5.477281	5.343627	2.374601
CLCNKB	205985_x_at	1.888756	1.879407	1.863561	1.864892	1.878104	1.870323	1.876317	1.879518	1.873048	1.867177	1.860516	1.871062	1.82764
AATK	205986_at	5.211268	5.258334	4.392089	4.235185	5.040234	5.021028	5.534937	5.681297	5.073902	5.662736	5.75705	5.898029	5.529723
CD1C	205987_at	3.494017	3.47365	2.743453	2.791506	2.885775	4.36559	2.686989	2.669278	2.630861	2.656054	2.439089	2.57538	2.378391
CD84	205988_at	5.423942	5.328108	5.58455	5.757621	5.599085	5.451886	5.704454	6.171131	5.362161	5.215056	4.907759	6.407363	3.451241
MOG	205989_s_at	2.989227	2.908649	2.816734	2.88508	2.981575	2.836756	2.895389	2.905393	2.88286	2.844784	2.899479	2.969822	2.623319
WNT5A	205990_s_at	5.981133	5.910626	5.463892	4.947738	4.861029	4.004601	4.384224	3.457182	3.364841	4.809526	5.309016	4.373841	10.63579
PRRX1	205991_s_at	2.293018	2.266627	2.252094	2.253184	2.293848	2.256097	2.257447	2.26803	2.254975	2.248873	2.252543	2.261244	8.124764
IL15	205992_s_at	4.470126	4.4726	4.357671	4.401608	4.843921	4.478846	3.55923	3.050367	3.821845	3.70817	3.777816	3.131816	4.05888
TBX2	205993_s_at	2.725368	2.802117	2.647946	2.690936	2.947213	2.873578	2.516089	2.712883	2.70023	2.676312	2.721629	2.730139	3.719875
ELK4	205994_at	3.219178	3.237842	3.160238	3.112221	3.138568	3.107615	3.08468	3.091275	3.041757	3.058085	3.108181	3.12122	2.762166
IQCB1	205995_x_at	4.008573	4.002465	3.98362	3.979595	3.168218	3.302605	5.307047	4.07299	4.293879	4.786302	3.979904	3.90264	5.685928
AK2	205996_s_at	5.447929	5.158949	6.504796	5.819276	4.745078	3.925592	6.411964	4.974336	6.496293	6.788003	4.588812	4.887754	6.447184
ADAM28	205997_at	2.713822	2.659244	2.616573	2.646097	3.215254	2.637761	2.639781	2.665472	2.64398	2.623245	2.634634	2.665723	2.822147
CYP3A4	205998_x_at	12.73562	12.67923	13.21994	13.33142	13.16533	12.92475	8.39368	9.561328	8.475736	8.604473	9.266125	8.920379	4.563282
CYP3A4	205999_x_at	13.73189	13.63597	14.2559	14.14473	14.06126	13.46254	8.801815	8.211181	9.308744	9.615079	6.821659	7.947417	4.879968

MEP1A	206000_at	2.237045	2.216643	2.212993	2.204762	2.223944	2.215817	2.966499	5.371744	5.517753	4.566417	3.220722	4.332468	2.177361
NPY	206001_at	2.199817	2.177791	2.154699	2.158185	2.177443	2.158553	2.166509	2.175114	2.161587	2.154044	2.867104	2.169148	4.235884
GPR64	206002_at	2.288286	2.255058	2.551806	2.709557	2.260159	2.2457	2.242476	2.25659	2.24033	2.226216	2.238426	2.244887	7.35683
CEP135	206003_at	4.962987	4.859598	4.925398	4.86661	4.947261	4.415999	7.173636	6.912737	7.473834	7.864425	6.665062	6.898963	6.53957
TGM3	206004_at	2.372168	2.335555	2.315751	2.321218	2.340198	2.536886	2.327072	2.334779	2.3228	2.306246	2.319049	2.329929	2.203692
KIAA1009	206005_s_at	3.939646	3.930068	3.820178	3.912542	4.068555	4.377848	4.63116	4.743421	4.691031	4.55108	4.729125	4.229665	4.68132
KIAA1009	206006_s_at	2.726839	2.68507	2.659022	2.705708	2.715658	2.821653	3.907914	3.201667	2.70018	3.296628	2.696695	2.714416	4.421975
PRG4	206007_at	7.640006	7.254303	8.619739	8.696178	6.764747	6.112141	3.041147	3.042453	3.026141	2.876716	2.875799	2.922023	2.64742
TGM1	206008_at	2.309781	2.286815	2.250981	2.273443	2.292998	2.270032	2.281389	2.287826	2.273014	2.266753	2.272298	2.279962	2.202653
ITGA9	206009_at	3.470421	3.395033	3.421974	3.426751	3.27283	2.46704	3.598202	3.219756	3.471577	3.395052	3.431807	3.437677	3.368037
HABP2	206010_at	11.43332	11.46099	11.07322	11.39876	11.38821	11.4943	9.204344	9.611773	8.988973	8.804798	8.408447	8.946828	2.255559
CASP1	206011_at	3.478158	3.470571	3.490151	3.460804	3.737072	3.45876	3.466475	2.581286	3.46318	3.455577	2.613978	2.582491	2.620815
LEFTY2	206012_at	2.18054	2.159189	2.130004	2.133052	2.160947	2.135642	2.148836	2.158272	2.138933	2.135509	2.13358	2.173819	3.371236
ACTL6B	206013_s_at	2.362681	2.317059	2.296224	2.304789	2.32818	2.288632	2.314893	2.321522	2.30647	2.300475	2.308712	2.317447	3.685754
ACTL6B	206014_at	2.722375	2.67115	2.621695	2.659915	2.706519	2.638472	2.672158	2.68205	2.669219	2.649253	2.668473	2.686927	4.065656
FOXJ3	206015_s_at	8.126696	8.080153	8.262164	8.237065	8.16874	7.538476	8.231841	7.887378	8.375117	8.288354	8.021986	7.976921	8.930601
CCDC22	206016_at	6.345268	6.449348	5.932026	6.294996	5.974679	6.312495	6.216367	6.231099	6.258433	6.369522	6.194399	6.757772	6.161135
KIAA0319	206017_at	2.59845	2.548596	2.523766	2.538005	2.605339	2.541247	2.544834	2.560787	2.545698	2.524218	2.542267	2.563163	2.417347
FOXG1	206018_at	2.363305	2.339858	2.302578	2.322147	2.596537	2.320516	2.331466	2.339317	2.36514	2.326377	6.407485	2.335509	5.324023
RBM19	206019_at	3.810788	3.756913	3.593385	3.740586	3.748481	3.745388	3.753149	3.751077	3.794153	3.742754	3.738841	3.744007	3.574765
SOCS6	206020_at	6.287496	6.534526	7.127204	7.115477	5.893882	5.872548	4.617611	5.476515	5.278438	5.087966	4.963635	5.190615	5.285081
SCAND2	206021_at	2.834003	2.772625	2.686691	2.781275	2.861067	2.799069	3.066803	2.773432	2.802159	2.796973	2.7782	2.802992	2.784644
NDP	206022_at	2.438854	2.407314	2.383887	2.388603	2.433358	2.387145	2.393819	2.404783	2.391105	2.377925	2.389862	2.400176	5.488432
NMU	206023_at	2.810769	2.784359	2.744745	2.773179	2.799408	2.755444	5.335057	3.049452	4.955776	6.291084	5.607884	6.398448	4.157203
HPD	206024_at	12.70091	12.68567	13.17326	13.26702	12.25081	12.03038	8.667475	7.358096	8.133716	9.011544	7.501541	7.628358	2.625323
TNFAIP6	206025_s_at	2.459325	2.447613	2.427096	2.428	2.45873	2.436456	2.413193	2.424861	2.407264	2.400411	2.410364	2.429865	6.456808
TNFAIP6	206026_s_at	3.05204	3.023597	3.020847	2.988517	3.038389	3.005963	3.001502	3.011193	2.99765	3.001798	3.013986	3.02358	7.134772
S100A3	206027_at	2.547029	2.509748	2.442157	2.494181	2.530616	2.48107	2.503262	2.527332	2.503553	2.486533	2.504454	3.564902	2.362926
MERTK	206028_s_at	5.281123	4.949679	6.865656	6.471763	4.867914	4.807065	6.631927	4.773072	5.597959	5.769912	5.23944	5.108249	4.97651
ANKRD1	206029_at	2.657989	2.605202	2.551375	2.592675	2.629966	2.582882	2.599176	2.614789	2.592025	2.579588	2.598694	2.623128	2.547271
ASPA	206030_at	7.069803	6.744533	7.877361	7.888991	7.700441	7.217978	2.270797	2.34715	2.262234	2.254043	2.009063	2.270525	2.496456
USP5	206031_s_at	4.381494	4.370715	4.402664	4.357998	4.117164	4.325377	5.433994	5.293858	5.017796	5.671981	5.303393	5.45585	4.230534
DSC3	206032_at	3.007557	2.911728	2.831688	2.922789	2.997378	2.862047	2.939844	2.912819	2.911246	2.927138	2.934282	2.959074	5.568977
DSC3	206033_s_at	2.506976	2.47892	2.429639	2.451549	2.487632	2.458787	2.555864	2.471612	2.458471	2.455327	2.462376	2.472493	4.324512
SERPINB8	206034_at	4.243856	3.973853	3.957239	3.908927	3.963284	3.837181	2.485364	3.956643	2.476109	2.961119	3.178961	3.02609	2.433396
REL	206035_at	2.961296	2.92794	2.837277	3.34704	3.579092	4.100019	2.468608	2.929591	2.465947	2.456705	2.467533	2.645335	2.408652
REL	206036_s_at	4.627992	4.517725	6.55559	5.979778	4.093156	3.106193	4.799095	3.359627	5.192027	5.124431	3.362506	3.597877	4.488071
CCBL1	206037_at	5.64732	6.152098	5.993591	5.672875	5.414184	5.612072	5.631768	5.650695	5.923831	5.886877	5.380729	5.755815	4.706936

NR2C2	206038_s_at	2.378971	2.348579	2.320604	2.579471	2.367823	2.331787	2.351576	2.353361	2.501746	2.710927	2.321898	2.439271	2.327429
RAB33A	206039_at	2.318965	2.305767	2.28256	2.277888	2.307415	2.298733	2.311644	2.303678	2.287111	2.264773	2.286956	2.301911	3.691277
MAPK11	206040_s_at	2.188518	2.167272	2.13665	2.148117	2.171734	2.149602	2.155883	2.164136	2.151862	2.14283	2.148469	2.160983	2.070832
SNRPN	206042_x_at	5.726023	5.698673	6.688747	6.136333	5.706053	5.284098	8.813054	6.412561	8.758135	8.77003	6.614524	6.922584	9.975817
ATP2C2	206043_s_at	2.165139	2.147217	2.126094	2.133581	2.14821	2.13431	2.144788	2.160647	2.136413	2.130625	2.129092	2.145157	2.067004
BRAF	206044_s_at	4.5376	4.501652	4.431654	4.433657	4.623745	4.522191	4.446315	4.676854	4.408203	4.362443	4.178555	4.654998	3.166266
NOL4	206045_s_at	2.213997	2.190442	2.167828	2.174722	2.200088	2.306784	2.184863	2.193374	2.177039	2.166917	2.171516	2.187519	2.114366
ADAM23	206046_at	2.281269	2.262458	2.226824	2.24407	2.272768	2.236152	2.25356	2.262241	2.247218	2.237012	2.246548	2.253933	4.448673
GNB3	206047_at	5.339116	5.309435	5.081423	5.132883	5.144522	5.132537	5.119149	5.144887	4.966111	5.105834	5.12726	5.137291	3.982139
OVOL2	206048_at	3.481029	3.367265	3.199998	3.356693	4.119145	3.311789	3.332885	3.33412	3.352078	3.344799	3.394009	3.448733	2.957765
SELP	206049_at	4.592221	4.16454	2.705845	2.054553	4.160167	4.244494	5.083964	4.715498	5.671008	5.304624	4.513515	5.838497	2.672459
RNH1	206050_s_at	7.899749	7.716276	8.014466	7.92706	7.953536	7.680186	7.90365	8.094668	8.286945	8.142744	7.921414	8.014063	7.889149
ELAVL4	206051_at	2.204237	2.192164	2.167852	2.177718	2.191764	2.178749	2.188359	2.196831	2.180139	2.175856	2.19676	2.19038	7.049689
SLBP	206052_s_at	7.450142	7.316803	7.186187	7.218359	6.558414	5.609343	10.05376	8.952503	10.1052	10.13984	8.687533	8.803221	9.003195
ZNF510	206053_at	2.295044	2.280514	2.2355	2.247396	2.281466	2.263137	2.275422	2.267262	2.552005	2.550142	2.443955	2.303702	4.515125
KNG1	206054_at	14.36892	14.36936	14.05837	14.15369	14.28842	14.17744	13.21463	12.6661	13.21576	12.94475	12.51703	13.06003	6.789838
SNRPA1	206055_s_at	9.144737	9.217357	9.122819	9.419668	9.321148	9.196469	10.22087	10.61448	10.30409	10.36966	10.41274	10.39192	9.481797
SPN	206056_x_at	5.364382	5.716311	4.853553	5.190216	5.482821	5.744251	5.296536	5.645842	5.068059	5.05267	5.578797	5.501191	4.954849
SPN	206057_x_at	2.240604	2.243283	2.204701	2.205634	2.245942	2.221247	2.59955	2.684811	2.22642	2.297093	2.224964	2.252287	2.160262
SLC6A12	206058_at	7.267372	7.154714	6.126441	6.149004	7.165928	6.761699	2.676721	3.551794	2.921126	2.313946	3.433815	2.518561	2.266043
ZNF91	206059_at	8.22461	8.189577	8.323199	8.244959	7.498983	7.163049	7.673293	7.029685	7.842031	7.77245	7.55606	7.040053	9.317382
PTPN22	206060_s_at	2.49947	2.465237	2.44529	2.46947	2.68418	2.463404	2.454987	2.468447	2.451414	2.441121	2.450839	2.461939	2.353343
DICER1	206061_s_at	6.355064	6.176446	7.86152	6.629996	5.321581	4.684536	8.212061	5.214603	8.009081	7.630609	5.267333	5.390989	9.440657
GUCA1A	206062_at	2.247667	2.238896	2.221178	2.22365	2.236649	2.223178	2.236064	2.238305	2.224734	2.212759	2.221391	2.215824	2.220166
PPIL2	206063_x_at	3.634219	3.886486	3.311877	3.310833	3.567961	3.60095	3.655543	4.366099	3.577406	3.621722	3.645072	4.10755	3.247066
PPIL2	206064_s_at	2.272363	2.260286	2.234709	2.23998	2.266046	2.24678	2.250011	2.259898	2.245151	2.360541	2.237486	2.253451	2.195587
DPYS	206065_s_at	12.00535	11.99086	12.35705	12.2896	11.55189	10.93707	10.27263	9.12592	9.905107	9.794587	8.657153	8.891584	4.281623
RAD51C	206066_s_at	2.51277	2.498419	2.479531	2.454112	2.472556	2.482034	4.06958	3.711488	4.204454	4.94234	2.794699	3.285461	3.419089
WT1	206067_s_at	2.994455	3.065871	3.386134	3.078926	3.147655	3.03529	4.10526	3.28443	3.184849	4.238361	7.416135	4.369824	7.713077
ACADL	206068_s_at	7.881745	7.67171	9.265427	9.197236	8.783741	8.348911	6.071887	5.327044	4.941398	4.934923	4.596696	4.775102	3.104647
ACADL	206069_s_at	5.399202	5.354331	7.256361	6.773393	5.759461	5.067687	3.452043	2.85798	3.595609	3.264781	2.639983	2.667909	2.457749
EPHA3	206070_s_at	4.990071	5.282926	4.839737	3.889883	3.50948	3.269821	2.849777	2.858519	2.848667	2.81865	3.385252	2.885629	8.534548
EPHA3	206071_s_at	2.315583	2.293129	2.265797	2.267213	2.298468	2.271543	2.281772	2.286983	2.27411	2.259945	2.27265	2.283902	6.043863
UCN	206072_at	2.609182	2.414666	2.367124	2.400404	2.386941	2.354143	2.354811	2.365083	2.261267	2.342241	2.340348	2.334104	2.235577
COLQ	206073_at	2.662545	2.931747	2.608	2.616287	2.644941	2.614431	2.624931	2.594272	2.621382	2.60763	4.580965	2.633487	4.088345
HMGA1	206074_s_at	6.814007	6.368191	6.306106	6.347342	6.362974	6.399381	12.40914	12.26289	12.39467	12.22115	12.06934	12.33444	8.440767
CSNK2A1	206075_s_at	2.586323	2.732623	3.462043	3.284384	2.568607	2.519106	5.89367	3.482708	6.132771	6.08091	3.297902	3.554246	6.944488
LRRC23	206076_at	2.304872	2.264622	2.267371	2.381664	2.278018	2.270751	2.213855	3.122766	2.369776	2.247174	2.362026	2.397668	2.940283

KEL	206077_at	2.143042	2.127941	2.108729	2.110524	2.126035	2.109927	8.860458	8.45834	8.714895	8.689903	8.133091	8.252816	2.076498
KALRN	206078_at	4.267466	4.066457	6.227763	6.389038	6.36741	6.084898	3.540645	2.738379	3.521127	2.878377	2.597481	3.156285	2.498973
CHML	206079_at	2.569794	2.541474	2.518955	2.521395	2.551828	2.525865	2.537089	2.547259	2.531863	2.524539	2.526265	2.620342	2.450174
PLCH2	206080_at	2.311139	2.305935	2.280357	2.446994	2.30097	3.090984	2.295053	2.291803	2.28552	2.288832	2.282258	2.291776	2.232229
SLC24A1	206081_at	4.938907	5.269481	4.910321	5.011061	5.688015	5.258202	5.327269	5.094928	5.15092	5.242769	5.794387	5.355197	5.441525
BAI1	206083_at	2.364934	2.401114	2.327362	2.31506	2.331603	2.33901	2.350752	2.884904	2.347096	2.389896	2.347136	2.558556	2.251791
PTPRR	206084_at	2.228353	2.197407	2.172187	2.181298	2.204466	2.178519	2.189786	2.196496	2.181439	2.176359	2.265876	2.190748	3.543975
CTH	206085_s_at	5.071536	5.348627	8.961126	8.465553	4.601969	2.244004	3.247408	2.068964	3.97426	3.174885	2.006605	1.990768	2.410905
HFE	206086_x_at	2.558785	2.536562	2.550682	2.554754	2.564905	2.556792	2.566775	2.565212	2.560974	2.552289	2.552043	2.55931	2.518624
HFE	206087_x_at	6.666451	7.174175	6.579363	6.668169	6.4258	6.394784	6.107551	6.073876	6.192796	6.343674	5.780258	6.066359	5.952514
LRRC37A2	206088_at	2.661897	2.631143	2.726282	2.628957	2.744978	2.868446	2.766766	2.651911	4.023539	3.427374	2.623013	3.409187	3.386796
NELL1	206089_at	1.889741	1.877571	1.857614	1.861092	1.876943	1.859758	1.872184	1.873931	1.862659	1.857196	2.666065	1.866876	5.563503
DISC1	206090_s_at	2.931131	2.920234	2.877541	2.993422	2.876779	2.98458	3.24531	2.920923	2.907694	2.889227	2.916494	2.916971	4.330749
MATN3	206091_at	3.043239	2.927486	2.845135	2.914917	3.042238	2.884346	2.923338	2.929864	2.928962	2.906336	2.941765	2.978795	5.093056
RTEL1	206092_x_at	2.46824	2.449099	2.431744	2.437219	2.4664	2.435217	2.44683	2.449819	2.437117	2.430497	2.490868	2.47407	2.39814
TNXB	206093_x_at	7.061737	7.577521	6.520778	6.85803	8.55887	9.142541	3.842821	5.706059	3.852091	4.570318	6.004317	4.591728	6.831646
UGT1A6	206094_x_at	12.65741	12.58878	13.02462	13.07488	11.91239	11.34119	4.128113	3.24651	3.488207	2.806876	2.349767	3.074645	2.222054
FUSIP1	206095_s_at	7.96412	7.840095	8.456662	8.284842	7.361512	6.834186	9.224579	7.951516	9.168131	9.395113	7.730489	7.747254	9.042676
ZNF35	206096_at	2.742719	3.251435	3.221895	3.052198	3.183355	2.976314	3.421424	4.063806	3.354891	3.675139	3.811104	3.870528	4.514843
SLC22A18A	206097_at	2.988394	2.970829	3.921585	3.385348	4.30661	3.227562	2.936663	2.949439	2.93585	2.936575	2.69403	2.931945	2.572142
ZBTB6	206098_at	2.609088	2.571643	2.52179	2.637452	2.590287	2.539053	2.58688	2.552928	2.571931	2.906969	2.562098	2.574845	5.085429
PRKCH	206099_at	2.553112	2.539961	2.522822	2.524698	2.539318	2.50397	2.537265	2.538948	2.522365	2.520115	2.520812	2.532241	2.557026
CPM	206100_at	6.408902	6.460648	7.800904	7.338036	6.925865	6.556003	6.579214	4.947542	6.035045	5.9369	5.255084	6.000938	4.31175
ECM2	206101_at	9.455768	9.339127	8.789657	8.757989	8.78776	8.692126	8.357563	8.246133	8.608031	7.553915	7.350754	7.542599	4.98975
GINS1	206102_at	5.356424	4.985999	2.694697	2.715394	2.77835	2.58751	9.111852	8.799078	8.845715	8.893802	8.586338	8.788056	8.174269
RAC3	206103_at	2.79108	2.758079	2.728735	2.738639	2.763532	2.736954	2.603217	2.754436	2.724433	2.722061	2.740079	2.738001	2.666823
ISL1	206104_at	2.216739	2.19842	2.175054	2.180513	2.200021	2.180066	2.193866	2.196193	2.185341	2.176199	2.889803	2.191615	7.972949
AFF2	206105_at	2.027048	2.018291	1.998248	2.002136	2.016263	2.004944	2.015973	2.023674	2.004385	2.003553	1.999716	2.012946	4.612609
MAPK12	206106_at	2.517275	3.172789	2.41629	2.487546	2.661141	2.647097	2.497473	2.513147	2.492815	3.047599	3.666093	3.055564	2.676638
RGS11	206107_at	2.329469	2.311111	2.260024	2.293932	2.310505	2.293485	2.310189	2.310645	2.322369	2.299283	2.293697	2.303171	2.232668
SFRS6	206108_s_at	2.683637	2.439651	3.04152	3.33178	2.988513	3.021143	4.924116	2.549456	6.035135	6.265529	3.312729	3.078766	5.38117
FUT1	206109_at	2.485567	2.473586	2.427721	2.398297	2.499778	3.455592	2.968059	3.459194	3.402037	2.559758	2.463715	2.853233	2.127816
HIST1H3H	206110_at	2.728599	2.610475	2.594528	2.562102	2.442158	2.384468	3.715302	5.606189	5.139702	5.667934	4.518532	4.994119	2.573666
RNASE2	206111_at	3.008143	2.945747	2.918114	2.922092	2.948168	2.924475	3.095444	2.955291	3.016353	3.120372	2.948008	2.990894	2.644011
ANKRD7	206112_at	2.733499	2.710246	2.631836	2.68827	2.721858	2.700415	2.696697	2.706422	2.695366	2.687476	2.695508	2.730312	2.613491
RAB5A	206113_s_at	2.919719	2.922746	3.726746	3.122189	2.906413	2.787606	4.965788	2.903829	4.805618	4.728968	2.884413	2.781494	6.899219
EPHA4	206114_at	2.250323	2.242411	2.222718	2.228655	2.285747	2.230354	2.235876	2.246249	2.233108	2.229624	5.79295	2.343048	6.991459
EGR3	206115_at	3.490832	3.324314	6.109196	5.43604	7.71169	7.433132	3.481621	3.481364	3.476876	3.470918	4.083515	3.56097	4.763235

TPM1	206116_s_at	5.554914	5.654795	6.982656	6.027842	5.78692	4.918018	8.003078	4.999252	7.695769	7.743153	9.986758	5.7783	12.23721
TPM1	206117_at	2.638356	2.716606	2.723397	2.73725	3.732768	3.951904	2.72373	2.756826	2.722139	2.703378	9.130212	2.751403	10.77055
STAT4	206118_at	6.666534	6.710902	4.972323	4.413773	3.547399	4.989604	2.477775	2.456838	2.658504	2.465423	2.462473	2.450239	3.451803
BHMT	206119_at	12.08758	11.92559	12.96357	13.09224	12.87037	12.7076	10.029	10.32283	10.11954	10.94252	9.434519	8.99334	4.798378
CD33	206120_at	2.217129	2.172116	2.186336	2.190982	2.227524	2.357056	2.20312	2.211557	2.196703	2.195839	2.192078	2.393692	2.024581
AMPD1	206121_at	2.195809	2.181262	2.156224	2.162157	2.251763	2.160785	2.172055	2.177447	2.163735	2.155316	2.476295	2.173034	2.584193
SOX15	206122_at	2.609498	2.560916	2.530573	2.543612	2.587365	2.542422	2.551537	2.598514	2.545924	2.538175	2.603418	2.559169	3.155284
LLGL1	206123_at	5.605734	5.975499	5.526653	5.831572	5.905605	6.129039	6.288887	6.346825	5.938907	5.946283	6.015145	6.248119	5.601603
LLGL1	206124_s_at	2.795589	2.791237	2.761686	2.702191	2.781905	3.006075	2.775621	2.786122	2.766724	2.775794	2.770752	3.144867	2.6715
KLK8	206125_s_at	2.085144	2.068815	2.043019	2.04975	2.068315	2.04839	2.059795	2.064321	2.052372	2.053994	2.198526	2.056731	1.986182
BLR1	206126_at	2.959878	2.929333	3.484654	2.912336	2.937055	2.915225	2.924838	2.95399	2.915544	2.911123	2.920549	2.927592	2.817005
ELK3	206127_at	2.128237	2.119942	2.085613	2.102592	2.138522	2.101248	2.11195	2.117571	2.104146	2.083248	2.106555	2.1156	2.316689
ADRA2C	206128_at	3.741625	3.770928	3.750612	3.734673	3.846493	3.921236	3.765253	3.766769	3.574256	3.750018	3.833927	3.760434	3.706523
ARSB	206129_s_at	2.298367	2.279829	2.263856	2.265453	2.284101	2.266866	2.279308	2.283263	2.275209	2.262733	2.269057	2.277447	2.201671
ASGR2	206130_s_at	11.81664	11.82841	11.65575	11.8869	11.94841	11.88867	11.07815	11.28537	11.07746	11.06995	11.25521	11.38944	4.359776
CLPS	206131_at	2.054127	2.296413	2.021243	2.02536	2.040845	2.021927	2.036513	2.038137	2.027036	2.02135	2.019392	2.032355	1.943128
MCC	206132_at	3.167513	3.469012	4.827829	3.32442	3.156504	3.358519	4.394969	3.155514	4.027287	4.436174	3.028554	3.460568	5.167507
XAF1	206133_at	5.366432	5.370127	6.60094	5.81793	5.852783	6.423623	3.967129	3.712351	3.963523	3.680539	3.785959	3.712797	3.697262
ADAMDEC1	206134_at	2.373878	2.369954	2.31434	2.311961	2.375732	2.336835	2.330192	2.342244	2.332599	2.323379	2.327913	2.339819	2.237677
ST18	206135_at	2.680155	2.602643	2.544778	2.586659	2.639067	2.565424	2.596033	2.603678	2.595543	2.574187	2.625777	2.612841	5.509662
FZD5	206136_at	4.82889	4.437711	5.133834	5.348802	4.445344	4.132492	4.403965	3.906571	4.426053	4.347072	3.522491	3.901466	2.121581
RIMS2	206137_at	2.226309	2.211152	2.187809	2.195045	2.208709	2.195681	2.206432	2.209262	2.200759	2.194366	2.190776	2.200586	2.922198
PI4KB	206138_s_at	7.784221	7.675466	7.488808	7.573403	7.854642	8.110996	7.669449	8.271949	7.951827	7.941347	8.274874	8.289869	8.156022
PI4KB	206139_at	5.263906	5.375109	4.488525	4.578338	5.201354	5.341412	5.681959	5.969195	5.547463	5.342772	5.48622	6.031151	4.392582
LHX2	206140_at	6.045337	5.378247	5.801032	6.111005	6.973769	7.021633	5.753204	5.220363	4.939979	4.580781	7.164997	5.895664	4.430064
MOCS3	206141_at	2.703836	2.675623	2.654475	2.747881	2.710722	2.678165	2.809359	2.667592	3.525977	2.740415	2.653023	2.702428	3.115731
ZNF135	206142_at	3.032761	3.049293	2.959647	2.973972	3.011918	2.970156	3.146538	2.998783	2.983706	3.230295	3.180617	3.164214	6.209227
SLC26A3	206143_at	2.71949	2.652272	2.590361	2.642282	2.737036	2.614074	7.526343	6.570274	8.19275	7.565161	6.946903	6.61306	2.485004
MAGI1	206144_at	4.085825	3.271711	3.214113	3.48241	3.080839	3.226392	2.488692	2.730911	2.982176	2.562029	2.493696	2.743534	4.122555
RHAG	206145_at	2.515015	2.474641	2.447253	2.467044	2.505098	2.463689	11.91956	11.86	12.0415	11.8245	11.08694	11.6091	4.845032
RHAG	206146_s_at	2.434223	2.345106	2.381002	2.383552	2.508396	2.514868	9.031843	6.775248	8.965921	8.932723	6.524411	6.514915	2.375901
SCML2	206147_x_at	6.941563	6.936811	6.926523	6.920801	6.67227	6.913804	7.512802	7.33311	7.1771	7.642411	7.156849	7.008018	6.532319
IL3RA	206148_at	2.904888	2.889899	2.790556	2.874797	2.915385	2.866434	2.88448	2.895338	2.881854	2.866666	2.877789	2.892349	2.783196
LOC63928	206149_at	2.126421	2.116629	2.096319	2.098346	2.115304	2.106299	2.117136	2.121519	2.115203	2.338389	2.170483	2.150062	2.075199
CD27	206150_at	3.386464	3.424426	3.314528	3.358273	3.369374	3.358636	3.369465	3.370082	3.351557	3.3568	3.144318	3.361891	2.462023
ELA3B	206151_x_at	2.540756	2.50568	2.468089	2.493273	2.504929	2.498731	2.505001	2.506043	2.526227	2.491545	2.494896	2.513622	2.377277
CENTG1	206152_at	3.219616	3.210124	3.190696	3.167213	3.204566	3.193656	3.209383	3.209669	3.190021	3.197093	3.212245	3.20388	3.143522
CYP4F11	206153_at	8.294541	8.199733	9.173893	9.090216	7.245291	6.561161	2.295627	2.967801	2.681581	2.979424	2.973706	2.977948	2.034342

RLBP1	206154_at	1.877471	1.869093	1.846766	1.851416	1.882894	1.851155	1.868213	1.909385	1.853629	1.856044	1.851264	1.861317	1.777649
ABCC2	206155_at	7.476969	7.486907	8.579898	8.209767	6.985554	6.291607	6.355805	4.646831	6.006361	6.246609	4.338336	4.81682	2.145067
GJB5	206156_at	1.945322	1.931184	1.910296	1.914161	1.935432	1.913256	1.926233	1.92809	1.916123	1.908985	1.913591	1.922713	1.863735
PTX3	206157_at	3.375973	3.259179	3.212995	3.134265	3.368567	3.163868	3.262431	3.283307	3.368998	3.245228	6.561864	3.361384	10.51741
CNBP	206158_s_at	11.68334	11.67943	12.47665	12.21431	11.77883	10.69527	12.26998	10.94271	12.26488	12.31126	10.95978	11.00737	12.5946
GDF10	206159_at	2.497585	2.460039	2.413388	2.443349	2.479472	2.442371	2.453139	2.463836	2.449277	2.433933	2.474129	2.458383	6.06862
APOBEC2	206160_at	2.855298	2.887024	2.872818	2.811762	2.898611	2.861459	2.824939	3.090824	2.886269	3.577017	6.112461	2.880976	8.023852
SYT5	206161_s_at	2.548196	2.538285	2.518896	2.523442	2.53649	2.52482	2.533371	2.534517	2.524643	2.514379	2.516528	2.52808	2.482945
SYT5	206162_x_at	2.968565	2.915053	2.853914	2.91351	2.946209	2.900114	2.922008	2.935349	2.918431	2.906237	2.934644	2.954822	2.695188
MAB21L1	206163_at	2.289655	2.262751	2.226334	2.242335	2.273874	2.245235	2.251511	2.263166	2.247807	2.235923	2.248719	2.256744	8.361668
CLCA2	206164_at	2.783333	2.67359	2.606772	2.660243	2.70857	2.625792	2.653792	2.673591	2.657731	2.636616	2.653368	2.681196	2.475854
CLCA2	206165_s_at	1.944644	1.93033	1.909365	1.916781	1.930214	1.912337	1.925204	1.927126	1.914407	1.908084	1.908991	1.92178	1.863591
CLCA2	206166_s_at	2.460545	2.429453	2.391502	2.409697	2.482025	2.402083	2.417154	2.429694	2.414939	2.403445	2.422846	2.48288	2.304019
ARHGAP6	206167_s_at	5.133088	4.868718	4.705374	4.946506	5.357623	5.820884	6.606834	6.987697	6.622625	6.423459	7.650315	7.848624	6.972007
ZC3H7B	206168_at	2.039355	2.020437	1.998239	2.004532	2.022426	2.001541	2.013328	2.018228	2.005091	2.004115	2.002637	2.013278	1.942036
ZC3H7B	206169_x_at	8.93839	8.974592	7.831641	8.107211	9.067202	9.850047	7.431406	8.964771	7.229078	7.572508	9.182494	8.598516	7.569695
ADRB2	206170_at	7.000358	6.923282	7.615538	7.878547	7.880754	7.666291	4.096221	4.788297	4.698101	4.278088	4.58178	5.491326	3.112996
ADORA3	206171_at	2.196902	2.189329	2.440456	2.180858	2.322169	2.183334	2.193247	2.191615	2.177594	2.181131	2.177918	2.209807	2.089332
IL13RA2	206172_at	7.036819	6.699504	7.370047	7.858986	5.575872	5.364085	2.754229	2.477167	2.484158	2.438368	2.742108	2.550798	2.995631
GABPB2	206173_x_at	3.001772	2.968365	2.950796	3.028931	2.979984	2.940619	4.588955	3.78534	4.786724	4.799153	3.194768	3.293173	4.616517
PPP6C	206174_s_at	6.779674	6.612959	7.301728	6.833645	6.017199	5.516866	8.747836	7.190517	8.613489	8.666779	6.740795	7.12117	8.196288
ZNF222	206175_x_at	3.591006	3.456589	3.971872	3.639175	3.465636	3.437199	3.918553	3.432709	4.273173	4.265049	3.302967	3.470125	5.131862
BMP6	206176_at	2.043412	2.022421	2.716373	2.10909	2.024431	2.00628	2.018268	2.026463	2.009243	2.003456	2.004937	2.016125	2.634324
ARG1	206177_s_at	13.82194	13.79043	14.07766	14.21822	13.77843	13.51699	13.17409	12.7219	13.47158	13.47683	12.04636	12.38231	6.82128
PLA2G5	206178_at	3.211045	3.191546	3.647682	3.250203	3.31026	3.388996	3.171327	3.152859	3.172472	3.164085	3.175423	3.188445	3.085503
TPPP	206179_s_at	2.447669	2.405829	2.409088	2.395161	2.427388	2.41097	2.408449	2.424127	2.402677	2.39399	2.401794	2.418772	2.570237
ZNF747	206180_x_at	2.946843	2.911501	2.841883	2.919824	3.178917	2.918983	3.391676	3.178142	2.922721	3.239117	2.920546	2.948316	2.864673
SLAMF1	206181_at	2.045527	2.031414	2.016015	2.018302	2.034705	2.227192	2.024378	2.031646	2.017339	2.009587	2.013205	2.025234	1.968645
ZNF134	206182_at	4.452804	4.396526	4.400106	4.400224	4.447179	4.538422	5.016978	5.237595	4.798084	4.999946	4.498259	5.155242	4.836578
HERC3	206183_s_at	3.318464	3.12729	3.474993	3.443185	3.178503	3.07721	3.292744	3.080945	3.178623	3.401918	3.165731	3.182593	2.786599
CRKL	206184_at	2.254153	2.24928	2.226235	2.232272	2.242916	2.238438	2.246849	2.244096	2.241813	2.23537	2.231999	2.237372	3.098437
CRYBB1	206185_at	1.8071	1.798412	1.779877	1.784232	1.797743	1.784719	1.798181	1.796589	1.787699	1.785227	1.778138	2.023863	4.257249
MPP3	206186_at	2.306168	2.289203	2.305145	2.270976	2.291328	2.275374	2.278401	2.292305	2.275445	2.271548	2.278418	2.27977	3.369684
PTGIR	206187_at	3.60115	3.903513	3.71109	3.195263	3.741325	4.271638	3.883032	3.740693	3.97156	3.726716	4.5441	3.732978	3.120737
ZNF623	206188_at	5.846598	5.600635	5.184762	5.085823	5.187093	5.199785	5.285141	5.689466	5.421811	5.53283	5.420243	5.307655	6.685825
UNC5C	206189_at	2.347761	2.32379	2.291929	2.3086	2.323737	2.300413	2.314358	2.325625	2.312453	2.300959	2.31217	2.324982	3.019424
GPR17	206190_at	2.159433	2.143006	2.12337	2.127247	2.144475	2.126659	2.139324	2.141807	2.127971	2.121979	2.129349	2.162134	2.271385
ENTPD3	206191_at	2.112879	2.085934	2.061827	2.067061	2.087151	2.066297	2.078757	2.082467	2.070384	2.061412	2.07051	2.077173	3.178828

CDSN	206192_at	2.12206	2.112826	2.091236	2.093184	2.114961	2.099832	2.105749	2.110936	2.095902	2.092761	2.095351	2.100807	2.057975
CDSN	206193_s_at	2.466919	2.437858	2.386515	2.415291	2.510427	2.418105	2.423202	2.434636	2.418997	2.406004	2.42158	2.457464	2.286818
HOXC4	206194_at	3.417906	3.330288	3.293149	3.372331	3.464096	3.36663	3.724226	3.179663	3.35463	3.301538	4.212435	3.385823	4.372765
GH2	206195_x_at	2.340636	2.317173	2.275424	2.302794	2.313461	2.30138	2.305386	2.312642	2.362605	2.300754	2.298293	2.304964	2.254244
RUNDC3A	206196_s_at	2.055603	2.067667	2.02266	2.026642	2.06633	2.045028	8.064719	8.478109	8.595823	8.44645	8.256046	7.765964	3.961831
NME5	206197_at	2.91311	2.428449	2.371208	2.408856	2.428127	2.385085	2.40746	2.424074	2.420999	2.392894	2.405758	2.420235	4.212269
CEACAM7	206198_s_at	2.620108	2.587626	2.554498	2.567891	2.620459	2.543659	2.578177	2.589695	2.57407	2.556737	2.574794	2.599762	2.408924
CEACAM7	206199_at	2.47052	2.433937	2.380648	2.417749	2.440801	2.422221	2.427749	2.43666	2.423066	2.40804	2.421468	2.431231	2.341594
ANXA11	206200_s_at	8.771454	8.946713	8.683436	8.945066	9.09125	9.281713	7.897167	8.123036	7.873524	8.106333	8.375399	8.126859	8.113652
MEOX2	206201_s_at	3.916527	3.840838	3.384644	3.403049	4.311904	4.106715	3.382642	3.388303	3.373216	3.256968	7.649838	3.42478	10.36706
MEOX2	206202_at	2.555953	2.511814	2.469931	2.491733	2.538473	2.482019	2.502684	2.513345	2.500404	2.485285	2.50264	2.514914	2.350866
RCVRN	206203_at	1.962374	1.946841	1.92776	1.93064	1.948385	1.964583	1.943055	1.944049	1.934145	1.926747	1.926315	1.940011	1.877452
GRB14	206204_at	10.10442	10.01728	10.53377	10.49557	9.884772	9.159578	3.158337	2.507085	4.707881	4.491341	3.717424	2.911998	5.282576
MPHOSPH9	206205_at	5.015692	4.956655	4.925845	4.946139	4.266789	3.806508	6.844109	6.307584	6.893891	6.994651	5.788236	6.143452	4.986769
CD180	206206_at	3.628273	3.799936	3.669511	3.646093	3.654351	3.621106	3.61439	3.624888	3.563264	3.520377	3.555326	3.662484	2.91497
CLC	206207_at	2.370554	2.360229	3.073112	2.691359	2.357071	2.342771	2.532772	4.461369	4.157526	4.919565	2.526699	3.213836	2.209813
CA4	206208_at	3.006872	2.95792	2.946547	2.968343	3.133499	2.938895	2.973602	3.498976	2.932835	3.095472	2.938056	2.989487	2.871375
CA4	206209_s_at	2.326701	2.30075	2.279923	2.286268	2.339183	2.278931	2.285697	2.297998	2.287534	2.271	2.278926	2.292657	2.245291
CETP	206210_s_at	6.529126	6.465834	7.435018	7.739532	6.934451	6.729244	6.213063	6.475084	6.498915	5.355129	5.503906	6.228301	2.59483
SELE	206211_at	2.806602	2.731746	3.570707	3.08023	3.493161	3.351025	2.651909	2.747029	2.688608	2.637185	2.679516	2.657255	2.520095
CPA2	206212_at	2.173351	2.148428	2.122755	2.128898	2.119007	2.127714	2.144089	2.148746	2.137772	2.124308	2.147236	2.168395	3.332546
WNT10B	206213_at	3.443981	3.852704	3.371136	3.420091	3.428861	3.478516	3.570044	3.421673	3.413807	4.000428	3.542092	3.467903	3.329999
PLA2G7	206214_at	4.311636	4.232455	5.25804	5.192288	5.68291	6.047195	9.59036	8.8499	9.600472	8.935799	8.237963	8.69475	4.059376
OPCML	206215_at	2.26402	2.252744	2.234367	2.234	2.254025	2.227006	2.249312	2.251948	2.241118	2.23377	2.238275	2.246501	2.403568
SRPK3	206216_at	2.618196	2.59014	2.521744	2.567228	2.617158	2.551436	2.577479	2.615964	2.573556	2.579832	5.038775	2.583065	5.087206
EDA	206217_at	3.157476	3.146217	3.223089	3.259992	3.253569	3.339296	3.244197	3.412956	3.238098	3.232015	3.373705	3.236344	3.266958
MAGEB2	206218_at	2.541589	2.316035	2.283522	2.300777	2.34874	2.307578	2.311765	2.320499	2.309732	2.305636	2.311864	2.34368	2.221375
VAV1	206219_s_at	2.854188	2.828008	2.742048	2.803412	2.953781	3.169811	2.835054	2.827777	2.828106	2.816497	2.825851	2.837352	2.63621
RASA3	206220_s_at	2.596451	2.574793	2.469447	2.55982	2.601715	2.56096	2.574918	2.578244	2.56521	2.557058	2.560403	2.607836	3.310641
RASA3	206221_at	2.415904	2.381841	2.344701	2.363549	2.39803	2.351574	2.372126	2.383766	2.369261	2.355244	2.368678	2.378498	2.348014
TNFRSF10C	206222_at	2.590362	2.517878	2.473667	2.516351	2.528198	2.504617	2.505906	2.517653	2.502038	2.434616	2.62577	2.511506	2.362852
LMTK2	206223_at	2.169685	2.15179	2.127457	2.134349	2.152862	2.135003	2.147525	2.149889	2.137211	2.12881	2.13719	2.144384	2.077243
CST1	206224_at	2.805614	2.756868	2.722448	2.753415	2.800472	2.735814	2.763077	2.765374	2.757265	2.760261	2.894051	2.770714	2.620031
ZNF507	206225_at	1.741428	1.732322	1.712991	1.717184	1.728099	1.716205	1.732067	1.731232	1.727565	1.716338	1.717158	1.72343	1.704009
HRG	206226_at	14.09008	14.09381	13.90724	14.10527	14.27391	14.05631	11.75782	11.39447	11.88968	11.72168	11.21556	12.20431	4.20424
CILP	206227_at	2.090038	2.078886	2.061969	2.066837	2.079351	2.505338	2.041246	2.076625	2.063118	2.054931	2.05897	2.041755	6.012057
PAX2	206228_at	1.806176	1.770405	1.749171	1.761443	1.773966	1.75213	1.764856	1.77322	1.765918	1.758731	1.755318	1.767209	4.039201
PAX2	206229_x_at	2.813697	2.803708	2.784137	2.787937	2.803063	2.794472	2.798818	2.804845	2.78911	2.792922	2.789395	2.796048	2.768522

LHX1	206230_at	2.239903	2.218399	2.165697	2.207018	2.265783	2.21457	2.228664	2.557205	2.218126	2.212669	2.215757	2.242261	2.18877
KCNN1	206231_at	2.811268	2.779647	2.742303	2.619761	2.60872	2.752864	3.400662	2.896035	2.767854	2.768155	2.758062	2.776102	2.692617
B4GALT6	206232_s_at	1.992816	1.974227	1.951465	1.955063	1.973345	1.955829	1.967375	1.970701	1.96477	1.950832	1.955998	1.96506	2.299478
B4GALT6	206233_at	3.04299	2.904414	2.871736	2.912764	3.08174	3.009569	3.675139	3.158489	5.122162	4.470441	3.124964	3.058746	6.236305
MMP17	206234_s_at	2.314815	2.36618	2.279813	2.271672	2.300796	2.611425	2.279886	2.290467	2.407835	2.283262	2.2792	2.306365	2.252591
LIG4	206235_at	3.292004	3.93001	4.69257	3.490512	3.114283	2.951701	3.840909	3.061538	3.90955	4.572032	3.30661	3.21128	4.543649
GPR4	206236_at	3.074022	3.066895	3.009056	3.172741	3.732719	3.829783	3.037409	2.81503	3.045988	2.969773	2.947518	3.057685	2.601387
NRG1	206237_s_at	3.549332	3.238713	2.740267	2.842236	2.914685	3.44785	2.523194	2.705912	2.51287	2.759082	2.521801	2.700439	2.137731
YAF2	206238_s_at	2.955946	3.005089	3.082309	3.31046	2.946163	2.878465	3.044411	2.906527	3.040022	3.107852	2.939112	2.967001	3.829773
SPINK1	206239_s_at	4.404132	4.760937	5.786112	4.607609	5.838689	7.765173	11.23812	11.16485	7.999645	7.77502	12.85664	12.80411	8.294524
ZNF136	206240_s_at	6.261568	6.196596	5.265193	5.542491	6.186795	6.108755	5.701364	5.647557	5.874386	5.893329	6.145152	5.797194	6.144883
KPNA5	206241_at	2.799852	2.76079	2.779892	2.737528	2.796903	2.715144	2.758439	2.748278	2.747334	2.738463	2.736523	2.769994	4.889137
TM4SF5	206242_at	9.33683	9.357836	8.131247	8.392084	9.724469	9.489704	8.796588	8.598663	9.181447	9.085451	8.580772	8.891656	2.758486
TIMP4	206243_at	2.352817	2.339246	2.318685	2.327293	2.392958	2.422757	2.335807	2.339164	2.328334	2.317854	2.326493	2.338399	2.265391
CR1	206244_at	2.789839	2.75562	2.797479	2.713469	2.7937	2.68749	2.824772	2.902818	3.396869	3.470155	2.738021	3.4482	2.536626
IVNS1ABP	206245_s_at	6.635666	6.760109	7.950484	7.746521	7.832436	7.541687	8.667171	7.941648	8.588546	8.664523	7.815571	7.942493	9.033773
PFKFB4	206246_at	2.398664	2.389609	2.373386	2.37678	2.386675	2.53725	2.389302	2.388061	2.382851	2.375981	2.370407	2.381168	2.186875
MICB	206247_at	5.73303	5.437758	4.707658	4.218449	5.394199	6.451158	5.130247	5.653841	4.243656	4.552973	5.965757	5.857625	3.032728
PRKCE	206248_at	2.212902	2.194575	2.168818	2.175179	2.19697	2.181576	2.185198	2.192747	2.177796	2.170307	2.173761	2.187405	2.109303
MAP3K13	206249_at	6.29116	6.439733	6.04418	5.937987	6.233391	5.181955	4.428759	3.654884	4.480639	4.311731	3.47106	3.972155	4.831315
AVPR1A	206250_x_at	2.454565	2.445712	2.430116	2.434257	2.528705	2.430376	2.440255	2.434576	2.440823	2.425064	2.417537	2.430128	2.393278
AVPR1A	206251_s_at	2.552734	2.516031	2.47418	2.499538	2.533564	2.493429	2.507579	2.515604	2.504135	2.493881	2.500521	2.512752	2.379088
AVPR1A	206252_s_at	2.541335	2.500181	2.99394	3.934084	3.26228	2.572617	2.515333	2.327908	2.499776	2.478593	2.3646	2.375109	2.242497
DLG2	206253_at	2.795036	2.759767	2.716772	2.73379	2.778879	2.700242	2.738634	2.733412	2.730297	2.65122	2.741694	2.760978	3.08049
EGF	206254_at	3.142283	3.097318	2.96927	3.103613	3.254969	3.084276	3.119344	3.117984	3.102757	3.335835	3.104379	3.183495	2.827091
BLK	206255_at	2.266666	2.241651	2.213947	2.224547	2.24439	2.222939	2.228026	2.23738	2.223556	2.218479	2.218393	2.233095	2.12667
CPN1	206256_at	8.120925	8.285627	8.78866	8.741017	8.877016	8.446127	8.164847	8.051242	8.258815	8.34883	7.489439	8.543195	1.800068
CCDC9	206257_at	3.568068	3.353016	3.534179	3.207077	3.575591	3.563089	3.580066	4.365278	3.575361	3.558995	3.897272	3.943054	3.176434
ST8SIA5	206258_at	2.608414	2.580346	2.531363	2.551753	2.574646	2.564493	2.57538	2.578598	2.566092	2.549346	2.559417	2.588032	2.555606
PROC	206259_at	12.05453	11.98767	11.71426	11.91356	12.26793	12.3221	9.280701	9.732198	9.272809	9.276886	9.651994	9.967268	3.162406
TGM4	206260_at	2.262482	2.244783	2.223144	2.226734	2.245751	2.22757	2.237559	2.242389	2.228532	2.222841	2.227504	2.238789	2.180779
ZNF239	206261_at	3.036772	3.02884	2.995477	3.006524	3.018459	2.93354	4.364756	3.038063	3.322773	3.92248	4.87892	4.620446	5.161306
ADH1A	206262_at	13.70524	13.70184	13.81668	13.96613	12.95474	12.63717	4.353317	4.17706	4.301655	3.867684	4.214536	4.992781	3.949923
FMO4	206263_at	7.032206	6.771603	7.238294	7.211147	6.182956	6.18215	3.542733	3.592596	2.934201	2.668506	2.68858	2.700692	2.624468
GPLD1	206264_at	4.364705	4.548446	5.598167	4.854708	4.416411	4.319111	3.103094	3.431916	3.585963	2.960427	2.836211	3.311278	2.867928
GPLD1	206265_s_at	4.07634	3.86475	6.423897	6.500764	4.304191	4.670803	2.468819	2.927277	2.388653	2.806256	2.407637	2.621062	2.047779
GPLD1	206266_at	2.508494	2.490384	2.450779	2.471113	2.504527	2.462275	2.481974	2.490977	2.476371	2.46235	2.472472	2.48868	2.363712
MATK	206267_s_at	4.092314	4.280049	4.251393	4.254998	4.164987	4.147497	4.266813	4.322768	4.256443	4.587986	4.25044	4.67517	3.784051

LEFTY1	206268_at	2.060256	2.050929	1.983911	2.028532	2.040173	2.010463	4.167643	3.173944	3.497968	2.256849	2.045673	2.82493	1.976516
GCM1	206269_at	2.413276	2.385616	2.342858	2.362863	2.437978	2.369117	2.370916	2.381294	2.367719	2.352677	2.365899	2.375994	2.254391
PRKCG	206270_at	2.168308	2.151427	2.138457	2.138662	2.150427	2.133953	2.140086	2.147341	2.142202	2.13737	2.131826	2.144599	2.075552
TLR3	206271_at	6.244391	5.801046	5.789297	5.871438	4.855224	4.65239	2.027807	2.512545	2.581316	1.998318	2.027895	2.063072	2.580837
SPHAR	206272_at	6.946404	6.883238	7.678298	7.733353	7.179001	6.961862	7.141126	6.955054	7.386166	7.200357	6.848301	7.152256	7.227726
SLMO1	206273_at	2.288863	2.279673	2.248389	2.260821	2.282387	2.26822	2.281583	2.280111	2.271776	2.256215	2.457648	2.291849	2.401709
CROCC	206274_s_at	3.029216	3.030099	2.956289	3.012861	3.069912	3.012237	3.017803	3.023695	3.014394	2.994941	3.288783	3.043814	3.898684
MICAL2	206275_s_at	2.394547	2.380959	2.350234	2.365551	2.397376	2.349846	2.374194	2.382001	2.36804	2.361476	2.366068	2.435131	2.284235
LY6D	206276_at	2.646462	2.635856	2.61625	2.60246	2.636858	3.327212	2.627784	2.615944	2.606272	2.59746	2.636165	2.625433	2.439217
P2RY2	206277_at	2.277021	2.246197	2.230599	2.233319	2.259238	2.223718	2.237818	2.245773	2.23539	2.232447	2.229768	2.241614	2.13642
PTAFR	206278_at	4.913489	4.847298	4.7861	4.815679	4.922967	4.807968	4.823882	4.836059	4.761399	4.740706	4.377612	4.818125	4.120378
PRKY	206279_at	2.985779	3.586214	2.810864	2.797233	2.781572	2.780534	2.865063	2.817573	2.85134	2.809719	2.589654	2.823308	2.847862
CDH18	206280_at	2.462366	2.66645	2.430644	2.433928	2.452549	2.433622	2.445573	2.44855	2.43649	2.428417	2.432783	2.442641	3.22425
ADCYAP1	206281_at	2.220116	2.196687	2.172311	2.176949	2.202421	2.178408	2.187934	2.195629	2.180512	2.172189	2.177619	2.190264	2.699522
NEUROD1	206282_at	2.1845	2.162852	2.134579	2.142378	2.165823	2.139412	2.150887	2.162029	2.145151	2.137509	2.146973	2.157405	3.639958
TAL1	206283_s_at	4.222279	4.161082	4.692584	4.558655	4.83304	4.90108	11.75673	11.98441	11.81705	11.89755	11.24627	11.5521	4.648862
CLTB	206284_x_at	6.850805	6.687696	6.633072	6.734211	6.974066	7.033069	6.080416	6.306748	5.901566	5.819658	6.72117	6.724326	6.751892
NPHP1	206285_at	2.849172	2.77611	2.717452	2.751642	2.808655	2.763877	2.771606	2.774655	2.759493	2.721891	2.745076	2.774056	2.531059
TDGF1	206286_s_at	5.598137	5.078753	6.056854	6.169947	3.444064	4.953419	3.707906	4.394137	4.224954	3.41261	3.888006	3.122571	3.709871
ITIH4	206287_s_at	13.81025	13.85291	13.73995	13.84092	14.14379	14.13196	6.205872	8.3266	7.859102	6.45687	6.555258	7.069823	3.433391
PGGT1B	206288_at	3.036176	3.04402	2.998207	3.011401	3.019494	3.00087	3.207972	3.021306	3.100239	3.012356	3.009452	3.015223	3.893297
HOXA4	206289_at	3.861293	3.828922	3.753426	3.801416	3.962658	3.804402	3.834783	3.828652	3.8289	3.805671	4.004312	3.841244	4.487125
RGS7	206290_s_at	2.237139	2.211375	2.196347	2.207718	2.234619	2.183761	2.202676	2.217423	2.199241	2.185722	2.197387	2.209571	5.256252
NTS	206291_at	4.612897	3.596658	2.62101	2.521571	3.477433	3.476365	5.06661	4.543793	5.402088	4.887588	6.226187	5.413243	5.189521
SULT2A1	206292_s_at	12.17561	12.02044	12.01314	11.97587	10.64565	10.00791	8.014764	7.37719	8.653292	7.733656	7.757985	6.811338	6.453482
SULT2A1	206293_at	13.78855	13.63772	13.26954	13.33255	13.02944	13.02766	9.724318	10.31304	10.71711	10.26146	10.37149	8.483086	7.873543
HSD3B2	206294_at	2.572182	2.548857	2.519053	2.529987	2.554669	2.526537	2.538257	2.545684	2.53174	2.525976	2.526563	2.561881	2.814184
IL18	206295_at	3.890356	3.607621	3.835973	3.730776	4.4033	4.148333	3.499699	3.617767	3.56775	3.525708	3.856278	3.703121	2.75126
MAP4K1	206296_x_at	2.874399	2.867366	2.813541	2.85005	2.885011	2.895622	2.855321	2.858015	2.8318	2.848581	2.838888	2.85145	2.793193
CTRC	206297_at	2.44031	2.402039	2.389766	2.387676	2.409895	2.383073	2.399498	2.407809	2.392657	2.392956	2.396611	2.400012	2.293592
ARHGAP22	206298_at	2.236272	2.225066	2.178139	2.205622	2.241668	2.207011	2.220043	2.273171	2.222748	2.223834	2.308939	2.429054	2.129503
TMEM28	206299_at	2.235246	2.638259	2.187341	2.201725	2.190405	2.214505	2.210105	2.213884	2.168958	2.191438	2.170481	2.248537	2.360859
PTHLH	206300_s_at	2.657456	2.638632	2.563779	2.618904	2.689553	2.621918	2.631538	2.63157	2.618501	2.61426	2.619643	2.631331	2.561611
TEC	206301_at	2.269989	2.253346	2.290653	2.241156	2.25585	2.226949	2.252416	2.24994	2.242789	2.237376	2.234664	2.244905	2.148485
NUDT4	206302_s_at	7.737391	7.571927	8.29263	8.321711	8.022013	7.758766	9.936984	9.924011	10.4995	10.26868	9.420735	10.09433	8.743855
NUDT4	206303_s_at	3.891531	3.951548	4.783802	4.66919	4.061103	3.596336	6.919213	5.160261	6.735977	6.988293	5.080001	5.611373	5.195758
MYBPH	206304_at	2.243086	2.212547	2.192851	2.202029	2.225182	2.206279	2.203495	2.215665	2.198919	2.188712	7.697077	2.208097	6.737381
C8A	206305_s_at	11.43155	11.4601	12.17752	12.2025	11.87179	11.76575	9.387659	9.524708	8.779718	9.416622	9.028915	9.52855	2.75596

RYR3	206306_at	2.444216	2.405324	2.381132	2.389067	2.426839	2.371799	2.398895	2.437827	2.395271	2.38493	2.949445	2.40892	5.261927
FOXD1	206307_s_at	2.692418	2.650753	2.365763	2.651437	2.693161	2.652997	2.657608	2.670185	2.654282	2.642362	2.907078	2.677658	6.915442
TRDMT1	206308_at	2.751602	2.737142	2.729743	2.453542	2.867687	2.749991	4.21667	3.966224	4.436085	4.433461	4.017579	3.20984	4.555184
LECT1	206309_at	1.981989	1.96646	1.944577	1.951619	1.967024	1.947935	1.956881	1.963628	1.951225	1.943326	1.947053	1.95841	8.570451
SPINK2	206310_at	2.144444	2.13234	2.107711	2.109247	2.13347	2.112147	2.123966	2.218643	2.133261	2.781888	2.125714	2.247349	2.122755
PLA2G1B	206311_s_at	2.90725	2.889709	2.866349	2.870503	2.890526	2.870831	3.713006	3.391922	3.909736	3.060832	2.892013	3.962506	2.583507
GUCY2C	206312_at	2.096639	2.075964	2.052067	2.058345	2.079392	2.057909	2.062533	2.071947	2.06056	2.054218	2.053438	2.072251	1.99558
HLA-DOA	206313_at	2.269894	2.254026	2.212756	2.237	2.25583	2.238146	2.245213	2.253748	2.240439	2.601501	2.243003	2.250276	2.151423
ZNF167	206314_at	3.511203	3.852115	2.803176	2.649523	3.282967	2.921713	3.410958	3.292817	3.911971	3.967935	4.267033	3.257742	5.935875
CRLF1	206315_at	2.520989	2.509178	2.485245	2.492773	2.518801	2.491259	2.465795	2.505152	2.396905	2.488598	2.608187	2.522234	3.954377
KNTC1	206316_s_at	3.65533	3.477871	3.226612	3.192065	3.167298	3.098266	7.638718	6.523399	7.677489	7.815006	6.515798	6.570734	6.608427
ABCB8	206317_s_at	3.059094	3.055346	3.034809	3.778274	3.040748	3.034208	3.049973	3.043226	3.045918	3.127909	3.182573	3.042096	3.000698
SPINLW1	206318_at	2.434682	2.397521	2.361293	2.387755	2.421346	2.391354	2.388185	2.398021	2.385484	2.368386	2.3848	2.391752	2.28062
SPINLW1	206319_s_at	1.936576	1.915392	1.894675	1.899786	1.914923	1.898437	1.90989	1.913481	1.90142	1.895882	1.89357	1.906572	1.84224
SMAD9	206320_s_at	2.339229	2.326622	2.301619	2.30575	2.317207	2.270016	2.317231	2.319557	2.307822	2.306289	2.303838	2.334458	2.534196
RFX1	206321_at	2.323447	2.313657	2.274862	2.293472	2.312756	2.859677	2.312669	2.313146	2.303786	2.286707	2.299033	2.305679	2.278059
SYN3	206322_at	2.495533	2.468039	2.438654	2.449818	2.477893	2.448404	2.463892	2.470879	2.465289	2.451289	2.475587	2.483543	2.312906
OPHN1	206323_x_at	9.603795	10.05002	9.324776	9.643017	9.811186	10.32234	9.408771	9.489839	9.49859	9.391019	9.517868	9.455881	9.897611
DAPK2	206324_s_at	3.698538	3.467983	3.433086	3.440347	3.462117	4.029066	3.433029	3.44409	3.358088	3.317015	3.426445	3.446131	3.447183
SERPINA6	206325_at	11.59966	11.71172	11.47906	11.58882	10.86897	10.53212	11.87357	11.24277	11.67769	11.7826	11.51632	11.61432	5.120453
GRP	206326_at	2.726308	2.688637	2.66349	2.677652	2.711428	2.65676	2.685808	2.692509	2.681339	2.656139	2.67534	2.707842	4.610149
CDH15	206327_s_at	2.74779	2.667807	2.649542	2.655881	2.66854	2.660981	2.665521	2.671122	2.649469	2.615087	5.156759	2.565528	3.470811
CDH15	206328_at	2.259875	2.244279	2.213292	2.229696	2.246507	2.229731	2.241675	2.604153	2.232042	2.226112	3.070107	2.242528	2.655878
EXTL1	206329_at	2.568223	2.619388	2.718008	2.545573	2.580985	2.612802	2.586784	2.623519	2.554203	2.530772	2.544575	2.548803	2.448583
SHC3	206330_s_at	2.555714	2.533968	2.501173	2.514221	2.554928	2.509382	2.529627	2.53088	2.517313	2.509452	2.517388	2.525832	2.544778
CALCRL	206331_at	2.810447	2.649055	4.185347	3.11448	2.749675	2.747827	2.758073	2.735147	2.753607	2.745962	2.344059	2.714314	5.548851
IFI16	206332_s_at	6.883543	7.025682	7.453031	7.316365	7.138484	7.123089	7.21995	6.386817	6.769732	6.817714	7.163091	6.725401	7.764569
MSI1	206333_at	3.180444	3.071543	2.988505	3.047725	3.291492	3.060803	3.0791	3.075676	3.07549	3.066324	3.229761	3.130464	2.798739
LIPF	206334_at	2.613984	2.566666	2.505367	2.542803	2.585435	2.521987	2.555955	2.584766	2.551999	2.540948	2.553451	2.562202	2.40925
GALNS	206335_at	6.523098	6.538332	6.569092	6.611953	7.03996	6.906615	5.453511	5.810348	5.673758	5.098477	5.97412	4.725308	7.315261
CXCL6	206336_at	3.664803	4.484074	4.168354	3.629016	5.24583	5.299916	3.144306	3.144405	3.13971	3.175929	3.192185	3.214356	3.028994
CCR7	206337_at	2.41478	2.387264	2.347226	2.154846	2.405397	3.911325	2.377103	2.387798	2.267775	2.359107	2.371079	2.384079	2.248582
ELAVL3	206338_at	3.906786	3.910162	3.727757	3.85529	3.881161	3.871479	3.863184	3.825935	3.858934	3.79362	3.860607	4.021085	3.64178
CARTPT	206339_at	3.239134	3.180908	3.067295	3.193736	3.231182	3.19751	3.208479	3.213653	3.198713	3.175786	3.22419	3.253987	3.129675
NR1H4	206340_at	10.04704	10.00849	10.21861	10.30203	10.1328	9.921145	9.573654	9.036258	9.016647	9.294943	8.411526	8.912484	4.082933
IL2RA	206341_at	2.915034	3.121137	2.800275	2.867624	2.907717	3.466247	2.8395	3.056773	2.836587	2.8098	2.903026	2.936286	2.604988
IDS	206342_x_at	4.390374	4.108945	5.850926	5.857207	5.714808	5.674337	2.922587	3.564978	3.491811	2.939589	3.084102	2.510428	4.615071
NRG1	206343_s_at	6.353764	6.547001	4.696039	5.108348	6.324056	6.008754	3.790558	3.938556	3.392369	3.194605	2.661818	3.354416	3.043949

PON1	206344_at	9.154695	9.002767	9.712501	10.0165	9.316065	8.969268	2.49479	5.510371	2.470193	4.581614	3.488082	4.009189	2.301041
PON1	206345_s_at	12.29951	12.27961	13.33054	13.35602	12.0159	11.80737	6.520724	8.155812	2.460733	7.886331	5.810766	2.868721	2.07119
PRLR	206346_at	3.319673	3.081132	3.430331	3.216172	3.272679	3.046645	3.551848	3.077262	3.225762	3.085594	3.077358	3.079069	2.688219
PDK3	206347_at	3.031584	2.943348	3.000124	2.952848	3.014275	2.867321	3.035522	3.104388	3.246102	3.028413	2.994508	3.226299	2.879265
PDK3	206348_s_at	2.816784	2.803987	2.879328	2.790111	2.805748	2.701341	2.747314	3.403879	3.268459	3.185924	2.789526	3.021636	2.692014
LGI1	206349_at	2.377342	2.338482	2.327366	2.332696	2.362616	2.336355	2.33618	2.346043	2.333535	2.321388	2.348189	2.358447	6.327116
APCS	206350_at	12.93796	12.94071	12.90496	12.92189	12.80879	12.61567	2.447878	2.843485	2.90098	3.162389	2.344223	2.451771	2.066753
PEX10	206351_s_at	3.502942	3.560051	3.539031	3.540478	3.475377	3.319727	3.874648	3.55825	4.041069	3.536885	3.52582	3.622253	3.51327
PEX10	206352_s_at	4.076718	4.068149	4.071702	4.117805	4.065229	4.037995	4.067311	4.611907	4.065018	4.055111	4.075744	4.68675	3.654628
COX6A2	206353_at	6.389272	6.551424	6.332575	6.362951	6.658945	6.838653	6.319664	6.371614	6.354523	6.302947	6.943434	6.364894	7.448129
SLCO1B3	206354_at	11.78444	11.5979	12.71254	12.46524	10.28531	9.529236	10.1503	9.320424	10.12862	10.91796	8.429594	8.010683	3.498689
GNAL	206355_at	2.710344	2.670474	2.634053	2.656051	2.693497	2.659716	2.66629	2.67485	2.663183	2.647768	2.669341	2.677533	4.252294
GNAL	206356_s_at	2.13006	2.101942	2.081751	2.082573	2.105656	2.072614	2.098008	2.098936	2.08824	2.08115	2.085031	2.092262	2.069703
OPA3	206357_at	3.441387	3.71894	4.149307	3.520001	3.848795	3.943135	3.169599	3.207958	3.195619	3.13794	3.225422	3.467631	2.808787
PRM1	206358_at	2.44077	2.680581	2.40258	2.410299	2.425181	2.417888	2.422457	2.424051	2.409596	2.401685	2.405854	2.419043	2.280038
SOCS3	206359_at	3.342814	3.263763	3.987586	3.253106	3.331812	3.554141	2.965324	3.266499	3.262696	3.238419	3.258686	3.25528	3.093731
SOCS3	206360_s_at	3.181054	3.463757	3.07375	2.995495	3.211254	3.196224	3.209012	4.03651	3.200597	3.195227	3.250566	3.409866	2.54778
GPR44	206361_at	2.233483	2.239676	2.181033	2.223574	2.240736	2.233508	6.08657	5.411318	6.147823	5.720856	5.55675	5.677849	2.207492
MAP3K10	206362_x_at	5.234495	5.224521	4.967475	5.213143	5.226497	5.217086	5.225928	5.225015	5.221477	5.198896	5.149357	5.361087	4.540561
MAF	206363_at	8.367028	8.11028	8.581039	8.856082	8.225933	8.180933	7.337305	6.228437	6.900174	6.545602	6.253623	6.925012	6.493863
KIF14	206364_at	3.934608	3.927924	3.290086	3.347564	3.394708	3.457298	8.062238	7.116009	8.328026	8.570456	6.678651	7.181763	6.595482
XCL1	206365_at	2.138929	2.119253	2.091491	2.101148	2.121899	2.099533	2.1094	2.118556	2.103378	2.094984	2.103036	2.11322	2.026701
XCL2	206366_x_at	2.117181	2.11378	2.212029	2.202736	2.105657	2.067083	1.996443	2.107976	2.098733	1.979633	2.06927	2.080113	2.004499
REN	206367_at	2.132634	2.116567	2.097833	2.09822	2.115969	2.099527	2.109434	2.113803	2.100189	2.092442	2.097056	2.107283	2.080184
CPLX2	206368_at	2.481426	2.448378	2.434008	2.437846	2.463723	2.437474	2.444545	2.452725	2.444037	2.413777	2.437604	2.447843	2.301309
PIK3CG	206369_s_at	2.330261	2.300816	2.262689	2.282591	2.309664	2.284314	2.289064	2.298454	2.286043	2.277503	2.282453	2.295165	2.185892
PIK3CG	206370_at	3.723362	3.535309	3.349033	3.600268	3.906037	3.566352	3.795013	3.634061	3.540357	3.534077	3.654127	3.731737	3.194811
FOLR3	206371_at	2.703875	2.643656	2.58493	2.58487	2.657292	2.646256	2.64758	2.655332	2.637667	2.629476	2.640482	2.668336	2.48359
MYF6	206372_at	2.400837	2.374861	2.326438	2.361018	2.379928	2.347132	2.363194	2.428918	2.363667	2.368237	3.040053	2.369032	5.367508
ZIC1	206373_at	2.037634	2.7322	5.820804	6.440683	8.048884	8.074695	2.049358	2.063596	2.046701	2.033987	2.277813	2.058376	10.58114
DUSP8	206374_at	3.119994	3.081659	2.403191	2.875895	2.535642	2.519026	3.18418	4.222886	2.209108	3.062013	3.644206	3.070452	4.044318
HSPB3	206375_s_at	2.46942	2.449499	2.42814	2.43545	2.451163	2.432489	2.443805	2.448286	2.431495	2.427115	6.535024	2.441292	8.887254
SLC6A15	206376_at	2.656912	2.624686	2.605206	2.610084	2.642258	2.602244	2.62095	2.628702	2.613116	2.602613	2.614864	2.628273	5.73259
FOXF2	206377_at	2.028391	2.006444	1.988177	1.991144	2.011823	2.002701	2.003461	2.006293	1.993161	1.986235	1.99032	2.003527	5.120166
SCGB2A2	206378_at	3.061949	3.036428	2.925821	3.011491	3.138342	2.992651	3.027736	3.033412	3.020957	3.000806	3.036666	3.08097	2.869908
EYA3	206379_at	2.29454	2.290581	2.264777	2.27064	2.278178	2.269753	2.294072	2.290945	2.273642	2.26627	2.276757	2.283113	2.238356
CFP	206380_s_at	6.170149	5.878116	7.334658	7.550272	6.940293	6.926767	4.962281	5.794588	5.903578	5.186739	5.196934	5.577742	3.130607
SCN2A	206381_at	2.23338	2.210555	2.185727	2.304068	2.214186	2.193642	2.205942	2.208037	2.195695	2.188064	2.194417	2.26936	3.1836

BDNF	206382_s_at	2.57015	2.521825	2.471175	2.516537	2.58347	2.492059	2.522588	2.536347	2.551558	2.500383	2.649193	2.600473	3.224957
G3BP2	206383_s_at	3.063544	3.016403	3.889675	3.004264	3.394437	3.031993	4.119659	3.025614	4.067789	3.529918	3.032202	3.043989	6.164913
CACNG3	206384_at	2.444874	2.409408	2.372477	2.393052	2.425956	2.385838	2.345649	2.411504	2.397216	2.383726	2.329424	2.404981	2.301752
ANK3	206385_s_at	8.890834	8.953143	9.145558	9.05257	9.701401	9.650474	8.385628	7.667883	8.055004	7.974248	8.973474	8.38786	10.80331
SERPINA7	206386_at	11.50475	11.47431	11.50237	11.65354	11.78389	11.64709	8.893816	9.497847	9.07032	9.004309	8.749009	8.379909	2.233157
CDX2	206387_at	2.744702	2.734183	2.716434	2.721569	2.726103	2.721652	2.730753	2.731426	2.721606	2.719706	2.713153	2.725572	2.678781
PDE3A	206388_at	2.184461	2.170967	2.140791	2.153509	2.171264	2.159404	2.162619	2.170384	2.155663	2.151957	2.154648	2.16505	2.08238
PDE3A	206389_s_at	2.681623	2.642013	2.603328	2.619324	2.715866	2.610126	2.63328	2.646043	2.633873	2.61671	2.629103	2.656576	2.6892
PF4	206390_x_at	2.837042	4.178659	3.215877	3.773266	5.419705	4.264127	11.9046	10.85572	11.34424	11.64831	11.16938	11.96036	4.943424
RARRES1	206391_at	2.256868	2.249366	2.21481	2.227233	2.250455	2.263251	2.230455	2.243677	2.235492	2.229836	2.22545	2.306168	2.192556
RARRES1	206392_s_at	2.413935	2.38243	2.365827	2.391695	2.399082	2.36915	2.405241	2.403587	2.600289	2.395837	2.388233	2.387551	3.249127
TNNI2	206393_at	2.301015	2.273236	2.238221	2.252608	2.286579	2.246172	2.260194	2.271541	2.257157	2.246091	7.898501	2.266147	8.416216
MYBPC2	206394_at	2.094082	2.077316	2.049198	2.059011	2.091669	2.056564	2.065723	2.077572	2.061775	2.050655	2.491339	2.069	2.231631
DGKG	206395_at	2.146107	2.133436	2.113905	2.117638	2.145337	2.123684	2.126819	2.129144	2.123222	2.118993	2.113778	2.123949	2.039233
SLC1A1	206396_at	2.108933	2.078493	4.308249	3.268699	2.236484	2.304991	2.08136	2.088664	1.988746	2.054256	1.985153	1.996781	2.073625
GDF1	206397_x_at	2.293235	2.281582	2.244474	2.256623	2.292969	2.264182	2.276572	2.281843	2.2661	2.25168	2.7902	2.275696	3.908704
CD19	206398_s_at	2.282635	2.273522	2.253454	2.257325	2.280904	2.265422	2.261512	2.271042	2.255015	2.253957	2.259111	2.265707	2.156103
CACNA1A	206399_x_at	2.826414	2.946325	2.838467	2.823497	2.915879	2.841779	2.842905	2.847644	2.752329	2.968619	2.779992	2.784123	2.787271
LGALS7	206400_at	2.181213	2.151375	2.129305	2.131642	2.160296	2.133972	2.14673	2.151255	2.140259	2.133612	2.137051	2.15108	3.269508
MAPT	206401_s_at	2.716169	2.688011	2.61637	2.671792	2.709246	2.659142	2.677109	2.678145	2.668153	2.643808	2.670029	2.692278	7.144981
NPFF	206402_s_at	2.8866	3.02993	2.861912	2.863854	3.03704	3.329911	2.880286	2.880348	2.868433	2.867613	3.054241	2.872412	2.976873
ZNF536	206403_at	2.140043	2.122499	2.098134	2.104589	2.12872	2.107913	2.119219	2.119885	2.107246	2.102629	2.110398	2.114198	4.225526
FGF9	206404_at	2.300976	2.284425	2.260071	2.267433	2.287035	2.269989	2.275836	2.284073	2.270832	2.264439	5.644592	2.304797	7.197883
USP6	206405_x_at	6.482634	6.484443	6.014806	6.025768	6.329459	6.312457	7.904453	6.396062	6.482281	6.593623	7.057899	7.071686	8.722958
SMCP	206406_at	2.309577	2.291484	2.271496	2.268764	2.292178	2.279722	2.285923	2.29105	2.283456	2.271328	2.276687	2.28328	2.148696
CCL13	206407_s_at	2.655027	2.579546	2.576596	2.570183	2.644507	2.557374	2.582063	2.592359	2.583593	2.549009	2.581743	2.585696	2.490177
LRRTM2	206408_at	2.935379	2.733459	2.719119	2.723579	2.867823	2.722759	2.725757	2.685769	2.723996	2.66057	2.721131	2.731649	5.335228
TIAM1	206409_at	3.731135	3.638758	3.913524	3.914111	3.808494	4.006218	3.677872	4.220921	3.374336	3.631947	3.774327	3.383565	4.147522
NR0B2	206410_at	9.515755	9.483879	8.162775	8.810299	9.041712	8.901664	6.570291	7.055759	6.873592	7.221365	6.557683	7.184218	1.733769
ABL2	206411_s_at	1.815338	1.805222	1.783056	1.789863	1.804484	1.79342	1.929625	1.836415	1.793447	1.79172	1.794191	1.82198	1.747453
FER	206412_at	2.686296	2.661988	2.546805	2.64955	2.673347	2.629454	3.015973	3.009441	2.657365	2.634589	2.653326	2.718694	4.579928
TCL1B	206413_s_at	2.350034	2.334108	2.296496	2.325409	2.332349	2.318196	2.336225	2.714034	2.403688	2.326216	2.321557	2.329043	2.262148
DDEF2	206414_s_at	5.608402	5.586513	5.909675	5.835481	5.498585	5.406125	6.679871	6.108481	6.423949	6.53251	7.104488	6.512814	8.679703
TLL1	206415_at	3.184328	3.08338	2.994345	3.042917	3.182062	3.012705	3.042479	3.074817	3.068211	3.022785	3.0926	3.13218	2.813732
ZNF205	206416_at	5.7228	5.279725	5.192046	5.19148	6.295219	6.081379	5.24135	5.968399	4.551455	5.008402	5.266034	5.694317	4.943435
CNGA1	206417_at	7.128746	7.06271	7.682966	7.874005	7.424346	6.969841	7.143204	6.505365	7.386379	7.160266	6.525592	7.166393	2.14201
NOX1	206418_at	2.459599	2.426033	2.379144	2.407554	2.443853	2.402725	2.418663	2.42652	2.413386	2.402915	2.412033	2.424527	2.292107
RORC	206419_at	3.735809	4.141095	5.442265	4.156594	3.237806	3.120998	2.855814	2.590019	2.184773	2.72823	2.513978	2.520152	1.847105

IGSF6	206420_at	5.346428	4.568791	4.492307	4.905532	6.538946	6.758176	5.409206	5.14879	4.455676	4.479953	4.352158	5.037076	2.611923
[truncated: 807,192 more chars]
